# The Effect of Age, Parity and Body Mass Index on the Efficacy, Safety, Placement and User Satisfaction Associated With Two Low-Dose Levonorgestrel Intrauterine Contraceptive Systems: Subgroup Analyses of Data From a Phase III Trial

**DOI:** 10.1371/journal.pone.0135309

**Published:** 2015-09-17

**Authors:** Kristina Gemzell-Danielsson, Dan Apter, Brian Hauck, Thomas Schmelter, Sarah Rybowski, Kimberly Rosen, Anita Nelson

**Affiliations:** 1 Department of Women’s and Children’s Health, Division of Obstetrics and Gynecology, Karolinska Institutet and Karolinska University Hospital, Stockholm, SE-171 76, Sweden; 2 Sexual Health Clinic (Family Federation of Finland), Väestöliitto, Helsinki, 00101, Finland; 3 Department of Obstetrics and Gynecology, Foothills Hospital, University of Calgary, Calgary, Alberta, T2N 4J8, Canada; 4 Bayer Pharma AG, Müllerstrasse 178, Berlin, 13353, Germany; 5 Bayer HealthCare Pharmaceuticals, Whippany, New Jersey, United States of America; 6 Los Angeles Biomedical Research Institute, Harbor-UCLA, 1000 West Carson Street Torrance, California, 90405, United States of America; NHS lothian and University of Edinburgh, UNITED KINGDOM

## Abstract

**Objective:**

Two low-dose levonorgestrel intrauterine contraceptive systems (LNG-IUSs; total content 13.5 mg [average approx. 8 μg/24 hours over the first year; LNG-IUS 8] and total content 19.5 mg [average approx. 13 μg/24 hours over the first year; LNG-IUS 13]) have previously been shown to be highly effective (3-year Pearl Indices: 0.33 and 0.31, respectively), safe and well tolerated. The present subgroup analyses evaluated whether or not outcomes were affected by parity, age (18–25 vs 26–35 years), or body mass index (BMI, <30 vs ≥30 kg/m^2^).

**Methods:**

Nulliparous and parous women aged 18‒35 years with regular menstrual cycles (21‒35 days) requesting contraception were randomized to 3 years of LNG-IUS 8 or LNG-IUS 13 use.

**Results:**

In the LNG-IUS 8 and LNG-IUS 13 groups, 1432 and 1452 women, respectively, had a placement attempted and were included in the full analysis set; 39.2%, 39.2% and 17.1% were 18–25 years old, nulliparous and had a BMI ≥30 kg/m^2^, respectively. Both systems were similarly effective regardless of age, parity or BMI; the subgroup Pearl Indices had widely overlapping 95% confidence intervals. Placement of LNG-IUS 8 and LNG-IUS 13 was easier (p < 0.0001) and less painful (p < 0.0001) in women who had delivered vaginally than in women who had not. The complete/partial expulsion rate was 2.2–4.2% across all age and parity subgroups and higher in parous than in nulliparous women (p = 0.004). The incidence of pelvic inflammatory disease was 0.1–0.6% across all age and parity subgroups: nulliparous and younger women were not at higher risk than parous and older women, respectively. The ectopic pregnancy rate was 0.3–0.4% across all age and parity subgroups. Across all age and parity subgroups, the 3-year completion rate was 50.9–61.3% for LNG-IUS 8 and 57.9–61.1% for LNG-IUS 13, and was higher (p = 0.0001) among older than younger women in the LNG-IUS 8 group only.

**Conclusions:**

LNG-IUS 8 and LNG-IUS 13 were highly effective, safe and well tolerated regardless of age or parity.

**Trial Registration:**

Clinical trials.gov NCT00528112

## Introduction

Although national and international guidance supports the use of intrauterine contraception (IUC) in a wide range of women, regardless of age and parity [1–4], IUC is used less frequently in younger and nulliparous women. In a US-based survey that analysed healthcare providers’ attitudes to providing IUC, more than 60% of providers reported they infrequently provided IUC to nulliparous women [5].

Bayer HealthCare has developed two low-dose levonorgestrel intrauterine contraceptive systems (LNG-IUSs); total content 13.5 mg (average approx. 8 μg/24 hours over the first year; hereafter referred to as LNG-IUS 8) and total content 19.5 mg (average approx. 13 μg/24 hours over the first year; hereafter referred to as LNG-IUS 13), designed to be potentially more suitable for women with either a tighter cervical canal or a smaller uterine cavity. Compared with LNG-IUS total content 52 mg (average approx. 20 μg/24 hours over the first year; hereafter referred to as LNG-IUS 20; Mirena), LNG-IUS 8 and LNG-IUS 13 have smaller T-frames (28 × 30 mm vs 32 × 32 mm) and smaller hormone reservoirs, allowing them to be placed using a smaller diameter placement tube (3.80 mm vs 4.75 mm or 4.4 mm, with the recently marketed EvoInserter).

In a Phase II study, LNG-IUS 8 and LNG-IUS 13 demonstrated good contraceptive efficacy over 3 years with no apparent dose–response, were well tolerated and were significantly easier and less painful to place compared with placement of LNG-IUS 20 using the 4.75 mm diameter placement tube [6]. LNG-IUS 8 and LNG-IUS 13 subsequently underwent further evaluation in a Phase III study; the overall results of this study have been reported previously [7]. Based on the results of these two studies, LNG-IUS 8 was approved by the US Food and Drug Administration (as Skyla) for up to 3 years of contraceptive use. In addition, LNG-IUS 8 was deemed ‘approvable’ by the Decentralized Procedure in the EU and was subsequently approved (as Jaydess) in most European countries, Canada and some countries in Latin America [8,9].

Here, we present *post hoc* subgroup analyses of Phase III data that evaluate the effect of age, parity and body mass index (BMI) on the efficacy of LNG-IUS 8 and LNG-IUS 13; the effect of age and parity on safety outcomes, continuation rates, reasons for discontinuation and user satisfaction; and the effect of parity on the ease and pain of placement.

## Materials and Methods

This open-label, randomized Phase III study (clinical trials.gov; NCT00528112; https://clinicaltrials.gov/ct2/show/NCT00528112) was conducted at 138 study centres across 11 countries between August 2007 and June 2011. The protocol (see S1 Protocol) and its amendments were reviewed and approved by the independent ethics committee or institutional review board at each study site and the study was conducted in accordance with the Declaration of Helsinki and Good Clinical Practice guidelines. Written informed consent was obtained from all participants before study entry.

The methodology and overall results have been published previously [7]. The pertinent elements of the protocol were as follows: Healthy nulliparous and parous women aged 18–35 years (inclusive) with regular menstrual cycles (21–35 days) requesting contraception were recruited. Women were required to have uterine conditions considered by the investigating physician to be suitable for placement of an LNG-IUS, although no maximum or minimum uterine dimensions were specified and no screening failures were reported to be due to an investigator considering the uterus too small.

Subjects were randomized 1:1 to 3 years of treatment with either LNG-IUS 8 or LNG-IUS 13. Treatment assignment was blinded to participating women. However, it was not possible to blind investigators to randomization because of visible differences between LNG-IUS 8 and LNG-IUS 13 in the length of the hormone reservoir.

LNG-IUS 8 and LNG-IUS 13 were placed during the first 7 days of the woman’s menstrual cycle. Two placement attempts were permitted per woman and if both attempts failed, the subject was withdrawn from the study. The use of local anaesthesia, oral analgesics and cervical dilation was permitted at the discretion of individual investigators. After placement, ultrasound was used to confirm the ‘compliant’ positioning of the system (i.e. completely within the uterine cavity).

At the LNG-IUS placement visit, investigators rated the ease of placement (from their own perspective) as ‘easy’, ‘slightly difficult’ or ‘very difficult’, based on their own perception of what these terms meant. In addition, women rated their pain on placement as ‘none’, ‘mild’, ‘moderate’ or ‘severe’, again based on their own perception of what these terms meant.

All uterine perforations and ectopic pregnancies were to be reported as serious adverse events (SAEs). Complete or partial expulsions were to be reported as AEs. During the study, a protocol amendment in 2009 clarified the definition of a partial expulsion (LNG-IUS at least partially located in the cervical canal by ultrasound and/or partially visible in the vagina on clinical examination) and confirmed that it was not necessary to remove an LNG-IUS that was completely within the uterine cavity (not in the cervical canal) even if it was not in the fundal position.

Another protocol amendment in 2009 required that participating women were asked to complete a user satisfaction questionnaire either at the end of 3 years (those who completed 3 years of treatment) or at their final study visit (those who discontinued prematurely). Women rated their overall satisfaction with study treatment as: ‘very satisfied’, ‘somewhat satisfied’, ‘neither satisfied/dissatisfied’, ‘dissatisfied’ or ‘very dissatisfied’. For women who experienced bleeding while on treatment, satisfaction with their menstrual bleeding pattern was rated as: ‘very satisfied’, ‘somewhat satisfied’, ‘neither satisfied/dissatisfied’, ‘dissatisfied’ or ‘very dissatisfied’. For women who did not experience bleeding while on treatment, their satisfaction with their menstrual bleeding pattern was recorded as ‘not applicable’. When asked about their preference for use of contraception after the study, women answered from the following options: ‘continue with study treatment’, ‘use a different hormonal contraceptive’, ‘use a different contraceptive method’, ‘discontinue use of all types of contraception’ or ‘don’t know’.

The pregnancy rate was expressed as the Pearl Index (number of pregnancies per 100 woman-years; the primary efficacy variable). Pearl Index and the corresponding confidence interval (CI) calculations assumed that the number of pregnancies followed a Poisson distribution. The cumulative failure rate was also calculated using the Kaplan–Meier method. Exposure calculations were based on the time from LNG-IUS placement until expulsion/removal of the system, or the end of 3 years of use. Months during which women used concomitant contraception (e.g. condoms) or prohibited medications such as sex steroid hormones were subtracted from respective exposure times. If a pregnancy occurred during such a month, the pregnancy would have been considered but the month would have been subtracted from the exposure time. Women who had a failed placement attempt were assumed to have an exposure time of 1 day (because they were considered to have been briefly exposed during the placement attempt) and were included in the efficacy analysis. Pregnancies that occurred after LNG-IUS removal or known expulsion were not included in Pearl Index calculations.

Analyses included data from all randomized women for whom LNG-IUS placement was attempted (full analysis set), with the exception of the analysis of expulsion rates, which included only women who had had a successful placement. Subgroup analyses were performed to evaluate the effect of age, parity and BMI on the efficacy of LNG-IUS 8 and LNG-IUS 13; the effect of age and parity on safety outcomes, continuation rates, reasons for discontinuation and user satisfaction; and the effect of parity on the ease and pain of placement. P values for differences in outcomes between the subgroups were calculated using log rank tests and Fisher’s exact tests. All statistical tests were performed using SAS software.

As previously reported [7], the sample size for the study was calculated so that sufficient women were recruited to give, in both treatment groups, a Pearl Index (primary endpoint) with a two-sided 95% CI, such that the difference between the upper limit of the CI and the point estimate (expressed in pregnancies per 100 woman-years) did not exceed 1 for each considered year of treatment. However, it is difficult to evaluate power for *post hoc* subgroup analyses because this would require the determination of what constitutes a relevant difference for each outcome variable under consideration, before performing a power calculation for each of the subgroup analyses. Power analyses for the *post hoc* analyses were therefore not performed.

### Ethics statement

The study protocol and its amendments were approved by the following ethics committees and institutional review boards (IRB):

#### Argentina

Comité Independiente Etica para Ensayos Farmacologia Clinica "Prof. Dr. Luis Maria Zieher", Buenos Aires; Comité de Ética Julio Cesar Maiztegui, Santa Fe; Hospital Bernardino Rivadavia Comité de Bioética, Buenos Aires.

#### Canada

Biomedical Research Ethics Board, University of Manitoba, Winnipeg; Ethics Review Board, Queens University, Kingston; Office of Research Ethics, University of Western Ontario; Conjoint Health Research Ethics Board, University of Calgary, Calgary.

#### Chile

Comité de Etica Servicio de Salud Metropolitano Norte, Santiago; Servicio de Salud Metropolitano Oriente Comité Etico Cientifico, Providencia.

#### Finland

Ethics Committee of HUS (University Hospital District of Southern Finland), Helsinki.

#### France

Comité de Protection des Personnes Sud Méditerranée III, Nimes.

#### Hungary

Medical Research Council EC for Clinical Pharmacology, Budapest; Regional Human Medical Research Ethical Committee, University of Szeged; Institutional Ethics Committee, Bács-Kiskun County Hospital, Kecskemét; Institutional Research Ethics Committee, Bekes County Hospital “Rethy Pal”, Bekescsaba.

#### Mexico

Comité de Bioética de la Facultad de Medicina de Torreón, Torreón; Comité de Etica, Hospital Integral de la Mujer del Estado De Sonora, Hermosillo; Comité Bioético para la Investigación Clinica S. C., Mexico D. F.; Comité de Etica de la Facultad de Medicina, Hospital Universitario “José Eleuterio González”, Monterrey.

#### Netherlands

IRB, Prinsengracht 83, Amsterdam.

#### Norway

Reg. Komité for Medisinsk og Helsefaglig Forskningsetikk, St Olavs Hospital, Trondheim.

#### Sweden

Regionala Etikprövningsnämnden, Stockholm.

#### USA

Independent Investigational Review Board, Inc., Florida; IRB, Columbia University Medical Center, NY; Chesapeake IRB, Columbia, Maryland; Scripps Cancer Center IRB, La Jolla, California; Research and Clinical Trials Administration Office, Rush University Medical Center, Chicago, Illinois; IRB, Oregon Health and Science University, Portland, Oregon; IRB University of Cincinnati, Ohio; Western University IRB, Washington, USA; John F Wolf Human Subjects Committee, LA Biomedical Research Institute, Harbor-UCLA Medical Center, Torrance, California; Crescent City IRB, New Orleans, Louisiana; Independent IRB, 1550 Sawgrass Corporate Parkway, Florida.

## Results

The disposition of women in the full analysis set has previously been reported [7]. The distribution of women among the subgroups analysed is shown in Table 1 (S1 Data). Among 2884 subjects in the full analysis set, 39.2% were nulliparous and 51.5% had never had a vaginal delivery. The maximum depth to which a woman’s uterus sounded was 10 cm among nulliparous women and 11 cm among parous women. However, no maximum or minimum uterine dimensions were specified in the eligibility criteria.

**Table 1:** Baseline Characteristics (Full Analysis Set).

Variable	LNG-IUS 8 (n = 1432)	LNG-IUS 13 (n = 1452)	Total (n = 2884)
**Age category, n (%)**			
18−25 years	566 (39.5)	564 (38.8)	1130 (39.2)
26–35 years	866 (60.5)	888 (61.2)	1754 (60.8)
**Body mass index, n (%)**			
<30 kg/m^2^	1187 (82.9)	1198 (82.5)	2385 (82.7)
≥30 kg/m^2^	244 (17.0)	250 (17.2)	494 (17.1)
Data missing	1 (0.1)	4 (0.3)	5 (0.2)
**Number of births, n (%)**			
0 (nulliparous)	556 (38.8)	574 (39.5)	1130 (39.2)
1	320 (22.3)	333 (22.9)	653 (22.6)
≥2	556 (38.9)	545 (37.6)	1101 (38.2)
**Number of vaginal deliveries, n (%)**			
0	743 (51.9)	743 (51.2)	1486 (51.5)
1	269 (18.8)	275 (18.9)	544 (18.9)
≥2	418 (29.2)	433 (29.8)	851 (29.5)
Data missing	2 (0.1)	1 (<0.1)	3 (0.1)
**Parous; Caesarean section delivery only, n (%)**	188 (13.1)	169 (11.6)	357 (12.4)

LNG-IUS 8; levonorgestrel intrauterine system total content 13.5 mg (average approx. 8 μg/24 hours over the first year); LNG-IUS 13, levonorgestrel intrauterine system total content 19.5 mg (average approx. 13 μg/24 hours over the first year)

The first-year and 3-year unadjusted Pearl Indices and the unadjusted Kaplan–Meier estimates for the first-year and 3-year cumulative failure rates for LNG-IUS 8 and LNG-IUS 13 are shown in Tables 2 and 3 (S1 Data). These efficacy outcomes were not significantly affected by women’s age, parity status or BMI (95% CIs overlapped).

**Table 2:** Unadjusted First-year and 3-year Pearl Indices According to Age, Parity Status and BMI (Full Analysis Set).

		LNG-IUS 8				LNG-IUS 13			
		Number of pregnancies/number of subjects	Relevant exposure* [WY]	Pearl Index	95% CI	Number of pregnancies/number of subjects	Relevant exposure* [WY]	Pearl Index	95% CI
**Analysis period**	**Age category**								
First year	18–25 years	1/566	455.62	**0.22**	0.01–1.22	1/564	473.33	**0.21**	0.01–1.18
	26–35 years	4/866	762.15	**0.52**	0.14–1.34	1/888	779.45	**0.13**	0.00–0.71
3-year	18–25 years	4/566	1114.21	**0.36**	0.10–0.92	2/564	1207.19	**0.17**	0.02–0.60
	26–35 years	6/866	1944.41	**0.31**	0.11–0.67	8/888	2004.17	**0.40**	0.17–0.79
**Analysis period**	**Parity status**								
First year	Nulliparous	2/556	446.88	**0.45**	0.05–1.62	0/574	473.39	**0.00**	0.00–0.78
	Parous	3/876	770.90	**0.39**	0.08–1.14	2/878	779.38	**0.26**	0.03–0.93
3-year	Nulliparous	4/556	1110.63	**0.36**	0.10–0.92	3/574	1205.33	**0.25**	0.05–0.73
	Parous	6/876	1947.99	**0.31**	0.11–0.67	7/878	2006.03	**0.35**	0.14–0.72
**Analysis period**	**BMI category**								
First year	<30 kg/m^2^	4/1187	1009.73	**0.40**	0.11–1.01	1/1198	1038.10	**0.10**	0.00–0.54
	≥30 kg/m^2^	1/244	207.13	**0.48**	0.01–2.69	1/250	211.13	**0.47**	0.01–2.64
3-year	<30 kg/m^2^	9/1187	2547.32	**0.35**	0.16–0.67	6/1198	2664.82	**0.23**	0.08–0.49
	≥30 kg/m^2^	1/244	509.34	**0.20**	0.00–1.09	4/250	538.41	**0.74**	0.20–1.90

* Relevant exposure was calculated from the total exposure minus the time in which back-up contraception was used or sex hormones were taken for other reasons.

BMI, body mass index; CI, confidence interval; WY, woman-year (1 WY = 365 days); LNG-IUS 8; levonorgestrel intrauterine system total content 13.5 mg (average approx. 8 μg/24 hours over the first year); LNG-IUS 13, levonorgestrel intrauterine system total content 19.5 mg (average approx. 13 μg/24 hours over the first year).

**Table 3:** First-year and 3-year Unadjusted Cumulative Kaplan–Meier Failure Rates: Subgroup Analyses by BMI, Age and Parity (Full Analysis Set).

		Unadjusted cumulative Kaplan–Meier estimated failure rate (95% CI)	
		LNG-IUS 8	LNG-IUS 13
**Analysis period**	**Age category**		
First year	18–25 years	**0.002** (0.000–0.013)	**0.002** (0.000–0.018)
	26–35 years	**0.005** (0.002–0.014)	**0.001** (0.000–0.010)
3-year	18–25 years	**0.010** (0.004–0.027)	**0.005** (0.001–0.019)
	26–35 years	**0.008** (0.004–0.019)	**0.012** (0.006–0.025)
**Analysis period**	**Parity status**		
First year	Nulliparous	**0.004** (0.001–0.017)	**0.000** (0.000–0.000)
	Parous	**0.004** (0.001–0.012)	**0.003** (0.001–0.011)
3-year	Nulliparous	**0.010** (0.004–0.026)	**0.008** (0.003–0.026)
	Parous	**0.009** (0.004–0.019)	**0.010** (0.005–0.022)
**Analysis period**	**BMI category**		
First year	<30 kg/m^2^	**0.004** (0.001–0.010)	**0.001** (0.000–0.008)
	≥30 kg/m^2^	**0.005** (0.001–0.034)	**0.005** (0.001–0.037)
3-year	<30 kg/m^2^	**0.010** (0.005–0.019)	**0.007** (0.003–0.016)
	≥30 kg/m^2^	**0.005** (0.001–0.033)	**0.022** (0.008–0.057)

BMI, body mass index; CI, confidence interval; LNG-IUS 8; levonorgestrel intrauterine system total content 13.5 mg (average approx. 8 μg/24 hours over the first year); LNG-IUS 13, levonorgestrel intrauterine system total content 19.5 mg (average approx. 13 μg/24 hours over the first year).

**Fig 1:**
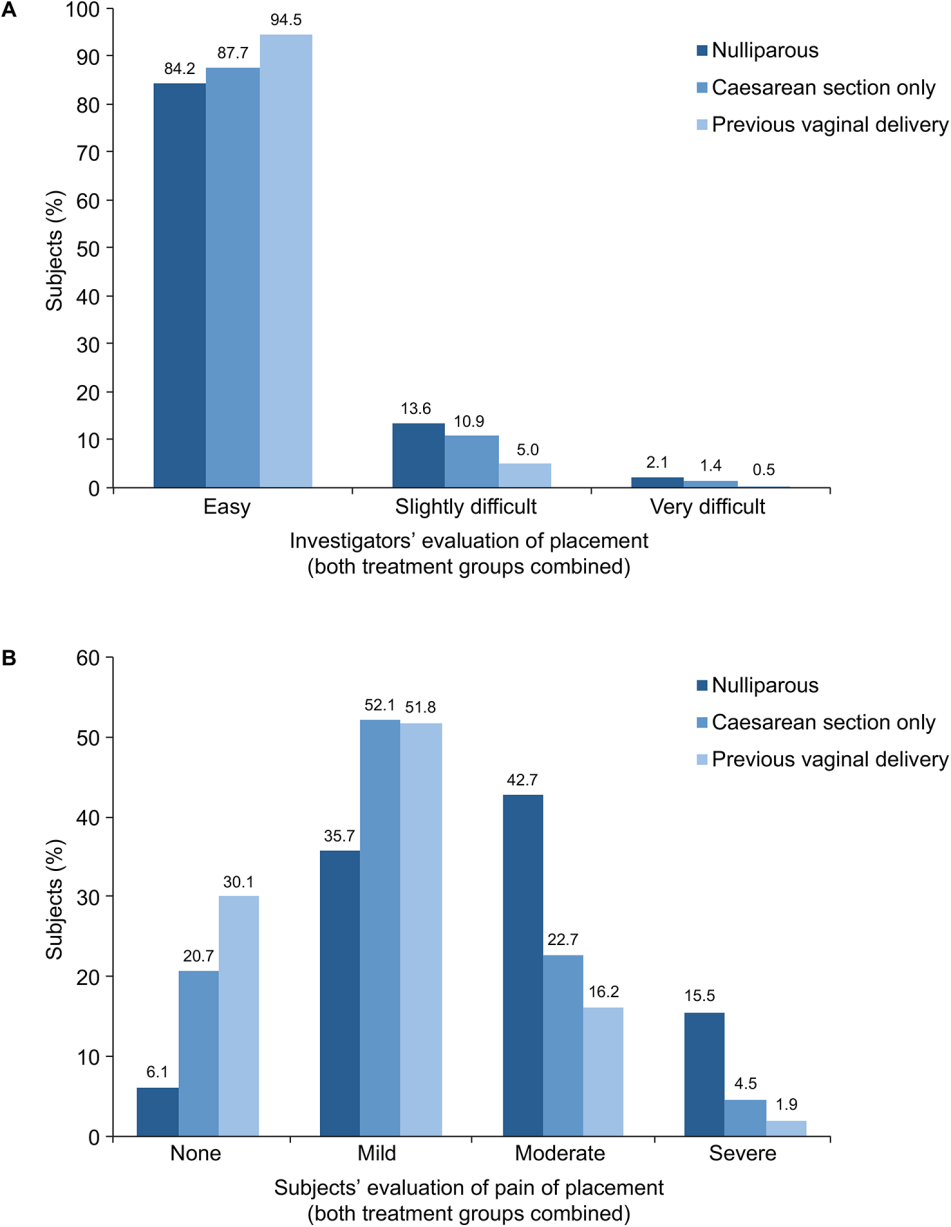
(A) Investigators’ assessment of ease of placement (both treatment groups combined); (B) Subjects’ assessment of pain of placement (both treatment groups combined).

Placement success rates, use of cervical dilation for placement and administration of pain medications for placement, by parity status, are summarized in Table 4 (S2 Data). Ease of placement from the investigator’s perspective is shown in Fig 1A and pain on placement from the woman’s perspective is summarized in Fig 1B. Because LNG-IUS 8 and LNG-IUS 13 had identical T-frame dimensions and were placed using identical placement tubes, these data were analysed for both treatment groups combined.

**Table 4:** Placement Success Rates, Use of Dilation and Administration of Pain Medication on Placement (Both Treatment Groups Combined) by Parity Status (Full Analysis Set).

	Parity status		
	Nulliparous (n = 1130)	Caesarean section delivery only (n = 357)	Previous vaginal delivery (n = 1397)
**Placement successful at first attempt, %**	95.0	96.1	96.9
**Placement successful at second attempt, % (n; subgroup who had a second attempt)**	94.0 (47/50)	92.3 (12/13)	97.7 (42/43)
**Subjects for whom placement was performed without dilation, %**	90.8	93.8	97.6
**Subjects administered local anaesthesia, %**			
Before the procedure	14.5	5.9	4.5
When the procedure proved difficult	0.5	0.0	<0.1
When the procedure proved painful	0.3	0.0	<0.1
**Subjects administered analgesia, %**			
Before the procedure	48.6	24.1	21.0
When the procedure proved difficult	0.4	0.0	0.0
When the procedure proved painful	4.3	0.6	1.1

Placement was successful at the first attempt in at least 95% of women, regardless of parity. Overall, allowing up to two attempts per woman, a successful placement was achieved in 2871 of 2884 women (99.5%). Placement was unsuccessful in a total of 13 women; this included nulliparous and parous women. Placement was performed without cervical dilation in most women (>90%), regardless of parity. Nulliparous women more frequently received local anaesthesia and oral analgesia compared with parous women; however, pain medications were mostly administered prophylactically (i.e. before the procedure), rather than reactively if the procedure proved painful (Table 4).

Placements were generally easier from the investigator’s perspective in women who had previously had a vaginal delivery than in nulliparous women or parous women who had delivered by Caesarean section only (no vaginal deliveries) (p < 0.0001 for overall difference). Nevertheless, placement was rated as ‘easy’ in more than 80% of women who had not had a vaginal delivery (Fig 1A).

Parous women generally reported less placement-related discomfort than nulliparous women (p < 0.0001 for overall difference). Nevertheless, among nulliparous women, 41.8% reported no more than ‘mild’ pain and 84.5% reported no more than ‘moderate’ pain. Women who had previously had a vaginal delivery generally experienced less discomfort compared with nulliparous women and women who had had a Caesarean section delivery only (Fig 1B; p < 0.0001 for overall difference).

In both treatment groups combined, the Kaplan–Meier cumulative probability of complete/partial expulsion was higher in parous women than in nulliparous women (p = 0.0002) (Table 5) and slightly higher in younger women (aged 18–25 years) than in women aged 26–35 years (Table 5), although the difference was not statistically significant (p = 0.07). The crude complete/partial expulsion rates for parous women and for younger women (aged 18–25 years) over up to 3 years were 4.2% and 3.9%, respectively. The risk of expulsion was greatest in the first 12 months after placement, regardless of parity status or age category (Table 5).

**Table 5:** Adverse Events (Both Treatment Groups Combined) According to Parity and Age (Full Analysis Set).

	Parity status			Age category, years	
	Nulliparous (n = 1130)	Parous (n = 1754)	p-value	18–25 (n = 1130)	26–35 (n = 1754)
**Kaplan–Meier estimated cumulative probability of complete or partial expulsion, %**					
At 6 months	0.66	1.77^†^		1.67	1.12
At 12 months	1.70	2.72^†^		2.59	2.15
At 3 years	2.63	4.92^†^		4.78	3.61
**Crude complete or partial expulsion rate over up to 3 years, %**					
Subjects with at least partial expulsion	2.2	4.2	0.004^‡^	3.9	3.1
**Crude rate of PID over up to 3 years, n (%)**					
Subjects with PID	1 (0.1)	11 (0.6)	0.035^†^	2 (0.2)	10 (0.6)
**Crude ectopic pregnancy rate over up to 3 years, n (%)**					
Subjects with ectopic pregnancy	5 (0.4)	5 (0.3)		4 (0.4)	6 (0.3)

Only statistically significant p values (< 0.05) are shown.

^†^ p = 0.0002 for Log rank test, comparison between nulliparous and parous subjects, across all time points.

^‡^ Fisher’s exact test, comparison between nulliparous and parous subjects.

PID, pelvic inflammatory disease.

One partial uterine perforation was reported during the study (a case of myometrial embedment with LNG-IUS 13 in a nulliparous woman); the IUS was removed vaginally.

Over up to 3 years of LNG-IUS 8 or LNG-IUS 13 use, nulliparous women were at no higher risk of pelvic inflammatory disease (PID) than their parous counterparts (crude PID incidence: 0.1% vs 0.6%, respectively) and younger women aged 18–25 years were no more likely to be diagnosed with PID than older women aged 26–35 years (crude incidence: 0.2% vs 0.6%, respectively) (Table 5).

The crude incidence of ectopic pregnancy over up to 3 years of LNG-IUS 8 or LNG-IUS 13 use was 0.3–0.4% across all age and parity subgroups (Table 5). Of the 20 pregnancies that occurred during the study, 10 (50%) were ectopic.

The percentages of women who completed 1 year and 3 years of treatment with LNG-IUS 8 or LNG-IUS 13 were favourable across all age and parity subgroups (Table 6). The Year 1 completion rate was significantly higher among parous women than nulliparous women in both treatment groups (p = 0.043 and p = 0.009 in the LNG-IUS 8 and LNG-IUS 13 groups, respectively). In the LNG-IUS 8 system group, the Year 1 and 3-year completion rates were significantly higher among women aged 26–35 years than among women aged 18–25 years (p = 0.0011 and p = 0.0001, respectively) (Table 6).

**Table 6:** Completion and Discontinuation Rates According to Parity Status and Age.

	Parity			Age category, years		
Treatment group	Nulliparous (n = 1130)	Parous (n = 1754)	p-value	18–25 (n = 1130)	26–35 (n = 1754)	p-value
**Year 1 completion rate, %**						
LNG-IUS 8	78.8	83.1	0.043	77.2	84.2	0.0011
LNG-IUS 13	79.8	85.1	0.009	81.4	84.0	
**3-year completion rate, %**						
LNG-IUS 8	54.3	59.0		50.9	61.3	0.0001
LNG-IUS 13	58.1	61.1		57.9	61.1	
Cumulative 3-year discontinuation rate owing to AEs, %						
LNG-IUS 8	26.1	19.2	0.0025	25.1	19.7	0.0185
LNG-IUS 13	20.6	18.2		20.0	18.6	
Cumulative 3-year discontinuation rate owing to change in menstrual bleeding pattern (including amenorrhoea),* %						
LNG-IUS 8	5.2	4.5		3.4	5.7	
LNG-IUS 13	5.6	4.4		4.4	5.2	
**Cumulative 3-year discontinuation rate for reasons unrelated to AEs,** ^**†**^ **%**						
LNG-IUS 8	19.6	21.7		23.9	18.9	0.0281
LNG-IUS 13	21.3	20.6		21.8	20.3	
**Cumulative 3-year discontinuation rate owing to desire for pregnancy,** ^**‡**^ **%**						
LNG-IUS 8	7.6	8.3		8.5	7.7	
LNG-IUS 13	7.8	8.3		6.9	8.9	

All p values were calculated using Fisher’s exact tests. Only statistically significant p values (p < 0.05) are shown.

* Women who discontinued owing to change in menstrual bleeding pattern (including amenorrhoea) are a subset of those who discontinued owing to AEs.

^†^ Non-AE-related reasons for premature discontinuation included withdrawal of consent, protocol deviation, death, subject was lost to follow-up, pregnancy, desire for pregnancy and ‘other’ reason.

^‡^ Women who discontinued due to desire for pregnancy were a subset of those who discontinued due to non-AE-related reasons.

AE, adverse event; LNG-IUS 8; levonorgestrel intrauterine system total content 13.5 mg (average approx. 8 μg/24 hours over the first year); LNG-IUS 13, levonorgestrel intrauterine system total content 19.5 mg (average approx. 13 μg/24 hours over the first year).

The percentages of women who discontinued study treatment before the end of 3 years for reasons unrelated to AEs were favourable in both treatment groups and across all age and parity subgroups. In the LNG-IUS 8 group, the 3-year discontinuation rate for reasons unrelated to AEs was significantly higher among women aged 18–25 years than in those aged 26–35 years (p = 0.0281) (Table 6). The desire to become pregnant before the end of the 3-year study period was a major reason for premature discontinuation in both treatment groups (Table 6). In both treatment groups, there were slightly more discontinuations owing to AEs among nulliparous women than among parous women (the difference was statistically significant for LNG-IUS 8 only; p = 0.0025) and among women aged 18–25 years than those aged 26–35 years (the difference was statistically significant for LNG-IUS 8 only; p = 0.0185) (Table 6).

Rates of premature discontinuation (before 3 years) owing to change in menstrual bleeding pattern (including development of amenorrhoea) were approximately 5% in both treatment groups, with no significant differences across the age and parity subgroups (range 3.4–5.7%) (Table 6). In total, 2 subjects reported that they discontinued due to amenorrhoea.

Overall, 2116 of 2884 women in the full analysis set completed a user satisfaction questionnaire either at the end of 3 years (those who completed the study) or at their last study visit (those who discontinued prematurely): 802 and 1314 were nulliparous and parous, respectively; 784 and 1332 were 18–25 and 26–35 years of age, respectively. Across all age and parity subgroups, >90% of women were either ‘very satisfied’ or ‘somewhat satisfied’ with LNG-IUS 8 and LNG-IUS 13, >70% of women were either ‘very satisfied’ or ‘somewhat satisfied’ with their menstrual bleeding pattern during use of LNG-IUS 8 and LNG-IUS 13, and >70% of women reported they would prefer to continue their use of study treatment after the study (Table 7).

**Table 7:** User Satisfaction According to Parity Status and Age.

	Parity		Age category, years	
	Nulliparous (n = 802)	Parous (n = 1314)	18–25 (n = 784)	26–35 (n = 1332)
**User ‘very satisfied’ or ‘somewhat satisfied’ with study treatment, %**				
LNG-IUS 8	94.2	95.0	96.4	93.6
LNG-IUS 13	95.8	95.9	96.4	95.5
User ‘very satisfied’ or ‘somewhat satisfied’ with their menstrual bleeding pattern,* %				
LNG-IUS 8	72.8	79.0	74.3	78.1
LNG-IUS 13	71.4	79.0	76.0	76.1
**User preference to use study treatment after the study, %**				
LNG-IUS 8	73.3	79.3	74.5	78.5
LNG-IUS 13	79.8	83.4	78.5	84.1

* For women who did not experience bleeding while on treatment, their satisfaction with their bleeding pattern was recorded as ‘not applicable’.

LNG-IUS 8; levonorgestrel intrauterine system total content 13.5 mg (average approx. 8 μg/24 hours over the first year); LNG-IUS 13, levonorgestrel intrauterine system total content 19.5 mg (average approx. 13 μg/24 hours over the first year).

## Discussion

The *post hoc* subgroup analyses reported here confirm that LNG-IUS 8 and LNG-IUS 13 were similarly effective regardless of the age, parity status or BMI of the user, demonstrated by first year and 3-year Pearl Indices and Kaplan–Meier cumulative failure rates for the subgroups that had widely overlapping 95% CIs. However, a limitation of the analysis according to BMI was that there were relatively few women (n = 494) in the higher BMI (≥30 kg/m^2^) subgroup, hence the wide 95% CIs for the first year and 3-year Pearl Indices and cumulative failure rates for this subgroup.

There is the perception among some healthcare providers that IUC is much more difficult to place in nulliparous women than in women who had already had a child [10]. However, parous women who have had elective Caesarean section deliveries only (and not laboured at all) may be considered to be similar to nulliparous women with regard to the tightness of their cervical canals. Investigators rated the placement of LNG-IUS 8 and LNG-IUS 13 as ‘easy’ in most women, regardless of parity. However, placement was rated by investigators as ‘easy’ in a higher percentage of women who had previously had a vaginal delivery (94.5%) compared with those who had had Caesarean section deliveries only (87.7%; it was not known when during the course of pregnancy or labour Caesarean section deliveries were performed) or nulliparous women (84.2%). However, a placement that is rated as ‘easy’ the investigator is not necessarily considered ‘easy’ or ‘pain-free’ by the woman. Investigators rated placement as ‘very difficult’ in only 2.1% of nulliparous women and 1.4% of women who had had Caesarean section deliveries only.

In total, 41.8% and 84.5% of nulliparous women reported their pain on placement as no more than ‘mild’ and no more than ‘moderate’, respectively. However, parous women generally reported less placement-related discomfort than their nulliparous counterparts and, among parous women, those who had had a vaginal delivery generally reported less discomfort than those who had had Caesarean section deliveries only: no more than ‘mild’ pain was reported by 41.8% of nulliparous women, 72.8% of parous women who had had Caesarean section deliveries only, and 81.9% of parous women who had previously had a vaginal delivery.

The analysis of placement-related pain did not take into account that more nulliparous women received pre-placement local anaesthesia (14.5%) and analgesia (48.6%) compared with the parous subgroups. However, these interventions are readily available and commonly used in routine medical practice [11]. In addition, the reporting of ease of placement and pain of placement was subjective; investigators may have varied in what they considered an ‘easy’ placement and women may have varied in what they considered to be ‘mild’, ‘moderate’ or ‘severe’ pain.

Placement was performed without cervical dilation in 90.8% of nulliparous women, 93.8% of women who had had Caesarean section deliveries only and 97.6% of women who had previously had a vaginal delivery. In a retrospective study evaluating ease of placement and clinical performance of LNG-IUS 20 (Mirena) in nulligravid women, a similar proportion of nulligravid women (92.3%) underwent placement without cervical dilation [12].

The incidences of at least partial expulsion, uterine perforation, PID and ectopic pregnancy were low across all age and parity subgroups. Concern exists in the literature regarding an increased risk of expulsion in nulliparous women [13]. However, the results from this study reinforce evidence from other studies showing that nulliparous women are at no higher risk of expulsion than parous women [14,15].

Younger women are generally considered to be at higher risk of contracting sexually transmitted infections compared with older women and therefore might be expected to have a higher risk of developing PID, particularly in this study, in which condom use was not encouraged. However, no difference in the incidence of PID between the older and younger women was observed.

The absolute incidence of ectopic pregnancy was low across all age and parity subgroups in both treatment arms. Furthermore, the absolute incidence of ectopic pregnancy for the LNG-IUS 8 and LNG-IUS 13 groups combined (0.13–0.22 per 100 woman-years across all age and parity subgroups) was within the reported range for oral contraceptives (absolute incidence: 0.07–1.99 per 100 woman-years for combined oral contraceptives; 0.3–2.0 per 100 woman-years for progestogen-only pills) [16].

One-year and 3-year completion rates were favourable for both LNG-IUS 8 and LNG-IUS 13 regardless of age or parity, reflecting high levels of satisfaction with study treatment across all subgroups. The 3-year completion rates for LNG-IUS 8 and LNG-IUS 13 also compare favourably with 3-year continuation rates for combined oral contraceptives. The number of women who prematurely discontinued owing to a desire for pregnancy was similar across all age and parity subgroups. Importantly, few women discontinued because of changes in menstrual bleeding patterns, regardless of age or parity. The percentages of women who discontinued because of AEs were favourable in both treatment groups and across all age and parity subgroups. However, in the LNG-IUS 8 group, significantly more nulliparous women than parous women and significantly more women aged 18–25 years than women aged 26–35 years discontinued because of AEs, whereas no significant difference by parity or age was observed in the LNG-IUS 13 group. The reason for this dose difference is unclear; however, it could be a chance finding between non-randomized parity and age subgroups.

Overall, these subgroup analyses demonstrate that LNG-IUS 8 and LNG-IUS 13 are highly effective, safe, well tolerated and associated with high levels of user satisfaction and favourable continuation rates, regardless of age or parity. These data refute the misconceptions held by some healthcare providers that IUC is not suitable for younger women or nulliparous women and add to the scientific evidence supporting the more widespread use of IUC among these groups.

## Supporting Information

S1 CONSORT Checklist CONSORT Checklist. (DOC)

S1 Data Demographics, Efficacy and Safety. (PDF)

S2 Data *Post Hoc* Analyses of Discontinuations, Insertion and User Evaluation Outcomes. (PDF)

S1 Protocol Study Protocol. (PDF)
