# Supplementary material for: The Effect of Age, Parity and Body Mass Index on the Efficacy, Safety, Placement and User Satisfaction Associated With Two Low-Dose Levonorgestrel Intrauterine Contraceptive Systems: Subgroup Analyses of Data From a Phase III Trial
Source: PLoS One. 2015 Sep 17;10(9):e0135309. doi: 10.1371/journal.pone.0135309 (PMC4574776; doi:10.1371/journal.pone.0135309)
Supplement: S1 Data — (PDF) [file pone.0135309.s002.pdf]

14. Tables and figures referred to but not included in the text

Table of contents

14. Tables and figures referred to but not included in the text ... 1
14.1 Demographics ... 2
14.1.1 Disposition and baseline characteristics ... 3
14.1.2 Study drug administration and compliance ... 145
14.1.3 Exposure ... 205
14.2 Efficacy ... 207
14.2.1 Contraceptive efficacy ... 208
14.2.2 Bleeding pattern (not included) ... 276
14.2.3 Other efficacy variables ... 277
14.3 Safety ... 280
14.3.1 Analyses of adverse events ... 281
14.3.2 Listings of deaths, other serious and significant adverse events ... 784
14.3.3 Pregnancies (see efficacy) ... 823

14.1 Demographics

14.1.1 Disposition and baseline characteristics

Table 14.1.1 / 2: Subject validity and primary reasons for exclusion from analysis (all subjects randomized)

	LCS12	LCS16	Total
Subjects randomized	1432 (100.0%)	1453 (100.0%)	2885 (100.0%)
Subjects valid for FAS	1432 (100.0%)	1452 (>99.9%)	2884 (>99.9%)
Subjects enrolled to subset 1	20 (1.4%)	19 (1.3%)	39 (1.4%)
Subjects enrolled to subset 2	31 (2.2%)	30 (2.1%)	61 (2.1%)
Subjects enrolled to subset 3	12 (0.8%)	11 (0.8%)	23 (0.8%)
Subjects valid for PKS	7 (0.5%)	6 (0.4%)	13 (0.5%)
Subjects enrolled to subset 4	102 (7.1%)	103 (7.1%)	205 (7.1%)
Excluded from FAS	0	1 (<0.1%)	1 (<0.1%)
No insertion attempts	0	1 (<0.1%)	1 (<0.1%)
Excluded from PKS	5 (0.3%)	5 (0.3%)	10 (0.3%)
no 3 year sample	4 (0.3%)	5 (0.3%)	9 (0.3%)
no sample around Tmax	1 (<0.1%)	0	1 (<0.1%)

Note: FAS = full analysis set, PKS = pharmacokinetic analysis set
Global Biostatistics: /by-sasp/patdb/projects/de04209/310442/stat/prod_interim03/pgms/t-ds2.sas epkll 07OCT2011 10:47
End of table

Table 14.1.1 / 3: Disposition: End of screening (all screened subjects)

	Total (N=3661)
Screened	3661 (100.0%)
Ongoing	2885 (78.8%)
prem. discontinued	776 (21.2%)
Premature termination	776 (21.2%)
Primary reason	
withdrawal of consent	164 (4.5%)
in-/ exclusion criteria not met	401 (11.0%)
pat. lost, no further information avail.	85 (2.3%)
pregnancy	44 (1.2%)
other	82 (2.2%)

Global Biostatistics: /by-sasp/patdb/projects/de04209/310442/stat/prod_interim03/pgms/t-ds3-1.sas epkll 07OCT2011 10:47
End of table

Table 14.1.1 / 4: Disposition: End of study (all randomized subjects)

	LCS12 (N=1432)	LCS16 (N=1453)	Total (N=2885)
Randomized	1432 (100.0%)	1453 (100.0%)	2885 (100.0%)
Ongoing to extension study	0	707 (48.7%)	707 (24.5%)
Completed	819 (57.2%)	163 (11.2%)	982 (34.0%)
prem. discontinued	613 (42.8%)	583 (40.1%)	1196 (41.5%)
Premature termination	613 (42.8%)	583 (40.1%)	1196 (41.5%)
Primary reason			
adverse event	311 (21.7%)	277 (19.1%)	588 (20.4%)
death	0	1 (<0.1%)	1 (<0.1%)
other	189 (13.2%)	188 (12.9%)	377 (13.1%)
pat. lost, no further information avail.	63 (4.4%)	61 (4.2%)	124 (4.3%)
pregnancy	9 (0.6%)	10 (0.7%)	19 (0.7%)
protocol deviation	16 (1.1%)	15 (1.0%)	31 (1.1%)
withdrawal of consent	25 (1.7%)	31 (2.1%)	56 (1.9%)

Global Biostatistics: /by-sasp/patdb/projects/de04209/310442/stat/prod_interim03/pgms/t-ds3-1.sas epkll 07OCT2011 10:47
End of table

Table 14.1.1 / 5: Number of subjects who prematurely discontinued the study medication (including main reason) after randomization by treatment and year (all randomized subjects)

YEAR AFTER INSERTION: 1st year

	LCS12 (N=1432)	LCS16 (N=1453)	Total (N=2885)
Study medication, administration status			
n	1432 (100.0%)	1453 (100.0%)	2885 (100.0%)
study medication never administered	0	2 (0.1%)	2 (<0.1%)
completed	0	0	0
prematurely discontinued	266 (18.6%)	245 (16.9%)	511 (17.7%)
ongoing	1166 (81.4%)	1206 (83.0%)	2372 (82.2%)
missing	0	0	0
Premature EOSM or never taken, reason			
n	266 (100.0%)	247 (100.0%)	513 (100.0%)
withdrawal of consent	11 (4.1%)	9 (3.6%)	20 (3.9%)
protocol deviation	3 (1.1%)	0	3 (0.6%)
adverse event	175 (65.8%)	168 (68.0%)	343 (66.9%)
death	0	0	0
pat. lost, no further information avail.	25 (9.4%)	21 (8.5%)	46 (9.0%)
pregnancy	5 (1.9%)	2 (0.8%)	7 (1.4%)
other	47 (17.7%)	47 (19.0%)	94 (18.3%)

Note: Year is calculated using the formula year = (last day on study (imputed)- insertion date)/365
Global Biostatistics: /by-sasp/patdb/projects/de04209/310442/stat/prod_interim03/pgms/t-ds3-2.sas epkll 07OCT2011 10:47

Table 14.1.1 / 5: Number of subjects who prematurely discontinued the study medication (including main reason) after randomization by treatment and year (all randomized subjects) (cont.)

YEAR AFTER INSERTION: 2nd year

	LCS12 (N=1432)	LCS16 (N=1453)	Total (N=2885)
Study medication, administration status			
n	1166 (100.0%)	1206 (100.0%)	2372 (100.0%)
study medication never administered	0	0	0
completed	0	0	0
prematurely discontinued	203 (17.4%)	194 (16.1%)	397 (16.7%)
ongoing	963 (82.6%)	1012 (83.9%)	1975 (83.3%)
missing	0	0	0
Premature EOSM or never taken, reason			
n	203 (100.0%)	194 (100.0%)	397 (100.0%)
withdrawal of consent	9 (4.4%)	14 (7.2%)	23 (5.8%)
protocol deviation	5 (2.5%)	8 (4.1%)	13 (3.3%)
adverse event	85 (41.9%)	66 (34.0%)	151 (38.0%)
death	0	0	0
pat. lost, no further information avail.	23 (11.3%)	21 (10.8%)	44 (11.1%)
pregnancy	3 (1.5%)	3 (1.5%)	6 (1.5%)
other	78 (38.4%)	82 (42.3%)	160 (40.3%)

Note: Year is calculated using the formula year = (last day on study (imputed)- insertion date)/365
Global Biostatistics: /by-sasp/patdb/projects/de04209/310442/stat/prod_interim03/pgms/t-ds3-2.sas epkll 07OCT2011 10:47

Table 14.1.1 / 5: Number of subjects who prematurely discontinued the study medication (including main reason) after randomization by treatment and year (all randomized subjects) (cont.)

YEAR AFTER INSERTION: 3rd year

	LCS12 (N=1432)	LCS16 (N=1453)	Total (N=2885)
Study medication, administration status			
n	963 (100.0%)	1012 (100.0%)	1975 (100.0%)
study medication never administered	0	0	0
completed	819 (85.0%)	163 (16.1%)	982 (49.7%)
prematurely discontinued	143 (14.8%)	142 (14.0%)	285 (14.4%)
ongoing	0	707 (69.9%)	707 (35.8%)
missing	1 (0.1%)	0	1 (<0.1%)
Premature EOSM or never taken, reason			
n	144 (100.0%)	142 (100.0%)	286 (100.0%)
withdrawal of consent	6 (4.2%)	8 (5.6%)	14 (4.9%)
protocol deviation	8 (5.6%)	8 (5.6%)	16 (5.6%)
adverse event	53 (36.8%)	44 (31.0%)	97 (33.9%)
death	0	1 (0.7%)	1 (0.3%)
pat. lost, no further information avail.	15 (10.4%)	19 (13.4%)	34 (11.9%)
pregnancy	1 (0.7%)	5 (3.5%)	6 (2.1%)
other	61 (42.4%)	57 (40.1%)	118 (41.3%)

Note: Year is calculated using the formula year = (last day on study (imputed)- insertion date)/365
Global Biostatistics: /by-sasp/patdb/projects/de04209/310442/stat/prod_interim03/pgms/t-ds3-2.sas epkll 07OCT2011 10:47

Table 14.1.1 / 5: Number of subjects who prematurely discontinued the study medication (including main reason) after randomization by treatment and year (all randomized subjects) (cont.)

YEAR AFTER INSERTION: Overall

	LCS12 (N=1432)	LCS16 (N=1453)	Total (N=2885)
Study medication, administration status			
n	1432 (100.0%)	1453 (100.0%)	2885 (100.0%)
study medication never administered	0	2 (0.1%)	2 (<0.1%)
completed	819 (57.2%)	163 (11.2%)	982 (34.0%)
prematurely discontinued	612 (42.7%)	581 (40.0%)	1193 (41.4%)
ongoing	0	707 (48.7%)	707 (24.5%)
missing	1 (<0.1%)	0	1 (<0.1%)
Premature EOSM or never taken, reason			
n	613 (100.0%)	583 (100.0%)	1196 (100.0%)
withdrawal of consent	26 (4.2%)	31 (5.3%)	57 (4.8%)
protocol deviation	16 (2.6%)	16 (2.7%)	32 (2.7%)
adverse event	313 (51.1%)	278 (47.7%)	591 (49.4%)
death	0	1 (0.2%)	1 (<0.1%)
pat. lost, no further information avail.	63 (10.3%)	61 (10.5%)	124 (10.4%)
pregnancy	9 (1.5%)	10 (1.7%)	19 (1.6%)
other	186 (30.3%)	186 (31.9%)	372 (31.1%)

Note: Year is calculated using the formula year = (last day on study (imputed)- insertion date)/365
Global Biostatistics: /by-sasp/patdb/projects/de04209/310442/stat/prod_interim03/pgms/t-ds3-2.sas epkll 07OCT2011 10:47
End of table

Table 14.1.1 / 6: Listing of subjects who prematurely discontinued study medication with reason 'other' by treatment (all randomized subjects)

TREATMENT: LCS12

Subject number	Premature EOSM or never taken, reason	Premature EOSM other reason
120109	other	WISH OF PREGNANCY
120212	other	WISH OF PREGNANCY.
120403	other	THE PATIENT WILL TRAVELED TO OTHER COUNTRY
120409	other	WISH FOR PREGNANCY
120412	other	WISH OF PREGNANCY
120414	other	WISH FOR PREGNANCY
120608	other	WISH TO BE PREGNANT.
140212	other	WISH FOR PREGNANCY
140501	other	INSERTION FAILURE
140701	other	PT. NON - COMPLIANT WITH DIARIES
140711	other	PT DID NOT WANT IUD ANYMORE.
140921	other	WANTS TO GET PREGNANT.
141017	other	'WISH FOR PREGNANCY'
141201	other	PARTNER UNCOMFORTABLE/SAME-SEX RELATIONSHIP, LAST HETEROSEXUAL RELATIONSHIP ENDED 31/OCT/2009
141301	other	NO LONGER NEEDS CONTRACEPTION
150106	other	WISH OF PREGNANCY
150110	other	DESIRE TO BECOME PREGNANT
150156	other	SHE WILL MOVE OUT OF THE COUNTRY FOR TWO YEARS.
150506	other	IUS WAS ACCIDENTALY REMOVED IN OTHER CLINIC CENTRE
160208	other	HOPE TO GET PREGNANT
160306	other	NO SEXUAL RELATION
160321	other	WISH OF PREGNANCY
160431	other	WISH TO GET PREGNANT
160514	other	WISH TO HAVE A BABY
160524	other	WISH TO HAVE A BABY
160550	other	NO NEED TO CONTRACEPTION
160557	other	WANT TO BE PREGNANT
160564	other	WANTS PREGNANCY
160566	other	SHE IS LIVING IN INDIA AND IS COMING TO FINLAND IN SUMMER 2011
160567	other	FEAR FOR HORMONES
160573	other	WANTS PREGNANCY
160574	other	GOING ABROAD (AUSTRALIA) FOR ONE YEAR.
160704	other	WISH TO BE PREGNANT
160721	other	WANTS TO GET PREGNANT
160725	other	WISH FOR PREGNANCY
160736	other	WANTS TO GET PREGNANT
160801	other	WISH OF PREGNANCY
160904	other	WISH FOR PREGNANCY
160982	other	WISH FOR PREGNANCY
161003	other	MOVING ABROAD
161106	other	WISH FOR PREGNANCY
161110	other	WISH FOR PREGNANCY

Table 14.1.1 / 6: Listing of subjects who prematurely discontinued study medication with reason 'other' by treatment (all randomized subjects)

TREATMENT: LCS12

Subject number	Premature EOSM or never taken, reason	Premature EOSM other reason
161112	other	EMIGRATE TO USA
161210	other	LCS ACCIDENTALLY REMOVED
161407	other	WISH FOR PREGNANCY
161435	other	WISH FOR PREGNANCY
161437	other	WISH TO GET PREGNANT
161511	other	WISH OF PREGNANCY
161514	other	PREGNANCY WISH
170101	other	WISHES FOR PREGNANCY
170302	other	WISHES FOR PREGNANCY
170410	other	WISH OF PREGNANCY
170414	other	WISH OF PREGNANCY
170801	other	WISHES FOR PREGNANCY
180307	other	WISH OF PREGNANCY
180617	other	WISH OF PREGNANCY
180658	other	WISH FOR PREGNANCY
180662	other	PREGNANCY WISH
180813	other	WISH OF PREGNANCY
190110	other	PATIENT WANTS TO GET PREGNANT
190411	other	SHE WANTS TO GET PREGNANT
190412	other	THE PATIENT WISHES TO GET PREGNANT.
190602	other	THE PATIENT WANTS TO GET PREGNANT.
200104	other	UNSATISFIED WITH CYCLIC BLEEDINGS
200106	other	FUTURE WISH PREGNANCY STARTING FROM AUG'09
200201	other	WANTS TO BE PREGNANT
200205	other	WISHES TO BE PREGNANT.
200303	other	WISH FOR PREGNANCY
200309	other	WANTS TO GET PREGNANT
200501	other	PREGNANCY WISH
200503	other	PREGNANCY WISH
200612	other	WISH FOR PREGNANCY.
200614	other	WANTS A BABY
200629	other	WISH FOR PREGNANCY.
200634	other	WANTS TO BECOME PREGNANT.
200803	other	MOVING TO OTHER COUNTRY
200804	other	WISH FOR CHILDREN
200904	other	EMIGRATION
200910	other	WISH FOR PREGNANCY
210125	other	PREGNANCY WISH
210127	other	SHE WANTS TO GET PREGNANT
210211	other	THE PATIENT WANTS / WISHES PREGNANCY
210404	other	MOVED TO NORTHERN NORWAY (1000KM. NORTH OF STUDY PLACE
210409	other	PREGNANCY WISH
210514	other	SHE WANT TO BE PREGNANT

Table 14.1.1 / 6: Listing of subjects who prematurely discontinued study medication with reason 'other' by treatment (all randomized subjects)

TREATMENT: LCS12

Subject number	Premature EOSM or never taken, reason	Premature EOSM other reason
230103	other	WISH PREGNANCY
230107	other	WISH FOR PREGNANCY
230115	other	BREAST CANCER EVENT WITHIN HER FAMILY
230208	other	WISH FOR PREGNANCY
230304	other	WISH TO GET PREGNANT
230401	other	PLANNING PREGNANCY
230408	other	PLANNING PREGNANCY
230512	other	WISH TO BECOME PREGNANT
230824	other	WISH OF PREGNANCY.
231112	other	NO SEXUAL ACTIVITY, NO PARTNER
240205	other	RELOCATION
240227	other	PATIENT NO LONGER WANTED IUS
240302	other	DESIRE TO BECOME PREGNANT
240310	other	DESIRE TO GET PREGNANT
240316	other	MOVE OUT OF STATE
240503	other	SUBJECT MOVING OUT OF AREA.
240505	other	DESIRES TO BECOME PREGNANT IN 4 TO 5 MONTHS
240521	other	SUBJECT REQUEST DUE TO UPCOMING MAJOR SURGERY (COLECTOMY)
240712	other	DESIRES PREGNANCY
240721	other	SUBJECT TO HAVE ESSURE AND NOVASURE PROCEDURES
240820	other	PATIENT MOVING
240838	other	MOVING
241011	other	WISH FOR PREGNANCY
241014	other	PARTNER COULD FEEL STRING
241018	other	FAILED IUD INSERTION. NO SECOND ATTEMPT.
241019	other	DESIRES TO CONCEIVE
241024	other	INTERNAL OS STENOTIC, COULD NOT PASS SOUND, TRIED TO DILATE WITHOUT SUCCESS
241033	other	PARTNER BOTHERED BY STRINGS.
241105	other	WISH FOR PREGNANCY
241108	other	DESIRES TO CONCEIVE
241110	other	WANTS TO CONCEIVE
241115	other	FAILED INSERTION
241120	other	WISH FOR PREGNANCY
241148	other	WISH FOR PREGNANCY
241162	other	WISH FOR PREGNANCY
241167	other	WISH FOR PREGNANCY
241174	other	MOVING OUT OF STATE & HUSBAND HAVING VASECTOMY.
241175	other	"WISH FOR PREGNANCY"
241190	other	MOVING OUT OF AREA
241193	other	SEEKING PREGNANCY
241202	other	SUBJECT WANTS TO TRY A DIFFERENT BIRTH CONTROL
241209	other	MOVING OUT OF THE COUNTRY.
241210	other	PREGNANCY WISH

Table 14.1.1 / 6: Listing of subjects who prematurely discontinued study medication with reason 'other' by treatment (all randomized subjects)

TREATMENT: LCS12

Subject number	Premature EOSM or never taken, reason	Premature EOSM other reason
241231	other	MOVING
241307	other	WISH FOR PREGNANCY
241405	other	PT. STATES STRINGS BOTHER HER PARTNER
241406	other	PT. HUSBAND HAD VASECTOMY.
241416	other	PATIENT DESIRES PREGNANCY
241428	other	IUS CAME OUT IMMEDIATELY AFTER INSERTION
241532	other	ACCIDENTALLY REMOVED WHEN CUTTING STRING
241541	other	RELOCATING
241545	other	PATIENT MOVING
241547	other	IUD NOT ALLOWED IN MILITARY SUBJECT JOINING MILITARY
241553	other	PLANNING FUTURE PREGNANCY
241605	other	SUBJECT REQUESTING CONTRACEPTION WITHOUT HORMONES
241805	other	SUBJECT NOT COMPLIANT WITH VISIT SCHEDULE
242203	other	PT WANTS TO GET PREGNANT
242213	other	PT COULD NO LONGER PARTICIPATE DUE TO WORK SCHEDULE
242708	other	SUBJECT MOVING OUT OF STATE
242816	other	PREGNANCYWISH
242907	other	MOVING TO ANOTHER COUNTRY
242910	other	MOVED AWAY
243005	other	DESIRES CONCEPTION
243009	other	DESIRES PREGNANCY
243106	other	IUS APPEARS TO BE TRANSVERSE LIE IN FUNDUS
243207	other	WISH FOR PREGNANCY
243309	other	PATIENT MOVING OUT OF STATE
243314	other	SUBJECT DESIRES PREGNANCY
243322	other	SUBJECT DESIRES PREGNANCY
243611	other	PATIENT MOVING OUT OF STATE
243618	other	PT. WISHES TO BECOME PREGNANT
243946	other	PATIENT IS MOVING TO FLORIDA AND WILL NOT RETURN WITHIN WINDOW TO GET IUD OUT
243965	other	PATIENT IS HAVING A BILATERAL TUBAL LIGATION
243968	other	NON COMPLIANCE
243973	other	HUSBAND FELT STRINGS OF IUD
244008	other	INSERTION FAILURE
244125	other	PATIENT MOVING OUT OF TOWN.
244302	other	PT MOVED OUT OF STATE
244306	other	SUBJECT MOVING OUT OF STATE
244407	other	SUBJECT TRAVELLING OUT OF THE COUNTRY
244426	other	SUBJECTWANTING TO CONCIEVE
244442	other	DESIRES PREGNANCY.
244604	other	PT. DESIRES CONCEPTION
244608	other	DESIRE TO CONCEIVE
244610	other	PT STATED SHE DIDN'T WANT TO HAVE TO WORRY ABOUT IUD ANYMORE.
245004	other	PT. MOVED OUT OF STATE

Table 14.1.1 / 6: Listing of subjects who prematurely discontinued study medication with reason 'other' by treatment (all randomized subjects)

TREATMENT: LCS12

Subject number	Premature EOSM or never taken, reason	Premature EOSM other reason
245007	other	DESIRE FOR PREGNANCY
245021	other	BASELINE FINDING OF "VAGINAL BLEEDING"
245022	other	DESIRE FOR PREGNANCY.
245026	other	DESIRE FOR PREGNANCY
245028	other	PT. MOVING OUT OF STATE
245032	other	DESIRE FOR PREGNANCY.
245431	other	DESIRES CONCEPTION
245439	other	DESIRES PREGNANCY
245525	other	MOVING OUT OF STATE.
245603	other	SUBJECT WISHES TO BECOME PREGNANT
245904	other	DESIRES PREGNANCY
245910	other	DESIRES PREGNANCY
246150	other	MOVING TO ANOTHER STATE
246210	other	DESIRES TO CONCEIVE
246211	other	"WISH FOR PREGNANCY"

Table 14.1.1 / 6: Listing of subjects who prematurely discontinued study medication with reason 'other' by treatment (all randomized subjects) (cont.)

TREATMENT: LCS16

Subject number	Premature EOSM or never taken, reason	Premature EOSM other reason
120334	other	WISH OF PREGNANCY
120406	other	WISH OF PREGNANCY
120408	other	WISH OF PREGNANCY
120424	other	WISH OF PREGNANCY
140120	other	DESIRE PREGNANCY
140211	other	DESIRE OF CHILD BEARING
140603	other	MOVING ABROAD
140710	other	PT. WISHES TO GET PREGNANT
140712	other	PT MOVING OUT OF PROVINCE
140906	other	HUSBAND HAD VASECTOMY
141002	other	WANTS TO BE PREGNANT.
141012	other	PATIENT WISHES PREGNANCY.
141202	other	SUBJECT PARTNER UNCOMFORTABLE WITH DEVICE.
150113	other	MOVING OUT OF THE CITY.
150122	other	THE IUS WAS REMOVED BY HER GYNECOLOGIST ON APRIL 20, 2010 BECAUSE HER HUSBAND MADE A VASECTOMY ON MARCH 15, 2010
150147	other	INSERTION FAILURE (2) WAS NOT POSSIBLE TO PASS THROUGH CERVIX
160115	other	WISH OF PREGNANCY
160123	other	WANTS TO GET PREGNANT
160204	other	UNABLE TO CONTINUE BECAUSE OF HER JOB
160211	other	HOPE TO GET PREGNANT
160327	other	WISH OF PREGNANCY
160330	other	WISH OF PREGNANCY
160412	other	WANTS GET PREGNANT
160429	other	WISH FOR PREGNANCY
160435	other	WISH FOR PREGNANCY.
160510	other	WANT TO HAVE A BABY
160521	other	PREGNANCY WISH
160527	other	WANTS TO GET PREGNANT
160530	other	WANTS PREGNANCY
160560	other	WANTS PREGNANCY
160620	other	WISH TO BECOME PREGNANT
160707	other	WANTS TO GET PREGNANT
160715	other	WANTS TO GET PREGNANT
160808	other	PREGNANCY WISH
160915	other	WISH FOR PREGNANCY
160941	other	HOPE FOR PREGNANCY
160945	other	WISH FOR PREGNANCY
161016	other	PREGNANCY IS DESIRED
161022	other	WISH OF PREGNANCY
161113	other	HOPE OF PREGNANCY
161307	other	WISH OF PREGNANCY
161412	other	WISH FOR PREGNANCY

Table 14.1.1 / 6: Listing of subjects who prematurely discontinued study medication with reason 'other' by treatment (all randomized subjects) (cont.)

TREATMENT: LCS16

Subject number	Premature EOSM or never taken, reason	Premature EOSM other reason
161427	other	WISH FOR PREGNANCY
161434	other	PATIENT MOVED AWAY
161507	other	WISH OF PREGNANCY
161528	other	PREGNANCY WISHES
161535	other	WISH OF PREGNANCY
170401	other	PREGNANCY WISH
170501	other	THE PATIENT WISHES FOR PREGNANCY NOW
170604	other	WISH OF PREGNANCY
180218	other	WISH OF PREGNANCY
180333	other	WISH OF PREGNANCY
180413	other	PLANNING OF PREGNANCY
180526	other	WISH OF PREGNANCY
180639	other	WISH FOR PREGNANCY
180678	other	WANTS TO BE PREGNANT
180688	other	PREGNANCY WISH
180802	other	PATIENT'S PARTNER FEELS DISCOMFORTABLE THREAD
180819	other	PATIENT WANT TO BE PREGNANT
190102	other	SHE WENT TO LIVE IN ANOTHER CITY
190113	other	SHE LIKES TO PREGNANT.
190122	other	WISH FOR PREGNANCY
190127	other	WANTS TO GET PREGNANT
190129	other	DESIRE OF PREGNANCY
190631	other	WISH TO GET PREGNANT
200107	other	PREGNANCY WISH
200113	other	PREGNANCY WISH
200114	other	PREGNANCY WISH
200212	other	MOVEMENT ABROAD.
200218	other	WOULD LIKE TO GET PREGNANT
200220	other	WHISH FOR PREGNANCY
200225	other	WISH TO BECOME PREGNANT
200305	other	WISH FOR PREGNANCY
200407	other	WISH TO CONCEIVE.
200508	other	CHILD WISH
200622	other	WANTS TO BECOME PREGNANT.
200907	other	WISH FOR PREGNANCY.
210204	other	THE WOMAN WISHES TO BE PREGNANT
210523	other	PREGNANCY DESIRE
210604	other	IUD EXTRACTED ACCIDENTALLY DURING BIOPSY
230113	other	INSERTION FAILURED
230118	other	PAT UNABLE TO DO SCHEDULED VISITS DUE TO TRAVELLING
230306	other	WISH TO GET PREGNANT
230404	other	PATIENT REQUEST IS MOVING TO NEW YORK
230407	other	INSERTION FAILURE

Table 14.1.1 / 6: Listing of subjects who prematurely discontinued study medication with reason 'other' by treatment (all randomized subjects) (cont.)

TREATMENT: LCS16

Subject number	Premature EOSM or never taken, reason	Premature EOSM other reason
230601	other	PATIENT MOVED TO LONDON
230708	other	PREGNANCY WISH
230915	other	PATIENT WISHES TO PREGNANT
230916	other	NO NEED OF CONTRACEPTION
231015	other	PREGNANCY WISH
231021	other	WISH FOR PREGNANCY
231109	other	PATIENT WANTS TO GET PREGNANT.
231110	other	WISH TO GET PREGNANT
240128	other	IUS STRING POKING PARTNER
240212	other	DESIRE FOR PREGNANCY
240343	other	DESIRE TO BECOME PREGNANT
240361	other	INSERTION FAILURE
240511	other	PI WILL NO LONGER BE PRACTICING.
240522	other	DESIRES TO BECOME PREGNANT
240523	other	SUBJECT REQUEST DUE TO RECURRENT PAST LEFT OVARIAN CYSTS
240645	other	HUSBAND GETTING VASECTOMY
240717	other	SUBJECT IS GOING TO BE AN INVITRO EGG DONOR FOR HER SISTER
240733	other	DYSPAREUNIA FOR PARTNER
240802	other	MOVED OUT OF STATE
240804	other	WISH FOR PREGNANCY
240807	other	SUBJECT UNABLE TO COMPLY WITH VISITS
240819	other	DESIRES CONCEPTION
240821	other	PATIENT WANTS TO GET PREGNANT
240830	other	PATIENT DESIRES CONCEPTION
240907	other	SUBJECT MOVING OUT OF STATE
241022	other	WANTS TO BECOME PREGNANT
241030	other	SUBJECT HAD NO NEED FOR CONTRACEPTION
241101	other	SUBJECT NO LONGER NEEDS CONTRACEPTION
241103	other	SUBJECT WANTS TO CONCIEVE
241118	other	WISH FOR PREGNANCY
241121	other	SEEKING PREGNANCY
241134	other	WISH FOR PREGNANCY
241135	other	WANTS TO CONCEIVE
241155	other	DESIRES CONCEPTION
241157	other	WISH FOR PREGNANCY
241189	other	WISH FOR PREGNANCY
241196	other	WISHES TO CONCIEVE
241203	other	SUBJECT IS MOVING OUT OF THE COUNTRY
241220	other	LOST TO FOLLOW UP
241235	other	SUBJECT TOO BUSY TO COMMIT TO WORK SCHEDULE
241243	other	SUBJECT MOVING
241422	other	SUBJECT MOVED OUT OF STATE
241511	other	WANT TO PLAN FOR PREGNANCY NEXT YEAR

Table 14.1.1 / 6: Listing of subjects who prematurely discontinued study medication with reason 'other' by treatment (all randomized subjects) (cont.)

TREATMENT: LCS16

Subject number	Premature EOSM or never taken, reason	Premature EOSM other reason
241524	other	ALTERNATIVE CONTRACEPTION TUBAL LIGATION
241529	other	MOVED TO COSTA RICO UNABLE TO CONTINUE FOLLOW-UP VISIT
241533	other	SUBJECT NO LONGER NEEDS CONTRACEPTION
241608	other	REMOVED BY PRIVATE PHYSICIAN DURING LOOP ELECTROSURGICAL EXCISION PROCEDURE
241703	other	DESIRES PREGNANCY
241705	other	SUBJECT IS RELOCATING TO CALIFORNIA.
241722	other	DESIRES PREGNANCY
242225	other	PT. MOVING
242314	other	PATIENT MOVED TO CONNECTICUT
242703	other	MARITAL STATUS
242808	other	SUBJECT HAD LCS REMOVED BY PRIVATE PHYSICIAN
242810	other	COULD NOT TRAVEL TO OFFICE APPOINTMENT
242817	other	REMOVED ACCIDENTALLY DUE TO CONFUSION RELATED TO IUS LOCATION
242912	other	DESIRES PREGNANCY
243006	other	HARD TO MAKE APPOINTMENTS
243017	other	MOVING OUT OF STATE
243205	other	DESIRE FOR PREGNANCY
243211	other	WISH FOR PREGNANCY
243216	other	WISH FOR PREGNANCY
243318	other	SUBJECT DESIRES PREGNANCY
243606	other	WISH FOR PREGNANCY
243808	other	SUBJECT IS MOVING OUT OF STATE.
243815	other	DUE TO DESIRES PREGNANCY
243824	other	WISH FOR PREGNANCY
243827	other	MOVING OUT OF TOWN.
243901	other	MOVING TO NEW YORK
243956	other	DIARY NON-COMPLIANCE
243966	other	NON COMPLIANCE
243970	other	HUSBAND COMPLAINS OF FEELING IUD
244006	other	TVUS PERFORMED IN U/S FACILITY IN THE NEXT BUILDING INDICATED LCS WERE LOCATED AT CERVICAL/VAGINAL CANAL
244007	other	INSERTION FAILURE
244126	other	RELOCATING TO PITTSBURGH
244129	other	DESIRES PREGNANCY
244133	other	DESIRES PREGNANCY
244405	other	IMPROPER POSITION OF DEVICE
244418	other	WEIGHT GAIN WORSENING OF ACNE
244424	other	SUBJECT CURRENTLY MEETS EXCLUSION CRITERIA #24.
244429	other	WANTING TO GET PREGNANT
244449	other	WISHES PREGNANCY
244611	other	PT. DESIRES PREGNANCY
244614	other	NO LONGER NEEDS CONTRACEPTION
244712	other	WANTS TO BECOME PREGNANT

Table 14.1.1 / 6: Listing of subjects who prematurely discontinued study medication with reason 'other' by treatment (all randomized subjects) (cont.)

TREATMENT: LCS16

Subject number	Premature EOSM or never taken, reason	Premature EOSM other reason
244801	other	STRINGS WERE BOTHERING PARTNER DURING SEX
244808	other	NON-COMPLIANCE WITH STUDY PROCEDURES
244925	other	DESIRE FOR PREGNANCY
245001	other	DESIRE FOR PREGNANCY
245011	other	DESIRE FOR PREGNANCY.
245416	other	DESIRE FOR PREGNANCY
245511	other	DESIRE FOR PREGNANCY.
245522	other	DESIRE TO ENTER HCG WEIGHT LOSS PROGRAM.
245532	other	NO INSERTION ATTEMPTED DUE TO UNSURE UTERINE DEPTH AS OBSERVED BY ULTRASOUND
245602	other	DESIRES INTRAUTERINE PREGNANCY
245707	other	WISHES TO BECOME PREGNANT
245917	other	SUBJECT WANTS TO GET PREGNANT
245922	other	PT. MOVED OUT OF THE STUDY
246124	other	SUBJECT HAD IUD REMOVED AT PRIVATE DOCTORS OFFICE
246204	other	PARTNER BOTHERED BY STRING
246221	other	WANTS TO CONCEIVE

Global Biostatistics: /by-sasp/patdb/projects/de04209/310442/stat/prod_interim03/pgms/t-ds3-2.sas epkll 07OCT2011 10:47
End of table

Table 14.1.1 / 7: Subjects Disposition (all screened subjects)

Disposition (number of subjects)	LCS12	LCS16	Total
Enrolled			3661
Not randomized			776
Randomized	1432 (100.0%)	1453 (100.0%)	2885 (100.0%)
Study drug never administered	0	2 (0.1%)	2 (<0.1%)
Treated	1432 (100.0%)	1451 (99.9%)	2883 (>99.9%)
Not completed study	612 (42.7%)	583 (40.1%)	1195 (41.4%)
Completed study	819 (57.2%)	870 (59.9%)	1689 (58.5%)

Global Biostatistics: /by-sasp/patdb/projects/de04209/310442/stat/prod_interim03/pgms/t-ds3-2.sas epkll 07OCT2011 10:47
End of table

Table 14.1.1 / 8: Reason for discontinuation of study medication due to withdrawal of consent or AE by treatment and parity status (FAS)

Parity		LCS12	LCS16	Total
0 births	n	556 (100.0%)	574 (100.0%)	1130 (100.0%)
	SM discontinued due to withdrawal of consent or AE			
	no	401 (72.1%)	444 (77.4%)	845 (74.8%)
	yes	155 (27.9%)	130 (22.6%)	285 (25.2%)
	missing	2 (0.4%)	1 (0.2%)	3 (0.3%)
	progestin-related side effect	21 (3.8%)	20 (3.5%)	41 (3.6%)
	bleeding/non bleeding problem	29 (5.2%)	32 (5.6%)	61 (5.4%)
	UNK	5 (0.9%)	5 (0.9%)	10 (0.9%)
	other	98 (17.6%)	72 (12.5%)	170 (15.0%)
1 birth or more	n	876 (100.0%)	878 (100.0%)	1754 (100.0%)
	SM discontinued due to withdrawal of consent or AE			
	no	692 (79.0%)	699 (79.6%)	1391 (79.3%)
	yes	184 (21.0%)	179 (20.4%)	363 (20.7%)
	missing	4 (0.5%)	2 (0.2%)	6 (0.3%)
	progestin-related side effect	27 (3.1%)	20 (2.3%)	47 (2.7%)
	bleeding/non bleeding problem	39 (4.5%)	39 (4.4%)	78 (4.4%)
	UNK	9 (1.0%)	11 (1.3%)	20 (1.1%)
	other	105 (12.0%)	107 (12.2%)	212 (12.1%)
total	n	1432 (100.0%)	1452 (100.0%)	2884 (100.0%)
	SM discontinued due to withdrawal of consent or AE			
	no	1093 (76.3%)	1143 (78.7%)	2236 (77.5%)
	yes	339 (23.7%)	309 (21.3%)	648 (22.5%)
	missing	6 (0.4%)	3 (0.2%)	9 (0.3%)
	progestin-related side effect	48 (3.4%)	40 (2.8%)	88 (3.1%)
	bleeding/non bleeding problem	68 (4.7%)	71 (4.9%)	139 (4.8%)
	UNK	14 (1.0%)	16 (1.1%)	30 (1.0%)
	other	203 (14.2%)	179 (12.3%)	382 (13.2%)

Note: SM = Study medication
Global Biostatistics: /by-sasp/patdb/projects/de04209/310442/stat/prod_interim03/pgms/t-ds3-3.sas epkll 07OCT2011 10:47
End of table

Table 14.1.1 / 9: Half-yearly drop-out rates (Kaplan-Meier analysis) (FAS)

Treatment	Period	half yearly drop-out rate [%]	cumulative drop-out rate [%]
LCS12	1st half year	10.00	10.00
	2nd half year	9.48	18.53
	3rd half year	8.93	25.80
	4th half yesr	9.24	32.66
	5th half year	7.48	37.69
	6th half year	7.74	42.52
LCS16	1st half year	8.78	8.78
	2nd half year	8.94	16.93
	3rd half year	8.15	23.70
	4th half yesr	8.70	30.34
	5th half year	8.23	36.07
	6th half year	5.62	39.67
Total	1st half year	9.38	9.38
	2nd half year	9.21	17.73
	3rd half year	8.53	24.75
	4th half yesr	8.96	31.49
	5th half year	7.86	36.88
	6th half year	6.66	41.08

Global Biostatistics: /by-sasp/patdb/projects/de04209/310442/stat/prod_interim03/pgms/tf-km-drop-out.sas epkll 07OCT2011 10:47
End of table

Figure 14.1.1 / 1: Kaplan-Meier plot of time to drop-out (FAS)

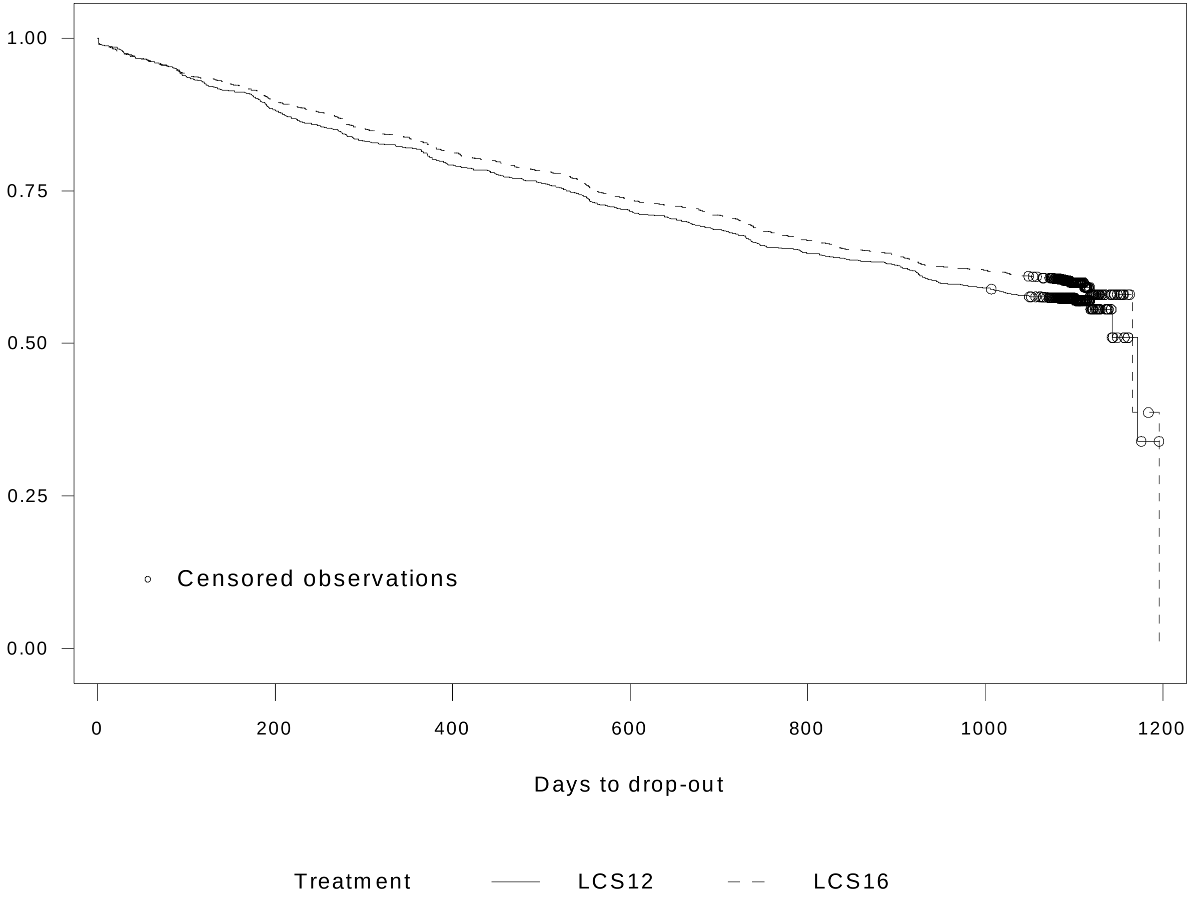

Table 14.1.1 / 10: Number of subjects with at least one minor protocol deviation by treatment (FAS)

	LCS12		LCS16		Total	
Visit	Events	N=1432 (100%)	Events	N=1452 (100%)	Events	N=2884 (100%)
at least one minor protocol deviation	4123	1259 (87.9%)	3797	1162 (80.0%)	7920	2421 (83.9%)
inclusion/exclusion error at study entry	63	56 (3.9%)	64	60 (4.1%)	127	116 (4.0%)
randomization/ registration error	10	10 (0.7%)	7	7 (0.5%)	17	17 (0.6%)
withdrawal crit. pres. but not withdrawn	5	4 (0.3%)	3	3 (0.2%)	8	7 (0.2%)
excluded concomitant treatment	20	20 (1.4%)	13	13 (0.9%)	33	33 (1.1%)
treatment deviation	59	59 (4.1%)	71	71 (4.9%)	130	130 (4.5%)
time schedule deviation	1864	716 (50.0%)	1918	741 (51.0%)	3782	1457 (50.5%)
procedure deviation	2102	1088 (76.0%)	1721	883 (60.8%)	3823	1971 (68.3%)

Global Biostatistics: /by-sasp/patdb/projects/de04209/310442/stat/prod_interim03/pgms/t-dv.sas epkll 07OCT2011 10:48
End of table

Table 14.1.1 / 11: Number of subjects with at least one major protocol deviation by treatment (FAS)

	LCS12		LCS16		Total	
Visit	Events	N=1432 (100%)	Events	N=1452 (100%)	Events	N=2884 (100%)
at least one major protocol deviation	45	39 (2.7%)	23	22 (1.5%)	68	61 (2.1%)
inclusion/exclusion error at study entry	2	2 (0.1%)	1	1 (<0.1%)	3	3 (0.1%)
excluded concomitant treatment	43	38 (2.7%)	22	22 (1.5%)	65	60 (2.1%)

Global Biostatistics: /by-sasp/patdb/projects/de04209/310442/stat/prod_interim03/pgms/t-dv.sas epkll 07OCT2011 10:48
End of table

Table 14.1.1 / 12: Listing of subjects with at least one major protocol deviation by treatment (FAS)

TREATMENT	Subject number	Time key first level	Protocol deviation type
LCS12	140911	End of study-Month 36	excluded concomitant treatment
	150503	Baseline	excluded concomitant treatment
	160105	Baseline	excluded concomitant treatment
	160208	Month 12	excluded concomitant treatment
	160406	Month 24	excluded concomitant treatment
	160710	Screening	inclusion/exclusion error at study entry
	170603	Month 12	excluded concomitant treatment
	170801	Month 6	excluded concomitant treatment
	180652	Month 18	excluded concomitant treatment
	180672	Baseline	excluded concomitant treatment
	180705	Month 6	excluded concomitant treatment
	180722	Screening	inclusion/exclusion error at study entry
		Month 6	excluded concomitant treatment
		Month 18	excluded concomitant treatment
	180733	End of study-Month 36	excluded concomitant treatment
	180739	End of study-Month 36	excluded concomitant treatment
	180740	End of study-Month 36	excluded concomitant treatment
	190110	Month 6	excluded concomitant treatment
	190418	Year 3	excluded concomitant treatment
	200104	Month 6	excluded concomitant treatment
		End of study-Month 36	excluded concomitant treatment
	200111	Month 3	excluded concomitant treatment
	200503	Month 3	excluded concomitant treatment
			excluded concomitant treatment
	200611	Month 9	excluded concomitant treatment
	200616	End of study-Month 36	excluded concomitant treatment
	200619	End of study-Month 36	excluded concomitant treatment
	200629	Month 18	excluded concomitant treatment
	200904	Month 24	excluded concomitant treatment
		End of study-Month 36	excluded concomitant treatment
	200908	Month 9	excluded concomitant treatment
		End of study-Month 36	excluded concomitant treatment
	200910	Month 3	excluded concomitant treatment
	200914	Month 12	excluded concomitant treatment
	230515	Month 12	excluded concomitant treatment
	241707	Month 24	excluded concomitant treatment
	241710	Month 30	excluded concomitant treatment
	242902	Baseline	excluded concomitant treatment
	243910	Month 3	excluded concomitant treatment
	243943	Baseline	excluded concomitant treatment
	244428	Month 9	excluded concomitant treatment
	244523	Year 3	excluded concomitant treatment
	244701	End of study-Month 36	excluded concomitant treatment
	244917	Baseline	excluded concomitant treatment

Table 14.1.1 / 12: Listing of subjects with at least one major protocol deviation by treatment (FAS)

TREATMENT	Subject number	Time key first level	Protocol deviation type
	246141	Month 24	excluded concomitant treatment
LCS16	160211	End of study-Month 36	excluded concomitant treatment
	160533	End of study-Month 36	excluded concomitant treatment
	161217	Baseline	excluded concomitant treatment
	180632	End of study-Month 36	excluded concomitant treatment
	180706	Month 30	excluded concomitant treatment
	180708	Month 18	excluded concomitant treatment
	190134	Month 9	excluded concomitant treatment
	200301	Baseline	excluded concomitant treatment
	200507	Treatment	excluded concomitant treatment
	200606	Screening	inclusion/exclusion error at study entry
		Month 9	excluded concomitant treatment
	200609	Month 9	excluded concomitant treatment
	200633	End of study-Month 36	excluded concomitant treatment
	200917	Baseline	excluded concomitant treatment
	241176	Month 30	excluded concomitant treatment
	241709	Month 3	excluded concomitant treatment
	242914	Baseline	excluded concomitant treatment
	243901	Month 30	excluded concomitant treatment
	243963	Month 12	excluded concomitant treatment
	244010	End of study-Month 36	excluded concomitant treatment
	244505	End of study-Month 36	excluded concomitant treatment
	244609	Baseline	excluded concomitant treatment
	244925	Treatment	excluded concomitant treatment

Global Biostatistics: /by-sasp/patdb/projects/de04209/310442/stat/prod_interim03/pgms/t-dv.sas epkll 07OCT2011 10:48
End of table

Table 14.1.1 / 13: Listing of inclusion- and exclusion criteria

Inclusion/exclusion question	Valid after amendment	Inclusion/exclusion question text
IN 1	0	SIGNED INFORMED CONSENT
IN 2	0	AGE BETWEEN 18 AND 35 YEARS (INCLUSIVE), IN GOOD GENERAL HEALTH AND REQUESTING CONTRACEPTION
IN 3	0	IN THE OPINION OF THE INVESTIGATOR, SUITABLE GENERAL AND UTERINE CONDITIONS FOR INSERTING THE LCS
IN 4	0	CLINICALLY NORMAL SAFETY LABORATORY RESULTS (I.E. INSIDE THE SPECIFIED RANGE FOR INCLUSION)
IN 5	0	WILLINGNESS AND ABILITY TO ATTEND THE SCHEDULED VISITS AND TO COMPLY WITH THE STUDY PROCEDURES
IN 6	0	REGULAR MENSTRUAL CYCLES (LENGTH OF CYCLE 21-35 DAYS) (I.E. ENDOGENOUS CYCLICITY WITHOUT HORMONAL CONTRACEPTIVE USE)
EX 1	0	KNOWN OR SUSPECTED PREGNANCY OR IS LACTATING
EX 2	0	VAGINAL DELIVERY, CESAREAN DELIVERY, OR ABORTION WITHIN 6 WEEKS PRIOR TO VISIT 1*
EX 3	0	HISTORY OF ECTOPIC PREGNANCIES
EX 4	0	INFECTED ABORTION OR POSTPARTUM ENDOMETRITIS WITHIN 3 MONTHS PRIOR TO VISIT 1
EX 5	0	ABNORMAL UTERINE BLEEDING OF UNKNOWN ORIGIN
EX 6	0	ANY GENITAL INFECTION (UNTIL SUCCESSFULLY TREATED)
EX 7	0	DESCRIPTIVE DIAGNOSES OF EPITHELIAL CELL ATYPIAS (NOT BENIGN ATYPIAS) OR MORE SERIOUS DISORDER IN CERVICAL SMEAR (ACCORDING TO THE BETHESDA SYSTEM) AT SCREENING AND NOT RESPONDING TO TREATMENT
	2	ABNORMAL CERVICAL SMEAR RESULT
EX 8	0	HISTORY OF, OR CURRENT, PELVIC INFLAMMATORY DISEASE
EX 9	0	CONGENITAL OR ACQUIRED UTERINE ANOMALY
EX 10	0	ANY DISTORTION OF THE UTERINE CAVITY (BY E.G. FIBROIDS) LIKELY TO CAUSE PROBLEMS (IN THE OPINION OF THE INVESTIGATOR) DURING INSERTION, RETENTION OR REMOVAL OF THE LCS
EX 11	0	HISTORY OF, DIAGNOSED OR SUSPECTED GENITAL MALIGNANCY, AND UNTREATED CERVICAL DYSPLASIA

Table 14.1.1 / 13: Listing of inclusion- and exclusion criteria

Inclusion/exclusion question	Valid after amendment	Inclusion/exclusion question text
EX 12	0	CURRENT DEEP VENOUS THROMBOSIS OR THROMBOPHLEBITIS; HISTORY OF DEEP VENOUS THROMBOSIS
EX 13	0	CURRENT ENDOMETRIAL POLYP(S)
	2	CLINICALLY SIGNIFICANT ENDOMETRIAL POLYP(S), WHICH, IN THE OPINION OF THE INVESTIGATOR, WILL INTERFERE WITH THE ASSESSMENT OF THE BLEEDING PROFILE DURING THE STUDY
EX 14	0	OVARIAN CYST(S) WITH DIAMETER > 3 CM
	2	CLINICALLY SIGNIFICANT OVARIAN CYST(S)
EX 15	0	CONCOMITANT USE OF OTHER SEX-HORMONE CONTAINING PREPARATIONS OR INTRAUTERINE DEVICE
EX 16	0	AND IF ENTERING SUBSET 2 OR SUBSET 3: ANY SEX-HORMONE ADMINISTRATION WITHIN ONE MONTH PRIOR TO START OF THE STUDY MEDICATION<br>USE OF ANY LONG-ACTING INJECTABLE SEX-HORMONE PREPARATIONS WITHIN 12 MONTHS PRIOR TO START OF STUDY MEDICATION,
EX 17	0	IF ENTERING SUBSET 2: ANY DRUG THAT MIGHT AFFECT THE BLOOD COAGULATION (E.G. HEPARIN, COUMARIN) WITIN ONE MONTH PRIOR TO START OF THE STUDY MEDICATION
EX 18	0	IF ENTERING SUBSET 2: ANY KNOWN CONDITION THAT MIGHT AFFECT THE BLOOD COAGULATION
EX 19	0	ESTABLISHED IMMUNODEFICIENCY
EX 20	0	ANY KNOWN HYPERSENSITIVITY TO THE CONSTITUENTS OF THE LCS
EX 21	0	DIAGNOSED OR SUSPECTED MALIGNANT OR PREMALIGNANT DISEASE AT SCREENING
EX 22	0	ARTERIAL HYPERTENSION NOT RESPONDING TO TREATMENT, WITH SYSTOLIC PRESSURE > 140 MMHG OR DIASTOLIC PRESSURE > 90 MMHG
EX 23	0	CURRENT (OR HISTORY OF) SEVERE HEPATIC DISEASES INCLUDING BENIGN OR MALIGNANT TUMORS. THERE SHOULD BE AN INTERVAL OF A LEAST 3 MONTHS BETWEEN THE START OF STUDY TREATMENT (I.E. LCS INSERTION) AND THE RETURN OF LIVER FUNCTION VALUES TO NORMAL
EX 24	0	HISTORY OF CHRONIC ALCOHOLISM, DRUG DEPENDENCE OR ABUSE, PSYCHOTIC STATES OR SEVERE NEUROSIS OR ANY OTHER CONDITION THAT, BY JUDGMENT OF THE INVESTIGATOR, MIGHT IMPAIR PATIENT'S ABILITY TO COOPERATE
EX 25	0	KNOWN OR SUSPECTED HIV INFECTION OR HIGH RISK FOR SEXUALLY TRANSMITTED DISEASE

Table 14.1.1 / 13: Listing of inclusion- and exclusion criteria

Inclusion/exclusion question	Valid after amendment	Inclusion/exclusion question text
EX 26	0	ANY CLINICALLY SIGNIFICANT CONDITION OR LABORATORY RESULT THAT, IN THE OPINION OF THE INVESTIGATOR, COMPROMISES PATIENT'S SAFETY, MIGHT INTERFERE WITH THE EVALUATIONS OR PREVENTS THE COMPLETION OF THE STUDY
EX 27	0	PARTICIPATED IN ANOTHER CLINICAL STUDY OR CONSUMED ANOTHER EXPERIMENTAL DRUG WITHIN 1 MONTH PRIOR TO VISIT 1
EX 28	0	PREVIOUS PARTICIPATION IN THIS STUDY
EX 29	0	CLOSE AFFILIATION WITH THE INVESTIGATIONAL SITE; E.G. CLOSE RELATIVE OF THE INVESTIGATOR, DEPENDANT PERSON, EMPLOYEE OR STUDENT OF THE INVESTIGATIONAL SITE

Note: IN=Inclusion criterion, EX=Exclusion criterion. There were 5 amendments to the original study protocol.
Note: Valid after amendment=0 indicates this criterion was valid for all subjects using the original study protocol if it was not amended later.
Global Biostatistics: /by-sasp/patdb/projects/de04209/310442/stat/prod_interim03/pgms/t-dv.sas epkll 07OCT2011 10:48
End of table

Table 14.1.1 / 14: Number of subjects by answer to inclusion- and exclusion criteria (FAS)

Inclusion/exclusion question		LCS12 (N=1432)	LCS16 (N=1452)	Total (N=2884)
IN 1	Inclusion/exclusion question answer			
	Number of subjects	1432 (100.0%)	1452 (100.0%)	2884 (100.0%)
	yes	1432 (100.0%)	1452 (100.0%)	2884 (100.0%)
IN 2	Inclusion/exclusion question answer			
	Number of subjects	1432 (100.0%)	1452 (100.0%)	2884 (100.0%)
	yes	1432 (100.0%)	1452 (100.0%)	2884 (100.0%)
IN 3	Inclusion/exclusion question answer			
	Number of subjects	1432 (100.0%)	1452 (100.0%)	2884 (100.0%)
	missing	1 (<0.1%)	0	1 (<0.1%)
	yes	1431 (>99.9%)	1452 (100.0%)	2883 (>99.9%)
IN 4	Inclusion/exclusion question answer			
	Number of subjects	1432 (100.0%)	1452 (100.0%)	2884 (100.0%)
	missing	1 (<0.1%)	4 (0.3%)	5 (0.2%)
	no	1 (<0.1%)	3 (0.2%)	4 (0.1%)
	yes	1430 (99.9%)	1445 (99.5%)	2875 (99.7%)
IN 5	Inclusion/exclusion question answer			
	Number of subjects	1432 (100.0%)	1452 (100.0%)	2884 (100.0%)
	yes	1432 (100.0%)	1452 (100.0%)	2884 (100.0%)
IN 6	Inclusion/exclusion question answer			
	Number of subjects	1432 (100.0%)	1452 (100.0%)	2884 (100.0%)
	no	1 (<0.1%)	4 (0.3%)	5 (0.2%)
	yes	1431 (>99.9%)	1448 (99.7%)	2879 (99.8%)
EX 1	Inclusion/exclusion question answer			
	Number of subjects	1432 (100.0%)	1452 (100.0%)	2884 (100.0%)
	no	1432 (100.0%)	1452 (100.0%)	2884 (100.0%)
EX 2	Inclusion/exclusion question answer			
	Number of subjects	1432 (100.0%)	1452 (100.0%)	2884 (100.0%)
	no	1430 (99.9%)	1452 (100.0%)	2882 (>99.9%)
	yes	2 (0.1%)	0	2 (<0.1%)
EX 3	Inclusion/exclusion question answer			
	Number of subjects	1432 (100.0%)	1452 (100.0%)	2884 (100.0%)
	no	1432 (100.0%)	1452 (100.0%)	2884 (100.0%)

Table 14.1.1 / 14: Number of subjects by answer to inclusion- and exclusion criteria (FAS)

Inclusion/exclusion question		LCS12 (N=1432)	LCS16 (N=1452)	Total (N=2884)
EX 4	Inclusion/exclusion question answer			
	Number of subjects	1432 (100.0%)	1452 (100.0%)	2884 (100.0%)
	no	1432 (100.0%)	1452 (100.0%)	2884 (100.0%)
EX 5	Inclusion/exclusion question answer			
	Number of subjects	1432 (100.0%)	1452 (100.0%)	2884 (100.0%)
	no	1431 (>99.9%)	1452 (100.0%)	2883 (>99.9%)
	yes	1 (<0.1%)	0	1 (<0.1%)
EX 6	Inclusion/exclusion question answer			
	Number of subjects	1432 (100.0%)	1452 (100.0%)	2884 (100.0%)
	missing	0	1 (<0.1%)	1 (<0.1%)
	no	1432 (100.0%)	1449 (99.8%)	2881 (99.9%)
	yes	0	2 (0.1%)	2 (<0.1%)
EX 7	Inclusion/exclusion question answer			
	Number of subjects	1432 (100.0%)	1452 (100.0%)	2884 (100.0%)
	missing	1 (<0.1%)	3 (0.2%)	4 (0.1%)
	no	1429 (99.8%)	1448 (99.7%)	2877 (99.8%)
	yes	2 (0.1%)	1 (<0.1%)	3 (0.1%)
EX 8	Inclusion/exclusion question answer			
	Number of subjects	1432 (100.0%)	1452 (100.0%)	2884 (100.0%)
	no	1431 (>99.9%)	1452 (100.0%)	2883 (>99.9%)
	yes	1 (<0.1%)	0	1 (<0.1%)
EX 9	Inclusion/exclusion question answer			
	Number of subjects	1432 (100.0%)	1452 (100.0%)	2884 (100.0%)
	missing	0	1 (<0.1%)	1 (<0.1%)
	no	1432 (100.0%)	1451 (>99.9%)	2883 (>99.9%)
EX 10	Inclusion/exclusion question answer			
	Number of subjects	1432 (100.0%)	1452 (100.0%)	2884 (100.0%)
	missing	1 (<0.1%)	1 (<0.1%)	2 (<0.1%)
	no	1431 (>99.9%)	1451 (>99.9%)	2882 (>99.9%)
EX 11	Inclusion/exclusion question answer			
	Number of subjects	1432 (100.0%)	1452 (100.0%)	2884 (100.0%)
	missing	0	1 (<0.1%)	1 (<0.1%)
	no	1432 (100.0%)	1451 (>99.9%)	2883 (>99.9%)

Table 14.1.1 / 14: Number of subjects by answer to inclusion- and exclusion criteria (FAS)

Inclusion/exclusion question		LCS12 (N=1432)	LCS16 (N=1452)	Total (N=2884)
EX 12	Inclusion/exclusion question answer			
	Number of subjects	1432 (100.0%)	1452 (100.0%)	2884 (100.0%)
	no	1432 (100.0%)	1452 (100.0%)	2884 (100.0%)
EX 13	Inclusion/exclusion question answer			
	Number of subjects	1432 (100.0%)	1452 (100.0%)	2884 (100.0%)
	no	1432 (100.0%)	1452 (100.0%)	2884 (100.0%)
EX 14	Inclusion/exclusion question answer			
	Number of subjects	1432 (100.0%)	1452 (100.0%)	2884 (100.0%)
	missing	0	1 (<0.1%)	1 (<0.1%)
	no	1427 (99.7%)	1446 (99.6%)	2873 (99.6%)
	yes	5 (0.3%)	5 (0.3%)	10 (0.3%)
EX 15	Inclusion/exclusion question answer			
	Number of subjects	1432 (100.0%)	1452 (100.0%)	2884 (100.0%)
	no	1431 (>99.9%)	1451 (>99.9%)	2882 (>99.9%)
	yes	1 (<0.1%)	1 (<0.1%)	2 (<0.1%)
EX 16	Inclusion/exclusion question answer			
	Number of subjects	1432 (100.0%)	1452 (100.0%)	2884 (100.0%)
	no	1431 (>99.9%)	1452 (100.0%)	2883 (>99.9%)
	yes	1 (<0.1%)	0	1 (<0.1%)
EX 17	Inclusion/exclusion question answer			
	Number of subjects	1432 (100.0%)	1451 (100.0%)	2883 (100.0%)
	no	31 (2.2%)	30 (2.1%)	61 (2.1%)
	not applicable	1401 (97.8%)	1421 (97.9%)	2822 (97.9%)
EX 18	Inclusion/exclusion question answer			
	Number of subjects	1432 (100.0%)	1451 (100.0%)	2883 (100.0%)
	no	32 (2.2%)	30 (2.1%)	62 (2.2%)
	not applicable	1400 (97.8%)	1421 (97.9%)	2821 (97.8%)
EX 19	Inclusion/exclusion question answer			
	Number of subjects	1432 (100.0%)	1452 (100.0%)	2884 (100.0%)
	no	1432 (100.0%)	1452 (100.0%)	2884 (100.0%)
EX 20	Inclusion/exclusion question answer			
	Number of subjects	1432 (100.0%)	1452 (100.0%)	2884 (100.0%)
	no	1432 (100.0%)	1452 (100.0%)	2884 (100.0%)

Table 14.1.1 / 14: Number of subjects by answer to inclusion- and exclusion criteria (FAS)

Inclusion/exclusion question		LCS12 (N=1432)	LCS16 (N=1452)	Total (N=2884)
EX 21	Inclusion/exclusion question answer			
	Number of subjects	1432 (100.0%)	1452 (100.0%)	2884 (100.0%)
	missing	1 (<0.1%)	1 (<0.1%)	2 (<0.1%)
	no	1431 (>99.9%)	1451 (>99.9%)	2882 (>99.9%)
EX 22	Inclusion/exclusion question answer			
	Number of subjects	1432 (100.0%)	1452 (100.0%)	2884 (100.0%)
	no	1431 (>99.9%)	1449 (99.8%)	2880 (99.9%)
	yes	1 (<0.1%)	3 (0.2%)	4 (0.1%)
EX 23	Inclusion/exclusion question answer			
	Number of subjects	1432 (100.0%)	1452 (100.0%)	2884 (100.0%)
	no	1432 (100.0%)	1452 (100.0%)	2884 (100.0%)
EX 24	Inclusion/exclusion question answer			
	Number of subjects	1432 (100.0%)	1452 (100.0%)	2884 (100.0%)
	no	1432 (100.0%)	1452 (100.0%)	2884 (100.0%)
EX 25	Inclusion/exclusion question answer			
	Number of subjects	1432 (100.0%)	1452 (100.0%)	2884 (100.0%)
	no	1432 (100.0%)	1452 (100.0%)	2884 (100.0%)
EX 26	Inclusion/exclusion question answer			
	Number of subjects	1432 (100.0%)	1452 (100.0%)	2884 (100.0%)
	missing	0	6 (0.4%)	6 (0.2%)
	no	1432 (100.0%)	1444 (99.4%)	2876 (99.7%)
	yes	0	2 (0.1%)	2 (<0.1%)
EX 27	Inclusion/exclusion question answer			
	Number of subjects	1432 (100.0%)	1452 (100.0%)	2884 (100.0%)
	no	1431 (>99.9%)	1452 (100.0%)	2883 (>99.9%)
	yes	1 (<0.1%)	0	1 (<0.1%)
EX 28	Inclusion/exclusion question answer			
	Number of subjects	1432 (100.0%)	1452 (100.0%)	2884 (100.0%)
	no	1432 (100.0%)	1452 (100.0%)	2884 (100.0%)
EX 29	Inclusion/exclusion question answer			
	Number of subjects	1432 (100.0%)	1452 (100.0%)	2884 (100.0%)
	no	1432 (100.0%)	1452 (100.0%)	2884 (100.0%)

Global Biostatistics: /by-sasp/patdb/projects/de04209/310442/stat/prod_interim03/pgms/t-dv.sas epkll 07OCT2011 10:48
End of table

Table 14.1.1 / 15: Number of subjects who fulfilled all inclusion criteria and no exclusion criteria present by treatment (FAS)

	LCS12	LCS16	Total
Number of subjects	1432 (100.0%)	1452 (100.0%)	2884 (100.0%)
All criteria fulfilled			
no	22 (1.5%)	35 (2.4%)	57 (2.0%)
yes	1410 (98.5%)	1417 (97.6%)	2827 (98.0%)

Note: For some subjects, answers to inclusion or exclusion criteria are missing.
For inclusion criteria, missing answers were set to NO. For exclusion criteria, missing answers were set to YES
Global Biostatistics: /by-sasp/patdb/projects/de04209/310442/stat/prod_interim03/pgms/t-dv.sas epkll 07OCT2011 10:48
End of table

Table 14.1.1 / 16: Demographics (FAS)

	LCS12 (N=1432)	LCS16 (N=1452)	Total (N=2884)
Number of Subjects	1432 (100.0%)	1452 (100.0%)	2884 (100.0%)
Sex			
female	1432 (100.0%)	1452 (100.0%)	2884 (100.0%)
Race			
Caucasian	1142 (79.7%)	1164 (80.2%)	2306 (80.0%)
Black	75 (5.2%)	74 (5.1%)	149 (5.2%)
Hispanic	165 (11.5%)	159 (11.0%)	324 (11.2%)
Asian	11 (0.8%)	17 (1.2%)	28 (1.0%)
other	39 (2.7%)	38 (2.6%)	77 (2.7%)
Age (years)			
n	1432	1452	2884
Nmiss	0	0	0
Min	18	18	18
Mean	27.2	27.1	27.1
SD	4.7	4.9	4.8
Q1	23.0	23.0	23.0
Median	27.0	27.0	27.0
Q3	31.0	31.0	31.0
Max	35	35	35
Weight derived (kg)			
n	1431	1448	2879
Nmiss	1	4	5
Min	38	38	38
Mean	68.7	68.7	68.7
SD	15.3	15.5	15.4
Q1	58.0	58.0	58.0
Median	65.0	65.0	65.0
Q3	76.0	76.0	76.0
Max	155	173	173
Height derived (cm)			
n	1431	1449	2880
Nmiss	1	3	4
Min	130	124	124
Mean	164.7	164.7	164.7
SD	6.8	7.1	7.0
Q1	160.0	160.0	160.0
Median	165.0	165.0	165.0
Q3	170.0	170.0	170.0
Max	186	188	188

Table 14.1.1 / 16: Demographics (FAS)

	LCS12 (N=1432)	LCS16 (N=1452)	Total (N=2884)
BMI (kg/m[2])			
n	1431	1448	2879
Nmiss	1	4	5
Min	15.6	15.2	15.2
Mean	25.32	25.32	25.32
SD	5.42	5.49	5.46
Q1	21.50	21.50	21.50
Median	23.90	24.00	24.00
Q3	28.00	27.90	27.90
Max	54.9	57.6	57.6

Global Biostatistics: /by-sasp/patdb/projects/de04209/310442/stat/prod_interim03/pgms/t-dm.sas epkll 07OCT2011 10:48
End of table

Table 14.1.1 / 17: Ethnic group: Listing of specification for ethnic group 'Other' by treatment (FAS)

TREATMENT: LCS12

Subject number	Race other
120104	SOUTH AMERICAN
120117	SOUTH AMERICAN
120119	SOUTH AMERICAN
120127	SOUTH AMERICAN
120224	SOUTH AMERICAN
120304	AMERICAN INDIAN
120310	AMERICAN INDIAN
120312	AMERICAN INDIAN
120315	AMERICAN INDIAN
120320	AMERICAN INDIAN
120321	AMERICAN INDIAN
120322	AMERICAN INDIAN
120323	AMERICAN INDIAN
120326	AMERICAN INDIAN
120330	AMERICAN INDIAN
120335	AMERICAN INDIAN
141004	ITALIAN
141109	CAUCASIAN BLACK(25%)
141402	SPANISH
141403	METIS
141405	ABORIGINAL
141419	CAUCASIAN / BLACK
200101	MEDITERANIAN
200516	MEDITERANIAN
200611	50% CAUCASIAN, 50% ASIAN
200615	50% CAUCASIAN 50% ASIAN
240209	NATIVE AMERICAN CHEROKCE INDIAN
240606	FILIPINO
240701	PACIFIC ISLAND
241154	ALASKAN NATIVE
241307	PACIFIC ISLANDER
241536	BI - RACIAL ASIAN / CAUCASIAN
242204	FILIPINO
243311	PACIFIC ISLANDIER
243609	HISPANIC / NATIVE AMERICAN
243611	AMERICAN INDIAN / HISPANIC
244425	CAUCASIAN AND BLACK
245520	ASSRIYON
246150	JAMAICAN/CAUCASIAN

Table 14.1.1 / 17: Ethnic group: Listing of specification for ethnic group 'Other' by treatment (FAS) (cont.)

TREATMENT: LCS16

Subject number	Race other
120101	SOUT AMERICAN
120102	SOUTH AMERICAN.
120103	SOUTH AMERICAN.
120118	SOUTH AMERICAN
120302	AMERICAN INDIAN
120303	AMERICAN INDIAN
120306	AMERICAN INDIAN
120308	AMERICAN INDIAN
120309	AMERICAN INDIAN
120311	AMERICAN INDIAN
120313	AMERICAN INDIAN
120317	AMERICAN INDIAN
120318	AMERICAN INDIAN
120319	AMERICAN INDIAN
120324	AMERICAN INDIAN
120325	AMERICAN INDIAN
120328	AMERICAN INDIAN
120333	AMERICAN INDIAN
120336	AMERICAN INDIAN
120337	AMERICAN INDIAN
141414	METIS
141415	ENGLISH, RUSSIAN, METIS GERMAN, IRISH, SCOTTISH
200632	1 PARENT IS CAUCASIAN AND 1 PARENT IS ASIAN
240128	PACIFIC ISLANDER
240618	ASIAN ' CAUCASIAN
240907	ROMANIAN
241206	BAHAMIAN
241519	MULTI
242211	FILIPINO
242303	EGYPTIAN
242306	BRAZILIAN
242321	CAUCASIAN / WEST AFRICAN
243808	CREOLE
243923	LATINO
244433	CAUCASIAN AND HISPANIC
244516	HISPANIC / CAUCASIAN
244916	HISPANIC & ASIAN + NATIVE AMERICAN
244926	BOTH CAUCASIAN AND HISPANIC

Global Biostatistics: /by-sasp/patdb/projects/de04209/310442/stat/prod_interim03/pgms/t-dm.sas epkll 07OCT2011 10:48
End of table

Table 14.1.1 / 18: Number of subjects by age group and treatment (FAS)

	LCS12 (N=1432)	LCS16 (N=1452)	Total (N=2884)
Number of subjects	1432 (100.0%)	1452 (100.0%)	2884 (100.0%)
Age category			
age <= 25	566 (39.5%)	564 (38.8%)	1130 (39.2%)
25 < age <= 35	866 (60.5%)	888 (61.2%)	1754 (60.8%)

Global Biostatistics: /by-sasp/patdb/projects/de04209/310442/stat/prod_interim03/pgms/t-dm.sas epkll 07OCT2011 10:48
End of table

Table 14.1.1 / 19: Education level by treatment (FAS)

	LCS12	LCS16	Total
Number of subjects	1007 (100.0%)	1030 (100.0%)	2037 (100.0%)
Educational level			
some elementary education	37 (3.7%)	34 (3.3%)	71 (3.5%)
some secondary education	291 (28.9%)	315 (30.6%)	606 (29.7%)
some college or university education	679 (67.4%)	681 (66.1%)	1360 (66.8%)

Data only collected after Amendment 3 to study protocol
Global Biostatistics: /by-sasp/patdb/projects/de04209/310442/stat/prod_interim03/pgms/t-dm.sas epkll 07OCT2011 10:48
End of table

Table 14.1.1 / 20: Sexual relation/activity at screening by treatment (FAS)

	LCS12	LCS16	Total
Number of subjects	1432 (100.0%)	1452 (100.0%)	2884 (100.0%)
Sexual relations/activity			
no	16 (1.1%)	17 (1.2%)	33 (1.1%)
yes	1416 (98.9%)	1435 (98.8%)	2851 (98.9%)

Global Biostatistics: /by-sasp/patdb/projects/de04209/310442/stat/prod_interim03/pgms/t-dm.sas epkll 07OCT2011 10:48
End of table

Table 14.1.1 / 21: Number of subjects currently smoking by treatment (FAS)

	LCS12	LCS16	Total
Number of subjects	1432 (100.0%)	1452 (100.0%)	2884 (100.0%)
Still smoking			
no	1098 (76.7%)	1092 (75.2%)	2190 (75.9%)
yes	334 (23.3%)	360 (24.8%)	694 (24.1%)

Global Biostatistics: /by-sasp/patdb/projects/de04209/310442/stat/prod_interim03/pgms/t-dm.sas epkll 07OCT2011 10:48
End of table

Table 14.1.1 / 22: Average number of cigarettes smoked per day in subjects who are currently smoking by treatment (FAS)

	TREATMENT	n	Nmiss	Mean	SD	Min	Q1	Median	Q3	Max
No of cigarettes/day	LCS12	323	11	7.8	5.6	0	3.0	7.0	10.0	30
	LCS16	339	21	7.9	5.9	0	3.0	6.0	10.0	25
	Total	662	32	7.9	5.8	0	3.0	7.0	10.0	30

Global Biostatistics: /by-sasp/patdb/projects/de04209/310442/stat/prod_interim03/pgms/t-dm.sas epkll 07OCT2011 10:48
End of table

Table 14.1.1 / 23: Subjects with other smoking habits by treatment (FAS)

TREATMENT: LCS12

Subject number	Still smoking	No of cigarettes/day	Smoking habits text
140105	yes	5	MARIJUANA 1 TIME/MONTH
141101	yes	12	NONE
141409	no	.	QUIT 3 WEEKS AGO 7 CIG/DAY
150138	yes	1	NO
161215	no	.	HAVE FINISHED ONE MONTH AGO
161514	yes	.	SELDOM
161525	yes	.	OCCASIONALLY
170901	yes	1	5 CIGARETTES/WEEK -
190403	yes	1	1/WEEK
200601	yes	20	MARIHUANA, INCIDENTAL
200613	yes	.	JOINT. ONCE/WEEK.
200624	yes	20	JOINT 1 DAILY
200625	yes	5	WEED, ONE/14 DAYS.
200634	yes	2	HASH. 1/3MONTHS.
200709	yes	10	NO
200710	yes	6	NO
200911	yes	8	CANNABIS (ONCE IN 3 MONTHS)
210412	yes	.	OCCASIONAL SMOKER
230406	yes	.	SNUFF
230512	yes	.	3 CIGARETTES/WEEK
230615	no	.	SNUFF OCCASIONALLY
230702	yes	.	PARTY SMOKER
230816	yes	2	NOT EVERY DAY, TRIES TO QUIT
231005	yes	2	PARTY SMOKING.
231102	yes	3	SNUFF "CATCH - MINI" 5 - 6 PER DAY
240104	yes	1	NONE
240115	yes	1	NONE
240123	yes	1	NONE
240131	no	.	NONE
240132	no	.	NONE
240135	no	.	NONE
240719	yes	.	1-2 CIGARETTES PER MONTH
240736	yes	.	SMOKES 1-2 CIGARETTES PER WEEK
240826	yes	1	SOCIAL SMOKER
241231	yes	.	1 TO 2 CIGARETTES OVER A WEEKEND.
241530	yes	15	NO
241547	yes	.	4 CIGARS PER WEEK
241807	yes	6	NO
242103	yes	2	NONE
243327	yes	4	NONE
243806	yes	0	1 CIGAR EVERY 14 DAYS
243938	no	.	NONE
243939	no	.	NONE
243946	no	.	NONE
243948	no	.	NONE

Table 14.1.1 / 23: Subjects with other smoking habits by treatment (FAS)

TREATMENT: LCS12

Subject number	Still smoking	No of cigarettes/day	Smoking habits text
243950	yes	10	NO
243962	no	.	NONE
243968	no	.	NONE
243969	no	.	NONE
243972	no	.	NONE
244412	yes	2	2 PACKS PER DAY
244427	yes	20	ONE PACK PER DAY
244442	no	.	PREVIOUS SMOKER - STOP DATE 2006
244710	no	.	NONE
244711	yes	10	NONE
244717	yes	5	NONE
245528	yes	1	SMOKES MAY BE 1 CIGARETTE EVERY 3-4 MONTHS

Table 14.1.1 / 23: Subjects with other smoking habits by treatment (FAS) (cont.)

TREATMENT: LCS16

Subject number	Still smoking	No of cigarettes/day	Smoking habits text
140201	yes	2	N/A
140203	yes	12	N/A
140509	yes	1	1 CIG .12 WEEKS X 4 YEARS
150116	yes	3	NO
150118	yes	6	NONE
150120	yes	1	NO
150141	yes	2	NONE
160204	yes	0	5/MONTH
160417	yes	.	ONCE A WEEK, NOT DAILY
160501	yes	.	ONCE A MONTH
160532	yes	.	2 CIGARETTES A WEEK
160543	yes	.	TWO TIMES IN A YEAR
160545	yes	.	1 CIGARETTE IN MONTH
161213	yes	1	OCCASIONALLY
180633	yes	.	SELDOM
190142	yes	.	1 EVERY WEEK
200306	yes	.	SMOKES MARIHUANA
200711	yes	25	NO
210110	yes	.	SHE SMOKE ONLY ON PARTY
210122	yes	1	PARTY SMOKER
210601	yes	.	PARTY SMOKER
210605	yes	.	8 PR. MONTH
230309	yes	.	PARTY SMOKER, 6 CIGARRETTES / WEEK.
230404	yes	.	SNUFF (3"BOXES"/WEEK)
230415	yes	.	SNUFF 1 BOX PER MONTH.
230416	yes	.	SNUFF 3 BOXES/WEEK
230601	yes	.	SNUFF 6 TIMES DAILY
230609	yes	.	SNUFFS ONE SNUFF EACH DAY
230815	yes	.	BUT SELDOM
230822	yes	3	TRY TO STOP SMOKE
240103	no	.	NONE
240106	yes	2	NONE
240113	yes	7	NONE
240117	no	.	NONE
240118	yes	15	NONE
240126	no	.	NONE
240127	no	.	NONE
240128	no	.	NONE
240133	no	.	NONE
240603	yes	.	SOCIAL, WEEKENDS 1-2 WK
240620	yes	10	NOT EVERY DAY, 10 MAXIMUM
240624	yes	.	1-2X/WEEK
243223	yes	10	NONE
243902	yes	3	0
243936	no	.	NONE

Table 14.1.1 / 23: Subjects with other smoking habits by treatment (FAS) (cont.)

TREATMENT: LCS16

Subject number	Still smoking	No of cigarettes/day	Smoking habits text
243937	yes	1	NONE
243949	no	.	NONE
243954	yes	10	NONE
243957	no	.	NONE
243960	no	.	NONE
243963	no	.	NONE
243966	yes	10	NONE
243967	yes	2	NONE
243975	yes	20	NO
243976	no	.	NONE
244420	yes	20	1 PACK PER DAY
244933	yes	.	MARIJUANA 2 TIMES A MONTH

Global Biostatistics: /by-sasp/patdb/projects/de04209/310442/stat/prod_interim03/pgms/t-dm.sas epkll 07OCT2011 10:48
End of table

Table 14.1.1 / 24: Number of subjects with alcohol consumption by treatment (FAS)

	LCS12	LCS16	Total
Number of subjects	1432 (100.0%)	1452 (100.0%)	2884 (100.0%)
Alcohol frequency			
never	315 (22.0%)	317 (21.8%)	632 (21.9%)
seldom	521 (36.4%)	503 (34.6%)	1024 (35.5%)
occasionally	546 (38.1%)	576 (39.7%)	1122 (38.9%)
regularly	50 (3.5%)	56 (3.9%)	106 (3.7%)

Global Biostatistics: /by-sasp/patdb/projects/de04209/310442/stat/prod_interim03/pgms/t-dm.sas epkll 07OCT2011 10:48
End of table

Table 14.1.1 / 25: Listing of subjects with comments about alcohol consumption by treatment (FAS)

Subject number	Alcohol frequency	Alcohol details
140101	occasionally	3 - 7/WEEK
140103	occasionally	4 BEERS / WEEK
140105	occasionally	1 1/2 WINE BOTTLE PER WEEK
140106	occasionally	6 BEERS ON WEEK-END
140110	seldom	1 GLASS OF WINE/MONTH
140113	regularly	10-15 CONSUMPTIONS/2WEEKS
140114	seldom	1 CONSUMPTION / WEEK
140116	seldom	1-2 CONSUMPTIONS / MONTH
140117	seldom	2 CONSUMPTIONS / WEEK
140118	seldom	1 CONSUMPTION / WEEK
140119	seldom	1 BEER / MONTH
140120	occasionally	7-8 CONSUMPTIONS/WEEK
140201	regularly	02 PER WEEK
140202	occasionally	ONE PER MONTH
140203	occasionally	1 PER WEEK
140206	occasionally	2 PER WEEK
140207	seldom	1 PER 3 MONTHS
140208	seldom	1 PER TWO MONTH
140210	regularly	2 PER WEEK
140211	regularly	4 PER WEEK
140212	occasionally	1 PER WEEK
140214	occasionally	4 PER WEEK
140215	seldom	2 PER MONTH
140216	seldom	4 PER MONTH
140218	seldom	4 PER YEAR
140219	occasionally	4 PER WEEK
140220	occasionally	4 PER WEEK
140221	regularly	7 PER WEEK
140222	seldom	1 PER MONTH
140223	occasionally	3 PER WEEK.
140224	occasionally	1 PER WEEK
140225	occasionally	3 PER WEEK
140227	seldom	1 PER MONTH
140228	regularly	7 PER WEEK
140229	regularly	7 PER WEEK
140301	occasionally	2 GLASSES PER MONTH
140302	regularly	3-4 DRINKS/WEEK
140304	regularly	2-3 GLASSES/WEEK
140506	occasionally	3-4 GLASSES OF WINE PER WEEK.
140507	occasionally	2 GLASS OF WINE / WEEK
140509	occasionally	3 OR 4 BEERS A WEEK
140514	occasionally	APPROXIMATELY 6 BEERS A MONTH
140518	regularly	3-4 GLASSES OF WINE 3 TIMES/WEEK
140519	occasionally	ONCE A WEEK
140520	seldom	2-3 TIMES/YEAR
140521	regularly	EVERY WEEKENDS

Table 14.1.1 / 25: Listing of subjects with comments about alcohol consumption by treatment (FAS)

Subject number	Alcohol frequency	Alcohol details
140523	regularly	3-4 BEERS A WEEK
140526	occasionally	3 BEERS A WEEK
140529	seldom	DURING HOLIDAYS
140916	regularly	1 GLASS WINE PER DAY
141002	occasionally	3 DRINKS PER WEEK
141005	regularly	2 DRINKS/WEEK
141006	regularly	2 DRINKS / WEEK
141011	regularly	5-6 DRINKS/WEEK
141012	occasionally	1-2 DRINKS/WEEK
141013	regularly	2-3 DRINKS/WEEK
141014	occasionally	2 DRINKS / WEEK
141016	occasionally	2 DRINKS PER WEEK OR LESS
141018	regularly	2 DRINKS / WEEK
141020	regularly	5 DRINKS / WEEK
141022	occasionally	2 DRINKS / WEEK
141023	regularly	6 DRINKS / WEEK
141025	occasionally	2 DRINKS PER WEEK
141028	occasionally	3 DRINKS PER WEEK
141030	seldom	1 DRINK PER MONTH
141032	occasionally	1 DRINK / WEEK
141401	occasionally	6/MONTH
141405	occasionally	1/WEEK
141407	occasionally	2 / WEEK
141410	occasionally	2 DRINKS PER MONTH
141411	occasionally	2 PER MONTH
141413	occasionally	2 PER MONTH
141414	occasionally	2/MONTH
141419	occasionally	12/MONTH
141420	seldom	1 EVERY 2 MONTHS OR LESS
141421	seldom	1/YEAR
141423	occasionally	1-2 PER MONTH
141426	occasionally	1 PER WEEK
150101	occasionally	1 TIME IN MONTH
150102	occasionally	2 TIMES ON THE MONTH
150103	occasionally	2 TIMES ON THE MONTH
150104	occasionally	1 PER WEEK
150108	occasionally	2 TIMES ON THE MOUTH
150109	occasionally	1 TIME EVERY THREE MONTHS.
150110	occasionally	ONE DRINKS PER MONTH
150111	seldom	1 DRINK PER MONTH
150112	occasionally	1 TIME EVERY ONE MONTH
150113	occasionally	2 TIMES EVERY 1 MONTH
150115	occasionally	1 DRINK PER MONTH
150116	occasionally	1 TIMES FOR MONTH
150117	occasionally	1 DRINK PER WEEK
150118	occasionally	2 DRINKS PER WEEK

Table 14.1.1 / 25: Listing of subjects with comments about alcohol consumption by treatment (FAS)

Subject number	Alcohol frequency	Alcohol details
150119	occasionally	1 PER MONTH
150120	occasionally	2 DRINKS PER WEEK
150121	occasionally	2 TIMES ON THE MONTH
150123	occasionally	TWO DRINKS PER MONTHS
150126	occasionally	ONE DRINKS PER WEEK.
150136	occasionally	1 DRINK FOR MONTH
150137	occasionally	1 DRINK ON MONTH
150139	occasionally	EACH 6 MONTHS
150140	occasionally	2 PER WEEK .
150142	occasionally	1 DRINKS PER WEEK
150143	occasionally	1 DRINKO PER MONTH
150145	occasionally	1 PER MONTH.
150146	occasionally	1 PER WEEK
150147	occasionally	2 PER MONTH.
150149	occasionally	2 PER WEEK.
150150	occasionally	1 PER MONTH.
150151	occasionally	1 PER MONTH.
150153	occasionally	2 PER MONTH.
150154	occasionally	1 PER MONTH.
150155	occasionally	2 PER MONTH.
150156	occasionally	3 TIMES FOR MONTH
150157	seldom	EACH 6 MONTHS
150159	occasionally	1 DRINK PER WEEK
150160	occasionally	1 DRINK FOR MONTH
150161	occasionally	1 DRINK PER WEEK
150162	occasionally	1 PER MONTH
150165	occasionally	2 PER WEEK
150167	occasionally	2 TIMES ON THE MONTH
150168	occasionally	1 PER MONTH
160602	occasionally	ABOUT ONCE A WEEK
160613	occasionally	2-3 AMOUNT/WEEK
160620	regularly	ONE GLASS OF WINE ONCE A WEEK
160633	occasionally	5 AMOUNT/ WEEK
160709	occasionally	2-3 GLASSES OF WINE PER WEEK
160720	occasionally	TWO GLASSES OF WINE PER WEEK
160749	occasionally	2 GLASSES OF WINE PER WEEK
160750	occasionally	TWO BOTLES OF WINE PER MONTH
160752	seldom	TWO GLASSES OF WINE IN ABOUT TWO MONTHS
160754	occasionally	2 GLASSES OF WINE PER WEEK
160757	occasionally	4 GLASSES OF WINE PER WEEK
160760	occasionally	ABOUT 5 PORTIONS A WEEK
160803	regularly	TWICE A MONTH, 7 DRINKS PER TIME
160806	seldom	ONCE A MONTH OR LESS
160915	occasionally	2 DOSES / WEEK
160927	occasionally	4 DOSES / WEEK
160940	occasionally	3-4 DOSES/WEEK

Table 14.1.1 / 25: Listing of subjects with comments about alcohol consumption by treatment (FAS)

Subject number	Alcohol frequency	Alcohol details
160941	occasionally	1 DOSE/WEEK
160950	occasionally	10 DOSES / WEEK
160971	occasionally	7-8 DOSES/WEEK
190604	occasionally	3 BEERS ON WEEKENDS
190622	seldom	SOCIALLY
190629	occasionally	SOCIALLY ON WEEKENDS MAX. 2 DRINKS PER WEEKEND
230614	regularly	AVERAGE TWO GLASSES PER WEEK
230707	regularly	"STUDENT LIFE"
240103	occasionally	BEER AND WINES DURING WEEKENDS
240104	occasionally	2 BEERS A MONTH
240106	seldom	1 MIXED COCKTAIL 2 TIMES A MONTH
240107	seldom	6 WINE COOLERS EVERY 6 MONTHS
240110	seldom	WINE AND/OR LIQUOR 2 TIMES A WEEK
240112	seldom	5 BEERS EVERY OTHER WEEK
240113	occasionally	3 BEERS 2 TIMES A WEEK
240115	seldom	1 BEER A WEEK
240116	seldom	3 BEERS A WEEK
240118	seldom	2 BEERS A WEEK
240123	occasionally	2 BEERS OR 2 GLASSES OF WINE PER WEEK
240127	occasionally	3 BOTTLES OF BEER AND / OR 2 OZ LIQUOR EVERY 2 WEEKS.
240128	seldom	2 OZ LIQUOR EVERY 2 MONTHS
240132	seldom	1 MARGARITA EVERY 3 MONTHS
240133	occasionally	2 GLASSES OF WINE, 2 BEERS, OR 2OZ OF LIQUOR 2 TIMES A WEEK
240135	seldom	1 WINE COOLER A MONTH
240201	regularly	3 GLASSES OF WINE PER WEEK
240205	regularly	3 DRINKS PER WEEK
240208	regularly	5 DRINKS PER WEEK
240209	occasionally	1 DRINK PER MONTH
240211	regularly	3 DRINKS PER WEEK
240221	occasionally	ONE GLASS PER WEEK
240223	occasionally	2 DRINKS PER WEEK
240224	occasionally	2 DRINKS PER WEEK
240230	occasionally	1 PER MONTH
240235	occasionally	2 PER MONTH
240240	seldom	ONE DRINK EVERY 3 MONTHS
240315	regularly	ONCE A WEEK
240330	regularly	ONCE A WEEK
240363	regularly	TWICE A WEEK
240401	occasionally	1 GLASS OF WINE 2 TIMES/WEAK
240704	regularly	BEER ON WEEKENDS
240807	occasionally	WEEKENDS ONLY
240901	regularly	1 DRINK PER NIGHT
240903	seldom	1 DRINK TWICE PER YEAR
240904	occasionally	1 DRINK PER MONTH
240905	occasionally	1 DRINK PER MONTH
240906	regularly	ONE DRINK PER WEEK

Table 14.1.1 / 25: Listing of subjects with comments about alcohol consumption by treatment (FAS)

Subject number	Alcohol frequency	Alcohol details
241131	occasionally	X1 WK
241203	occasionally	SOCIAL DRINKER
241204	occasionally	SOCIAL DRINKER
241237	regularly	1 GLASS OF WINE EVERY DAY
241305	occasionally	1 GLASS WINE 4X/WK
241307	seldom	~ 1X / MONTH
241504	seldom	2 PER YEAR
241519	seldom	1 BEER A MONTH
241533	occasionally	TWO DRINKS OF WINE PER MONTH
241536	occasionally	ONE TO TWO BEERS OR WINE A WEEK.
241801	occasionally	SOMETIMES 2 DRINKS/WEEK
241802	occasionally	1-2 BOTTLES OF A BEER OR 2 GLASSES OF WINE/WK
241807	occasionally	4 BEERS / 2WKS
241809	occasionally	2 GLASSES OF WINE / WEEK
241811	occasionally	1 BEER/WK
242208	seldom	2/WEEK
242211	seldom	1/WEEK
242212	regularly	4-6 / WEEK
242213	occasionally	4 / WEEK
242215	regularly	3/WEEK
242216	seldom	1 / WK
242218	occasionally	2/WEEK
242219	occasionally	4 / WEEK
242223	occasionally	2/WEEK
242224	occasionally	3/WEEK
242225	occasionally	2/WEEK
242227	seldom	1/WEEK
242228	occasionally	2/WEEK
242320	occasionally	SOCIALLY EVERY NOW AND THEN
242321	occasionally	A GLASS OF WINE ONCE A MONTH APPROXIMATELY.
242324	seldom	1-2 DRINKS PER MONTH
242326	seldom	SPECIAL OCCASIONS ONLY
243002	seldom	SOCIAL
243006	occasionally	SOCIAL
243015	occasionally	WINE OR BEER
243022	occasionally	WEEKENDS
243023	seldom	SOCIALLY
243025	occasionally	SOCIALLY 3 X PER WEEK
243026	seldom	SOCIALLY
243030	seldom	SOCIAL
243101	occasionally	< 1 PER WEEK
243122	occasionally	1/WK
243125	occasionally	<1 PER WEEK
243303	occasionally	BEER ON WEEKENDS
243402	occasionally	0-4 A WEEK
243403	occasionally	2 DRINKS A WEEK

Table 14.1.1 / 25: Listing of subjects with comments about alcohol consumption by treatment (FAS)

Subject number	Alcohol frequency	Alcohol details
243406	seldom	TWICE A MONTH
243606	occasionally	0-2 PER WK
243609	seldom	0~1 / WK
243611	seldom	1-2 / WK
243616	seldom	SPECIAL OCCASIONS
243618	seldom	0-1 UNITS PER WEEK
243702	occasionally	4 DRINKS PER MONTH
243703	occasionally	1 DRINK PER MONTH
243709	occasionally	2 DRINKS PER MONTH
243712	occasionally	1 DRINK PER MONTH
243717	occasionally	20 PER MONTH
243719	occasionally	7 PER MONTH
243720	occasionally	4 PER MONTH
243805	occasionally	2 DRINKS EVERY 3 WEEKS
243806	occasionally	2 DRINKS EVERY 14 DAYS
243807	occasionally	1 DRINK EVERY 2 - 3 WEEKS
243808	seldom	CELEBRATIONS ONLY
243813	occasionally	2 DRINKS PER MONTH
243819	regularly	3 GLASSES PER WEEK
243820	occasionally	1 DRINK PER WEEK
243822	regularly	2 GLASSES OF WINE PER WEEK
243823	regularly	3 DRINKS PER WEEK
243824	occasionally	1 GLASS WINE EVERY 2 WEEKS
243826	seldom	AT HOLIDAYS 2-3 DRINKS
243829	occasionally	2-3 DRINKS PER WEEKEND
243833	seldom	AT HOLIDAYS
243901	seldom	<1 DRINK / WK
243902	seldom	<1 PER WEEK
243904	occasionally	1 DRINK/WEEK
243907	occasionally	2-3 DRINKS/MO.
243909	regularly	2-3 DRINKS / WEEK
243910	occasionally	2/WK.
243911	regularly	1-2 PER WEEK
243913	seldom	<1 PER WEEK
243914	occasionally	3-4 PER WEEK
243916	seldom	1/YEAR
243918	occasionally	2 PER WEEK
243919	occasionally	4 PER MONTH
243921	occasionally	2 PER MONTH
243925	regularly	10 GLASSES PER WEEK
243928	seldom	1 PER WEEK
243932	regularly	7 GLASSES PER WEEK
243933	occasionally	2 PER MONTH
243936	seldom	1 PER MONTH
243937	occasionally	1 PER MONTH
243939	occasionally	3 PER WEEK

Table 14.1.1 / 25: Listing of subjects with comments about alcohol consumption by treatment (FAS)

Subject number	Alcohol frequency	Alcohol details
243941	occasionally	2 PER MONTH
243944	seldom	4 PER YEAR
243946	seldom	4 PER YEAR
243948	occasionally	2 PER MONTH
243949	occasionally	1 PER WEEK
243950	occasionally	3 PER WEEK
243952	occasionally	1 PER WEEK
243958	regularly	7 PER WEEK
243959	occasionally	6 PER WEEK
243962	seldom	2 PER MONTH
243964	regularly	30 OZ PER WEEK
243965	occasionally	2 PER WEEK
243967	occasionally	4 DRINKS PER WEEK
243968	occasionally	24 0Z PER MONTH
243969	regularly	30 OZ PER WEEK
243971	occasionally	2 GLASSES WINE PER MONTH
243972	occasionally	16 OZ PER MONTH
243975	occasionally	24 OZ PER MONTH
243978	occasionally	8 OZ PER MONTH
244002	seldom	ONE DRINK EVERY 6 MONTHS
244003	seldom	ONE WINE COOLER EVERY 2 WEEKS
244006	occasionally	1 - 2 BEERS A MONTH
244007	occasionally	3-5 GLASSES OF WINE PER MONTH
244008	occasionally	1-2 GIN/TONIC
244203	occasionally	SOCIALLY
244302	seldom	EVERY FEW MONTHS
244305	seldom	3 - 4 TIMES PER YEAR
244306	occasionally	SOCIALLY
244309	occasionally	2 BEERS A MONTH
244310	occasionally	ON WEEKENDS OCCASSIONALLY
244312	occasionally	ONCE A WEEK
244401	regularly	5 PER WEEK
244402	regularly	6 PER WEEK
244403	regularly	10 PER WEEK
244405	occasionally	4 DRINKS PER WEEK
244407	regularly	DRINKS PER WEEK ON AVERAGE 5
244408	occasionally	5 DRINKS PER WEEK
244412	occasionally	5 PER WEEK ON AVERAGE
244418	occasionally	2 PER WEEK
244420	seldom	LESS THAN 1 PR. WEEK
244424	occasionally	3 PER WEEK
244425	occasionally	5-7 DRINKS PER WEEK
244428	seldom	6 DRINKS PR WEEK
244429	regularly	4-5 DRINKS PER WEEK
244433	occasionally	1 DRINK PER WEEK ON AVERAGE
244435	seldom	1 OR 2 DRINKS PER MONTH

Table 14.1.1 / 25: Listing of subjects with comments about alcohol consumption by treatment (FAS)

Subject number	Alcohol frequency	Alcohol details
244437	occasionally	1-2 DRINKS PER WEEK
244439	occasionally	1 DRINK PER WEEK ON AVERAGE
244441	occasionally	3 PER WEEK ON AVERAGE
244442	occasionally	2 DRINKS PER WEEK
244446	seldom	2 DRINKS PER WEEK ON AVERAGE
244447	regularly	2-3 DRINKS PER WEEK
244449	occasionally	5 DRINKS PER WEEK ON AVGERAGE
244450	occasionally	7 DRINKS PER WEEK ON AVERAGE
244451	regularly	4-5 DRINKS PER WEEK ON AVERAGE
244503	seldom	ONCE / WEEK ON WEEKENDS
244504	occasionally	ONE OR TWO DRINKS A WEEK
244505	occasionally	1-2 GLASSES OF WINE PER WEEK
244508	occasionally	APPROX 3 DRINKS A WEEK
244509	regularly	TWICE A WEEK
244521	regularly	3 DRINKS WEEK
244701	occasionally	2-3 DRINKS PER MONTH
244704	occasionally	1 DRINK PER MONTH
244707	seldom	1-2 DRINKS PER YEAR
244710	occasionally	10 - 12 DRINKS PER MONTH
244711	regularly	12 DRINKS PER WEEK
244714	occasionally	3 DRINKS PER WEEK
244715	occasionally	2 DRINKS PER WEEK
244716	occasionally	3-4 DRINKS PER WEEK
244717	occasionally	10 DRINKS PER WEEK
244802	seldom	ONE DRINK EVERY 3-4 MONTHS
244917	occasionally	ONCE A MONTH
244918	occasionally	LESS THAN 3 PER WEEK
244922	regularly	2-3 GLASSES WINE A WEEK
244926	occasionally	5 DRINKS PER WEEK
244927	occasionally	1 DRINK PER WEEK
244931	regularly	2 DRINKS @ WEEK
244934	regularly	8 DRINKS EACH WEEK
245001	occasionally	4 BEER A WEEK
245002	seldom	<1 GLASS OF WINE
245003	occasionally	2 GLASSES OF WINE PER WEEK
245004	seldom	0 CONSUMED PER WEEK
245009	seldom	3 BEERS PER WEEK
245011	occasionally	<1 PER WEEK
245013	occasionally	<1 AMOUNT CONSUMED PER WEEK
245014	seldom	<1 AMOUNT CONSUMED PER WEEK
245015	occasionally	<1 PER WEEK
245016	seldom	<1 PER WEEK
245018	occasionally	1 PER MONTH
245019	seldom	1A MONTH CONSUMED
245020	seldom	1 PER WEEK
245021	regularly	2 BEERS PER DAY

Table 14.1.1 / 25: Listing of subjects with comments about alcohol consumption by treatment (FAS)

Subject number	Alcohol frequency	Alcohol details
245022	seldom	<1 PER WEEK
245024	occasionally	8 BEERS A WEEK
245026	occasionally	< 1 PER WEEK
245027	seldom	<1 PER WEEK
245028	seldom	<1 PER WEEK
245029	occasionally	4 DRINKS PER WEEK
245030	seldom	<1 PER WEEK
245031	occasionally	1 PER WEEK
245032	occasionally	4 PER WEEK
245035	occasionally	2-3 TIMES A WEEK
245502	seldom	0-1 DRINK PER WEEK SELDOM.
245505	occasionally	1-2 PER WEEK.
245507	seldom	1 DRINK PER WEEK.
245508	occasionally	2 DRINKS PER WEEK.
245516	occasionally	2 DRINKS PER WEEK.
245518	occasionally	2 DRINKS PER WEEK
245522	occasionally	1 DRINK PER WEEK
245524	regularly	3 OR 4 DRINKS PER WEEK
245530	occasionally	1-2 DRINKS PER WEEK.
245601	occasionally	SOCIAL
245701	occasionally	1-2 X 1 MONTH
245704	occasionally	WEEKENDS
245709	occasionally	1-2/WK
245801	regularly	TWO GLASSES OF WINE ON WEEKENDS.
245802	occasionally	SOCIAL
245807	regularly	SOCIALLY ON WEEKENDS
245808	occasionally	SOCIALLY

Global Biostatistics: /by-sasp/patdb/projects/de04209/310442/stat/prod_interim03/pgms/t-dm.sas epkll 07OCT2011 10:48
End of table

Table 14.1.1 / 26: Number of subjects with medical history findings by primary system organ class, high level term, preferred term by treatment (FAS)

Primary system organ class<br>High level term<br>Preferred term<br>MedDRA Version 14.0	LCS12<br>N=1432 (100%)	LCS16<br>N=1452 (100%)	Total<br>N=2884 (100%)
Number of subjects (%) with at least one medical history finding	1103 (77.0%)	1100 (75.8%)	2203 (76.4%)
Blood and lymphatic system disorders	30 (2.1%)	33 (2.3%)	63 (2.2%)
Anaemia deficiencies	5 (0.3%)	2 (0.1%)	7 (0.2%)
Anaemia of pregnancy	3 (0.2%)	0	3 (0.1%)
Iron deficiency anaemia	2 (0.1%)	2 (0.1%)	4 (0.1%)
Anaemias NEC	22 (1.5%)	27 (1.9%)	49 (1.7%)
Anaemia	22 (1.5%)	27 (1.9%)	49 (1.7%)
Coagulopathies	1 (<0.1%)	0	1 (<0.1%)
Activated protein C resistance	1 (<0.1%)	0	1 (<0.1%)
Lymphatic system disorders NEC	1 (<0.1%)	3 (0.2%)	4 (0.1%)
Lymphadenitis	0	2 (0.1%)	2 (<0.1%)
Lymphadenopathy	0	2 (0.1%)	2 (<0.1%)
Lymphoid tissue hyperplasia	1 (<0.1%)	0	1 (<0.1%)
Thrombocytopenias	1 (<0.1%)	1 (<0.1%)	2 (<0.1%)
Idiopathic thrombocytopenic purpura	0	1 (<0.1%)	1 (<0.1%)
Thrombocytopenia	1 (<0.1%)	0	1 (<0.1%)
Thrombocytoses	0	1 (<0.1%)	1 (<0.1%)
Thrombocytosis	0	1 (<0.1%)	1 (<0.1%)
Cardiac disorders	6 (0.4%)	19 (1.3%)	25 (0.9%)
Aortic valvular disorders	0	1 (<0.1%)	1 (<0.1%)
Aortic valve disease	0	1 (<0.1%)	1 (<0.1%)
Cardiac conduction disorders	0	1 (<0.1%)	1 (<0.1%)
Wolff-Parkinson-White syndrome	0	1 (<0.1%)	1 (<0.1%)
Cardiac signs and symptoms NEC	1 (<0.1%)	3 (0.2%)	4 (0.1%)
Palpitations	1 (<0.1%)	3 (0.2%)	4 (0.1%)
Ischaemic coronary artery disorders	0	1 (<0.1%)	1 (<0.1%)
Angina pectoris	0	1 (<0.1%)	1 (<0.1%)
Mitral valvular disorders	2 (0.1%)	6 (0.4%)	8 (0.3%)
Mitral valve disease	0	1 (<0.1%)	1 (<0.1%)
Mitral valve prolapse	2 (0.1%)	5 (0.3%)	7 (0.2%)
Noninfectious myocarditis	1 (<0.1%)	1 (<0.1%)	2 (<0.1%)
Myocarditis	1 (<0.1%)	1 (<0.1%)	2 (<0.1%)
Pulmonary valvular disorders	0	1 (<0.1%)	1 (<0.1%)
Pulmonary valve stenosis	0	1 (<0.1%)	1 (<0.1%)
Rate and rhythm disorders NEC	3 (0.2%)	5 (0.3%)	8 (0.3%)
Arrhythmia	2 (0.1%)	2 (0.1%)	4 (0.1%)
Tachycardia	1 (<0.1%)	3 (0.2%)	4 (0.1%)

Table 14.1.1 / 26: Number of subjects with medical history findings by primary system organ class, high level term, preferred term by treatment (FAS)

Primary system organ class<br>High level term<br>Preferred term<br>MedDRA Version 14.0	LCS12<br>N=1432 (100%)	LCS16<br>N=1452 (100%)	Total<br>N=2884 (100%)
Supraventricular arrhythmias	0	3 (0.2%)	3 (0.1%)
Supraventricular tachycardia	0	3 (0.2%)	3 (0.1%)
Congenital, familial and genetic disorders	20 (1.4%)	17 (1.2%)	37 (1.3%)
Breast disorders congenital	0	1 (<0.1%)	1 (<0.1%)
Accessory breast	0	1 (<0.1%)	1 (<0.1%)
Cardiac septal defects congenital	2 (0.1%)	3 (0.2%)	5 (0.2%)
Atrial septal defect	1 (<0.1%)	0	1 (<0.1%)
Ventricular septal defect	1 (<0.1%)	3 (0.2%)	4 (0.1%)
Chromosomal abnormalities NEC	1 (<0.1%)	0	1 (<0.1%)
Gene mutation	1 (<0.1%)	0	1 (<0.1%)
Coagulation disorders congenital	2 (0.1%)	2 (0.1%)	4 (0.1%)
Factor V Leiden mutation	1 (<0.1%)	1 (<0.1%)	2 (<0.1%)
Protein S deficiency	1 (<0.1%)	0	1 (<0.1%)
Von Willebrand's disease	1 (<0.1%)	1 (<0.1%)	2 (<0.1%)
Congenital disorders NEC	0	1 (<0.1%)	1 (<0.1%)
Foetal malformation	0	1 (<0.1%)	1 (<0.1%)
Connective tissue disorders congenital	1 (<0.1%)	0	1 (<0.1%)
Ehlers-Danlos syndrome	1 (<0.1%)	0	1 (<0.1%)
External ear disorders congenital	0	1 (<0.1%)	1 (<0.1%)
Anomaly of external ear congenital	0	1 (<0.1%)	1 (<0.1%)
Gastric disorders congenital	1 (<0.1%)	0	1 (<0.1%)
Pyloric stenosis	1 (<0.1%)	0	1 (<0.1%)
Haematological disorders congenital NEC	1 (<0.1%)	0	1 (<0.1%)
Hereditary spherocytosis	1 (<0.1%)	0	1 (<0.1%)
Haemoglobinopathies congenital	1 (<0.1%)	2 (0.1%)	3 (0.1%)
Sickle cell trait	1 (<0.1%)	2 (0.1%)	3 (0.1%)
Hearing disorders congenital	0	1 (<0.1%)	1 (<0.1%)
Deafness congenital	0	1 (<0.1%)	1 (<0.1%)
Intestinal disorders congenital	0	1 (<0.1%)	1 (<0.1%)
Congenital intestinal malformation	0	1 (<0.1%)	1 (<0.1%)
Musculoskeletal and connective tissue disorders of face, neck and jaw congenital	1 (<0.1%)	0	1 (<0.1%)
Branchial cleft cyst	1 (<0.1%)	0	1 (<0.1%)
Musculoskeletal and connective tissue disorders of limbs congenital	6 (0.4%)	1 (<0.1%)	7 (0.2%)
Congenital foot malformation	1 (<0.1%)	1 (<0.1%)	2 (<0.1%)
Congenital hand malformation	0	1 (<0.1%)	1 (<0.1%)
Congenital knee deformity	1 (<0.1%)	0	1 (<0.1%)
Hip dysplasia	3 (0.2%)	0	3 (0.1%)
Syndactyly	1 (<0.1%)	0	1 (<0.1%)
Talipes	1 (<0.1%)	0	1 (<0.1%)
Non-site specific bone disorders congenital	0	1 (<0.1%)	1 (<0.1%)
Fibrous dysplasia of bone	0	1 (<0.1%)	1 (<0.1%)

Table 14.1.1 / 26: Number of subjects with medical history findings by primary system organ class, high level term, preferred term by treatment (FAS)

Primary system organ class<br>High level term<br>Preferred term<br>MedDRA Version 14.0	LCS12<br>N=1432 (100%)	LCS16<br>N=1452 (100%)	Total<br>N=2884 (100%)
Pulmonary and bronchial disorders congenital	1 (<0.1%)	0	1 (<0.1%)
Pulmonary malformation	1 (<0.1%)	0	1 (<0.1%)
Renal and urinary tract disorders congenital NEC	1 (<0.1%)	0	1 (<0.1%)
Urethral intrinsic sphincter deficiency	1 (<0.1%)	0	1 (<0.1%)
Renal disorders congenital	1 (<0.1%)	1 (<0.1%)	2 (<0.1%)
Pelvic kidney	1 (<0.1%)	0	1 (<0.1%)
Solitary kidney	0	1 (<0.1%)	1 (<0.1%)
Retinal disorders congenital	1 (<0.1%)	0	1 (<0.1%)
Retinal anomaly congenital	1 (<0.1%)	0	1 (<0.1%)
Skin and subcutaneous tissue disorders congenital NEC	0	1 (<0.1%)	1 (<0.1%)
Birth mark	0	1 (<0.1%)	1 (<0.1%)
Ureteric disorders congenital	0	1 (<0.1%)	1 (<0.1%)
Congenital ureteric anomaly	0	1 (<0.1%)	1 (<0.1%)
Ear and labyrinth disorders	9 (0.6%)	5 (0.3%)	14 (0.5%)
Hearing losses	2 (0.1%)	1 (<0.1%)	3 (0.1%)
Deafness bilateral	1 (<0.1%)	0	1 (<0.1%)
Hearing impaired	1 (<0.1%)	1 (<0.1%)	2 (<0.1%)
Inner ear signs and symptoms	3 (0.2%)	2 (0.1%)	5 (0.2%)
Motion sickness	1 (<0.1%)	1 (<0.1%)	2 (<0.1%)
Tinnitus	2 (0.1%)	0	2 (<0.1%)
Vertigo	0	1 (<0.1%)	1 (<0.1%)
Middle ear disorders NEC	0	1 (<0.1%)	1 (<0.1%)
Otosclerosis	0	1 (<0.1%)	1 (<0.1%)
Tympanic membrane disorders (excl infections)	4 (0.3%)	1 (<0.1%)	5 (0.2%)
Tympanic membrane perforation	4 (0.3%)	1 (<0.1%)	5 (0.2%)
Endocrine disorders	15 (1.0%)	21 (1.4%)	36 (1.2%)
Acute and chronic thyroiditis	0	3 (0.2%)	3 (0.1%)
Thyroiditis	0	3 (0.2%)	3 (0.1%)
Adrenal cortical hyperfunctions	1 (<0.1%)	0	1 (<0.1%)
Primary hyperaldosteronism	1 (<0.1%)	0	1 (<0.1%)
Anterior pituitary hyperfunction	0	1 (<0.1%)	1 (<0.1%)
Hyperprolactinaemia	0	1 (<0.1%)	1 (<0.1%)
Thyroid disorders NEC	1 (<0.1%)	5 (0.3%)	6 (0.2%)
Goitre	1 (<0.1%)	4 (0.3%)	5 (0.2%)
Thyroid dysfunction in pregnancy	0	1 (<0.1%)	1 (<0.1%)
Thyroid hyperfunction disorders	4 (0.3%)	2 (0.1%)	6 (0.2%)
Basedow's disease	1 (<0.1%)	0	1 (<0.1%)
Hyperthyroidism	3 (0.2%)	2 (0.1%)	5 (0.2%)
Thyroid hypofunction disorders	7 (0.5%)	12 (0.8%)	19 (0.7%)
Hypothyroidism	7 (0.5%)	12 (0.8%)	19 (0.7%)

Table 14.1.1 / 26: Number of subjects with medical history findings by primary system organ class, high level term, preferred term by treatment (FAS)

Primary system organ class<br>High level term<br>Preferred term<br>MedDRA Version 14.0	LCS12<br>N=1432 (100%)	LCS16<br>N=1452 (100%)	Total<br>N=2884 (100%)
Thyroid neoplasms	2 (0.1%)	0	2 (<0.1%)
Thyroid cyst	2 (0.1%)	0	2 (<0.1%)
Eye disorders	34 (2.4%)	43 (3.0%)	77 (2.7%)
Amblyopic vision impairment	0	3 (0.2%)	3 (0.1%)
Amblyopia	0	3 (0.2%)	3 (0.1%)
Conjunctival infections, irritations and inflammations	3 (0.2%)	2 (0.1%)	5 (0.2%)
Conjunctivitis	2 (0.1%)	2 (0.1%)	4 (0.1%)
Conjunctivitis allergic	1 (<0.1%)	0	1 (<0.1%)
Corneal infections, oedemas and inflammations	1 (<0.1%)	0	1 (<0.1%)
Ulcerative keratitis	1 (<0.1%)	0	1 (<0.1%)
Iris and uveal tract infections, irritations and inflammations	1 (<0.1%)	1 (<0.1%)	2 (<0.1%)
Iritis	1 (<0.1%)	1 (<0.1%)	2 (<0.1%)
Lacrimal disorders	1 (<0.1%)	1 (<0.1%)	2 (<0.1%)
Dry eye	1 (<0.1%)	1 (<0.1%)	2 (<0.1%)
Lid, lash and lacrimal infections, irritations and inflammations	1 (<0.1%)	0	1 (<0.1%)
Blepharitis	1 (<0.1%)	0	1 (<0.1%)
Ocular infections, inflammations and associated manifestations	0	2 (0.1%)	2 (<0.1%)
Eye allergy	0	1 (<0.1%)	1 (<0.1%)
Eye pruritus	0	1 (<0.1%)	1 (<0.1%)
Ocular nerve and muscle disorders	0	3 (0.2%)	3 (0.1%)
Strabismus	0	3 (0.2%)	3 (0.1%)
Ocular sensation disorders	0	1 (<0.1%)	1 (<0.1%)
Asthenopia	0	1 (<0.1%)	1 (<0.1%)
Optic disc abnormalities NEC	1 (<0.1%)	0	1 (<0.1%)
Papilloedema	1 (<0.1%)	0	1 (<0.1%)
Partial vision loss	1 (<0.1%)	1 (<0.1%)	2 (<0.1%)
Visual acuity reduced	1 (<0.1%)	1 (<0.1%)	2 (<0.1%)
Refractive and accommodative disorders	23 (1.6%)	28 (1.9%)	51 (1.8%)
Astigmatism	5 (0.3%)	10 (0.7%)	15 (0.5%)
Hypermetropia	3 (0.2%)	3 (0.2%)	6 (0.2%)
Myopia	19 (1.3%)	17 (1.2%)	36 (1.2%)
Refraction disorder	0	1 (<0.1%)	1 (<0.1%)
Retinal structural change, deposit and degeneration	1 (<0.1%)	0	1 (<0.1%)
Retinal detachment	1 (<0.1%)	0	1 (<0.1%)
Visual disorders NEC	0	2 (0.1%)	2 (<0.1%)
Vision blurred	0	2 (0.1%)	2 (<0.1%)
Visual field disorders	1 (<0.1%)	0	1 (<0.1%)
Scotoma	1 (<0.1%)	0	1 (<0.1%)
Gastrointestinal disorders	116 (8.1%)	103 (7.1%)	219 (7.6%)

Table 14.1.1 / 26: Number of subjects with medical history findings by primary system organ class, high level term, preferred term by treatment (FAS)

Primary system organ class<br>High level term<br>Preferred term<br>MedDRA Version 14.0	LCS12<br>N=1432 (100%)	LCS16<br>N=1452 (100%)	Total<br>N=2884 (100%)
Abdominal cavity hernias NEC	0	1 (<0.1%)	1 (<0.1%)
Ischiorectal hernia	0	1 (<0.1%)	1 (<0.1%)
Abdominal hernias, site unspecified	1 (<0.1%)	0	1 (<0.1%)
Abdominal hernia	1 (<0.1%)	0	1 (<0.1%)
Abdominal wall conditions NEC	1 (<0.1%)	0	1 (<0.1%)
Abdominal wall cyst	1 (<0.1%)	0	1 (<0.1%)
Acute and chronic pancreatitis	0	3 (0.2%)	3 (0.1%)
Pancreatitis	0	2 (0.1%)	2 (<0.1%)
Pancreatitis acute	0	1 (<0.1%)	1 (<0.1%)
Anal and rectal disorders NEC	2 (0.1%)	2 (0.1%)	4 (0.1%)
Anal fissure	1 (<0.1%)	1 (<0.1%)	2 (<0.1%)
Rectal fissure	1 (<0.1%)	1 (<0.1%)	2 (<0.1%)
Colitis (excl infective)	4 (0.3%)	1 (<0.1%)	5 (0.2%)
Colitis	1 (<0.1%)	0	1 (<0.1%)
Colitis ulcerative	2 (0.1%)	1 (<0.1%)	3 (0.1%)
Necrotising colitis	1 (<0.1%)	0	1 (<0.1%)
Dental and periodontal infections and inflammations	0	1 (<0.1%)	1 (<0.1%)
Dental caries	0	1 (<0.1%)	1 (<0.1%)
Dental disorders NEC	2 (0.1%)	2 (0.1%)	4 (0.1%)
Malocclusion	1 (<0.1%)	0	1 (<0.1%)
Tooth disorder	1 (<0.1%)	0	1 (<0.1%)
Tooth impacted	0	2 (0.1%)	2 (<0.1%)
Dental pain and sensation disorders	1 (<0.1%)	2 (0.1%)	3 (0.1%)
Toothache	1 (<0.1%)	2 (0.1%)	3 (0.1%)
Diaphragmatic hernias	0	1 (<0.1%)	1 (<0.1%)
Hiatus hernia	0	1 (<0.1%)	1 (<0.1%)
Diarrhoea (excl infective)	7 (0.5%)	2 (0.1%)	9 (0.3%)
Diarrhoea	7 (0.5%)	2 (0.1%)	9 (0.3%)
Dyspeptic signs and symptoms	9 (0.6%)	8 (0.6%)	17 (0.6%)
Dyspepsia	9 (0.6%)	8 (0.6%)	17 (0.6%)
Femoral hernias	0	1 (<0.1%)	1 (<0.1%)
Femoral hernia	0	1 (<0.1%)	1 (<0.1%)
Flatulence, bloating and distension	0	3 (0.2%)	3 (0.1%)
Abdominal distension	0	2 (0.1%)	2 (<0.1%)
Flatulence	0	1 (<0.1%)	1 (<0.1%)
Gastric ulcers and perforation	7 (0.5%)	2 (0.1%)	9 (0.3%)
Gastric ulcer	7 (0.5%)	2 (0.1%)	9 (0.3%)
Gastritis (excl infective)	7 (0.5%)	7 (0.5%)	14 (0.5%)
Gastritis	7 (0.5%)	7 (0.5%)	14 (0.5%)

Table 14.1.1 / 26: Number of subjects with medical history findings by primary system organ class, high level term, preferred term by treatment (FAS)

Primary system organ class<br>High level term<br>Preferred term<br>MedDRA Version 14.0	LCS12<br>N=1432 (100%)	LCS16<br>N=1452 (100%)	Total<br>N=2884 (100%)
Gastrointestinal and abdominal pains (excl oral and throat)	12 (0.8%)	10 (0.7%)	22 (0.8%)
Abdominal pain	9 (0.6%)	4 (0.3%)	13 (0.5%)
Abdominal pain lower	1 (<0.1%)	2 (0.1%)	3 (0.1%)
Abdominal pain upper	2 (0.1%)	4 (0.3%)	6 (0.2%)
Gastrointestinal atonic and hypomotility disorders NEC	30 (2.1%)	24 (1.7%)	54 (1.9%)
Constipation	16 (1.1%)	14 (1.0%)	30 (1.0%)
Gastrooesophageal reflux disease	17 (1.2%)	11 (0.8%)	28 (1.0%)
Impaired gastric emptying	1 (<0.1%)	0	1 (<0.1%)
Gastrointestinal disorders NEC	2 (0.1%)	0	2 (<0.1%)
Bezoar	1 (<0.1%)	0	1 (<0.1%)
Gastric disorder	1 (<0.1%)	0	1 (<0.1%)
Gastrointestinal dyskinetic disorders	0	1 (<0.1%)	1 (<0.1%)
Bowel movement irregularity	0	1 (<0.1%)	1 (<0.1%)
Gastrointestinal fistulae	0	1 (<0.1%)	1 (<0.1%)
Anal fistula	0	1 (<0.1%)	1 (<0.1%)
Gastrointestinal inflammatory disorders NEC	1 (<0.1%)	0	1 (<0.1%)
Crohn's disease	1 (<0.1%)	0	1 (<0.1%)
Gastrointestinal signs and symptoms NEC	1 (<0.1%)	3 (0.2%)	4 (0.1%)
Abdominal discomfort	1 (<0.1%)	1 (<0.1%)	2 (<0.1%)
Dysphagia	0	2 (0.1%)	2 (<0.1%)
Gastrointestinal spastic and hypermotility disorders	24 (1.7%)	13 (0.9%)	37 (1.3%)
Irritable bowel syndrome	24 (1.7%)	13 (0.9%)	37 (1.3%)
Gastrointestinal stenosis and obstruction NEC	1 (<0.1%)	0	1 (<0.1%)
Ileus	1 (<0.1%)	0	1 (<0.1%)
Gastrointestinal ulcers and perforation, site unspecified	0	3 (0.2%)	3 (0.1%)
Gastrointestinal ulcer	0	3 (0.2%)	3 (0.1%)
Haemorrhoids and gastrointestinal varices (excl oesophageal)	10 (0.7%)	6 (0.4%)	16 (0.6%)
Haemorrhoids	10 (0.7%)	6 (0.4%)	16 (0.6%)
Inguinal hernias	4 (0.3%)	4 (0.3%)	8 (0.3%)
Inguinal hernia	4 (0.3%)	4 (0.3%)	8 (0.3%)
Intestinal haemorrhages	0	2 (0.1%)	2 (<0.1%)
Rectal haemorrhage	0	2 (0.1%)	2 (<0.1%)
Malabsorption syndromes	3 (0.2%)	1 (<0.1%)	4 (0.1%)
Coeliac disease	3 (0.2%)	1 (<0.1%)	4 (0.1%)
Nausea and vomiting symptoms	0	4 (0.3%)	4 (0.1%)
Nausea	0	4 (0.3%)	4 (0.1%)
Oesophagitis (excl infective)	0	1 (<0.1%)	1 (<0.1%)
Oesophagitis	0	1 (<0.1%)	1 (<0.1%)
Peptic ulcers and perforation	1 (<0.1%)	0	1 (<0.1%)
Peptic ulcer	1 (<0.1%)	0	1 (<0.1%)
Peritoneal and retroperitoneal disorders	0	1 (<0.1%)	1 (<0.1%)
Peritonitis	0	1 (<0.1%)	1 (<0.1%)

Table 14.1.1 / 26: Number of subjects with medical history findings by primary system organ class, high level term, preferred term by treatment (FAS)

Primary system organ class<br>High level term<br>Preferred term<br>MedDRA Version 14.0	LCS12<br>N=1432 (100%)	LCS16<br>N=1452 (100%)	Total<br>N=2884 (100%)
Salivary gland infections and inflammations	0	1 (<0.1%)	1 (<0.1%)
Parotid gland inflammation	0	1 (<0.1%)	1 (<0.1%)
Umbilical hernias	1 (<0.1%)	1 (<0.1%)	2 (<0.1%)
Umbilical hernia	1 (<0.1%)	1 (<0.1%)	2 (<0.1%)
General disorders and administration site conditions	23 (1.6%)	16 (1.1%)	39 (1.4%)
Asthenic conditions	7 (0.5%)	3 (0.2%)	10 (0.3%)
Asthenia	2 (0.1%)	1 (<0.1%)	3 (0.1%)
Chronic fatigue syndrome	1 (<0.1%)	0	1 (<0.1%)
Fatigue	5 (0.3%)	2 (0.1%)	7 (0.2%)
Breast complications associated with device	0	1 (<0.1%)	1 (<0.1%)
Capsular contracture associated with breast implant	0	1 (<0.1%)	1 (<0.1%)
Complications associated with device NEC	1 (<0.1%)	0	1 (<0.1%)
Medical device pain	1 (<0.1%)	0	1 (<0.1%)
Device issues NEC	0	1 (<0.1%)	1 (<0.1%)
Device dislocation	0	1 (<0.1%)	1 (<0.1%)
Device operational issues NEC	1 (<0.1%)	0	1 (<0.1%)
Device difficult to use	1 (<0.1%)	0	1 (<0.1%)
General signs and symptoms NEC	1 (<0.1%)	1 (<0.1%)	2 (<0.1%)
Irritability	0	1 (<0.1%)	1 (<0.1%)
Unevaluable event	1 (<0.1%)	0	1 (<0.1%)
Hernias NEC	3 (0.2%)	2 (0.1%)	5 (0.2%)
Hernia	3 (0.2%)	2 (0.1%)	5 (0.2%)
Mass conditions NEC	3 (0.2%)	0	3 (0.1%)
Cyst	2 (0.1%)	0	2 (<0.1%)
Haemorrhagic cyst	1 (<0.1%)	0	1 (<0.1%)
Oedema NEC	0	1 (<0.1%)	1 (<0.1%)
Oedema peripheral	0	1 (<0.1%)	1 (<0.1%)
Pain and discomfort NEC	5 (0.3%)	6 (0.4%)	11 (0.4%)
Chest discomfort	1 (<0.1%)	0	1 (<0.1%)
Chest pain	1 (<0.1%)	1 (<0.1%)	2 (<0.1%)
Facial pain	1 (<0.1%)	0	1 (<0.1%)
Pain	2 (0.1%)	5 (0.3%)	7 (0.2%)
Therapeutic and nontherapeutic responses	2 (0.1%)	1 (<0.1%)	3 (0.1%)
Drug intolerance	2 (0.1%)	1 (<0.1%)	3 (0.1%)
Trophic disorders	0	1 (<0.1%)	1 (<0.1%)
Metaplasia	0	1 (<0.1%)	1 (<0.1%)
Hepatobiliary disorders	22 (1.5%)	15 (1.0%)	37 (1.3%)
Cholecystitis and cholelithiasis	15 (1.0%)	12 (0.8%)	27 (0.9%)
Cholecystitis	0	3 (0.2%)	3 (0.1%)
Cholelithiasis	15 (1.0%)	9 (0.6%)	24 (0.8%)

Table 14.1.1 / 26: Number of subjects with medical history findings by primary system organ class, high level term, preferred term by treatment (FAS)

Primary system organ class<br>High level term<br>Preferred term<br>MedDRA Version 14.0	LCS12<br>N=1432 (100%)	LCS16<br>N=1452 (100%)	Total<br>N=2884 (100%)
Cholestasis and jaundice	3 (0.2%)	1 (<0.1%)	4 (0.1%)
Cholestasis	0	1 (<0.1%)	1 (<0.1%)
Cholestasis of pregnancy	3 (0.2%)	0	3 (0.1%)
Gallbladder disorders NEC	3 (0.2%)	1 (<0.1%)	4 (0.1%)
Gallbladder disorder	3 (0.2%)	1 (<0.1%)	4 (0.1%)
Hepatocellular damage and hepatitis NEC	1 (<0.1%)	1 (<0.1%)	2 (<0.1%)
Hepatic steatosis	1 (<0.1%)	0	1 (<0.1%)
Hepatitis	0	1 (<0.1%)	1 (<0.1%)
Immune system disorders	241 (16.8%)	265 (18.3%)	506 (17.5%)
Allergic conditions NEC	40 (2.8%)	56 (3.9%)	96 (3.3%)
Allergy to animal	20 (1.4%)	27 (1.9%)	47 (1.6%)
Allergy to arthropod bite	0	1 (<0.1%)	1 (<0.1%)
Allergy to arthropod sting	3 (0.2%)	1 (<0.1%)	4 (0.1%)
Allergy to metals	6 (0.4%)	6 (0.4%)	12 (0.4%)
Allergy to plants	1 (<0.1%)	1 (<0.1%)	2 (<0.1%)
Hypersensitivity	7 (0.5%)	17 (1.2%)	24 (0.8%)
Multiple allergies	2 (0.1%)	4 (0.3%)	6 (0.2%)
Mycotic allergy	1 (<0.1%)	2 (0.1%)	3 (0.1%)
Allergies to foods, food additives, drugs and other chemicals	113 (7.9%)	123 (8.5%)	236 (8.2%)
Allergy to chemicals	1 (<0.1%)	4 (0.3%)	5 (0.2%)
Allergy to vaccine	1 (<0.1%)	1 (<0.1%)	2 (<0.1%)
Drug hypersensitivity	95 (6.6%)	97 (6.7%)	192 (6.7%)
Food allergy	17 (1.2%)	24 (1.7%)	41 (1.4%)
Iodine allergy	0	3 (0.2%)	3 (0.1%)
Latex allergy	3 (0.2%)	7 (0.5%)	10 (0.3%)
Milk allergy	0	1 (<0.1%)	1 (<0.1%)
Perfume sensitivity	1 (<0.1%)	0	1 (<0.1%)
Anaphylactic responses	1 (<0.1%)	0	1 (<0.1%)
Anaphylactic shock	1 (<0.1%)	0	1 (<0.1%)
Atopic disorders	130 (9.1%)	136 (9.4%)	266 (9.2%)
Atopy	6 (0.4%)	5 (0.3%)	11 (0.4%)
House dust allergy	6 (0.4%)	7 (0.5%)	13 (0.5%)
Perennial allergy	0	1 (<0.1%)	1 (<0.1%)
Seasonal allergy	123 (8.6%)	128 (8.8%)	251 (8.7%)
Infections and infestations	363 (25.3%)	347 (23.9%)	710 (24.6%)

Table 14.1.1 / 26: Number of subjects with medical history findings by primary system organ class, high level term, preferred term by treatment (FAS)

Primary system organ class<br>High level term<br>Preferred term<br>MedDRA Version 14.0	LCS12<br>N=1432 (100%)	LCS16<br>N=1452 (100%)	Total<br>N=2884 (100%)
Abdominal and gastrointestinal infections	32 (2.2%)	27 (1.9%)	59 (2.0%)
Appendicitis	28 (2.0%)	25 (1.7%)	53 (1.8%)
Appendicitis perforated	1 (<0.1%)	0	1 (<0.1%)
Diverticulitis	1 (<0.1%)	0	1 (<0.1%)
Enterocolitis infectious	1 (<0.1%)	1 (<0.1%)	2 (<0.1%)
Gastroenteritis	1 (<0.1%)	1 (<0.1%)	2 (<0.1%)
Bacterial infections NEC	37 (2.6%)	26 (1.8%)	63 (2.2%)
Bacteriuria	1 (<0.1%)	0	1 (<0.1%)
Cellulitis	1 (<0.1%)	1 (<0.1%)	2 (<0.1%)
Enterocolitis bacterial	0	1 (<0.1%)	1 (<0.1%)
Folliculitis	1 (<0.1%)	1 (<0.1%)	2 (<0.1%)
Vaginitis bacterial	34 (2.4%)	23 (1.6%)	57 (2.0%)
Bone and joint infections	2 (0.1%)	1 (<0.1%)	3 (0.1%)
Osteomyelitis	2 (0.1%)	1 (<0.1%)	3 (0.1%)
Borrelial infections	2 (0.1%)	0	2 (<0.1%)
Lyme disease	2 (0.1%)	0	2 (<0.1%)
Breast infections	4 (0.3%)	3 (0.2%)	7 (0.2%)
Breast abscess	1 (<0.1%)	2 (0.1%)	3 (0.1%)
Breast infection	1 (<0.1%)	0	1 (<0.1%)
Mastitis	2 (0.1%)	0	2 (<0.1%)
Mastitis postpartum	0	1 (<0.1%)	1 (<0.1%)
Candida infections	23 (1.6%)	19 (1.3%)	42 (1.5%)
Candidiasis	5 (0.3%)	7 (0.5%)	12 (0.4%)
Genital candidiasis	0	1 (<0.1%)	1 (<0.1%)
Vulvovaginal candidiasis	18 (1.3%)	11 (0.8%)	29 (1.0%)
Central nervous system and spinal infections	1 (<0.1%)	2 (0.1%)	3 (0.1%)
Encephalitic infection	0	1 (<0.1%)	1 (<0.1%)
Meningitis	1 (<0.1%)	1 (<0.1%)	2 (<0.1%)
Chlamydial infections	48 (3.4%)	48 (3.3%)	96 (3.3%)
Chlamydial cervicitis	17 (1.2%)	16 (1.1%)	33 (1.1%)
Chlamydial infection	7 (0.5%)	10 (0.7%)	17 (0.6%)
Genitourinary chlamydia infection	5 (0.3%)	4 (0.3%)	9 (0.3%)
Gynaecological chlamydia infection	6 (0.4%)	6 (0.4%)	12 (0.4%)
Vaginitis chlamydial	13 (0.9%)	12 (0.8%)	25 (0.9%)
Clostridia infections	0	2 (0.1%)	2 (<0.1%)
Clostridial infection	0	2 (0.1%)	2 (<0.1%)
Coccidioides infections	2 (0.1%)	0	2 (<0.1%)
Coccidioidomycosis	2 (0.1%)	0	2 (<0.1%)
Coxsackie viral infections	1 (<0.1%)	0	1 (<0.1%)
Hand-foot-and-mouth disease	1 (<0.1%)	0	1 (<0.1%)
Cytomegaloviral infections	0	1 (<0.1%)	1 (<0.1%)
Cytomegalovirus infection	0	1 (<0.1%)	1 (<0.1%)

Table 14.1.1 / 26: Number of subjects with medical history findings by primary system organ class, high level term, preferred term by treatment (FAS)

Primary system organ class<br>High level term<br>Preferred term<br>MedDRA Version 14.0	LCS12<br>N=1432 (100%)	LCS16<br>N=1452 (100%)	Total<br>N=2884 (100%)
Dental and oral soft tissue infections	5 (0.3%)	4 (0.3%)	9 (0.3%)
Periodontal infection	1 (<0.1%)	0	1 (<0.1%)
Tooth abscess	1 (<0.1%)	2 (0.1%)	3 (0.1%)
Tooth infection	3 (0.2%)	2 (0.1%)	5 (0.2%)
Ear infections	13 (0.9%)	12 (0.8%)	25 (0.9%)
Ear infection	7 (0.5%)	9 (0.6%)	16 (0.6%)
Labyrinthitis	1 (<0.1%)	0	1 (<0.1%)
Mastoiditis	1 (<0.1%)	0	1 (<0.1%)
Otitis externa	3 (0.2%)	0	3 (0.1%)
Otitis media	2 (0.1%)	3 (0.2%)	5 (0.2%)
Epstein-Barr viral infections	7 (0.5%)	5 (0.3%)	12 (0.4%)
Epstein-Barr virus infection	0	1 (<0.1%)	1 (<0.1%)
Infectious mononucleosis	7 (0.5%)	4 (0.3%)	11 (0.4%)
Eye and eyelid infections	0	3 (0.2%)	3 (0.1%)
Conjunctivitis infective	0	1 (<0.1%)	1 (<0.1%)
Hordeolum	0	2 (0.1%)	2 (<0.1%)
Female reproductive tract infections	24 (1.7%)	21 (1.4%)	45 (1.6%)
Cervicitis	3 (0.2%)	6 (0.4%)	9 (0.3%)
Endometritis decidual	1 (<0.1%)	0	1 (<0.1%)
Pelvic inflammatory disease	2 (0.1%)	1 (<0.1%)	3 (0.1%)
Salpingitis	0	1 (<0.1%)	1 (<0.1%)
Uterine infection	1 (<0.1%)	0	1 (<0.1%)
Vaginal infection	14 (1.0%)	12 (0.8%)	26 (0.9%)
Vulval abscess	0	1 (<0.1%)	1 (<0.1%)
Vulvitis	3 (0.2%)	0	3 (0.1%)
Vulvovaginitis	2 (0.1%)	0	2 (<0.1%)
Fungal infections NEC	48 (3.4%)	55 (3.8%)	103 (3.6%)
Fungal infection	30 (2.1%)	36 (2.5%)	66 (2.3%)
Fungal skin infection	0	1 (<0.1%)	1 (<0.1%)
Genital infection fungal	1 (<0.1%)	1 (<0.1%)	2 (<0.1%)
Onychomycosis	1 (<0.1%)	0	1 (<0.1%)
Vulvovaginal mycotic infection	16 (1.1%)	17 (1.2%)	33 (1.1%)
Gardnerella infections	0	2 (0.1%)	2 (<0.1%)
Gardnerella infection	0	1 (<0.1%)	1 (<0.1%)
Vaginitis gardnerella	0	1 (<0.1%)	1 (<0.1%)
Giardia infections	0	1 (<0.1%)	1 (<0.1%)
Giardiasis	0	1 (<0.1%)	1 (<0.1%)
Helicobacter infections	2 (0.1%)	0	2 (<0.1%)
Helicobacter gastritis	1 (<0.1%)	0	1 (<0.1%)
Helicobacter infection	1 (<0.1%)	0	1 (<0.1%)

Table 14.1.1 / 26: Number of subjects with medical history findings by primary system organ class, high level term, preferred term by treatment (FAS)

Primary system organ class<br>High level term<br>Preferred term<br>MedDRA Version 14.0	LCS12<br>N=1432 (100%)	LCS16<br>N=1452 (100%)	Total<br>N=2884 (100%)
Hepatitis viral infections	3 (0.2%)	8 (0.6%)	11 (0.4%)
Hepatitis A	2 (0.1%)	8 (0.6%)	10 (0.3%)
Hepatitis B	1 (<0.1%)	0	1 (<0.1%)
Herpes viral infections	28 (2.0%)	28 (1.9%)	56 (1.9%)
Genital herpes	12 (0.8%)	12 (0.8%)	24 (0.8%)
Herpes ophthalmic	1 (<0.1%)	0	1 (<0.1%)
Herpes simplex	5 (0.3%)	5 (0.3%)	10 (0.3%)
Herpes virus infection	0	1 (<0.1%)	1 (<0.1%)
Herpes zoster	0	2 (0.1%)	2 (<0.1%)
Oral herpes	6 (0.4%)	6 (0.4%)	12 (0.4%)
Varicella	4 (0.3%)	2 (0.1%)	6 (0.2%)
Infections NEC	5 (0.3%)	2 (0.1%)	7 (0.2%)
Localised infection	0	2 (0.1%)	2 (<0.1%)
Pelvic infection	1 (<0.1%)	0	1 (<0.1%)
Postoperative wound infection	1 (<0.1%)	0	1 (<0.1%)
Respiratory tract infection	3 (0.2%)	0	3 (0.1%)
Influenza viral infections	4 (0.3%)	10 (0.7%)	14 (0.5%)
Influenza	4 (0.3%)	10 (0.7%)	14 (0.5%)
Lower respiratory tract and lung infections	16 (1.1%)	19 (1.3%)	35 (1.2%)
Bronchitis	5 (0.3%)	8 (0.6%)	13 (0.5%)
Lower respiratory tract infection	0	1 (<0.1%)	1 (<0.1%)
Pneumonia	11 (0.8%)	11 (0.8%)	22 (0.8%)
Mycoplasma infections	0	1 (<0.1%)	1 (<0.1%)
Pneumonia mycoplasmal	0	1 (<0.1%)	1 (<0.1%)
Neisseria infections	6 (0.4%)	4 (0.3%)	10 (0.3%)
Gonorrhoea	6 (0.4%)	4 (0.3%)	10 (0.3%)
Papilloma viral infections	29 (2.0%)	13 (0.9%)	42 (1.5%)
Anogenital warts	28 (2.0%)	11 (0.8%)	39 (1.4%)
Papilloma viral infection	1 (<0.1%)	2 (0.1%)	3 (0.1%)
Rubella viral infections	1 (<0.1%)	0	1 (<0.1%)
Rubella	1 (<0.1%)	0	1 (<0.1%)
Salmonella infections	2 (0.1%)	1 (<0.1%)	3 (0.1%)
Gastroenteritis salmonella	1 (<0.1%)	0	1 (<0.1%)
Paratyphoid fever	0	1 (<0.1%)	1 (<0.1%)
Typhoid fever	1 (<0.1%)	0	1 (<0.1%)
Skin structures and soft tissue infections	2 (0.1%)	4 (0.3%)	6 (0.2%)
Eczema infected	1 (<0.1%)	0	1 (<0.1%)
Paronychia	0	1 (<0.1%)	1 (<0.1%)
Pilonidal cyst	1 (<0.1%)	1 (<0.1%)	2 (<0.1%)
Pseudofolliculitis barbae	0	1 (<0.1%)	1 (<0.1%)
Skin infection	0	1 (<0.1%)	1 (<0.1%)

Table 14.1.1 / 26: Number of subjects with medical history findings by primary system organ class, high level term, preferred term by treatment (FAS)

Primary system organ class<br>High level term<br>Preferred term<br>MedDRA Version 14.0	LCS12<br>N=1432 (100%)	LCS16<br>N=1452 (100%)	Total<br>N=2884 (100%)
Staphylococcal infections	0	2 (0.1%)	2 (<0.1%)
Staphylococcal infection	0	2 (0.1%)	2 (<0.1%)
Streptococcal infections	3 (0.2%)	12 (0.8%)	15 (0.5%)
Pharyngitis streptococcal	3 (0.2%)	7 (0.5%)	10 (0.3%)
Scarlet fever	0	3 (0.2%)	3 (0.1%)
Vulvovaginitis streptococcal	0	2 (0.1%)	2 (<0.1%)
Tinea infections	3 (0.2%)	2 (0.1%)	5 (0.2%)
Tinea infection	1 (<0.1%)	1 (<0.1%)	2 (<0.1%)
Tinea pedis	1 (<0.1%)	0	1 (<0.1%)
Tinea versicolour	1 (<0.1%)	1 (<0.1%)	2 (<0.1%)
Treponema infections	0	1 (<0.1%)	1 (<0.1%)
Syphilis	0	1 (<0.1%)	1 (<0.1%)
Trichomonas infections	3 (0.2%)	5 (0.3%)	8 (0.3%)
Cervicitis trichomonal	1 (<0.1%)	0	1 (<0.1%)
Trichomoniasis	0	2 (0.1%)	2 (<0.1%)
Vulvovaginitis trichomonal	2 (0.1%)	3 (0.2%)	5 (0.2%)
Tuberculous infections	0	3 (0.2%)	3 (0.1%)
Lymph node tuberculosis	0	1 (<0.1%)	1 (<0.1%)
Tuberculosis	0	3 (0.2%)	3 (0.1%)
Upper respiratory tract infections	76 (5.3%)	65 (4.5%)	141 (4.9%)
Acute tonsillitis	1 (<0.1%)	1 (<0.1%)	2 (<0.1%)
Chronic sinusitis	7 (0.5%)	2 (0.1%)	9 (0.3%)
Chronic tonsillitis	6 (0.4%)	6 (0.4%)	12 (0.4%)
Laryngitis	1 (<0.1%)	1 (<0.1%)	2 (<0.1%)
Nasopharyngitis	12 (0.8%)	7 (0.5%)	19 (0.7%)
Peritonsillar abscess	0	1 (<0.1%)	1 (<0.1%)
Peritonsillitis	0	1 (<0.1%)	1 (<0.1%)
Pharyngitis	2 (0.1%)	2 (0.1%)	4 (0.1%)
Rhinitis	1 (<0.1%)	0	1 (<0.1%)
Sinobronchitis	0	1 (<0.1%)	1 (<0.1%)
Sinusitis	25 (1.7%)	19 (1.3%)	44 (1.5%)
Tonsillitis	22 (1.5%)	18 (1.2%)	40 (1.4%)
Upper respiratory tract infection	5 (0.3%)	7 (0.5%)	12 (0.4%)
Urinary tract infections	79 (5.5%)	77 (5.3%)	156 (5.4%)
Cystitis	9 (0.6%)	9 (0.6%)	18 (0.6%)
Kidney infection	7 (0.5%)	11 (0.8%)	18 (0.6%)
Pyelonephritis	4 (0.3%)	0	4 (0.1%)
Pyelonephritis acute	0	1 (<0.1%)	1 (<0.1%)
Urethritis	1 (<0.1%)	0	1 (<0.1%)
Urinary tract infection	65 (4.5%)	57 (3.9%)	122 (4.2%)

Table 14.1.1 / 26: Number of subjects with medical history findings by primary system organ class, high level term, preferred term by treatment (FAS)

Primary system organ class<br>High level term<br>Preferred term<br>MedDRA Version 14.0	LCS12<br>N=1432 (100%)	LCS16<br>N=1452 (100%)	Total<br>N=2884 (100%)
Viral infections NEC	3 (0.2%)	4 (0.3%)	7 (0.2%)
Gastroenteritis viral	0	1 (<0.1%)	1 (<0.1%)
Genital infection viral	2 (0.1%)	1 (<0.1%)	3 (0.1%)
Meningitis viral	1 (<0.1%)	2 (0.1%)	3 (0.1%)
Injury, poisoning and procedural complications	68 (4.7%)	95 (6.5%)	163 (5.7%)
Abdominal injuries NEC	1 (<0.1%)	0	1 (<0.1%)
Splenic rupture	1 (<0.1%)	0	1 (<0.1%)
Cerebral injuries NEC	3 (0.2%)	4 (0.3%)	7 (0.2%)
Brain contusion	0	1 (<0.1%)	1 (<0.1%)
Concussion	2 (0.1%)	3 (0.2%)	5 (0.2%)
Traumatic brain injury	1 (<0.1%)	0	1 (<0.1%)
Chest and lung injuries NEC	1 (<0.1%)	0	1 (<0.1%)
Collapse of lung	1 (<0.1%)	0	1 (<0.1%)
Fractures and dislocations NEC	2 (0.1%)	6 (0.4%)	8 (0.3%)
Avulsion fracture	0	1 (<0.1%)	1 (<0.1%)
Joint dislocation	2 (0.1%)	5 (0.3%)	7 (0.2%)
Limb injuries NEC (incl traumatic amputation)	13 (0.9%)	15 (1.0%)	28 (1.0%)
Cartilage injury	0	1 (<0.1%)	1 (<0.1%)
Joint injury	3 (0.2%)	2 (0.1%)	5 (0.2%)
Joint sprain	4 (0.3%)	6 (0.4%)	10 (0.3%)
Limb injury	4 (0.3%)	1 (<0.1%)	5 (0.2%)
Meniscus lesion	2 (0.1%)	5 (0.3%)	7 (0.2%)
Synovial rupture	0	1 (<0.1%)	1 (<0.1%)
Lower limb fractures and dislocations	11 (0.8%)	20 (1.4%)	31 (1.1%)
Ankle fracture	2 (0.1%)	7 (0.5%)	9 (0.3%)
Fibula fracture	0	1 (<0.1%)	1 (<0.1%)
Foot fracture	6 (0.4%)	6 (0.4%)	12 (0.4%)
Hip fracture	0	2 (0.1%)	2 (<0.1%)
Lower limb fracture	3 (0.2%)	4 (0.3%)	7 (0.2%)
Tibia fracture	0	1 (<0.1%)	1 (<0.1%)
Muscle, tendon and ligament injuries	2 (0.1%)	13 (0.9%)	15 (0.5%)
Ligament rupture	1 (<0.1%)	6 (0.4%)	7 (0.2%)
Muscle injury	0	2 (0.1%)	2 (<0.1%)
Tendon injury	0	1 (<0.1%)	1 (<0.1%)
Tendon rupture	1 (<0.1%)	1 (<0.1%)	2 (<0.1%)
Whiplash injury	0	3 (0.2%)	3 (0.1%)

Table 14.1.1 / 26: Number of subjects with medical history findings by primary system organ class, high level term, preferred term by treatment (FAS)

Primary system organ class<br>High level term<br>Preferred term<br>MedDRA Version 14.0	LCS12<br>N=1432 (100%)	LCS16<br>N=1452 (100%)	Total<br>N=2884 (100%)
Non-site specific injuries NEC	6 (0.4%)	5 (0.3%)	11 (0.4%)
Animal bite	1 (<0.1%)	0	1 (<0.1%)
Arthropod bite	2 (0.1%)	0	2 (<0.1%)
Foreign body	0	1 (<0.1%)	1 (<0.1%)
Injury	0	2 (0.1%)	2 (<0.1%)
Road traffic accident	1 (<0.1%)	2 (0.1%)	3 (0.1%)
Traumatic haematoma	1 (<0.1%)	0	1 (<0.1%)
Wound	1 (<0.1%)	0	1 (<0.1%)
Non-site specific procedural complications	1 (<0.1%)	0	1 (<0.1%)
Post procedural complication	1 (<0.1%)	0	1 (<0.1%)
Pelvic fractures and dislocations	0	1 (<0.1%)	1 (<0.1%)
Pelvic fracture	0	1 (<0.1%)	1 (<0.1%)
Peripheral nerve injuries	1 (<0.1%)	0	1 (<0.1%)
Sciatic nerve injury	1 (<0.1%)	0	1 (<0.1%)
Reproductive system and breast injuries	1 (<0.1%)	0	1 (<0.1%)
Uterine rupture	1 (<0.1%)	0	1 (<0.1%)
Reproductive tract and breast procedural complications	1 (<0.1%)	0	1 (<0.1%)
Uterine perforation	1 (<0.1%)	0	1 (<0.1%)
Site specific injuries NEC	2 (0.1%)	6 (0.4%)	8 (0.3%)
Face injury	1 (<0.1%)	0	1 (<0.1%)
Head injury	0	4 (0.3%)	4 (0.1%)
Neck injury	1 (<0.1%)	0	1 (<0.1%)
Spinal column injury	0	1 (<0.1%)	1 (<0.1%)
Tooth injury	0	1 (<0.1%)	1 (<0.1%)
Skin injuries NEC	2 (0.1%)	1 (<0.1%)	3 (0.1%)
Contusion	2 (0.1%)	1 (<0.1%)	3 (0.1%)
Skull fractures, facial bone fractures and dislocations	4 (0.3%)	4 (0.3%)	8 (0.3%)
Facial bones fracture	3 (0.2%)	4 (0.3%)	7 (0.2%)
Jaw fracture	1 (<0.1%)	0	1 (<0.1%)
Spinal fractures and dislocations	3 (0.2%)	2 (0.1%)	5 (0.2%)
Cervical vertebral fracture	1 (<0.1%)	1 (<0.1%)	2 (<0.1%)
Dislocation of vertebra	0	1 (<0.1%)	1 (<0.1%)
Fractured coccyx	1 (<0.1%)	0	1 (<0.1%)
Lumbar vertebral fracture	1 (<0.1%)	0	1 (<0.1%)
Spinal fracture	0	1 (<0.1%)	1 (<0.1%)
Thermal burns	1 (<0.1%)	2 (0.1%)	3 (0.1%)
Thermal burn	1 (<0.1%)	2 (0.1%)	3 (0.1%)
Thoracic cage fractures and dislocations	2 (0.1%)	2 (0.1%)	4 (0.1%)
Rib fracture	2 (0.1%)	2 (0.1%)	4 (0.1%)

Table 14.1.1 / 26: Number of subjects with medical history findings by primary system organ class, high level term, preferred term by treatment (FAS)

Primary system organ class<br>High level term<br>Preferred term<br>MedDRA Version 14.0	LCS12<br>N=1432 (100%)	LCS16<br>N=1452 (100%)	Total<br>N=2884 (100%)
Upper limb fractures and dislocations	22 (1.5%)	31 (2.1%)	53 (1.8%)
Clavicle fracture	2 (0.1%)	3 (0.2%)	5 (0.2%)
Forearm fracture	1 (<0.1%)	1 (<0.1%)	2 (<0.1%)
Hand fracture	3 (0.2%)	4 (0.3%)	7 (0.2%)
Humerus fracture	1 (<0.1%)	2 (0.1%)	3 (0.1%)
Radius fracture	1 (<0.1%)	0	1 (<0.1%)
Ulna fracture	2 (0.1%)	0	2 (<0.1%)
Upper limb fracture	8 (0.6%)	13 (0.9%)	21 (0.7%)
Wrist fracture	5 (0.3%)	8 (0.6%)	13 (0.5%)
Investigations	136 (9.5%)	130 (9.0%)	266 (9.2%)
Bacteria identification and serology (excl mycobacteria)	7 (0.5%)	4 (0.3%)	11 (0.4%)
Chlamydia test positive	7 (0.5%)	3 (0.2%)	10 (0.3%)
Streptococcus test positive	0	1 (<0.1%)	1 (<0.1%)
Cardiac auscultatory investigations	7 (0.5%)	2 (0.1%)	9 (0.3%)
Cardiac murmur	7 (0.5%)	2 (0.1%)	9 (0.3%)
Cell marker procedures	0	1 (<0.1%)	1 (<0.1%)
Leukocyte antigen B-27 positive	0	1 (<0.1%)	1 (<0.1%)
Cholesterol analyses	1 (<0.1%)	0	1 (<0.1%)
Blood cholesterol increased	1 (<0.1%)	0	1 (<0.1%)
Gastrointestinal and abdominal imaging procedures	18 (1.3%)	18 (1.2%)	36 (1.2%)
Colonoscopy	1 (<0.1%)	5 (0.3%)	6 (0.2%)
Endoscopy upper gastrointestinal tract	1 (<0.1%)	1 (<0.1%)	2 (<0.1%)
Laparoscopy	16 (1.1%)	12 (0.8%)	28 (1.0%)
Oesophagogastroscopy	0	1 (<0.1%)	1 (<0.1%)
Heart rate and pulse investigations	1 (<0.1%)	2 (0.1%)	3 (0.1%)
Heart rate increased	0	2 (0.1%)	2 (<0.1%)
Heart rate irregular	1 (<0.1%)	0	1 (<0.1%)
Hepatobiliary imaging procedures	0	1 (<0.1%)	1 (<0.1%)
Endoscopic retrograde cholangiopancreatography	0	1 (<0.1%)	1 (<0.1%)
Histopathology procedures NEC	0	1 (<0.1%)	1 (<0.1%)
Biopsy site unspecified normal	0	1 (<0.1%)	1 (<0.1%)
Imaging procedures NEC	2 (0.1%)	1 (<0.1%)	3 (0.1%)
Endoscopy	2 (0.1%)	1 (<0.1%)	3 (0.1%)
Investigations NEC	0	1 (<0.1%)	1 (<0.1%)
Paracentesis	0	1 (<0.1%)	1 (<0.1%)
Liver function analyses	1 (<0.1%)	0	1 (<0.1%)
Gamma-glutamyltransferase increased	1 (<0.1%)	0	1 (<0.1%)
Mineral and electrolyte analyses	1 (<0.1%)	1 (<0.1%)	2 (<0.1%)
Blood iron decreased	1 (<0.1%)	1 (<0.1%)	2 (<0.1%)
Musculoskeletal and soft tissue imaging procedures	10 (0.7%)	12 (0.8%)	22 (0.8%)
Arthroscopy	10 (0.7%)	12 (0.8%)	22 (0.8%)

Table 14.1.1 / 26: Number of subjects with medical history findings by primary system organ class, high level term, preferred term by treatment (FAS)

Primary system organ class<br>High level term<br>Preferred term<br>MedDRA Version 14.0	LCS12<br>N=1432 (100%)	LCS16<br>N=1452 (100%)	Total<br>N=2884 (100%)
Physical examination procedures	1 (<0.1%)	2 (0.1%)	3 (0.1%)
Weight decreased	1 (<0.1%)	1 (<0.1%)	2 (<0.1%)
Weight increased	0	1 (<0.1%)	1 (<0.1%)
Red blood cell analyses	1 (<0.1%)	0	1 (<0.1%)
Haemoglobin decreased	1 (<0.1%)	0	1 (<0.1%)
Reproductive hormone analyses	1 (<0.1%)	0	1 (<0.1%)
Blood testosterone increased	1 (<0.1%)	0	1 (<0.1%)
Reproductive organ and breast histopathology procedures	62 (4.3%)	56 (3.9%)	118 (4.1%)
Aspiration breast	0	1 (<0.1%)	1 (<0.1%)
Biopsy breast	3 (0.2%)	4 (0.3%)	7 (0.2%)
Biopsy breast normal	1 (<0.1%)	0	1 (<0.1%)
Biopsy cervix	8 (0.6%)	7 (0.5%)	15 (0.5%)
Smear cervix abnormal	56 (3.9%)	44 (3.0%)	100 (3.5%)
Smear cervix normal	0	2 (0.1%)	2 (<0.1%)
Reproductive organ and breast imaging procedures	28 (2.0%)	29 (2.0%)	57 (2.0%)
Colposcopy	27 (1.9%)	24 (1.7%)	51 (1.8%)
Colposcopy normal	0	1 (<0.1%)	1 (<0.1%)
Hysteroscopy	1 (<0.1%)	3 (0.2%)	4 (0.1%)
Mammogram abnormal	0	1 (<0.1%)	1 (<0.1%)
Thyroid histopathology procedures	0	1 (<0.1%)	1 (<0.1%)
Biopsy thyroid gland	0	1 (<0.1%)	1 (<0.1%)
Urinary tract histopathology procedures	1 (<0.1%)	0	1 (<0.1%)
Biopsy kidney	1 (<0.1%)	0	1 (<0.1%)
Urinary tract imaging procedures	0	1 (<0.1%)	1 (<0.1%)
Cystoscopy	0	1 (<0.1%)	1 (<0.1%)
Virus identification and serology	28 (2.0%)	30 (2.1%)	58 (2.0%)
Human papilloma virus test negative	0	1 (<0.1%)	1 (<0.1%)
Human papilloma virus test positive	28 (2.0%)	29 (2.0%)	57 (2.0%)
Vitamin analyses	0	1 (<0.1%)	1 (<0.1%)
Vitamin B12 decreased	0	1 (<0.1%)	1 (<0.1%)
Metabolism and nutrition disorders	24 (1.7%)	23 (1.6%)	47 (1.6%)
Appetite disorders	1 (<0.1%)	1 (<0.1%)	2 (<0.1%)
Decreased appetite	1 (<0.1%)	1 (<0.1%)	2 (<0.1%)
Diabetes mellitus (incl subtypes)	4 (0.3%)	10 (0.7%)	14 (0.5%)
Gestational diabetes	4 (0.3%)	10 (0.7%)	14 (0.5%)
Elevated cholesterol	4 (0.3%)	3 (0.2%)	7 (0.2%)
Hypercholesterolaemia	4 (0.3%)	3 (0.2%)	7 (0.2%)
Food malabsorption and intolerance syndromes (excl sugar intolerance)	1 (<0.1%)	0	1 (<0.1%)
Food intolerance	1 (<0.1%)	0	1 (<0.1%)

Table 14.1.1 / 26: Number of subjects with medical history findings by primary system organ class, high level term, preferred term by treatment (FAS)

Primary system organ class<br>High level term<br>Preferred term<br>MedDRA Version 14.0	LCS12<br>N=1432 (100%)	LCS16<br>N=1452 (100%)	Total<br>N=2884 (100%)
General nutritional disorders NEC	3 (0.2%)	1 (<0.1%)	4 (0.1%)
Obesity	2 (0.1%)	1 (<0.1%)	3 (0.1%)
Overweight	1 (<0.1%)	0	1 (<0.1%)
Hyperglycaemic conditions NEC	1 (<0.1%)	0	1 (<0.1%)
Glucose tolerance impaired	1 (<0.1%)	0	1 (<0.1%)
Hyperlipidaemias NEC	1 (<0.1%)	0	1 (<0.1%)
Hyperlipidaemia	1 (<0.1%)	0	1 (<0.1%)
Hypoglycaemic conditions NEC	2 (0.1%)	1 (<0.1%)	3 (0.1%)
Hypoglycaemia	1 (<0.1%)	1 (<0.1%)	2 (<0.1%)
Hypoglycaemic seizure	1 (<0.1%)	0	1 (<0.1%)
Lipid metabolism and deposit disorders NEC	1 (<0.1%)	1 (<0.1%)	2 (<0.1%)
Body fat disorder	0	1 (<0.1%)	1 (<0.1%)
Dyslipidaemia	1 (<0.1%)	0	1 (<0.1%)
Sugar intolerance (excl glucose intolerance)	6 (0.4%)	4 (0.3%)	10 (0.3%)
Lactose intolerance	6 (0.4%)	4 (0.3%)	10 (0.3%)
Total fluid volume increased	1 (<0.1%)	2 (0.1%)	3 (0.1%)
Fluid retention	1 (<0.1%)	2 (0.1%)	3 (0.1%)
Musculoskeletal and connective tissue disorders	87 (6.1%)	90 (6.2%)	177 (6.1%)
Arthropathies NEC	5 (0.3%)	0	5 (0.2%)
Arthritis	2 (0.1%)	0	2 (<0.1%)
Arthritis reactive	1 (<0.1%)	0	1 (<0.1%)
Arthropathy	1 (<0.1%)	0	1 (<0.1%)
Sacroiliitis	1 (<0.1%)	0	1 (<0.1%)
Bone disorders NEC	1 (<0.1%)	0	1 (<0.1%)
Osteonecrosis	1 (<0.1%)	0	1 (<0.1%)
Bone related signs and symptoms	0	1 (<0.1%)	1 (<0.1%)
Pain in jaw	0	1 (<0.1%)	1 (<0.1%)
Bursal disorders	3 (0.2%)	2 (0.1%)	5 (0.2%)
Bunion	2 (0.1%)	0	2 (<0.1%)
Bursitis	1 (<0.1%)	2 (0.1%)	3 (0.1%)
Cartilage disorders	4 (0.3%)	1 (<0.1%)	5 (0.2%)
Cartilage hypertrophy	1 (<0.1%)	0	1 (<0.1%)
Costochondritis	0	1 (<0.1%)	1 (<0.1%)
Osteochondrosis	3 (0.2%)	0	3 (0.1%)
Connective tissue disorders (excl LE)	0	1 (<0.1%)	1 (<0.1%)
Scleroderma	0	1 (<0.1%)	1 (<0.1%)
Extremity deformities	2 (0.1%)	2 (0.1%)	4 (0.1%)
Foot deformity	1 (<0.1%)	2 (0.1%)	3 (0.1%)
Knee deformity	1 (<0.1%)	0	1 (<0.1%)

Table 14.1.1 / 26: Number of subjects with medical history findings by primary system organ class, high level term, preferred term by treatment (FAS)

Primary system organ class<br>High level term<br>Preferred term<br>MedDRA Version 14.0	LCS12<br>N=1432 (100%)	LCS16<br>N=1452 (100%)	Total<br>N=2884 (100%)
Intervertebral disc disorders NEC	3 (0.2%)	6 (0.4%)	9 (0.3%)
Intervertebral disc degeneration	1 (<0.1%)	0	1 (<0.1%)
Intervertebral disc displacement	0	1 (<0.1%)	1 (<0.1%)
Intervertebral disc protrusion	2 (0.1%)	5 (0.3%)	7 (0.2%)
Joint related disorders NEC	6 (0.4%)	5 (0.3%)	11 (0.4%)
Chondromalacia	0	1 (<0.1%)	1 (<0.1%)
Joint instability	1 (<0.1%)	0	1 (<0.1%)
Patellofemoral pain syndrome	1 (<0.1%)	2 (0.1%)	3 (0.1%)
Rotator cuff syndrome	0	1 (<0.1%)	1 (<0.1%)
Temporomandibular joint syndrome	4 (0.3%)	1 (<0.1%)	5 (0.2%)
Joint related signs and symptoms	15 (1.0%)	18 (1.2%)	33 (1.1%)
Arthralgia	15 (1.0%)	18 (1.2%)	33 (1.1%)
Ligament disorders	0	1 (<0.1%)	1 (<0.1%)
Ligament calcification	0	1 (<0.1%)	1 (<0.1%)
Muscle infections and inflammations	1 (<0.1%)	0	1 (<0.1%)
Myositis	1 (<0.1%)	0	1 (<0.1%)
Muscle pains	7 (0.5%)	5 (0.3%)	12 (0.4%)
Fibromyalgia	1 (<0.1%)	1 (<0.1%)	2 (<0.1%)
Myalgia	7 (0.5%)	4 (0.3%)	11 (0.4%)
Muscle related signs and symptoms NEC	8 (0.6%)	7 (0.5%)	15 (0.5%)
Muscle disorder	0	1 (<0.1%)	1 (<0.1%)
Muscle spasms	3 (0.2%)	5 (0.3%)	8 (0.3%)
Muscle tightness	5 (0.3%)	1 (<0.1%)	6 (0.2%)
Muscle weakness conditions	1 (<0.1%)	0	1 (<0.1%)
Muscular weakness	1 (<0.1%)	0	1 (<0.1%)
Musculoskeletal and connective tissue infections and inflammations NEC	1 (<0.1%)	2 (0.1%)	3 (0.1%)
Plantar fasciitis	1 (<0.1%)	2 (0.1%)	3 (0.1%)
Musculoskeletal and connective tissue pain and discomfort	31 (2.2%)	37 (2.5%)	68 (2.4%)
Back pain	22 (1.5%)	32 (2.2%)	54 (1.9%)
Musculoskeletal discomfort	1 (<0.1%)	0	1 (<0.1%)
Musculoskeletal pain	4 (0.3%)	4 (0.3%)	8 (0.3%)
Neck pain	1 (<0.1%)	1 (<0.1%)	2 (<0.1%)
Pain in extremity	4 (0.3%)	3 (0.2%)	7 (0.2%)
Musculoskeletal and connective tissue signs and symptoms NEC	1 (<0.1%)	0	1 (<0.1%)
Back disorder	1 (<0.1%)	0	1 (<0.1%)
Osteoarthropathies	1 (<0.1%)	3 (0.2%)	4 (0.1%)
Osteoarthritis	1 (<0.1%)	2 (0.1%)	3 (0.1%)
Spinal osteoarthritis	0	1 (<0.1%)	1 (<0.1%)
Rheumatoid arthropathies	2 (0.1%)	2 (0.1%)	4 (0.1%)
Juvenile arthritis	2 (0.1%)	1 (<0.1%)	3 (0.1%)
Rheumatoid arthritis	0	1 (<0.1%)	1 (<0.1%)

Table 14.1.1 / 26: Number of subjects with medical history findings by primary system organ class, high level term, preferred term by treatment (FAS)

Primary system organ class<br>High level term<br>Preferred term<br>MedDRA Version 14.0	LCS12<br>N=1432 (100%)	LCS16<br>N=1452 (100%)	Total<br>N=2884 (100%)
Soft tissue disorders NEC	0	1 (<0.1%)	1 (<0.1%)
Neck mass	0	1 (<0.1%)	1 (<0.1%)
Spine and neck deformities	2 (0.1%)	4 (0.3%)	6 (0.2%)
Scoliosis	2 (0.1%)	4 (0.3%)	6 (0.2%)
Synovial disorders	1 (<0.1%)	3 (0.2%)	4 (0.1%)
Synovial cyst	1 (<0.1%)	2 (0.1%)	3 (0.1%)
Tenosynovitis stenosans	0	1 (<0.1%)	1 (<0.1%)
Tendon disorders	4 (0.3%)	3 (0.2%)	7 (0.2%)
Tendonitis	3 (0.2%)	2 (0.1%)	5 (0.2%)
Tenosynovitis	1 (<0.1%)	0	1 (<0.1%)
Trigger finger	0	1 (<0.1%)	1 (<0.1%)
Neoplasms benign, malignant and unspecified (incl cysts and polyps)	41 (2.9%)	54 (3.7%)	95 (3.3%)
Bone neoplasms benign (excl cysts)	2 (0.1%)	0	2 (<0.1%)
Benign bone neoplasm	1 (<0.1%)	0	1 (<0.1%)
Bone giant cell tumour benign	1 (<0.1%)	0	1 (<0.1%)
Bone neoplasms unspecified malignancy	1 (<0.1%)	0	1 (<0.1%)
Bone neoplasm	1 (<0.1%)	0	1 (<0.1%)
Breast and nipple neoplasms benign	9 (0.6%)	6 (0.4%)	15 (0.5%)
Benign breast neoplasm	0	5 (0.3%)	5 (0.2%)
Breast fibroma	0	1 (<0.1%)	1 (<0.1%)
Fibroadenoma of breast	8 (0.6%)	0	8 (0.3%)
Lipoma of breast	1 (<0.1%)	0	1 (<0.1%)
Cervix neoplasms benign	13 (0.9%)	16 (1.1%)	29 (1.0%)
Benign neoplasm of cervix uteri	1 (<0.1%)	0	1 (<0.1%)
Cervicitis human papilloma virus	12 (0.8%)	16 (1.1%)	28 (1.0%)
Leukaemias acute lymphocytic	0	1 (<0.1%)	1 (<0.1%)
Acute lymphocytic leukaemia	0	1 (<0.1%)	1 (<0.1%)
Lip and oral cavity neoplasms benign	1 (<0.1%)	2 (0.1%)	3 (0.1%)
Ameloblastoma	0	1 (<0.1%)	1 (<0.1%)
Benign salivary gland neoplasm	1 (<0.1%)	1 (<0.1%)	2 (<0.1%)
Neoplasms benign site unspecified NEC	0	2 (0.1%)	2 (<0.1%)
Adenoma benign	0	1 (<0.1%)	1 (<0.1%)
Benign neoplasm	0	1 (<0.1%)	1 (<0.1%)
Ovarian neoplasms benign	4 (0.3%)	3 (0.2%)	7 (0.2%)
Ovarian adenoma	1 (<0.1%)	0	1 (<0.1%)
Ovarian fibroma	1 (<0.1%)	0	1 (<0.1%)
Ovarian germ cell teratoma benign	2 (0.1%)	3 (0.2%)	5 (0.2%)
Reproductive neoplasms female benign NEC	0	1 (<0.1%)	1 (<0.1%)
Benign hydatidiform mole	0	1 (<0.1%)	1 (<0.1%)

Table 14.1.1 / 26: Number of subjects with medical history findings by primary system organ class, high level term, preferred term by treatment (FAS)

Primary system organ class<br>High level term<br>Preferred term<br>MedDRA Version 14.0	LCS12<br>N=1432 (100%)	LCS16<br>N=1452 (100%)	Total<br>N=2884 (100%)
Skin neoplasms benign	4 (0.3%)	6 (0.4%)	10 (0.3%)
Acrochordon	0	1 (<0.1%)	1 (<0.1%)
Benign neoplasm of skin	2 (0.1%)	2 (0.1%)	4 (0.1%)
Haemangioma of skin	1 (<0.1%)	1 (<0.1%)	2 (<0.1%)
Juvenile melanoma benign	0	1 (<0.1%)	1 (<0.1%)
Melanocytic naevus	1 (<0.1%)	0	1 (<0.1%)
Skin papilloma	0	1 (<0.1%)	1 (<0.1%)
Soft tissue neoplasms benign NEC	3 (0.2%)	0	3 (0.1%)
Glomus tumour	1 (<0.1%)	0	1 (<0.1%)
Lipoma	2 (0.1%)	0	2 (<0.1%)
Thyroid neoplasms benign	1 (<0.1%)	0	1 (<0.1%)
Benign neoplasm of thyroid gland	1 (<0.1%)	0	1 (<0.1%)
Upper gastrointestinal neoplasms benign	0	1 (<0.1%)	1 (<0.1%)
Benign gastric neoplasm	0	1 (<0.1%)	1 (<0.1%)
Urinary tract neoplasms benign	1 (<0.1%)	0	1 (<0.1%)
Benign neoplasm of urethra	1 (<0.1%)	0	1 (<0.1%)
Uterine neoplasms benign	0	4 (0.3%)	4 (0.1%)
Uterine leiomyoma	0	4 (0.3%)	4 (0.1%)
Vaginal neoplasms benign	3 (0.2%)	12 (0.8%)	15 (0.5%)
Vulvovaginal human papilloma virus infection	3 (0.2%)	12 (0.8%)	15 (0.5%)
Nervous system disorders	247 (17.2%)	264 (18.2%)	511 (17.7%)
Acute polyneuropathies	0	1 (<0.1%)	1 (<0.1%)
Guillain-Barre syndrome	0	1 (<0.1%)	1 (<0.1%)
Central nervous system haemorrhages and cerebrovascular accidents	1 (<0.1%)	1 (<0.1%)	2 (<0.1%)
Cerebral infarction	0	1 (<0.1%)	1 (<0.1%)
Cerebrovascular accident	1 (<0.1%)	0	1 (<0.1%)
Coma states	0	1 (<0.1%)	1 (<0.1%)
Coma	0	1 (<0.1%)	1 (<0.1%)
Cortical dysfunction NEC	1 (<0.1%)	0	1 (<0.1%)
Central auditory processing disorder	1 (<0.1%)	0	1 (<0.1%)
Disturbances in consciousness NEC	2 (0.1%)	1 (<0.1%)	3 (0.1%)
Syncope	2 (0.1%)	1 (<0.1%)	3 (0.1%)
Dystonias	2 (0.1%)	0	2 (<0.1%)
Myotonia	2 (0.1%)	0	2 (<0.1%)
Headaches NEC	170 (11.9%)	181 (12.5%)	351 (12.2%)
Cervicogenic headache	1 (<0.1%)	0	1 (<0.1%)
Cluster headache	2 (0.1%)	0	2 (<0.1%)
Headache	138 (9.6%)	152 (10.5%)	290 (10.1%)
Sinus headache	4 (0.3%)	3 (0.2%)	7 (0.2%)
Tension headache	27 (1.9%)	26 (1.8%)	53 (1.8%)
Vascular headache	0	1 (<0.1%)	1 (<0.1%)

Table 14.1.1 / 26: Number of subjects with medical history findings by primary system organ class, high level term, preferred term by treatment (FAS)

Primary system organ class<br>High level term<br>Preferred term<br>MedDRA Version 14.0	LCS12<br>N=1432 (100%)	LCS16<br>N=1452 (100%)	Total<br>N=2884 (100%)
Increased intracranial pressure disorders	1 (<0.1%)	1 (<0.1%)	2 (<0.1%)
Benign intracranial hypertension	1 (<0.1%)	1 (<0.1%)	2 (<0.1%)
Lumbar spinal cord and nerve root disorders	2 (0.1%)	0	2 (<0.1%)
Sciatica	2 (0.1%)	0	2 (<0.1%)
Memory loss (excl dementia)	0	1 (<0.1%)	1 (<0.1%)
Memory impairment	0	1 (<0.1%)	1 (<0.1%)
Migraine headaches	83 (5.8%)	85 (5.9%)	168 (5.8%)
Migraine	76 (5.3%)	79 (5.4%)	155 (5.4%)
Migraine with aura	6 (0.4%)	6 (0.4%)	12 (0.4%)
Migraine without aura	1 (<0.1%)	0	1 (<0.1%)
Mononeuropathies	2 (0.1%)	3 (0.2%)	5 (0.2%)
Carpal tunnel syndrome	2 (0.1%)	2 (0.1%)	4 (0.1%)
Nerve compression	0	1 (<0.1%)	1 (<0.1%)
Muscle tone abnormal	1 (<0.1%)	0	1 (<0.1%)
Hypertonia	1 (<0.1%)	0	1 (<0.1%)
Neurologic visual problems NEC	1 (<0.1%)	0	1 (<0.1%)
Hemianopia	1 (<0.1%)	0	1 (<0.1%)
Neurological signs and symptoms NEC	0	1 (<0.1%)	1 (<0.1%)
Dizziness	0	1 (<0.1%)	1 (<0.1%)
Optic nerve disorders NEC	1 (<0.1%)	0	1 (<0.1%)
Optic neuritis	1 (<0.1%)	0	1 (<0.1%)
Paraesthesias and dysaesthesias	0	1 (<0.1%)	1 (<0.1%)
Paraesthesia	0	1 (<0.1%)	1 (<0.1%)
Paralysis and paresis (excl cranial nerve)	1 (<0.1%)	0	1 (<0.1%)
Paraparesis	1 (<0.1%)	0	1 (<0.1%)
Seizures and seizure disorders NEC	5 (0.3%)	9 (0.6%)	14 (0.5%)
Convulsion	2 (0.1%)	5 (0.3%)	7 (0.2%)
Epilepsy	2 (0.1%)	4 (0.3%)	6 (0.2%)
Febrile convulsion	1 (<0.1%)	0	1 (<0.1%)
Sensory abnormalities NEC	2 (0.1%)	1 (<0.1%)	3 (0.1%)
Complex regional pain syndrome	1 (<0.1%)	0	1 (<0.1%)
Restless legs syndrome	1 (<0.1%)	1 (<0.1%)	2 (<0.1%)
Pregnancy, puerperium and perinatal conditions	78 (5.4%)	88 (6.1%)	166 (5.8%)
Abortions not specified as induced or spontaneous	28 (2.0%)	26 (1.8%)	54 (1.9%)
Abortion	27 (1.9%)	25 (1.7%)	52 (1.8%)
Abortion incomplete	1 (<0.1%)	0	1 (<0.1%)
Abortion missed	0	1 (<0.1%)	1 (<0.1%)
Abortions spontaneous	16 (1.1%)	21 (1.4%)	37 (1.3%)
Abortion spontaneous	16 (1.1%)	20 (1.4%)	36 (1.2%)
Abortion spontaneous incomplete	0	1 (<0.1%)	1 (<0.1%)

Table 14.1.1 / 26: Number of subjects with medical history findings by primary system organ class, high level term, preferred term by treatment (FAS)

Primary system organ class<br>High level term<br>Preferred term<br>MedDRA Version 14.0	LCS12<br>N=1432 (100%)	LCS16<br>N=1452 (100%)	Total<br>N=2884 (100%)
Failed labour	1 (<0.1%)	0	1 (<0.1%)
Arrested labour	1 (<0.1%)	0	1 (<0.1%)
Foetal complications NEC	3 (0.2%)	1 (<0.1%)	4 (0.1%)
Foetal distress syndrome	3 (0.2%)	1 (<0.1%)	4 (0.1%)
Haemorrhagic complications of pregnancy	3 (0.2%)	4 (0.3%)	7 (0.2%)
Antepartum haemorrhage	2 (0.1%)	3 (0.2%)	5 (0.2%)
Placenta praevia haemorrhage	1 (<0.1%)	0	1 (<0.1%)
Premature separation of placenta	0	1 (<0.1%)	1 (<0.1%)
Hypertension associated disorders of pregnancy	7 (0.5%)	9 (0.6%)	16 (0.6%)
Gestational hypertension	2 (0.1%)	4 (0.3%)	6 (0.2%)
HELLP syndrome	1 (<0.1%)	0	1 (<0.1%)
Pre-eclampsia	4 (0.3%)	5 (0.3%)	9 (0.3%)
Labour onset and length abnormalities	1 (<0.1%)	0	1 (<0.1%)
Premature labour	1 (<0.1%)	0	1 (<0.1%)
Maternal complications of delivery NEC	0	1 (<0.1%)	1 (<0.1%)
Retained placenta or membranes	0	1 (<0.1%)	1 (<0.1%)
Maternal complications of pregnancy NEC	2 (0.1%)	0	2 (<0.1%)
Cervical incompetence	1 (<0.1%)	0	1 (<0.1%)
Ectopic pregnancy	1 (<0.1%)	0	1 (<0.1%)
Multiple pregnancies	1 (<0.1%)	1 (<0.1%)	2 (<0.1%)
Twin pregnancy	1 (<0.1%)	1 (<0.1%)	2 (<0.1%)
Normal pregnancy, labour and delivery	21 (1.5%)	31 (2.1%)	52 (1.8%)
Delivery	21 (1.5%)	31 (2.1%)	52 (1.8%)
Placental abnormalities (excl neoplasms)	1 (<0.1%)	0	1 (<0.1%)
Placental insufficiency	1 (<0.1%)	0	1 (<0.1%)
Postpartum complications NEC	1 (<0.1%)	3 (0.2%)	4 (0.1%)
Postpartum haemorrhage	1 (<0.1%)	3 (0.2%)	4 (0.1%)
Stillbirth and foetal death	0	1 (<0.1%)	1 (<0.1%)
Stillbirth	0	1 (<0.1%)	1 (<0.1%)
Unintended pregnancies	1 (<0.1%)	1 (<0.1%)	2 (<0.1%)
Pregnancy with contraceptive device	1 (<0.1%)	1 (<0.1%)	2 (<0.1%)
Psychiatric disorders	90 (6.3%)	101 (7.0%)	191 (6.6%)
Anxiety disorders NEC	2 (0.1%)	1 (<0.1%)	3 (0.1%)
Anxiety disorder	1 (<0.1%)	1 (<0.1%)	2 (<0.1%)
Generalised anxiety disorder	1 (<0.1%)	0	1 (<0.1%)
Anxiety symptoms	24 (1.7%)	29 (2.0%)	53 (1.8%)
Anxiety	24 (1.7%)	28 (1.9%)	52 (1.8%)
Stress	1 (<0.1%)	1 (<0.1%)	2 (<0.1%)
Attention deficit and disruptive behaviour disorders	2 (0.1%)	4 (0.3%)	6 (0.2%)
Attention deficit/hyperactivity disorder	2 (0.1%)	4 (0.3%)	6 (0.2%)

Table 14.1.1 / 26: Number of subjects with medical history findings by primary system organ class, high level term, preferred term by treatment (FAS)

Primary system organ class<br>High level term<br>Preferred term<br>MedDRA Version 14.0	LCS12<br>N=1432 (100%)	LCS16<br>N=1452 (100%)	Total<br>N=2884 (100%)
Bipolar disorders	0	3 (0.2%)	3 (0.1%)
Bipolar disorder	0	3 (0.2%)	3 (0.1%)
Brief psychotic disorder	0	1 (<0.1%)	1 (<0.1%)
Brief psychotic disorder, with postpartum onset	0	1 (<0.1%)	1 (<0.1%)
Depressive disorders	46 (3.2%)	39 (2.7%)	85 (2.9%)
Depression	42 (2.9%)	32 (2.2%)	74 (2.6%)
Major depression	1 (<0.1%)	0	1 (<0.1%)
Postpartum depression	3 (0.2%)	8 (0.6%)	11 (0.4%)
Disturbances in initiating and maintaining sleep	12 (0.8%)	20 (1.4%)	32 (1.1%)
Insomnia	12 (0.8%)	20 (1.4%)	32 (1.1%)
Eating disorders NEC	3 (0.2%)	2 (0.1%)	5 (0.2%)
Bulimia nervosa	1 (<0.1%)	2 (0.1%)	3 (0.1%)
Eating disorder	2 (0.1%)	0	2 (<0.1%)
Emotional and mood disturbances NEC	1 (<0.1%)	0	1 (<0.1%)
Mood altered	1 (<0.1%)	0	1 (<0.1%)
Fear symptoms and phobic disorders (incl social phobia)	1 (<0.1%)	1 (<0.1%)	2 (<0.1%)
Phobia of flying	1 (<0.1%)	1 (<0.1%)	2 (<0.1%)
Fluctuating mood symptoms	2 (0.1%)	1 (<0.1%)	3 (0.1%)
Mood swings	2 (0.1%)	1 (<0.1%)	3 (0.1%)
Mood disorders NEC	1 (<0.1%)	1 (<0.1%)	2 (<0.1%)
Seasonal affective disorder	1 (<0.1%)	1 (<0.1%)	2 (<0.1%)
Orgasmic disorders and disturbances	0	1 (<0.1%)	1 (<0.1%)
Anorgasmia	0	1 (<0.1%)	1 (<0.1%)
Panic attacks and disorders	3 (0.2%)	6 (0.4%)	9 (0.3%)
Panic attack	2 (0.1%)	5 (0.3%)	7 (0.2%)
Panic disorder	1 (<0.1%)	1 (<0.1%)	2 (<0.1%)
Personality disorders with dramatic behaviour (Cluster B)	0	1 (<0.1%)	1 (<0.1%)
Borderline personality disorder	0	1 (<0.1%)	1 (<0.1%)
Sexual desire disorders	3 (0.2%)	4 (0.3%)	7 (0.2%)
Libido decreased	3 (0.2%)	4 (0.3%)	7 (0.2%)
Sleep disorders NEC	2 (0.1%)	1 (<0.1%)	3 (0.1%)
Sleep disorder	2 (0.1%)	1 (<0.1%)	3 (0.1%)
Somatoform disorders	1 (<0.1%)	0	1 (<0.1%)
Hypochondriasis	1 (<0.1%)	0	1 (<0.1%)
Stress disorders	1 (<0.1%)	0	1 (<0.1%)
Burnout syndrome	1 (<0.1%)	0	1 (<0.1%)
Substance-related disorders	1 (<0.1%)	4 (0.3%)	5 (0.2%)
Alcohol abuse	0	2 (0.1%)	2 (<0.1%)
Alcoholism	0	1 (<0.1%)	1 (<0.1%)
Drug abuse	0	4 (0.3%)	4 (0.1%)
Nicotine dependence	1 (<0.1%)	0	1 (<0.1%)

Table 14.1.1 / 26: Number of subjects with medical history findings by primary system organ class, high level term, preferred term by treatment (FAS)

Primary system organ class<br>High level term<br>Preferred term<br>MedDRA Version 14.0	LCS12<br>N=1432 (100%)	LCS16<br>N=1452 (100%)	Total<br>N=2884 (100%)
Renal and urinary disorders	23 (1.6%)	27 (1.9%)	50 (1.7%)
Bladder and urethral symptoms	8 (0.6%)	8 (0.6%)	16 (0.6%)
Dysuria	1 (<0.1%)	1 (<0.1%)	2 (<0.1%)
Incontinence	0	1 (<0.1%)	1 (<0.1%)
Micturition urgency	1 (<0.1%)	1 (<0.1%)	2 (<0.1%)
Pollakiuria	1 (<0.1%)	1 (<0.1%)	2 (<0.1%)
Stress urinary incontinence	4 (0.3%)	3 (0.2%)	7 (0.2%)
Urinary incontinence	1 (<0.1%)	0	1 (<0.1%)
Urinary retention	0	1 (<0.1%)	1 (<0.1%)
Bladder infections and inflammations	1 (<0.1%)	1 (<0.1%)	2 (<0.1%)
Cystitis interstitial	1 (<0.1%)	1 (<0.1%)	2 (<0.1%)
Bladder neoplasms	1 (<0.1%)	0	1 (<0.1%)
Bladder cyst	1 (<0.1%)	0	1 (<0.1%)
Genital and urinary tract disorders NEC	1 (<0.1%)	0	1 (<0.1%)
Urinary tract disorder	1 (<0.1%)	0	1 (<0.1%)
Genitourinary tract infections and inflammations NEC	0	1 (<0.1%)	1 (<0.1%)
Urinary tract inflammation	0	1 (<0.1%)	1 (<0.1%)
Renal disorders NEC	0	1 (<0.1%)	1 (<0.1%)
Renal disorder in pregnancy	0	1 (<0.1%)	1 (<0.1%)
Renal lithiasis	9 (0.6%)	14 (1.0%)	23 (0.8%)
Nephrolithiasis	9 (0.6%)	14 (1.0%)	23 (0.8%)
Renal obstructive disorders	1 (<0.1%)	0	1 (<0.1%)
Hydronephrosis	1 (<0.1%)	0	1 (<0.1%)
Urinary abnormalities	1 (<0.1%)	0	1 (<0.1%)
Haematuria	1 (<0.1%)	0	1 (<0.1%)
Urinary tract signs and symptoms NEC	1 (<0.1%)	3 (0.2%)	4 (0.1%)
Renal colic	1 (<0.1%)	3 (0.2%)	4 (0.1%)
Reproductive system and breast disorders	240 (16.8%)	243 (16.7%)	483 (16.7%)
Benign and malignant breast neoplasms	10 (0.7%)	9 (0.6%)	19 (0.7%)
Breast cyst	4 (0.3%)	2 (0.1%)	6 (0.2%)
Fibrocystic breast disease	6 (0.4%)	7 (0.5%)	13 (0.5%)
Breast disorders NEC	6 (0.4%)	3 (0.2%)	9 (0.3%)
Breast calcifications	0	1 (<0.1%)	1 (<0.1%)
Breast enlargement	1 (<0.1%)	0	1 (<0.1%)
Breast mass	4 (0.3%)	2 (0.1%)	6 (0.2%)
Mastoptosis	1 (<0.1%)	0	1 (<0.1%)
Breast signs and symptoms	3 (0.2%)	3 (0.2%)	6 (0.2%)
Breast discomfort	1 (<0.1%)	1 (<0.1%)	2 (<0.1%)
Breast pain	2 (0.1%)	2 (0.1%)	4 (0.1%)

Table 14.1.1 / 26: Number of subjects with medical history findings by primary system organ class, high level term, preferred term by treatment (FAS)

Primary system organ class<br>High level term<br>Preferred term<br>MedDRA Version 14.0	LCS12<br>N=1432 (100%)	LCS16<br>N=1452 (100%)	Total<br>N=2884 (100%)
Cervix disorders NEC	41 (2.9%)	45 (3.1%)	86 (3.0%)
Cervical dysplasia	41 (2.9%)	43 (3.0%)	84 (2.9%)
Uterine cervical squamous metaplasia	0	1 (<0.1%)	1 (<0.1%)
Uterine cervix stenosis	0	1 (<0.1%)	1 (<0.1%)
Cervix neoplasms	1 (<0.1%)	0	1 (<0.1%)
Cervical cyst	1 (<0.1%)	0	1 (<0.1%)
Lactation disorders	1 (<0.1%)	1 (<0.1%)	2 (<0.1%)
Galactorrhoea	1 (<0.1%)	1 (<0.1%)	2 (<0.1%)
Menstruation and uterine bleeding NEC	131 (9.1%)	135 (9.3%)	266 (9.2%)
Dysfunctional uterine bleeding	0	1 (<0.1%)	1 (<0.1%)
Dysmenorrhoea	121 (8.4%)	128 (8.8%)	249 (8.6%)
Menstruation irregular	5 (0.3%)	2 (0.1%)	7 (0.2%)
Metrorrhagia	2 (0.1%)	4 (0.3%)	6 (0.2%)
Premenstrual syndrome	10 (0.7%)	3 (0.2%)	13 (0.5%)
Menstruation with decreased bleeding	3 (0.2%)	2 (0.1%)	5 (0.2%)
Amenorrhoea	3 (0.2%)	2 (0.1%)	5 (0.2%)
Menstruation with increased bleeding	16 (1.1%)	14 (1.0%)	30 (1.0%)
Menometrorrhagia	2 (0.1%)	0	2 (<0.1%)
Menorrhagia	14 (1.0%)	13 (0.9%)	27 (0.9%)
Polymenorrhagia	0	1 (<0.1%)	1 (<0.1%)
Ovarian and fallopian tube cysts and neoplasms	30 (2.1%)	32 (2.2%)	62 (2.1%)
Fallopian tube cyst	2 (0.1%)	0	2 (<0.1%)
Haemorrhagic ovarian cyst	0	1 (<0.1%)	1 (<0.1%)
Ovarian cyst	22 (1.5%)	26 (1.8%)	48 (1.7%)
Ovarian cyst ruptured	2 (0.1%)	1 (<0.1%)	3 (0.1%)
Polycystic ovaries	5 (0.3%)	4 (0.3%)	9 (0.3%)
Ovarian and fallopian tube disorders NEC	0	1 (<0.1%)	1 (<0.1%)
Fallopian tube obstruction	0	1 (<0.1%)	1 (<0.1%)
Pelvic prolapse conditions	1 (<0.1%)	0	1 (<0.1%)
Pelvic prolapse	1 (<0.1%)	0	1 (<0.1%)
Pelvis and broad ligament disorders NEC	0	1 (<0.1%)	1 (<0.1%)
Pelvic adhesions	0	1 (<0.1%)	1 (<0.1%)
Reproductive tract signs and symptoms NEC	3 (0.2%)	8 (0.6%)	11 (0.4%)
Genital discharge	0	1 (<0.1%)	1 (<0.1%)
Pelvic discomfort	1 (<0.1%)	0	1 (<0.1%)
Pelvic pain	2 (0.1%)	7 (0.5%)	9 (0.3%)
Scrotal disorders NEC	1 (<0.1%)	0	1 (<0.1%)
Varicocele	1 (<0.1%)	0	1 (<0.1%)

Table 14.1.1 / 26: Number of subjects with medical history findings by primary system organ class, high level term, preferred term by treatment (FAS)

Primary system organ class<br>High level term<br>Preferred term<br>MedDRA Version 14.0	LCS12<br>N=1432 (100%)	LCS16<br>N=1452 (100%)	Total<br>N=2884 (100%)
Sexual function and fertility disorders NEC	10 (0.7%)	10 (0.7%)	20 (0.7%)
Dyspareunia	7 (0.5%)	9 (0.6%)	16 (0.6%)
Infertility	2 (0.1%)	0	2 (<0.1%)
Infertility female	1 (<0.1%)	0	1 (<0.1%)
Infertility male	0	1 (<0.1%)	1 (<0.1%)
Uterine disorders NEC	12 (0.8%)	18 (1.2%)	30 (1.0%)
Adenomyosis	0	1 (<0.1%)	1 (<0.1%)
Endometriosis	12 (0.8%)	16 (1.1%)	28 (1.0%)
Uterine haemorrhage	0	1 (<0.1%)	1 (<0.1%)
Uterine neoplasms	0	1 (<0.1%)	1 (<0.1%)
Uterine polyp	0	1 (<0.1%)	1 (<0.1%)
Uterine tone disorders	2 (0.1%)	2 (0.1%)	4 (0.1%)
Uterine atony	0	1 (<0.1%)	1 (<0.1%)
Uterine spasm	2 (0.1%)	1 (<0.1%)	3 (0.1%)
Vaginal and vulval infections and inflammations	2 (0.1%)	0	2 (<0.1%)
Bartholinitis	2 (0.1%)	0	2 (<0.1%)
Vulvovaginal cysts and neoplasms	1 (<0.1%)	1 (<0.1%)	2 (<0.1%)
Vaginal cyst	1 (<0.1%)	0	1 (<0.1%)
Vulva cyst	0	1 (<0.1%)	1 (<0.1%)
Vulvovaginal disorders NEC	8 (0.6%)	5 (0.3%)	13 (0.5%)
Vaginal disorder	3 (0.2%)	0	3 (0.1%)
Vaginal haemorrhage	5 (0.3%)	2 (0.1%)	7 (0.2%)
Vaginal stricture	0	1 (<0.1%)	1 (<0.1%)
Vulval disorder	0	1 (<0.1%)	1 (<0.1%)
Vulval leukoplakia	0	1 (<0.1%)	1 (<0.1%)
Vulvovaginal signs and symptoms	8 (0.6%)	6 (0.4%)	14 (0.5%)
Coital bleeding	2 (0.1%)	0	2 (<0.1%)
Haematocolpos	1 (<0.1%)	0	1 (<0.1%)
Labia enlarged	0	1 (<0.1%)	1 (<0.1%)
Vaginal discharge	1 (<0.1%)	4 (0.3%)	5 (0.2%)
Vaginal lesion	0	1 (<0.1%)	1 (<0.1%)
Vulvovaginal erythema	1 (<0.1%)	0	1 (<0.1%)
Vulvovaginal pain	3 (0.2%)	0	3 (0.1%)
Respiratory, thoracic and mediastinal disorders	87 (6.1%)	97 (6.7%)	184 (6.4%)
Breathing abnormalities	1 (<0.1%)	1 (<0.1%)	2 (<0.1%)
Dyspnoea	1 (<0.1%)	0	1 (<0.1%)
Hyperventilation	0	1 (<0.1%)	1 (<0.1%)

Table 14.1.1 / 26: Number of subjects with medical history findings by primary system organ class, high level term, preferred term by treatment (FAS)

Primary system organ class<br>High level term<br>Preferred term<br>MedDRA Version 14.0	LCS12<br>N=1432 (100%)	LCS16<br>N=1452 (100%)	Total<br>N=2884 (100%)
Bronchospasm and obstruction	50 (3.5%)	58 (4.0%)	108 (3.7%)
Asthma	39 (2.7%)	51 (3.5%)	90 (3.1%)
Asthma exercise induced	5 (0.3%)	6 (0.4%)	11 (0.4%)
Asthmatic crisis	1 (<0.1%)	0	1 (<0.1%)
Bronchitis chronic	4 (0.3%)	0	4 (0.1%)
Bronchospasm	1 (<0.1%)	0	1 (<0.1%)
Infantile asthma	1 (<0.1%)	1 (<0.1%)	2 (<0.1%)
Coughing and associated symptoms	3 (0.2%)	1 (<0.1%)	4 (0.1%)
Cough	3 (0.2%)	1 (<0.1%)	4 (0.1%)
Productive cough	1 (<0.1%)	0	1 (<0.1%)
Laryngeal and adjacent sites disorders NEC (excl infections and neoplasms)	1 (<0.1%)	0	1 (<0.1%)
Vocal cord thickening	1 (<0.1%)	0	1 (<0.1%)
Nasal congestion and inflammations	21 (1.5%)	21 (1.4%)	42 (1.5%)
Nasal congestion	0	1 (<0.1%)	1 (<0.1%)
Rhinitis allergic	10 (0.7%)	11 (0.8%)	21 (0.7%)
Rhinitis perennial	0	1 (<0.1%)	1 (<0.1%)
Rhinitis seasonal	11 (0.8%)	9 (0.6%)	20 (0.7%)
Nasal disorders NEC	5 (0.3%)	6 (0.4%)	11 (0.4%)
Epistaxis	0	2 (0.1%)	2 (<0.1%)
Nasal septum deviation	5 (0.3%)	4 (0.3%)	9 (0.3%)
Paranasal sinus disorders (excl infections and neoplasms)	2 (0.1%)	6 (0.4%)	8 (0.3%)
Allergic sinusitis	1 (<0.1%)	0	1 (<0.1%)
Sinus congestion	1 (<0.1%)	5 (0.3%)	6 (0.2%)
Sinus disorder	0	1 (<0.1%)	1 (<0.1%)
Pharyngeal disorders (excl infections and neoplasms)	2 (0.1%)	0	2 (<0.1%)
Adenoidal disorder	1 (<0.1%)	0	1 (<0.1%)
Tonsillar hypertrophy	1 (<0.1%)	0	1 (<0.1%)
Pneumothorax and pleural effusions NEC	1 (<0.1%)	0	1 (<0.1%)
Pneumothorax	1 (<0.1%)	0	1 (<0.1%)
Respiratory tract disorders NEC	1 (<0.1%)	0	1 (<0.1%)
Chronic respiratory disease	1 (<0.1%)	0	1 (<0.1%)
Upper respiratory tract signs and symptoms	4 (0.3%)	7 (0.5%)	11 (0.4%)
Dysphonia	0	1 (<0.1%)	1 (<0.1%)
Oropharyngeal pain	3 (0.2%)	5 (0.3%)	8 (0.3%)
Rhinorrhoea	1 (<0.1%)	0	1 (<0.1%)
Throat irritation	0	1 (<0.1%)	1 (<0.1%)
Skin and subcutaneous tissue disorders	92 (6.4%)	80 (5.5%)	172 (6.0%)
Acnes	42 (2.9%)	46 (3.2%)	88 (3.1%)
Acne	41 (2.9%)	45 (3.1%)	86 (3.0%)
Acne cystic	1 (<0.1%)	1 (<0.1%)	2 (<0.1%)

Table 14.1.1 / 26: Number of subjects with medical history findings by primary system organ class, high level term, preferred term by treatment (FAS)

Primary system organ class<br>High level term<br>Preferred term<br>MedDRA Version 14.0	LCS12<br>N=1432 (100%)	LCS16<br>N=1452 (100%)	Total<br>N=2884 (100%)
Alopecias	1 (<0.1%)	0	1 (<0.1%)
Alopecia	1 (<0.1%)	0	1 (<0.1%)
Apocrine and eccrine gland disorders	3 (0.2%)	1 (<0.1%)	4 (0.1%)
Heat rash	0	1 (<0.1%)	1 (<0.1%)
Hyperhidrosis	2 (0.1%)	0	2 (<0.1%)
Night sweats	1 (<0.1%)	1 (<0.1%)	2 (<0.1%)
Dermal and epidermal conditions NEC	2 (0.1%)	1 (<0.1%)	3 (0.1%)
Dry skin	2 (0.1%)	0	2 (<0.1%)
Skin fissures	0	1 (<0.1%)	1 (<0.1%)
Dermatitis and eczema	28 (2.0%)	20 (1.4%)	48 (1.7%)
Dermatitis	1 (<0.1%)	0	1 (<0.1%)
Dermatitis allergic	1 (<0.1%)	0	1 (<0.1%)
Dermatitis atopic	9 (0.6%)	6 (0.4%)	15 (0.5%)
Dermatitis contact	2 (0.1%)	5 (0.3%)	7 (0.2%)
Eczema	16 (1.1%)	9 (0.6%)	25 (0.9%)
Granulomatous and deep cutaneous inflammatory conditions	1 (<0.1%)	0	1 (<0.1%)
Granuloma annulare	1 (<0.1%)	0	1 (<0.1%)
Hyperkeratoses	1 (<0.1%)	1 (<0.1%)	2 (<0.1%)
Hyperkeratosis	1 (<0.1%)	0	1 (<0.1%)
Keratosis pilaris	0	1 (<0.1%)	1 (<0.1%)
Hyperpigmentation disorders	0	1 (<0.1%)	1 (<0.1%)
Chloasma	0	1 (<0.1%)	1 (<0.1%)
Hypertrichoses	1 (<0.1%)	2 (0.1%)	3 (0.1%)
Hirsutism	1 (<0.1%)	1 (<0.1%)	2 (<0.1%)
Hypertrichosis	0	1 (<0.1%)	1 (<0.1%)
Hypopigmentation disorders	1 (<0.1%)	0	1 (<0.1%)
Vitiligo	1 (<0.1%)	0	1 (<0.1%)
Nail and nail bed conditions (excl infections and infestations)	2 (0.1%)	0	2 (<0.1%)
Ingrowing nail	2 (0.1%)	0	2 (<0.1%)
Panniculitides	1 (<0.1%)	0	1 (<0.1%)
Erythema nodosum	1 (<0.1%)	0	1 (<0.1%)
Photosensitivity conditions	2 (0.1%)	0	2 (<0.1%)
Photosensitivity allergic reaction	1 (<0.1%)	0	1 (<0.1%)
Photosensitivity reaction	1 (<0.1%)	0	1 (<0.1%)
Pruritus NEC	0	2 (0.1%)	2 (<0.1%)
Pruritus	0	1 (<0.1%)	1 (<0.1%)
Pruritus generalised	0	1 (<0.1%)	1 (<0.1%)
Psoriatic conditions	6 (0.4%)	4 (0.3%)	10 (0.3%)
Psoriasis	6 (0.4%)	4 (0.3%)	10 (0.3%)
Purpura and related conditions	0	1 (<0.1%)	1 (<0.1%)
Increased tendency to bruise	0	1 (<0.1%)	1 (<0.1%)

Table 14.1.1 / 26: Number of subjects with medical history findings by primary system organ class, high level term, preferred term by treatment (FAS)

Primary system organ class<br>High level term<br>Preferred term<br>MedDRA Version 14.0	LCS12<br>N=1432 (100%)	LCS16<br>N=1452 (100%)	Total<br>N=2884 (100%)
Rosaceas	2 (0.1%)	0	2 (<0.1%)
Rosacea	2 (0.1%)	0	2 (<0.1%)
Scaly conditions	1 (<0.1%)	0	1 (<0.1%)
Pityriasis	1 (<0.1%)	0	1 (<0.1%)
Sebaceous gland disorders	0	1 (<0.1%)	1 (<0.1%)
Seborrhoea	0	1 (<0.1%)	1 (<0.1%)
Skin and subcutaneous conditions NEC	0	1 (<0.1%)	1 (<0.1%)
Skin mass	0	1 (<0.1%)	1 (<0.1%)
Skin cysts and polyps	1 (<0.1%)	0	1 (<0.1%)
Dermal cyst	1 (<0.1%)	0	1 (<0.1%)
Skin hypoplasias and atrophies	0	1 (<0.1%)	1 (<0.1%)
Skin striae	0	1 (<0.1%)	1 (<0.1%)
Skin injuries and mechanical dermatoses	4 (0.3%)	0	4 (0.1%)
Scar	4 (0.3%)	0	4 (0.1%)
Telangiectasia and related conditions	1 (<0.1%)	0	1 (<0.1%)
Telangiectasia	1 (<0.1%)	0	1 (<0.1%)
Urticarias	2 (0.1%)	3 (0.2%)	5 (0.2%)
Urticaria	2 (0.1%)	3 (0.2%)	5 (0.2%)
Social circumstances	16 (1.1%)	17 (1.2%)	33 (1.1%)
Disability issues	6 (0.4%)	7 (0.5%)	13 (0.5%)
Corrective lens user	6 (0.4%)	5 (0.3%)	11 (0.4%)
Hearing aid user	0	1 (<0.1%)	1 (<0.1%)
Orthosis user	0	1 (<0.1%)	1 (<0.1%)
Family and partner issues	0	1 (<0.1%)	1 (<0.1%)
Familial risk factor	0	1 (<0.1%)	1 (<0.1%)
Pregnancy related circumstances	7 (0.5%)	7 (0.5%)	14 (0.5%)
Aborted pregnancy	7 (0.5%)	6 (0.4%)	13 (0.5%)
Nulliparous	0	1 (<0.1%)	1 (<0.1%)
Social issues NEC	2 (0.1%)	3 (0.2%)	5 (0.2%)
Cosmetic body piercing	1 (<0.1%)	0	1 (<0.1%)
Organ donor	0	1 (<0.1%)	1 (<0.1%)
Tattoo	2 (0.1%)	2 (0.1%)	4 (0.1%)
Tobacco use	1 (<0.1%)	0	1 (<0.1%)
Tobacco user	1 (<0.1%)	0	1 (<0.1%)
Surgical and medical procedures	616 (43.0%)	579 (39.9%)	1195 (41.4%)
Abdominal therapeutic procedures NEC	9 (0.6%)	1 (<0.1%)	10 (0.3%)
Abdominoplasty	9 (0.6%)	1 (<0.1%)	10 (0.3%)

Table 14.1.1 / 26: Number of subjects with medical history findings by primary system organ class, high level term, preferred term by treatment (FAS)

Primary system organ class<br>High level term<br>Preferred term<br>MedDRA Version 14.0	LCS12<br>N=1432 (100%)	LCS16<br>N=1452 (100%)	Total<br>N=2884 (100%)
Anorectal therapeutic procedures	4 (0.3%)	1 (<0.1%)	5 (0.2%)
Anal fissure excision	1 (<0.1%)	0	1 (<0.1%)
Anal fistula excision	1 (<0.1%)	0	1 (<0.1%)
Haemorrhoid operation	2 (0.1%)	1 (<0.1%)	3 (0.1%)
Biliary tract and gallbladder therapeutic procedures	35 (2.4%)	39 (2.7%)	74 (2.6%)
Cholecystectomy	33 (2.3%)	37 (2.5%)	70 (2.4%)
Cholelithotomy	1 (<0.1%)	0	1 (<0.1%)
Gallbladder operation	1 (<0.1%)	2 (0.1%)	3 (0.1%)
Bladder therapeutic procedures	2 (0.1%)	0	2 (<0.1%)
Bladder irrigation	1 (<0.1%)	0	1 (<0.1%)
Bladder repair	1 (<0.1%)	0	1 (<0.1%)
Blood and blood product treatment	0	1 (<0.1%)	1 (<0.1%)
Transfusion	0	1 (<0.1%)	1 (<0.1%)
Bone therapeutic procedures NEC	7 (0.5%)	6 (0.4%)	13 (0.5%)
Bone cyst excision	1 (<0.1%)	0	1 (<0.1%)
Bone graft	1 (<0.1%)	0	1 (<0.1%)
Bone lesion excision	0	2 (0.1%)	2 (<0.1%)
Bone operation	1 (<0.1%)	2 (0.1%)	3 (0.1%)
Epiphyseal stapling	1 (<0.1%)	0	1 (<0.1%)
Ostectomy	1 (<0.1%)	0	1 (<0.1%)
Osteosynthesis	2 (0.1%)	2 (0.1%)	4 (0.1%)
Osteotomy	2 (0.1%)	0	2 (<0.1%)
Removal of internal fixation	1 (<0.1%)	0	1 (<0.1%)
Breast neoplasm removal	7 (0.5%)	3 (0.2%)	10 (0.3%)
Breast cyst excision	4 (0.3%)	2 (0.1%)	6 (0.2%)
Breast lump removal	3 (0.2%)	1 (<0.1%)	4 (0.1%)
Breast therapeutic procedures NEC	48 (3.4%)	65 (4.5%)	113 (3.9%)
Breast cyst drainage	0	1 (<0.1%)	1 (<0.1%)
Breast prosthesis implantation	16 (1.1%)	21 (1.4%)	37 (1.3%)
Breast prosthesis removal	1 (<0.1%)	0	1 (<0.1%)
Breast reconstruction	1 (<0.1%)	0	1 (<0.1%)
Mammoplasty	31 (2.2%)	44 (3.0%)	75 (2.6%)
Cardiac device therapeutic procedures	0	1 (<0.1%)	1 (<0.1%)
Cardiac pacemaker insertion	0	1 (<0.1%)	1 (<0.1%)
Cardiac therapeutic procedures NEC	0	1 (<0.1%)	1 (<0.1%)
Cardiac ablation	0	1 (<0.1%)	1 (<0.1%)

Table 14.1.1 / 26: Number of subjects with medical history findings by primary system organ class, high level term, preferred term by treatment (FAS)

Primary system organ class<br>High level term<br>Preferred term<br>MedDRA Version 14.0	LCS12<br>N=1432 (100%)	LCS16<br>N=1452 (100%)	Total<br>N=2884 (100%)
Cervix therapeutic procedures	31 (2.2%)	35 (2.4%)	66 (2.3%)
Cervical conisation	3 (0.2%)	3 (0.2%)	6 (0.2%)
Cervical laser therapy	2 (0.1%)	2 (0.1%)	4 (0.1%)
Cervix cautery	4 (0.3%)	6 (0.4%)	10 (0.3%)
Cervix cerclage procedure	2 (0.1%)	1 (<0.1%)	3 (0.1%)
Cervix operation	0	2 (0.1%)	2 (<0.1%)
Endocervical curettage	1 (<0.1%)	4 (0.3%)	5 (0.2%)
Loop electrosurgical excision procedure	19 (1.3%)	17 (1.2%)	36 (1.2%)
Chest wall and mediastinal therapeutic procedures	1 (<0.1%)	0	1 (<0.1%)
Rib excision	1 (<0.1%)	0	1 (<0.1%)
Contraceptive methods female	1 (<0.1%)	0	1 (<0.1%)
Intra-uterine contraceptive device	1 (<0.1%)	0	1 (<0.1%)
Corneal and scleral therapeutic procedures	3 (0.2%)	3 (0.2%)	6 (0.2%)
Keratomileusis	3 (0.2%)	3 (0.2%)	6 (0.2%)
Dental and gingival therapeutic procedures	64 (4.5%)	63 (4.3%)	127 (4.4%)
Dental care	0	1 (<0.1%)	1 (<0.1%)
Dental operation	2 (0.1%)	2 (0.1%)	4 (0.1%)
Endodontic procedure	0	2 (0.1%)	2 (<0.1%)
Gingival graft	1 (<0.1%)	0	1 (<0.1%)
Gingival operation	0	1 (<0.1%)	1 (<0.1%)
Gingivectomy	1 (<0.1%)	0	1 (<0.1%)
Periodontal operation	0	1 (<0.1%)	1 (<0.1%)
Tooth extraction	7 (0.5%)	5 (0.3%)	12 (0.4%)
Wisdom teeth removal	55 (3.8%)	52 (3.6%)	107 (3.7%)
External ear therapeutic procedures	1 (<0.1%)	0	1 (<0.1%)
Otoplasty	1 (<0.1%)	0	1 (<0.1%)
External female genital therapeutic procedures	5 (0.3%)	4 (0.3%)	9 (0.3%)
Bartholin's cyst removal	1 (<0.1%)	1 (<0.1%)	2 (<0.1%)
Genital labial operation	1 (<0.1%)	0	1 (<0.1%)
Vulval warts removal	3 (0.2%)	2 (0.1%)	5 (0.2%)
Vulvectomy	0	1 (<0.1%)	1 (<0.1%)
Eye therapeutic procedures NEC	6 (0.4%)	5 (0.3%)	11 (0.4%)
Eye laser surgery	1 (<0.1%)	1 (<0.1%)	2 (<0.1%)
Eye operation	5 (0.3%)	3 (0.2%)	8 (0.3%)
Eyeglasses therapy	0	1 (<0.1%)	1 (<0.1%)
Facial therapeutic procedures	8 (0.6%)	5 (0.3%)	13 (0.5%)
Jaw operation	6 (0.4%)	5 (0.3%)	11 (0.4%)
Maxillofacial operation	1 (<0.1%)	0	1 (<0.1%)
Plastic surgery to the face	1 (<0.1%)	0	1 (<0.1%)
Fallopian tube therapeutic procedures	0	2 (0.1%)	2 (<0.1%)
Salpingectomy	0	1 (<0.1%)	1 (<0.1%)
Salpingo-oophorectomy unilateral	0	1 (<0.1%)	1 (<0.1%)

Table 14.1.1 / 26: Number of subjects with medical history findings by primary system organ class, high level term, preferred term by treatment (FAS)

Primary system organ class<br>High level term<br>Preferred term<br>MedDRA Version 14.0	LCS12<br>N=1432 (100%)	LCS16<br>N=1452 (100%)	Total<br>N=2884 (100%)
Fertility and fertilisation interventions female	2 (0.1%)	3 (0.2%)	5 (0.2%)
In vitro fertilisation	2 (0.1%)	3 (0.2%)	5 (0.2%)
Gastric therapeutic procedures	0	6 (0.4%)	6 (0.2%)
Gastric banding	0	1 (<0.1%)	1 (<0.1%)
Gastric bypass	0	5 (0.3%)	5 (0.2%)
Head, neck and oral cavity therapeutic procedures NEC	4 (0.3%)	1 (<0.1%)	5 (0.2%)
Ear operation	1 (<0.1%)	1 (<0.1%)	2 (<0.1%)
Oral surgery	3 (0.2%)	0	3 (0.1%)
Hernia repairs	15 (1.0%)	15 (1.0%)	30 (1.0%)
Abdominal hernia repair	2 (0.1%)	0	2 (<0.1%)
Hernia repair	0	2 (0.1%)	2 (<0.1%)
Inguinal hernia repair	8 (0.6%)	11 (0.8%)	19 (0.7%)
Umbilical hernia repair	5 (0.3%)	2 (0.1%)	7 (0.2%)
Immunisations	1 (<0.1%)	0	1 (<0.1%)
Immunisation	1 (<0.1%)	0	1 (<0.1%)
Induced abortions	18 (1.3%)	19 (1.3%)	37 (1.3%)
Abortion induced	18 (1.3%)	19 (1.3%)	37 (1.3%)
Joint therapeutic procedures	35 (2.4%)	32 (2.2%)	67 (2.3%)
Ankle operation	5 (0.3%)	2 (0.1%)	7 (0.2%)
Bunion operation	6 (0.4%)	2 (0.1%)	8 (0.3%)
Elbow operation	1 (<0.1%)	1 (<0.1%)	2 (<0.1%)
Joint dislocation reduction	1 (<0.1%)	0	1 (<0.1%)
Joint injection	0	1 (<0.1%)	1 (<0.1%)
Knee operation	7 (0.5%)	12 (0.8%)	19 (0.7%)
Ligament operation	6 (0.4%)	9 (0.6%)	15 (0.5%)
Meniscus operation	2 (0.1%)	0	2 (<0.1%)
Meniscus removal	2 (0.1%)	2 (0.1%)	4 (0.1%)
Shoulder operation	3 (0.2%)	3 (0.2%)	6 (0.2%)
Synovectomy	1 (<0.1%)	0	1 (<0.1%)
Wrist surgery	1 (<0.1%)	1 (<0.1%)	2 (<0.1%)
Large intestine therapeutic procedures	85 (5.9%)	61 (4.2%)	146 (5.1%)
Appendicectomy	84 (5.9%)	61 (4.2%)	145 (5.0%)
Proctocolectomy	1 (<0.1%)	0	1 (<0.1%)
Lens therapeutic procedures	0	1 (<0.1%)	1 (<0.1%)
Cataract operation	0	1 (<0.1%)	1 (<0.1%)
Limb therapeutic procedures	7 (0.5%)	9 (0.6%)	16 (0.6%)
Foot operation	1 (<0.1%)	3 (0.2%)	4 (0.1%)
Limb operation	4 (0.3%)	4 (0.3%)	8 (0.3%)
Toe amputation	0	1 (<0.1%)	1 (<0.1%)
Toe operation	2 (0.1%)	1 (<0.1%)	3 (0.1%)

Table 14.1.1 / 26: Number of subjects with medical history findings by primary system organ class, high level term, preferred term by treatment (FAS)

Primary system organ class<br>High level term<br>Preferred term<br>MedDRA Version 14.0	LCS12<br>N=1432 (100%)	LCS16<br>N=1452 (100%)	Total<br>N=2884 (100%)
Lymphoid tissue therapeutic procedures	2 (0.1%)	3 (0.2%)	5 (0.2%)
Lymphadenectomy	1 (<0.1%)	1 (<0.1%)	2 (<0.1%)
Lymphoid tissue operation	0	1 (<0.1%)	1 (<0.1%)
Splenectomy	1 (<0.1%)	0	1 (<0.1%)
Thymectomy	0	1 (<0.1%)	1 (<0.1%)
Mastoid therapeutic procedures	1 (<0.1%)	0	1 (<0.1%)
Mastoidectomy	1 (<0.1%)	0	1 (<0.1%)
Middle ear therapeutic procedures	12 (0.8%)	9 (0.6%)	21 (0.7%)
Ear tube insertion	6 (0.4%)	5 (0.3%)	11 (0.4%)
Ear tube removal	1 (<0.1%)	0	1 (<0.1%)
Myringotomy	3 (0.2%)	2 (0.1%)	5 (0.2%)
Tympanoplasty	2 (0.1%)	3 (0.2%)	5 (0.2%)
Nasal therapeutic procedures	16 (1.1%)	9 (0.6%)	25 (0.9%)
Cautery to nose	1 (<0.1%)	1 (<0.1%)	2 (<0.1%)
Nasal operation	4 (0.3%)	0	4 (0.1%)
Nasal polypectomy	1 (<0.1%)	1 (<0.1%)	2 (<0.1%)
Nasal septal operation	4 (0.3%)	1 (<0.1%)	5 (0.2%)
Rhinoplasty	6 (0.4%)	6 (0.4%)	12 (0.4%)
Obstetric therapeutic procedures	188 (13.1%)	159 (11.0%)	347 (12.0%)
Caesarean section	187 (13.1%)	158 (10.9%)	345 (12.0%)
Evacuation of retained products of conception	1 (<0.1%)	1 (<0.1%)	2 (<0.1%)
Orbit and globe therapeutic procedures	4 (0.3%)	8 (0.6%)	12 (0.4%)
Eye muscle operation	0	3 (0.2%)	3 (0.1%)
Strabismus correction	4 (0.3%)	5 (0.3%)	9 (0.3%)
Ovarian therapeutic procedures	13 (0.9%)	11 (0.8%)	24 (0.8%)
Oophorectomy	0	1 (<0.1%)	1 (<0.1%)
Ovarian cystectomy	11 (0.8%)	10 (0.7%)	21 (0.7%)
Ovarian operation	2 (0.1%)	0	2 (<0.1%)
Palatal therapeutic procedures	1 (<0.1%)	0	1 (<0.1%)
Uvulectomy	1 (<0.1%)	0	1 (<0.1%)
Paranasal therapeutic procedures	2 (0.1%)	3 (0.2%)	5 (0.2%)
Sinus operation	2 (0.1%)	3 (0.2%)	5 (0.2%)
Peripheral nerve therapeutic procedures	1 (<0.1%)	1 (<0.1%)	2 (<0.1%)
Carpal tunnel decompression	1 (<0.1%)	0	1 (<0.1%)
Peripheral nerve operation	0	1 (<0.1%)	1 (<0.1%)
Phototherapies	1 (<0.1%)	0	1 (<0.1%)
Laser therapy	1 (<0.1%)	0	1 (<0.1%)
Prophylactic procedures NEC	1 (<0.1%)	2 (0.1%)	3 (0.1%)
Antibiotic prophylaxis	0	1 (<0.1%)	1 (<0.1%)
Antiviral prophylaxis	0	1 (<0.1%)	1 (<0.1%)
Prophylaxis	1 (<0.1%)	0	1 (<0.1%)

Table 14.1.1 / 26: Number of subjects with medical history findings by primary system organ class, high level term, preferred term by treatment (FAS)

Primary system organ class<br>High level term<br>Preferred term<br>MedDRA Version 14.0	LCS12<br>N=1432 (100%)	LCS16<br>N=1452 (100%)	Total<br>N=2884 (100%)
Salivary gland therapeutic procedures	1 (<0.1%)	0	1 (<0.1%)
Salivary gland resection	1 (<0.1%)	0	1 (<0.1%)
Skin and subcutaneous tissue therapeutic procedures NEC	1 (<0.1%)	3 (0.2%)	4 (0.1%)
Pilonidal sinus repair	0	3 (0.2%)	3 (0.1%)
Skin operation	1 (<0.1%)	0	1 (<0.1%)
Skin grafts	2 (0.1%)	2 (0.1%)	4 (0.1%)
Skin graft	2 (0.1%)	2 (0.1%)	4 (0.1%)
Skin lesion excisions	15 (1.0%)	9 (0.6%)	24 (0.8%)
Acrochordon excision	1 (<0.1%)	0	1 (<0.1%)
Mole excision	5 (0.3%)	2 (0.1%)	7 (0.2%)
Scar excision	2 (0.1%)	1 (<0.1%)	3 (0.1%)
Sebaceous cyst excision	1 (<0.1%)	0	1 (<0.1%)
Skin neoplasm excision	0	1 (<0.1%)	1 (<0.1%)
Wart excision	6 (0.4%)	5 (0.3%)	11 (0.4%)
Skull and brain therapeutic procedures	0	1 (<0.1%)	1 (<0.1%)
Cranioplasty	0	1 (<0.1%)	1 (<0.1%)
Small intestine therapeutic procedures	0	2 (0.1%)	2 (<0.1%)
Ileal operation	0	1 (<0.1%)	1 (<0.1%)
Small intestinal resection	0	1 (<0.1%)	1 (<0.1%)
Soft tissue therapeutic procedures NEC	10 (0.7%)	4 (0.3%)	14 (0.5%)
Lipoma excision	2 (0.1%)	0	2 (<0.1%)
Liposuction	8 (0.6%)	4 (0.3%)	12 (0.4%)
Spine and spinal cord therapeutic procedures	3 (0.2%)	6 (0.4%)	9 (0.3%)
Intervertebral disc operation	2 (0.1%)	4 (0.3%)	6 (0.2%)
Scoliosis surgery	1 (<0.1%)	2 (0.1%)	3 (0.1%)
Tendon therapeutic procedures	4 (0.3%)	6 (0.4%)	10 (0.3%)
Tendon operation	1 (<0.1%)	2 (0.1%)	3 (0.1%)
Tendon sheath incision	1 (<0.1%)	0	1 (<0.1%)
Tendon sheath lesion excision	2 (0.1%)	4 (0.3%)	6 (0.2%)
Therapeutic procedures NEC	16 (1.1%)	17 (1.2%)	33 (1.1%)
Abscess drainage	1 (<0.1%)	2 (0.1%)	3 (0.1%)
Adhesiolysis	2 (0.1%)	0	2 (<0.1%)
Benign tumour excision	0	3 (0.2%)	3 (0.1%)
Cryotherapy	2 (0.1%)	5 (0.3%)	7 (0.2%)
Cyst drainage	1 (<0.1%)	0	1 (<0.1%)
Cyst removal	7 (0.5%)	4 (0.3%)	11 (0.4%)
Exploratory operation	1 (<0.1%)	0	1 (<0.1%)
Lithotripsy	1 (<0.1%)	0	1 (<0.1%)
Polypectomy	0	1 (<0.1%)	1 (<0.1%)
Surgery	1 (<0.1%)	1 (<0.1%)	2 (<0.1%)
Therapeutic procedure	0	1 (<0.1%)	1 (<0.1%)

Table 14.1.1 / 26: Number of subjects with medical history findings by primary system organ class, high level term, preferred term by treatment (FAS)

Primary system organ class<br>High level term<br>Preferred term<br>MedDRA Version 14.0	LCS12<br>N=1432 (100%)	LCS16<br>N=1452 (100%)	Total<br>N=2884 (100%)
Thyroid radiotherapies	1 (<0.1%)	0	1 (<0.1%)
Radioactive iodine therapy	1 (<0.1%)	0	1 (<0.1%)
Thyroid therapeutic procedures	3 (0.2%)	5 (0.3%)	8 (0.3%)
Thyroid adenoma removal	1 (<0.1%)	0	1 (<0.1%)
Thyroidectomy	2 (0.1%)	5 (0.3%)	7 (0.2%)
Tongue therapeutic procedures	0	1 (<0.1%)	1 (<0.1%)
Tongue tie operation	0	1 (<0.1%)	1 (<0.1%)
Tonsillar therapeutic procedures	150 (10.5%)	118 (8.1%)	268 (9.3%)
Adenoidectomy	36 (2.5%)	21 (1.4%)	57 (2.0%)
Adenotonsillectomy	4 (0.3%)	18 (1.2%)	22 (0.8%)
Tonsillectomy	127 (8.9%)	89 (6.1%)	216 (7.5%)
Urethral therapeutic procedures	0	1 (<0.1%)	1 (<0.1%)
Urethral dilation procedure	0	1 (<0.1%)	1 (<0.1%)
Uterine therapeutic procedures	25 (1.7%)	38 (2.6%)	63 (2.2%)
Endometriosis ablation	1 (<0.1%)	4 (0.3%)	5 (0.2%)
Myomectomy	0	2 (0.1%)	2 (<0.1%)
Uterine dilation and curettage	19 (1.3%)	25 (1.7%)	44 (1.5%)
Uterine dilation and evacuation	3 (0.2%)	3 (0.2%)	6 (0.2%)
Uterine operation	0	2 (0.1%)	2 (<0.1%)
Uterine polypectomy	2 (0.1%)	3 (0.2%)	5 (0.2%)
Vaginal therapeutic procedures	3 (0.2%)	2 (0.1%)	5 (0.2%)
Hymenectomy	2 (0.1%)	1 (<0.1%)	3 (0.1%)
Vaginal cyst excision	1 (<0.1%)	0	1 (<0.1%)
Vaginal operation	0	1 (<0.1%)	1 (<0.1%)
Venous therapeutic procedures	3 (0.2%)	6 (0.4%)	9 (0.3%)
Varicose vein operation	3 (0.2%)	6 (0.4%)	9 (0.3%)
Vascular disorders	6 (0.4%)	9 (0.6%)	15 (0.5%)
Aortic necrosis and vascular insufficiency	0	1 (<0.1%)	1 (<0.1%)
Aortic stenosis	0	1 (<0.1%)	1 (<0.1%)
Arterial inflammations	0	1 (<0.1%)	1 (<0.1%)
Kawasaki's disease	0	1 (<0.1%)	1 (<0.1%)
Lymphoedemas	1 (<0.1%)	0	1 (<0.1%)
Lymphoedema	1 (<0.1%)	0	1 (<0.1%)
Non-site specific vascular disorders NEC	0	1 (<0.1%)	1 (<0.1%)
Vein pain	0	1 (<0.1%)	1 (<0.1%)
Peripheral vascular disorders NEC	0	2 (0.1%)	2 (<0.1%)
Hot flush	0	1 (<0.1%)	1 (<0.1%)
Peripheral vascular disorder	0	1 (<0.1%)	1 (<0.1%)
Varicose veins non-site specific	3 (0.2%)	1 (<0.1%)	4 (0.1%)
Varicose vein	3 (0.2%)	1 (<0.1%)	4 (0.1%)

Table 14.1.1 / 26: Number of subjects with medical history findings by primary system organ class, high level term, preferred term by treatment (FAS)

Primary system organ class<br>High level term<br>Preferred term<br>MedDRA Version 14.0	LCS12<br>N=1432 (100%)	LCS16<br>N=1452 (100%)	Total<br>N=2884 (100%)
Vascular hypertensive disorders NEC	2 (0.1%)	3 (0.2%)	5 (0.2%)
Hypertension	2 (0.1%)	3 (0.2%)	5 (0.2%)

Note: A subject is counted only once within each SOC/high level term.
Global Biostatistics: /by-sasp/patdb/projects/de04209/310442/stat/prod_interim03/pgms/t-mh.sas epkll 07OCT2011 10:49
End of table

Table 14.1.1 / 27: Gynecological history: Age at menarche, No. of births, vaginal deliveries, abortions, cesarean sections and ectopic pregnancies by treatment - frequencies (FAS)

	LCS12 (N=1432)	LCS16 (N=1452)	Total (N=2884)
Number of subjects	1432 (100.0%)	1452 (100.0%)	2884 (100.0%)
Menarche, age			
8	1 (<0.1%)	0	1 (<0.1%)
9	26 (1.8%)	28 (1.9%)	54 (1.9%)
10	70 (4.9%)	44 (3.0%)	114 (4.0%)
11	194 (13.5%)	217 (14.9%)	411 (14.3%)
12	398 (27.8%)	441 (30.4%)	839 (29.1%)
13	391 (27.3%)	384 (26.4%)	775 (26.9%)
14	203 (14.2%)	216 (14.9%)	419 (14.5%)
15	99 (6.9%)	80 (5.5%)	179 (6.2%)
16	39 (2.7%)	26 (1.8%)	65 (2.3%)
17	6 (0.4%)	12 (0.8%)	18 (0.6%)
18	4 (0.3%)	4 (0.3%)	8 (0.3%)
19	1 (<0.1%)	0	1 (<0.1%)
No. of births			
0	556 (38.8%)	574 (39.5%)	1130 (39.2%)
1	320 (22.3%)	333 (22.9%)	653 (22.6%)
2	397 (27.7%)	407 (28.0%)	804 (27.9%)
3	128 (8.9%)	113 (7.8%)	241 (8.4%)
4	26 (1.8%)	20 (1.4%)	46 (1.6%)
5	3 (0.2%)	4 (0.3%)	7 (0.2%)
6	2 (0.1%)	1 (<0.1%)	3 (0.1%)
Vaginal deliveries, number			
missing	2 (0.1%)	1 (<0.1%)	3 (0.1%)
0	743 (51.9%)	743 (51.2%)	1486 (51.5%)
1	269 (18.8%)	275 (18.9%)	544 (18.9%)
2	290 (20.3%)	328 (22.6%)	618 (21.4%)
3	102 (7.1%)	87 (6.0%)	189 (6.6%)
4	22 (1.5%)	14 (1.0%)	36 (1.2%)
5	1 (<0.1%)	4 (0.3%)	5 (0.2%)
6	3 (0.2%)	0	3 (0.1%)

Table 14.1.1 / 27: Gynecological history: Age at menarche, No. of births, vaginal deliveries, abortions, cesarean sections and ectopic pregnancies by treatment - frequencies (FAS)

	LCS12 (N=1432)	LCS16 (N=1452)	Total (N=2884)
No. of abortions			
missing	1 (<0.1%)	1 (<0.1%)	2 (<0.1%)
0	1041 (72.7%)	1054 (72.6%)	2095 (72.6%)
1	261 (18.2%)	277 (19.1%)	538 (18.7%)
2	96 (6.7%)	87 (6.0%)	183 (6.3%)
3	24 (1.7%)	21 (1.4%)	45 (1.6%)
4	7 (0.5%)	10 (0.7%)	17 (0.6%)
5	1 (<0.1%)	2 (0.1%)	3 (0.1%)
7	1 (<0.1%)	0	1 (<0.1%)
Cesarean sections, number			
missing	1 (<0.1%)	1 (<0.1%)	2 (<0.1%)
0	1166 (81.4%)	1216 (83.7%)	2382 (82.6%)
1	173 (12.1%)	160 (11.0%)	333 (11.5%)
2	84 (5.9%)	64 (4.4%)	148 (5.1%)
3	8 (0.6%)	11 (0.8%)	19 (0.7%)
Ectopic pregnancies, number			
missing	2 (0.1%)	3 (0.2%)	5 (0.2%)
0	1430 (99.9%)	1449 (99.8%)	2879 (99.8%)

Global Biostatistics: /by-sasp/patdb/projects/de04209/310442/stat/prod_interim03/pgms/t-dm-gyn-history.sas epkll 07OCT2011 10:49
End of table

Table 14.1.1 / 28: Gynecological history: Age at menarche, No. of births, vaginal deliveries, abortions, cesarean sections, and ectopic pregnancies - descriptive statistics by treatment (FAS)

		TREATMENT	n	Nmiss	Mean	SD	Min	Q1	Median	Q3	Max
Menarche, age	years	LCS12	1432	0	12.6	1.5	8	12.0	13.0	13.0	19
		LCS16	1452	0	12.6	1.4	9	12.0	12.0	13.0	18
		Total	2884	0	12.6	1.5	8	12.0	13.0	13.0	19
No. of births		LCS12	1432	0	1.1	1.1	0	0.0	1.0	2.0	6
		LCS16	1452	0	1.1	1.1	0	0.0	1.0	2.0	6
		Total	2884	0	1.1	1.1	0	0.0	1.0	2.0	6
Vaginal deliveries, number		LCS12	1430	2	0.9	1.1	0	0.0	0.0	2.0	6
		LCS16	1451	1	0.9	1.0	0	0.0	0.0	2.0	5
		Total	2881	3	0.9	1.1	0	0.0	0.0	2.0	6
No. of abortions		LCS12	1431	1	0.4	0.8	0	0.0	0.0	1.0	7
		LCS16	1451	1	0.4	0.7	0	0.0	0.0	1.0	5
		Total	2882	2	0.4	0.8	0	0.0	0.0	1.0	7
Cesarean sections, number		LCS12	1431	1	0.3	0.6	0	0.0	0.0	0.0	3
		LCS16	1451	1	0.2	0.6	0	0.0	0.0	0.0	3
		Total	2882	2	0.2	0.6	0	0.0	0.0	0.0	3
Ectopic pregnancies, number		LCS12	1430	2	0.0	0.0	0	0.0	0.0	0.0	0
		LCS16	1449	3	0.0	0.0	0	0.0	0.0	0.0	0
		Total	2879	5	0.0	0.0	0	0.0	0.0	0.0	0

Global Biostatistics: /by-sasp/patdb/projects/de04209/310442/stat/prod_interim03/pgms/t-dm-gyn-history.sas epkll 07OCT2011 10:49
End of table

Table 14.1.1 / 29: Contraceptive methods at screening visit by treatment (FAS)

	LCS12 N=1432 (100%)	LCS16 N=1452 (100%)	Total N=2884 (100%)
Contraceptive method			
no	152 (10.6%)	163 (11.2%)	315 (10.9%)
condom	592 (41.3%)	585 (40.3%)	1177 (40.8%)
OC	461 (32.2%)	493 (34.0%)	954 (33.1%)
diaphragm	1 (<0.1%)	0	1 (<0.1%)
IUD containing hormone	48 (3.4%)	40 (2.8%)	88 (3.1%)
implant	7 (0.5%)	6 (0.4%)	13 (0.5%)
IUD non hormonal	69 (4.8%)	59 (4.1%)	128 (4.4%)
other	102 (7.1%)	106 (7.3%)	208 (7.2%)

Global Biostatistics: /by-sasp/patdb/projects/de04209/310442/stat/prod_interim03/pgms/t-dm-gyn-history.sas epkll 07OCT2011 10:49
End of table

Table 14.1.1 / 30: Menstrual history: Cycle regularity, length of cycle, duration of menstruation, intensity of bleeding and intracyclic vaginal bleeding by treatment (FAS)

	LCS12 (N=1432)	LCS16 (N=1452)	Total (N=2884)
Cycle, regularity			
Number of Subjects	1432 (100.0%)	1452 (100.0%)	2884 (100.0%)
missing	0	2 (0.1%)	2 (<0.1%)
regular	1419 (99.1%)	1433 (98.7%)	2852 (98.9%)
irregular	13 (0.9%)	17 (1.2%)	30 (1.0%)
Cycle average length (days)			
Mean	28.5	28.3	28.4
SD	2.6	2.2	2.4
Min	21	0	0
Q1	28.0	28.0	28.0
Median	28.0	28.0	28.0
Q3	30.0	29.0	30.0
Max	90	36	90
Menstruation average length (days)			
Mean	4.9	5.0	5.0
SD	1.3	1.3	1.3
Min	1	0	0
Q1	4.0	4.0	4.0
Median	5.0	5.0	5.0
Q3	6.0	6.0	6.0
Max	14	12	14
Average intensity of bleeding			
Number of Subjects	1432 (100.0%)	1452 (100.0%)	2884 (100.0%)
missing	1 (<0.1%)	0	1 (<0.1%)
none	1 (<0.1%)	3 (0.2%)	4 (0.1%)
spotting	8 (0.6%)	5 (0.3%)	13 (0.5%)
light	189 (13.2%)	187 (12.9%)	376 (13.0%)
normal	907 (63.3%)	925 (63.7%)	1832 (63.5%)
heavy	326 (22.8%)	332 (22.9%)	658 (22.8%)
Vaginal bleeding, intracyclic			
Number of Subjects	1432 (100.0%)	1452 (100.0%)	2884 (100.0%)
missing	2 (0.1%)	0	2 (<0.1%)
no	1404 (98.0%)	1417 (97.6%)	2821 (97.8%)
yes	26 (1.8%)	35 (2.4%)	61 (2.1%)

Global Biostatistics: /by-sasp/patdb/projects/de04209/310442/stat/prod_interim03/pgms/t-dm-gyn-history.sas epkll 07OCT2011 10:49
End of table

Table 14.1.1 / 31: Vaginal ultrasound: Uterus (portio-fundus) by treatment (FAS)

		TREATMENT	n	Nmiss	Mean	SD	Min	Q1	Median	Q3	Max
Uterus length	(mm)	LCS12	1214	7	72.0	12.3	7	64.0	72.0	80.0	120
		LCS16	1221	6	72.2	12.4	8	64.0	72.0	80.0	124
		Total	2435	13	72.1	12.4	7	64.0	72.0	80.0	124

Global Biostatistics: /by-sasp/patdb/projects/de04209/310442/stat/prod_interim03/pgms/t-dm-gyn-history.sas epkll 07OCT2011 10:49
End of table

Table 14.1.1 / 32: Vaginal ultrasound: Uterus: Max. width anteroposterior by treatment (FAS)

		TREATMENT	n	Nmiss	Mean	SD	Min	Q1	Median	Q3	Max
Uterus maximal anteroposterior width	(mm)	LCS12	1214	7	37.4	13.7	3	31.0	37.0	42.0	426
		LCS16	1222	5	37.0	8.1	4	31.0	37.0	42.0	70
		Total	2436	12	37.2	11.3	3	31.0	37.0	42.0	426

Global Biostatistics: /by-sasp/patdb/projects/de04209/310442/stat/prod_interim03/pgms/t-dm-gyn-history.sas epkll 07OCT2011 10:49
End of table

Table 14.1.1 / 33: Vaginal ultrasound: Uterus: Max. width transverse by treatment (FAS)

		TREATMENT	n	Nmiss	Mean	SD	Min	Q1	Median	Q3	Max
Uterus maximal transverse width	(mm)	LCS12	1205	16	46.4	10.4	4	40.0	46.0	53.0	86
		LCS16	1214	13	46.9	10.2	23	40.0	46.0	54.0	92
		Total	2419	29	46.6	10.3	4	40.0	46.0	53.0	92

Global Biostatistics: /by-sasp/patdb/projects/de04209/310442/stat/prod_interim03/pgms/t-dm-gyn-history.sas epkll 07OCT2011 10:49
End of table

Table 14.1.1 / 34: Vaginal ultrasound: Uterus: Length of the cavity by treatment (FAS)

		TREATMENT	n	Nmiss	Mean	SD	Min	Q1	Median	Q3	Max
Uterus, cavity length	(mm)	LCS12	1177	44	41.1	13.4	3	32.0	38.0	47.0	90
		LCS16	1184	43	41.3	13.6	3	32.0	38.0	48.0	96
		Total	2361	87	41.2	13.5	3	32.0	38.0	47.0	96

Global Biostatistics: /by-sasp/patdb/projects/de04209/310442/stat/prod_interim03/pgms/t-dm-gyn-history.sas epkll 07OCT2011 10:49
End of table

Table 14.1.1 / 35: Vaginal ultrasound: Other uterine abnormalities by treatment (FAS)

	LCS12 N=1432 (100%)	LCS16 N=1452 (100%)	Total N=2884 (100%)
Uterus, other abnormalities			
missing	2 (0.1%)	1 (<0.1%)	3 (0.1%)
no	1206 (84.2%)	1209 (83.3%)	2415 (83.7%)
yes	13 (0.9%)	17 (1.2%)	30 (1.0%)

Global Biostatistics: /by-sasp/patdb/projects/de04209/310442/stat/prod_interim03/pgms/t-dm-gyn-history.sas epkll 07OCT2011 10:49
End of table

Table 14.1.1 / 36: Listing of specified other uterine abnormalities by treatment (FAS)

TREATMENT	Subject number	Uterus, other abnormalities text
LCS12	140105	UTERINE RETROVERSION.
	140113	CERVIX NABOTH CYST
	140605	C-SECTION SCAR
	140807	IUD IN PLACE.
	140904	QUERY ADENOMYOSIS
	140911	ARCUATE
	160962	CS SCAR IN LOWER SEGMENT OF THE UTERUS
	180603	NON HORMONAL IUD IN SITU
	230501	UTERUS VERY CURVED.
	240209	WIDENED UPPER BODY AND BREAK OF THE ENDOMETRIUM SUSPECT UTERUS SUBSEPTUS - NCS
	241113	LT. HORN SMALL HYPERECHOIC AREA, LINING
	243309	EVIDENCE OF C-SECTION INCISION AT UTERINE NECK
	245907	FOCAL ADENOMYSOS NO DEVIATION OF CAVITY WITH SALINE ULTRASOUND NCS
LCS16	140901	ARCUATE UTERUS
	140909	HETEROGENOUS C-SECTION SCAN.
	140916	ARCUATE
	160719	THE UTERINE CAVITY IS SMALL
	161222	CYSTA/MYOMA INTRAMURALIS 4X6MM
	170803	CESAREAN SCAR VISIBLE.
	200232	PREGNANT
	241117	RETROVERTED UTERUS
	241118	RETROVERTED UTERUS
	241138	HYPERECHOIC AREA LINING - NCS SMALL FLAT POLYP
	241152	NABOTHIAN CYSTS - NCS
	242803	ADENOMYOSIS
	242813	ADENOMYOSIS
	242814	ADENOMYOSIS
	243316	INCREASED VASCULARITY NOTED IN THE PERIPHERY OF THE UTERINE WALLS
	243401	HYPERECHORIC SMALL AREAS WITHIN POSTERIOR
	244405	4ML FLUID IN ENDOMETRIUM

Global Biostatistics: /by-sasp/patdb/projects/de04209/310442/stat/prod_interim03/pgms/t-dm-gyn-history.sas epkll 07OCT2011 10:49
End of table

Table 14.1.1 / 37: Number of subjects who took at least one prior medication (FAS)

Substance	LCS12 N=1432 (100%)	LCS16 N=1452 (100%)	Total N=2884 (100%)
Number of subjects (%) with at least one prior medication	792 (55.3%)	822 (56.6%)	1614 (56.0%)
ETHINYLESTRADIOL	427 (29.8%)	474 (32.6%)	901 (31.2%)
LEVONORGESTREL	133 (9.3%)	129 (8.9%)	262 (9.1%)
DROSPIRENONE	109 (7.6%)	126 (8.7%)	235 (8.1%)
IBUPROFEN	74 (5.2%)	77 (5.3%)	151 (5.2%)
PARACETAMOL	70 (4.9%)	75 (5.2%)	145 (5.0%)
DESOGESTREL	65 (4.5%)	69 (4.8%)	134 (4.6%)
ETONOGESTREL	51 (3.6%)	61 (4.2%)	112 (3.9%)
GESTODENE	46 (3.2%)	52 (3.6%)	98 (3.4%)
NORGESTIMATE	36 (2.5%)	41 (2.8%)	77 (2.7%)
INTRAUTERINE CONTRACEPTIVE DEVICE	36 (2.5%)	29 (2.0%)	65 (2.3%)
NORETHISTERONE ACETATE	27 (1.9%)	31 (2.1%)	58 (2.0%)
AZITHROMYCIN	28 (2.0%)	27 (1.9%)	55 (1.9%)
NORETHISTERONE	31 (2.2%)	21 (1.4%)	52 (1.8%)
METRONIDAZOLE	26 (1.8%)	23 (1.6%)	49 (1.7%)
FERROUS FUMARATE	20 (1.4%)	21 (1.4%)	41 (1.4%)
CYPROTERONE ACETATE	20 (1.4%)	19 (1.3%)	39 (1.4%)
FLUCONAZOLE	18 (1.3%)	17 (1.2%)	35 (1.2%)
PSEUDOEPHEDRINE HYDROCHLORIDE	16 (1.1%)	19 (1.3%)	35 (1.2%)
NORELGESTROMIN	16 (1.1%)	11 (0.8%)	27 (0.9%)
AMOXICILLIN	17 (1.2%)	9 (0.6%)	26 (0.9%)
DEXTROMETHORPHAN HYDROBROMIDE	12 (0.8%)	10 (0.7%)	22 (0.8%)
CAFFEINE	10 (0.7%)	8 (0.6%)	18 (0.6%)
DOXYCYCLINE	5 (0.3%)	13 (0.9%)	18 (0.6%)
MISOPROSTOL	8 (0.6%)	9 (0.6%)	17 (0.6%)
NAPROXEN	9 (0.6%)	7 (0.5%)	16 (0.6%)
ACETYLSALICYLIC ACID	8 (0.6%)	7 (0.5%)	15 (0.5%)
NAPROXEN SODIUM	8 (0.6%)	7 (0.5%)	15 (0.5%)
CEFALEXIN	6 (0.4%)	8 (0.6%)	14 (0.5%)
CODEINE PHOSPHATE	10 (0.7%)	4 (0.3%)	14 (0.5%)
NITROFURANTOIN	7 (0.5%)	7 (0.5%)	14 (0.5%)
CLINDAMYCIN PHOSPHATE	7 (0.5%)	6 (0.4%)	13 (0.5%)
CLOTRIMAZOLE	7 (0.5%)	6 (0.4%)	13 (0.5%)
NEOMYCIN SULFATE	9 (0.6%)	4 (0.3%)	13 (0.5%)
NYSTATIN	7 (0.5%)	6 (0.4%)	13 (0.5%)
CHLORPHENAMINE MALEATE	8 (0.6%)	4 (0.3%)	12 (0.4%)
INTRAUTERINE CONTRACEPTIVES	6 (0.4%)	5 (0.3%)	11 (0.4%)
LORATADINE	5 (0.3%)	6 (0.4%)	11 (0.4%)
POLYMYXIN B SULFATE	7 (0.5%)	4 (0.3%)	11 (0.4%)
CIPROFLOXACIN	5 (0.3%)	5 (0.3%)	10 (0.3%)
DICLOFENAC	7 (0.5%)	3 (0.2%)	10 (0.3%)
TRIMETHOPRIM	4 (0.3%)	6 (0.4%)	10 (0.3%)
CLAVULANATE POTASSIUM	6 (0.4%)	3 (0.2%)	9 (0.3%)

Table 14.1.1 / 37: Number of subjects who took at least one prior medication (FAS)

Substance	LCS12 N=1432 (100%)	LCS16 N=1452 (100%)	Total N=2884 (100%)
MINERALS NOS	4 (0.3%)	5 (0.3%)	9 (0.3%)
ASCORBIC ACID	4 (0.3%)	4 (0.3%)	8 (0.3%)
DIPHENHYDRAMINE HYDROCHLORIDE	4 (0.3%)	4 (0.3%)	8 (0.3%)
DOXYLAMINE SUCCINATE	5 (0.3%)	3 (0.2%)	8 (0.3%)
ETHANOL	5 (0.3%)	3 (0.2%)	8 (0.3%)
FERROUS SULFATE	4 (0.3%)	4 (0.3%)	8 (0.3%)
MICONAZOLE NITRATE	5 (0.3%)	3 (0.2%)	8 (0.3%)
POVIDONE-IODINE	3 (0.2%)	5 (0.3%)	8 (0.3%)
AMPICILLIN	3 (0.2%)	4 (0.3%)	7 (0.2%)
EPHEDRINE SULFATE	5 (0.3%)	2 (0.1%)	7 (0.2%)
LEVOFLOXACIN	4 (0.3%)	3 (0.2%)	7 (0.2%)
PHENOXYMETHYLPENICILLIN POTASSIUM	5 (0.3%)	2 (0.1%)	7 (0.2%)
PHENYLEPHRINE HYDROCHLORIDE	3 (0.2%)	4 (0.3%)	7 (0.2%)
AMOXICILLIN TRIHYDRATE	3 (0.2%)	3 (0.2%)	6 (0.2%)
CHLORPHENAMINE	2 (0.1%)	4 (0.3%)	6 (0.2%)
GUAIFENESIN	2 (0.1%)	4 (0.3%)	6 (0.2%)
HEPATITIS A VACCINE	3 (0.2%)	3 (0.2%)	6 (0.2%)
HYDROCODONE	2 (0.1%)	4 (0.3%)	6 (0.2%)
HYDROCODONE BITARTRATE	2 (0.1%)	4 (0.3%)	6 (0.2%)
SULFAMETHOXAZOLE	1 (<0.1%)	5 (0.3%)	6 (0.2%)
VIRAL VACCINES	2 (0.1%)	4 (0.3%)	6 (0.2%)
VITAMIN B NOS	1 (<0.1%)	5 (0.3%)	6 (0.2%)
AMOXICILLIN SODIUM	3 (0.2%)	2 (0.1%)	5 (0.2%)
BIOTIN	1 (<0.1%)	4 (0.3%)	5 (0.2%)
CLARITHROMYCIN	4 (0.3%)	1 (<0.1%)	5 (0.2%)
CLINDAMYCIN HYDROCHLORIDE	4 (0.3%)	1 (<0.1%)	5 (0.2%)
DOXYCYCLINE MONOHYDRATE	4 (0.3%)	1 (<0.1%)	5 (0.2%)
DYDROGESTERONE	4 (0.3%)	1 (<0.1%)	5 (0.2%)
FOLIC ACID	2 (0.1%)	3 (0.2%)	5 (0.2%)
LOPERAMIDE HYDROCHLORIDE	3 (0.2%)	2 (0.1%)	5 (0.2%)
NICOTINIC ACID	1 (<0.1%)	4 (0.3%)	5 (0.2%)
PIVMECILLINAM	4 (0.3%)	1 (<0.1%)	5 (0.2%)
RETINOL	1 (<0.1%)	4 (0.3%)	5 (0.2%)
SERTRALINE	3 (0.2%)	2 (0.1%)	5 (0.2%)
TOCOPHEROL	1 (<0.1%)	4 (0.3%)	5 (0.2%)
VITAMIN D NOS	1 (<0.1%)	4 (0.3%)	5 (0.2%)
VITAMINS NOS	4 (0.3%)	1 (<0.1%)	5 (0.2%)
BETA-LACTAMASE SENSITIVE PENICILLINS	1 (<0.1%)	3 (0.2%)	4 (0.1%)
BUDESONIDE	4 (0.3%)	0	4 (0.1%)
CLINDAMYCIN	2 (0.1%)	2 (0.1%)	4 (0.1%)
CYCLOBENZAPRINE HYDROCHLORIDE	3 (0.2%)	1 (<0.1%)	4 (0.1%)
HYDROCORTISONE	3 (0.2%)	1 (<0.1%)	4 (0.1%)
MEPYRAMINE MALEATE	0	4 (0.3%)	4 (0.1%)
MESTRANOL	3 (0.2%)	1 (<0.1%)	4 (0.1%)
METHOCARBAMOL	2 (0.1%)	2 (0.1%)	4 (0.1%)

Table 14.1.1 / 37: Number of subjects who took at least one prior medication (FAS)

Substance	LCS12 N=1432 (100%)	LCS16 N=1452 (100%)	Total N=2884 (100%)
NORGESTREL	1 (<0.1%)	3 (0.2%)	4 (0.1%)
OMEPRAZOLE	1 (<0.1%)	3 (0.2%)	4 (0.1%)
PARGEVERINE	1 (<0.1%)	3 (0.2%)	4 (0.1%)
PHENAZOPYRIDINE HYDROCHLORIDE	2 (0.1%)	2 (0.1%)	4 (0.1%)
SUMATRIPTAN	2 (0.1%)	2 (0.1%)	4 (0.1%)
TIZANIDINE HYDROCHLORIDE	1 (<0.1%)	3 (0.2%)	4 (0.1%)
UNCODEABLE "UNCLASSIFIABLE"	0	4 (0.3%)	4 (0.1%)
ZOLPIDEM TARTRATE	2 (0.1%)	2 (0.1%)	4 (0.1%)
ACICLOVIR	1 (<0.1%)	2 (0.1%)	3 (0.1%)
ACRIVASTINE	2 (0.1%)	1 (<0.1%)	3 (0.1%)
ALPRAZOLAM	1 (<0.1%)	2 (0.1%)	3 (0.1%)
AMBROXOL	1 (<0.1%)	2 (0.1%)	3 (0.1%)
ANILIDES	2 (0.1%)	1 (<0.1%)	3 (0.1%)
BETAMETHASONE	0	3 (0.2%)	3 (0.1%)
BROMHEXINE HYDROCHLORIDE	1 (<0.1%)	2 (0.1%)	3 (0.1%)
BUPROPION	2 (0.1%)	1 (<0.1%)	3 (0.1%)
CETIRIZINE HYDROCHLORIDE	1 (<0.1%)	2 (0.1%)	3 (0.1%)
CLONIXIN LYSINATE	2 (0.1%)	1 (<0.1%)	3 (0.1%)
CO-TRIMOXAZOLE	1 (<0.1%)	2 (0.1%)	3 (0.1%)
CYANOCOBALAMIN	1 (<0.1%)	2 (0.1%)	3 (0.1%)
DEXTROPROPOXYPHENE NAPSILATE	1 (<0.1%)	2 (0.1%)	3 (0.1%)
DIET FORMULATIONS FOR TREATMENT OF OBESITY	1 (<0.1%)	2 (0.1%)	3 (0.1%)
DULOXETINE HYDROCHLORIDE	2 (0.1%)	1 (<0.1%)	3 (0.1%)
ECONAZOLE	2 (0.1%)	1 (<0.1%)	3 (0.1%)
ERGOTAMINE TARTRATE	3 (0.2%)	0	3 (0.1%)
ESTRADIOL VALERATE	0	3 (0.2%)	3 (0.1%)
ETYNODIOL DIACETATE	2 (0.1%)	1 (<0.1%)	3 (0.1%)
GYNECOLOGICAL ANTIINFECTIVES AND ANTISEPTICS	3 (0.2%)	0	3 (0.1%)
HEPATITIS B VACCINE	2 (0.1%)	1 (<0.1%)	3 (0.1%)
HERBAL NOS	2 (0.1%)	1 (<0.1%)	3 (0.1%)
KETOPROFEN	2 (0.1%)	1 (<0.1%)	3 (0.1%)
LIDOCAINE	1 (<0.1%)	2 (0.1%)	3 (0.1%)
MEDROXYPROGESTERONE ACETATE	2 (0.1%)	1 (<0.1%)	3 (0.1%)
MELOXICAM	2 (0.1%)	1 (<0.1%)	3 (0.1%)
MOXIFLOXACIN HYDROCHLORIDE	3 (0.2%)	0	3 (0.1%)
NORETHISTERONE ENANTATE	0	3 (0.2%)	3 (0.1%)
PROMETHAZINE	1 (<0.1%)	2 (0.1%)	3 (0.1%)
PSEUDOEPHEDRINE	1 (<0.1%)	2 (0.1%)	3 (0.1%)
PSEUDOEPHEDRINE SULFATE	3 (0.2%)	0	3 (0.1%)
RIZATRIPTAN BENZOATE	2 (0.1%)	1 (<0.1%)	3 (0.1%)
TERCONAZOLE	3 (0.2%)	0	3 (0.1%)
ANTIDIARRHOEAL MICROORGANISMS	2 (0.1%)	0	2 (<0.1%)
ANTIFUNGALS	2 (0.1%)	0	2 (<0.1%)
BROXYQUINOLINE	1 (<0.1%)	1 (<0.1%)	2 (<0.1%)
CEFPROZIL	1 (<0.1%)	1 (<0.1%)	2 (<0.1%)

Table 14.1.1 / 37: Number of subjects who took at least one prior medication (FAS)

Substance	LCS12 N=1432 (100%)	LCS16 N=1452 (100%)	Total N=2884 (100%)
CIPROFLOXACIN HYDROCHLORIDE	2 (0.1%)	0	2 (<0.1%)
CITALOPRAM	1 (<0.1%)	1 (<0.1%)	2 (<0.1%)
CITRIC ACID	1 (<0.1%)	1 (<0.1%)	2 (<0.1%)
CLAVULANIC ACID	1 (<0.1%)	1 (<0.1%)	2 (<0.1%)
CLIDINIUM BROMIDE	1 (<0.1%)	1 (<0.1%)	2 (<0.1%)
COUGH AND COLD PREPARATIONS	0	2 (0.1%)	2 (<0.1%)
DESLORATADINE	0	2 (0.1%)	2 (<0.1%)
DICLOFENAC SODIUM	1 (<0.1%)	1 (<0.1%)	2 (<0.1%)
DIMENHYDRINATE	2 (0.1%)	0	2 (<0.1%)
DIOSMIN	0	2 (0.1%)	2 (<0.1%)
DOXYCYCLINE HYCLATE	0	2 (0.1%)	2 (<0.1%)
EPHEDRINE HYDROCHLORIDE	1 (<0.1%)	1 (<0.1%)	2 (<0.1%)
ERYTHROMYCIN	1 (<0.1%)	1 (<0.1%)	2 (<0.1%)
ESOMEPRAZOLE MAGNESIUM	0	2 (0.1%)	2 (<0.1%)
FLUCLOXACILLIN SODIUM	0	2 (0.1%)	2 (<0.1%)
GATIFLOXACIN	1 (<0.1%)	1 (<0.1%)	2 (<0.1%)
GLUCOSAMINE	0	2 (0.1%)	2 (<0.1%)
HERBAL PREPARATION	0	2 (0.1%)	2 (<0.1%)
HESPERIDIN	0	2 (0.1%)	2 (<0.1%)
HYDROXYZINE	0	2 (0.1%)	2 (<0.1%)
HYOSCINE BUTYLBROMIDE	1 (<0.1%)	1 (<0.1%)	2 (<0.1%)
INOSINE PRANOBEX	2 (0.1%)	0	2 (<0.1%)
INTRAVAGINAL CONTRACEPTIVES	0	2 (0.1%)	2 (<0.1%)
IRON	2 (0.1%)	0	2 (<0.1%)
ITRACONAZOLE	2 (0.1%)	0	2 (<0.1%)
KETOROLAC TROMETHAMINE	1 (<0.1%)	1 (<0.1%)	2 (<0.1%)
LEVOCETIRIZINE DIHYDROCHLORIDE	1 (<0.1%)	1 (<0.1%)	2 (<0.1%)
LORAZEPAM	1 (<0.1%)	1 (<0.1%)	2 (<0.1%)
METAXALONE	0	2 (0.1%)	2 (<0.1%)
METHYLERGOMETRINE MALEATE	0	2 (0.1%)	2 (<0.1%)
MIRTAZAPINE	1 (<0.1%)	1 (<0.1%)	2 (<0.1%)
MOMETASONE FUROATE	2 (0.1%)	0	2 (<0.1%)
NONOXINOL	2 (0.1%)	0	2 (<0.1%)
OPIUM DERIVATIVES AND EXPECTORANTS	0	2 (0.1%)	2 (<0.1%)
ORLISTAT	2 (0.1%)	0	2 (<0.1%)
ORPHENADRINE CITRATE	0	2 (0.1%)	2 (<0.1%)
OXYCODONE HYDROCHLORIDE	0	2 (0.1%)	2 (<0.1%)
PENTOXYVERINE CITRATE	1 (<0.1%)	1 (<0.1%)	2 (<0.1%)
PHENOXYMETHYLPENICILLIN	0	2 (0.1%)	2 (<0.1%)
PHENYLPROPANOLAMINE HYDROCHLORIDE	0	2 (0.1%)	2 (<0.1%)
PREDNISONE	2 (0.1%)	0	2 (<0.1%)
RIZATRIPTAN	2 (0.1%)	0	2 (<0.1%)
SALICYLAMIDE	2 (0.1%)	0	2 (<0.1%)
SODIUM BICARBONATE	1 (<0.1%)	1 (<0.1%)	2 (<0.1%)
TINIDAZOLE	0	2 (0.1%)	2 (<0.1%)

Table 14.1.1 / 37: Number of subjects who took at least one prior medication (FAS)

Substance	LCS12 N=1432 (100%)	LCS16 N=1452 (100%)	Total N=2884 (100%)
TOLFENAMIC ACID	0	2 (0.1%)	2 (<0.1%)
TOPIRAMATE	2 (0.1%)	0	2 (<0.1%)
TRAMADOL	0	2 (0.1%)	2 (<0.1%)
TRIAMCINOLONE ACETONIDE	2 (0.1%)	0	2 (<0.1%)
VALACICLOVIR HYDROCHLORIDE	1 (<0.1%)	1 (<0.1%)	2 (<0.1%)
VARENICLINE TARTRATE	0	2 (0.1%)	2 (<0.1%)
VENLAFAXINE HYDROCHLORIDE	0	2 (0.1%)	2 (<0.1%)
ACETYLCYSTEINE	0	1 (<0.1%)	1 (<0.1%)
ALLIUM SATIVUM	1 (<0.1%)	0	1 (<0.1%)
AMBROXOL HYDROCHLORIDE	1 (<0.1%)	0	1 (<0.1%)
AMCINONIDE	1 (<0.1%)	0	1 (<0.1%)
AMITRIPTYLINE HYDROCHLORIDE	1 (<0.1%)	0	1 (<0.1%)
AMMONIUM CARBONATE	0	1 (<0.1%)	1 (<0.1%)
ANAESTHETICS	0	1 (<0.1%)	1 (<0.1%)
ANAESTHETICS, LOCAL	0	1 (<0.1%)	1 (<0.1%)
ANTIINFECTIVES/ANTISEPT.,EXCL COMB WITH CORTI	0	1 (<0.1%)	1 (<0.1%)
ANTISPASMODICS IN COMBINATION WITH ANALGESICS	0	1 (<0.1%)	1 (<0.1%)
BACITRACIN	1 (<0.1%)	0	1 (<0.1%)
BACITRACIN ZINC	1 (<0.1%)	0	1 (<0.1%)
BACTERIA NOS	1 (<0.1%)	0	1 (<0.1%)
BAMIPINE	0	1 (<0.1%)	1 (<0.1%)
BENZALKONIUM CHLORIDE	1 (<0.1%)	0	1 (<0.1%)
BENZYLPENICILLIN PROCAINE	0	1 (<0.1%)	1 (<0.1%)
BETAMETHASONE VALERATE	0	1 (<0.1%)	1 (<0.1%)
BIFIDOBACTERIUM BIFIDUM	1 (<0.1%)	0	1 (<0.1%)
BISOPROLOL	1 (<0.1%)	0	1 (<0.1%)
BOTULINUM TOXIN TYPE A	0	1 (<0.1%)	1 (<0.1%)
BROMAZEPAM	1 (<0.1%)	0	1 (<0.1%)
BROMOCRIPTINE MESILATE	1 (<0.1%)	0	1 (<0.1%)
BUTALBITAL	1 (<0.1%)	0	1 (<0.1%)
BUTAMIRATE CITRATE	1 (<0.1%)	0	1 (<0.1%)
BUTOCONAZOLE NITRATE	0	1 (<0.1%)	1 (<0.1%)
CABERGOLINE	1 (<0.1%)	0	1 (<0.1%)
CALCIUM CARBONATE	1 (<0.1%)	0	1 (<0.1%)
CAMPHOR	0	1 (<0.1%)	1 (<0.1%)
CARBAMAZEPINE	0	1 (<0.1%)	1 (<0.1%)
CARBASALATE CALCIUM	0	1 (<0.1%)	1 (<0.1%)
CARBOCISTEINE	1 (<0.1%)	0	1 (<0.1%)
CARISOPRODOL	0	1 (<0.1%)	1 (<0.1%)
CEFADROXIL	0	1 (<0.1%)	1 (<0.1%)
CEFDINIR	1 (<0.1%)	0	1 (<0.1%)
CEFIXIME	1 (<0.1%)	0	1 (<0.1%)
CEFTRIAXONE	0	1 (<0.1%)	1 (<0.1%)
CEFUROXIME	1 (<0.1%)	0	1 (<0.1%)
CEFUROXIME SODIUM	1 (<0.1%)	0	1 (<0.1%)

Table 14.1.1 / 37: Number of subjects who took at least one prior medication (FAS)

Substance	LCS12 N=1432 (100%)	LCS16 N=1452 (100%)	Total N=2884 (100%)
CEPHAELIS SPP. FLUID EXTRACT	0	1 (<0.1%)	1 (<0.1%)
CHLORAMPHENICOL	0	1 (<0.1%)	1 (<0.1%)
CHLORDIAZEPOXIDE	1 (<0.1%)	0	1 (<0.1%)
CHLORDIAZEPOXIDE HYDROCHLORIDE	0	1 (<0.1%)	1 (<0.1%)
CHLOROFORM	0	1 (<0.1%)	1 (<0.1%)
CHLOROQUINE PHOSPHATE	1 (<0.1%)	0	1 (<0.1%)
CHONDROITIN	0	1 (<0.1%)	1 (<0.1%)
CITALOPRAM HYDROBROMIDE	1 (<0.1%)	0	1 (<0.1%)
CLONIXIN	1 (<0.1%)	0	1 (<0.1%)
COMBINATIONS OF ANTIBACTERIALS	1 (<0.1%)	0	1 (<0.1%)
CORTICOSTEROID NOS	1 (<0.1%)	0	1 (<0.1%)
CORTISONE	0	1 (<0.1%)	1 (<0.1%)
CYCLIZINE HYDROCHLORIDE	1 (<0.1%)	0	1 (<0.1%)
CYCLOBENZAPRINE	1 (<0.1%)	0	1 (<0.1%)
DARIFENACIN HYDROBROMIDE	1 (<0.1%)	0	1 (<0.1%)
DEXAMETHASONE	1 (<0.1%)	0	1 (<0.1%)
DICLOFENAC POTASSIUM	1 (<0.1%)	0	1 (<0.1%)
DICLOFENAMIDE	1 (<0.1%)	0	1 (<0.1%)
DIMETICONE	1 (<0.1%)	0	1 (<0.1%)
DIMETINDENE MALEATE	1 (<0.1%)	0	1 (<0.1%)
DIPHENHYDRAMINE	0	1 (<0.1%)	1 (<0.1%)
DIPYRIDAMOLE	0	1 (<0.1%)	1 (<0.1%)
DOCOSAHEXANOIC ACID	1 (<0.1%)	0	1 (<0.1%)
DOCUSATE SODIUM	0	1 (<0.1%)	1 (<0.1%)
DOMPERIDONE	1 (<0.1%)	0	1 (<0.1%)
DOXYCYCLINE HYDROCHLORIDE	1 (<0.1%)	0	1 (<0.1%)
DRUGS USED IN NICOTINE DEPENDENCE	1 (<0.1%)	0	1 (<0.1%)
ECHINACEA PURPUREA	1 (<0.1%)	0	1 (<0.1%)
ECONAZOLE NITRATE	1 (<0.1%)	0	1 (<0.1%)
EICOSAPENTAENOIC ACID	1 (<0.1%)	0	1 (<0.1%)
ENOXAPARIN SODIUM	1 (<0.1%)	0	1 (<0.1%)
ENTEROCOCCUS FAECIUM	1 (<0.1%)	0	1 (<0.1%)
ESCITALOPRAM	0	1 (<0.1%)	1 (<0.1%)
ESCITALOPRAM OXALATE	1 (<0.1%)	0	1 (<0.1%)
ESTRADIOL	0	1 (<0.1%)	1 (<0.1%)
ESZOPICLONE	1 (<0.1%)	0	1 (<0.1%)
ETHYLMORPHINE HYDROCHLORIDE	1 (<0.1%)	0	1 (<0.1%)
ETORICOXIB	0	1 (<0.1%)	1 (<0.1%)
FAMOTIDINE	1 (<0.1%)	0	1 (<0.1%)
FELYPRESSIN	0	1 (<0.1%)	1 (<0.1%)
FENTICONAZOLE NITRATE	0	1 (<0.1%)	1 (<0.1%)
FERRIC SODIUM GLUCONATE COMPLEX	1 (<0.1%)	0	1 (<0.1%)
FEXOFENADINE	1 (<0.1%)	0	1 (<0.1%)
FLUNARIZINE	1 (<0.1%)	0	1 (<0.1%)
FLUVOXAMINE MALEATE	1 (<0.1%)	0	1 (<0.1%)

Table 14.1.1 / 37: Number of subjects who took at least one prior medication (FAS)

Substance	LCS12 N=1432 (100%)	LCS16 N=1452 (100%)	Total N=2884 (100%)
FORMOTEROL FUMARATE	1 (<0.1%)	0	1 (<0.1%)
GELATIN	1 (<0.1%)	0	1 (<0.1%)
GENTAMICIN	0	1 (<0.1%)	1 (<0.1%)
GRAMICIDIN	1 (<0.1%)	0	1 (<0.1%)
GUAREA GUIDONIA	1 (<0.1%)	0	1 (<0.1%)
GYNAECOLOGICAL ANTIINFECTIVES AND ANTISEPTICS	0	1 (<0.1%)	1 (<0.1%)
HAEMOPHILUS INFLUENZAE	1 (<0.1%)	0	1 (<0.1%)
HEDERA HELIX	0	1 (<0.1%)	1 (<0.1%)
HOMATROPINE METHYLBROMIDE	1 (<0.1%)	0	1 (<0.1%)
HOMEOPATIC PREPARATION	1 (<0.1%)	0	1 (<0.1%)
HOMEOPATICS NOS	1 (<0.1%)	0	1 (<0.1%)
HYDROCHLOROTHIAZIDE	0	1 (<0.1%)	1 (<0.1%)
HYDROMORPHONE HYDROCHLORIDE	0	1 (<0.1%)	1 (<0.1%)
HYOSCYAMINE SULFATE	0	1 (<0.1%)	1 (<0.1%)
IMIDAZOLE DERIVATIVES	1 (<0.1%)	0	1 (<0.1%)
IMIQUIMOD	1 (<0.1%)	0	1 (<0.1%)
INFLUENZA VACCINE	0	1 (<0.1%)	1 (<0.1%)
IRON IN OTHER COMBINATIONS	0	1 (<0.1%)	1 (<0.1%)
ISOTRETINOIN	1 (<0.1%)	0	1 (<0.1%)
KETOCONAZOLE	0	1 (<0.1%)	1 (<0.1%)
KETOROLAC	0	1 (<0.1%)	1 (<0.1%)
KLEBSIELLA PNEUMONIAE	1 (<0.1%)	0	1 (<0.1%)
LANSOPRAZOLE	0	1 (<0.1%)	1 (<0.1%)
LEVOTHYROXINE SODIUM	0	1 (<0.1%)	1 (<0.1%)
LYNESTRENOL	0	1 (<0.1%)	1 (<0.1%)
MACROCYSTIS PYRIFERA	1 (<0.1%)	0	1 (<0.1%)
MACROGOL	0	1 (<0.1%)	1 (<0.1%)
MAGNESIUM CARBONATE	1 (<0.1%)	0	1 (<0.1%)
MECLOZINE	1 (<0.1%)	0	1 (<0.1%)
MENINGOCOCCAL VACCINES	1 (<0.1%)	0	1 (<0.1%)
MENTHOL	0	1 (<0.1%)	1 (<0.1%)
MESALAZINE	1 (<0.1%)	0	1 (<0.1%)
METFORMIN	0	1 (<0.1%)	1 (<0.1%)
METHENAMINE	0	1 (<0.1%)	1 (<0.1%)
METHYLCELLULOSE	1 (<0.1%)	0	1 (<0.1%)
METHYLPREDNISOLONE	0	1 (<0.1%)	1 (<0.1%)
METHYLTHIONINIUM CHLORIDE	0	1 (<0.1%)	1 (<0.1%)
METOCLOPRAMIDE	0	1 (<0.1%)	1 (<0.1%)
METOCLOPRAMIDE HYDROCHLORIDE	1 (<0.1%)	0	1 (<0.1%)
MICONAZOLE	0	1 (<0.1%)	1 (<0.1%)
MIDAZOLAM HYDROCHLORIDE	0	1 (<0.1%)	1 (<0.1%)
MINOCYCLINE	0	1 (<0.1%)	1 (<0.1%)
NASAL PREPARATIONS	1 (<0.1%)	0	1 (<0.1%)
NATAMYCIN	1 (<0.1%)	0	1 (<0.1%)
NEISSERIA CATARRHALIS	1 (<0.1%)	0	1 (<0.1%)

Table 14.1.1 / 37: Number of subjects who took at least one prior medication (FAS)

Substance	LCS12 N=1432 (100%)	LCS16 N=1452 (100%)	Total N=2884 (100%)
NOMEGESTROL ACETATE	0	1 (<0.1%)	1 (<0.1%)
NORFLOXACIN	1 (<0.1%)	0	1 (<0.1%)
NORMETHADONE HYDROCHLORIDE	1 (<0.1%)	0	1 (<0.1%)
ONDANSETRON	0	1 (<0.1%)	1 (<0.1%)
OXILOFRINE HYDROCHLORIDE	1 (<0.1%)	0	1 (<0.1%)
PANAX GINSENG	1 (<0.1%)	0	1 (<0.1%)
PANTOPRAZOLE SODIUM	0	1 (<0.1%)	1 (<0.1%)
PARGEVERINE HYDROCHLORIDE	0	1 (<0.1%)	1 (<0.1%)
PENTOSAN POLYSULFATE SODIUM	0	1 (<0.1%)	1 (<0.1%)
PHENIRAMINE MALEATE	0	1 (<0.1%)	1 (<0.1%)
PHENTERMINE	1 (<0.1%)	0	1 (<0.1%)
PHENYL SALICYLATE	0	1 (<0.1%)	1 (<0.1%)
PHENYLPROPANOLAMINE	0	1 (<0.1%)	1 (<0.1%)
PHOSPHORIC ACID SODIUM	0	1 (<0.1%)	1 (<0.1%)
PIROXICAM BETADEX	0	1 (<0.1%)	1 (<0.1%)
PODOPHYLLOTOXIN	0	1 (<0.1%)	1 (<0.1%)
POLICRESULEN	1 (<0.1%)	0	1 (<0.1%)
POLIOMYELITIS VACCINE	0	1 (<0.1%)	1 (<0.1%)
POLYACRYLIC ACID	1 (<0.1%)	0	1 (<0.1%)
POLYSORBATE 20	0	1 (<0.1%)	1 (<0.1%)
POTASSIUM BICARBONATE	0	1 (<0.1%)	1 (<0.1%)
POTASSIUM CHLORIDE	0	1 (<0.1%)	1 (<0.1%)
PREDNISOLONE	0	1 (<0.1%)	1 (<0.1%)
PREDNISOLONE ACETATE	1 (<0.1%)	0	1 (<0.1%)
PREDNISOLONE SODIUM PHOSPHATE	1 (<0.1%)	0	1 (<0.1%)
PRIDINOL MESILATE	0	1 (<0.1%)	1 (<0.1%)
PRILOCAINE	0	1 (<0.1%)	1 (<0.1%)
PROCHLORPERAZINE EDISYLATE	0	1 (<0.1%)	1 (<0.1%)
PROMETHAZINE HYDROCHLORIDE	0	1 (<0.1%)	1 (<0.1%)
PROPYPHENAZONE	0	1 (<0.1%)	1 (<0.1%)
PSYLLIUM HYDROPHILIC MUCILLOID	1 (<0.1%)	0	1 (<0.1%)
PYRIDOXINE HYDROCHLORIDE	0	1 (<0.1%)	1 (<0.1%)
RAMIPRIL	0	1 (<0.1%)	1 (<0.1%)
ROXITHROMYCIN	1 (<0.1%)	0	1 (<0.1%)
SALBUTAMOL	1 (<0.1%)	0	1 (<0.1%)
SIBUTRAMINE	1 (<0.1%)	0	1 (<0.1%)
SIBUTRAMINE HYDROCHLORIDE	1 (<0.1%)	0	1 (<0.1%)
SILYBUM MARIANUM	1 (<0.1%)	0	1 (<0.1%)
SODIUM CHLORIDE	0	1 (<0.1%)	1 (<0.1%)
SODIUM CITRATE	0	1 (<0.1%)	1 (<0.1%)
SPIRONOLACTONE	1 (<0.1%)	0	1 (<0.1%)
STREPTOCOCCUS PNEUMONIAE	1 (<0.1%)	0	1 (<0.1%)
STREPTOCOCCUS PYOGENES	1 (<0.1%)	0	1 (<0.1%)
STREPTOCOCCUS VIRIDANS	1 (<0.1%)	0	1 (<0.1%)
SULFANILAMIDE	0	1 (<0.1%)	1 (<0.1%)

Table 14.1.1 / 37: Number of subjects who took at least one prior medication (FAS)

Substance	LCS12 N=1432 (100%)	LCS16 N=1452 (100%)	Total N=2884 (100%)
SULFOGAIACOL	0	1 (<0.1%)	1 (<0.1%)
TEMAZEPAM	0	1 (<0.1%)	1 (<0.1%)
TERBUTALINE SULFATE	1 (<0.1%)	0	1 (<0.1%)
TETANUS VACCINE	0	1 (<0.1%)	1 (<0.1%)
TETRACYCLINE HYDROCHLORIDE	1 (<0.1%)	0	1 (<0.1%)
TETRYZOLINE HYDROCHLORIDE	1 (<0.1%)	0	1 (<0.1%)
TRAZODONE	0	1 (<0.1%)	1 (<0.1%)
TRIAMTERENE	0	1 (<0.1%)	1 (<0.1%)
TRIPROLIDINE HYDROCHLORIDE	1 (<0.1%)	0	1 (<0.1%)
TYPHOID VACCINE	0	1 (<0.1%)	1 (<0.1%)
VENLAFAXINE	1 (<0.1%)	0	1 (<0.1%)
VITAMINS	0	1 (<0.1%)	1 (<0.1%)
VITAMINS WITH MINERALS	0	1 (<0.1%)	1 (<0.1%)
XYLOMETAZOLINE HYDROCHLORIDE	0	1 (<0.1%)	1 (<0.1%)
ZINC	0	1 (<0.1%)	1 (<0.1%)
ZINC OXIDE	1 (<0.1%)	0	1 (<0.1%)
ZOLMITRIPTAN	1 (<0.1%)	0	1 (<0.1%)
ZOPICLONE	0	1 (<0.1%)	1 (<0.1%)

Note: Multiple substances per drug are possible. Therefore, the same drug may be counted for more than one substance for the same subject
Note: All documented medications that ended before start of treatment are included in this table
Global Biostatistics: /by-sasp/patdb/projects/de04209/310442/stat/prod_interim03/pgms/t-cm.sas epkll 07OCT2011 10:50
End of table

Table 14.1.1 / 38: Number of subjects who took at least one concomitant medication (FAS)

Substance	LCS12 N=1432 (100%)	LCS16 N=1452 (100%)	Total N=2884 (100%)
Number of subjects (%) with at least one concomitant medication	1191 (83.2%)	1215 (83.7%)	2406 (83.4%)
IBUPROFEN	572 (39.9%)	598 (41.2%)	1170 (40.6%)
PARACETAMOL	442 (30.9%)	467 (32.2%)	909 (31.5%)
METRONIDAZOLE	150 (10.5%)	174 (12.0%)	324 (11.2%)
FLUCONAZOLE	138 (9.6%)	151 (10.4%)	289 (10.0%)
AMOXICILLIN	94 (6.6%)	99 (6.8%)	193 (6.7%)
AZITHROMYCIN	89 (6.2%)	93 (6.4%)	182 (6.3%)
LIDOCAINE	81 (5.7%)	91 (6.3%)	172 (6.0%)
CIPROFLOXACIN	94 (6.6%)	77 (5.3%)	171 (5.9%)
VITAMINS NOS	82 (5.7%)	87 (6.0%)	169 (5.9%)
NAPROXEN	80 (5.6%)	88 (6.1%)	168 (5.8%)
PSEUDOEPHEDRINE HYDROCHLORIDE	77 (5.4%)	84 (5.8%)	161 (5.6%)
ETHINYLESTRADIOL	78 (5.4%)	81 (5.6%)	159 (5.5%)
CAFFEINE	80 (5.6%)	74 (5.1%)	154 (5.3%)
CEFALEXIN	78 (5.4%)	75 (5.2%)	153 (5.3%)
DICLOFENAC	70 (4.9%)	79 (5.4%)	149 (5.2%)
NAPROXEN SODIUM	78 (5.4%)	69 (4.8%)	147 (5.1%)
ACETYLSALICYLIC ACID	72 (5.0%)	64 (4.4%)	136 (4.7%)
ASCORBIC ACID	76 (5.3%)	51 (3.5%)	127 (4.4%)
CLOTRIMAZOLE	65 (4.5%)	59 (4.1%)	124 (4.3%)
TRIMETHOPRIM	57 (4.0%)	61 (4.2%)	118 (4.1%)
MICONAZOLE NITRATE	52 (3.6%)	62 (4.3%)	114 (4.0%)
CODEINE PHOSPHATE	47 (3.3%)	57 (3.9%)	104 (3.6%)
SALBUTAMOL	52 (3.6%)	52 (3.6%)	104 (3.6%)
CETIRIZINE HYDROCHLORIDE	47 (3.3%)	55 (3.8%)	102 (3.5%)
LORATADINE	54 (3.8%)	47 (3.2%)	101 (3.5%)
NITROFURANTOIN	63 (4.4%)	38 (2.6%)	101 (3.5%)
DOXYCYCLINE	48 (3.4%)	52 (3.6%)	100 (3.5%)
AMOXICILLIN TRIHYDRATE	47 (3.3%)	49 (3.4%)	96 (3.3%)
LIDOCAINE HYDROCHLORIDE	48 (3.4%)	48 (3.3%)	96 (3.3%)
DEXTROMETHORPHAN HYDROBROMIDE	51 (3.6%)	44 (3.0%)	95 (3.3%)
CLAVULANATE POTASSIUM	43 (3.0%)	44 (3.0%)	87 (3.0%)
HYDROCODONE BITARTRATE	40 (2.8%)	47 (3.2%)	87 (3.0%)
DIPHENHYDRAMINE HYDROCHLORIDE	35 (2.4%)	44 (3.0%)	79 (2.7%)
PHENOXYMETHYLPENICILLIN POTASSIUM	43 (3.0%)	35 (2.4%)	78 (2.7%)
LEVOTHYROXINE SODIUM	33 (2.3%)	39 (2.7%)	72 (2.5%)
FLUTICASONE PROPIONATE	36 (2.5%)	31 (2.1%)	67 (2.3%)
GUAIFENESIN	36 (2.5%)	28 (1.9%)	64 (2.2%)
FOLIC ACID	29 (2.0%)	34 (2.3%)	63 (2.2%)
OXYCODONE HYDROCHLORIDE	26 (1.8%)	35 (2.4%)	61 (2.1%)
SULFAMETHOXAZOLE	28 (2.0%)	33 (2.3%)	61 (2.1%)
CLINDAMYCIN PHOSPHATE	28 (2.0%)	32 (2.2%)	60 (2.1%)
PIVMECILLINAM	39 (2.7%)	21 (1.4%)	60 (2.1%)

Table 14.1.1 / 38: Number of subjects who took at least one concomitant medication (FAS)

Substance	LCS12<br>N=1432 (100%)	LCS16<br>N=1452 (100%)	Total<br>N=2884 (100%)
MOMETASONE FUROATE	32 (2.2%)	27 (1.9%)	59 (2.0%)
CHLORPHENAMINE MALEATE	31 (2.2%)	27 (1.9%)	58 (2.0%)
CITALOPRAM HYDROBROMIDE	34 (2.4%)	22 (1.5%)	56 (1.9%)
DICLOFENAC SODIUM	27 (1.9%)	26 (1.8%)	53 (1.8%)
INFLUENZA VACCINE	22 (1.5%)	31 (2.1%)	53 (1.8%)
HYDROCORTISONE	28 (2.0%)	24 (1.7%)	52 (1.8%)
ONDANSETRON	28 (2.0%)	24 (1.7%)	52 (1.8%)
NYSTATIN	25 (1.7%)	25 (1.7%)	50 (1.7%)
ESCITALOPRAM OXALATE	29 (2.0%)	20 (1.4%)	49 (1.7%)
IMIDAZOLE DERIVATIVES	18 (1.3%)	31 (2.1%)	49 (1.7%)
DOXYLAMINE SUCCINATE	23 (1.6%)	25 (1.7%)	48 (1.7%)
SERTRALINE	21 (1.5%)	27 (1.9%)	48 (1.7%)
ACICLOVIR	24 (1.7%)	23 (1.6%)	47 (1.6%)
BUDESONIDE	25 (1.7%)	22 (1.5%)	47 (1.6%)
MINERALS NOS	24 (1.7%)	23 (1.6%)	47 (1.6%)
ACRIVASTINE	20 (1.4%)	26 (1.8%)	46 (1.6%)
RETINOL	31 (2.2%)	15 (1.0%)	46 (1.6%)
CLARITHROMYCIN	24 (1.7%)	21 (1.4%)	45 (1.6%)
CLINDAMYCIN	21 (1.5%)	24 (1.7%)	45 (1.6%)
CLINDAMYCIN HYDROCHLORIDE	24 (1.7%)	21 (1.4%)	45 (1.6%)
KETOPROFEN	18 (1.3%)	27 (1.9%)	45 (1.6%)
MISOPROSTOL	22 (1.5%)	23 (1.6%)	45 (1.6%)
HYDROCODONE	21 (1.5%)	23 (1.6%)	44 (1.5%)
OMEPRAZOLE	23 (1.6%)	21 (1.4%)	44 (1.5%)
ALPRAZOLAM	19 (1.3%)	24 (1.7%)	43 (1.5%)
PREDNISONE	20 (1.4%)	23 (1.6%)	43 (1.5%)
ETHANOL	19 (1.3%)	23 (1.6%)	42 (1.5%)
METOCLOPRAMIDE	23 (1.6%)	19 (1.3%)	42 (1.5%)
DOXYCYCLINE HYDROCHLORIDE	18 (1.3%)	23 (1.6%)	41 (1.4%)
TRIAMCINOLONE ACETONIDE	23 (1.6%)	18 (1.2%)	41 (1.4%)
DEXAMETHASONE	24 (1.7%)	16 (1.1%)	40 (1.4%)
DIAZEPAM	19 (1.3%)	21 (1.4%)	40 (1.4%)
LEVOFLOXACIN	24 (1.7%)	16 (1.1%)	40 (1.4%)
IRON	15 (1.0%)	24 (1.7%)	39 (1.4%)
AMOXICILLIN SODIUM	26 (1.8%)	12 (0.8%)	38 (1.3%)
EPHEDRINE SULFATE	18 (1.3%)	20 (1.4%)	38 (1.3%)
VALACICLOVIR HYDROCHLORIDE	21 (1.5%)	17 (1.2%)	38 (1.3%)
KETOROLAC TROMETHAMINE	20 (1.4%)	17 (1.2%)	37 (1.3%)
TERCONAZOLE	18 (1.3%)	19 (1.3%)	37 (1.3%)
COLECALCIFEROL	14 (1.0%)	22 (1.5%)	36 (1.2%)
PROMETHAZINE	16 (1.1%)	20 (1.4%)	36 (1.2%)
DROSPIRENONE	18 (1.3%)	17 (1.2%)	35 (1.2%)
CYANOCOBALAMIN	15 (1.0%)	19 (1.3%)	34 (1.2%)
ESOMEPRAZOLE MAGNESIUM	13 (0.9%)	21 (1.4%)	34 (1.2%)
LEVONORGESTREL	21 (1.5%)	13 (0.9%)	34 (1.2%)

Table 14.1.1 / 38: Number of subjects who took at least one concomitant medication (FAS)

Substance	LCS12 N=1432 (100%)	LCS16 N=1452 (100%)	Total N=2884 (100%)
SODIUM CHLORIDE	16 (1.1%)	18 (1.2%)	34 (1.2%)
SUMATRIPTAN	16 (1.1%)	18 (1.2%)	34 (1.2%)
ECONAZOLE	17 (1.2%)	16 (1.1%)	33 (1.1%)
ERGOCALCIFEROL	22 (1.5%)	11 (0.8%)	33 (1.1%)
ERYTHROMYCIN	13 (0.9%)	20 (1.4%)	33 (1.1%)
MORPHINE	16 (1.1%)	17 (1.2%)	33 (1.1%)
PHENYLEPHRINE HYDROCHLORIDE	14 (1.0%)	19 (1.3%)	33 (1.1%)
THIAMINE HYDROCHLORIDE	19 (1.3%)	14 (1.0%)	33 (1.1%)
VENLAFAXINE	20 (1.4%)	13 (0.9%)	33 (1.1%)
FEXOFENADINE	11 (0.8%)	21 (1.4%)	32 (1.1%)
HYOSCINE BUTYLBROMIDE	15 (1.0%)	17 (1.2%)	32 (1.1%)
NICOTINAMIDE	19 (1.3%)	13 (0.9%)	32 (1.1%)
RIBOFLAVIN	19 (1.3%)	13 (0.9%)	32 (1.1%)
DESLORATADINE	18 (1.3%)	13 (0.9%)	31 (1.1%)
DEXTROPROPOXYPHENE NAPSILATE	19 (1.3%)	12 (0.8%)	31 (1.1%)
EPINEPHRINE	14 (1.0%)	17 (1.2%)	31 (1.1%)
ESCITALOPRAM	13 (0.9%)	18 (1.2%)	31 (1.1%)
FENTANYL	19 (1.3%)	12 (0.8%)	31 (1.1%)
TIZANIDINE HYDROCHLORIDE	14 (1.0%)	17 (1.2%)	31 (1.1%)
CALCIUM	14 (1.0%)	16 (1.1%)	30 (1.0%)
FLUOXETINE HYDROCHLORIDE	17 (1.2%)	13 (0.9%)	30 (1.0%)
PROPOFOL	17 (1.2%)	13 (0.9%)	30 (1.0%)
BUPROPION	16 (1.1%)	13 (0.9%)	29 (1.0%)
METAMIZOLE SODIUM	12 (0.8%)	17 (1.2%)	29 (1.0%)
PHENTERMINE	15 (1.0%)	14 (1.0%)	29 (1.0%)
BENZOCAINE	10 (0.7%)	18 (1.2%)	28 (1.0%)
BENZOYL PEROXIDE	14 (1.0%)	14 (1.0%)	28 (1.0%)
BETA-LACTAMASE SENSITIVE PENICILLINS	13 (0.9%)	15 (1.0%)	28 (1.0%)
EBASTINE	13 (0.9%)	15 (1.0%)	28 (1.0%)
ZOLPIDEM TARTRATE	11 (0.8%)	17 (1.2%)	28 (1.0%)
CLONAZEPAM	11 (0.8%)	16 (1.1%)	27 (0.9%)
ETORICOXIB	17 (1.2%)	10 (0.7%)	27 (0.9%)
KETOROLAC	11 (0.8%)	16 (1.1%)	27 (0.9%)
LANSOPRAZOLE	11 (0.8%)	16 (1.1%)	27 (0.9%)
MINOCYCLINE	11 (0.8%)	16 (1.1%)	27 (0.9%)
MONTELUKAST	6 (0.4%)	21 (1.4%)	27 (0.9%)
PANTHENOL	18 (1.3%)	9 (0.6%)	27 (0.9%)
PHENAZOPYRIDINE HYDROCHLORIDE	13 (0.9%)	14 (1.0%)	27 (0.9%)
PSEUDOEPHEDRINE SULFATE	16 (1.1%)	11 (0.8%)	27 (0.9%)
BETAMETHASONE	12 (0.8%)	14 (1.0%)	26 (0.9%)
LEVOCETIRIZINE DIHYDROCHLORIDE	12 (0.8%)	14 (1.0%)	26 (0.9%)
LOPERAMIDE HYDROCHLORIDE	12 (0.8%)	14 (1.0%)	26 (0.9%)
MULTIVITAMINS, PLAIN	10 (0.7%)	16 (1.1%)	26 (0.9%)
TRETINOIN	13 (0.9%)	13 (0.9%)	26 (0.9%)
CALCIUM CARBONATE	10 (0.7%)	15 (1.0%)	25 (0.9%)

Table 14.1.1 / 38: Number of subjects who took at least one concomitant medication (FAS)

Substance	LCS12 N=1432 (100%)	LCS16 N=1452 (100%)	Total N=2884 (100%)
FERROUS FUMARATE	15 (1.0%)	10 (0.7%)	25 (0.9%)
FISH OIL	11 (0.8%)	14 (1.0%)	25 (0.9%)
MELOXICAM	12 (0.8%)	13 (0.9%)	25 (0.9%)
TETRACYCLINE HYDROCHLORIDE	14 (1.0%)	11 (0.8%)	25 (0.9%)
MEPYRAMINE MALEATE	9 (0.6%)	15 (1.0%)	24 (0.8%)
RANITIDINE HYDROCHLORIDE	13 (0.9%)	11 (0.8%)	24 (0.8%)
TOCOPHEROL	14 (1.0%)	10 (0.7%)	24 (0.8%)
TRAMADOL HYDROCHLORIDE	11 (0.8%)	13 (0.9%)	24 (0.8%)
CYCLOBENZAPRINE HYDROCHLORIDE	10 (0.7%)	13 (0.9%)	23 (0.8%)
DICLOFENAC POTASSIUM	12 (0.8%)	11 (0.8%)	23 (0.8%)
HYDROCHLOROTHIAZIDE	12 (0.8%)	11 (0.8%)	23 (0.8%)
ISOTRETINOIN	15 (1.0%)	8 (0.6%)	23 (0.8%)
LORAZEPAM	8 (0.6%)	15 (1.0%)	23 (0.8%)
NORFLOXACIN	16 (1.1%)	7 (0.5%)	23 (0.8%)
POTASSIUM CHLORIDE	12 (0.8%)	11 (0.8%)	23 (0.8%)
ERGOTAMINE TARTRATE	10 (0.7%)	12 (0.8%)	22 (0.8%)
HEPATITIS A VACCINE	10 (0.7%)	12 (0.8%)	22 (0.8%)
NEOMYCIN SULFATE	10 (0.7%)	12 (0.8%)	22 (0.8%)
TERBUTALINE SULFATE	8 (0.6%)	14 (1.0%)	22 (0.8%)
TETRACYCLINE	11 (0.8%)	11 (0.8%)	22 (0.8%)
VIRAL VACCINES	9 (0.6%)	13 (0.9%)	22 (0.8%)
ANESTHETICS, GENERAL	11 (0.8%)	10 (0.7%)	21 (0.7%)
FERROUS SULFATE	8 (0.6%)	13 (0.9%)	21 (0.7%)
HEPATITIS B VACCINE	9 (0.6%)	12 (0.8%)	21 (0.7%)
OTHER NUTRIENTS	6 (0.4%)	15 (1.0%)	21 (0.7%)
PARGEVERINE	12 (0.8%)	9 (0.6%)	21 (0.7%)
PREDNISOLONE	11 (0.8%)	10 (0.7%)	21 (0.7%)
PRILOCAINE HYDROCHLORIDE	12 (0.8%)	9 (0.6%)	21 (0.7%)
TRAMADOL	9 (0.6%)	12 (0.8%)	21 (0.7%)
CITALOPRAM	10 (0.7%)	10 (0.7%)	20 (0.7%)
CLONIXIN LYSINATE	10 (0.7%)	10 (0.7%)	20 (0.7%)
TINIDAZOLE	8 (0.6%)	12 (0.8%)	20 (0.7%)
TOPIRAMATE	14 (1.0%)	6 (0.4%)	20 (0.7%)
BROMHEXINE HYDROCHLORIDE	9 (0.6%)	10 (0.7%)	19 (0.7%)
CHLORAMPHENICOL	9 (0.6%)	10 (0.7%)	19 (0.7%)
CLAVULANIC ACID	10 (0.7%)	9 (0.6%)	19 (0.7%)
FORMOTEROL FUMARATE	12 (0.8%)	7 (0.5%)	19 (0.7%)
HYDROMORPHONE HYDROCHLORIDE	10 (0.7%)	9 (0.6%)	19 (0.7%)
LAMOTRIGINE	9 (0.6%)	10 (0.7%)	19 (0.7%)
MEFENAMIC ACID	12 (0.8%)	7 (0.5%)	19 (0.7%)
OSELTAMIVIR	8 (0.6%)	11 (0.8%)	19 (0.7%)
SALBUTAMOL SULFATE	8 (0.6%)	11 (0.8%)	19 (0.7%)
SALMETEROL XINAFOATE	9 (0.6%)	10 (0.7%)	19 (0.7%)
ANILIDES	8 (0.6%)	10 (0.7%)	18 (0.6%)
CO-TRIMOXAZOLE	10 (0.7%)	8 (0.6%)	18 (0.6%)

Table 14.1.1 / 38: Number of subjects who took at least one concomitant medication (FAS)

Substance	LCS12 N=1432 (100%)	LCS16 N=1452 (100%)	Total N=2884 (100%)
ETONOGESTREL	5 (0.3%)	13 (0.9%)	18 (0.6%)
FUSIDIC ACID	8 (0.6%)	10 (0.7%)	18 (0.6%)
MICONAZOLE	4 (0.3%)	14 (1.0%)	18 (0.6%)
NORETHISTERONE ACETATE	10 (0.7%)	8 (0.6%)	18 (0.6%)
PANTOPRAZOLE	9 (0.6%)	9 (0.6%)	18 (0.6%)
PANTOPRAZOLE SODIUM	8 (0.6%)	10 (0.7%)	18 (0.6%)
PROPRANOLOL	10 (0.7%)	8 (0.6%)	18 (0.6%)
ADAPALENE	7 (0.5%)	10 (0.7%)	17 (0.6%)
ANTI-ACNE PREPARATIONS FOR TOPICAL USE	9 (0.6%)	8 (0.6%)	17 (0.6%)
ANTIDIARRHOEAL MICROORGANISMS	7 (0.5%)	10 (0.7%)	17 (0.6%)
CIPROFLOXACIN HYDROCHLORIDE	8 (0.6%)	9 (0.6%)	17 (0.6%)
LEVOTHYROXINE	8 (0.6%)	9 (0.6%)	17 (0.6%)
NORGESTIMATE	10 (0.7%)	7 (0.5%)	17 (0.6%)
PSEUDOEPHEDRINE	8 (0.6%)	9 (0.6%)	17 (0.6%)
ZOPICLONE	7 (0.5%)	10 (0.7%)	17 (0.6%)
AMFETAMINE SULFATE	8 (0.6%)	8 (0.6%)	16 (0.6%)
COUGH AND COLD PREPARATIONS	6 (0.4%)	10 (0.7%)	16 (0.6%)
DEXAMFETAMINE SULFATE	7 (0.5%)	9 (0.6%)	16 (0.6%)
DOCUSATE SODIUM	7 (0.5%)	9 (0.6%)	16 (0.6%)
FAMOTIDINE	9 (0.6%)	7 (0.5%)	16 (0.6%)
FLUOXETINE	4 (0.3%)	12 (0.8%)	16 (0.6%)
MINOCYCLINE HYDROCHLORIDE	12 (0.8%)	4 (0.3%)	16 (0.6%)
ORPHENADRINE CITRATE	6 (0.4%)	10 (0.7%)	16 (0.6%)
PHLOROGLUCINOL	7 (0.5%)	9 (0.6%)	16 (0.6%)
VITAMIN B NOS	8 (0.6%)	8 (0.6%)	16 (0.6%)
AMFETAMINE ASPARTATE	7 (0.5%)	8 (0.6%)	15 (0.5%)
BIOTIN	7 (0.5%)	8 (0.6%)	15 (0.5%)
CODEINE	10 (0.7%)	5 (0.3%)	15 (0.5%)
DEXAMFETAMINE SACCHARATE	7 (0.5%)	8 (0.6%)	15 (0.5%)
DOXYCYCLINE MONOHYDRATE	5 (0.3%)	10 (0.7%)	15 (0.5%)
EPHEDRINE HYDROCHLORIDE	6 (0.4%)	9 (0.6%)	15 (0.5%)
HERBAL PREPARATION	4 (0.3%)	11 (0.8%)	15 (0.5%)
PRILOCAINE	6 (0.4%)	9 (0.6%)	15 (0.5%)
VITAMINS	8 (0.6%)	7 (0.5%)	15 (0.5%)
ANTIBIOTICS	9 (0.6%)	5 (0.3%)	14 (0.5%)
CEFTRIAXONE	5 (0.3%)	9 (0.6%)	14 (0.5%)
CYCLOBENZAPRINE	7 (0.5%)	7 (0.5%)	14 (0.5%)
DESOGESTREL	6 (0.4%)	8 (0.6%)	14 (0.5%)
DIMENHYDRINATE	5 (0.3%)	9 (0.6%)	14 (0.5%)
DULOXETINE HYDROCHLORIDE	9 (0.6%)	5 (0.3%)	14 (0.5%)
FLUTICASONE	8 (0.6%)	6 (0.4%)	14 (0.5%)
MIDAZOLAM HYDROCHLORIDE	10 (0.7%)	4 (0.3%)	14 (0.5%)
NICOTINIC ACID	10 (0.7%)	4 (0.3%)	14 (0.5%)
NORETHISTERONE	9 (0.6%)	5 (0.3%)	14 (0.5%)
PETHIDINE HYDROCHLORIDE	7 (0.5%)	7 (0.5%)	14 (0.5%)

Table 14.1.1 / 38: Number of subjects who took at least one concomitant medication (FAS)

Substance	LCS12 N=1432 (100%)	LCS16 N=1452 (100%)	Total N=2884 (100%)
PODOPHYLLOTOXIN	7 (0.5%)	7 (0.5%)	14 (0.5%)
RIZATRIPTAN	7 (0.5%)	7 (0.5%)	14 (0.5%)
VITAMIN D NOS	7 (0.5%)	7 (0.5%)	14 (0.5%)
ANTISPASMODICS IN COMBINATION WITH ANALGESICS	7 (0.5%)	6 (0.4%)	13 (0.5%)
BECLOMETASONE DIPROPIONATE	5 (0.3%)	8 (0.6%)	13 (0.5%)
BETAMETHASONE VALERATE	4 (0.3%)	9 (0.6%)	13 (0.5%)
BUPROPION HYDROCHLORIDE	8 (0.6%)	5 (0.3%)	13 (0.5%)
CEFAZOLIN SODIUM	8 (0.6%)	5 (0.3%)	13 (0.5%)
CROMOGLICATE SODIUM	7 (0.5%)	6 (0.4%)	13 (0.5%)
EPINEPHRINE HYDROCHLORIDE	8 (0.6%)	5 (0.3%)	13 (0.5%)
ETHYLMORPHINE HYDROCHLORIDE	6 (0.4%)	7 (0.5%)	13 (0.5%)
FELYPRESSIN	6 (0.4%)	7 (0.5%)	13 (0.5%)
MAGNESIUM	5 (0.3%)	8 (0.6%)	13 (0.5%)
METHYLPREDNISOLONE	7 (0.5%)	6 (0.4%)	13 (0.5%)
MIRTAZAPINE	6 (0.4%)	7 (0.5%)	13 (0.5%)
OXAZEPAM	6 (0.4%)	7 (0.5%)	13 (0.5%)
PHENYLPROPANOLAMINE HYDROCHLORIDE	4 (0.3%)	9 (0.6%)	13 (0.5%)
POLYMYXIN B SULFATE	6 (0.4%)	7 (0.5%)	13 (0.5%)
PRIDINOL MESILATE	7 (0.5%)	6 (0.4%)	13 (0.5%)
PYRIDOXINE HYDROCHLORIDE	6 (0.4%)	7 (0.5%)	13 (0.5%)
QUETIAPINE	4 (0.3%)	9 (0.6%)	13 (0.5%)
TIOCONAZOLE	9 (0.6%)	4 (0.3%)	13 (0.5%)
TRANEXAMIC ACID	9 (0.6%)	4 (0.3%)	13 (0.5%)
UNCODEABLE "UNCLASSIFIABLE"	6 (0.4%)	7 (0.5%)	13 (0.5%)
ANTIINFECTIVES/ANTISEPT.,EXCL COMB WITH CORTI	5 (0.3%)	7 (0.5%)	12 (0.4%)
ATOVAQUONE	7 (0.5%)	5 (0.3%)	12 (0.4%)
AZELAIC ACID	5 (0.3%)	7 (0.5%)	12 (0.4%)
BUPIVACAINE	5 (0.3%)	7 (0.5%)	12 (0.4%)
CEFUROXIME	3 (0.2%)	9 (0.6%)	12 (0.4%)
CETIRIZINE	5 (0.3%)	7 (0.5%)	12 (0.4%)
GESTODENE	8 (0.6%)	4 (0.3%)	12 (0.4%)
HYDROXYZINE	4 (0.3%)	8 (0.6%)	12 (0.4%)
MACROGOL	5 (0.3%)	7 (0.5%)	12 (0.4%)
METFORMIN	5 (0.3%)	7 (0.5%)	12 (0.4%)
METHOCARBAMOL	6 (0.4%)	6 (0.4%)	12 (0.4%)
OFLOXACIN	6 (0.4%)	6 (0.4%)	12 (0.4%)
PAROXETINE HYDROCHLORIDE	6 (0.4%)	6 (0.4%)	12 (0.4%)
PROGUANIL HYDROCHLORIDE	7 (0.5%)	5 (0.3%)	12 (0.4%)
TERBINAFINE	7 (0.5%)	5 (0.3%)	12 (0.4%)
TRIMETHYLPHLOROGLUCINOL	6 (0.4%)	6 (0.4%)	12 (0.4%)
VARENICLINE TARTRATE	5 (0.3%)	7 (0.5%)	12 (0.4%)
XYLOMETAZOLINE HYDROCHLORIDE	2 (0.1%)	10 (0.7%)	12 (0.4%)
AMITRIPTYLINE HYDROCHLORIDE	5 (0.3%)	6 (0.4%)	11 (0.4%)
ANTIINFECTIVES/ANTISEPTICS IN COMB.WITH CORTI	6 (0.4%)	5 (0.3%)	11 (0.4%)
BETAMETHASONE SODIUM PHOSPHATE	7 (0.5%)	4 (0.3%)	11 (0.4%)

Table 14.1.1 / 38: Number of subjects who took at least one concomitant medication (FAS)

Substance	LCS12 N=1432 (100%)	LCS16 N=1452 (100%)	Total N=2884 (100%)
BISMUTH SUBSALICYLATE	3 (0.2%)	8 (0.6%)	11 (0.4%)
BUPIVACAINE HYDROCHLORIDE	6 (0.4%)	5 (0.3%)	11 (0.4%)
BUTALBITAL	6 (0.4%)	5 (0.3%)	11 (0.4%)
BUTOCONAZOLE NITRATE	6 (0.4%)	5 (0.3%)	11 (0.4%)
CYCLIZINE HYDROCHLORIDE	4 (0.3%)	7 (0.5%)	11 (0.4%)
DOXYCYCLINE HYCLATE	4 (0.3%)	7 (0.5%)	11 (0.4%)
DRUGS USED IN NICOTINE DEPENDENCE	9 (0.6%)	2 (0.1%)	11 (0.4%)
FLUTICASONE FUROATE	5 (0.3%)	6 (0.4%)	11 (0.4%)
KETOCONAZOLE	4 (0.3%)	7 (0.5%)	11 (0.4%)
LISINOPRIL	6 (0.4%)	5 (0.3%)	11 (0.4%)
METOCLOPRAMIDE HYDROCHLORIDE	8 (0.6%)	3 (0.2%)	11 (0.4%)
MOXIFLOXACIN HYDROCHLORIDE	6 (0.4%)	5 (0.3%)	11 (0.4%)
RANITIDINE	7 (0.5%)	4 (0.3%)	11 (0.4%)
SIBUTRAMINE HYDROCHLORIDE	8 (0.6%)	3 (0.2%)	11 (0.4%)
AMPICILLIN	6 (0.4%)	4 (0.3%)	10 (0.3%)
CALCIUM CHLORIDE DIHYDRATE	5 (0.3%)	5 (0.3%)	10 (0.3%)
ENOXAPARIN SODIUM	1 (<0.1%)	9 (0.6%)	10 (0.3%)
IMIQUIMOD	4 (0.3%)	6 (0.4%)	10 (0.3%)
INFLUENZA VACCINE INACTIVATED	4 (0.3%)	6 (0.4%)	10 (0.3%)
LACTOBACILLUS ACIDOPHILUS	4 (0.3%)	6 (0.4%)	10 (0.3%)
MELATONIN	6 (0.4%)	4 (0.3%)	10 (0.3%)
MESALAZINE	7 (0.5%)	3 (0.2%)	10 (0.3%)
METAMIZOLE	4 (0.3%)	6 (0.4%)	10 (0.3%)
MIDAZOLAM	4 (0.3%)	6 (0.4%)	10 (0.3%)
OXYCODONE	6 (0.4%)	4 (0.3%)	10 (0.3%)
PAROXETINE	7 (0.5%)	3 (0.2%)	10 (0.3%)
ROXITHROMYCIN	5 (0.3%)	5 (0.3%)	10 (0.3%)
SODIUM LACTATE	5 (0.3%)	5 (0.3%)	10 (0.3%)
TRIAMTERENE	4 (0.3%)	6 (0.4%)	10 (0.3%)
ZOLMITRIPTAN	6 (0.4%)	4 (0.3%)	10 (0.3%)
ATROPINE SULFATE	3 (0.2%)	6 (0.4%)	9 (0.3%)
BUSPIRONE HYDROCHLORIDE	5 (0.3%)	4 (0.3%)	9 (0.3%)
CARISOPRODOL	6 (0.4%)	3 (0.2%)	9 (0.3%)
CLONIXIN	6 (0.4%)	3 (0.2%)	9 (0.3%)
DOCOSAHEXANOIC ACID	5 (0.3%)	4 (0.3%)	9 (0.3%)
EICOSAPENTAENOIC ACID	5 (0.3%)	4 (0.3%)	9 (0.3%)
FEXOFENADINE HYDROCHLORIDE	6 (0.4%)	3 (0.2%)	9 (0.3%)
MAGNESIUM HYDROXIDE	2 (0.1%)	7 (0.5%)	9 (0.3%)
MEFLOQUINE	5 (0.3%)	4 (0.3%)	9 (0.3%)
METAXALONE	2 (0.1%)	7 (0.5%)	9 (0.3%)
POTASSIUM	4 (0.3%)	5 (0.3%)	9 (0.3%)
RIZATRIPTAN BENZOATE	5 (0.3%)	4 (0.3%)	9 (0.3%)
ROCURONIUM BROMIDE	2 (0.1%)	7 (0.5%)	9 (0.3%)
SALICYLAMIDE	4 (0.3%)	5 (0.3%)	9 (0.3%)
SIBUTRAMINE	6 (0.4%)	3 (0.2%)	9 (0.3%)

Table 14.1.1 / 38: Number of subjects who took at least one concomitant medication (FAS)

Substance	LCS12 N=1432 (100%)	LCS16 N=1452 (100%)	Total N=2884 (100%)
SODIUM BICARBONATE	3 (0.2%)	6 (0.4%)	9 (0.3%)
SPIRONOLACTONE	5 (0.3%)	4 (0.3%)	9 (0.3%)
TEMAZEPAM	3 (0.2%)	6 (0.4%)	9 (0.3%)
AMINOPHENAZONE	2 (0.1%)	6 (0.4%)	8 (0.3%)
DESVENLAFAXINE SUCCINATE	2 (0.1%)	6 (0.4%)	8 (0.3%)
DIET FORMULATIONS FOR TREATMENT OF OBESITY	5 (0.3%)	3 (0.2%)	8 (0.3%)
DIPHENHYDRAMINE	4 (0.3%)	4 (0.3%)	8 (0.3%)
ESTRIOL	2 (0.1%)	6 (0.4%)	8 (0.3%)
GLUCOSAMINE	2 (0.1%)	6 (0.4%)	8 (0.3%)
GLYCOPYRRONIUM BROMIDE	5 (0.3%)	3 (0.2%)	8 (0.3%)
GUAREA GUIDONIA	4 (0.3%)	4 (0.3%)	8 (0.3%)
INFLUENZA VIRUS VACCINE POLYVALENT	3 (0.2%)	5 (0.3%)	8 (0.3%)
LACTULOSE	3 (0.2%)	5 (0.3%)	8 (0.3%)
MECLOZINE	3 (0.2%)	5 (0.3%)	8 (0.3%)
NICOTINE	1 (<0.1%)	7 (0.5%)	8 (0.3%)
NORELGESTROMIN	4 (0.3%)	4 (0.3%)	8 (0.3%)
PROMETHAZINE HYDROCHLORIDE	5 (0.3%)	3 (0.2%)	8 (0.3%)
RABEPRAZOLE SODIUM	5 (0.3%)	3 (0.2%)	8 (0.3%)
SIMETICONE	3 (0.2%)	5 (0.3%)	8 (0.3%)
SUXAMETHONIUM CHLORIDE	7 (0.5%)	1 (<0.1%)	8 (0.3%)
VENLAFAXINE HYDROCHLORIDE	2 (0.1%)	6 (0.4%)	8 (0.3%)
XYLOMETAZOLINE	5 (0.3%)	3 (0.2%)	8 (0.3%)
ZINC	3 (0.2%)	5 (0.3%)	8 (0.3%)
AMITRIPTYLINE	5 (0.3%)	2 (0.1%)	7 (0.2%)
ARIPIPRAZOLE	3 (0.2%)	4 (0.3%)	7 (0.2%)
ATORVASTATIN CALCIUM	4 (0.3%)	3 (0.2%)	7 (0.2%)
CEFADROXIL	2 (0.1%)	5 (0.3%)	7 (0.2%)
CEFPROZIL	4 (0.3%)	3 (0.2%)	7 (0.2%)
CEFUROXIME SODIUM	5 (0.3%)	2 (0.1%)	7 (0.2%)
CELECOXIB	2 (0.1%)	5 (0.3%)	7 (0.2%)
CLOBETASOL PROPIONATE	1 (<0.1%)	6 (0.4%)	7 (0.2%)
CORTISONE	4 (0.3%)	3 (0.2%)	7 (0.2%)
GABAPENTIN	3 (0.2%)	4 (0.3%)	7 (0.2%)
GLUCOSE	5 (0.3%)	2 (0.1%)	7 (0.2%)
GRAMICIDIN	4 (0.3%)	3 (0.2%)	7 (0.2%)
HYDROCORTISONE ACETATE	4 (0.3%)	3 (0.2%)	7 (0.2%)
HYOSCYAMINE SULFATE	1 (<0.1%)	6 (0.4%)	7 (0.2%)
ITRACONAZOLE	1 (<0.1%)	6 (0.4%)	7 (0.2%)
MEPIVACAINE HYDROCHLORIDE	2 (0.1%)	5 (0.3%)	7 (0.2%)
METHYLPHENIDATE HYDROCHLORIDE	3 (0.2%)	4 (0.3%)	7 (0.2%)
NIMESULIDE	2 (0.1%)	5 (0.3%)	7 (0.2%)
OLANZAPINE	4 (0.3%)	3 (0.2%)	7 (0.2%)
OXYMETAZOLINE HYDROCHLORIDE	5 (0.3%)	2 (0.1%)	7 (0.2%)
PHENIRAMINE MALEATE	3 (0.2%)	4 (0.3%)	7 (0.2%)
PREGABALIN	4 (0.3%)	3 (0.2%)	7 (0.2%)

Table 14.1.1 / 38: Number of subjects who took at least one concomitant medication (FAS)

Substance	LCS12 N=1432 (100%)	LCS16 N=1452 (100%)	Total N=2884 (100%)
SALICYLIC ACID	5 (0.3%)	2 (0.1%)	7 (0.2%)
TOBRAMYCIN	5 (0.3%)	2 (0.1%)	7 (0.2%)
TOLFENAMIC ACID	1 (<0.1%)	6 (0.4%)	7 (0.2%)
YELLOW FEVER VACCINE	2 (0.1%)	5 (0.3%)	7 (0.2%)
ANAESTHETICS, LOCAL	3 (0.2%)	3 (0.2%)	6 (0.2%)
ANTIFUNGALS FOR TOPICAL USE	2 (0.1%)	4 (0.3%)	6 (0.2%)
BETAMETHASONE DIPROPIONATE	2 (0.1%)	4 (0.3%)	6 (0.2%)
BISOPROLOL	3 (0.2%)	3 (0.2%)	6 (0.2%)
CARBAMAZEPINE	2 (0.1%)	4 (0.3%)	6 (0.2%)
CHLORPHENAMINE	4 (0.3%)	2 (0.1%)	6 (0.2%)
CYPROTERONE ACETATE	2 (0.1%)	4 (0.3%)	6 (0.2%)
DICHLORALPHENAZONE	2 (0.1%)	4 (0.3%)	6 (0.2%)
DIPHTHERIA AND TETANUS VACCINES	3 (0.2%)	3 (0.2%)	6 (0.2%)
ECONAZOLE NITRATE	4 (0.3%)	2 (0.1%)	6 (0.2%)
FOSFOMYCIN TROMETAMOL	3 (0.2%)	3 (0.2%)	6 (0.2%)
GENTAMICIN	5 (0.3%)	1 (<0.1%)	6 (0.2%)
HYDROCORTISONE BUTYRATE	3 (0.2%)	3 (0.2%)	6 (0.2%)
MEBEVERINE	5 (0.3%)	1 (<0.1%)	6 (0.2%)
METHOTREXATE	2 (0.1%)	4 (0.3%)	6 (0.2%)
METOPROLOL SUCCINATE	3 (0.2%)	3 (0.2%)	6 (0.2%)
NATAMYCIN	3 (0.2%)	3 (0.2%)	6 (0.2%)
PAMABROM	2 (0.1%)	4 (0.3%)	6 (0.2%)
PHENAZOPYRIDINE	4 (0.3%)	2 (0.1%)	6 (0.2%)
PIMECROLIMUS	3 (0.2%)	3 (0.2%)	6 (0.2%)
PIROXICAM	4 (0.3%)	2 (0.1%)	6 (0.2%)
PYRVINIUM EMBONATE	4 (0.3%)	2 (0.1%)	6 (0.2%)
SALMETEROL	1 (<0.1%)	5 (0.3%)	6 (0.2%)
SUCRALFATE	2 (0.1%)	4 (0.3%)	6 (0.2%)
SULFADIAZINE	4 (0.3%)	2 (0.1%)	6 (0.2%)
TRAZODONE	2 (0.1%)	4 (0.3%)	6 (0.2%)
TYPHOID VACCINE	2 (0.1%)	4 (0.3%)	6 (0.2%)
ZOLPIDEM	2 (0.1%)	4 (0.3%)	6 (0.2%)
ACETYLCYSTEINE	2 (0.1%)	3 (0.2%)	5 (0.2%)
ALMOTRIPTAN MALATE	3 (0.2%)	2 (0.1%)	5 (0.2%)
ALUMINIUM HYDROXIDE	0	5 (0.3%)	5 (0.2%)
AMLODIPINE	3 (0.2%)	2 (0.1%)	5 (0.2%)
ANTI-ACNE PREPARATIONS	3 (0.2%)	2 (0.1%)	5 (0.2%)
ASTEMIZOLE	5 (0.3%)	0	5 (0.2%)
ATENOLOL	1 (<0.1%)	4 (0.3%)	5 (0.2%)
BACITRACIN	1 (<0.1%)	4 (0.3%)	5 (0.2%)
CHOLERA VACCINE	3 (0.2%)	2 (0.1%)	5 (0.2%)
CHORIONIC GONADOTROPHIN	4 (0.3%)	1 (<0.1%)	5 (0.2%)
CINCHOCAINE HYDROCHLORIDE	4 (0.3%)	1 (<0.1%)	5 (0.2%)
DEXTROPROPOXYPHENE	2 (0.1%)	3 (0.2%)	5 (0.2%)
DIOSMIN	2 (0.1%)	3 (0.2%)	5 (0.2%)

Table 14.1.1 / 38: Number of subjects who took at least one concomitant medication (FAS)

Substance	LCS12 N=1432 (100%)	LCS16 N=1452 (100%)	Total N=2884 (100%)
DIPHENHYDRAMINE, COMBINATIONS	2 (0.1%)	3 (0.2%)	5 (0.2%)
EPHEDRINE	4 (0.3%)	1 (<0.1%)	5 (0.2%)
ERGOT ALKALOIDS	2 (0.1%)	3 (0.2%)	5 (0.2%)
ESTRADIOL	3 (0.2%)	2 (0.1%)	5 (0.2%)
ESZOPICLONE	2 (0.1%)	3 (0.2%)	5 (0.2%)
FERROUS GLYCINE SULFATE	3 (0.2%)	2 (0.1%)	5 (0.2%)
HERBAL NOS	1 (<0.1%)	4 (0.3%)	5 (0.2%)
HESPERIDIN	2 (0.1%)	3 (0.2%)	5 (0.2%)
HYDROXYZINE HYDROCHLORIDE	4 (0.3%)	1 (<0.1%)	5 (0.2%)
ISOMETHEPTENE	2 (0.1%)	3 (0.2%)	5 (0.2%)
LACTIC ACID	1 (<0.1%)	4 (0.3%)	5 (0.2%)
LACTOBACILLUS ACIDOPHILUS, LYOPHILIZED	2 (0.1%)	3 (0.2%)	5 (0.2%)
MEDROXYPROGESTERONE ACETATE	2 (0.1%)	3 (0.2%)	5 (0.2%)
NOSCAPINE	5 (0.3%)	0	5 (0.2%)
NOSCAPINE HYDROCHLORIDE	5 (0.3%)	0	5 (0.2%)
OTHER ANTISPASMODICS AND ANTICHOLINERGICS IN	3 (0.2%)	2 (0.1%)	5 (0.2%)
PIPERACILLIN SODIUM	3 (0.2%)	2 (0.1%)	5 (0.2%)
POLYCARBOPHIL CALCIUM	1 (<0.1%)	4 (0.3%)	5 (0.2%)
POLYGALA SENEGA EXTRACT	1 (<0.1%)	4 (0.3%)	5 (0.2%)
PREDNISOLONE ACETATE	1 (<0.1%)	4 (0.3%)	5 (0.2%)
PROGESTERONE	2 (0.1%)	3 (0.2%)	5 (0.2%)
PSYLLIUM HYDROPHILIC MUCILLOID	2 (0.1%)	3 (0.2%)	5 (0.2%)
REMIFENTANIL HYDROCHLORIDE	4 (0.3%)	1 (<0.1%)	5 (0.2%)
TACROLIMUS	1 (<0.1%)	4 (0.3%)	5 (0.2%)
TAZOBACTAM SODIUM	3 (0.2%)	2 (0.1%)	5 (0.2%)
TETANUS VACCINE	1 (<0.1%)	4 (0.3%)	5 (0.2%)
VITAMIN B	2 (0.1%)	3 (0.2%)	5 (0.2%)
VITAMIN-B-KOMPLEX STANDARD	2 (0.1%)	3 (0.2%)	5 (0.2%)
AMBROXOL HYDROCHLORIDE	4 (0.3%)	0	4 (0.1%)
ANTIHEMORRHOIDALS FOR TOPICAL USE	4 (0.3%)	0	4 (0.1%)
ANTIMIGRAINE PREPARATIONS	2 (0.1%)	2 (0.1%)	4 (0.1%)
BENZONATATE	3 (0.2%)	1 (<0.1%)	4 (0.1%)
BETAHISTINE HYDROCHLORIDE	3 (0.2%)	1 (<0.1%)	4 (0.1%)
BISACODYL	3 (0.2%)	1 (<0.1%)	4 (0.1%)
CALCIUM PANTOTHENATE	0	4 (0.3%)	4 (0.1%)
CALCIUM PHOSPHATE	2 (0.1%)	2 (0.1%)	4 (0.1%)
CAMPHOR	2 (0.1%)	2 (0.1%)	4 (0.1%)
CARBOMER	3 (0.2%)	1 (<0.1%)	4 (0.1%)
CEFAZOLIN	1 (<0.1%)	3 (0.2%)	4 (0.1%)
CEFIXIME	3 (0.2%)	1 (<0.1%)	4 (0.1%)
CHLORDIAZEPOXIDE	2 (0.1%)	2 (0.1%)	4 (0.1%)
CHLORHEXIDINE GLUCONATE	2 (0.1%)	2 (0.1%)	4 (0.1%)
CHONDROITIN	0	4 (0.3%)	4 (0.1%)
DALTEPARIN SODIUM	2 (0.1%)	2 (0.1%)	4 (0.1%)
DESONIDE	2 (0.1%)	2 (0.1%)	4 (0.1%)

Table 14.1.1 / 38: Number of subjects who took at least one concomitant medication (FAS)

Substance	LCS12 N=1432 (100%)	LCS16 N=1452 (100%)	Total N=2884 (100%)
DEXAMFETAMINE	2 (0.1%)	2 (0.1%)	4 (0.1%)
DICLOFENAC DIETHYLAMINE	0	4 (0.3%)	4 (0.1%)
DICYCLOVERINE HYDROCHLORIDE	2 (0.1%)	2 (0.1%)	4 (0.1%)
DOMPERIDONE	3 (0.2%)	1 (<0.1%)	4 (0.1%)
DROTAVERINE HYDROCHLORIDE	2 (0.1%)	2 (0.1%)	4 (0.1%)
FENTICONAZOLE NITRATE	1 (<0.1%)	3 (0.2%)	4 (0.1%)
FLUCLOXACILLIN SODIUM	1 (<0.1%)	3 (0.2%)	4 (0.1%)
FLURBIPROFEN	1 (<0.1%)	3 (0.2%)	4 (0.1%)
GENTAMICIN SULFATE	1 (<0.1%)	3 (0.2%)	4 (0.1%)
GYNECOLOGICAL ANTIINFECTIVES AND ANTISEPTICS	1 (<0.1%)	3 (0.2%)	4 (0.1%)
HOMEOPATIC PREPARATION	2 (0.1%)	2 (0.1%)	4 (0.1%)
HYDROQUINONE	3 (0.2%)	1 (<0.1%)	4 (0.1%)
INSULIN LISPRO	2 (0.1%)	2 (0.1%)	4 (0.1%)
KETAMINE	4 (0.3%)	0	4 (0.1%)
KETOBEMIDONE HYDROCHLORIDE	4 (0.3%)	0	4 (0.1%)
LINUM USITATISSIMUM SEED OIL	3 (0.2%)	1 (<0.1%)	4 (0.1%)
LOPERAMIDE	3 (0.2%)	1 (<0.1%)	4 (0.1%)
MACROCYSTIS PYRIFERA	2 (0.1%)	2 (0.1%)	4 (0.1%)
MAGNESIUM CARBONATE	1 (<0.1%)	3 (0.2%)	4 (0.1%)
MENTHOL	2 (0.1%)	2 (0.1%)	4 (0.1%)
METHENAMINE	1 (<0.1%)	3 (0.2%)	4 (0.1%)
NALBUPHINE	3 (0.2%)	1 (<0.1%)	4 (0.1%)
NORTRIPTYLINE	1 (<0.1%)	3 (0.2%)	4 (0.1%)
OENOTHERA BIENNIS OIL	1 (<0.1%)	3 (0.2%)	4 (0.1%)
ORLISTAT	3 (0.2%)	1 (<0.1%)	4 (0.1%)
PHENOBARBITAL	2 (0.1%)	2 (0.1%)	4 (0.1%)
PREDNISOLONE CAPROATE	3 (0.2%)	1 (<0.1%)	4 (0.1%)
PROCHLORPERAZINE EDISYLATE	2 (0.1%)	2 (0.1%)	4 (0.1%)
PROPYLTHIOURACIL	2 (0.1%)	2 (0.1%)	4 (0.1%)
PROPYPHENAZONE	2 (0.1%)	2 (0.1%)	4 (0.1%)
RAMIPRIL	1 (<0.1%)	3 (0.2%)	4 (0.1%)
RESORCINOL	3 (0.2%)	1 (<0.1%)	4 (0.1%)
SENNOSIDE A+B	0	4 (0.3%)	4 (0.1%)
SERRAPEPTASE	2 (0.1%)	2 (0.1%)	4 (0.1%)
SEVOFLURANE	3 (0.2%)	1 (<0.1%)	4 (0.1%)
SILVER NITRATE	2 (0.1%)	2 (0.1%)	4 (0.1%)
SODIUM PHOSPHATE DIBASIC	1 (<0.1%)	3 (0.2%)	4 (0.1%)
SULFASALAZINE	2 (0.1%)	2 (0.1%)	4 (0.1%)
SUMATRIPTAN SUCCINATE	1 (<0.1%)	3 (0.2%)	4 (0.1%)
TETANUS TOXOID	1 (<0.1%)	3 (0.2%)	4 (0.1%)
TOLPERISONE HYDROCHLORIDE	3 (0.2%)	1 (<0.1%)	4 (0.1%)
TRICHLOROACETIC ACID	4 (0.3%)	0	4 (0.1%)
TRIMEBUTINE	3 (0.2%)	1 (<0.1%)	4 (0.1%)
VACCINIUM MACROCARPON	0	4 (0.3%)	4 (0.1%)
VERAPAMIL	3 (0.2%)	1 (<0.1%)	4 (0.1%)

Table 14.1.1 / 38: Number of subjects who took at least one concomitant medication (FAS)

Substance	LCS12 N=1432 (100%)	LCS16 N=1452 (100%)	Total N=2884 (100%)
VITAMIN B12 NOS	0	4 (0.3%)	4 (0.1%)
ACECLOFENAC	2 (0.1%)	1 (<0.1%)	3 (0.1%)
ALFENTANIL HYDROCHLORIDE	2 (0.1%)	1 (<0.1%)	3 (0.1%)
AMBROXOL	3 (0.2%)	0	3 (0.1%)
AMILORIDE HYDROCHLORIDE	2 (0.1%)	1 (<0.1%)	3 (0.1%)
ANALGESICS	1 (<0.1%)	2 (0.1%)	3 (0.1%)
ANTIFUNGALS	2 (0.1%)	1 (<0.1%)	3 (0.1%)
ANTIHISTAMINES	1 (<0.1%)	2 (0.1%)	3 (0.1%)
ANTIINFLAM. AGENTS AND ANTIINFECT. OPHTHALMIC	2 (0.1%)	1 (<0.1%)	3 (0.1%)
ARTICAINE HYDROCHLORIDE	2 (0.1%)	1 (<0.1%)	3 (0.1%)
BACITRACIN ZINC	1 (<0.1%)	2 (0.1%)	3 (0.1%)
BENZATHINE BENZYLPENICILLIN	3 (0.2%)	0	3 (0.1%)
BISOPROLOL FUMARATE	3 (0.2%)	0	3 (0.1%)
BORIC ACID	1 (<0.1%)	2 (0.1%)	3 (0.1%)
BOTULINUM TOXIN TYPE A	0	3 (0.2%)	3 (0.1%)
BUSPIRONE	2 (0.1%)	1 (<0.1%)	3 (0.1%)
CALCIPOTRIOL	1 (<0.1%)	2 (0.1%)	3 (0.1%)
CALCIUM CITRATE	1 (<0.1%)	2 (0.1%)	3 (0.1%)
CALCIUM SODIUM LACTATE	1 (<0.1%)	2 (0.1%)	3 (0.1%)
CANDESARTAN CILEXETIL	1 (<0.1%)	2 (0.1%)	3 (0.1%)
CEFDINIR	2 (0.1%)	1 (<0.1%)	3 (0.1%)
CHLORDIAZEPOXIDE HYDROCHLORIDE	1 (<0.1%)	2 (0.1%)	3 (0.1%)
CHLORMEZANONE	2 (0.1%)	1 (<0.1%)	3 (0.1%)
CHROMIUM	2 (0.1%)	1 (<0.1%)	3 (0.1%)
CINEOLE	1 (<0.1%)	2 (0.1%)	3 (0.1%)
CORTICOSTEROIDS, DERMATOLOGICAL PREPARATIONS	2 (0.1%)	1 (<0.1%)	3 (0.1%)
CYAMOPSIS TETRAGONOLOBA GUM	1 (<0.1%)	2 (0.1%)	3 (0.1%)
DESFLURANE	1 (<0.1%)	2 (0.1%)	3 (0.1%)
DEXIBUPROFEN	2 (0.1%)	1 (<0.1%)	3 (0.1%)
DEXTROMETHORPHAN	1 (<0.1%)	2 (0.1%)	3 (0.1%)
DIMETHYL-3,3-DIPHENYL-1-METHYLALLYLAMINE HCL	3 (0.2%)	0	3 (0.1%)
DIPHTHERIA TOXOID	0	3 (0.2%)	3 (0.1%)
DRIMIA MARITIMA SYRUP	1 (<0.1%)	2 (0.1%)	3 (0.1%)
DRUGS FOR FUNCTIONAL GASTROINTEST. DISORDERS	1 (<0.1%)	2 (0.1%)	3 (0.1%)
ENALAPRIL MALEATE	2 (0.1%)	1 (<0.1%)	3 (0.1%)
EPINEPHRINE BITARTRATE	2 (0.1%)	1 (<0.1%)	3 (0.1%)
ERDOSTEINE	1 (<0.1%)	2 (0.1%)	3 (0.1%)
EUPHORBIA HIRTA	1 (<0.1%)	2 (0.1%)	3 (0.1%)
FLUCLOXACILLIN	2 (0.1%)	1 (<0.1%)	3 (0.1%)
FLUOCINOLONE ACETONIDE	1 (<0.1%)	2 (0.1%)	3 (0.1%)
FRANGULIN	1 (<0.1%)	2 (0.1%)	3 (0.1%)
FROVATRIPTAN SUCCINATE MONOHYDRATE	0	3 (0.2%)	3 (0.1%)
FUROSEMIDE	0	3 (0.2%)	3 (0.1%)
GLUCOCORTICOIDS	1 (<0.1%)	2 (0.1%)	3 (0.1%)
GLUCOSAMINE SULFATE	0	3 (0.2%)	3 (0.1%)

Table 14.1.1 / 38: Number of subjects who took at least one concomitant medication (FAS)

Substance	LCS12 N=1432 (100%)	LCS16 N=1452 (100%)	Total N=2884 (100%)
GRANISETRON	2 (0.1%)	1 (<0.1%)	3 (0.1%)
GUAREA GUIDONIA LIQUID EXTRACT	1 (<0.1%)	2 (0.1%)	3 (0.1%)
HEPARIN	1 (<0.1%)	2 (0.1%)	3 (0.1%)
HYOSCINE	2 (0.1%)	1 (<0.1%)	3 (0.1%)
HYPNOTICS AND SEDATIVES	2 (0.1%)	1 (<0.1%)	3 (0.1%)
INDOMETACIN	2 (0.1%)	1 (<0.1%)	3 (0.1%)
IPRATROPIUM BROMIDE	1 (<0.1%)	2 (0.1%)	3 (0.1%)
ISOCONAZOLE NITRATE	2 (0.1%)	1 (<0.1%)	3 (0.1%)
LACTUCA VIROSA EXTRACT	1 (<0.1%)	2 (0.1%)	3 (0.1%)
LEVOCABASTINE HYDROCHLORIDE	2 (0.1%)	1 (<0.1%)	3 (0.1%)
LEVOCETIRIZINE	0	3 (0.2%)	3 (0.1%)
LIOTHYRONINE SODIUM	1 (<0.1%)	2 (0.1%)	3 (0.1%)
LOSARTAN	2 (0.1%)	1 (<0.1%)	3 (0.1%)
LUBIPROSTONE	1 (<0.1%)	2 (0.1%)	3 (0.1%)
MAGNESIUM OXIDE	1 (<0.1%)	2 (0.1%)	3 (0.1%)
MEPREDNISONE	1 (<0.1%)	2 (0.1%)	3 (0.1%)
METHYLPREDNISOLONE ACETATE	2 (0.1%)	1 (<0.1%)	3 (0.1%)
METHYLPREDNISOLONE SODIUM SUCCINATE	2 (0.1%)	1 (<0.1%)	3 (0.1%)
METOPROLOL TARTRATE	1 (<0.1%)	2 (0.1%)	3 (0.1%)
NEOMYCIN	2 (0.1%)	1 (<0.1%)	3 (0.1%)
NEOSTIGMINE METILSULFATE	1 (<0.1%)	2 (0.1%)	3 (0.1%)
OTHER COLD COMBINATION PREPARATIONS	3 (0.2%)	0	3 (0.1%)
OXITRIPTAN	2 (0.1%)	1 (<0.1%)	3 (0.1%)
PAPAVERINE HYDROCHLORIDE	2 (0.1%)	1 (<0.1%)	3 (0.1%)
PARAFFIN	0	3 (0.2%)	3 (0.1%)
PHENOL	2 (0.1%)	1 (<0.1%)	3 (0.1%)
PHENOXYMETHYLPENICILLIN	1 (<0.1%)	2 (0.1%)	3 (0.1%)
PHENTERMINE HYDROCHLORIDE	1 (<0.1%)	2 (0.1%)	3 (0.1%)
POLIOMYELITIS VACCINE	1 (<0.1%)	2 (0.1%)	3 (0.1%)
POLYSORBATE 20	0	3 (0.2%)	3 (0.1%)
PRAMOCAINE HYDROCHLORIDE	0	3 (0.2%)	3 (0.1%)
PROCAINE HYDROCHLORIDE	1 (<0.1%)	2 (0.1%)	3 (0.1%)
PROPIOMAZINE MALEATE	3 (0.2%)	0	3 (0.1%)
PYRIDOXINE	2 (0.1%)	1 (<0.1%)	3 (0.1%)
RABIES VACCINE	1 (<0.1%)	2 (0.1%)	3 (0.1%)
RISPERIDONE	0	3 (0.2%)	3 (0.1%)
ROCURONIUM	3 (0.2%)	0	3 (0.1%)
ROPIVACAINE HYDROCHLORIDE	1 (<0.1%)	2 (0.1%)	3 (0.1%)
SACCHAROMYCES BOULARDII	3 (0.2%)	0	3 (0.1%)
SECNIDAZOLE	2 (0.1%)	1 (<0.1%)	3 (0.1%)
SERTACONAZOLE NITRATE	2 (0.1%)	1 (<0.1%)	3 (0.1%)
SERTRALINE HYDROCHLORIDE	2 (0.1%)	1 (<0.1%)	3 (0.1%)
SIMVASTATIN	1 (<0.1%)	2 (0.1%)	3 (0.1%)
SODIUM CITRATE	1 (<0.1%)	2 (0.1%)	3 (0.1%)
SODIUM PHOSPHATE MONOBASIC	1 (<0.1%)	2 (0.1%)	3 (0.1%)

Table 14.1.1 / 38: Number of subjects who took at least one concomitant medication (FAS)

Substance	LCS12 N=1432 (100%)	LCS16 N=1452 (100%)	Total N=2884 (100%)
SUFENTANIL	1 (<0.1%)	2 (0.1%)	3 (0.1%)
SULBACTAM	2 (0.1%)	1 (<0.1%)	3 (0.1%)
SULFADIAZINE SILVER	0	3 (0.2%)	3 (0.1%)
TESTOSTERONE	2 (0.1%)	1 (<0.1%)	3 (0.1%)
THIAMINE	2 (0.1%)	1 (<0.1%)	3 (0.1%)
THIOCTIC ACID	2 (0.1%)	1 (<0.1%)	3 (0.1%)
TRAZODONE HYDROCHLORIDE	1 (<0.1%)	2 (0.1%)	3 (0.1%)
TRIAMCINOLONE	2 (0.1%)	1 (<0.1%)	3 (0.1%)
TRIMEBUTINE MALEATE	2 (0.1%)	1 (<0.1%)	3 (0.1%)
VACCINES	2 (0.1%)	1 (<0.1%)	3 (0.1%)
VALPROATE SEMISODIUM	1 (<0.1%)	2 (0.1%)	3 (0.1%)
VALPROATE SODIUM	3 (0.2%)	0	3 (0.1%)
VITAMINS WITH MINERALS	0	3 (0.2%)	3 (0.1%)
ADIPHENINE	2 (0.1%)	0	2 (<0.1%)
ALIMEMAZINE TARTRATE	2 (0.1%)	0	2 (<0.1%)
ALIMENTARY TRACT AND METABOLISM	0	2 (0.1%)	2 (<0.1%)
ALLIUM SATIVUM	2 (0.1%)	0	2 (<0.1%)
ALOE VERA	2 (0.1%)	0	2 (<0.1%)
AMCINONIDE	2 (0.1%)	0	2 (<0.1%)
AMFEPRAMONE HYDROCHLORIDE	1 (<0.1%)	1 (<0.1%)	2 (<0.1%)
AMINOSALICYLIC ACID	2 (0.1%)	0	2 (<0.1%)
AMMONIUM CHLORIDE	2 (0.1%)	0	2 (<0.1%)
ANTACIDS	2 (0.1%)	0	2 (<0.1%)
ANTIBIOTICS FOR TOPICAL USE	0	2 (0.1%)	2 (<0.1%)
ARTICAINE	1 (<0.1%)	1 (<0.1%)	2 (<0.1%)
ATOMOXETINE HYDROCHLORIDE	1 (<0.1%)	1 (<0.1%)	2 (<0.1%)
ATROPINE	2 (0.1%)	0	2 (<0.1%)
AZATHIOPRINE	1 (<0.1%)	1 (<0.1%)	2 (<0.1%)
AZELASTINE	1 (<0.1%)	1 (<0.1%)	2 (<0.1%)
BENZALKONIUM CHLORIDE	0	2 (0.1%)	2 (<0.1%)
BENZOIC ACID	0	2 (0.1%)	2 (<0.1%)
BETACAROTENE	0	2 (0.1%)	2 (<0.1%)
BROMELAINS	1 (<0.1%)	1 (<0.1%)	2 (<0.1%)
BROMPHENIRAMINE MALEATE	1 (<0.1%)	1 (<0.1%)	2 (<0.1%)
CALCIUM AMINO ACID CHELATE	0	2 (0.1%)	2 (<0.1%)
CALCIUM GLUCONATE	2 (0.1%)	0	2 (<0.1%)
CAMELLIA SINENSIS	2 (0.1%)	0	2 (<0.1%)
CAPTOPRIL	0	2 (0.1%)	2 (<0.1%)
CARBASALATE CALCIUM	1 (<0.1%)	1 (<0.1%)	2 (<0.1%)
CEFATRIZINE PROPYLENGLYCOLATE SULFATE	1 (<0.1%)	1 (<0.1%)	2 (<0.1%)
CEFOXITIN	0	2 (0.1%)	2 (<0.1%)
CEFUROXIME AXETIL	0	2 (0.1%)	2 (<0.1%)
CETYLPYRIDINIUM CHLORIDE	1 (<0.1%)	1 (<0.1%)	2 (<0.1%)
CHARCOAL, ACTIVATED	1 (<0.1%)	1 (<0.1%)	2 (<0.1%)
CHLOROPYRAMINE HYDROCHLORIDE	2 (0.1%)	0	2 (<0.1%)

Table 14.1.1 / 38: Number of subjects who took at least one concomitant medication (FAS)

Substance	LCS12<br>N=1432 (100%)	LCS16<br>N=1452 (100%)	Total<br>N=2884 (100%)
CHLOROQUINE PHOSPHATE	1 (<0.1%)	1 (<0.1%)	2 (<0.1%)
CHROMIUM AMINO ACID CHELATE	0	2 (0.1%)	2 (<0.1%)
CICLESONIDE	1 (<0.1%)	1 (<0.1%)	2 (<0.1%)
CITRIC ACID	1 (<0.1%)	1 (<0.1%)	2 (<0.1%)
CLIDINIUM BROMIDE	0	2 (0.1%)	2 (<0.1%)
CLOBETASOL	1 (<0.1%)	1 (<0.1%)	2 (<0.1%)
CLOBETASONE BUTYRATE	0	2 (0.1%)	2 (<0.1%)
CLOFEDANOL	2 (0.1%)	0	2 (<0.1%)
CLONIDINE	1 (<0.1%)	1 (<0.1%)	2 (<0.1%)
COD-LIVER OIL	1 (<0.1%)	1 (<0.1%)	2 (<0.1%)
COLESTYRAMINE	1 (<0.1%)	1 (<0.1%)	2 (<0.1%)
COPPER AMINO ACID CHELATE	0	2 (0.1%)	2 (<0.1%)
COSMETICS	1 (<0.1%)	1 (<0.1%)	2 (<0.1%)
DERMATOLOGICALS	2 (0.1%)	0	2 (<0.1%)
DESOXIMETASONE	1 (<0.1%)	1 (<0.1%)	2 (<0.1%)
DEXCHLORPHENIRAMINE MALEATE	2 (0.1%)	0	2 (<0.1%)
DEXKETOPROFEN TROMETAMOL	0	2 (0.1%)	2 (<0.1%)
DEXTROPROPOXYPHENE HYDROCHLORIDE	0	2 (0.1%)	2 (<0.1%)
DICHLOROBENZYL ALCOHOL	1 (<0.1%)	1 (<0.1%)	2 (<0.1%)
DIFLUCORTOLONE VALERATE	2 (0.1%)	0	2 (<0.1%)
DILTIAZEM	1 (<0.1%)	1 (<0.1%)	2 (<0.1%)
DOCOSANOL	0	2 (0.1%)	2 (<0.1%)
DROPERIDOL	0	2 (0.1%)	2 (<0.1%)
DROPERIDOL LACTATE	1 (<0.1%)	1 (<0.1%)	2 (<0.1%)
ECHINACEA PURPUREA	1 (<0.1%)	1 (<0.1%)	2 (<0.1%)
ELETRIPTAN HYDROBROMIDE	1 (<0.1%)	1 (<0.1%)	2 (<0.1%)
EQUISETUM ARVENSE STEM	0	2 (0.1%)	2 (<0.1%)
ESOMEPRAZOLE	0	2 (0.1%)	2 (<0.1%)
ESTRADIOL CIPIONATE	1 (<0.1%)	1 (<0.1%)	2 (<0.1%)
ETODOLAC	0	2 (0.1%)	2 (<0.1%)
EXENATIDE	2 (0.1%)	0	2 (<0.1%)
FENOVERINE	1 (<0.1%)	1 (<0.1%)	2 (<0.1%)
FENTANYL CITRATE	1 (<0.1%)	1 (<0.1%)	2 (<0.1%)
FLUOCINONIDE	1 (<0.1%)	1 (<0.1%)	2 (<0.1%)
FLUPENTIXOL DIHYDROCHLORIDE	1 (<0.1%)	1 (<0.1%)	2 (<0.1%)
FRAMYCETIN SULFATE	2 (0.1%)	0	2 (<0.1%)
GLYCEROL	0	2 (0.1%)	2 (<0.1%)
HALOPERIDOL	0	2 (0.1%)	2 (<0.1%)
HAMAMELIS VIRGINIANA	1 (<0.1%)	1 (<0.1%)	2 (<0.1%)
HEPARIN SODIUM	1 (<0.1%)	1 (<0.1%)	2 (<0.1%)
HERBAL EXTRACT NOS	1 (<0.1%)	1 (<0.1%)	2 (<0.1%)
HOMEOPATICS NOS	0	2 (0.1%)	2 (<0.1%)
HORMONAL CONTRACEPTIVES FOR SYSTEMIC USE	1 (<0.1%)	1 (<0.1%)	2 (<0.1%)
HYDROMORPHONE	1 (<0.1%)	1 (<0.1%)	2 (<0.1%)
HYDROXYCHLOROQUINE PHOSPHATE	0	2 (0.1%)	2 (<0.1%)

Table 14.1.1 / 38: Number of subjects who took at least one concomitant medication (FAS)

Substance	LCS12 N=1432 (100%)	LCS16 N=1452 (100%)	Total N=2884 (100%)
HYDROXYCHLOROQUINE SULFATE	1 (<0.1%)	1 (<0.1%)	2 (<0.1%)
HYOSCINE HYDROBROMIDE	0	2 (0.1%)	2 (<0.1%)
HYPROMELLOSE	0	2 (0.1%)	2 (<0.1%)
I.V. SOLUTIONS	1 (<0.1%)	1 (<0.1%)	2 (<0.1%)
IOHEXOL	2 (0.1%)	0	2 (<0.1%)
IRON IN OTHER COMBINATIONS	0	2 (0.1%)	2 (<0.1%)
ISOCONAZOLE	0	2 (0.1%)	2 (<0.1%)
KETOTIFEN FUMARATE	0	2 (0.1%)	2 (<0.1%)
LAMINARIA DIGITATA POWDER	0	2 (0.1%)	2 (<0.1%)
LEVETIRACETAM	0	2 (0.1%)	2 (<0.1%)
LYNESTRENOL	1 (<0.1%)	1 (<0.1%)	2 (<0.1%)
LYSINE	1 (<0.1%)	1 (<0.1%)	2 (<0.1%)
MANGANESE AMINO ACID CHELATE	0	2 (0.1%)	2 (<0.1%)
MEBENDAZOLE	0	2 (0.1%)	2 (<0.1%)
MECLOFENAMATE SODIUM	1 (<0.1%)	1 (<0.1%)	2 (<0.1%)
MEDAZEPAM	2 (0.1%)	0	2 (<0.1%)
MELALEUCA ALTERNIFOLIA OIL	1 (<0.1%)	1 (<0.1%)	2 (<0.1%)
MENINGOCOCCAL VACCINES	1 (<0.1%)	1 (<0.1%)	2 (<0.1%)
MERCAPTOPURINE	1 (<0.1%)	1 (<0.1%)	2 (<0.1%)
METHYLSULFONYLMETHANE	0	2 (0.1%)	2 (<0.1%)
METHYLTHIONINIUM CHLORIDE	0	2 (0.1%)	2 (<0.1%)
METOPROLOL	1 (<0.1%)	1 (<0.1%)	2 (<0.1%)
MODAFINIL	1 (<0.1%)	1 (<0.1%)	2 (<0.1%)
MOMETASONE	1 (<0.1%)	1 (<0.1%)	2 (<0.1%)
MORPHINE SULFATE	2 (0.1%)	0	2 (<0.1%)
MOXIFLOXACIN	1 (<0.1%)	1 (<0.1%)	2 (<0.1%)
MULTIVITAMINS WITH MINERALS [MULTIVITAMINS WI	2 (0.1%)	0	2 (<0.1%)
MUPIROCIN	0	2 (0.1%)	2 (<0.1%)
NABUMETONE	1 (<0.1%)	1 (<0.1%)	2 (<0.1%)
NADROPARIN CALCIUM	2 (0.1%)	0	2 (<0.1%)
NARATRIPTAN HYDROCHLORIDE	1 (<0.1%)	1 (<0.1%)	2 (<0.1%)
NEBIVOLOL HYDROCHLORIDE	1 (<0.1%)	1 (<0.1%)	2 (<0.1%)
NIFEDIPINE	0	2 (0.1%)	2 (<0.1%)
NIFLUMIC ACID	1 (<0.1%)	1 (<0.1%)	2 (<0.1%)
NIFUROXAZIDE	0	2 (0.1%)	2 (<0.1%)
NORGESTREL	2 (0.1%)	0	2 (<0.1%)
OCTATROPINE METHYLBROMIDE	1 (<0.1%)	1 (<0.1%)	2 (<0.1%)
OLOPATADINE	1 (<0.1%)	1 (<0.1%)	2 (<0.1%)
OPIOIDS	2 (0.1%)	0	2 (<0.1%)
OTHER ANTIPRURITICS	0	2 (0.1%)	2 (<0.1%)
OTHER EMOLLIENTS AND PROTECTIVES	0	2 (0.1%)	2 (<0.1%)
OTHER OPHTHALMOLOGICALS	0	2 (0.1%)	2 (<0.1%)
OTHER RESPIRATORY SYSTEM PRODUCTS	2 (0.1%)	0	2 (<0.1%)
OXCARBAZEPINE	2 (0.1%)	0	2 (<0.1%)
OXYMETAZOLINE	1 (<0.1%)	1 (<0.1%)	2 (<0.1%)

Table 14.1.1 / 38: Number of subjects who took at least one concomitant medication (FAS)

Substance	LCS12 N=1432 (100%)	LCS16 N=1452 (100%)	Total N=2884 (100%)
OXYTETRACYCLINE HYDROCHLORIDE	2 (0.1%)	0	2 (<0.1%)
PARGEVERINE HYDROCHLORIDE	1 (<0.1%)	1 (<0.1%)	2 (<0.1%)
PENCICLOVIR	0	2 (0.1%)	2 (<0.1%)
PETHIDINE	1 (<0.1%)	1 (<0.1%)	2 (<0.1%)
PETROLATUM	0	2 (0.1%)	2 (<0.1%)
PHENYL SALICYLATE	0	2 (0.1%)	2 (<0.1%)
PHENYLEPHRINE	2 (0.1%)	0	2 (<0.1%)
PHENYLPROPANOLAMINE	1 (<0.1%)	1 (<0.1%)	2 (<0.1%)
PHENYLTOLOXAMINE	1 (<0.1%)	1 (<0.1%)	2 (<0.1%)
PIROXICAM BETADEX	1 (<0.1%)	1 (<0.1%)	2 (<0.1%)
PLANTAGO AFRA	2 (0.1%)	0	2 (<0.1%)
PNEUMOCOCCAL VACCINE	1 (<0.1%)	1 (<0.1%)	2 (<0.1%)
POLIOVIRUS VACCINE, INACTIVATED	1 (<0.1%)	1 (<0.1%)	2 (<0.1%)
POLYNOXYLIN	2 (0.1%)	0	2 (<0.1%)
POTASSIUM AMINO ACID CHELATE	0	2 (0.1%)	2 (<0.1%)
POTASSIUM IODIDE	2 (0.1%)	0	2 (<0.1%)
PRAMIPEXOLE DIHYDROCHLORIDE	2 (0.1%)	0	2 (<0.1%)
PRASTERONE	1 (<0.1%)	1 (<0.1%)	2 (<0.1%)
PROMESTRIENE	2 (0.1%)	0	2 (<0.1%)
PROPOXYPHENE	1 (<0.1%)	1 (<0.1%)	2 (<0.1%)
QUILLAJA SAPONARIA BARK EXTRACT	2 (0.1%)	0	2 (<0.1%)
RAMELTEON	1 (<0.1%)	1 (<0.1%)	2 (<0.1%)
SALICYLIC ACID AND DERIVATIVES	0	2 (0.1%)	2 (<0.1%)
SELENIUM AMINO ACID CHELATE	0	2 (0.1%)	2 (<0.1%)
SILYBUM MARIANUM	2 (0.1%)	0	2 (<0.1%)
SODIUM CITRATE ACID	1 (<0.1%)	1 (<0.1%)	2 (<0.1%)
SOLIFENACIN SUCCINATE	0	2 (0.1%)	2 (<0.1%)
TAMSULOSIN HYDROCHLORIDE	1 (<0.1%)	1 (<0.1%)	2 (<0.1%)
TBE VIRUS ANTIGEN	0	2 (0.1%)	2 (<0.1%)
TELITHROMYCIN	1 (<0.1%)	1 (<0.1%)	2 (<0.1%)
TERBINAFINE HYDROCHLORIDE	0	2 (0.1%)	2 (<0.1%)
THIAMINE MONONITRATE	0	2 (0.1%)	2 (<0.1%)
THIETHYLPERAZINE MALEATE	2 (0.1%)	0	2 (<0.1%)
TILACTASE	2 (0.1%)	0	2 (<0.1%)
TIZANIDINE	2 (0.1%)	0	2 (<0.1%)
TOLNAFTATE	0	2 (0.1%)	2 (<0.1%)
VALACICLOVIR	1 (<0.1%)	1 (<0.1%)	2 (<0.1%)
VALERIANA OFFICINALIS	1 (<0.1%)	1 (<0.1%)	2 (<0.1%)
VALSARTAN	2 (0.1%)	0	2 (<0.1%)
VANCOMYCIN	1 (<0.1%)	1 (<0.1%)	2 (<0.1%)
ZINC AMINO ACID CHELATE	0	2 (0.1%)	2 (<0.1%)
ZIPRASIDONE HYDROCHLORIDE	0	2 (0.1%)	2 (<0.1%)
5-ETHYL-6-PHENYLPERHYDRO-1,3-THIAZIN-2,4-DION	0	1 (<0.1%)	1 (<0.1%)
ACEMETACIN	0	1 (<0.1%)	1 (<0.1%)
ACETAZOLAMIDE	0	1 (<0.1%)	1 (<0.1%)

Table 14.1.1 / 38: Number of subjects who took at least one concomitant medication (FAS)

Substance	LCS12 N=1432 (100%)	LCS16 N=1452 (100%)	Total N=2884 (100%)
ACETIC ACID DERIVATIVES AND RELATED SUBST.	0	1 (<0.1%)	1 (<0.1%)
ACETYLSALICYLATE LYSINE	0	1 (<0.1%)	1 (<0.1%)
ADALIMUMAB	1 (<0.1%)	0	1 (<0.1%)
ALBUMIN TANNATE	1 (<0.1%)	0	1 (<0.1%)
ALCLOMETASONE DIPROPIONATE	0	1 (<0.1%)	1 (<0.1%)
ALENDRONATE SODIUM	0	1 (<0.1%)	1 (<0.1%)
ALENDRONIC ACID	0	1 (<0.1%)	1 (<0.1%)
ALFA-HEDERINA	0	1 (<0.1%)	1 (<0.1%)
ALLERGEN EXTRACTS	0	1 (<0.1%)	1 (<0.1%)
ALLERGENS, POLLEN & PLANT EXTRACT	0	1 (<0.1%)	1 (<0.1%)
ALTEPLASE	1 (<0.1%)	0	1 (<0.1%)
ALUMINIUM HYDROXIDE GEL	1 (<0.1%)	0	1 (<0.1%)
AMANTADINE	1 (<0.1%)	0	1 (<0.1%)
AMANTADINE HYDROCHLORIDE	1 (<0.1%)	0	1 (<0.1%)
AMIKACIN	0	1 (<0.1%)	1 (<0.1%)
AMINO ACIDS NOS	0	1 (<0.1%)	1 (<0.1%)
AMYLASE	0	1 (<0.1%)	1 (<0.1%)
AMYLMETACRESOL	1 (<0.1%)	0	1 (<0.1%)
ANAESTHETICS	1 (<0.1%)	0	1 (<0.1%)
ANESTHETICS, LOCAL	0	1 (<0.1%)	1 (<0.1%)
ANETHOLE	0	1 (<0.1%)	1 (<0.1%)
ANTHRAX VACCINE	1 (<0.1%)	0	1 (<0.1%)
ANTIDOTES	1 (<0.1%)	0	1 (<0.1%)
ANTIHISTAMINES FOR SYSTEMIC USE	0	1 (<0.1%)	1 (<0.1%)
ANTIINFLAMMATORY/ANTIRHEUMATIC NON-STEROIDS	0	1 (<0.1%)	1 (<0.1%)
ANTIINFLAMMATORY/ANTIRHEUMATIC PRODUCTS	1 (<0.1%)	0	1 (<0.1%)
ANTIMALARIALS	0	1 (<0.1%)	1 (<0.1%)
ANTIPSORIATICS	1 (<0.1%)	0	1 (<0.1%)
APPETITE STIMULANTS	1 (<0.1%)	0	1 (<0.1%)
ARACHIS HYPOGAEA OIL	0	1 (<0.1%)	1 (<0.1%)
ATRACURIUM	0	1 (<0.1%)	1 (<0.1%)
ATRACURIUM BESILATE	0	1 (<0.1%)	1 (<0.1%)
ATROPA BELLADONNA EXTRACT	1 (<0.1%)	0	1 (<0.1%)
ATTAPULGITE	0	1 (<0.1%)	1 (<0.1%)
BACLOFEN	1 (<0.1%)	0	1 (<0.1%)
BECLOMETASONE	1 (<0.1%)	0	1 (<0.1%)
BENACTYZINE HYDROCHLORIDE	0	1 (<0.1%)	1 (<0.1%)
BENAZEPRIL	1 (<0.1%)	0	1 (<0.1%)
BENZALKONIUM BROMIDE	0	1 (<0.1%)	1 (<0.1%)
BENZATROPINE MESILATE	0	1 (<0.1%)	1 (<0.1%)
BENZYDAMINE HYDROCHLORIDE	1 (<0.1%)	0	1 (<0.1%)
BENZYL BENZOATE	1 (<0.1%)	0	1 (<0.1%)
BENZYLPENICILLIN	1 (<0.1%)	0	1 (<0.1%)
BENZYLPENICILLIN POTASSIUM	0	1 (<0.1%)	1 (<0.1%)
BENZYLPENICILLIN SODIUM	1 (<0.1%)	0	1 (<0.1%)

Table 14.1.1 / 38: Number of subjects who took at least one concomitant medication (FAS)

Substance	LCS12 N=1432 (100%)	LCS16 N=1452 (100%)	Total N=2884 (100%)
BETA-LACTAM ANTIBACTERIALS, PENICILLINS	1 (<0.1%)	0	1 (<0.1%)
BETAINE HYDROCHLORIDE	0	1 (<0.1%)	1 (<0.1%)
BETAMETHASONE BENZOATE	0	1 (<0.1%)	1 (<0.1%)
BIBROCATHOL	1 (<0.1%)	0	1 (<0.1%)
BORNEOL	0	1 (<0.1%)	1 (<0.1%)
BROMOCRIPTINE MESILATE	0	1 (<0.1%)	1 (<0.1%)
BUPRENORPHINE	1 (<0.1%)	0	1 (<0.1%)
BUTOCONAZOLE	1 (<0.1%)	0	1 (<0.1%)
BUTORPHANOL TARTRATE	1 (<0.1%)	0	1 (<0.1%)
CABERGOLINE	0	1 (<0.1%)	1 (<0.1%)
CAFFEINE CITRATE	0	1 (<0.1%)	1 (<0.1%)
CALCIFEDIOL	1 (<0.1%)	0	1 (<0.1%)
CALCITONIN, SALMON	0	1 (<0.1%)	1 (<0.1%)
CALCIUM CHLORIDE ANHYDROUS	1 (<0.1%)	0	1 (<0.1%)
CALCIUM DOBESILATE	0	1 (<0.1%)	1 (<0.1%)
CALCIUM FOLINATE	1 (<0.1%)	0	1 (<0.1%)
CALENDULA OFFICINALIS	0	1 (<0.1%)	1 (<0.1%)
CAMPHENE	0	1 (<0.1%)	1 (<0.1%)
CAMPHOR MONOBROMIDE	1 (<0.1%)	0	1 (<0.1%)
CAMYLOFIN HYDROCHLORIDE	1 (<0.1%)	0	1 (<0.1%)
CANDIDA ALBICANS	1 (<0.1%)	0	1 (<0.1%)
CANDIDA PARAPSILOSIS	1 (<0.1%)	0	1 (<0.1%)
CAPILLARY STABILIZING AGENTS	0	1 (<0.1%)	1 (<0.1%)
CAPSAICIN	1 (<0.1%)	0	1 (<0.1%)
CARBOCISTEINE	0	1 (<0.1%)	1 (<0.1%)
CARBOHYDRATES/PROTEINS/MINERALS/VITAMINS, COM	1 (<0.1%)	0	1 (<0.1%)
CARBROMAL	0	1 (<0.1%)	1 (<0.1%)
CARVEDILOL	1 (<0.1%)	0	1 (<0.1%)
CEFACLOR	1 (<0.1%)	0	1 (<0.1%)
CEFALOTIN	1 (<0.1%)	0	1 (<0.1%)
CEFEPIME	0	1 (<0.1%)	1 (<0.1%)
CEFOTAXIME	0	1 (<0.1%)	1 (<0.1%)
CEFOXITIN SODIUM	0	1 (<0.1%)	1 (<0.1%)
CEFTRIAXONE SODIUM	1 (<0.1%)	0	1 (<0.1%)
CELLULASE	0	1 (<0.1%)	1 (<0.1%)
CENTELLA ASIATICA	0	1 (<0.1%)	1 (<0.1%)
CEPHAELIS SPP. FLUID EXTRACT	1 (<0.1%)	0	1 (<0.1%)
CHLORHEXIDINE HYDROCHLORIDE	1 (<0.1%)	0	1 (<0.1%)
CHLORINE	1 (<0.1%)	0	1 (<0.1%)
CHLORMADINONE ACETATE	0	1 (<0.1%)	1 (<0.1%)
CHLOROFORM	1 (<0.1%)	0	1 (<0.1%)
CHLOROQUINE	0	1 (<0.1%)	1 (<0.1%)
CHLORQUINALDOL	1 (<0.1%)	0	1 (<0.1%)
CHLORZOXAZONE	1 (<0.1%)	0	1 (<0.1%)
CHROMIC CHLORIDE	1 (<0.1%)	0	1 (<0.1%)

Table 14.1.1 / 38: Number of subjects who took at least one concomitant medication (FAS)

Substance	LCS12 N=1432 (100%)	LCS16 N=1452 (100%)	Total N=2884 (100%)
CHROMIUM PICOLINATE	1 (<0.1%)	0	1 (<0.1%)
CICLOPIROX OLAMINE	0	1 (<0.1%)	1 (<0.1%)
CIMETIDINE	0	1 (<0.1%)	1 (<0.1%)
CIPROFLOXACIN LACTATE	0	1 (<0.1%)	1 (<0.1%)
CISAPRIDE	1 (<0.1%)	0	1 (<0.1%)
CITICOLINE	0	1 (<0.1%)	1 (<0.1%)
CLEMASTINE	1 (<0.1%)	0	1 (<0.1%)
CLENBUTEROL HYDROCHLORIDE	1 (<0.1%)	0	1 (<0.1%)
CLIOQUINOL	0	1 (<0.1%)	1 (<0.1%)
CLOMIPRAMINE HYDROCHLORIDE	1 (<0.1%)	0	1 (<0.1%)
CLOXACILLIN	1 (<0.1%)	0	1 (<0.1%)
CODEINE HYDROCHLORIDE	1 (<0.1%)	0	1 (<0.1%)
CODEINE PHOSPHATE HEMIHYDRATE	0	1 (<0.1%)	1 (<0.1%)
COLESEVELAM HYDROCHLORIDE	1 (<0.1%)	0	1 (<0.1%)
COLESTIPOL	0	1 (<0.1%)	1 (<0.1%)
COMBINATIONS OF VITAMINS	1 (<0.1%)	0	1 (<0.1%)
CONTACT LAXATIVES	1 (<0.1%)	0	1 (<0.1%)
CONTRACEPTIVES FOR TOPICAL USE	1 (<0.1%)	0	1 (<0.1%)
COPPER	0	1 (<0.1%)	1 (<0.1%)
COPPER GLUCONATE	1 (<0.1%)	0	1 (<0.1%)
CORTICOSTEROID NOS	0	1 (<0.1%)	1 (<0.1%)
CORTICOSTEROIDS AND ANTIINFECTIVES IN COMB.	0	1 (<0.1%)	1 (<0.1%)
CUPRIC CARBONATE, BASIC	1 (<0.1%)	0	1 (<0.1%)
CYANOCOBALAMIN-TANNIN COMPLEX	1 (<0.1%)	0	1 (<0.1%)
CYCLOPENTOLATE HYDROCHLORIDE	1 (<0.1%)	0	1 (<0.1%)
CYPROHEPTADINE	1 (<0.1%)	0	1 (<0.1%)
CYSTINE	0	1 (<0.1%)	1 (<0.1%)
D-MANNOSE	1 (<0.1%)	0	1 (<0.1%)
DABIGATRAN ETEXILATE	0	1 (<0.1%)	1 (<0.1%)
DALTEPARIN	1 (<0.1%)	0	1 (<0.1%)
DAPSONE	0	1 (<0.1%)	1 (<0.1%)
DAPTOMYCIN	0	1 (<0.1%)	1 (<0.1%)
DARIFENACIN	0	1 (<0.1%)	1 (<0.1%)
DESVENLAFAXINE	0	1 (<0.1%)	1 (<0.1%)
DEXAMETHASONE ACETATE	1 (<0.1%)	0	1 (<0.1%)
DEXAMETHASONE SODIUM PHOSPHATE	1 (<0.1%)	0	1 (<0.1%)
DEXMETHYLPHENIDATE HYDROCHLORIDE	1 (<0.1%)	0	1 (<0.1%)
DICLOXACILLIN	0	1 (<0.1%)	1 (<0.1%)
DICLOXACILLIN SODIUM MONOHYDRATE	0	1 (<0.1%)	1 (<0.1%)
DIGOXIN	0	1 (<0.1%)	1 (<0.1%)
DIHYDROXYALUMINUM SODIUM CARBONATE	1 (<0.1%)	0	1 (<0.1%)
DIMETHYL SULFOXIDE	1 (<0.1%)	0	1 (<0.1%)
DIMETICONE	0	1 (<0.1%)	1 (<0.1%)
DIPHENHYDRAMINE CITRATE	1 (<0.1%)	0	1 (<0.1%)
DIPHENOXYLATE HYDROCHLORIDE	0	1 (<0.1%)	1 (<0.1%)

Table 14.1.1 / 38: Number of subjects who took at least one concomitant medication (FAS)

Substance	LCS12 N=1432 (100%)	LCS16 N=1452 (100%)	Total N=2884 (100%)
DIPHENYLPYRALINE HYDROCHLORIDE	1 (<0.1%)	0	1 (<0.1%)
DIPHTHERIA AND TETANUS TOXOIDS AND PERTUSSIS	0	1 (<0.1%)	1 (<0.1%)
DISULFIRAM	0	1 (<0.1%)	1 (<0.1%)
DL- LACTIC ACID	0	1 (<0.1%)	1 (<0.1%)
DOMPERIDONE MALEATE	1 (<0.1%)	0	1 (<0.1%)
DONEPEZIL HYDROCHLORIDE	1 (<0.1%)	0	1 (<0.1%)
DROTAVERINE	1 (<0.1%)	0	1 (<0.1%)
DRUG USED IN DIABETES	1 (<0.1%)	0	1 (<0.1%)
DULOXETINE	1 (<0.1%)	0	1 (<0.1%)
DYDROGESTERONE	1 (<0.1%)	0	1 (<0.1%)
EDETIC ACID	1 (<0.1%)	0	1 (<0.1%)
EFLORNITHINE	0	1 (<0.1%)	1 (<0.1%)
ELECTROLYTE SOLUTIONS	0	1 (<0.1%)	1 (<0.1%)
EMEDASTINE	1 (<0.1%)	0	1 (<0.1%)
EMOLLIENTS AND PROTECTIVES	0	1 (<0.1%)	1 (<0.1%)
ENOXACIN SESQUIHYDRATE	1 (<0.1%)	0	1 (<0.1%)
ERTAPENEM SODIUM	1 (<0.1%)	0	1 (<0.1%)
ERYTHROMYCIN ETHYLSUCCINATE	0	1 (<0.1%)	1 (<0.1%)
ESCULOSIDE	1 (<0.1%)	0	1 (<0.1%)
ESSENTIAL OILS NOS	0	1 (<0.1%)	1 (<0.1%)
ESTROGENS CONJUGATED	0	1 (<0.1%)	1 (<0.1%)
ETANERCEPT	0	1 (<0.1%)	1 (<0.1%)
ETHAMBUTOL	1 (<0.1%)	0	1 (<0.1%)
ETIFOXINE	0	1 (<0.1%)	1 (<0.1%)
ETOFENAMATE	0	1 (<0.1%)	1 (<0.1%)
ETYNODIOL DIACETATE	0	1 (<0.1%)	1 (<0.1%)
EXPECTORANTS	0	1 (<0.1%)	1 (<0.1%)
FAMCICLOVIR	1 (<0.1%)	0	1 (<0.1%)
FENCHONE	0	1 (<0.1%)	1 (<0.1%)
FENOTEROL	1 (<0.1%)	0	1 (<0.1%)
FENOXAZOLINE HYDROCHLORIDE	0	1 (<0.1%)	1 (<0.1%)
FERRIC HYDROXIDE POLYMALTOSE	1 (<0.1%)	0	1 (<0.1%)
FERRIC HYDROXIDE POLYMALTOSE COMPLEX	0	1 (<0.1%)	1 (<0.1%)
FERRIC SODIUM GLUCONATE COMPLEX	0	1 (<0.1%)	1 (<0.1%)
FERROUS GLUCONATE	0	1 (<0.1%)	1 (<0.1%)
FIBRE, DIETARY	1 (<0.1%)	0	1 (<0.1%)
FLUNARIZINE	1 (<0.1%)	0	1 (<0.1%)
FLUOCORTOLONE	0	1 (<0.1%)	1 (<0.1%)
FLUOCORTOLONE PIVALATE	0	1 (<0.1%)	1 (<0.1%)
FLUOROMETHOLONE	0	1 (<0.1%)	1 (<0.1%)
FLUOROQUINOLONES	0	1 (<0.1%)	1 (<0.1%)
FLUOROURACIL	0	1 (<0.1%)	1 (<0.1%)
FLUTEMAZEPAM	0	1 (<0.1%)	1 (<0.1%)
FLUVOXAMINE MALEATE	0	1 (<0.1%)	1 (<0.1%)
FORMOTEROL	1 (<0.1%)	0	1 (<0.1%)

Table 14.1.1 / 38: Number of subjects who took at least one concomitant medication (FAS)

Substance	LCS12 N=1432 (100%)	LCS16 N=1452 (100%)	Total N=2884 (100%)
FOSINOPRIL SODIUM	1 (<0.1%)	0	1 (<0.1%)
FROVATRIPTAN	0	1 (<0.1%)	1 (<0.1%)
FUROSEMIDE SODIUM	1 (<0.1%)	0	1 (<0.1%)
GEMIFLOXACIN	0	1 (<0.1%)	1 (<0.1%)
GENITO URINARY SYSTEM AND SEX HORMONES	0	1 (<0.1%)	1 (<0.1%)
GINKGO BILOBA	1 (<0.1%)	0	1 (<0.1%)
GLIBENCLAMIDE	1 (<0.1%)	0	1 (<0.1%)
GLUCOMANNAN	0	1 (<0.1%)	1 (<0.1%)
GLUCONIC ACID	1 (<0.1%)	0	1 (<0.1%)
GLYCERYL TRINITRATE	1 (<0.1%)	0	1 (<0.1%)
GLYCOLIC ACID	1 (<0.1%)	0	1 (<0.1%)
GLYCYRRHIZIC ACID, AMMONIUM SALT	1 (<0.1%)	0	1 (<0.1%)
GOSERELIN	0	1 (<0.1%)	1 (<0.1%)
GUAIACOL	0	1 (<0.1%)	1 (<0.1%)
GUAIACOL CARBONATE	1 (<0.1%)	0	1 (<0.1%)
GYNAECOLOGICAL ANTIINFECTIVES AND ANTISEPTICS	1 (<0.1%)	0	1 (<0.1%)
HAEMOPHILUS INFLUENZA LYSATE	0	1 (<0.1%)	1 (<0.1%)
HAEMOPHILUS INFLUENZAE TYPE B POLYSACCHARIDE	1 (<0.1%)	0	1 (<0.1%)
HALOPERIDOL DECANOATE	0	1 (<0.1%)	1 (<0.1%)
HEDERA HELIX	1 (<0.1%)	0	1 (<0.1%)
HELICIDINE	0	1 (<0.1%)	1 (<0.1%)
HEPARIN CALCIUM	1 (<0.1%)	0	1 (<0.1%)
HETASTARCH	1 (<0.1%)	0	1 (<0.1%)
HOMATROPINE	0	1 (<0.1%)	1 (<0.1%)
HOMATROPINE METHYLBROMIDE	1 (<0.1%)	0	1 (<0.1%)
HYALURONIC ACID	1 (<0.1%)	0	1 (<0.1%)
HYDROCORTISONE CAPRONATE	1 (<0.1%)	0	1 (<0.1%)
HYDROCORTISONE SODIUM SUCCINATE	1 (<0.1%)	0	1 (<0.1%)
HYDROXOCOBALAMIN	1 (<0.1%)	0	1 (<0.1%)
HYDROXYZINE EMBONATE	0	1 (<0.1%)	1 (<0.1%)
HYETELLOSE	0	1 (<0.1%)	1 (<0.1%)
HYOSCINE METHOBROMIDE	0	1 (<0.1%)	1 (<0.1%)
HYOSCINE METHONITRATE	1 (<0.1%)	0	1 (<0.1%)
HYPERICUM PERFORATUM	1 (<0.1%)	0	1 (<0.1%)
IMIPENEM	1 (<0.1%)	0	1 (<0.1%)
IMIPRAMINE HYDROCHLORIDE	1 (<0.1%)	0	1 (<0.1%)
IMMUNOGLOBULIN HUMAN ANTI-RH	0	1 (<0.1%)	1 (<0.1%)
IMMUNOSUPPRESSIVE AGENTS	0	1 (<0.1%)	1 (<0.1%)
INOSINE PRANOBEX	0	1 (<0.1%)	1 (<0.1%)
INSULIN	0	1 (<0.1%)	1 (<0.1%)
INSULIN ASPART	1 (<0.1%)	0	1 (<0.1%)
INSULIN GLARGINE	0	1 (<0.1%)	1 (<0.1%)
INTESTINAL ANTIINFECTIVES	0	1 (<0.1%)	1 (<0.1%)
INVERT SUGAR	1 (<0.1%)	0	1 (<0.1%)
IODINE	0	1 (<0.1%)	1 (<0.1%)

Table 14.1.1 / 38: Number of subjects who took at least one concomitant medication (FAS)

Substance	LCS12 N=1432 (100%)	LCS16 N=1452 (100%)	Total N=2884 (100%)
IOPAMIDOL	0	1 (<0.1%)	1 (<0.1%)
IPRATROPIUM	1 (<0.1%)	0	1 (<0.1%)
IPRAZOCHROME	0	1 (<0.1%)	1 (<0.1%)
IRON IN COMBINATION WITH FOLIC ACID	1 (<0.1%)	0	1 (<0.1%)
ISOMETHEPTENE MUCATE	0	1 (<0.1%)	1 (<0.1%)
ISONIAZID	1 (<0.1%)	0	1 (<0.1%)
IVERMECTIN	1 (<0.1%)	0	1 (<0.1%)
JAPANESE ENCEPHALITIS VACCINE	1 (<0.1%)	0	1 (<0.1%)
KETAMINE HYDROCHLORIDE	1 (<0.1%)	0	1 (<0.1%)
KLEBSIELLA OZAENAE LYSATE	0	1 (<0.1%)	1 (<0.1%)
KLEBSIELLA PNEUMONIAE LYSATE	0	1 (<0.1%)	1 (<0.1%)
LABETALOL	1 (<0.1%)	0	1 (<0.1%)
LABETALOL HYDROCHLORIDE	1 (<0.1%)	0	1 (<0.1%)
LACTOBACILLUS CASEI	0	1 (<0.1%)	1 (<0.1%)
LACTOBACILLUS RHAMNOSUS	1 (<0.1%)	0	1 (<0.1%)
LANOLIN	1 (<0.1%)	0	1 (<0.1%)
LAXATIVES	1 (<0.1%)	0	1 (<0.1%)
LEFLUNOMIDE	1 (<0.1%)	0	1 (<0.1%)
LEVOBUPIVACAINE	1 (<0.1%)	0	1 (<0.1%)
LINOLEIC ACID	1 (<0.1%)	0	1 (<0.1%)
LINUM USITATISSIMUM	0	1 (<0.1%)	1 (<0.1%)
LIOTHYRONINE	0	1 (<0.1%)	1 (<0.1%)
LITHIUM	0	1 (<0.1%)	1 (<0.1%)
LODOXAMIDE	1 (<0.1%)	0	1 (<0.1%)
LOSARTAN POTASSIUM	1 (<0.1%)	0	1 (<0.1%)
LOXOPROFEN	1 (<0.1%)	0	1 (<0.1%)
LYMECYCLINE	0	1 (<0.1%)	1 (<0.1%)
MAGNESIUM AMINO ACID CHELATE	1 (<0.1%)	0	1 (<0.1%)
MAGNESIUM CHLORIDE ANHYDROUS	1 (<0.1%)	0	1 (<0.1%)
MAGNESIUM CITRATE	1 (<0.1%)	0	1 (<0.1%)
MAGNESIUM GLUCONATE	0	1 (<0.1%)	1 (<0.1%)
MANGANESE CHLORIDE	1 (<0.1%)	0	1 (<0.1%)
MANGANESE GLUCONATE	1 (<0.1%)	0	1 (<0.1%)
MEBEVERINE HYDROCHLORIDE	0	1 (<0.1%)	1 (<0.1%)
MECLOZINE HYDROCHLORIDE	1 (<0.1%)	0	1 (<0.1%)
MEGLUMINE AMIDOTRIZOATE	0	1 (<0.1%)	1 (<0.1%)
MELILOTUS OFFICINALIS	1 (<0.1%)	0	1 (<0.1%)
MENADIONE	0	1 (<0.1%)	1 (<0.1%)
MEPACRINE HYDROCHLORIDE	0	1 (<0.1%)	1 (<0.1%)
METFORMIN HYDROCHLORIDE	0	1 (<0.1%)	1 (<0.1%)
METHADONE	0	1 (<0.1%)	1 (<0.1%)
METHENAMINE HIPPURATE	1 (<0.1%)	0	1 (<0.1%)
METHYLPHENIDATE	1 (<0.1%)	0	1 (<0.1%)
METHYLPREDNISOLONE ACEPONATE	1 (<0.1%)	0	1 (<0.1%)
METOPIMAZINE	0	1 (<0.1%)	1 (<0.1%)

Table 14.1.1 / 38: Number of subjects who took at least one concomitant medication (FAS)

Substance	LCS12<br>N=1432 (100%)	LCS16<br>N=1452 (100%)	Total<br>N=2884 (100%)
METRONIDAZOLE HYDROCHLORIDE	1 (<0.1%)	0	1 (<0.1%)
MEXILETINE	0	1 (<0.1%)	1 (<0.1%)
MIANSERIN	1 (<0.1%)	0	1 (<0.1%)
MIANSERIN HYDROCHLORIDE	0	1 (<0.1%)	1 (<0.1%)
MILNACIPRAN HYDROCHLORIDE	0	1 (<0.1%)	1 (<0.1%)
MINERAL OIL EMULSION	0	1 (<0.1%)	1 (<0.1%)
MINERAL SUPPLEMENTS	0	1 (<0.1%)	1 (<0.1%)
MIVACURIUM CHLORIDE	0	1 (<0.1%)	1 (<0.1%)
MOCLOBEMIDE	1 (<0.1%)	0	1 (<0.1%)
MORPHINE HYDROCHLORIDE	1 (<0.1%)	0	1 (<0.1%)
MOXONIDINE	0	1 (<0.1%)	1 (<0.1%)
MULTIVITAMINS AND IRON	1 (<0.1%)	0	1 (<0.1%)
NALOXONE	1 (<0.1%)	0	1 (<0.1%)
NAPHAZOLINE HYDROCHLORIDE	1 (<0.1%)	0	1 (<0.1%)
NASAL DECONGESTANTS FOR TOPICAL USE	1 (<0.1%)	0	1 (<0.1%)
NEBIVOLOL	1 (<0.1%)	0	1 (<0.1%)
NEISSERIA CATARRHALIS LYSATE	0	1 (<0.1%)	1 (<0.1%)
NEOSTIGMINE	0	1 (<0.1%)	1 (<0.1%)
NEOSTIGMINE BROMIDE	1 (<0.1%)	0	1 (<0.1%)
NIMODIPINE	1 (<0.1%)	0	1 (<0.1%)
NITAZOXANIDE	1 (<0.1%)	0	1 (<0.1%)
NITROFURAL	1 (<0.1%)	0	1 (<0.1%)
NITROUS OXIDE	0	1 (<0.1%)	1 (<0.1%)
OLMESARTAN MEDOXOMIL	0	1 (<0.1%)	1 (<0.1%)
OMALIZUMAB	0	1 (<0.1%)	1 (<0.1%)
OMEGA-3 FATTY ACIDS	0	1 (<0.1%)	1 (<0.1%)
OPIUM ALKALOIDS AND DERIVATIVES	0	1 (<0.1%)	1 (<0.1%)
OPIUM ALKALOIDS TOTAL	1 (<0.1%)	0	1 (<0.1%)
OTHER ANTIDEPRESSANTS	1 (<0.1%)	0	1 (<0.1%)
OTHER ANTIINFLAMMATORY AGENTS IN COMB.	0	1 (<0.1%)	1 (<0.1%)
OTHER DERMATOLOGICAL PREPARATIONS	0	1 (<0.1%)	1 (<0.1%)
OTHER HYPNOTICS AND SEDATIVES	0	1 (<0.1%)	1 (<0.1%)
OTOLOGICALS	1 (<0.1%)	0	1 (<0.1%)
OXAPROZIN	1 (<0.1%)	0	1 (<0.1%)
OXETORONE FUMARATE	1 (<0.1%)	0	1 (<0.1%)
OXYBUTYNIN	0	1 (<0.1%)	1 (<0.1%)
OXYBUTYNIN HYDROCHLORIDE	1 (<0.1%)	0	1 (<0.1%)
OXYTOCIN	1 (<0.1%)	0	1 (<0.1%)
PANCREATIN	0	1 (<0.1%)	1 (<0.1%)
PANICUM MILIACEUM	0	1 (<0.1%)	1 (<0.1%)
PAPAIN	0	1 (<0.1%)	1 (<0.1%)
PARAFFIN, LIQUID	0	1 (<0.1%)	1 (<0.1%)
PARAHYDROXYBENZOIC ACID	1 (<0.1%)	0	1 (<0.1%)
PECTIN	0	1 (<0.1%)	1 (<0.1%)
PELARGONIUM PULVERULENTUM ROOT	0	1 (<0.1%)	1 (<0.1%)

Table 14.1.1 / 38: Number of subjects who took at least one concomitant medication (FAS)

Substance	LCS12 N=1432 (100%)	LCS16 N=1452 (100%)	Total N=2884 (100%)
PELARGONIUM SIDOIDES	0	1 (<0.1%)	1 (<0.1%)
PELARGONIUM SIDOIDES ROOT	0	1 (<0.1%)	1 (<0.1%)
PENTOSAN POLYSULFATE SODIUM	0	1 (<0.1%)	1 (<0.1%)
PENTOXYVERINE CITRATE	0	1 (<0.1%)	1 (<0.1%)
PERINDOPRIL	1 (<0.1%)	0	1 (<0.1%)
PERTUSSIS VACCINE ACELLULAR	0	1 (<0.1%)	1 (<0.1%)
PHENAZONE	1 (<0.1%)	0	1 (<0.1%)
PHENETICILLIN POTASSIUM	0	1 (<0.1%)	1 (<0.1%)
PHENYLTOLOXAMINE CITRATE	0	1 (<0.1%)	1 (<0.1%)
PHENYTOIN	1 (<0.1%)	0	1 (<0.1%)
PHOSPHORIC ACID	1 (<0.1%)	0	1 (<0.1%)
PHOSPHORIC ACID SODIUM	0	1 (<0.1%)	1 (<0.1%)
PHTHALYLSULFATHIAZOLE	0	1 (<0.1%)	1 (<0.1%)
PINAVERIUM	0	1 (<0.1%)	1 (<0.1%)
PINAVERIUM BROMIDE	1 (<0.1%)	0	1 (<0.1%)
PINENE	0	1 (<0.1%)	1 (<0.1%)
PIRITRAMIDE	0	1 (<0.1%)	1 (<0.1%)
PIROMIDIC ACID	0	1 (<0.1%)	1 (<0.1%)
PITOFENONE HYDROCHLORIDE	0	1 (<0.1%)	1 (<0.1%)
PLANTAGO AFRA SEED	0	1 (<0.1%)	1 (<0.1%)
PLANTAGO OVATA	0	1 (<0.1%)	1 (<0.1%)
POLICRESULEN	1 (<0.1%)	0	1 (<0.1%)
POLIOMYELITIS VACCINE INACTIVATED	0	1 (<0.1%)	1 (<0.1%)
POLIOMYELITIS VACCINES	1 (<0.1%)	0	1 (<0.1%)
POTASSIUM CITRATE	1 (<0.1%)	0	1 (<0.1%)
POTASSIUM PHOSPHATE MONOBASIC	0	1 (<0.1%)	1 (<0.1%)
POVIDONE-IODINE	1 (<0.1%)	0	1 (<0.1%)
PRAZEPAM	0	1 (<0.1%)	1 (<0.1%)
PREDNISOLONE SODIUM PHOSPHATE	1 (<0.1%)	0	1 (<0.1%)
PRENOXDIAZIN HYDROCHLORIDE	1 (<0.1%)	0	1 (<0.1%)
PRISTINAMYCIN	1 (<0.1%)	0	1 (<0.1%)
PROBENECID	1 (<0.1%)	0	1 (<0.1%)
PROCAINE	1 (<0.1%)	0	1 (<0.1%)
PROCHLORPERAZINE	0	1 (<0.1%)	1 (<0.1%)
PROPOLIS	0	1 (<0.1%)	1 (<0.1%)
PROPRANOLOL HYDROCHLORIDE	1 (<0.1%)	0	1 (<0.1%)
PROPYLENE GLYCOL	1 (<0.1%)	0	1 (<0.1%)
PROTECTIVES AGAINST UV-RADIATION	1 (<0.1%)	0	1 (<0.1%)
PROTEIN SUPPLEMENTS	1 (<0.1%)	0	1 (<0.1%)
PROXIBARBAL	0	1 (<0.1%)	1 (<0.1%)
PULSATILLA PRATENSIS	1 (<0.1%)	0	1 (<0.1%)
PYRAZINAMIDE	1 (<0.1%)	0	1 (<0.1%)
PYRIDOSTIGMINE BROMIDE	0	1 (<0.1%)	1 (<0.1%)
PYRIMETHAMINE	0	1 (<0.1%)	1 (<0.1%)
QUINAPRIL HYDROCHLORIDE	1 (<0.1%)	0	1 (<0.1%)

Table 14.1.1 / 38: Number of subjects who took at least one concomitant medication (FAS)

Substance	LCS12 N=1432 (100%)	LCS16 N=1452 (100%)	Total N=2884 (100%)
RABIES IMMUNEGLOBULIN	0	1 (<0.1%)	1 (<0.1%)
REBOXETINE	1 (<0.1%)	0	1 (<0.1%)
RED BLOOD CELLS, CONCENTRATED	0	1 (<0.1%)	1 (<0.1%)
REMIFENTANIL	0	1 (<0.1%)	1 (<0.1%)
RETINOL ACETATE	1 (<0.1%)	0	1 (<0.1%)
RETINOL PALMITATE	1 (<0.1%)	0	1 (<0.1%)
RIFAMPICIN	1 (<0.1%)	0	1 (<0.1%)
RIFAXIMIN	0	1 (<0.1%)	1 (<0.1%)
RUSCUS ACULEATUS	1 (<0.1%)	0	1 (<0.1%)
SALICYLATE SODIUM	0	1 (<0.1%)	1 (<0.1%)
SALSALATE	0	1 (<0.1%)	1 (<0.1%)
SALVIA OFFICINALIS	0	1 (<0.1%)	1 (<0.1%)
SELENOMETHIONINE	1 (<0.1%)	0	1 (<0.1%)
SENNA ALEXANDRINA	1 (<0.1%)	0	1 (<0.1%)
SERTACONAZOLE	0	1 (<0.1%)	1 (<0.1%)
SEX HORMONES AND MODULATORS OF THE GENI. SYS.	1 (<0.1%)	0	1 (<0.1%)
SHARK-LIVER OIL	0	1 (<0.1%)	1 (<0.1%)
SMALLPOX VACCINE	1 (<0.1%)	0	1 (<0.1%)
SODIUM	1 (<0.1%)	0	1 (<0.1%)
SODIUM ACETATE	1 (<0.1%)	0	1 (<0.1%)
SODIUM AMIDOTRIZOATE	0	1 (<0.1%)	1 (<0.1%)
SODIUM ASCORBATE	1 (<0.1%)	0	1 (<0.1%)
SODIUM LAURYL SULFATE	0	1 (<0.1%)	1 (<0.1%)
SODIUM LAURYL SULFOACETATE	0	1 (<0.1%)	1 (<0.1%)
SODIUM PHOSPHATE MONOBASIC (ANHYDRATE)	0	1 (<0.1%)	1 (<0.1%)
SODIUM SULFATE	0	1 (<0.1%)	1 (<0.1%)
SOFT PARAFFIN AND FAT PRODUCTS	0	1 (<0.1%)	1 (<0.1%)
SOFTENERS, EMOLLIENTS	1 (<0.1%)	0	1 (<0.1%)
SOLIFENACIN	0	1 (<0.1%)	1 (<0.1%)
SORBIC ACID	0	1 (<0.1%)	1 (<0.1%)
SORBITOL	0	1 (<0.1%)	1 (<0.1%)
SPIRAMYCIN	1 (<0.1%)	0	1 (<0.1%)
SPIRULINA SPP.	0	1 (<0.1%)	1 (<0.1%)
STAPHYLOCOCCUS AUREUS LYSATE	0	1 (<0.1%)	1 (<0.1%)
STOMATOLOGICALS, MOUTH PREPARATIONS	0	1 (<0.1%)	1 (<0.1%)
STREPTOCOCCUS PNEUMONIAE LYSATE	0	1 (<0.1%)	1 (<0.1%)
STREPTOCOCCUS PYROGENES LYSATE	0	1 (<0.1%)	1 (<0.1%)
STREPTOCOCCUS VIRIDANS LYSATE	0	1 (<0.1%)	1 (<0.1%)
STREPTOMYCIN	0	1 (<0.1%)	1 (<0.1%)
SUFENTANIL CITRATE	1 (<0.1%)	0	1 (<0.1%)
SULCONAZOLE NITRATE	1 (<0.1%)	0	1 (<0.1%)
SULFACETAMIDE	1 (<0.1%)	0	1 (<0.1%)
SULFACETAMIDE SODIUM	0	1 (<0.1%)	1 (<0.1%)
SULFAGUANIDINE	1 (<0.1%)	0	1 (<0.1%)
SULFOGAIACOL	1 (<0.1%)	0	1 (<0.1%)

Table 14.1.1 / 38: Number of subjects who took at least one concomitant medication (FAS)

Substance	LCS12 N=1432 (100%)	LCS16 N=1452 (100%)	Total N=2884 (100%)
SULFUR	0	1 (<0.1%)	1 (<0.1%)
SULINDAC	0	1 (<0.1%)	1 (<0.1%)
SYMPATHOMIMETICS	1 (<0.1%)	0	1 (<0.1%)
TAZAROTENE	1 (<0.1%)	0	1 (<0.1%)
TERPIN HYDRATE	0	1 (<0.1%)	1 (<0.1%)
TETANUS IMMUNE GLOBULIN (HUMAN)	1 (<0.1%)	0	1 (<0.1%)
TETRAZEPAM	1 (<0.1%)	0	1 (<0.1%)
THEOPHYLLINE	1 (<0.1%)	0	1 (<0.1%)
THIAMAZOLE	1 (<0.1%)	0	1 (<0.1%)
THIOPENTAL	0	1 (<0.1%)	1 (<0.1%)
TICK-BORNE ENCEPHALITIS VACCINE	0	1 (<0.1%)	1 (<0.1%)
TINZAPARIN SODIUM	1 (<0.1%)	0	1 (<0.1%)
TIOTROPIUM BROMIDE	1 (<0.1%)	0	1 (<0.1%)
TOCOPHERYL ACETATE	1 (<0.1%)	0	1 (<0.1%)
TOLPERISONE	0	1 (<0.1%)	1 (<0.1%)
TONICS	1 (<0.1%)	0	1 (<0.1%)
TRANYLCYPROMINE	0	1 (<0.1%)	1 (<0.1%)
TRIAMCINOLONE HEXACETONIDE	0	1 (<0.1%)	1 (<0.1%)
TRICHLORMETHIAZIDE	0	1 (<0.1%)	1 (<0.1%)
TRIMIPRAMINE	1 (<0.1%)	0	1 (<0.1%)
TRIPROLIDINE HYDROCHLORIDE	1 (<0.1%)	0	1 (<0.1%)
TRITICUM AESTIVUM GERM OIL	0	1 (<0.1%)	1 (<0.1%)
TUBERCULIN PPD	0	1 (<0.1%)	1 (<0.1%)
TYPHOID VACCINE, LIVE ORAL	0	1 (<0.1%)	1 (<0.1%)
TYPHUS VACCINE	1 (<0.1%)	0	1 (<0.1%)
TYROTHRICIN	1 (<0.1%)	0	1 (<0.1%)
UBIDECARENONE	1 (<0.1%)	0	1 (<0.1%)
VALPROIC ACID	1 (<0.1%)	0	1 (<0.1%)
VECURONIUM	1 (<0.1%)	0	1 (<0.1%)
VITAMIN B-COMPLEX WITH MINERALS	1 (<0.1%)	0	1 (<0.1%)
VITAMINS, OTHER COMBINATIONS	1 (<0.1%)	0	1 (<0.1%)
VITEX AGNUS-CASTUS	0	1 (<0.1%)	1 (<0.1%)
WARFARIN SODIUM	1 (<0.1%)	0	1 (<0.1%)
ZINC ACETATE	0	1 (<0.1%)	1 (<0.1%)
ZINC CITRATE	1 (<0.1%)	0	1 (<0.1%)
ZINC SULFATE	1 (<0.1%)	0	1 (<0.1%)

Note: Multiple substances per drug are possible. Therefore, the same drug may be counted for more than one substance for the same subject
Note: Medications that are ongoing at the start of treatment or medications that began any time from the start of study drug until the last day of study drug (includes start and stop dates of study drug) are included in this table.
Global Biostatistics: /by-sasp/patdb/projects/de04209/310442/stat/prod_interim03/pgms/t-cm.sas epkll 07OCT2011 10:50
End of table

Table 14.1.1 / 39: Number of subjects who took at least one post-treatment medication (FAS)

Substance	LCS12 N=1432 (100%)	LCS16 N=1452 (100%)	Total N=2884 (100%)
Number of subjects (%) with at least one post-treatment medication	20 (1.4%)	17 (1.2%)	37 (1.3%)
ETHINYLESTRADIOL	3 (0.2%)	4 (0.3%)	7 (0.2%)
METRONIDAZOLE	3 (0.2%)	3 (0.2%)	6 (0.2%)
PARACETAMOL	3 (0.2%)	1 (<0.1%)	4 (0.1%)
ETONOGESTREL	1 (<0.1%)	2 (0.1%)	3 (0.1%)
LIDOCAINE HYDROCHLORIDE	1 (<0.1%)	2 (0.1%)	3 (0.1%)
NITROFURANTOIN	1 (<0.1%)	2 (0.1%)	3 (0.1%)
ONDANSETRON	2 (0.1%)	1 (<0.1%)	3 (0.1%)
CEFTRIAXONE	1 (<0.1%)	1 (<0.1%)	2 (<0.1%)
CIPROFLOXACIN	2 (0.1%)	0	2 (<0.1%)
DOXYCYCLINE	1 (<0.1%)	1 (<0.1%)	2 (<0.1%)
EPINEPHRINE	0	2 (0.1%)	2 (<0.1%)
FENTANYL	1 (<0.1%)	1 (<0.1%)	2 (<0.1%)
IBUPROFEN	1 (<0.1%)	1 (<0.1%)	2 (<0.1%)
LEVONORGESTREL	2 (0.1%)	0	2 (<0.1%)
MIDAZOLAM HYDROCHLORIDE	2 (0.1%)	0	2 (<0.1%)
MORPHINE	2 (0.1%)	0	2 (<0.1%)
VALACICLOVIR HYDROCHLORIDE	2 (0.1%)	0	2 (<0.1%)
ACICLOVIR	1 (<0.1%)	0	1 (<0.1%)
ACRIVASTINE	0	1 (<0.1%)	1 (<0.1%)
ALLOPURINOL	1 (<0.1%)	0	1 (<0.1%)
AMPICILLIN	1 (<0.1%)	0	1 (<0.1%)
ANESTHETICS, GENERAL	0	1 (<0.1%)	1 (<0.1%)
CEFALEXIN	0	1 (<0.1%)	1 (<0.1%)
CEFAZOLIN SODIUM	1 (<0.1%)	0	1 (<0.1%)
CILASTATIN SODIUM	1 (<0.1%)	0	1 (<0.1%)
CLARITHROMYCIN	0	1 (<0.1%)	1 (<0.1%)
CLINDAMYCIN HYDROCHLORIDE	1 (<0.1%)	0	1 (<0.1%)
CLINDAMYCIN PHOSPHATE	1 (<0.1%)	0	1 (<0.1%)
DEXAMETHASONE	1 (<0.1%)	0	1 (<0.1%)
DIAZEPAM	0	1 (<0.1%)	1 (<0.1%)
DIPHENHYDRAMINE HYDROCHLORIDE	1 (<0.1%)	0	1 (<0.1%)
DOXYCYCLINE MONOHYDRATE	0	1 (<0.1%)	1 (<0.1%)
DROSPIRENONE	1 (<0.1%)	0	1 (<0.1%)
FAMOTIDINE	1 (<0.1%)	0	1 (<0.1%)
FLUCONAZOLE	1 (<0.1%)	0	1 (<0.1%)
HYDROCHLOROTHIAZIDE	1 (<0.1%)	0	1 (<0.1%)
HYDROMORPHONE	0	1 (<0.1%)	1 (<0.1%)
HYDROMORPHONE HYDROCHLORIDE	0	1 (<0.1%)	1 (<0.1%)
IMIPENEM	1 (<0.1%)	0	1 (<0.1%)
IMMUNOGLOBULIN HUMAN ANTI-RH	0	1 (<0.1%)	1 (<0.1%)
LAMOTRIGINE	0	1 (<0.1%)	1 (<0.1%)
LEVOFLOXACIN	1 (<0.1%)	0	1 (<0.1%)

Table 14.1.1 / 39: Number of subjects who took at least one post-treatment medication (FAS)

Substance	LCS12 N=1432 (100%)	LCS16 N=1452 (100%)	Total N=2884 (100%)
LEVOTHYROXINE SODIUM	1 (<0.1%)	0	1 (<0.1%)
LIDOCAINE	1 (<0.1%)	0	1 (<0.1%)
METHOTREXATE	0	1 (<0.1%)	1 (<0.1%)
NORELGESTROMIN	0	1 (<0.1%)	1 (<0.1%)
NORGESTIMATE	0	1 (<0.1%)	1 (<0.1%)
NORTRIPTYLINE	0	1 (<0.1%)	1 (<0.1%)
OXYCODONE HYDROCHLORIDE	1 (<0.1%)	0	1 (<0.1%)
PILOCARPINE HYDROCHLORIDE	1 (<0.1%)	0	1 (<0.1%)
PROMETHAZINE	0	1 (<0.1%)	1 (<0.1%)
PROPOFOL	1 (<0.1%)	0	1 (<0.1%)
PSEUDOEPHEDRINE HYDROCHLORIDE	0	1 (<0.1%)	1 (<0.1%)
ROCURONIUM BROMIDE	1 (<0.1%)	0	1 (<0.1%)
SODIUM CHLORIDE	1 (<0.1%)	0	1 (<0.1%)
SODIUM IODIDE (131 I)	1 (<0.1%)	0	1 (<0.1%)
SULBACTAM	1 (<0.1%)	0	1 (<0.1%)
SULFAMETHOXAZOLE	0	1 (<0.1%)	1 (<0.1%)
TRIMETHOPRIM	0	1 (<0.1%)	1 (<0.1%)

Note: Multiple substances per drug are possible. Therefore, the same drug may be counted for more than one substance for the same subject
Note: Medications that began after the last day of study drug are included in this table
Global Biostatistics: /by-sasp/patdb/projects/de04209/310442/stat/prod_interim03/pgms/t-cm.sas epkll 07OCT2011 10:50
End of table

14.1.2 Study drug administration and compliance

Table 14.1.2 / 1: Number of successful insertions by treatment (FAS)

	LCS12	LCS16	Total
Number of insertions	1481 (100.0%)	1509 (100.0%)	2990 (100.0%)
IUS insertion completed			
no	55 (3.7%)	64 (4.2%)	119 (4.0%)
yes	1426 (96.3%)	1445 (95.8%)	2871 (96.0%)

Note: Table shows all first and second insertion attempts
Global Biostatistics: /by-sasp/patdb/projects/de04209/310442/stat/prod_interim03/pgms/t-admin-compl.sas epkll 07OCT2011 10:51
End of table

Table 14.1.2 / 2: Number of successful first insertion by treatment (FAS)

	LCS12	LCS16	Total
Number of insertions	1432 (100.0%)	1452 (100.0%)	2884 (100.0%)
IUS insertion completed			
no	52 (3.6%)	62 (4.3%)	114 (4.0%)
yes	1380 (96.4%)	1390 (95.7%)	2770 (96.0%)

Global Biostatistics: /by-sasp/patdb/projects/de04209/310442/stat/prod_interim03/pgms/t-admin-compl.sas epkll 07OCT2011 10:51
End of table

Table 14.1.2 / 3: Reason for failure of first insertion by treatment (FAS)

	LCS12	LCS16	Total
Number of insertions	52 (100.0%)	62 (100.0%)	114 (100.0%)
IUS insertion failed, reason			
IUS became unsterile	0	3 (4.8%)	3 (2.6%)
cervix too tight	3 (5.8%)	7 (11.3%)	10 (8.8%)
IUS came out immediately after insertion	14 (26.9%)	23 (37.1%)	37 (32.5%)
IUS inserter became unsterile	1 (1.9%)	1 (1.6%)	2 (1.8%)
malfunction of the inserter	15 (28.8%)	12 (19.4%)	27 (23.7%)
small uterus	1 (1.9%)	1 (1.6%)	2 (1.8%)
vasovagal attack	0	1 (1.6%)	1 (0.9%)
pain	3 (5.8%)	0	3 (2.6%)
position of the uterus	2 (3.8%)	1 (1.6%)	3 (2.6%)
other	13 (25.0%)	13 (21.0%)	26 (22.8%)

Global Biostatistics: /by-sasp/patdb/projects/de04209/310442/stat/prod_interim03/pgms/t-admin-compl.sas epkll 07OCT2011 10:51
End of table

Table 14.1.2 / 4: Listing of specified 'other' reason for not completed first insertion attempt by treatment and subject (FAS)

TREATMENT	Subject number	IUS insertion failed, reason	IUS insertion failed, other reason
LCS12	140229	.	N/A
	161539	other	IUD WAS NOT IN SITU
	200223	other	PULLED OUT LCS WITH SISCORS
	240715	other	THE STRING WOULD NOT RELEASE FROM INSIDE THE INSERTER. THE END WOULD NOT DISENGAGE FROM THE INSERTER.
	240719	other	THE STRINGS WOULD NOT RELEASE OR SLIDE THROUGH THE INSERTER
	241015	other	NOT INSERTED DEEP ENOUGH
	241019	other	NOT FAR ENOUGH INTO UTERUS
	241531	other	IUD LOW ON SONOGRAM, THUS REMOVED AND REPLACED
	241536	other	PLACEMENT TOO LOW
	241707	other	SHARPLY ANTEVERTED UTERUS
	241721	other	IUS APPLICATOR BROKE DURING PLACEMENT
	243952	other	INSERTION PROVED TO BE DIFFICULT
	244417	other	SCISSORS CUT ONLY 1 OF 2 STRINGS, IUD PULLED OUT WHEN WITHDRAWN
	244422	other	PINK BOTTON DIDN'T CLICK AT THE END OF INSERTION
LCS16	150117	other	NOTE:THE IUS #12673 WAS INSERTED ONLY INTO THE CERVIX
	200405	IUS came out immediately after insertion	TOO LOW, THERE FORE REMOVED
	230105	other	THREADS SPONTANEOSLY GOT LOOSE
	240126	other	INSERTER INADVERTENTLY PULLED OUT THE IUS DUE TO THE STRING GOT CAUGHT IN THE HINGE OF THE SCISSORS.
	240404	other	1ST INSERTION ATTEMPTED, 1ST IUD BENT, SECOND ONE USED
	240602	other	ULTRASOUND OF FIRST ATTEMPT INDICATED THAT THE IUS WAS NOT LOCATED IN THE FUNDUS. DEVICE WAS REMOVED
	241535	other	SLIGHTLY TOO LOW
	242104	other	CERVICAL PLACEMENT
	242808	other	DURING ULTRASOUND - IUS TOO LOW IN CERVICAL CANAL
	243928	other	SLIDER MOVED BACK TO QUICKLY & TO FAR TO ALLOW PROPER INSERTION IN CAVITY
	244405	other	DISPLACED CERVICAL CANAL
	244449	other	PHYSICIAN NOT SATISFIED WITH PLACEMENT
	244916	other	ULTRASOUND SHOWED IUD DISPLACED
	246209	other	IUD WAS ACCIDENTLY PULLED OUT WHEN TRIMMING IUD STRING.

Global Biostatistics: /by-sasp/patdb/projects/de04209/310442/stat/prod_interim03/pgms/t-admin-compl.sas epkll 07OCT2011 10:51
End of table

Table 14.1.2 / 5: Number of successful second insertion by treatment (FAS)

	LCS12	LCS16	Total
Number of insertions	49 (100.0%)	57 (100.0%)	106 (100.0%)
IUS insertion completed			
no	3 (6.1%)	2 (3.5%)	5 (4.7%)
yes	46 (93.9%)	55 (96.5%)	101 (95.3%)

Global Biostatistics: /by-sasp/patdb/projects/de04209/310442/stat/prod_interim03/pgms/t-admin-compl.sas epkll 07OCT2011 10:51
End of table

Table 14.1.2 / 6: Reason for failure of second insertion by treatment (FAS)

	LCS12	LCS16	Total
Number of insertions	3 (100.0%)	2 (100.0%)	5 (100.0%)
IUS insertion failed, reason			
missing	1 (33.3%)	0	1 (20.0%)
cervix too tight	0	1 (50.0%)	1 (20.0%)
IUS came out immediately after insertion	1 (33.3%)	0	1 (20.0%)
malfunction of the inserter	0	1 (50.0%)	1 (20.0%)
small uterus	1 (33.3%)	0	1 (20.0%)

Global Biostatistics: /by-sasp/patdb/projects/de04209/310442/stat/prod_interim03/pgms/t-admin-compl.sas epkll 07OCT2011 10:51
End of table

Table 14.1.2 / 7: Listing of specified 'other' reason for not completed second insertion attempt by treatment and subject (FAS)

TREATMENT	Subject number	IUS insertion failed, reason	IUS insertion failed, other reason
.	.	.	

NO DATA AVAILABLE

Global Biostatistics: /by-sasp/patdb/projects/de04209/310442/stat/prod_interim03/pgms/t-admin-compl.sas epkll 07OCT2011 10:51
End of table

Table 14.1.2 / 8: Sound depth of the uterus by treatment (FAS)

		TREATMENT	n	Nmiss	Mean	SD	Min	Q1	Median	Q3	Max
Uterus depth	(cm)	LCS12	1426	6	7.27	0.89	2.0	7.00	7.00	8.00	11.0
		LCS16	1450	2	7.23	0.91	2.0	7.00	7.00	8.00	11.0
		Total	2876	8	7.25	0.90	2.0	7.00	7.00	8.00	11.0

Global Biostatistics: /by-sasp/patdb/projects/de04209/310442/stat/prod_interim03/pgms/t-admin-compl.sas epkll 07OCT2011 10:51
End of table

Table 14.1.2 / 9: Position of the uterus by treatment (FAS)

	LCS12	LCS16	Total
Number of insertions	1432 (100.0%)	1452 (100.0%)	2884 (100.0%)
Uterus position			
anteversion	959 (67.0%)	968 (66.7%)	1927 (66.8%)
mid position	248 (17.3%)	247 (17.0%)	495 (17.2%)
retroversion	221 (15.4%)	233 (16.0%)	454 (15.7%)
other	4 (0.3%)	4 (0.3%)	8 (0.3%)

Global Biostatistics: /by-sasp/patdb/projects/de04209/310442/stat/prod_interim03/pgms/t-admin-compl.sas epkll 07OCT2011 10:51
End of table

Table 14.1.2 / 10: Investigator's evaluation of IUS insertion procedure by treatment (FAS)

	LCS12	LCS16	Total
Number of insertions	1432 (100.0%)	1452 (100.0%)	2884 (100.0%)
IUS insertion, general assessment			
easy	1283 (89.6%)	1302 (89.7%)	2585 (89.6%)
slightly difficult	131 (9.1%)	132 (9.1%)	263 (9.1%)
very difficult	18 (1.3%)	18 (1.2%)	36 (1.2%)

Global Biostatistics: /by-sasp/patdb/projects/de04209/310442/stat/prod_interim03/pgms/t-admin-compl.sas epkll 07OCT2011 10:51
End of table

Table 14.1.2 / 11: Investigator's evaluation of IUS insertion procedure by dilatation performed (yes/no) and treatment (FAS)

	LCS12	LCS16	Total
Number of insertions	1432 (100.0%)	1452 (100.0%)	2884 (100.0%)
IUS insertion, dilatation used			
no	1355 (94.6%)	1370 (94.4%)	2725 (94.5%)
- of these: easy	1247 (92.0%)	1261 (92.0%)	2508 (92.0%)
slightly difficult	100 (7.4%)	101 (7.4%)	201 (7.4%)
very difficult	8 (0.6%)	8 (0.6%)	16 (0.6%)
yes	77 (5.4%)	82 (5.6%)	159 (5.5%)
- of these: easy	36 (46.8%)	41 (50.0%)	77 (48.4%)
slightly difficult	31 (40.3%)	31 (37.8%)	62 (39.0%)
very difficult	10 (13.0%)	10 (12.2%)	20 (12.6%)

Global Biostatistics: /by-sasp/patdb/projects/de04209/310442/stat/prod_interim03/pgms/t-admin-compl.sas epkll 07OCT2011 10:51
End of table

Table 14.1.2 / 12: Investigator's evaluation of IUS insertion procedure by parity (0 births vs. 1 or more) and treatment (FAS)

	LCS12	LCS16	Total
Number of subjects	1432 (100.0%)	1452 (100.0%)	2884 (100.0%)
Parity			
0 births	556 (38.8%)	574 (39.5%)	1130 (39.2%)
- of these: easy	468 (84.2%)	484 (84.3%)	952 (84.2%)
slightly difficult	76 (13.7%)	78 (13.6%)	154 (13.6%)
very difficult	12 (2.2%)	12 (2.1%)	24 (2.1%)
1 birth or more	876 (61.2%)	878 (60.5%)	1754 (60.8%)
- of these: easy	815 (93.0%)	818 (93.2%)	1633 (93.1%)
slightly difficult	55 (6.3%)	54 (6.2%)	109 (6.2%)
very difficult	6 (0.7%)	6 (0.7%)	12 (0.7%)

Global Biostatistics: /by-sasp/patdb/projects/de04209/310442/stat/prod_interim03/pgms/t-admin-compl.sas epkll 07OCT2011 10:51
End of table

Table 14.1.2 / 13: Subject's evaluation of pain during the IUS insertion procedure by treatment (FAS)

	LCS12	LCS16	Total
Number of insertions	1432 (100.0%)	1452 (100.0%)	2884 (100.0%)
IUS insertion, pain			
missing	0	1 (<0.1%)	1 (<0.1%)
none	295 (20.6%)	268 (18.5%)	563 (19.5%)
mild	629 (43.9%)	683 (47.0%)	1312 (45.5%)
moderate	390 (27.2%)	400 (27.5%)	790 (27.4%)
severe	118 (8.2%)	100 (6.9%)	218 (7.6%)

Global Biostatistics: /by-sasp/patdb/projects/de04209/310442/stat/prod_interim03/pgms/t-admin-compl.sas epkll 07OCT2011 10:51
End of table

Table 14.1.2 / 14: Subject's evaluation of pain during the IUS insertion procedure by dilatation performed (yes/no) and treatment (FAS)

	LCS12	LCS16	Total
Number of insertions	1432 (100.0%)	1452 (100.0%)	2884 (100.0%)
IUS insertion, dilatation used			
no	1355 (94.6%)	1370 (94.4%)	2725 (94.5%)
- of these: missing	0	1 (<0.1%)	1 (<0.1%)
none	291 (21.5%)	265 (19.3%)	556 (20.4%)
mild	605 (44.6%)	658 (48.0%)	1263 (46.3%)
moderate	353 (26.1%)	356 (26.0%)	709 (26.0%)
severe	106 (7.8%)	90 (6.6%)	196 (7.2%)
yes	77 (5.4%)	82 (5.6%)	159 (5.5%)
- of these: none	4 (5.2%)	3 (3.7%)	7 (4.4%)
mild	24 (31.2%)	25 (30.5%)	49 (30.8%)
moderate	37 (48.1%)	44 (53.7%)	81 (50.9%)
severe	12 (15.6%)	10 (12.2%)	22 (13.8%)

Global Biostatistics: /by-sasp/patdb/projects/de04209/310442/stat/prod_interim03/pgms/t-admin-compl.sas epkll 07OCT2011 10:51
End of table

Table 14.1.2 / 15: Subject's evaluation of pain during IUS insertion procedure by parity (0 births vs. 1 or more) and treatment (FAS)

	LCS12	LCS16	Total
Number of insertions	1432 (100.0%)	1452 (100.0%)	2884 (100.0%)
Parity			
0 births	556 (38.8%)	574 (39.5%)	1130 (39.2%)
- of these: missing	0	1 (0.2%)	1 (<0.1%)
none	40 (7.2%)	29 (5.1%)	69 (6.1%)
mild	178 (32.0%)	225 (39.2%)	403 (35.7%)
moderate	246 (44.2%)	236 (41.1%)	482 (42.7%)
severe	92 (16.5%)	83 (14.5%)	175 (15.5%)
1 birth or more	876 (61.2%)	878 (60.5%)	1754 (60.8%)
- of these: none	255 (29.1%)	239 (27.2%)	494 (28.2%)
mild	451 (51.5%)	458 (52.2%)	909 (51.8%)
moderate	144 (16.4%)	164 (18.7%)	308 (17.6%)
severe	26 (3.0%)	17 (1.9%)	43 (2.5%)

Global Biostatistics: /by-sasp/patdb/projects/de04209/310442/stat/prod_interim03/pgms/t-admin-compl.sas epkll 07OCT2011 10:51
End of table

Table 14.1.2 / 16: Number of subjects with dilatation performed including 'when' by treatment (FAS)

	LCS12	LCS16	Total
Number of insertions	1432 (100.0%)	1452 (100.0%)	2884 (100.0%)
IUS insertion, dilatation used			
no	1355 (94.6%)	1370 (94.4%)	2725 (94.5%)
missing	1355 (94.6%)	1369 (94.3%)	2724 (94.5%)
before procedure was performed	0	1 (<0.1%)	1 (<0.1%)
yes	77 (5.4%)	82 (5.6%)	159 (5.5%)
before procedure was performed	54 (3.8%)	51 (3.5%)	105 (3.6%)
when procedure proved to be difficult	22 (1.5%)	31 (2.1%)	53 (1.8%)
when procedure proved to be painful	1 (<0.1%)	0	1 (<0.1%)

Global Biostatistics: /by-sasp/patdb/projects/de04209/310442/stat/prod_interim03/pgms/t-admin-compl.sas epkll 07OCT2011 10:51
End of table

Table 14.1.2 / 17: Hegar size used by treatment (FAS)

	TREATMENT	n	Nmiss	Mean	SD	Min	Q1	Median	Q3	Max
Hegar size	LCS12 (N=1432)	40	37	5.1	3.0	1	3.0	4.0	5.0	14
	LCS16 (N=1452)	49	33	5.6	3.5	1	4.0	4.0	5.0	14
	Total (N=2884)	89	70	5.3	3.3	1	4.0	4.0	5.0	14

Global Biostatistics: /by-sasp/patdb/projects/de04209/310442/stat/prod_interim03/pgms/t-admin-compl.sas epkll 07OCT2011 10:51
End of table

Table 14.1.2 / 18: Number of subjects with dilatation performed including 'when' by parity and treatment (FAS)

Parity		LCS12	LCS16	Total
0 births	Number of insertions	556 (100.0%)	574 (100.0%)	1130 (100.0%)
	IUS insertion, dilatation used			
	no	505 (90.8%)	521 (90.8%)	1026 (90.8%)
	missing	505 (90.8%)	520 (90.6%)	1025 (90.7%)
	before procedure was performed	0	1 (0.2%)	1 (<0.1%)
	yes	51 (9.2%)	53 (9.2%)	104 (9.2%)
	before procedure was performed	36 (6.5%)	31 (5.4%)	67 (5.9%)
	when procedure proved to be difficult	14 (2.5%)	22 (3.8%)	36 (3.2%)
	when procedure proved to be painful	1 (0.2%)	0	1 (<0.1%)
1 birth or more	Number of insertions	876 (100.0%)	878 (100.0%)	1754 (100.0%)
	IUS insertion, dilatation used			
	no	850 (97.0%)	849 (96.7%)	1699 (96.9%)
	missing	850 (97.0%)	849 (96.7%)	1699 (96.9%)
	before procedure was performed	0	0	0
	yes	26 (3.0%)	29 (3.3%)	55 (3.1%)
	before procedure was performed	18 (2.1%)	20 (2.3%)	38 (2.2%)
	when procedure proved to be difficult	8 (0.9%)	9 (1.0%)	17 (1.0%)
	when procedure proved to be painful	0	0	0

Global Biostatistics: /by-sasp/patdb/projects/de04209/310442/stat/prod_interim03/pgms/t-admin-compl.sas epkll 07OCT2011 10:51
End of table

Table 14.1.2 / 19: Number of subjects with local anesthesia given including 'when' by treatment (FAS)

	LCS12	LCS16	Total
Number of subjects	1432 (100.0%)	1452 (100.0%)	2884 (100.0%)
Local anesthesia administered			
missing	1 (<0.1%)	0	1 (<0.1%)
no	1306 (91.2%)	1318 (90.8%)	2624 (91.0%)
yes	125 (8.7%)	134 (9.2%)	259 (9.0%)
before procedure was performed	120 (8.4%)	128 (8.8%)	248 (8.6%)
when procedure proved to be difficult	1 (<0.1%)	6 (0.4%)	7 (0.2%)
when procedure proved to be painful	4 (0.3%)	0	4 (0.1%)

Global Biostatistics: /by-sasp/patdb/projects/de04209/310442/stat/prod_interim03/pgms/t-admin-compl.sas epkll 07OCT2011 10:51
End of table

Table 14.1.2 / 20: Number of subjects with local anesthesia given including 'when' by parity and treatment (FAS)

Parity		LCS12	LCS16	Total
0 births	Number of subjects	556 (100.0%)	574 (100.0%)	1130 (100.0%)
	Local anesthesia administered			
	missing	0	0	0
	no	477 (85.8%)	480 (83.6%)	957 (84.7%)
	yes	79 (14.2%)	94 (16.4%)	173 (15.3%)
	before procedure was performed	75 (13.5%)	89 (15.5%)	164 (14.5%)
	when procedure proved to be difficult	1 (0.2%)	5 (0.9%)	6 (0.5%)
	when procedure proved to be painful	3 (0.5%)	0	3 (0.3%)
1 birth or more	Number of subjects	876 (100.0%)	878 (100.0%)	1754 (100.0%)
	Local anesthesia administered			
	missing	1 (0.1%)	0	1 (<0.1%)
	no	829 (94.6%)	838 (95.4%)	1667 (95.0%)
	yes	46 (5.3%)	40 (4.6%)	86 (4.9%)
	before procedure was performed	45 (5.1%)	39 (4.4%)	84 (4.8%)
	when procedure proved to be difficult	0	1 (0.1%)	1 (<0.1%)
	when procedure proved to be painful	1 (0.1%)	0	1 (<0.1%)

Global Biostatistics: /by-sasp/patdb/projects/de04209/310442/stat/prod_interim03/pgms/t-admin-compl.sas epkll 07OCT2011 10:51
End of table

Table 14.1.2 / 21: Number of subjects with analgesics given including 'when' by treatment (FAS)

	LCS12	LCS16	Total
Number of subjects	1432 (100.0%)	1452 (100.0%)	2884 (100.0%)
Analgetics administered			
no	934 (65.2%)	950 (65.4%)	1884 (65.3%)
yes	498 (34.8%)	502 (34.6%)	1000 (34.7%)
missing	1 (<0.1%)	0	1 (<0.1%)
before procedure was performed	463 (32.3%)	466 (32.1%)	929 (32.2%)
when procedure proved to be difficult	2 (0.1%)	2 (0.1%)	4 (0.1%)
when procedure proved to be painful	32 (2.2%)	34 (2.3%)	66 (2.3%)

Global Biostatistics: /by-sasp/patdb/projects/de04209/310442/stat/prod_interim03/pgms/t-admin-compl.sas epkll 07OCT2011 10:51
End of table

Table 14.1.2 / 22: Number of subjects with analgesics given including 'when' by parity and treatment (FAS)

Parity		LCS12	LCS16	Total
0 births	Number of subjects	556 (100.0%)	574 (100.0%)	1130 (100.0%)
	Analgetics administered			
	no	255 (45.9%)	272 (47.4%)	527 (46.6%)
	yes	301 (54.1%)	302 (52.6%)	603 (53.4%)
	missing	1 (0.2%)	0	1 (<0.1%)
	before procedure was performed	271 (48.7%)	278 (48.4%)	549 (48.6%)
	when procedure proved to be difficult	2 (0.4%)	2 (0.3%)	4 (0.4%)
	when procedure proved to be painful	27 (4.9%)	22 (3.8%)	49 (4.3%)
1 birth or more	Number of subjects	876 (100.0%)	878 (100.0%)	1754 (100.0%)
	Analgetics administered			
	no	679 (77.5%)	678 (77.2%)	1357 (77.4%)
	yes	197 (22.5%)	200 (22.8%)	397 (22.6%)
	missing	0	0	0
	before procedure was performed	192 (21.9%)	188 (21.4%)	380 (21.7%)
	when procedure proved to be difficult	0	0	0
	when procedure proved to be painful	5 (0.6%)	12 (1.4%)	17 (1.0%)

Global Biostatistics: /by-sasp/patdb/projects/de04209/310442/stat/prod_interim03/pgms/t-admin-compl.sas epkll 07OCT2011 10:51
End of table

Table 14.1.2 / 23: Number of subjects with other medication given including 'when' by treatment (FAS)

	LCS12	LCS16	Total
Number of subjects	1432 (100.0%)	1452 (100.0%)	2884 (100.0%)
Other medication administered			
no	1371 (95.7%)	1391 (95.8%)	2762 (95.8%)
yes	61 (4.3%)	61 (4.2%)	122 (4.2%)
before procedure was performed	48 (3.4%)	51 (3.5%)	99 (3.4%)
when procedure proved to be difficult	1 (<0.1%)	4 (0.3%)	5 (0.2%)
when procedure proved to be painful	12 (0.8%)	6 (0.4%)	18 (0.6%)

Global Biostatistics: /by-sasp/patdb/projects/de04209/310442/stat/prod_interim03/pgms/t-admin-compl.sas epkll 07OCT2011 10:51
End of table

Table 14.1.2 / 24: IUS detectable by ultrasound by time point and treatment (FAS)

Time key first level		LCS12	LCS16	Total
Baseline	Number of subjects	1428 (100.0%)	1447 (100.0%)	2875 (100.0%)
	IUS detectable by ultrasound			
	no	4 (0.3%)	1 (<0.1%)	5 (0.2%)
	yes	1424 (99.7%)	1446 (>99.9%)	2870 (99.8%)
Month 3	Number of subjects	1337 (100.0%)	1353 (100.0%)	2690 (100.0%)
	IUS detectable by ultrasound			
	no	3 (0.2%)	2 (0.1%)	5 (0.2%)
	yes	1334 (99.8%)	1351 (99.9%)	2685 (99.8%)
Month 6	Number of subjects	1235 (100.0%)	1278 (100.0%)	2513 (100.0%)
	IUS detectable by ultrasound			
	no	0	1 (<0.1%)	1 (<0.1%)
	yes	1235 (100.0%)	1277 (>99.9%)	2512 (>99.9%)
Month 9	Number of subjects	1174 (100.0%)	1215 (100.0%)	2389 (100.0%)
	IUS detectable by ultrasound			
	no	1 (<0.1%)	1 (<0.1%)	2 (<0.1%)
	yes	1173 (>99.9%)	1214 (>99.9%)	2387 (>99.9%)
Month 12	Number of subjects	1133 (100.0%)	1177 (100.0%)	2310 (100.0%)
	IUS detectable by ultrasound			
	no	1 (<0.1%)	0	1 (<0.1%)
	yes	1132 (>99.9%)	1177 (100.0%)	2309 (>99.9%)
Month 18	Number of subjects	1039 (100.0%)	1074 (100.0%)	2113 (100.0%)
	IUS detectable by ultrasound			
	no	1 (<0.1%)	0	1 (<0.1%)
	yes	1038 (>99.9%)	1074 (100.0%)	2112 (>99.9%)

Table 14.1.2 / 24: IUS detectable by ultrasound by time point and treatment (FAS)

Time key first level		LCS12	LCS16	Total
Month 24	Number of subjects	929 (100.0%)	984 (100.0%)	1913 (100.0%)
	IUS detectable by ultrasound			
	no	0	0	0
	yes	929 (100.0%)	984 (100.0%)	1913 (100.0%)
Month 30	Number of subjects	851 (100.0%)	901 (100.0%)	1752 (100.0%)
	IUS detectable by ultrasound			
	no	1 (0.1%)	1 (0.1%)	2 (0.1%)
	yes	850 (99.9%)	900 (99.9%)	1750 (99.9%)
End of study-Month 36	Number of subjects	1326 (100.0%)	1344 (100.0%)	2670 (100.0%)
	IUS detectable by ultrasound			
	no	49 (3.7%)	38 (2.8%)	87 (3.3%)
	yes	1277 (96.3%)	1306 (97.2%)	2583 (96.7%)

Global Biostatistics: /by-sasp/patdb/projects/de04209/310442/stat/prod_interim03/pgms/t-admin-compl.sas epkll 07OCT2011 10:51
End of table

Table 14.1.2 / 25: IUS location by ultrasound by time point and treatment (FAS)

Time key first level		LCS12	LCS16	Total
Baseline	Number of subjects	1429 (100.0%)	1448 (100.0%)	2877 (100.0%)
	IUS location			
	missing	3 (0.2%)	1 (<0.1%)	4 (0.1%)
	in situ	1415 (99.0%)	1437 (99.2%)	2852 (99.1%)
	displaced, cervical canal	0	4 (0.3%)	4 (0.1%)
	displaced, intrauterin	9 (0.6%)	5 (0.3%)	14 (0.5%)
	expelled in vagina	0	1 (<0.1%)	1 (<0.1%)
	myometrial perforation	0	0	0
	absent	1 (<0.1%)	0	1 (<0.1%)
	other	1 (<0.1%)	0	1 (<0.1%)
Month 3	Number of subjects	1341 (100.0%)	1355 (100.0%)	2696 (100.0%)
	IUS location			
	missing	5 (0.4%)	2 (0.1%)	7 (0.3%)
	in situ	1324 (98.7%)	1336 (98.6%)	2660 (98.7%)
	displaced, cervical canal	2 (0.1%)	6 (0.4%)	8 (0.3%)
	displaced, intrauterin	8 (0.6%)	9 (0.7%)	17 (0.6%)
	expelled in vagina	0	0	0
	myometrial perforation	0	0	0
	absent	2 (0.1%)	1 (<0.1%)	3 (0.1%)
	other	0	1 (<0.1%)	1 (<0.1%)
Month 6	Number of subjects	1239 (100.0%)	1284 (100.0%)	2523 (100.0%)
	IUS location			
	missing	4 (0.3%)	6 (0.5%)	10 (0.4%)
	in situ	1225 (98.9%)	1259 (98.1%)	2484 (98.5%)
	displaced, cervical canal	3 (0.2%)	7 (0.5%)	10 (0.4%)
	displaced, intrauterin	7 (0.6%)	11 (0.9%)	18 (0.7%)
	expelled in vagina	0	0	0
	myometrial perforation	0	0	0
	absent	0	1 (<0.1%)	1 (<0.1%)
	other	0	0	0

Table 14.1.2 / 25: IUS location by ultrasound by time point and treatment (FAS)

Time key first level		LCS12	LCS16	Total
Month 9	Number of subjects	1176 (100.0%)	1219 (100.0%)	2395 (100.0%)
	IUS location			
	missing	3 (0.3%)	5 (0.4%)	8 (0.3%)
	in situ	1158 (98.5%)	1196 (98.1%)	2354 (98.3%)
	displaced, cervical canal	6 (0.5%)	3 (0.2%)	9 (0.4%)
	displaced, intrauterin	8 (0.7%)	15 (1.2%)	23 (1.0%)
	expelled in vagina	0	0	0
	myometrial perforation	0	0	0
	absent	0	0	0
	other	1 (<0.1%)	0	1 (<0.1%)
Month 12	Number of subjects	1135 (100.0%)	1179 (100.0%)	2314 (100.0%)
	IUS location			
	missing	3 (0.3%)	2 (0.2%)	5 (0.2%)
	in situ	1126 (99.2%)	1161 (98.5%)	2287 (98.8%)
	displaced, cervical canal	2 (0.2%)	1 (<0.1%)	3 (0.1%)
	displaced, intrauterin	4 (0.4%)	14 (1.2%)	18 (0.8%)
	expelled in vagina	0	0	0
	myometrial perforation	0	0	0
	absent	0	0	0
	other	0	1 (<0.1%)	1 (<0.1%)
Month 18	Number of subjects	1042 (100.0%)	1078 (100.0%)	2120 (100.0%)
	IUS location			
	missing	3 (0.3%)	4 (0.4%)	7 (0.3%)
	in situ	1031 (98.9%)	1066 (98.9%)	2097 (98.9%)
	displaced, cervical canal	1 (<0.1%)	1 (<0.1%)	2 (<0.1%)
	displaced, intrauterin	6 (0.6%)	7 (0.6%)	13 (0.6%)
	expelled in vagina	0	0	0
	myometrial perforation	0	0	0
	absent	1 (<0.1%)	0	1 (<0.1%)
	other	0	0	0

Table 14.1.2 / 25: IUS location by ultrasound by time point and treatment (FAS)

Time key first level		LCS12	LCS16	Total
Month 24	Number of subjects	933 (100.0%)	986 (100.0%)	1919 (100.0%)
	IUS location			
	missing	4 (0.4%)	2 (0.2%)	6 (0.3%)
	in situ	925 (99.1%)	974 (98.8%)	1899 (99.0%)
	displaced, cervical canal	1 (0.1%)	1 (0.1%)	2 (0.1%)
	displaced, intrauterin	3 (0.3%)	7 (0.7%)	10 (0.5%)
	expelled in vagina	0	0	0
	myometrial perforation	0	1 (0.1%)	1 (<0.1%)
	absent	0	0	0
	other	0	1 (0.1%)	1 (<0.1%)
Month 30	Number of subjects	851 (100.0%)	901 (100.0%)	1752 (100.0%)
	IUS location			
	missing	0	0	0
	in situ	846 (99.4%)	894 (99.2%)	1740 (99.3%)
	displaced, cervical canal	1 (0.1%)	0	1 (<0.1%)
	displaced, intrauterin	3 (0.4%)	7 (0.8%)	10 (0.6%)
	expelled in vagina	0	0	0
	myometrial perforation	0	0	0
	absent	1 (0.1%)	0	1 (<0.1%)
	other	0	0	0
End of study-Month 36	Number of subjects	1357 (100.0%)	1379 (100.0%)	2736 (100.0%)
	IUS location			
	missing	33 (2.4%)	37 (2.7%)	70 (2.6%)
	in situ	1243 (91.6%)	1261 (91.4%)	2504 (91.5%)
	displaced, cervical canal	19 (1.4%)	23 (1.7%)	42 (1.5%)
	displaced, intrauterin	12 (0.9%)	17 (1.2%)	29 (1.1%)
	expelled in vagina	1 (<0.1%)	4 (0.3%)	5 (0.2%)
	myometrial perforation	0	1 (<0.1%)	1 (<0.1%)
	absent	24 (1.8%)	15 (1.1%)	39 (1.4%)
	other	25 (1.8%)	21 (1.5%)	46 (1.7%)

Global Biostatistics: /by-sasp/patdb/projects/de04209/310442/stat/prod_interim03/pgms/t-admin-compl.sas epkll 07OCT2011 10:51
End of table

Table 14.1.2 / 26: IUS location by ultrasound by time point, parity status and treatment (FAS)

Time key first level	Parity		LCS12	LCS16	Total
Baseline	0 births	Number of subjects	553 (100.0%)	571 (100.0%)	1124 (100.0%)
		IUS location			
		missing	2 (0.4%)	1 (0.2%)	3 (0.3%)
		in situ	545 (98.6%)	566 (99.1%)	1111 (98.8%)
		displaced, cervical canal	0	0	0
		displaced, intrauterin	4 (0.7%)	3 (0.5%)	7 (0.6%)
		expelled in vagina	0	1 (0.2%)	1 (<0.1%)
		myometrial perforation	0	0	0
		absent	1 (0.2%)	0	1 (<0.1%)
		other	1 (0.2%)	0	1 (<0.1%)
	1 birth or more	Number of subjects	876 (100.0%)	877 (100.0%)	1753 (100.0%)
		IUS location			
		missing	1 (0.1%)	0	1 (<0.1%)
		in situ	870 (99.3%)	871 (99.3%)	1741 (99.3%)
		displaced, cervical canal	0	4 (0.5%)	4 (0.2%)
		displaced, intrauterin	5 (0.6%)	2 (0.2%)	7 (0.4%)
		expelled in vagina	0	0	0
		myometrial perforation	0	0	0
		absent	0	0	0
		other	0	0	0
Month 3	0 births	Number of subjects	514 (100.0%)	525 (100.0%)	1039 (100.0%)
		IUS location			
		missing	2 (0.4%)	1 (0.2%)	3 (0.3%)
		in situ	509 (99.0%)	519 (98.9%)	1028 (98.9%)
		displaced, cervical canal	0	1 (0.2%)	1 (<0.1%)
		displaced, intrauterin	3 (0.6%)	3 (0.6%)	6 (0.6%)
		expelled in vagina	0	0	0
		myometrial perforation	0	0	0
		absent	0	0	0
		other	0	1 (0.2%)	1 (<0.1%)

Table 14.1.2 / 26: IUS location by ultrasound by time point, parity status and treatment (FAS)

Time key first level	Parity		LCS12	LCS16	Total
	1 birth or more	Number of subjects	827 (100.0%)	830 (100.0%)	1657 (100.0%)
		IUS location			
		missing	3 (0.4%)	1 (0.1%)	4 (0.2%)
		in situ	815 (98.5%)	817 (98.4%)	1632 (98.5%)
		displaced, cervical canal	2 (0.2%)	5 (0.6%)	7 (0.4%)
		displaced, intrauterin	5 (0.6%)	6 (0.7%)	11 (0.7%)
		expelled in vagina	0	0	0
		myometrial perforation	0	0	0
		absent	2 (0.2%)	1 (0.1%)	3 (0.2%)
		other	0	0	0
Month 6	0 births	Number of subjects	469 (100.0%)	498 (100.0%)	967 (100.0%)
		IUS location			
		missing	1 (0.2%)	2 (0.4%)	3 (0.3%)
		in situ	462 (98.5%)	490 (98.4%)	952 (98.4%)
		displaced, cervical canal	1 (0.2%)	1 (0.2%)	2 (0.2%)
		displaced, intrauterin	5 (1.1%)	4 (0.8%)	9 (0.9%)
		expelled in vagina	0	0	0
		myometrial perforation	0	0	0
		absent	0	1 (0.2%)	1 (0.1%)
		other	0	0	0
	1 birth or more	Number of subjects	770 (100.0%)	786 (100.0%)	1556 (100.0%)
		IUS location			
		missing	3 (0.4%)	4 (0.5%)	7 (0.4%)
		in situ	763 (99.1%)	769 (97.8%)	1532 (98.5%)
		displaced, cervical canal	2 (0.3%)	6 (0.8%)	8 (0.5%)
		displaced, intrauterin	2 (0.3%)	7 (0.9%)	9 (0.6%)
		expelled in vagina	0	0	0
		myometrial perforation	0	0	0
		absent	0	0	0
		other	0	0	0

Table 14.1.2 / 26: IUS location by ultrasound by time point, parity status and treatment (FAS)

Time key first level	Parity		LCS12	LCS16	Total
Month 9	0 births	Number of subjects	442 (100.0%)	466 (100.0%)	908 (100.0%)
		IUS location			
		missing	2 (0.5%)	1 (0.2%)	3 (0.3%)
		in situ	433 (98.0%)	458 (98.3%)	891 (98.1%)
		displaced, cervical canal	4 (0.9%)	1 (0.2%)	5 (0.6%)
		displaced, intrauterin	3 (0.7%)	6 (1.3%)	9 (1.0%)
		expelled in vagina	0	0	0
		myometrial perforation	0	0	0
		absent	0	0	0
		other	0	0	0
	1 birth or more	Number of subjects	734 (100.0%)	753 (100.0%)	1487 (100.0%)
		IUS location			
		missing	1 (0.1%)	4 (0.5%)	5 (0.3%)
		in situ	725 (98.8%)	738 (98.0%)	1463 (98.4%)
		displaced, cervical canal	2 (0.3%)	2 (0.3%)	4 (0.3%)
		displaced, intrauterin	5 (0.7%)	9 (1.2%)	14 (0.9%)
		expelled in vagina	0	0	0
		myometrial perforation	0	0	0
		absent	0	0	0
		other	1 (0.1%)	0	1 (<0.1%)
Month 12	0 births	Number of subjects	422 (100.0%)	452 (100.0%)	874 (100.0%)
		IUS location			
		missing	1 (0.2%)	0	1 (0.1%)
		in situ	419 (99.3%)	446 (98.7%)	865 (99.0%)
		displaced, cervical canal	1 (0.2%)	1 (0.2%)	2 (0.2%)
		displaced, intrauterin	1 (0.2%)	4 (0.9%)	5 (0.6%)
		expelled in vagina	0	0	0
		myometrial perforation	0	0	0
		absent	0	0	0
		other	0	1 (0.2%)	1 (0.1%)

Table 14.1.2 / 26: IUS location by ultrasound by time point, parity status and treatment (FAS)

Time key first level	Parity		LCS12	LCS16	Total
	1 birth or more	Number of subjects	713 (100.0%)	727 (100.0%)	1440 (100.0%)
		IUS location			
		missing	2 (0.3%)	2 (0.3%)	4 (0.3%)
		in situ	707 (99.2%)	715 (98.3%)	1422 (98.8%)
		displaced, cervical canal	1 (0.1%)	0	1 (<0.1%)
		displaced, intrauterin	3 (0.4%)	10 (1.4%)	13 (0.9%)
		expelled in vagina	0	0	0
		myometrial perforation	0	0	0
		absent	0	0	0
		other	0	0	0
Month 18	0 births	Number of subjects	387 (100.0%)	407 (100.0%)	794 (100.0%)
		IUS location			
		missing	2 (0.5%)	0	2 (0.3%)
		in situ	380 (98.2%)	403 (99.0%)	783 (98.6%)
		displaced, cervical canal	1 (0.3%)	1 (0.2%)	2 (0.3%)
		displaced, intrauterin	3 (0.8%)	3 (0.7%)	6 (0.8%)
		expelled in vagina	0	0	0
		myometrial perforation	0	0	0
		absent	1 (0.3%)	0	1 (0.1%)
		other	0	0	0
	1 birth or more	Number of subjects	655 (100.0%)	671 (100.0%)	1326 (100.0%)
		IUS location			
		missing	1 (0.2%)	4 (0.6%)	5 (0.4%)
		in situ	651 (99.4%)	663 (98.8%)	1314 (99.1%)
		displaced, cervical canal	0	0	0
		displaced, intrauterin	3 (0.5%)	4 (0.6%)	7 (0.5%)
		expelled in vagina	0	0	0
		myometrial perforation	0	0	0
		absent	0	0	0
		other	0	0	0

Table 14.1.2 / 26: IUS location by ultrasound by time point, parity status and treatment (FAS)

Time key first level	Parity		LCS12	LCS16	Total
Month 24	0 births	Number of subjects	342 (100.0%)	373 (100.0%)	715 (100.0%)
		IUS location			
		missing	1 (0.3%)	0	1 (0.1%)
		in situ	341 (99.7%)	369 (98.9%)	710 (99.3%)
		displaced, cervical canal	0	0	0
		displaced, intrauterin	0	3 (0.8%)	3 (0.4%)
		expelled in vagina	0	0	0
		myometrial perforation	0	1 (0.3%)	1 (0.1%)
		absent	0	0	0
		other	0	0	0
	1 birth or more	Number of subjects	591 (100.0%)	613 (100.0%)	1204 (100.0%)
		IUS location			
		missing	3 (0.5%)	2 (0.3%)	5 (0.4%)
		in situ	584 (98.8%)	605 (98.7%)	1189 (98.8%)
		displaced, cervical canal	1 (0.2%)	1 (0.2%)	2 (0.2%)
		displaced, intrauterin	3 (0.5%)	4 (0.7%)	7 (0.6%)
		expelled in vagina	0	0	0
		myometrial perforation	0	0	0
		absent	0	0	0
		other	0	1 (0.2%)	1 (<0.1%)
Month 30	0 births	Number of subjects	318 (100.0%)	341 (100.0%)	659 (100.0%)
		IUS location			
		missing	0	0	0
		in situ	317 (99.7%)	339 (99.4%)	656 (99.5%)
		displaced, cervical canal	0	0	0
		displaced, intrauterin	0	2 (0.6%)	2 (0.3%)
		expelled in vagina	0	0	0
		myometrial perforation	0	0	0
		absent	1 (0.3%)	0	1 (0.2%)
		other	0	0	0

Table 14.1.2 / 26: IUS location by ultrasound by time point, parity status and treatment (FAS)

Time key first level	Parity		LCS12	LCS16	Total
	1 birth or more	Number of subjects	533 (100.0%)	560 (100.0%)	1093 (100.0%)
		IUS location			
		missing	0	0	0
		in situ	529 (99.2%)	555 (99.1%)	1084 (99.2%)
		displaced, cervical canal	1 (0.2%)	0	1 (<0.1%)
		displaced, intrauterin	3 (0.6%)	5 (0.9%)	8 (0.7%)
		expelled in vagina	0	0	0
		myometrial perforation	0	0	0
		absent	0	0	0
		other	0	0	0
End of study-Month 36	0 births	Number of subjects	536 (100.0%)	545 (100.0%)	1081 (100.0%)
		IUS location			
		missing	11 (2.1%)	20 (3.7%)	31 (2.9%)
		in situ	496 (92.5%)	497 (91.2%)	993 (91.9%)
		displaced, cervical canal	9 (1.7%)	4 (0.7%)	13 (1.2%)
		displaced, intrauterin	4 (0.7%)	8 (1.5%)	12 (1.1%)
		expelled in vagina	0	1 (0.2%)	1 (<0.1%)
		myometrial perforation	0	1 (0.2%)	1 (<0.1%)
		absent	4 (0.7%)	3 (0.6%)	7 (0.6%)
		other	12 (2.2%)	11 (2.0%)	23 (2.1%)
	1 birth or more	Number of subjects	821 (100.0%)	834 (100.0%)	1655 (100.0%)
		IUS location			
		missing	22 (2.7%)	17 (2.0%)	39 (2.4%)
		in situ	747 (91.0%)	764 (91.6%)	1511 (91.3%)
		displaced, cervical canal	10 (1.2%)	19 (2.3%)	29 (1.8%)
		displaced, intrauterin	8 (1.0%)	9 (1.1%)	17 (1.0%)
		expelled in vagina	1 (0.1%)	3 (0.4%)	4 (0.2%)
		myometrial perforation	0	0	0
		absent	20 (2.4%)	12 (1.4%)	32 (1.9%)
		other	13 (1.6%)	10 (1.2%)	23 (1.4%)

Global Biostatistics: /by-sasp/patdb/projects/de04209/310442/stat/prod_interim03/pgms/t-admin-compl.sas epkll 07OCT2011 10:51
End of table

Table 14.1.2 / 27: IUS location by ultrasound (compliant vs. non-compliant) by time point and treatment (FAS)

Time key first level		LCS12	LCS16	Total
Baseline	Number of subjects	1429 (100.0%)	1448 (100.0%)	2877 (100.0%)
	Compliance			
	missing	3 (0.2%)	1 (<0.1%)	4 (0.1%)
	compliant	1424 (99.7%)	1442 (99.6%)	2866 (99.6%)
	not compliant	2 (0.1%)	5 (0.3%)	7 (0.2%)
Month 3	Number of subjects	1341 (100.0%)	1355 (100.0%)	2696 (100.0%)
	Compliance			
	missing	5 (0.4%)	2 (0.1%)	7 (0.3%)
	compliant	1332 (99.3%)	1345 (99.3%)	2677 (99.3%)
	not compliant	4 (0.3%)	8 (0.6%)	12 (0.4%)
Month 6	Number of subjects	1239 (100.0%)	1284 (100.0%)	2523 (100.0%)
	Compliance			
	missing	4 (0.3%)	6 (0.5%)	10 (0.4%)
	compliant	1232 (99.4%)	1270 (98.9%)	2502 (99.2%)
	not compliant	3 (0.2%)	8 (0.6%)	11 (0.4%)
Month 9	Number of subjects	1176 (100.0%)	1219 (100.0%)	2395 (100.0%)
	Compliance			
	missing	3 (0.3%)	5 (0.4%)	8 (0.3%)
	compliant	1166 (99.1%)	1211 (99.3%)	2377 (99.2%)
	not compliant	7 (0.6%)	3 (0.2%)	10 (0.4%)
Month 12	Number of subjects	1135 (100.0%)	1179 (100.0%)	2314 (100.0%)
	Compliance			
	missing	3 (0.3%)	2 (0.2%)	5 (0.2%)
	compliant	1130 (99.6%)	1175 (99.7%)	2305 (99.6%)
	not compliant	2 (0.2%)	2 (0.2%)	4 (0.2%)
Month 18	Number of subjects	1042 (100.0%)	1078 (100.0%)	2120 (100.0%)
	Compliance			
	missing	3 (0.3%)	4 (0.4%)	7 (0.3%)
	compliant	1037 (99.5%)	1073 (99.5%)	2110 (99.5%)
	not compliant	2 (0.2%)	1 (<0.1%)	3 (0.1%)

Table 14.1.2 / 27: IUS location by ultrasound (compliant vs. non-compliant) by time point and treatment (FAS)

Time key first level		LCS12	LCS16	Total
Month 24	Number of subjects	933 (100.0%)	986 (100.0%)	1919 (100.0%)
	Compliance			
	missing	4 (0.4%)	2 (0.2%)	6 (0.3%)
	compliant	928 (99.5%)	981 (99.5%)	1909 (99.5%)
	not compliant	1 (0.1%)	3 (0.3%)	4 (0.2%)
Month 30	Number of subjects	851 (100.0%)	901 (100.0%)	1752 (100.0%)
	Compliance			
	missing	0	0	0
	compliant	849 (99.8%)	901 (100.0%)	1750 (99.9%)
	not compliant	2 (0.2%)	0	2 (0.1%)
End of study-Month 36	Number of subjects	1357 (100.0%)	1379 (100.0%)	2736 (100.0%)
	Compliance			
	missing	33 (2.4%)	37 (2.7%)	70 (2.6%)
	compliant	1255 (92.5%)	1278 (92.7%)	2533 (92.6%)
	not compliant	69 (5.1%)	64 (4.6%)	133 (4.9%)

Note: Compliant: location of the IUS in situ or displaced intrauterine.
All other categories counted as not compliant.
Global Biostatistics: /by-sasp/patdb/projects/de04209/310442/stat/prod_interim03/pgms/t-admin-compl.sas epkll 07OCT2011 10:51
End of table

Table 14.1.2 / 28: IUS location by ultrasound (compliant vs. non-compliant) by time point, parity status and treatment (FAS)

Time key first level		LCS12	LCS16	Total
Baseline	Number of subjects	1429 (100.0%)	1448 (100.0%)	2877 (100.0%)
	Parity			
	0 births	553 (38.7%)	571 (39.4%)	1124 (39.1%)
	missing	2 (0.1%)	1 (<0.1%)	3 (0.1%)
	compliant	549 (38.4%)	569 (39.3%)	1118 (38.9%)
	not compliant	2 (0.1%)	1 (<0.1%)	3 (0.1%)
	1 birth or more	876 (61.3%)	877 (60.6%)	1753 (60.9%)
	missing	1 (<0.1%)	0	1 (<0.1%)
	compliant	875 (61.2%)	873 (60.3%)	1748 (60.8%)
	not compliant	0	4 (0.3%)	4 (0.1%)
Month 3	Number of subjects	1341 (100.0%)	1355 (100.0%)	2696 (100.0%)
	Parity			
	0 births	514 (38.3%)	525 (38.7%)	1039 (38.5%)
	missing	2 (0.1%)	1 (<0.1%)	3 (0.1%)
	compliant	512 (38.2%)	522 (38.5%)	1034 (38.4%)
	not compliant	0	2 (0.1%)	2 (<0.1%)
	1 birth or more	827 (61.7%)	830 (61.3%)	1657 (61.5%)
	missing	3 (0.2%)	1 (<0.1%)	4 (0.1%)
	compliant	820 (61.1%)	823 (60.7%)	1643 (60.9%)
	not compliant	4 (0.3%)	6 (0.4%)	10 (0.4%)
Month 6	Number of subjects	1239 (100.0%)	1284 (100.0%)	2523 (100.0%)
	Parity			
	0 births	469 (37.9%)	498 (38.8%)	967 (38.3%)
	missing	1 (<0.1%)	2 (0.2%)	3 (0.1%)
	compliant	467 (37.7%)	494 (38.5%)	961 (38.1%)
	not compliant	1 (<0.1%)	2 (0.2%)	3 (0.1%)
	1 birth or more	770 (62.1%)	786 (61.2%)	1556 (61.7%)
	missing	3 (0.2%)	4 (0.3%)	7 (0.3%)
	compliant	765 (61.7%)	776 (60.4%)	1541 (61.1%)
	not compliant	2 (0.2%)	6 (0.5%)	8 (0.3%)

Table 14.1.2 / 28: IUS location by ultrasound (compliant vs. non-compliant) by time point, parity status and treatment (FAS)

Time key first level		LCS12	LCS16	Total
Month 9	Number of subjects	1176 (100.0%)	1219 (100.0%)	2395 (100.0%)
	Parity			
	0 births	442 (37.6%)	466 (38.2%)	908 (37.9%)
	missing	2 (0.2%)	1 (<0.1%)	3 (0.1%)
	compliant	436 (37.1%)	464 (38.1%)	900 (37.6%)
	not compliant	4 (0.3%)	1 (<0.1%)	5 (0.2%)
	1 birth or more	734 (62.4%)	753 (61.8%)	1487 (62.1%)
	missing	1 (<0.1%)	4 (0.3%)	5 (0.2%)
	compliant	730 (62.1%)	747 (61.3%)	1477 (61.7%)
	not compliant	3 (0.3%)	2 (0.2%)	5 (0.2%)
Month 12	Number of subjects	1135 (100.0%)	1179 (100.0%)	2314 (100.0%)
	Parity			
	0 births	422 (37.2%)	452 (38.3%)	874 (37.8%)
	missing	1 (<0.1%)	0	1 (<0.1%)
	compliant	420 (37.0%)	450 (38.2%)	870 (37.6%)
	not compliant	1 (<0.1%)	2 (0.2%)	3 (0.1%)
	1 birth or more	713 (62.8%)	727 (61.7%)	1440 (62.2%)
	missing	2 (0.2%)	2 (0.2%)	4 (0.2%)
	compliant	710 (62.6%)	725 (61.5%)	1435 (62.0%)
	not compliant	1 (<0.1%)	0	1 (<0.1%)
Month 18	Number of subjects	1042 (100.0%)	1078 (100.0%)	2120 (100.0%)
	Parity			
	0 births	387 (37.1%)	407 (37.8%)	794 (37.5%)
	missing	2 (0.2%)	0	2 (<0.1%)
	compliant	383 (36.8%)	406 (37.7%)	789 (37.2%)
	not compliant	2 (0.2%)	1 (<0.1%)	3 (0.1%)
	1 birth or more	655 (62.9%)	671 (62.2%)	1326 (62.5%)
	missing	1 (<0.1%)	4 (0.4%)	5 (0.2%)
	compliant	654 (62.8%)	667 (61.9%)	1321 (62.3%)
	not compliant	0	0	0

Table 14.1.2 / 28: IUS location by ultrasound (compliant vs. non-compliant) by time point, parity status and treatment (FAS)

Time key first level		LCS12	LCS16	Total
Month 24	Number of subjects	933 (100.0%)	986 (100.0%)	1919 (100.0%)
	Parity			
	0 births	342 (36.7%)	373 (37.8%)	715 (37.3%)
	missing	1 (0.1%)	0	1 (<0.1%)
	compliant	341 (36.5%)	372 (37.7%)	713 (37.2%)
	not compliant	0	1 (0.1%)	1 (<0.1%)
	1 birth or more	591 (63.3%)	613 (62.2%)	1204 (62.7%)
	missing	3 (0.3%)	2 (0.2%)	5 (0.3%)
	compliant	587 (62.9%)	609 (61.8%)	1196 (62.3%)
	not compliant	1 (0.1%)	2 (0.2%)	3 (0.2%)
Month 30	Number of subjects	851 (100.0%)	901 (100.0%)	1752 (100.0%)
	Parity			
	0 births	318 (37.4%)	341 (37.8%)	659 (37.6%)
	missing	0	0	0
	compliant	317 (37.3%)	341 (37.8%)	658 (37.6%)
	not compliant	1 (0.1%)	0	1 (<0.1%)
	1 birth or more	533 (62.6%)	560 (62.2%)	1093 (62.4%)
	missing	0	0	0
	compliant	532 (62.5%)	560 (62.2%)	1092 (62.3%)
	not compliant	1 (0.1%)	0	1 (<0.1%)
End of study-Month 36	Number of subjects	1357 (100.0%)	1379 (100.0%)	2736 (100.0%)
	Parity			
	0 births	536 (39.5%)	545 (39.5%)	1081 (39.5%)
	missing	11 (0.8%)	20 (1.5%)	31 (1.1%)
	compliant	500 (36.8%)	505 (36.6%)	1005 (36.7%)
	not compliant	25 (1.8%)	20 (1.5%)	45 (1.6%)
	1 birth or more	821 (60.5%)	834 (60.5%)	1655 (60.5%)
	missing	22 (1.6%)	17 (1.2%)	39 (1.4%)
	compliant	755 (55.6%)	773 (56.1%)	1528 (55.8%)
	not compliant	44 (3.2%)	44 (3.2%)	88 (3.2%)

Note: Compliant: location of the IUS in situ or displaced intrauterine.
All other categories counted as not compliant.
Global Biostatistics: /by-sasp/patdb/projects/de04209/310442/stat/prod_interim03/pgms/t-admin-compl.sas epkll 07OCT2011 10:51
End of table

Table 14.1.2 / 29: Investigators evaluation of IUS removal procedure by treatment (FAS)

	LCS12	LCS16	Total
Number of subjects	1333 (100.0%)	660 (100.0%)	1993 (100.0%)
IUS removal, general assessment			
missing	16 (1.2%)	27 (4.1%)	43 (2.2%)
easy	1211 (90.8%)	588 (89.1%)	1799 (90.3%)
slightly difficult	82 (6.2%)	33 (5.0%)	115 (5.8%)
very difficult	24 (1.8%)	12 (1.8%)	36 (1.8%)

Note: Only subjects with documented removal of IUS
Global Biostatistics: /by-sasp/patdb/projects/de04209/310442/stat/prod_interim03/pgms/t-admin-compl.sas epkll 07OCT2011 10:51
End of table

Table 14.1.2 / 30: Investigators evaluation of IUS removal procedure by parity (0 births vs. 1 or more) and treatment (FAS)

	LCS12	LCS16	Total
Number of subjects	1333 (100.0%)	660 (100.0%)	1993 (100.0%)
Parity			
0 births	534 (40.1%)	283 (42.9%)	817 (41.0%)
- of these: missing	3 (0.6%)	10 (3.5%)	13 (1.6%)
easy	495 (92.7%)	251 (88.7%)	746 (91.3%)
slightly difficult	29 (5.4%)	18 (6.4%)	47 (5.8%)
very difficult	7 (1.3%)	4 (1.4%)	11 (1.3%)
1 birth or more	799 (59.9%)	377 (57.1%)	1176 (59.0%)
- of these: missing	13 (1.6%)	17 (4.5%)	30 (2.6%)
easy	716 (89.6%)	337 (89.4%)	1053 (89.5%)
slightly difficult	53 (6.6%)	15 (4.0%)	68 (5.8%)
very difficult	17 (2.1%)	8 (2.1%)	25 (2.1%)

Note: Only subjects with documented removal of IUS
Global Biostatistics: /by-sasp/patdb/projects/de04209/310442/stat/prod_interim03/pgms/t-admin-compl.sas epkll 07OCT2011 10:51
End of table

Table 14.1.2 / 31: Subjects evaluation of pain during IUS removal procedure by treatment (FAS)

	LCS12	LCS16	Total
Number of subjects	1333 (100.0%)	660 (100.0%)	1993 (100.0%)
IUS removal, pain			
missing	10 (0.8%)	21 (3.2%)	31 (1.6%)
none	460 (34.5%)	260 (39.4%)	720 (36.1%)
mild	634 (47.6%)	281 (42.6%)	915 (45.9%)
moderate	185 (13.9%)	82 (12.4%)	267 (13.4%)
severe	44 (3.3%)	16 (2.4%)	60 (3.0%)

Note: Only subjects with documented removal of IUS
Global Biostatistics: /by-sasp/patdb/projects/de04209/310442/stat/prod_interim03/pgms/t-admin-compl.sas epkll 07OCT2011 10:51
End of table

Table 14.1.2 / 32: Subjects evaluation of pain during IUS removal procedure by parity (0 births vs. 1 or more) and treatment (FAS)

	LCS12	LCS16	Total
Number of subjects	1333 (100.0%)	660 (100.0%)	1993 (100.0%)
Parity			
0 births	534 (40.1%)	283 (42.9%)	817 (41.0%)
- of these: missing	2 (0.4%)	9 (3.2%)	11 (1.3%)
none	95 (17.8%)	65 (23.0%)	160 (19.6%)
mild	294 (55.1%)	148 (52.3%)	442 (54.1%)
moderate	116 (21.7%)	51 (18.0%)	167 (20.4%)
severe	27 (5.1%)	10 (3.5%)	37 (4.5%)
1 birth or more	799 (59.9%)	377 (57.1%)	1176 (59.0%)
- of these: missing	8 (1.0%)	12 (3.2%)	20 (1.7%)
none	365 (45.7%)	195 (51.7%)	560 (47.6%)
mild	340 (42.6%)	133 (35.3%)	473 (40.2%)
moderate	69 (8.6%)	31 (8.2%)	100 (8.5%)
severe	17 (2.1%)	6 (1.6%)	23 (2.0%)

Note: Only subjects with documented removal of IUS
Global Biostatistics: /by-sasp/patdb/projects/de04209/310442/stat/prod_interim03/pgms/t-admin-compl.sas epkll 07OCT2011 10:51
End of table

Table 14.1.2 / 33: Number of subjects with expulsions (FAS)

	LCS12 (N=1432)	LCS16 (N=1452)	Total (N=2884)
Number of Subjects	1426 (100.0%)	1445 (100.0%)	2871 (100.0%)
IUS partially expelled			
no	1402 (98.3%)	1415 (97.9%)	2817 (98.1%)
yes	24 (1.7%)	30 (2.1%)	54 (1.9%)
IUS totally expelled			
no	1397 (98.0%)	1429 (98.9%)	2826 (98.4%)
yes	29 (2.0%)	16 (1.1%)	45 (1.6%)
Partial or total expulsion			
no	1373 (96.3%)	1399 (96.8%)	2772 (96.6%)
yes	53 (3.7%)	46 (3.2%)	99 (3.4%)

Note: Only subjects with a successful insertion are considered
Global Biostatistics: /by-sasp/patdb/projects/de04209/310442/stat/prod_interim03/pgms/t-expul.sas epkll 07OCT2011 10:52
End of table

Table 14.1.2 / 34: Number of subjects with expulsions by parity (FAS)

Parity		LCS12 (N=1432)	LCS16 (N=1452)	Total (N=2884)
0 births	Number of Subjects	551 (100.0%)	570 (100.0%)	1121 (100.0%)
	IUS partially expelled			
	no	540 (98.0%)	563 (98.8%)	1103 (98.4%)
	yes	11 (2.0%)	7 (1.2%)	18 (1.6%)
	IUS totally expelled			
	no	546 (99.1%)	568 (99.6%)	1114 (99.4%)
	yes	5 (0.9%)	2 (0.4%)	7 (0.6%)
	Partial or total expulsion			
	no	535 (97.1%)	561 (98.4%)	1096 (97.8%)
	yes	16 (2.9%)	9 (1.6%)	25 (2.2%)
1 birth or more	Number of Subjects	875 (100.0%)	875 (100.0%)	1750 (100.0%)
	IUS partially expelled			
	no	862 (98.5%)	852 (97.4%)	1714 (97.9%)
	yes	13 (1.5%)	23 (2.6%)	36 (2.1%)
	IUS totally expelled			
	no	851 (97.3%)	861 (98.4%)	1712 (97.8%)
	yes	24 (2.7%)	14 (1.6%)	38 (2.2%)
	Partial or total expulsion			
	no	838 (95.8%)	838 (95.8%)	1676 (95.8%)
	yes	37 (4.2%)	37 (4.2%)	74 (4.2%)

Note: Only subjects with a successful insertion are considered
Global Biostatistics: /by-sasp/patdb/projects/de04209/310442/stat/prod_interim03/pgms/t-expul.sas epkll 07OCT2011 10:52
End of table

Table 14.1.2 / 35: Listing of subjects with expulsions (FAS)

TREATMENT	Subject number	Parity	Age category	IUS partially expelled	IUS totally expelled	Time to at least partial expulsion [days]	Time to total expulsion [days]
LCS12	120240	1 birth or more	25 < age <= 35	no	yes	574	574
	120242	0 births	age <= 25	no	yes	372	372
	120329	1 birth or more	age <= 25	no	yes	871	871
	120623	1 birth or more	age <= 25	no	yes	1029	1029
	140605	1 birth or more	age <= 25	yes	no	913	931
	140905	0 births	25 < age <= 35	yes	no	275	524
	141008	1 birth or more	25 < age <= 35	yes	no	63	63
	141416	1 birth or more	age <= 25	no	yes	270	270
	141426	0 births	age <= 25	no	yes	915	915
	150155	0 births	25 < age <= 35	yes	no	274	279
	160220	1 birth or more	25 < age <= 35	no	yes	712	712
	160309	0 births	age <= 25	yes	no	210	504
	161431	0 births	age <= 25	yes	no	572	580
	161540	0 births	age <= 25	yes	no	294	294
	180302	1 birth or more	25 < age <= 35	yes	no	423	423
	180315	1 birth or more	25 < age <= 35	yes	no	193	193
	180636	1 birth or more	25 < age <= 35	no	yes	1	1
	190104	0 births	age <= 25	yes	no	1103	1103
	190218	1 birth or more	age <= 25	yes	no	185	189
	190635	1 birth or more	age <= 25	no	yes	1083	1083
	200604	1 birth or more	age <= 25	no	yes	35	35
	200705	0 births	25 < age <= 35	yes	no	728	728
	210519	1 birth or more	25 < age <= 35	yes	no	643	643
	230312	1 birth or more	25 < age <= 35	no	yes	25	25
	230816	1 birth or more	25 < age <= 35	no	yes	981	981
	240351	1 birth or more	25 < age <= 35	no	yes	92	92
	240364	1 birth or more	age <= 25	no	yes	183	183
	240625	1 birth or more	age <= 25	yes	no	182	212
	240634	1 birth or more	25 < age <= 35	no	yes	1072	1072
	240638	1 birth or more	age <= 25	yes	no	33	34
	240808	0 births	25 < age <= 35	yes	no	365	365
	241187	1 birth or more	age <= 25	yes	no	176	176
	241201	1 birth or more	age <= 25	yes	no	784	792
	241704	1 birth or more	age <= 25	no	yes	751	751
	242909	0 births	age <= 25	no	yes	132	132
	243511	1 birth or more	25 < age <= 35	no	yes	745	745
	243803	1 birth or more	age <= 25	no	yes	120	120
	243810	1 birth or more	25 < age <= 35	no	yes	36	36
	244003	1 birth or more	25 < age <= 35	yes	no	84	451
	244109	1 birth or more	25 < age <= 35	no	yes	443	443
	244316	0 births	25 < age <= 35	yes	no	189	189
	244422	0 births	age <= 25	no	yes	741	741
	244601	1 birth or more	age <= 25	no	yes	29	29
	244937	1 birth or more	25 < age <= 35	no	yes	185	185

Table 14.1.2 / 35: Listing of subjects with expulsions (FAS)

TREATMENT	Subject number	Parity	Age category	IUS partially expelled	IUS totally expelled	Time to at least partial expulsion [days]	Time to total expulsion [days]
	245003	1 birth or more	25 < age <= 35	yes	no	277	311
	245402	1 birth or more	25 < age <= 35	no	yes	544	544
	245415	1 birth or more	25 < age <= 35	no	yes	561	561
	245907	0 births	25 < age <= 35	yes	no	85	85
	245932	0 births	25 < age <= 35	yes	no	277	295
	245934	1 birth or more	age <= 25	yes	no	92	92
	246121	1 birth or more	25 < age <= 35	no	yes	317	317
	246125	1 birth or more	25 < age <= 35	no	yes	638	638
	246212	0 births	age <= 25	no	yes	923	923
LCS16	120318	1 birth or more	25 < age <= 35	no	yes	816	816
	120319	0 births	age <= 25	no	yes	93	93
	160303	1 birth or more	25 < age <= 35	yes	no	92	290
	160318	1 birth or more	age <= 25	yes	no	65	85
	160621	1 birth or more	25 < age <= 35	yes	no	174	392
	170103	1 birth or more	25 < age <= 35	yes	no	92	92
	170303	1 birth or more	25 < age <= 35	yes	no	771	877
	180121	1 birth or more	25 < age <= 35	yes	no	594	594
	180134	1 birth or more	25 < age <= 35	yes	no	371	371
	180219	1 birth or more	25 < age <= 35	no	yes	43	43
	180226	1 birth or more	25 < age <= 35	no	yes	313	313
	180634	1 birth or more	25 < age <= 35	yes	no	1	288
	180653	1 birth or more	25 < age <= 35	yes	no	90	244
	180656	1 birth or more	25 < age <= 35	yes	no	92	287
	180679	1 birth or more	25 < age <= 35	yes	no	101	275
	180682	1 birth or more	25 < age <= 35	no	yes	1	1
	190611	1 birth or more	25 < age <= 35	yes	no	275	1119
	190627	1 birth or more	age <= 25	no	yes	84	84
	240133	1 birth or more	age <= 25	yes	no	622	622
	240354	1 birth or more	age <= 25	yes	no	56	56
	240822	0 births	age <= 25	yes	no	183	183
	240901	0 births	25 < age <= 35	yes	no	76	76
	241195	1 birth or more	age <= 25	no	yes	153	153
	241540	1 birth or more	25 < age <= 35	yes	no	190	190
	241732	1 birth or more	age <= 25	yes	no	32	32
	242507	0 births	25 < age <= 35	no	yes	279	279
	243034	1 birth or more	age <= 25	no	yes	494	494
	243101	1 birth or more	25 < age <= 35	yes	no	1097	1097
	243108	1 birth or more	age <= 25	yes	no	1	32
	243109	1 birth or more	age <= 25	yes	no	191	609
	243208	1 birth or more	25 < age <= 35	no	yes	410	410
	243604	1 birth or more	age <= 25	yes	no	303	1097
	243703	1 birth or more	25 < age <= 35	no	yes	791	791
	243918	0 births	age <= 25	yes	no	908	908

Table 14.1.2 / 35: Listing of subjects with expulsions (FAS)

TREATMENT	Subject number	Parity	Age category	IUS partially expelled	IUS totally expelled	Time to at least partial expulsion [days]	Time to total expulsion [days]
	243936	0 births	25 < age <= 35	yes	no	90	664
	243954	1 birth or more	age <= 25	no	yes	788	788
	244114	0 births	age <= 25	yes	no	92	92
	244309	1 birth or more	age <= 25	yes	no	28	28
	244715	1 birth or more	25 < age <= 35	yes	no	100	100
	244916	0 births	age <= 25	yes	no	1	1113
	244933	1 birth or more	25 < age <= 35	no	yes	276	276
	245031	0 births	age <= 25	yes	no	243	243
	245404	1 birth or more	25 < age <= 35	yes	no	268	268
	245406	1 birth or more	25 < age <= 35	no	yes	205	205
	245408	1 birth or more	25 < age <= 35	no	yes	881	881
	246120	1 birth or more	age <= 25	no	yes	1037	1037

Global Biostatistics: /by-sasp/patdb/projects/de04209/310442/stat/prod_interim03/pgms/t-expul.sas epkll 07OCT2011 10:52
End of table

Table 14.1.2 / 36: Probability of at least partial expulsion by treatment (Kaplan-Meier analysis) (FAS)

Treatment	Month	number of subjects still under treatment	cumulative number of expulsions	estimated cumulative probability of expulsion [%]
LCS12	0	1426	0	0.00
	6	1283	15	1.10
	12	1162	29	2.22
	18	1060	33	2.57
	24	960	40	3.25
	30	889	45	3.76
	36	271	52	4.56
LCS16	0	1445	0	0.00
	6	1314	22	1.58
	12	1199	33	2.43
	18	1104	36	2.68
	24	1008	38	2.86
	30	924	44	3.47
	36	305	45	3.58
Total	0	2871	0	0.00
	6	2597	37	1.34
	12	2361	62	2.32
	18	2164	69	2.62
	24	1968	78	3.05
	30	1813	89	3.61
	36	576	97	4.05

GB: /by-sasp/patdb/projects/de04209/310442/stat/prod_interim03/pgms/t-expul-km-part-all.sas epkll 07OCT2011 10:52
End of table

Table 14.1.2 / 37: Probability of at least partial expulsion by treatment by parity (Kaplan-Meier analysis) (FAS)

Parity: 0 births

Treatment	Month	number of subjects still under treatment	cumulative number of expulsions	estimated cumulative probability of expulsion [%]
LCS12	0	551	0	0.00
	6	492	2	0.39
	12	438	9	1.90
	18	397	10	2.13
	24	354	12	2.65
	30	336	13	2.93
	36	85	15	3.51
LCS16	0	570	0	0.00
	6	509	5	0.92
	12	457	8	1.52
	18	422	8	1.52
	24	380	8	1.52
	30	355	9	1.80
	36	107	9	1.80
Total	0	1121	0	0.00
	6	1001	7	0.66
	12	895	17	1.70
	18	819	18	1.81
	24	734	20	2.07
	30	691	22	2.35
	36	192	24	2.63

Table 14.1.2 / 37: Probability of at least partial expulsion by treatment by parity (Kaplan-Meier analysis) (FAS) (cont.)

Parity: 1 birth or more

Treatment	Month	number of subjects still under treatment	cumulative number of expulsions	estimated cumulative probability of expulsion [%]
LCS12	0	875	0	0.00
	6	791	13	1.54
	12	723	20	2.43
	18	663	23	2.86
	24	606	28	3.62
	30	553	32	4.28
	36	186	37	5.19
LCS16	0	875	0	0.00
	6	805	17	1.99
	12	742	25	3.00
	18	682	28	3.40
	24	628	30	3.69
	30	569	35	4.49
	36	198	36	4.67
Total	0	1750	0	0.00
	6	1596	30	1.77
	12	1465	45	2.72
	18	1345	51	3.13
	24	1234	58	3.65
	30	1122	67	4.38
	36	384	73	4.92

GB: /by-sasp/patdb/projects/de04209/310442/stat/prod_interim03/pgms/t-expul-km-part-par.sas epkll 07OCT2011 10:52
End of table

Table 14.1.2 / 38: Probability of at least partial expulsion by treatment by age group (Kaplan-Meier analysis) (FAS)

Age category: age <= 25

Treatment	Month	number of subjects still under treatment	cumulative number of expulsions	estimated cumulative probability of expulsion [%]
LCS12	0	560	0	0.00
	6	493	8	1.52
	12	434	13	2.56
	18	392	14	2.79
	24	354	15	3.04
	30	326	19	4.16
	36	86	24	5.73
LCS16	0	562	0	0.00
	6	508	10	1.83
	12	457	14	2.62
	18	424	15	2.85
	24	381	16	3.09
	30	351	18	3.63
	36	107	19	3.91
Total	0	1122	0	0.00
	6	1001	18	1.67
	12	891	27	2.59
	18	816	29	2.81
	24	735	31	3.06
	30	677	37	3.88
	36	193	43	4.78

Table 14.1.2 / 38: Probability of at least partial expulsion by treatment by age group (Kaplan-Meier analysis) (FAS) (cont.)

Age category: 25 < age <= 35

Treatment	Month	number of subjects still under treatment	cumulative number of expulsions	estimated cumulative probability of expulsion [%]
LCS12	0	866	0	0.00
	6	790	7	0.83
	12	726	16	2.00
	18	668	19	2.43
	24	606	25	3.35
	30	563	26	3.51
	36	185	28	3.87
LCS16	0	883	0	0.00
	6	806	12	1.41
	12	742	19	2.30
	18	680	21	2.57
	24	627	22	2.71
	30	573	26	3.36
	36	198	26	3.36
Total	0	1749	0	0.00
	6	1596	19	1.12
	12	1468	35	2.15
	18	1348	40	2.50
	24	1233	47	3.03
	30	1136	52	3.43
	36	383	54	3.61

GB: /by-sasp/patdb/projects/de04209/310442/stat/prod_interim03/pgms/t-expul-km-part-age.sas epkll 07OCT2011 10:53
End of table

Table 14.1.2 / 39: Probability of at total expulsion by treatment (Kaplan-Meier analysis) (FAS)

Treatment	Month	number of subjects still under treatment	cumulative number of expulsions	estimated cumulative probability of expulsion [%]
LCS12	0	1426	0	0.00
	6	1285	8	0.58
	12	1162	12	0.90
	18	1060	15	1.17
	24	960	19	1.56
	30	889	23	1.98
	36	271	29	2.68
LCS16	0	1445	0	0.00
	6	1322	5	0.36
	12	1205	9	0.68
	18	1109	11	0.85
	24	1011	11	0.85
	30	927	15	1.26
	36	308	16	1.37
Total	0	2871	0	0.00
	6	2607	13	0.47
	12	2367	21	0.79
	18	2169	26	1.01
	24	1971	30	1.20
	30	1816	38	1.61
	36	579	45	2.01

GB: /by-sasp/patdb/projects/de04209/310442/stat/prod_interim03/pgms/t-expul-km-tot-all.sas epkll 07OCT2011 10:53
End of table

Table 14.1.2 / 40: Probability of at total expulsion by treatment by parity (Kaplan-Meier analysis) (FAS)

Parity: 0 births

Treatment	Month	number of subjects still under treatment	cumulative number of expulsions	estimated cumulative probability of expulsion [%]
LCS12	0	551	0	0.00
	6	492	1	0.20
	12	438	1	0.20
	18	397	2	0.43
	24	354	2	0.43
	30	336	3	0.71
	36	85	5	1.31
LCS16	0	570	0	0.00
	6	511	1	0.19
	12	459	2	0.40
	18	424	2	0.40
	24	381	2	0.40
	30	356	2	0.40
	36	108	2	0.40
Total	0	1121	0	0.00
	6	1003	2	0.19
	12	897	3	0.30
	18	821	4	0.41
	24	735	4	0.41
	30	692	5	0.55
	36	193	7	0.84

GB: /by-sasp/patdb/projects/de04209/310442/stat/prod_interim03/pgms/t-expul-km-tot-par.sas epkll 07OCT2011 10:53

Table 14.1.2 / 40: Probability of at total expulsion by treatment by parity (Kaplan-Meier analysis) (FAS) (cont.)

Parity: 1 birth or more

Treatment	Month	number of subjects still under treatment	cumulative number of expulsions	estimated cumulative probability of expulsion [%]
LCS12	0	875	0	0.00
	6	793	7	0.82
	12	724	11	1.34
	18	663	13	1.63
	24	606	17	2.24
	30	553	20	2.74
	36	186	24	3.50
LCS16	0	875	0	0.00
	6	811	4	0.47
	12	746	7	0.85
	18	685	9	1.13
	24	630	9	1.13
	30	571	13	1.79
	36	200	14	1.97
Total	0	1750	0	0.00
	6	1604	11	0.65
	12	1470	18	1.09
	18	1348	22	1.38
	24	1236	26	1.68
	30	1124	33	2.26
	36	386	38	2.72

GB: /by-sasp/patdb/projects/de04209/310442/stat/prod_interim03/pgms/t-expul-km-tot-par.sas epkll 07OCT2011 10:53
End of table

Table 14.1.2 / 41: Probability of at total expulsion by treatment by age group (Kaplan-Meier analysis) (FAS)

Age category: age <= 25

Treatment	Month	number of subjects still under treatment	cumulative number of expulsions	estimated cumulative probability of expulsion [%]
LCS12	0	560	0	0.00
	6	494	4	0.76
	12	435	6	1.17
	18	392	7	1.40
	24	354	7	1.40
	30	326	10	2.26
	36	86	14	3.55
LCS16	0	562	0	0.00
	6	509	3	0.56
	12	460	3	0.56
	18	427	4	0.79
	24	383	4	0.79
	30	353	5	1.06
	36	109	6	1.36
Total	0	1122	0	0.00
	6	1003	7	0.66
	12	895	9	0.86
	18	819	11	1.09
	24	737	11	1.09
	30	679	15	1.65
	36	195	20	2.42

Table 14.1.2 / 41: Probability of at total expulsion by treatment by age group (Kaplan-Meier analysis) (FAS) (cont.)

Age category: 25 < age <= 35

Treatment	Month	number of subjects still under treatment	cumulative number of expulsions	estimated cumulative probability of expulsion [%]
LCS12	0	866	0	0.00
	6	791	4	0.47
	12	727	6	0.73
	18	668	8	1.02
	24	606	12	1.64
	30	563	13	1.80
	36	185	15	2.17
LCS16	0	883	0	0.00
	6	813	2	0.23
	12	745	6	0.74
	18	682	7	0.88
	24	628	7	0.88
	30	574	10	1.38
	36	199	10	1.38
Total	0	1749	0	0.00
	6	1604	6	0.35
	12	1472	12	0.74
	18	1350	15	0.95
	24	1234	19	1.26
	30	1137	23	1.59
	36	384	25	1.77

GB: /by-sasp/patdb/projects/de04209/310442/stat/prod_interim03/pgms/t-expul-km-tot-age.sas epkll 07OCT2011 10:53
End of table

Table 14.1.2 / 42: Proportion of subjects who are compliant by time point (Kaplan-Meier analysis) (FAS)

Treatment	Period	half yearly rate of non-compliance [%]	cumulative rate of non-compliance [%]	cumulative rate of compliance [%]
LCS12	1st half year	1.68	1.68	98.32
	2nd half year	1.87	3.51	96.49
	3rd half year	0.63	4.13	95.87
	4th half yesr	1.10	5.18	94.82
	5th half year	0.86	6.00	94.00
	6th half year	1.73	7.62	92.38
LCS16	1st half year	2.57	2.57	97.43
	2nd half year	1.35	3.88	96.12
	3rd half year	0.26	4.13	95.87
	4th half yesr	0.66	4.76	95.24
	5th half year	0.94	5.65	94.35
	6th half year	0.33	5.97	94.03
Total	1st half year	2.13	2.13	97.87
	2nd half year	1.60	3.70	96.30
	3rd half year	0.44	4.12	95.88
	4th half yesr	0.87	4.96	95.04
	5th half year	0.90	5.82	94.18
	6th half year	1.01	6.77	93.23

Note: Any documented IUS position other than 'in situ' or 'displaced, intrauterine' was considered non-compliant regardless of the reason
Global Biostatistics: /by-sasp/patdb/projects/de04209/310442/stat/prod_interim03/pgms/t-compl-km.sas epkll 07OCT2011 10:53
End of table

14.1.3 Exposure

Table 14.1.3 / 1: Extent of exposure - descriptive statistics by treatment (FAS)

		LCS12	LCS16	Total
Exposure to treatment [days]	n	1432	1452	2884
	Mean	820.5	843.0	831.8
	SD	376.0	365.5	370.8
	Min	1	1	1
	Q1	528.0	562.5	550.0
	Median	1085.0	1086.0	1085.0
	Q3	1093.5	1094.0	1094.0
	Max	1205	1248	1248
	Sum	1174915.0	1223998.0	2398913.0
Woman-years	n	1432	1452	2884
	Mean	2.25	2.31	2.28
	SD	1.03	1.00	1.02
	Min	0.0	0.0	0.0
	Q1	1.45	1.54	1.51
	Median	2.97	2.98	2.97
	Q3	3.00	3.00	3.00
	Max	3.3	3.4	3.4
	Sum	3218.95	3353.42	6572.36

For this presentation a woman-year equals 365 days
Global Biostatistics: /by-sasp/patdb/projects/de04209/310442/stat/prod_interim03/pgms/t-expos-saf.sas   epkll   07OCT2011 10:53
End of table

14.2 Efficacy

14.2.1 Contraceptive efficacy

Table 14.2.1 / 1: Contraceptive efficacy by treatment (FAS)

Pearl Index: unadj.

Time	TREATMENT	Number of subjects	Total exposure [WY]	Excluded exposure [WY]	Relevant exposure [WY]	Number of pregnancies	Pearl index	Lower 95% CIL	Upper 95% CIL
overall	LCS12	1432	3217.01	153.31	3063.70	10	0.33	0.16	0.60
	LCS16	1452	3351.99	129.92	3222.07	10	0.31	0.15	0.57
year 1	LCS12	1432	1280.22	62.44	1217.78	5	0.41	0.13	0.96
	LCS16	1452	1316.37	63.59	1252.78	2	0.16	0.02	0.58
year 2	LCS12	1162	1056.98	41.31	1015.67	3	0.30	0.06	0.86
	LCS16	1206	1105.32	37.83	1067.49	4	0.37	0.10	0.96
year 3	LCS12	960	870.42	45.25	825.17	2	0.24	0.03	0.88
	LCS16	1010	918.30	27.21	891.09	4	0.45	0.12	1.15
2 years	LCS12	1432	2337.20	103.75	2233.45	8	0.36	0.15	0.71
	LCS16	1452	2421.69	101.42	2320.27	6	0.26	0.09	0.56
3 years	LCS12	1432	3207.62	149.00	3058.62	10	0.33	0.16	0.60
	LCS16	1452	3339.99	128.63	3211.36	10	0.31	0.15	0.57

Note: WY = women years, 1 WY equals to 365 days, CIL = confidence interval limit
Global Biostatistics: /by-sasp/patdb/projects/de04209/310442/stat/prod_interim03/pgms/t-eff-pi-all.sas sgrpp 27OCT2011 12:52

Table 14.2.1 / 1: Contraceptive efficacy by treatment (FAS) (cont.)

Pearl Index: adj.

Time	TREATMENT	Number of subjects	Total exposure [WY]	Excluded exposure [WY]	Relevant exposure [WY]	Number of pregnancies	Pearl index	Lower 95% CIL	Upper 95% CIL
overall	LCS12	1432	3191.55	150.61	3040.95	10	0.33	0.16	0.60
	LCS16	1452	3320.24	127.77	3192.47	10	0.31	0.15	0.58
year 1	LCS12	1432	1270.42	61.59	1208.82	5	0.41	0.13	0.97
	LCS16	1452	1300.92	62.29	1238.62	2	0.16	0.02	0.58
year 2	LCS12	1155	1048.64	40.08	1008.56	3	0.30	0.06	0.87
	LCS16	1198	1095.63	37.27	1058.36	4	0.38	0.10	0.97
year 3	LCS12	953	863.44	44.76	818.68	2	0.24	0.03	0.88
	LCS16	1000	911.70	26.92	884.78	4	0.45	0.12	1.16
2 years	LCS12	1432	2319.06	101.67	2217.38	8	0.36	0.16	0.71
	LCS16	1452	2396.55	99.56	2296.99	6	0.26	0.10	0.57
3 years	LCS12	1432	3182.50	146.43	3036.07	10	0.33	0.16	0.61
	LCS16	1452	3308.24	126.47	3181.77	10	0.31	0.15	0.58

Note: WY = women years, 1 WY equals to 365 days, CIL = confidence interval limit
Global Biostatistics: /by-sasp/patdb/projects/de04209/310442/stat/prod_interim03/pgms/t-eff-pi-all.sas sgrpp 27OCT2011 12:52
End of table

Table 14.2.1 / 2: Contraceptive efficacy by treatment and age-group (FAS)

Pearl Index: unadj.
Age group: age <= 25

Time	TREATMENT	Number of subjects	Total exposure [WY]	Excluded exposure [WY]	Relevant exposure [WY]	Number of pregnancies	Pearl index	Lower 95% CIL	Upper 95% CIL
overall	LCS12	566	1202.58	86.18	1116.41	4	0.36	0.10	0.92
	LCS16	564	1284.62	73.22	1211.40	2	0.17	0.02	0.60
year 1	LCS12	566	491.49	35.87	455.62	1	0.22	0.01	1.22
	LCS16	564	508.48	35.16	473.33	1	0.21	0.01	1.18
year 2	LCS12	435	392.29	24.00	368.29	2	0.54	0.07	1.96
	LCS16	461	423.65	23.01	400.65	0	0.00	0.00	0.92
year 3	LCS12	354	315.24	24.95	290.30	1	0.34	0.01	1.92
	LCS16	383	347.48	14.27	333.21	1	0.30	0.01	1.67
2 years	LCS12	566	883.78	59.87	823.92	3	0.36	0.08	1.06
	LCS16	564	932.14	58.16	873.98	1	0.11	0.00	0.64
3 years	LCS12	566	1199.03	84.82	1114.21	4	0.36	0.10	0.92
	LCS16	564	1279.62	72.43	1207.19	2	0.17	0.02	0.60

Note: WY = women years, 1 WY equals to 365 days, CIL = confidence interval limit
Global Biostatistics: /by-sasp/patdb/projects/de04209/310442/stat/prod_interim03/pgms/t-eff-pi-age.sas sgrpp 27OCT2011 12:52

Table 14.2.1 / 2: Contraceptive efficacy by treatment and age-group (FAS) (cont.)

Pearl Index: unadj.
Age group: 25 < age <= 35

Time	TREATMENT	Number of subjects	Total exposure [WY]	Excluded exposure [WY]	Relevant exposure [WY]	Number of pregnancies	Pearl index	Lower 95% CIL	Upper 95% CIL
overall	LCS12	866	2014.42	67.13	1947.29	6	0.31	0.11	0.67
	LCS16	888	2067.37	56.70	2010.67	8	0.40	0.17	0.78
year 1	LCS12	866	788.73	26.57	762.15	4	0.52	0.14	1.34
	LCS16	888	807.88	28.43	779.45	1	0.13	0.00	0.71
year 2	LCS12	727	664.69	17.31	647.38	1	0.15	0.00	0.86
	LCS16	745	681.67	14.82	666.84	4	0.60	0.16	1.54
year 3	LCS12	606	555.18	20.30	534.87	1	0.19	0.00	1.04
	LCS16	627	570.82	12.94	557.88	3	0.54	0.11	1.57
2 years	LCS12	866	1453.42	43.88	1409.54	5	0.35	0.12	0.83
	LCS16	888	1489.55	43.26	1446.29	5	0.35	0.11	0.81
3 years	LCS12	866	2008.59	64.18	1944.41	6	0.31	0.11	0.67
	LCS16	888	2060.37	56.19	2004.17	8	0.40	0.17	0.79

Note: WY = women years, 1 WY equals to 365 days, CIL = confidence interval limit
Global Biostatistics: /by-sasp/patdb/projects/de04209/310442/stat/prod_interim03/pgms/t-eff-pi-age.sas sgrpp 27OCT2011 12:52

Table 14.2.1 / 2: Contraceptive efficacy by treatment and age-group (FAS) (cont.)

Pearl Index: adj.
Age group: age <= 25

Time	TREATMENT	Number of subjects	Total exposure [WY]	Excluded exposure [WY]	Relevant exposure [WY]	Number of pregnancies	Pearl index	Lower 95% CIL	Upper 95% CIL
overall	LCS12	566	1189.59	84.86	1104.73	4	0.36	0.10	0.93
	LCS16	564	1269.51	71.85	1197.66	2	0.17	0.02	0.60
year 1	LCS12	566	486.04	35.47	450.56	1	0.22	0.01	1.24
	LCS16	564	502.10	34.24	467.86	1	0.21	0.01	1.19
year 2	LCS12	432	388.32	23.35	364.97	2	0.55	0.07	1.98
	LCS16	458	417.98	22.70	395.28	0	0.00	0.00	0.93
year 3	LCS12	349	311.81	24.72	287.09	1	0.35	0.01	1.94
	LCS16	378	344.44	14.13	330.32	1	0.30	0.01	1.69
2 years	LCS12	566	874.36	58.83	815.53	3	0.37	0.08	1.08
	LCS16	564	920.07	56.93	863.14	1	0.12	0.00	0.65
3 years	LCS12	566	1186.17	83.55	1102.62	4	0.36	0.10	0.93
	LCS16	564	1264.52	71.06	1193.45	2	0.17	0.02	0.61

Note: WY = women years, 1 WY equals to 365 days, CIL = confidence interval limit
Global Biostatistics: /by-sasp/patdb/projects/de04209/310442/stat/prod_interim03/pgms/t-eff-pi-age.sas sgrpp 27OCT2011 12:52

Table 14.2.1 / 2: Contraceptive efficacy by treatment and age-group (FAS) (cont.)

Pearl Index: adj.
Age group: 25 < age <= 35

Time	TREATMENT	Number of subjects	Total exposure [WY]	Excluded exposure [WY]	Relevant exposure [WY]	Number of pregnancies	Pearl index	Lower 95% CIL	Upper 95% CIL
overall	LCS12	866	2001.96	65.75	1936.22	6	0.31	0.11	0.67
	LCS16	888	2050.73	55.92	1994.80	8	0.40	0.17	0.79
year 1	LCS12	866	784.38	26.12	758.26	4	0.53	0.14	1.35
	LCS16	888	798.82	28.05	770.76	1	0.13	0.00	0.72
year 2	LCS12	723	660.32	16.73	643.59	1	0.16	0.00	0.87
	LCS16	740	677.65	14.57	663.08	4	0.60	0.16	1.54
year 3	LCS12	604	551.63	20.04	531.59	1	0.19	0.00	1.05
	LCS16	622	567.25	12.79	554.47	3	0.54	0.11	1.58
2 years	LCS12	866	1444.70	42.85	1401.85	5	0.36	0.12	0.83
	LCS16	888	1476.47	42.62	1433.85	5	0.35	0.11	0.81
3 years	LCS12	866	1996.33	62.88	1933.44	6	0.31	0.11	0.68
	LCS16	888	2043.73	55.41	1988.31	8	0.40	0.17	0.79

Note: WY = women years, 1 WY equals to 365 days, CIL = confidence interval limit
Global Biostatistics: /by-sasp/patdb/projects/de04209/310442/stat/prod_interim03/pgms/t-eff-pi-age.sas sgrpp 27OCT2011 12:52
End of table

Table 14.2.1 / 3: Contraceptive efficacy by treatment and parity (FAS)

Pearl Index: unadj.
Parity: 0 births

Time	TREATMENT	Number of subjects	Total exposure [WY]	Excluded exposure [WY]	Relevant exposure [WY]	Number of pregnancies	Pearl index	Lower 95% CIL	Upper 95% CIL
overall	LCS12	556	1211.65	99.27	1112.38	4	0.36	0.10	0.92
	LCS16	574	1283.30	74.15	1209.16	3	0.25	0.05	0.73
year 1	LCS12	556	489.40	42.52	446.88	2	0.45	0.05	1.62
	LCS16	574	508.51	35.12	473.39	0	0.00	0.00	0.78
year 2	LCS12	438	395.52	28.17	367.34	1	0.27	0.01	1.52
	LCS16	459	420.01	21.54	398.47	1	0.25	0.01	1.40
year 3	LCS12	354	323.63	27.22	296.41	1	0.34	0.01	1.88
	LCS16	380	350.26	16.78	333.48	2	0.60	0.07	2.17
2 years	LCS12	556	884.92	70.69	814.22	3	0.37	0.08	1.08
	LCS16	574	928.52	56.66	871.86	1	0.11	0.00	0.64
3 years	LCS12	556	1208.55	97.92	1110.63	4	0.36	0.10	0.92
	LCS16	574	1278.78	73.44	1205.33	3	0.25	0.05	0.73

Note: WY = women years, 1 WY equals to 365 days, CIL = confidence interval limit
Global Biostatistics: /by-sasp/patdb/projects/de04209/310442/stat/prod_interim03/pgms/t-eff-pi-parity.sas sgrpp 27OCT2011 12:52

Table 14.2.1 / 3: Contraceptive efficacy by treatment and parity (FAS) (cont.)

Pearl Index: unadj.
Parity: 1 birth or more

Time	TREATMENT	Number of subjects	Total exposure [WY]	Excluded exposure [WY]	Relevant exposure [WY]	Number of pregnancies	Pearl index	Lower 95% CIL	Upper 95% CIL
overall	LCS12	876	2005.35	54.04	1951.32	6	0.31	0.11	0.67
	LCS16	878	2068.68	55.78	2012.91	7	0.35	0.14	0.72
year 1	LCS12	876	790.82	19.92	770.90	3	0.39	0.08	1.14
	LCS16	878	807.86	28.47	779.38	2	0.26	0.03	0.93
year 2	LCS12	724	661.47	13.14	648.33	2	0.31	0.04	1.11
	LCS16	747	685.32	16.29	669.03	3	0.45	0.09	1.31
year 3	LCS12	606	546.79	18.03	528.76	1	0.19	0.00	1.05
	LCS16	630	568.04	10.42	557.62	2	0.36	0.04	1.30
2 years	LCS12	876	1452.29	33.06	1419.23	5	0.35	0.11	0.82
	LCS16	878	1493.17	44.76	1448.41	5	0.35	0.11	0.81
3 years	LCS12	876	1999.07	51.08	1947.99	6	0.31	0.11	0.67
	LCS16	878	2061.21	55.19	2006.03	7	0.35	0.14	0.72

Note: WY = women years, 1 WY equals to 365 days, CIL = confidence interval limit
Global Biostatistics: /by-sasp/patdb/projects/de04209/310442/stat/prod_interim03/pgms/t-eff-pi-parity.sas sgrpp 27OCT2011 12:52

Table 14.2.1 / 3: Contraceptive efficacy by treatment and parity (FAS) (cont.)

Pearl Index: adj.
Parity: 0 births

Time	TREATMENT	Number of subjects	Total exposure [WY]	Excluded exposure [WY]	Relevant exposure [WY]	Number of pregnancies	Pearl index	Lower 95% CIL	Upper 95% CIL
overall	LCS12	556	1200.91	97.98	1102.93	4	0.36	0.10	0.93
	LCS16	574	1271.13	72.95	1198.18	3	0.25	0.05	0.73
year 1	LCS12	556	485.87	42.09	443.78	2	0.45	0.05	1.63
	LCS16	574	502.21	34.22	467.99	0	0.00	0.00	0.79
year 2	LCS12	436	391.53	27.55	363.99	1	0.27	0.01	1.53
	LCS16	456	416.31	21.37	394.93	1	0.25	0.01	1.41
year 3	LCS12	351	320.55	27.04	293.51	1	0.34	0.01	1.90
	LCS16	376	348.08	16.65	331.44	2	0.60	0.07	2.18
2 years	LCS12	556	877.41	69.64	807.77	3	0.37	0.08	1.09
	LCS16	574	918.52	55.60	862.92	1	0.12	0.00	0.65
3 years	LCS12	556	1197.95	96.68	1101.28	4	0.36	0.10	0.93
	LCS16	574	1266.60	72.24	1194.36	3	0.25	0.05	0.73

Note: WY = women years, 1 WY equals to 365 days, CIL = confidence interval limit
Global Biostatistics: /by-sasp/patdb/projects/de04209/310442/stat/prod_interim03/pgms/t-eff-pi-parity.sas sgrpp 27OCT2011 12:52

Table 14.2.1 / 3: Contraceptive efficacy by treatment and parity (FAS) (cont.)

Pearl Index: adj.
Parity: 1 birth or more

Time	TREATMENT	Number of subjects	Total exposure [WY]	Excluded exposure [WY]	Relevant exposure [WY]	Number of pregnancies	Pearl index	Lower 95% CIL	Upper 95% CIL
overall	LCS12	876	1990.64	52.63	1938.01	6	0.31	0.11	0.67
	LCS16	878	2049.11	54.82	1994.29	7	0.35	0.14	0.72
year 1	LCS12	876	784.55	19.50	765.04	3	0.39	0.08	1.15
	LCS16	878	798.70	28.07	770.64	2	0.26	0.03	0.94
year 2	LCS12	719	657.10	12.53	644.57	2	0.31	0.04	1.12
	LCS16	742	679.32	15.90	663.43	3	0.45	0.09	1.32
year 3	LCS12	602	542.89	17.72	525.18	1	0.19	0.00	1.06
	LCS16	624	563.61	10.27	553.35	2	0.36	0.04	1.31
2 years	LCS12	876	1441.65	32.04	1409.61	5	0.35	0.12	0.83
	LCS16	878	1478.03	43.96	1434.07	5	0.35	0.11	0.81
3 years	LCS12	876	1984.54	49.75	1934.79	6	0.31	0.11	0.67
	LCS16	878	2041.64	54.23	1987.41	7	0.35	0.14	0.73

Note: WY = women years, 1 WY equals to 365 days, CIL = confidence interval limit
Global Biostatistics: /by-sasp/patdb/projects/de04209/310442/stat/prod_interim03/pgms/t-eff-pi-parity.sas sgrpp 27OCT2011 12:52
End of table

Table 14.2.1 / 4: Contraceptive efficacy by treatment and BMI-group (FAS)

Pearl Index: unadj.
BMI group: missing

Time	TREATMENT	Number of subjects	Total exposure [WY]	Excluded exposure [WY]	Relevant exposure [WY]	Number of pregnancies	Pearl index	Lower 95% CIL	Upper 95% CIL
overall	LCS12	1	2.07	0.10	1.96	0	0.00	0.00	188.05
	LCS16	4	8.15	0.02	8.13	0	0.00	0.00	45.37
year 1	LCS12	1	1.00	0.08	0.92	0	0.00	0.00	403.13
	LCS16	4	3.57	0.02	3.55	0	0.00	0.00	103.89
year 2	LCS12	1	1.00	0.00	1.00	0	0.00	0.00	368.89
	LCS16	3	2.59	0.00	2.59	0	0.00	0.00	142.18
year 3	LCS12	1	0.07	0.02	0.05	0	0.00	0.00	7920.24
	LCS16	2	1.99	0.00	1.99	0	0.00	0.00	185.72
2 years	LCS12	1	2.00	0.08	1.92	0	0.00	0.00	192.62
	LCS16	4	6.16	0.02	6.15	0	0.00	0.00	60.03
3 years	LCS12	1	2.07	0.10	1.96	0	0.00	0.00	188.05
	LCS16	4	8.15	0.02	8.13	0	0.00	0.00	45.37

Note: WY = women years, 1 WY equals to 365 days, CIL = confidence interval limit
Global Biostatistics: /by-sasp/patdb/projects/de04209/310442/stat/prod_interim03/pgms/t-eff-pi-bmi.sas sgrpp 27OCT2011 12:52

Table 14.2.1 / 4: Contraceptive efficacy by treatment and BMI-group (FAS) (cont.)

Pearl Index: unadj.
BMI group: < 30 kg/m2

Time	TREATMENT	Number of subjects	Total exposure [WY]	Excluded exposure [WY]	Relevant exposure [WY]	Number of pregnancies	Pearl index	Lower 95% CIL	Upper 95% CIL
overall	LCS12	1187	2672.43	121.88	2550.55	9	0.35	0.16	0.67
	LCS16	1198	2781.56	108.49	2673.07	6	0.22	0.08	0.49
year 1	LCS12	1187	1059.53	49.80	1009.73	4	0.40	0.11	1.01
	LCS16	1198	1090.09	51.99	1038.10	1	0.10	0.00	0.54
year 2	LCS12	960	879.05	30.98	848.07	3	0.35	0.07	1.03
	LCS16	1000	917.49	31.77	885.72	2	0.23	0.03	0.82
year 3	LCS12	801	727.47	37.96	689.51	2	0.29	0.04	1.05
	LCS16	840	764.71	23.71	741.01	3	0.40	0.08	1.18
2 years	LCS12	1187	1938.58	80.77	1857.81	7	0.38	0.15	0.78
	LCS16	1198	2007.58	83.76	1923.81	3	0.16	0.03	0.46
3 years	LCS12	1187	2666.05	118.73	2547.32	9	0.35	0.16	0.67
	LCS16	1198	2772.29	107.47	2664.82	6	0.23	0.08	0.49

Note: WY = women years, 1 WY equals to 365 days, CIL = confidence interval limit
Global Biostatistics: /by-sasp/patdb/projects/de04209/310442/stat/prod_interim03/pgms/t-eff-pi-bmi.sas sgrpp 27OCT2011 12:52

Table 14.2.1 / 4: Contraceptive efficacy by treatment and BMI-group (FAS) (cont.)

Pearl Index: unadj.
BMI group: >= 30 kg/m2

Time	TREATMENT	Number of subjects	Total exposure [WY]	Excluded exposure [WY]	Relevant exposure [WY]	Number of pregnancies	Pearl index	Lower 95% CIL	Upper 95% CIL
overall	LCS12	244	542.51	31.32	511.19	1	0.20	0.00	1.09
	LCS16	250	562.28	21.42	540.87	4	0.74	0.20	1.89
year 1	LCS12	244	219.69	12.56	207.13	1	0.48	0.01	2.69
	LCS16	250	222.71	11.58	211.13	1	0.47	0.01	2.64
year 2	LCS12	201	176.93	10.33	166.60	0	0.00	0.00	2.21
	LCS16	203	185.24	6.06	179.18	2	1.12	0.14	4.03
year 3	LCS12	158	142.88	7.27	135.61	0	0.00	0.00	2.72
	LCS16	168	151.60	3.50	148.10	1	0.68	0.02	3.76
2 years	LCS12	244	396.62	22.89	373.73	1	0.27	0.01	1.49
	LCS16	250	407.95	17.64	390.31	3	0.77	0.16	2.25
3 years	LCS12	244	539.51	30.16	509.34	1	0.20	0.00	1.09
	LCS16	250	559.55	21.14	538.41	4	0.74	0.20	1.90

Note: WY = women years, 1 WY equals to 365 days, CIL = confidence interval limit
Global Biostatistics: /by-sasp/patdb/projects/de04209/310442/stat/prod_interim03/pgms/t-eff-pi-bmi.sas sgrpp 27OCT2011 12:52

Table 14.2.1 / 4: Contraceptive efficacy by treatment and BMI-group (FAS) (cont.)

Pearl Index: adj.
BMI group: missing

Time	TREATMENT	Number of subjects	Total exposure [WY]	Excluded exposure [WY]	Relevant exposure [WY]	Number of pregnancies	Pearl index	Lower 95% CIL	Upper 95% CIL
overall	LCS12	1	2.07	0.10	1.96	0	0.00	0.00	188.05
	LCS16	4	8.15	0.02	8.13	0	0.00	0.00	45.37
year 1	LCS12	1	1.00	0.08	0.92	0	0.00	0.00	403.13
	LCS16	4	3.57	0.02	3.55	0	0.00	0.00	103.89
year 2	LCS12	1	1.00	0.00	1.00	0	0.00	0.00	368.89
	LCS16	3	2.59	0.00	2.59	0	0.00	0.00	142.18
year 3	LCS12	1	0.07	0.02	0.05	0	0.00	0.00	7920.24
	LCS16	2	1.99	0.00	1.99	0	0.00	0.00	185.72
2 years	LCS12	1	2.00	0.08	1.92	0	0.00	0.00	192.62
	LCS16	4	6.16	0.02	6.15	0	0.00	0.00	60.03
3 years	LCS12	1	2.07	0.10	1.96	0	0.00	0.00	188.05
	LCS16	4	8.15	0.02	8.13	0	0.00	0.00	45.37

Note: WY = women years, 1 WY equals to 365 days, CIL = confidence interval limit
Global Biostatistics: /by-sasp/patdb/projects/de04209/310442/stat/prod_interim03/pgms/t-eff-pi-bmi.sas sgrpp 27OCT2011 12:52

Table 14.2.1 / 4: Contraceptive efficacy by treatment and BMI-group (FAS) (cont.)

Pearl Index: adj.
BMI group: < 30 kg/m2

Time	TREATMENT	Number of subjects	Total exposure [WY]	Excluded exposure [WY]	Relevant exposure [WY]	Number of pregnancies	Pearl index	Lower 95% CIL	Upper 95% CIL
overall	LCS12	1187	2650.97	119.51	2531.46	9	0.36	0.16	0.67
	LCS16	1198	2757.19	106.86	2650.33	6	0.23	0.08	0.49
year 1	LCS12	1187	1052.56	49.17	1003.39	4	0.40	0.11	1.02
	LCS16	1198	1079.20	51.15	1028.05	1	0.10	0.00	0.54
year 2	LCS12	955	870.88	29.78	841.09	3	0.36	0.07	1.04
	LCS16	994	909.24	31.26	877.98	2	0.23	0.03	0.82
year 3	LCS12	794	721.39	37.50	683.89	2	0.29	0.04	1.06
	LCS16	831	759.48	23.44	736.05	3	0.41	0.08	1.19
2 years	LCS12	1187	1923.44	78.95	1844.48	7	0.38	0.15	0.78
	LCS16	1198	1988.44	82.41	1906.03	3	0.16	0.03	0.46
3 years	LCS12	1187	2644.82	116.45	2528.38	9	0.36	0.16	0.68
	LCS16	1198	2747.92	105.84	2642.08	6	0.23	0.08	0.49

Note: WY = women years, 1 WY equals to 365 days, CIL = confidence interval limit
Global Biostatistics: /by-sasp/patdb/projects/de04209/310442/stat/prod_interim03/pgms/t-eff-pi-bmi.sas sgrpp 27OCT2011 12:52

Table 14.2.1 / 4: Contraceptive efficacy by treatment and BMI-group (FAS) (cont.)

Pearl Index: adj.
BMI group: >= 30 kg/m2

Time	TREATMENT	Number of subjects	Total exposure [WY]	Excluded exposure [WY]	Relevant exposure [WY]	Number of pregnancies	Pearl index	Lower 95% CIL	Upper 95% CIL
overall	LCS12	244	538.51	30.99	507.52	1	0.20	0.00	1.10
	LCS16	250	554.90	20.89	534.01	4	0.75	0.20	1.92
year 1	LCS12	244	216.86	12.34	204.52	1	0.49	0.01	2.72
	LCS16	250	218.15	11.13	207.02	1	0.48	0.01	2.69
year 2	LCS12	199	176.76	10.30	166.47	0	0.00	0.00	2.22
	LCS16	201	183.80	6.01	177.79	2	1.12	0.14	4.06
year 3	LCS12	158	141.98	7.24	134.74	0	0.00	0.00	2.74
	LCS16	167	150.23	3.48	146.75	1	0.68	0.02	3.80
2 years	LCS12	244	393.62	22.64	370.99	1	0.27	0.01	1.50
	LCS16	250	401.95	17.13	384.81	3	0.78	0.16	2.28
3 years	LCS12	244	535.61	29.88	505.73	1	0.20	0.01	1.10
	LCS16	250	552.17	20.61	531.56	4	0.75	0.21	1.93

Note: WY = women years, 1 WY equals to 365 days, CIL = confidence interval limit
Global Biostatistics: /by-sasp/patdb/projects/de04209/310442/stat/prod_interim03/pgms/t-eff-pi-bmi.sas sgrpp 27OCT2011 12:52
End of table

Table 14.2.1 / 5: Listing with details on pregnancies (FAS)

TREATMENT	Subject number	Age/BMI(kg/m2) /parity	Pregnancy implantation	Pregnancy implantation, text	IUS insertion date	Last day on study medication (imputed)	Conception date / Conception date (imputed)
LCS12	120329	23 / 22.5 / 1 birth or more	.		18APR2008	05SEP2010	07AUG2010 / 07AUG10
	120419	30 / 21.8 / 1 birth or more	ectopic		14MAY2008	24FEB2009	04JAN2009 / 04JAN09
	140202	27 / 29 / 0 births	other	TRANSVAGINAL ULTRASOUND INCONCLUSIVE FOR IMPLANTATION	20FEB2008	03SEP2010	01AUG2010 / 01AUG10
	141008	26 / 21.9 / 1 birth or more	.		27FEB2008	29APR2008	07APR2008 / 07APR08
	160743	18 / 19.8 / 0 births	ectopic		18JAN2008	27APR2008	APR2008 / 01APR08
	190636	23 / 26.2 / 1 birth or more	.		19MAY2008	24SEP2009	23JUN2009 / 23JUN09
	210519	34 / 23.4 / 1 birth or more	.		12MAR2008	14DEC2009	29OCT2009 / 29OCT09
	230303	20 / 22.5 / 0 births	ectopic		14JAN2008	16JUN2009	MAY2009 / 01MAY09
	245003	27 / 21 / 1 birth or more	.		07JAN2008	12NOV2008	03OCT2008 / 03OCT08
	245932	27 / 36.7 / 0 births	.		05MAY2008	23FEB2009	JAN2009 / 01JAN09
LCS16	160423	26 / 35 / 1 birth or more	ectopic		18DEC2007	09OCT2008	26SEP2008 / 26SEP08
	160927	33 / 19.9 / 1 birth or more	other	BLIGHTED OVUM	22OCT2007	10FEB2010	10DEC2009 / 10DEC09
	180112	33 / 33.8 / 1 birth or more	.		04DEC2007	04JUN2009	02MAY2009 / 02MAY09
	180317	33 / 31.5 / 1 birth or more	ectopic		22NOV2007	03JAN2009	07DEC2008 / 07DEC08
	200609	32 / 19.9 / 1 birth or more	ectopic		03JAN2008	21DEC2009	OCT2009 / 01OCT09
	242321	31 / 21.1 / 0 births	ectopic		21APR2008	19JUL2010	2010 / 01JAN10
	243228	23 / 27.6 / 1 birth or more	ectopic		20MAY2008	06MAY2009	28MAR2009 / 28MAR09
	243703	26 / 42.9 / 1 birth or more	.		13DEC2007	10FEB2010	08FEB2010 / 08FEB10
	243932	29 / 21.3 / 0 births	ectopic		21FEB2008	08MAR2011	FEB2011 / 01FEB11

Table 14.2.1 / 5: Listing with details on pregnancies (FAS)

TREATMENT	Subject number	Age/BMI(kg/m2) /parity	Pregnancy implantation	Pregnancy implantation, text	IUS insertion date	Last day on study medication (imputed)	Conception date / Conception date (imputed)
	244519	25 / 26.9 / 0 births	ectopic		10APR2008	18JUN2010	08JUN2010 / 08JUN10
	245406	26 / 27.2 / 1 birth or more	.		27FEB2008	18SEP2008	08OCT2008 / 08OCT08

Global Biostatistics: /by-sasp/patdb/projects/de04209/310442/stat/prod_interim03/pgms/t-preg1.sas sgrpp 27OCT2011 12:52

Table 14.2.1 / 5: Listing with details on pregnancies (FAS) (cont.)

TREATMENT	Partial expulsion / total expulsion	Conception relative to insertion [days]	Conception relative to removal/expulsion [days]
LCS12	/ 05SEP2010	841	-29
	/	235	-51
	/	893	-33
	/	40	-22
	/	74	-26
	/	400	-93
	14DEC2009 /	596	-46
	/	473	-46
	/	270	-40
	05FEB2009 /	241	-53
LCS16	/	283	-13
	/	780	-62
	/	515	-33
	/	381	-27
	/	637	-81
	/	620	-199
	/	312	-39
	/ 2010	788	-2
	/	1076	-35
	/	789	-10
	/ 18SEP2008	224	20

Global Biostatistics: /by-sasp/patdb/projects/de04209/310442/stat/prod_interim03/pgms/t-preg1.sas sgrpp 27OCT2011 12:52
End of table

Table 14.2.1 / 6: Contraceptive efficacy (ectopic pregnancies) by treatment (FAS)

Pearl Index: unadj.

Time	TREATMENT	Number of subjects	Total exposure [WY]	Excluded exposure [WY]	Relevant exposure [WY]	Number of pregnancies	Pearl index	Lower 95% CIL	Upper 95% CIL
overall	LCS12	1432	3217.01	153.31	3063.70	3	0.10	0.02	0.29
	LCS16	1452	3351.99	129.92	3222.07	7	0.22	0.09	0.45
year 1	LCS12	1432	1280.22	62.44	1217.78	2	0.16	0.02	0.59
	LCS16	1452	1316.37	63.59	1252.78	2	0.16	0.02	0.58
year 2	LCS12	1162	1056.98	41.31	1015.67	1	0.10	0.00	0.55
	LCS16	1206	1105.32	37.83	1067.49	3	0.28	0.06	0.82
year 3	LCS12	960	870.42	45.25	825.17	0	0.00	0.00	0.45
	LCS16	1010	918.30	27.21	891.09	2	0.22	0.03	0.81
2 years	LCS12	1432	2337.20	103.75	2233.45	3	0.13	0.03	0.39
	LCS16	1452	2421.69	101.42	2320.27	5	0.22	0.07	0.50
3 years	LCS12	1432	3207.62	149.00	3058.62	3	0.10	0.02	0.29
	LCS16	1452	3339.99	128.63	3211.36	7	0.22	0.09	0.45

Note: WY = women years, 1 WY equals to 365 days, CIL = confidence interval limit
Global Biostatistics: /by-sasp/patdb/projects/de04209/310442/stat/prod_interim03/pgms/t-eff-pi-ect-all.sas sgrpp 27OCT2011 12:52

Table 14.2.1 / 6: Contraceptive efficacy (ectopic pregnancies) by treatment (FAS) (cont.)

Pearl Index: adj.

Time	TREATMENT	Number of subjects	Total exposure [WY]	Excluded exposure [WY]	Relevant exposure [WY]	Number of pregnancies	Pearl index	Lower 95% CIL	Upper 95% CIL
overall	LCS12	1432	3191.55	150.61	3040.95	3	0.10	0.02	0.29
	LCS16	1452	3320.24	127.77	3192.47	7	0.22	0.09	0.45
year 1	LCS12	1432	1270.42	61.59	1208.82	2	0.17	0.02	0.60
	LCS16	1452	1300.92	62.29	1238.62	2	0.16	0.02	0.58
year 2	LCS12	1155	1048.64	40.08	1008.56	1	0.10	0.00	0.55
	LCS16	1198	1095.63	37.27	1058.36	3	0.28	0.06	0.83
year 3	LCS12	953	863.44	44.76	818.68	0	0.00	0.00	0.45
	LCS16	1000	911.70	26.92	884.78	2	0.23	0.03	0.82
2 years	LCS12	1432	2319.06	101.67	2217.38	3	0.14	0.03	0.40
	LCS16	1452	2396.55	99.56	2296.99	5	0.22	0.07	0.51
3 years	LCS12	1432	3182.50	146.43	3036.07	3	0.10	0.02	0.29
	LCS16	1452	3308.24	126.47	3181.77	7	0.22	0.09	0.45

Note: WY = women years, 1 WY equals to 365 days, CIL = confidence interval limit
Global Biostatistics: /by-sasp/patdb/projects/de04209/310442/stat/prod_interim03/pgms/t-eff-pi-ect-all.sas sgrpp 27OCT2011 12:52
End of table

Table 14.2.1 / 7: Contraceptive efficacy (ectopic pregnancies) by treatment and age-group (FAS)

Pearl Index: unadj.
Age group: age <= 25

Time	TREATMENT	Number of subjects	Total exposure [WY]	Excluded exposure [WY]	Relevant exposure [WY]	Number of pregnancies	Pearl index	Lower 95% CIL	Upper 95% CIL
overall	LCS12	566	1202.58	86.18	1116.41	2	0.18	0.02	0.65
	LCS16	564	1284.62	73.22	1211.40	2	0.17	0.02	0.60
year 1	LCS12	566	491.49	35.87	455.62	1	0.22	0.01	1.22
	LCS16	564	508.48	35.16	473.33	1	0.21	0.01	1.18
year 2	LCS12	435	392.29	24.00	368.29	1	0.27	0.01	1.51
	LCS16	461	423.65	23.01	400.65	0	0.00	0.00	0.92
year 3	LCS12	354	315.24	24.95	290.30	0	0.00	0.00	1.27
	LCS16	383	347.48	14.27	333.21	1	0.30	0.01	1.67
2 years	LCS12	566	883.78	59.87	823.92	2	0.24	0.03	0.88
	LCS16	564	932.14	58.16	873.98	1	0.11	0.00	0.64
3 years	LCS12	566	1199.03	84.82	1114.21	2	0.18	0.02	0.65
	LCS16	564	1279.62	72.43	1207.19	2	0.17	0.02	0.60

Note: WY = women years, 1 WY equals to 365 days, CIL = confidence interval limit
Global Biostatistics: /by-sasp/patdb/projects/de04209/310442/stat/prod_interim03/pgms/t-eff-pi-ect-age.sas sgrpp 27OCT2011 12:52

Table 14.2.1 / 7: Contraceptive efficacy (ectopic pregnancies) by treatment and age-group (FAS) (cont.)

Pearl Index: unadj.
Age group: 25 < age <= 35

Time	TREATMENT	Number of subjects	Total exposure [WY]	Excluded exposure [WY]	Relevant exposure [WY]	Number of pregnancies	Pearl index	Lower 95% CIL	Upper 95% CIL
overall	LCS12	866	2014.42	67.13	1947.29	1	0.05	0.00	0.29
	LCS16	888	2067.37	56.70	2010.67	5	0.25	0.08	0.58
year 1	LCS12	866	788.73	26.57	762.15	1	0.13	0.00	0.73
	LCS16	888	807.88	28.43	779.45	1	0.13	0.00	0.71
year 2	LCS12	727	664.69	17.31	647.38	0	0.00	0.00	0.57
	LCS16	745	681.67	14.82	666.84	3	0.45	0.09	1.31
year 3	LCS12	606	555.18	20.30	534.87	0	0.00	0.00	0.69
	LCS16	627	570.82	12.94	557.88	1	0.18	0.00	1.00
2 years	LCS12	866	1453.42	43.88	1409.54	1	0.07	0.00	0.40
	LCS16	888	1489.55	43.26	1446.29	4	0.28	0.08	0.71
3 years	LCS12	866	2008.59	64.18	1944.41	1	0.05	0.00	0.29
	LCS16	888	2060.37	56.19	2004.17	5	0.25	0.08	0.58

Note: WY = women years, 1 WY equals to 365 days, CIL = confidence interval limit
Global Biostatistics: /by-sasp/patdb/projects/de04209/310442/stat/prod_interim03/pgms/t-eff-pi-ect-age.sas sgrpp 27OCT2011 12:52

Table 14.2.1 / 7: Contraceptive efficacy (ectopic pregnancies) by treatment and age-group (FAS) (cont.)

Pearl Index: adj.
Age group: age <= 25

Time	TREATMENT	Number of subjects	Total exposure [WY]	Excluded exposure [WY]	Relevant exposure [WY]	Number of pregnancies	Pearl index	Lower 95% CIL	Upper 95% CIL
overall	LCS12	566	1189.59	84.86	1104.73	2	0.18	0.02	0.65
	LCS16	564	1269.51	71.85	1197.66	2	0.17	0.02	0.60
year 1	LCS12	566	486.04	35.47	450.56	1	0.22	0.01	1.24
	LCS16	564	502.10	34.24	467.86	1	0.21	0.01	1.19
year 2	LCS12	432	388.32	23.35	364.97	1	0.27	0.01	1.53
	LCS16	458	417.98	22.70	395.28	0	0.00	0.00	0.93
year 3	LCS12	349	311.81	24.72	287.09	0	0.00	0.00	1.28
	LCS16	378	344.44	14.13	330.32	1	0.30	0.01	1.69
2 years	LCS12	566	874.36	58.83	815.53	2	0.25	0.03	0.89
	LCS16	564	920.07	56.93	863.14	1	0.12	0.00	0.65
3 years	LCS12	566	1186.17	83.55	1102.62	2	0.18	0.02	0.66
	LCS16	564	1264.52	71.06	1193.45	2	0.17	0.02	0.61

Note: WY = women years, 1 WY equals to 365 days, CIL = confidence interval limit
Global Biostatistics: /by-sasp/patdb/projects/de04209/310442/stat/prod_interim03/pgms/t-eff-pi-ect-age.sas sgrpp 27OCT2011 12:52

Table 14.2.1 / 7: Contraceptive efficacy (ectopic pregnancies) by treatment and age-group (FAS) (cont.)

Pearl Index: adj.
Age group: 25 < age <= 35

Time	TREATMENT	Number of subjects	Total exposure [WY]	Excluded exposure [WY]	Relevant exposure [WY]	Number of pregnancies	Pearl index	Lower 95% CIL	Upper 95% CIL
overall	LCS12	866	2001.96	65.75	1936.22	1	0.05	0.00	0.29
	LCS16	888	2050.73	55.92	1994.80	5	0.25	0.08	0.58
year 1	LCS12	866	784.38	26.12	758.26	1	0.13	0.00	0.73
	LCS16	888	798.82	28.05	770.76	1	0.13	0.00	0.72
year 2	LCS12	723	660.32	16.73	643.59	0	0.00	0.00	0.57
	LCS16	740	677.65	14.57	663.08	3	0.45	0.09	1.32
year 3	LCS12	604	551.63	20.04	531.59	0	0.00	0.00	0.69
	LCS16	622	567.25	12.79	554.47	1	0.18	0.00	1.00
2 years	LCS12	866	1444.70	42.85	1401.85	1	0.07	0.00	0.40
	LCS16	888	1476.47	42.62	1433.85	4	0.28	0.08	0.71
3 years	LCS12	866	1996.33	62.88	1933.44	1	0.05	0.00	0.29
	LCS16	888	2043.73	55.41	1988.31	5	0.25	0.08	0.59

Note: WY = women years, 1 WY equals to 365 days, CIL = confidence interval limit
Global Biostatistics: /by-sasp/patdb/projects/de04209/310442/stat/prod_interim03/pgms/t-eff-pi-ect-age.sas sgrpp 27OCT2011 12:52
End of table

Table 14.2.1 / 8: Contraceptive efficacy (ectopic pregnacies) by treatment and parity (FAS)

Pearl Index: unadj.
Parity: 0 births

Time	TREATMENT	Number of subjects	Total exposure [WY]	Excluded exposure [WY]	Relevant exposure [WY]	Number of pregnancies	Pearl index	Lower 95% CIL	Upper 95% CIL
overall	LCS12	556	1211.65	99.27	1112.38	2	0.18	0.02	0.65
	LCS16	574	1283.30	74.15	1209.16	3	0.25	0.05	0.73
year 1	LCS12	556	489.40	42.52	446.88	1	0.22	0.01	1.25
	LCS16	574	508.51	35.12	473.39	0	0.00	0.00	0.78
year 2	LCS12	438	395.52	28.17	367.34	1	0.27	0.01	1.52
	LCS16	459	420.01	21.54	398.47	1	0.25	0.01	1.40
year 3	LCS12	354	323.63	27.22	296.41	0	0.00	0.00	1.24
	LCS16	380	350.26	16.78	333.48	2	0.60	0.07	2.17
2 years	LCS12	556	884.92	70.69	814.22	2	0.25	0.03	0.89
	LCS16	574	928.52	56.66	871.86	1	0.11	0.00	0.64
3 years	LCS12	556	1208.55	97.92	1110.63	2	0.18	0.02	0.65
	LCS16	574	1278.78	73.44	1205.33	3	0.25	0.05	0.73

Note: WY = women years, 1 WY equals to 365 days, CIL = confidence interval limit
GB: /by-sasp/patdb/projects/de04209/310442/stat/prod_interim03/pgms/t-eff-pi-ect-parity.sas sgrpp 27OCT2011 12:53

Table 14.2.1 / 8: Contraceptive efficacy (ectopic pregnacies) by treatment and parity (FAS) (cont.)

Pearl Index: unadj.
Parity: 1 birth or more

Time	TREATMENT	Number of subjects	Total exposure [WY]	Excluded exposure [WY]	Relevant exposure [WY]	Number of pregnancies	Pearl index	Lower 95% CIL	Upper 95% CIL
overall	LCS12	876	2005.35	54.04	1951.32	1	0.05	0.00	0.29
	LCS16	878	2068.68	55.78	2012.91	4	0.20	0.05	0.51
year 1	LCS12	876	790.82	19.92	770.90	1	0.13	0.00	0.72
	LCS16	878	807.86	28.47	779.38	2	0.26	0.03	0.93
year 2	LCS12	724	661.47	13.14	648.33	0	0.00	0.00	0.57
	LCS16	747	685.32	16.29	669.03	2	0.30	0.04	1.08
year 3	LCS12	606	546.79	18.03	528.76	0	0.00	0.00	0.70
	LCS16	630	568.04	10.42	557.62	0	0.00	0.00	0.66
2 years	LCS12	876	1452.29	33.06	1419.23	1	0.07	0.00	0.39
	LCS16	878	1493.17	44.76	1448.41	4	0.28	0.08	0.71
3 years	LCS12	876	1999.07	51.08	1947.99	1	0.05	0.00	0.29
	LCS16	878	2061.21	55.19	2006.03	4	0.20	0.05	0.51

Note: WY = women years, 1 WY equals to 365 days, CIL = confidence interval limit
GB: /by-sasp/patdb/projects/de04209/310442/stat/prod_interim03/pgms/t-eff-pi-ect-parity.sas sgrpp 27OCT2011 12:53

Table 14.2.1 / 8: Contraceptive efficacy (ectopic pregnancies) by treatment and parity (FAS) (cont.)

Pearl Index: adj.
Parity: 0 births

Time	TREATMENT	Number of subjects	Total exposure [WY]	Excluded exposure [WY]	Relevant exposure [WY]	Number of pregnancies	Pearl index	Lower 95% CIL	Upper 95% CIL
overall	LCS12	556	1200.91	97.98	1102.93	2	0.18	0.02	0.66
	LCS16	574	1271.13	72.95	1198.18	3	0.25	0.05	0.73
year 1	LCS12	556	485.87	42.09	443.78	1	0.23	0.01	1.26
	LCS16	574	502.21	34.22	467.99	0	0.00	0.00	0.79
year 2	LCS12	436	391.53	27.55	363.99	1	0.27	0.01	1.53
	LCS16	456	416.31	21.37	394.93	1	0.25	0.01	1.41
year 3	LCS12	351	320.55	27.04	293.51	0	0.00	0.00	1.26
	LCS16	376	348.08	16.65	331.44	2	0.60	0.07	2.18
2 years	LCS12	556	877.41	69.64	807.77	2	0.25	0.03	0.89
	LCS16	574	918.52	55.60	862.92	1	0.12	0.00	0.65
3 years	LCS12	556	1197.95	96.68	1101.28	2	0.18	0.02	0.66
	LCS16	574	1266.60	72.24	1194.36	3	0.25	0.05	0.73

Note: WY = women years, 1 WY equals to 365 days, CIL = confidence interval limit
GB: /by-sasp/patdb/projects/de04209/310442/stat/prod_interim03/pgms/t-eff-pi-ect-parity.sas sgrpp 27OCT2011 12:53

Table 14.2.1 / 8: Contraceptive efficacy (ectopic pregnancies) by treatment and parity (FAS) (cont.)

Pearl Index: adj.
Parity: 1 birth or more

Time	TREATMENT	Number of subjects	Total exposure [WY]	Excluded exposure [WY]	Relevant exposure [WY]	Number of pregnancies	Pearl index	Lower 95% CIL	Upper 95% CIL
overall	LCS12	876	1990.64	52.63	1938.01	1	0.05	0.00	0.29
	LCS16	878	2049.11	54.82	1994.29	4	0.20	0.05	0.51
year 1	LCS12	876	784.55	19.50	765.04	1	0.13	0.00	0.73
	LCS16	878	798.70	28.07	770.64	2	0.26	0.03	0.94
year 2	LCS12	719	657.10	12.53	644.57	0	0.00	0.00	0.57
	LCS16	742	679.32	15.90	663.43	2	0.30	0.04	1.09
year 3	LCS12	602	542.89	17.72	525.18	0	0.00	0.00	0.70
	LCS16	624	563.61	10.27	553.35	0	0.00	0.00	0.67
2 years	LCS12	876	1441.65	32.04	1409.61	1	0.07	0.00	0.40
	LCS16	878	1478.03	43.96	1434.07	4	0.28	0.08	0.71
3 years	LCS12	876	1984.54	49.75	1934.79	1	0.05	0.00	0.29
	LCS16	878	2041.64	54.23	1987.41	4	0.20	0.05	0.52

Note: WY = women years, 1 WY equals to 365 days, CIL = confidence interval limit
GB: /by-sasp/patdb/projects/de04209/310442/stat/prod_interim03/pgms/t-eff-pi-ect-parity.sas sgrpp 27OCT2011 12:53
End of table

Table 14.2.1 / 9: Contraceptive efficacy (ectopic pregnancies) by treatment and BMI-group (FAS)

Pearl Index: unadj.
BMI group: missing

Time	TREATMENT	Number of subjects	Total exposure [WY]	Excluded exposure [WY]	Relevant exposure [WY]	Number of pregnancies	Pearl index	Lower 95% CIL	Upper 95% CIL
overall	LCS12	1	2.07	0.10	1.96	0	0.00	0.00	188.05
	LCS16	4	8.15	0.02	8.13	0	0.00	0.00	45.37
year 1	LCS12	1	1.00	0.08	0.92	0	0.00	0.00	403.13
	LCS16	4	3.57	0.02	3.55	0	0.00	0.00	103.89
year 2	LCS12	1	1.00	0.00	1.00	0	0.00	0.00	368.89
	LCS16	3	2.59	0.00	2.59	0	0.00	0.00	142.18
year 3	LCS12	1	0.07	0.02	0.05	0	0.00	0.00	7920.24
	LCS16	2	1.99	0.00	1.99	0	0.00	0.00	185.72
2 years	LCS12	1	2.00	0.08	1.92	0	0.00	0.00	192.62
	LCS16	4	6.16	0.02	6.15	0	0.00	0.00	60.03
3 years	LCS12	1	2.07	0.10	1.96	0	0.00	0.00	188.05
	LCS16	4	8.15	0.02	8.13	0	0.00	0.00	45.37

Note: WY = women years, 1 WY equals to 365 days, CIL = confidence interval limit
Global Biostatistics: /by-sasp/patdb/projects/de04209/310442/stat/prod_interim03/pgms/t-eff-pi-ect-bmi.sas sgrpp 27OCT2011 12:53

Table 14.2.1 / 9: Contraceptive efficacy (ectopic pregnancies) by treatment and BMI-group (FAS) (cont.)

Pearl Index: unadj.
BMI group: < 30 kg/m2

Time	TREATMENT	Number of subjects	Total exposure [WY]	Excluded exposure [WY]	Relevant exposure [WY]	Number of pregnancies	Pearl index	Lower 95% CIL	Upper 95% CIL
overall	LCS12	1187	2672.43	121.88	2550.55	3	0.12	0.02	0.34
	LCS16	1198	2781.56	108.49	2673.07	5	0.19	0.06	0.44
year 1	LCS12	1187	1059.53	49.80	1009.73	2	0.20	0.02	0.72
	LCS16	1198	1090.09	51.99	1038.10	1	0.10	0.00	0.54
year 2	LCS12	960	879.05	30.98	848.07	1	0.12	0.00	0.66
	LCS16	1000	917.49	31.77	885.72	2	0.23	0.03	0.82
year 3	LCS12	801	727.47	37.96	689.51	0	0.00	0.00	0.54
	LCS16	840	764.71	23.71	741.01	2	0.27	0.03	0.97
2 years	LCS12	1187	1938.58	80.77	1857.81	3	0.16	0.03	0.47
	LCS16	1198	2007.58	83.76	1923.81	3	0.16	0.03	0.46
3 years	LCS12	1187	2666.05	118.73	2547.32	3	0.12	0.02	0.34
	LCS16	1198	2772.29	107.47	2664.82	5	0.19	0.06	0.44

Note: WY = women years, 1 WY equals to 365 days, CIL = confidence interval limit
Global Biostatistics: /by-sasp/patdb/projects/de04209/310442/stat/prod_interim03/pgms/t-eff-pi-ect-bmi.sas sgrpp 27OCT2011 12:53

Table 14.2.1 / 9: Contraceptive efficacy (ectopic pregnancies) by treatment and BMI-group (FAS) (cont.)

Pearl Index: unadj.
BMI group: >= 30 kg/m2

Time	TREATMENT	Number of subjects	Total exposure [WY]	Excluded exposure [WY]	Relevant exposure [WY]	Number of pregnancies	Pearl index	Lower 95% CIL	Upper 95% CIL
overall	LCS12	244	542.51	31.32	511.19	0	0.00	0.00	0.72
	LCS16	250	562.28	21.42	540.87	2	0.37	0.04	1.34
year 1	LCS12	244	219.69	12.56	207.13	0	0.00	0.00	1.78
	LCS16	250	222.71	11.58	211.13	1	0.47	0.01	2.64
year 2	LCS12	201	176.93	10.33	166.60	0	0.00	0.00	2.21
	LCS16	203	185.24	6.06	179.18	1	0.56	0.01	3.11
year 3	LCS12	158	142.88	7.27	135.61	0	0.00	0.00	2.72
	LCS16	168	151.60	3.50	148.10	0	0.00	0.00	2.49
2 years	LCS12	244	396.62	22.89	373.73	0	0.00	0.00	0.99
	LCS16	250	407.95	17.64	390.31	2	0.51	0.06	1.85
3 years	LCS12	244	539.51	30.16	509.34	0	0.00	0.00	0.72
	LCS16	250	559.55	21.14	538.41	2	0.37	0.04	1.34

Note: WY = women years, 1 WY equals to 365 days, CIL = confidence interval limit
Global Biostatistics: /by-sasp/patdb/projects/de04209/310442/stat/prod_interim03/pgms/t-eff-pi-ect-bmi.sas sgrpp 27OCT2011 12:53

Table 14.2.1 / 9: Contraceptive efficacy (ectopic pregnancies) by treatment and BMI-group (FAS) (cont.)

Pearl Index: adj.
BMI group: missing

Time	TREATMENT	Number of subjects	Total exposure [WY]	Excluded exposure [WY]	Relevant exposure [WY]	Number of pregnancies	Pearl index	Lower 95% CIL	Upper 95% CIL
overall	LCS12	1	2.07	0.10	1.96	0	0.00	0.00	188.05
	LCS16	4	8.15	0.02	8.13	0	0.00	0.00	45.37
year 1	LCS12	1	1.00	0.08	0.92	0	0.00	0.00	403.13
	LCS16	4	3.57	0.02	3.55	0	0.00	0.00	103.89
year 2	LCS12	1	1.00	0.00	1.00	0	0.00	0.00	368.89
	LCS16	3	2.59	0.00	2.59	0	0.00	0.00	142.18
year 3	LCS12	1	0.07	0.02	0.05	0	0.00	0.00	7920.24
	LCS16	2	1.99	0.00	1.99	0	0.00	0.00	185.72
2 years	LCS12	1	2.00	0.08	1.92	0	0.00	0.00	192.62
	LCS16	4	6.16	0.02	6.15	0	0.00	0.00	60.03
3 years	LCS12	1	2.07	0.10	1.96	0	0.00	0.00	188.05
	LCS16	4	8.15	0.02	8.13	0	0.00	0.00	45.37

Note: WY = women years, 1 WY equals to 365 days, CIL = confidence interval limit
Global Biostatistics: /by-sasp/patdb/projects/de04209/310442/stat/prod_interim03/pgms/t-eff-pi-ect-bmi.sas sgrpp 27OCT2011 12:53

Table 14.2.1 / 9: Contraceptive efficacy (ectopic pregnancies) by treatment and BMI-group (FAS) (cont.)

Pearl Index: adj.
BMI group: < 30 kg/m2

Time	TREATMENT	Number of subjects	Total exposure [WY]	Excluded exposure [WY]	Relevant exposure [WY]	Number of pregnancies	Pearl index	Lower 95% CIL	Upper 95% CIL
overall	LCS12	1187	2650.97	119.51	2531.46	3	0.12	0.02	0.35
	LCS16	1198	2757.19	106.86	2650.33	5	0.19	0.06	0.44
year 1	LCS12	1187	1052.56	49.17	1003.39	2	0.20	0.02	0.72
	LCS16	1198	1079.20	51.15	1028.05	1	0.10	0.00	0.54
year 2	LCS12	955	870.88	29.78	841.09	1	0.12	0.00	0.66
	LCS16	994	909.24	31.26	877.98	2	0.23	0.03	0.82
year 3	LCS12	794	721.39	37.50	683.89	0	0.00	0.00	0.54
	LCS16	831	759.48	23.44	736.05	2	0.27	0.03	0.98
2 years	LCS12	1187	1923.44	78.95	1844.48	3	0.16	0.03	0.48
	LCS16	1198	1988.44	82.41	1906.03	3	0.16	0.03	0.46
3 years	LCS12	1187	2644.82	116.45	2528.38	3	0.12	0.02	0.35
	LCS16	1198	2747.92	105.84	2642.08	5	0.19	0.06	0.44

Note: WY = women years, 1 WY equals to 365 days, CIL = confidence interval limit
Global Biostatistics: /by-sasp/patdb/projects/de04209/310442/stat/prod_interim03/pgms/t-eff-pi-ect-bmi.sas sgrpp 27OCT2011 12:53

Table 14.2.1 / 9: Contraceptive efficacy (ectopic pregnancies) by treatment and BMI-group (FAS) (cont.)

Pearl Index: adj.
BMI group: >= 30 kg/m2

Time	TREATMENT	Number of subjects	Total exposure [WY]	Excluded exposure [WY]	Relevant exposure [WY]	Number of pregnancies	Pearl index	Lower 95% CIL	Upper 95% CIL
overall	LCS12	244	538.51	30.99	507.52	0	0.00	0.00	0.73
	LCS16	250	554.90	20.89	534.01	2	0.37	0.05	1.35
year 1	LCS12	244	216.86	12.34	204.52	0	0.00	0.00	1.80
	LCS16	250	218.15	11.13	207.02	1	0.48	0.01	2.69
year 2	LCS12	199	176.76	10.30	166.47	0	0.00	0.00	2.22
	LCS16	201	183.80	6.01	177.79	1	0.56	0.01	3.13
year 3	LCS12	158	141.98	7.24	134.74	0	0.00	0.00	2.74
	LCS16	167	150.23	3.48	146.75	0	0.00	0.00	2.51
2 years	LCS12	244	393.62	22.64	370.99	0	0.00	0.00	0.99
	LCS16	250	401.95	17.13	384.81	2	0.52	0.06	1.88
3 years	LCS12	244	535.61	29.88	505.73	0	0.00	0.00	0.73
	LCS16	250	552.17	20.61	531.56	2	0.38	0.05	1.36

Note: WY = women years, 1 WY equals to 365 days, CIL = confidence interval limit
Global Biostatistics: /by-sasp/patdb/projects/de04209/310442/stat/prod_interim03/pgms/t-eff-pi-ect-bmi.sas sgrpp 27OCT2011 12:53
End of table

Table 14.2.1 / 10: Probability of getting pregnant by treatment (Kaplan-Meier analysis) (FAS)

Analysis type: unadj.

Time	TREATMENT	Number of subjects	Total exposure [WY]	Excluded exposure [WY]	Relevant exposure [WY]	Number of pregnancies	Cumulative probability	lower 95% CIL	upper 95% CIL
year 1	LCS12	1432	1280.22	62.44	1217.78	5	0.004	0.002	0.010
	LCS16	1452	1316.37	63.59	1252.78	2	0.002	0.000	0.007
year 2	LCS12	1162	1056.98	41.31	1015.67	3	0.003	0.001	0.009
	LCS16	1206	1105.32	37.83	1067.49	4	0.004	0.001	0.010
year 3	LCS12	960	870.42	45.25	825.17	2	0.002	0.001	0.009
	LCS16	1010	918.30	27.21	891.09	4	0.004	0.002	0.011
2 years	LCS12	1432	2337.20	103.75	2233.45	8	0.007	0.003	0.014
	LCS16	1452	2421.69	101.42	2320.27	6	0.005	0.002	0.012
3 years	LCS12	1432	3207.62	149.00	3058.62	10	0.009	0.005	0.017
	LCS16	1452	3339.99	128.63	3211.36	10	0.010	0.005	0.018

Note: WY = women years, 1 WY equals to 365 days, CIL = confidence interval limit
Global Biostatistics: /by-sasp/patdb/projects/de04209/310442/stat/prod_interim03/pgms/i-eff-km-all.sas sgrpp 27OCT2011 12:53

Table 14.2.1 / 10: Probability of getting pregnant by treatment (Kaplan-Meier analysis) (FAS) (cont.)

Analysis type: adj.

Time	TREATMENT	Number of subjects	Total exposure [WY]	Excluded exposure [WY]	Relevant exposure [WY]	Number of pregnancies	Cumulative probability	lower 95% CIL	upper 95% CIL
year 1	LCS12	1432	1270.42	61.59	1208.82	5	0.004	0.002	0.010
	LCS16	1452	1300.92	62.29	1238.62	2	0.002	0.000	0.007
year 2	LCS12	1155	1048.64	40.08	1008.56	3	0.003	0.001	0.009
	LCS16	1198	1095.63	37.27	1058.36	4	0.004	0.001	0.010
year 3	LCS12	953	863.44	44.76	818.68	2	0.002	0.001	0.009
	LCS16	1000	911.70	26.92	884.78	4	0.004	0.002	0.011
2 years	LCS12	1432	2319.06	101.67	2217.38	8	0.007	0.003	0.014
	LCS16	1452	2396.55	99.56	2296.99	6	0.005	0.002	0.012
3 years	LCS12	1432	3182.50	146.43	3036.07	10	0.009	0.005	0.017
	LCS16	1452	3308.24	126.47	3181.77	10	0.010	0.005	0.018

Note: WY = women years, 1 WY equals to 365 days, CIL = confidence interval limit
Global Biostatistics: /by-sasp/patdb/projects/de04209/310442/stat/prod_interim03/pgms/i-eff-km-all.sas sgrpp 27OCT2011 12:53
End of table

Table 14.2.1 / 11: Probability of getting pregnant by treatment and age-group (Kaplan-Meier analysis) (FAS)

Analysis type: unadj.
Age category: age <= 25

Time	TREATMENT	Number of subjects	Total exposure [WY]	Excluded exposure [WY]	Relevant exposure [WY]	Number of pregnancies	Cumulative probability	lower 95% CIL	upper 95% CIL
year 1	LCS12	566	491.49	35.87	455.62	1	0.002	0.000	0.013
	LCS16	564	508.48	35.16	473.33	1	0.002	0.000	0.018
year 2	LCS12	435	392.29	24.00	368.29	2	0.005	0.001	0.019
	LCS16	461	423.65	23.01	400.65	0	0.000	0.000	0.000
year 3	LCS12	354	315.24	24.95	290.30	1	0.003	0.000	0.022
	LCS16	383	347.48	14.27	333.21	1	0.003	0.000	0.019
2 years	LCS12	566	883.78	59.87	823.92	3	0.007	0.002	0.022
	LCS16	564	932.14	58.16	873.98	1	0.002	0.000	0.015
3 years	LCS12	566	1199.03	84.82	1114.21	4	0.010	0.004	0.027
	LCS16	564	1279.62	72.43	1207.19	2	0.005	0.001	0.019

Note: WY = women years, 1 WY equals to 365 days, CIL = confidence interval limit
Global Biostatistics: /by-sasp/patdb/projects/de04209/310442/stat/prod_interim03/pgms/i-eff-km-age.sas sgrpp 27OCT2011 12:53

Table 14.2.1 / 11: Probability of getting pregnant by treatment and age-group (Kaplan-Meier analysis) (FAS) (cont.)

Analysis type: unadj.
Age category: 25 < age <= 35

Time	TREATMENT	Number of subjects	Total exposure [WY]	Excluded exposure [WY]	Relevant exposure [WY]	Number of pregnancies	Cumulative probability	lower 95% CIL	upper 95% CIL
year 1	LCS12	866	788.73	26.57	762.15	4	0.005	0.002	0.014
	LCS16	888	807.88	28.43	779.45	1	0.001	0.000	0.010
year 2	LCS12	727	664.69	17.31	647.38	1	0.002	0.000	0.011
	LCS16	745	681.67	14.82	666.84	4	0.006	0.002	0.016
year 3	LCS12	606	555.18	20.30	534.87	1	0.002	0.000	0.013
	LCS16	627	570.82	12.94	557.88	3	0.005	0.002	0.016
2 years	LCS12	866	1453.42	43.88	1409.54	5	0.007	0.003	0.016
	LCS16	888	1489.55	43.26	1446.29	5	0.007	0.003	0.017
3 years	LCS12	866	2008.59	64.18	1944.41	6	0.008	0.004	0.019
	LCS16	888	2060.37	56.19	2004.17	8	0.012	0.006	0.025

Note: WY = women years, 1 WY equals to 365 days, CIL = confidence interval limit
Global Biostatistics: /by-sasp/patdb/projects/de04209/310442/stat/prod_interim03/pgms/i-eff-km-age.sas sgrpp 27OCT2011 12:53

Table 14.2.1 / 11: Probability of getting pregnant by treatment and age-group (Kaplan-Meier analysis) (FAS) (cont.)

Analysis type: adj.
Age category: age <= 25

Time	TREATMENT	Number of subjects	Total exposure [WY]	Excluded exposure [WY]	Relevant exposure [WY]	Number of pregnancies	Cumulative probability	lower 95% CIL	upper 95% CIL
year 1	LCS12	566	486.04	35.47	450.56	1	0.002	0.000	0.014
	LCS16	564	502.10	34.24	467.86	1	0.003	0.000	0.018
year 2	LCS12	432	388.32	23.35	364.97	2	0.005	0.001	0.020
	LCS16	458	417.98	22.70	395.28	0	0.000	0.000	0.000
year 3	LCS12	349	311.81	24.72	287.09	1	0.003	0.000	0.022
	LCS16	378	344.44	14.13	330.32	1	0.003	0.000	0.019
2 years	LCS12	566	874.36	58.83	815.53	3	0.007	0.002	0.022
	LCS16	564	920.07	56.93	863.14	1	0.002	0.000	0.015
3 years	LCS12	566	1186.17	83.55	1102.62	4	0.010	0.004	0.027
	LCS16	564	1264.52	71.06	1193.45	2	0.005	0.001	0.020

Note: WY = women years, 1 WY equals to 365 days, CIL = confidence interval limit
Global Biostatistics: /by-sasp/patdb/projects/de04209/310442/stat/prod_interim03/pgms/i-eff-km-age.sas sgrpp 27OCT2011 12:53

Table 14.2.1 / 11: Probability of getting pregnant by treatment and age-group (Kaplan-Meier analysis) (FAS) (cont.)

Analysis type: adj.
Age category: 25 < age <= 35

Time	TREATMENT	Number of subjects	Total exposure [WY]	Excluded exposure [WY]	Relevant exposure [WY]	Number of pregnancies	Cumulative probability	lower 95% CIL	upper 95% CIL
year 1	LCS12	866	784.38	26.12	758.26	4	0.005	0.002	0.014
	LCS16	888	798.82	28.05	770.76	1	0.001	0.000	0.010
year 2	LCS12	723	660.32	16.73	643.59	1	0.002	0.000	0.011
	LCS16	740	677.65	14.57	663.08	4	0.006	0.002	0.016
year 3	LCS12	604	551.63	20.04	531.59	1	0.002	0.000	0.013
	LCS16	622	567.25	12.79	554.47	3	0.005	0.002	0.016
2 years	LCS12	866	1444.70	42.85	1401.85	5	0.007	0.003	0.016
	LCS16	888	1476.47	42.62	1433.85	5	0.007	0.003	0.018
3 years	LCS12	866	1996.33	62.88	1933.44	6	0.009	0.004	0.019
	LCS16	888	2043.73	55.41	1988.31	8	0.012	0.006	0.025

Note: WY = women years, 1 WY equals to 365 days, CIL = confidence interval limit
Global Biostatistics: /by-sasp/patdb/projects/de04209/310442/stat/prod_interim03/pgms/i-eff-km-age.sas sgrpp 27OCT2011 12:53
End of table

Table 14.2.1 / 12: Probability of getting pregnant by treatment and parity (Kaplan-Meier analysis) (FAS)

Analysis type: unadj.
Parity: 0 births

Time	TREATMENT	Number of subjects	Total exposure [WY]	Excluded exposure [WY]	Relevant exposure [WY]	Number of pregnancies	Cumulative probability	lower 95% CIL	upper 95% CIL
year 1	LCS12	556	489.40	42.52	446.88	2	0.004	0.001	0.017
	LCS16	574	508.51	35.12	473.39	0	0.000	0.000	0.000
year 2	LCS12	438	395.52	28.17	367.34	1	0.003	0.000	0.018
	LCS16	459	420.01	21.54	398.47	1	0.003	0.000	0.018
year 3	LCS12	354	323.63	27.22	296.41	1	0.003	0.000	0.022
	LCS16	380	350.26	16.78	333.48	2	0.006	0.001	0.023
2 years	LCS12	556	884.92	70.69	814.22	3	0.007	0.002	0.020
	LCS16	574	928.52	56.66	871.86	1	0.003	0.000	0.020
3 years	LCS12	556	1208.55	97.92	1110.63	4	0.010	0.004	0.026
	LCS16	574	1278.78	73.44	1205.33	3	0.008	0.003	0.026

Note: WY = women years, 1 WY equals to 365 days, CIL = confidence interval limit
Global Biostatistics: /by-sasp/patdb/projects/de04209/310442/stat/prod_interim03/pgms/i-eff-km-parity.sas sgrpp 27OCT2011 12:53

Table 14.2.1 / 12: Probability of getting pregnant by treatment and parity (Kaplan-Meier analysis) (FAS) (cont.)

Analysis type: unadj.
Parity: 1 birth or more

Time	TREATMENT	Number of subjects	Total exposure [WY]	Excluded exposure [WY]	Relevant exposure [WY]	Number of pregnancies	Cumulative probability	lower 95% CIL	upper 95% CIL
year 1	LCS12	876	790.82	19.92	770.90	3	0.004	0.001	0.012
	LCS16	878	807.86	28.47	779.38	2	0.003	0.001	0.011
year 2	LCS12	724	661.47	13.14	648.33	2	0.003	0.001	0.012
	LCS16	747	685.32	16.29	669.03	3	0.004	0.001	0.014
year 3	LCS12	606	546.79	18.03	528.76	1	0.002	0.000	0.013
	LCS16	630	568.04	10.42	557.62	2	0.003	0.001	0.013
2 years	LCS12	876	1452.29	33.06	1419.23	5	0.007	0.003	0.017
	LCS16	878	1493.17	44.76	1448.41	5	0.007	0.003	0.017
3 years	LCS12	876	1999.07	51.08	1947.99	6	0.009	0.004	0.019
	LCS16	878	2061.21	55.19	2006.03	7	0.010	0.005	0.022

Note: WY = women years, 1 WY equals to 365 days, CIL = confidence interval limit
Global Biostatistics: /by-sasp/patdb/projects/de04209/310442/stat/prod_interim03/pgms/i-eff-km-parity.sas sgrpp 27OCT2011 12:53

Table 14.2.1 / 12: Probability of getting pregnant by treatment and parity (Kaplan-Meier analysis) (FAS) (cont.)

Analysis type: adj.
Parity: 0 births

Time	TREATMENT	Number of subjects	Total exposure [WY]	Excluded exposure [WY]	Relevant exposure [WY]	Number of pregnancies	Cumulative probability	lower 95% CIL	upper 95% CIL
year 1	LCS12	556	485.87	42.09	443.78	2	0.004	0.001	0.017
	LCS16	574	502.21	34.22	467.99	0	0.000	0.000	0.000
year 2	LCS12	436	391.53	27.55	363.99	1	0.003	0.000	0.018
	LCS16	456	416.31	21.37	394.93	1	0.003	0.000	0.019
year 3	LCS12	351	320.55	27.04	293.51	1	0.003	0.000	0.022
	LCS16	376	348.08	16.65	331.44	2	0.006	0.001	0.023
2 years	LCS12	556	877.41	69.64	807.77	3	0.007	0.002	0.021
	LCS16	574	918.52	55.60	862.92	1	0.003	0.000	0.020
3 years	LCS12	556	1197.95	96.68	1101.28	4	0.010	0.004	0.026
	LCS16	574	1266.60	72.24	1194.36	3	0.008	0.003	0.026

Note: WY = women years, 1 WY equals to 365 days, CIL = confidence interval limit
Global Biostatistics: /by-sasp/patdb/projects/de04209/310442/stat/prod_interim03/pgms/i-eff-km-parity.sas sgrpp 27OCT2011 12:53

Table 14.2.1 / 12: Probability of getting pregnant by treatment and parity (Kaplan-Meier analysis) (FAS) (cont.)

Analysis type: adj.
Parity: 1 birth or more

Time	TREATMENT	Number of subjects	Total exposure [WY]	Excluded exposure [WY]	Relevant exposure [WY]	Number of pregnancies	Cumulative probability	lower 95% CIL	upper 95% CIL
year 1	LCS12	876	784.55	19.50	765.04	3	0.004	0.001	0.012
	LCS16	878	798.70	28.07	770.64	2	0.003	0.001	0.011
year 2	LCS12	719	657.10	12.53	644.57	2	0.003	0.001	0.012
	LCS16	742	679.32	15.90	663.43	3	0.004	0.001	0.014
year 3	LCS12	602	542.89	17.72	525.18	1	0.002	0.000	0.013
	LCS16	624	563.61	10.27	553.35	2	0.003	0.001	0.013
2 years	LCS12	876	1441.65	32.04	1409.61	5	0.007	0.003	0.017
	LCS16	878	1478.03	43.96	1434.07	5	0.007	0.003	0.017
3 years	LCS12	876	1984.54	49.75	1934.79	6	0.009	0.004	0.019
	LCS16	878	2041.64	54.23	1987.41	7	0.010	0.005	0.022

Note: WY = women years, 1 WY equals to 365 days, CIL = confidence interval limit
Global Biostatistics: /by-sasp/patdb/projects/de04209/310442/stat/prod_interim03/pgms/i-eff-km-parity.sas sgrpp 27OCT2011 12:53
End of table

Table 14.2.1 / 13: Probability of getting pregnant by treatment and BMI-group (Kaplan-Meier analysis) (FAS)

Analysis type: unadj.
BMI group: missing

Time	TREATMENT	Number of subjects	Total exposure [WY]	Excluded exposure [WY]	Relevant exposure [WY]	Number of pregnancies	Cumulative probability	lower 95% CIL	upper 95% CIL
year 1	LCS12	1	1.00	0.08	0.92	0	0.000	0.000	0.000
	LCS16	4	3.57	0.02	3.55	0	0.000	0.000	0.000
year 2	LCS12	1	1.00	0.00	1.00	0	0.000	0.000	0.000
	LCS16	3	2.59	0.00	2.59	0	0.000	0.000	0.000
year 3	LCS12	1	0.07	0.02	0.05	0	0.000	0.000	0.000
	LCS16	2	1.99	0.00	1.99	0	0.000	0.000	0.000
2 years	LCS12	1	2.00	0.08	1.92	0	0.000	0.000	0.000
	LCS16	4	6.16	0.02	6.15	0	0.000	0.000	0.000
3 years	LCS12	1	2.07	0.10	1.96	0	0.000	0.000	0.000
	LCS16	4	8.15	0.02	8.13	0	0.000	0.000	0.000

Note: WY = women years, 1 WY equals to 365 days, CIL = confidence interval limit
Global Biostatistics: /by-sasp/patdb/projects/de04209/310442/stat/prod_interim03/pgms/i-eff-km-bmi.sas sgrpp 27OCT2011 12:53

Table 14.2.1 / 13: Probability of getting pregnant by treatment and BMI-group (Kaplan-Meier analysis) (FAS) (cont.)

Analysis type: unadj.
BMI group: < 30 kg/m2

Time	TREATMENT	Number of subjects	Total exposure [WY]	Excluded exposure [WY]	Relevant exposure [WY]	Number of pregnancies	Cumulative probability	lower 95% CIL	upper 95% CIL
year 1	LCS12	1187	1059.53	49.80	1009.73	4	0.004	0.001	0.010
	LCS16	1198	1090.09	51.99	1038.10	1	0.001	0.000	0.008
year 2	LCS12	960	879.05	30.98	848.07	3	0.003	0.001	0.011
	LCS16	1000	917.49	31.77	885.72	2	0.002	0.001	0.009
year 3	LCS12	801	727.47	37.96	689.51	2	0.003	0.001	0.011
	LCS16	840	764.71	23.71	741.01	3	0.004	0.001	0.012
2 years	LCS12	1187	1938.58	80.77	1857.81	7	0.007	0.003	0.015
	LCS16	1198	2007.58	83.76	1923.81	3	0.003	0.001	0.010
3 years	LCS12	1187	2666.05	118.73	2547.32	9	0.010	0.005	0.019
	LCS16	1198	2772.29	107.47	2664.82	6	0.007	0.003	0.016

Note: WY = women years, 1 WY equals to 365 days, CIL = confidence interval limit
Global Biostatistics: /by-sasp/patdb/projects/de04209/310442/stat/prod_interim03/pgms/i-eff-km-bmi.sas sgrpp 27OCT2011 12:53

Table 14.2.1 / 13: Probability of getting pregnant by treatment and BMI-group (Kaplan-Meier analysis) (FAS) (cont.)

Analysis type: unadj.
BMI group: >= 30 kg/m2

Time	TREATMENT	Number of subjects	Total exposure [WY]	Excluded exposure [WY]	Relevant exposure [WY]	Number of pregnancies	Cumulative probability	lower 95% CIL	upper 95% CIL
year 1	LCS12	244	219.69	12.56	207.13	1	0.005	0.001	0.034
	LCS16	250	222.71	11.58	211.13	1	0.005	0.001	0.037
year 2	LCS12	201	176.93	10.33	166.60	0	0.000	0.000	0.000
	LCS16	203	185.24	6.06	179.18	2	0.010	0.003	0.041
year 3	LCS12	158	142.88	7.27	135.61	0	0.000	0.000	0.000
	LCS16	168	151.60	3.50	148.10	1	0.006	0.001	0.044
2 years	LCS12	244	396.62	22.89	373.73	1	0.005	0.001	0.033
	LCS16	250	407.95	17.64	390.31	3	0.015	0.005	0.047
3 years	LCS12	244	539.51	30.16	509.34	1	0.005	0.001	0.033
	LCS16	250	559.55	21.14	538.41	4	0.022	0.008	0.057

Note: WY = women years, 1 WY equals to 365 days, CIL = confidence interval limit
Global Biostatistics: /by-sasp/patdb/projects/de04209/310442/stat/prod_interim03/pgms/i-eff-km-bmi.sas   sgrpp   27OCT2011 12:53

Table 14.2.1 / 13: Probability of getting pregnant by treatment and BMI-group (Kaplan-Meier analysis) (FAS) (cont.)

Analysis type: adj.
BMI group: missing

Time	TREATMENT	Number of subjects	Total exposure [WY]	Excluded exposure [WY]	Relevant exposure [WY]	Number of pregnancies	Cumulative probability	lower 95% CIL	upper 95% CIL
year 1	LCS12	1	1.00	0.08	0.92	0	0.000	0.000	0.000
	LCS16	4	3.57	0.02	3.55	0	0.000	0.000	0.000
year 2	LCS12	1	1.00	0.00	1.00	0	0.000	0.000	0.000
	LCS16	3	2.59	0.00	2.59	0	0.000	0.000	0.000
year 3	LCS12	1	0.07	0.02	0.05	0	0.000	0.000	0.000
	LCS16	2	1.99	0.00	1.99	0	0.000	0.000	0.000
2 years	LCS12	1	2.00	0.08	1.92	0	0.000	0.000	0.000
	LCS16	4	6.16	0.02	6.15	0	0.000	0.000	0.000
3 years	LCS12	1	2.07	0.10	1.96	0	0.000	0.000	0.000
	LCS16	4	8.15	0.02	8.13	0	0.000	0.000	0.000

Note: WY = women years, 1 WY equals to 365 days, CIL = confidence interval limit
Global Biostatistics: /by-sasp/patdb/projects/de04209/310442/stat/prod_interim03/pgms/i-eff-km-bmi.sas sgrpp 27OCT2011 12:53

Table 14.2.1 / 13: Probability of getting pregnant by treatment and BMI-group (Kaplan-Meier analysis) (FAS) (cont.)

Analysis type: adj.
BMI group: < 30 kg/m2

Time	TREATMENT	Number of subjects	Total exposure [WY]	Excluded exposure [WY]	Relevant exposure [WY]	Number of pregnancies	Cumulative probability	lower 95% CIL	upper 95% CIL
year 1	LCS12	1187	1052.56	49.17	1003.39	4	0.004	0.001	0.010
	LCS16	1198	1079.20	51.15	1028.05	1	0.001	0.000	0.008
year 2	LCS12	955	870.88	29.78	841.09	3	0.003	0.001	0.011
	LCS16	994	909.24	31.26	877.98	2	0.002	0.001	0.010
year 3	LCS12	794	721.39	37.50	683.89	2	0.003	0.001	0.011
	LCS16	831	759.48	23.44	736.05	3	0.004	0.001	0.012
2 years	LCS12	1187	1923.44	78.95	1844.48	7	0.007	0.003	0.015
	LCS16	1198	1988.44	82.41	1906.03	3	0.003	0.001	0.011
3 years	LCS12	1187	2644.82	116.45	2528.38	9	0.010	0.005	0.019
	LCS16	1198	2747.92	105.84	2642.08	6	0.007	0.003	0.016

Note: WY = women years, 1 WY equals to 365 days, CIL = confidence interval limit
Global Biostatistics: /by-sasp/patdb/projects/de04209/310442/stat/prod_interim03/pgms/i-eff-km-bmi.sas sgrpp 27OCT2011 12:53

Table 14.2.1 / 13: Probability of getting pregnant by treatment and BMI-group (Kaplan-Meier analysis) (FAS) (cont.)

Analysis type: adj.
BMI group: >= 30 kg/m2

Time	TREATMENT	Number of subjects	Total exposure [WY]	Excluded exposure [WY]	Relevant exposure [WY]	Number of pregnancies	Cumulative probability	lower 95% CIL	upper 95% CIL
year 1	LCS12	244	216.86	12.34	204.52	1	0.005	0.001	0.034
	LCS16	250	218.15	11.13	207.02	1	0.005	0.001	0.037
year 2	LCS12	199	176.76	10.30	166.47	0	0.000	0.000	0.000
	LCS16	201	183.80	6.01	177.79	2	0.010	0.003	0.041
year 3	LCS12	158	141.98	7.24	134.74	0	0.000	0.000	0.000
	LCS16	167	150.23	3.48	146.75	1	0.006	0.001	0.044
2 years	LCS12	244	393.62	22.64	370.99	1	0.005	0.001	0.033
	LCS16	250	401.95	17.13	384.81	3	0.016	0.005	0.048
3 years	LCS12	244	535.61	29.88	505.73	1	0.005	0.001	0.033
	LCS16	250	552.17	20.61	531.56	4	0.022	0.008	0.058

Note: WY = women years, 1 WY equals to 365 days, CIL = confidence interval limit
Global Biostatistics: /by-sasp/patdb/projects/de04209/310442/stat/prod_interim03/pgms/i-eff-km-bmi.sas sgrpp 27OCT2011 12:53
End of table

Table 14.2.1 / 14: Probability of getting pregnant (ectopic pregnancies) by treatment (Kaplan-Meier analysis) (FAS)

Analysis type: unadj.

Time	TREATMENT	Number of subjects	Total exposure [WY]	Excluded exposure [WY]	Relevant exposure [WY]	Number of pregnancies	Cumulative probability	lower 95% CIL	upper 95% CIL
year 1	LCS12	1432	1280.22	62.44	1217.78	2	0.002	0.000	0.006
	LCS16	1452	1316.37	63.59	1252.78	2	0.002	0.000	0.007
year 2	LCS12	1162	1056.98	41.31	1015.67	1	0.001	0.000	0.007
	LCS16	1206	1105.32	37.83	1067.49	3	0.003	0.001	0.009
year 3	LCS12	960	870.42	45.25	825.17	0	0.000	0.000	0.000
	LCS16	1010	918.30	27.21	891.09	2	0.002	0.001	0.009
2 years	LCS12	1432	2337.20	103.75	2233.45	3	0.002	0.001	0.008
	LCS16	1452	2421.69	101.42	2320.27	5	0.004	0.002	0.011
3 years	LCS12	1432	3207.62	149.00	3058.62	3	0.002	0.001	0.008
	LCS16	1452	3339.99	128.63	3211.36	7	0.007	0.003	0.014

Note: WY = women years, 1 WY equals to 365 days, CIL = confidence interval limit
Global Biostatistics: /by-sasp/patdb/projects/de04209/310442/stat/prod_interim03/pgms/i-eff-km-ect-all.sas sgrpp 27OCT2011 12:53

Table 14.2.1 / 14: Probability of getting pregnant (ectopic pregnancies) by treatment (Kaplan-Meier analysis) (FAS) (cont.)

Analysis type: adj.

Time	TREATMENT	Number of subjects	Total exposure [WY]	Excluded exposure [WY]	Relevant exposure [WY]	Number of pregnancies	Cumulative probability	lower 95% CIL	upper 95% CIL
year 1	LCS12	1432	1270.42	61.59	1208.82	2	0.002	0.000	0.006
	LCS16	1452	1300.92	62.29	1238.62	2	0.002	0.000	0.007
year 2	LCS12	1155	1048.64	40.08	1008.56	1	0.001	0.000	0.007
	LCS16	1198	1095.63	37.27	1058.36	3	0.003	0.001	0.009
year 3	LCS12	953	863.44	44.76	818.68	0	0.000	0.000	0.000
	LCS16	1000	911.70	26.92	884.78	2	0.002	0.001	0.009
2 years	LCS12	1432	2319.06	101.67	2217.38	3	0.003	0.001	0.008
	LCS16	1452	2396.55	99.56	2296.99	5	0.005	0.002	0.011
3 years	LCS12	1432	3182.50	146.43	3036.07	3	0.002	0.001	0.008
	LCS16	1452	3308.24	126.47	3181.77	7	0.007	0.003	0.014

Note: WY = women years, 1 WY equals to 365 days, CIL = confidence interval limit
Global Biostatistics: /by-sasp/patdb/projects/de04209/310442/stat/prod_interim03/pgms/i-eff-km-ect-all.sas sgrpp 27OCT2011 12:53
End of table

Table 14.2.1 / 15: Probability of getting pregnant (ectopic pregnancies) by treatment and age-group (Kaplan-Meier analysis) (FAS)

Analysis type: unadj.
Age category: age <= 25

Time	TREATMENT	Number of subjects	Total exposure [WY]	Excluded exposure [WY]	Relevant exposure [WY]	Number of pregnancies	Cumulative probability	lower 95% CIL	upper 95% CIL
year 1	LCS12	566	491.49	35.87	455.62	1	0.002	0.000	0.013
	LCS16	564	508.48	35.16	473.33	1	0.002	0.000	0.018
year 2	LCS12	435	392.29	24.00	368.29	1	0.002	0.000	0.018
	LCS16	461	423.65	23.01	400.65	0	0.000	0.000	0.000
year 3	LCS12	354	315.24	24.95	290.30	0	0.000	0.000	0.000
	LCS16	383	347.48	14.27	333.21	1	0.003	0.000	0.019
2 years	LCS12	566	883.78	59.87	823.92	2	0.004	0.001	0.018
	LCS16	564	932.14	58.16	873.98	1	0.002	0.000	0.015
3 years	LCS12	566	1199.03	84.82	1114.21	2	0.004	0.001	0.018
	LCS16	564	1279.62	72.43	1207.19	2	0.005	0.001	0.019

Note: WY = women years, 1 WY equals to 365 days, CIL = confidence interval limit
Global Biostatistics: /by-sasp/patdb/projects/de04209/310442/stat/prod_interim03/pgms/i-eff-km-ect-age.sas sgrpp 27OCT2011 12:54

Table 14.2.1 / 15: Probability of getting pregnant (ectopic pregnancies) by treatment and age-group (Kaplan-Meier analysis) (FAS) (cont.)

Analysis type: unadj.
Age category: 25 < age <= 35

Time	TREATMENT	Number of subjects	Total exposure [WY]	Excluded exposure [WY]	Relevant exposure [WY]	Number of pregnancies	Cumulative probability	lower 95% CIL	upper 95% CIL
year 1	LCS12	866	788.73	26.57	762.15	1	0.001	0.000	0.010
	LCS16	888	807.88	28.43	779.45	1	0.001	0.000	0.010
year 2	LCS12	727	664.69	17.31	647.38	0	0.000	0.000	0.000
	LCS16	745	681.67	14.82	666.84	3	0.005	0.001	0.014
year 3	LCS12	606	555.18	20.30	534.87	0	0.000	0.000	0.000
	LCS16	627	570.82	12.94	557.88	1	0.002	0.000	0.013
2 years	LCS12	866	1453.42	43.88	1409.54	1	0.001	0.000	0.009
	LCS16	888	1489.55	43.26	1446.29	4	0.006	0.002	0.016
3 years	LCS12	866	2008.59	64.18	1944.41	1	0.001	0.000	0.009
	LCS16	888	2060.37	56.19	2004.17	5	0.008	0.003	0.018

Note: WY = women years, 1 WY equals to 365 days, CIL = confidence interval limit
Global Biostatistics: /by-sasp/patdb/projects/de04209/310442/stat/prod_interim03/pgms/i-eff-km-ect-age.sas sgrpp 27OCT2011 12:54

Table 14.2.1 / 15: Probability of getting pregnant (ectopic pregnancies) by treatment and age-group (Kaplan-Meier analysis) (FAS) (cont.)

Analysis type: adj.
Age category: age <= 25

Time	TREATMENT	Number of subjects	Total exposure [WY]	Excluded exposure [WY]	Relevant exposure [WY]	Number of pregnancies	Cumulative probability	lower 95% CIL	upper 95% CIL
year 1	LCS12	566	486.04	35.47	450.56	1	0.002	0.000	0.014
	LCS16	564	502.10	34.24	467.86	1	0.003	0.000	0.018
year 2	LCS12	432	388.32	23.35	364.97	1	0.003	0.000	0.018
	LCS16	458	417.98	22.70	395.28	0	0.000	0.000	0.000
year 3	LCS12	349	311.81	24.72	287.09	0	0.000	0.000	0.000
	LCS16	378	344.44	14.13	330.32	1	0.003	0.000	0.019
2 years	LCS12	566	874.36	58.83	815.53	2	0.004	0.001	0.018
	LCS16	564	920.07	56.93	863.14	1	0.002	0.000	0.015
3 years	LCS12	566	1186.17	83.55	1102.62	2	0.004	0.001	0.018
	LCS16	564	1264.52	71.06	1193.45	2	0.005	0.001	0.020

Note: WY = women years, 1 WY equals to 365 days, CIL = confidence interval limit
Global Biostatistics: /by-sasp/patdb/projects/de04209/310442/stat/prod_interim03/pgms/i-eff-km-ect-age.sas sgrpp 27OCT2011 12:54

Table 14.2.1 / 15: Probability of getting pregnant (ectopic pregnancies) by treatment and age-group (Kaplan-Meier analysis) (FAS) (cont.)

Analysis type: adj.
Age category: 25 < age <= 35

Time	TREATMENT	Number of subjects	Total exposure [WY]	Excluded exposure [WY]	Relevant exposure [WY]	Number of pregnancies	Cumulative probability	lower 95% CIL	upper 95% CIL
year 1	LCS12	866	784.38	26.12	758.26	1	0.001	0.000	0.010
	LCS16	888	798.82	28.05	770.76	1	0.001	0.000	0.010
year 2	LCS12	723	660.32	16.73	643.59	0	0.000	0.000	0.000
	LCS16	740	677.65	14.57	663.08	3	0.005	0.001	0.014
year 3	LCS12	604	551.63	20.04	531.59	0	0.000	0.000	0.000
	LCS16	622	567.25	12.79	554.47	1	0.002	0.000	0.013
2 years	LCS12	866	1444.70	42.85	1401.85	1	0.001	0.000	0.009
	LCS16	888	1476.47	42.62	1433.85	4	0.006	0.002	0.016
3 years	LCS12	866	1996.33	62.88	1933.44	1	0.001	0.000	0.009
	LCS16	888	2043.73	55.41	1988.31	5	0.008	0.003	0.018

Note: WY = women years, 1 WY equals to 365 days, CIL = confidence interval limit
Global Biostatistics: /by-sasp/patdb/projects/de04209/310442/stat/prod_interim03/pgms/i-eff-km-ect-age.sas sgrpp 27OCT2011 12:54
End of table

Table 14.2.1 / 16: Probability of getting pregnant (ectopic pregnancies) by treatment and parity (Kaplan-Meier analysis) (FAS)

Analysis type: unadj.
Parity: 0 births

Time	TREATMENT	Number of subjects	Total exposure [WY]	Excluded exposure [WY]	Relevant exposure [WY]	Number of pregnancies	Cumulative probability	lower 95% CIL	upper 95% CIL
year 1	LCS12	556	489.40	42.52	446.88	1	0.002	0.000	0.014
	LCS16	574	508.51	35.12	473.39	0	0.000	0.000	0.000
year 2	LCS12	438	395.52	28.17	367.34	1	0.003	0.000	0.018
	LCS16	459	420.01	21.54	398.47	1	0.003	0.000	0.018
year 3	LCS12	354	323.63	27.22	296.41	0	0.000	0.000	0.000
	LCS16	380	350.26	16.78	333.48	2	0.006	0.001	0.023
2 years	LCS12	556	884.92	70.69	814.22	2	0.004	0.001	0.018
	LCS16	574	928.52	56.66	871.86	1	0.003	0.000	0.020
3 years	LCS12	556	1208.55	97.92	1110.63	2	0.004	0.001	0.018
	LCS16	574	1278.78	73.44	1205.33	3	0.008	0.003	0.026

Note: WY = women years, 1 WY equals to 365 days, CIL = confidence interval limit
GB: /by-sasp/patdb/projects/de04209/310442/stat/prod_interim03/pgms/i-eff-km-ect-parity.sas sgrpp 27OCT2011 12:54

Table 14.2.1 / 16: Probability of getting pregnant (ectopic pregnancies) by treatment and parity (Kaplan-Meier analysis) (FAS) (cont.)

Analysis type: unadj.
Parity: 1 birth or more

Time	TREATMENT	Number of subjects	Total exposure [WY]	Excluded exposure [WY]	Relevant exposure [WY]	Number of pregnancies	Cumulative probability	lower 95% CIL	upper 95% CIL
year 1	LCS12	876	790.82	19.92	770.90	1	0.001	0.000	0.009
	LCS16	878	807.86	28.47	779.38	2	0.003	0.001	0.011
year 2	LCS12	724	661.47	13.14	648.33	0	0.000	0.000	0.000
	LCS16	747	685.32	16.29	669.03	2	0.003	0.001	0.012
year 3	LCS12	606	546.79	18.03	528.76	0	0.000	0.000	0.000
	LCS16	630	568.04	10.42	557.62	0	0.000	0.000	0.000
2 years	LCS12	876	1452.29	33.06	1419.23	1	0.001	0.000	0.009
	LCS16	878	1493.17	44.76	1448.41	4	0.006	0.002	0.015
3 years	LCS12	876	1999.07	51.08	1947.99	1	0.001	0.000	0.009
	LCS16	878	2061.21	55.19	2006.03	4	0.005	0.002	0.015

Note: WY = women years, 1 WY equals to 365 days, CIL = confidence interval limit
GB: /by-sasp/patdb/projects/de04209/310442/stat/prod_interim03/pgms/i-eff-km-ect-parity.sas sgrpp 27OCT2011 12:54

Table 14.2.1 / 16: Probability of getting pregnant (ectopic pregnancies) by treatment and parity (Kaplan-Meier analysis) (FAS) (cont.)

Analysis type: adj.
Parity: 0 births

Time	TREATMENT	Number of subjects	Total exposure [WY]	Excluded exposure [WY]	Relevant exposure [WY]	Number of pregnancies	Cumulative probability	lower 95% CIL	upper 95% CIL
year 1	LCS12	556	485.87	42.09	443.78	1	0.002	0.000	0.014
	LCS16	574	502.21	34.22	467.99	0	0.000	0.000	0.000
year 2	LCS12	436	391.53	27.55	363.99	1	0.003	0.000	0.018
	LCS16	456	416.31	21.37	394.93	1	0.003	0.000	0.019
year 3	LCS12	351	320.55	27.04	293.51	0	0.000	0.000	0.000
	LCS16	376	348.08	16.65	331.44	2	0.006	0.001	0.023
2 years	LCS12	556	877.41	69.64	807.77	2	0.005	0.001	0.018
	LCS16	574	918.52	55.60	862.92	1	0.003	0.000	0.020
3 years	LCS12	556	1197.95	96.68	1101.28	2	0.004	0.001	0.018
	LCS16	574	1266.60	72.24	1194.36	3	0.008	0.003	0.026

Note: WY = women years, 1 WY equals to 365 days, CIL = confidence interval limit
GB: /by-sasp/patdb/projects/de04209/310442/stat/prod_interim03/pgms/i-eff-km-ect-parity.sas sgrpp 27OCT2011 12:54

Table 14.2.1 / 16: Probability of getting pregnant (ectopic pregnancies) by treatment and parity (Kaplan-Meier analysis) (FAS) (cont.)

Analysis type: adj.
Parity: 1 birth or more

Time	TREATMENT	Number of subjects	Total exposure [WY]	Excluded exposure [WY]	Relevant exposure [WY]	Number of pregnancies	Cumulative probability	lower 95% CIL	upper 95% CIL
year 1	LCS12	876	784.55	19.50	765.04	1	0.001	0.000	0.010
	LCS16	878	798.70	28.07	770.64	2	0.003	0.001	0.011
year 2	LCS12	719	657.10	12.53	644.57	0	0.000	0.000	0.000
	LCS16	742	679.32	15.90	663.43	2	0.003	0.001	0.012
year 3	LCS12	602	542.89	17.72	525.18	0	0.000	0.000	0.000
	LCS16	624	563.61	10.27	553.35	0	0.000	0.000	0.000
2 years	LCS12	876	1441.65	32.04	1409.61	1	0.001	0.000	0.009
	LCS16	878	1478.03	43.96	1434.07	4	0.006	0.002	0.015
3 years	LCS12	876	1984.54	49.75	1934.79	1	0.001	0.000	0.009
	LCS16	878	2041.64	54.23	1987.41	4	0.006	0.002	0.015

Note: WY = women years, 1 WY equals to 365 days, CIL = confidence interval limit
GB: /by-sasp/patdb/projects/de04209/310442/stat/prod_interim03/pgms/i-eff-km-ect-parity.sas sgrpp 27OCT2011 12:54
End of table

Table 14.2.1 / 17: Probability of getting pregnant (ectopic pregnancies) by treatment and BMI-group (Kaplan-Meier analysis) (FAS)

Analysis type: unadj.
BMI group: missing

Time	TREATMENT	Number of subjects	Total exposure [WY]	Excluded exposure [WY]	Relevant exposure [WY]	Number of pregnancies	Cumulative probability	lower 95% CIL	upper 95% CIL
year 1	LCS12	1	1.00	0.08	0.92	0	0.000	0.000	0.000
	LCS16	4	3.57	0.02	3.55	0	0.000	0.000	0.000
year 2	LCS12	1	1.00	0.00	1.00	0	0.000	0.000	0.000
	LCS16	3	2.59	0.00	2.59	0	0.000	0.000	0.000
year 3	LCS12	1	0.07	0.02	0.05	0	0.000	0.000	0.000
	LCS16	2	1.99	0.00	1.99	0	0.000	0.000	0.000
2 years	LCS12	1	2.00	0.08	1.92	0	0.000	0.000	0.000
	LCS16	4	6.16	0.02	6.15	0	0.000	0.000	0.000
3 years	LCS12	1	2.07	0.10	1.96	0	0.000	0.000	0.000
	LCS16	4	8.15	0.02	8.13	0	0.000	0.000	0.000

Note: WY = women years, 1 WY equals to 365 days, CIL = confidence interval limit
Global Biostatistics: /by-sasp/patdb/projects/de04209/310442/stat/prod_interim03/pgms/i-eff-km-ect-bmi.sas sgrpp 27OCT2011 12:54

Table 14.2.1 / 17: Probability of getting pregnant (ectopic pregnancies) by treatment and BMI-group (Kaplan-Meier analysis) (FAS) (cont.)

Analysis type: unadj.
BMI group: < 30 kg/m2

Time	TREATMENT	Number of subjects	Total exposure [WY]	Excluded exposure [WY]	Relevant exposure [WY]	Number of pregnancies	Cumulative probability	lower 95% CIL	upper 95% CIL
year 1	LCS12	1187	1059.53	49.80	1009.73	2	0.002	0.000	0.008
	LCS16	1198	1090.09	51.99	1038.10	1	0.001	0.000	0.008
year 2	LCS12	960	879.05	30.98	848.07	1	0.001	0.000	0.008
	LCS16	1000	917.49	31.77	885.72	2	0.002	0.001	0.009
year 3	LCS12	801	727.47	37.96	689.51	0	0.000	0.000	0.000
	LCS16	840	764.71	23.71	741.01	2	0.003	0.001	0.010
2 years	LCS12	1187	1938.58	80.77	1857.81	3	0.003	0.001	0.009
	LCS16	1198	2007.58	83.76	1923.81	3	0.003	0.001	0.010
3 years	LCS12	1187	2666.05	118.73	2547.32	3	0.003	0.001	0.009
	LCS16	1198	2772.29	107.47	2664.82	5	0.006	0.002	0.014

Note: WY = women years, 1 WY equals to 365 days, CIL = confidence interval limit
Global Biostatistics: /by-sasp/patdb/projects/de04209/310442/stat/prod_interim03/pgms/i-eff-km-ect-bmi.sas sgrpp 27OCT2011 12:54

Table 14.2.1 / 17: Probability of getting pregnant (ectopic pregnancies) by treatment and BMI-group (Kaplan-Meier analysis) (FAS) (cont.)

Analysis type: unadj.
BMI group: >= 30 kg/m2

Time	TREATMENT	Number of subjects	Total exposure [WY]	Excluded exposure [WY]	Relevant exposure [WY]	Number of pregnancies	Cumulative probability	lower 95% CIL	upper 95% CIL
year 1	LCS12	244	219.69	12.56	207.13	0	0.000	0.000	0.000
	LCS16	250	222.71	11.58	211.13	1	0.005	0.001	0.037
year 2	LCS12	201	176.93	10.33	166.60	0	0.000	0.000	0.000
	LCS16	203	185.24	6.06	179.18	1	0.005	0.001	0.035
year 3	LCS12	158	142.88	7.27	135.61	0	0.000	0.000	0.000
	LCS16	168	151.60	3.50	148.10	0	0.000	0.000	0.000
2 years	LCS12	244	396.62	22.89	373.73	0	0.000	0.000	0.000
	LCS16	250	407.95	17.64	390.31	2	0.010	0.003	0.039
3 years	LCS12	244	539.51	30.16	509.34	0	0.000	0.000	0.000
	LCS16	250	559.55	21.14	538.41	2	0.010	0.002	0.039

Note: WY = women years, 1 WY equals to 365 days, CIL = confidence interval limit
Global Biostatistics: /by-sasp/patdb/projects/de04209/310442/stat/prod_interim03/pgms/i-eff-km-ect-bmi.sas sgrpp 27OCT2011 12:54

Table 14.2.1 / 17: Probability of getting pregnant (ectopic pregnancies) by treatment and BMI-group (Kaplan-Meier analysis) (FAS) (cont.)

Analysis type: adj.
BMI group: missing

Time	TREATMENT	Number of subjects	Total exposure [WY]	Excluded exposure [WY]	Relevant exposure [WY]	Number of pregnancies	Cumulative probability	lower 95% CIL	upper 95% CIL
year 1	LCS12	1	1.00	0.08	0.92	0	0.000	0.000	0.000
	LCS16	4	3.57	0.02	3.55	0	0.000	0.000	0.000
year 2	LCS12	1	1.00	0.00	1.00	0	0.000	0.000	0.000
	LCS16	3	2.59	0.00	2.59	0	0.000	0.000	0.000
year 3	LCS12	1	0.07	0.02	0.05	0	0.000	0.000	0.000
	LCS16	2	1.99	0.00	1.99	0	0.000	0.000	0.000
2 years	LCS12	1	2.00	0.08	1.92	0	0.000	0.000	0.000
	LCS16	4	6.16	0.02	6.15	0	0.000	0.000	0.000
3 years	LCS12	1	2.07	0.10	1.96	0	0.000	0.000	0.000
	LCS16	4	8.15	0.02	8.13	0	0.000	0.000	0.000

Note: WY = women years, 1 WY equals to 365 days, CIL = confidence interval limit
Global Biostatistics: /by-sasp/patdb/projects/de04209/310442/stat/prod_interim03/pgms/i-eff-km-ect-bmi.sas sgrpp 27OCT2011 12:54

Table 14.2.1 / 17: Probability of getting pregnant (ectopic pregnancies) by treatment and BMI-group (Kaplan-Meier analysis) (FAS) (cont.)

Analysis type: adj.
BMI group: < 30 kg/m2

Time	TREATMENT	Number of subjects	Total exposure [WY]	Excluded exposure [WY]	Relevant exposure [WY]	Number of pregnancies	Cumulative probability	lower 95% CIL	upper 95% CIL
year 1	LCS12	1187	1052.56	49.17	1003.39	2	0.002	0.000	0.008
	LCS16	1198	1079.20	51.15	1028.05	1	0.001	0.000	0.008
year 2	LCS12	955	870.88	29.78	841.09	1	0.001	0.000	0.008
	LCS16	994	909.24	31.26	877.98	2	0.002	0.001	0.010
year 3	LCS12	794	721.39	37.50	683.89	0	0.000	0.000	0.000
	LCS16	831	759.48	23.44	736.05	2	0.003	0.001	0.010
2 years	LCS12	1187	1923.44	78.95	1844.48	3	0.003	0.001	0.009
	LCS16	1198	1988.44	82.41	1906.03	3	0.003	0.001	0.011
3 years	LCS12	1187	2644.82	116.45	2528.38	3	0.003	0.001	0.009
	LCS16	1198	2747.92	105.84	2642.08	5	0.006	0.002	0.014

Note: WY = women years, 1 WY equals to 365 days, CIL = confidence interval limit
Global Biostatistics: /by-sasp/patdb/projects/de04209/310442/stat/prod_interim03/pgms/i-eff-km-ect-bmi.sas sgrpp 27OCT2011 12:54

Table 14.2.1 / 17: Probability of getting pregnant (ectopic pregnancies) by treatment and BMI-group (Kaplan-Meier analysis) (FAS) (cont.)

Analysis type: adj.
BMI group: >= 30 kg/m2

Time	TREATMENT	Number of subjects	Total exposure [WY]	Excluded exposure [WY]	Relevant exposure [WY]	Number of pregnancies	Cumulative probability	lower 95% CIL	upper 95% CIL
year 1	LCS12	244	216.86	12.34	204.52	0	0.000	0.000	0.000
	LCS16	250	218.15	11.13	207.02	1	0.005	0.001	0.037
year 2	LCS12	199	176.76	10.30	166.47	0	0.000	0.000	0.000
	LCS16	201	183.80	6.01	177.79	1	0.005	0.001	0.035
year 3	LCS12	158	141.98	7.24	134.74	0	0.000	0.000	0.000
	LCS16	167	150.23	3.48	146.75	0	0.000	0.000	0.000
2 years	LCS12	244	393.62	22.64	370.99	0	0.000	0.000	0.000
	LCS16	250	401.95	17.13	384.81	2	0.010	0.003	0.040
3 years	LCS12	244	535.61	29.88	505.73	0	0.000	0.000	0.000
	LCS16	250	552.17	20.61	531.56	2	0.010	0.003	0.039

Note: WY = women years, 1 WY equals to 365 days, CIL = confidence interval limit
Global Biostatistics: /by-sasp/patdb/projects/de04209/310442/stat/prod_interim03/pgms/i-eff-km-ect-bmi.sas sgrpp 27OCT2011 12:54
End of table

14.2.2 Bleeding pattern (not included)

14.2.3 Other efficacy variables

Table 14.2.3 / 1: User satisfaction questionnaire (FAS)

Volunteer satisf. questionn., questions	Volunteer satisfaction questionnaire	LCS12	LCS16	Total
study treat, overall satisfaction	n	1053 (100.0%)	1063 (100.0%)	2116 (100.0%)
	missing	1 (<0.1%)	1 (<0.1%)	2 (<0.1%)
	very satisfied	796 (75.6%)	842 (79.2%)	1638 (77.4%)
	somewhat satisfied	201 (19.1%)	177 (16.7%)	378 (17.9%)
	neither satisfied / dissatisfied	31 (2.9%)	22 (2.1%)	53 (2.5%)
	dissatisfied	23 (2.2%)	18 (1.7%)	41 (1.9%)
	very dissatisfied	1 (<0.1%)	3 (0.3%)	4 (0.2%)
change of regimen, likelihood	n	1053 (100.0%)	1063 (100.0%)	2116 (100.0%)
	missing	2 (0.2%)	1 (<0.1%)	3 (0.1%)
	continue with LCS	811 (77.0%)	872 (82.0%)	1683 (79.5%)
	use a different horm. contr.	86 (8.2%)	45 (4.2%)	131 (6.2%)
	use a different contr. meth.	79 (7.5%)	68 (6.4%)	147 (6.9%)
	discontinue use of all types of c	28 (2.7%)	27 (2.5%)	55 (2.6%)
	don't know	47 (4.5%)	50 (4.7%)	97 (4.6%)
menstrual bleeding, comparison	n	1053 (100.0%)	1063 (100.0%)	2116 (100.0%)
	missing	2 (0.2%)	1 (<0.1%)	3 (0.1%)
	decreased	913 (86.7%)	985 (92.7%)	1898 (89.7%)
	no change	85 (8.1%)	48 (4.5%)	133 (6.3%)
	increased	53 (5.0%)	29 (2.7%)	82 (3.9%)
menstrual bleeding pattern	n	1053 (100.0%)	1063 (100.0%)	2116 (100.0%)
	missing	2 (0.2%)	5 (0.5%)	7 (0.3%)
	very satisfied	514 (48.8%)	537 (50.5%)	1051 (49.7%)
	somewhat satisfied	293 (27.8%)	272 (25.6%)	565 (26.7%)
	neither satisfied / dissatisfied	100 (9.5%)	77 (7.2%)	177 (8.4%)
	dissatisfied	66 (6.3%)	59 (5.6%)	125 (5.9%)
	very dissatisfied	20 (1.9%)	13 (1.2%)	33 (1.6%)
	not applicable	58 (5.5%)	100 (9.4%)	158 (7.5%)
menstrual bleeding, unexpected	n	1053 (100.0%)	1063 (100.0%)	2116 (100.0%)
	missing	3 (0.3%)	1 (<0.1%)	4 (0.2%)
	never	342 (32.5%)	327 (30.8%)	669 (31.6%)
	seldom	589 (55.9%)	634 (59.6%)	1223 (57.8%)
	often	98 (9.3%)	79 (7.4%)	177 (8.4%)
	very often	21 (2.0%)	22 (2.1%)	43 (2.0%)

Table 14.2.3 / 1: User satisfaction questionnaire (FAS)

Volunteer satisf. questionn., questions	Volunteer satisfaction questionnaire	LCS12	LCS16	Total
menstrual bleeding, absence	n	1053 (100.0%)	1063 (100.0%)	2116 (100.0%)
	missing	918 (87.2%)	833 (78.4%)	1751 (82.8%)
	very satisfied	115 (10.9%)	189 (17.8%)	304 (14.4%)
	somewhat satisfied	12 (1.1%)	26 (2.4%)	38 (1.8%)
	neither satisfied / dissatisfied	6 (0.6%)	11 (1.0%)	17 (0.8%)
	dissatisfied	2 (0.2%)	4 (0.4%)	6 (0.3%)
menstrual pain, treatment	n	1053 (100.0%)	1063 (100.0%)	2116 (100.0%)
	missing	3 (0.3%)	1 (<0.1%)	4 (0.2%)
	none	425 (40.4%)	448 (42.1%)	873 (41.3%)
	mild	469 (44.5%)	480 (45.2%)	949 (44.8%)
	moderate	130 (12.3%)	119 (11.2%)	249 (11.8%)
	severe	26 (2.5%)	15 (1.4%)	41 (1.9%)
menstrual pain, comparison	n	1053 (100.0%)	1063 (100.0%)	2116 (100.0%)
	missing	139 (13.2%)	114 (10.7%)	253 (12.0%)
	decreased	602 (57.2%)	681 (64.1%)	1283 (60.6%)
	no change	195 (18.5%)	197 (18.5%)	392 (18.5%)
	increased	117 (11.1%)	71 (6.7%)	188 (8.9%)

Note: Only collected after Amendment 3 to study protocol
Global Biostatistics: /by-sasp/patdb/projects/de04209/310442/stat/prod_interim03/pgms/t-usq.sas sgrpp 27OCT2011 13:09
End of table

14.3 Safety

14.3.1 Analyses of adverse events

Table 14.3.1 / 1: Number of subjects by number of baseline findings (FAS)

	LCS12	LCS16	Total
Number of subjects	1432 (100.0%)	1452 (100.0%)	2884 (100.0%)
Number of Baseline Findings			
0	702 (49.0%)	685 (47.2%)	1387 (48.1%)
1	391 (27.3%)	392 (27.0%)	783 (27.1%)
2	172 (12.0%)	197 (13.6%)	369 (12.8%)
3	90 (6.3%)	97 (6.7%)	187 (6.5%)
4	44 (3.1%)	45 (3.1%)	89 (3.1%)
5	21 (1.5%)	17 (1.2%)	38 (1.3%)
6	8 (0.6%)	8 (0.6%)	16 (0.6%)
7	2 (0.1%)	5 (0.3%)	7 (0.2%)
8	1 (<0.1%)	3 (0.2%)	4 (0.1%)
9	0	2 (0.1%)	2 (<0.1%)
=> 10	1 (<0.1%)	1 (<0.1%)	2 (<0.1%)

Global Biostatistics: /by-sasp/patdb/projects/de04209/310442/stat/prod_interim03/pgms/t-basfin.sas epkll 12OCT2011 11:19
End of table

Table 14.3.1 / 2: Number of subjects with baseline findings and number of baseline findings by MedDRA SOC/PT (FAS)

Primary system organ class<br>Preferred term<br>MedDRA version 14.0	LCS12<br>Events	 N=1432 (100%)	LCS16<br>Events	 N=1452 (100%)	Total<br>Events	 N=2884 (100%)
ANY EVENT	1367	730 (51.0%)	1478	767 (52.8%)	2845	1497 (51.9%)
Blood and lymphatic system disorders	13	13 (0.9%)	27	26 (1.8%)	40	39 (1.4%)
Anaemia	10	10 (0.7%)	23	23 (1.6%)	33	33 (1.1%)
Coagulopathy	1	1 (<0.1%)	0	0	1	1 (<0.1%)
Haemoconcentration	0	0	1	1 (<0.1%)	1	1 (<0.1%)
Iron deficiency anaemia	0	0	1	1 (<0.1%)	1	1 (<0.1%)
Lymphadenopathy	0	0	2	2 (0.1%)	2	2 (<0.1%)
Pernicious anaemia	1	1 (<0.1%)	0	0	1	1 (<0.1%)
Splenomegaly	1	1 (<0.1%)	0	0	1	1 (<0.1%)
Cardiac disorders	4	4 (0.3%)	10	9 (0.6%)	14	13 (0.5%)
Arrhythmia	0	0	2	2 (0.1%)	2	2 (<0.1%)
Extrasystoles	1	1 (<0.1%)	1	1 (<0.1%)	2	2 (<0.1%)
Long QT syndrome	0	0	1	1 (<0.1%)	1	1 (<0.1%)
Mitral valve incompetence	0	0	1	1 (<0.1%)	1	1 (<0.1%)
Mitral valve prolapse	3	3 (0.2%)	2	2 (0.1%)	5	5 (0.2%)
Palpitations	0	0	1	1 (<0.1%)	1	1 (<0.1%)
Tachycardia	0	0	2	2 (0.1%)	2	2 (<0.1%)
Congenital, familial and genetic disorders	5	5 (0.3%)	8	8 (0.6%)	13	13 (0.5%)
Bicuspid aortic valve	0	0	1	1 (<0.1%)	1	1 (<0.1%)
Dermoid cyst	0	0	1	1 (<0.1%)	1	1 (<0.1%)
Ehlers-Danlos syndrome	1	1 (<0.1%)	0	0	1	1 (<0.1%)
Factor V Leiden mutation	1	1 (<0.1%)	1	1 (<0.1%)	2	2 (<0.1%)
Gilbert's syndrome	1	1 (<0.1%)	0	0	1	1 (<0.1%)
Haemangioma congenital	1	1 (<0.1%)	0	0	1	1 (<0.1%)
Peroneal muscular atrophy	0	0	1	1 (<0.1%)	1	1 (<0.1%)
Sickle cell trait	0	0	1	1 (<0.1%)	1	1 (<0.1%)
Ventricular septal defect	1	1 (<0.1%)	0	0	1	1 (<0.1%)
Von Willebrand's disease	0	0	3	3 (0.2%)	3	3 (0.1%)

Table 14.3.1 / 2: Number of subjects with baseline findings and number of baseline findings by MedDRA SOC/PT (FAS)

Primary system organ class<br>Preferred term<br>MedDRA version 14.0	LCS12<br>Events	 N=1432 (100%)	LCS16<br>Events	 N=1452 (100%)	Total<br>Events	 N=2884 (100%)
Ear and labyrinth disorders	12	10 (0.7%)	5	5 (0.3%)	17	15 (0.5%)
Cerumen impaction	0	0	1	1 (<0.1%)	1	1 (<0.1%)
Deafness bilateral	1	1 (<0.1%)	0	0	1	1 (<0.1%)
Ear discomfort	1	1 (<0.1%)	0	0	1	1 (<0.1%)
Hypoacusis	1	1 (<0.1%)	0	0	1	1 (<0.1%)
Meniere's disease	2	2 (0.1%)	0	0	2	2 (<0.1%)
Motion sickness	1	1 (<0.1%)	0	0	1	1 (<0.1%)
Tinnitus	2	2 (0.1%)	2	2 (0.1%)	4	4 (0.1%)
Tympanic membrane perforation	2	2 (0.1%)	0	0	2	2 (<0.1%)
Tympanic membrane scarring	1	1 (<0.1%)	0	0	1	1 (<0.1%)
Vertigo	1	1 (<0.1%)	2	2 (0.1%)	3	3 (0.1%)
Endocrine disorders	31	31 (2.2%)	33	32 (2.2%)	64	63 (2.2%)
Goitre	2	2 (0.1%)	2	2 (0.1%)	4	4 (0.1%)
Hyperprolactinaemia	0	0	2	2 (0.1%)	2	2 (<0.1%)
Hyperthyroidism	3	3 (0.2%)	1	1 (<0.1%)	4	4 (0.1%)
Hypothyroidism	25	25 (1.7%)	26	26 (1.8%)	51	51 (1.8%)
Thyroiditis	1	1 (<0.1%)	2	2 (0.1%)	3	3 (0.1%)
Eye disorders	53	50 (3.5%)	58	55 (3.8%)	111	105 (3.6%)
Asthenopia	1	1 (<0.1%)	0	0	1	1 (<0.1%)
Astigmatism	2	2 (0.1%)	5	5 (0.3%)	7	7 (0.2%)
Chalazion	0	0	1	1 (<0.1%)	1	1 (<0.1%)
Conjunctivitis	1	1 (<0.1%)	2	2 (0.1%)	3	3 (0.1%)
Conjunctivitis allergic	0	0	1	1 (<0.1%)	1	1 (<0.1%)
Dry eye	0	0	2	2 (0.1%)	2	2 (<0.1%)
Ectropion	0	0	1	1 (<0.1%)	1	1 (<0.1%)
Eyelid ptosis	1	1 (<0.1%)	0	0	1	1 (<0.1%)
Hypermetropia	6	6 (0.4%)	6	6 (0.4%)	12	12 (0.4%)
Keratoconus	1	1 (<0.1%)	0	0	1	1 (<0.1%)
Myopia	40	40 (2.8%)	39	39 (2.7%)	79	79 (2.7%)
Presbyopia	0	0	1	1 (<0.1%)	1	1 (<0.1%)
Ulcerative keratitis	1	1 (<0.1%)	0	0	1	1 (<0.1%)

Table 14.3.1 / 2: Number of subjects with baseline findings and number of baseline findings by MedDRA SOC/PT (FAS)

Primary system organ class<br>Preferred term<br>MedDRA version 14.0	LCS12<br>Events	 N=1432 (100%)	LCS16<br>Events	 N=1452 (100%)	Total<br>Events	 N=2884 (100%)
Gastrointestinal disorders	79	70 (4.9%)	64	58 (4.0%)	143	128 (4.4%)
Abdominal discomfort	0	0	1	1 (<0.1%)	1	1 (<0.1%)
Abdominal distension	2	2 (0.1%)	0	0	2	2 (<0.1%)
Abdominal pain	6	6 (0.4%)	4	4 (0.3%)	10	10 (0.3%)
Abdominal pain lower	0	0	2	2 (0.1%)	2	2 (<0.1%)
Abdominal pain upper	0	0	1	1 (<0.1%)	1	1 (<0.1%)
Anal fissure	1	1 (<0.1%)	0	0	1	1 (<0.1%)
Coeliac disease	2	2 (0.1%)	2	2 (0.1%)	4	4 (0.1%)
Colitis	1	1 (<0.1%)	0	0	1	1 (<0.1%)
Colitis ulcerative	4	4 (0.3%)	2	2 (0.1%)	6	6 (0.2%)
Constipation	6	6 (0.4%)	9	9 (0.6%)	15	15 (0.5%)
Crohn's disease	2	2 (0.1%)	1	1 (<0.1%)	3	3 (0.1%)
Diarrhoea	2	2 (0.1%)	3	3 (0.2%)	5	5 (0.2%)
Dyspepsia	8	8 (0.6%)	4	4 (0.3%)	12	12 (0.4%)
Faecal incontinence	1	1 (<0.1%)	0	0	1	1 (<0.1%)
Flatulence	3	3 (0.2%)	0	0	3	3 (0.1%)
Food poisoning	0	0	1	1 (<0.1%)	1	1 (<0.1%)
Gastric disorder	1	1 (<0.1%)	0	0	1	1 (<0.1%)
Gastritis	6	6 (0.4%)	4	4 (0.3%)	10	10 (0.3%)
Gastrooesophageal reflux disease	5	5 (0.3%)	11	11 (0.8%)	16	16 (0.6%)
Glossitis	1	1 (<0.1%)	0	0	1	1 (<0.1%)
Haemorrhoids	2	2 (0.1%)	1	1 (<0.1%)	3	3 (0.1%)
Hiatus hernia	2	2 (0.1%)	1	1 (<0.1%)	3	3 (0.1%)
Inguinal hernia	1	1 (<0.1%)	0	0	1	1 (<0.1%)
Irritable bowel syndrome	12	12 (0.8%)	8	8 (0.6%)	20	20 (0.7%)
Malocclusion	1	1 (<0.1%)	0	0	1	1 (<0.1%)
Nausea	4	4 (0.3%)	4	4 (0.3%)	8	8 (0.3%)
Oesophagitis	0	0	1	1 (<0.1%)	1	1 (<0.1%)
Periodontitis	0	0	1	1 (<0.1%)	1	1 (<0.1%)
Proctalgia	1	1 (<0.1%)	0	0	1	1 (<0.1%)
Reflux oesophagitis	1	1 (<0.1%)	0	0	1	1 (<0.1%)
Toothache	1	1 (<0.1%)	1	1 (<0.1%)	2	2 (<0.1%)
Umbilical hernia	3	3 (0.2%)	1	1 (<0.1%)	4	4 (0.1%)
Vomiting	0	0	1	1 (<0.1%)	1	1 (<0.1%)

Table 14.3.1 / 2: Number of subjects with baseline findings and number of baseline findings by MedDRA SOC/PT (FAS)

Primary system organ class / Preferred term / MedDRA version 14.0	LCS12 Events	LCS12 N=1432 (100%)	LCS16 Events	LCS16 N=1452 (100%)	Total Events	Total N=2884 (100%)
General disorders and administration site conditions	15	15 (1.0%)	9	9 (0.6%)	24	24 (0.8%)
Asthenia	1	1 (<0.1%)	0	0	1	1 (<0.1%)
Chest pain	0	0	1	1 (<0.1%)	1	1 (<0.1%)
Cyst	0	0	1	1 (<0.1%)	1	1 (<0.1%)
Fatigue	4	4 (0.3%)	3	3 (0.2%)	7	7 (0.2%)
Influenza like illness	1	1 (<0.1%)	0	0	1	1 (<0.1%)
Irritability	1	1 (<0.1%)	1	1 (<0.1%)	2	2 (<0.1%)
Oedema peripheral	2	2 (0.1%)	1	1 (<0.1%)	3	3 (0.1%)
Pain	5	5 (0.3%)	1	1 (<0.1%)	6	6 (0.2%)
Pyrexia	1	1 (<0.1%)	1	1 (<0.1%)	2	2 (<0.1%)
Hepatobiliary disorders	1	1 (<0.1%)	5	5 (0.3%)	6	6 (0.2%)
Cholelithiasis	1	1 (<0.1%)	3	3 (0.2%)	4	4 (0.1%)
Hepatic steatosis	0	0	2	2 (0.1%)	2	2 (<0.1%)
Immune system disorders	65	51 (3.6%)	67	51 (3.5%)	132	102 (3.5%)
Allergy to animal	3	3 (0.2%)	9	8 (0.6%)	12	11 (0.4%)
Allergy to arthropod sting	2	2 (0.1%)	0	0	2	2 (<0.1%)
Allergy to chemicals	0	0	1	1 (<0.1%)	1	1 (<0.1%)
Allergy to metals	3	3 (0.2%)	2	1 (<0.1%)	5	4 (0.1%)
Atopy	1	1 (<0.1%)	0	0	1	1 (<0.1%)
Drug hypersensitivity	8	5 (0.3%)	7	6 (0.4%)	15	11 (0.4%)
Food allergy	3	3 (0.2%)	6	4 (0.3%)	9	7 (0.2%)
House dust allergy	6	6 (0.4%)	3	3 (0.2%)	9	9 (0.3%)
Hypersensitivity	11	11 (0.8%)	7	7 (0.5%)	18	18 (0.6%)
Latex allergy	0	0	2	2 (0.1%)	2	2 (<0.1%)
Milk allergy	1	1 (<0.1%)	1	1 (<0.1%)	2	2 (<0.1%)
Multiple allergies	1	1 (<0.1%)	1	1 (<0.1%)	2	2 (<0.1%)
Seasonal allergy	26	26 (1.8%)	28	28 (1.9%)	54	54 (1.9%)

Table 14.3.1 / 2: Number of subjects with baseline findings and number of baseline findings by MedDRA SOC/PT (FAS)

Primary system organ class / Preferred term / MedDRA version 14.0	LCS12 Events	LCS12 N=1432 (100%)	LCS16 Events	LCS16 N=1452 (100%)	Total Events	Total N=2884 (100%)
Infections and infestations	194	168 (11.7%)	182	158 (10.9%)	376	326 (11.3%)
Acute tonsillitis	1	1 (<0.1%)	1	1 (<0.1%)	2	2 (<0.1%)
Anogenital warts	1	1 (<0.1%)	1	1 (<0.1%)	2	2 (<0.1%)
Body tinea	1	1 (<0.1%)	0	0	1	1 (<0.1%)
Bronchitis	5	5 (0.3%)	5	5 (0.3%)	10	10 (0.3%)
Cellulitis	0	0	1	1 (<0.1%)	1	1 (<0.1%)
Cervicitis	2	2 (0.1%)	1	1 (<0.1%)	3	3 (0.1%)
Cervicitis gonococcal	1	1 (<0.1%)	0	0	1	1 (<0.1%)
Chlamydial cervicitis	6	6 (0.4%)	5	5 (0.3%)	11	11 (0.4%)
Chlamydial infection	3	3 (0.2%)	3	3 (0.2%)	6	6 (0.2%)
Chronic sinusitis	2	2 (0.1%)	0	0	2	2 (<0.1%)
Cystitis	5	4 (0.3%)	1	1 (<0.1%)	6	5 (0.2%)
Ear infection	1	1 (<0.1%)	1	1 (<0.1%)	2	2 (<0.1%)
Folliculitis	1	1 (<0.1%)	1	1 (<0.1%)	2	2 (<0.1%)
Fungal infection	1	1 (<0.1%)	5	5 (0.3%)	6	6 (0.2%)
Fungal skin infection	1	1 (<0.1%)	0	0	1	1 (<0.1%)
Gastroenteritis	2	2 (0.1%)	2	2 (0.1%)	4	4 (0.1%)
Genital candidiasis	0	0	1	1 (<0.1%)	1	1 (<0.1%)
Genital herpes	4	4 (0.3%)	9	9 (0.6%)	13	13 (0.5%)
Genital infection bacterial	1	1 (<0.1%)	0	0	1	1 (<0.1%)
Gonorrhoea	1	1 (<0.1%)	1	1 (<0.1%)	2	2 (<0.1%)
Gynaecological chlamydia infection	2	2 (0.1%)	0	0	2	2 (<0.1%)
Herpes dermatitis	1	1 (<0.1%)	0	0	1	1 (<0.1%)
Herpes simplex	2	2 (0.1%)	1	1 (<0.1%)	3	3 (0.1%)
Infection	0	0	1	1 (<0.1%)	1	1 (<0.1%)
Influenza	11	11 (0.8%)	5	5 (0.3%)	16	16 (0.6%)
Lactobacillus infection	1	1 (<0.1%)	0	0	1	1 (<0.1%)
Laryngitis	2	2 (0.1%)	1	1 (<0.1%)	3	3 (0.1%)
Localised infection	1	1 (<0.1%)	0	0	1	1 (<0.1%)
Molluscum contagiosum	1	1 (<0.1%)	1	1 (<0.1%)	2	2 (<0.1%)
Nasopharyngitis	15	15 (1.0%)	24	22 (1.5%)	39	37 (1.3%)
Onychomycosis	2	2 (0.1%)	3	3 (0.2%)	5	5 (0.2%)
Oral herpes	3	3 (0.2%)	6	6 (0.4%)	9	9 (0.3%)
Otitis media	2	2 (0.1%)	0	0	2	2 (<0.1%)
Otitis media bacterial	1	1 (<0.1%)	0	0	1	1 (<0.1%)
Papilloma viral infection	1	1 (<0.1%)	0	0	1	1 (<0.1%)
Pelvic infection	0	0	1	1 (<0.1%)	1	1 (<0.1%)
Pharyngitis	5	5 (0.3%)	3	3 (0.2%)	8	8 (0.3%)
Pharyngitis streptococcal	1	1 (<0.1%)	2	2 (0.1%)	3	3 (0.1%)
Pneumonia	0	0	3	3 (0.2%)	3	3 (0.1%)
Respiratory tract infection	2	2 (0.1%)	2	2 (0.1%)	4	4 (0.1%)
Rhinitis	2	2 (0.1%)	1	1 (<0.1%)	3	3 (0.1%)
Sinusitis	14	14 (1.0%)	14	14 (1.0%)	28	28 (1.0%)

Table 14.3.1 / 2: Number of subjects with baseline findings and number of baseline findings by MedDRA SOC/PT (FAS)

Primary system organ class<br>Preferred term<br>MedDRA version 14.0	LCS12<br>Events	LCS12<br>N=1432 (100%)	LCS16<br>Events	LCS16<br>N=1452 (100%)	Total<br>Events	Total<br>N=2884 (100%)
Skin infection	3	3 (0.2%)	1	1 (<0.1%)	4	4 (0.1%)
Staphylococcal skin infection	1	1 (<0.1%)	0	0	1	1 (<0.1%)
Tinea infection	2	1 (<0.1%)	0	0	2	1 (<0.1%)
Tinea pedis	2	2 (0.1%)	0	0	2	2 (<0.1%)
Tonsillitis	4	4 (0.3%)	2	2 (0.1%)	6	6 (0.2%)
Tooth abscess	1	1 (<0.1%)	0	0	1	1 (<0.1%)
Tooth infection	1	1 (<0.1%)	2	2 (0.1%)	3	3 (0.1%)
Upper respiratory tract infection	8	7 (0.5%)	10	10 (0.7%)	18	17 (0.6%)
Urinary tract infection	13	13 (0.9%)	16	15 (1.0%)	29	28 (1.0%)
Vaginal infection	3	3 (0.2%)	6	6 (0.4%)	9	9 (0.3%)
Vaginitis bacterial	27	27 (1.9%)	20	20 (1.4%)	47	47 (1.6%)
Vaginitis chlamydial	2	2 (0.1%)	1	1 (<0.1%)	3	3 (0.1%)
Viral upper respiratory tract infection	1	1 (<0.1%)	3	3 (0.2%)	4	4 (0.1%)
Vulvitis	2	2 (0.1%)	0	0	2	2 (<0.1%)
Vulvovaginal candidiasis	9	9 (0.6%)	9	9 (0.6%)	18	18 (0.6%)
Vulvovaginal mycotic infection	6	5 (0.3%)	5	5 (0.3%)	11	10 (0.3%)
Vulvovaginitis streptococcal	0	0	1	1 (<0.1%)	1	1 (<0.1%)
Vulvovaginitis trichomonal	1	1 (<0.1%)	0	0	1	1 (<0.1%)
Injury, poisoning and procedural complications	13	11 (0.8%)	18	15 (1.0%)	31	26 (0.9%)
Ankle fracture	0	0	2	2 (0.1%)	2	2 (<0.1%)
Cartilage injury	0	0	1	1 (<0.1%)	1	1 (<0.1%)
Concussion	3	2 (0.1%)	0	0	3	2 (<0.1%)
Epicondylitis	0	0	1	1 (<0.1%)	1	1 (<0.1%)
Head injury	1	1 (<0.1%)	0	0	1	1 (<0.1%)
Incision site pain	0	0	1	1 (<0.1%)	1	1 (<0.1%)
Joint injury	2	2 (0.1%)	0	0	2	2 (<0.1%)
Ligament rupture	0	0	1	1 (<0.1%)	1	1 (<0.1%)
Limb injury	1	1 (<0.1%)	0	0	1	1 (<0.1%)
Muscle strain	0	0	2	2 (0.1%)	2	2 (<0.1%)
Neck injury	0	0	1	1 (<0.1%)	1	1 (<0.1%)
Post-traumatic pain	0	0	2	2 (0.1%)	2	2 (<0.1%)
Procedural pain	3	3 (0.2%)	1	1 (<0.1%)	4	4 (0.1%)
Procedural site reaction	0	0	1	1 (<0.1%)	1	1 (<0.1%)
Spinal column injury	0	0	3	1 (<0.1%)	3	1 (<0.1%)
Tooth fracture	1	1 (<0.1%)	0	0	1	1 (<0.1%)
Upper limb fracture	0	0	1	1 (<0.1%)	1	1 (<0.1%)
Whiplash injury	0	0	1	1 (<0.1%)	1	1 (<0.1%)
Wound	1	1 (<0.1%)	0	0	1	1 (<0.1%)
Wrist fracture	1	1 (<0.1%)	0	0	1	1 (<0.1%)

Table 14.3.1 / 2: Number of subjects with baseline findings and number of baseline findings by MedDRA SOC/PT (FAS)

Primary system organ class<br>Preferred term<br>MedDRA version 14.0	LCS12<br>Events	 N=1432 (100%)	LCS16<br>Events	 N=1452 (100%)	Total<br>Events	 N=2884 (100%)
Investigations	108	89 (6.2%)	125	98 (6.7%)	233	187 (6.5%)
Alanine aminotransferase increased	10	10 (0.7%)	8	8 (0.6%)	18	18 (0.6%)
Aspartate aminotransferase increased	2	2 (0.1%)	2	2 (0.1%)	4	4 (0.1%)
Biopsy	1	1 (<0.1%)	0	0	1	1 (<0.1%)
Blood albumin decreased	1	1 (<0.1%)	0	0	1	1 (<0.1%)
Blood albumin increased	1	1 (<0.1%)	0	0	1	1 (<0.1%)
Blood alkaline phosphatase increased	1	1 (<0.1%)	0	0	1	1 (<0.1%)
Blood cholesterol decreased	1	1 (<0.1%)	0	0	1	1 (<0.1%)
Blood cholesterol increased	5	5 (0.3%)	4	4 (0.3%)	9	9 (0.3%)
Blood creatinine decreased	2	2 (0.1%)	6	6 (0.4%)	8	8 (0.3%)
Blood iron decreased	0	0	1	1 (<0.1%)	1	1 (<0.1%)
Blood potassium decreased	1	1 (<0.1%)	0	0	1	1 (<0.1%)
Blood potassium increased	5	5 (0.3%)	5	5 (0.3%)	10	10 (0.3%)
Blood pressure increased	1	1 (<0.1%)	0	0	1	1 (<0.1%)
Blood pressure systolic increased	0	0	1	1 (<0.1%)	1	1 (<0.1%)
Blood sodium decreased	0	0	1	1 (<0.1%)	1	1 (<0.1%)
Blood sodium increased	4	4 (0.3%)	2	2 (0.1%)	6	6 (0.2%)
Blood triglycerides increased	6	6 (0.4%)	10	10 (0.7%)	16	16 (0.6%)
Blood urine present	1	1 (<0.1%)	2	2 (0.1%)	3	3 (0.1%)
Cardiac murmur	1	1 (<0.1%)	0	0	1	1 (<0.1%)
Chlamydia test	1	1 (<0.1%)	0	0	1	1 (<0.1%)
Chlamydia test positive	5	5 (0.3%)	12	12 (0.8%)	17	17 (0.6%)
Colposcopy	1	1 (<0.1%)	0	0	1	1 (<0.1%)
Fungal test positive	0	0	1	1 (<0.1%)	1	1 (<0.1%)
Gamma-glutamyltransferase increased	5	5 (0.3%)	7	6 (0.4%)	12	11 (0.4%)
Glycosylated haemoglobin increased	0	0	2	2 (0.1%)	2	2 (<0.1%)
Haematocrit decreased	2	2 (0.1%)	4	4 (0.3%)	6	6 (0.2%)
Haematocrit increased	1	1 (<0.1%)	0	0	1	1 (<0.1%)
Haematology test abnormal	0	0	1	1 (<0.1%)	1	1 (<0.1%)
Haemoglobin decreased	5	5 (0.3%)	8	8 (0.6%)	13	13 (0.5%)
Haemoglobin increased	0	0	1	1 (<0.1%)	1	1 (<0.1%)
Haemoglobin urine present	0	0	2	2 (0.1%)	2	2 (<0.1%)
Heart sounds abnormal	1	1 (<0.1%)	0	0	1	1 (<0.1%)
Hepatic enzyme increased	2	2 (0.1%)	1	1 (<0.1%)	3	3 (0.1%)
High density lipoprotein abnormal	2	2 (0.1%)	0	0	2	2 (<0.1%)
High density lipoprotein decreased	3	3 (0.2%)	3	3 (0.2%)	6	6 (0.2%)
Human papilloma virus test positive	0	0	2	2 (0.1%)	2	2 (<0.1%)
Lipids increased	1	1 (<0.1%)	0	0	1	1 (<0.1%)
Liver function test abnormal	1	1 (<0.1%)	0	0	1	1 (<0.1%)
Low density lipoprotein decreased	0	0	1	1 (<0.1%)	1	1 (<0.1%)
Low density lipoprotein increased	7	7 (0.5%)	5	5 (0.3%)	12	12 (0.4%)
Platelet count decreased	0	0	2	2 (0.1%)	2	2 (<0.1%)
Platelet count increased	0	0	1	1 (<0.1%)	1	1 (<0.1%)

Table 14.3.1 / 2: Number of subjects with baseline findings and number of baseline findings by MedDRA SOC/PT (FAS)

Primary system organ class<br>Preferred term<br>MedDRA version 14.0	LCS12<br>Events	 N=1432 (100%)	LCS16<br>Events	 N=1452 (100%)	Total<br>Events	 N=2884 (100%)
Pregnancy test positive	1	1 (<0.1%)	0	0	1	1 (<0.1%)
Protein total decreased	2	2 (0.1%)	1	1 (<0.1%)	3	3 (0.1%)
Protein total increased	1	1 (<0.1%)	2	2 (0.1%)	3	3 (0.1%)
Red blood cell count decreased	0	0	1	1 (<0.1%)	1	1 (<0.1%)
Red blood cell count increased	1	1 (<0.1%)	1	1 (<0.1%)	2	2 (<0.1%)
Rhesus antibodies negative	0	0	1	1 (<0.1%)	1	1 (<0.1%)
Smear cervix abnormal	10	10 (0.7%)	5	5 (0.3%)	15	15 (0.5%)
Ultrasound uterus abnormal	1	1 (<0.1%)	0	0	1	1 (<0.1%)
Urine leukocyte esterase positive	2	2 (0.1%)	1	1 (<0.1%)	3	3 (0.1%)
Weight decreased	1	1 (<0.1%)	0	0	1	1 (<0.1%)
Weight increased	0	0	2	2 (0.1%)	2	2 (<0.1%)
White blood cell count decreased	3	3 (0.2%)	7	7 (0.5%)	10	10 (0.3%)
White blood cell count increased	6	6 (0.4%)	9	9 (0.6%)	15	15 (0.5%)
Metabolism and nutrition disorders	26	25 (1.7%)	35	33 (2.3%)	61	58 (2.0%)
Diabetes mellitus	0	0	1	1 (<0.1%)	1	1 (<0.1%)
Fluid retention	0	0	2	2 (0.1%)	2	2 (<0.1%)
Hypercholesterolaemia	3	3 (0.2%)	4	4 (0.3%)	7	7 (0.2%)
Hyperlipidaemia	2	2 (0.1%)	2	2 (0.1%)	4	4 (0.1%)
Hypertriglyceridaemia	1	1 (<0.1%)	0	0	1	1 (<0.1%)
Hypocholesterolaemia	1	1 (<0.1%)	0	0	1	1 (<0.1%)
Hypoglycaemia	1	1 (<0.1%)	2	2 (0.1%)	3	3 (0.1%)
Hypokalaemia	0	0	1	1 (<0.1%)	1	1 (<0.1%)
Insulin resistance	4	4 (0.3%)	6	6 (0.4%)	10	10 (0.3%)
Iron deficiency	0	0	2	2 (0.1%)	2	2 (<0.1%)
Lactose intolerance	5	5 (0.3%)	7	7 (0.5%)	12	12 (0.4%)
Obesity	5	5 (0.3%)	7	7 (0.5%)	12	12 (0.4%)
Overweight	1	1 (<0.1%)	0	0	1	1 (<0.1%)
Type 1 diabetes mellitus	3	3 (0.2%)	1	1 (<0.1%)	4	4 (0.1%)

Table 14.3.1 / 2: Number of subjects with baseline findings and number of baseline findings by MedDRA SOC/PT (FAS)

Primary system organ class<br>Preferred term<br>MedDRA version 14.0	LCS12 Events	LCS12 N=1432 (100%)	LCS16 Events	LCS16 N=1452 (100%)	Total Events	Total N=2884 (100%)
Musculoskeletal and connective tissue disorders	67	63 (4.4%)	79	64 (4.4%)	146	127 (4.4%)
Ankylosing spondylitis	1	1 (<0.1%)	0	0	1	1 (<0.1%)
Arthralgia	6	6 (0.4%)	4	4 (0.3%)	10	10 (0.3%)
Arthritis	1	1 (<0.1%)	7	4 (0.3%)	8	5 (0.2%)
Back pain	21	21 (1.5%)	20	20 (1.4%)	41	41 (1.4%)
Bone pain	0	0	1	1 (<0.1%)	1	1 (<0.1%)
Fibromyalgia	1	1 (<0.1%)	2	2 (0.1%)	3	3 (0.1%)
Foot deformity	0	0	3	2 (0.1%)	3	2 (<0.1%)
Intervertebral disc degeneration	1	1 (<0.1%)	0	0	1	1 (<0.1%)
Intervertebral disc protrusion	4	4 (0.3%)	1	1 (<0.1%)	5	5 (0.2%)
Juvenile arthritis	1	1 (<0.1%)	0	0	1	1 (<0.1%)
Medial tibial stress syndrome	1	1 (<0.1%)	0	0	1	1 (<0.1%)
Muscle contracture	1	1 (<0.1%)	0	0	1	1 (<0.1%)
Muscle spasms	2	2 (0.1%)	0	0	2	2 (<0.1%)
Muscle tightness	4	4 (0.3%)	2	2 (0.1%)	6	6 (0.2%)
Muscular weakness	0	0	1	1 (<0.1%)	1	1 (<0.1%)
Musculoskeletal chest pain	1	1 (<0.1%)	0	0	1	1 (<0.1%)
Musculoskeletal pain	4	4 (0.3%)	6	6 (0.4%)	10	10 (0.3%)
Myalgia	3	2 (0.1%)	4	4 (0.3%)	7	6 (0.2%)
Myokymia	0	0	2	2 (0.1%)	2	2 (<0.1%)
Neck pain	1	1 (<0.1%)	7	7 (0.5%)	8	8 (0.3%)
Osteitis	0	0	1	1 (<0.1%)	1	1 (<0.1%)
Osteoarthritis	2	1 (<0.1%)	0	0	2	1 (<0.1%)
Osteopenia	1	1 (<0.1%)	1	1 (<0.1%)	2	2 (<0.1%)
Pain in extremity	3	3 (0.2%)	4	4 (0.3%)	7	7 (0.2%)
Patellofemoral pain syndrome	0	0	3	3 (0.2%)	3	3 (0.1%)
Rheumatoid arthritis	3	3 (0.2%)	1	1 (<0.1%)	4	4 (0.1%)
Scoliosis	5	5 (0.3%)	3	3 (0.2%)	8	8 (0.3%)
Seronegative arthritis	0	0	1	1 (<0.1%)	1	1 (<0.1%)
Tendonitis	0	0	5	5 (0.3%)	5	5 (0.2%)

Table 14.3.1 / 2: Number of subjects with baseline findings and number of baseline findings by MedDRA SOC/PT (FAS)

Primary system organ class<br>Preferred term<br>MedDRA version 14.0	LCS12<br>Events	 N=1432 (100%)	LCS16<br>Events	 N=1452 (100%)	Total<br>Events	 N=2884 (100%)
Neoplasms benign, malignant and unspecified (incl cysts and polyps)	19	19 (1.3%)	14	14 (1.0%)	33	33 (1.1%)
Acrochordon	0	0	1	1 (<0.1%)	1	1 (<0.1%)
Benign breast neoplasm	0	0	1	1 (<0.1%)	1	1 (<0.1%)
Breast fibroma	1	1 (<0.1%)	0	0	1	1 (<0.1%)
Cervicitis human papilloma virus	2	2 (0.1%)	0	0	2	2 (<0.1%)
Fibroadenoma of breast	2	2 (0.1%)	1	1 (<0.1%)	3	3 (0.1%)
Fibroma	0	0	1	1 (<0.1%)	1	1 (<0.1%)
Glomus tumour	1	1 (<0.1%)	0	0	1	1 (<0.1%)
Lipoma	2	2 (0.1%)	0	0	2	2 (<0.1%)
Melanocytic naevus	3	3 (0.2%)	2	2 (0.1%)	5	5 (0.2%)
Prolactinoma	1	1 (<0.1%)	0	0	1	1 (<0.1%)
Skin papilloma	1	1 (<0.1%)	0	0	1	1 (<0.1%)
Uterine leiomyoma	6	6 (0.4%)	7	7 (0.5%)	13	13 (0.5%)
Vulvovaginal human papilloma virus infection	0	0	1	1 (<0.1%)	1	1 (<0.1%)
Nervous system disorders	152	142 (9.9%)	164	155 (10.7%)	316	297 (10.3%)
Ageusia	0	0	1	1 (<0.1%)	1	1 (<0.1%)
Carpal tunnel syndrome	1	1 (<0.1%)	0	0	1	1 (<0.1%)
Cervicobrachial syndrome	0	0	1	1 (<0.1%)	1	1 (<0.1%)
Convulsion	0	0	1	1 (<0.1%)	1	1 (<0.1%)
Disturbance in attention	0	0	1	1 (<0.1%)	1	1 (<0.1%)
Dizziness	1	1 (<0.1%)	1	1 (<0.1%)	2	2 (<0.1%)
Epilepsy	5	5 (0.3%)	3	3 (0.2%)	8	8 (0.3%)
Essential tremor	0	0	1	1 (<0.1%)	1	1 (<0.1%)
Headache	79	77 (5.4%)	77	77 (5.3%)	156	154 (5.3%)
Hypersomnia	0	0	1	1 (<0.1%)	1	1 (<0.1%)
Hypertonia	0	0	1	1 (<0.1%)	1	1 (<0.1%)
Migraine	42	42 (2.9%)	52	52 (3.6%)	94	94 (3.3%)
Migraine with aura	0	0	1	1 (<0.1%)	1	1 (<0.1%)
Migraine without aura	0	0	1	1 (<0.1%)	1	1 (<0.1%)
Myasthenia gravis	0	0	1	1 (<0.1%)	1	1 (<0.1%)
Nerve compression	0	0	1	1 (<0.1%)	1	1 (<0.1%)
Nystagmus	1	1 (<0.1%)	0	0	1	1 (<0.1%)
Restless legs syndrome	1	1 (<0.1%)	0	0	1	1 (<0.1%)
Sciatica	3	3 (0.2%)	0	0	3	3 (0.1%)
Sinus headache	1	1 (<0.1%)	2	2 (0.1%)	3	3 (0.1%)
Tension headache	17	17 (1.2%)	18	18 (1.2%)	35	35 (1.2%)
Tremor	1	1 (<0.1%)	0	0	1	1 (<0.1%)

Table 14.3.1 / 2: Number of subjects with baseline findings and number of baseline findings by MedDRA SOC/PT (FAS)

Primary system organ class<br>Preferred term<br>MedDRA version 14.0	LCS12<br>Events	 N=1432 (100%)	LCS16<br>Events	 N=1452 (100%)	Total<br>Events	 N=2884 (100%)
Pregnancy, puerperium and perinatal conditions	2	1 (<0.1%)	2	2 (0.1%)	4	3 (0.1%)
Abortion	1	1 (<0.1%)	0	0	1	1 (<0.1%)
Pregnancy	1	1 (<0.1%)	1	1 (<0.1%)	2	2 (<0.1%)
Traumatic delivery	0	0	1	1 (<0.1%)	1	1 (<0.1%)
Psychiatric disorders	135	113 (7.9%)	137	114 (7.9%)	272	227 (7.9%)
Alcohol abuse	0	0	1	1 (<0.1%)	1	1 (<0.1%)
Anorgasmia	0	0	1	1 (<0.1%)	1	1 (<0.1%)
Anxiety	33	33 (2.3%)	33	33 (2.3%)	66	66 (2.3%)
Attention deficit/hyperactivity disorder	6	6 (0.4%)	7	7 (0.5%)	13	13 (0.5%)
Bipolar I disorder	1	1 (<0.1%)	0	0	1	1 (<0.1%)
Bipolar disorder	1	1 (<0.1%)	4	4 (0.3%)	5	5 (0.2%)
Borderline personality disorder	0	0	1	1 (<0.1%)	1	1 (<0.1%)
Bruxism	1	1 (<0.1%)	0	0	1	1 (<0.1%)
Burnout syndrome	0	0	2	2 (0.1%)	2	2 (<0.1%)
Depressed mood	1	1 (<0.1%)	1	1 (<0.1%)	2	2 (<0.1%)
Depression	62	62 (4.3%)	51	51 (3.5%)	113	113 (3.9%)
Encopresis	0	0	1	1 (<0.1%)	1	1 (<0.1%)
Generalised anxiety disorder	2	2 (0.1%)	0	0	2	2 (<0.1%)
Insomnia	16	16 (1.1%)	16	16 (1.1%)	32	32 (1.1%)
Libido decreased	2	2 (0.1%)	3	3 (0.2%)	5	5 (0.2%)
Mood swings	0	0	1	1 (<0.1%)	1	1 (<0.1%)
Obsessive-compulsive disorder	2	2 (0.1%)	1	1 (<0.1%)	3	3 (0.1%)
Panic disorder	1	1 (<0.1%)	4	4 (0.3%)	5	5 (0.2%)
Panic reaction	0	0	1	1 (<0.1%)	1	1 (<0.1%)
Phobia	1	1 (<0.1%)	0	0	1	1 (<0.1%)
Post-traumatic stress disorder	0	0	1	1 (<0.1%)	1	1 (<0.1%)
Postpartum depression	5	5 (0.3%)	5	5 (0.3%)	10	10 (0.3%)
Psychotic disorder	0	0	1	1 (<0.1%)	1	1 (<0.1%)
Seasonal affective disorder	0	0	1	1 (<0.1%)	1	1 (<0.1%)
Sleep disorder	1	1 (<0.1%)	0	0	1	1 (<0.1%)
Social phobia	0	0	1	1 (<0.1%)	1	1 (<0.1%)

Table 14.3.1 / 2: Number of subjects with baseline findings and number of baseline findings by MedDRA SOC/PT (FAS)

Primary system organ class<br>Preferred term<br>MedDRA version 14.0	LCS12<br>Events	LCS12<br>N=1432 (100%)	LCS16<br>Events	LCS16<br>N=1452 (100%)	Total<br>Events	Total<br>N=2884 (100%)
Renal and urinary disorders	12	10 (0.7%)	3	3 (0.2%)	15	13 (0.5%)
Calculus urinary	1	1 (<0.1%)	0	0	1	1 (<0.1%)
Cystitis interstitial	2	2 (0.1%)	2	2 (0.1%)	4	4 (0.1%)
Dysuria	1	1 (<0.1%)	0	0	1	1 (<0.1%)
Haematuria	1	1 (<0.1%)	0	0	1	1 (<0.1%)
Micturition urgency	1	1 (<0.1%)	0	0	1	1 (<0.1%)
Pollakiuria	1	1 (<0.1%)	0	0	1	1 (<0.1%)
Renal failure chronic	1	1 (<0.1%)	0	0	1	1 (<0.1%)
Stress urinary incontinence	3	3 (0.2%)	1	1 (<0.1%)	4	4 (0.1%)
Urinary incontinence	1	1 (<0.1%)	0	0	1	1 (<0.1%)

Table 14.3.1 / 2: Number of subjects with baseline findings and number of baseline findings by MedDRA SOC/PT (FAS)

Primary system organ class<br>Preferred term<br>MedDRA version 14.0	LCS12<br>Events	 N=1432 (100%)	LCS16<br>Events	 N=1452 (100%)	Total<br>Events	 N=2884 (100%)
Reproductive system and breast disorders	216	186 (13.0%)	261	215 (14.8%)	477	401 (13.9%)
Adnexa uteri mass	1	1 (<0.1%)	0	0	1	1 (<0.1%)
Adnexa uteri pain	1	1 (<0.1%)	0	0	1	1 (<0.1%)
Amenorrhoea	0	0	3	3 (0.2%)	3	3 (0.1%)
Bartholin's cyst	2	2 (0.1%)	0	0	2	2 (<0.1%)
Breast atrophy	1	1 (<0.1%)	0	0	1	1 (<0.1%)
Breast cyst	1	1 (<0.1%)	1	1 (<0.1%)	2	2 (<0.1%)
Breast discomfort	2	2 (0.1%)	5	5 (0.3%)	7	7 (0.2%)
Breast enlargement	0	0	1	1 (<0.1%)	1	1 (<0.1%)
Breast mass	3	3 (0.2%)	1	1 (<0.1%)	4	4 (0.1%)
Breast pain	1	1 (<0.1%)	1	1 (<0.1%)	2	2 (<0.1%)
Breast tenderness	0	0	1	1 (<0.1%)	1	1 (<0.1%)
Cervical cyst	0	0	1	1 (<0.1%)	1	1 (<0.1%)
Cervical dysplasia	20	20 (1.4%)	24	24 (1.7%)	44	44 (1.5%)
Cervical friability	0	0	1	1 (<0.1%)	1	1 (<0.1%)
Cervical polyp	1	1 (<0.1%)	2	2 (0.1%)	3	3 (0.1%)
Cervix haemorrhage uterine	0	0	1	1 (<0.1%)	1	1 (<0.1%)
Cervix inflammation	2	2 (0.1%)	1	1 (<0.1%)	3	3 (0.1%)
Dysmenorrhoea	95	95 (6.6%)	120	119 (8.2%)	215	214 (7.4%)
Dyspareunia	0	0	2	2 (0.1%)	2	2 (<0.1%)
Ectropion of cervix	2	2 (0.1%)	2	2 (0.1%)	4	4 (0.1%)
Endometriosis	4	4 (0.3%)	4	4 (0.3%)	8	8 (0.3%)
Fibrocystic breast disease	5	5 (0.3%)	3	3 (0.2%)	8	8 (0.3%)
Galactorrhoea	2	2 (0.1%)	2	2 (0.1%)	4	4 (0.1%)
Genital discharge	1	1 (<0.1%)	4	4 (0.3%)	5	5 (0.2%)
Haemorrhagic ovarian cyst	1	1 (<0.1%)	0	0	1	1 (<0.1%)
Hydrometra	1	1 (<0.1%)	0	0	1	1 (<0.1%)
Menorrhagia	4	4 (0.3%)	4	4 (0.3%)	8	8 (0.3%)
Menstruation delayed	1	1 (<0.1%)	0	0	1	1 (<0.1%)
Menstruation irregular	1	1 (<0.1%)	0	0	1	1 (<0.1%)
Metrorrhagia	1	1 (<0.1%)	2	2 (0.1%)	3	3 (0.1%)
Nipple disorder	0	0	1	1 (<0.1%)	1	1 (<0.1%)
Ovarian atrophy	1	1 (<0.1%)	0	0	1	1 (<0.1%)
Ovarian cyst	22	21 (1.5%)	20	20 (1.4%)	42	41 (1.4%)
Ovulation pain	1	1 (<0.1%)	0	0	1	1 (<0.1%)
Parovarian cyst	2	2 (0.1%)	0	0	2	2 (<0.1%)
Pelvic pain	0	0	1	1 (<0.1%)	1	1 (<0.1%)
Polycystic ovaries	1	1 (<0.1%)	2	2 (0.1%)	3	3 (0.1%)
Premenstrual syndrome	26	26 (1.8%)	35	35 (2.4%)	61	61 (2.1%)
Rectocele	1	1 (<0.1%)	0	0	1	1 (<0.1%)
Uterine cervical erosion	1	1 (<0.1%)	0	0	1	1 (<0.1%)
Uterine cervical pain	1	1 (<0.1%)	0	0	1	1 (<0.1%)
Uterine cervix stenosis	0	0	1	1 (<0.1%)	1	1 (<0.1%)

Table 14.3.1 / 2: Number of subjects with baseline findings and number of baseline findings by MedDRA SOC/PT (FAS)

Primary system organ class<br>Preferred term<br>MedDRA version 14.0	LCS12<br>Events	LCS12<br>N=1432 (100%)	LCS16<br>Events	LCS16<br>N=1452 (100%)	Total<br>Events	Total<br>N=2884 (100%)
Uterine spasm	1	1 (<0.1%)	0	0	1	1 (<0.1%)
Vaginal cyst	0	0	1	1 (<0.1%)	1	1 (<0.1%)
Vaginal discharge	0	0	6	6 (0.4%)	6	6 (0.2%)
Vaginal disorder	1	1 (<0.1%)	1	1 (<0.1%)	2	2 (<0.1%)
Vaginal haemorrhage	1	1 (<0.1%)	0	0	1	1 (<0.1%)
Varicose veins vaginal	1	1 (<0.1%)	0	0	1	1 (<0.1%)
Vulvovaginal burning sensation	1	1 (<0.1%)	1	1 (<0.1%)	2	2 (<0.1%)
Vulvovaginal discomfort	0	0	1	1 (<0.1%)	1	1 (<0.1%)
Vulvovaginal dryness	1	1 (<0.1%)	2	2 (0.1%)	3	3 (0.1%)
Vulvovaginal erythema	1	1 (<0.1%)	0	0	1	1 (<0.1%)
Vulvovaginal pain	0	0	1	1 (<0.1%)	1	1 (<0.1%)
Vulvovaginal pruritus	0	0	2	2 (0.1%)	2	2 (<0.1%)
Respiratory, thoracic and mediastinal disorders	44	44 (3.1%)	59	58 (4.0%)	103	102 (3.5%)
Allergic bronchitis	1	1 (<0.1%)	0	0	1	1 (<0.1%)
Allergic respiratory disease	0	0	1	1 (<0.1%)	1	1 (<0.1%)
Allergic sinusitis	1	1 (<0.1%)	0	0	1	1 (<0.1%)
Asthma	30	30 (2.1%)	42	42 (2.9%)	72	72 (2.5%)
Asthma exercise induced	3	3 (0.2%)	1	1 (<0.1%)	4	4 (0.1%)
Bronchospasm	1	1 (<0.1%)	0	0	1	1 (<0.1%)
Cough	0	0	4	4 (0.3%)	4	4 (0.1%)
Dysphonia	0	0	1	1 (<0.1%)	1	1 (<0.1%)
Nasal disorder	1	1 (<0.1%)	0	0	1	1 (<0.1%)
Nasal obstruction	0	0	1	1 (<0.1%)	1	1 (<0.1%)
Nasal septum deviation	0	0	1	1 (<0.1%)	1	1 (<0.1%)
Oropharyngeal pain	1	1 (<0.1%)	0	0	1	1 (<0.1%)
Rhinitis allergic	3	3 (0.2%)	5	5 (0.3%)	8	8 (0.3%)
Sinus congestion	2	2 (0.1%)	3	3 (0.2%)	5	5 (0.2%)
Vasomotor rhinitis	1	1 (<0.1%)	0	0	1	1 (<0.1%)

Table 14.3.1 / 2: Number of subjects with baseline findings and number of baseline findings by MedDRA SOC/PT (FAS)

Primary system organ class<br>Preferred term<br>MedDRA version 14.0	LCS12<br>Events	 N=1432 (100%)	LCS16<br>Events	 N=1452 (100%)	Total<br>Events	 N=2884 (100%)
Skin and subcutaneous tissue disorders	71	64 (4.5%)	83	74 (5.1%)	154	138 (4.8%)
Acanthosis nigricans	0	0	1	1 (<0.1%)	1	1 (<0.1%)
Acne	32	32 (2.2%)	31	31 (2.1%)	63	63 (2.2%)
Alopecia	0	0	1	1 (<0.1%)	1	1 (<0.1%)
Chloasma	0	0	1	1 (<0.1%)	1	1 (<0.1%)
Cutaneous lupus erythematosus	0	0	2	2 (0.1%)	2	2 (<0.1%)
Dermal cyst	0	0	1	1 (<0.1%)	1	1 (<0.1%)
Dermatitis	1	1 (<0.1%)	2	2 (0.1%)	3	3 (0.1%)
Dermatitis allergic	1	1 (<0.1%)	1	1 (<0.1%)	2	2 (<0.1%)
Dermatitis atopic	7	7 (0.5%)	6	6 (0.4%)	13	13 (0.5%)
Dermatitis contact	0	0	1	1 (<0.1%)	1	1 (<0.1%)
Dry skin	1	1 (<0.1%)	3	3 (0.2%)	4	4 (0.1%)
Ecchymosis	0	0	1	1 (<0.1%)	1	1 (<0.1%)
Eczema	10	10 (0.7%)	9	9 (0.6%)	19	19 (0.7%)
Hirsutism	4	4 (0.3%)	1	1 (<0.1%)	5	5 (0.2%)
Hyperhidrosis	1	1 (<0.1%)	0	0	1	1 (<0.1%)
Hypertrichosis	0	0	1	1 (<0.1%)	1	1 (<0.1%)
Increased tendency to bruise	0	0	1	1 (<0.1%)	1	1 (<0.1%)
Keloid scar	0	0	1	1 (<0.1%)	1	1 (<0.1%)
Lichen sclerosus	0	0	1	1 (<0.1%)	1	1 (<0.1%)
Psoriasis	4	4 (0.3%)	7	7 (0.5%)	11	11 (0.4%)
Purpura	1	1 (<0.1%)	0	0	1	1 (<0.1%)
Rash	2	1 (<0.1%)	4	4 (0.3%)	6	5 (0.2%)
Rash generalised	1	1 (<0.1%)	0	0	1	1 (<0.1%)
Rosacea	1	1 (<0.1%)	3	3 (0.2%)	4	4 (0.1%)
Scar	2	2 (0.1%)	2	2 (0.1%)	4	4 (0.1%)
Skin hypertrophy	0	0	1	1 (<0.1%)	1	1 (<0.1%)
Skin irritation	1	1 (<0.1%)	0	0	1	1 (<0.1%)
Urticaria	2	2 (0.1%)	1	1 (<0.1%)	3	3 (0.1%)
Social circumstances	1	1 (<0.1%)	4	4 (0.3%)	5	5 (0.2%)
Corrective lens user	1	1 (<0.1%)	4	4 (0.3%)	5	5 (0.2%)

Table 14.3.1 / 2: Number of subjects with baseline findings and number of baseline findings by MedDRA SOC/PT (FAS)

Primary system organ class<br>Preferred term<br>MedDRA version 14.0	LCS12<br>Events	LCS12<br>N=1432 (100%)	LCS16<br>Events	LCS16<br>N=1452 (100%)	Total<br>Events	Total<br>N=2884 (100%)
Surgical and medical procedures	8	8 (0.6%)	5	5 (0.3%)	13	13 (0.5%)
Abortion induced	0	0	1	1 (<0.1%)	1	1 (<0.1%)
Breast prosthesis implantation	1	1 (<0.1%)	0	0	1	1 (<0.1%)
Contraception	1	1 (<0.1%)	0	0	1	1 (<0.1%)
Ear tube insertion	1	1 (<0.1%)	0	0	1	1 (<0.1%)
Endodontic procedure	1	1 (<0.1%)	0	0	1	1 (<0.1%)
Mammoplasty	1	1 (<0.1%)	1	1 (<0.1%)	2	2 (<0.1%)
Medical device removal	0	0	1	1 (<0.1%)	1	1 (<0.1%)
Mole excision	0	0	1	1 (<0.1%)	1	1 (<0.1%)
Obesity surgery	1	1 (<0.1%)	0	0	1	1 (<0.1%)
Oral contraception	0	0	1	1 (<0.1%)	1	1 (<0.1%)
Pterygium operation	1	1 (<0.1%)	0	0	1	1 (<0.1%)
Tonsillectomy	1	1 (<0.1%)	0	0	1	1 (<0.1%)
Vascular disorders	21	21 (1.5%)	21	20 (1.4%)	42	41 (1.4%)
Hot flush	0	0	1	1 (<0.1%)	1	1 (<0.1%)
Hypertension	16	16 (1.1%)	12	12 (0.8%)	28	28 (1.0%)
Hypotension	2	2 (0.1%)	4	4 (0.3%)	6	6 (0.2%)
Orthostatic hypotension	0	0	1	1 (<0.1%)	1	1 (<0.1%)
Raynaud's phenomenon	0	0	1	1 (<0.1%)	1	1 (<0.1%)
Varicose vein	3	3 (0.2%)	2	2 (0.1%)	5	5 (0.2%)

Note: Adverse events are sorted in alphabetical order by primary SOC and preferred term.
Global Biostatistics: /by-sasp/patdb/projects/de04209/310442/stat/prod_interim03/pgms/t-basfin.sas epkll 12OCT2011 11:19
End of table

Table 14.3.1 / 3: Listing of serious baseline findings by subject (FAS)

TREATMENT	Subject number	BF specification	BF onset date	BF stop date	BF still continuing	BF intensity
LCS12	160637	UNSPECIFIC PAIN	.	.	yes	moderate

Global Biostatistics: /by-sasp/patdb/projects/de04209/310442/stat/prod_interim03/pgms/t-basfin.sas epkll 12OCT2011 11:19
End of table

Table 14.3.1 / 4: Number of subjects with baseline findings or medical history by MedDRA SOC/PT (FAS)

Primary system organ class<br>Preferred term<br>MedDRA version 14.0	LCS12<br>N=1432 (100%)	LCS16<br>N=1452 (100%)	Total<br>N=2884 (100%)
Number of subjects (%) with at least one such event	1236 (86.3%)	1258 (86.6%)	2494 (86.5%)
Blood and lymphatic system disorders	42 (2.9%)	56 (3.9%)	98 (3.4%)
Activated protein C resistance	1 (<0.1%)	0	1 (<0.1%)
Anaemia	32 (2.2%)	47 (3.2%)	79 (2.7%)
Anaemia of pregnancy	3 (0.2%)	0	3 (0.1%)
Coagulopathy	1 (<0.1%)	0	1 (<0.1%)
Haemoconcentration	0	1 (<0.1%)	1 (<0.1%)
Idiopathic thrombocytopenic purpura	0	1 (<0.1%)	1 (<0.1%)
Iron deficiency anaemia	2 (0.1%)	3 (0.2%)	5 (0.2%)
Lymphadenitis	0	2 (0.1%)	2 (<0.1%)
Lymphadenopathy	0	4 (0.3%)	4 (0.1%)
Lymphoid tissue hyperplasia	1 (<0.1%)	0	1 (<0.1%)
Pernicious anaemia	1 (<0.1%)	0	1 (<0.1%)
Splenomegaly	1 (<0.1%)	0	1 (<0.1%)
Thrombocytopenia	1 (<0.1%)	0	1 (<0.1%)
Thrombocytosis	0	1 (<0.1%)	1 (<0.1%)
Cardiac disorders	10 (0.7%)	28 (1.9%)	38 (1.3%)
Angina pectoris	0	1 (<0.1%)	1 (<0.1%)
Aortic valve disease	0	1 (<0.1%)	1 (<0.1%)
Arrhythmia	2 (0.1%)	4 (0.3%)	6 (0.2%)
Extrasystoles	1 (<0.1%)	1 (<0.1%)	2 (<0.1%)
Long QT syndrome	0	1 (<0.1%)	1 (<0.1%)
Mitral valve disease	0	1 (<0.1%)	1 (<0.1%)
Mitral valve incompetence	0	1 (<0.1%)	1 (<0.1%)
Mitral valve prolapse	5 (0.3%)	7 (0.5%)	12 (0.4%)
Myocarditis	1 (<0.1%)	1 (<0.1%)	2 (<0.1%)
Palpitations	1 (<0.1%)	4 (0.3%)	5 (0.2%)
Pulmonary valve stenosis	0	1 (<0.1%)	1 (<0.1%)
Supraventricular tachycardia	0	3 (0.2%)	3 (0.1%)
Tachycardia	1 (<0.1%)	5 (0.3%)	6 (0.2%)
Wolff-Parkinson-White syndrome	0	1 (<0.1%)	1 (<0.1%)

Table 14.3.1 / 4: Number of subjects with baseline findings or medical history by MedDRA SOC/PT (FAS)

Primary system organ class<br>Preferred term<br>MedDRA version 14.0	LCS12<br>N=1432 (100%)	LCS16<br>N=1452 (100%)	Total<br>N=2884 (100%)
Congenital, familial and genetic disorders	25 (1.7%)	25 (1.7%)	50 (1.7%)
Accessory breast	0	1 (<0.1%)	1 (<0.1%)
Anomaly of external ear congenital	0	1 (<0.1%)	1 (<0.1%)
Atrial septal defect	1 (<0.1%)	0	1 (<0.1%)
Bicuspid aortic valve	0	1 (<0.1%)	1 (<0.1%)
Birth mark	0	1 (<0.1%)	1 (<0.1%)
Branchial cleft cyst	1 (<0.1%)	0	1 (<0.1%)
Congenital foot malformation	1 (<0.1%)	1 (<0.1%)	2 (<0.1%)
Congenital hand malformation	0	1 (<0.1%)	1 (<0.1%)
Congenital intestinal malformation	0	1 (<0.1%)	1 (<0.1%)
Congenital knee deformity	1 (<0.1%)	0	1 (<0.1%)
Congenital ureteric anomaly	0	1 (<0.1%)	1 (<0.1%)
Deafness congenital	0	1 (<0.1%)	1 (<0.1%)
Dermoid cyst	0	1 (<0.1%)	1 (<0.1%)
Ehlers-Danlos syndrome	2 (0.1%)	0	2 (<0.1%)
Factor V Leiden mutation	2 (0.1%)	2 (0.1%)	4 (0.1%)
Fibrous dysplasia of bone	0	1 (<0.1%)	1 (<0.1%)
Foetal malformation	0	1 (<0.1%)	1 (<0.1%)
Gene mutation	1 (<0.1%)	0	1 (<0.1%)
Gilbert's syndrome	1 (<0.1%)	0	1 (<0.1%)
Haemangioma congenital	1 (<0.1%)	0	1 (<0.1%)
Hereditary spherocytosis	1 (<0.1%)	0	1 (<0.1%)
Hip dysplasia	3 (0.2%)	0	3 (0.1%)
Pelvic kidney	1 (<0.1%)	0	1 (<0.1%)
Peroneal muscular atrophy	0	1 (<0.1%)	1 (<0.1%)
Protein S deficiency	1 (<0.1%)	0	1 (<0.1%)
Pulmonary malformation	1 (<0.1%)	0	1 (<0.1%)
Pyloric stenosis	1 (<0.1%)	0	1 (<0.1%)
Retinal anomaly congenital	1 (<0.1%)	0	1 (<0.1%)
Sickle cell trait	1 (<0.1%)	3 (0.2%)	4 (0.1%)
Solitary kidney	0	1 (<0.1%)	1 (<0.1%)
Syndactyly	1 (<0.1%)	0	1 (<0.1%)
Talipes	1 (<0.1%)	0	1 (<0.1%)
Urethral intrinsic sphincter deficiency	1 (<0.1%)	0	1 (<0.1%)
Ventricular septal defect	2 (0.1%)	3 (0.2%)	5 (0.2%)
Von Willebrand's disease	1 (<0.1%)	4 (0.3%)	5 (0.2%)

Table 14.3.1 / 4: Number of subjects with baseline findings or medical history by MedDRA SOC/PT (FAS)

Primary system organ class<br>Preferred term<br>MedDRA version 14.0	LCS12<br>N=1432 (100%)	LCS16<br>N=1452 (100%)	Total<br>N=2884 (100%)
Ear and labyrinth disorders	19 (1.3%)	10 (0.7%)	29 (1.0%)
Cerumen impaction	0	1 (<0.1%)	1 (<0.1%)
Deafness bilateral	2 (0.1%)	0	2 (<0.1%)
Ear discomfort	1 (<0.1%)	0	1 (<0.1%)
Hearing impaired	1 (<0.1%)	1 (<0.1%)	2 (<0.1%)
Hypoacusis	1 (<0.1%)	0	1 (<0.1%)
Meniere's disease	2 (0.1%)	0	2 (<0.1%)
Motion sickness	2 (0.1%)	1 (<0.1%)	3 (0.1%)
Otosclerosis	0	1 (<0.1%)	1 (<0.1%)
Tinnitus	4 (0.3%)	2 (0.1%)	6 (0.2%)
Tympanic membrane perforation	6 (0.4%)	1 (<0.1%)	7 (0.2%)
Tympanic membrane scarring	1 (<0.1%)	0	1 (<0.1%)
Vertigo	1 (<0.1%)	3 (0.2%)	4 (0.1%)
Endocrine disorders	42 (2.9%)	48 (3.3%)	90 (3.1%)
Basedow's disease	1 (<0.1%)	0	1 (<0.1%)
Goitre	3 (0.2%)	6 (0.4%)	9 (0.3%)
Hyperprolactinaemia	0	3 (0.2%)	3 (0.1%)
Hyperthyroidism	6 (0.4%)	3 (0.2%)	9 (0.3%)
Hypothyroidism	31 (2.2%)	36 (2.5%)	67 (2.3%)
Primary hyperaldosteronism	1 (<0.1%)	0	1 (<0.1%)
Thyroid cyst	2 (0.1%)	0	2 (<0.1%)
Thyroid dysfunction in pregnancy	0	1 (<0.1%)	1 (<0.1%)
Thyroiditis	1 (<0.1%)	4 (0.3%)	5 (0.2%)

Table 14.3.1 / 4: Number of subjects with baseline findings or medical history by MedDRA SOC/PT (FAS)

Primary system organ class<br>Preferred term<br>MedDRA version 14.0	LCS12<br>N=1432 (100%)	LCS16<br>N=1452 (100%)	Total<br>N=2884 (100%)
Eye disorders	83 (5.8%)	95 (6.5%)	178 (6.2%)
Amblyopia	0	3 (0.2%)	3 (0.1%)
Asthenopia	1 (<0.1%)	1 (<0.1%)	2 (<0.1%)
Astigmatism	7 (0.5%)	15 (1.0%)	22 (0.8%)
Blepharitis	1 (<0.1%)	0	1 (<0.1%)
Chalazion	0	1 (<0.1%)	1 (<0.1%)
Conjunctivitis	2 (0.1%)	4 (0.3%)	6 (0.2%)
Conjunctivitis allergic	1 (<0.1%)	1 (<0.1%)	2 (<0.1%)
Dry eye	1 (<0.1%)	3 (0.2%)	4 (0.1%)
Ectropion	0	1 (<0.1%)	1 (<0.1%)
Eye allergy	0	1 (<0.1%)	1 (<0.1%)
Eye pruritus	0	1 (<0.1%)	1 (<0.1%)
Eyelid ptosis	1 (<0.1%)	0	1 (<0.1%)
Hypermetropia	9 (0.6%)	9 (0.6%)	18 (0.6%)
Iritis	1 (<0.1%)	1 (<0.1%)	2 (<0.1%)
Keratoconus	1 (<0.1%)	0	1 (<0.1%)
Myopia	59 (4.1%)	56 (3.9%)	115 (4.0%)
Papilloedema	1 (<0.1%)	0	1 (<0.1%)
Presbyopia	0	1 (<0.1%)	1 (<0.1%)
Refraction disorder	0	1 (<0.1%)	1 (<0.1%)
Retinal detachment	1 (<0.1%)	0	1 (<0.1%)
Scotoma	1 (<0.1%)	0	1 (<0.1%)
Strabismus	0	3 (0.2%)	3 (0.1%)
Ulcerative keratitis	2 (0.1%)	0	2 (<0.1%)
Vision blurred	0	2 (0.1%)	2 (<0.1%)
Visual acuity reduced	1 (<0.1%)	1 (<0.1%)	2 (<0.1%)

Table 14.3.1 / 4: Number of subjects with baseline findings or medical history by MedDRA SOC/PT (FAS)

Primary system organ class<br>Preferred term<br>MedDRA version 14.0	LCS12<br>N=1432 (100%)	LCS16<br>N=1452 (100%)	Total<br>N=2884 (100%)
Gastrointestinal disorders	172 (12.0%)	149 (10.3%)	321 (11.1%)
Abdominal discomfort	1 (<0.1%)	2 (0.1%)	3 (0.1%)
Abdominal distension	2 (0.1%)	2 (0.1%)	4 (0.1%)
Abdominal hernia	1 (<0.1%)	0	1 (<0.1%)
Abdominal pain	15 (1.0%)	8 (0.6%)	23 (0.8%)
Abdominal pain lower	1 (<0.1%)	4 (0.3%)	5 (0.2%)
Abdominal pain upper	2 (0.1%)	5 (0.3%)	7 (0.2%)
Abdominal wall cyst	1 (<0.1%)	0	1 (<0.1%)
Anal fissure	1 (<0.1%)	1 (<0.1%)	2 (<0.1%)
Anal fistula	0	1 (<0.1%)	1 (<0.1%)
Bezoar	1 (<0.1%)	0	1 (<0.1%)
Bowel movement irregularity	0	1 (<0.1%)	1 (<0.1%)
Coeliac disease	5 (0.3%)	3 (0.2%)	8 (0.3%)
Colitis	2 (0.1%)	0	2 (<0.1%)
Colitis ulcerative	5 (0.3%)	3 (0.2%)	8 (0.3%)
Constipation	22 (1.5%)	22 (1.5%)	44 (1.5%)
Crohn's disease	3 (0.2%)	1 (<0.1%)	4 (0.1%)
Dental caries	0	1 (<0.1%)	1 (<0.1%)
Diarrhoea	9 (0.6%)	5 (0.3%)	14 (0.5%)
Dyspepsia	17 (1.2%)	12 (0.8%)	29 (1.0%)
Dysphagia	0	2 (0.1%)	2 (<0.1%)
Faecal incontinence	1 (<0.1%)	0	1 (<0.1%)
Femoral hernia	0	1 (<0.1%)	1 (<0.1%)
Flatulence	3 (0.2%)	1 (<0.1%)	4 (0.1%)
Food poisoning	0	1 (<0.1%)	1 (<0.1%)
Gastric disorder	2 (0.1%)	0	2 (<0.1%)
Gastric ulcer	7 (0.5%)	2 (0.1%)	9 (0.3%)
Gastritis	13 (0.9%)	11 (0.8%)	24 (0.8%)
Gastrointestinal ulcer	0	3 (0.2%)	3 (0.1%)
Gastrooesophageal reflux disease	21 (1.5%)	21 (1.4%)	42 (1.5%)
Glossitis	1 (<0.1%)	0	1 (<0.1%)
Haemorrhoids	12 (0.8%)	7 (0.5%)	19 (0.7%)
Hiatus hernia	2 (0.1%)	2 (0.1%)	4 (0.1%)
Ileus	1 (<0.1%)	0	1 (<0.1%)
Impaired gastric emptying	1 (<0.1%)	0	1 (<0.1%)
Inguinal hernia	4 (0.3%)	4 (0.3%)	8 (0.3%)
Irritable bowel syndrome	36 (2.5%)	21 (1.4%)	57 (2.0%)
Ischiorectal hernia	0	1 (<0.1%)	1 (<0.1%)
Malocclusion	2 (0.1%)	0	2 (<0.1%)
Nausea	4 (0.3%)	8 (0.6%)	12 (0.4%)
Necrotising colitis	1 (<0.1%)	0	1 (<0.1%)
Oesophagitis	0	1 (<0.1%)	1 (<0.1%)
Pancreatitis	0	2 (0.1%)	2 (<0.1%)
Pancreatitis acute	0	1 (<0.1%)	1 (<0.1%)

Table 14.3.1 / 4: Number of subjects with baseline findings or medical history by MedDRA SOC/PT (FAS)

Primary system organ class<br>Preferred term<br>MedDRA version 14.0	LCS12<br>N=1432 (100%)	LCS16<br>N=1452 (100%)	Total<br>N=2884 (100%)
Parotid gland inflammation	0	1 (<0.1%)	1 (<0.1%)
Peptic ulcer	1 (<0.1%)	0	1 (<0.1%)
Periodontitis	0	1 (<0.1%)	1 (<0.1%)
Peritonitis	0	1 (<0.1%)	1 (<0.1%)
Proctalgia	1 (<0.1%)	0	1 (<0.1%)
Rectal fissure	1 (<0.1%)	1 (<0.1%)	2 (<0.1%)
Rectal haemorrhage	0	2 (0.1%)	2 (<0.1%)
Reflux oesophagitis	1 (<0.1%)	0	1 (<0.1%)
Tooth disorder	1 (<0.1%)	0	1 (<0.1%)
Tooth impacted	0	2 (0.1%)	2 (<0.1%)
Toothache	2 (0.1%)	3 (0.2%)	5 (0.2%)
Umbilical hernia	4 (0.3%)	2 (0.1%)	6 (0.2%)
Vomiting	0	1 (<0.1%)	1 (<0.1%)
General disorders and administration site conditions	37 (2.6%)	25 (1.7%)	62 (2.1%)
Asthenia	3 (0.2%)	1 (<0.1%)	4 (0.1%)
Capsular contracture associated with breast implant	0	1 (<0.1%)	1 (<0.1%)
Chest discomfort	1 (<0.1%)	0	1 (<0.1%)
Chest pain	1 (<0.1%)	2 (0.1%)	3 (0.1%)
Chronic fatigue syndrome	1 (<0.1%)	0	1 (<0.1%)
Cyst	2 (0.1%)	1 (<0.1%)	3 (0.1%)
Device difficult to use	1 (<0.1%)	0	1 (<0.1%)
Device dislocation	0	1 (<0.1%)	1 (<0.1%)
Drug intolerance	2 (0.1%)	1 (<0.1%)	3 (0.1%)
Facial pain	1 (<0.1%)	0	1 (<0.1%)
Fatigue	9 (0.6%)	5 (0.3%)	14 (0.5%)
Haemorrhagic cyst	1 (<0.1%)	0	1 (<0.1%)
Hernia	3 (0.2%)	2 (0.1%)	5 (0.2%)
Influenza like illness	1 (<0.1%)	0	1 (<0.1%)
Irritability	1 (<0.1%)	2 (0.1%)	3 (0.1%)
Medical device pain	1 (<0.1%)	0	1 (<0.1%)
Metaplasia	0	1 (<0.1%)	1 (<0.1%)
Oedema peripheral	2 (0.1%)	2 (0.1%)	4 (0.1%)
Pain	7 (0.5%)	6 (0.4%)	13 (0.5%)
Pyrexia	1 (<0.1%)	1 (<0.1%)	2 (<0.1%)
Unevaluable event	1 (<0.1%)	0	1 (<0.1%)

Table 14.3.1 / 4: Number of subjects with baseline findings or medical history by MedDRA SOC/PT (FAS)

Primary system organ class<br>Preferred term<br>MedDRA version 14.0	LCS12<br>N=1432 (100%)	LCS16<br>N=1452 (100%)	Total<br>N=2884 (100%)
Hepatobiliary disorders	23 (1.6%)	20 (1.4%)	43 (1.5%)
Cholecystitis	0	3 (0.2%)	3 (0.1%)
Cholelithiasis	16 (1.1%)	12 (0.8%)	28 (1.0%)
Cholestasis	0	1 (<0.1%)	1 (<0.1%)
Cholestasis of pregnancy	3 (0.2%)	0	3 (0.1%)
Gallbladder disorder	3 (0.2%)	1 (<0.1%)	4 (0.1%)
Hepatic steatosis	1 (<0.1%)	2 (0.1%)	3 (0.1%)
Hepatitis	0	1 (<0.1%)	1 (<0.1%)
Immune system disorders	283 (19.8%)	310 (21.3%)	593 (20.6%)
Allergy to animal	23 (1.6%)	35 (2.4%)	58 (2.0%)
Allergy to arthropod bite	0	1 (<0.1%)	1 (<0.1%)
Allergy to arthropod sting	5 (0.3%)	1 (<0.1%)	6 (0.2%)
Allergy to chemicals	1 (<0.1%)	5 (0.3%)	6 (0.2%)
Allergy to metals	9 (0.6%)	7 (0.5%)	16 (0.6%)
Allergy to plants	1 (<0.1%)	1 (<0.1%)	2 (<0.1%)
Allergy to vaccine	1 (<0.1%)	1 (<0.1%)	2 (<0.1%)
Anaphylactic shock	1 (<0.1%)	0	1 (<0.1%)
Atopy	7 (0.5%)	5 (0.3%)	12 (0.4%)
Drug hypersensitivity	100 (7.0%)	103 (7.1%)	203 (7.0%)
Food allergy	19 (1.3%)	28 (1.9%)	47 (1.6%)
House dust allergy	12 (0.8%)	10 (0.7%)	22 (0.8%)
Hypersensitivity	18 (1.3%)	24 (1.7%)	42 (1.5%)
Iodine allergy	0	3 (0.2%)	3 (0.1%)
Latex allergy	3 (0.2%)	9 (0.6%)	12 (0.4%)
Milk allergy	1 (<0.1%)	2 (0.1%)	3 (0.1%)
Multiple allergies	3 (0.2%)	5 (0.3%)	8 (0.3%)
Mycotic allergy	1 (<0.1%)	2 (0.1%)	3 (0.1%)
Perennial allergy	0	1 (<0.1%)	1 (<0.1%)
Perfume sensitivity	1 (<0.1%)	0	1 (<0.1%)
Seasonal allergy	145 (10.1%)	154 (10.6%)	299 (10.4%)

Table 14.3.1 / 4: Number of subjects with baseline findings or medical history by MedDRA SOC/PT (FAS)

Primary system organ class<br>Preferred term<br>MedDRA version 14.0	LCS12<br>N=1432 (100%)	LCS16<br>N=1452 (100%)	Total<br>N=2884 (100%)
Infections and infestations	478 (33.4%)	452 (31.1%)	930 (32.2%)
Acute tonsillitis	2 (0.1%)	2 (0.1%)	4 (0.1%)
Anogenital warts	29 (2.0%)	12 (0.8%)	41 (1.4%)
Appendicitis	28 (2.0%)	25 (1.7%)	53 (1.8%)
Appendicitis perforated	1 (<0.1%)	0	1 (<0.1%)
Bacteriuria	1 (<0.1%)	0	1 (<0.1%)
Body tinea	1 (<0.1%)	0	1 (<0.1%)
Breast abscess	1 (<0.1%)	2 (0.1%)	3 (0.1%)
Breast infection	1 (<0.1%)	0	1 (<0.1%)
Bronchitis	10 (0.7%)	13 (0.9%)	23 (0.8%)
Candidiasis	5 (0.3%)	7 (0.5%)	12 (0.4%)
Cellulitis	1 (<0.1%)	2 (0.1%)	3 (0.1%)
Cervicitis	5 (0.3%)	7 (0.5%)	12 (0.4%)
Cervicitis gonococcal	1 (<0.1%)	0	1 (<0.1%)
Cervicitis trichomonal	1 (<0.1%)	0	1 (<0.1%)
Chlamydial cervicitis	21 (1.5%)	21 (1.4%)	42 (1.5%)
Chlamydial infection	10 (0.7%)	13 (0.9%)	23 (0.8%)
Chronic sinusitis	9 (0.6%)	2 (0.1%)	11 (0.4%)
Chronic tonsillitis	6 (0.4%)	6 (0.4%)	12 (0.4%)
Clostridial infection	0	2 (0.1%)	2 (<0.1%)
Coccidioidomycosis	2 (0.1%)	0	2 (<0.1%)
Conjunctivitis infective	0	1 (<0.1%)	1 (<0.1%)
Cystitis	12 (0.8%)	10 (0.7%)	22 (0.8%)
Cytomegalovirus infection	0	1 (<0.1%)	1 (<0.1%)
Diverticulitis	1 (<0.1%)	0	1 (<0.1%)
Ear infection	7 (0.5%)	10 (0.7%)	17 (0.6%)
Eczema infected	1 (<0.1%)	0	1 (<0.1%)
Encephalitic infection	0	1 (<0.1%)	1 (<0.1%)
Endometritis decidual	1 (<0.1%)	0	1 (<0.1%)
Enterocolitis bacterial	0	1 (<0.1%)	1 (<0.1%)
Enterocolitis infectious	1 (<0.1%)	1 (<0.1%)	2 (<0.1%)
Epstein-Barr virus infection	0	1 (<0.1%)	1 (<0.1%)
Folliculitis	2 (0.1%)	2 (0.1%)	4 (0.1%)
Fungal infection	31 (2.2%)	40 (2.8%)	71 (2.5%)
Fungal skin infection	1 (<0.1%)	1 (<0.1%)	2 (<0.1%)
Gardnerella infection	0	1 (<0.1%)	1 (<0.1%)
Gastroenteritis	3 (0.2%)	3 (0.2%)	6 (0.2%)
Gastroenteritis salmonella	1 (<0.1%)	0	1 (<0.1%)
Gastroenteritis viral	0	1 (<0.1%)	1 (<0.1%)
Genital candidiasis	0	2 (0.1%)	2 (<0.1%)
Genital herpes	16 (1.1%)	20 (1.4%)	36 (1.2%)
Genital infection bacterial	1 (<0.1%)	0	1 (<0.1%)
Genital infection fungal	1 (<0.1%)	1 (<0.1%)	2 (<0.1%)
Genital infection viral	2 (0.1%)	1 (<0.1%)	3 (0.1%)

Table 14.3.1 / 4: Number of subjects with baseline findings or medical history by MedDRA SOC/PT (FAS)

Primary system organ class<br>Preferred term<br>MedDRA version 14.0	LCS12<br>N=1432 (100%)	LCS16<br>N=1452 (100%)	Total<br>N=2884 (100%)
Genitourinary chlamydia infection	5 (0.3%)	4 (0.3%)	9 (0.3%)
Giardiasis	0	1 (<0.1%)	1 (<0.1%)
Gonorrhoea	7 (0.5%)	5 (0.3%)	12 (0.4%)
Gynaecological chlamydia infection	8 (0.6%)	6 (0.4%)	14 (0.5%)
Hand-foot-and-mouth disease	1 (<0.1%)	0	1 (<0.1%)
Helicobacter gastritis	1 (<0.1%)	0	1 (<0.1%)
Helicobacter infection	1 (<0.1%)	0	1 (<0.1%)
Hepatitis A	2 (0.1%)	8 (0.6%)	10 (0.3%)
Hepatitis B	1 (<0.1%)	0	1 (<0.1%)
Herpes dermatitis	1 (<0.1%)	0	1 (<0.1%)
Herpes ophthalmic	1 (<0.1%)	0	1 (<0.1%)
Herpes simplex	7 (0.5%)	6 (0.4%)	13 (0.5%)
Herpes virus infection	0	1 (<0.1%)	1 (<0.1%)
Herpes zoster	0	2 (0.1%)	2 (<0.1%)
Hordeolum	0	2 (0.1%)	2 (<0.1%)
Infection	0	1 (<0.1%)	1 (<0.1%)
Infectious mononucleosis	7 (0.5%)	4 (0.3%)	11 (0.4%)
Influenza	15 (1.0%)	15 (1.0%)	30 (1.0%)
Kidney infection	7 (0.5%)	11 (0.8%)	18 (0.6%)
Labyrinthitis	1 (<0.1%)	0	1 (<0.1%)
Lactobacillus infection	1 (<0.1%)	0	1 (<0.1%)
Laryngitis	3 (0.2%)	2 (0.1%)	5 (0.2%)
Localised infection	1 (<0.1%)	2 (0.1%)	3 (0.1%)
Lower respiratory tract infection	0	1 (<0.1%)	1 (<0.1%)
Lyme disease	2 (0.1%)	0	2 (<0.1%)
Lymph node tuberculosis	0	1 (<0.1%)	1 (<0.1%)
Mastitis	2 (0.1%)	0	2 (<0.1%)
Mastitis postpartum	0	1 (<0.1%)	1 (<0.1%)
Mastoiditis	1 (<0.1%)	0	1 (<0.1%)
Meningitis	1 (<0.1%)	1 (<0.1%)	2 (<0.1%)
Meningitis viral	1 (<0.1%)	2 (0.1%)	3 (0.1%)
Molluscum contagiosum	1 (<0.1%)	1 (<0.1%)	2 (<0.1%)
Nasopharyngitis	27 (1.9%)	28 (1.9%)	55 (1.9%)
Onychomycosis	2 (0.1%)	3 (0.2%)	5 (0.2%)
Oral herpes	8 (0.6%)	12 (0.8%)	20 (0.7%)
Osteomyelitis	2 (0.1%)	1 (<0.1%)	3 (0.1%)
Otitis externa	3 (0.2%)	0	3 (0.1%)
Otitis media	4 (0.3%)	3 (0.2%)	7 (0.2%)
Otitis media bacterial	1 (<0.1%)	0	1 (<0.1%)
Papilloma viral infection	2 (0.1%)	2 (0.1%)	4 (0.1%)
Paratyphoid fever	0	1 (<0.1%)	1 (<0.1%)
Paronychia	0	1 (<0.1%)	1 (<0.1%)
Pelvic infection	1 (<0.1%)	1 (<0.1%)	2 (<0.1%)
Pelvic inflammatory disease	2 (0.1%)	1 (<0.1%)	3 (0.1%)

Table 14.3.1 / 4: Number of subjects with baseline findings or medical history by MedDRA SOC/PT (FAS)

Primary system organ class<br>Preferred term<br>MedDRA version 14.0	LCS12<br>N=1432 (100%)	LCS16<br>N=1452 (100%)	Total<br>N=2884 (100%)
Periodontal infection	1 (<0.1%)	0	1 (<0.1%)
Peritonsillar abscess	0	1 (<0.1%)	1 (<0.1%)
Peritonsillitis	0	1 (<0.1%)	1 (<0.1%)
Pharyngitis	7 (0.5%)	4 (0.3%)	11 (0.4%)
Pharyngitis streptococcal	4 (0.3%)	9 (0.6%)	13 (0.5%)
Pilonidal cyst	1 (<0.1%)	1 (<0.1%)	2 (<0.1%)
Pneumonia	11 (0.8%)	14 (1.0%)	25 (0.9%)
Pneumonia mycoplasmal	0	1 (<0.1%)	1 (<0.1%)
Postoperative wound infection	1 (<0.1%)	0	1 (<0.1%)
Pseudofolliculitis barbae	0	1 (<0.1%)	1 (<0.1%)
Pyelonephritis	4 (0.3%)	0	4 (0.1%)
Pyelonephritis acute	0	1 (<0.1%)	1 (<0.1%)
Respiratory tract infection	5 (0.3%)	2 (0.1%)	7 (0.2%)
Rhinitis	3 (0.2%)	1 (<0.1%)	4 (0.1%)
Rubella	1 (<0.1%)	0	1 (<0.1%)
Salpingitis	0	1 (<0.1%)	1 (<0.1%)
Scarlet fever	0	3 (0.2%)	3 (0.1%)
Sinobronchitis	0	1 (<0.1%)	1 (<0.1%)
Sinusitis	37 (2.6%)	33 (2.3%)	70 (2.4%)
Skin infection	3 (0.2%)	2 (0.1%)	5 (0.2%)
Staphylococcal infection	0	2 (0.1%)	2 (<0.1%)
Staphylococcal skin infection	1 (<0.1%)	0	1 (<0.1%)
Syphilis	0	1 (<0.1%)	1 (<0.1%)
Tinea infection	1 (<0.1%)	1 (<0.1%)	2 (<0.1%)
Tinea pedis	3 (0.2%)	0	3 (0.1%)
Tinea versicolour	1 (<0.1%)	1 (<0.1%)	2 (<0.1%)
Tonsillitis	26 (1.8%)	20 (1.4%)	46 (1.6%)
Tooth abscess	1 (<0.1%)	2 (0.1%)	3 (0.1%)
Tooth infection	4 (0.3%)	4 (0.3%)	8 (0.3%)
Trichomoniasis	0	2 (0.1%)	2 (<0.1%)
Tuberculosis	0	3 (0.2%)	3 (0.1%)
Typhoid fever	1 (<0.1%)	0	1 (<0.1%)
Upper respiratory tract infection	12 (0.8%)	17 (1.2%)	29 (1.0%)
Urethritis	1 (<0.1%)	0	1 (<0.1%)
Urinary tract infection	77 (5.4%)	70 (4.8%)	147 (5.1%)
Uterine infection	1 (<0.1%)	0	1 (<0.1%)
Vaginal infection	17 (1.2%)	18 (1.2%)	35 (1.2%)
Vaginitis bacterial	59 (4.1%)	43 (3.0%)	102 (3.5%)
Vaginitis chlamydial	15 (1.0%)	13 (0.9%)	28 (1.0%)
Vaginitis gardnerella	0	1 (<0.1%)	1 (<0.1%)
Varicella	4 (0.3%)	2 (0.1%)	6 (0.2%)
Viral upper respiratory tract infection	1 (<0.1%)	3 (0.2%)	4 (0.1%)
Vulval abscess	0	1 (<0.1%)	1 (<0.1%)
Vulvitis	4 (0.3%)	0	4 (0.1%)

Table 14.3.1 / 4: Number of subjects with baseline findings or medical history by MedDRA SOC/PT (FAS)

Primary system organ class<br>Preferred term<br>MedDRA version 14.0	LCS12<br>N=1432 (100%)	LCS16<br>N=1452 (100%)	Total<br>N=2884 (100%)
Vulvovaginal candidiasis	27 (1.9%)	20 (1.4%)	47 (1.6%)
Vulvovaginal mycotic infection	21 (1.5%)	21 (1.4%)	42 (1.5%)
Vulvovaginitis	2 (0.1%)	0	2 (<0.1%)
Vulvovaginitis streptococcal	0	3 (0.2%)	3 (0.1%)
Vulvovaginitis trichomonal	3 (0.2%)	3 (0.2%)	6 (0.2%)

Table 14.3.1 / 4: Number of subjects with baseline findings or medical history by MedDRA SOC/PT (FAS)

Primary system organ class<br>Preferred term<br>MedDRA version 14.0	LCS12<br>N=1432 (100%)	LCS16<br>N=1452 (100%)	Total<br>N=2884 (100%)
Injury, poisoning and procedural complications	76 (5.3%)	106 (7.3%)	182 (6.3%)
Animal bite	1 (<0.1%)	0	1 (<0.1%)
Ankle fracture	2 (0.1%)	9 (0.6%)	11 (0.4%)
Arthropod bite	2 (0.1%)	0	2 (<0.1%)
Avulsion fracture	0	1 (<0.1%)	1 (<0.1%)
Brain contusion	0	1 (<0.1%)	1 (<0.1%)
Cartilage injury	0	2 (0.1%)	2 (<0.1%)
Cervical vertebral fracture	1 (<0.1%)	1 (<0.1%)	2 (<0.1%)
Clavicle fracture	2 (0.1%)	3 (0.2%)	5 (0.2%)
Collapse of lung	1 (<0.1%)	0	1 (<0.1%)
Concussion	4 (0.3%)	3 (0.2%)	7 (0.2%)
Contusion	2 (0.1%)	1 (<0.1%)	3 (0.1%)
Dislocation of vertebra	0	1 (<0.1%)	1 (<0.1%)
Epicondylitis	0	1 (<0.1%)	1 (<0.1%)
Face injury	1 (<0.1%)	0	1 (<0.1%)
Facial bones fracture	3 (0.2%)	4 (0.3%)	7 (0.2%)
Fibula fracture	0	1 (<0.1%)	1 (<0.1%)
Foot fracture	6 (0.4%)	6 (0.4%)	12 (0.4%)
Forearm fracture	1 (<0.1%)	1 (<0.1%)	2 (<0.1%)
Foreign body	0	1 (<0.1%)	1 (<0.1%)
Fractured coccyx	1 (<0.1%)	0	1 (<0.1%)
Hand fracture	3 (0.2%)	4 (0.3%)	7 (0.2%)
Head injury	1 (<0.1%)	4 (0.3%)	5 (0.2%)
Hip fracture	0	2 (0.1%)	2 (<0.1%)
Humerus fracture	1 (<0.1%)	2 (0.1%)	3 (0.1%)
Incision site pain	0	1 (<0.1%)	1 (<0.1%)
Injury	0	2 (0.1%)	2 (<0.1%)
Jaw fracture	1 (<0.1%)	0	1 (<0.1%)
Joint dislocation	2 (0.1%)	5 (0.3%)	7 (0.2%)
Joint injury	5 (0.3%)	2 (0.1%)	7 (0.2%)
Joint sprain	4 (0.3%)	6 (0.4%)	10 (0.3%)
Ligament rupture	1 (<0.1%)	7 (0.5%)	8 (0.3%)
Limb injury	5 (0.3%)	1 (<0.1%)	6 (0.2%)
Lower limb fracture	3 (0.2%)	4 (0.3%)	7 (0.2%)
Lumbar vertebral fracture	1 (<0.1%)	0	1 (<0.1%)
Meniscus lesion	2 (0.1%)	5 (0.3%)	7 (0.2%)
Muscle injury	0	2 (0.1%)	2 (<0.1%)
Muscle strain	0	2 (0.1%)	2 (<0.1%)
Neck injury	1 (<0.1%)	1 (<0.1%)	2 (<0.1%)
Pelvic fracture	0	1 (<0.1%)	1 (<0.1%)
Post procedural complication	1 (<0.1%)	0	1 (<0.1%)
Post-traumatic pain	0	2 (0.1%)	2 (<0.1%)
Procedural pain	3 (0.2%)	1 (<0.1%)	4 (0.1%)
Procedural site reaction	0	1 (<0.1%)	1 (<0.1%)

Table 14.3.1 / 4: Number of subjects with baseline findings or medical history by MedDRA SOC/PT (FAS)

Primary system organ class<br>Preferred term<br>MedDRA version 14.0	LCS12<br>N=1432 (100%)	LCS16<br>N=1452 (100%)	Total<br>N=2884 (100%)
Radius fracture	1 (<0.1%)	0	1 (<0.1%)
Rib fracture	2 (0.1%)	2 (0.1%)	4 (0.1%)
Road traffic accident	1 (<0.1%)	2 (0.1%)	3 (0.1%)
Sciatic nerve injury	1 (<0.1%)	0	1 (<0.1%)
Spinal column injury	0	1 (<0.1%)	1 (<0.1%)
Spinal fracture	0	1 (<0.1%)	1 (<0.1%)
Splenic rupture	1 (<0.1%)	0	1 (<0.1%)
Synovial rupture	0	1 (<0.1%)	1 (<0.1%)
Tendon injury	0	1 (<0.1%)	1 (<0.1%)
Tendon rupture	1 (<0.1%)	1 (<0.1%)	2 (<0.1%)
Thermal burn	1 (<0.1%)	2 (0.1%)	3 (0.1%)
Tibia fracture	0	1 (<0.1%)	1 (<0.1%)
Tooth fracture	1 (<0.1%)	0	1 (<0.1%)
Tooth injury	0	1 (<0.1%)	1 (<0.1%)
Traumatic brain injury	1 (<0.1%)	0	1 (<0.1%)
Traumatic haematoma	1 (<0.1%)	0	1 (<0.1%)
Ulna fracture	2 (0.1%)	0	2 (<0.1%)
Upper limb fracture	8 (0.6%)	14 (1.0%)	22 (0.8%)
Uterine perforation	1 (<0.1%)	0	1 (<0.1%)
Uterine rupture	1 (<0.1%)	0	1 (<0.1%)
Whiplash injury	0	4 (0.3%)	4 (0.1%)
Wound	2 (0.1%)	0	2 (<0.1%)
Wrist fracture	6 (0.4%)	8 (0.6%)	14 (0.5%)

Table 14.3.1 / 4: Number of subjects with baseline findings or medical history by MedDRA SOC/PT (FAS)

Primary system organ class<br>Preferred term<br>MedDRA version 14.0	LCS12<br>N=1432 (100%)	LCS16<br>N=1452 (100%)	Total<br>N=2884 (100%)
Investigations	210 (14.7%)	222 (15.3%)	432 (15.0%)
Alanine aminotransferase increased	10 (0.7%)	8 (0.6%)	18 (0.6%)
Arthroscopy	10 (0.7%)	12 (0.8%)	22 (0.8%)
Aspartate aminotransferase increased	2 (0.1%)	2 (0.1%)	4 (0.1%)
Aspiration breast	0	1 (<0.1%)	1 (<0.1%)
Biopsy	1 (<0.1%)	0	1 (<0.1%)
Biopsy breast	3 (0.2%)	4 (0.3%)	7 (0.2%)
Biopsy breast normal	1 (<0.1%)	0	1 (<0.1%)
Biopsy cervix	8 (0.6%)	7 (0.5%)	15 (0.5%)
Biopsy kidney	1 (<0.1%)	0	1 (<0.1%)
Biopsy site unspecified normal	0	1 (<0.1%)	1 (<0.1%)
Biopsy thyroid gland	0	1 (<0.1%)	1 (<0.1%)
Blood albumin decreased	1 (<0.1%)	0	1 (<0.1%)
Blood albumin increased	1 (<0.1%)	0	1 (<0.1%)
Blood alkaline phosphatase increased	1 (<0.1%)	0	1 (<0.1%)
Blood cholesterol decreased	1 (<0.1%)	0	1 (<0.1%)
Blood cholesterol increased	6 (0.4%)	4 (0.3%)	10 (0.3%)
Blood creatinine decreased	2 (0.1%)	6 (0.4%)	8 (0.3%)
Blood iron decreased	1 (<0.1%)	2 (0.1%)	3 (0.1%)
Blood potassium decreased	1 (<0.1%)	0	1 (<0.1%)
Blood potassium increased	5 (0.3%)	5 (0.3%)	10 (0.3%)
Blood pressure increased	1 (<0.1%)	0	1 (<0.1%)
Blood pressure systolic increased	0	1 (<0.1%)	1 (<0.1%)
Blood sodium decreased	0	1 (<0.1%)	1 (<0.1%)
Blood sodium increased	4 (0.3%)	2 (0.1%)	6 (0.2%)
Blood testosterone increased	1 (<0.1%)	0	1 (<0.1%)
Blood triglycerides increased	6 (0.4%)	10 (0.7%)	16 (0.6%)
Blood urine present	1 (<0.1%)	2 (0.1%)	3 (0.1%)
Cardiac murmur	8 (0.6%)	2 (0.1%)	10 (0.3%)
Chlamydia test	1 (<0.1%)	0	1 (<0.1%)
Chlamydia test positive	12 (0.8%)	14 (1.0%)	26 (0.9%)
Colonoscopy	1 (<0.1%)	5 (0.3%)	6 (0.2%)
Colposcopy	28 (2.0%)	24 (1.7%)	52 (1.8%)
Colposcopy normal	0	1 (<0.1%)	1 (<0.1%)
Cystoscopy	0	1 (<0.1%)	1 (<0.1%)
Endoscopic retrograde cholangiopancreatography	0	1 (<0.1%)	1 (<0.1%)
Endoscopy	2 (0.1%)	1 (<0.1%)	3 (0.1%)
Endoscopy upper gastrointestinal tract	1 (<0.1%)	1 (<0.1%)	2 (<0.1%)
Fungal test positive	0	1 (<0.1%)	1 (<0.1%)
Gamma-glutamyltransferase increased	6 (0.4%)	6 (0.4%)	12 (0.4%)
Glycosylated haemoglobin increased	0	2 (0.1%)	2 (<0.1%)
Haematocrit decreased	2 (0.1%)	4 (0.3%)	6 (0.2%)
Haematocrit increased	1 (<0.1%)	0	1 (<0.1%)
Haematology test abnormal	0	1 (<0.1%)	1 (<0.1%)

Table 14.3.1 / 4: Number of subjects with baseline findings or medical history by MedDRA SOC/PT (FAS)

Primary system organ class<br>Preferred term<br>MedDRA version 14.0	LCS12<br>N=1432 (100%)	LCS16<br>N=1452 (100%)	Total<br>N=2884 (100%)
Haemoglobin decreased	6 (0.4%)	8 (0.6%)	14 (0.5%)
Haemoglobin increased	0	1 (<0.1%)	1 (<0.1%)
Haemoglobin urine present	0	2 (0.1%)	2 (<0.1%)
Heart rate increased	0	2 (0.1%)	2 (<0.1%)
Heart rate irregular	1 (<0.1%)	0	1 (<0.1%)
Heart sounds abnormal	1 (<0.1%)	0	1 (<0.1%)
Hepatic enzyme increased	2 (0.1%)	1 (<0.1%)	3 (0.1%)
High density lipoprotein abnormal	2 (0.1%)	0	2 (<0.1%)
High density lipoprotein decreased	3 (0.2%)	3 (0.2%)	6 (0.2%)
Human papilloma virus test negative	0	1 (<0.1%)	1 (<0.1%)
Human papilloma virus test positive	28 (2.0%)	31 (2.1%)	59 (2.0%)
Hysteroscopy	1 (<0.1%)	3 (0.2%)	4 (0.1%)
Laparoscopy	16 (1.1%)	12 (0.8%)	28 (1.0%)
Leukocyte antigen B-27 positive	0	1 (<0.1%)	1 (<0.1%)
Lipids increased	1 (<0.1%)	0	1 (<0.1%)
Liver function test abnormal	1 (<0.1%)	0	1 (<0.1%)
Low density lipoprotein decreased	0	1 (<0.1%)	1 (<0.1%)
Low density lipoprotein increased	7 (0.5%)	5 (0.3%)	12 (0.4%)
Mammogram abnormal	0	1 (<0.1%)	1 (<0.1%)
Oesophagogastroscopy	0	1 (<0.1%)	1 (<0.1%)
Paracentesis	0	1 (<0.1%)	1 (<0.1%)
Platelet count decreased	0	2 (0.1%)	2 (<0.1%)
Platelet count increased	0	1 (<0.1%)	1 (<0.1%)
Pregnancy test positive	1 (<0.1%)	0	1 (<0.1%)
Protein total decreased	2 (0.1%)	1 (<0.1%)	3 (0.1%)
Protein total increased	1 (<0.1%)	2 (0.1%)	3 (0.1%)
Red blood cell count decreased	0	1 (<0.1%)	1 (<0.1%)
Red blood cell count increased	1 (<0.1%)	1 (<0.1%)	2 (<0.1%)
Rhesus antibodies negative	0	1 (<0.1%)	1 (<0.1%)
Smear cervix abnormal	64 (4.5%)	49 (3.4%)	113 (3.9%)
Smear cervix normal	0	2 (0.1%)	2 (<0.1%)
Streptococcus test positive	0	1 (<0.1%)	1 (<0.1%)
Ultrasound uterus abnormal	1 (<0.1%)	0	1 (<0.1%)
Urine leukocyte esterase positive	2 (0.1%)	1 (<0.1%)	3 (0.1%)
Vitamin B12 decreased	0	1 (<0.1%)	1 (<0.1%)
Weight decreased	2 (0.1%)	1 (<0.1%)	3 (0.1%)
Weight increased	0	3 (0.2%)	3 (0.1%)
White blood cell count decreased	3 (0.2%)	7 (0.5%)	10 (0.3%)
White blood cell count increased	6 (0.4%)	9 (0.6%)	15 (0.5%)

Table 14.3.1 / 4: Number of subjects with baseline findings or medical history by MedDRA SOC/PT (FAS)

Primary system organ class<br>Preferred term<br>MedDRA version 14.0	LCS12<br>N=1432 (100%)	LCS16<br>N=1452 (100%)	Total<br>N=2884 (100%)
Metabolism and nutrition disorders	48 (3.4%)	54 (3.7%)	102 (3.5%)
Body fat disorder	0	1 (<0.1%)	1 (<0.1%)
Decreased appetite	1 (<0.1%)	1 (<0.1%)	2 (<0.1%)
Diabetes mellitus	0	1 (<0.1%)	1 (<0.1%)
Dyslipidaemia	1 (<0.1%)	0	1 (<0.1%)
Fluid retention	1 (<0.1%)	4 (0.3%)	5 (0.2%)
Food intolerance	1 (<0.1%)	0	1 (<0.1%)
Gestational diabetes	4 (0.3%)	10 (0.7%)	14 (0.5%)
Glucose tolerance impaired	1 (<0.1%)	0	1 (<0.1%)
Hypercholesterolaemia	7 (0.5%)	7 (0.5%)	14 (0.5%)
Hyperlipidaemia	3 (0.2%)	2 (0.1%)	5 (0.2%)
Hypertriglyceridaemia	1 (<0.1%)	0	1 (<0.1%)
Hypocholesterolaemia	1 (<0.1%)	0	1 (<0.1%)
Hypoglycaemia	2 (0.1%)	3 (0.2%)	5 (0.2%)
Hypoglycaemic seizure	1 (<0.1%)	0	1 (<0.1%)
Hypokalaemia	0	1 (<0.1%)	1 (<0.1%)
Insulin resistance	4 (0.3%)	6 (0.4%)	10 (0.3%)
Iron deficiency	0	2 (0.1%)	2 (<0.1%)
Lactose intolerance	11 (0.8%)	10 (0.7%)	21 (0.7%)
Obesity	7 (0.5%)	8 (0.6%)	15 (0.5%)
Overweight	2 (0.1%)	0	2 (<0.1%)
Type 1 diabetes mellitus	3 (0.2%)	1 (<0.1%)	4 (0.1%)

Table 14.3.1 / 4: Number of subjects with baseline findings or medical history by MedDRA SOC/PT (FAS)

Primary system organ class<br>Preferred term<br>MedDRA version 14.0	LCS12<br>N=1432 (100%)	LCS16<br>N=1452 (100%)	Total<br>N=2884 (100%)
Musculoskeletal and connective tissue disorders	141 (9.8%)	142 (9.8%)	283 (9.8%)
Ankylosing spondylitis	1 (<0.1%)	0	1 (<0.1%)
Arthralgia	21 (1.5%)	21 (1.4%)	42 (1.5%)
Arthritis	3 (0.2%)	4 (0.3%)	7 (0.2%)
Arthritis reactive	1 (<0.1%)	0	1 (<0.1%)
Arthropathy	1 (<0.1%)	0	1 (<0.1%)
Back disorder	1 (<0.1%)	0	1 (<0.1%)
Back pain	42 (2.9%)	50 (3.4%)	92 (3.2%)
Bone pain	0	1 (<0.1%)	1 (<0.1%)
Bunion	2 (0.1%)	0	2 (<0.1%)
Bursitis	1 (<0.1%)	2 (0.1%)	3 (0.1%)
Cartilage hypertrophy	1 (<0.1%)	0	1 (<0.1%)
Chondromalacia	0	1 (<0.1%)	1 (<0.1%)
Costochondritis	0	1 (<0.1%)	1 (<0.1%)
Fibromyalgia	2 (0.1%)	3 (0.2%)	5 (0.2%)
Foot deformity	1 (<0.1%)	3 (0.2%)	4 (0.1%)
Intervertebral disc degeneration	2 (0.1%)	0	2 (<0.1%)
Intervertebral disc displacement	0	1 (<0.1%)	1 (<0.1%)
Intervertebral disc protrusion	6 (0.4%)	6 (0.4%)	12 (0.4%)
Joint instability	1 (<0.1%)	0	1 (<0.1%)
Juvenile arthritis	3 (0.2%)	1 (<0.1%)	4 (0.1%)
Knee deformity	1 (<0.1%)	0	1 (<0.1%)
Ligament calcification	0	1 (<0.1%)	1 (<0.1%)
Medial tibial stress syndrome	1 (<0.1%)	0	1 (<0.1%)
Muscle contracture	1 (<0.1%)	0	1 (<0.1%)
Muscle disorder	0	1 (<0.1%)	1 (<0.1%)
Muscle spasms	4 (0.3%)	5 (0.3%)	9 (0.3%)
Muscle tightness	9 (0.6%)	3 (0.2%)	12 (0.4%)
Muscular weakness	1 (<0.1%)	1 (<0.1%)	2 (<0.1%)
Musculoskeletal chest pain	1 (<0.1%)	0	1 (<0.1%)
Musculoskeletal discomfort	1 (<0.1%)	0	1 (<0.1%)
Musculoskeletal pain	7 (0.5%)	10 (0.7%)	17 (0.6%)
Myalgia	9 (0.6%)	8 (0.6%)	17 (0.6%)
Myokymia	0	2 (0.1%)	2 (<0.1%)
Myositis	1 (<0.1%)	0	1 (<0.1%)
Neck mass	0	1 (<0.1%)	1 (<0.1%)
Neck pain	2 (0.1%)	8 (0.6%)	10 (0.3%)
Osteitis	0	1 (<0.1%)	1 (<0.1%)
Osteoarthritis	2 (0.1%)	2 (0.1%)	4 (0.1%)
Osteochondrosis	3 (0.2%)	0	3 (0.1%)
Osteonecrosis	1 (<0.1%)	0	1 (<0.1%)
Osteopenia	1 (<0.1%)	1 (<0.1%)	2 (<0.1%)
Pain in extremity	6 (0.4%)	7 (0.5%)	13 (0.5%)
Pain in jaw	0	1 (<0.1%)	1 (<0.1%)

Table 14.3.1 / 4: Number of subjects with baseline findings or medical history by MedDRA SOC/PT (FAS)

Primary system organ class<br>Preferred term<br>MedDRA version 14.0	LCS12<br>N=1432 (100%)	LCS16<br>N=1452 (100%)	Total<br>N=2884 (100%)
Patellofemoral pain syndrome	1 (<0.1%)	5 (0.3%)	6 (0.2%)
Plantar fasciitis	1 (<0.1%)	2 (0.1%)	3 (0.1%)
Rheumatoid arthritis	3 (0.2%)	2 (0.1%)	5 (0.2%)
Rotator cuff syndrome	0	1 (<0.1%)	1 (<0.1%)
Sacroiliitis	1 (<0.1%)	0	1 (<0.1%)
Scleroderma	0	1 (<0.1%)	1 (<0.1%)
Scoliosis	7 (0.5%)	7 (0.5%)	14 (0.5%)
Seronegative arthritis	0	1 (<0.1%)	1 (<0.1%)
Spinal osteoarthritis	0	1 (<0.1%)	1 (<0.1%)
Synovial cyst	1 (<0.1%)	2 (0.1%)	3 (0.1%)
Temporomandibular joint syndrome	4 (0.3%)	1 (<0.1%)	5 (0.2%)
Tendonitis	3 (0.2%)	7 (0.5%)	10 (0.3%)
Tenosynovitis	1 (<0.1%)	0	1 (<0.1%)
Tenosynovitis stenosans	0	1 (<0.1%)	1 (<0.1%)
Trigger finger	0	1 (<0.1%)	1 (<0.1%)

Table 14.3.1 / 4: Number of subjects with baseline findings or medical history by MedDRA SOC/PT (FAS)

Primary system organ class<br>Preferred term<br>MedDRA version 14.0	LCS12<br>N=1432 (100%)	LCS16<br>N=1452 (100%)	Total<br>N=2884 (100%)
Neoplasms benign, malignant and unspecified (incl cysts and polyps)	57 (4.0%)	65 (4.5%)	122 (4.2%)
Acrochordon	0	2 (0.1%)	2 (<0.1%)
Acute lymphocytic leukaemia	0	1 (<0.1%)	1 (<0.1%)
Adenoma benign	0	1 (<0.1%)	1 (<0.1%)
Ameloblastoma	0	1 (<0.1%)	1 (<0.1%)
Benign bone neoplasm	1 (<0.1%)	0	1 (<0.1%)
Benign breast neoplasm	0	6 (0.4%)	6 (0.2%)
Benign gastric neoplasm	0	1 (<0.1%)	1 (<0.1%)
Benign hydatidiform mole	0	1 (<0.1%)	1 (<0.1%)
Benign neoplasm	0	1 (<0.1%)	1 (<0.1%)
Benign neoplasm of cervix uteri	1 (<0.1%)	0	1 (<0.1%)
Benign neoplasm of skin	2 (0.1%)	2 (0.1%)	4 (0.1%)
Benign neoplasm of thyroid gland	1 (<0.1%)	0	1 (<0.1%)
Benign neoplasm of urethra	1 (<0.1%)	0	1 (<0.1%)
Benign salivary gland neoplasm	1 (<0.1%)	1 (<0.1%)	2 (<0.1%)
Bone giant cell tumour benign	1 (<0.1%)	0	1 (<0.1%)
Bone neoplasm	1 (<0.1%)	0	1 (<0.1%)
Breast fibroma	1 (<0.1%)	1 (<0.1%)	2 (<0.1%)
Cervicitis human papilloma virus	14 (1.0%)	16 (1.1%)	30 (1.0%)
Fibroadenoma of breast	10 (0.7%)	1 (<0.1%)	11 (0.4%)
Fibroma	0	1 (<0.1%)	1 (<0.1%)
Glomus tumour	1 (<0.1%)	0	1 (<0.1%)
Haemangioma of skin	1 (<0.1%)	1 (<0.1%)	2 (<0.1%)
Juvenile melanoma benign	0	1 (<0.1%)	1 (<0.1%)
Lipoma	4 (0.3%)	0	4 (0.1%)
Lipoma of breast	1 (<0.1%)	0	1 (<0.1%)
Melanocytic naevus	4 (0.3%)	2 (0.1%)	6 (0.2%)
Ovarian adenoma	1 (<0.1%)	0	1 (<0.1%)
Ovarian fibroma	1 (<0.1%)	0	1 (<0.1%)
Ovarian germ cell teratoma benign	2 (0.1%)	3 (0.2%)	5 (0.2%)
Prolactinoma	1 (<0.1%)	0	1 (<0.1%)
Skin papilloma	1 (<0.1%)	1 (<0.1%)	2 (<0.1%)
Uterine leiomyoma	6 (0.4%)	10 (0.7%)	16 (0.6%)
Vulvovaginal human papilloma virus infection	3 (0.2%)	13 (0.9%)	16 (0.6%)

Table 14.3.1 / 4: Number of subjects with baseline findings or medical history by MedDRA SOC/PT (FAS)

Primary system organ class<br>Preferred term<br>MedDRA version 14.0	LCS12<br>N=1432 (100%)	LCS16<br>N=1452 (100%)	Total<br>N=2884 (100%)
Nervous system disorders	364 (25.4%)	394 (27.1%)	758 (26.3%)
Ageusia	0	1 (<0.1%)	1 (<0.1%)
Benign intracranial hypertension	1 (<0.1%)	1 (<0.1%)	2 (<0.1%)
Carpal tunnel syndrome	3 (0.2%)	2 (0.1%)	5 (0.2%)
Central auditory processing disorder	1 (<0.1%)	0	1 (<0.1%)
Cerebral infarction	0	1 (<0.1%)	1 (<0.1%)
Cerebrovascular accident	1 (<0.1%)	0	1 (<0.1%)
Cervicobrachial syndrome	0	1 (<0.1%)	1 (<0.1%)
Cervicogenic headache	1 (<0.1%)	0	1 (<0.1%)
Cluster headache	2 (0.1%)	0	2 (<0.1%)
Coma	0	1 (<0.1%)	1 (<0.1%)
Complex regional pain syndrome	1 (<0.1%)	0	1 (<0.1%)
Convulsion	2 (0.1%)	6 (0.4%)	8 (0.3%)
Disturbance in attention	0	1 (<0.1%)	1 (<0.1%)
Dizziness	1 (<0.1%)	2 (0.1%)	3 (0.1%)
Epilepsy	6 (0.4%)	7 (0.5%)	13 (0.5%)
Essential tremor	0	1 (<0.1%)	1 (<0.1%)
Febrile convulsion	1 (<0.1%)	0	1 (<0.1%)
Guillain-Barre syndrome	0	1 (<0.1%)	1 (<0.1%)
Headache	209 (14.6%)	226 (15.6%)	435 (15.1%)
Hemianopia	1 (<0.1%)	0	1 (<0.1%)
Hypersomnia	0	1 (<0.1%)	1 (<0.1%)
Hypertonia	1 (<0.1%)	1 (<0.1%)	2 (<0.1%)
Memory impairment	0	1 (<0.1%)	1 (<0.1%)
Migraine	115 (8.0%)	127 (8.7%)	242 (8.4%)
Migraine with aura	6 (0.4%)	7 (0.5%)	13 (0.5%)
Migraine without aura	1 (<0.1%)	1 (<0.1%)	2 (<0.1%)
Myasthenia gravis	0	1 (<0.1%)	1 (<0.1%)
Myotonia	2 (0.1%)	0	2 (<0.1%)
Nerve compression	0	2 (0.1%)	2 (<0.1%)
Nystagmus	1 (<0.1%)	0	1 (<0.1%)
Optic neuritis	1 (<0.1%)	0	1 (<0.1%)
Paraesthesia	0	1 (<0.1%)	1 (<0.1%)
Paraparesis	1 (<0.1%)	0	1 (<0.1%)
Restless legs syndrome	2 (0.1%)	1 (<0.1%)	3 (0.1%)
Sciatica	5 (0.3%)	0	5 (0.2%)
Sinus headache	5 (0.3%)	5 (0.3%)	10 (0.3%)
Syncope	2 (0.1%)	1 (<0.1%)	3 (0.1%)
Tension headache	42 (2.9%)	43 (3.0%)	85 (2.9%)
Tremor	1 (<0.1%)	0	1 (<0.1%)
Vascular headache	0	1 (<0.1%)	1 (<0.1%)

Table 14.3.1 / 4: Number of subjects with baseline findings or medical history by MedDRA SOC/PT (FAS)

Primary system organ class<br>Preferred term<br>MedDRA version 14.0	LCS12<br>N=1432 (100%)	LCS16<br>N=1452 (100%)	Total<br>N=2884 (100%)
Pregnancy, puerperium and perinatal conditions	79 (5.5%)	90 (6.2%)	169 (5.9%)
Abortion	28 (2.0%)	25 (1.7%)	53 (1.8%)
Abortion incomplete	1 (<0.1%)	0	1 (<0.1%)
Abortion missed	0	1 (<0.1%)	1 (<0.1%)
Abortion spontaneous	16 (1.1%)	20 (1.4%)	36 (1.2%)
Abortion spontaneous incomplete	0	1 (<0.1%)	1 (<0.1%)
Antepartum haemorrhage	2 (0.1%)	3 (0.2%)	5 (0.2%)
Arrested labour	1 (<0.1%)	0	1 (<0.1%)
Cervical incompetence	1 (<0.1%)	0	1 (<0.1%)
Delivery	21 (1.5%)	31 (2.1%)	52 (1.8%)
Ectopic pregnancy	1 (<0.1%)	0	1 (<0.1%)
Foetal distress syndrome	3 (0.2%)	1 (<0.1%)	4 (0.1%)
Gestational hypertension	2 (0.1%)	4 (0.3%)	6 (0.2%)
HELLP syndrome	1 (<0.1%)	0	1 (<0.1%)
Placenta praevia haemorrhage	1 (<0.1%)	0	1 (<0.1%)
Placental insufficiency	1 (<0.1%)	0	1 (<0.1%)
Postpartum haemorrhage	1 (<0.1%)	3 (0.2%)	4 (0.1%)
Pre-eclampsia	4 (0.3%)	5 (0.3%)	9 (0.3%)
Pregnancy	1 (<0.1%)	1 (<0.1%)	2 (<0.1%)
Pregnancy with contraceptive device	1 (<0.1%)	1 (<0.1%)	2 (<0.1%)
Premature labour	1 (<0.1%)	0	1 (<0.1%)
Premature separation of placenta	0	1 (<0.1%)	1 (<0.1%)
Retained placenta or membranes	0	1 (<0.1%)	1 (<0.1%)
Stillbirth	0	1 (<0.1%)	1 (<0.1%)
Traumatic delivery	0	1 (<0.1%)	1 (<0.1%)
Twin pregnancy	1 (<0.1%)	1 (<0.1%)	2 (<0.1%)

Table 14.3.1 / 4: Number of subjects with baseline findings or medical history by MedDRA SOC/PT (FAS)

Primary system organ class<br>Preferred term<br>MedDRA version 14.0	LCS12<br>N=1432 (100%)	LCS16<br>N=1452 (100%)	Total<br>N=2884 (100%)
Psychiatric disorders	190 (13.3%)	195 (13.4%)	385 (13.3%)
Alcohol abuse	0	3 (0.2%)	3 (0.1%)
Alcoholism	0	1 (<0.1%)	1 (<0.1%)
Anorgasmia	0	2 (0.1%)	2 (<0.1%)
Anxiety	57 (4.0%)	60 (4.1%)	117 (4.1%)
Anxiety disorder	1 (<0.1%)	1 (<0.1%)	2 (<0.1%)
Attention deficit/hyperactivity disorder	8 (0.6%)	11 (0.8%)	19 (0.7%)
Bipolar I disorder	1 (<0.1%)	0	1 (<0.1%)
Bipolar disorder	1 (<0.1%)	7 (0.5%)	8 (0.3%)
Borderline personality disorder	0	2 (0.1%)	2 (<0.1%)
Brief psychotic disorder, with postpartum onset	0	1 (<0.1%)	1 (<0.1%)
Bruxism	1 (<0.1%)	0	1 (<0.1%)
Bulimia nervosa	1 (<0.1%)	2 (0.1%)	3 (0.1%)
Burnout syndrome	1 (<0.1%)	2 (0.1%)	3 (0.1%)
Depressed mood	1 (<0.1%)	1 (<0.1%)	2 (<0.1%)
Depression	101 (7.1%)	82 (5.6%)	183 (6.3%)
Drug abuse	0	4 (0.3%)	4 (0.1%)
Eating disorder	2 (0.1%)	0	2 (<0.1%)
Encopresis	0	1 (<0.1%)	1 (<0.1%)
Generalised anxiety disorder	3 (0.2%)	0	3 (0.1%)
Hypochondriasis	1 (<0.1%)	0	1 (<0.1%)
Insomnia	28 (2.0%)	36 (2.5%)	64 (2.2%)
Libido decreased	5 (0.3%)	7 (0.5%)	12 (0.4%)
Major depression	1 (<0.1%)	0	1 (<0.1%)
Mood altered	1 (<0.1%)	0	1 (<0.1%)
Mood swings	2 (0.1%)	2 (0.1%)	4 (0.1%)
Nicotine dependence	1 (<0.1%)	0	1 (<0.1%)
Obsessive-compulsive disorder	2 (0.1%)	1 (<0.1%)	3 (0.1%)
Panic attack	2 (0.1%)	5 (0.3%)	7 (0.2%)
Panic disorder	2 (0.1%)	4 (0.3%)	6 (0.2%)
Panic reaction	0	1 (<0.1%)	1 (<0.1%)
Phobia	1 (<0.1%)	0	1 (<0.1%)
Phobia of flying	1 (<0.1%)	1 (<0.1%)	2 (<0.1%)
Post-traumatic stress disorder	0	1 (<0.1%)	1 (<0.1%)
Postpartum depression	8 (0.6%)	12 (0.8%)	20 (0.7%)
Psychotic disorder	0	1 (<0.1%)	1 (<0.1%)
Seasonal affective disorder	1 (<0.1%)	2 (0.1%)	3 (0.1%)
Sleep disorder	3 (0.2%)	1 (<0.1%)	4 (0.1%)
Social phobia	0	1 (<0.1%)	1 (<0.1%)
Stress	1 (<0.1%)	1 (<0.1%)	2 (<0.1%)

Table 14.3.1 / 4: Number of subjects with baseline findings or medical history by MedDRA SOC/PT (FAS)

Primary system organ class<br>Preferred term<br>MedDRA version 14.0	LCS12<br>N=1432 (100%)	LCS16<br>N=1452 (100%)	Total<br>N=2884 (100%)
Renal and urinary disorders	32 (2.2%)	30 (2.1%)	62 (2.1%)
Bladder cyst	1 (<0.1%)	0	1 (<0.1%)
Calculus urinary	1 (<0.1%)	0	1 (<0.1%)
Cystitis interstitial	3 (0.2%)	3 (0.2%)	6 (0.2%)
Dysuria	2 (0.1%)	1 (<0.1%)	3 (0.1%)
Haematuria	2 (0.1%)	0	2 (<0.1%)
Hydronephrosis	1 (<0.1%)	0	1 (<0.1%)
Incontinence	0	1 (<0.1%)	1 (<0.1%)
Micturition urgency	2 (0.1%)	1 (<0.1%)	3 (0.1%)
Nephrolithiasis	9 (0.6%)	14 (1.0%)	23 (0.8%)
Pollakiuria	2 (0.1%)	1 (<0.1%)	3 (0.1%)
Renal colic	1 (<0.1%)	3 (0.2%)	4 (0.1%)
Renal disorder in pregnancy	0	1 (<0.1%)	1 (<0.1%)
Renal failure chronic	1 (<0.1%)	0	1 (<0.1%)
Stress urinary incontinence	7 (0.5%)	4 (0.3%)	11 (0.4%)
Urinary incontinence	2 (0.1%)	0	2 (<0.1%)
Urinary retention	0	1 (<0.1%)	1 (<0.1%)
Urinary tract disorder	1 (<0.1%)	0	1 (<0.1%)
Urinary tract inflammation	0	1 (<0.1%)	1 (<0.1%)

Table 14.3.1 / 4: Number of subjects with baseline findings or medical history by MedDRA SOC/PT (FAS)

Primary system organ class<br>Preferred term<br>MedDRA version 14.0	LCS12<br>N=1432 (100%)	LCS16<br>N=1452 (100%)	Total<br>N=2884 (100%)
Reproductive system and breast disorders	386 (27.0%)	413 (28.4%)	799 (27.7%)
Adenomyosis	0	1 (<0.1%)	1 (<0.1%)
Adnexa uteri mass	1 (<0.1%)	0	1 (<0.1%)
Adnexa uteri pain	1 (<0.1%)	0	1 (<0.1%)
Amenorrhoea	3 (0.2%)	5 (0.3%)	8 (0.3%)
Bartholin's cyst	2 (0.1%)	0	2 (<0.1%)
Bartholinitis	2 (0.1%)	0	2 (<0.1%)
Breast atrophy	1 (<0.1%)	0	1 (<0.1%)
Breast calcifications	0	1 (<0.1%)	1 (<0.1%)
Breast cyst	4 (0.3%)	2 (0.1%)	6 (0.2%)
Breast discomfort	3 (0.2%)	6 (0.4%)	9 (0.3%)
Breast enlargement	1 (<0.1%)	1 (<0.1%)	2 (<0.1%)
Breast mass	7 (0.5%)	3 (0.2%)	10 (0.3%)
Breast pain	3 (0.2%)	3 (0.2%)	6 (0.2%)
Breast tenderness	0	1 (<0.1%)	1 (<0.1%)
Cervical cyst	1 (<0.1%)	1 (<0.1%)	2 (<0.1%)
Cervical dysplasia	61 (4.3%)	65 (4.5%)	126 (4.4%)
Cervical friability	0	1 (<0.1%)	1 (<0.1%)
Cervical polyp	1 (<0.1%)	2 (0.1%)	3 (0.1%)
Cervix haemorrhage uterine	0	1 (<0.1%)	1 (<0.1%)
Cervix inflammation	2 (0.1%)	1 (<0.1%)	3 (0.1%)
Coital bleeding	2 (0.1%)	0	2 (<0.1%)
Dysfunctional uterine bleeding	0	1 (<0.1%)	1 (<0.1%)
Dysmenorrhoea	211 (14.7%)	242 (16.7%)	453 (15.7%)
Dyspareunia	7 (0.5%)	11 (0.8%)	18 (0.6%)
Ectropion of cervix	2 (0.1%)	2 (0.1%)	4 (0.1%)
Endometriosis	15 (1.0%)	17 (1.2%)	32 (1.1%)
Fallopian tube cyst	2 (0.1%)	0	2 (<0.1%)
Fallopian tube obstruction	0	1 (<0.1%)	1 (<0.1%)
Fibrocystic breast disease	11 (0.8%)	10 (0.7%)	21 (0.7%)
Galactorrhoea	3 (0.2%)	3 (0.2%)	6 (0.2%)
Genital discharge	1 (<0.1%)	5 (0.3%)	6 (0.2%)
Haematocolpos	1 (<0.1%)	0	1 (<0.1%)
Haemorrhagic ovarian cyst	1 (<0.1%)	1 (<0.1%)	2 (<0.1%)
Hydrometra	1 (<0.1%)	0	1 (<0.1%)
Infertility	2 (0.1%)	0	2 (<0.1%)
Infertility female	1 (<0.1%)	0	1 (<0.1%)
Infertility male	0	1 (<0.1%)	1 (<0.1%)
Labia enlarged	0	1 (<0.1%)	1 (<0.1%)
Mastoptosis	1 (<0.1%)	0	1 (<0.1%)
Menometrorrhagia	2 (0.1%)	0	2 (<0.1%)
Menorrhagia	17 (1.2%)	17 (1.2%)	34 (1.2%)
Menstruation delayed	1 (<0.1%)	0	1 (<0.1%)
Menstruation irregular	5 (0.3%)	2 (0.1%)	7 (0.2%)

Table 14.3.1 / 4: Number of subjects with baseline findings or medical history by MedDRA SOC/PT (FAS)

Primary system organ class<br>Preferred term<br>MedDRA version 14.0	LCS12<br>N=1432 (100%)	LCS16<br>N=1452 (100%)	Total<br>N=2884 (100%)
Metrorrhagia	3 (0.2%)	6 (0.4%)	9 (0.3%)
Nipple disorder	0	1 (<0.1%)	1 (<0.1%)
Ovarian atrophy	1 (<0.1%)	0	1 (<0.1%)
Ovarian cyst	43 (3.0%)	42 (2.9%)	85 (2.9%)
Ovarian cyst ruptured	2 (0.1%)	1 (<0.1%)	3 (0.1%)
Ovulation pain	1 (<0.1%)	0	1 (<0.1%)
Parovarian cyst	2 (0.1%)	0	2 (<0.1%)
Pelvic adhesions	0	1 (<0.1%)	1 (<0.1%)
Pelvic discomfort	1 (<0.1%)	0	1 (<0.1%)
Pelvic pain	2 (0.1%)	8 (0.6%)	10 (0.3%)
Pelvic prolapse	1 (<0.1%)	0	1 (<0.1%)
Polycystic ovaries	6 (0.4%)	6 (0.4%)	12 (0.4%)
Polymenorrhagia	0	1 (<0.1%)	1 (<0.1%)
Premenstrual syndrome	35 (2.4%)	38 (2.6%)	73 (2.5%)
Rectocele	1 (<0.1%)	0	1 (<0.1%)
Uterine atony	0	1 (<0.1%)	1 (<0.1%)
Uterine cervical erosion	1 (<0.1%)	0	1 (<0.1%)
Uterine cervical pain	1 (<0.1%)	0	1 (<0.1%)
Uterine cervical squamous metaplasia	0	1 (<0.1%)	1 (<0.1%)
Uterine cervix stenosis	0	2 (0.1%)	2 (<0.1%)
Uterine haemorrhage	0	1 (<0.1%)	1 (<0.1%)
Uterine polyp	0	1 (<0.1%)	1 (<0.1%)
Uterine spasm	2 (0.1%)	1 (<0.1%)	3 (0.1%)
Vaginal cyst	1 (<0.1%)	1 (<0.1%)	2 (<0.1%)
Vaginal discharge	1 (<0.1%)	10 (0.7%)	11 (0.4%)
Vaginal disorder	4 (0.3%)	1 (<0.1%)	5 (0.2%)
Vaginal haemorrhage	6 (0.4%)	2 (0.1%)	8 (0.3%)
Vaginal lesion	0	1 (<0.1%)	1 (<0.1%)
Vaginal stricture	0	1 (<0.1%)	1 (<0.1%)
Varicocele	1 (<0.1%)	0	1 (<0.1%)
Varicose veins vaginal	1 (<0.1%)	0	1 (<0.1%)
Vulva cyst	0	1 (<0.1%)	1 (<0.1%)
Vulval disorder	0	1 (<0.1%)	1 (<0.1%)
Vulval leukoplakia	0	1 (<0.1%)	1 (<0.1%)
Vulvovaginal burning sensation	1 (<0.1%)	1 (<0.1%)	2 (<0.1%)
Vulvovaginal discomfort	0	1 (<0.1%)	1 (<0.1%)
Vulvovaginal dryness	1 (<0.1%)	2 (0.1%)	3 (0.1%)
Vulvovaginal erythema	2 (0.1%)	0	2 (<0.1%)
Vulvovaginal pain	3 (0.2%)	1 (<0.1%)	4 (0.1%)
Vulvovaginal pruritus	0	2 (0.1%)	2 (<0.1%)

Table 14.3.1 / 4: Number of subjects with baseline findings or medical history by MedDRA SOC/PT (FAS)

Primary system organ class<br>Preferred term<br>MedDRA version 14.0	LCS12<br>N=1432 (100%)	LCS16<br>N=1452 (100%)	Total<br>N=2884 (100%)
Respiratory, thoracic and mediastinal disorders	125 (8.7%)	151 (10.4%)	276 (9.6%)
Adenoidal disorder	1 (<0.1%)	0	1 (<0.1%)
Allergic bronchitis	1 (<0.1%)	0	1 (<0.1%)
Allergic respiratory disease	0	1 (<0.1%)	1 (<0.1%)
Allergic sinusitis	2 (0.1%)	0	2 (<0.1%)
Asthma	67 (4.7%)	92 (6.3%)	159 (5.5%)
Asthma exercise induced	8 (0.6%)	7 (0.5%)	15 (0.5%)
Asthmatic crisis	1 (<0.1%)	0	1 (<0.1%)
Bronchitis chronic	4 (0.3%)	0	4 (0.1%)
Bronchospasm	2 (0.1%)	0	2 (<0.1%)
Chronic respiratory disease	1 (<0.1%)	0	1 (<0.1%)
Cough	3 (0.2%)	5 (0.3%)	8 (0.3%)
Dysphonia	0	1 (<0.1%)	1 (<0.1%)
Dyspnoea	1 (<0.1%)	0	1 (<0.1%)
Epistaxis	0	2 (0.1%)	2 (<0.1%)
Hyperventilation	0	1 (<0.1%)	1 (<0.1%)
Infantile asthma	1 (<0.1%)	1 (<0.1%)	2 (<0.1%)
Nasal congestion	0	1 (<0.1%)	1 (<0.1%)
Nasal disorder	1 (<0.1%)	0	1 (<0.1%)
Nasal obstruction	0	1 (<0.1%)	1 (<0.1%)
Nasal septum deviation	5 (0.3%)	5 (0.3%)	10 (0.3%)
Oropharyngeal pain	4 (0.3%)	5 (0.3%)	9 (0.3%)
Pneumothorax	1 (<0.1%)	0	1 (<0.1%)
Productive cough	1 (<0.1%)	0	1 (<0.1%)
Rhinitis allergic	12 (0.8%)	16 (1.1%)	28 (1.0%)
Rhinitis perennial	0	1 (<0.1%)	1 (<0.1%)
Rhinitis seasonal	11 (0.8%)	9 (0.6%)	20 (0.7%)
Rhinorrhoea	1 (<0.1%)	0	1 (<0.1%)
Sinus congestion	3 (0.2%)	8 (0.6%)	11 (0.4%)
Sinus disorder	0	1 (<0.1%)	1 (<0.1%)
Throat irritation	0	1 (<0.1%)	1 (<0.1%)
Tonsillar hypertrophy	1 (<0.1%)	0	1 (<0.1%)
Vasomotor rhinitis	1 (<0.1%)	0	1 (<0.1%)
Vocal cord thickening	1 (<0.1%)	0	1 (<0.1%)

Table 14.3.1 / 4: Number of subjects with baseline findings or medical history by MedDRA SOC/PT (FAS)

Primary system organ class<br>Preferred term<br>MedDRA version 14.0	LCS12<br>N=1432 (100%)	LCS16<br>N=1452 (100%)	Total<br>N=2884 (100%)
Skin and subcutaneous tissue disorders	148 (10.3%)	146 (10.1%)	294 (10.2%)
Acanthosis nigricans	0	1 (<0.1%)	1 (<0.1%)
Acne	70 (4.9%)	73 (5.0%)	143 (5.0%)
Acne cystic	1 (<0.1%)	1 (<0.1%)	2 (<0.1%)
Alopecia	1 (<0.1%)	1 (<0.1%)	2 (<0.1%)
Chloasma	0	2 (0.1%)	2 (<0.1%)
Cutaneous lupus erythematosus	0	2 (0.1%)	2 (<0.1%)
Dermal cyst	1 (<0.1%)	1 (<0.1%)	2 (<0.1%)
Dermatitis	2 (0.1%)	2 (0.1%)	4 (0.1%)
Dermatitis allergic	2 (0.1%)	1 (<0.1%)	3 (0.1%)
Dermatitis atopic	15 (1.0%)	11 (0.8%)	26 (0.9%)
Dermatitis contact	2 (0.1%)	6 (0.4%)	8 (0.3%)
Dry skin	3 (0.2%)	3 (0.2%)	6 (0.2%)
Ecchymosis	0	1 (<0.1%)	1 (<0.1%)
Eczema	25 (1.7%)	18 (1.2%)	43 (1.5%)
Erythema nodosum	1 (<0.1%)	0	1 (<0.1%)
Granuloma annulare	1 (<0.1%)	0	1 (<0.1%)
Heat rash	0	1 (<0.1%)	1 (<0.1%)
Hirsutism	5 (0.3%)	2 (0.1%)	7 (0.2%)
Hyperhidrosis	3 (0.2%)	0	3 (0.1%)
Hyperkeratosis	1 (<0.1%)	0	1 (<0.1%)
Hypertrichosis	0	2 (0.1%)	2 (<0.1%)
Increased tendency to bruise	0	2 (0.1%)	2 (<0.1%)
Ingrowing nail	2 (0.1%)	0	2 (<0.1%)
Keloid scar	0	1 (<0.1%)	1 (<0.1%)
Keratosis pilaris	0	1 (<0.1%)	1 (<0.1%)
Lichen sclerosus	0	1 (<0.1%)	1 (<0.1%)
Night sweats	1 (<0.1%)	1 (<0.1%)	2 (<0.1%)
Photosensitivity allergic reaction	1 (<0.1%)	0	1 (<0.1%)
Photosensitivity reaction	1 (<0.1%)	0	1 (<0.1%)
Pityriasis	1 (<0.1%)	0	1 (<0.1%)
Pruritus	0	1 (<0.1%)	1 (<0.1%)
Pruritus generalised	0	1 (<0.1%)	1 (<0.1%)
Psoriasis	10 (0.7%)	11 (0.8%)	21 (0.7%)
Purpura	1 (<0.1%)	0	1 (<0.1%)
Rash	1 (<0.1%)	4 (0.3%)	5 (0.2%)
Rash generalised	1 (<0.1%)	0	1 (<0.1%)
Rosacea	3 (0.2%)	3 (0.2%)	6 (0.2%)
Scar	6 (0.4%)	2 (0.1%)	8 (0.3%)
Seborrhoea	0	1 (<0.1%)	1 (<0.1%)
Skin fissures	0	1 (<0.1%)	1 (<0.1%)
Skin hypertrophy	0	1 (<0.1%)	1 (<0.1%)
Skin irritation	1 (<0.1%)	0	1 (<0.1%)
Skin mass	0	1 (<0.1%)	1 (<0.1%)

Table 14.3.1 / 4: Number of subjects with baseline findings or medical history by MedDRA SOC/PT (FAS)

Primary system organ class<br>Preferred term<br>MedDRA version 14.0	LCS12<br>N=1432 (100%)	LCS16<br>N=1452 (100%)	Total<br>N=2884 (100%)
Skin striae	0	1 (<0.1%)	1 (<0.1%)
Telangiectasia	1 (<0.1%)	0	1 (<0.1%)
Urticaria	4 (0.3%)	4 (0.3%)	8 (0.3%)
Vitiligo	1 (<0.1%)	0	1 (<0.1%)
Social circumstances	17 (1.2%)	21 (1.4%)	38 (1.3%)
Aborted pregnancy	7 (0.5%)	6 (0.4%)	13 (0.5%)
Corrective lens user	7 (0.5%)	9 (0.6%)	16 (0.6%)
Cosmetic body piercing	1 (<0.1%)	0	1 (<0.1%)
Familial risk factor	0	1 (<0.1%)	1 (<0.1%)
Hearing aid user	0	1 (<0.1%)	1 (<0.1%)
Nulliparous	0	1 (<0.1%)	1 (<0.1%)
Organ donor	0	1 (<0.1%)	1 (<0.1%)
Orthosis user	0	1 (<0.1%)	1 (<0.1%)
Tattoo	2 (0.1%)	2 (0.1%)	4 (0.1%)
Tobacco user	1 (<0.1%)	0	1 (<0.1%)

Table 14.3.1 / 4: Number of subjects with baseline findings or medical history by MedDRA SOC/PT (FAS)

Primary system organ class<br>Preferred term<br>MedDRA version 14.0	LCS12<br>N=1432 (100%)	LCS16<br>N=1452 (100%)	Total<br>N=2884 (100%)
Surgical and medical procedures	620 (43.3%)	581 (40.0%)	1201 (41.6%)
Abdominal hernia repair	2 (0.1%)	0	2 (<0.1%)
Abdominoplasty	9 (0.6%)	1 (<0.1%)	10 (0.3%)
Abortion induced	18 (1.3%)	20 (1.4%)	38 (1.3%)
Abscess drainage	1 (<0.1%)	2 (0.1%)	3 (0.1%)
Acrochordon excision	1 (<0.1%)	0	1 (<0.1%)
Adenoidectomy	36 (2.5%)	21 (1.4%)	57 (2.0%)
Adenotonsillectomy	4 (0.3%)	18 (1.2%)	22 (0.8%)
Adhesiolysis	2 (0.1%)	0	2 (<0.1%)
Anal fissure excision	1 (<0.1%)	0	1 (<0.1%)
Anal fistula excision	1 (<0.1%)	0	1 (<0.1%)
Ankle operation	5 (0.3%)	2 (0.1%)	7 (0.2%)
Antibiotic prophylaxis	0	1 (<0.1%)	1 (<0.1%)
Antiviral prophylaxis	0	1 (<0.1%)	1 (<0.1%)
Appendicectomy	84 (5.9%)	61 (4.2%)	145 (5.0%)
Bartholin's cyst removal	1 (<0.1%)	1 (<0.1%)	2 (<0.1%)
Benign tumour excision	0	3 (0.2%)	3 (0.1%)
Bladder irrigation	1 (<0.1%)	0	1 (<0.1%)
Bladder repair	1 (<0.1%)	0	1 (<0.1%)
Bone cyst excision	1 (<0.1%)	0	1 (<0.1%)
Bone graft	1 (<0.1%)	0	1 (<0.1%)
Bone lesion excision	0	2 (0.1%)	2 (<0.1%)
Bone operation	1 (<0.1%)	2 (0.1%)	3 (0.1%)
Breast cyst drainage	0	1 (<0.1%)	1 (<0.1%)
Breast cyst excision	4 (0.3%)	2 (0.1%)	6 (0.2%)
Breast lump removal	3 (0.2%)	1 (<0.1%)	4 (0.1%)
Breast prosthesis implantation	17 (1.2%)	21 (1.4%)	38 (1.3%)
Breast prosthesis removal	1 (<0.1%)	0	1 (<0.1%)
Breast reconstruction	1 (<0.1%)	0	1 (<0.1%)
Bunion operation	6 (0.4%)	2 (0.1%)	8 (0.3%)
Caesarean section	187 (13.1%)	158 (10.9%)	345 (12.0%)
Cardiac ablation	0	1 (<0.1%)	1 (<0.1%)
Cardiac pacemaker insertion	0	1 (<0.1%)	1 (<0.1%)
Carpal tunnel decompression	1 (<0.1%)	0	1 (<0.1%)
Cataract operation	0	1 (<0.1%)	1 (<0.1%)
Cautery to nose	1 (<0.1%)	1 (<0.1%)	2 (<0.1%)
Cervical conisation	3 (0.2%)	3 (0.2%)	6 (0.2%)
Cervical laser therapy	2 (0.1%)	2 (0.1%)	4 (0.1%)
Cervix cautery	4 (0.3%)	6 (0.4%)	10 (0.3%)
Cervix cerclage procedure	2 (0.1%)	1 (<0.1%)	3 (0.1%)
Cervix operation	0	2 (0.1%)	2 (<0.1%)
Cholecystectomy	33 (2.3%)	37 (2.5%)	70 (2.4%)
Cholelithotomy	1 (<0.1%)	0	1 (<0.1%)
Contraception	1 (<0.1%)	0	1 (<0.1%)

Table 14.3.1 / 4: Number of subjects with baseline findings or medical history by MedDRA SOC/PT (FAS)

Primary system organ class<br>Preferred term<br>MedDRA version 14.0	LCS12<br>N=1432 (100%)	LCS16<br>N=1452 (100%)	Total<br>N=2884 (100%)
Cranioplasty	0	1 (<0.1%)	1 (<0.1%)
Cryotherapy	2 (0.1%)	5 (0.3%)	7 (0.2%)
Cyst drainage	1 (<0.1%)	0	1 (<0.1%)
Cyst removal	7 (0.5%)	4 (0.3%)	11 (0.4%)
Dental care	0	1 (<0.1%)	1 (<0.1%)
Dental operation	2 (0.1%)	2 (0.1%)	4 (0.1%)
Ear operation	1 (<0.1%)	1 (<0.1%)	2 (<0.1%)
Ear tube insertion	7 (0.5%)	5 (0.3%)	12 (0.4%)
Ear tube removal	1 (<0.1%)	0	1 (<0.1%)
Elbow operation	1 (<0.1%)	1 (<0.1%)	2 (<0.1%)
Endocervical curettage	1 (<0.1%)	4 (0.3%)	5 (0.2%)
Endodontic procedure	1 (<0.1%)	2 (0.1%)	3 (0.1%)
Endometriosis ablation	1 (<0.1%)	4 (0.3%)	5 (0.2%)
Epiphyseal stapling	1 (<0.1%)	0	1 (<0.1%)
Evacuation of retained products of conception	1 (<0.1%)	1 (<0.1%)	2 (<0.1%)
Exploratory operation	1 (<0.1%)	0	1 (<0.1%)
Eye laser surgery	1 (<0.1%)	1 (<0.1%)	2 (<0.1%)
Eye muscle operation	0	3 (0.2%)	3 (0.1%)
Eye operation	5 (0.3%)	3 (0.2%)	8 (0.3%)
Eyeglasses therapy	0	1 (<0.1%)	1 (<0.1%)
Foot operation	1 (<0.1%)	3 (0.2%)	4 (0.1%)
Gallbladder operation	1 (<0.1%)	2 (0.1%)	3 (0.1%)
Gastric banding	0	1 (<0.1%)	1 (<0.1%)
Gastric bypass	0	5 (0.3%)	5 (0.2%)
Genital labial operation	1 (<0.1%)	0	1 (<0.1%)
Gingival graft	1 (<0.1%)	0	1 (<0.1%)
Gingival operation	0	1 (<0.1%)	1 (<0.1%)
Gingivectomy	1 (<0.1%)	0	1 (<0.1%)
Haemorrhoid operation	2 (0.1%)	1 (<0.1%)	3 (0.1%)
Hernia repair	0	2 (0.1%)	2 (<0.1%)
Hymenectomy	2 (0.1%)	1 (<0.1%)	3 (0.1%)
Ileal operation	0	1 (<0.1%)	1 (<0.1%)
Immunisation	1 (<0.1%)	0	1 (<0.1%)
In vitro fertilisation	2 (0.1%)	3 (0.2%)	5 (0.2%)
Inguinal hernia repair	8 (0.6%)	11 (0.8%)	19 (0.7%)
Intervertebral disc operation	2 (0.1%)	4 (0.3%)	6 (0.2%)
Intra-uterine contraceptive device	1 (<0.1%)	0	1 (<0.1%)
Jaw operation	6 (0.4%)	5 (0.3%)	11 (0.4%)
Joint dislocation reduction	1 (<0.1%)	0	1 (<0.1%)
Joint injection	0	1 (<0.1%)	1 (<0.1%)
Keratomileusis	3 (0.2%)	3 (0.2%)	6 (0.2%)
Knee operation	7 (0.5%)	12 (0.8%)	19 (0.7%)
Laser therapy	1 (<0.1%)	0	1 (<0.1%)
Ligament operation	6 (0.4%)	9 (0.6%)	15 (0.5%)

Table 14.3.1 / 4: Number of subjects with baseline findings or medical history by MedDRA SOC/PT (FAS)

Primary system organ class<br>Preferred term<br>MedDRA version 14.0	LCS12<br>N=1432 (100%)	LCS16<br>N=1452 (100%)	Total<br>N=2884 (100%)
Limb operation	4 (0.3%)	4 (0.3%)	8 (0.3%)
Lipoma excision	2 (0.1%)	0	2 (<0.1%)
Liposuction	8 (0.6%)	4 (0.3%)	12 (0.4%)
Lithotripsy	1 (<0.1%)	0	1 (<0.1%)
Loop electrosurgical excision procedure	19 (1.3%)	17 (1.2%)	36 (1.2%)
Lymphadenectomy	1 (<0.1%)	1 (<0.1%)	2 (<0.1%)
Lymphoid tissue operation	0	1 (<0.1%)	1 (<0.1%)
Mammoplasty	31 (2.2%)	44 (3.0%)	75 (2.6%)
Mastoidectomy	1 (<0.1%)	0	1 (<0.1%)
Maxillofacial operation	1 (<0.1%)	0	1 (<0.1%)
Medical device removal	0	1 (<0.1%)	1 (<0.1%)
Meniscus operation	2 (0.1%)	0	2 (<0.1%)
Meniscus removal	2 (0.1%)	2 (0.1%)	4 (0.1%)
Mole excision	5 (0.3%)	3 (0.2%)	8 (0.3%)
Myomectomy	0	2 (0.1%)	2 (<0.1%)
Myringotomy	3 (0.2%)	2 (0.1%)	5 (0.2%)
Nasal operation	4 (0.3%)	0	4 (0.1%)
Nasal polypectomy	1 (<0.1%)	1 (<0.1%)	2 (<0.1%)
Nasal septal operation	4 (0.3%)	1 (<0.1%)	5 (0.2%)
Obesity surgery	1 (<0.1%)	0	1 (<0.1%)
Oophorectomy	0	1 (<0.1%)	1 (<0.1%)
Oral contraception	0	1 (<0.1%)	1 (<0.1%)
Oral surgery	3 (0.2%)	0	3 (0.1%)
Ostectomy	1 (<0.1%)	0	1 (<0.1%)
Osteosynthesis	2 (0.1%)	2 (0.1%)	4 (0.1%)
Osteotomy	2 (0.1%)	0	2 (<0.1%)
Otoplasty	1 (<0.1%)	0	1 (<0.1%)
Ovarian cystectomy	11 (0.8%)	10 (0.7%)	21 (0.7%)
Ovarian operation	2 (0.1%)	0	2 (<0.1%)
Periodontal operation	0	1 (<0.1%)	1 (<0.1%)
Peripheral nerve operation	0	1 (<0.1%)	1 (<0.1%)
Pilonidal sinus repair	0	3 (0.2%)	3 (0.1%)
Plastic surgery to the face	1 (<0.1%)	0	1 (<0.1%)
Polypectomy	0	1 (<0.1%)	1 (<0.1%)
Proctocolectomy	1 (<0.1%)	0	1 (<0.1%)
Prophylaxis	1 (<0.1%)	0	1 (<0.1%)
Pterygium operation	1 (<0.1%)	0	1 (<0.1%)
Radioactive iodine therapy	1 (<0.1%)	0	1 (<0.1%)
Removal of internal fixation	1 (<0.1%)	0	1 (<0.1%)
Rhinoplasty	6 (0.4%)	6 (0.4%)	12 (0.4%)
Rib excision	1 (<0.1%)	0	1 (<0.1%)
Salivary gland resection	1 (<0.1%)	0	1 (<0.1%)
Salpingectomy	0	1 (<0.1%)	1 (<0.1%)
Salpingo-oophorectomy unilateral	0	1 (<0.1%)	1 (<0.1%)

Table 14.3.1 / 4: Number of subjects with baseline findings or medical history by MedDRA SOC/PT (FAS)

Primary system organ class<br>Preferred term<br>MedDRA version 14.0	LCS12<br>N=1432 (100%)	LCS16<br>N=1452 (100%)	Total<br>N=2884 (100%)
Scar excision	2 (0.1%)	1 (<0.1%)	3 (0.1%)
Scoliosis surgery	1 (<0.1%)	2 (0.1%)	3 (0.1%)
Sebaceous cyst excision	1 (<0.1%)	0	1 (<0.1%)
Shoulder operation	3 (0.2%)	3 (0.2%)	6 (0.2%)
Sinus operation	2 (0.1%)	3 (0.2%)	5 (0.2%)
Skin graft	2 (0.1%)	2 (0.1%)	4 (0.1%)
Skin neoplasm excision	0	1 (<0.1%)	1 (<0.1%)
Skin operation	1 (<0.1%)	0	1 (<0.1%)
Small intestinal resection	0	1 (<0.1%)	1 (<0.1%)
Splenectomy	1 (<0.1%)	0	1 (<0.1%)
Strabismus correction	4 (0.3%)	5 (0.3%)	9 (0.3%)
Surgery	1 (<0.1%)	1 (<0.1%)	2 (<0.1%)
Synovectomy	1 (<0.1%)	0	1 (<0.1%)
Tendon operation	1 (<0.1%)	2 (0.1%)	3 (0.1%)
Tendon sheath incision	1 (<0.1%)	0	1 (<0.1%)
Tendon sheath lesion excision	2 (0.1%)	4 (0.3%)	6 (0.2%)
Therapeutic procedure	0	1 (<0.1%)	1 (<0.1%)
Thymectomy	0	1 (<0.1%)	1 (<0.1%)
Thyroid adenoma removal	1 (<0.1%)	0	1 (<0.1%)
Thyroidectomy	2 (0.1%)	5 (0.3%)	7 (0.2%)
Toe amputation	0	1 (<0.1%)	1 (<0.1%)
Toe operation	2 (0.1%)	1 (<0.1%)	3 (0.1%)
Tongue tie operation	0	1 (<0.1%)	1 (<0.1%)
Tonsillectomy	128 (8.9%)	89 (6.1%)	217 (7.5%)
Tooth extraction	7 (0.5%)	5 (0.3%)	12 (0.4%)
Transfusion	0	1 (<0.1%)	1 (<0.1%)
Tympanoplasty	2 (0.1%)	3 (0.2%)	5 (0.2%)
Umbilical hernia repair	5 (0.3%)	2 (0.1%)	7 (0.2%)
Urethral dilation procedure	0	1 (<0.1%)	1 (<0.1%)
Uterine dilation and curettage	19 (1.3%)	25 (1.7%)	44 (1.5%)
Uterine dilation and evacuation	3 (0.2%)	3 (0.2%)	6 (0.2%)
Uterine operation	0	2 (0.1%)	2 (<0.1%)
Uterine polypectomy	2 (0.1%)	3 (0.2%)	5 (0.2%)
Uvulectomy	1 (<0.1%)	0	1 (<0.1%)
Vaginal cyst excision	1 (<0.1%)	0	1 (<0.1%)
Vaginal operation	0	1 (<0.1%)	1 (<0.1%)
Varicose vein operation	3 (0.2%)	6 (0.4%)	9 (0.3%)
Vulval warts removal	3 (0.2%)	2 (0.1%)	5 (0.2%)
Vulvectomy	0	1 (<0.1%)	1 (<0.1%)
Wart excision	6 (0.4%)	5 (0.3%)	11 (0.4%)
Wisdom teeth removal	55 (3.8%)	52 (3.6%)	107 (3.7%)
Wrist surgery	1 (<0.1%)	1 (<0.1%)	2 (<0.1%)

Table 14.3.1 / 4: Number of subjects with baseline findings or medical history by MedDRA SOC/PT (FAS)

Primary system organ class<br>Preferred term<br>MedDRA version 14.0	LCS12<br>N=1432 (100%)	LCS16<br>N=1452 (100%)	Total<br>N=2884 (100%)
Vascular disorders	26 (1.8%)	29 (2.0%)	55 (1.9%)
Aortic stenosis	0	1 (<0.1%)	1 (<0.1%)
Hot flush	0	2 (0.1%)	2 (<0.1%)
Hypertension	17 (1.2%)	15 (1.0%)	32 (1.1%)
Hypotension	2 (0.1%)	4 (0.3%)	6 (0.2%)
Kawasaki's disease	0	1 (<0.1%)	1 (<0.1%)
Lymphoedema	1 (<0.1%)	0	1 (<0.1%)
Orthostatic hypotension	0	1 (<0.1%)	1 (<0.1%)
Peripheral vascular disorder	0	1 (<0.1%)	1 (<0.1%)
Raynaud's phenomenon	0	1 (<0.1%)	1 (<0.1%)
Varicose vein	6 (0.4%)	3 (0.2%)	9 (0.3%)
Vein pain	0	1 (<0.1%)	1 (<0.1%)

Note: All events recorded as medical history or as baseline finding are shown in this table.
Global Biostatistics: /by-sasp/patdb/projects/de04209/310442/stat/prod_interim03/pgms/t-basfin.sas epkll 12OCT2011 11:19
End of table

Table 14.3.1 / 5: Overall summary of number of subjects with adverse events by treatment in 1st year (FAS)

	LCS12<br>N=1432 (100%)	LCS16<br>N=1452 (100%)	Total<br>N=2884 (100%)
Number of subjects (%) with:			
Any AE	1035 (72.3%)	1068 (73.6%)	2103 (72.9%)
Any study drug related AE	591 (41.3%)	596 (41.0%)	1187 (41.2%)
Any AE related to procedures required by the protocol	180 (12.6%)	186 (12.8%)	366 (12.7%)
Maximum intensity for any AE			
mild	326 (22.8%)	378 (26.0%)	704 (24.4%)
moderate	521 (36.4%)	524 (36.1%)	1045 (36.2%)
severe	187 (13.1%)	161 (11.1%)	348 (12.1%)
Maximum intensity for study drug related AEs			
mild	228 (15.9%)	254 (17.5%)	482 (16.7%)
moderate	275 (19.2%)	258 (17.8%)	533 (18.5%)
severe	85 (5.9%)	84 (5.8%)	169 (5.9%)
AE-related Deaths	0	0	0
Any SAEs	35 (2.4%)	27 (1.9%)	62 (2.1%)
Study drug related SAEs	6 (0.4%)	7 (0.5%)	13 (0.5%)
SAEs related to procedures required by the protocol	2 (0.1%)	4 (0.3%)	6 (0.2%)
Discontinuation of study drug due to AEs	233 (16.3%)	207 (14.3%)	440 (15.3%)
Discontinuation of study drug due to SAEs	9 (0.6%)	7 (0.5%)	16 (0.6%)

Note: Missing, possible, probable and definite for drug relationship is counted as drug-related.
Note: Denominator is number of subjects available at the START of the year or all subjects for overall
Global Biostatistics: /by-sasp/patdb/projects/de04209/310442/stat/prod_interim03/pgms/t-ae-cat-summary.sas epkll 12OCT2011 11:20
End of table

Table 14.3.1 / 6: Overall summary of number of subjects with adverse events by treatment in 2nd year (FAS)

	LCS12 N=1166 (100%)	LCS16 N=1206 (100%)	Total N=2372 (100%)
Number of subjects (%) with:			
Any AE	603 (51.7%)	670 (55.6%)	1273 (53.7%)
Any study drug related AE	166 (14.2%)	200 (16.6%)	366 (15.4%)
Any AE related to procedures required by the protocol	27 (2.3%)	37 (3.1%)	64 (2.7%)
Maximum intensity for any AE			
mild	257 (22.0%)	286 (23.7%)	543 (22.9%)
moderate	284 (24.4%)	300 (24.9%)	584 (24.6%)
severe	61 (5.2%)	81 (6.7%)	142 (6.0%)
Maximum intensity for study drug related AEs			
mild	82 (7.0%)	105 (8.7%)	187 (7.9%)
moderate	64 (5.5%)	73 (6.1%)	137 (5.8%)
severe	20 (1.7%)	21 (1.7%)	41 (1.7%)
AE-related Deaths	0	0	0
Any SAEs	19 (1.6%)	22 (1.8%)	41 (1.7%)
Study drug related SAEs	2 (0.2%)	5 (0.4%)	7 (0.3%)
SAEs related to procedures required by the protocol	0	2 (0.2%)	2 (<0.1%)
Discontinuation of study drug due to AEs	56 (4.8%)	56 (4.6%)	112 (4.7%)
Discontinuation of study drug due to SAEs	2 (0.2%)	5 (0.4%)	7 (0.3%)

Note: Missing, possible, probable and definite for drug relationship is counted as drug-related.
Note: Denominator is number of subjects available at the START of the year or all subjects for overall
Global Biostatistics: /by-sasp/patdb/projects/de04209/310442/stat/prod_interim03/pgms/t-ae-cat-summary.sas epkll 12OCT2011 11:20
End of table

Table 14.3.1 / 7: Overall summary of number of subjects with adverse events by treatment in 3rd year (FAS)

	LCS12 N=963 (100%)	LCS16 N=1012 (100%)	Total N=1975 (100%)
Number of subjects (%) with:			
Any AE	466 (48.4%)	535 (52.9%)	1001 (50.7%)
Any study drug related AE	104 (10.8%)	141 (13.9%)	245 (12.4%)
Any AE related to procedures required by the protocol	49 (5.1%)	50 (4.9%)	99 (5.0%)
Maximum intensity for any AE			
mild	211 (21.9%)	228 (22.5%)	439 (22.2%)
moderate	195 (20.2%)	239 (23.6%)	434 (22.0%)
severe	50 (5.2%)	53 (5.2%)	103 (5.2%)
Maximum intensity for study drug related AEs			
mild	54 (5.6%)	80 (7.9%)	134 (6.8%)
moderate	37 (3.8%)	49 (4.8%)	86 (4.4%)
severe	11 (1.1%)	11 (1.1%)	22 (1.1%)
AE-related Deaths	0	0	0
Any SAEs	18 (1.9%)	23 (2.3%)	41 (2.1%)
Study drug related SAEs	1 (0.1%)	3 (0.3%)	4 (0.2%)
SAEs related to procedures required by the protocol	0	0	0
Discontinuation of study drug due to AEs	32 (3.3%)	33 (3.3%)	65 (3.3%)
Discontinuation of study drug due to SAEs	3 (0.3%)	6 (0.6%)	9 (0.5%)

Note: Missing, possible, probable and definite for drug relationship is counted as drug-related.
Note: Denominator is number of subjects available at the START of the year or all subjects for overall
Global Biostatistics: /by-sasp/patdb/projects/de04209/310442/stat/prod_interim03/pgms/t-ae-cat-summary.sas epkll 12OCT2011 11:20
End of table

Table 14.3.1 / 8: Overall summary of number of subjects with adverse events by treatment overall (FAS)

	LCS12 N=1432 (100%)	LCS16 N=1452 (100%)	Total N=2884 (100%)
Number of subjects (%) with:			
Any AE	1194 (83.4%)	1246 (85.8%)	2440 (84.6%)
Any study drug related AE	710 (49.6%)	756 (52.1%)	1466 (50.8%)
Any AE related to procedures required by the protocol	241 (16.8%)	260 (17.9%)	501 (17.4%)
Maximum intensity for any AE			
mild	313 (21.9%)	353 (24.3%)	666 (23.1%)
moderate	616 (43.0%)	633 (43.6%)	1249 (43.3%)
severe	261 (18.2%)	250 (17.2%)	511 (17.7%)
Maximum intensity for study drug related AEs			
mild	261 (18.2%)	315 (21.7%)	576 (20.0%)
moderate	333 (23.3%)	334 (23.0%)	667 (23.1%)
severe	112 (7.8%)	106 (7.3%)	218 (7.6%)
AE-related Deaths	0	0	0
Any SAEs	66 (4.6%)	71 (4.9%)	137 (4.8%)
Study drug related SAEs	8 (0.6%)	15 (1.0%)	23 (0.8%)
SAEs related to procedures required by the protocol	2 (0.1%)	6 (0.4%)	8 (0.3%)
Discontinuation of study drug due to AEs	319 (22.3%)	290 (20.0%)	609 (21.1%)
Discontinuation of study drug due to SAEs	14 (1.0%)	18 (1.2%)	32 (1.1%)

Note: Missing, possible, probable and definite for drug relationship is counted as drug-related.
Note: Denominator is number of subjects available at the START of the year or all subjects for overall
Global Biostatistics: /by-sasp/patdb/projects/de04209/310442/stat/prod_interim03/pgms/t-ae-cat-summary.sas epkll 12OCT2011 11:20
End of table

Table 14.3.1 / 9: Number of subjects with adverse events by primary system organ class, preferred term and year (FAS)

Primary system organ class<br>Preferred term<br>MedDRA version 14.0	YEAR OF ONSET	LCS12	LCS16	Total
Number of subjects (%) with at least one adverse event	1st year	1035/1432 (72.3%)	1068/1452 (73.6%)	2103/2884 (72.9%)
	2nd year	603/1166 (51.7%)	670/1206 (55.6%)	1273/2372 (53.7%)
	3rd year	466/ 963 (48.4%)	535/1012 (52.9%)	1001/1975 (50.7%)
	Overall	1194/1432 (83.4%)	1246/1452 (85.8%)	2440/2884 (84.6%)
Blood and lymphatic system disorders	1st year	10/1432 (0.7%)	3/1452 (0.2%)	13/2884 (0.5%)
	2nd year	3/1166 (0.3%)	2/1206 (0.2%)	5/2372 (0.2%)
	3rd year	3/ 963 (0.3%)	3/1012 (0.3%)	6/1975 (0.3%)
	Overall	16/1432 (1.1%)	8/1452 (0.6%)	24/2884 (0.8%)
Anaemia	1st year	5/1432 (0.3%)	1/1452 (<0.1%)	6/2884 (0.2%)
	2nd year	1/1166 (<0.1%)	1/1206 (<0.1%)	2/2372 (<0.1%)
	3rd year	1/ 963 (0.1%)	1/1012 (<0.1%)	2/1975 (0.1%)
	Overall	7/1432 (0.5%)	3/1452 (0.2%)	10/2884 (0.3%)
Iron deficiency anaemia	1st year	0/1432	1/1452 (<0.1%)	1/2884 (<0.1%)
	3rd year	1/ 963 (0.1%)	0/1012	1/1975 (<0.1%)
	Overall	1/1432 (<0.1%)	1/1452 (<0.1%)	2/2884 (<0.1%)
Leukocytosis	1st year	1/1432 (<0.1%)	0/1452	1/2884 (<0.1%)
	Overall	1/1432 (<0.1%)	0/1452	1/2884 (<0.1%)
Lymphadenitis	1st year	2/1432 (0.1%)	0/1452	2/2884 (<0.1%)
	2nd year	1/1166 (<0.1%)	0/1206	1/2372 (<0.1%)
	Overall	3/1432 (0.2%)	0/1452	3/2884 (0.1%)
Lymphadenopathy	1st year	1/1432 (<0.1%)	1/1452 (<0.1%)	2/2884 (<0.1%)
	2nd year	1/1166 (<0.1%)	0/1206	1/2372 (<0.1%)
	3rd year	1/ 963 (0.1%)	2/1012 (0.2%)	3/1975 (0.2%)
	Overall	3/1432 (0.2%)	3/1452 (0.2%)	6/2884 (0.2%)
Pancytopenia	2nd year	0/1166	1/1206 (<0.1%)	1/2372 (<0.1%)
	Overall	0/1432	1/1452 (<0.1%)	1/2884 (<0.1%)
Spherocytic anaemia	1st year	1/1432 (<0.1%)	0/1452	1/2884 (<0.1%)
	Overall	1/1432 (<0.1%)	0/1452	1/2884 (<0.1%)
Splenic cyst	1st year	1/1432 (<0.1%)	0/1452	1/2884 (<0.1%)
	Overall	1/1432 (<0.1%)	0/1452	1/2884 (<0.1%)

Table 14.3.1 / 9: Number of subjects with adverse events by primary system organ class, preferred term and year (FAS)

Primary system organ class<br>Preferred term<br>MedDRA version 14.0	YEAR OF ONSET	LCS12	LCS16	Total
Splenomegaly	1st year	1/1432 (<0.1%)	0/1452	1/2884 (<0.1%)
	Overall	1/1432 (<0.1%)	0/1452	1/2884 (<0.1%)
Cardiac disorders	1st year	2/1432 (0.1%)	8/1452 (0.6%)	10/2884 (0.3%)
	2nd year	4/1166 (0.3%)	4/1206 (0.3%)	8/2372 (0.3%)
	3rd year	3/ 963 (0.3%)	3/1012 (0.3%)	6/1975 (0.3%)
	Overall	9/1432 (0.6%)	14/1452 (1.0%)	23/2884 (0.8%)
Arrhythmia	1st year	1/1432 (<0.1%)	2/1452 (0.1%)	3/2884 (0.1%)
	Overall	1/1432 (<0.1%)	2/1452 (0.1%)	3/2884 (0.1%)
Extrasystoles	1st year	0/1432	1/1452 (<0.1%)	1/2884 (<0.1%)
	3rd year	0/ 963	1/1012 (<0.1%)	1/1975 (<0.1%)
	Overall	0/1432	2/1452 (0.1%)	2/2884 (<0.1%)
Mitral valve prolapse	1st year	0/1432	1/1452 (<0.1%)	1/2884 (<0.1%)
	2nd year	0/1166	1/1206 (<0.1%)	1/2372 (<0.1%)
	Overall	0/1432	2/1452 (0.1%)	2/2884 (<0.1%)
Palpitations	1st year	0/1432	2/1452 (0.1%)	2/2884 (<0.1%)
	2nd year	1/1166 (<0.1%)	3/1206 (0.2%)	4/2372 (0.2%)
	3rd year	3/ 963 (0.3%)	1/1012 (<0.1%)	4/1975 (0.2%)
	Overall	4/1432 (0.3%)	6/1452 (0.4%)	10/2884 (0.3%)
Sinus tachycardia	1st year	0/1432	1/1452 (<0.1%)	1/2884 (<0.1%)
	Overall	0/1432	1/1452 (<0.1%)	1/2884 (<0.1%)
Tachycardia	1st year	1/1432 (<0.1%)	2/1452 (0.1%)	3/2884 (0.1%)
	2nd year	3/1166 (0.3%)	0/1206	3/2372 (0.1%)
	Overall	4/1432 (0.3%)	2/1452 (0.1%)	6/2884 (0.2%)
Ventricular extrasystoles	3rd year	0/ 963	1/1012 (<0.1%)	1/1975 (<0.1%)
	Overall	0/1432	1/1452 (<0.1%)	1/2884 (<0.1%)
Congenital, familial and genetic disorders	1st year	0/1432	1/1452 (<0.1%)	1/2884 (<0.1%)
	2nd year	3/1166 (0.3%)	0/1206	3/2372 (0.1%)
	Overall	3/1432 (0.2%)	1/1452 (<0.1%)	4/2884 (0.1%)
Congenital hydronephrosis	2nd year	1/1166 (<0.1%)	0/1206	1/2372 (<0.1%)
	Overall	1/1432 (<0.1%)	0/1452	1/2884 (<0.1%)

Table 14.3.1 / 9: Number of subjects with adverse events by primary system organ class, preferred term and year (FAS)

Primary system organ class<br>Preferred term<br>MedDRA version 14.0	YEAR OF ONSET	LCS12	LCS16	Total
Dermoid cyst	2nd year	1/1166 (<0.1%)	0/1206	1/2372 (<0.1%)
	Overall	1/1432 (<0.1%)	0/1452	1/2884 (<0.1%)
Myotonia congenita	1st year	0/1432	1/1452 (<0.1%)	1/2884 (<0.1%)
	Overall	0/1432	1/1452 (<0.1%)	1/2884 (<0.1%)
Urethral intrinsic sphincter deficiency	2nd year	1/1166 (<0.1%)	0/1206	1/2372 (<0.1%)
	Overall	1/1432 (<0.1%)	0/1452	1/2884 (<0.1%)
Ear and labyrinth disorders	1st year	9/1432 (0.6%)	5/1452 (0.3%)	14/2884 (0.5%)
	2nd year	5/1166 (0.4%)	3/1206 (0.2%)	8/2372 (0.3%)
	3rd year	3/ 963 (0.3%)	7/1012 (0.7%)	10/1975 (0.5%)
	Overall	16/1432 (1.1%)	14/1452 (1.0%)	30/2884 (1.0%)
Deafness bilateral	2nd year	1/1166 (<0.1%)	0/1206	1/2372 (<0.1%)
	Overall	1/1432 (<0.1%)	0/1452	1/2884 (<0.1%)
Ear pain	1st year	0/1432	1/1452 (<0.1%)	1/2884 (<0.1%)
	3rd year	0/ 963	2/1012 (0.2%)	2/1975 (0.1%)
	Overall	0/1432	3/1452 (0.2%)	3/2884 (0.1%)
Ear pruritus	1st year	1/1432 (<0.1%)	0/1452	1/2884 (<0.1%)
	Overall	1/1432 (<0.1%)	0/1452	1/2884 (<0.1%)
Meniere's disease	3rd year	0/ 963	1/1012 (<0.1%)	1/1975 (<0.1%)
	Overall	0/1432	1/1452 (<0.1%)	1/2884 (<0.1%)
Middle ear effusion	1st year	0/1432	1/1452 (<0.1%)	1/2884 (<0.1%)
	2nd year	0/1166	1/1206 (<0.1%)	1/2372 (<0.1%)
	Overall	0/1432	2/1452 (0.1%)	2/2884 (<0.1%)
Motion sickness	1st year	3/1432 (0.2%)	1/1452 (<0.1%)	4/2884 (0.1%)
	3rd year	1/ 963 (0.1%)	2/1012 (0.2%)	3/1975 (0.2%)
	Overall	4/1432 (0.3%)	3/1452 (0.2%)	7/2884 (0.2%)
Otorrhoea	2nd year	0/1166	1/1206 (<0.1%)	1/2372 (<0.1%)
	3rd year	1/ 963 (0.1%)	0/1012	1/1975 (<0.1%)
	Overall	1/1432 (<0.1%)	1/1452 (<0.1%)	2/2884 (<0.1%)

Table 14.3.1 / 9: Number of subjects with adverse events by primary system organ class, preferred term and year (FAS)

Primary system organ class<br>Preferred term<br>MedDRA version 14.0	YEAR OF ONSET	LCS12	LCS16	Total
Sudden hearing loss	3rd year	1/ 963 (0.1%)	0/1012	1/1975 (<0.1%)
	Overall	1/1432 (<0.1%)	0/1452	1/2884 (<0.1%)
Tinnitus	2nd year	2/1166 (0.2%)	0/1206	2/2372 (<0.1%)
	Overall	2/1432 (0.1%)	0/1452	2/2884 (<0.1%)
Tympanic membrane perforation	1st year	1/1432 (<0.1%)	0/1452	1/2884 (<0.1%)
	2nd year	1/1166 (<0.1%)	0/1206	1/2372 (<0.1%)
	Overall	2/1432 (0.1%)	0/1452	2/2884 (<0.1%)
Vertigo	1st year	3/1432 (0.2%)	2/1452 (0.1%)	5/2884 (0.2%)
	2nd year	1/1166 (<0.1%)	1/1206 (<0.1%)	2/2372 (<0.1%)
	3rd year	0/ 963	3/1012 (0.3%)	3/1975 (0.2%)
	Overall	4/1432 (0.3%)	6/1452 (0.4%)	10/2884 (0.3%)
Vertigo labyrinthine	1st year	1/1432 (<0.1%)	0/1452	1/2884 (<0.1%)
	Overall	1/1432 (<0.1%)	0/1452	1/2884 (<0.1%)
Vertigo positional	1st year	1/1432 (<0.1%)	0/1452	1/2884 (<0.1%)
	2nd year	1/1166 (<0.1%)	0/1206	1/2372 (<0.1%)
	Overall	2/1432 (0.1%)	0/1452	2/2884 (<0.1%)
Endocrine disorders	1st year	3/1432 (0.2%)	14/1452 (1.0%)	17/2884 (0.6%)
	2nd year	5/1166 (0.4%)	1/1206 (<0.1%)	6/2372 (0.3%)
	3rd year	2/ 963 (0.2%)	6/1012 (0.6%)	8/1975 (0.4%)
	Overall	10/1432 (0.7%)	21/1452 (1.4%)	31/2884 (1.1%)
Goitre	1st year	0/1432	3/1452 (0.2%)	3/2884 (0.1%)
	2nd year	1/1166 (<0.1%)	1/1206 (<0.1%)	2/2372 (<0.1%)
	3rd year	1/ 963 (0.1%)	1/1012 (<0.1%)	2/1975 (0.1%)
	Overall	2/1432 (0.1%)	5/1452 (0.3%)	7/2884 (0.2%)
Hyperthyroidism	1st year	0/1432	1/1452 (<0.1%)	1/2884 (<0.1%)
	3rd year	0/ 963	1/1012 (<0.1%)	1/1975 (<0.1%)
	Overall	0/1432	2/1452 (0.1%)	2/2884 (<0.1%)
Hypothyroidism	1st year	2/1432 (0.1%)	9/1452 (0.6%)	11/2884 (0.4%)
	2nd year	4/1166 (0.3%)	0/1206	4/2372 (0.2%)
	3rd year	1/ 963 (0.1%)	4/1012 (0.4%)	5/1975 (0.3%)
	Overall	7/1432 (0.5%)	13/1452 (0.9%)	20/2884 (0.7%)

Table 14.3.1 / 9: Number of subjects with adverse events by primary system organ class, preferred term and year (FAS)

Primary system organ class<br>Preferred term<br>MedDRA version 14.0	YEAR OF<br>ONSET	LCS12	LCS16	Total
Thyroid mass	1st year	0/1432	1/1452 (<0.1%)	1/2884 (<0.1%)
	Overall	0/1432	1/1452 (<0.1%)	1/2884 (<0.1%)
Thyroiditis	1st year	1/1432 (<0.1%)	0/1452	1/2884 (<0.1%)
	Overall	1/1432 (<0.1%)	0/1452	1/2884 (<0.1%)
Eye disorders	1st year	14/1432 (1.0%)	11/1452 (0.8%)	25/2884 (0.9%)
	2nd year	4/1166 (0.3%)	5/1206 (0.4%)	9/2372 (0.4%)
	3rd year	7/ 963 (0.7%)	7/1012 (0.7%)	14/1975 (0.7%)
	Overall	25/1432 (1.7%)	23/1452 (1.6%)	48/2884 (1.7%)
Blepharitis	1st year	0/1432	2/1452 (0.1%)	2/2884 (<0.1%)
	2nd year	1/1166 (<0.1%)	0/1206	1/2372 (<0.1%)
	Overall	1/1432 (<0.1%)	2/1452 (0.1%)	3/2884 (0.1%)
Chalazion	1st year	0/1432	1/1452 (<0.1%)	1/2884 (<0.1%)
	Overall	0/1432	1/1452 (<0.1%)	1/2884 (<0.1%)
Conjunctivitis	1st year	11/1432 (0.8%)	6/1452 (0.4%)	17/2884 (0.6%)
	2nd year	2/1166 (0.2%)	4/1206 (0.3%)	6/2372 (0.3%)
	3rd year	2/ 963 (0.2%)	5/1012 (0.5%)	7/1975 (0.4%)
	Overall	15/1432 (1.0%)	15/1452 (1.0%)	30/2884 (1.0%)
Conjunctivitis allergic	3rd year	2/ 963 (0.2%)	2/1012 (0.2%)	4/1975 (0.2%)
	Overall	2/1432 (0.1%)	2/1452 (0.1%)	4/2884 (0.1%)
Dry eye	1st year	2/1432 (0.1%)	0/1452	2/2884 (<0.1%)
	Overall	2/1432 (0.1%)	0/1452	2/2884 (<0.1%)
Eye inflammation	2nd year	0/1166	1/1206 (<0.1%)	1/2372 (<0.1%)
	Overall	0/1432	1/1452 (<0.1%)	1/2884 (<0.1%)
Eyelid cyst	1st year	0/1432	1/1452 (<0.1%)	1/2884 (<0.1%)
	Overall	0/1432	1/1452 (<0.1%)	1/2884 (<0.1%)
Heterophoria	2nd year	1/1166 (<0.1%)	0/1206	1/2372 (<0.1%)
	Overall	1/1432 (<0.1%)	0/1452	1/2884 (<0.1%)

Table 14.3.1 / 9: Number of subjects with adverse events by primary system organ class, preferred term and year (FAS)

Primary system organ class<br>Preferred term<br>MedDRA version 14.0	YEAR OF<br>ONSET	LCS12	LCS16	Total
Iridocyclitis	1st year	1/1432 (<0.1%)	0/1452	1/2884 (<0.1%)
	Overall	1/1432 (<0.1%)	0/1452	1/2884 (<0.1%)
Iritis	1st year	0/1432	1/1452 (<0.1%)	1/2884 (<0.1%)
	2nd year	0/1166	1/1206 (<0.1%)	1/2372 (<0.1%)
	Overall	0/1432	2/1452 (0.1%)	2/2884 (<0.1%)
Meibomianitis	1st year	1/1432 (<0.1%)	0/1452	1/2884 (<0.1%)
	Overall	1/1432 (<0.1%)	0/1452	1/2884 (<0.1%)
Myopia	1st year	0/1432	1/1452 (<0.1%)	1/2884 (<0.1%)
	Overall	0/1432	1/1452 (<0.1%)	1/2884 (<0.1%)
Ocular icterus	1st year	0/1432	1/1452 (<0.1%)	1/2884 (<0.1%)
	Overall	0/1432	1/1452 (<0.1%)	1/2884 (<0.1%)
Panophthalmitis	3rd year	1/ 963 (0.1%)	0/1012	1/1975 (<0.1%)
	Overall	1/1432 (<0.1%)	0/1452	1/2884 (<0.1%)
Scotoma	3rd year	1/ 963 (0.1%)	0/1012	1/1975 (<0.1%)
	Overall	1/1432 (<0.1%)	0/1452	1/2884 (<0.1%)
Vision blurred	3rd year	1/ 963 (0.1%)	0/1012	1/1975 (<0.1%)
	Overall	1/1432 (<0.1%)	0/1452	1/2884 (<0.1%)
Gastrointestinal disorders	1st year	236/1432 (16.5%)	218/1452 (15.0%)	454/2884 (15.7%)
	2nd year	81/1166 (6.9%)	93/1206 (7.7%)	174/2372 (7.3%)
	3rd year	54/ 963 (5.6%)	63/1012 (6.2%)	117/1975 (5.9%)
	Overall	335/1432 (23.4%)	320/1452 (22.0%)	655/2884 (22.7%)
Abdominal adhesions	2nd year	1/1166 (<0.1%)	0/1206	1/2372 (<0.1%)
	Overall	1/1432 (<0.1%)	0/1452	1/2884 (<0.1%)
Abdominal discomfort	1st year	5/1432 (0.3%)	3/1452 (0.2%)	8/2884 (0.3%)
	2nd year	1/1166 (<0.1%)	3/1206 (0.2%)	4/2372 (0.2%)
	Overall	5/1432 (0.3%)	6/1452 (0.4%)	11/2884 (0.4%)

Table 14.3.1 / 9: Number of subjects with adverse events by primary system organ class, preferred term and year (FAS)

Primary system organ class<br>Preferred term<br>MedDRA version 14.0	YEAR OF ONSET	LCS12	LCS16	Total
Abdominal distension	1st year	17/1432 (1.2%)	10/1452 (0.7%)	27/2884 (0.9%)
	2nd year	3/1166 (0.3%)	2/1206 (0.2%)	5/2372 (0.2%)
	3rd year	1/ 963 (0.1%)	0/1012	1/1975 (<0.1%)
	Overall	21/1432 (1.5%)	11/1452 (0.8%)	32/2884 (1.1%)
Abdominal hernia	1st year	1/1432 (<0.1%)	0/1452	1/2884 (<0.1%)
	3rd year	1/ 963 (0.1%)	0/1012	1/1975 (<0.1%)
	Overall	2/1432 (0.1%)	0/1452	2/2884 (<0.1%)
Abdominal mass	1st year	1/1432 (<0.1%)	0/1452	1/2884 (<0.1%)
	Overall	1/1432 (<0.1%)	0/1452	1/2884 (<0.1%)
Abdominal pain	1st year	75/1432 (5.2%)	60/1452 (4.1%)	135/2884 (4.7%)
	2nd year	20/1166 (1.7%)	29/1206 (2.4%)	49/2372 (2.1%)
	3rd year	11/ 963 (1.1%)	21/1012 (2.1%)	32/1975 (1.6%)
	Overall	100/1432 (7.0%)	101/1452 (7.0%)	201/2884 (7.0%)
Abdominal pain lower	1st year	47/1432 (3.3%)	43/1452 (3.0%)	90/2884 (3.1%)
	2nd year	14/1166 (1.2%)	16/1206 (1.3%)	30/2372 (1.3%)
	3rd year	8/ 963 (0.8%)	9/1012 (0.9%)	17/1975 (0.9%)
	Overall	67/1432 (4.7%)	61/1452 (4.2%)	128/2884 (4.4%)
Abdominal pain upper	1st year	5/1432 (0.3%)	7/1452 (0.5%)	12/2884 (0.4%)
	2nd year	1/1166 (<0.1%)	2/1206 (0.2%)	3/2372 (0.1%)
	3rd year	1/ 963 (0.1%)	2/1012 (0.2%)	3/1975 (0.2%)
	Overall	7/1432 (0.5%)	11/1452 (0.8%)	18/2884 (0.6%)
Abdominal tenderness	1st year	2/1432 (0.1%)	0/1452	2/2884 (<0.1%)
	Overall	2/1432 (0.1%)	0/1452	2/2884 (<0.1%)
Anal fissure	1st year	0/1432	1/1452 (<0.1%)	1/2884 (<0.1%)
	2nd year	1/1166 (<0.1%)	1/1206 (<0.1%)	2/2372 (<0.1%)
	Overall	1/1432 (<0.1%)	2/1452 (0.1%)	3/2884 (0.1%)
Anal pruritus	1st year	1/1432 (<0.1%)	0/1452	1/2884 (<0.1%)
	2nd year	1/1166 (<0.1%)	1/1206 (<0.1%)	2/2372 (<0.1%)
	3rd year	1/ 963 (0.1%)	1/1012 (<0.1%)	2/1975 (0.1%)
	Overall	3/1432 (0.2%)	2/1452 (0.1%)	5/2884 (0.2%)

Table 14.3.1 / 9: Number of subjects with adverse events by primary system organ class, preferred term and year (FAS)

Primary system organ class<br>Preferred term<br>MedDRA version 14.0	YEAR OF ONSET	LCS12	LCS16	Total
Anal skin tags	2nd year	0/1166	1/1206 (<0.1%)	1/2372 (<0.1%)
	Overall	0/1432	1/1452 (<0.1%)	1/2884 (<0.1%)
Aphthous stomatitis	1st year	0/1432	1/1452 (<0.1%)	1/2884 (<0.1%)
	Overall	0/1432	1/1452 (<0.1%)	1/2884 (<0.1%)
Breath odour	2nd year	1/1166 (<0.1%)	0/1206	1/2372 (<0.1%)
	Overall	1/1432 (<0.1%)	0/1452	1/2884 (<0.1%)
Coeliac disease	2nd year	1/1166 (<0.1%)	0/1206	1/2372 (<0.1%)
	Overall	1/1432 (<0.1%)	0/1452	1/2884 (<0.1%)
Colitis	1st year	0/1432	2/1452 (0.1%)	2/2884 (<0.1%)
	2nd year	1/1166 (<0.1%)	1/1206 (<0.1%)	2/2372 (<0.1%)
	Overall	1/1432 (<0.1%)	3/1452 (0.2%)	4/2884 (0.1%)
Colitis ulcerative	1st year	1/1432 (<0.1%)	0/1452	1/2884 (<0.1%)
	2nd year	1/1166 (<0.1%)	0/1206	1/2372 (<0.1%)
	Overall	2/1432 (0.1%)	0/1452	2/2884 (<0.1%)
Colonic polyp	2nd year	1/1166 (<0.1%)	0/1206	1/2372 (<0.1%)
	Overall	1/1432 (<0.1%)	0/1452	1/2884 (<0.1%)
Constipation	1st year	11/1432 (0.8%)	9/1452 (0.6%)	20/2884 (0.7%)
	2nd year	3/1166 (0.3%)	6/1206 (0.5%)	9/2372 (0.4%)
	3rd year	3/ 963 (0.3%)	3/1012 (0.3%)	6/1975 (0.3%)
	Overall	16/1432 (1.1%)	18/1452 (1.2%)	34/2884 (1.2%)
Crohn's disease	1st year	2/1432 (0.1%)	0/1452	2/2884 (<0.1%)
	Overall	2/1432 (0.1%)	0/1452	2/2884 (<0.1%)
Dental caries	1st year	1/1432 (<0.1%)	1/1452 (<0.1%)	2/2884 (<0.1%)
	3rd year	0/ 963	2/1012 (0.2%)	2/1975 (0.1%)
	Overall	1/1432 (<0.1%)	3/1452 (0.2%)	4/2884 (0.1%)
Diarrhoea	1st year	13/1432 (0.9%)	16/1452 (1.1%)	29/2884 (1.0%)
	2nd year	2/1166 (0.2%)	7/1206 (0.6%)	9/2372 (0.4%)
	3rd year	4/ 963 (0.4%)	5/1012 (0.5%)	9/1975 (0.5%)
	Overall	19/1432 (1.3%)	27/1452 (1.9%)	46/2884 (1.6%)

Table 14.3.1 / 9: Number of subjects with adverse events by primary system organ class, preferred term and year (FAS)

Primary system organ class<br>Preferred term<br>MedDRA version 14.0	YEAR OF ONSET	LCS12	LCS16	Total
Dry mouth	3rd year	0/ 963	1/1012 (<0.1%)	1/1975 (<0.1%)
	Overall	0/1432	1/1452 (<0.1%)	1/2884 (<0.1%)
Duodenal ulcer	3rd year	1/ 963 (0.1%)	0/1012	1/1975 (<0.1%)
	Overall	1/1432 (<0.1%)	0/1452	1/2884 (<0.1%)
Dyspepsia	1st year	6/1432 (0.4%)	7/1452 (0.5%)	13/2884 (0.5%)
	2nd year	3/1166 (0.3%)	3/1206 (0.2%)	6/2372 (0.3%)
	3rd year	5/ 963 (0.5%)	2/1012 (0.2%)	7/1975 (0.4%)
	Overall	14/1432 (1.0%)	12/1452 (0.8%)	26/2884 (0.9%)
Dysphagia	1st year	1/1432 (<0.1%)	0/1452	1/2884 (<0.1%)
	2nd year	0/1166	1/1206 (<0.1%)	1/2372 (<0.1%)
	Overall	1/1432 (<0.1%)	1/1452 (<0.1%)	2/2884 (<0.1%)
Flatulence	1st year	2/1432 (0.1%)	2/1452 (0.1%)	4/2884 (0.1%)
	Overall	2/1432 (0.1%)	2/1452 (0.1%)	4/2884 (0.1%)
Food poisoning	1st year	1/1432 (<0.1%)	3/1452 (0.2%)	4/2884 (0.1%)
	2nd year	0/1166	4/1206 (0.3%)	4/2372 (0.2%)
	Overall	1/1432 (<0.1%)	6/1452 (0.4%)	7/2884 (0.2%)
Frequent bowel movements	1st year	2/1432 (0.1%)	0/1452	2/2884 (<0.1%)
	Overall	2/1432 (0.1%)	0/1452	2/2884 (<0.1%)
Gastric ulcer	1st year	1/1432 (<0.1%)	1/1452 (<0.1%)	2/2884 (<0.1%)
	2nd year	1/1166 (<0.1%)	0/1206	1/2372 (<0.1%)
	Overall	2/1432 (0.1%)	1/1452 (<0.1%)	3/2884 (0.1%)
Gastritis	1st year	6/1432 (0.4%)	5/1452 (0.3%)	11/2884 (0.4%)
	2nd year	2/1166 (0.2%)	8/1206 (0.7%)	10/2372 (0.4%)
	3rd year	5/ 963 (0.5%)	3/1012 (0.3%)	8/1975 (0.4%)
	Overall	13/1432 (0.9%)	16/1452 (1.1%)	29/2884 (1.0%)
Gastrointestinal disorder	2nd year	1/1166 (<0.1%)	0/1206	1/2372 (<0.1%)
	Overall	1/1432 (<0.1%)	0/1452	1/2884 (<0.1%)
Gastrointestinal pain	1st year	1/1432 (<0.1%)	1/1452 (<0.1%)	2/2884 (<0.1%)
	Overall	1/1432 (<0.1%)	1/1452 (<0.1%)	2/2884 (<0.1%)

Table 14.3.1 / 9: Number of subjects with adverse events by primary system organ class, preferred term and year (FAS)

Primary system organ class<br>Preferred term<br>MedDRA version 14.0	YEAR OF ONSET	LCS12	LCS16	Total
Gastrooesophageal reflux disease	1st year	6/1432 (0.4%)	6/1452 (0.4%)	12/2884 (0.4%)
	2nd year	2/1166 (0.2%)	3/1206 (0.2%)	5/2372 (0.2%)
	3rd year	1/ 963 (0.1%)	2/1012 (0.2%)	3/1975 (0.2%)
	Overall	9/1432 (0.6%)	11/1452 (0.8%)	20/2884 (0.7%)
Gingival oedema	3rd year	1/ 963 (0.1%)	0/1012	1/1975 (<0.1%)
	Overall	1/1432 (<0.1%)	0/1452	1/2884 (<0.1%)
Gingival recession	1st year	1/1432 (<0.1%)	0/1452	1/2884 (<0.1%)
	Overall	1/1432 (<0.1%)	0/1452	1/2884 (<0.1%)
Gingivitis	1st year	1/1432 (<0.1%)	0/1452	1/2884 (<0.1%)
	2nd year	0/1166	1/1206 (<0.1%)	1/2372 (<0.1%)
	Overall	1/1432 (<0.1%)	1/1452 (<0.1%)	2/2884 (<0.1%)
Haematochezia	2nd year	1/1166 (<0.1%)	1/1206 (<0.1%)	2/2372 (<0.1%)
	Overall	1/1432 (<0.1%)	1/1452 (<0.1%)	2/2884 (<0.1%)
Haemorrhoids	1st year	3/1432 (0.2%)	5/1452 (0.3%)	8/2884 (0.3%)
	2nd year	2/1166 (0.2%)	3/1206 (0.2%)	5/2372 (0.2%)
	3rd year	2/ 963 (0.2%)	0/1012	2/1975 (0.1%)
	Overall	7/1432 (0.5%)	8/1452 (0.6%)	15/2884 (0.5%)
Hiatus hernia	2nd year	0/1166	1/1206 (<0.1%)	1/2372 (<0.1%)
	Overall	0/1432	1/1452 (<0.1%)	1/2884 (<0.1%)
Hyperchlorhydria	1st year	1/1432 (<0.1%)	1/1452 (<0.1%)	2/2884 (<0.1%)
	2nd year	1/1166 (<0.1%)	0/1206	1/2372 (<0.1%)
	Overall	2/1432 (0.1%)	1/1452 (<0.1%)	3/2884 (0.1%)
Impaired gastric emptying	1st year	1/1432 (<0.1%)	0/1452	1/2884 (<0.1%)
	Overall	1/1432 (<0.1%)	0/1452	1/2884 (<0.1%)
Inflammatory bowel disease	3rd year	0/ 963	1/1012 (<0.1%)	1/1975 (<0.1%)
	Overall	0/1432	1/1452 (<0.1%)	1/2884 (<0.1%)
Inguinal hernia	2nd year	1/1166 (<0.1%)	0/1206	1/2372 (<0.1%)
	Overall	1/1432 (<0.1%)	0/1452	1/2884 (<0.1%)

Table 14.3.1 / 9: Number of subjects with adverse events by primary system organ class, preferred term and year (FAS)

Primary system organ class<br>Preferred term<br>MedDRA version 14.0	YEAR OF ONSET	LCS12	LCS16	Total
Irritable bowel syndrome	1st year	5/1432 (0.3%)	2/1452 (0.1%)	7/2884 (0.2%)
	2nd year	4/1166 (0.3%)	1/1206 (<0.1%)	5/2372 (0.2%)
	3rd year	2/ 963 (0.2%)	3/1012 (0.3%)	5/1975 (0.3%)
	Overall	11/1432 (0.8%)	5/1452 (0.3%)	16/2884 (0.6%)
Mouth ulceration	1st year	0/1432	2/1452 (0.1%)	2/2884 (<0.1%)
	Overall	0/1432	2/1452 (0.1%)	2/2884 (<0.1%)
Nausea	1st year	49/1432 (3.4%)	41/1452 (2.8%)	90/2884 (3.1%)
	2nd year	18/1166 (1.5%)	8/1206 (0.7%)	26/2372 (1.1%)
	3rd year	9/ 963 (0.9%)	12/1012 (1.2%)	21/1975 (1.1%)
	Overall	74/1432 (5.2%)	58/1452 (4.0%)	132/2884 (4.6%)
Odynophagia	2nd year	0/1166	1/1206 (<0.1%)	1/2372 (<0.1%)
	Overall	0/1432	1/1452 (<0.1%)	1/2884 (<0.1%)
Oesophageal stenosis	2nd year	0/1166	1/1206 (<0.1%)	1/2372 (<0.1%)
	Overall	0/1432	1/1452 (<0.1%)	1/2884 (<0.1%)
Oesophagitis	3rd year	1/ 963 (0.1%)	0/1012	1/1975 (<0.1%)
	Overall	1/1432 (<0.1%)	0/1452	1/2884 (<0.1%)
Oral pain	1st year	0/1432	1/1452 (<0.1%)	1/2884 (<0.1%)
	Overall	0/1432	1/1452 (<0.1%)	1/2884 (<0.1%)
Peptic ulcer	2nd year	1/1166 (<0.1%)	0/1206	1/2372 (<0.1%)
	Overall	1/1432 (<0.1%)	0/1452	1/2884 (<0.1%)
Periodontitis	1st year	1/1432 (<0.1%)	0/1452	1/2884 (<0.1%)
	3rd year	1/ 963 (0.1%)	1/1012 (<0.1%)	2/1975 (0.1%)
	Overall	2/1432 (0.1%)	1/1452 (<0.1%)	3/2884 (0.1%)
Peritonitis	2nd year	1/1166 (<0.1%)	0/1206	1/2372 (<0.1%)
	Overall	1/1432 (<0.1%)	0/1452	1/2884 (<0.1%)
Proctalgia	2nd year	2/1166 (0.2%)	1/1206 (<0.1%)	3/2372 (0.1%)
	Overall	2/1432 (0.1%)	1/1452 (<0.1%)	3/2884 (0.1%)

Table 14.3.1 / 9: Number of subjects with adverse events by primary system organ class, preferred term and year (FAS)

Primary system organ class<br>Preferred term<br>MedDRA version 14.0	YEAR OF ONSET	LCS12	LCS16	Total
Rectal haemorrhage	1st year	0/1432	2/1452 (0.1%)	2/2884 (<0.1%)
	2nd year	0/1166	1/1206 (<0.1%)	1/2372 (<0.1%)
	Overall	0/1432	3/1452 (0.2%)	3/2884 (0.1%)
Reflux oesophagitis	1st year	0/1432	1/1452 (<0.1%)	1/2884 (<0.1%)
	2nd year	0/1166	1/1206 (<0.1%)	1/2372 (<0.1%)
	Overall	0/1432	1/1452 (<0.1%)	1/2884 (<0.1%)
Salivary gland calculus	1st year	0/1432	1/1452 (<0.1%)	1/2884 (<0.1%)
	Overall	0/1432	1/1452 (<0.1%)	1/2884 (<0.1%)
Tooth disorder	1st year	3/1432 (0.2%)	0/1452	3/2884 (0.1%)
	2nd year	0/1166	1/1206 (<0.1%)	1/2372 (<0.1%)
	Overall	3/1432 (0.2%)	1/1452 (<0.1%)	4/2884 (0.1%)
Tooth impacted	1st year	1/1432 (<0.1%)	0/1452	1/2884 (<0.1%)
	2nd year	1/1166 (<0.1%)	0/1206	1/2372 (<0.1%)
	Overall	2/1432 (0.1%)	0/1452	2/2884 (<0.1%)
Toothache	1st year	12/1432 (0.8%)	15/1452 (1.0%)	27/2884 (0.9%)
	2nd year	2/1166 (0.2%)	9/1206 (0.7%)	11/2372 (0.5%)
	3rd year	4/ 963 (0.4%)	5/1012 (0.5%)	9/1975 (0.5%)
	Overall	17/1432 (1.2%)	26/1452 (1.8%)	43/2884 (1.5%)
Umbilical hernia	3rd year	0/ 963	1/1012 (<0.1%)	1/1975 (<0.1%)
	Overall	0/1432	1/1452 (<0.1%)	1/2884 (<0.1%)
Vomiting	1st year	12/1432 (0.8%)	13/1452 (0.9%)	25/2884 (0.9%)
	2nd year	4/1166 (0.3%)	6/1206 (0.5%)	10/2372 (0.4%)
	3rd year	1/ 963 (0.1%)	5/1012 (0.5%)	6/1975 (0.3%)
	Overall	17/1432 (1.2%)	24/1452 (1.7%)	41/2884 (1.4%)
General disorders and administration site conditions	1st year	63/1432 (4.4%)	72/1452 (5.0%)	135/2884 (4.7%)
	2nd year	34/1166 (2.9%)	30/1206 (2.5%)	64/2372 (2.7%)
	3rd year	22/ 963 (2.3%)	20/1012 (2.0%)	42/1975 (2.1%)
	Overall	113/1432 (7.9%)	119/1452 (8.2%)	232/2884 (8.0%)
Asthenia	1st year	1/1432 (<0.1%)	1/1452 (<0.1%)	2/2884 (<0.1%)
	2nd year	1/1166 (<0.1%)	0/1206	1/2372 (<0.1%)
	Overall	2/1432 (0.1%)	1/1452 (<0.1%)	3/2884 (0.1%)

Table 14.3.1 / 9: Number of subjects with adverse events by primary system organ class, preferred term and year (FAS)

Primary system organ class<br>Preferred term<br>MedDRA version 14.0	YEAR OF ONSET	LCS12	LCS16	Total
Axillary pain	2nd year	0/1166	1/1206 (<0.1%)	1/2372 (<0.1%)
	3rd year	1/ 963 (0.1%)	0/1012	1/1975 (<0.1%)
	Overall	1/1432 (<0.1%)	1/1452 (<0.1%)	2/2884 (<0.1%)
Chest pain	1st year	2/1432 (0.1%)	2/1452 (0.1%)	4/2884 (0.1%)
	2nd year	3/1166 (0.3%)	1/1206 (<0.1%)	4/2372 (0.2%)
	3rd year	1/ 963 (0.1%)	4/1012 (0.4%)	5/1975 (0.3%)
	Overall	5/1432 (0.3%)	7/1452 (0.5%)	12/2884 (0.4%)
Chills	1st year	0/1432	2/1452 (0.1%)	2/2884 (<0.1%)
	Overall	0/1432	2/1452 (0.1%)	2/2884 (<0.1%)
Cyst	1st year	1/1432 (<0.1%)	0/1452	1/2884 (<0.1%)
	2nd year	2/1166 (0.2%)	0/1206	2/2372 (<0.1%)
	3rd year	0/ 963	1/1012 (<0.1%)	1/1975 (<0.1%)
	Overall	3/1432 (0.2%)	1/1452 (<0.1%)	4/2884 (0.1%)
Device dislocation	1st year	2/1432 (0.1%)	3/1452 (0.2%)	5/2884 (0.2%)
	2nd year	0/1166	1/1206 (<0.1%)	1/2372 (<0.1%)
	3rd year	1/ 963 (0.1%)	1/1012 (<0.1%)	2/1975 (0.1%)
	Overall	3/1432 (0.2%)	5/1452 (0.3%)	8/2884 (0.3%)
Device expulsion	1st year	25/1432 (1.7%)	23/1452 (1.6%)	48/2884 (1.7%)
	2nd year	9/1166 (0.8%)	7/1206 (0.6%)	16/2372 (0.7%)
	3rd year	12/ 963 (1.2%)	7/1012 (0.7%)	19/1975 (1.0%)
	Overall	46/1432 (3.2%)	37/1452 (2.5%)	83/2884 (2.9%)
Discomfort	1st year	1/1432 (<0.1%)	0/1452	1/2884 (<0.1%)
	2nd year	1/1166 (<0.1%)	1/1206 (<0.1%)	2/2372 (<0.1%)
	Overall	2/1432 (0.1%)	1/1452 (<0.1%)	3/2884 (0.1%)
Drug withdrawal syndrome	3rd year	1/ 963 (0.1%)	0/1012	1/1975 (<0.1%)
	Overall	1/1432 (<0.1%)	0/1452	1/2884 (<0.1%)
Fatigue	1st year	12/1432 (0.8%)	19/1452 (1.3%)	31/2884 (1.1%)
	2nd year	8/1166 (0.7%)	6/1206 (0.5%)	14/2372 (0.6%)
	3rd year	5/ 963 (0.5%)	2/1012 (0.2%)	7/1975 (0.4%)
	Overall	24/1432 (1.7%)	27/1452 (1.9%)	51/2884 (1.8%)

Table 14.3.1 / 9: Number of subjects with adverse events by primary system organ class, preferred term and year (FAS)

Primary system organ class<br>Preferred term<br>MedDRA version 14.0	YEAR OF ONSET	LCS12	LCS16	Total
Feeling cold	1st year	0/1432	1/1452 (<0.1%)	1/2884 (<0.1%)
	2nd year	0/1166	1/1206 (<0.1%)	1/2372 (<0.1%)
	Overall	0/1432	2/1452 (0.1%)	2/2884 (<0.1%)
Feeling hot	1st year	0/1432	1/1452 (<0.1%)	1/2884 (<0.1%)
	Overall	0/1432	1/1452 (<0.1%)	1/2884 (<0.1%)
Generalised oedema	3rd year	1/ 963 (0.1%)	0/1012	1/1975 (<0.1%)
	Overall	1/1432 (<0.1%)	0/1452	1/2884 (<0.1%)
Hangover	1st year	1/1432 (<0.1%)	0/1452	1/2884 (<0.1%)
	Overall	1/1432 (<0.1%)	0/1452	1/2884 (<0.1%)
Hunger	2nd year	0/1166	1/1206 (<0.1%)	1/2372 (<0.1%)
	Overall	0/1432	1/1452 (<0.1%)	1/2884 (<0.1%)
Inflammation	1st year	1/1432 (<0.1%)	0/1452	1/2884 (<0.1%)
	3rd year	0/ 963	1/1012 (<0.1%)	1/1975 (<0.1%)
	Overall	1/1432 (<0.1%)	1/1452 (<0.1%)	2/2884 (<0.1%)
Influenza like illness	2nd year	1/1166 (<0.1%)	1/1206 (<0.1%)	2/2372 (<0.1%)
	Overall	1/1432 (<0.1%)	1/1452 (<0.1%)	2/2884 (<0.1%)
Injury associated with device	1st year	0/1432	1/1452 (<0.1%)	1/2884 (<0.1%)
	Overall	0/1432	1/1452 (<0.1%)	1/2884 (<0.1%)
Irritability	1st year	2/1432 (0.1%)	4/1452 (0.3%)	6/2884 (0.2%)
	2nd year	0/1166	1/1206 (<0.1%)	1/2372 (<0.1%)
	3rd year	0/ 963	1/1012 (<0.1%)	1/1975 (<0.1%)
	Overall	2/1432 (0.1%)	6/1452 (0.4%)	8/2884 (0.3%)
Localised oedema	1st year	0/1432	1/1452 (<0.1%)	1/2884 (<0.1%)
	2nd year	0/1166	1/1206 (<0.1%)	1/2372 (<0.1%)
	Overall	0/1432	1/1452 (<0.1%)	1/2884 (<0.1%)
Malaise	1st year	0/1432	1/1452 (<0.1%)	1/2884 (<0.1%)
	Overall	0/1432	1/1452 (<0.1%)	1/2884 (<0.1%)

Table 14.3.1 / 9: Number of subjects with adverse events by primary system organ class, preferred term and year (FAS)

Primary system organ class<br>Preferred term<br>MedDRA version 14.0	YEAR OF ONSET	LCS12	LCS16	Total
Oedema	1st year	0/1432	1/1452 (<0.1%)	1/2884 (<0.1%)
	Overall	0/1432	1/1452 (<0.1%)	1/2884 (<0.1%)
Oedema peripheral	1st year	3/1432 (0.2%)	4/1452 (0.3%)	7/2884 (0.2%)
	2nd year	1/1166 (<0.1%)	1/1206 (<0.1%)	2/2372 (<0.1%)
	3rd year	0/ 963	1/1012 (<0.1%)	1/1975 (<0.1%)
	Overall	4/1432 (0.3%)	6/1452 (0.4%)	10/2884 (0.3%)
Pain	1st year	5/1432 (0.3%)	3/1452 (0.2%)	8/2884 (0.3%)
	2nd year	1/1166 (<0.1%)	1/1206 (<0.1%)	2/2372 (<0.1%)
	3rd year	0/ 963	1/1012 (<0.1%)	1/1975 (<0.1%)
	Overall	6/1432 (0.4%)	5/1452 (0.3%)	11/2884 (0.4%)
Pyrexia	1st year	10/1432 (0.7%)	10/1452 (0.7%)	20/2884 (0.7%)
	2nd year	7/1166 (0.6%)	5/1206 (0.4%)	12/2372 (0.5%)
	3rd year	1/ 963 (0.1%)	2/1012 (0.2%)	3/1975 (0.2%)
	Overall	18/1432 (1.3%)	16/1452 (1.1%)	34/2884 (1.2%)
Vaccination site pain	2nd year	1/1166 (<0.1%)	1/1206 (<0.1%)	2/2372 (<0.1%)
	Overall	1/1432 (<0.1%)	1/1452 (<0.1%)	2/2884 (<0.1%)
Hepatobiliary disorders	1st year	5/1432 (0.3%)	6/1452 (0.4%)	11/2884 (0.4%)
	2nd year	2/1166 (0.2%)	3/1206 (0.2%)	5/2372 (0.2%)
	3rd year	2/ 963 (0.2%)	2/1012 (0.2%)	4/1975 (0.2%)
	Overall	8/1432 (0.6%)	11/1452 (0.8%)	19/2884 (0.7%)
Biliary colic	1st year	0/1432	2/1452 (0.1%)	2/2884 (<0.1%)
	Overall	0/1432	2/1452 (0.1%)	2/2884 (<0.1%)
Biliary dyskinesia	2nd year	0/1166	1/1206 (<0.1%)	1/2372 (<0.1%)
	3rd year	1/ 963 (0.1%)	0/1012	1/1975 (<0.1%)
	Overall	1/1432 (<0.1%)	1/1452 (<0.1%)	2/2884 (<0.1%)
Cholecystitis	1st year	1/1432 (<0.1%)	0/1452	1/2884 (<0.1%)
	2nd year	0/1166	1/1206 (<0.1%)	1/2372 (<0.1%)
	3rd year	2/ 963 (0.2%)	1/1012 (<0.1%)	3/1975 (0.2%)
	Overall	3/1432 (0.2%)	2/1452 (0.1%)	5/2884 (0.2%)

Table 14.3.1 / 9: Number of subjects with adverse events by primary system organ class, preferred term and year (FAS)

Primary system organ class<br>Preferred term<br>MedDRA version 14.0	YEAR OF ONSET	LCS12	LCS16	Total
Cholecystitis chronic	1st year	1/1432 (<0.1%)	0/1452	1/2884 (<0.1%)
	2nd year	1/1166 (<0.1%)	0/1206	1/2372 (<0.1%)
	Overall	2/1432 (0.1%)	0/1452	2/2884 (<0.1%)
Cholelithiasis	1st year	3/1432 (0.2%)	1/1452 (<0.1%)	4/2884 (0.1%)
	2nd year	1/1166 (<0.1%)	1/1206 (<0.1%)	2/2372 (<0.1%)
	3rd year	0/ 963	2/1012 (0.2%)	2/1975 (0.1%)
	Overall	4/1432 (0.3%)	4/1452 (0.3%)	8/2884 (0.3%)
Gallbladder pain	1st year	0/1432	1/1452 (<0.1%)	1/2884 (<0.1%)
	Overall	0/1432	1/1452 (<0.1%)	1/2884 (<0.1%)
Gallbladder polyp	1st year	1/1432 (<0.1%)	0/1452	1/2884 (<0.1%)
	Overall	1/1432 (<0.1%)	0/1452	1/2884 (<0.1%)
Hepatic steatosis	1st year	0/1432	1/1452 (<0.1%)	1/2884 (<0.1%)
	Overall	0/1432	1/1452 (<0.1%)	1/2884 (<0.1%)
Hyperbilirubinaemia	1st year	0/1432	1/1452 (<0.1%)	1/2884 (<0.1%)
	Overall	0/1432	1/1452 (<0.1%)	1/2884 (<0.1%)
Immune system disorders	1st year	39/1432 (2.7%)	37/1452 (2.5%)	76/2884 (2.6%)
	2nd year	25/1166 (2.1%)	22/1206 (1.8%)	47/2372 (2.0%)
	3rd year	18/ 963 (1.9%)	21/1012 (2.1%)	39/1975 (2.0%)
	Overall	63/1432 (4.4%)	66/1452 (4.5%)	129/2884 (4.5%)
Allergy to animal	1st year	0/1432	2/1452 (0.1%)	2/2884 (<0.1%)
	3rd year	1/ 963 (0.1%)	0/1012	1/1975 (<0.1%)
	Overall	1/1432 (<0.1%)	2/1452 (0.1%)	3/2884 (0.1%)
Allergy to arthropod bite	1st year	1/1432 (<0.1%)	0/1452	1/2884 (<0.1%)
	3rd year	0/ 963	1/1012 (<0.1%)	1/1975 (<0.1%)
	Overall	1/1432 (<0.1%)	1/1452 (<0.1%)	2/2884 (<0.1%)
Allergy to arthropod sting	3rd year	0/ 963	1/1012 (<0.1%)	1/1975 (<0.1%)
	Overall	0/1432	1/1452 (<0.1%)	1/2884 (<0.1%)
Allergy to chemicals	1st year	1/1432 (<0.1%)	0/1452	1/2884 (<0.1%)
	Overall	1/1432 (<0.1%)	0/1452	1/2884 (<0.1%)

Table 14.3.1 / 9: Number of subjects with adverse events by primary system organ class, preferred term and year (FAS)

Primary system organ class<br>Preferred term<br>MedDRA version 14.0	YEAR OF ONSET	LCS12	LCS16	Total
Anaphylactic reaction	3rd year	0/ 963	1/1012 (<0.1%)	1/1975 (<0.1%)
	Overall	0/1432	1/1452 (<0.1%)	1/2884 (<0.1%)
Drug hypersensitivity	1st year	5/1432 (0.3%)	4/1452 (0.3%)	9/2884 (0.3%)
	2nd year	2/1166 (0.2%)	0/1206	2/2372 (<0.1%)
	3rd year	2/ 963 (0.2%)	2/1012 (0.2%)	4/1975 (0.2%)
	Overall	9/1432 (0.6%)	6/1452 (0.4%)	15/2884 (0.5%)
Food allergy	1st year	1/1432 (<0.1%)	1/1452 (<0.1%)	2/2884 (<0.1%)
	Overall	1/1432 (<0.1%)	1/1452 (<0.1%)	2/2884 (<0.1%)
Hypersensitivity	1st year	8/1432 (0.6%)	6/1452 (0.4%)	14/2884 (0.5%)
	2nd year	3/1166 (0.3%)	5/1206 (0.4%)	8/2372 (0.3%)
	3rd year	3/ 963 (0.3%)	1/1012 (<0.1%)	4/1975 (0.2%)
	Overall	13/1432 (0.9%)	11/1452 (0.8%)	24/2884 (0.8%)
Seasonal allergy	1st year	24/1432 (1.7%)	24/1452 (1.7%)	48/2884 (1.7%)
	2nd year	20/1166 (1.7%)	17/1206 (1.4%)	37/2372 (1.6%)
	3rd year	12/ 963 (1.2%)	16/1012 (1.6%)	28/1975 (1.4%)
	Overall	39/1432 (2.7%)	44/1452 (3.0%)	83/2884 (2.9%)
Infections and infestations	1st year	534/1432 (37.3%)	529/1452 (36.4%)	1063/2884 (36.9%)
	2nd year	320/1166 (27.4%)	335/1206 (27.8%)	655/2372 (27.6%)
	3rd year	228/ 963 (23.7%)	259/1012 (25.6%)	487/1975 (24.7%)
	Overall	722/1432 (50.4%)	736/1452 (50.7%)	1458/2884 (50.6%)
Abscess limb	1st year	0/1432	1/1452 (<0.1%)	1/2884 (<0.1%)
	Overall	0/1432	1/1452 (<0.1%)	1/2884 (<0.1%)
Acarodermatitis	2nd year	1/1166 (<0.1%)	0/1206	1/2372 (<0.1%)
	3rd year	1/ 963 (0.1%)	0/1012	1/1975 (<0.1%)
	Overall	2/1432 (0.1%)	0/1452	2/2884 (<0.1%)
Acute sinusitis	1st year	1/1432 (<0.1%)	0/1452	1/2884 (<0.1%)
	2nd year	3/1166 (0.3%)	2/1206 (0.2%)	5/2372 (0.2%)
	3rd year	4/ 963 (0.4%)	1/1012 (<0.1%)	5/1975 (0.3%)
	Overall	7/1432 (0.5%)	3/1452 (0.2%)	10/2884 (0.3%)

Table 14.3.1 / 9: Number of subjects with adverse events by primary system organ class, preferred term and year (FAS)

Primary system organ class<br>Preferred term<br>MedDRA version 14.0	YEAR OF ONSET	LCS12	LCS16	Total
Acute tonsillitis	1st year	8/1432 (0.6%)	1/1452 (<0.1%)	9/2884 (0.3%)
	2nd year	1/1166 (<0.1%)	2/1206 (0.2%)	3/2372 (0.1%)
	3rd year	1/ 963 (0.1%)	2/1012 (0.2%)	3/1975 (0.2%)
	Overall	10/1432 (0.7%)	5/1452 (0.3%)	15/2884 (0.5%)
Anogenital warts	1st year	8/1432 (0.6%)	4/1452 (0.3%)	12/2884 (0.4%)
	2nd year	3/1166 (0.3%)	3/1206 (0.2%)	6/2372 (0.3%)
	3rd year	0/ 963	3/1012 (0.3%)	3/1975 (0.2%)
	Overall	11/1432 (0.8%)	10/1452 (0.7%)	21/2884 (0.7%)
Appendicitis	1st year	4/1432 (0.3%)	2/1452 (0.1%)	6/2884 (0.2%)
	2nd year	2/1166 (0.2%)	3/1206 (0.2%)	5/2372 (0.2%)
	3rd year	0/ 963	2/1012 (0.2%)	2/1975 (0.1%)
	Overall	6/1432 (0.4%)	7/1452 (0.5%)	13/2884 (0.5%)
Arthritis rubella	2nd year	1/1166 (<0.1%)	0/1206	1/2372 (<0.1%)
	Overall	1/1432 (<0.1%)	0/1452	1/2884 (<0.1%)
Bartholin's abscess	1st year	2/1432 (0.1%)	0/1452	2/2884 (<0.1%)
	3rd year	1/ 963 (0.1%)	0/1012	1/1975 (<0.1%)
	Overall	3/1432 (0.2%)	0/1452	3/2884 (0.1%)
Blastocystis infection	3rd year	0/ 963	1/1012 (<0.1%)	1/1975 (<0.1%)
	Overall	0/1432	1/1452 (<0.1%)	1/2884 (<0.1%)
Body tinea	1st year	1/1432 (<0.1%)	2/1452 (0.1%)	3/2884 (0.1%)
	3rd year	1/ 963 (0.1%)	0/1012	1/1975 (<0.1%)
	Overall	2/1432 (0.1%)	2/1452 (0.1%)	4/2884 (0.1%)
Breast abscess	1st year	1/1432 (<0.1%)	0/1452	1/2884 (<0.1%)
	Overall	1/1432 (<0.1%)	0/1452	1/2884 (<0.1%)
Bronchitis	1st year	32/1432 (2.2%)	21/1452 (1.4%)	53/2884 (1.8%)
	2nd year	17/1166 (1.5%)	15/1206 (1.2%)	32/2372 (1.3%)
	3rd year	15/ 963 (1.6%)	5/1012 (0.5%)	20/1975 (1.0%)
	Overall	58/1432 (4.1%)	38/1452 (2.6%)	96/2884 (3.3%)
Bronchitis viral	2nd year	1/1166 (<0.1%)	0/1206	1/2372 (<0.1%)
	Overall	1/1432 (<0.1%)	0/1452	1/2884 (<0.1%)

Table 14.3.1 / 9: Number of subjects with adverse events by primary system organ class, preferred term and year (FAS)

Primary system organ class<br>Preferred term<br>MedDRA version 14.0	YEAR OF<br>ONSET	LCS12	LCS16	Total
Bronchopneumonia	1st year	1/1432 (<0.1%)	0/1452	1/2884 (<0.1%)
	Overall	1/1432 (<0.1%)	0/1452	1/2884 (<0.1%)
Bursitis infective	1st year	1/1432 (<0.1%)	0/1452	1/2884 (<0.1%)
	Overall	1/1432 (<0.1%)	0/1452	1/2884 (<0.1%)
Campylobacter infection	1st year	1/1432 (<0.1%)	0/1452	1/2884 (<0.1%)
	Overall	1/1432 (<0.1%)	0/1452	1/2884 (<0.1%)
Campylobacter intestinal infection	1st year	0/1432	1/1452 (<0.1%)	1/2884 (<0.1%)
	Overall	0/1432	1/1452 (<0.1%)	1/2884 (<0.1%)
Candidiasis	1st year	10/1432 (0.7%)	5/1452 (0.3%)	15/2884 (0.5%)
	2nd year	2/1166 (0.2%)	2/1206 (0.2%)	4/2372 (0.2%)
	3rd year	1/ 963 (0.1%)	2/1012 (0.2%)	3/1975 (0.2%)
	Overall	11/1432 (0.8%)	9/1452 (0.6%)	20/2884 (0.7%)
Carbuncle	1st year	0/1432	1/1452 (<0.1%)	1/2884 (<0.1%)
	Overall	0/1432	1/1452 (<0.1%)	1/2884 (<0.1%)
Cellulitis	1st year	1/1432 (<0.1%)	3/1452 (0.2%)	4/2884 (0.1%)
	2nd year	1/1166 (<0.1%)	1/1206 (<0.1%)	2/2372 (<0.1%)
	3rd year	1/ 963 (0.1%)	1/1012 (<0.1%)	2/1975 (0.1%)
	Overall	3/1432 (0.2%)	4/1452 (0.3%)	7/2884 (0.2%)
Cervicitis	1st year	2/1432 (0.1%)	11/1452 (0.8%)	13/2884 (0.5%)
	2nd year	2/1166 (0.2%)	2/1206 (0.2%)	4/2372 (0.2%)
	3rd year	1/ 963 (0.1%)	3/1012 (0.3%)	4/1975 (0.2%)
	Overall	5/1432 (0.3%)	15/1452 (1.0%)	20/2884 (0.7%)
Cervicitis gonococcal	2nd year	1/1166 (<0.1%)	0/1206	1/2372 (<0.1%)
	Overall	1/1432 (<0.1%)	0/1452	1/2884 (<0.1%)
Chlamydial cervicitis	1st year	1/1432 (<0.1%)	1/1452 (<0.1%)	2/2884 (<0.1%)
	2nd year	1/1166 (<0.1%)	3/1206 (0.2%)	4/2372 (0.2%)
	3rd year	0/ 963	2/1012 (0.2%)	2/1975 (0.1%)
	Overall	2/1432 (0.1%)	6/1452 (0.4%)	8/2884 (0.3%)

Table 14.3.1 / 9: Number of subjects with adverse events by primary system organ class, preferred term and year (FAS)

Primary system organ class<br>Preferred term<br>MedDRA version 14.0	YEAR OF ONSET	LCS12	LCS16	Total
Chlamydial infection	1st year	0/1432	2/1452 (0.1%)	2/2884 (<0.1%)
	2nd year	1/1166 (<0.1%)	1/1206 (<0.1%)	2/2372 (<0.1%)
	3rd year	0/ 963	1/1012 (<0.1%)	1/1975 (<0.1%)
	Overall	1/1432 (<0.1%)	4/1452 (0.3%)	5/2884 (0.2%)
Chronic tonsillitis	3rd year	0/ 963	1/1012 (<0.1%)	1/1975 (<0.1%)
	Overall	0/1432	1/1452 (<0.1%)	1/2884 (<0.1%)
Clitoris abscess	3rd year	1/ 963 (0.1%)	0/1012	1/1975 (<0.1%)
	Overall	1/1432 (<0.1%)	0/1452	1/2884 (<0.1%)
Conjunctivitis bacterial	1st year	0/1432	1/1452 (<0.1%)	1/2884 (<0.1%)
	2nd year	0/1166	1/1206 (<0.1%)	1/2372 (<0.1%)
	3rd year	1/ 963 (0.1%)	0/1012	1/1975 (<0.1%)
	Overall	1/1432 (<0.1%)	2/1452 (0.1%)	3/2884 (0.1%)
Conjunctivitis infective	1st year	2/1432 (0.1%)	0/1452	2/2884 (<0.1%)
	Overall	2/1432 (0.1%)	0/1452	2/2884 (<0.1%)
Conjunctivitis viral	1st year	1/1432 (<0.1%)	1/1452 (<0.1%)	2/2884 (<0.1%)
	Overall	1/1432 (<0.1%)	1/1452 (<0.1%)	2/2884 (<0.1%)
Coxsackie viral infection	1st year	1/1432 (<0.1%)	1/1452 (<0.1%)	2/2884 (<0.1%)
	2nd year	1/1166 (<0.1%)	0/1206	1/2372 (<0.1%)
	Overall	2/1432 (0.1%)	1/1452 (<0.1%)	3/2884 (0.1%)
Cystitis	1st year	20/1432 (1.4%)	13/1452 (0.9%)	33/2884 (1.1%)
	2nd year	8/1166 (0.7%)	13/1206 (1.1%)	21/2372 (0.9%)
	3rd year	6/ 963 (0.6%)	6/1012 (0.6%)	12/1975 (0.6%)
	Overall	30/1432 (2.1%)	26/1452 (1.8%)	56/2884 (1.9%)
Dengue fever	1st year	1/1432 (<0.1%)	0/1452	1/2884 (<0.1%)
	2nd year	1/1166 (<0.1%)	0/1206	1/2372 (<0.1%)
	Overall	2/1432 (0.1%)	0/1452	2/2884 (<0.1%)
Diarrhoea infectious	1st year	1/1432 (<0.1%)	1/1452 (<0.1%)	2/2884 (<0.1%)
	3rd year	0/ 963	1/1012 (<0.1%)	1/1975 (<0.1%)
	Overall	1/1432 (<0.1%)	2/1452 (0.1%)	3/2884 (0.1%)

Table 14.3.1 / 9: Number of subjects with adverse events by primary system organ class, preferred term and year (FAS)

Primary system organ class<br>Preferred term<br>MedDRA version 14.0	YEAR OF ONSET	LCS12	LCS16	Total
Diverticulitis	1st year	1/1432 (<0.1%)	0/1452	1/2884 (<0.1%)
	2nd year	1/1166 (<0.1%)	1/1206 (<0.1%)	2/2372 (<0.1%)
	Overall	1/1432 (<0.1%)	1/1452 (<0.1%)	2/2884 (<0.1%)
Ear infection	1st year	15/1432 (1.0%)	7/1452 (0.5%)	22/2884 (0.8%)
	2nd year	9/1166 (0.8%)	5/1206 (0.4%)	14/2372 (0.6%)
	3rd year	6/ 963 (0.6%)	3/1012 (0.3%)	9/1975 (0.5%)
	Overall	27/1432 (1.9%)	13/1452 (0.9%)	40/2884 (1.4%)
Ear infection bacterial	1st year	1/1432 (<0.1%)	0/1452	1/2884 (<0.1%)
	Overall	1/1432 (<0.1%)	0/1452	1/2884 (<0.1%)
Endometritis	1st year	9/1432 (0.6%)	9/1452 (0.6%)	18/2884 (0.6%)
	2nd year	1/1166 (<0.1%)	3/1206 (0.2%)	4/2372 (0.2%)
	3rd year	2/ 963 (0.2%)	0/1012	2/1975 (0.1%)
	Overall	11/1432 (0.8%)	11/1452 (0.8%)	22/2884 (0.8%)
Enterobiasis	1st year	0/1432	1/1452 (<0.1%)	1/2884 (<0.1%)
	2nd year	1/1166 (<0.1%)	0/1206	1/2372 (<0.1%)
	3rd year	2/ 963 (0.2%)	0/1012	2/1975 (0.1%)
	Overall	3/1432 (0.2%)	1/1452 (<0.1%)	4/2884 (0.1%)
Enterocolitis infectious	1st year	1/1432 (<0.1%)	0/1452	1/2884 (<0.1%)
	2nd year	1/1166 (<0.1%)	0/1206	1/2372 (<0.1%)
	Overall	2/1432 (0.1%)	0/1452	2/2884 (<0.1%)
Epstein-Barr virus infection	1st year	0/1432	1/1452 (<0.1%)	1/2884 (<0.1%)
	Overall	0/1432	1/1452 (<0.1%)	1/2884 (<0.1%)
Erysipelas	2nd year	1/1166 (<0.1%)	0/1206	1/2372 (<0.1%)
	Overall	1/1432 (<0.1%)	0/1452	1/2884 (<0.1%)
Erythema infectiosum	2nd year	1/1166 (<0.1%)	0/1206	1/2372 (<0.1%)
	Overall	1/1432 (<0.1%)	0/1452	1/2884 (<0.1%)
Escherichia urinary tract infection	1st year	0/1432	1/1452 (<0.1%)	1/2884 (<0.1%)
	Overall	0/1432	1/1452 (<0.1%)	1/2884 (<0.1%)
Escherichia vaginitis	1st year	1/1432 (<0.1%)	0/1452	1/2884 (<0.1%)
	Overall	1/1432 (<0.1%)	0/1452	1/2884 (<0.1%)

Table 14.3.1 / 9: Number of subjects with adverse events by primary system organ class, preferred term and year (FAS)

Primary system organ class<br>Preferred term<br>MedDRA version 14.0	YEAR OF ONSET	LCS12	LCS16	Total
Eye infection	3rd year	0/ 963	1/1012 (<0.1%)	1/1975 (<0.1%)
	Overall	0/1432	1/1452 (<0.1%)	1/2884 (<0.1%)
Eyelid infection	2nd year	1/1166 (<0.1%)	0/1206	1/2372 (<0.1%)
	Overall	1/1432 (<0.1%)	0/1452	1/2884 (<0.1%)
Folliculitis	1st year	4/1432 (0.3%)	2/1452 (0.1%)	6/2884 (0.2%)
	2nd year	1/1166 (<0.1%)	0/1206	1/2372 (<0.1%)
	3rd year	0/ 963	4/1012 (0.4%)	4/1975 (0.2%)
	Overall	5/1432 (0.3%)	6/1452 (0.4%)	11/2884 (0.4%)
Fungal infection	1st year	5/1432 (0.3%)	6/1452 (0.4%)	11/2884 (0.4%)
	2nd year	0/1166	2/1206 (0.2%)	2/2372 (<0.1%)
	3rd year	0/ 963	1/1012 (<0.1%)	1/1975 (<0.1%)
	Overall	5/1432 (0.3%)	8/1452 (0.6%)	13/2884 (0.5%)
Fungal skin infection	1st year	1/1432 (<0.1%)	0/1452	1/2884 (<0.1%)
	2nd year	2/1166 (0.2%)	1/1206 (<0.1%)	3/2372 (0.1%)
	Overall	3/1432 (0.2%)	1/1452 (<0.1%)	4/2884 (0.1%)
Furuncle	1st year	1/1432 (<0.1%)	1/1452 (<0.1%)	2/2884 (<0.1%)
	Overall	1/1432 (<0.1%)	1/1452 (<0.1%)	2/2884 (<0.1%)
Gastritis bacterial	1st year	0/1432	1/1452 (<0.1%)	1/2884 (<0.1%)
	Overall	0/1432	1/1452 (<0.1%)	1/2884 (<0.1%)
Gastritis viral	1st year	0/1432	1/1452 (<0.1%)	1/2884 (<0.1%)
	Overall	0/1432	1/1452 (<0.1%)	1/2884 (<0.1%)
Gastroenteritis	1st year	19/1432 (1.3%)	16/1452 (1.1%)	35/2884 (1.2%)
	2nd year	9/1166 (0.8%)	6/1206 (0.5%)	15/2372 (0.6%)
	3rd year	6/ 963 (0.6%)	7/1012 (0.7%)	13/1975 (0.7%)
	Overall	31/1432 (2.2%)	29/1452 (2.0%)	60/2884 (2.1%)
Gastroenteritis rotavirus	1st year	0/1432	1/1452 (<0.1%)	1/2884 (<0.1%)
	Overall	0/1432	1/1452 (<0.1%)	1/2884 (<0.1%)

Table 14.3.1 / 9: Number of subjects with adverse events by primary system organ class, preferred term and year (FAS)

Primary system organ class<br>Preferred term<br>MedDRA version 14.0	YEAR OF ONSET	LCS12	LCS16	Total
Gastroenteritis viral	1st year	5/1432 (0.3%)	4/1452 (0.3%)	9/2884 (0.3%)
	2nd year	0/1166	4/1206 (0.3%)	4/2372 (0.2%)
	3rd year	3/ 963 (0.3%)	1/1012 (<0.1%)	4/1975 (0.2%)
	Overall	7/1432 (0.5%)	8/1452 (0.6%)	15/2884 (0.5%)
Gastrointestinal bacterial infection	1st year	0/1432	1/1452 (<0.1%)	1/2884 (<0.1%)
	Overall	0/1432	1/1452 (<0.1%)	1/2884 (<0.1%)
Gastrointestinal viral infection	1st year	1/1432 (<0.1%)	0/1452	1/2884 (<0.1%)
	2nd year	0/1166	1/1206 (<0.1%)	1/2372 (<0.1%)
	3rd year	0/ 963	1/1012 (<0.1%)	1/1975 (<0.1%)
	Overall	1/1432 (<0.1%)	2/1452 (0.1%)	3/2884 (0.1%)
Genital candidiasis	1st year	1/1432 (<0.1%)	2/1452 (0.1%)	3/2884 (0.1%)
	2nd year	0/1166	1/1206 (<0.1%)	1/2372 (<0.1%)
	Overall	1/1432 (<0.1%)	3/1452 (0.2%)	4/2884 (0.1%)
Genital herpes	1st year	4/1432 (0.3%)	8/1452 (0.6%)	12/2884 (0.4%)
	2nd year	4/1166 (0.3%)	3/1206 (0.2%)	7/2372 (0.3%)
	3rd year	4/ 963 (0.4%)	4/1012 (0.4%)	8/1975 (0.4%)
	Overall	10/1432 (0.7%)	14/1452 (1.0%)	24/2884 (0.8%)
Genital infection fungal	1st year	1/1432 (<0.1%)	0/1452	1/2884 (<0.1%)
	Overall	1/1432 (<0.1%)	0/1452	1/2884 (<0.1%)
Giardiasis	2nd year	1/1166 (<0.1%)	0/1206	1/2372 (<0.1%)
	Overall	1/1432 (<0.1%)	0/1452	1/2884 (<0.1%)
Gonorrhoea	1st year	0/1432	1/1452 (<0.1%)	1/2884 (<0.1%)
	2nd year	1/1166 (<0.1%)	0/1206	1/2372 (<0.1%)
	Overall	1/1432 (<0.1%)	1/1452 (<0.1%)	2/2884 (<0.1%)
Groin infection	1st year	1/1432 (<0.1%)	0/1452	1/2884 (<0.1%)
	2nd year	1/1166 (<0.1%)	0/1206	1/2372 (<0.1%)
	Overall	2/1432 (0.1%)	0/1452	2/2884 (<0.1%)
Gynaecological chlamydia infection	1st year	3/1432 (0.2%)	2/1452 (0.1%)	5/2884 (0.2%)
	2nd year	0/1166	2/1206 (0.2%)	2/2372 (<0.1%)
	3rd year	0/ 963	2/1012 (0.2%)	2/1975 (0.1%)
	Overall	3/1432 (0.2%)	5/1452 (0.3%)	8/2884 (0.3%)

Table 14.3.1 / 9: Number of subjects with adverse events by primary system organ class, preferred term and year (FAS)

Primary system organ class<br>Preferred term<br>MedDRA version 14.0	YEAR OF ONSET	LCS12	LCS16	Total
H1N1 influenza	2nd year	7/1166 (0.6%)	5/1206 (0.4%)	12/2372 (0.5%)
	Overall	7/1432 (0.5%)	5/1452 (0.3%)	12/2884 (0.4%)
Haemophilus infection	1st year	0/1432	1/1452 (<0.1%)	1/2884 (<0.1%)
	Overall	0/1432	1/1452 (<0.1%)	1/2884 (<0.1%)
Hand-foot-and-mouth disease	3rd year	0/ 963	1/1012 (<0.1%)	1/1975 (<0.1%)
	Overall	0/1432	1/1452 (<0.1%)	1/2884 (<0.1%)
Helicobacter gastritis	2nd year	1/1166 (<0.1%)	1/1206 (<0.1%)	2/2372 (<0.1%)
	Overall	1/1432 (<0.1%)	1/1452 (<0.1%)	2/2884 (<0.1%)
Helicobacter infection	1st year	1/1432 (<0.1%)	0/1452	1/2884 (<0.1%)
	Overall	1/1432 (<0.1%)	0/1452	1/2884 (<0.1%)
Herpes dermatitis	1st year	0/1432	1/1452 (<0.1%)	1/2884 (<0.1%)
	2nd year	1/1166 (<0.1%)	0/1206	1/2372 (<0.1%)
	3rd year	1/ 963 (0.1%)	0/1012	1/1975 (<0.1%)
	Overall	1/1432 (<0.1%)	1/1452 (<0.1%)	2/2884 (<0.1%)
Herpes simplex	2nd year	0/1166	2/1206 (0.2%)	2/2372 (<0.1%)
	3rd year	1/ 963 (0.1%)	1/1012 (<0.1%)	2/1975 (0.1%)
	Overall	1/1432 (<0.1%)	3/1452 (0.2%)	4/2884 (0.1%)
Herpes zoster	1st year	3/1432 (0.2%)	1/1452 (<0.1%)	4/2884 (0.1%)
	2nd year	3/1166 (0.3%)	2/1206 (0.2%)	5/2372 (0.2%)
	3rd year	1/ 963 (0.1%)	1/1012 (<0.1%)	2/1975 (0.1%)
	Overall	7/1432 (0.5%)	4/1452 (0.3%)	11/2884 (0.4%)
Impetigo	1st year	1/1432 (<0.1%)	1/1452 (<0.1%)	2/2884 (<0.1%)
	2nd year	2/1166 (0.2%)	1/1206 (<0.1%)	3/2372 (0.1%)
	3rd year	1/ 963 (0.1%)	0/1012	1/1975 (<0.1%)
	Overall	4/1432 (0.3%)	2/1452 (0.1%)	6/2884 (0.2%)
Infected bites	1st year	1/1432 (<0.1%)	0/1452	1/2884 (<0.1%)
	2nd year	1/1166 (<0.1%)	0/1206	1/2372 (<0.1%)
	Overall	2/1432 (0.1%)	0/1452	2/2884 (<0.1%)
Infected cyst	2nd year	0/1166	1/1206 (<0.1%)	1/2372 (<0.1%)
	Overall	0/1432	1/1452 (<0.1%)	1/2884 (<0.1%)

Table 14.3.1 / 9: Number of subjects with adverse events by primary system organ class, preferred term and year (FAS)

Primary system organ class<br>Preferred term<br>MedDRA version 14.0	YEAR OF ONSET	LCS12	LCS16	Total
Infection	2nd year	0/1166	1/1206 (<0.1%)	1/2372 (<0.1%)
	Overall	0/1432	1/1452 (<0.1%)	1/2884 (<0.1%)
Infectious mononucleosis	1st year	0/1432	2/1452 (0.1%)	2/2884 (<0.1%)
	2nd year	1/1166 (<0.1%)	4/1206 (0.3%)	5/2372 (0.2%)
	Overall	1/1432 (<0.1%)	6/1452 (0.4%)	7/2884 (0.2%)
Influenza	1st year	46/1432 (3.2%)	59/1452 (4.1%)	105/2884 (3.6%)
	2nd year	27/1166 (2.3%)	32/1206 (2.7%)	59/2372 (2.5%)
	3rd year	14/ 963 (1.5%)	14/1012 (1.4%)	28/1975 (1.4%)
	Overall	72/1432 (5.0%)	94/1452 (6.5%)	166/2884 (5.8%)
Keratitis viral	1st year	0/1432	1/1452 (<0.1%)	1/2884 (<0.1%)
	Overall	0/1432	1/1452 (<0.1%)	1/2884 (<0.1%)
Kidney infection	1st year	1/1432 (<0.1%)	1/1452 (<0.1%)	2/2884 (<0.1%)
	2nd year	1/1166 (<0.1%)	3/1206 (0.2%)	4/2372 (0.2%)
	Overall	2/1432 (0.1%)	4/1452 (0.3%)	6/2884 (0.2%)
Labyrinthitis	1st year	0/1432	1/1452 (<0.1%)	1/2884 (<0.1%)
	3rd year	1/ 963 (0.1%)	0/1012	1/1975 (<0.1%)
	Overall	1/1432 (<0.1%)	1/1452 (<0.1%)	2/2884 (<0.1%)
Laryngitis	1st year	1/1432 (<0.1%)	5/1452 (0.3%)	6/2884 (0.2%)
	2nd year	5/1166 (0.4%)	4/1206 (0.3%)	9/2372 (0.4%)
	3rd year	1/ 963 (0.1%)	2/1012 (0.2%)	3/1975 (0.2%)
	Overall	7/1432 (0.5%)	10/1452 (0.7%)	17/2884 (0.6%)
Laryngitis bacterial	1st year	0/1432	1/1452 (<0.1%)	1/2884 (<0.1%)
	Overall	0/1432	1/1452 (<0.1%)	1/2884 (<0.1%)
Localised infection	1st year	1/1432 (<0.1%)	2/1452 (0.1%)	3/2884 (0.1%)
	2nd year	2/1166 (0.2%)	0/1206	2/2372 (<0.1%)
	3rd year	1/ 963 (0.1%)	0/1012	1/1975 (<0.1%)
	Overall	4/1432 (0.3%)	2/1452 (0.1%)	6/2884 (0.2%)
Lower respiratory tract infection	1st year	2/1432 (0.1%)	0/1452	2/2884 (<0.1%)
	2nd year	1/1166 (<0.1%)	1/1206 (<0.1%)	2/2372 (<0.1%)
	Overall	3/1432 (0.2%)	1/1452 (<0.1%)	4/2884 (0.1%)

Table 14.3.1 / 9: Number of subjects with adverse events by primary system organ class, preferred term and year (FAS)

Primary system organ class<br>Preferred term<br>MedDRA version 14.0	YEAR OF ONSET	LCS12	LCS16	Total
Lower respiratory tract infection bacterial	3rd year	1/ 963 (0.1%)	0/1012	1/1975 (<0.1%)
	Overall	1/1432 (<0.1%)	0/1452	1/2884 (<0.1%)
Lymph gland infection	3rd year	1/ 963 (0.1%)	0/1012	1/1975 (<0.1%)
	Overall	1/1432 (<0.1%)	0/1452	1/2884 (<0.1%)
Malaria	1st year	1/1432 (<0.1%)	0/1452	1/2884 (<0.1%)
	2nd year	0/1166	1/1206 (<0.1%)	1/2372 (<0.1%)
	Overall	1/1432 (<0.1%)	1/1452 (<0.1%)	2/2884 (<0.1%)
Mastitis	1st year	1/1432 (<0.1%)	1/1452 (<0.1%)	2/2884 (<0.1%)
	3rd year	2/ 963 (0.2%)	0/1012	2/1975 (0.1%)
	Overall	3/1432 (0.2%)	1/1452 (<0.1%)	4/2884 (0.1%)
Meningitis	1st year	1/1432 (<0.1%)	0/1452	1/2884 (<0.1%)
	Overall	1/1432 (<0.1%)	0/1452	1/2884 (<0.1%)
Meningitis viral	3rd year	0/ 963	1/1012 (<0.1%)	1/1975 (<0.1%)
	Overall	0/1432	1/1452 (<0.1%)	1/2884 (<0.1%)
Molluscum contagiosum	3rd year	0/ 963	1/1012 (<0.1%)	1/1975 (<0.1%)
	Overall	0/1432	1/1452 (<0.1%)	1/2884 (<0.1%)
Nail infection	1st year	1/1432 (<0.1%)	1/1452 (<0.1%)	2/2884 (<0.1%)
	3rd year	1/ 963 (0.1%)	0/1012	1/1975 (<0.1%)
	Overall	2/1432 (0.1%)	1/1452 (<0.1%)	3/2884 (0.1%)
Nasopharyngitis	1st year	70/1432 (4.9%)	73/1452 (5.0%)	143/2884 (5.0%)
	2nd year	35/1166 (3.0%)	36/1206 (3.0%)	71/2372 (3.0%)
	3rd year	21/ 963 (2.2%)	24/1012 (2.4%)	45/1975 (2.3%)
	Overall	103/1432 (7.2%)	109/1452 (7.5%)	212/2884 (7.4%)
Nipple infection	3rd year	0/ 963	1/1012 (<0.1%)	1/1975 (<0.1%)
	Overall	0/1432	1/1452 (<0.1%)	1/2884 (<0.1%)
Omphalitis	1st year	0/1432	1/1452 (<0.1%)	1/2884 (<0.1%)
	Overall	0/1432	1/1452 (<0.1%)	1/2884 (<0.1%)

Table 14.3.1 / 9: Number of subjects with adverse events by primary system organ class, preferred term and year (FAS)

Primary system organ class<br>Preferred term<br>MedDRA version 14.0	YEAR OF ONSET	LCS12	LCS16	Total
Onychomycosis	1st year	2/1432 (0.1%)	2/1452 (0.1%)	4/2884 (0.1%)
	2nd year	0/1166	2/1206 (0.2%)	2/2372 (<0.1%)
	3rd year	1/ 963 (0.1%)	0/1012	1/1975 (<0.1%)
	Overall	3/1432 (0.2%)	4/1452 (0.3%)	7/2884 (0.2%)
Oophoritis	1st year	0/1432	1/1452 (<0.1%)	1/2884 (<0.1%)
	Overall	0/1432	1/1452 (<0.1%)	1/2884 (<0.1%)
Oral candidiasis	2nd year	0/1166	1/1206 (<0.1%)	1/2372 (<0.1%)
	Overall	0/1432	1/1452 (<0.1%)	1/2884 (<0.1%)
Oral herpes	1st year	6/1432 (0.4%)	3/1452 (0.2%)	9/2884 (0.3%)
	2nd year	3/1166 (0.3%)	4/1206 (0.3%)	7/2372 (0.3%)
	3rd year	3/ 963 (0.3%)	4/1012 (0.4%)	7/1975 (0.4%)
	Overall	11/1432 (0.8%)	10/1452 (0.7%)	21/2884 (0.7%)
Osteomyelitis	1st year	0/1432	1/1452 (<0.1%)	1/2884 (<0.1%)
	2nd year	0/1166	1/1206 (<0.1%)	1/2372 (<0.1%)
	Overall	0/1432	1/1452 (<0.1%)	1/2884 (<0.1%)
Otitis externa	2nd year	0/1166	2/1206 (0.2%)	2/2372 (<0.1%)
	3rd year	0/ 963	2/1012 (0.2%)	2/1975 (0.1%)
	Overall	0/1432	3/1452 (0.2%)	3/2884 (0.1%)
Otitis media	1st year	3/1432 (0.2%)	3/1452 (0.2%)	6/2884 (0.2%)
	2nd year	1/1166 (<0.1%)	3/1206 (0.2%)	4/2372 (0.2%)
	3rd year	2/ 963 (0.2%)	1/1012 (<0.1%)	3/1975 (0.2%)
	Overall	5/1432 (0.3%)	6/1452 (0.4%)	11/2884 (0.4%)
Otitis media acute	1st year	0/1432	1/1452 (<0.1%)	1/2884 (<0.1%)
	Overall	0/1432	1/1452 (<0.1%)	1/2884 (<0.1%)
Papilloma viral infection	1st year	2/1432 (0.1%)	1/1452 (<0.1%)	3/2884 (0.1%)
	Overall	2/1432 (0.1%)	1/1452 (<0.1%)	3/2884 (0.1%)
Parasitic gastroenteritis	1st year	1/1432 (<0.1%)	0/1452	1/2884 (<0.1%)
	Overall	1/1432 (<0.1%)	0/1452	1/2884 (<0.1%)

Table 14.3.1 / 9: Number of subjects with adverse events by primary system organ class, preferred term and year (FAS)

Primary system organ class<br>Preferred term<br>MedDRA version 14.0	YEAR OF ONSET	LCS12	LCS16	Total
Paronychia	1st year	0/1432	1/1452 (<0.1%)	1/2884 (<0.1%)
	2nd year	1/1166 (<0.1%)	1/1206 (<0.1%)	2/2372 (<0.1%)
	Overall	1/1432 (<0.1%)	2/1452 (0.1%)	3/2884 (0.1%)
Pelvic infection	1st year	0/1432	1/1452 (<0.1%)	1/2884 (<0.1%)
	Overall	0/1432	1/1452 (<0.1%)	1/2884 (<0.1%)
Pelvic inflammatory disease	1st year	2/1432 (0.1%)	4/1452 (0.3%)	6/2884 (0.2%)
	2nd year	1/1166 (<0.1%)	0/1206	1/2372 (<0.1%)
	3rd year	2/ 963 (0.2%)	1/1012 (<0.1%)	3/1975 (0.2%)
	Overall	5/1432 (0.3%)	5/1452 (0.3%)	10/2884 (0.3%)
Peritoneal abscess	2nd year	1/1166 (<0.1%)	0/1206	1/2372 (<0.1%)
	Overall	1/1432 (<0.1%)	0/1452	1/2884 (<0.1%)
Peritonsillar abscess	1st year	2/1432 (0.1%)	1/1452 (<0.1%)	3/2884 (0.1%)
	Overall	2/1432 (0.1%)	1/1452 (<0.1%)	3/2884 (0.1%)
Pharyngeal abscess	3rd year	1/ 963 (0.1%)	0/1012	1/1975 (<0.1%)
	Overall	1/1432 (<0.1%)	0/1452	1/2884 (<0.1%)
Pharyngitis	1st year	8/1432 (0.6%)	14/1452 (1.0%)	22/2884 (0.8%)
	2nd year	3/1166 (0.3%)	4/1206 (0.3%)	7/2372 (0.3%)
	3rd year	2/ 963 (0.2%)	4/1012 (0.4%)	6/1975 (0.3%)
	Overall	13/1432 (0.9%)	21/1452 (1.4%)	34/2884 (1.2%)
Pharyngitis streptococcal	1st year	11/1432 (0.8%)	12/1452 (0.8%)	23/2884 (0.8%)
	2nd year	6/1166 (0.5%)	5/1206 (0.4%)	11/2372 (0.5%)
	3rd year	7/ 963 (0.7%)	5/1012 (0.5%)	12/1975 (0.6%)
	Overall	23/1432 (1.6%)	21/1452 (1.4%)	44/2884 (1.5%)
Pharyngotonsillitis	3rd year	1/ 963 (0.1%)	0/1012	1/1975 (<0.1%)
	Overall	1/1432 (<0.1%)	0/1452	1/2884 (<0.1%)
Pilonidal cyst	2nd year	1/1166 (<0.1%)	0/1206	1/2372 (<0.1%)
	Overall	1/1432 (<0.1%)	0/1452	1/2884 (<0.1%)

Table 14.3.1 / 9: Number of subjects with adverse events by primary system organ class, preferred term and year (FAS)

Primary system organ class<br>Preferred term<br>MedDRA version 14.0	YEAR OF ONSET	LCS12	LCS16	Total
Pneumonia	1st year	7/1432 (0.5%)	6/1452 (0.4%)	13/2884 (0.5%)
	2nd year	3/1166 (0.3%)	8/1206 (0.7%)	11/2372 (0.5%)
	3rd year	4/ 963 (0.4%)	2/1012 (0.2%)	6/1975 (0.3%)
	Overall	14/1432 (1.0%)	15/1452 (1.0%)	29/2884 (1.0%)
Pneumonia mycoplasmal	2nd year	1/1166 (<0.1%)	0/1206	1/2372 (<0.1%)
	Overall	1/1432 (<0.1%)	0/1452	1/2884 (<0.1%)
Pogosta disease	2nd year	1/1166 (<0.1%)	0/1206	1/2372 (<0.1%)
	Overall	1/1432 (<0.1%)	0/1452	1/2884 (<0.1%)
Post procedural infection	1st year	3/1432 (0.2%)	0/1452	3/2884 (0.1%)
	3rd year	0/ 963	1/1012 (<0.1%)	1/1975 (<0.1%)
	Overall	3/1432 (0.2%)	1/1452 (<0.1%)	4/2884 (0.1%)
Pulpitis dental	2nd year	0/1166	1/1206 (<0.1%)	1/2372 (<0.1%)
	Overall	0/1432	1/1452 (<0.1%)	1/2884 (<0.1%)
Pyelonephritis	1st year	6/1432 (0.4%)	2/1452 (0.1%)	8/2884 (0.3%)
	2nd year	3/1166 (0.3%)	1/1206 (<0.1%)	4/2372 (0.2%)
	Overall	9/1432 (0.6%)	3/1452 (0.2%)	12/2884 (0.4%)
Pyelonephritis acute	1st year	1/1432 (<0.1%)	0/1452	1/2884 (<0.1%)
	3rd year	1/ 963 (0.1%)	0/1012	1/1975 (<0.1%)
	Overall	2/1432 (0.1%)	0/1452	2/2884 (<0.1%)
Q fever	2nd year	1/1166 (<0.1%)	0/1206	1/2372 (<0.1%)
	Overall	1/1432 (<0.1%)	0/1452	1/2884 (<0.1%)
Rash pustular	1st year	0/1432	1/1452 (<0.1%)	1/2884 (<0.1%)
	Overall	0/1432	1/1452 (<0.1%)	1/2884 (<0.1%)
Respiratory tract infection	1st year	6/1432 (0.4%)	7/1452 (0.5%)	13/2884 (0.5%)
	2nd year	5/1166 (0.4%)	5/1206 (0.4%)	10/2372 (0.4%)
	3rd year	0/ 963	9/1012 (0.9%)	9/1975 (0.5%)
	Overall	10/1432 (0.7%)	16/1452 (1.1%)	26/2884 (0.9%)
Respiratory tract infection viral	1st year	0/1432	1/1452 (<0.1%)	1/2884 (<0.1%)
	3rd year	2/ 963 (0.2%)	0/1012	2/1975 (0.1%)
	Overall	2/1432 (0.1%)	1/1452 (<0.1%)	3/2884 (0.1%)

Table 14.3.1 / 9: Number of subjects with adverse events by primary system organ class, preferred term and year (FAS)

Primary system organ class<br>Preferred term<br>MedDRA version 14.0	YEAR OF ONSET	LCS12	LCS16	Total
Rhinitis	1st year	6/1432 (0.4%)	7/1452 (0.5%)	13/2884 (0.5%)
	2nd year	0/1166	4/1206 (0.3%)	4/2372 (0.2%)
	3rd year	0/ 963	1/1012 (<0.1%)	1/1975 (<0.1%)
	Overall	6/1432 (0.4%)	12/1452 (0.8%)	18/2884 (0.6%)
Salpingitis	2nd year	1/1166 (<0.1%)	0/1206	1/2372 (<0.1%)
	Overall	1/1432 (<0.1%)	0/1452	1/2884 (<0.1%)
Salpingo-oophoritis	1st year	2/1432 (0.1%)	3/1452 (0.2%)	5/2884 (0.2%)
	Overall	2/1432 (0.1%)	3/1452 (0.2%)	5/2884 (0.2%)
Sialoadenitis	1st year	1/1432 (<0.1%)	0/1452	1/2884 (<0.1%)
	2nd year	1/1166 (<0.1%)	0/1206	1/2372 (<0.1%)
	Overall	1/1432 (<0.1%)	0/1452	1/2884 (<0.1%)
Sinobronchitis	3rd year	0/ 963	1/1012 (<0.1%)	1/1975 (<0.1%)
	Overall	0/1432	1/1452 (<0.1%)	1/2884 (<0.1%)
Sinusitis	1st year	55/1432 (3.8%)	47/1452 (3.2%)	102/2884 (3.5%)
	2nd year	33/1166 (2.8%)	34/1206 (2.8%)	67/2372 (2.8%)
	3rd year	26/ 963 (2.7%)	29/1012 (2.9%)	55/1975 (2.8%)
	Overall	90/1432 (6.3%)	96/1452 (6.6%)	186/2884 (6.4%)
Skin bacterial infection	1st year	0/1432	1/1452 (<0.1%)	1/2884 (<0.1%)
	3rd year	1/ 963 (0.1%)	1/1012 (<0.1%)	2/1975 (0.1%)
	Overall	1/1432 (<0.1%)	2/1452 (0.1%)	3/2884 (0.1%)
Skin candida	3rd year	1/ 963 (0.1%)	0/1012	1/1975 (<0.1%)
	Overall	1/1432 (<0.1%)	0/1452	1/2884 (<0.1%)
Skin infection	1st year	1/1432 (<0.1%)	2/1452 (0.1%)	3/2884 (0.1%)
	2nd year	0/1166	2/1206 (0.2%)	2/2372 (<0.1%)
	3rd year	0/ 963	2/1012 (0.2%)	2/1975 (0.1%)
	Overall	1/1432 (<0.1%)	6/1452 (0.4%)	7/2884 (0.2%)
Small intestinal bacterial overgrowth	3rd year	0/ 963	1/1012 (<0.1%)	1/1975 (<0.1%)
	Overall	0/1432	1/1452 (<0.1%)	1/2884 (<0.1%)
Staphylococcal infection	1st year	1/1432 (<0.1%)	1/1452 (<0.1%)	2/2884 (<0.1%)
	Overall	1/1432 (<0.1%)	1/1452 (<0.1%)	2/2884 (<0.1%)

Table 14.3.1 / 9: Number of subjects with adverse events by primary system organ class, preferred term and year (FAS)

Primary system organ class<br>Preferred term<br>MedDRA version 14.0	YEAR OF ONSET	LCS12	LCS16	Total
Staphylococcal skin infection	1st year	1/1432 (<0.1%)	0/1452	1/2884 (<0.1%)
	Overall	1/1432 (<0.1%)	0/1452	1/2884 (<0.1%)
Streptococcal infection	2nd year	1/1166 (<0.1%)	0/1206	1/2372 (<0.1%)
	Overall	1/1432 (<0.1%)	0/1452	1/2884 (<0.1%)
Streptococcal sepsis	2nd year	1/1166 (<0.1%)	0/1206	1/2372 (<0.1%)
	Overall	1/1432 (<0.1%)	0/1452	1/2884 (<0.1%)
Subcutaneous abscess	1st year	2/1432 (0.1%)	0/1452	2/2884 (<0.1%)
	2nd year	1/1166 (<0.1%)	2/1206 (0.2%)	3/2372 (0.1%)
	3rd year	1/ 963 (0.1%)	0/1012	1/1975 (<0.1%)
	Overall	3/1432 (0.2%)	2/1452 (0.1%)	5/2884 (0.2%)
Sweat gland infection	1st year	1/1432 (<0.1%)	0/1452	1/2884 (<0.1%)
	Overall	1/1432 (<0.1%)	0/1452	1/2884 (<0.1%)
Tinea infection	1st year	1/1432 (<0.1%)	0/1452	1/2884 (<0.1%)
	3rd year	0/ 963	2/1012 (0.2%)	2/1975 (0.1%)
	Overall	1/1432 (<0.1%)	2/1452 (0.1%)	3/2884 (0.1%)
Tinea pedis	1st year	0/1432	1/1452 (<0.1%)	1/2884 (<0.1%)
	3rd year	1/ 963 (0.1%)	0/1012	1/1975 (<0.1%)
	Overall	1/1432 (<0.1%)	1/1452 (<0.1%)	2/2884 (<0.1%)
Tinea versicolour	1st year	0/1432	1/1452 (<0.1%)	1/2884 (<0.1%)
	Overall	0/1432	1/1452 (<0.1%)	1/2884 (<0.1%)
Tonsillitis	1st year	15/1432 (1.0%)	8/1452 (0.6%)	23/2884 (0.8%)
	2nd year	9/1166 (0.8%)	8/1206 (0.7%)	17/2372 (0.7%)
	3rd year	6/ 963 (0.6%)	8/1012 (0.8%)	14/1975 (0.7%)
	Overall	25/1432 (1.7%)	24/1452 (1.7%)	49/2884 (1.7%)
Tonsillitis streptococcal	2nd year	1/1166 (<0.1%)	0/1206	1/2372 (<0.1%)
	3rd year	1/ 963 (0.1%)	0/1012	1/1975 (<0.1%)
	Overall	1/1432 (<0.1%)	0/1452	1/2884 (<0.1%)
Tooth abscess	1st year	5/1432 (0.3%)	3/1452 (0.2%)	8/2884 (0.3%)
	2nd year	0/1166	1/1206 (<0.1%)	1/2372 (<0.1%)
	Overall	5/1432 (0.3%)	4/1452 (0.3%)	9/2884 (0.3%)

Table 14.3.1 / 9: Number of subjects with adverse events by primary system organ class, preferred term and year (FAS)

Primary system organ class<br>Preferred term<br>MedDRA version 14.0	YEAR OF ONSET	LCS12	LCS16	Total
Tooth infection	1st year	11/1432 (0.8%)	7/1452 (0.5%)	18/2884 (0.6%)
	2nd year	4/1166 (0.3%)	3/1206 (0.2%)	7/2372 (0.3%)
	3rd year	10/ 963 (1.0%)	6/1012 (0.6%)	16/1975 (0.8%)
	Overall	25/1432 (1.7%)	15/1452 (1.0%)	40/2884 (1.4%)
Tracheitis	1st year	1/1432 (<0.1%)	0/1452	1/2884 (<0.1%)
	Overall	1/1432 (<0.1%)	0/1452	1/2884 (<0.1%)
Trichomoniasis	1st year	1/1432 (<0.1%)	0/1452	1/2884 (<0.1%)
	2nd year	1/1166 (<0.1%)	1/1206 (<0.1%)	2/2372 (<0.1%)
	3rd year	0/ 963	1/1012 (<0.1%)	1/1975 (<0.1%)
	Overall	2/1432 (0.1%)	2/1452 (0.1%)	4/2884 (0.1%)
Tuberculosis	3rd year	1/ 963 (0.1%)	0/1012	1/1975 (<0.1%)
	Overall	1/1432 (<0.1%)	0/1452	1/2884 (<0.1%)
Tubo-ovarian abscess	1st year	1/1432 (<0.1%)	0/1452	1/2884 (<0.1%)
	Overall	1/1432 (<0.1%)	0/1452	1/2884 (<0.1%)
Typhoid fever	1st year	0/1432	1/1452 (<0.1%)	1/2884 (<0.1%)
	Overall	0/1432	1/1452 (<0.1%)	1/2884 (<0.1%)
Upper respiratory tract infection	1st year	31/1432 (2.2%)	41/1452 (2.8%)	72/2884 (2.5%)
	2nd year	16/1166 (1.4%)	20/1206 (1.7%)	36/2372 (1.5%)
	3rd year	13/ 963 (1.3%)	8/1012 (0.8%)	21/1975 (1.1%)
	Overall	51/1432 (3.6%)	57/1452 (3.9%)	108/2884 (3.7%)
Ureaplasma infection	2nd year	1/1166 (<0.1%)	0/1206	1/2372 (<0.1%)
	Overall	1/1432 (<0.1%)	0/1452	1/2884 (<0.1%)
Urinary tract infection	1st year	94/1432 (6.6%)	87/1452 (6.0%)	181/2884 (6.3%)
	2nd year	42/1166 (3.6%)	38/1206 (3.2%)	80/2372 (3.4%)
	3rd year	43/ 963 (4.5%)	38/1012 (3.8%)	81/1975 (4.1%)
	Overall	158/1432 (11.0%)	145/1452 (10.0%)	303/2884 (10.5%)
Uterine infection	1st year	0/1432	1/1452 (<0.1%)	1/2884 (<0.1%)
	Overall	0/1432	1/1452 (<0.1%)	1/2884 (<0.1%)

Table 14.3.1 / 9: Number of subjects with adverse events by primary system organ class, preferred term and year (FAS)

Primary system organ class<br>Preferred term<br>MedDRA version 14.0	YEAR OF ONSET	LCS12	LCS16	Total
Vaginal infection	1st year	28/1432 (2.0%)	40/1452 (2.8%)	68/2884 (2.4%)
	2nd year	20/1166 (1.7%)	10/1206 (0.8%)	30/2372 (1.3%)
	3rd year	6/ 963 (0.6%)	15/1012 (1.5%)	21/1975 (1.1%)
	Overall	48/1432 (3.4%)	60/1452 (4.1%)	108/2884 (3.7%)
Vaginitis bacterial	1st year	52/1432 (3.6%)	61/1452 (4.2%)	113/2884 (3.9%)
	2nd year	36/1166 (3.1%)	50/1206 (4.1%)	86/2372 (3.6%)
	3rd year	30/ 963 (3.1%)	45/1012 (4.4%)	75/1975 (3.8%)
	Overall	105/1432 (7.3%)	127/1452 (8.7%)	232/2884 (8.0%)
Vaginitis chlamydial	1st year	1/1432 (<0.1%)	1/1452 (<0.1%)	2/2884 (<0.1%)
	2nd year	1/1166 (<0.1%)	0/1206	1/2372 (<0.1%)
	Overall	2/1432 (0.1%)	1/1452 (<0.1%)	3/2884 (0.1%)
Vaginitis gardnerella	1st year	0/1432	2/1452 (0.1%)	2/2884 (<0.1%)
	2nd year	0/1166	1/1206 (<0.1%)	1/2372 (<0.1%)
	3rd year	0/ 963	1/1012 (<0.1%)	1/1975 (<0.1%)
	Overall	0/1432	4/1452 (0.3%)	4/2884 (0.1%)
Vestibular neuronitis	1st year	2/1432 (0.1%)	0/1452	2/2884 (<0.1%)
	Overall	2/1432 (0.1%)	0/1452	2/2884 (<0.1%)
Viral infection	1st year	0/1432	2/1452 (0.1%)	2/2884 (<0.1%)
	2nd year	2/1166 (0.2%)	1/1206 (<0.1%)	3/2372 (0.1%)
	3rd year	1/ 963 (0.1%)	0/1012	1/1975 (<0.1%)
	Overall	3/1432 (0.2%)	3/1452 (0.2%)	6/2884 (0.2%)
Viral pharyngitis	1st year	0/1432	1/1452 (<0.1%)	1/2884 (<0.1%)
	2nd year	1/1166 (<0.1%)	0/1206	1/2372 (<0.1%)
	Overall	1/1432 (<0.1%)	1/1452 (<0.1%)	2/2884 (<0.1%)
Viral rhinitis	2nd year	1/1166 (<0.1%)	0/1206	1/2372 (<0.1%)
	Overall	1/1432 (<0.1%)	0/1452	1/2884 (<0.1%)
Viral upper respiratory tract infection	1st year	7/1432 (0.5%)	7/1452 (0.5%)	14/2884 (0.5%)
	2nd year	9/1166 (0.8%)	9/1206 (0.7%)	18/2372 (0.8%)
	3rd year	1/ 963 (0.1%)	3/1012 (0.3%)	4/1975 (0.2%)
	Overall	15/1432 (1.0%)	14/1452 (1.0%)	29/2884 (1.0%)

Table 14.3.1 / 9: Number of subjects with adverse events by primary system organ class, preferred term and year (FAS)

Primary system organ class<br>Preferred term<br>MedDRA version 14.0	YEAR OF ONSET	LCS12	LCS16	Total
Vulval abscess	1st year	0/1432	1/1452 (<0.1%)	1/2884 (<0.1%)
	Overall	0/1432	1/1452 (<0.1%)	1/2884 (<0.1%)
Vulvitis	1st year	5/1432 (0.3%)	5/1452 (0.3%)	10/2884 (0.3%)
	2nd year	3/1166 (0.3%)	1/1206 (<0.1%)	4/2372 (0.2%)
	3rd year	0/ 963	1/1012 (<0.1%)	1/1975 (<0.1%)
	Overall	8/1432 (0.6%)	7/1452 (0.5%)	15/2884 (0.5%)
Vulvovaginal candidiasis	1st year	40/1432 (2.8%)	39/1452 (2.7%)	79/2884 (2.7%)
	2nd year	25/1166 (2.1%)	30/1206 (2.5%)	55/2372 (2.3%)
	3rd year	16/ 963 (1.7%)	19/1012 (1.9%)	35/1975 (1.8%)
	Overall	72/1432 (5.0%)	72/1452 (5.0%)	144/2884 (5.0%)
Vulvovaginal mycotic infection	1st year	61/1432 (4.3%)	67/1452 (4.6%)	128/2884 (4.4%)
	2nd year	35/1166 (3.0%)	40/1206 (3.3%)	75/2372 (3.2%)
	3rd year	20/ 963 (2.1%)	31/1012 (3.1%)	51/1975 (2.6%)
	Overall	99/1432 (6.9%)	110/1452 (7.6%)	209/2884 (7.2%)
Vulvovaginitis	1st year	6/1432 (0.4%)	5/1452 (0.3%)	11/2884 (0.4%)
	2nd year	0/1166	1/1206 (<0.1%)	1/2372 (<0.1%)
	3rd year	1/ 963 (0.1%)	2/1012 (0.2%)	3/1975 (0.2%)
	Overall	6/1432 (0.4%)	7/1452 (0.5%)	13/2884 (0.5%)
Vulvovaginitis chlamydial	2nd year	0/1166	1/1206 (<0.1%)	1/2372 (<0.1%)
	Overall	0/1432	1/1452 (<0.1%)	1/2884 (<0.1%)
Vulvovaginitis streptococcal	1st year	1/1432 (<0.1%)	1/1452 (<0.1%)	2/2884 (<0.1%)
	3rd year	0/ 963	1/1012 (<0.1%)	1/1975 (<0.1%)
	Overall	1/1432 (<0.1%)	2/1452 (0.1%)	3/2884 (0.1%)
Vulvovaginitis trichomonal	1st year	1/1432 (<0.1%)	2/1452 (0.1%)	3/2884 (0.1%)
	2nd year	1/1166 (<0.1%)	1/1206 (<0.1%)	2/2372 (<0.1%)
	3rd year	2/ 963 (0.2%)	1/1012 (<0.1%)	3/1975 (0.2%)
	Overall	4/1432 (0.3%)	4/1452 (0.3%)	8/2884 (0.3%)
Wound infection	1st year	1/1432 (<0.1%)	1/1452 (<0.1%)	2/2884 (<0.1%)
	2nd year	1/1166 (<0.1%)	0/1206	1/2372 (<0.1%)
	3rd year	1/ 963 (0.1%)	0/1012	1/1975 (<0.1%)
	Overall	3/1432 (0.2%)	1/1452 (<0.1%)	4/2884 (0.1%)

Table 14.3.1 / 9: Number of subjects with adverse events by primary system organ class, preferred term and year (FAS)

Primary system organ class<br>Preferred term<br>MedDRA version 14.0	YEAR OF ONSET	LCS12	LCS16	Total
Wound infection staphylococcal	3rd year	0/ 963	1/1012 (<0.1%)	1/1975 (<0.1%)
	Overall	0/1432	1/1452 (<0.1%)	1/2884 (<0.1%)
Injury, poisoning and procedural complications	1st year	98/1432 (6.8%)	88/1452 (6.1%)	186/2884 (6.4%)
	2nd year	31/1166 (2.7%)	29/1206 (2.4%)	60/2372 (2.5%)
	3rd year	30/ 963 (3.1%)	30/1012 (3.0%)	60/1975 (3.0%)
	Overall	149/1432 (10.4%)	133/1452 (9.2%)	282/2884 (9.8%)
Abdominal wound dehiscence	3rd year	0/ 963	1/1012 (<0.1%)	1/1975 (<0.1%)
	Overall	0/1432	1/1452 (<0.1%)	1/2884 (<0.1%)
Accident	2nd year	0/1166	1/1206 (<0.1%)	1/2372 (<0.1%)
	Overall	0/1432	1/1452 (<0.1%)	1/2884 (<0.1%)
Animal bite	1st year	2/1432 (0.1%)	1/1452 (<0.1%)	3/2884 (0.1%)
	2nd year	1/1166 (<0.1%)	1/1206 (<0.1%)	2/2372 (<0.1%)
	3rd year	0/ 963	1/1012 (<0.1%)	1/1975 (<0.1%)
	Overall	3/1432 (0.2%)	2/1452 (0.1%)	5/2884 (0.2%)
Ankle fracture	1st year	0/1432	1/1452 (<0.1%)	1/2884 (<0.1%)
	2nd year	1/1166 (<0.1%)	1/1206 (<0.1%)	2/2372 (<0.1%)
	Overall	1/1432 (<0.1%)	2/1452 (0.1%)	3/2884 (0.1%)
Arthropod bite	1st year	3/1432 (0.2%)	2/1452 (0.1%)	5/2884 (0.2%)
	2nd year	1/1166 (<0.1%)	0/1206	1/2372 (<0.1%)
	3rd year	1/ 963 (0.1%)	0/1012	1/1975 (<0.1%)
	Overall	5/1432 (0.3%)	2/1452 (0.1%)	7/2884 (0.2%)
Arthropod sting	1st year	0/1432	1/1452 (<0.1%)	1/2884 (<0.1%)
	3rd year	0/ 963	1/1012 (<0.1%)	1/1975 (<0.1%)
	Overall	0/1432	2/1452 (0.1%)	2/2884 (<0.1%)
Back injury	2nd year	0/1166	1/1206 (<0.1%)	1/2372 (<0.1%)
	Overall	0/1432	1/1452 (<0.1%)	1/2884 (<0.1%)
Burns second degree	2nd year	0/1166	1/1206 (<0.1%)	1/2372 (<0.1%)
	Overall	0/1432	1/1452 (<0.1%)	1/2884 (<0.1%)
Burns third degree	1st year	1/1432 (<0.1%)	0/1452	1/2884 (<0.1%)
	Overall	1/1432 (<0.1%)	0/1452	1/2884 (<0.1%)

Table 14.3.1 / 9: Number of subjects with adverse events by primary system organ class, preferred term and year (FAS)

Primary system organ class<br>Preferred term<br>MedDRA version 14.0	YEAR OF ONSET	LCS12	LCS16	Total
Cartilage injury	2nd year	0/1166	1/1206 (<0.1%)	1/2372 (<0.1%)
	Overall	0/1432	1/1452 (<0.1%)	1/2884 (<0.1%)
Clavicle fracture	3rd year	1/ 963 (0.1%)	0/1012	1/1975 (<0.1%)
	Overall	1/1432 (<0.1%)	0/1452	1/2884 (<0.1%)
Concussion	1st year	1/1432 (<0.1%)	6/1452 (0.4%)	7/2884 (0.2%)
	2nd year	2/1166 (0.2%)	1/1206 (<0.1%)	3/2372 (0.1%)
	3rd year	0/ 963	1/1012 (<0.1%)	1/1975 (<0.1%)
	Overall	3/1432 (0.2%)	8/1452 (0.6%)	11/2884 (0.4%)
Contusion	1st year	5/1432 (0.3%)	2/1452 (0.1%)	7/2884 (0.2%)
	2nd year	0/1166	2/1206 (0.2%)	2/2372 (<0.1%)
	3rd year	2/ 963 (0.2%)	1/1012 (<0.1%)	3/1975 (0.2%)
	Overall	7/1432 (0.5%)	5/1452 (0.3%)	12/2884 (0.4%)
Epicondylitis	1st year	2/1432 (0.1%)	3/1452 (0.2%)	5/2884 (0.2%)
	2nd year	0/1166	1/1206 (<0.1%)	1/2372 (<0.1%)
	Overall	2/1432 (0.1%)	4/1452 (0.3%)	6/2884 (0.2%)
Excoriation	1st year	0/1432	1/1452 (<0.1%)	1/2884 (<0.1%)
	Overall	0/1432	1/1452 (<0.1%)	1/2884 (<0.1%)
Eye injury	1st year	0/1432	1/1452 (<0.1%)	1/2884 (<0.1%)
	3rd year	1/ 963 (0.1%)	0/1012	1/1975 (<0.1%)
	Overall	1/1432 (<0.1%)	1/1452 (<0.1%)	2/2884 (<0.1%)
Facial bones fracture	2nd year	2/1166 (0.2%)	0/1206	2/2372 (<0.1%)
	Overall	2/1432 (0.1%)	0/1452	2/2884 (<0.1%)
Fall	3rd year	0/ 963	1/1012 (<0.1%)	1/1975 (<0.1%)
	Overall	0/1432	1/1452 (<0.1%)	1/2884 (<0.1%)
Foot fracture	1st year	3/1432 (0.2%)	3/1452 (0.2%)	6/2884 (0.2%)
	2nd year	3/1166 (0.3%)	0/1206	3/2372 (0.1%)
	3rd year	4/ 963 (0.4%)	0/1012	4/1975 (0.2%)
	Overall	9/1432 (0.6%)	3/1452 (0.2%)	12/2884 (0.4%)
Forearm fracture	1st year	1/1432 (<0.1%)	0/1452	1/2884 (<0.1%)
	Overall	1/1432 (<0.1%)	0/1452	1/2884 (<0.1%)

Table 14.3.1 / 9: Number of subjects with adverse events by primary system organ class, preferred term and year (FAS)

Primary system organ class<br>Preferred term<br>MedDRA version 14.0	YEAR OF ONSET	LCS12	LCS16	Total
Frostbite	1st year	1/1432 (<0.1%)	0/1452	1/2884 (<0.1%)
	Overall	1/1432 (<0.1%)	0/1452	1/2884 (<0.1%)
Hand fracture	2nd year	0/1166	1/1206 (<0.1%)	1/2372 (<0.1%)
	3rd year	1/ 963 (0.1%)	2/1012 (0.2%)	3/1975 (0.2%)
	Overall	1/1432 (<0.1%)	3/1452 (0.2%)	4/2884 (0.1%)
Head injury	1st year	1/1432 (<0.1%)	0/1452	1/2884 (<0.1%)
	3rd year	0/ 963	1/1012 (<0.1%)	1/1975 (<0.1%)
	Overall	1/1432 (<0.1%)	1/1452 (<0.1%)	2/2884 (<0.1%)
Humerus fracture	3rd year	0/ 963	1/1012 (<0.1%)	1/1975 (<0.1%)
	Overall	0/1432	1/1452 (<0.1%)	1/2884 (<0.1%)
Injury	1st year	0/1432	1/1452 (<0.1%)	1/2884 (<0.1%)
	Overall	0/1432	1/1452 (<0.1%)	1/2884 (<0.1%)
Joint dislocation	1st year	3/1432 (0.2%)	1/1452 (<0.1%)	4/2884 (0.1%)
	2nd year	1/1166 (<0.1%)	0/1206	1/2372 (<0.1%)
	3rd year	1/ 963 (0.1%)	0/1012	1/1975 (<0.1%)
	Overall	5/1432 (0.3%)	1/1452 (<0.1%)	6/2884 (0.2%)
Joint injury	1st year	5/1432 (0.3%)	2/1452 (0.1%)	7/2884 (0.2%)
	2nd year	1/1166 (<0.1%)	1/1206 (<0.1%)	2/2372 (<0.1%)
	3rd year	1/ 963 (0.1%)	3/1012 (0.3%)	4/1975 (0.2%)
	Overall	7/1432 (0.5%)	6/1452 (0.4%)	13/2884 (0.5%)
Joint sprain	1st year	7/1432 (0.5%)	3/1452 (0.2%)	10/2884 (0.3%)
	2nd year	3/1166 (0.3%)	0/1206	3/2372 (0.1%)
	3rd year	3/ 963 (0.3%)	3/1012 (0.3%)	6/1975 (0.3%)
	Overall	13/1432 (0.9%)	6/1452 (0.4%)	19/2884 (0.7%)
Laceration	1st year	1/1432 (<0.1%)	2/1452 (0.1%)	3/2884 (0.1%)
	2nd year	1/1166 (<0.1%)	0/1206	1/2372 (<0.1%)
	3rd year	1/ 963 (0.1%)	0/1012	1/1975 (<0.1%)
	Overall	3/1432 (0.2%)	2/1452 (0.1%)	5/2884 (0.2%)
Laryngeal injury	1st year	1/1432 (<0.1%)	0/1452	1/2884 (<0.1%)
	Overall	1/1432 (<0.1%)	0/1452	1/2884 (<0.1%)

Table 14.3.1 / 9: Number of subjects with adverse events by primary system organ class, preferred term and year (FAS)

Primary system organ class / Preferred term / MedDRA version 14.0	YEAR OF ONSET	LCS12	LCS16	Total
Ligament rupture	1st year	2/1432 (0.1%)	1/1452 (<0.1%)	3/2884 (0.1%)
	3rd year	1/ 963 (0.1%)	0/1012	1/1975 (<0.1%)
	Overall	3/1432 (0.2%)	1/1452 (<0.1%)	4/2884 (0.1%)
Ligament sprain	2nd year	0/1166	1/1206 (<0.1%)	1/2372 (<0.1%)
	Overall	0/1432	1/1452 (<0.1%)	1/2884 (<0.1%)
Limb crushing injury	3rd year	1/ 963 (0.1%)	0/1012	1/1975 (<0.1%)
	Overall	1/1432 (<0.1%)	0/1452	1/2884 (<0.1%)
Limb injury	1st year	0/1432	3/1452 (0.2%)	3/2884 (0.1%)
	3rd year	1/ 963 (0.1%)	1/1012 (<0.1%)	2/1975 (0.1%)
	Overall	1/1432 (<0.1%)	4/1452 (0.3%)	5/2884 (0.2%)
Lower limb fracture	3rd year	1/ 963 (0.1%)	0/1012	1/1975 (<0.1%)
	Overall	1/1432 (<0.1%)	0/1452	1/2884 (<0.1%)
Lumbar vertebral fracture	3rd year	0/ 963	1/1012 (<0.1%)	1/1975 (<0.1%)
	Overall	0/1432	1/1452 (<0.1%)	1/2884 (<0.1%)
Meniscus lesion	1st year	1/1432 (<0.1%)	0/1452	1/2884 (<0.1%)
	2nd year	1/1166 (<0.1%)	0/1206	1/2372 (<0.1%)
	3rd year	1/ 963 (0.1%)	0/1012	1/1975 (<0.1%)
	Overall	3/1432 (0.2%)	0/1452	3/2884 (0.1%)
Muscle injury	1st year	3/1432 (0.2%)	1/1452 (<0.1%)	4/2884 (0.1%)
	2nd year	1/1166 (<0.1%)	1/1206 (<0.1%)	2/2372 (<0.1%)
	3rd year	1/ 963 (0.1%)	0/1012	1/1975 (<0.1%)
	Overall	5/1432 (0.3%)	2/1452 (0.1%)	7/2884 (0.2%)
Muscle rupture	2nd year	0/1166	1/1206 (<0.1%)	1/2372 (<0.1%)
	Overall	0/1432	1/1452 (<0.1%)	1/2884 (<0.1%)
Muscle strain	1st year	4/1432 (0.3%)	5/1452 (0.3%)	9/2884 (0.3%)
	2nd year	0/1166	1/1206 (<0.1%)	1/2372 (<0.1%)
	3rd year	3/ 963 (0.3%)	4/1012 (0.4%)	7/1975 (0.4%)
	Overall	7/1432 (0.5%)	10/1452 (0.7%)	17/2884 (0.6%)
Nail avulsion	3rd year	1/ 963 (0.1%)	0/1012	1/1975 (<0.1%)
	Overall	1/1432 (<0.1%)	0/1452	1/2884 (<0.1%)

Table 14.3.1 / 9: Number of subjects with adverse events by primary system organ class, preferred term and year (FAS)

Primary system organ class<br>Preferred term<br>MedDRA version 14.0	YEAR OF ONSET	LCS12	LCS16	Total
Overdose	3rd year	1/ 963 (0.1%)	0/1012	1/1975 (<0.1%)
	Overall	1/1432 (<0.1%)	0/1452	1/2884 (<0.1%)
Post concussion syndrome	2nd year	0/1166	1/1206 (<0.1%)	1/2372 (<0.1%)
	Overall	0/1432	1/1452 (<0.1%)	1/2884 (<0.1%)
Post procedural haemorrhage	1st year	1/1432 (<0.1%)	1/1452 (<0.1%)	2/2884 (<0.1%)
	2nd year	1/1166 (<0.1%)	0/1206	1/2372 (<0.1%)
	3rd year	0/ 963	1/1012 (<0.1%)	1/1975 (<0.1%)
	Overall	2/1432 (0.1%)	2/1452 (0.1%)	4/2884 (0.1%)
Post-traumatic pain	1st year	2/1432 (0.1%)	3/1452 (0.2%)	5/2884 (0.2%)
	2nd year	1/1166 (<0.1%)	1/1206 (<0.1%)	2/2372 (<0.1%)
	Overall	3/1432 (0.2%)	4/1452 (0.3%)	7/2884 (0.2%)
Postoperative ileus	3rd year	0/ 963	1/1012 (<0.1%)	1/1975 (<0.1%)
	Overall	0/1432	1/1452 (<0.1%)	1/2884 (<0.1%)
Procedural pain	1st year	48/1432 (3.4%)	43/1452 (3.0%)	91/2884 (3.2%)
	2nd year	8/1166 (0.7%)	6/1206 (0.5%)	14/2372 (0.6%)
	3rd year	4/ 963 (0.4%)	7/1012 (0.7%)	11/1975 (0.6%)
	Overall	58/1432 (4.1%)	54/1452 (3.7%)	112/2884 (3.9%)
Procedural vomiting	1st year	1/1432 (<0.1%)	0/1452	1/2884 (<0.1%)
	Overall	1/1432 (<0.1%)	0/1452	1/2884 (<0.1%)
Radius fracture	1st year	0/1432	1/1452 (<0.1%)	1/2884 (<0.1%)
	2nd year	1/1166 (<0.1%)	0/1206	1/2372 (<0.1%)
	Overall	1/1432 (<0.1%)	1/1452 (<0.1%)	2/2884 (<0.1%)
Rib fracture	1st year	1/1432 (<0.1%)	1/1452 (<0.1%)	2/2884 (<0.1%)
	3rd year	0/ 963	1/1012 (<0.1%)	1/1975 (<0.1%)
	Overall	1/1432 (<0.1%)	2/1452 (0.1%)	3/2884 (0.1%)
Road traffic accident	1st year	3/1432 (0.2%)	0/1452	3/2884 (0.1%)
	3rd year	0/ 963	2/1012 (0.2%)	2/1975 (0.1%)
	Overall	3/1432 (0.2%)	2/1452 (0.1%)	5/2884 (0.2%)
Seroma	3rd year	1/ 963 (0.1%)	0/1012	1/1975 (<0.1%)
	Overall	1/1432 (<0.1%)	0/1452	1/2884 (<0.1%)

Table 14.3.1 / 9: Number of subjects with adverse events by primary system organ class, preferred term and year (FAS)

Primary system organ class<br>Preferred term<br>MedDRA version 14.0	YEAR OF ONSET	LCS12	LCS16	Total
Shunt occlusion	1st year	1/1432 (<0.1%)	0/1452	1/2884 (<0.1%)
	Overall	1/1432 (<0.1%)	0/1452	1/2884 (<0.1%)
Skeletal injury	3rd year	0/ 963	1/1012 (<0.1%)	1/1975 (<0.1%)
	Overall	0/1432	1/1452 (<0.1%)	1/2884 (<0.1%)
Skin injury	2nd year	0/1166	1/1206 (<0.1%)	1/2372 (<0.1%)
	Overall	0/1432	1/1452 (<0.1%)	1/2884 (<0.1%)
Spinal column injury	1st year	0/1432	2/1452 (0.1%)	2/2884 (<0.1%)
	Overall	0/1432	2/1452 (0.1%)	2/2884 (<0.1%)
Stress fracture	2nd year	0/1166	1/1206 (<0.1%)	1/2372 (<0.1%)
	Overall	0/1432	1/1452 (<0.1%)	1/2884 (<0.1%)
Subdural haematoma	1st year	1/1432 (<0.1%)	0/1452	1/2884 (<0.1%)
	Overall	1/1432 (<0.1%)	0/1452	1/2884 (<0.1%)
Tendon injury	1st year	0/1432	1/1452 (<0.1%)	1/2884 (<0.1%)
	Overall	0/1432	1/1452 (<0.1%)	1/2884 (<0.1%)
Tendon rupture	1st year	1/1432 (<0.1%)	1/1452 (<0.1%)	2/2884 (<0.1%)
	3rd year	2/ 963 (0.2%)	0/1012	2/1975 (0.1%)
	Overall	3/1432 (0.2%)	1/1452 (<0.1%)	4/2884 (0.1%)
Thermal burn	2nd year	0/1166	1/1206 (<0.1%)	1/2372 (<0.1%)
	3rd year	0/ 963	2/1012 (0.2%)	2/1975 (0.1%)
	Overall	0/1432	3/1452 (0.2%)	3/2884 (0.1%)
Tibia fracture	2nd year	1/1166 (<0.1%)	1/1206 (<0.1%)	2/2372 (<0.1%)
	Overall	1/1432 (<0.1%)	1/1452 (<0.1%)	2/2884 (<0.1%)
Traumatic fracture	1st year	1/1432 (<0.1%)	0/1452	1/2884 (<0.1%)
	Overall	1/1432 (<0.1%)	0/1452	1/2884 (<0.1%)
Upper limb fracture	2nd year	1/1166 (<0.1%)	1/1206 (<0.1%)	2/2372 (<0.1%)
	3rd year	0/ 963	1/1012 (<0.1%)	1/1975 (<0.1%)
	Overall	1/1432 (<0.1%)	2/1452 (0.1%)	3/2884 (0.1%)

Table 14.3.1 / 9: Number of subjects with adverse events by primary system organ class, preferred term and year (FAS)

Primary system organ class<br>Preferred term<br>MedDRA version 14.0	YEAR OF ONSET	LCS12	LCS16	Total
Vulval laceration	3rd year	0/ 963	1/1012 (<0.1%)	1/1975 (<0.1%)
	Overall	0/1432	1/1452 (<0.1%)	1/2884 (<0.1%)
Whiplash injury	1st year	1/1432 (<0.1%)	2/1452 (0.1%)	3/2884 (0.1%)
	2nd year	0/1166	2/1206 (0.2%)	2/2372 (<0.1%)
	3rd year	0/ 963	1/1012 (<0.1%)	1/1975 (<0.1%)
	Overall	1/1432 (<0.1%)	5/1452 (0.3%)	6/2884 (0.2%)
Wound	1st year	1/1432 (<0.1%)	0/1452	1/2884 (<0.1%)
	Overall	1/1432 (<0.1%)	0/1452	1/2884 (<0.1%)
Wrist fracture	1st year	0/1432	1/1452 (<0.1%)	1/2884 (<0.1%)
	2nd year	0/1166	1/1206 (<0.1%)	1/2372 (<0.1%)
	Overall	0/1432	2/1452 (0.1%)	2/2884 (<0.1%)
Investigations	1st year	57/1432 (4.0%)	64/1452 (4.4%)	121/2884 (4.2%)
	2nd year	37/1166 (3.2%)	42/1206 (3.5%)	79/2372 (3.3%)
	3rd year	61/ 963 (6.3%)	60/1012 (5.9%)	121/1975 (6.1%)
	Overall	150/1432 (10.5%)	158/1452 (10.9%)	308/2884 (10.7%)
Alanine aminotransferase increased	1st year	1/1432 (<0.1%)	1/1452 (<0.1%)	2/2884 (<0.1%)
	2nd year	1/1166 (<0.1%)	1/1206 (<0.1%)	2/2372 (<0.1%)
	3rd year	6/ 963 (0.6%)	8/1012 (0.8%)	14/1975 (0.7%)
	Overall	9/1432 (0.6%)	10/1452 (0.7%)	19/2884 (0.7%)
Antibiotic resistant Staphylococcus test positive	1st year	1/1432 (<0.1%)	0/1452	1/2884 (<0.1%)
	3rd year	1/ 963 (0.1%)	0/1012	1/1975 (<0.1%)
	Overall	2/1432 (0.1%)	0/1452	2/2884 (<0.1%)
Aspartate aminotransferase increased	1st year	1/1432 (<0.1%)	0/1452	1/2884 (<0.1%)
	2nd year	0/1166	1/1206 (<0.1%)	1/2372 (<0.1%)
	3rd year	4/ 963 (0.4%)	3/1012 (0.3%)	7/1975 (0.4%)
	Overall	6/1432 (0.4%)	4/1452 (0.3%)	10/2884 (0.3%)
Biopsy muscle	1st year	1/1432 (<0.1%)	0/1452	1/2884 (<0.1%)
	Overall	1/1432 (<0.1%)	0/1452	1/2884 (<0.1%)
Blood alkaline phosphatase decreased	3rd year	1/ 963 (0.1%)	0/1012	1/1975 (<0.1%)
	Overall	1/1432 (<0.1%)	0/1452	1/2884 (<0.1%)

Table 14.3.1 / 9: Number of subjects with adverse events by primary system organ class, preferred term and year (FAS)

Primary system organ class<br>Preferred term<br>MedDRA version 14.0	YEAR OF ONSET	LCS12	LCS16	Total
Blood amylase increased	1st year	0/1432	1/1452 (<0.1%)	1/2884 (<0.1%)
	Overall	0/1432	1/1452 (<0.1%)	1/2884 (<0.1%)
Blood cholesterol increased	1st year	3/1432 (0.2%)	0/1452	3/2884 (0.1%)
	3rd year	0/ 963	1/1012 (<0.1%)	1/1975 (<0.1%)
	Overall	3/1432 (0.2%)	1/1452 (<0.1%)	4/2884 (0.1%)
Blood iron decreased	1st year	0/1432	1/1452 (<0.1%)	1/2884 (<0.1%)
	Overall	0/1432	1/1452 (<0.1%)	1/2884 (<0.1%)
Blood potassium abnormal	3rd year	1/ 963 (0.1%)	0/1012	1/1975 (<0.1%)
	Overall	1/1432 (<0.1%)	0/1452	1/2884 (<0.1%)
Blood potassium increased	1st year	0/1432	1/1452 (<0.1%)	1/2884 (<0.1%)
	Overall	0/1432	1/1452 (<0.1%)	1/2884 (<0.1%)
Blood pressure diastolic increased	1st year	0/1432	1/1452 (<0.1%)	1/2884 (<0.1%)
	Overall	0/1432	1/1452 (<0.1%)	1/2884 (<0.1%)
Blood pressure increased	2nd year	2/1166 (0.2%)	0/1206	2/2372 (<0.1%)
	3rd year	1/ 963 (0.1%)	1/1012 (<0.1%)	2/1975 (0.1%)
	Overall	3/1432 (0.2%)	1/1452 (<0.1%)	4/2884 (0.1%)
Blood pressure systolic increased	2nd year	0/1166	1/1206 (<0.1%)	1/2372 (<0.1%)
	Overall	0/1432	1/1452 (<0.1%)	1/2884 (<0.1%)
Blood sodium decreased	3rd year	1/ 963 (0.1%)	0/1012	1/1975 (<0.1%)
	Overall	1/1432 (<0.1%)	0/1452	1/2884 (<0.1%)
Blood triglycerides increased	1st year	1/1432 (<0.1%)	1/1452 (<0.1%)	2/2884 (<0.1%)
	3rd year	0/ 963	4/1012 (0.4%)	4/1975 (0.2%)
	Overall	1/1432 (<0.1%)	5/1452 (0.3%)	6/2884 (0.2%)
Blood urine present	3rd year	3/ 963 (0.3%)	1/1012 (<0.1%)	4/1975 (0.2%)
	Overall	3/1432 (0.2%)	1/1452 (<0.1%)	4/2884 (0.1%)
Carbohydrate antigen 125 increased	3rd year	1/ 963 (0.1%)	0/1012	1/1975 (<0.1%)
	Overall	1/1432 (<0.1%)	0/1452	1/2884 (<0.1%)

Table 14.3.1 / 9: Number of subjects with adverse events by primary system organ class, preferred term and year (FAS)

Primary system organ class / Preferred term / MedDRA version 14.0	YEAR OF ONSET	LCS12	LCS16	Total
Cardiac murmur	2nd year	1/1166 (<0.1%)	0/1206	1/2372 (<0.1%)
	Overall	1/1432 (<0.1%)	0/1452	1/2884 (<0.1%)
Chlamydia test positive	1st year	2/1432 (0.1%)	0/1452	2/2884 (<0.1%)
	2nd year	2/1166 (0.2%)	1/1206 (<0.1%)	3/2372 (0.1%)
	3rd year	3/ 963 (0.3%)	0/1012	3/1975 (0.2%)
	Overall	7/1432 (0.5%)	1/1452 (<0.1%)	8/2884 (0.3%)
Escherichia test positive	3rd year	1/ 963 (0.1%)	0/1012	1/1975 (<0.1%)
	Overall	1/1432 (<0.1%)	0/1452	1/2884 (<0.1%)
Gamma-glutamyltransferase increased	1st year	0/1432	2/1452 (0.1%)	2/2884 (<0.1%)
	2nd year	0/1166	1/1206 (<0.1%)	1/2372 (<0.1%)
	3rd year	4/ 963 (0.4%)	10/1012 (1.0%)	14/1975 (0.7%)
	Overall	4/1432 (0.3%)	13/1452 (0.9%)	17/2884 (0.6%)
Gastric pH decreased	2nd year	0/1166	1/1206 (<0.1%)	1/2372 (<0.1%)
	Overall	0/1432	1/1452 (<0.1%)	1/2884 (<0.1%)
Glucose urine present	3rd year	0/ 963	1/1012 (<0.1%)	1/1975 (<0.1%)
	Overall	0/1432	1/1452 (<0.1%)	1/2884 (<0.1%)
Glycosylated haemoglobin increased	2nd year	1/1166 (<0.1%)	0/1206	1/2372 (<0.1%)
	3rd year	4/ 963 (0.4%)	0/1012	4/1975 (0.2%)
	Overall	5/1432 (0.3%)	0/1452	5/2884 (0.2%)
Haematocrit decreased	1st year	1/1432 (<0.1%)	0/1452	1/2884 (<0.1%)
	2nd year	0/1166	1/1206 (<0.1%)	1/2372 (<0.1%)
	Overall	1/1432 (<0.1%)	1/1452 (<0.1%)	2/2884 (<0.1%)
Haematocrit increased	2nd year	1/1166 (<0.1%)	0/1206	1/2372 (<0.1%)
	3rd year	1/ 963 (0.1%)	2/1012 (0.2%)	3/1975 (0.2%)
	Overall	2/1432 (0.1%)	2/1452 (0.1%)	4/2884 (0.1%)
Haemoglobin decreased	1st year	2/1432 (0.1%)	0/1452	2/2884 (<0.1%)
	2nd year	1/1166 (<0.1%)	2/1206 (0.2%)	3/2372 (0.1%)
	3rd year	2/ 963 (0.2%)	3/1012 (0.3%)	5/1975 (0.3%)
	Overall	5/1432 (0.3%)	5/1452 (0.3%)	10/2884 (0.3%)

Table 14.3.1 / 9: Number of subjects with adverse events by primary system organ class, preferred term and year (FAS)

Primary system organ class<br>Preferred term<br>MedDRA version 14.0	YEAR OF ONSET	LCS12	LCS16	Total
Heart rate increased	1st year	0/1432	1/1452 (<0.1%)	1/2884 (<0.1%)
	Overall	0/1432	1/1452 (<0.1%)	1/2884 (<0.1%)
Hepatic enzyme increased	1st year	1/1432 (<0.1%)	1/1452 (<0.1%)	2/2884 (<0.1%)
	Overall	1/1432 (<0.1%)	1/1452 (<0.1%)	2/2884 (<0.1%)
High density lipoprotein decreased	1st year	0/1432	1/1452 (<0.1%)	1/2884 (<0.1%)
	3rd year	3/ 963 (0.3%)	5/1012 (0.5%)	8/1975 (0.4%)
	Overall	3/1432 (0.2%)	6/1452 (0.4%)	9/2884 (0.3%)
High density lipoprotein increased	3rd year	0/ 963	1/1012 (<0.1%)	1/1975 (<0.1%)
	Overall	0/1432	1/1452 (<0.1%)	1/2884 (<0.1%)
Human papilloma virus test positive	1st year	1/1432 (<0.1%)	2/1452 (0.1%)	3/2884 (0.1%)
	2nd year	3/1166 (0.3%)	1/1206 (<0.1%)	4/2372 (0.2%)
	3rd year	1/ 963 (0.1%)	2/1012 (0.2%)	3/1975 (0.2%)
	Overall	5/1432 (0.3%)	5/1452 (0.3%)	10/2884 (0.3%)
Liver function test abnormal	1st year	0/1432	1/1452 (<0.1%)	1/2884 (<0.1%)
	3rd year	0/ 963	1/1012 (<0.1%)	1/1975 (<0.1%)
	Overall	0/1432	2/1452 (0.1%)	2/2884 (<0.1%)
Low density lipoprotein increased	1st year	1/1432 (<0.1%)	1/1452 (<0.1%)	2/2884 (<0.1%)
	3rd year	0/ 963	1/1012 (<0.1%)	1/1975 (<0.1%)
	Overall	1/1432 (<0.1%)	2/1452 (0.1%)	3/2884 (0.1%)
Neisseria test positive	1st year	1/1432 (<0.1%)	0/1452	1/2884 (<0.1%)
	2nd year	1/1166 (<0.1%)	0/1206	1/2372 (<0.1%)
	Overall	2/1432 (0.1%)	0/1452	2/2884 (<0.1%)
Platelet count decreased	3rd year	2/ 963 (0.2%)	3/1012 (0.3%)	5/1975 (0.3%)
	Overall	2/1432 (0.1%)	3/1452 (0.2%)	5/2884 (0.2%)
Protein total decreased	3rd year	0/ 963	1/1012 (<0.1%)	1/1975 (<0.1%)
	Overall	0/1432	1/1452 (<0.1%)	1/2884 (<0.1%)
Protein urine present	3rd year	1/ 963 (0.1%)	1/1012 (<0.1%)	2/1975 (0.1%)
	Overall	1/1432 (<0.1%)	1/1452 (<0.1%)	2/2884 (<0.1%)

Table 14.3.1 / 9: Number of subjects with adverse events by primary system organ class, preferred term and year (FAS)

Primary system organ class<br>Preferred term<br>MedDRA version 14.0	YEAR OF<br>ONSET	LCS12	LCS16	Total
Red blood cell count decreased	2nd year	0/1166	1/1206 (<0.1%)	1/2372 (<0.1%)
	3rd year	0/ 963	1/1012 (<0.1%)	1/1975 (<0.1%)
	Overall	0/1432	2/1452 (0.1%)	2/2884 (<0.1%)
Red blood cell count increased	2nd year	1/1166 (<0.1%)	0/1206	1/2372 (<0.1%)
	Overall	1/1432 (<0.1%)	0/1452	1/2884 (<0.1%)
Simplex virus test positive	1st year	0/1432	1/1452 (<0.1%)	1/2884 (<0.1%)
	2nd year	1/1166 (<0.1%)	0/1206	1/2372 (<0.1%)
	3rd year	1/ 963 (0.1%)	0/1012	1/1975 (<0.1%)
	Overall	2/1432 (0.1%)	1/1452 (<0.1%)	3/2884 (0.1%)
Smear cervix abnormal	1st year	2/1432 (0.1%)	7/1452 (0.5%)	9/2884 (0.3%)
	2nd year	9/1166 (0.8%)	9/1206 (0.7%)	18/2372 (0.8%)
	3rd year	7/ 963 (0.7%)	12/1012 (1.2%)	19/1975 (1.0%)
	Overall	18/1432 (1.3%)	28/1452 (1.9%)	46/2884 (1.6%)
Streptococcus test positive	2nd year	1/1166 (<0.1%)	0/1206	1/2372 (<0.1%)
	Overall	1/1432 (<0.1%)	0/1452	1/2884 (<0.1%)
Transaminases increased	2nd year	0/1166	1/1206 (<0.1%)	1/2372 (<0.1%)
	Overall	0/1432	1/1452 (<0.1%)	1/2884 (<0.1%)
Ultrasound ovary abnormal	2nd year	1/1166 (<0.1%)	0/1206	1/2372 (<0.1%)
	Overall	1/1432 (<0.1%)	0/1452	1/2884 (<0.1%)
Urine leukocyte esterase positive	1st year	0/1432	1/1452 (<0.1%)	1/2884 (<0.1%)
	Overall	0/1432	1/1452 (<0.1%)	1/2884 (<0.1%)
Vitamin D decreased	3rd year	0/ 963	1/1012 (<0.1%)	1/1975 (<0.1%)
	Overall	0/1432	1/1452 (<0.1%)	1/2884 (<0.1%)
Weight decreased	1st year	2/1432 (0.1%)	3/1452 (0.2%)	5/2884 (0.2%)
	2nd year	3/1166 (0.3%)	3/1206 (0.2%)	6/2372 (0.3%)
	3rd year	4/ 963 (0.4%)	1/1012 (<0.1%)	5/1975 (0.3%)
	Overall	9/1432 (0.6%)	7/1452 (0.5%)	16/2884 (0.6%)

Table 14.3.1 / 9: Number of subjects with adverse events by primary system organ class, preferred term and year (FAS)

Primary system organ class<br>Preferred term<br>MedDRA version 14.0	YEAR OF ONSET	LCS12	LCS16	Total
Weight increased	1st year	39/1432 (2.7%)	41/1452 (2.8%)	80/2884 (2.8%)
	2nd year	11/1166 (0.9%)	22/1206 (1.8%)	33/2372 (1.4%)
	3rd year	7/ 963 (0.7%)	5/1012 (0.5%)	12/1975 (0.6%)
	Overall	56/1432 (3.9%)	68/1452 (4.7%)	124/2884 (4.3%)
White blood cell count decreased	3rd year	4/ 963 (0.4%)	2/1012 (0.2%)	6/1975 (0.3%)
	Overall	4/1432 (0.3%)	2/1452 (0.1%)	6/2884 (0.2%)
White blood cell count increased	2nd year	1/1166 (<0.1%)	0/1206	1/2372 (<0.1%)
	3rd year	4/ 963 (0.4%)	2/1012 (0.2%)	6/1975 (0.3%)
	Overall	5/1432 (0.3%)	2/1452 (0.1%)	7/2884 (0.2%)
White blood cells urine positive	2nd year	1/1166 (<0.1%)	0/1206	1/2372 (<0.1%)
	3rd year	2/ 963 (0.2%)	2/1012 (0.2%)	4/1975 (0.2%)
	Overall	3/1432 (0.2%)	2/1452 (0.1%)	5/2884 (0.2%)
Metabolism and nutrition disorders	1st year	11/1432 (0.8%)	11/1452 (0.8%)	22/2884 (0.8%)
	2nd year	2/1166 (0.2%)	4/1206 (0.3%)	6/2372 (0.3%)
	3rd year	5/ 963 (0.5%)	3/1012 (0.3%)	8/1975 (0.4%)
	Overall	18/1432 (1.3%)	17/1452 (1.2%)	35/2884 (1.2%)
Abnormal loss of weight	1st year	0/1432	1/1452 (<0.1%)	1/2884 (<0.1%)
	Overall	0/1432	1/1452 (<0.1%)	1/2884 (<0.1%)
Abnormal weight gain	1st year	0/1432	1/1452 (<0.1%)	1/2884 (<0.1%)
	Overall	0/1432	1/1452 (<0.1%)	1/2884 (<0.1%)
Cell death	2nd year	1/1166 (<0.1%)	0/1206	1/2372 (<0.1%)
	Overall	1/1432 (<0.1%)	0/1452	1/2884 (<0.1%)
Cholesterosis	1st year	1/1432 (<0.1%)	0/1452	1/2884 (<0.1%)
	Overall	1/1432 (<0.1%)	0/1452	1/2884 (<0.1%)
Decreased appetite	1st year	0/1432	1/1452 (<0.1%)	1/2884 (<0.1%)
	3rd year	2/ 963 (0.2%)	0/1012	2/1975 (0.1%)
	Overall	2/1432 (0.1%)	1/1452 (<0.1%)	3/2884 (0.1%)
Dehydration	1st year	0/1432	1/1452 (<0.1%)	1/2884 (<0.1%)
	3rd year	0/ 963	1/1012 (<0.1%)	1/1975 (<0.1%)
	Overall	0/1432	2/1452 (0.1%)	2/2884 (<0.1%)

Table 14.3.1 / 9: Number of subjects with adverse events by primary system organ class, preferred term and year (FAS)

Primary system organ class<br>Preferred term<br>MedDRA version 14.0	YEAR OF ONSET	LCS12	LCS16	Total
Diabetic ketoacidosis	2nd year	0/1166	1/1206 (<0.1%)	1/2372 (<0.1%)
	Overall	0/1432	1/1452 (<0.1%)	1/2884 (<0.1%)
Dyslipidaemia	3rd year	0/ 963	1/1012 (<0.1%)	1/1975 (<0.1%)
	Overall	0/1432	1/1452 (<0.1%)	1/2884 (<0.1%)
Fluid retention	1st year	3/1432 (0.2%)	2/1452 (0.1%)	5/2884 (0.2%)
	Overall	3/1432 (0.2%)	2/1452 (0.1%)	5/2884 (0.2%)
Glucose tolerance impaired	3rd year	1/ 963 (0.1%)	0/1012	1/1975 (<0.1%)
	Overall	1/1432 (<0.1%)	0/1452	1/2884 (<0.1%)
Hypokalaemia	1st year	1/1432 (<0.1%)	0/1452	1/2884 (<0.1%)
	3rd year	1/ 963 (0.1%)	0/1012	1/1975 (<0.1%)
	Overall	2/1432 (0.1%)	0/1452	2/2884 (<0.1%)
Hyponatraemia	1st year	1/1432 (<0.1%)	0/1452	1/2884 (<0.1%)
	Overall	1/1432 (<0.1%)	0/1452	1/2884 (<0.1%)
Increased appetite	1st year	1/1432 (<0.1%)	2/1452 (0.1%)	3/2884 (0.1%)
	Overall	1/1432 (<0.1%)	2/1452 (0.1%)	3/2884 (0.1%)
Insulin resistance	1st year	2/1432 (0.1%)	0/1452	2/2884 (<0.1%)
	2nd year	0/1166	1/1206 (<0.1%)	1/2372 (<0.1%)
	Overall	2/1432 (0.1%)	1/1452 (<0.1%)	3/2884 (0.1%)
Lactose intolerance	1st year	2/1432 (0.1%)	0/1452	2/2884 (<0.1%)
	Overall	2/1432 (0.1%)	0/1452	2/2884 (<0.1%)
Obesity	1st year	0/1432	1/1452 (<0.1%)	1/2884 (<0.1%)
	Overall	0/1432	1/1452 (<0.1%)	1/2884 (<0.1%)
Overweight	1st year	0/1432	1/1452 (<0.1%)	1/2884 (<0.1%)
	Overall	0/1432	1/1452 (<0.1%)	1/2884 (<0.1%)
Type 2 diabetes mellitus	2nd year	1/1166 (<0.1%)	0/1206	1/2372 (<0.1%)
	Overall	1/1432 (<0.1%)	0/1452	1/2884 (<0.1%)

Table 14.3.1 / 9: Number of subjects with adverse events by primary system organ class, preferred term and year (FAS)

Primary system organ class<br>Preferred term<br>MedDRA version 14.0	YEAR OF ONSET	LCS12	LCS16	Total
Vitamin B12 deficiency	1st year	1/1432 (<0.1%)	0/1452	1/2884 (<0.1%)
	2nd year	0/1166	1/1206 (<0.1%)	1/2372 (<0.1%)
	Overall	1/1432 (<0.1%)	1/1452 (<0.1%)	2/2884 (<0.1%)
Vitamin D deficiency	2nd year	0/1166	1/1206 (<0.1%)	1/2372 (<0.1%)
	3rd year	1/ 963 (0.1%)	1/1012 (<0.1%)	2/1975 (0.1%)
	Overall	1/1432 (<0.1%)	2/1452 (0.1%)	3/2884 (0.1%)
Weight fluctuation	1st year	0/1432	1/1452 (<0.1%)	1/2884 (<0.1%)
	Overall	0/1432	1/1452 (<0.1%)	1/2884 (<0.1%)
Musculoskeletal and connective tissue disorders	1st year	89/1432 (6.2%)	109/1452 (7.5%)	198/2884 (6.9%)
	2nd year	37/1166 (3.2%)	50/1206 (4.1%)	87/2372 (3.7%)
	3rd year	33/ 963 (3.4%)	55/1012 (5.4%)	88/1975 (4.5%)
	Overall	144/1432 (10.1%)	187/1452 (12.9%)	331/2884 (11.5%)
Ankylosing spondylitis	1st year	1/1432 (<0.1%)	0/1452	1/2884 (<0.1%)
	Overall	1/1432 (<0.1%)	0/1452	1/2884 (<0.1%)
Arthralgia	1st year	15/1432 (1.0%)	19/1452 (1.3%)	34/2884 (1.2%)
	2nd year	4/1166 (0.3%)	8/1206 (0.7%)	12/2372 (0.5%)
	3rd year	3/ 963 (0.3%)	5/1012 (0.5%)	8/1975 (0.4%)
	Overall	21/1432 (1.5%)	31/1452 (2.1%)	52/2884 (1.8%)
Arthritis	1st year	0/1432	1/1452 (<0.1%)	1/2884 (<0.1%)
	2nd year	1/1166 (<0.1%)	2/1206 (0.2%)	3/2372 (0.1%)
	3rd year	0/ 963	2/1012 (0.2%)	2/1975 (0.1%)
	Overall	1/1432 (<0.1%)	5/1452 (0.3%)	6/2884 (0.2%)
Axillary mass	2nd year	0/1166	1/1206 (<0.1%)	1/2372 (<0.1%)
	Overall	0/1432	1/1452 (<0.1%)	1/2884 (<0.1%)
Back pain	1st year	32/1432 (2.2%)	41/1452 (2.8%)	73/2884 (2.5%)
	2nd year	11/1166 (0.9%)	15/1206 (1.2%)	26/2372 (1.1%)
	3rd year	18/ 963 (1.9%)	14/1012 (1.4%)	32/1975 (1.6%)
	Overall	59/1432 (4.1%)	65/1452 (4.5%)	124/2884 (4.3%)
Bone pain	1st year	1/1432 (<0.1%)	1/1452 (<0.1%)	2/2884 (<0.1%)
	3rd year	0/ 963	2/1012 (0.2%)	2/1975 (0.1%)
	Overall	1/1432 (<0.1%)	3/1452 (0.2%)	4/2884 (0.1%)

Table 14.3.1 / 9: Number of subjects with adverse events by primary system organ class, preferred term and year (FAS)

Primary system organ class<br>Preferred term<br>MedDRA version 14.0	YEAR OF ONSET	LCS12	LCS16	Total
Bursitis	1st year	1/1432 (<0.1%)	1/1452 (<0.1%)	2/2884 (<0.1%)
	2nd year	1/1166 (<0.1%)	0/1206	1/2372 (<0.1%)
	Overall	2/1432 (0.1%)	1/1452 (<0.1%)	3/2884 (0.1%)
Compartment syndrome	2nd year	0/1166	1/1206 (<0.1%)	1/2372 (<0.1%)
	Overall	0/1432	1/1452 (<0.1%)	1/2884 (<0.1%)
Costochondritis	1st year	1/1432 (<0.1%)	0/1452	1/2884 (<0.1%)
	2nd year	2/1166 (0.2%)	0/1206	2/2372 (<0.1%)
	3rd year	0/ 963	1/1012 (<0.1%)	1/1975 (<0.1%)
	Overall	3/1432 (0.2%)	1/1452 (<0.1%)	4/2884 (0.1%)
Exostosis	1st year	1/1432 (<0.1%)	1/1452 (<0.1%)	2/2884 (<0.1%)
	2nd year	0/1166	2/1206 (0.2%)	2/2372 (<0.1%)
	Overall	1/1432 (<0.1%)	3/1452 (0.2%)	4/2884 (0.1%)
Fibromyalgia	1st year	1/1432 (<0.1%)	0/1452	1/2884 (<0.1%)
	2nd year	0/1166	1/1206 (<0.1%)	1/2372 (<0.1%)
	3rd year	0/ 963	1/1012 (<0.1%)	1/1975 (<0.1%)
	Overall	1/1432 (<0.1%)	2/1452 (0.1%)	3/2884 (0.1%)
Flank pain	1st year	1/1432 (<0.1%)	2/1452 (0.1%)	3/2884 (0.1%)
	2nd year	1/1166 (<0.1%)	0/1206	1/2372 (<0.1%)
	3rd year	0/ 963	1/1012 (<0.1%)	1/1975 (<0.1%)
	Overall	2/1432 (0.1%)	3/1452 (0.2%)	5/2884 (0.2%)
Groin pain	1st year	2/1432 (0.1%)	2/1452 (0.1%)	4/2884 (0.1%)
	3rd year	0/ 963	2/1012 (0.2%)	2/1975 (0.1%)
	Overall	2/1432 (0.1%)	4/1452 (0.3%)	6/2884 (0.2%)
Hand deformity	1st year	0/1432	1/1452 (<0.1%)	1/2884 (<0.1%)
	Overall	0/1432	1/1452 (<0.1%)	1/2884 (<0.1%)
Intervertebral disc disorder	1st year	1/1432 (<0.1%)	1/1452 (<0.1%)	2/2884 (<0.1%)
	Overall	1/1432 (<0.1%)	1/1452 (<0.1%)	2/2884 (<0.1%)
Intervertebral disc protrusion	2nd year	0/1166	2/1206 (0.2%)	2/2372 (<0.1%)
	3rd year	1/ 963 (0.1%)	1/1012 (<0.1%)	2/1975 (0.1%)
	Overall	1/1432 (<0.1%)	3/1452 (0.2%)	4/2884 (0.1%)

Table 14.3.1 / 9: Number of subjects with adverse events by primary system organ class, preferred term and year (FAS)

Primary system organ class / Preferred term / MedDRA version 14.0	YEAR OF ONSET	LCS12	LCS16	Total
Joint effusion	1st year	1/1432 (<0.1%)	0/1452	1/2884 (<0.1%)
	Overall	1/1432 (<0.1%)	0/1452	1/2884 (<0.1%)
Joint instability	1st year	1/1432 (<0.1%)	0/1452	1/2884 (<0.1%)
	2nd year	0/1166	1/1206 (<0.1%)	1/2372 (<0.1%)
	3rd year	0/ 963	1/1012 (<0.1%)	1/1975 (<0.1%)
	Overall	1/1432 (<0.1%)	1/1452 (<0.1%)	2/2884 (<0.1%)
Joint stiffness	2nd year	0/1166	1/1206 (<0.1%)	1/2372 (<0.1%)
	Overall	0/1432	1/1452 (<0.1%)	1/2884 (<0.1%)
Joint swelling	1st year	0/1432	1/1452 (<0.1%)	1/2884 (<0.1%)
	3rd year	1/ 963 (0.1%)	0/1012	1/1975 (<0.1%)
	Overall	1/1432 (<0.1%)	1/1452 (<0.1%)	2/2884 (<0.1%)
Loose body in joint	2nd year	0/1166	1/1206 (<0.1%)	1/2372 (<0.1%)
	Overall	0/1432	1/1452 (<0.1%)	1/2884 (<0.1%)
Medial tibial stress syndrome	1st year	1/1432 (<0.1%)	0/1452	1/2884 (<0.1%)
	2nd year	1/1166 (<0.1%)	1/1206 (<0.1%)	2/2372 (<0.1%)
	3rd year	0/ 963	1/1012 (<0.1%)	1/1975 (<0.1%)
	Overall	2/1432 (0.1%)	2/1452 (0.1%)	4/2884 (0.1%)
Muscle contracture	1st year	0/1432	1/1452 (<0.1%)	1/2884 (<0.1%)
	2nd year	0/1166	1/1206 (<0.1%)	1/2372 (<0.1%)
	3rd year	1/ 963 (0.1%)	0/1012	1/1975 (<0.1%)
	Overall	1/1432 (<0.1%)	2/1452 (0.1%)	3/2884 (0.1%)
Muscle spasms	1st year	3/1432 (0.2%)	6/1452 (0.4%)	9/2884 (0.3%)
	2nd year	2/1166 (0.2%)	0/1206	2/2372 (<0.1%)
	3rd year	0/ 963	2/1012 (0.2%)	2/1975 (0.1%)
	Overall	5/1432 (0.3%)	8/1452 (0.6%)	13/2884 (0.5%)
Muscle tightness	1st year	4/1432 (0.3%)	4/1452 (0.3%)	8/2884 (0.3%)
	2nd year	3/1166 (0.3%)	1/1206 (<0.1%)	4/2372 (0.2%)
	3rd year	1/ 963 (0.1%)	6/1012 (0.6%)	7/1975 (0.4%)
	Overall	8/1432 (0.6%)	10/1452 (0.7%)	18/2884 (0.6%)

Table 14.3.1 / 9: Number of subjects with adverse events by primary system organ class, preferred term and year (FAS)

Primary system organ class<br>Preferred term<br>MedDRA version 14.0	YEAR OF ONSET	LCS12	LCS16	Total
Musculoskeletal chest pain	1st year	0/1432	2/1452 (0.1%)	2/2884 (<0.1%)
	3rd year	2/ 963 (0.2%)	0/1012	2/1975 (0.1%)
	Overall	2/1432 (0.1%)	2/1452 (0.1%)	4/2884 (0.1%)
Musculoskeletal discomfort	2nd year	0/1166	1/1206 (<0.1%)	1/2372 (<0.1%)
	Overall	0/1432	1/1452 (<0.1%)	1/2884 (<0.1%)
Musculoskeletal pain	1st year	6/1432 (0.4%)	7/1452 (0.5%)	13/2884 (0.5%)
	2nd year	1/1166 (<0.1%)	4/1206 (0.3%)	5/2372 (0.2%)
	3rd year	2/ 963 (0.2%)	1/1012 (<0.1%)	3/1975 (0.2%)
	Overall	9/1432 (0.6%)	11/1452 (0.8%)	20/2884 (0.7%)
Musculoskeletal stiffness	2nd year	0/1166	1/1206 (<0.1%)	1/2372 (<0.1%)
	Overall	0/1432	1/1452 (<0.1%)	1/2884 (<0.1%)
Myalgia	1st year	7/1432 (0.5%)	8/1452 (0.6%)	15/2884 (0.5%)
	2nd year	2/1166 (0.2%)	2/1206 (0.2%)	4/2372 (0.2%)
	3rd year	1/ 963 (0.1%)	2/1012 (0.2%)	3/1975 (0.2%)
	Overall	9/1432 (0.6%)	12/1452 (0.8%)	21/2884 (0.7%)
Myositis	1st year	0/1432	1/1452 (<0.1%)	1/2884 (<0.1%)
	Overall	0/1432	1/1452 (<0.1%)	1/2884 (<0.1%)
Neck pain	1st year	3/1432 (0.2%)	6/1452 (0.4%)	9/2884 (0.3%)
	2nd year	5/1166 (0.4%)	2/1206 (0.2%)	7/2372 (0.3%)
	3rd year	4/ 963 (0.4%)	2/1012 (0.2%)	6/1975 (0.3%)
	Overall	12/1432 (0.8%)	10/1452 (0.7%)	22/2884 (0.8%)
Osteitis	1st year	1/1432 (<0.1%)	0/1452	1/2884 (<0.1%)
	2nd year	1/1166 (<0.1%)	0/1206	1/2372 (<0.1%)
	Overall	2/1432 (0.1%)	0/1452	2/2884 (<0.1%)
Osteoarthritis	1st year	0/1432	2/1452 (0.1%)	2/2884 (<0.1%)
	Overall	0/1432	2/1452 (0.1%)	2/2884 (<0.1%)
Osteochondrosis	3rd year	0/ 963	2/1012 (0.2%)	2/1975 (0.1%)
	Overall	0/1432	2/1452 (0.1%)	2/2884 (<0.1%)
Osteopenia	2nd year	0/1166	1/1206 (<0.1%)	1/2372 (<0.1%)
	Overall	0/1432	1/1452 (<0.1%)	1/2884 (<0.1%)

Table 14.3.1 / 9: Number of subjects with adverse events by primary system organ class, preferred term and year (FAS)

Primary system organ class<br>Preferred term<br>MedDRA version 14.0	YEAR OF ONSET	LCS12	LCS16	Total
Pain in extremity	1st year	4/1432 (0.3%)	8/1452 (0.6%)	12/2884 (0.4%)
	2nd year	1/1166 (<0.1%)	3/1206 (0.2%)	4/2372 (0.2%)
	3rd year	1/ 963 (0.1%)	4/1012 (0.4%)	5/1975 (0.3%)
	Overall	6/1432 (0.4%)	15/1452 (1.0%)	21/2884 (0.7%)
Pain in jaw	3rd year	0/ 963	1/1012 (<0.1%)	1/1975 (<0.1%)
	Overall	0/1432	1/1452 (<0.1%)	1/2884 (<0.1%)
Patellofemoral pain syndrome	1st year	1/1432 (<0.1%)	0/1452	1/2884 (<0.1%)
	Overall	1/1432 (<0.1%)	0/1452	1/2884 (<0.1%)
Plantar fasciitis	1st year	0/1432	1/1452 (<0.1%)	1/2884 (<0.1%)
	Overall	0/1432	1/1452 (<0.1%)	1/2884 (<0.1%)
Psoriatic arthropathy	3rd year	1/ 963 (0.1%)	0/1012	1/1975 (<0.1%)
	Overall	1/1432 (<0.1%)	0/1452	1/2884 (<0.1%)
Rotator cuff syndrome	1st year	1/1432 (<0.1%)	1/1452 (<0.1%)	2/2884 (<0.1%)
	2nd year	0/1166	3/1206 (0.2%)	3/2372 (0.1%)
	3rd year	0/ 963	2/1012 (0.2%)	2/1975 (0.1%)
	Overall	1/1432 (<0.1%)	6/1452 (0.4%)	7/2884 (0.2%)
Scleroderma	2nd year	1/1166 (<0.1%)	0/1206	1/2372 (<0.1%)
	Overall	1/1432 (<0.1%)	0/1452	1/2884 (<0.1%)
Sensation of heaviness	3rd year	0/ 963	1/1012 (<0.1%)	1/1975 (<0.1%)
	Overall	0/1432	1/1452 (<0.1%)	1/2884 (<0.1%)
Spinal osteoarthritis	2nd year	1/1166 (<0.1%)	0/1206	1/2372 (<0.1%)
	Overall	1/1432 (<0.1%)	0/1452	1/2884 (<0.1%)
Synovitis	1st year	1/1432 (<0.1%)	0/1452	1/2884 (<0.1%)
	Overall	1/1432 (<0.1%)	0/1452	1/2884 (<0.1%)
Temporomandibular joint syndrome	1st year	1/1432 (<0.1%)	0/1452	1/2884 (<0.1%)
	3rd year	0/ 963	1/1012 (<0.1%)	1/1975 (<0.1%)
	Overall	1/1432 (<0.1%)	1/1452 (<0.1%)	2/2884 (<0.1%)

Table 14.3.1 / 9: Number of subjects with adverse events by primary system organ class, preferred term and year (FAS)

Primary system organ class<br>Preferred term<br>MedDRA version 14.0	YEAR OF ONSET	LCS12	LCS16	Total
Tendon pain	1st year	0/1432	1/1452 (<0.1%)	1/2884 (<0.1%)
	2nd year	1/1166 (<0.1%)	0/1206	1/2372 (<0.1%)
	Overall	1/1432 (<0.1%)	1/1452 (<0.1%)	2/2884 (<0.1%)
Tendonitis	1st year	3/1432 (0.2%)	4/1452 (0.3%)	7/2884 (0.2%)
	2nd year	2/1166 (0.2%)	0/1206	2/2372 (<0.1%)
	3rd year	0/ 963	1/1012 (<0.1%)	1/1975 (<0.1%)
	Overall	5/1432 (0.3%)	5/1452 (0.3%)	10/2884 (0.3%)
Tenosynovitis	1st year	0/1432	1/1452 (<0.1%)	1/2884 (<0.1%)
	3rd year	0/ 963	1/1012 (<0.1%)	1/1975 (<0.1%)
	Overall	0/1432	1/1452 (<0.1%)	1/2884 (<0.1%)
Torticollis	1st year	1/1432 (<0.1%)	0/1452	1/2884 (<0.1%)
	2nd year	0/1166	1/1206 (<0.1%)	1/2372 (<0.1%)
	3rd year	0/ 963	1/1012 (<0.1%)	1/1975 (<0.1%)
	Overall	1/1432 (<0.1%)	2/1452 (0.1%)	3/2884 (0.1%)
Neoplasms benign, malignant and unspecified (incl cysts and polyps)	1st year	20/1432 (1.4%)	17/1452 (1.2%)	37/2884 (1.3%)
	2nd year	14/1166 (1.2%)	18/1206 (1.5%)	32/2372 (1.3%)
	3rd year	14/ 963 (1.5%)	10/1012 (1.0%)	24/1975 (1.2%)
	Overall	45/1432 (3.1%)	43/1452 (3.0%)	88/2884 (3.1%)
Acoustic neuroma	3rd year	0/ 963	1/1012 (<0.1%)	1/1975 (<0.1%)
	Overall	0/1432	1/1452 (<0.1%)	1/2884 (<0.1%)
Acrochordon	1st year	0/1432	1/1452 (<0.1%)	1/2884 (<0.1%)
	2nd year	1/1166 (<0.1%)	1/1206 (<0.1%)	2/2372 (<0.1%)
	3rd year	0/ 963	1/1012 (<0.1%)	1/1975 (<0.1%)
	Overall	1/1432 (<0.1%)	3/1452 (0.2%)	4/2884 (0.1%)
Acute leukaemia	3rd year	1/ 963 (0.1%)	0/1012	1/1975 (<0.1%)
	Overall	1/1432 (<0.1%)	0/1452	1/2884 (<0.1%)
Adenoma benign	2nd year	0/1166	1/1206 (<0.1%)	1/2372 (<0.1%)
	Overall	0/1432	1/1452 (<0.1%)	1/2884 (<0.1%)
Astrocytoma, low grade	1st year	1/1432 (<0.1%)	0/1452	1/2884 (<0.1%)
	Overall	1/1432 (<0.1%)	0/1452	1/2884 (<0.1%)

Table 14.3.1 / 9: Number of subjects with adverse events by primary system organ class, preferred term and year (FAS)

Primary system organ class<br>Preferred term<br>MedDRA version 14.0	YEAR OF ONSET	LCS12	LCS16	Total
Benign breast neoplasm	1st year	1/1432 (<0.1%)	1/1452 (<0.1%)	2/2884 (<0.1%)
	2nd year	1/1166 (<0.1%)	0/1206	1/2372 (<0.1%)
	Overall	2/1432 (0.1%)	1/1452 (<0.1%)	3/2884 (0.1%)
Cervicitis human papilloma virus	1st year	0/1432	1/1452 (<0.1%)	1/2884 (<0.1%)
	2nd year	0/1166	3/1206 (0.2%)	3/2372 (0.1%)
	3rd year	1/ 963 (0.1%)	0/1012	1/1975 (<0.1%)
	Overall	1/1432 (<0.1%)	4/1452 (0.3%)	5/2884 (0.2%)
Cervix carcinoma stage 0	3rd year	0/ 963	1/1012 (<0.1%)	1/1975 (<0.1%)
	Overall	0/1432	1/1452 (<0.1%)	1/2884 (<0.1%)
Cervix neoplasm	3rd year	1/ 963 (0.1%)	0/1012	1/1975 (<0.1%)
	Overall	1/1432 (<0.1%)	0/1452	1/2884 (<0.1%)
Enchondroma	2nd year	0/1166	1/1206 (<0.1%)	1/2372 (<0.1%)
	Overall	0/1432	1/1452 (<0.1%)	1/2884 (<0.1%)
Fibroadenoma of breast	1st year	2/1432 (0.1%)	1/1452 (<0.1%)	3/2884 (0.1%)
	2nd year	4/1166 (0.3%)	1/1206 (<0.1%)	5/2372 (0.2%)
	3rd year	3/ 963 (0.3%)	0/1012	3/1975 (0.2%)
	Overall	7/1432 (0.5%)	2/1452 (0.1%)	9/2884 (0.3%)
Glomus tumour	1st year	1/1432 (<0.1%)	0/1452	1/2884 (<0.1%)
	2nd year	1/1166 (<0.1%)	0/1206	1/2372 (<0.1%)
	Overall	1/1432 (<0.1%)	0/1452	1/2884 (<0.1%)
Haemangioma	1st year	1/1432 (<0.1%)	0/1452	1/2884 (<0.1%)
	Overall	1/1432 (<0.1%)	0/1452	1/2884 (<0.1%)
Haemangioma of liver	2nd year	0/1166	1/1206 (<0.1%)	1/2372 (<0.1%)
	Overall	0/1432	1/1452 (<0.1%)	1/2884 (<0.1%)
Leiomyoma	2nd year	0/1166	1/1206 (<0.1%)	1/2372 (<0.1%)
	Overall	0/1432	1/1452 (<0.1%)	1/2884 (<0.1%)
Lipoma of breast	2nd year	2/1166 (0.2%)	0/1206	2/2372 (<0.1%)
	Overall	2/1432 (0.1%)	0/1452	2/2884 (<0.1%)

Table 14.3.1 / 9: Number of subjects with adverse events by primary system organ class, preferred term and year (FAS)

Primary system organ class<br>Preferred term<br>MedDRA version 14.0	YEAR OF ONSET	LCS12	LCS16	Total
Malignant melanoma	2nd year	0/1166	1/1206 (<0.1%)	1/2372 (<0.1%)
	Overall	0/1432	1/1452 (<0.1%)	1/2884 (<0.1%)
Melanocytic naevus	1st year	4/1432 (0.3%)	2/1452 (0.1%)	6/2884 (0.2%)
	2nd year	1/1166 (<0.1%)	1/1206 (<0.1%)	2/2372 (<0.1%)
	3rd year	1/ 963 (0.1%)	1/1012 (<0.1%)	2/1975 (0.1%)
	Overall	6/1432 (0.4%)	4/1452 (0.3%)	10/2884 (0.3%)
Neurilemmoma	1st year	0/1432	1/1452 (<0.1%)	1/2884 (<0.1%)
	Overall	0/1432	1/1452 (<0.1%)	1/2884 (<0.1%)
Ovarian germ cell teratoma benign	1st year	3/1432 (0.2%)	3/1452 (0.2%)	6/2884 (0.2%)
	3rd year	2/ 963 (0.2%)	0/1012	2/1975 (0.1%)
	Overall	5/1432 (0.3%)	3/1452 (0.2%)	8/2884 (0.3%)
Ovarian neoplasm	1st year	0/1432	2/1452 (0.1%)	2/2884 (<0.1%)
	Overall	0/1432	2/1452 (0.1%)	2/2884 (<0.1%)
Pancreatic carcinoma	1st year	0/1432	1/1452 (<0.1%)	1/2884 (<0.1%)
	Overall	0/1432	1/1452 (<0.1%)	1/2884 (<0.1%)
Skin papilloma	1st year	1/1432 (<0.1%)	1/1452 (<0.1%)	2/2884 (<0.1%)
	2nd year	0/1166	1/1206 (<0.1%)	1/2372 (<0.1%)
	Overall	1/1432 (<0.1%)	2/1452 (0.1%)	3/2884 (0.1%)
Teratoma benign	2nd year	0/1166	1/1206 (<0.1%)	1/2372 (<0.1%)
	Overall	0/1432	1/1452 (<0.1%)	1/2884 (<0.1%)
Thyroid cancer	2nd year	1/1166 (<0.1%)	0/1206	1/2372 (<0.1%)
	3rd year	1/ 963 (0.1%)	0/1012	1/1975 (<0.1%)
	Overall	2/1432 (0.1%)	0/1452	2/2884 (<0.1%)
Thyroid neoplasm	2nd year	0/1166	1/1206 (<0.1%)	1/2372 (<0.1%)
	3rd year	0/ 963	1/1012 (<0.1%)	1/1975 (<0.1%)
	Overall	0/1432	2/1452 (0.1%)	2/2884 (<0.1%)
Uterine leiomyoma	1st year	4/1432 (0.3%)	2/1452 (0.1%)	6/2884 (0.2%)
	2nd year	3/1166 (0.3%)	2/1206 (0.2%)	5/2372 (0.2%)
	3rd year	1/ 963 (0.1%)	4/1012 (0.4%)	5/1975 (0.3%)
	Overall	8/1432 (0.6%)	6/1452 (0.4%)	14/2884 (0.5%)

Table 14.3.1 / 9: Number of subjects with adverse events by primary system organ class, preferred term and year (FAS)

Primary system organ class<br>Preferred term<br>MedDRA version 14.0	YEAR OF ONSET	LCS12	LCS16	Total
Vulvovaginal human papilloma virus infection	1st year	2/1432 (0.1%)	2/1452 (0.1%)	4/2884 (0.1%)
	2nd year	0/1166	3/1206 (0.2%)	3/2372 (0.1%)
	3rd year	3/ 963 (0.3%)	1/1012 (<0.1%)	4/1975 (0.2%)
	Overall	5/1432 (0.3%)	6/1452 (0.4%)	11/2884 (0.4%)
Nervous system disorders	1st year	154/1432 (10.8%)	170/1452 (11.7%)	324/2884 (11.2%)
	2nd year	44/1166 (3.8%)	48/1206 (4.0%)	92/2372 (3.9%)
	3rd year	27/ 963 (2.8%)	39/1012 (3.9%)	66/1975 (3.3%)
	Overall	200/1432 (14.0%)	221/1452 (15.2%)	421/2884 (14.6%)
Amnesia	1st year	1/1432 (<0.1%)	0/1452	1/2884 (<0.1%)
	2nd year	0/1166	1/1206 (<0.1%)	1/2372 (<0.1%)
	Overall	1/1432 (<0.1%)	1/1452 (<0.1%)	2/2884 (<0.1%)
Aphonia	1st year	0/1432	1/1452 (<0.1%)	1/2884 (<0.1%)
	Overall	0/1432	1/1452 (<0.1%)	1/2884 (<0.1%)
Burning sensation	1st year	1/1432 (<0.1%)	0/1452	1/2884 (<0.1%)
	Overall	1/1432 (<0.1%)	0/1452	1/2884 (<0.1%)
Carpal tunnel syndrome	1st year	0/1432	4/1452 (0.3%)	4/2884 (0.1%)
	2nd year	0/1166	2/1206 (0.2%)	2/2372 (<0.1%)
	3rd year	1/ 963 (0.1%)	0/1012	1/1975 (<0.1%)
	Overall	1/1432 (<0.1%)	6/1452 (0.4%)	7/2884 (0.2%)
Cervicobrachial syndrome	1st year	0/1432	1/1452 (<0.1%)	1/2884 (<0.1%)
	Overall	0/1432	1/1452 (<0.1%)	1/2884 (<0.1%)
Cervicogenic headache	2nd year	1/1166 (<0.1%)	0/1206	1/2372 (<0.1%)
	Overall	1/1432 (<0.1%)	0/1452	1/2884 (<0.1%)
Cluster headache	2nd year	0/1166	1/1206 (<0.1%)	1/2372 (<0.1%)
	Overall	0/1432	1/1452 (<0.1%)	1/2884 (<0.1%)
Convulsion	1st year	0/1432	2/1452 (0.1%)	2/2884 (<0.1%)
	2nd year	1/1166 (<0.1%)	0/1206	1/2372 (<0.1%)
	Overall	1/1432 (<0.1%)	2/1452 (0.1%)	3/2884 (0.1%)

Table 14.3.1 / 9: Number of subjects with adverse events by primary system organ class, preferred term and year (FAS)

Primary system organ class<br>Preferred term<br>MedDRA version 14.0	YEAR OF ONSET	LCS12	LCS16	Total
Disturbance in attention	2nd year	0/1166	1/1206 (<0.1%)	1/2372 (<0.1%)
	3rd year	0/ 963	1/1012 (<0.1%)	1/1975 (<0.1%)
	Overall	0/1432	2/1452 (0.1%)	2/2884 (<0.1%)
Dizziness	1st year	7/1432 (0.5%)	14/1452 (1.0%)	21/2884 (0.7%)
	2nd year	2/1166 (0.2%)	3/1206 (0.2%)	5/2372 (0.2%)
	3rd year	0/ 963	5/1012 (0.5%)	5/1975 (0.3%)
	Overall	9/1432 (0.6%)	22/1452 (1.5%)	31/2884 (1.1%)
Dizziness postural	1st year	1/1432 (<0.1%)	0/1452	1/2884 (<0.1%)
	Overall	1/1432 (<0.1%)	0/1452	1/2884 (<0.1%)
Facial spasm	3rd year	1/ 963 (0.1%)	0/1012	1/1975 (<0.1%)
	Overall	1/1432 (<0.1%)	0/1452	1/2884 (<0.1%)
Headache	1st year	102/1432 (7.1%)	108/1452 (7.4%)	210/2884 (7.3%)
	2nd year	29/1166 (2.5%)	28/1206 (2.3%)	57/2372 (2.4%)
	3rd year	15/ 963 (1.6%)	23/1012 (2.3%)	38/1975 (1.9%)
	Overall	133/1432 (9.3%)	137/1452 (9.4%)	270/2884 (9.4%)
Hypoaesthesia	1st year	1/1432 (<0.1%)	2/1452 (0.1%)	3/2884 (0.1%)
	2nd year	1/1166 (<0.1%)	1/1206 (<0.1%)	2/2372 (<0.1%)
	3rd year	0/ 963	1/1012 (<0.1%)	1/1975 (<0.1%)
	Overall	2/1432 (0.1%)	4/1452 (0.3%)	6/2884 (0.2%)
Loss of consciousness	1st year	0/1432	1/1452 (<0.1%)	1/2884 (<0.1%)
	Overall	0/1432	1/1452 (<0.1%)	1/2884 (<0.1%)
Migraine	1st year	22/1432 (1.5%)	25/1452 (1.7%)	47/2884 (1.6%)
	2nd year	5/1166 (0.4%)	8/1206 (0.7%)	13/2372 (0.5%)
	3rd year	8/ 963 (0.8%)	6/1012 (0.6%)	14/1975 (0.7%)
	Overall	34/1432 (2.4%)	38/1452 (2.6%)	72/2884 (2.5%)
Migraine with aura	1st year	3/1432 (0.2%)	1/1452 (<0.1%)	4/2884 (0.1%)
	2nd year	1/1166 (<0.1%)	0/1206	1/2372 (<0.1%)
	3rd year	0/ 963	1/1012 (<0.1%)	1/1975 (<0.1%)
	Overall	4/1432 (0.3%)	2/1452 (0.1%)	6/2884 (0.2%)
Migraine without aura	1st year	1/1432 (<0.1%)	0/1452	1/2884 (<0.1%)
	Overall	1/1432 (<0.1%)	0/1452	1/2884 (<0.1%)

Table 14.3.1 / 9: Number of subjects with adverse events by primary system organ class, preferred term and year (FAS)

Primary system organ class<br>Preferred term<br>MedDRA version 14.0	YEAR OF ONSET	LCS12	LCS16	Total
Morton's neuralgia	2nd year	1/1166 (<0.1%)	0/1206	1/2372 (<0.1%)
	Overall	1/1432 (<0.1%)	0/1452	1/2884 (<0.1%)
Multiple sclerosis	3rd year	1/ 963 (0.1%)	0/1012	1/1975 (<0.1%)
	Overall	1/1432 (<0.1%)	0/1452	1/2884 (<0.1%)
Muscle contractions involuntary	1st year	0/1432	1/1452 (<0.1%)	1/2884 (<0.1%)
	Overall	0/1432	1/1452 (<0.1%)	1/2884 (<0.1%)
Nerve compression	2nd year	1/1166 (<0.1%)	0/1206	1/2372 (<0.1%)
	3rd year	1/ 963 (0.1%)	0/1012	1/1975 (<0.1%)
	Overall	2/1432 (0.1%)	0/1452	2/2884 (<0.1%)
Nervous system disorder	1st year	0/1432	1/1452 (<0.1%)	1/2884 (<0.1%)
	Overall	0/1432	1/1452 (<0.1%)	1/2884 (<0.1%)
Paraesthesia	1st year	0/1432	4/1452 (0.3%)	4/2884 (0.1%)
	3rd year	0/ 963	1/1012 (<0.1%)	1/1975 (<0.1%)
	Overall	0/1432	5/1452 (0.3%)	5/2884 (0.2%)
Presyncope	1st year	4/1432 (0.3%)	8/1452 (0.6%)	12/2884 (0.4%)
	3rd year	0/ 963	1/1012 (<0.1%)	1/1975 (<0.1%)
	Overall	4/1432 (0.3%)	9/1452 (0.6%)	13/2884 (0.5%)
Restless legs syndrome	1st year	1/1432 (<0.1%)	0/1452	1/2884 (<0.1%)
	Overall	1/1432 (<0.1%)	0/1452	1/2884 (<0.1%)
Sciatica	1st year	3/1432 (0.2%)	3/1452 (0.2%)	6/2884 (0.2%)
	2nd year	1/1166 (<0.1%)	0/1206	1/2372 (<0.1%)
	Overall	4/1432 (0.3%)	3/1452 (0.2%)	7/2884 (0.2%)
Sinus headache	1st year	1/1432 (<0.1%)	0/1452	1/2884 (<0.1%)
	2nd year	0/1166	1/1206 (<0.1%)	1/2372 (<0.1%)
	3rd year	0/ 963	1/1012 (<0.1%)	1/1975 (<0.1%)
	Overall	1/1432 (<0.1%)	2/1452 (0.1%)	3/2884 (0.1%)
Somnolence	1st year	2/1432 (0.1%)	0/1452	2/2884 (<0.1%)
	2nd year	0/1166	1/1206 (<0.1%)	1/2372 (<0.1%)
	Overall	2/1432 (0.1%)	1/1452 (<0.1%)	3/2884 (0.1%)

Table 14.3.1 / 9: Number of subjects with adverse events by primary system organ class, preferred term and year (FAS)

Primary system organ class<br>Preferred term<br>MedDRA version 14.0	YEAR OF ONSET	LCS12	LCS16	Total
Spinal cord herniation	2nd year	0/1166	1/1206 (<0.1%)	1/2372 (<0.1%)
	Overall	0/1432	1/1452 (<0.1%)	1/2884 (<0.1%)
Status migrainosus	1st year	1/1432 (<0.1%)	0/1452	1/2884 (<0.1%)
	Overall	1/1432 (<0.1%)	0/1452	1/2884 (<0.1%)
Syncope	1st year	6/1432 (0.4%)	4/1452 (0.3%)	10/2884 (0.3%)
	3rd year	0/ 963	1/1012 (<0.1%)	1/1975 (<0.1%)
	Overall	6/1432 (0.4%)	5/1452 (0.3%)	11/2884 (0.4%)
Tension headache	1st year	6/1432 (0.4%)	4/1452 (0.3%)	10/2884 (0.3%)
	2nd year	3/1166 (0.3%)	3/1206 (0.2%)	6/2372 (0.3%)
	3rd year	3/ 963 (0.3%)	3/1012 (0.3%)	6/1975 (0.3%)
	Overall	12/1432 (0.8%)	9/1452 (0.6%)	21/2884 (0.7%)
Thoracic outlet syndrome	2nd year	1/1166 (<0.1%)	0/1206	1/2372 (<0.1%)
	Overall	1/1432 (<0.1%)	0/1452	1/2884 (<0.1%)
VIIth nerve paralysis	1st year	2/1432 (0.1%)	0/1452	2/2884 (<0.1%)
	Overall	2/1432 (0.1%)	0/1452	2/2884 (<0.1%)
Pregnancy, puerperium and perinatal conditions	1st year	3/1432 (0.2%)	3/1452 (0.2%)	6/2884 (0.2%)
	2nd year	2/1166 (0.2%)	3/1206 (0.2%)	5/2372 (0.2%)
	3rd year	1/ 963 (0.1%)	4/1012 (0.4%)	5/1975 (0.3%)
	Overall	6/1432 (0.4%)	10/1452 (0.7%)	16/2884 (0.6%)
Abortion spontaneous	1st year	1/1432 (<0.1%)	0/1452	1/2884 (<0.1%)
	2nd year	1/1166 (<0.1%)	0/1206	1/2372 (<0.1%)
	3rd year	1/ 963 (0.1%)	0/1012	1/1975 (<0.1%)
	Overall	3/1432 (0.2%)	0/1452	3/2884 (0.1%)
Abortion spontaneous incomplete	2nd year	0/1166	1/1206 (<0.1%)	1/2372 (<0.1%)
	Overall	0/1432	1/1452 (<0.1%)	1/2884 (<0.1%)
Blighted ovum	3rd year	0/ 963	1/1012 (<0.1%)	1/1975 (<0.1%)
	Overall	0/1432	1/1452 (<0.1%)	1/2884 (<0.1%)

Table 14.3.1 / 9: Number of subjects with adverse events by primary system organ class, preferred term and year (FAS)

Primary system organ class<br>Preferred term<br>MedDRA version 14.0	YEAR OF<br>ONSET	LCS12	LCS16	Total
Ectopic pregnancy	1st year	2/1432 (0.1%)	2/1452 (0.1%)	4/2884 (0.1%)
	2nd year	1/1166 (<0.1%)	2/1206 (0.2%)	3/2372 (0.1%)
	3rd year	0/ 963	2/1012 (0.2%)	2/1975 (0.1%)
	Overall	3/1432 (0.2%)	6/1452 (0.4%)	9/2884 (0.3%)
Premature separation of placenta	2nd year	1/1166 (<0.1%)	0/1206	1/2372 (<0.1%)
	Overall	1/1432 (<0.1%)	0/1452	1/2884 (<0.1%)
Ruptured ectopic pregnancy	3rd year	0/ 963	1/1012 (<0.1%)	1/1975 (<0.1%)
	Overall	0/1432	1/1452 (<0.1%)	1/2884 (<0.1%)
Uterine contractions abnormal	1st year	0/1432	1/1452 (<0.1%)	1/2884 (<0.1%)
	Overall	0/1432	1/1452 (<0.1%)	1/2884 (<0.1%)
Psychiatric disorders	1st year	107/1432 (7.5%)	98/1452 (6.7%)	205/2884 (7.1%)
	2nd year	55/1166 (4.7%)	53/1206 (4.4%)	108/2372 (4.6%)
	3rd year	26/ 963 (2.7%)	37/1012 (3.7%)	63/1975 (3.2%)
	Overall	173/1432 (12.1%)	172/1452 (11.8%)	345/2884 (12.0%)
Acute stress disorder	3rd year	0/ 963	1/1012 (<0.1%)	1/1975 (<0.1%)
	Overall	0/1432	1/1452 (<0.1%)	1/2884 (<0.1%)
Adjustment disorder	1st year	1/1432 (<0.1%)	0/1452	1/2884 (<0.1%)
	Overall	1/1432 (<0.1%)	0/1452	1/2884 (<0.1%)
Affect lability	1st year	2/1432 (0.1%)	1/1452 (<0.1%)	3/2884 (0.1%)
	2nd year	2/1166 (0.2%)	0/1206	2/2372 (<0.1%)
	Overall	4/1432 (0.3%)	1/1452 (<0.1%)	5/2884 (0.2%)
Affective disorder	2nd year	0/1166	1/1206 (<0.1%)	1/2372 (<0.1%)
	Overall	0/1432	1/1452 (<0.1%)	1/2884 (<0.1%)
Aggression	1st year	1/1432 (<0.1%)	0/1452	1/2884 (<0.1%)
	Overall	1/1432 (<0.1%)	0/1452	1/2884 (<0.1%)
Alcohol abuse	2nd year	0/1166	1/1206 (<0.1%)	1/2372 (<0.1%)
	Overall	0/1432	1/1452 (<0.1%)	1/2884 (<0.1%)

Table 14.3.1 / 9: Number of subjects with adverse events by primary system organ class, preferred term and year (FAS)

Primary system organ class<br>Preferred term<br>MedDRA version 14.0	YEAR OF ONSET	LCS12	LCS16	Total
Alcoholism	2nd year	0/1166	1/1206 (<0.1%)	1/2372 (<0.1%)
	Overall	0/1432	1/1452 (<0.1%)	1/2884 (<0.1%)
Anxiety	1st year	17/1432 (1.2%)	17/1452 (1.2%)	34/2884 (1.2%)
	2nd year	13/1166 (1.1%)	12/1206 (1.0%)	25/2372 (1.1%)
	3rd year	9/ 963 (0.9%)	10/1012 (1.0%)	19/1975 (1.0%)
	Overall	37/1432 (2.6%)	37/1452 (2.5%)	74/2884 (2.6%)
Anxiety disorder	1st year	0/1432	2/1452 (0.1%)	2/2884 (<0.1%)
	2nd year	0/1166	1/1206 (<0.1%)	1/2372 (<0.1%)
	3rd year	1/ 963 (0.1%)	1/1012 (<0.1%)	2/1975 (0.1%)
	Overall	1/1432 (<0.1%)	4/1452 (0.3%)	5/2884 (0.2%)
Attention deficit/hyperactivity disorder	1st year	1/1432 (<0.1%)	3/1452 (0.2%)	4/2884 (0.1%)
	2nd year	1/1166 (<0.1%)	1/1206 (<0.1%)	2/2372 (<0.1%)
	3rd year	2/ 963 (0.2%)	0/1012	2/1975 (0.1%)
	Overall	4/1432 (0.3%)	4/1452 (0.3%)	8/2884 (0.3%)
Bipolar I disorder	1st year	1/1432 (<0.1%)	0/1452	1/2884 (<0.1%)
	Overall	1/1432 (<0.1%)	0/1452	1/2884 (<0.1%)
Bipolar disorder	1st year	0/1432	1/1452 (<0.1%)	1/2884 (<0.1%)
	2nd year	1/1166 (<0.1%)	2/1206 (0.2%)	3/2372 (0.1%)
	3rd year	0/ 963	3/1012 (0.3%)	3/1975 (0.2%)
	Overall	1/1432 (<0.1%)	6/1452 (0.4%)	7/2884 (0.2%)
Bulimia nervosa	1st year	0/1432	1/1452 (<0.1%)	1/2884 (<0.1%)
	Overall	0/1432	1/1452 (<0.1%)	1/2884 (<0.1%)
Burnout syndrome	1st year	1/1432 (<0.1%)	1/1452 (<0.1%)	2/2884 (<0.1%)
	2nd year	0/1166	1/1206 (<0.1%)	1/2372 (<0.1%)
	Overall	1/1432 (<0.1%)	2/1452 (0.1%)	3/2884 (0.1%)
Completed suicide	3rd year	0/ 963	1/1012 (<0.1%)	1/1975 (<0.1%)
	Overall	0/1432	1/1452 (<0.1%)	1/2884 (<0.1%)
Daydreaming	2nd year	1/1166 (<0.1%)	0/1206	1/2372 (<0.1%)
	Overall	1/1432 (<0.1%)	0/1452	1/2884 (<0.1%)

Table 14.3.1 / 9: Number of subjects with adverse events by primary system organ class, preferred term and year (FAS)

Primary system organ class<br>Preferred term<br>MedDRA version 14.0	YEAR OF ONSET	LCS12	LCS16	Total
Depressed mood	1st year	4/1432 (0.3%)	3/1452 (0.2%)	7/2884 (0.2%)
	2nd year	1/1166 (<0.1%)	0/1206	1/2372 (<0.1%)
	Overall	5/1432 (0.3%)	3/1452 (0.2%)	8/2884 (0.3%)
Depression	1st year	27/1432 (1.9%)	26/1452 (1.8%)	53/2884 (1.8%)
	2nd year	18/1166 (1.5%)	14/1206 (1.2%)	32/2372 (1.3%)
	3rd year	8/ 963 (0.8%)	12/1012 (1.2%)	20/1975 (1.0%)
	Overall	51/1432 (3.6%)	49/1452 (3.4%)	100/2884 (3.5%)
Depression suicidal	2nd year	1/1166 (<0.1%)	0/1206	1/2372 (<0.1%)
	Overall	1/1432 (<0.1%)	0/1452	1/2884 (<0.1%)
Depressive symptom	1st year	1/1432 (<0.1%)	0/1452	1/2884 (<0.1%)
	Overall	1/1432 (<0.1%)	0/1452	1/2884 (<0.1%)
Disorientation	1st year	0/1432	1/1452 (<0.1%)	1/2884 (<0.1%)
	Overall	0/1432	1/1452 (<0.1%)	1/2884 (<0.1%)
Drug dependence	1st year	1/1432 (<0.1%)	0/1452	1/2884 (<0.1%)
	2nd year	0/1166	1/1206 (<0.1%)	1/2372 (<0.1%)
	Overall	1/1432 (<0.1%)	1/1452 (<0.1%)	2/2884 (<0.1%)
Fear	1st year	0/1432	1/1452 (<0.1%)	1/2884 (<0.1%)
	3rd year	1/ 963 (0.1%)	0/1012	1/1975 (<0.1%)
	Overall	1/1432 (<0.1%)	1/1452 (<0.1%)	2/2884 (<0.1%)
Generalised anxiety disorder	1st year	3/1432 (0.2%)	0/1452	3/2884 (0.1%)
	Overall	3/1432 (0.2%)	0/1452	3/2884 (0.1%)
Hallucination, auditory	1st year	1/1432 (<0.1%)	0/1452	1/2884 (<0.1%)
	Overall	1/1432 (<0.1%)	0/1452	1/2884 (<0.1%)
Insomnia	1st year	16/1432 (1.1%)	17/1452 (1.2%)	33/2884 (1.1%)
	2nd year	4/1166 (0.3%)	10/1206 (0.8%)	14/2372 (0.6%)
	3rd year	3/ 963 (0.3%)	4/1012 (0.4%)	7/1975 (0.4%)
	Overall	22/1432 (1.5%)	29/1452 (2.0%)	51/2884 (1.8%)

Table 14.3.1 / 9: Number of subjects with adverse events by primary system organ class, preferred term and year (FAS)

Primary system organ class<br>Preferred term<br>MedDRA version 14.0	YEAR OF ONSET	LCS12	LCS16	Total
Libido decreased	1st year	18/1432 (1.3%)	16/1452 (1.1%)	34/2884 (1.2%)
	2nd year	8/1166 (0.7%)	8/1206 (0.7%)	16/2372 (0.7%)
	3rd year	2/ 963 (0.2%)	1/1012 (<0.1%)	3/1975 (0.2%)
	Overall	27/1432 (1.9%)	25/1452 (1.7%)	52/2884 (1.8%)
Libido increased	1st year	1/1432 (<0.1%)	1/1452 (<0.1%)	2/2884 (<0.1%)
	2nd year	1/1166 (<0.1%)	1/1206 (<0.1%)	2/2372 (<0.1%)
	Overall	2/1432 (0.1%)	2/1452 (0.1%)	4/2884 (0.1%)
Loss of libido	1st year	3/1432 (0.2%)	1/1452 (<0.1%)	4/2884 (0.1%)
	2nd year	0/1166	1/1206 (<0.1%)	1/2372 (<0.1%)
	Overall	4/1432 (0.3%)	2/1452 (0.1%)	6/2884 (0.2%)
Mental status changes	1st year	0/1432	1/1452 (<0.1%)	1/2884 (<0.1%)
	Overall	0/1432	1/1452 (<0.1%)	1/2884 (<0.1%)
Mood altered	1st year	10/1432 (0.7%)	8/1452 (0.6%)	18/2884 (0.6%)
	2nd year	5/1166 (0.4%)	2/1206 (0.2%)	7/2372 (0.3%)
	Overall	15/1432 (1.0%)	10/1452 (0.7%)	25/2884 (0.9%)
Mood swings	1st year	9/1432 (0.6%)	8/1452 (0.6%)	17/2884 (0.6%)
	2nd year	3/1166 (0.3%)	0/1206	3/2372 (0.1%)
	3rd year	0/ 963	1/1012 (<0.1%)	1/1975 (<0.1%)
	Overall	12/1432 (0.8%)	9/1452 (0.6%)	21/2884 (0.7%)
Nervousness	1st year	0/1432	1/1452 (<0.1%)	1/2884 (<0.1%)
	2nd year	0/1166	1/1206 (<0.1%)	1/2372 (<0.1%)
	Overall	0/1432	2/1452 (0.1%)	2/2884 (<0.1%)
Nicotine dependence	3rd year	1/ 963 (0.1%)	0/1012	1/1975 (<0.1%)
	Overall	1/1432 (<0.1%)	0/1452	1/2884 (<0.1%)
Obsessive-compulsive personality disorder	1st year	0/1432	1/1452 (<0.1%)	1/2884 (<0.1%)
	Overall	0/1432	1/1452 (<0.1%)	1/2884 (<0.1%)
Panic attack	1st year	0/1432	1/1452 (<0.1%)	1/2884 (<0.1%)
	2nd year	1/1166 (<0.1%)	0/1206	1/2372 (<0.1%)
	Overall	1/1432 (<0.1%)	1/1452 (<0.1%)	2/2884 (<0.1%)

Table 14.3.1 / 9: Number of subjects with adverse events by primary system organ class, preferred term and year (FAS)

Primary system organ class<br>Preferred term<br>MedDRA version 14.0	YEAR OF ONSET	LCS12	LCS16	Total
Panic disorder	1st year	0/1432	1/1452 (<0.1%)	1/2884 (<0.1%)
	2nd year	1/1166 (<0.1%)	1/1206 (<0.1%)	2/2372 (<0.1%)
	3rd year	0/ 963	3/1012 (0.3%)	3/1975 (0.2%)
	Overall	1/1432 (<0.1%)	5/1452 (0.3%)	6/2884 (0.2%)
Personality disorder	3rd year	1/ 963 (0.1%)	0/1012	1/1975 (<0.1%)
	Overall	1/1432 (<0.1%)	0/1452	1/2884 (<0.1%)
Phobia of flying	3rd year	0/ 963	1/1012 (<0.1%)	1/1975 (<0.1%)
	Overall	0/1432	1/1452 (<0.1%)	1/2884 (<0.1%)
Polysubstance dependence	2nd year	0/1166	1/1206 (<0.1%)	1/2372 (<0.1%)
	Overall	0/1432	1/1452 (<0.1%)	1/2884 (<0.1%)
Post-traumatic stress disorder	2nd year	1/1166 (<0.1%)	1/1206 (<0.1%)	2/2372 (<0.1%)
	Overall	1/1432 (<0.1%)	1/1452 (<0.1%)	2/2884 (<0.1%)
Psychotic disorder	2nd year	0/1166	1/1206 (<0.1%)	1/2372 (<0.1%)
	Overall	0/1432	1/1452 (<0.1%)	1/2884 (<0.1%)
Sleep disorder	1st year	3/1432 (0.2%)	1/1452 (<0.1%)	4/2884 (0.1%)
	2nd year	2/1166 (0.2%)	1/1206 (<0.1%)	3/2372 (0.1%)
	3rd year	1/ 963 (0.1%)	0/1012	1/1975 (<0.1%)
	Overall	5/1432 (0.3%)	2/1452 (0.1%)	7/2884 (0.2%)
Social phobia	1st year	0/1432	1/1452 (<0.1%)	1/2884 (<0.1%)
	Overall	0/1432	1/1452 (<0.1%)	1/2884 (<0.1%)
Stress	1st year	2/1432 (0.1%)	0/1452	2/2884 (<0.1%)
	2nd year	0/1166	3/1206 (0.2%)	3/2372 (0.1%)
	3rd year	1/ 963 (0.1%)	1/1012 (<0.1%)	2/1975 (0.1%)
	Overall	3/1432 (0.2%)	4/1452 (0.3%)	7/2884 (0.2%)
Suicide attempt	2nd year	1/1166 (<0.1%)	0/1206	1/2372 (<0.1%)
	Overall	1/1432 (<0.1%)	0/1452	1/2884 (<0.1%)
Renal and urinary disorders	1st year	25/1432 (1.7%)	16/1452 (1.1%)	41/2884 (1.4%)
	2nd year	8/1166 (0.7%)	8/1206 (0.7%)	16/2372 (0.7%)
	3rd year	10/ 963 (1.0%)	9/1012 (0.9%)	19/1975 (1.0%)
	Overall	41/1432 (2.9%)	31/1452 (2.1%)	72/2884 (2.5%)

Table 14.3.1 / 9: Number of subjects with adverse events by primary system organ class, preferred term and year (FAS)

Primary system organ class<br>Preferred term<br>MedDRA version 14.0	YEAR OF ONSET	LCS12	LCS16	Total
Calculus bladder	1st year	1/1432 (<0.1%)	0/1452	1/2884 (<0.1%)
	Overall	1/1432 (<0.1%)	0/1452	1/2884 (<0.1%)
Calculus urinary	1st year	0/1432	1/1452 (<0.1%)	1/2884 (<0.1%)
	Overall	0/1432	1/1452 (<0.1%)	1/2884 (<0.1%)
Chromaturia	1st year	0/1432	1/1452 (<0.1%)	1/2884 (<0.1%)
	Overall	0/1432	1/1452 (<0.1%)	1/2884 (<0.1%)
Cystitis haemorrhagic	1st year	1/1432 (<0.1%)	0/1452	1/2884 (<0.1%)
	Overall	1/1432 (<0.1%)	0/1452	1/2884 (<0.1%)
Dysuria	1st year	9/1432 (0.6%)	5/1452 (0.3%)	14/2884 (0.5%)
	2nd year	3/1166 (0.3%)	2/1206 (0.2%)	5/2372 (0.2%)
	3rd year	3/ 963 (0.3%)	0/1012	3/1975 (0.2%)
	Overall	14/1432 (1.0%)	7/1452 (0.5%)	21/2884 (0.7%)
Haematuria	1st year	1/1432 (<0.1%)	1/1452 (<0.1%)	2/2884 (<0.1%)
	3rd year	1/ 963 (0.1%)	1/1012 (<0.1%)	2/1975 (0.1%)
	Overall	2/1432 (0.1%)	2/1452 (0.1%)	4/2884 (0.1%)
Hypertonic bladder	3rd year	0/ 963	2/1012 (0.2%)	2/1975 (0.1%)
	Overall	0/1432	2/1452 (0.1%)	2/2884 (<0.1%)
Micturition urgency	1st year	1/1432 (<0.1%)	0/1452	1/2884 (<0.1%)
	2nd year	1/1166 (<0.1%)	2/1206 (0.2%)	3/2372 (0.1%)
	3rd year	1/ 963 (0.1%)	1/1012 (<0.1%)	2/1975 (0.1%)
	Overall	2/1432 (0.1%)	3/1452 (0.2%)	5/2884 (0.2%)
Nephrolithiasis	1st year	4/1432 (0.3%)	0/1452	4/2884 (0.1%)
	2nd year	1/1166 (<0.1%)	2/1206 (0.2%)	3/2372 (0.1%)
	3rd year	3/ 963 (0.3%)	1/1012 (<0.1%)	4/1975 (0.2%)
	Overall	8/1432 (0.6%)	3/1452 (0.2%)	11/2884 (0.4%)
Pollakiuria	1st year	3/1432 (0.2%)	7/1452 (0.5%)	10/2884 (0.3%)
	2nd year	0/1166	1/1206 (<0.1%)	1/2372 (<0.1%)
	3rd year	1/ 963 (0.1%)	0/1012	1/1975 (<0.1%)
	Overall	4/1432 (0.3%)	8/1452 (0.6%)	12/2884 (0.4%)

Table 14.3.1 / 9: Number of subjects with adverse events by primary system organ class, preferred term and year (FAS)

Primary system organ class<br>Preferred term<br>MedDRA version 14.0	YEAR OF<br>ONSET	LCS12	LCS16	Total
Polyuria	1st year	0/1432	1/1452 (<0.1%)	1/2884 (<0.1%)
	2nd year	1/1166 (<0.1%)	0/1206	1/2372 (<0.1%)
	Overall	1/1432 (<0.1%)	1/1452 (<0.1%)	2/2884 (<0.1%)
Renal cyst	3rd year	0/ 963	1/1012 (<0.1%)	1/1975 (<0.1%)
	Overall	0/1432	1/1452 (<0.1%)	1/2884 (<0.1%)
Renal pain	1st year	1/1432 (<0.1%)	0/1452	1/2884 (<0.1%)
	Overall	1/1432 (<0.1%)	0/1452	1/2884 (<0.1%)
Strangury	1st year	1/1432 (<0.1%)	0/1452	1/2884 (<0.1%)
	Overall	1/1432 (<0.1%)	0/1452	1/2884 (<0.1%)
Stress urinary incontinence	1st year	0/1432	2/1452 (0.1%)	2/2884 (<0.1%)
	3rd year	1/ 963 (0.1%)	0/1012	1/1975 (<0.1%)
	Overall	1/1432 (<0.1%)	2/1452 (0.1%)	3/2884 (0.1%)
Ureteric obstruction	1st year	0/1432	1/1452 (<0.1%)	1/2884 (<0.1%)
	Overall	0/1432	1/1452 (<0.1%)	1/2884 (<0.1%)
Urethral cyst	3rd year	0/ 963	1/1012 (<0.1%)	1/1975 (<0.1%)
	Overall	0/1432	1/1452 (<0.1%)	1/2884 (<0.1%)
Urinary incontinence	1st year	2/1432 (0.1%)	0/1452	2/2884 (<0.1%)
	2nd year	1/1166 (<0.1%)	1/1206 (<0.1%)	2/2372 (<0.1%)
	3rd year	0/ 963	1/1012 (<0.1%)	1/1975 (<0.1%)
	Overall	3/1432 (0.2%)	1/1452 (<0.1%)	4/2884 (0.1%)
Urinary retention	3rd year	0/ 963	1/1012 (<0.1%)	1/1975 (<0.1%)
	Overall	0/1432	1/1452 (<0.1%)	1/2884 (<0.1%)
Urinary tract disorder	1st year	1/1432 (<0.1%)	0/1452	1/2884 (<0.1%)
	Overall	1/1432 (<0.1%)	0/1452	1/2884 (<0.1%)
Urinary tract inflammation	1st year	1/1432 (<0.1%)	0/1452	1/2884 (<0.1%)
	Overall	1/1432 (<0.1%)	0/1452	1/2884 (<0.1%)

Table 14.3.1 / 9: Number of subjects with adverse events by primary system organ class, preferred term and year (FAS)

Primary system organ class<br>Preferred term<br>MedDRA version 14.0	YEAR OF ONSET	LCS12	LCS16	Total
Urinary tract pain	1st year	1/1432 (<0.1%)	1/1452 (<0.1%)	2/2884 (<0.1%)
	2nd year	1/1166 (<0.1%)	0/1206	1/2372 (<0.1%)
	Overall	2/1432 (0.1%)	1/1452 (<0.1%)	3/2884 (0.1%)
Urine odour abnormal	1st year	0/1432	1/1452 (<0.1%)	1/2884 (<0.1%)
	Overall	0/1432	1/1452 (<0.1%)	1/2884 (<0.1%)
Reproductive system and breast disorders	1st year	478/1432 (33.4%)	536/1452 (36.9%)	1014/2884 (35.2%)
	2nd year	213/1166 (18.3%)	257/1206 (21.3%)	470/2372 (19.8%)
	3rd year	161/ 963 (16.7%)	195/1012 (19.3%)	356/1975 (18.0%)
	Overall	681/1432 (47.6%)	763/1452 (52.5%)	1444/2884 (50.1%)
Adenomyosis	3rd year	1/ 963 (0.1%)	0/1012	1/1975 (<0.1%)
	Overall	1/1432 (<0.1%)	0/1452	1/2884 (<0.1%)
Adnexa uteri mass	3rd year	1/ 963 (0.1%)	0/1012	1/1975 (<0.1%)
	Overall	1/1432 (<0.1%)	0/1452	1/2884 (<0.1%)
Adnexa uteri pain	1st year	2/1432 (0.1%)	3/1452 (0.2%)	5/2884 (0.2%)
	2nd year	0/1166	2/1206 (0.2%)	2/2372 (<0.1%)
	3rd year	1/ 963 (0.1%)	0/1012	1/1975 (<0.1%)
	Overall	3/1432 (0.2%)	5/1452 (0.3%)	8/2884 (0.3%)
Amenorrhoea	1st year	0/1432	2/1452 (0.1%)	2/2884 (<0.1%)
	2nd year	0/1166	4/1206 (0.3%)	4/2372 (0.2%)
	Overall	0/1432	6/1452 (0.4%)	6/2884 (0.2%)
Atrophic vulvovaginitis	2nd year	1/1166 (<0.1%)	0/1206	1/2372 (<0.1%)
	Overall	1/1432 (<0.1%)	0/1452	1/2884 (<0.1%)
Bartholin's cyst	3rd year	0/ 963	1/1012 (<0.1%)	1/1975 (<0.1%)
	Overall	0/1432	1/1452 (<0.1%)	1/2884 (<0.1%)
Breast calcifications	1st year	1/1432 (<0.1%)	0/1452	1/2884 (<0.1%)
	Overall	1/1432 (<0.1%)	0/1452	1/2884 (<0.1%)
Breast cyst	1st year	2/1432 (0.1%)	0/1452	2/2884 (<0.1%)
	2nd year	1/1166 (<0.1%)	2/1206 (0.2%)	3/2372 (0.1%)
	Overall	3/1432 (0.2%)	2/1452 (0.1%)	5/2884 (0.2%)

Table 14.3.1 / 9: Number of subjects with adverse events by primary system organ class, preferred term and year (FAS)

Primary system organ class<br>Preferred term<br>MedDRA version 14.0	YEAR OF<br>ONSET	LCS12	LCS16	Total
Breast discharge	1st year	0/1432	5/1452 (0.3%)	5/2884 (0.2%)
	2nd year	0/1166	2/1206 (0.2%)	2/2372 (<0.1%)
	3rd year	0/ 963	3/1012 (0.3%)	3/1975 (0.2%)
	Overall	0/1432	10/1452 (0.7%)	10/2884 (0.3%)
Breast discomfort	1st year	4/1432 (0.3%)	6/1452 (0.4%)	10/2884 (0.3%)
	2nd year	0/1166	1/1206 (<0.1%)	1/2372 (<0.1%)
	3rd year	0/ 963	1/1012 (<0.1%)	1/1975 (<0.1%)
	Overall	4/1432 (0.3%)	8/1452 (0.6%)	12/2884 (0.4%)
Breast disorder female	2nd year	0/1166	1/1206 (<0.1%)	1/2372 (<0.1%)
	Overall	0/1432	1/1452 (<0.1%)	1/2884 (<0.1%)
Breast dysplasia	3rd year	1/ 963 (0.1%)	0/1012	1/1975 (<0.1%)
	Overall	1/1432 (<0.1%)	0/1452	1/2884 (<0.1%)
Breast engorgement	2nd year	1/1166 (<0.1%)	0/1206	1/2372 (<0.1%)
	Overall	1/1432 (<0.1%)	0/1452	1/2884 (<0.1%)
Breast enlargement	1st year	0/1432	2/1452 (0.1%)	2/2884 (<0.1%)
	2nd year	0/1166	2/1206 (0.2%)	2/2372 (<0.1%)
	Overall	0/1432	4/1452 (0.3%)	4/2884 (0.1%)
Breast mass	1st year	3/1432 (0.2%)	2/1452 (0.1%)	5/2884 (0.2%)
	2nd year	1/1166 (<0.1%)	3/1206 (0.2%)	4/2372 (0.2%)
	3rd year	3/ 963 (0.3%)	2/1012 (0.2%)	5/1975 (0.3%)
	Overall	7/1432 (0.5%)	7/1452 (0.5%)	14/2884 (0.5%)
Breast pain	1st year	23/1432 (1.6%)	23/1452 (1.6%)	46/2884 (1.6%)
	2nd year	9/1166 (0.8%)	11/1206 (0.9%)	20/2372 (0.8%)
	3rd year	6/ 963 (0.6%)	12/1012 (1.2%)	18/1975 (0.9%)
	Overall	36/1432 (2.5%)	43/1452 (3.0%)	79/2884 (2.7%)
Breast swelling	1st year	1/1432 (<0.1%)	2/1452 (0.1%)	3/2884 (0.1%)
	Overall	1/1432 (<0.1%)	2/1452 (0.1%)	3/2884 (0.1%)
Breast tenderness	1st year	20/1432 (1.4%)	22/1452 (1.5%)	42/2884 (1.5%)
	2nd year	11/1166 (0.9%)	11/1206 (0.9%)	22/2372 (0.9%)
	3rd year	2/ 963 (0.2%)	4/1012 (0.4%)	6/1975 (0.3%)
	Overall	32/1432 (2.2%)	35/1452 (2.4%)	67/2884 (2.3%)

Table 14.3.1 / 9: Number of subjects with adverse events by primary system organ class, preferred term and year (FAS)

Primary system organ class<br>Preferred term<br>MedDRA version 14.0	YEAR OF ONSET	LCS12	LCS16	Total
Cervical cyst	1st year	0/1432	1/1452 (<0.1%)	1/2884 (<0.1%)
	Overall	0/1432	1/1452 (<0.1%)	1/2884 (<0.1%)
Cervical discharge	1st year	1/1432 (<0.1%)	0/1452	1/2884 (<0.1%)
	2nd year	1/1166 (<0.1%)	0/1206	1/2372 (<0.1%)
	Overall	2/1432 (0.1%)	0/1452	2/2884 (<0.1%)
Cervical dysplasia	1st year	17/1432 (1.2%)	24/1452 (1.7%)	41/2884 (1.4%)
	2nd year	46/1166 (3.9%)	53/1206 (4.4%)	99/2372 (4.2%)
	3rd year	50/ 963 (5.2%)	48/1012 (4.7%)	98/1975 (5.0%)
	Overall	107/1432 (7.5%)	115/1452 (7.9%)	222/2884 (7.7%)
Cervical friability	2nd year	0/1166	1/1206 (<0.1%)	1/2372 (<0.1%)
	Overall	0/1432	1/1452 (<0.1%)	1/2884 (<0.1%)
Cervical polyp	1st year	1/1432 (<0.1%)	0/1452	1/2884 (<0.1%)
	3rd year	0/ 963	2/1012 (0.2%)	2/1975 (0.1%)
	Overall	1/1432 (<0.1%)	2/1452 (0.1%)	3/2884 (0.1%)
Cervix disorder	1st year	0/1432	1/1452 (<0.1%)	1/2884 (<0.1%)
	Overall	0/1432	1/1452 (<0.1%)	1/2884 (<0.1%)
Cervix erythema	1st year	0/1432	1/1452 (<0.1%)	1/2884 (<0.1%)
	3rd year	0/ 963	1/1012 (<0.1%)	1/1975 (<0.1%)
	Overall	0/1432	2/1452 (0.1%)	2/2884 (<0.1%)
Cervix haemorrhage uterine	1st year	0/1432	1/1452 (<0.1%)	1/2884 (<0.1%)
	Overall	0/1432	1/1452 (<0.1%)	1/2884 (<0.1%)
Cervix inflammation	2nd year	1/1166 (<0.1%)	0/1206	1/2372 (<0.1%)
	Overall	1/1432 (<0.1%)	0/1452	1/2884 (<0.1%)
Cervix oedema	2nd year	0/1166	1/1206 (<0.1%)	1/2372 (<0.1%)
	Overall	0/1432	1/1452 (<0.1%)	1/2884 (<0.1%)
Coital bleeding	1st year	8/1432 (0.6%)	7/1452 (0.5%)	15/2884 (0.5%)
	2nd year	3/1166 (0.3%)	4/1206 (0.3%)	7/2372 (0.3%)
	3rd year	3/ 963 (0.3%)	3/1012 (0.3%)	6/1975 (0.3%)
	Overall	14/1432 (1.0%)	14/1452 (1.0%)	28/2884 (1.0%)

Table 14.3.1 / 9: Number of subjects with adverse events by primary system organ class, preferred term and year (FAS)

Primary system organ class<br>Preferred term<br>MedDRA version 14.0	YEAR OF ONSET	LCS12	LCS16	Total
Dysfunctional uterine bleeding	1st year	3/1432 (0.2%)	3/1452 (0.2%)	6/2884 (0.2%)
	2nd year	0/1166	1/1206 (<0.1%)	1/2372 (<0.1%)
	Overall	3/1432 (0.2%)	4/1452 (0.3%)	7/2884 (0.2%)
Dysmenorrhoea	1st year	104/1432 (7.3%)	75/1452 (5.2%)	179/2884 (6.2%)
	2nd year	20/1166 (1.7%)	26/1206 (2.2%)	46/2372 (1.9%)
	3rd year	13/ 963 (1.3%)	14/1012 (1.4%)	27/1975 (1.4%)
	Overall	130/1432 (9.1%)	108/1452 (7.4%)	238/2884 (8.3%)
Dyspareunia	1st year	24/1432 (1.7%)	18/1452 (1.2%)	42/2884 (1.5%)
	2nd year	7/1166 (0.6%)	6/1206 (0.5%)	13/2372 (0.5%)
	3rd year	3/ 963 (0.3%)	4/1012 (0.4%)	7/1975 (0.4%)
	Overall	33/1432 (2.3%)	28/1452 (1.9%)	61/2884 (2.1%)
Ectropion of cervix	1st year	2/1432 (0.1%)	1/1452 (<0.1%)	3/2884 (0.1%)
	Overall	2/1432 (0.1%)	1/1452 (<0.1%)	3/2884 (0.1%)
Endometriosis	1st year	4/1432 (0.3%)	1/1452 (<0.1%)	5/2884 (0.2%)
	2nd year	3/1166 (0.3%)	1/1206 (<0.1%)	4/2372 (0.2%)
	3rd year	1/ 963 (0.1%)	1/1012 (<0.1%)	2/1975 (0.1%)
	Overall	8/1432 (0.6%)	3/1452 (0.2%)	11/2884 (0.4%)
Fibrocystic breast disease	1st year	1/1432 (<0.1%)	1/1452 (<0.1%)	2/2884 (<0.1%)
	2nd year	2/1166 (0.2%)	3/1206 (0.2%)	5/2372 (0.2%)
	3rd year	1/ 963 (0.1%)	1/1012 (<0.1%)	2/1975 (0.1%)
	Overall	3/1432 (0.2%)	5/1452 (0.3%)	8/2884 (0.3%)
Galactorrhoea	1st year	3/1432 (0.2%)	5/1452 (0.3%)	8/2884 (0.3%)
	2nd year	1/1166 (<0.1%)	1/1206 (<0.1%)	2/2372 (<0.1%)
	3rd year	0/ 963	2/1012 (0.2%)	2/1975 (0.1%)
	Overall	4/1432 (0.3%)	8/1452 (0.6%)	12/2884 (0.4%)
Genital cyst	1st year	0/1432	1/1452 (<0.1%)	1/2884 (<0.1%)
	Overall	0/1432	1/1452 (<0.1%)	1/2884 (<0.1%)
Genital discharge	1st year	4/1432 (0.3%)	4/1452 (0.3%)	8/2884 (0.3%)
	2nd year	5/1166 (0.4%)	5/1206 (0.4%)	10/2372 (0.4%)
	3rd year	1/ 963 (0.1%)	0/1012	1/1975 (<0.1%)
	Overall	9/1432 (0.6%)	9/1452 (0.6%)	18/2884 (0.6%)

Table 14.3.1 / 9: Number of subjects with adverse events by primary system organ class, preferred term and year (FAS)

Primary system organ class<br>Preferred term<br>MedDRA version 14.0	YEAR OF ONSET	LCS12	LCS16	Total
Genital haemorrhage	1st year	1/1432 (<0.1%)	0/1452	1/2884 (<0.1%)
	Overall	1/1432 (<0.1%)	0/1452	1/2884 (<0.1%)
Genital lesion	1st year	2/1432 (0.1%)	0/1452	2/2884 (<0.1%)
	Overall	2/1432 (0.1%)	0/1452	2/2884 (<0.1%)
Genital rash	2nd year	0/1166	1/1206 (<0.1%)	1/2372 (<0.1%)
	Overall	0/1432	1/1452 (<0.1%)	1/2884 (<0.1%)
Haemorrhagic ovarian cyst	1st year	11/1432 (0.8%)	11/1452 (0.8%)	22/2884 (0.8%)
	2nd year	2/1166 (0.2%)	4/1206 (0.3%)	6/2372 (0.3%)
	3rd year	2/ 963 (0.2%)	4/1012 (0.4%)	6/1975 (0.3%)
	Overall	13/1432 (0.9%)	19/1452 (1.3%)	32/2884 (1.1%)
Hydrometra	1st year	1/1432 (<0.1%)	0/1452	1/2884 (<0.1%)
	2nd year	1/1166 (<0.1%)	0/1206	1/2372 (<0.1%)
	Overall	2/1432 (0.1%)	0/1452	2/2884 (<0.1%)
Hypomenorrhoea	2nd year	1/1166 (<0.1%)	0/1206	1/2372 (<0.1%)
	3rd year	1/ 963 (0.1%)	1/1012 (<0.1%)	2/1975 (0.1%)
	Overall	2/1432 (0.1%)	1/1452 (<0.1%)	3/2884 (0.1%)
Menometrorrhagia	1st year	1/1432 (<0.1%)	0/1452	1/2884 (<0.1%)
	2nd year	1/1166 (<0.1%)	0/1206	1/2372 (<0.1%)
	Overall	2/1432 (0.1%)	0/1452	2/2884 (<0.1%)
Menorrhagia	1st year	5/1432 (0.3%)	9/1452 (0.6%)	14/2884 (0.5%)
	3rd year	1/ 963 (0.1%)	1/1012 (<0.1%)	2/1975 (0.1%)
	Overall	6/1432 (0.4%)	10/1452 (0.7%)	16/2884 (0.6%)
Menstrual disorder	1st year	2/1432 (0.1%)	0/1452	2/2884 (<0.1%)
	2nd year	1/1166 (<0.1%)	0/1206	1/2372 (<0.1%)
	3rd year	0/ 963	1/1012 (<0.1%)	1/1975 (<0.1%)
	Overall	3/1432 (0.2%)	1/1452 (<0.1%)	4/2884 (0.1%)
Menstruation irregular	1st year	1/1432 (<0.1%)	3/1452 (0.2%)	4/2884 (0.1%)
	2nd year	1/1166 (<0.1%)	0/1206	1/2372 (<0.1%)
	3rd year	2/ 963 (0.2%)	0/1012	2/1975 (0.1%)
	Overall	4/1432 (0.3%)	3/1452 (0.2%)	7/2884 (0.2%)

Table 14.3.1 / 9: Number of subjects with adverse events by primary system organ class, preferred term and year (FAS)

Primary system organ class<br>Preferred term<br>MedDRA version 14.0	YEAR OF ONSET	LCS12	LCS16	Total
Metrorrhagia	1st year	11/1432 (0.8%)	10/1452 (0.7%)	21/2884 (0.7%)
	2nd year	1/1166 (<0.1%)	1/1206 (<0.1%)	2/2372 (<0.1%)
	3rd year	4/ 963 (0.4%)	1/1012 (<0.1%)	5/1975 (0.3%)
	Overall	16/1432 (1.1%)	12/1452 (0.8%)	28/2884 (1.0%)
Nipple exudate bloody	3rd year	0/ 963	1/1012 (<0.1%)	1/1975 (<0.1%)
	Overall	0/1432	1/1452 (<0.1%)	1/2884 (<0.1%)
Nipple pain	1st year	2/1432 (0.1%)	1/1452 (<0.1%)	3/2884 (0.1%)
	2nd year	0/1166	1/1206 (<0.1%)	1/2372 (<0.1%)
	3rd year	1/ 963 (0.1%)	0/1012	1/1975 (<0.1%)
	Overall	3/1432 (0.2%)	2/1452 (0.1%)	5/2884 (0.2%)
Oligomenorrhoea	1st year	0/1432	2/1452 (0.1%)	2/2884 (<0.1%)
	Overall	0/1432	2/1452 (0.1%)	2/2884 (<0.1%)
Ovarian cyst	1st year	115/1432 (8.0%)	204/1452 (14.0%)	319/2884 (11.1%)
	2nd year	57/1166 (4.9%)	76/1206 (6.3%)	133/2372 (5.6%)
	3rd year	40/ 963 (4.2%)	71/1012 (7.0%)	111/1975 (5.6%)
	Overall	186/1432 (13.0%)	304/1452 (20.9%)	490/2884 (17.0%)
Ovarian cyst ruptured	1st year	2/1432 (0.1%)	6/1452 (0.4%)	8/2884 (0.3%)
	2nd year	0/1166	1/1206 (<0.1%)	1/2372 (<0.1%)
	Overall	2/1432 (0.1%)	7/1452 (0.5%)	9/2884 (0.3%)
Ovarian cyst torsion	1st year	0/1432	1/1452 (<0.1%)	1/2884 (<0.1%)
	Overall	0/1432	1/1452 (<0.1%)	1/2884 (<0.1%)
Ovarian disorder	3rd year	0/ 963	1/1012 (<0.1%)	1/1975 (<0.1%)
	Overall	0/1432	1/1452 (<0.1%)	1/2884 (<0.1%)
Ovarian enlargement	1st year	1/1432 (<0.1%)	0/1452	1/2884 (<0.1%)
	2nd year	0/1166	1/1206 (<0.1%)	1/2372 (<0.1%)
	Overall	1/1432 (<0.1%)	1/1452 (<0.1%)	2/2884 (<0.1%)
Ovarian mass	2nd year	1/1166 (<0.1%)	2/1206 (0.2%)	3/2372 (0.1%)
	Overall	1/1432 (<0.1%)	2/1452 (0.1%)	3/2884 (0.1%)

Table 14.3.1 / 9: Number of subjects with adverse events by primary system organ class, preferred term and year (FAS)

Primary system organ class<br>Preferred term<br>MedDRA version 14.0	YEAR OF ONSET	LCS12	LCS16	Total
Ovulation pain	1st year	4/1432 (0.3%)	2/1452 (0.1%)	6/2884 (0.2%)
	2nd year	1/1166 (<0.1%)	0/1206	1/2372 (<0.1%)
	3rd year	1/ 963 (0.1%)	0/1012	1/1975 (<0.1%)
	Overall	6/1432 (0.4%)	2/1452 (0.1%)	8/2884 (0.3%)
Parovarian cyst	1st year	2/1432 (0.1%)	0/1452	2/2884 (<0.1%)
	3rd year	1/ 963 (0.1%)	0/1012	1/1975 (<0.1%)
	Overall	3/1432 (0.2%)	0/1452	3/2884 (0.1%)
Pelvic adhesions	3rd year	0/ 963	1/1012 (<0.1%)	1/1975 (<0.1%)
	Overall	0/1432	1/1452 (<0.1%)	1/2884 (<0.1%)
Pelvic congestion	1st year	1/1432 (<0.1%)	0/1452	1/2884 (<0.1%)
	Overall	1/1432 (<0.1%)	0/1452	1/2884 (<0.1%)
Pelvic discomfort	1st year	4/1432 (0.3%)	0/1452	4/2884 (0.1%)
	2nd year	1/1166 (<0.1%)	1/1206 (<0.1%)	2/2372 (<0.1%)
	3rd year	1/ 963 (0.1%)	1/1012 (<0.1%)	2/1975 (0.1%)
	Overall	6/1432 (0.4%)	2/1452 (0.1%)	8/2884 (0.3%)
Pelvic fluid collection	3rd year	1/ 963 (0.1%)	0/1012	1/1975 (<0.1%)
	Overall	1/1432 (<0.1%)	0/1452	1/2884 (<0.1%)
Pelvic pain	1st year	71/1432 (5.0%)	97/1452 (6.7%)	168/2884 (5.8%)
	2nd year	21/1166 (1.8%)	24/1206 (2.0%)	45/2372 (1.9%)
	3rd year	10/ 963 (1.0%)	12/1012 (1.2%)	22/1975 (1.1%)
	Overall	96/1432 (6.7%)	123/1452 (8.5%)	219/2884 (7.6%)
Pelvic prolapse	1st year	1/1432 (<0.1%)	0/1452	1/2884 (<0.1%)
	Overall	1/1432 (<0.1%)	0/1452	1/2884 (<0.1%)
Perineal pain	2nd year	1/1166 (<0.1%)	0/1206	1/2372 (<0.1%)
	Overall	1/1432 (<0.1%)	0/1452	1/2884 (<0.1%)
Polycystic ovaries	1st year	2/1432 (0.1%)	1/1452 (<0.1%)	3/2884 (0.1%)
	2nd year	0/1166	1/1206 (<0.1%)	1/2372 (<0.1%)
	3rd year	3/ 963 (0.3%)	0/1012	3/1975 (0.2%)
	Overall	4/1432 (0.3%)	2/1452 (0.1%)	6/2884 (0.2%)

Table 14.3.1 / 9: Number of subjects with adverse events by primary system organ class, preferred term and year (FAS)

Primary system organ class<br>Preferred term<br>MedDRA version 14.0	YEAR OF ONSET	LCS12	LCS16	Total
Polymenorrhoea	2nd year	1/1166 (<0.1%)	1/1206 (<0.1%)	2/2372 (<0.1%)
	Overall	1/1432 (<0.1%)	1/1452 (<0.1%)	2/2884 (<0.1%)
Premenstrual syndrome	1st year	9/1432 (0.6%)	8/1452 (0.6%)	17/2884 (0.6%)
	2nd year	6/1166 (0.5%)	2/1206 (0.2%)	8/2372 (0.3%)
	3rd year	2/ 963 (0.2%)	2/1012 (0.2%)	4/1975 (0.2%)
	Overall	17/1432 (1.2%)	12/1452 (0.8%)	29/2884 (1.0%)
Pruritus genital	1st year	1/1432 (<0.1%)	0/1452	1/2884 (<0.1%)
	Overall	1/1432 (<0.1%)	0/1452	1/2884 (<0.1%)
Uterine cervical erosion	1st year	1/1432 (<0.1%)	0/1452	1/2884 (<0.1%)
	Overall	1/1432 (<0.1%)	0/1452	1/2884 (<0.1%)
Uterine cervical pain	1st year	2/1432 (0.1%)	0/1452	2/2884 (<0.1%)
	Overall	2/1432 (0.1%)	0/1452	2/2884 (<0.1%)
Uterine haemorrhage	1st year	6/1432 (0.4%)	4/1452 (0.3%)	10/2884 (0.3%)
	2nd year	1/1166 (<0.1%)	2/1206 (0.2%)	3/2372 (0.1%)
	3rd year	1/ 963 (0.1%)	1/1012 (<0.1%)	2/1975 (0.1%)
	Overall	8/1432 (0.6%)	7/1452 (0.5%)	15/2884 (0.5%)
Uterine inflammation	2nd year	0/1166	1/1206 (<0.1%)	1/2372 (<0.1%)
	Overall	0/1432	1/1452 (<0.1%)	1/2884 (<0.1%)
Uterine pain	1st year	1/1432 (<0.1%)	2/1452 (0.1%)	3/2884 (0.1%)
	2nd year	1/1166 (<0.1%)	0/1206	1/2372 (<0.1%)
	Overall	2/1432 (0.1%)	2/1452 (0.1%)	4/2884 (0.1%)
Uterine polyp	1st year	1/1432 (<0.1%)	0/1452	1/2884 (<0.1%)
	2nd year	1/1166 (<0.1%)	0/1206	1/2372 (<0.1%)
	3rd year	0/ 963	1/1012 (<0.1%)	1/1975 (<0.1%)
	Overall	2/1432 (0.1%)	1/1452 (<0.1%)	3/2884 (0.1%)
Uterine prolapse	3rd year	1/ 963 (0.1%)	0/1012	1/1975 (<0.1%)
	Overall	1/1432 (<0.1%)	0/1452	1/2884 (<0.1%)

Table 14.3.1 / 9: Number of subjects with adverse events by primary system organ class, preferred term and year (FAS)

Primary system organ class<br>Preferred term<br>MedDRA version 14.0	YEAR OF ONSET	LCS12	LCS16	Total
Uterine spasm	1st year	27/1432 (1.9%)	37/1452 (2.5%)	64/2884 (2.2%)
	2nd year	3/1166 (0.3%)	0/1206	3/2372 (0.1%)
	3rd year	1/ 963 (0.1%)	2/1012 (0.2%)	3/1975 (0.2%)
	Overall	30/1432 (2.1%)	39/1452 (2.7%)	69/2884 (2.4%)
Uterine tenderness	1st year	1/1432 (<0.1%)	2/1452 (0.1%)	3/2884 (0.1%)
	Overall	1/1432 (<0.1%)	2/1452 (0.1%)	3/2884 (0.1%)
Vaginal cyst	1st year	1/1432 (<0.1%)	0/1452	1/2884 (<0.1%)
	Overall	1/1432 (<0.1%)	0/1452	1/2884 (<0.1%)
Vaginal discharge	1st year	36/1432 (2.5%)	33/1452 (2.3%)	69/2884 (2.4%)
	2nd year	20/1166 (1.7%)	24/1206 (2.0%)	44/2372 (1.9%)
	3rd year	6/ 963 (0.6%)	15/1012 (1.5%)	21/1975 (1.1%)
	Overall	55/1432 (3.8%)	58/1452 (4.0%)	113/2884 (3.9%)
Vaginal disorder	1st year	3/1432 (0.2%)	2/1452 (0.1%)	5/2884 (0.2%)
	3rd year	1/ 963 (0.1%)	2/1012 (0.2%)	3/1975 (0.2%)
	Overall	4/1432 (0.3%)	4/1452 (0.3%)	8/2884 (0.3%)
Vaginal haemorrhage	1st year	51/1432 (3.6%)	53/1452 (3.7%)	104/2884 (3.6%)
	2nd year	13/1166 (1.1%)	15/1206 (1.2%)	28/2372 (1.2%)
	3rd year	3/ 963 (0.3%)	5/1012 (0.5%)	8/1975 (0.4%)
	Overall	66/1432 (4.6%)	73/1452 (5.0%)	139/2884 (4.8%)
Vaginal lesion	1st year	1/1432 (<0.1%)	0/1452	1/2884 (<0.1%)
	Overall	1/1432 (<0.1%)	0/1452	1/2884 (<0.1%)
Vaginal odour	1st year	1/1432 (<0.1%)	5/1452 (0.3%)	6/2884 (0.2%)
	2nd year	1/1166 (<0.1%)	3/1206 (0.2%)	4/2372 (0.2%)
	3rd year	4/ 963 (0.4%)	1/1012 (<0.1%)	5/1975 (0.3%)
	Overall	6/1432 (0.4%)	7/1452 (0.5%)	13/2884 (0.5%)
Vaginal perforation	2nd year	0/1166	1/1206 (<0.1%)	1/2372 (<0.1%)
	Overall	0/1432	1/1452 (<0.1%)	1/2884 (<0.1%)
Vaginal ulceration	2nd year	0/1166	1/1206 (<0.1%)	1/2372 (<0.1%)
	Overall	0/1432	1/1452 (<0.1%)	1/2884 (<0.1%)

Table 14.3.1 / 9: Number of subjects with adverse events by primary system organ class, preferred term and year (FAS)

Primary system organ class<br>Preferred term<br>MedDRA version 14.0	YEAR OF ONSET	LCS12	LCS16	Total
Vaginal wall congestion	2nd year	1/1166 (<0.1%)	0/1206	1/2372 (<0.1%)
	Overall	1/1432 (<0.1%)	0/1452	1/2884 (<0.1%)
Vulval disorder	3rd year	1/ 963 (0.1%)	0/1012	1/1975 (<0.1%)
	Overall	1/1432 (<0.1%)	0/1452	1/2884 (<0.1%)
Vulvovaginal burning sensation	1st year	1/1432 (<0.1%)	1/1452 (<0.1%)	2/2884 (<0.1%)
	2nd year	0/1166	1/1206 (<0.1%)	1/2372 (<0.1%)
	3rd year	0/ 963	2/1012 (0.2%)	2/1975 (0.1%)
	Overall	1/1432 (<0.1%)	4/1452 (0.3%)	5/2884 (0.2%)
Vulvovaginal discomfort	1st year	4/1432 (0.3%)	1/1452 (<0.1%)	5/2884 (0.2%)
	2nd year	0/1166	1/1206 (<0.1%)	1/2372 (<0.1%)
	3rd year	2/ 963 (0.2%)	0/1012	2/1975 (0.1%)
	Overall	6/1432 (0.4%)	2/1452 (0.1%)	8/2884 (0.3%)
Vulvovaginal dryness	1st year	2/1432 (0.1%)	5/1452 (0.3%)	7/2884 (0.2%)
	2nd year	1/1166 (<0.1%)	0/1206	1/2372 (<0.1%)
	Overall	3/1432 (0.2%)	5/1452 (0.3%)	8/2884 (0.3%)
Vulvovaginal erythema	1st year	0/1432	1/1452 (<0.1%)	1/2884 (<0.1%)
	2nd year	0/1166	1/1206 (<0.1%)	1/2372 (<0.1%)
	Overall	0/1432	2/1452 (0.1%)	2/2884 (<0.1%)
Vulvovaginal pain	1st year	2/1432 (0.1%)	2/1452 (0.1%)	4/2884 (0.1%)
	3rd year	1/ 963 (0.1%)	0/1012	1/1975 (<0.1%)
	Overall	3/1432 (0.2%)	2/1452 (0.1%)	5/2884 (0.2%)
Vulvovaginal pruritus	1st year	10/1432 (0.7%)	11/1452 (0.8%)	21/2884 (0.7%)
	2nd year	4/1166 (0.3%)	4/1206 (0.3%)	8/2372 (0.3%)
	3rd year	3/ 963 (0.3%)	4/1012 (0.4%)	7/1975 (0.4%)
	Overall	16/1432 (1.1%)	17/1452 (1.2%)	33/2884 (1.1%)
Vulvovaginal swelling	2nd year	1/1166 (<0.1%)	1/1206 (<0.1%)	2/2372 (<0.1%)
	Overall	1/1432 (<0.1%)	1/1452 (<0.1%)	2/2884 (<0.1%)
Respiratory, thoracic and mediastinal disorders	1st year	36/1432 (2.5%)	40/1452 (2.8%)	76/2884 (2.6%)
	2nd year	31/1166 (2.7%)	20/1206 (1.7%)	51/2372 (2.2%)
	3rd year	14/ 963 (1.5%)	18/1012 (1.8%)	32/1975 (1.6%)
	Overall	71/1432 (5.0%)	70/1452 (4.8%)	141/2884 (4.9%)

Table 14.3.1 / 9: Number of subjects with adverse events by primary system organ class, preferred term and year (FAS)

Primary system organ class<br>Preferred term<br>MedDRA version 14.0	YEAR OF ONSET	LCS12	LCS16	Total
Allergic sinusitis	1st year	1/1432 (<0.1%)	1/1452 (<0.1%)	2/2884 (<0.1%)
	Overall	1/1432 (<0.1%)	1/1452 (<0.1%)	2/2884 (<0.1%)
Asthma	1st year	4/1432 (0.3%)	4/1452 (0.3%)	8/2884 (0.3%)
	2nd year	6/1166 (0.5%)	4/1206 (0.3%)	10/2372 (0.4%)
	3rd year	3/ 963 (0.3%)	4/1012 (0.4%)	7/1975 (0.4%)
	Overall	11/1432 (0.8%)	11/1452 (0.8%)	22/2884 (0.8%)
Atelectasis	3rd year	0/ 963	1/1012 (<0.1%)	1/1975 (<0.1%)
	Overall	0/1432	1/1452 (<0.1%)	1/2884 (<0.1%)
Bronchospasm	1st year	2/1432 (0.1%)	0/1452	2/2884 (<0.1%)
	2nd year	0/1166	1/1206 (<0.1%)	1/2372 (<0.1%)
	3rd year	0/ 963	1/1012 (<0.1%)	1/1975 (<0.1%)
	Overall	2/1432 (0.1%)	2/1452 (0.1%)	4/2884 (0.1%)
Cough	1st year	3/1432 (0.2%)	11/1452 (0.8%)	14/2884 (0.5%)
	2nd year	5/1166 (0.4%)	3/1206 (0.2%)	8/2372 (0.3%)
	3rd year	6/ 963 (0.6%)	1/1012 (<0.1%)	7/1975 (0.4%)
	Overall	13/1432 (0.9%)	13/1452 (0.9%)	26/2884 (0.9%)
Diaphragmatic hernia	1st year	0/1432	1/1452 (<0.1%)	1/2884 (<0.1%)
	Overall	0/1432	1/1452 (<0.1%)	1/2884 (<0.1%)
Dyspnoea	2nd year	1/1166 (<0.1%)	0/1206	1/2372 (<0.1%)
	3rd year	0/ 963	2/1012 (0.2%)	2/1975 (0.1%)
	Overall	1/1432 (<0.1%)	2/1452 (0.1%)	3/2884 (0.1%)
Epiglottic cyst	1st year	1/1432 (<0.1%)	0/1452	1/2884 (<0.1%)
	Overall	1/1432 (<0.1%)	0/1452	1/2884 (<0.1%)
Epistaxis	1st year	2/1432 (0.1%)	0/1452	2/2884 (<0.1%)
	2nd year	1/1166 (<0.1%)	0/1206	1/2372 (<0.1%)
	3rd year	0/ 963	1/1012 (<0.1%)	1/1975 (<0.1%)
	Overall	3/1432 (0.2%)	1/1452 (<0.1%)	4/2884 (0.1%)
Hyperventilation	1st year	1/1432 (<0.1%)	1/1452 (<0.1%)	2/2884 (<0.1%)
	Overall	1/1432 (<0.1%)	1/1452 (<0.1%)	2/2884 (<0.1%)

Table 14.3.1 / 9: Number of subjects with adverse events by primary system organ class, preferred term and year (FAS)

Primary system organ class<br>Preferred term<br>MedDRA version 14.0	YEAR OF<br>ONSET	LCS12	LCS16	Total
Hypoxia	3rd year	0/ 963	1/1012 (<0.1%)	1/1975 (<0.1%)
	Overall	0/1432	1/1452 (<0.1%)	1/2884 (<0.1%)
Nasal congestion	1st year	1/1432 (<0.1%)	1/1452 (<0.1%)	2/2884 (<0.1%)
	2nd year	1/1166 (<0.1%)	0/1206	1/2372 (<0.1%)
	Overall	2/1432 (0.1%)	1/1452 (<0.1%)	3/2884 (0.1%)
Nasal disorder	3rd year	1/ 963 (0.1%)	0/1012	1/1975 (<0.1%)
	Overall	1/1432 (<0.1%)	0/1452	1/2884 (<0.1%)
Nasal oedema	1st year	1/1432 (<0.1%)	0/1452	1/2884 (<0.1%)
	Overall	1/1432 (<0.1%)	0/1452	1/2884 (<0.1%)
Nasal polyps	1st year	1/1432 (<0.1%)	0/1452	1/2884 (<0.1%)
	2nd year	0/1166	1/1206 (<0.1%)	1/2372 (<0.1%)
	Overall	1/1432 (<0.1%)	1/1452 (<0.1%)	2/2884 (<0.1%)
Oropharyngeal pain	1st year	7/1432 (0.5%)	10/1452 (0.7%)	17/2884 (0.6%)
	2nd year	9/1166 (0.8%)	7/1206 (0.6%)	16/2372 (0.7%)
	3rd year	3/ 963 (0.3%)	2/1012 (0.2%)	5/1975 (0.3%)
	Overall	17/1432 (1.2%)	17/1452 (1.2%)	34/2884 (1.2%)
Pleural effusion	3rd year	0/ 963	1/1012 (<0.1%)	1/1975 (<0.1%)
	Overall	0/1432	1/1452 (<0.1%)	1/2884 (<0.1%)
Pleurisy	1st year	0/1432	1/1452 (<0.1%)	1/2884 (<0.1%)
	2nd year	1/1166 (<0.1%)	0/1206	1/2372 (<0.1%)
	Overall	1/1432 (<0.1%)	1/1452 (<0.1%)	2/2884 (<0.1%)
Pneumonitis	1st year	0/1432	1/1452 (<0.1%)	1/2884 (<0.1%)
	Overall	0/1432	1/1452 (<0.1%)	1/2884 (<0.1%)
Pneumothorax	2nd year	1/1166 (<0.1%)	0/1206	1/2372 (<0.1%)
	Overall	1/1432 (<0.1%)	0/1452	1/2884 (<0.1%)
Pulmonary congestion	1st year	1/1432 (<0.1%)	0/1452	1/2884 (<0.1%)
	Overall	1/1432 (<0.1%)	0/1452	1/2884 (<0.1%)

Table 14.3.1 / 9: Number of subjects with adverse events by primary system organ class, preferred term and year (FAS)

Primary system organ class<br>Preferred term<br>MedDRA version 14.0	YEAR OF ONSET	LCS12	LCS16	Total
Respiratory tract congestion	2nd year	1/1166 (<0.1%)	0/1206	1/2372 (<0.1%)
	Overall	1/1432 (<0.1%)	0/1452	1/2884 (<0.1%)
Respiratory tract inflammation	2nd year	1/1166 (<0.1%)	0/1206	1/2372 (<0.1%)
	Overall	1/1432 (<0.1%)	0/1452	1/2884 (<0.1%)
Rhinitis allergic	1st year	6/1432 (0.4%)	7/1452 (0.5%)	13/2884 (0.5%)
	2nd year	3/1166 (0.3%)	2/1206 (0.2%)	5/2372 (0.2%)
	3rd year	2/ 963 (0.2%)	4/1012 (0.4%)	6/1975 (0.3%)
	Overall	10/1432 (0.7%)	11/1452 (0.8%)	21/2884 (0.7%)
Rhinitis seasonal	1st year	1/1432 (<0.1%)	2/1452 (0.1%)	3/2884 (0.1%)
	Overall	1/1432 (<0.1%)	2/1452 (0.1%)	3/2884 (0.1%)
Rhinorrhoea	1st year	1/1432 (<0.1%)	0/1452	1/2884 (<0.1%)
	Overall	1/1432 (<0.1%)	0/1452	1/2884 (<0.1%)
Sinus congestion	1st year	4/1432 (0.3%)	0/1452	4/2884 (0.1%)
	2nd year	1/1166 (<0.1%)	2/1206 (0.2%)	3/2372 (0.1%)
	3rd year	1/ 963 (0.1%)	0/1012	1/1975 (<0.1%)
	Overall	6/1432 (0.4%)	2/1452 (0.1%)	8/2884 (0.3%)
Sinus polyp	2nd year	0/1166	1/1206 (<0.1%)	1/2372 (<0.1%)
	Overall	0/1432	1/1452 (<0.1%)	1/2884 (<0.1%)
Sleep apnoea syndrome	3rd year	0/ 963	1/1012 (<0.1%)	1/1975 (<0.1%)
	Overall	0/1432	1/1452 (<0.1%)	1/2884 (<0.1%)
Tonsillar disorder	3rd year	0/ 963	1/1012 (<0.1%)	1/1975 (<0.1%)
	Overall	0/1432	1/1452 (<0.1%)	1/2884 (<0.1%)
Upper respiratory tract inflammation	1st year	0/1432	1/1452 (<0.1%)	1/2884 (<0.1%)
	2nd year	0/1166	1/1206 (<0.1%)	1/2372 (<0.1%)
	3rd year	0/ 963	1/1012 (<0.1%)	1/1975 (<0.1%)
	Overall	0/1432	2/1452 (0.1%)	2/2884 (<0.1%)
Wheezing	1st year	1/1432 (<0.1%)	0/1452	1/2884 (<0.1%)
	Overall	1/1432 (<0.1%)	0/1452	1/2884 (<0.1%)

Table 14.3.1 / 9: Number of subjects with adverse events by primary system organ class, preferred term and year (FAS)

Primary system organ class<br>Preferred term<br>MedDRA version 14.0	YEAR OF ONSET	LCS12	LCS16	Total
Skin and subcutaneous tissue disorders	1st year	190/1432 (13.3%)	183/1452 (12.6%)	373/2884 (12.9%)
	2nd year	45/1166 (3.9%)	65/1206 (5.4%)	110/2372 (4.6%)
	3rd year	25/ 963 (2.6%)	28/1012 (2.8%)	53/1975 (2.7%)
	Overall	242/1432 (16.9%)	254/1452 (17.5%)	496/2884 (17.2%)
Acne	1st year	139/1432 (9.7%)	128/1452 (8.8%)	267/2884 (9.3%)
	2nd year	20/1166 (1.7%)	33/1206 (2.7%)	53/2372 (2.2%)
	3rd year	10/ 963 (1.0%)	13/1012 (1.3%)	23/1975 (1.2%)
	Overall	163/1432 (11.4%)	169/1452 (11.6%)	332/2884 (11.5%)
Acne cystic	1st year	1/1432 (<0.1%)	0/1452	1/2884 (<0.1%)
	2nd year	1/1166 (<0.1%)	0/1206	1/2372 (<0.1%)
	Overall	2/1432 (0.1%)	0/1452	2/2884 (<0.1%)
Alopecia	1st year	10/1432 (0.7%)	10/1452 (0.7%)	20/2884 (0.7%)
	2nd year	6/1166 (0.5%)	1/1206 (<0.1%)	7/2372 (0.3%)
	3rd year	0/ 963	2/1012 (0.2%)	2/1975 (0.1%)
	Overall	15/1432 (1.0%)	13/1452 (0.9%)	28/2884 (1.0%)
Alopecia areata	2nd year	1/1166 (<0.1%)	0/1206	1/2372 (<0.1%)
	3rd year	1/ 963 (0.1%)	0/1012	1/1975 (<0.1%)
	Overall	2/1432 (0.1%)	0/1452	2/2884 (<0.1%)
Chloasma	1st year	3/1432 (0.2%)	0/1452	3/2884 (0.1%)
	Overall	3/1432 (0.2%)	0/1452	3/2884 (0.1%)
Cold sweat	3rd year	1/ 963 (0.1%)	0/1012	1/1975 (<0.1%)
	Overall	1/1432 (<0.1%)	0/1452	1/2884 (<0.1%)
Dandruff	1st year	1/1432 (<0.1%)	1/1452 (<0.1%)	2/2884 (<0.1%)
	Overall	1/1432 (<0.1%)	1/1452 (<0.1%)	2/2884 (<0.1%)
Dermal cyst	1st year	2/1432 (0.1%)	0/1452	2/2884 (<0.1%)
	Overall	2/1432 (0.1%)	0/1452	2/2884 (<0.1%)
Dermatitis	1st year	4/1432 (0.3%)	5/1452 (0.3%)	9/2884 (0.3%)
	2nd year	3/1166 (0.3%)	2/1206 (0.2%)	5/2372 (0.2%)
	3rd year	1/ 963 (0.1%)	0/1012	1/1975 (<0.1%)
	Overall	8/1432 (0.6%)	7/1452 (0.5%)	15/2884 (0.5%)

Table 14.3.1 / 9: Number of subjects with adverse events by primary system organ class, preferred term and year (FAS)

Primary system organ class<br>Preferred term<br>MedDRA version 14.0	YEAR OF ONSET	LCS12	LCS16	Total
Dermatitis allergic	1st year	2/1432 (0.1%)	1/1452 (<0.1%)	3/2884 (0.1%)
	2nd year	1/1166 (<0.1%)	2/1206 (0.2%)	3/2372 (0.1%)
	3rd year	3/ 963 (0.3%)	2/1012 (0.2%)	5/1975 (0.3%)
	Overall	6/1432 (0.4%)	5/1452 (0.3%)	11/2884 (0.4%)
Dermatitis atopic	1st year	0/1432	3/1452 (0.2%)	3/2884 (0.1%)
	2nd year	2/1166 (0.2%)	1/1206 (<0.1%)	3/2372 (0.1%)
	3rd year	0/ 963	2/1012 (0.2%)	2/1975 (0.1%)
	Overall	2/1432 (0.1%)	5/1452 (0.3%)	7/2884 (0.2%)
Dermatitis contact	1st year	1/1432 (<0.1%)	3/1452 (0.2%)	4/2884 (0.1%)
	2nd year	1/1166 (<0.1%)	0/1206	1/2372 (<0.1%)
	3rd year	0/ 963	2/1012 (0.2%)	2/1975 (0.1%)
	Overall	2/1432 (0.1%)	5/1452 (0.3%)	7/2884 (0.2%)
Drug eruption	2nd year	1/1166 (<0.1%)	0/1206	1/2372 (<0.1%)
	Overall	1/1432 (<0.1%)	0/1452	1/2884 (<0.1%)
Dry skin	2nd year	0/1166	1/1206 (<0.1%)	1/2372 (<0.1%)
	Overall	0/1432	1/1452 (<0.1%)	1/2884 (<0.1%)
Dyshidrosis	1st year	1/1432 (<0.1%)	0/1452	1/2884 (<0.1%)
	Overall	1/1432 (<0.1%)	0/1452	1/2884 (<0.1%)
Eczema	1st year	4/1432 (0.3%)	4/1452 (0.3%)	8/2884 (0.3%)
	2nd year	2/1166 (0.2%)	3/1206 (0.2%)	5/2372 (0.2%)
	3rd year	1/ 963 (0.1%)	2/1012 (0.2%)	3/1975 (0.2%)
	Overall	6/1432 (0.4%)	8/1452 (0.6%)	14/2884 (0.5%)
Erythema	1st year	1/1432 (<0.1%)	0/1452	1/2884 (<0.1%)
	2nd year	1/1166 (<0.1%)	0/1206	1/2372 (<0.1%)
	Overall	2/1432 (0.1%)	0/1452	2/2884 (<0.1%)
Erythema multiforme	2nd year	1/1166 (<0.1%)	0/1206	1/2372 (<0.1%)
	3rd year	1/ 963 (0.1%)	0/1012	1/1975 (<0.1%)
	Overall	1/1432 (<0.1%)	0/1452	1/2884 (<0.1%)
Hidradenitis	1st year	1/1432 (<0.1%)	0/1452	1/2884 (<0.1%)
	Overall	1/1432 (<0.1%)	0/1452	1/2884 (<0.1%)

Table 14.3.1 / 9: Number of subjects with adverse events by primary system organ class, preferred term and year (FAS)

Primary system organ class<br>Preferred term<br>MedDRA version 14.0	YEAR OF ONSET	LCS12	LCS16	Total
Hirsutism	1st year	7/1432 (0.5%)	5/1452 (0.3%)	12/2884 (0.4%)
	2nd year	0/1166	6/1206 (0.5%)	6/2372 (0.3%)
	Overall	7/1432 (0.5%)	10/1452 (0.7%)	17/2884 (0.6%)
Hyperhidrosis	1st year	3/1432 (0.2%)	2/1452 (0.1%)	5/2884 (0.2%)
	Overall	3/1432 (0.2%)	2/1452 (0.1%)	5/2884 (0.2%)
Hyperkeratosis	1st year	1/1432 (<0.1%)	0/1452	1/2884 (<0.1%)
	Overall	1/1432 (<0.1%)	0/1452	1/2884 (<0.1%)
Hypertrichosis	1st year	2/1432 (0.1%)	0/1452	2/2884 (<0.1%)
	2nd year	0/1166	1/1206 (<0.1%)	1/2372 (<0.1%)
	Overall	2/1432 (0.1%)	1/1452 (<0.1%)	3/2884 (0.1%)
Idiopathic urticaria	1st year	1/1432 (<0.1%)	0/1452	1/2884 (<0.1%)
	Overall	1/1432 (<0.1%)	0/1452	1/2884 (<0.1%)
Increased tendency to bruise	3rd year	1/ 963 (0.1%)	0/1012	1/1975 (<0.1%)
	Overall	1/1432 (<0.1%)	0/1452	1/2884 (<0.1%)
Ingrown hair	2nd year	1/1166 (<0.1%)	0/1206	1/2372 (<0.1%)
	Overall	1/1432 (<0.1%)	0/1452	1/2884 (<0.1%)
Intertrigo	2nd year	1/1166 (<0.1%)	0/1206	1/2372 (<0.1%)
	Overall	1/1432 (<0.1%)	0/1452	1/2884 (<0.1%)
Lentigo	3rd year	1/ 963 (0.1%)	0/1012	1/1975 (<0.1%)
	Overall	1/1432 (<0.1%)	0/1452	1/2884 (<0.1%)
Lichen sclerosus	1st year	0/1432	2/1452 (0.1%)	2/2884 (<0.1%)
	2nd year	1/1166 (<0.1%)	0/1206	1/2372 (<0.1%)
	Overall	1/1432 (<0.1%)	2/1452 (0.1%)	3/2884 (0.1%)
Neurodermatitis	1st year	0/1432	1/1452 (<0.1%)	1/2884 (<0.1%)
	Overall	0/1432	1/1452 (<0.1%)	1/2884 (<0.1%)
Night sweats	2nd year	0/1166	1/1206 (<0.1%)	1/2372 (<0.1%)
	Overall	0/1432	1/1452 (<0.1%)	1/2884 (<0.1%)

Table 14.3.1 / 9: Number of subjects with adverse events by primary system organ class, preferred term and year (FAS)

Primary system organ class<br>Preferred term<br>MedDRA version 14.0	YEAR OF ONSET	LCS12	LCS16	Total
Photosensitivity reaction	2nd year	0/1166	1/1206 (<0.1%)	1/2372 (<0.1%)
	3rd year	0/ 963	1/1012 (<0.1%)	1/1975 (<0.1%)
	Overall	0/1432	1/1452 (<0.1%)	1/2884 (<0.1%)
Pityriasis rosea	1st year	1/1432 (<0.1%)	1/1452 (<0.1%)	2/2884 (<0.1%)
	2nd year	0/1166	1/1206 (<0.1%)	1/2372 (<0.1%)
	Overall	1/1432 (<0.1%)	2/1452 (0.1%)	3/2884 (0.1%)
Precancerous skin lesion	2nd year	0/1166	1/1206 (<0.1%)	1/2372 (<0.1%)
	Overall	0/1432	1/1452 (<0.1%)	1/2884 (<0.1%)
Pruritus	1st year	3/1432 (0.2%)	1/1452 (<0.1%)	4/2884 (0.1%)
	2nd year	0/1166	2/1206 (0.2%)	2/2372 (<0.1%)
	3rd year	1/ 963 (0.1%)	0/1012	1/1975 (<0.1%)
	Overall	4/1432 (0.3%)	3/1452 (0.2%)	7/2884 (0.2%)
Pruritus allergic	1st year	0/1432	1/1452 (<0.1%)	1/2884 (<0.1%)
	2nd year	0/1166	1/1206 (<0.1%)	1/2372 (<0.1%)
	Overall	0/1432	2/1452 (0.1%)	2/2884 (<0.1%)
Psoriasis	1st year	0/1432	1/1452 (<0.1%)	1/2884 (<0.1%)
	2nd year	0/1166	1/1206 (<0.1%)	1/2372 (<0.1%)
	Overall	0/1432	2/1452 (0.1%)	2/2884 (<0.1%)
Rash	1st year	6/1432 (0.4%)	7/1452 (0.5%)	13/2884 (0.5%)
	2nd year	2/1166 (0.2%)	0/1206	2/2372 (<0.1%)
	3rd year	2/ 963 (0.2%)	1/1012 (<0.1%)	3/1975 (0.2%)
	Overall	10/1432 (0.7%)	8/1452 (0.6%)	18/2884 (0.6%)
Rash macular	3rd year	0/ 963	1/1012 (<0.1%)	1/1975 (<0.1%)
	Overall	0/1432	1/1452 (<0.1%)	1/2884 (<0.1%)
Rash papular	1st year	1/1432 (<0.1%)	0/1452	1/2884 (<0.1%)
	2nd year	1/1166 (<0.1%)	1/1206 (<0.1%)	2/2372 (<0.1%)
	Overall	2/1432 (0.1%)	1/1452 (<0.1%)	3/2884 (0.1%)
Rosacea	1st year	0/1432	2/1452 (0.1%)	2/2884 (<0.1%)
	2nd year	1/1166 (<0.1%)	0/1206	1/2372 (<0.1%)
	Overall	1/1432 (<0.1%)	2/1452 (0.1%)	3/2884 (0.1%)

Table 14.3.1 / 9: Number of subjects with adverse events by primary system organ class, preferred term and year (FAS)

Primary system organ class<br>Preferred term<br>MedDRA version 14.0	YEAR OF ONSET	LCS12	LCS16	Total
Scar	2nd year	0/1166	1/1206 (<0.1%)	1/2372 (<0.1%)
	Overall	0/1432	1/1452 (<0.1%)	1/2884 (<0.1%)
Seborrhoea	1st year	6/1432 (0.4%)	8/1452 (0.6%)	14/2884 (0.5%)
	2nd year	0/1166	4/1206 (0.3%)	4/2372 (0.2%)
	Overall	7/1432 (0.5%)	10/1452 (0.7%)	17/2884 (0.6%)
Skin dystrophy	1st year	1/1432 (<0.1%)	0/1452	1/2884 (<0.1%)
	Overall	1/1432 (<0.1%)	0/1452	1/2884 (<0.1%)
Skin fissures	1st year	0/1432	1/1452 (<0.1%)	1/2884 (<0.1%)
	2nd year	0/1166	1/1206 (<0.1%)	1/2372 (<0.1%)
	Overall	0/1432	2/1452 (0.1%)	2/2884 (<0.1%)
Skin hypopigmentation	3rd year	0/ 963	1/1012 (<0.1%)	1/1975 (<0.1%)
	Overall	0/1432	1/1452 (<0.1%)	1/2884 (<0.1%)
Skin irritation	1st year	1/1432 (<0.1%)	2/1452 (0.1%)	3/2884 (0.1%)
	Overall	1/1432 (<0.1%)	2/1452 (0.1%)	3/2884 (0.1%)
Skin lesion	3rd year	0/ 963	1/1012 (<0.1%)	1/1975 (<0.1%)
	Overall	0/1432	1/1452 (<0.1%)	1/2884 (<0.1%)
Skin odour abnormal	2nd year	0/1166	1/1206 (<0.1%)	1/2372 (<0.1%)
	Overall	0/1432	1/1452 (<0.1%)	1/2884 (<0.1%)
Skin reaction	1st year	2/1432 (0.1%)	0/1452	2/2884 (<0.1%)
	Overall	2/1432 (0.1%)	0/1452	2/2884 (<0.1%)
Urticaria	1st year	7/1432 (0.5%)	5/1452 (0.3%)	12/2884 (0.4%)
	2nd year	2/1166 (0.2%)	2/1206 (0.2%)	4/2372 (0.2%)
	3rd year	3/ 963 (0.3%)	3/1012 (0.3%)	6/1975 (0.3%)
	Overall	11/1432 (0.8%)	10/1452 (0.7%)	21/2884 (0.7%)
Vitiligo	3rd year	0/ 963	1/1012 (<0.1%)	1/1975 (<0.1%)
	Overall	0/1432	1/1452 (<0.1%)	1/2884 (<0.1%)
Social circumstances	1st year	1/1432 (<0.1%)	1/1452 (<0.1%)	2/2884 (<0.1%)
	Overall	1/1432 (<0.1%)	1/1452 (<0.1%)	2/2884 (<0.1%)

Table 14.3.1 / 9: Number of subjects with adverse events by primary system organ class, preferred term and year (FAS)

Primary system organ class<br>Preferred term<br>MedDRA version 14.0	YEAR OF ONSET	LCS12	LCS16	Total
Exposure to communicable disease	1st year	1/1432 (<0.1%)	0/1452	1/2884 (<0.1%)
	Overall	1/1432 (<0.1%)	0/1452	1/2884 (<0.1%)
Hearing disability	1st year	0/1432	1/1452 (<0.1%)	1/2884 (<0.1%)
	Overall	0/1432	1/1452 (<0.1%)	1/2884 (<0.1%)
Surgical and medical procedures	1st year	17/1432 (1.2%)	22/1452 (1.5%)	39/2884 (1.4%)
	2nd year	14/1166 (1.2%)	18/1206 (1.5%)	32/2372 (1.3%)
	3rd year	10/ 963 (1.0%)	8/1012 (0.8%)	18/1975 (0.9%)
	Overall	40/1432 (2.8%)	45/1452 (3.1%)	85/2884 (2.9%)
Abdominoplasty	1st year	2/1432 (0.1%)	1/1452 (<0.1%)	3/2884 (0.1%)
	2nd year	1/1166 (<0.1%)	0/1206	1/2372 (<0.1%)
	3rd year	1/ 963 (0.1%)	0/1012	1/1975 (<0.1%)
	Overall	4/1432 (0.3%)	1/1452 (<0.1%)	5/2884 (0.2%)
Anal fissure excision	2nd year	1/1166 (<0.1%)	0/1206	1/2372 (<0.1%)
	Overall	1/1432 (<0.1%)	0/1452	1/2884 (<0.1%)
Anal sphincterotomy	2nd year	0/1166	1/1206 (<0.1%)	1/2372 (<0.1%)
	Overall	0/1432	1/1452 (<0.1%)	1/2884 (<0.1%)
Apicectomy	1st year	0/1432	1/1452 (<0.1%)	1/2884 (<0.1%)
	2nd year	0/1166	1/1206 (<0.1%)	1/2372 (<0.1%)
	Overall	0/1432	1/1452 (<0.1%)	1/2884 (<0.1%)
Breast prosthesis implantation	1st year	2/1432 (0.1%)	0/1452	2/2884 (<0.1%)
	2nd year	1/1166 (<0.1%)	0/1206	1/2372 (<0.1%)
	3rd year	0/ 963	1/1012 (<0.1%)	1/1975 (<0.1%)
	Overall	3/1432 (0.2%)	1/1452 (<0.1%)	4/2884 (0.1%)
Bunion operation	1st year	1/1432 (<0.1%)	0/1452	1/2884 (<0.1%)
	2nd year	1/1166 (<0.1%)	0/1206	1/2372 (<0.1%)
	3rd year	0/ 963	1/1012 (<0.1%)	1/1975 (<0.1%)
	Overall	2/1432 (0.1%)	1/1452 (<0.1%)	3/2884 (0.1%)
Cervix cautery	1st year	0/1432	1/1452 (<0.1%)	1/2884 (<0.1%)
	Overall	0/1432	1/1452 (<0.1%)	1/2884 (<0.1%)

Table 14.3.1 / 9: Number of subjects with adverse events by primary system organ class, preferred term and year (FAS)

Primary system organ class<br>Preferred term<br>MedDRA version 14.0	YEAR OF ONSET	LCS12	LCS16	Total
Cholecystectomy	2nd year	1/1166 (<0.1%)	0/1206	1/2372 (<0.1%)
	Overall	1/1432 (<0.1%)	0/1452	1/2884 (<0.1%)
Dental cosmetic procedure	3rd year	1/ 963 (0.1%)	0/1012	1/1975 (<0.1%)
	Overall	1/1432 (<0.1%)	0/1452	1/2884 (<0.1%)
Dental operation	1st year	0/1432	3/1452 (0.2%)	3/2884 (0.1%)
	2nd year	1/1166 (<0.1%)	0/1206	1/2372 (<0.1%)
	Overall	1/1432 (<0.1%)	3/1452 (0.2%)	4/2884 (0.1%)
Detoxification	2nd year	0/1166	1/1206 (<0.1%)	1/2372 (<0.1%)
	3rd year	1/ 963 (0.1%)	0/1012	1/1975 (<0.1%)
	Overall	1/1432 (<0.1%)	1/1452 (<0.1%)	2/2884 (<0.1%)
Endodontic procedure	1st year	0/1432	1/1452 (<0.1%)	1/2884 (<0.1%)
	2nd year	0/1166	1/1206 (<0.1%)	1/2372 (<0.1%)
	Overall	0/1432	2/1452 (0.1%)	2/2884 (<0.1%)
Eye laser surgery	2nd year	0/1166	1/1206 (<0.1%)	1/2372 (<0.1%)
	Overall	0/1432	1/1452 (<0.1%)	1/2884 (<0.1%)
Gallbladder operation	1st year	0/1432	1/1452 (<0.1%)	1/2884 (<0.1%)
	Overall	0/1432	1/1452 (<0.1%)	1/2884 (<0.1%)
Gastric banding	1st year	1/1432 (<0.1%)	0/1452	1/2884 (<0.1%)
	Overall	1/1432 (<0.1%)	0/1452	1/2884 (<0.1%)
Gastric bypass	2nd year	0/1166	1/1206 (<0.1%)	1/2372 (<0.1%)
	Overall	0/1432	1/1452 (<0.1%)	1/2884 (<0.1%)
Haemorrhoid operation	2nd year	0/1166	1/1206 (<0.1%)	1/2372 (<0.1%)
	Overall	0/1432	1/1452 (<0.1%)	1/2884 (<0.1%)
Keratomileusis	3rd year	0/ 963	1/1012 (<0.1%)	1/1975 (<0.1%)
	Overall	0/1432	1/1452 (<0.1%)	1/2884 (<0.1%)
Ligament operation	3rd year	1/ 963 (0.1%)	0/1012	1/1975 (<0.1%)
	Overall	1/1432 (<0.1%)	0/1452	1/2884 (<0.1%)

Table 14.3.1 / 9: Number of subjects with adverse events by primary system organ class, preferred term and year (FAS)

Primary system organ class<br>Preferred term<br>MedDRA version 14.0	YEAR OF ONSET	LCS12	LCS16	Total
Lipoma excision	2nd year	1/1166 (<0.1%)	0/1206	1/2372 (<0.1%)
	Overall	1/1432 (<0.1%)	0/1452	1/2884 (<0.1%)
Mammoplasty	1st year	2/1432 (0.1%)	0/1452	2/2884 (<0.1%)
	2nd year	2/1166 (0.2%)	0/1206	2/2372 (<0.1%)
	3rd year	3/ 963 (0.3%)	3/1012 (0.3%)	6/1975 (0.3%)
	Overall	6/1432 (0.4%)	3/1452 (0.2%)	9/2884 (0.3%)
Maxillary antrum operation	1st year	1/1432 (<0.1%)	0/1452	1/2884 (<0.1%)
	Overall	1/1432 (<0.1%)	0/1452	1/2884 (<0.1%)
Meniscus operation	2nd year	0/1166	1/1206 (<0.1%)	1/2372 (<0.1%)
	Overall	0/1432	1/1452 (<0.1%)	1/2884 (<0.1%)
Mole excision	1st year	2/1432 (0.1%)	1/1452 (<0.1%)	3/2884 (0.1%)
	2nd year	0/1166	1/1206 (<0.1%)	1/2372 (<0.1%)
	Overall	2/1432 (0.1%)	2/1452 (0.1%)	4/2884 (0.1%)
Nasal polypectomy	1st year	0/1432	1/1452 (<0.1%)	1/2884 (<0.1%)
	Overall	0/1432	1/1452 (<0.1%)	1/2884 (<0.1%)
Parasitic infection prophylaxis	1st year	0/1432	1/1452 (<0.1%)	1/2884 (<0.1%)
	Overall	0/1432	1/1452 (<0.1%)	1/2884 (<0.1%)
Plastic surgery	1st year	0/1432	1/1452 (<0.1%)	1/2884 (<0.1%)
	Overall	0/1432	1/1452 (<0.1%)	1/2884 (<0.1%)
Postoperative analgesia	2nd year	1/1166 (<0.1%)	0/1206	1/2372 (<0.1%)
	Overall	1/1432 (<0.1%)	0/1452	1/2884 (<0.1%)
Rotator cuff repair	1st year	0/1432	1/1452 (<0.1%)	1/2884 (<0.1%)
	2nd year	0/1166	1/1206 (<0.1%)	1/2372 (<0.1%)
	Overall	0/1432	2/1452 (0.1%)	2/2884 (<0.1%)
Scar excision	2nd year	0/1166	1/1206 (<0.1%)	1/2372 (<0.1%)
	Overall	0/1432	1/1452 (<0.1%)	1/2884 (<0.1%)
Skin neoplasm excision	2nd year	0/1166	1/1206 (<0.1%)	1/2372 (<0.1%)
	Overall	0/1432	1/1452 (<0.1%)	1/2884 (<0.1%)

Table 14.3.1 / 9: Number of subjects with adverse events by primary system organ class, preferred term and year (FAS)

Primary system organ class<br>Preferred term<br>MedDRA version 14.0	YEAR OF ONSET	LCS12	LCS16	Total
Tenolysis	3rd year	0/ 963	1/1012 (<0.1%)	1/1975 (<0.1%)
	Overall	0/1432	1/1452 (<0.1%)	1/2884 (<0.1%)
Tonsillectomy	1st year	0/1432	1/1452 (<0.1%)	1/2884 (<0.1%)
	2nd year	1/1166 (<0.1%)	1/1206 (<0.1%)	2/2372 (<0.1%)
	Overall	1/1432 (<0.1%)	2/1452 (0.1%)	3/2884 (0.1%)
Tooth extraction	1st year	1/1432 (<0.1%)	5/1452 (0.3%)	6/2884 (0.2%)
	2nd year	2/1166 (0.2%)	3/1206 (0.2%)	5/2372 (0.2%)
	3rd year	3/ 963 (0.3%)	1/1012 (<0.1%)	4/1975 (0.2%)
	Overall	6/1432 (0.4%)	9/1452 (0.6%)	15/2884 (0.5%)
Umbilical hernia repair	1st year	0/1432	1/1452 (<0.1%)	1/2884 (<0.1%)
	Overall	0/1432	1/1452 (<0.1%)	1/2884 (<0.1%)
Varicose vein operation	3rd year	1/ 963 (0.1%)	0/1012	1/1975 (<0.1%)
	Overall	1/1432 (<0.1%)	0/1452	1/2884 (<0.1%)
Wisdom teeth removal	1st year	6/1432 (0.4%)	3/1452 (0.2%)	9/2884 (0.3%)
	2nd year	1/1166 (<0.1%)	3/1206 (0.2%)	4/2372 (0.2%)
	Overall	7/1432 (0.5%)	5/1452 (0.3%)	12/2884 (0.4%)
Wrist surgery	1st year	0/1432	1/1452 (<0.1%)	1/2884 (<0.1%)
	Overall	0/1432	1/1452 (<0.1%)	1/2884 (<0.1%)
Vascular disorders	1st year	14/1432 (1.0%)	11/1452 (0.8%)	25/2884 (0.9%)
	2nd year	4/1166 (0.3%)	11/1206 (0.9%)	15/2372 (0.6%)
	3rd year	4/ 963 (0.4%)	6/1012 (0.6%)	10/1975 (0.5%)
	Overall	20/1432 (1.4%)	26/1452 (1.8%)	46/2884 (1.6%)
Deep vein thrombosis	1st year	1/1432 (<0.1%)	0/1452	1/2884 (<0.1%)
	Overall	1/1432 (<0.1%)	0/1452	1/2884 (<0.1%)
Hot flush	1st year	4/1432 (0.3%)	1/1452 (<0.1%)	5/2884 (0.2%)
	2nd year	0/1166	1/1206 (<0.1%)	1/2372 (<0.1%)
	3rd year	0/ 963	2/1012 (0.2%)	2/1975 (0.1%)
	Overall	4/1432 (0.3%)	3/1452 (0.2%)	7/2884 (0.2%)

Table 14.3.1 / 9: Number of subjects with adverse events by primary system organ class, preferred term and year (FAS)

Primary system organ class<br>Preferred term<br>MedDRA version 14.0	YEAR OF<br>ONSET	LCS12	LCS16	Total
Hypertension	1st year	6/1432 (0.4%)	6/1452 (0.4%)	12/2884 (0.4%)
	2nd year	3/1166 (0.3%)	7/1206 (0.6%)	10/2372 (0.4%)
	3rd year	3/ 963 (0.3%)	3/1012 (0.3%)	6/1975 (0.3%)
	Overall	11/1432 (0.8%)	16/1452 (1.1%)	27/2884 (0.9%)
Hypotension	1st year	1/1432 (<0.1%)	2/1452 (0.1%)	3/2884 (0.1%)
	Overall	1/1432 (<0.1%)	2/1452 (0.1%)	3/2884 (0.1%)
Neurogenic shock	1st year	2/1432 (0.1%)	0/1452	2/2884 (<0.1%)
	Overall	2/1432 (0.1%)	0/1452	2/2884 (<0.1%)
Phlebitis	2nd year	0/1166	1/1206 (<0.1%)	1/2372 (<0.1%)
	Overall	0/1432	1/1452 (<0.1%)	1/2884 (<0.1%)
Thrombophlebitis superficial	2nd year	1/1166 (<0.1%)	0/1206	1/2372 (<0.1%)
	Overall	1/1432 (<0.1%)	0/1452	1/2884 (<0.1%)
Varicose vein	1st year	0/1432	2/1452 (0.1%)	2/2884 (<0.1%)
	2nd year	0/1166	3/1206 (0.2%)	3/2372 (0.1%)
	3rd year	1/ 963 (0.1%)	1/1012 (<0.1%)	2/1975 (0.1%)
	Overall	1/1432 (<0.1%)	5/1452 (0.3%)	6/2884 (0.2%)
Vein pain	2nd year	0/1166	1/1206 (<0.1%)	1/2372 (<0.1%)
	Overall	0/1432	1/1452 (<0.1%)	1/2884 (<0.1%)

Note: A subject is counted only once within each preferred term of any primary SOC.
Note: Denominator is number of subjects available at the START of the year or all subjects for overall
Note: Adverse events are sorted in alphabetical order by primary SOC and preferred term.
Global Biostatistics: /by-sasp/patdb/projects/de04209/310442/stat/prod_interim03/pgms/t-ae-cat.sas epkll 12OCT2011 11:21
End of table

Table 14.3.1 / 10: Number of subjects with adverse events including subject identifier and year by treatment (FAS)

TREATMENT: LCS12

Primary system organ class<br>Preferred term<br>MedDRA version 14.0	YEAR OF ONSET	N (%)	subject identifier
Number of subjects (%) with at least one adverse event	1st year	1035/1432 (72.3%)	
	2nd year	603/1166 (51.7%)	
	3rd year	466/ 963 (48.4%)	
	Overall	1194/1432 (83.4%)	
Blood and lymphatic system disorders	1st year	10/1432 (0.7%)	
	2nd year	3/1166 (0.3%)	
	3rd year	3/ 963 (0.3%)	
	Overall	16/1432 (1.1%)	
Anaemia	1st year	5/1432 (0.3%)	120417, 161210, 241410, 244125, 246211
	2nd year	1/1166 (<0.1%)	244430
	3rd year	1/ 963 (0.1%)	150142
	Overall	7/1432 (0.5%)	120417, 150142, 161210, 241410, 244125, 244430, 246211
Iron deficiency anaemia	3rd year	1/ 963 (0.1%)	245431
	Overall	1/1432 (<0.1%)	245431
Leukocytosis	1st year	1/1432 (<0.1%)	241410
	Overall	1/1432 (<0.1%)	241410
Lymphadenitis	1st year	2/1432 (0.1%)	160701, 244117
	2nd year	1/1166 (<0.1%)	180630
	Overall	3/1432 (0.2%)	160701, 180630, 244117
Lymphadenopathy	1st year	1/1432 (<0.1%)	140105
	2nd year	1/1166 (<0.1%)	242812
	3rd year	1/ 963 (0.1%)	210124
	Overall	3/1432 (0.2%)	140105, 210124, 242812
Spherocytic anaemia	1st year	1/1432 (<0.1%)	243938
	Overall	1/1432 (<0.1%)	243938
Splenic cyst	1st year	1/1432 (<0.1%)	120104
	Overall	1/1432 (<0.1%)	120104

Table 14.3.1 / 10: Number of subjects with adverse events including subject identifier and year by treatment (FAS)

TREATMENT: LCS12

Primary system organ class / Preferred term / MedDRA version 14.0	YEAR OF ONSET	N (%)	subject identifier
Splenomegaly	1st year	1/1432 (<0.1%)	243938
	Overall	1/1432 (<0.1%)	243938
Cardiac disorders	1st year	2/1432 (0.1%)	
	2nd year	4/1166 (0.3%)	
	3rd year	3/ 963 (0.3%)	
	Overall	9/1432 (0.6%)	
Arrhythmia	1st year	1/1432 (<0.1%)	160551
	Overall	1/1432 (<0.1%)	160551
Palpitations	2nd year	1/1166 (<0.1%)	160541
	3rd year	3/ 963 (0.3%)	161440, 161517, 244310
	Overall	4/1432 (0.3%)	160541, 161440, 161517, 244310
Tachycardia	1st year	1/1432 (<0.1%)	160401
	2nd year	3/1166 (0.3%)	180620, 180686, 230401
	Overall	4/1432 (0.3%)	160401, 180620, 180686, 230401
Congenital, familial and genetic disorders	2nd year	3/1166 (0.3%)	
	Overall	3/1432 (0.2%)	
Congenital hydronephrosis	2nd year	1/1166 (<0.1%)	190126
	Overall	1/1432 (<0.1%)	190126
Dermoid cyst	2nd year	1/1166 (<0.1%)	244407
	Overall	1/1432 (<0.1%)	244407
Urethral intrinsic sphincter deficiency	2nd year	1/1166 (<0.1%)	246220
	Overall	1/1432 (<0.1%)	246220
Ear and labyrinth disorders	1st year	9/1432 (0.6%)	
	2nd year	5/1166 (0.4%)	
	3rd year	3/ 963 (0.3%)	
	Overall	16/1432 (1.1%)	
Deafness bilateral	2nd year	1/1166 (<0.1%)	242806
	Overall	1/1432 (<0.1%)	242806

Table 14.3.1 / 10: Number of subjects with adverse events including subject identifier and year by treatment (FAS)

TREATMENT: LCS12

Primary system organ class<br>Preferred term<br>MedDRA version 14.0	YEAR OF ONSET	N (%)	subject identifier
Ear pruritus	1st year	1/1432 (<0.1%)	140802
	Overall	1/1432 (<0.1%)	140802
Motion sickness	1st year	3/1432 (0.2%)	160415, 161316, 190135
	3rd year	1/ 963 (0.1%)	243913
	Overall	4/1432 (0.3%)	160415, 161316, 190135, 243913
Otorrhoea	3rd year	1/ 963 (0.1%)	241406
	Overall	1/1432 (<0.1%)	241406
Sudden hearing loss	3rd year	1/ 963 (0.1%)	230205
	Overall	1/1432 (<0.1%)	230205
Tinnitus	2nd year	2/1166 (0.2%)	120632, 161117
	Overall	2/1432 (0.1%)	120632, 161117
Tympanic membrane perforation	1st year	1/1432 (<0.1%)	243327
	2nd year	1/1166 (<0.1%)	200923
	Overall	2/1432 (0.1%)	200923, 243327
Vertigo	1st year	3/1432 (0.2%)	150110, 180624, 241193
	2nd year	1/1166 (<0.1%)	242806
	Overall	4/1432 (0.3%)	150110, 180624, 241193, 242806
Vertigo labyrinthine	1st year	1/1432 (<0.1%)	150110
	Overall	1/1432 (<0.1%)	150110
Vertigo positional	1st year	1/1432 (<0.1%)	160803
	2nd year	1/1166 (<0.1%)	140802
	Overall	2/1432 (0.1%)	140802, 160803
Endocrine disorders	1st year	3/1432 (0.2%)	
	2nd year	5/1166 (0.4%)	
	3rd year	2/ 963 (0.2%)	
	Overall	10/1432 (0.7%)	
Goitre	2nd year	1/1166 (<0.1%)	241172
	3rd year	1/ 963 (0.1%)	141419
	Overall	2/1432 (0.1%)	141419, 241172

Table 14.3.1 / 10: Number of subjects with adverse events including subject identifier and year by treatment (FAS)

TREATMENT: LCS12

Primary system organ class<br>Preferred term<br>MedDRA version 14.0	YEAR OF ONSET	N (%)	subject identifier
Hypothyroidism	1st year	2/1432 (0.1%)	120403, 240352
	2nd year	4/1166 (0.3%)	120305, 231005, 241536, 242807
	3rd year	1/ 963 (0.1%)	240828
	Overall	7/1432 (0.5%)	120305, 120403, 231005, 240352, 240828, 241536, 242807
Thyroiditis	1st year	1/1432 (<0.1%)	141403
	Overall	1/1432 (<0.1%)	141403
Eye disorders	1st year	14/1432 (1.0%)	
	2nd year	4/1166 (0.3%)	
	3rd year	7/ 963 (0.7%)	
	Overall	25/1432 (1.7%)	
Blepharitis	2nd year	1/1166 (<0.1%)	200601
	Overall	1/1432 (<0.1%)	200601
Conjunctivitis	1st year	11/1432 (0.8%)	140224, 160118, 160321, 160749, 160801, 161109, 161218, 161406, 200506, 240644, 244412
	2nd year	2/1166 (0.2%)	120329, 150142
	3rd year	2/ 963 (0.2%)	160631, 161313
	Overall	15/1432 (1.0%)	120329, 140224, 150142, 160118, 160321, 160631, 160749, 160801, 161109, 161218, 161313, 161406, 200506, 240644, 244412
Conjunctivitis allergic	3rd year	2/ 963 (0.2%)	230308, 230603
	Overall	2/1432 (0.1%)	230308, 230603
Dry eye	1st year	2/1432 (0.1%)	161122, 161403
	Overall	2/1432 (0.1%)	161122, 161403
Heterophoria	2nd year	1/1166 (<0.1%)	230613
	Overall	1/1432 (<0.1%)	230613
Iridocyclitis	1st year	1/1432 (<0.1%)	180501
	Overall	1/1432 (<0.1%)	180501
Meibomianitis	1st year	1/1432 (<0.1%)	160321
	Overall	1/1432 (<0.1%)	160321

Table 14.3.1 / 10: Number of subjects with adverse events including subject identifier and year by treatment (FAS)

TREATMENT: LCS12

Primary system organ class<br>Preferred term<br>MedDRA version 14.0	YEAR OF ONSET	N (%)	subject identifier
Panophthalmitis	3rd year	1/ 963 (0.1%)	160984
	Overall	1/1432 (<0.1%)	160984
Scotoma	3rd year	1/ 963 (0.1%)	140105
	Overall	1/1432 (<0.1%)	140105
Vision blurred	3rd year	1/ 963 (0.1%)	244430
	Overall	1/1432 (<0.1%)	244430
Gastrointestinal disorders	1st year	236/1432 (16.5%)	
	2nd year	81/1166 (6.9%)	
	3rd year	54/ 963 (5.6%)	
	Overall	335/1432 (23.4%)	
Abdominal adhesions	2nd year	1/1166 (<0.1%)	200908
	Overall	1/1432 (<0.1%)	200908
Abdominal discomfort	1st year	5/1432 (0.3%)	200121, 230607, 241805, 243943, 243948
	2nd year	1/1166 (<0.1%)	230607
	Overall	5/1432 (0.3%)	200121, 230607, 241805, 243943, 243948
Abdominal distension	1st year	17/1432 (1.2%)	120201, 141027, 141405, 141409, 160301, 160944, 200521, 210415, 230824, 241154, 243009, 243220, 243224, 243828, 244524, 245903, 245928
	2nd year	3/1166 (0.3%)	190225, 243026, 243903
	3rd year	1/ 963 (0.1%)	161109
	Overall	21/1432 (1.5%)	120201, 141027, 141405, 141409, 160301, 160944, 161109, 190225, 200521, 210415, 230824, 241154, 243009, 243026, 243220, 243224, 243828, 243903, 244524, 245903, 245928
Abdominal hernia	1st year	1/1432 (<0.1%)	240713
	3rd year	1/ 963 (0.1%)	160613
	Overall	2/1432 (0.1%)	160613, 240713
Abdominal mass	1st year	1/1432 (<0.1%)	245911
	Overall	1/1432 (<0.1%)	245911

Table 14.3.1 / 10: Number of subjects with adverse events including subject identifier and year by treatment (FAS)

TREATMENT: LCS12

Primary system organ class<br>Preferred term<br>MedDRA version 14.0	YEAR OF ONSET	N (%)	subject identifier
Abdominal pain	1st year	75/1432 (5.2%)	120104, 120320, 120411, 120412, 120414, 120419, 120611, 120619, 120623, 120626, 120630, 120632, 140105, 140116, 140117, 140118, 141405, 141409, 150149, 160102, 160119, 160425, 160434, 160514, 160536, 160565, 160572, 160601, 160701, 160712, 160717, 160736, 161026, 161316, 161511, 161512, 161531, 161540, 180714, 190403, 200101, 200219, 200503, 200527, 200613, 200623, 200629, 200708, 200908, 200923, 210109, 210136, 210411, 230809, 230901, 231022, 231103, 240622, 240814, 240841, 241169, 241410, 241414, 241424, 241518, 241718, 243121, 243125, 243320, 243939, 243969, 244125, 244438, 245929, 245932
	2nd year	20/1166 (1.7%)	120320, 120411, 140113, 141412, 160205, 160405, 160427, 160440, 161436, 180136, 180733, 200908, 210136, 230401, 240110, 241541, 241725, 243220, 243616, 244314
	3rd year	11/ 963 (1.1%)	140802, 180416, 200916, 210136, 210203, 231014, 240131, 241414, 242320, 244407, 244430
	Overall	100/1432 (7.0%)	120104, 120320, 120411, 120412, 120414, 120419, 120611, 120619, 120623, 120626, 120630, 120632, 140105, 140113, 140116, 140117, 140118, 140802, 141405, 141409, 141412, 150149, 160102, 160119, 160205, 160405, 160425, 160427, 160434, 160440, 160514, 160536, 160565, 160572, 160601, 160701, 160712, 160717, 160736, 161026, 161316, 161436, 161511, 161512, 161531, 161540, 180136, 180416, 180714, 180733, 190403, 200101, 200219, 200503, 200527, 200613, 200623, 200629, 200708, 200908, 200916, 200923, 210109, 210136, 210203, 210411, 230401, 230809, 230901, 231014, 231022, 231103, 240110, 240131, 240622, 240814, 240841, 241169, 241410, 241414, 241424, 241518, 241541, 241718, 241725, 242320, 243121, 243125, 243220, 243320, 243616, 243939, 243969, 244125, 244314, 244407, 244430, 244438, 245929, 245932

Table 14.3.1 / 10: Number of subjects with adverse events including subject identifier and year by treatment (FAS)

TREATMENT: LCS12

Primary system organ class<br>Preferred term<br>MedDRA version 14.0	YEAR OF ONSET	N (%)	subject identifier
Abdominal pain lower	1st year	47/1432 (3.3%)	140301, 160106, 160110, 160130, 160212, 160213, 160217, 160415, 160514, 160525, 160536, 160576, 160605, 160615, 160640, 160911, 160925, 160933, 160949, 160976, 161001, 161102, 161124, 161318, 161440, 161518, 161540, 180605, 180673, 190110, 190133, 200111, 200802, 200904, 200908, 240521, 240627, 241507, 241811, 242227, 243327, 243611, 243806, 243807, 244117, 244602, 246121
	2nd year	14/1166 (1.2%)	160918, 160988, 161504, 161514, 200709, 241130, 241133, 241525, 241548, 243103, 243314, 243320, 244610, 245923
	3rd year	8/ 963 (0.8%)	160113, 160525, 160633, 160949, 161109, 161322, 190132, 240715
	Overall	67/1432 (4.7%)	140301, 160106, 160110, 160113, 160130, 160212, 160213, 160217, 160415, 160514, 160525, 160536, 160576, 160605, 160615, 160633, 160640, 160911, 160918, 160925, 160933, 160949, 160976, 160988, 161001, 161102, 161109, 161124, 161318, 161322, 161440, 161504, 161514, 161518, 161540, 180605, 180673, 190110, 190132, 190133, 200111, 200709, 200802, 200904, 200908, 240521, 240627, 240715, 241130, 241133, 241507, 241525, 241548, 241811, 242227, 243103, 243314, 243320, 243327, 243611, 243806, 243807, 244117, 244602, 244610, 245923, 246121
Abdominal pain upper	1st year	5/1432 (0.3%)	120310, 120320, 200517, 200619, 230604
	2nd year	1/1166 (<0.1%)	243617
	3rd year	1/ 963 (0.1%)	160631
	Overall	7/1432 (0.5%)	120310, 120320, 160631, 200517, 200619, 230604, 243617
Abdominal tenderness	1st year	2/1432 (0.1%)	244117, 245929
	Overall	2/1432 (0.1%)	244117, 245929
Anal fissure	2nd year	1/1166 (<0.1%)	141004
	Overall	1/1432 (<0.1%)	141004

Table 14.3.1 / 10: Number of subjects with adverse events including subject identifier and year by treatment (FAS)

TREATMENT: LCS12

Primary system organ class<br>Preferred term<br>MedDRA version 14.0	YEAR OF ONSET	N (%)	subject identifier
Anal pruritus	1st year	1/1432 (<0.1%)	160734
	2nd year	1/1166 (<0.1%)	160567
	3rd year	1/ 963 (0.1%)	161117
	Overall	3/1432 (0.2%)	160567, 160734, 161117
Breath odour	2nd year	1/1166 (<0.1%)	161410
	Overall	1/1432 (<0.1%)	161410
Coeliac disease	2nd year	1/1166 (<0.1%)	242907
	Overall	1/1432 (<0.1%)	242907
Colitis	2nd year	1/1166 (<0.1%)	190411
	Overall	1/1432 (<0.1%)	190411
Colitis ulcerative	1st year	1/1432 (<0.1%)	140202
	2nd year	1/1166 (<0.1%)	140212
	Overall	2/1432 (0.1%)	140202, 140212
Colonic polyp	2nd year	1/1166 (<0.1%)	242212
	Overall	1/1432 (<0.1%)	242212
Constipation	1st year	11/1432 (0.8%)	120104, 200521, 200901, 200908, 210111, 240525, 240825, 241545, 242326, 243121, 244117
	2nd year	3/1166 (0.3%)	141004, 243904, 245916
	3rd year	3/ 963 (0.3%)	240828, 241545, 244412
	Overall	16/1432 (1.1%)	120104, 141004, 200521, 200901, 200908, 210111, 240525, 240825, 240828, 241545, 242326, 243121, 243904, 244117, 244412, 245916
Crohn's disease	1st year	2/1432 (0.1%)	161537, 240209
	Overall	2/1432 (0.1%)	161537, 240209
Dental caries	1st year	1/1432 (<0.1%)	244004
	Overall	1/1432 (<0.1%)	244004

Table 14.3.1 / 10: Number of subjects with adverse events including subject identifier and year by treatment (FAS)

TREATMENT: LCS12

Primary system organ class<br>Preferred term<br>MedDRA version 14.0	YEAR OF ONSET	N (%)	subject identifier
Diarrhoea	1st year	13/1432 (0.9%)	120104, 120417, 120632, 140105, 140111, 141301, 161026, 161410, 210125, 242910, 243125, 243323, 243904
	2nd year	2/1166 (0.2%)	140802, 242902
	3rd year	4/ 963 (0.4%)	120320, 150153, 161017, 161539
	Overall	19/1432 (1.3%)	120104, 120320, 120417, 120632, 140105, 140111, 140802, 141301, 150153, 161017, 161026, 161410, 161539, 210125, 242902, 242910, 243125, 243323, 243904
Duodenal ulcer	3rd year	1/ 963 (0.1%)	150109
	Overall	1/1432 (<0.1%)	150109
Dyspepsia	1st year	6/1432 (0.4%)	160522, 160571, 160806, 210203, 240713, 241536
	2nd year	3/1166 (0.3%)	160102, 160606, 244523
	3rd year	5/ 963 (0.5%)	140302, 141032, 160624, 161109, 161308
	Overall	14/1432 (1.0%)	140302, 141032, 160102, 160522, 160571, 160606, 160624, 160806, 161109, 161308, 210203, 240713, 241536, 244523
Dysphagia	1st year	1/1432 (<0.1%)	244707
	Overall	1/1432 (<0.1%)	244707
Flatulence	1st year	2/1432 (0.1%)	200521, 210411
	Overall	2/1432 (0.1%)	200521, 210411
Food poisoning	1st year	1/1432 (<0.1%)	244518
	Overall	1/1432 (<0.1%)	244518
Frequent bowel movements	1st year	2/1432 (0.1%)	210111, 244524
	Overall	2/1432 (0.1%)	210111, 244524
Gastric ulcer	1st year	1/1432 (<0.1%)	230713
	2nd year	1/1166 (<0.1%)	243978
	Overall	2/1432 (0.1%)	230713, 243978

Table 14.3.1 / 10: Number of subjects with adverse events including subject identifier and year by treatment (FAS)

TREATMENT: LCS12

Primary system organ class<br>Preferred term<br>MedDRA version 14.0	YEAR OF ONSET	N (%)	subject identifier
Gastritis	1st year	6/1432 (0.4%)	161026, 200710, 230310, 230908, 231002, 243125
	2nd year	2/1166 (0.2%)	120119, 200914
	3rd year	5/ 963 (0.5%)	120307, 150109, 200505, 210106, 230318
	Overall	13/1432 (0.9%)	120119, 120307, 150109, 161026, 200505, 200710, 200914, 210106, 230310, 230318, 230908, 231002, 243125
Gastrointestinal disorder	2nd year	1/1166 (<0.1%)	241513
	Overall	1/1432 (<0.1%)	241513
Gastrointestinal pain	1st year	1/1432 (<0.1%)	150123
	Overall	1/1432 (<0.1%)	150123
Gastrooesophageal reflux disease	1st year	6/1432 (0.4%)	140914, 240505, 241536, 243938, 244117, 245910
	2nd year	2/1166 (0.2%)	140802, 244443
	3rd year	1/ 963 (0.1%)	246212
	Overall	9/1432 (0.6%)	140802, 140914, 240505, 241536, 243938, 244117, 244443, 245910, 246212
Gingival oedema	3rd year	1/ 963 (0.1%)	140808
	Overall	1/1432 (<0.1%)	140808
Gingival recession	1st year	1/1432 (<0.1%)	245531
	Overall	1/1432 (<0.1%)	245531
Gingivitis	1st year	1/1432 (<0.1%)	160606
	Overall	1/1432 (<0.1%)	160606
Haematochezia	2nd year	1/1166 (<0.1%)	246119
	Overall	1/1432 (<0.1%)	246119
Haemorrhoids	1st year	3/1432 (0.2%)	140918, 240223, 240818
	2nd year	2/1166 (0.2%)	141031, 160606
	3rd year	2/ 963 (0.2%)	160555, 243914
	Overall	7/1432 (0.5%)	140918, 141031, 160555, 160606, 240223, 240818, 243914

Table 14.3.1 / 10: Number of subjects with adverse events including subject identifier and year by treatment (FAS)

TREATMENT: LCS12

Primary system organ class<br>Preferred term<br>MedDRA version 14.0	YEAR OF<br>ONSET	N (%)	subject identifier
Hyperchlorhydria	1st year	1/1432 (<0.1%)	180707
	2nd year	1/1166 (<0.1%)	180733
	Overall	2/1432 (0.1%)	180707, 180733
Impaired gastric emptying	1st year	1/1432 (<0.1%)	240209
	Overall	1/1432 (<0.1%)	240209
Inguinal hernia	2nd year	1/1166 (<0.1%)	246220
	Overall	1/1432 (<0.1%)	246220
Irritable bowel syndrome	1st year	5/1432 (0.3%)	150104, 150109, 150142, 150166, 240505
	2nd year	4/1166 (0.3%)	150101, 150138, 150168, 244918
	3rd year	2/ 963 (0.2%)	150149, 200505
	Overall	11/1432 (0.8%)	150101, 150104, 150109, 150138, 150142, 150149, 150166, 150168, 200505, 240505, 244918

Table 14.3.1 / 10: Number of subjects with adverse events including subject identifier and year by treatment (FAS)

TREATMENT: LCS12

Primary system organ class<br>Preferred term<br>MedDRA version 14.0	YEAR OF ONSET	N (%)	subject identifier
Nausea	1st year	49/1432 (3.4%)	120320, 120335, 140118, 140206, 140304, 140802, 140808, 141301, 141405, 141409, 150106, 160129, 160311, 160601, 160738, 160944, 160973, 161026, 180342, 190402, 190409, 200505, 200517, 200521, 200629, 230408, 230615, 230816, 230904, 231112, 240217, 240223, 240521, 241119, 241410, 241414, 241536, 241805, 242326, 242807, 243323, 243502, 244117, 244427, 244438, 244711, 244917, 244931, 246205
	2nd year	18/1166 (1.5%)	120623, 140817, 150153, 180409, 231018, 240110, 241019, 241130, 241133, 241513, 242907, 242910, 243222, 243314, 243611, 243965, 245910, 246220
	3rd year	9/ 963 (0.9%)	190402, 210124, 230406, 240110, 240131, 241548, 242812, 243231, 244412
	Overall	74/1432 (5.2%)	120320, 120335, 120623, 140118, 140206, 140304, 140802, 140808, 140817, 141301, 141405, 141409, 150106, 150153, 160129, 160311, 160601, 160738, 160944, 160973, 161026, 180342, 180409, 190402, 190409, 200505, 200517, 200521, 200629, 210124, 230406, 230408, 230615, 230816, 230904, 231018, 231112, 240110, 240131, 240217, 240223, 240521, 241019, 241119, 241130, 241133, 241410, 241414, 241513, 241536, 241548, 241805, 242326, 242807, 242812, 242907, 242910, 243222, 243231, 243314, 243323, 243502, 243611, 243965, 244117, 244412, 244427, 244438, 244711, 244917, 244931, 245910, 246205, 246220
Oesophagitis	3rd year	1/ 963 (0.1%)	161532
	Overall	1/1432 (<0.1%)	161532
Peptic ulcer	2nd year	1/1166 (<0.1%)	240223
	Overall	1/1432 (<0.1%)	240223
Periodontitis	1st year	1/1432 (<0.1%)	190117
	3rd year	1/ 963 (0.1%)	200516
	Overall	2/1432 (0.1%)	190117, 200516

Table 14.3.1 / 10: Number of subjects with adverse events including subject identifier and year by treatment (FAS)

TREATMENT: LCS12

Primary system organ class<br>Preferred term<br>MedDRA version 14.0	YEAR OF ONSET	N (%)	subject identifier
Peritonitis	2nd year	1/1166 (<0.1%)	120330
	Overall	1/1432 (<0.1%)	120330
Proctalgia	2nd year	2/1166 (0.2%)	160606, 243616
	Overall	2/1432 (0.1%)	160606, 243616
Tooth disorder	1st year	3/1432 (0.2%)	120326, 160615, 160924
	Overall	3/1432 (0.2%)	120326, 160615, 160924
Tooth impacted	1st year	1/1432 (<0.1%)	241168
	2nd year	1/1166 (<0.1%)	245914
	Overall	2/1432 (0.1%)	241168, 245914
Toothache	1st year	12/1432 (0.8%)	120310, 120322, 120613, 120623, 120634, 140113, 160624, 160625, 160722, 190203, 241606, 244509
	2nd year	2/1166 (0.2%)	120412, 120623
	3rd year	4/ 963 (0.4%)	161308, 161517, 244407, 245525
	Overall	17/1432 (1.2%)	120310, 120322, 120412, 120613, 120623, 120634, 140113, 160624, 160625, 160722, 161308, 161517, 190203, 241606, 244407, 244509, 245525
Vomiting	1st year	12/1432 (0.8%)	140808, 160511, 161023, 200517, 240707, 241410, 241536, 243323, 244117, 244403, 244438, 244711
	2nd year	4/1166 (0.3%)	120602, 160405, 243222, 244523
	3rd year	1/ 963 (0.1%)	242812
	Overall	17/1432 (1.2%)	120602, 140808, 160405, 160511, 161023, 200517, 240707, 241410, 241536, 242812, 243222, 243323, 244117, 244403, 244438, 244523, 244711
General disorders and administration site conditions	1st year	63/1432 (4.4%)	
	2nd year	34/1166 (2.9%)	
	3rd year	22/ 963 (2.3%)	
	Overall	113/1432 (7.9%)	
Asthenia	1st year	1/1432 (<0.1%)	243702
	2nd year	1/1166 (<0.1%)	150108
	Overall	2/1432 (0.1%)	150108, 243702

Table 14.3.1 / 10: Number of subjects with adverse events including subject identifier and year by treatment (FAS)

TREATMENT: LCS12

Primary system organ class<br>Preferred term<br>MedDRA version 14.0	YEAR OF ONSET	N (%)	subject identifier
Axillary pain	3rd year	1/ 963 (0.1%)	244430
	Overall	1/1432 (<0.1%)	244430
Chest pain	1st year	2/1432 (0.1%)	242320, 243219
	2nd year	3/1166 (0.3%)	160625, 240739, 242506
	3rd year	1/ 963 (0.1%)	242320
	Overall	5/1432 (0.3%)	160625, 240739, 242320, 242506, 243219
Cyst	1st year	1/1432 (<0.1%)	242714
	2nd year	2/1166 (0.2%)	140802, 141022
	Overall	3/1432 (0.2%)	140802, 141022, 242714
Device dislocation	1st year	2/1432 (0.1%)	240808, 241733
	3rd year	1/ 963 (0.1%)	140605
	Overall	3/1432 (0.2%)	140605, 240808, 241733
Device expulsion	1st year	25/1432 (1.7%)	141416, 150155, 160309, 180315, 180636, 190218, 200604, 230312, 240351, 240364, 240625, 240638, 241187, 242909, 243803, 243810, 244003, 244316, 244601, 244937, 245907, 245932, 245934, 246121, 246125
	2nd year	9/1166 (0.8%)	120240, 120242, 160220, 161431, 180302, 200705, 210519, 244109, 245402
	3rd year	12/ 963 (1.2%)	120329, 120623, 141426, 190635, 230816, 240634, 241201, 241704, 243511, 244422, 245415, 246212
	Overall	46/1432 (3.2%)	120240, 120242, 120329, 120623, 141416, 141426, 150155, 160220, 160309, 161431, 180302, 180315, 180636, 190218, 190635, 200604, 200705, 210519, 230312, 230816, 240351, 240364, 240625, 240634, 240638, 241187, 241201, 241704, 242909, 243511, 243803, 243810, 244003, 244109, 244316, 244422, 244601, 244937, 245402, 245415, 245907, 245932, 245934, 246121, 246125, 246212
Discomfort	1st year	1/1432 (<0.1%)	160984
	2nd year	1/1166 (<0.1%)	200908
	Overall	2/1432 (0.1%)	160984, 200908

Table 14.3.1 / 10: Number of subjects with adverse events including subject identifier and year by treatment (FAS)

TREATMENT: LCS12

Primary system organ class Preferred term MedDRA version 14.0	YEAR OF ONSET	N (%)	subject identifier
Drug withdrawal syndrome	3rd year	1/ 963 (0.1%)	241708
	Overall	1/1432 (<0.1%)	241708
Fatigue	1st year	12/1432 (0.8%)	140515, 141402, 141405, 141426, 180624, 200505, 230515, 240217, 241410, 243213, 244524, 244618
	2nd year	8/1166 (0.7%)	140802, 241305, 241704, 243213, 243611, 243830, 244931, 245910
	3rd year	5/ 963 (0.5%)	140911, 160519, 200521, 241130, 241302
	Overall	24/1432 (1.7%)	140515, 140802, 140911, 141402, 141405, 141426, 160519, 180624, 200505, 200521, 230515, 240217, 241130, 241302, 241305, 241410, 241704, 243213, 243611, 243830, 244524, 244618, 244931, 245910
Generalised oedema	3rd year	1/ 963 (0.1%)	244105
	Overall	1/1432 (<0.1%)	244105
Hangover	1st year	1/1432 (<0.1%)	160749
	Overall	1/1432 (<0.1%)	160749
Inflammation	1st year	1/1432 (<0.1%)	200908
	Overall	1/1432 (<0.1%)	200908
Influenza like illness	2nd year	1/1166 (<0.1%)	170702
	Overall	1/1432 (<0.1%)	170702
Irritability	1st year	2/1432 (0.1%)	240309, 241193
	Overall	2/1432 (0.1%)	240309, 241193
Oedema peripheral	1st year	3/1432 (0.2%)	120401, 140301, 141405
	2nd year	1/1166 (<0.1%)	160564
	Overall	4/1432 (0.3%)	120401, 140301, 141405, 160564
Pain	1st year	5/1432 (0.3%)	160524, 200901, 241410, 244931, 245027
	2nd year	1/1166 (<0.1%)	242907
	Overall	6/1432 (0.4%)	160524, 200901, 241410, 242907, 244931, 245027

Table 14.3.1 / 10: Number of subjects with adverse events including subject identifier and year by treatment (FAS)

TREATMENT: LCS12

Primary system organ class<br>Preferred term<br>MedDRA version 14.0	YEAR OF ONSET	N (%)	subject identifier
Pyrexia	1st year	10/1432 (0.7%)	160506, 210404, 210409, 231017, 241202, 241410, 241424, 241536, 243322, 244117
	2nd year	7/1166 (0.6%)	160623, 160633, 161104, 190636, 200624, 210119, 242910
	3rd year	1/ 963 (0.1%)	120623
	Overall	18/1432 (1.3%)	120623, 160506, 160623, 160633, 161104, 190636, 200624, 210119, 210404, 210409, 231017, 241202, 241410, 241424, 241536, 242910, 243322, 244117
Vaccination site pain	2nd year	1/1166 (<0.1%)	160427
	Overall	1/1432 (<0.1%)	160427
Hepatobiliary disorders	1st year	5/1432 (0.3%)	
	2nd year	2/1166 (0.2%)	
	3rd year	2/ 963 (0.2%)	
	Overall	8/1432 (0.6%)	
Biliary dyskinesia	3rd year	1/ 963 (0.1%)	230201
	Overall	1/1432 (<0.1%)	230201
Cholecystitis	1st year	1/1432 (<0.1%)	240215
	3rd year	2/ 963 (0.2%)	230201, 242308
	Overall	3/1432 (0.2%)	230201, 240215, 242308
Cholecystitis chronic	1st year	1/1432 (<0.1%)	241536
	2nd year	1/1166 (<0.1%)	243617
	Overall	2/1432 (0.1%)	241536, 243617
Cholelithiasis	1st year	3/1432 (0.2%)	160520, 230201, 243938
	2nd year	1/1166 (<0.1%)	240739
	Overall	4/1432 (0.3%)	160520, 230201, 240739, 243938
Gallbladder polyp	1st year	1/1432 (<0.1%)	240215
	Overall	1/1432 (<0.1%)	240215
Immune system disorders	1st year	39/1432 (2.7%)	
	2nd year	25/1166 (2.1%)	
	3rd year	18/ 963 (1.9%)	
	Overall	63/1432 (4.4%)	

Table 14.3.1 / 10: Number of subjects with adverse events including subject identifier and year by treatment (FAS)

TREATMENT: LCS12

Primary system organ class<br>Preferred term<br>MedDRA version 14.0	YEAR OF ONSET	N (%)	subject identifier
Allergy to animal	3rd year	1/ 963 (0.1%)	140105
	Overall	1/1432 (<0.1%)	140105
Allergy to arthropod bite	1st year	1/1432 (<0.1%)	161003
	Overall	1/1432 (<0.1%)	161003
Allergy to chemicals	1st year	1/1432 (<0.1%)	140105
	Overall	1/1432 (<0.1%)	140105
Drug hypersensitivity	1st year	5/1432 (0.3%)	160617, 160935, 161517, 200901, 243929
	2nd year	2/1166 (0.2%)	160806, 200908
	3rd year	2/ 963 (0.2%)	242320, 245701
	Overall	9/1432 (0.6%)	160617, 160806, 160935, 161517, 200901, 200908, 242320, 243929, 245701
Food allergy	1st year	1/1432 (<0.1%)	242308
	Overall	1/1432 (<0.1%)	242308
Hypersensitivity	1st year	8/1432 (0.6%)	120312, 120403, 120604, 120621, 120633, 140221, 161210, 180707
	2nd year	3/1166 (0.3%)	120623, 161202, 241302
	3rd year	3/ 963 (0.3%)	140511, 161202, 244523
	Overall	13/1432 (0.9%)	120312, 120403, 120604, 120621, 120623, 120633, 140221, 140511, 161202, 161210, 180707, 241302, 244523

Table 14.3.1 / 10: Number of subjects with adverse events including subject identifier and year by treatment (FAS)

TREATMENT: LCS12

Primary system organ class / Preferred term / MedDRA version 14.0	YEAR OF ONSET	N (%)	subject identifier
Seasonal allergy	1st year	24/1432 (1.7%)	140215, 160212, 160416, 160525, 160615, 160717, 160741, 160801, 160917, 160951, 161003, 161015, 161018, 161316, 161410, 161414, 161424, 161426, 200506, 231108, 241153, 243919, 244307, 244422
	2nd year	20/1166 (1.7%)	140302, 160416, 160569, 160717, 160741, 160944, 160951, 161316, 161407, 161410, 161414, 161424, 161426, 180734, 241305, 241704, 243322, 244608, 245402, 245808
	3rd year	12/ 963 (1.2%)	160416, 160525, 160616, 160741, 160951, 161316, 161410, 161414, 161426, 180511, 244430, 244607
	Overall	39/1432 (2.7%)	140215, 140302, 160212, 160416, 160525, 160569, 160615, 160616, 160717, 160741, 160801, 160917, 160944, 160951, 161003, 161015, 161018, 161316, 161407, 161410, 161414, 161424, 161426, 180511, 180734, 200506, 231108, 241153, 241305, 241704, 243322, 243919, 244307, 244422, 244430, 244607, 244608, 245402, 245808
Infections and infestations	1st year	534/1432 (37.3%)	
	2nd year	320/1166 (27.4%)	
	3rd year	228/ 963 (23.7%)	
	Overall	722/1432 (50.4%)	
Acarodermatitis	2nd year	1/1166 (<0.1%)	200911
	3rd year	1/ 963 (0.1%)	120619
	Overall	2/1432 (0.1%)	120619, 200911
Acute sinusitis	1st year	1/1432 (<0.1%)	160985
	2nd year	3/1166 (0.3%)	160548, 160749, 160754
	3rd year	4/ 963 (0.4%)	160749, 161012, 161221, 241305
	Overall	7/1432 (0.5%)	160548, 160749, 160754, 160985, 161012, 161221, 241305
Acute tonsillitis	1st year	8/1432 (0.6%)	150138, 160217, 160520, 160539, 160564, 160729, 161304, 170901
	2nd year	1/1166 (<0.1%)	161202
	3rd year	1/ 963 (0.1%)	120401
	Overall	10/1432 (0.7%)	120401, 150138, 160217, 160520, 160539, 160564, 160729, 161202, 161304, 170901

Table 14.3.1 / 10: Number of subjects with adverse events including subject identifier and year by treatment (FAS)

TREATMENT: LCS12

Primary system organ class<br>Preferred term<br>MedDRA version 14.0	YEAR OF ONSET	N (%)	subject identifier
Anogenital warts	1st year	8/1432 (0.6%)	120623, 210136, 210409, 230405, 243609, 243907, 244509, 245923
	2nd year	3/1166 (0.3%)	160406, 200505, 243925
	Overall	11/1432 (0.8%)	120623, 160406, 200505, 210136, 210409, 230405, 243609, 243907, 243925, 244509, 245923
Appendicitis	1st year	4/1432 (0.3%)	150111, 160416, 241106, 241172
	2nd year	2/1166 (0.2%)	120330, 160973
	Overall	6/1432 (0.4%)	120330, 150111, 160416, 160973, 241106, 241172
Arthritis rubella	2nd year	1/1166 (<0.1%)	170306
	Overall	1/1432 (<0.1%)	170306
Bartholin's abscess	1st year	2/1432 (0.1%)	243933, 244617
	3rd year	1/ 963 (0.1%)	240226
	Overall	3/1432 (0.2%)	240226, 243933, 244617
Body tinea	1st year	1/1432 (<0.1%)	210111
	3rd year	1/ 963 (0.1%)	140802
	Overall	2/1432 (0.1%)	140802, 210111
Breast abscess	1st year	1/1432 (<0.1%)	243030
	Overall	1/1432 (<0.1%)	243030

Table 14.3.1 / 10: Number of subjects with adverse events including subject identifier and year by treatment (FAS)

TREATMENT: LCS12

Primary system organ class<br>Preferred term<br>MedDRA version 14.0	YEAR OF ONSET	N (%)	subject identifier
Bronchitis	1st year	32/1432 (2.2%)	120403, 120632, 141032, 150108, 150123, 160207, 160434, 160436, 160576, 160610, 160640, 160720, 160924, 160954, 160985, 161532, 200703, 200912, 210411, 240312, 240710, 240715, 241148, 241167, 241185, 241421, 243919, 243941, 243978, 244511, 245701, 245903
	2nd year	17/1166 (1.5%)	140111, 140301, 150110, 160328, 160576, 160609, 160610, 160910, 160928, 160954, 240337, 240627, 240826, 241113, 243206, 243978, 245531
	3rd year	15/ 963 (1.6%)	120401, 120403, 140811, 160416, 160551, 160576, 160623, 160624, 160717, 160729, 161303, 180715, 180735, 240739, 244105
	Overall	58/1432 (4.1%)	120401, 120403, 120632, 140111, 140301, 140811, 141032, 150108, 150110, 150123, 160207, 160328, 160416, 160434, 160436, 160551, 160576, 160609, 160610, 160623, 160624, 160640, 160717, 160720, 160729, 160910, 160924, 160928, 160954, 160985, 161303, 161532, 180715, 180735, 200703, 200912, 210411, 240312, 240337, 240627, 240710, 240715, 240739, 240826, 241113, 241148, 241167, 241185, 241421, 243206, 243919, 243941, 243978, 244105, 244511, 245531, 245701, 245903
Bronchitis viral	2nd year	1/1166 (<0.1%)	160328
	Overall	1/1432 (<0.1%)	160328
Bronchopneumonia	1st year	1/1432 (<0.1%)	140117
	Overall	1/1432 (<0.1%)	140117
Bursitis infective	1st year	1/1432 (<0.1%)	160520
	Overall	1/1432 (<0.1%)	160520
Campylobacter infection	1st year	1/1432 (<0.1%)	140817
	Overall	1/1432 (<0.1%)	140817

Table 14.3.1 / 10: Number of subjects with adverse events including subject identifier and year by treatment (FAS)

TREATMENT: LCS12

Primary system organ class<br>Preferred term<br>MedDRA version 14.0	YEAR OF ONSET	N (%)	subject identifier
Candidiasis	1st year	10/1432 (0.7%)	160323, 160954, 200705, 200910, 230412, 244302, 246104, 246121, 246122, 246125
	2nd year	2/1166 (0.2%)	160954, 200910
	3rd year	1/ 963 (0.1%)	160328
	Overall	11/1432 (0.8%)	160323, 160328, 160954, 200705, 200910, 230412, 244302, 246104, 246121, 246122, 246125
Cellulitis	1st year	1/1432 (<0.1%)	120613
	2nd year	1/1166 (<0.1%)	241536
	3rd year	1/ 963 (0.1%)	243323
	Overall	3/1432 (0.2%)	120613, 241536, 243323
Cervicitis	1st year	2/1432 (0.1%)	190118, 243979
	2nd year	2/1166 (0.2%)	140605, 245413
	3rd year	1/ 963 (0.1%)	210125
	Overall	5/1432 (0.3%)	140605, 190118, 210125, 243979, 245413
Cervicitis gonococcal	2nd year	1/1166 (<0.1%)	244710
	Overall	1/1432 (<0.1%)	244710
Chlamydial cervicitis	1st year	1/1432 (<0.1%)	243720
	2nd year	1/1166 (<0.1%)	244710
	Overall	2/1432 (0.1%)	243720, 244710
Chlamydial infection	2nd year	1/1166 (<0.1%)	244422
	Overall	1/1432 (<0.1%)	244422
Clitoris abscess	3rd year	1/ 963 (0.1%)	210411
	Overall	1/1432 (<0.1%)	210411
Conjunctivitis bacterial	3rd year	1/ 963 (0.1%)	160633
	Overall	1/1432 (<0.1%)	160633
Conjunctivitis infective	1st year	2/1432 (0.1%)	245701, 245911
	Overall	2/1432 (0.1%)	245701, 245911
Conjunctivitis viral	1st year	1/1432 (<0.1%)	120632
	Overall	1/1432 (<0.1%)	120632

Table 14.3.1 / 10: Number of subjects with adverse events including subject identifier and year by treatment (FAS)

TREATMENT: LCS12

Primary system organ class<br>Preferred term<br>MedDRA version 14.0	YEAR OF ONSET	N (%)	subject identifier
Coxsackie viral infection	1st year	1/1432 (<0.1%)	161316
	2nd year	1/1166 (<0.1%)	160734
	Overall	2/1432 (0.1%)	160734, 161316
Cystitis	1st year	20/1432 (1.4%)	120305, 120322, 120401, 140118, 140608, 141416, 160402, 160427, 161221, 180120, 180352, 180701, 180728, 180733, 190126, 200104, 210203, 230509, 243903, 245506
	2nd year	8/1166 (0.7%)	120411, 120630, 141006, 160402, 160952, 161202, 170606, 190126
	3rd year	6/ 963 (0.6%)	160402, 180707, 180728, 200601, 200901, 210119
	Overall	30/1432 (2.1%)	120305, 120322, 120401, 120411, 120630, 140118, 140608, 141006, 141416, 160402, 160427, 160952, 161202, 161221, 170606, 180120, 180352, 180701, 180707, 180728, 180733, 190126, 200104, 200601, 200901, 210119, 210203, 230509, 243903, 245506
Dengue fever	1st year	1/1432 (<0.1%)	160415
	2nd year	1/1166 (<0.1%)	190118
	Overall	2/1432 (0.1%)	160415, 190118
Diarrhoea infectious	1st year	1/1432 (<0.1%)	244511
	Overall	1/1432 (<0.1%)	244511
Diverticulitis	1st year	1/1432 (<0.1%)	240521
	2nd year	1/1166 (<0.1%)	240521
	Overall	1/1432 (<0.1%)	240521

Table 14.3.1 / 10: Number of subjects with adverse events including subject identifier and year by treatment (FAS)

TREATMENT: LCS12

Primary system organ class<br>Preferred term<br>MedDRA version 14.0	YEAR OF ONSET	N (%)	subject identifier
Ear infection	1st year	15/1432 (1.0%)	120626, 140224, 140811, 150110, 160548, 160564, 160625, 160973, 161203, 200908, 230515, 240520, 240813, 241406, 241536
	2nd year	9/1166 (0.8%)	160416, 160625, 161308, 200625, 200923, 210136, 243222, 243806, 244426
	3rd year	6/ 963 (0.6%)	140502, 160416, 160625, 160911, 161316, 243214
	Overall	27/1432 (1.9%)	120626, 140224, 140502, 140811, 150110, 160416, 160548, 160564, 160625, 160911, 160973, 161203, 161308, 161316, 200625, 200908, 200923, 210136, 230515, 240520, 240813, 241406, 241536, 243214, 243222, 243806, 244426
Ear infection bacterial	1st year	1/1432 (<0.1%)	243903
	Overall	1/1432 (<0.1%)	243903
Endometritis	1st year	9/1432 (0.6%)	160434, 160637, 160705, 160732, 180630, 180644, 180668, 230809, 243910
	2nd year	1/1166 (<0.1%)	160705
	3rd year	2/ 963 (0.2%)	140520, 243012
	Overall	11/1432 (0.8%)	140520, 160434, 160637, 160705, 160732, 180630, 180644, 180668, 230809, 243012, 243910
Enterobiasis	2nd year	1/1166 (<0.1%)	161407
	3rd year	2/ 963 (0.2%)	160129, 161517
	Overall	3/1432 (0.2%)	160129, 161407, 161517
Enterocolitis infectious	1st year	1/1432 (<0.1%)	120419
	2nd year	1/1166 (<0.1%)	242212
	Overall	2/1432 (0.1%)	120419, 242212
Erysipelas	2nd year	1/1166 (<0.1%)	120411
	Overall	1/1432 (<0.1%)	120411
Erythema infectiosum	2nd year	1/1166 (<0.1%)	140808
	Overall	1/1432 (<0.1%)	140808
Escherichia vaginitis	1st year	1/1432 (<0.1%)	242307
	Overall	1/1432 (<0.1%)	242307

Table 14.3.1 / 10: Number of subjects with adverse events including subject identifier and year by treatment (FAS)

TREATMENT: LCS12

Primary system organ class<br>Preferred term<br>MedDRA version 14.0	YEAR OF ONSET	N (%)	subject identifier
Eyelid infection	2nd year	1/1166 (<0.1%)	161018
	Overall	1/1432 (<0.1%)	161018
Folliculitis	1st year	4/1432 (0.3%)	140113, 140608, 150167, 241606
	2nd year	1/1166 (<0.1%)	160566
	Overall	5/1432 (0.3%)	140113, 140608, 150167, 160566, 241606
Fungal infection	1st year	5/1432 (0.3%)	160564, 240357, 241185, 241424, 241547
	Overall	5/1432 (0.3%)	160564, 240357, 241185, 241424, 241547
Fungal skin infection	1st year	1/1432 (<0.1%)	230602
	2nd year	2/1166 (0.2%)	120320, 120411
	Overall	3/1432 (0.2%)	120320, 120411, 230602
Furuncle	1st year	1/1432 (<0.1%)	243720
	Overall	1/1432 (<0.1%)	243720
Gastroenteritis	1st year	19/1432 (1.3%)	120315, 120608, 120611, 140114, 140219, 160121, 160129, 160416, 160637, 160757, 160806, 160973, 161109, 161111, 161312, 161408, 200919, 230303, 231012
	2nd year	9/1166 (0.8%)	120315, 150101, 160110, 160118, 160548, 160952, 161316, 161420, 161424
	3rd year	6/ 963 (0.6%)	120611, 160106, 160712, 160952, 200911, 245911
	Overall	31/1432 (2.2%)	120315, 120608, 120611, 140114, 140219, 150101, 160106, 160110, 160118, 160121, 160129, 160416, 160548, 160637, 160712, 160757, 160806, 160952, 160973, 161109, 161111, 161312, 161316, 161408, 161420,<br>161424, 200911, 200919, 230303, 231012, 245911
Gastroenteritis viral	1st year	5/1432 (0.3%)	240521, 243307, 243309, 243323, 244412
	3rd year	3/ 963 (0.3%)	200503, 243323, 243946
	Overall	7/1432 (0.5%)	200503, 240521, 243307, 243309, 243323, 243946, 244412
Gastrointestinal viral infection	1st year	1/1432 (<0.1%)	241305
	Overall	1/1432 (<0.1%)	241305

Table 14.3.1 / 10: Number of subjects with adverse events including subject identifier and year by treatment (FAS)

TREATMENT: LCS12

Primary system organ class<br>Preferred term<br>MedDRA version 14.0	YEAR OF ONSET	N (%)	subject identifier
Genital candidiasis	1st year	1/1432 (<0.1%)	230908
	Overall	1/1432 (<0.1%)	230908
Genital herpes	1st year	4/1432 (0.3%)	140210, 141026, 161525, 240625
	2nd year	4/1166 (0.3%)	141026, 160129, 160739, 160928
	3rd year	4/ 963 (0.4%)	140507, 141026, 160418, 160575
	Overall	10/1432 (0.7%)	140210, 140507, 141026, 160129, 160418, 160575, 160739, 160928, 161525, 240625
Genital infection fungal	1st year	1/1432 (<0.1%)	170801
	Overall	1/1432 (<0.1%)	170801
Giardiasis	2nd year	1/1166 (<0.1%)	160566
	Overall	1/1432 (<0.1%)	160566
Gonorrhoea	2nd year	1/1166 (<0.1%)	240623
	Overall	1/1432 (<0.1%)	240623
Groin infection	1st year	1/1432 (<0.1%)	120401
	2nd year	1/1166 (<0.1%)	160567
	Overall	2/1432 (0.1%)	120401, 160567
Gynaecological chlamydia infection	1st year	3/1432 (0.2%)	140221, 230818, 230908
	Overall	3/1432 (0.2%)	140221, 230818, 230908
H1N1 influenza	2nd year	7/1166 (0.6%)	140111, 160606, 160623, 160631, 160753, 170702, 244508
	Overall	7/1432 (0.5%)	140111, 160606, 160623, 160631, 160753, 170702, 244508
Helicobacter gastritis	2nd year	1/1166 (<0.1%)	242907
	Overall	1/1432 (<0.1%)	242907
Helicobacter infection	1st year	1/1432 (<0.1%)	161210
	Overall	1/1432 (<0.1%)	161210
Herpes dermatitis	2nd year	1/1166 (<0.1%)	245431
	3rd year	1/ 963 (0.1%)	245431
	Overall	1/1432 (<0.1%)	245431

Table 14.3.1 / 10: Number of subjects with adverse events including subject identifier and year by treatment (FAS)

TREATMENT: LCS12

Primary system organ class<br>Preferred term<br>MedDRA version 14.0	YEAR OF ONSET	N (%)	subject identifier
Herpes simplex	3rd year	1/ 963 (0.1%)	241015
	Overall	1/1432 (<0.1%)	241015
Herpes zoster	1st year	3/1432 (0.2%)	120401, 160404, 244203
	2nd year	3/1166 (0.3%)	160713, 161001, 161425
	3rd year	1/ 963 (0.1%)	230908
	Overall	7/1432 (0.5%)	120401, 160404, 160713, 161001, 161425, 230908, 244203
Impetigo	1st year	1/1432 (<0.1%)	230223
	2nd year	2/1166 (0.2%)	160928, 161114
	3rd year	1/ 963 (0.1%)	231005
	Overall	4/1432 (0.3%)	160928, 161114, 230223, 231005
Infected bites	1st year	1/1432 (<0.1%)	200709
	2nd year	1/1166 (<0.1%)	160924
	Overall	2/1432 (0.1%)	160924, 200709
Infectious mononucleosis	2nd year	1/1166 (<0.1%)	200104
	Overall	1/1432 (<0.1%)	200104

Table 14.3.1 / 10: Number of subjects with adverse events including subject identifier and year by treatment (FAS)

TREATMENT: LCS12

Primary system organ class<br>Preferred term<br>MedDRA version 14.0	YEAR OF ONSET	N (%)	subject identifier
Influenza	1st year	46/1432 (3.2%)	120305, 120307, 120310, 120315, 120320, 120403, 120409, 120611, 140105, 140117, 140118, 141301, 141421, 160309, 160328, 160525, 160574, 160605, 160610, 160615, 160616, 160617, 160624, 160633, 160637, 161301, 161305, 161313, 161316, 190118, 200506, 200521, 200629, 200703, 210124, 210201, 210409, 230201, 230216, 241307, 241414, 241531, 242316, 242320, 243219, 245528
	2nd year	27/1166 (2.3%)	120315, 120323, 120604, 150151, 160310, 160525, 160574, 160606, 160613, 160615, 160623, 160624, 160631, 160633, 161313, 161316, 200703, 210409, 231012, 240729, 242320, 242506, 243222, 243231, 244515, 244701, 244934
	3rd year	14/ 963 (1.5%)	120305, 120623, 150101, 160328, 160427, 160571, 160624, 160625, 160631, 190128, 200115, 200912, 210101, 240710
	Overall	72/1432 (5.0%)	120305, 120307, 120310, 120315, 120320, 120323, 120403, 120409, 120604, 120611, 120623, 140105, 140117, 140118, 141301, 141421, 150101, 150151, 160309, 160310, 160328, 160427, 160525, 160571, 160574, 160605, 160606, 160610, 160613, 160615, 160616, 160617, 160623, 160624, 160625, 160631, 160633, 160637, 161301, 161305, 161313, 161316, 190118, 190128, 200115, 200506, 200521, 200629, 200703, 200912, 210101, 210124, 210201, 210409, 230201, 230216, 231012, 240710, 240729, 241307, 241414, 241531, 242316, 242320, 242506, 243219, 243222, 243231, 244515, 244701, 244934, 245528
Kidney infection	1st year	1/1432 (<0.1%)	244427
	2nd year	1/1166 (<0.1%)	244710
	Overall	2/1432 (0.1%)	244427, 244710
Labyrinthitis	3rd year	1/ 963 (0.1%)	141032
	Overall	1/1432 (<0.1%)	141032

Table 14.3.1 / 10: Number of subjects with adverse events including subject identifier and year by treatment (FAS)

TREATMENT: LCS12

Primary system organ class<br>Preferred term<br>MedDRA version 14.0	YEAR OF ONSET	N (%)	subject identifier
Laryngitis	1st year	1/1432 (<0.1%)	200516
	2nd year	5/1166 (0.4%)	140511, 140519, 140811, 141412, 150123
	3rd year	1/ 963 (0.1%)	245531
	Overall	7/1432 (0.5%)	140511, 140519, 140811, 141412, 150123, 200516, 245531
Localised infection	1st year	1/1432 (<0.1%)	243510
	2nd year	2/1166 (0.2%)	160905, 244427
	3rd year	1/ 963 (0.1%)	246212
	Overall	4/1432 (0.3%)	160905, 243510, 244427, 246212
Lower respiratory tract infection	1st year	2/1432 (0.1%)	140215, 244306
	2nd year	1/1166 (<0.1%)	244310
	Overall	3/1432 (0.2%)	140215, 244306, 244310
Lower respiratory tract infection bacterial	3rd year	1/ 963 (0.1%)	242506
	Overall	1/1432 (<0.1%)	242506
Lymph gland infection	3rd year	1/ 963 (0.1%)	245506
	Overall	1/1432 (<0.1%)	245506
Malaria	1st year	1/1432 (<0.1%)	161018
	Overall	1/1432 (<0.1%)	161018
Mastitis	1st year	1/1432 (<0.1%)	140518
	3rd year	2/ 963 (0.2%)	120305, 160701
	Overall	3/1432 (0.2%)	120305, 140518, 160701
Meningitis	1st year	1/1432 (<0.1%)	160329
	Overall	1/1432 (<0.1%)	160329
Nail infection	1st year	1/1432 (<0.1%)	160722
	3rd year	1/ 963 (0.1%)	244412
	Overall	2/1432 (0.1%)	160722, 244412

Table 14.3.1 / 10: Number of subjects with adverse events including subject identifier and year by treatment (FAS)

TREATMENT: LCS12

Primary system organ class<br>Preferred term<br>MedDRA version 14.0	YEAR OF ONSET	N (%)	subject identifier
Nasopharyngitis	1st year	70/1432 (4.9%)	120608, 120610, 140111, 140113, 140116, 140228, 140515, 140808, 140817, 140905, 140911, 140921, 141405, 150108, 150149, 150163, 150166, 150168, 160105, 160106, 160108, 160129, 160720, 160757, 160801, 160806, 161305, 161322, 161517, 180703, 180705, 180707, 180734, 180735, 200521, 200911, 210119, 230115, 230120, 230216, 230303, 230314, 230401, 230607, 230713, 230714, 230809, 230816, 230819, 230824, 231012, 231108, 241159, 241307, 241553, 241807, 242313, 242316, 242504, 242906, 243206, 243213, 243919, 243948, 244127, 244518, 244707, 244711, 244909, 245506
	2nd year	35/1166 (3.0%)	120310, 120329, 120611, 140105, 140111, 140218, 140228, 140510, 150151, 150156, 160108, 160118, 160806, 161104, 161305, 161313, 161316, 161322, 210119, 210120, 210201, 210404, 230314, 230318, 230603, 230607, 230819, 231102, 231108, 242504, 242902, 243204, 244105, 244412, 245506
	3rd year	21/ 963 (2.2%)	120326, 120610, 140116, 140518, 140808, 140811, 150123, 150142, 161012, 161305, 161313, 161316, 200521, 200911, 210116, 230114, 230223, 230602, 230819, 243946, 245506
	Overall	103/1432 (7.2%)	120310, 120326, 120329, 120608, 120610, 120611, 140105, 140111, 140113, 140116, 140218, 140228, 140510, 140515, 140518, 140808, 140811, 140817, 140905, 140911, 140921, 141405, 150108, 150123, 150142, 150149, 150151, 150156, 150163, 150166, 150168, 160105, 160106, 160108, 160118, 160129, 160720, 160757, 160801, 160806, 161012, 161104, 161305, 161313, 161316, 161322, 161517, 180703, 180705, 180707, 180734, 180735, 200521, 200911, 210116, 210119, 210120, 210201, 210404, 230114, 230115, 230120, 230216, 230223, 230303, 230314, 230318, 230401, 230602, 230603, 230607, 230713, 230714, 230809, 230816,

Table 14.3.1 / 10: Number of subjects with adverse events including subject identifier and year by treatment (FAS)

TREATMENT: LCS12

Primary system organ class / Preferred term / MedDRA version 14.0	YEAR OF ONSET	N (%)	subject identifier
			230819, 230824, 231012, 231102, 231108, 241159, 241307, 241553, 241807, 242313, 242316, 242504, 242902, 242906, 243204, 243206, 243213, 243919, 243946, 243948, 244105, 244127, 244412, 244518, 244707, 244711, 244909, 245506
Onychomycosis	1st year	2/1432 (0.1%)	120412, 161411
	3rd year	1/ 963 (0.1%)	200528
	Overall	3/1432 (0.2%)	120412, 161411, 200528
Oral herpes	1st year	6/1432 (0.4%)	140116, 150166, 160208, 161205, 210136, 244125
	2nd year	3/1166 (0.3%)	120611, 160967, 241421
	3rd year	3/ 963 (0.3%)	120602, 140228, 241421
	Overall	11/1432 (0.8%)	120602, 120611, 140116, 140228, 150166, 160208, 160967, 161205, 210136, 241421, 244125
Otitis media	1st year	3/1432 (0.2%)	160217, 161202, 244408
	2nd year	1/1166 (<0.1%)	161225
	3rd year	2/ 963 (0.2%)	160217, 160551
	Overall	5/1432 (0.3%)	160217, 160551, 161202, 161225, 244408
Papilloma viral infection	1st year	2/1432 (0.1%)	161514, 161525
	Overall	2/1432 (0.1%)	161514, 161525
Parasitic gastroenteritis	1st year	1/1432 (<0.1%)	190216
	Overall	1/1432 (<0.1%)	190216
Paronychia	2nd year	1/1166 (<0.1%)	160416
	Overall	1/1432 (<0.1%)	160416
Pelvic inflammatory disease	1st year	2/1432 (0.1%)	140221, 244438
	2nd year	1/1166 (<0.1%)	243220
	3rd year	2/ 963 (0.2%)	180116, 180618
	Overall	5/1432 (0.3%)	140221, 180116, 180618, 243220, 244438
Peritoneal abscess	2nd year	1/1166 (<0.1%)	160973
	Overall	1/1432 (<0.1%)	160973

Table 14.3.1 / 10: Number of subjects with adverse events including subject identifier and year by treatment (FAS)

TREATMENT: LCS12

Primary system organ class<br>Preferred term<br>MedDRA version 14.0	YEAR OF ONSET	N (%)	subject identifier
Peritonsillar abscess	1st year	2/1432 (0.1%)	160904, 244613
	Overall	2/1432 (0.1%)	160904, 244613
Pharyngeal abscess	3rd year	1/ 963 (0.1%)	160729
	Overall	1/1432 (<0.1%)	160729
Pharyngitis	1st year	8/1432 (0.6%)	120417, 120610, 120611, 120632, 140210, 160749, 180733, 244425
	2nd year	3/1166 (0.3%)	150123, 243320, 245803
	3rd year	2/ 963 (0.2%)	140502, 244430
	Overall	13/1432 (0.9%)	120417, 120610, 120611, 120632, 140210, 140502, 150123, 160749, 180733, 243320, 244425, 244430, 245803
Pharyngitis streptococcal	1st year	11/1432 (0.8%)	140224, 240115, 240516, 241110, 241202, 243903, 243978, 244422, 244447, 245502, 246125
	2nd year	6/1166 (0.5%)	140703, 141004, 240739, 243502, 244443, 244610
	3rd year	7/ 963 (0.7%)	240726, 240739, 241548, 242818, 243206, 243222, 243913
	Overall	23/1432 (1.6%)	140224, 140703, 141004, 240115, 240516, 240726, 240739, 241110, 241202, 241548, 242818, 243206, 243222, 243502, 243903, 243913, 243978, 244422, 244443, 244447, 244610, 245502, 246125
Pharyngotonsillitis	3rd year	1/ 963 (0.1%)	150166
	Overall	1/1432 (<0.1%)	150166
Pilonidal cyst	2nd year	1/1166 (<0.1%)	245516
	Overall	1/1432 (<0.1%)	245516
Pneumonia	1st year	7/1432 (0.5%)	120315, 140113, 140507, 160129, 200501, 230604, 230809
	2nd year	3/1166 (0.3%)	160623, 161003, 241113
	3rd year	4/ 963 (0.4%)	120619, 160551, 180420, 244707
	Overall	14/1432 (1.0%)	120315, 120619, 140113, 140507, 160129, 160551, 160623, 161003, 180420, 200501, 230604, 230809, 241113, 244707
Pneumonia mycoplasmal	2nd year	1/1166 (<0.1%)	210411
	Overall	1/1432 (<0.1%)	210411

Table 14.3.1 / 10: Number of subjects with adverse events including subject identifier and year by treatment (FAS)

TREATMENT: LCS12

Primary system organ class<br>Preferred term<br>MedDRA version 14.0	YEAR OF ONSET	N (%)	subject identifier
Pogosta disease	2nd year	1/1166 (<0.1%)	161017
	Overall	1/1432 (<0.1%)	161017
Post procedural infection	1st year	3/1432 (0.2%)	120326, 200519, 200709
	Overall	3/1432 (0.2%)	120326, 200519, 200709
Pyelonephritis	1st year	6/1432 (0.4%)	140118, 141013, 141109, 200705, 200901, 241410
	2nd year	3/1166 (0.3%)	170406, 241725, 242807
	Overall	9/1432 (0.6%)	140118, 141013, 141109, 170406, 200705, 200901, 241410, 241725, 242807
Pyelonephritis acute	1st year	1/1432 (<0.1%)	161512
	3rd year	1/ 963 (0.1%)	161403
	Overall	2/1432 (0.1%)	161403, 161512
Q fever	2nd year	1/1166 (<0.1%)	200519
	Overall	1/1432 (<0.1%)	200519
Respiratory tract infection	1st year	6/1432 (0.4%)	160129, 160746, 160749, 161215, 161532, 161537
	2nd year	5/1166 (0.4%)	160729, 160738, 161203, 161205, 161215
	Overall	10/1432 (0.7%)	160129, 160729, 160738, 160746, 160749, 161203, 161205, 161215, 161532, 161537
Respiratory tract infection viral	3rd year	2/ 963 (0.2%)	243320, 243325
	Overall	2/1432 (0.1%)	243320, 243325
Rhinitis	1st year	6/1432 (0.4%)	120412, 120611, 160415, 160616, 180736, 180740
	Overall	6/1432 (0.4%)	120412, 120611, 160415, 160616, 180736, 180740
Salpingitis	2nd year	1/1166 (<0.1%)	161027
	Overall	1/1432 (<0.1%)	161027
Salpingo-oophoritis	1st year	2/1432 (0.1%)	150104, 180140
	Overall	2/1432 (0.1%)	150104, 180140
Sialoadenitis	1st year	1/1432 (<0.1%)	140111
	2nd year	1/1166 (<0.1%)	140111
	Overall	1/1432 (<0.1%)	140111

Table 14.3.1 / 10: Number of subjects with adverse events including subject identifier and year by treatment (FAS)

TREATMENT: LCS12

Primary system organ class<br>Preferred term<br>MedDRA version 14.0	YEAR OF ONSET	N (%)	subject identifier
Sinusitis	1st year	55/1432 (3.8%)	120401, 140113, 140301, 150156, 160129, 160305, 160320, 160321, 160328, 160404, 160541, 160548, 160566, 160572, 160613, 160626, 160721, 160725, 161026, 161221, 161440, 170606, 180652, 180733, 200523, 210203, 230515, 230809, 231012, 240525, 240644, 241110, 241185, 241305, 241406, 241414, 241424, 241536, 241553, 241717, 241718, 242313, 243220, 243222, 243323, 243325, 243327, 243904, 243914, 243919, 243965, 243969, 243978, 244203, 244711
	2nd year	33/1166 (2.8%)	140116, 140519, 160320, 160328, 160434, 160576, 160610, 160613, 160615, 160616, 160721, 161015, 161221, 161532, 180673, 200625, 231107, 241536, 241708, 241712, 243220, 243222, 243308, 243309, 243323, 243325, 243913, 243914, 244430, 245502, 245701, 245910, 245933
	3rd year	26/ 963 (2.7%)	140116, 140811, 141032, 160127, 160320, 160328, 160576, 160606, 160613, 160631, 160949, 161203, 161205, 161219, 161221, 161414, 200625, 210203, 231012, 240627, 241708, 243219, 243904, 244430, 245701, 245709
	Overall	90/1432 (6.3%)	120401, 140113, 140116, 140301, 140519, 140811, 141032, 150156, 160127, 160129, 160305, 160320, 160321, 160328, 160404, 160434, 160541, 160548, 160566, 160572, 160576, 160606, 160610, 160613, 160615, 160616, 160626, 160631, 160721, 160725, 160949, 161015, 161026, 161203, 161205, 161219, 161221, 161414, 161440, 161532, 170606, 180652, 180673, 180733, 200523, 200625, 210203, 230515, 230809, 231012, 231107, 240525, 240627, 240644, 241110, 241185, 241305, 241406, 241414, 241424, 241536, 241553, 241708, 241712, 241717, 241718, 242313, 243219, 243220, 243222, 243308, 243309, 243323, 243325, 243327, 243904, 243913, 243914, 243919, 243965, 243969, 243978, 244203, 244430, 244711, 245502, 245701, 245709, 245910, 245933

Table 14.3.1 / 10: Number of subjects with adverse events including subject identifier and year by treatment (FAS)

TREATMENT: LCS12

Primary system organ class<br>Preferred term<br>MedDRA version 14.0	YEAR OF ONSET	N (%)	subject identifier
Skin bacterial infection	3rd year	1/ 963 (0.1%)	160754
	Overall	1/1432 (<0.1%)	160754
Skin candida	3rd year	1/ 963 (0.1%)	244127
	Overall	1/1432 (<0.1%)	244127
Skin infection	1st year	1/1432 (<0.1%)	160113
	Overall	1/1432 (<0.1%)	160113
Staphylococcal infection	1st year	1/1432 (<0.1%)	243510
	Overall	1/1432 (<0.1%)	243510
Staphylococcal skin infection	1st year	1/1432 (<0.1%)	244417
	Overall	1/1432 (<0.1%)	244417
Streptococcal infection	2nd year	1/1166 (<0.1%)	120632
	Overall	1/1432 (<0.1%)	120632
Streptococcal sepsis	2nd year	1/1166 (<0.1%)	244602
	Overall	1/1432 (<0.1%)	244602
Subcutaneous abscess	1st year	2/1432 (0.1%)	120403, 241548
	2nd year	1/1166 (<0.1%)	241548
	3rd year	1/ 963 (0.1%)	120307
	Overall	3/1432 (0.2%)	120307, 120403, 241548
Sweat gland infection	1st year	1/1432 (<0.1%)	160547
	Overall	1/1432 (<0.1%)	160547
Tinea infection	1st year	1/1432 (<0.1%)	230217
	Overall	1/1432 (<0.1%)	230217
Tinea pedis	3rd year	1/ 963 (0.1%)	200528
	Overall	1/1432 (<0.1%)	200528

Table 14.3.1 / 10: Number of subjects with adverse events including subject identifier and year by treatment (FAS)

TREATMENT: LCS12

Primary system organ class<br>Preferred term<br>MedDRA version 14.0	YEAR OF ONSET	N (%)	subject identifier
Tonsillitis	1st year	15/1432 (1.0%)	120626, 140212, 150123, 150142, 150143, 160402, 160555, 160610, 160617, 160633, 180501, 230607, 230713, 241805, 245508
	2nd year	9/1166 (0.8%)	120632, 141004, 150143, 160566, 160633, 160701, 160729, 161517, 180630
	3rd year	6/ 963 (0.6%)	120619, 120632, 150143, 160625, 160633, 180645
	Overall	25/1432 (1.7%)	120619, 120626, 120632, 140212, 141004, 150123, 150142, 150143, 160402, 160555, 160566, 160610, 160617, 160625, 160633, 160701, 160729, 161517, 180501, 180630, 180645, 230607, 230713, 241805, 245508
Tonsillitis streptococcal	2nd year	1/1166 (<0.1%)	160952
	3rd year	1/ 963 (0.1%)	160952
	Overall	1/1432 (<0.1%)	160952
Tooth abscess	1st year	5/1432 (0.3%)	140113, 140502, 241718, 244132, 244618
	Overall	5/1432 (0.3%)	140113, 140502, 241718, 244132, 244618
Tooth infection	1st year	11/1432 (0.8%)	160119, 160205, 160613, 160705, 160758, 160949, 160967, 161117, 161539, 180652, 243712
	2nd year	4/1166 (0.3%)	120307, 160538, 161221, 243611
	3rd year	10/ 963 (1.0%)	120613, 150167, 160539, 160928, 161017, 161303, 161517, 242812, 243806, 245916
	Overall	25/1432 (1.7%)	120307, 120613, 150167, 160119, 160205, 160538, 160539, 160613, 160705, 160758, 160928, 160949, 160967, 161017, 161117, 161221, 161303, 161517, 161539, 180652, 242812, 243611, 243712, 243806, 245916
Tracheitis	1st year	1/1432 (<0.1%)	140114
	Overall	1/1432 (<0.1%)	140114
Trichomoniasis	1st year	1/1432 (<0.1%)	246132
	2nd year	1/1166 (<0.1%)	241547
	Overall	2/1432 (0.1%)	241547, 246132
Tuberculosis	3rd year	1/ 963 (0.1%)	120321
	Overall	1/1432 (<0.1%)	120321

Table 14.3.1 / 10: Number of subjects with adverse events including subject identifier and year by treatment (FAS)

TREATMENT: LCS12

Primary system organ class<br>Preferred term<br>MedDRA version 14.0	YEAR OF ONSET	N (%)	subject identifier
Tubo-ovarian abscess	1st year	1/1432 (<0.1%)	244438
	Overall	1/1432 (<0.1%)	244438
Upper respiratory tract infection	1st year	31/1432 (2.2%)	141104, 161015, 161017, 161026, 180619, 180624, 230917, 241238, 241307, 241405, 241414, 243206, 243307, 243322, 243323, 243327, 243720, 244125, 244402, 244407, 244408, 244412, 244422, 244426, 244447, 244504, 244508, 244515, 244523, 244604, 245702
	2nd year	16/1166 (1.4%)	140808, 161001, 161015, 161023, 180604, 180624, 180644, 240521, 241531, 242805, 243314, 243322, 244508, 244602, 244608, 244618
	3rd year	13/ 963 (1.3%)	140807, 160119, 161001, 161015, 161017, 161023, 230216, 241302, 241305, 242510, 243206, 244607, 244707
	Overall	51/1432 (3.6%)	140807, 140808, 141104, 160119, 161001, 161015, 161017, 161023, 161026, 180604, 180619, 180624, 180644, 230216, 230917, 240521, 241238, 241302, 241305, 241307, 241405, 241414, 241531, 242510, 242805, 243206, 243307, 243314, 243322, 243323, 243327, 243720, 244125, 244402, 244407, 244408, 244412, 244422, 244426, 244447, 244504, 244508, 244515, 244523, 244602, 244604, 244607, 244608, 244618, 244707, 245702
Ureaplasma infection	2nd year	1/1166 (<0.1%)	150104
	Overall	1/1432 (<0.1%)	150104

Table 14.3.1 / 10: Number of subjects with adverse events including subject identifier and year by treatment (FAS)

TREATMENT: LCS12

Primary system organ class<br>Preferred term<br>MedDRA version 14.0	YEAR OF ONSET	N (%)	subject identifier
Urinary tract infection	1st year	94/1432 (6.6%)	120201, 120307, 120611, 120619, 120626, 120632, 140228, 141004, 141022, 141109, 150104, 150125, 150128, 150143, 150163, 160402, 160425, 160506, 160515, 160520, 160539, 160547, 160548, 160564, 160571, 160623, 160626, 160712, 160736, 160743, 160806, 160910, 160952, 160967, 161012, 161023, 161127, 161304, 161511, 170406, 180630, 180643, 180662, 190118, 190204, 190205, 210124, 210125, 210404, 210411, 230103, 230115, 230310, 230501, 230603, 231009, 231112, 240131, 240217, 240520, 240617, 240716, 240904, 241009, 241128, 241142, 241167, 241228, 241231, 241307, 241410, 241545, 242204, 242315, 242712, 242714, 243103, 243220, 243224, 243308, 243320, 243814, 243830, 243950, 243972, 244125, 244425, 244427, 244447, 244701, 245014, 245439, 245525, 245929
	2nd year	42/1166 (3.6%)	120321, 120330, 140302, 140518, 141106, 141402, 141423, 150104, 150110, 150149, 160566, 160571, 161003, 161012, 161017, 161215, 161218, 161322, 161440, 170101, 170406, 180676, 210136, 230603, 230818, 240209, 240326, 240715, 241130, 241231, 241302, 241525, 241708, 242316, 243307, 243502, 243904, 243943, 243972, 244408, 244602, 244934
	3rd year	43/ 963 (4.5%)	120119, 120307, 120310, 120321, 120417, 120619, 150104, 150506, 160102, 160113, 160201, 160218, 160571, 160616, 160705, 160712, 160911, 161001, 161109, 161127, 161203, 161218, 161219, 161221, 161303, 161440, 170404, 230401, 230908, 231016, 241009, 241128, 241142, 241149, 241185, 241302, 241421, 241736, 244425, 244523, 244707, 244914, 246214
	Overall	158/1432 (11.0%)	120119, 120201, 120307, 120310, 120321, 120330, 120417, 120611, 120619, 120626, 120632, 140228, 140302, 140518, 141004, 141022, 141106, 141109, 141402, 141423, 150104, 150110, 150125, 150128, 150143,

Table 14.3.1 / 10: Number of subjects with adverse events including subject identifier and year by treatment (FAS)

TREATMENT: LCS12

Primary system organ class<br>Preferred term<br>MedDRA version 14.0	YEAR OF ONSET	N (%)	subject identifier
			150149, 150163, 150506, 160102, 160113, 160201, 160218, 160402, 160425, 160506, 160515, 160520, 160539, 160547, 160548, 160564, 160566, 160571, 160616, 160623, 160626, 160705, 160712, 160736, 160743, 160806, 160910, 160911, 160952, 160967, 161001, 161003, 161012, 161017, 161023, 161109, 161127, 161203, 161215, 161218, 161219, 161221, 161303, 161304, 161322, 161440, 161511, 170101, 170404, 170406, 180630, 180643, 180662, 180676, 190118, 190204, 190205, 210124, 210125, 210136, 210404, 210411, 230103, 230115, 230310, 230401, 230501, 230603, 230818, 230908, 231009, 231016, 231112, 240131, 240209, 240217, 240326, 240520, 240617, 240715, 240716, 240904, 241009, 241128, 241130, 241142, 241149, 241167, 241185, 241228, 241231, 241302, 241307, 241410, 241421, 241525, 241545, 241708, 241736, 242204, 242315, 242316, 242712, 242714, 243103, 243220, 243224, 243307, 243308, 243320, 243502, 243814, 243830, 243904, 243943, 243950, 243972, 244125, 244408, 244425, 244427, 244447, 244523, 244602, 244701, 244707, 244914, 244934, 245014, 245439, 245525, 245929, 246214

Table 14.3.1 / 10: Number of subjects with adverse events including subject identifier and year by treatment (FAS)

TREATMENT: LCS12

Primary system organ class<br>Preferred term<br>MedDRA version 14.0	YEAR OF<br>ONSET	N (%)	subject identifier
Vaginal infection	1st year	28/1432 (2.0%)	140113, 140502, 140511, 140905, 160205, 160522, 160536, 160571, 160601, 160615, 160633, 161225, 161303, 161540, 180136, 180705, 180733, 190132, 190135, 190140, 190203, 190412, 190418, 240366, 242805, 242818, 243118, 246104
	2nd year	20/1166 (1.7%)	140518, 141013, 141022, 160102, 160201, 160207, 160538, 160571, 160623, 160625, 160633, 180503, 180733, 190126, 190132, 190216, 210124, 242315, 242906, 243907
	3rd year	6/ 963 (0.6%)	140302, 141011, 160555, 160623, 160625, 240828
	Overall	48/1432 (3.4%)	140113, 140302, 140502, 140511, 140518, 140905, 141011, 141013, 141022, 160102, 160201, 160205, 160207, 160522, 160536, 160538, 160555, 160571, 160601, 160615, 160623, 160625, 160633, 161225, 161303, 161540, 180136, 180503, 180705, 180733, 190126, 190132, 190135, 190140, 190203, 190216, 190412, 190418, 210124, 240366, 240828, 242315, 242805, 242818, 242906, 243118, 243907, 246104

Table 14.3.1 / 10: Number of subjects with adverse events including subject identifier and year by treatment (FAS)

TREATMENT: LCS12

Primary system organ class<br>Preferred term<br>MedDRA version 14.0	YEAR OF ONSET	N (%)	subject identifier
Vaginitis bacterial	1st year	52/1432 (3.6%)	140210, 140802, 141101, 141201, 150104, 150149, 160431, 160515, 160726, 160754, 161436, 180644, 190203, 200908, 210109, 210514, 231016, 231112, 240116, 240304, 240312, 240327, 240357, 240622, 240720, 240801, 240827, 241521, 241547, 241605, 242506, 243030, 243314, 243510, 243712, 243903, 244003, 244316, 244402, 244441, 244447, 244508, 244909, 244917, 244919, 245004, 245423, 245516, 246106, 246121, 246122, 246138
	2nd year	36/1166 (3.1%)	120323, 140113, 140802, 140807, 141034, 141426, 160402, 160525, 160609, 160913, 160982, 161001, 190619, 210124, 230818, 230908, 231103, 241129, 241405, 241704, 241902, 242218, 242506, 242805, 242807, 243010, 243709, 243978, 244402, 244515, 244617, 244931, 245004, 245519, 246122, 246212
	3rd year	30/ 963 (3.1%)	120323, 160425, 160520, 160746, 160914, 160949, 190605, 190618, 230406, 240622, 241015, 241227, 241421, 242506, 243117, 243709, 243807, 243914, 243978, 244105, 244425, 244509, 244809, 244914, 244931, 245432, 245518, 245807, 246106, 246122
	Overall	105/1432 (7.3%)	120323, 140113, 140210, 140802, 140807, 141034, 141101, 141201, 141426, 150104, 150149, 160402, 160425, 160431, 160515, 160520, 160525, 160609, 160726, 160746, 160754, 160913, 160914, 160949, 160982, 161001, 161436, 180644, 190203, 190605, 190618, 190619, 200908, 210109, 210124, 210514, 230406, 230818, 230908, 231016, 231103, 231112, 240116, 240304, 240312, 240327, 240357, 240622, 240720, 240801, 240827, 241015, 241129, 241227, 241405, 241421, 241521, 241547, 241605, 241704, 241902, 242218, 242506, 242805, 242807, 243010, 243030, 243117, 243314, 243510, 243709, 243712, 243807, 243903, 243914,

Table 14.3.1 / 10: Number of subjects with adverse events including subject identifier and year by treatment (FAS)

TREATMENT: LCS12

Primary system organ class<br>Preferred term<br>MedDRA version 14.0	YEAR OF ONSET	N (%)	subject identifier
			243978, 244003, 244105, 244316, 244402, 244425, 244441, 244447, 244508, 244509, 244515, 244617, 244809, 244909, 244914, 244917, 244919, 244931, 245004, 245423, 245432, 245516, 245518, 245519, 245807, 246106, 246121, 246122, 246138, 246212
Vaginitis chlamydial	1st year	1/1432 (<0.1%)	243720
	2nd year	1/1166 (<0.1%)	241129
	Overall	2/1432 (0.1%)	241129, 243720
Vestibular neuronitis	1st year	2/1432 (0.1%)	160725, 230408
	Overall	2/1432 (0.1%)	160725, 230408
Viral infection	2nd year	2/1166 (0.2%)	140802, 241302
	3rd year	1/ 963 (0.1%)	242712
	Overall	3/1432 (0.2%)	140802, 241302, 242712
Viral pharyngitis	2nd year	1/1166 (<0.1%)	243320
	Overall	1/1432 (<0.1%)	243320
Viral rhinitis	2nd year	1/1166 (<0.1%)	243311
	Overall	1/1432 (<0.1%)	243311
Viral upper respiratory tract infection	1st year	7/1432 (0.5%)	161403, 161407, 161408, 161426, 161435, 161436, 161437
	2nd year	9/1166 (0.8%)	161403, 161406, 161410, 161414, 161424, 161426, 161431, 161440, 243323
	3rd year	1/ 963 (0.1%)	242315
	Overall	15/1432 (1.0%)	161403, 161406, 161407, 161408, 161410, 161414, 161424, 161426, 161431, 161435, 161436, 161437, 161440, 242315, 243323
Vulvitis	1st year	5/1432 (0.3%)	120411, 140529, 150166, 160619, 190401
	2nd year	3/1166 (0.3%)	150138, 231103, 243302
	Overall	8/1432 (0.6%)	120411, 140529, 150138, 150166, 160619, 190401, 231103, 243302

Table 14.3.1 / 10: Number of subjects with adverse events including subject identifier and year by treatment (FAS)

TREATMENT: LCS12

Primary system organ class<br>Preferred term<br>MedDRA version 14.0	YEAR OF ONSET	N (%)	subject identifier
Vulvovaginal candidiasis	1st year	40/1432 (2.8%)	140206, 140911, 160220, 160402, 160404, 160515, 160555, 160928, 160935, 160954, 160981, 161102, 161106, 161117, 161124, 161218, 161408, 161424, 161426, 161431, 200104, 200703, 200916, 230408, 230517, 230816, 230817, 230904, 231012, 231016, 231017, 240357, 240516, 240520, 241204, 242818, 244518, 244917, 245003, 245019
	2nd year	25/1166 (2.1%)	140210, 140511, 160102, 160402, 160551, 160569, 160615, 160928, 160960, 161112, 161114, 161117, 161122, 161224, 161408, 161511, 190629, 200703, 230401, 231009, 231012, 231018, 231112, 242805, 246122
	3rd year	16/ 963 (1.7%)	140518, 160576, 160623, 160624, 160746, 160911, 161109, 161122, 161127, 161203, 161303, 200121, 200703, 230604, 242818, 244931
	Overall	72/1432 (5.0%)	140206, 140210, 140511, 140518, 140911, 160102, 160220, 160402, 160404, 160515, 160551, 160555, 160569, 160576, 160615, 160623, 160624, 160746, 160911, 160928, 160935, 160954, 160960, 160981, 161102, 161106, 161109, 161112, 161114, 161117, 161122, 161124, 161127, 161203, 161218, 161224, 161303, 161408, 161424, 161426, 161431, 161511, 190629, 200104, 200121, 200703, 200916, 230401, 230408, 230517, 230604, 230816, 230817, 230904, 231009, 231012, 231016, 231017, 231018, 231112, 240357, 240516, 240520, 241204, 242805, 242818, 244518, 244917, 244931, 245003, 245019, 246122

Table 14.3.1 / 10: Number of subjects with adverse events including subject identifier and year by treatment (FAS)

TREATMENT: LCS12

Primary system organ class<br>Preferred term<br>MedDRA version 14.0	YEAR OF<br>ONSET	N (%)	subject identifier
Vulvovaginal mycotic infection	1st year	61/1432 (4.3%)	141004, 141201, 150101, 150123, 150124, 150125, 150128, 150138, 150151, 150153, 150156, 150163, 160564, 160566, 160571, 160701, 160710, 160721, 160732, 160741, 160756, 161003, 170603, 180618, 180624, 230310, 240110, 240242, 240312, 240326, 240327, 240335, 240357, 240366, 240644, 240701, 241133, 241424, 241513, 241545, 241547, 242307, 242315, 242320, 243003, 243009, 243012, 243030, 243222, 243309, 243314, 243323, 243907, 244108, 244117, 244203, 244447, 244509, 244701, 245027, 245710
	2nd year	35/1166 (3.0%)	120323, 120626, 141426, 150109, 150112, 150123, 150138, 160418, 160566, 160705, 170101, 180621, 180662, 240357, 241414, 241525, 241541, 241725, 241902, 242302, 243117, 243220, 243307, 243309, 243314, 243502, 243829, 244203, 244408, 244430, 244602, 245514, 245519, 245808, 246122
	3rd year	20/ 963 (2.1%)	120611, 140116, 150104, 150112, 150123, 150151, 150166, 160548, 160559, 160571, 160701, 160741, 170702, 242302, 243511, 243914, 244408, 244509, 245519, 245803
	Overall	99/1432 (6.9%)	120323, 120611, 120626, 140116, 141004, 141201, 141426, 150101, 150104, 150109, 150112, 150123, 150124, 150125, 150128, 150138, 150151, 150153, 150156, 150163, 150166, 160418, 160548, 160559, 160564, 160566, 160571, 160701, 160705, 160710, 160721, 160732, 160741, 160756, 161003, 170101, 170603, 170702, 180618, 180621, 180624, 180662, 230310, 240110, 240242, 240312, 240326, 240327, 240335, 240357, 240366, 240644, 240701, 241133, 241414, 241424, 241513, 241525, 241541, 241545, 241547, 241725, 241902, 242302, 242307, 242315, 242320, 243003, 243009, 243012, 243030, 243117, 243220, 243222, 243307, 243309, 243314, 243323, 243502, 243511, 243829, 243907, 243914, 244108, 244117, 244203, 244408, 244430, 244447, 244509, 244602, 244701, 245027, 245514, 245519, 245710, 245803, 245808, 246122

Table 14.3.1 / 10: Number of subjects with adverse events including subject identifier and year by treatment (FAS)

TREATMENT: LCS12

Primary system organ class<br>Preferred term<br>MedDRA version 14.0	YEAR OF ONSET	N (%)	subject identifier
Vulvovaginitis	1st year	6/1432 (0.4%)	120414, 120626, 120630, 150123, 180346, 190402
	3rd year	1/ 963 (0.1%)	180346
	Overall	6/1432 (0.4%)	120414, 120626, 120630, 150123, 180346, 190402
Vulvovaginitis streptococcal	1st year	1/1432 (<0.1%)	245025
	Overall	1/1432 (<0.1%)	245025
Vulvovaginitis trichomonal	1st year	1/1432 (<0.1%)	140608
	2nd year	1/1166 (<0.1%)	245808
	3rd year	2/ 963 (0.2%)	120214, 240623
	Overall	4/1432 (0.3%)	120214, 140608, 240623, 245808
Wound infection	1st year	1/1432 (<0.1%)	140802
	2nd year	1/1166 (<0.1%)	161301
	3rd year	1/ 963 (0.1%)	160520
	Overall	3/1432 (0.2%)	140802, 160520, 161301
Injury, poisoning and procedural complications	1st year	98/1432 (6.8%)	
	2nd year	31/1166 (2.7%)	
	3rd year	30/ 963 (3.1%)	
	Overall	149/1432 (10.4%)	
Animal bite	1st year	2/1432 (0.1%)	160572, 160928
	2nd year	1/1166 (<0.1%)	120312
	Overall	3/1432 (0.2%)	120312, 160572, 160928
Ankle fracture	2nd year	1/1166 (<0.1%)	120411
	Overall	1/1432 (<0.1%)	120411
Arthropod bite	1st year	3/1432 (0.2%)	150163, 180728, 244518
	2nd year	1/1166 (<0.1%)	243611
	3rd year	1/ 963 (0.1%)	161001
	Overall	5/1432 (0.3%)	150163, 161001, 180728, 243611, 244518
Burns third degree	1st year	1/1432 (<0.1%)	241190
	Overall	1/1432 (<0.1%)	241190
Clavicle fracture	3rd year	1/ 963 (0.1%)	161408
	Overall	1/1432 (<0.1%)	161408

Table 14.3.1 / 10: Number of subjects with adverse events including subject identifier and year by treatment (FAS)

TREATMENT: LCS12

Primary system organ class<br>Preferred term<br>MedDRA version 14.0	YEAR OF ONSET	N (%)	subject identifier
Concussion	1st year	1/1432 (<0.1%)	230219
	2nd year	2/1166 (0.2%)	160633, 200505
	Overall	3/1432 (0.2%)	160633, 200505, 230219
Contusion	1st year	5/1432 (0.3%)	150124, 161406, 200631, 243327, 243711
	3rd year	2/ 963 (0.2%)	160605, 244515
	Overall	7/1432 (0.5%)	150124, 160605, 161406, 200631, 243327, 243711, 244515
Epicondylitis	1st year	2/1432 (0.1%)	160558, 160619
	Overall	2/1432 (0.1%)	160558, 160619
Eye injury	3rd year	1/ 963 (0.1%)	160506
	Overall	1/1432 (<0.1%)	160506
Facial bones fracture	2nd year	2/1166 (0.2%)	200901, 244412
	Overall	2/1432 (0.1%)	200901, 244412
Foot fracture	1st year	3/1432 (0.2%)	241805, 245911, 245932
	2nd year	3/1166 (0.3%)	241406, 242902, 245808
	3rd year	4/ 963 (0.4%)	180511, 240508, 242902, 245933
	Overall	9/1432 (0.6%)	180511, 240508, 241406, 241805, 242902, 245808, 245911, 245932, 245933
Forearm fracture	1st year	1/1432 (<0.1%)	140520
	Overall	1/1432 (<0.1%)	140520
Frostbite	1st year	1/1432 (<0.1%)	141426
	Overall	1/1432 (<0.1%)	141426
Hand fracture	3rd year	1/ 963 (0.1%)	160623
	Overall	1/1432 (<0.1%)	160623
Head injury	1st year	1/1432 (<0.1%)	243117
	Overall	1/1432 (<0.1%)	243117

Table 14.3.1 / 10: Number of subjects with adverse events including subject identifier and year by treatment (FAS)

TREATMENT: LCS12

Primary system organ class / Preferred term / MedDRA version 14.0	YEAR OF ONSET	N (%)	subject identifier
Joint dislocation	1st year	3/1432 (0.2%)	160506, 180707, 200119
	2nd year	1/1166 (<0.1%)	160405
	3rd year	1/ 963 (0.1%)	160984
	Overall	5/1432 (0.3%)	160405, 160506, 160984, 180707, 200119
Joint injury	1st year	5/1432 (0.3%)	120326, 120621, 160615, 180642, 244523
	2nd year	1/1166 (<0.1%)	160121
	3rd year	1/ 963 (0.1%)	244504
	Overall	7/1432 (0.5%)	120326, 120621, 160121, 160615, 180642, 244504, 244523
Joint sprain	1st year	7/1432 (0.5%)	140301, 160108, 160130, 160605, 200911, 210409, 243711
	2nd year	3/1166 (0.3%)	160803, 160806, 170413
	3rd year	3/ 963 (0.3%)	120307, 120315, 242906
	Overall	13/1432 (0.9%)	120307, 120315, 140301, 160108, 160130, 160605, 160803, 160806, 170413, 200911, 210409, 242906, 243711
Laceration	1st year	1/1432 (<0.1%)	140802
	2nd year	1/1166 (<0.1%)	244422
	3rd year	1/ 963 (0.1%)	243916
	Overall	3/1432 (0.2%)	140802, 243916, 244422
Laryngeal injury	1st year	1/1432 (<0.1%)	230613
	Overall	1/1432 (<0.1%)	230613
Ligament rupture	1st year	2/1432 (0.1%)	230104, 240716
	3rd year	1/ 963 (0.1%)	241717
	Overall	3/1432 (0.2%)	230104, 240716, 241717
Limb crushing injury	3rd year	1/ 963 (0.1%)	160108
	Overall	1/1432 (<0.1%)	160108
Limb injury	3rd year	1/ 963 (0.1%)	160127
	Overall	1/1432 (<0.1%)	160127
Lower limb fracture	3rd year	1/ 963 (0.1%)	161523
	Overall	1/1432 (<0.1%)	161523

Table 14.3.1 / 10: Number of subjects with adverse events including subject identifier and year by treatment (FAS)

TREATMENT: LCS12

Primary system organ class<br>Preferred term<br>MedDRA version 14.0	YEAR OF ONSET	N (%)	subject identifier
Meniscus lesion	1st year	1/1432 (<0.1%)	180642
	2nd year	1/1166 (<0.1%)	242807
	3rd year	1/ 963 (0.1%)	240701
	Overall	3/1432 (0.2%)	180642, 240701, 242807
Muscle injury	1st year	3/1432 (0.2%)	140118, 140605, 243327
	2nd year	1/1166 (<0.1%)	140202
	3rd year	1/ 963 (0.1%)	140511
	Overall	5/1432 (0.3%)	140118, 140202, 140511, 140605, 243327
Muscle strain	1st year	4/1432 (0.3%)	140118, 140605, 244407, 244701
	3rd year	3/ 963 (0.3%)	140511, 160427, 243617
	Overall	7/1432 (0.5%)	140118, 140511, 140605, 160427, 243617, 244407, 244701
Nail avulsion	3rd year	1/ 963 (0.1%)	245933
	Overall	1/1432 (<0.1%)	245933
Overdose	3rd year	1/ 963 (0.1%)	230720
	Overall	1/1432 (<0.1%)	230720
Post procedural haemorrhage	1st year	1/1432 (<0.1%)	150136
	2nd year	1/1166 (<0.1%)	241113
	Overall	2/1432 (0.1%)	150136, 241113
Post-traumatic pain	1st year	2/1432 (0.1%)	120326, 160803
	2nd year	1/1166 (<0.1%)	241421
	Overall	3/1432 (0.2%)	120326, 160803, 241421

Table 14.3.1 / 10: Number of subjects with adverse events including subject identifier and year by treatment (FAS)

TREATMENT: LCS12

Primary system organ class<br>Preferred term<br>MedDRA version 14.0	YEAR OF ONSET	N (%)	subject identifier
Procedural pain	1st year	48/1432 (3.4%)	120418, 140808, 150503, 160201, 160329, 160520, 160525, 160538, 160615, 160626, 160704, 160705, 160710, 160713, 160738, 160739, 160743, 161002, 161017, 161018, 161102, 161104, 161110, 161112, 161114, 161117, 161121, 161122, 161127, 161531, 170704, 180621, 200112, 200501, 200503, 200708, 210205, 230607, 231009, 240215, 241405, 241536, 242906, 242909, 243314, 243916, 244302, 244807
	2nd year	8/1166 (0.7%)	160506, 160746, 210127, 240510, 242307, 242315, 243121, 244509
	3rd year	4/ 963 (0.4%)	160506, 242308, 242315, 244408
	Overall	58/1432 (4.1%)	120418, 140808, 150503, 160201, 160329, 160506, 160520, 160525, 160538, 160615, 160626, 160704, 160705, 160710, 160713, 160738, 160739, 160743, 160746, 161002, 161017, 161018, 161102, 161104, 161110, 161112, 161114, 161117, 161121, 161122, 161127, 161531, 170704, 180621, 200112, 200501, 200503, 200708, 210127, 210205, 230607, 231009, 240215, 240510, 241405, 241536, 242307, 242308, 242315, 242906, 242909, 243121, 243314, 243916, 244302, 244408, 244509, 244807
Procedural vomiting	1st year	1/1432 (<0.1%)	160712
	Overall	1/1432 (<0.1%)	160712
Radius fracture	2nd year	1/1166 (<0.1%)	240904
	Overall	1/1432 (<0.1%)	240904
Rib fracture	1st year	1/1432 (<0.1%)	210107
	Overall	1/1432 (<0.1%)	210107
Road traffic accident	1st year	3/1432 (0.2%)	190610, 243327, 245702
	Overall	3/1432 (0.2%)	190610, 243327, 245702
Seroma	3rd year	1/ 963 (0.1%)	242906
	Overall	1/1432 (<0.1%)	242906

Table 14.3.1 / 10: Number of subjects with adverse events including subject identifier and year by treatment (FAS)

TREATMENT: LCS12

Primary system organ class<br>Preferred term<br>MedDRA version 14.0	YEAR OF ONSET	N (%)	subject identifier
Shunt occlusion	1st year	1/1432 (<0.1%)	160329
	Overall	1/1432 (<0.1%)	160329
Subdural haematoma	1st year	1/1432 (<0.1%)	160329
	Overall	1/1432 (<0.1%)	160329
Tendon rupture	1st year	1/1432 (<0.1%)	160108
	3rd year	2/ 963 (0.2%)	160105, 160130
	Overall	3/1432 (0.2%)	160105, 160108, 160130
Tibia fracture	2nd year	1/1166 (<0.1%)	241545
	Overall	1/1432 (<0.1%)	241545
Traumatic fracture	1st year	1/1432 (<0.1%)	160528
	Overall	1/1432 (<0.1%)	160528
Upper limb fracture	2nd year	1/1166 (<0.1%)	150143
	Overall	1/1432 (<0.1%)	150143
Whiplash injury	1st year	1/1432 (<0.1%)	243220
	Overall	1/1432 (<0.1%)	243220
Wound	1st year	1/1432 (<0.1%)	150149
	Overall	1/1432 (<0.1%)	150149
Investigations	1st year	57/1432 (4.0%)	
	2nd year	37/1166 (3.2%)	
	3rd year	61/ 963 (6.3%)	
	Overall	150/1432 (10.5%)	
Alanine aminotransferase increased	1st year	1/1432 (<0.1%)	160213
	2nd year	1/1166 (<0.1%)	241605
	3rd year	6/ 963 (0.6%)	150112, 160519, 170608, 243806, 244508, 245710
	Overall	9/1432 (0.6%)	150112, 160213, 160519, 170608, 241605, 242313, 243806, 244508, 245710
Antibiotic resistant Staphylococcus test positive	1st year	1/1432 (<0.1%)	243948
	3rd year	1/ 963 (0.1%)	243323
	Overall	2/1432 (0.1%)	243323, 243948

Table 14.3.1 / 10: Number of subjects with adverse events including subject identifier and year by treatment (FAS)

TREATMENT: LCS12

Primary system organ class<br>Preferred term<br>MedDRA version 14.0	YEAR OF ONSET	N (%)	subject identifier
Aspartate aminotransferase increased	1st year	1/1432 (<0.1%)	160213
	3rd year	4/ 963 (0.4%)	150112, 160519, 244508, 245710
	Overall	6/1432 (0.4%)	150112, 160213, 160519, 242313, 244508, 245710
Biopsy muscle	1st year	1/1432 (<0.1%)	230613
	Overall	1/1432 (<0.1%)	230613
Blood alkaline phosphatase decreased	3rd year	1/ 963 (0.1%)	150123
	Overall	1/1432 (<0.1%)	150123
Blood cholesterol increased	1st year	3/1432 (0.2%)	160758, 200908, 240112
	Overall	3/1432 (0.2%)	160758, 200908, 240112
Blood potassium abnormal	3rd year	1/ 963 (0.1%)	200205
	Overall	1/1432 (<0.1%)	200205
Blood pressure increased	2nd year	2/1166 (0.2%)	160637, 230720
	3rd year	1/ 963 (0.1%)	245528
	Overall	3/1432 (0.2%)	160637, 230720, 245528
Blood sodium decreased	3rd year	1/ 963 (0.1%)	150142
	Overall	1/1432 (<0.1%)	150142
Blood triglycerides increased	1st year	1/1432 (<0.1%)	200908
	Overall	1/1432 (<0.1%)	200908
Blood urine present	3rd year	3/ 963 (0.3%)	244417, 244425, 246220
	Overall	3/1432 (0.2%)	244417, 244425, 246220
Carbohydrate antigen 125 increased	3rd year	1/ 963 (0.1%)	241305
	Overall	1/1432 (<0.1%)	241305
Cardiac murmur	2nd year	1/1166 (<0.1%)	241416
	Overall	1/1432 (<0.1%)	241416

Table 14.3.1 / 10: Number of subjects with adverse events including subject identifier and year by treatment (FAS)

TREATMENT: LCS12

Primary system organ class<br>Preferred term<br>MedDRA version 14.0	YEAR OF ONSET	N (%)	subject identifier
Chlamydia test positive	1st year	2/1432 (0.1%)	160575, 245004
	2nd year	2/1166 (0.2%)	240622, 241547
	3rd year	3/ 963 (0.3%)	200611, 200923, 242818
	Overall	7/1432 (0.5%)	160575, 200611, 200923, 240622, 241547, 242818, 245004
Escherichia test positive	3rd year	1/ 963 (0.1%)	150506
	Overall	1/1432 (<0.1%)	150506
Gamma-glutamyltransferase increased	3rd year	4/ 963 (0.4%)	150168, 160437, 160619, 180416
	Overall	4/1432 (0.3%)	150168, 160437, 160619, 180416
Glycosylated haemoglobin increased	2nd year	1/1166 (<0.1%)	243302
	3rd year	4/ 963 (0.4%)	240234, 244427, 245525, 246119
	Overall	5/1432 (0.3%)	240234, 243302, 244427, 245525, 246119
Haematocrit decreased	1st year	1/1432 (<0.1%)	244125
	Overall	1/1432 (<0.1%)	244125
Haematocrit increased	2nd year	1/1166 (<0.1%)	150110
	3rd year	1/ 963 (0.1%)	150123
	Overall	2/1432 (0.1%)	150110, 150123
Haemoglobin decreased	1st year	2/1432 (0.1%)	140116, 244125
	2nd year	1/1166 (<0.1%)	160201
	3rd year	2/ 963 (0.2%)	241414, 245525
	Overall	5/1432 (0.3%)	140116, 160201, 241414, 244125, 245525
Hepatic enzyme increased	1st year	1/1432 (<0.1%)	190418
	Overall	1/1432 (<0.1%)	190418
High density lipoprotein decreased	3rd year	3/ 963 (0.3%)	150123, 150138, 150151
	Overall	3/1432 (0.2%)	150123, 150138, 150151
Human papilloma virus test positive	1st year	1/1432 (<0.1%)	160976
	2nd year	3/1166 (0.3%)	241005, 241028, 245603
	3rd year	1/ 963 (0.1%)	141029
	Overall	5/1432 (0.3%)	141029, 160976, 241005, 241028, 245603

Table 14.3.1 / 10: Number of subjects with adverse events including subject identifier and year by treatment (FAS)

TREATMENT: LCS12

Primary system organ class<br>Preferred term<br>MedDRA version 14.0	YEAR OF ONSET	N (%)	subject identifier
Low density lipoprotein increased	1st year	1/1432 (<0.1%)	243207
	Overall	1/1432 (<0.1%)	243207
Neisseria test positive	1st year	1/1432 (<0.1%)	240623
	2nd year	1/1166 (<0.1%)	240622
	Overall	2/1432 (0.1%)	240622, 240623
Platelet count decreased	3rd year	2/ 963 (0.2%)	150145, 242902
	Overall	2/1432 (0.1%)	150145, 242902
Protein urine present	3rd year	1/ 963 (0.1%)	244809
	Overall	1/1432 (<0.1%)	244809
Red blood cell count increased	2nd year	1/1166 (<0.1%)	150110
	Overall	1/1432 (<0.1%)	150110
Simplex virus test positive	2nd year	1/1166 (<0.1%)	243933
	3rd year	1/ 963 (0.1%)	241015
	Overall	2/1432 (0.1%)	241015, 243933
Smear cervix abnormal	1st year	2/1432 (0.1%)	200208, 245411
	2nd year	9/1166 (0.8%)	160406, 160514, 180662, 200210, 200908, 240712, 241706, 243219, 245603
	3rd year	7/ 963 (0.7%)	180225, 200304, 240514, 241172, 241717, 243611, 244523
	Overall	18/1432 (1.3%)	160406, 160514, 180225, 180662, 200208, 200210, 200304, 200908, 240514, 240712, 241172, 241706, 241717, 243219, 243611, 244523, 245411, 245603
Streptococcus test positive	2nd year	1/1166 (<0.1%)	160129
	Overall	1/1432 (<0.1%)	160129
Ultrasound ovary abnormal	2nd year	1/1166 (<0.1%)	140612
	Overall	1/1432 (<0.1%)	140612

Table 14.3.1 / 10: Number of subjects with adverse events including subject identifier and year by treatment (FAS)

TREATMENT: LCS12

Primary system organ class<br>Preferred term<br>MedDRA version 14.0	YEAR OF ONSET	N (%)	subject identifier
Weight decreased	1st year	2/1432 (0.1%)	140105, 243004
	2nd year	3/1166 (0.3%)	140204, 241119, 243611
	3rd year	4/ 963 (0.4%)	120315, 230501, 241102, 245431
	Overall	9/1432 (0.6%)	120315, 140105, 140204, 230501, 241102, 241119, 243004, 243611, 245431
Weight increased	1st year	39/1432 (2.7%)	120201, 120401, 140210, 140302, 140520, 141412, 141426, 160550, 160576, 160615, 160619, 160746, 180110, 210124, 230214, 230501, 230517, 231112, 240123, 240826, 241123, 241133, 241162, 241187, 241406, 241531, 241545, 241553, 243022, 243030, 243952, 244122, 244314, 244414, 244441, 245414, 245418, 245928, 245932
	2nd year	11/1166 (0.9%)	160734, 230517, 230720, 240525, 241005, 241110, 242902, 242910, 243830, 244914, 246141
	3rd year	7/ 963 (0.7%)	140718, 140817, 230501, 240321, 241149, 244430, 244909
	Overall	56/1432 (3.9%)	120201, 120401, 140210, 140302, 140520, 140718, 140817, 141412, 141426, 160550, 160576, 160615, 160619, 160734, 160746, 161111, 180110, 210124, 230214, 230501, 230517, 230720, 231112, 240123, 240321, 240525, 240826, 241005, 241110, 241123, 241133, 241149, 241162, 241187, 241406, 241531, 241545, 241553, 242902, 242910, 243022, 243030, 243830, 243952, 244122, 244314, 244414, 244430, 244441, 244909, 244914, 245414, 245418, 245928, 245932, 246141
White blood cell count decreased	3rd year	4/ 963 (0.4%)	161221, 180418, 190203, 241130
	Overall	4/1432 (0.3%)	161221, 180418, 190203, 241130
White blood cell count increased	2nd year	1/1166 (<0.1%)	140212
	3rd year	4/ 963 (0.4%)	160506, 180645, 190225, 244430
	Overall	5/1432 (0.3%)	140212, 160506, 180645, 190225, 244430
White blood cells urine positive	2nd year	1/1166 (<0.1%)	140302
	3rd year	2/ 963 (0.2%)	160741, 241159
	Overall	3/1432 (0.2%)	140302, 160741, 241159

Table 14.3.1 / 10: Number of subjects with adverse events including subject identifier and year by treatment (FAS)

TREATMENT: LCS12

Primary system organ class<br>Preferred term<br>MedDRA version 14.0	YEAR OF ONSET	N (%)	subject identifier
Metabolism and nutrition disorders	1st year	11/1432 (0.8%)	
	2nd year	2/1166 (0.2%)	
	3rd year	5/ 963 (0.5%)	
	Overall	18/1432 (1.3%)	
Cell death	2nd year	1/1166 (<0.1%)	242706
	Overall	1/1432 (<0.1%)	242706
Cholesterosis	1st year	1/1432 (<0.1%)	241536
	Overall	1/1432 (<0.1%)	241536
Decreased appetite	3rd year	2/ 963 (0.2%)	120315, 243323
	Overall	2/1432 (0.1%)	120315, 243323
Fluid retention	1st year	3/1432 (0.2%)	241406, 242307, 243403
	Overall	3/1432 (0.2%)	241406, 242307, 243403
Glucose tolerance impaired	3rd year	1/ 963 (0.1%)	243514
	Overall	1/1432 (<0.1%)	243514
Hypokalaemia	1st year	1/1432 (<0.1%)	241410
	3rd year	1/ 963 (0.1%)	242320
	Overall	2/1432 (0.1%)	241410, 242320
Hyponatraemia	1st year	1/1432 (<0.1%)	241410
	Overall	1/1432 (<0.1%)	241410
Increased appetite	1st year	1/1432 (<0.1%)	140802
	Overall	1/1432 (<0.1%)	140802
Insulin resistance	1st year	2/1432 (0.1%)	241108, 241123
	Overall	2/1432 (0.1%)	241108, 241123
Lactose intolerance	1st year	2/1432 (0.1%)	230804, 241807
	Overall	2/1432 (0.1%)	230804, 241807
Type 2 diabetes mellitus	2nd year	1/1166 (<0.1%)	240135
	Overall	1/1432 (<0.1%)	240135

Table 14.3.1 / 10: Number of subjects with adverse events including subject identifier and year by treatment (FAS)

TREATMENT: LCS12

Primary system organ class<br>Preferred term<br>MedDRA version 14.0	YEAR OF ONSET	N (%)	subject identifier
Vitamin B12 deficiency	1st year	1/1432 (<0.1%)	230401
	Overall	1/1432 (<0.1%)	230401
Vitamin D deficiency	3rd year	1/ 963 (0.1%)	241302
	Overall	1/1432 (<0.1%)	241302
Musculoskeletal and connective tissue disorders	1st year	89/1432 (6.2%)	
	2nd year	37/1166 (3.2%)	
	3rd year	33/ 963 (3.4%)	
	Overall	144/1432 (10.1%)	
Ankylosing spondylitis	1st year	1/1432 (<0.1%)	160935
	Overall	1/1432 (<0.1%)	160935
Arthralgia	1st year	15/1432 (1.0%)	120104, 120411, 160119, 160557, 160609, 160624, 160801, 180642, 241167, 241605, 242307, 243231, 243933, 245932, 245933
	2nd year	4/1166 (0.3%)	230501, 244918, 245431, 245910
	3rd year	3/ 963 (0.3%)	150149, 243219, 245933
	Overall	21/1432 (1.5%)	120104, 120411, 150149, 160119, 160557, 160609, 160624, 160801, 180642, 230501, 241167, 241605, 242307, 243219, 243231, 243933, 244918, 245431, 245910, 245932, 245933
Arthritis	2nd year	1/1166 (<0.1%)	200303
	Overall	1/1432 (<0.1%)	200303

Table 14.3.1 / 10: Number of subjects with adverse events including subject identifier and year by treatment (FAS)

TREATMENT: LCS12

Primary system organ class / Preferred term / MedDRA version 14.0	YEAR OF ONSET	N (%)	subject identifier
Back pain	1st year	32/1432 (2.2%)	120310, 120414, 120608, 120630, 140215, 160205, 160541, 160624, 160637, 160705, 161218, 161301, 170402, 170606, 200802, 200910, 210514, 230310, 230809, 240627, 240814, 241015, 241131, 241518, 242506, 243224, 243702, 243978, 244511, 245702, 245803, 246217
	2nd year	11/1166 (0.9%)	120320, 140608, 150109, 160217, 160624, 160720, 180734, 242812, 243616, 244412, 244610
	3rd year	18/ 963 (1.9%)	120335, 120621, 140507, 150142, 160105, 160110, 160328, 160427, 160626, 160944, 160954, 160973, 161403, 161435, 241507, 241606, 244407, 244412
	Overall	59/1432 (4.1%)	120310, 120320, 120335, 120414, 120608, 120621, 120630, 140215, 140507, 140608, 150109, 150142, 160105, 160110, 160205, 160217, 160328, 160427, 160541, 160624, 160626, 160637, 160705, 160720, 160944, 160954, 160973, 161218, 161301, 161403, 161435, 170402, 170606, 180734, 200802, 200910, 210514, 230310, 230809, 240627, 240814, 241015, 241131, 241507, 241518, 241606, 242506, 242812, 243224, 243616, 243702, 243978, 244407, 244412, 244511, 244610, 245702, 245803, 246217
Bone pain	1st year	1/1432 (<0.1%)	161440
	Overall	1/1432 (<0.1%)	161440
Bursitis	1st year	1/1432 (<0.1%)	242812
	2nd year	1/1166 (<0.1%)	160520
	Overall	2/1432 (0.1%)	160520, 242812
Costochondritis	1st year	1/1432 (<0.1%)	244809
	2nd year	2/1166 (0.2%)	242206, 244430
	Overall	3/1432 (0.2%)	242206, 244430, 244809
Exostosis	1st year	1/1432 (<0.1%)	244407
	Overall	1/1432 (<0.1%)	244407

Table 14.3.1 / 10: Number of subjects with adverse events including subject identifier and year by treatment (FAS)

TREATMENT: LCS12

Primary system organ class<br>Preferred term<br>MedDRA version 14.0	YEAR OF ONSET	N (%)	subject identifier
Fibromyalgia	1st year	1/1432 (<0.1%)	241174
	Overall	1/1432 (<0.1%)	241174
Flank pain	1st year	1/1432 (<0.1%)	240704
	2nd year	1/1166 (<0.1%)	140808
	Overall	2/1432 (0.1%)	140808, 240704
Groin pain	1st year	2/1432 (0.1%)	160130, 245914
	Overall	2/1432 (0.1%)	160130, 245914
Intervertebral disc disorder	1st year	1/1432 (<0.1%)	140301
	Overall	1/1432 (<0.1%)	140301
Intervertebral disc protrusion	3rd year	1/ 963 (0.1%)	160803
	Overall	1/1432 (<0.1%)	160803
Joint effusion	1st year	1/1432 (<0.1%)	240716
	Overall	1/1432 (<0.1%)	240716
Joint instability	1st year	1/1432 (<0.1%)	230613
	Overall	1/1432 (<0.1%)	230613
Joint swelling	3rd year	1/ 963 (0.1%)	244707
	Overall	1/1432 (<0.1%)	244707
Medial tibial stress syndrome	1st year	1/1432 (<0.1%)	241424
	2nd year	1/1166 (<0.1%)	161440
	Overall	2/1432 (0.1%)	161440, 241424
Muscle contracture	3rd year	1/ 963 (0.1%)	120602
	Overall	1/1432 (<0.1%)	120602
Muscle spasms	1st year	3/1432 (0.2%)	161517, 200904, 245702
	2nd year	2/1166 (0.2%)	241513, 244511
	Overall	5/1432 (0.3%)	161517, 200904, 241513, 244511, 245702

Table 14.3.1 / 10: Number of subjects with adverse events including subject identifier and year by treatment (FAS)

TREATMENT: LCS12

Primary system organ class<br>Preferred term<br>MedDRA version 14.0	YEAR OF ONSET	N (%)	subject identifier
Muscle tightness	1st year	4/1432 (0.3%)	141421, 160427, 160601, 161420
	2nd year	3/1166 (0.3%)	160328, 161305, 161313
	3rd year	1/ 963 (0.1%)	161301
	Overall	8/1432 (0.6%)	141421, 160328, 160427, 160601, 161301, 161305, 161313, 161420
Musculoskeletal chest pain	3rd year	2/ 963 (0.2%)	244412, 245528
	Overall	2/1432 (0.1%)	244412, 245528
Musculoskeletal pain	1st year	6/1432 (0.4%)	160129, 160624, 160625, 160756, 241142, 242307
	2nd year	1/1166 (<0.1%)	161221
	3rd year	2/ 963 (0.2%)	242906, 244707
	Overall	9/1432 (0.6%)	160129, 160624, 160625, 160756, 161221, 241142, 242307, 242906, 244707
Myalgia	1st year	7/1432 (0.5%)	120604, 140921, 161017, 210136, 241807, 243958, 244509
	2nd year	2/1166 (0.2%)	140105, 160569
	3rd year	1/ 963 (0.1%)	160569
	Overall	9/1432 (0.6%)	120604, 140105, 140921, 160569, 161017, 210136, 241807, 243958, 244509
Neck pain	1st year	3/1432 (0.2%)	140212, 190228, 240622
	2nd year	5/1166 (0.4%)	120119, 161440, 210201, 230115, 244511
	3rd year	4/ 963 (0.4%)	120315, 120320, 120611, 120621
	Overall	12/1432 (0.8%)	120119, 120315, 120320, 120611, 120621, 140212, 161440, 190228, 210201, 230115, 240622, 244511
Osteitis	1st year	1/1432 (<0.1%)	200904
	2nd year	1/1166 (<0.1%)	180652
	Overall	2/1432 (0.1%)	180652, 200904
Pain in extremity	1st year	4/1432 (0.3%)	140304, 160801, 200709, 244117
	2nd year	1/1166 (<0.1%)	161301
	3rd year	1/ 963 (0.1%)	244105
	Overall	6/1432 (0.4%)	140304, 160801, 161301, 200709, 244105, 244117
Patellofemoral pain syndrome	1st year	1/1432 (<0.1%)	161421
	Overall	1/1432 (<0.1%)	161421

Table 14.3.1 / 10: Number of subjects with adverse events including subject identifier and year by treatment (FAS)

TREATMENT: LCS12

Primary system organ class<br>Preferred term<br>MedDRA version 14.0	YEAR OF ONSET	N (%)	subject identifier
Psoriatic arthropathy	3rd year	1/ 963 (0.1%)	231012
	Overall	1/1432 (<0.1%)	231012
Rotator cuff syndrome	1st year	1/1432 (<0.1%)	160110
	Overall	1/1432 (<0.1%)	160110
Scleroderma	2nd year	1/1166 (<0.1%)	161301
	Overall	1/1432 (<0.1%)	161301
Spinal osteoarthritis	2nd year	1/1166 (<0.1%)	180605
	Overall	1/1432 (<0.1%)	180605
Synovitis	1st year	1/1432 (<0.1%)	180642
	Overall	1/1432 (<0.1%)	180642
Temporomandibular joint syndrome	1st year	1/1432 (<0.1%)	150145
	Overall	1/1432 (<0.1%)	150145
Tendon pain	2nd year	1/1166 (<0.1%)	161440
	Overall	1/1432 (<0.1%)	161440
Tendonitis	1st year	3/1432 (0.2%)	180715, 200528, 244402
	2nd year	2/1166 (0.2%)	200709, 240712
	Overall	5/1432 (0.3%)	180715, 200528, 200709, 240712, 244402
Torticollis	1st year	1/1432 (<0.1%)	161109
	Overall	1/1432 (<0.1%)	161109
Neoplasms benign, malignant and unspecified (incl cysts and polyps)	1st year	20/1432 (1.4%)	
	2nd year	14/1166 (1.2%)	
	3rd year	14/ 963 (1.5%)	
	Overall	45/1432 (3.1%)	
Acrochordon	2nd year	1/1166 (<0.1%)	241706
	Overall	1/1432 (<0.1%)	241706

Table 14.3.1 / 10: Number of subjects with adverse events including subject identifier and year by treatment (FAS)

TREATMENT: LCS12

Primary system organ class<br>Preferred term<br>MedDRA version 14.0	YEAR OF ONSET	N (%)	subject identifier
Acute leukaemia	3rd year	1/ 963 (0.1%)	246212
	Overall	1/1432 (<0.1%)	246212
Astrocytoma, low grade	1st year	1/1432 (<0.1%)	160329
	Overall	1/1432 (<0.1%)	160329
Benign breast neoplasm	1st year	1/1432 (<0.1%)	244119
	2nd year	1/1166 (<0.1%)	160904
	Overall	2/1432 (0.1%)	160904, 244119
Cervicitis human papilloma virus	3rd year	1/ 963 (0.1%)	161406
	Overall	1/1432 (<0.1%)	161406
Cervix neoplasm	3rd year	1/ 963 (0.1%)	190225
	Overall	1/1432 (<0.1%)	190225
Fibroadenoma of breast	1st year	2/1432 (0.1%)	170407, 242806
	2nd year	4/1166 (0.3%)	230804, 240622, 241531, 244509
	3rd year	3/ 963 (0.3%)	170407, 242806, 243916
	Overall	7/1432 (0.5%)	170407, 230804, 240622, 241531, 242806, 243916, 244509
Glomus tumour	1st year	1/1432 (<0.1%)	230401
	2nd year	1/1166 (<0.1%)	230401
	Overall	1/1432 (<0.1%)	230401
Haemangioma	1st year	1/1432 (<0.1%)	243965
	Overall	1/1432 (<0.1%)	243965
Lipoma of breast	2nd year	2/1166 (0.2%)	160928, 242906
	Overall	2/1432 (0.1%)	160928, 242906
Melanocytic naevus	1st year	4/1432 (0.3%)	161421, 161436, 161437, 245911
	2nd year	1/1166 (<0.1%)	241414
	3rd year	1/ 963 (0.1%)	242712
	Overall	6/1432 (0.4%)	161421, 161436, 161437, 241414, 242712, 245911

Table 14.3.1 / 10: Number of subjects with adverse events including subject identifier and year by treatment (FAS)

TREATMENT: LCS12

Primary system organ class / Preferred term / MedDRA version 14.0	YEAR OF ONSET	N (%)	subject identifier
Ovarian germ cell teratoma benign	1st year	3/1432 (0.2%)	140808, 161431, 244448
	3rd year	2/ 963 (0.2%)	160317, 161114
	Overall	5/1432 (0.3%)	140808, 160317, 161114, 161431, 244448
Skin papilloma	1st year	1/1432 (<0.1%)	140518
	Overall	1/1432 (<0.1%)	140518
Thyroid cancer	2nd year	1/1166 (<0.1%)	241153
	3rd year	1/ 963 (0.1%)	160519
	Overall	2/1432 (0.1%)	160519, 241153
Uterine leiomyoma	1st year	4/1432 (0.3%)	120242, 240841, 241190, 244132
	2nd year	3/1166 (0.3%)	160606, 180740, 244109
	3rd year	1/ 963 (0.1%)	200207
	Overall	8/1432 (0.6%)	120242, 160606, 180740, 200207, 240841, 241190, 244109, 244132
Vulvovaginal human papilloma virus infection	1st year	2/1432 (0.1%)	160609, 243830
	3rd year	3/ 963 (0.3%)	150112, 160418, 161512
	Overall	5/1432 (0.3%)	150112, 160418, 160609, 161512, 243830
Nervous system disorders	1st year	154/1432 (10.8%)	
	2nd year	44/1166 (3.8%)	
	3rd year	27/ 963 (2.8%)	
	Overall	200/1432 (14.0%)	
Amnesia	1st year	1/1432 (<0.1%)	243950
	Overall	1/1432 (<0.1%)	243950
Burning sensation	1st year	1/1432 (<0.1%)	120634
	Overall	1/1432 (<0.1%)	120634
Carpal tunnel syndrome	3rd year	1/ 963 (0.1%)	140105
	Overall	1/1432 (<0.1%)	140105
Cervicogenic headache	2nd year	1/1166 (<0.1%)	140802
	Overall	1/1432 (<0.1%)	140802

Table 14.3.1 / 10: Number of subjects with adverse events including subject identifier and year by treatment (FAS)

TREATMENT: LCS12

Primary system organ class<br>Preferred term<br>MedDRA version 14.0	YEAR OF ONSET	N (%)	subject identifier
Convulsion	2nd year	1/1166 (<0.1%)	241544
	Overall	1/1432 (<0.1%)	241544
Dizziness	1st year	7/1432 (0.5%)	140206, 140802, 141405, 160624, 200304, 244618, 246147
	2nd year	2/1166 (0.2%)	200112, 240739
	Overall	9/1432 (0.6%)	140206, 140802, 141405, 160624, 200112, 200304, 240739, 244618, 246147
Dizziness postural	1st year	1/1432 (<0.1%)	244438
	Overall	1/1432 (<0.1%)	244438
Facial spasm	3rd year	1/ 963 (0.1%)	200521
	Overall	1/1432 (<0.1%)	200521

Table 14.3.1 / 10: Number of subjects with adverse events including subject identifier and year by treatment (FAS)

TREATMENT: LCS12

Primary system organ class<br>Preferred term<br>MedDRA version 14.0	YEAR OF ONSET	N (%)	subject identifier
Headache	1st year	102/1432 (7.1%)	120104, 120113, 120315, 120320, 120411, 120419, 120602, 120604, 120608, 120610, 120611, 120613, 120621, 120626, 120630, 120632, 140105, 140206, 140301, 140302, 140304, 140518, 140605, 140905, 141301, 150106, 160105, 160208, 160302, 160331, 160506, 160511, 160569, 160605, 160606, 160610, 160616, 160624, 160631, 160637, 160640, 160717, 160756, 160962, 161003, 161018, 161023, 161027, 161301, 161303, 161304, 161305, 161313, 161316, 161403, 161407, 161532, 161533, 170704, 180735, 180736, 190402, 190409, 200919, 210409, 210412, 210415, 230214, 230303, 230406, 230603, 230819, 230824, 240217, 240345, 240701, 241102, 241122, 241131, 241159, 241185, 241190, 241228, 241305, 241525, 242307, 242320, 242903, 243720, 243904, 243929, 243979, 244109, 244125, 244430, 244438, 244518, 244914, 245518, 245519, 245803, 245928
	2nd year	29/1166 (2.5%)	120305, 120315, 120320, 120412, 120414, 120602, 140228, 140708, 150166, 160913, 161305, 161313, 180821, 190409, 190601, 200402, 200616, 200703, 210409, 230515, 230819, 231108, 240712, 242212, 243720, 244105, 244408, 244412, 244443
	3rd year	15/ 963 (1.6%)	120307, 120320, 120630, 140210, 140518, 141032, 160218, 160525, 160605, 160613, 190205, 241302, 242302, 243702, 244407
	Overall	133/1432 (9.3%)	120104, 120113, 120305, 120307, 120315, 120320, 120411, 120412, 120414, 120419, 120602, 120604, 120608, 120610, 120611, 120613, 120621, 120626, 120630, 120632, 140105, 140206, 140210, 140228, 140301, 140302, 140304, 140518, 140605, 140708, 140905, 141032, 141301, 150106, 150166, 160105, 160208, 160218, 160302, 160331, 160506, 160511, 160525, 160569, 160605, 160606, 160610, 160613, 160616, 160624,

Table 14.3.1 / 10: Number of subjects with adverse events including subject identifier and year by treatment (FAS)

TREATMENT: LCS12

Primary system organ class<br>Preferred term<br>MedDRA version 14.0	YEAR OF ONSET	N (%)	subject identifier
			160631, 160637, 160640, 160717, 160756, 160913, 160962, 161003, 161018, 161023, 161027, 161301, 161303, 161304, 161305, 161313, 161316, 161403, 161407, 161532, 161533, 170704, 180735, 180736, 180821, 190205, 190402, 190409, 190601, 200402, 200616, 200703, 200919, 210409, 210412, 210415, 230214, 230303, 230406, 230515, 230603, 230819, 230824, 231108, 240217, 240345, 240701, 240712, 241102, 241122, 241131, 241159, 241185, 241190, 241228, 241302, 241305, 241525, 242212, 242302, 242307, 242320, 242903, 243702, 243720, 243904, 243929, 243979, 244105, 244109, 244125, 244407, 244408, 244412, 244430, 244438, 244443, 244518, 244914, 245518, 245519, 245803, 245928
Hypoaesthesia	1st year	1/1432 (<0.1%)	246147
	2nd year	1/1166 (<0.1%)	244408
	Overall	2/1432 (0.1%)	244408, 246147
Migraine	1st year	22/1432 (1.5%)	140114, 160511, 160569, 161002, 161109, 170605, 200516, 200527, 200803, 230817, 240725, 241531, 241544, 241718, 242504, 243323, 243618, 243904, 244412, 244427, 244919, 245516
	2nd year	5/1166 (0.4%)	120602, 160625, 161301, 240728, 245403
	3rd year	8/ 963 (0.8%)	140105, 160625, 160637, 160738, 160960, 160981, 241159, 244408
	Overall	34/1432 (2.4%)	120602, 140105, 140114, 160511, 160569, 160625, 160637, 160738, 160960, 160981, 161002, 161109, 161301, 170605, 200516, 200527, 200803, 230817, 240725, 240728, 241159, 241531, 241544, 241718, 242504, 243323, 243618, 243904, 244408, 244412, 244427, 244919, 245403, 245516
Migraine with aura	1st year	3/1432 (0.2%)	200703, 241530, 245508
	2nd year	1/1166 (<0.1%)	161102
	Overall	4/1432 (0.3%)	161102, 200703, 241530, 245508

Table 14.3.1 / 10: Number of subjects with adverse events including subject identifier and year by treatment (FAS)

TREATMENT: LCS12

Primary system organ class<br>Preferred term<br>MedDRA version 14.0	YEAR OF ONSET	N (%)	subject identifier
Migraine without aura	1st year	1/1432 (<0.1%)	241551
	Overall	1/1432 (<0.1%)	241551
Morton's neuralgia	2nd year	1/1166 (<0.1%)	200614
	Overall	1/1432 (<0.1%)	200614
Multiple sclerosis	3rd year	1/ 963 (0.1%)	160637
	Overall	1/1432 (<0.1%)	160637
Nerve compression	2nd year	1/1166 (<0.1%)	244711
	3rd year	1/ 963 (0.1%)	244407
	Overall	2/1432 (0.1%)	244407, 244711
Presyncope	1st year	4/1432 (0.3%)	140521, 160985, 170609, 200613
	Overall	4/1432 (0.3%)	140521, 160985, 170609, 200613
Restless legs syndrome	1st year	1/1432 (<0.1%)	230401
	Overall	1/1432 (<0.1%)	230401
Sciatica	1st year	3/1432 (0.2%)	161112, 170605, 200612
	2nd year	1/1166 (<0.1%)	180733
	Overall	4/1432 (0.3%)	161112, 170605, 180733, 200612
Sinus headache	1st year	1/1432 (<0.1%)	244511
	Overall	1/1432 (<0.1%)	244511
Somnolence	1st year	2/1432 (0.1%)	120335, 140114
	Overall	2/1432 (0.1%)	120335, 140114
Status migrainosus	1st year	1/1432 (<0.1%)	242807
	Overall	1/1432 (<0.1%)	242807
Syncope	1st year	6/1432 (0.4%)	120619, 200412, 200908, 200923, 245701, 245710
	Overall	6/1432 (0.4%)	120619, 200412, 200908, 200923, 245701, 245710

Table 14.3.1 / 10: Number of subjects with adverse events including subject identifier and year by treatment (FAS)

TREATMENT: LCS12

Primary system organ class<br>Preferred term<br>MedDRA version 14.0	YEAR OF ONSET	N (%)	subject identifier
Tension headache	1st year	6/1432 (0.4%)	150125, 150166, 161312, 241718, 243206, 243231
	2nd year	3/1166 (0.3%)	150104, 242816, 243213
	3rd year	3/ 963 (0.3%)	161313, 230216, 245506
	Overall	12/1432 (0.8%)	150104, 150125, 150166, 161312, 161313, 230216, 241718, 242816, 243206, 243213, 243231, 245506
Thoracic outlet syndrome	2nd year	1/1166 (<0.1%)	200614
	Overall	1/1432 (<0.1%)	200614
VIIth nerve paralysis	1st year	2/1432 (0.1%)	160511, 190221
	Overall	2/1432 (0.1%)	160511, 190221
Pregnancy, puerperium and perinatal conditions	1st year	3/1432 (0.2%)	
	2nd year	2/1166 (0.2%)	
	3rd year	1/ 963 (0.1%)	
	Overall	6/1432 (0.4%)	
Abortion spontaneous	1st year	1/1432 (<0.1%)	245003
	2nd year	1/1166 (<0.1%)	190636
	3rd year	1/ 963 (0.1%)	140202
	Overall	3/1432 (0.2%)	140202, 190636, 245003
Ectopic pregnancy	1st year	2/1432 (0.1%)	120419, 160743
	2nd year	1/1166 (<0.1%)	230303
	Overall	3/1432 (0.2%)	120419, 160743, 230303
Premature separation of placenta	2nd year	1/1166 (<0.1%)	190636
	Overall	1/1432 (<0.1%)	190636
Psychiatric disorders	1st year	107/1432 (7.5%)	
	2nd year	55/1166 (4.7%)	
	3rd year	26/ 963 (2.7%)	
	Overall	173/1432 (12.1%)	
Adjustment disorder	1st year	1/1432 (<0.1%)	140817
	Overall	1/1432 (<0.1%)	140817

Table 14.3.1 / 10: Number of subjects with adverse events including subject identifier and year by treatment (FAS)

TREATMENT: LCS12

Primary system organ class<br>Preferred term<br>MedDRA version 14.0	YEAR OF ONSET	N (%)	subject identifier
Affect lability	1st year	2/1432 (0.1%)	140808, 200923
	2nd year	2/1166 (0.2%)	244931, 245910
	Overall	4/1432 (0.3%)	140808, 200923, 244931, 245910
Aggression	1st year	1/1432 (<0.1%)	140114
	Overall	1/1432 (<0.1%)	140114
Anxiety	1st year	17/1432 (1.2%)	140807, 240209, 240217, 240510, 240704, 241142, 242818, 243121, 243913, 243965, 244608, 245024, 245027, 245411, 245514, 245910, 245923
	2nd year	13/1166 (1.1%)	120632, 140507, 141028, 210519, 230720, 240337, 240634, 241015, 241531, 243904, 243946, 244918, 245904
	3rd year	9/ 963 (0.9%)	120630, 150153, 230720, 240505, 242818, 244412, 244430, 244613, 245410
	Overall	37/1432 (2.6%)	120630, 120632, 140507, 140807, 141028, 150153, 210519, 230720, 240209, 240217, 240337, 240505, 240510, 240634, 240704, 241015, 241142, 241531, 242818, 243121, 243904, 243913, 243946, 243965, 244412, 244430, 244608, 244613, 244918, 245024, 245027, 245410, 245411, 245514, 245904, 245910, 245923
Anxiety disorder	3rd year	1/ 963 (0.1%)	180511
	Overall	1/1432 (<0.1%)	180511
Attention deficit/hyperactivity disorder	1st year	1/1432 (<0.1%)	241414
	2nd year	1/1166 (<0.1%)	200115
	3rd year	2/ 963 (0.2%)	244412, 244428
	Overall	4/1432 (0.3%)	200115, 241414, 244412, 244428
Bipolar I disorder	1st year	1/1432 (<0.1%)	241174
	Overall	1/1432 (<0.1%)	241174
Bipolar disorder	2nd year	1/1166 (<0.1%)	240116
	Overall	1/1432 (<0.1%)	240116
Burnout syndrome	1st year	1/1432 (<0.1%)	160509
	Overall	1/1432 (<0.1%)	160509

Table 14.3.1 / 10: Number of subjects with adverse events including subject identifier and year by treatment (FAS)

TREATMENT: LCS12

Primary system organ class<br>Preferred term<br>MedDRA version 14.0	YEAR OF ONSET	N (%)	subject identifier
Daydreaming	2nd year	1/1166 (<0.1%)	243611
	Overall	1/1432 (<0.1%)	243611
Depressed mood	1st year	4/1432 (0.3%)	141301, 141426, 230214, 242917
	2nd year	1/1166 (<0.1%)	160734
	Overall	5/1432 (0.3%)	141301, 141426, 160734, 230214, 242917
Depression	1st year	27/1432 (1.9%)	140817, 141034, 150109, 150128, 160431, 160434, 160550, 160605, 160806, 160905, 160935, 161221, 200619, 210203, 210404, 230310, 240352, 240644, 241108, 241119, 241531, 241541, 241717, 241718, 243821, 243950, 244402
	2nd year	18/1166 (1.5%)	120417, 140520, 141402, 141419, 160329, 160509, 170410, 230720, 241541, 243103, 243611, 243830, 243946, 243973, 244417, 244422, 244918, 245808
	3rd year	8/ 963 (0.8%)	150138, 150166, 150202, 161403, 161513, 210411, 230720, 243907
	Overall	51/1432 (3.6%)	120417, 140520, 140817, 141034, 141402, 141419, 150109, 150128, 150138, 150166, 150202, 160329, 160431, 160434, 160509, 160550, 160605, 160806, 160905, 160935, 161221, 161403, 161513, 170410, 200619, 210203, 210404, 210411, 230310, 230720, 240352, 240644, 241108, 241119, 241531, 241541, 241717, 241718, 243103, 243611, 243821, 243830, 243907, 243946, 243950, 243973, 244402, 244417, 244422, 244918, 245808
Depression suicidal	2nd year	1/1166 (<0.1%)	244422
	Overall	1/1432 (<0.1%)	244422
Depressive symptom	1st year	1/1432 (<0.1%)	230714
	Overall	1/1432 (<0.1%)	230714
Drug dependence	1st year	1/1432 (<0.1%)	241708
	Overall	1/1432 (<0.1%)	241708

Table 14.3.1 / 10: Number of subjects with adverse events including subject identifier and year by treatment (FAS)

TREATMENT: LCS12

Primary system organ class<br>Preferred term<br>MedDRA version 14.0	YEAR OF ONSET	N (%)	subject identifier
Fear	3rd year	1/ 963 (0.1%)	160551
	Overall	1/1432 (<0.1%)	160551
Generalised anxiety disorder	1st year	3/1432 (0.2%)	161026, 243302, 243502
	Overall	3/1432 (0.2%)	161026, 243302, 243502
Hallucination, auditory	1st year	1/1432 (<0.1%)	140114
	Overall	1/1432 (<0.1%)	140114
Insomnia	1st year	16/1432 (1.1%)	140807, 160110, 161411, 190203, 210203, 230117, 230401, 230720, 240217, 240505, 241174, 241405, 243323, 243617, 243950, 244417
	2nd year	4/1166 (0.3%)	120310, 140802, 161440, 231107
	3rd year	3/ 963 (0.3%)	160904, 240505, 243907
	Overall	22/1432 (1.5%)	120310, 140802, 140807, 160110, 160904, 161411, 161440, 190203, 210203, 230117, 230401, 230720, 231107, 240217, 240505, 241174, 241405, 243323, 243617, 243907, 243950, 244417
Libido decreased	1st year	18/1432 (1.3%)	140113, 140813, 160522, 161303, 200919, 210120, 230122, 230824, 240401, 241102, 241149, 241174, 241193, 243026, 243929, 244443, 245027, 245531
	2nd year	8/1166 (0.7%)	161002, 170801, 240401, 243103, 243314, 244430, 244522, 244918
	3rd year	2/ 963 (0.2%)	180620, 230804
	Overall	27/1432 (1.9%)	140113, 140813, 160522, 161002, 161303, 170801, 180620, 200919, 210120, 230122, 230804, 230824, 240401, 241102, 241149, 241174, 241193, 243026, 243103, 243314, 243929, 244430, 244443, 244522, 244918, 245027, 245531
Libido increased	1st year	1/1432 (<0.1%)	160520
	2nd year	1/1166 (<0.1%)	245910
	Overall	2/1432 (0.1%)	160520, 245910
Loss of libido	1st year	3/1432 (0.2%)	161110, 230413, 240217
	Overall	4/1432 (0.3%)	161110, 161111, 230413, 240217

Table 14.3.1 / 10: Number of subjects with adverse events including subject identifier and year by treatment (FAS)

TREATMENT: LCS12

Primary system organ class<br>Preferred term<br>MedDRA version 14.0	YEAR OF ONSET	N (%)	subject identifier
Mood altered	1st year	10/1432 (0.7%)	160301, 210503, 230613, 230903, 230917, 241014, 242326, 242701, 245024, 245026
	2nd year	5/1166 (0.4%)	141409, 160960, 161532, 180409, 241414
	Overall	15/1432 (1.0%)	141409, 160301, 160960, 161532, 180409, 210503, 230613, 230903, 230917, 241014, 241414, 242326, 242701, 245024, 245026
Mood swings	1st year	9/1432 (0.6%)	200919, 240217, 241530, 241531, 243213, 243907, 244122, 244203, 246144
	2nd year	3/1166 (0.3%)	141412, 241133, 244402
	Overall	12/1432 (0.8%)	141412, 200919, 240217, 241133, 241530, 241531, 243213, 243907, 244122, 244203, 244402, 246144
Nicotine dependence	3rd year	1/ 963 (0.1%)	140605
	Overall	1/1432 (<0.1%)	140605
Panic attack	2nd year	1/1166 (<0.1%)	241531
	Overall	1/1432 (<0.1%)	241531
Panic disorder	2nd year	1/1166 (<0.1%)	160212
	Overall	1/1432 (<0.1%)	160212
Personality disorder	3rd year	1/ 963 (0.1%)	140817
	Overall	1/1432 (<0.1%)	140817
Post-traumatic stress disorder	2nd year	1/1166 (<0.1%)	200612
	Overall	1/1432 (<0.1%)	200612
Sleep disorder	1st year	3/1432 (0.2%)	160541, 161117, 230613
	2nd year	2/1166 (0.2%)	160541, 160615
	3rd year	1/ 963 (0.1%)	230603
	Overall	5/1432 (0.3%)	160541, 160615, 161117, 230603, 230613
Stress	1st year	2/1432 (0.1%)	200528, 240217
	3rd year	1/ 963 (0.1%)	120315
	Overall	3/1432 (0.2%)	120315, 200528, 240217
Suicide attempt	2nd year	1/1166 (<0.1%)	244422
	Overall	1/1432 (<0.1%)	244422

Table 14.3.1 / 10: Number of subjects with adverse events including subject identifier and year by treatment (FAS)

TREATMENT: LCS12

Primary system organ class<br>Preferred term<br>MedDRA version 14.0	YEAR OF ONSET	N (%)	subject identifier
Renal and urinary disorders	1st year	25/1432 (1.7%)	
	2nd year	8/1166 (0.7%)	
	3rd year	10/ 963 (1.0%)	
	Overall	41/1432 (2.9%)	
Calculus bladder	1st year	1/1432 (<0.1%)	120634
	Overall	1/1432 (<0.1%)	120634
Cystitis haemorrhagic	1st year	1/1432 (<0.1%)	140208
	Overall	1/1432 (<0.1%)	140208
Dysuria	1st year	9/1432 (0.6%)	140510, 161304, 200901, 210101, 210602, 241133, 243972, 244117, 245803
	2nd year	3/1166 (0.3%)	150108, 150143, 150506
	3rd year	3/ 963 (0.3%)	150143, 150151, 200911
	Overall	14/1432 (1.0%)	140510, 150108, 150143, 150151, 150506, 161304, 200901, 200911, 210101, 210602, 241133, 243972, 244117, 245803
Haematuria	1st year	1/1432 (<0.1%)	240209
	3rd year	1/ 963 (0.1%)	245702
	Overall	2/1432 (0.1%)	240209, 245702
Micturition urgency	1st year	1/1432 (<0.1%)	244701
	2nd year	1/1166 (<0.1%)	244607
	3rd year	1/ 963 (0.1%)	244607
	Overall	2/1432 (0.1%)	244607, 244701
Nephrolithiasis	1st year	4/1432 (0.3%)	240525, 240721, 243903, 244707
	2nd year	1/1166 (<0.1%)	180821
	3rd year	3/ 963 (0.3%)	141104, 241725, 244523
	Overall	8/1432 (0.6%)	141104, 180821, 240525, 240721, 241725, 243903, 244523, 244707
Pollakiuria	1st year	3/1432 (0.2%)	200219, 244117, 244701
	3rd year	1/ 963 (0.1%)	161322
	Overall	4/1432 (0.3%)	161322, 200219, 244117, 244701

Table 14.3.1 / 10: Number of subjects with adverse events including subject identifier and year by treatment (FAS)

TREATMENT: LCS12

Primary system organ class<br>Preferred term<br>MedDRA version 14.0	YEAR OF ONSET	N (%)	subject identifier
Polyuria	2nd year	1/1166 (<0.1%)	244918
	Overall	1/1432 (<0.1%)	244918
Renal pain	1st year	1/1432 (<0.1%)	242506
	Overall	1/1432 (<0.1%)	242506
Strangury	1st year	1/1432 (<0.1%)	230501
	Overall	1/1432 (<0.1%)	230501
Stress urinary incontinence	3rd year	1/ 963 (0.1%)	245503
	Overall	1/1432 (<0.1%)	245503
Urinary incontinence	1st year	2/1432 (0.1%)	160402, 161539
	2nd year	1/1166 (<0.1%)	170414
	Overall	3/1432 (0.2%)	160402, 161539, 170414
Urinary tract disorder	1st year	1/1432 (<0.1%)	161318
	Overall	1/1432 (<0.1%)	161318
Urinary tract inflammation	1st year	1/1432 (<0.1%)	160220
	Overall	1/1432 (<0.1%)	160220
Urinary tract pain	1st year	1/1432 (<0.1%)	150167
	2nd year	1/1166 (<0.1%)	150123
	Overall	2/1432 (0.1%)	150123, 150167
Reproductive system and breast disorders	1st year	478/1432 (33.4%)	
	2nd year	213/1166 (18.3%)	
	3rd year	161/ 963 (16.7%)	
	Overall	681/1432 (47.6%)	
Adenomyosis	3rd year	1/ 963 (0.1%)	242806
	Overall	1/1432 (<0.1%)	242806
Adnexa uteri mass	3rd year	1/ 963 (0.1%)	241305
	Overall	1/1432 (<0.1%)	241305

Table 14.3.1 / 10: Number of subjects with adverse events including subject identifier and year by treatment (FAS)

TREATMENT: LCS12

Primary system organ class<br>Preferred term<br>MedDRA version 14.0	YEAR OF ONSET	N (%)	subject identifier
Adnexa uteri pain	1st year	2/1432 (0.1%)	243828, 243929
	3rd year	1/ 963 (0.1%)	140105
	Overall	3/1432 (0.2%)	140105, 243828, 243929
Atrophic vulvovaginitis	2nd year	1/1166 (<0.1%)	244918
	Overall	1/1432 (<0.1%)	244918
Breast calcifications	1st year	1/1432 (<0.1%)	241605
	Overall	1/1432 (<0.1%)	241605
Breast cyst	1st year	2/1432 (0.1%)	150125, 170407
	2nd year	1/1166 (<0.1%)	141019
	Overall	3/1432 (0.2%)	141019, 150125, 170407
Breast discomfort	1st year	4/1432 (0.3%)	161212, 180342, 180668, 230917
	Overall	4/1432 (0.3%)	161212, 180342, 180668, 230917
Breast dysplasia	3rd year	1/ 963 (0.1%)	120401
	Overall	1/1432 (<0.1%)	120401
Breast engorgement	2nd year	1/1166 (<0.1%)	190216
	Overall	1/1432 (<0.1%)	190216
Breast mass	1st year	3/1432 (0.2%)	141026, 241704, 243929
	2nd year	1/1166 (<0.1%)	200914
	3rd year	3/ 963 (0.3%)	242320, 243914, 244919
	Overall	7/1432 (0.5%)	141026, 200914, 241704, 242320, 243914, 243929, 244919

Table 14.3.1 / 10: Number of subjects with adverse events including subject identifier and year by treatment (FAS)

TREATMENT: LCS12

Primary system organ class<br>Preferred term<br>MedDRA version 14.0	YEAR OF ONSET	N (%)	subject identifier
Breast pain	1st year	23/1432 (1.6%)	120320, 120403, 120613, 140105, 140117, 141027, 150104, 150125, 150142, 160803, 160951, 161102, 170402, 170407, 170605, 180136, 190128, 200708, 200802, 210127, 230908, 241416, 244412
	2nd year	9/1166 (0.8%)	120322, 120323, 140519, 150166, 150214, 180409, 200908, 230613, 240622
	3rd year	6/ 963 (0.6%)	160571, 160717, 180122, 190132, 200708, 230613
	Overall	36/1432 (2.5%)	120320, 120322, 120323, 120403, 120613, 140105, 140117, 140519, 141027, 150104, 150125, 150142, 150166, 150214, 160571, 160717, 160803, 160951, 161102, 170402, 170407, 170605, 180122, 180136, 180409, 190128, 190132, 200708, 200802, 200908, 210127, 230613, 230908, 240622, 241416, 244412
Breast swelling	1st year	1/1432 (<0.1%)	161102
	Overall	1/1432 (<0.1%)	161102
Breast tenderness	1st year	20/1432 (1.4%)	120411, 120630, 120632, 141201, 141412, 160113, 160624, 160631, 161406, 210415, 230603, 231112, 241307, 241504, 242917, 243022, 243907, 244618, 245702, 246116
	2nd year	11/1166 (0.9%)	120613, 141409, 160405, 160439, 160963, 231018, 241019, 242218, 242307, 245710, 245910
	3rd year	2/ 963 (0.2%)	140202, 160631
	Overall	32/1432 (2.2%)	120411, 120613, 120630, 120632, 140202, 141201, 141409, 141412, 160113, 160405, 160439, 160624, 160631, 160963, 161406, 210415, 230603, 231018, 231112, 241019, 241307, 241504, 242218, 242307, 242917, 243022, 243907, 244618, 245702, 245710, 245910, 246116
Cervical discharge	1st year	1/1432 (<0.1%)	243322
	2nd year	1/1166 (<0.1%)	241127
	Overall	2/1432 (0.1%)	241127, 243322

Table 14.3.1 / 10: Number of subjects with adverse events including subject identifier and year by treatment (FAS)

TREATMENT: LCS12

Primary system organ class<br>Preferred term<br>MedDRA version 14.0	YEAR OF ONSET	N (%)	subject identifier
Cervical dysplasia	1st year	17/1432 (1.2%)	140609, 141101, 160536, 160539, 161514, 180412, 180426, 200111, 230120, 230511, 240612, 240623, 240638, 241704, 242807, 243816, 243820
	2nd year	46/1166 (3.9%)	120608, 140204, 140519, 150506, 160103, 160118, 160129, 160201, 160806, 160911, 160913, 160985, 161210, 170304, 170306, 180125, 180345, 180520, 180524, 190225, 200304, 210124, 210136, 210205, 230816, 230824, 231018, 240310, 240643, 240712, 241005, 241201, 241204, 241414, 241541, 242313, 242316, 243012, 243206, 243220, 243311, 244422, 244504, 244710, 245423, 245525
	3rd year	50/ 963 (5.2%)	120310, 140301, 141423, 150142, 150506, 160437, 160624, 160711, 160982, 160985, 161406, 161414, 170608, 180136, 180325, 180425, 180514, 180520, 180603, 180707, 180728, 190203, 190409, 200626, 210124, 210125, 210136, 210203, 210411, 230406, 230413, 230607, 230913, 240215, 240357, 240736, 241028, 241536, 241704, 242212, 242302, 242818, 243012, 243609, 243913, 244425, 245014, 245432, 245904, 246214
	Overall	107/1432 (7.5%)	120310, 120608, 140204, 140301, 140519, 140609, 141101, 141423, 150142, 150506, 160103, 160118, 160129, 160201, 160437, 160536, 160539, 160624, 160711, 160806, 160911, 160913, 160982, 160985, 161210, 161406, 161414, 161514, 170304, 170306, 170608, 180125, 180136, 180325, 180345, 180412, 180425, 180426, 180514, 180520, 180524, 180603, 180707, 180728, 190203, 190225, 190409, 200111, 200304, 200626, 210124, 210125, 210136, 210203, 210205, 210411, 230120, 230406, 230413, 230511, 230607, 230816, 230824, 230913, 231018, 240215, 240310, 240357, 240612, 240623, 240638, 240643, 240712, 240736, 240813, 241005, 241028, 241201, 241204, 241414, 241536, 241541, 241704, 242212, 242302, 242313, 242316, 242807, 242818, 243012, 243206, 243220, 243311, 243609, 243816, 243820, 243913, 244422, 244425, 244504,

Table 14.3.1 / 10: Number of subjects with adverse events including subject identifier and year by treatment (FAS)

TREATMENT: LCS12

Primary system organ class<br>Preferred term<br>MedDRA version 14.0	YEAR OF ONSET	N (%)	subject identifier
			244710, 245014, 245423, 245432, 245525, 245904, 246214
Cervical polyp	1st year	1/1432 (<0.1%)	240520
	Overall	1/1432 (<0.1%)	240520
Cervix inflammation	2nd year	1/1166 (<0.1%)	170608
	Overall	1/1432 (<0.1%)	170608
Coital bleeding	1st year	8/1432 (0.6%)	200504, 240825, 240904, 243830, 243939, 244119, 245503, 245508
	2nd year	3/1166 (0.3%)	150110, 190613, 245910
	3rd year	3/ 963 (0.3%)	120623, 161511, 240701
	Overall	14/1432 (1.0%)	120623, 150110, 161511, 190613, 200504, 240701, 240825, 240904, 243830, 243939, 244119, 245503, 245508, 245910
Dysfunctional uterine bleeding	1st year	3/1432 (0.2%)	243311, 243314, 243327
	Overall	3/1432 (0.2%)	243311, 243314, 243327

Table 14.3.1 / 10: Number of subjects with adverse events including subject identifier and year by treatment (FAS)

TREATMENT: LCS12

Primary system organ class<br>Preferred term<br>MedDRA version 14.0	YEAR OF ONSET	N (%)	subject identifier
Dysmenorrhoea	1st year	104/1432 (7.3%)	120310, 120326, 120329, 120613, 140529, 140609, 140904, 141032, 141201, 150104, 150106, 150119, 150136, 150142, 150151, 150153, 150163, 150167, 160106, 160209, 160309, 160328, 160625, 160626, 160631, 160633, 160704, 160712, 160713, 160720, 160721, 160725, 160757, 160801, 160803, 160971, 160988, 161001, 161002, 161003, 161012, 161018, 161027, 161111, 161112, 161114, 161212, 161305, 161411, 161424, 190118, 190119, 190225, 200104, 200119, 200234, 200527, 200611, 200615, 200629, 200634, 200911, 200923, 210120, 210130, 210404, 210412, 210415, 230310, 230406, 230408, 230603, 230613, 230712, 230719, 230802, 230804, 230805, 230817, 230819, 230824, 231018, 240309, 240366, 240904, 241033, 241307, 241708, 242504, 243021, 243925, 243958, 243972, 244112, 244119, 244122, 244125, 244132, 244414, 244508, 244509, 244511, 245809, 245934
	2nd year	20/1166 (1.7%)	120307, 120310, 120320, 150110, 160328, 161026, 161106, 161424, 190216, 190225, 200304, 240135, 241156, 241302, 241525, 241551, 242302, 242907, 245514, 245518
	3rd year	13/ 963 (1.3%)	120320, 160525, 160639, 160918, 161109, 161301, 190132, 190133, 190225, 200109, 230603, 243820, 244407
	Overall	130/1432 (9.1%)	120307, 120310, 120320, 120326, 120329, 120613, 140529, 140609, 140904, 141032, 141201, 150104, 150106, 150110, 150119, 150136, 150142, 150151, 150153, 150163, 150167, 160106, 160209, 160309, 160328, 160525, 160625, 160626, 160631, 160633, 160639, 160704, 160712, 160713, 160720, 160721, 160725, 160757, 160801, 160803, 160918, 160971, 160988, 161001, 161002, 161003, 161012, 161018, 161026, 161027, 161106, 161109, 161111, 161112, 161114, 161212, 161301, 161305, 161411, 161424, 190118, 190119, 190132, 190133, 190216, 190225, 200104, 200109, 200119, 200234, 200304, 200527, 200611, 200615, 200629,

Table 14.3.1 / 10: Number of subjects with adverse events including subject identifier and year by treatment (FAS)

TREATMENT: LCS12

Primary system organ class<br>Preferred term<br>MedDRA version 14.0	YEAR OF ONSET	N (%)	subject identifier
			200634, 200911, 200923, 210120, 210130, 210404, 210412, 210415, 230310, 230406, 230408, 230603, 230613, 230712, 230719, 230802, 230804, 230805, 230817, 230819, 230824, 231018, 240135, 240309, 240366, 240904, 241033, 241156, 241302, 241307, 241525, 241551, 241708, 242302, 242504, 242907, 243021, 243820, 243925, 243958, 243972, 244112, 244119, 244122, 244125, 244132, 244407, 244414, 244508, 244509, 244511, 245514, 245518, 245809, 245934
Dyspareunia	1st year	24/1432 (1.7%)	140502, 140515, 140529, 141423, 160207, 160803, 210101, 210111, 231016, 240209, 241133, 241532, 242917, 243009, 243012, 243220, 243224, 243939, 243948, 243979, 244450, 244917, 244919, 245809
	2nd year	7/1166 (0.6%)	140817, 150125, 231103, 240520, 241547, 241725, 243220
	3rd year	3/ 963 (0.3%)	190132, 245421, 245519
	Overall	33/1432 (2.3%)	140502, 140515, 140529, 140817, 141423, 150125, 160207, 160803, 190132, 210101, 210111, 231016, 231103, 240209, 240520, 241133, 241532, 241547, 241725, 242917, 243009, 243012, 243220, 243224, 243939, 243948, 243979, 244450, 244917, 244919, 245421, 245519, 245809
Ectropion of cervix	1st year	2/1432 (0.1%)	190411, 200517
	Overall	2/1432 (0.1%)	190411, 200517
Endometriosis	1st year	4/1432 (0.3%)	200705, 240841, 244701, 245025
	2nd year	3/1166 (0.3%)	160973, 200708, 240110
	3rd year	1/ 963 (0.1%)	161114
	Overall	8/1432 (0.6%)	160973, 161114, 200705, 200708, 240110, 240841, 244701, 245025
Fibrocystic breast disease	1st year	1/1432 (<0.1%)	241536
	2nd year	2/1166 (0.2%)	140807, 244918
	3rd year	1/ 963 (0.1%)	244918
	Overall	3/1432 (0.2%)	140807, 241536, 244918

Table 14.3.1 / 10: Number of subjects with adverse events including subject identifier and year by treatment (FAS)

TREATMENT: LCS12

Primary system organ class<br>Preferred term<br>MedDRA version 14.0	YEAR OF ONSET	N (%)	subject identifier
Galactorrhoea	1st year	3/1432 (0.2%)	230221, 240115, 243514
	2nd year	1/1166 (<0.1%)	245808
	Overall	4/1432 (0.3%)	230221, 240115, 243514, 245808
Genital discharge	1st year	4/1432 (0.3%)	140811, 150156, 170702, 190409
	2nd year	5/1166 (0.4%)	141402, 150123, 150168, 160631, 245516
	3rd year	1/ 963 (0.1%)	190409
	Overall	9/1432 (0.6%)	140811, 141402, 150123, 150156, 150168, 160631, 170702, 190409, 245516
Genital haemorrhage	1st year	1/1432 (<0.1%)	241005
	Overall	1/1432 (<0.1%)	241005
Genital lesion	1st year	2/1432 (0.1%)	244105, 244302
	Overall	2/1432 (0.1%)	244105, 244302
Haemorrhagic ovarian cyst	1st year	11/1432 (0.8%)	120104, 161027, 210411, 240508, 241023, 241231, 243307, 243502, 243948, 244430, 245516
	2nd year	2/1166 (0.2%)	160803, 161027
	3rd year	2/ 963 (0.2%)	140608, 243307
	Overall	13/1432 (0.9%)	120104, 140608, 160803, 161027, 210411, 240508, 241023, 241231, 243307, 243502, 243948, 244430, 245516
Hydrometra	1st year	1/1432 (<0.1%)	200505
	2nd year	1/1166 (<0.1%)	230714
	Overall	2/1432 (0.1%)	200505, 230714
Hypomenorrhoea	2nd year	1/1166 (<0.1%)	190203
	3rd year	1/ 963 (0.1%)	190204
	Overall	2/1432 (0.1%)	190203, 190204
Menometrorrhagia	1st year	1/1432 (<0.1%)	200213
	2nd year	1/1166 (<0.1%)	200908
	Overall	2/1432 (0.1%)	200213, 200908
Menorrhagia	1st year	5/1432 (0.3%)	141008, 200307, 240904, 243979, 245809
	3rd year	1/ 963 (0.1%)	200216
	Overall	6/1432 (0.4%)	141008, 200216, 200307, 240904, 243979, 245809

Table 14.3.1 / 10: Number of subjects with adverse events including subject identifier and year by treatment (FAS)

TREATMENT: LCS12

Primary system organ class<br>Preferred term<br>MedDRA version 14.0	YEAR OF ONSET	N (%)	subject identifier
Menstrual disorder	1st year	2/1432 (0.1%)	140905, 180666
	2nd year	1/1166 (<0.1%)	180652
	Overall	3/1432 (0.2%)	140905, 180652, 180666
Menstruation irregular	1st year	1/1432 (<0.1%)	242103
	2nd year	1/1166 (<0.1%)	230613
	3rd year	2/ 963 (0.2%)	180740, 244523
	Overall	4/1432 (0.3%)	180740, 230613, 242103, 244523
Metrorrhagia	1st year	11/1432 (0.8%)	120111, 140609, 150207, 160207, 160209, 180722, 230219, 230411, 242307, 242709, 243004
	2nd year	1/1166 (<0.1%)	200908
	3rd year	4/ 963 (0.4%)	170404, 190104, 190203, 200207
	Overall	16/1432 (1.1%)	120111, 140609, 150207, 160207, 160209, 170404, 180722, 190104, 190203, 200207, 200908, 230219, 230411, 242307, 242709, 243004
Nipple pain	1st year	2/1432 (0.1%)	240814, 241416
	3rd year	1/ 963 (0.1%)	245702
	Overall	3/1432 (0.2%)	240814, 241416, 245702

Table 14.3.1 / 10: Number of subjects with adverse events including subject identifier and year by treatment (FAS)

TREATMENT: LCS12

Primary system organ class<br>Preferred term<br>MedDRA version 14.0	YEAR OF ONSET	N (%)	subject identifier
Ovarian cyst	1st year	115/1432 (8.0%)	120127, 120218, 120240, 120242, 120305, 120320, 120418, 120613, 120621, 140105, 140301, 140807, 140808, 140904, 140911, 141028, 150143, 150503, 160207, 160208, 160213, 160321, 160538, 160547, 160551, 160557, 160567, 160569, 160732, 160803, 160806, 160951, 161224, 170901, 180116, 180615, 180662, 180681, 190110, 190126, 190133, 190218, 200111, 200201, 200219, 200412, 200625, 200629, 200705, 200709, 200911, 200923, 210101, 210116, 230117, 230303, 230904, 231102, 240309, 240337, 240357, 240366, 240370, 240521, 240606, 240625, 240630, 240639, 240824, 240827, 241004, 241015, 241023, 241033, 241105, 241123, 241156, 241167, 241190, 241410, 241424, 241521, 241545, 241548, 241553, 241704, 242326, 242903, 243103, 243121, 243231, 243609, 243816, 243939, 243948, 244003, 244004, 244422, 244427, 244428, 244430, 244442, 244509, 245004, 245008, 245433, 245439, 245702, 245709, 245807, 245923, 245934, 246122, 246210, 246216
	2nd year	57/1166 (4.9%)	120403, 120412, 120611, 140301, 140706, 140811, 150136, 160208, 160220, 160323, 160331, 160416, 160437, 160564, 160758, 160951, 161122, 161212, 161411, 180603, 180681, 190204, 190610, 200109, 200219, 200506, 200927, 210205, 210602, 230401, 230613, 231102, 240311, 240525, 240712, 240715, 241108, 241142, 241190, 241513, 241525, 241531, 241545, 241712, 242315, 243103, 243302, 243830, 244407, 244414, 244430, 244602, 244613, 244711, 245709, 245911, 245916
	3rd year	40/ 963 (4.2%)	120204, 120312, 120330, 120621, 140206, 140219, 140811, 160551, 160722, 160739, 160753, 180614, 180734, 190204, 200625, 231012, 231014, 240623, 240715, 240827, 241015, 241159, 241185, 241227, 241414, 241521, 241531, 241805, 242315, 242504, 242714, 242902, 243510, 244407, 244430, 245401, 245433, 245528, 245702, 246119

Table 14.3.1 / 10: Number of subjects with adverse events including subject identifier and year by treatment (FAS)

TREATMENT: LCS12

Primary system organ class<br>Preferred term<br>MedDRA version 14.0	YEAR OF ONSET	N (%)	subject identifier
	Overall	186/1432 (13.0%)	120127, 120204, 120218, 120240, 120242, 120305, 120312, 120320, 120330, 120403, 120412, 120418, 120611, 120613, 120621, 140105, 140206, 140219, 140301, 140706, 140807, 140808, 140811, 140904, 140911, 141028, 150136, 150143, 150503, 160207, 160208, 160213, 160220, 160321, 160323, 160331, 160416, 160437, 160538, 160547, 160551, 160557, 160564, 160567, 160569, 160722, 160732, 160739, 160753, 160758, 160803, 160806, 160951, 161122, 161212, 161224, 161411, 170901, 180116, 180603, 180614, 180615, 180662, 180681, 180734, 190110, 190126, 190133, 190204, 190218, 190610, 200109, 200111, 200201, 200219, 200412, 200506, 200625, 200629, 200705, 200709, 200911, 200923, 200927, 210101, 210116, 210205, 210602, 230117, 230303, 230401, 230613, 230904, 231012, 231014, 231102, 240309, 240311, 240337, 240357, 240366, 240370, 240521, 240525, 240606, 240623, 240625, 240630, 240639, 240712, 240715, 240824, 240827, 241004, 241015, 241023, 241033, 241105, 241108, 241123, 241142, 241156, 241159, 241167, 241185, 241190, 241227, 241410, 241414, 241424, 241513, 241521, 241525, 241531, 241545, 241548, 241553, 241704, 241712, 241805, 242315, 242326, 242504, 242714, 242902, 242903, 243103, 243121, 243231, 243302, 243510, 243609, 243816, 243830, 243939, 243948, 244003, 244004, 244407, 244414, 244422, 244427, 244428, 244430, 244442, 244509, 244602, 244613, 244711, 245004, 245008, 245401, 245433, 245439, 245528, 245702, 245709, 245807, 245911, 245916, 245923, 245934, 246119, 246122, 246210, 246216
Ovarian cyst ruptured	1st year	2/1432 (0.1%)	161416, 245923
	Overall	2/1432 (0.1%)	161416, 245923

Table 14.3.1 / 10: Number of subjects with adverse events including subject identifier and year by treatment (FAS)

TREATMENT: LCS12

Primary system organ class<br>Preferred term<br>MedDRA version 14.0	YEAR OF ONSET	N (%)	subject identifier
Ovarian enlargement	1st year	1/1432 (<0.1%)	141034
	Overall	1/1432 (<0.1%)	141034
Ovarian mass	2nd year	1/1166 (<0.1%)	243121
	Overall	1/1432 (<0.1%)	243121
Ovulation pain	1st year	4/1432 (0.3%)	160129, 160213, 242504, 244117
	2nd year	1/1166 (<0.1%)	161017
	3rd year	1/ 963 (0.1%)	243941
	Overall	6/1432 (0.4%)	160129, 160213, 161017, 242504, 243941, 244117
Parovarian cyst	1st year	2/1432 (0.1%)	161122, 210136
	3rd year	1/ 963 (0.1%)	161114
	Overall	3/1432 (0.2%)	161114, 161122, 210136
Pelvic congestion	1st year	1/1432 (<0.1%)	190409
	Overall	1/1432 (<0.1%)	190409
Pelvic discomfort	1st year	4/1432 (0.3%)	241120, 241133, 243009, 246217
	2nd year	1/1166 (<0.1%)	140302
	3rd year	1/ 963 (0.1%)	243231
	Overall	6/1432 (0.4%)	140302, 241120, 241133, 243009, 243231, 246217
Pelvic fluid collection	3rd year	1/ 963 (0.1%)	242902
	Overall	1/1432 (<0.1%)	242902

Table 14.3.1 / 10: Number of subjects with adverse events including subject identifier and year by treatment (FAS)

TREATMENT: LCS12

Primary system organ class<br>Preferred term<br>MedDRA version 14.0	YEAR OF ONSET	N (%)	subject identifier
Pelvic pain	1st year	71/1432 (5.0%)	120111, 120205, 120304, 120310, 120611, 120613, 120630, 120632, 140202, 140204, 140218, 140301, 140304, 150109, 150123, 150149, 150155, 150156, 150158, 150219, 160711, 160742, 161121, 161127, 161219, 161304, 170304, 170901, 200501, 200927, 210603, 230112, 230117, 230120, 230310, 230405, 230712, 230809, 230904, 240818, 240825, 241154, 241167, 241532, 241536, 241544, 241545, 241734, 242103, 242209, 242302, 242902, 242907, 242917, 243026, 243207, 243220, 243224, 243231, 243702, 243925, 243929, 244931, 245025, 245601, 245807, 246104, 246112, 246116, 246122, 246216
	2nd year	21/1166 (1.8%)	120320, 140301, 150110, 150136, 150153, 150214, 161219, 190601, 200908, 200911, 230112, 230602, 230613, 230818, 240209, 240326, 242218, 243220, 243222, 243511, 244402
	3rd year	10/ 963 (1.0%)	120108, 120310, 150142, 160710, 161121, 180116, 200304, 200528, 230125, 240828
	Overall	96/1432 (6.7%)	120108, 120111, 120205, 120304, 120310, 120320, 120611, 120613, 120630, 120632, 140202, 140204, 140218, 140301, 140304, 150109, 150110, 150123, 150136, 150142, 150149, 150153, 150155, 150156, 150158, 150214, 150219, 160710, 160711, 160742, 161121, 161127, 161219, 161304, 170304, 170901, 180116, 190601, 200304, 200501, 200528, 200908, 200911, 200927, 210603, 230112, 230117, 230120, 230125, 230310, 230405, 230602, 230613, 230712, 230809, 230818, 230904, 240209, 240326, 240818, 240825, 240828, 241154, 241167, 241532, 241536, 241544, 241545, 241734, 242103, 242209, 242218, 242302, 242902, 242907, 242917, 243026, 243207, 243220, 243222, 243224, 243231, 243511, 243702, 243925, 243929, 244402, 244931, 245025, 245601, 245807, 246104, 246112, 246116, 246122, 246216

Table 14.3.1 / 10: Number of subjects with adverse events including subject identifier and year by treatment (FAS)

TREATMENT: LCS12

Primary system organ class<br>Preferred term<br>MedDRA version 14.0	YEAR OF ONSET	N (%)	subject identifier
Pelvic prolapse	1st year	1/1432 (<0.1%)	240841
	Overall	1/1432 (<0.1%)	240841
Perineal pain	2nd year	1/1166 (<0.1%)	245910
	Overall	1/1432 (<0.1%)	245910
Polycystic ovaries	1st year	2/1432 (0.1%)	180739, 244918
	3rd year	3/ 963 (0.3%)	140918, 190418, 244918
	Overall	4/1432 (0.3%)	140918, 180739, 190418, 244918
Polymenorrhoea	2nd year	1/1166 (<0.1%)	190216
	Overall	1/1432 (<0.1%)	190216
Premenstrual syndrome	1st year	9/1432 (0.6%)	141423, 160918, 161539, 200614, 210106, 230517, 240721, 241175, 244132
	2nd year	6/1166 (0.5%)	200619, 230318, 231018, 241305, 244203, 245410
	3rd year	2/ 963 (0.2%)	160952, 242714
	Overall	17/1432 (1.2%)	141423, 160918, 160952, 161539, 200614, 200619, 210106, 230318, 230517, 231018, 240721, 241175, 241305, 242714, 244132, 244203, 245410
Pruritus genital	1st year	1/1432 (<0.1%)	240503
	Overall	1/1432 (<0.1%)	240503
Uterine cervical erosion	1st year	1/1432 (<0.1%)	200503
	Overall	1/1432 (<0.1%)	200503
Uterine cervical pain	1st year	2/1432 (0.1%)	140111, 244919
	Overall	2/1432 (0.1%)	140111, 244919
Uterine haemorrhage	1st year	6/1432 (0.4%)	120236, 210412, 240806, 241733, 244112, 244122
	2nd year	1/1166 (<0.1%)	161219
	3rd year	1/ 963 (0.1%)	180210
	Overall	8/1432 (0.6%)	120236, 161219, 180210, 210412, 240806, 241733, 244112, 244122
Uterine pain	1st year	1/1432 (<0.1%)	170304
	2nd year	1/1166 (<0.1%)	244914
	Overall	2/1432 (0.1%)	170304, 244914

Table 14.3.1 / 10: Number of subjects with adverse events including subject identifier and year by treatment (FAS)

TREATMENT: LCS12

Primary system organ class<br>Preferred term<br>MedDRA version 14.0	YEAR OF ONSET	N (%)	subject identifier
Uterine polyp	1st year	1/1432 (<0.1%)	200207
	2nd year	1/1166 (<0.1%)	245709
	Overall	2/1432 (0.1%)	200207, 245709
Uterine prolapse	3rd year	1/ 963 (0.1%)	245503
	Overall	1/1432 (<0.1%)	245503
Uterine spasm	1st year	27/1432 (1.9%)	140921, 141104, 161403, 161406, 161407, 161410, 161411, 161414, 161416, 161431, 161435, 161437, 161440, 200503, 210603, 210606, 230615, 241302, 241307, 241721, 242812, 242818, 244005, 244441, 244450, 244524, 245903
	2nd year	3/1166 (0.3%)	240223, 241725, 245807
	3rd year	1/ 963 (0.1%)	245807
	Overall	30/1432 (2.1%)	140921, 141104, 161403, 161406, 161407, 161410, 161411, 161414, 161416, 161431, 161435, 161437, 161440, 200503, 210603, 210606, 230615, 240223, 241302, 241307, 241721, 241725, 242812, 242818, 244005, 244441, 244450, 244524, 245807, 245903
Uterine tenderness	1st year	1/1432 (<0.1%)	241721
	Overall	1/1432 (<0.1%)	241721
Vaginal cyst	1st year	1/1432 (<0.1%)	241530
	Overall	1/1432 (<0.1%)	241530

Table 14.3.1 / 10: Number of subjects with adverse events including subject identifier and year by treatment (FAS)

TREATMENT: LCS12

Primary system organ class<br>Preferred term<br>MedDRA version 14.0	YEAR OF ONSET	N (%)	subject identifier
Vaginal discharge	1st year	36/1432 (2.5%)	120304, 120307, 120401, 120610, 120613, 120632, 120633, 140113, 140701, 160928, 160973, 161110, 180102, 180644, 200115, 200631, 200709, 200912, 210201, 210602, 230112, 230818, 230901, 241127, 241172, 241553, 242212, 243117, 243907, 243929, 244125, 244203, 244711, 245518, 245809, 246216
	2nd year	20/1166 (1.7%)	120418, 120611, 120623, 120630, 120632, 120633, 141402, 160914, 161117, 180109, 180136, 230509, 230613, 231112, 240135, 241129, 241421, 241553, 244109, 244914
	3rd year	6/ 963 (0.6%)	120113, 120307, 120610, 120623, 240612, 244711
	Overall	55/1432 (3.8%)	120113, 120304, 120307, 120401, 120418, 120610, 120611, 120613, 120623, 120630, 120632, 120633, 140113, 140701, 141402, 160914, 160928, 160973, 161110, 161117, 180102, 180109, 180136, 180644, 200115, 200631, 200709, 200912, 210201, 210602, 230112, 230509, 230613, 230818, 230901, 231112, 240135, 240612, 241127, 241129, 241172, 241421, 241553, 242212, 243117, 243907, 243929, 244109, 244125, 244203, 244711, 244914, 245518, 245809, 246216
Vaginal disorder	1st year	3/1432 (0.2%)	120409, 120411, 140802
	3rd year	1/ 963 (0.1%)	190133
	Overall	4/1432 (0.3%)	120409, 120411, 140802, 190133

Table 14.3.1 / 10: Number of subjects with adverse events including subject identifier and year by treatment (FAS)

TREATMENT: LCS12

Primary system organ class Preferred term MedDRA version 14.0	YEAR OF ONSET	N (%)	subject identifier
Vaginal haemorrhage	1st year	51/1432 (3.6%)	140813, 150119, 150158, 150163, 160436, 160505, 160535, 160712, 160726, 160729, 160732, 161225, 170101, 170304, 170801, 170802, 180335, 180635, 180741, 200119, 200503, 200710, 200908, 200914, 210109, 210126, 210516, 230405, 230817, 230904, 231017, 231022, 240112, 240366, 240639, 241122, 241221, 241410, 241715, 241811, 242103, 242223, 242701, 243224, 243720, 243910, 243919, 244524, 245809, 245929, 246217
	2nd year	13/1166 (1.1%)	140111, 150125, 180722, 200230, 200629, 200709, 210602, 240135, 240520, 240740, 241172, 244710, 244931
	3rd year	3/ 963 (0.3%)	160712, 240357, 240715
	Overall	66/1432 (4.6%)	140111, 140813, 150119, 150125, 150158, 150163, 160436, 160505, 160535, 160712, 160726, 160729, 160732, 161225, 170101, 170304, 170801, 170802, 180335, 180635, 180722, 180741, 200119, 200230, 200503, 200629, 200709, 200710, 200908, 200914, 210109, 210126, 210516, 210602, 230405, 230817, 230904, 231017, 231022, 240112, 240135, 240357, 240366, 240520, 240639, 240715, 240740, 241122, 241172, 241221, 241410, 241715, 241811, 242103, 242223, 242701, 243224, 243720, 243910, 243919, 244524, 244710, 244931, 245809, 245929, 246217
Vaginal lesion	1st year	1/1432 (<0.1%)	244132
	Overall	1/1432 (<0.1%)	244132
Vaginal odour	1st year	1/1432 (<0.1%)	245518
	2nd year	1/1166 (<0.1%)	244105
	3rd year	4/ 963 (0.4%)	141412, 244711, 245701, 245710
	Overall	6/1432 (0.4%)	141412, 244105, 244711, 245518, 245701, 245710
Vaginal wall congestion	2nd year	1/1166 (<0.1%)	244918
	Overall	1/1432 (<0.1%)	244918

Table 14.3.1 / 10: Number of subjects with adverse events including subject identifier and year by treatment (FAS)

TREATMENT: LCS12

Primary system organ class<br>Preferred term<br>MedDRA version 14.0	YEAR OF ONSET	N (%)	subject identifier
Vulval disorder	3rd year	1/ 963 (0.1%)	244105
	Overall	1/1432 (<0.1%)	244105
Vulvovaginal burning sensation	1st year	1/1432 (<0.1%)	140515
	Overall	1/1432 (<0.1%)	140515
Vulvovaginal discomfort	1st year	4/1432 (0.3%)	200916, 240623, 242103, 243003
	3rd year	2/ 963 (0.2%)	190203, 240622
	Overall	6/1432 (0.4%)	190203, 200916, 240622, 240623, 242103, 243003
Vulvovaginal dryness	1st year	2/1432 (0.1%)	160976, 230310
	2nd year	1/1166 (<0.1%)	241707
	Overall	3/1432 (0.2%)	160976, 230310, 241707
Vulvovaginal pain	1st year	2/1432 (0.1%)	160756, 243903
	3rd year	1/ 963 (0.1%)	244408
	Overall	3/1432 (0.2%)	160756, 243903, 244408
Vulvovaginal pruritus	1st year	10/1432 (0.7%)	120419, 140502, 140811, 160717, 190126, 190128, 200910, 242212, 242805, 243222
	2nd year	4/1166 (0.3%)	160406, 230604, 240740, 245807
	3rd year	3/ 963 (0.3%)	160576, 160712, 230604
	Overall	16/1432 (1.1%)	120419, 140502, 140811, 160406, 160576, 160712, 160717, 190126, 190128, 200910, 230604, 240740, 242212, 242805, 243222, 245807
Vulvovaginal swelling	2nd year	1/1166 (<0.1%)	244934
	Overall	1/1432 (<0.1%)	244934
Respiratory, thoracic and mediastinal disorders	1st year	36/1432 (2.5%)	
	2nd year	31/1166 (2.7%)	
	3rd year	14/ 963 (1.5%)	
	Overall	71/1432 (5.0%)	
Allergic sinusitis	1st year	1/1432 (<0.1%)	243941
	Overall	1/1432 (<0.1%)	243941

Table 14.3.1 / 10: Number of subjects with adverse events including subject identifier and year by treatment (FAS)

TREATMENT: LCS12

Primary system organ class<br>Preferred term<br>MedDRA version 14.0	YEAR OF ONSET	N (%)	subject identifier
Asthma	1st year	4/1432 (0.3%)	160564, 161203, 200202, 230221
	2nd year	6/1166 (0.5%)	120205, 160909, 161203, 170606, 243609, 245502
	3rd year	3/ 963 (0.3%)	160576, 160623, 161203
	Overall	11/1432 (0.8%)	120205, 160564, 160576, 160623, 160909, 161203, 170606, 200202, 230221, 243609, 245502
Bronchospasm	1st year	2/1432 (0.1%)	120604, 120621
	Overall	2/1432 (0.1%)	120604, 120621
Cough	1st year	3/1432 (0.2%)	140519, 200911, 242307
	2nd year	5/1166 (0.4%)	160572, 161532, 200901, 230607, 244934
	3rd year	6/ 963 (0.6%)	160625, 161532, 242906, 244105, 244407, 245933
	Overall	13/1432 (0.9%)	140519, 160572, 160625, 161532, 200901, 200911, 230607, 242307, 242906, 244105, 244407, 244934, 245933
Dyspnoea	2nd year	1/1166 (<0.1%)	160623
	Overall	1/1432 (<0.1%)	160623
Epiglottic cyst	1st year	1/1432 (<0.1%)	244707
	Overall	1/1432 (<0.1%)	244707
Epistaxis	1st year	2/1432 (0.1%)	140301, 190135
	2nd year	1/1166 (<0.1%)	160806
	Overall	3/1432 (0.2%)	140301, 160806, 190135
Hyperventilation	1st year	1/1432 (<0.1%)	160741
	Overall	1/1432 (<0.1%)	160741
Nasal congestion	1st year	1/1432 (<0.1%)	241807
	2nd year	1/1166 (<0.1%)	160631
	Overall	2/1432 (0.1%)	160631, 241807
Nasal disorder	3rd year	1/ 963 (0.1%)	200624
	Overall	1/1432 (<0.1%)	200624
Nasal oedema	1st year	1/1432 (<0.1%)	243969
	Overall	1/1432 (<0.1%)	243969

Table 14.3.1 / 10: Number of subjects with adverse events including subject identifier and year by treatment (FAS)

TREATMENT: LCS12

Primary system organ class<br>Preferred term<br>MedDRA version 14.0	YEAR OF ONSET	N (%)	subject identifier
Nasal polyps	1st year	1/1432 (<0.1%)	170606
	Overall	1/1432 (<0.1%)	170606
Oropharyngeal pain	1st year	7/1432 (0.5%)	160609, 200911, 230604, 242907, 244125, 244407, 245528
	2nd year	9/1166 (0.8%)	120632, 141403, 161426, 161440, 170702, 230604, 242812, 242907, 244426
	3rd year	3/ 963 (0.3%)	160623, 245431, 246212
	Overall	17/1432 (1.2%)	120632, 141403, 160609, 160623, 161426, 161440, 170702, 200911, 230604, 242812, 242907, 244125, 244407, 244426, 245431, 245528, 246212
Pleurisy	2nd year	1/1166 (<0.1%)	244430
	Overall	1/1432 (<0.1%)	244430
Pneumothorax	2nd year	1/1166 (<0.1%)	240510
	Overall	1/1432 (<0.1%)	240510
Pulmonary congestion	1st year	1/1432 (<0.1%)	241532
	Overall	1/1432 (<0.1%)	241532
Respiratory tract congestion	2nd year	1/1166 (<0.1%)	244310
	Overall	1/1432 (<0.1%)	244310
Respiratory tract inflammation	2nd year	1/1166 (<0.1%)	160985
	Overall	1/1432 (<0.1%)	160985
Rhinitis allergic	1st year	6/1432 (0.4%)	140116, 141032, 150101, 160505, 160519, 160573
	2nd year	3/1166 (0.3%)	160519, 240729, 244412
	3rd year	2/ 963 (0.2%)	200505, 210411
	Overall	10/1432 (0.7%)	140116, 141032, 150101, 160505, 160519, 160573, 200505, 210411, 240729, 244412
Rhinitis seasonal	1st year	1/1432 (<0.1%)	243969
	Overall	1/1432 (<0.1%)	243969
Rhinorrhoea	1st year	1/1432 (<0.1%)	240508
	Overall	1/1432 (<0.1%)	240508

Table 14.3.1 / 10: Number of subjects with adverse events including subject identifier and year by treatment (FAS)

TREATMENT: LCS12

Primary system organ class<br>Preferred term<br>MedDRA version 14.0	YEAR OF ONSET	N (%)	subject identifier
Sinus congestion	1st year	4/1432 (0.3%)	243972, 244934, 245701, 245910
	2nd year	1/1166 (<0.1%)	243702
	3rd year	1/ 963 (0.1%)	246212
	Overall	6/1432 (0.4%)	243702, 243972, 244934, 245701, 245910, 246212
Wheezing	1st year	1/1432 (<0.1%)	244438
	Overall	1/1432 (<0.1%)	244438
Skin and subcutaneous tissue disorders	1st year	190/1432 (13.3%)	
	2nd year	45/1166 (3.9%)	
	3rd year	25/ 963 (2.6%)	
	Overall	242/1432 (16.9%)	

Table 14.3.1 / 10: Number of subjects with adverse events including subject identifier and year by treatment (FAS)

TREATMENT: LCS12

Primary system organ class<br>Preferred term<br>MedDRA version 14.0	YEAR OF ONSET	N (%)	subject identifier
Acne	1st year	139/1432 (9.7%)	120201, 120208, 120228, 120329, 120401, 120412, 120414, 120633, 140114, 140118, 140204, 140206, 140301, 140502, 140518, 140519, 140529, 140802, 140813, 140904, 140914, 141022, 141029, 141106, 141109, 141409, 150111, 150136, 150151, 150153, 160106, 160217, 160220, 160305, 160310, 160311, 160323, 160416, 160418, 160434, 160505, 160509, 160615, 160617, 160624, 160625, 160705, 160722, 160725, 160726, 160734, 160742, 160746, 160754, 160758, 160935, 160959, 160965, 160982, 160985, 161130, 161203, 161215, 161301, 161312, 161322, 161411, 161414, 161440, 161516, 161533, 170304, 170704, 180612, 180645, 180655, 180739, 200109, 200208, 200216, 200227, 200408, 200501, 200521, 200527, 200914, 200919, 210130, 210205, 210404, 210606, 230221, 230310, 230406, 230603, 230824, 230903, 231002, 240217, 240223, 240345, 240352, 240508, 240811, 240836, 241028, 241153, 241175, 241231, 241405, 241512, 241553, 241902, 242215, 242316, 242320, 242510, 243003, 243320, 243323, 243820, 243929, 244203, 244407, 244441, 244448, 244511, 244518, 244707, 245432, 245502, 245506, 245508, 245528, 245709, 245903, 245916, 246116, 246144
	2nd year	20/1166 (1.7%)	140208, 141412, 160310, 160416, 160440, 160914, 161106, 161322, 161511, 180621, 180652, 200201, 200709, 242212, 243617, 243830, 243945, 244522, 244918, 245508
	3rd year	10/ 963 (1.0%)	141029, 160910, 161117, 161122, 161301, 170608, 200115, 243514, 245019, 245519
	Overall	163/1432 (11.4%)	120201, 120208, 120228, 120329, 120401, 120412, 120414, 120633, 140114, 140118, 140204, 140206, 140208, 140301, 140502, 140518, 140519, 140529, 140802, 140813, 140904, 140914, 141022, 141029, 141106,

Table 14.3.1 / 10: Number of subjects with adverse events including subject identifier and year by treatment (FAS)

TREATMENT: LCS12

Primary system organ class<br>Preferred term<br>MedDRA version 14.0	YEAR OF<br>ONSET	N (%)	subject identifier
			141109, 141409, 141412, 150111, 150136, 150151, 150153, 160106, 160217, 160220, 160305, 160310, 160311, 160323, 160416, 160418, 160434, 160440, 160505, 160509, 160615, 160617, 160624, 160625, 160705, 160722, 160725, 160726, 160734, 160742, 160746, 160754, 160758, 160910, 160914, 160935, 160959, 160965, 160982, 160985, 161106, 161117, 161122, 161130, 161203, 161215, 161301, 161312, 161322, 161411, 161414, 161440, 161511, 161516, 161533, 170304, 170608, 170704, 180612, 180621, 180645, 180652, 180655, 180739, 200109, 200115, 200201, 200208, 200216, 200227, 200408, 200501, 200521, 200527, 200709, 200914, 200919, 210130, 210205, 210404, 210606, 230221, 230310, 230406, 230603, 230824, 230903, 231002, 240217, 240223, 240345, 240352, 240508, 240811, 240836, 241028, 241153, 241175, 241231, 241405, 241512, 241553, 241902, 242212, 242215, 242316, 242320, 242510, 243003, 243320, 243323, 243514, 243617, 243820, 243830, 243929, 243945, 244203, 244407, 244441, 244448, 244511, 244518, 244522, 244707, 244918, 245019, 245432, 245502, 245506, 245508, 245519, 245528, 245709, 245903, 245916, 246116, 246144
Acne cystic	1st year	1/1432 (<0.1%)	240359
	2nd year	1/1166 (<0.1%)	245410
	Overall	2/1432 (0.1%)	240359, 245410
Alopecia	1st year	10/1432 (0.7%)	160509, 160606, 160954, 200904, 231103, 241507, 243969, 244203, 244518, 246138
	2nd year	6/1166 (0.5%)	120323, 120626, 141412, 241305, 244203, 244918
	Overall	15/1432 (1.0%)	120323, 120626, 141412, 160509, 160606, 160954, 200904, 231103, 241305, 241507, 243969, 244203, 244518, 244918, 246138

Table 14.3.1 / 10: Number of subjects with adverse events including subject identifier and year by treatment (FAS)

TREATMENT: LCS12

Primary system organ class<br>Preferred term<br>MedDRA version 14.0	YEAR OF ONSET	N (%)	subject identifier
Alopecia areata	2nd year	1/1166 (<0.1%)	150112
	3rd year	1/ 963 (0.1%)	241504
	Overall	2/1432 (0.1%)	150112, 241504
Chloasma	1st year	3/1432 (0.2%)	120414, 244448, 245928
	Overall	3/1432 (0.2%)	120414, 244448, 245928
Cold sweat	3rd year	1/ 963 (0.1%)	245807
	Overall	1/1432 (<0.1%)	245807
Dandruff	1st year	1/1432 (<0.1%)	160705
	Overall	1/1432 (<0.1%)	160705
Dermal cyst	1st year	2/1432 (0.1%)	241536, 244710
	Overall	2/1432 (0.1%)	241536, 244710
Dermatitis	1st year	4/1432 (0.3%)	120602, 140210, 160951, 210136
	2nd year	3/1166 (0.3%)	150167, 160960, 243015
	3rd year	1/ 963 (0.1%)	161411
	Overall	8/1432 (0.6%)	120602, 140210, 150167, 160951, 160960, 161411, 210136, 243015
Dermatitis allergic	1st year	2/1432 (0.1%)	140117, 161205
	2nd year	1/1166 (<0.1%)	141004
	3rd year	3/ 963 (0.3%)	141032, 161018, 200121
	Overall	6/1432 (0.4%)	140117, 141004, 141032, 161018, 161205, 200121
Dermatitis atopic	2nd year	2/1166 (0.2%)	150112, 161114
	Overall	2/1432 (0.1%)	150112, 161114
Dermatitis contact	1st year	1/1432 (<0.1%)	245808
	2nd year	1/1166 (<0.1%)	241305
	Overall	2/1432 (0.1%)	241305, 245808
Drug eruption	2nd year	1/1166 (<0.1%)	200910
	Overall	1/1432 (<0.1%)	200910
Dyshidrosis	1st year	1/1432 (<0.1%)	120632
	Overall	1/1432 (<0.1%)	120632

Table 14.3.1 / 10: Number of subjects with adverse events including subject identifier and year by treatment (FAS)

TREATMENT: LCS12

Primary system organ class<br>Preferred term<br>MedDRA version 14.0	YEAR OF ONSET	N (%)	subject identifier
Eczema	1st year	4/1432 (0.3%)	140519, 160323, 160576, 244414
	2nd year	2/1166 (0.2%)	140105, 141419
	3rd year	1/ 963 (0.1%)	140105
	Overall	6/1432 (0.4%)	140105, 140519, 141419, 160323, 160576, 244414
Erythema	1st year	1/1432 (<0.1%)	140802
	2nd year	1/1166 (<0.1%)	141101
	Overall	2/1432 (0.1%)	140802, 141101
Erythema multiforme	2nd year	1/1166 (<0.1%)	245431
	3rd year	1/ 963 (0.1%)	245431
	Overall	1/1432 (<0.1%)	245431
Hidradenitis	1st year	1/1432 (<0.1%)	230908
	Overall	1/1432 (<0.1%)	230908
Hirsutism	1st year	7/1432 (0.5%)	120414, 150111, 200614, 230221, 241302, 241512, 244412
	Overall	7/1432 (0.5%)	120414, 150111, 200614, 230221, 241302, 241512, 244412
Hyperhidrosis	1st year	3/1432 (0.2%)	161511, 200919, 241406
	Overall	3/1432 (0.2%)	161511, 200919, 241406
Hyperkeratosis	1st year	1/1432 (<0.1%)	200601
	Overall	1/1432 (<0.1%)	200601
Hypertrichosis	1st year	2/1432 (0.1%)	160935, 210205
	Overall	2/1432 (0.1%)	160935, 210205
Idiopathic urticaria	1st year	1/1432 (<0.1%)	241712
	Overall	1/1432 (<0.1%)	241712
Increased tendency to bruise	3rd year	1/ 963 (0.1%)	244919
	Overall	1/1432 (<0.1%)	244919
Ingrown hair	2nd year	1/1166 (<0.1%)	245808
	Overall	1/1432 (<0.1%)	245808

Table 14.3.1 / 10: Number of subjects with adverse events including subject identifier and year by treatment (FAS)

TREATMENT: LCS12

Primary system organ class<br>Preferred term<br>MedDRA version 14.0	YEAR OF ONSET	N (%)	subject identifier
Intertrigo	2nd year	1/1166 (<0.1%)	140817
	Overall	1/1432 (<0.1%)	140817
Lentigo	3rd year	1/ 963 (0.1%)	241504
	Overall	1/1432 (<0.1%)	241504
Lichen sclerosus	2nd year	1/1166 (<0.1%)	160962
	Overall	1/1432 (<0.1%)	160962
Pityriasis rosea	1st year	1/1432 (<0.1%)	246111
	Overall	1/1432 (<0.1%)	246111
Pruritus	1st year	3/1432 (0.2%)	140519, 241545, 244518
	3rd year	1/ 963 (0.1%)	120312
	Overall	4/1432 (0.3%)	120312, 140519, 241545, 244518
Rash	1st year	6/1432 (0.4%)	160415, 210120, 241416, 241507, 242907, 243204
	2nd year	2/1166 (0.2%)	241536, 241708
	3rd year	2/ 963 (0.2%)	140808, 160617
	Overall	10/1432 (0.7%)	140808, 160415, 160617, 210120, 241416, 241507, 241536, 241708, 242907, 243204
Rash papular	1st year	1/1432 (<0.1%)	243308
	2nd year	1/1166 (<0.1%)	244412
	Overall	2/1432 (0.1%)	243308, 244412
Rosacea	2nd year	1/1166 (<0.1%)	160952
	Overall	1/1432 (<0.1%)	160952
Seborrhoea	1st year	6/1432 (0.4%)	160301, 160509, 160574, 160806, 161403, 161406
	Overall	7/1432 (0.5%)	160301, 160509, 160574, 160806, 161111, 161403, 161406
Skin dystrophy	1st year	1/1432 (<0.1%)	200901
	Overall	1/1432 (<0.1%)	200901
Skin irritation	1st year	1/1432 (<0.1%)	160803
	Overall	1/1432 (<0.1%)	160803

Table 14.3.1 / 10: Number of subjects with adverse events including subject identifier and year by treatment (FAS)

TREATMENT: LCS12

Primary system organ class / Preferred term / MedDRA version 14.0	YEAR OF ONSET	N (%)	subject identifier
Skin reaction	1st year	2/1432 (0.1%)	241531, 241605
	Overall	2/1432 (0.1%)	241531, 241605
Urticaria	1st year	7/1432 (0.5%)	150123, 160415, 160924, 161301, 230112, 243948, 244927
	2nd year	2/1166 (0.2%)	161301, 245807
	3rd year	3/ 963 (0.3%)	140519, 160613, 242320
	Overall	11/1432 (0.8%)	140519, 150123, 160415, 160613, 160924, 161301, 230112, 242320, 243948, 244927, 245807
Social circumstances	1st year	1/1432 (<0.1%)	
	Overall	1/1432 (<0.1%)	
Exposure to communicable disease	1st year	1/1432 (<0.1%)	240503
	Overall	1/1432 (<0.1%)	240503
Surgical and medical procedures	1st year	17/1432 (1.2%)	
	2nd year	14/1166 (1.2%)	
	3rd year	10/ 963 (1.0%)	
	Overall	40/1432 (2.8%)	
Abdominoplasty	1st year	2/1432 (0.1%)	120604, 160436
	2nd year	1/1166 (<0.1%)	242307
	3rd year	1/ 963 (0.1%)	245503
	Overall	4/1432 (0.3%)	120604, 160436, 242307, 245503
Anal fissure excision	2nd year	1/1166 (<0.1%)	141004
	Overall	1/1432 (<0.1%)	141004
Breast prosthesis implantation	1st year	2/1432 (0.1%)	160436, 240348
	2nd year	1/1166 (<0.1%)	120626
	Overall	3/1432 (0.2%)	120626, 160436, 240348
Bunion operation	1st year	1/1432 (<0.1%)	210411
	2nd year	1/1166 (<0.1%)	160746
	Overall	2/1432 (0.1%)	160746, 210411
Cholecystectomy	2nd year	1/1166 (<0.1%)	243617
	Overall	1/1432 (<0.1%)	243617

Table 14.3.1 / 10: Number of subjects with adverse events including subject identifier and year by treatment (FAS)

TREATMENT: LCS12

Primary system organ class<br>Preferred term<br>MedDRA version 14.0	YEAR OF ONSET	N (%)	subject identifier
Dental cosmetic procedure	3rd year	1/ 963 (0.1%)	244430
	Overall	1/1432 (<0.1%)	244430
Dental operation	2nd year	1/1166 (<0.1%)	120312
	Overall	1/1432 (<0.1%)	120312
Detoxification	3rd year	1/ 963 (0.1%)	241708
	Overall	1/1432 (<0.1%)	241708
Gastric banding	1st year	1/1432 (<0.1%)	243502
	Overall	1/1432 (<0.1%)	243502
Ligament operation	3rd year	1/ 963 (0.1%)	230613
	Overall	1/1432 (<0.1%)	230613
Lipoma excision	2nd year	1/1166 (<0.1%)	140116
	Overall	1/1432 (<0.1%)	140116
Mammoplasty	1st year	2/1432 (0.1%)	230318, 230607
	2nd year	2/1166 (0.2%)	141026, 242315
	3rd year	3/ 963 (0.3%)	241302, 242315, 245503
	Overall	6/1432 (0.4%)	141026, 230318, 230607, 241302, 242315, 245503
Maxillary antrum operation	1st year	1/1432 (<0.1%)	200501
	Overall	1/1432 (<0.1%)	200501
Mole excision	1st year	2/1432 (0.1%)	200709, 210111
	Overall	2/1432 (0.1%)	200709, 210111
Postoperative analgesia	2nd year	1/1166 (<0.1%)	246220
	Overall	1/1432 (<0.1%)	246220
Tonsillectomy	2nd year	1/1166 (<0.1%)	241113
	Overall	1/1432 (<0.1%)	241113

Table 14.3.1 / 10: Number of subjects with adverse events including subject identifier and year by treatment (FAS)

TREATMENT: LCS12

Primary system organ class<br>Preferred term<br>MedDRA version 14.0	YEAR OF ONSET	N (%)	subject identifier
Tooth extraction	1st year	1/1432 (<0.1%)	150108
	2nd year	2/1166 (0.2%)	120623, 150167
	3rd year	3/ 963 (0.3%)	120613, 160981, 244408
	Overall	6/1432 (0.4%)	120613, 120623, 150108, 150167, 160981, 244408
Varicose vein operation	3rd year	1/ 963 (0.1%)	160928
	Overall	1/1432 (<0.1%)	160928
Wisdom teeth removal	1st year	6/1432 (0.4%)	150156, 160949, 161424, 240634, 240739, 243919
	2nd year	1/1166 (<0.1%)	243121
	Overall	7/1432 (0.5%)	150156, 160949, 161424, 240634, 240739, 243121, 243919
Vascular disorders	1st year	14/1432 (1.0%)	
	2nd year	4/1166 (0.3%)	
	3rd year	4/ 963 (0.4%)	
	Overall	20/1432 (1.4%)	
Deep vein thrombosis	1st year	1/1432 (<0.1%)	140807
	Overall	1/1432 (<0.1%)	140807
Hot flush	1st year	4/1432 (0.3%)	140802, 240217, 241168, 244618
	Overall	4/1432 (0.3%)	140802, 240217, 241168, 244618
Hypertension	1st year	6/1432 (0.4%)	160331, 180707, 200705, 242320, 243302, 244523
	2nd year	3/1166 (0.3%)	160509, 180669, 200912
	3rd year	3/ 963 (0.3%)	160567, 180420, 242320
	Overall	11/1432 (0.8%)	160331, 160509, 160567, 180420, 180669, 180707, 200705, 200912, 242320, 243302, 244523
Hypotension	1st year	1/1432 (<0.1%)	120208
	Overall	1/1432 (<0.1%)	120208
Neurogenic shock	1st year	2/1432 (0.1%)	140105, 140114
	Overall	2/1432 (0.1%)	140105, 140114
Thrombophlebitis superficial	2nd year	1/1166 (<0.1%)	140807
	Overall	1/1432 (<0.1%)	140807

Table 14.3.1 / 10: Number of subjects with adverse events including subject identifier and year by treatment (FAS)

TREATMENT: LCS12

Primary system organ class<br>Preferred term<br>MedDRA version 14.0	YEAR OF ONSET	N (%)	subject identifier
Varicose vein	3rd year	1/ 963 (0.1%)	120401
	Overall	1/1432 (<0.1%)	120401

Note: A subject is counted only once within each preferred term of any primary SOC.
Note: Denominator is number of subjects available at the START of the year or all subjects for overall
Note: Adverse events are sorted in alphabetical order by primary SOC and preferred term.
Global Biostatistics: /by-sasp/patdb/projects/de04209/310442/stat/prod_interim03/pgms/t-ae-cat.sas epkll 12OCT2011 11:21

Table 14.3.1 / 10: Number of subjects with adverse events including subject identifier and year by treatment (FAS) (cont.)

TREATMENT: LCS16

Primary system organ class<br>Preferred term<br>MedDRA version 14.0	YEAR OF ONSET	N (%)	subject identifier
Number of subjects (%) with at least one adverse event	1st year	1068/1452 (73.6%)	
	2nd year	670/1206 (55.6%)	
	3rd year	535/1012 (52.9%)	
	Overall	1246/1452 (85.8%)	
Blood and lymphatic system disorders	1st year	3/1452 (0.2%)	
	2nd year	2/1206 (0.2%)	
	3rd year	3/1012 (0.3%)	
	Overall	8/1452 (0.6%)	
Anaemia	1st year	1/1452 (<0.1%)	161019
	2nd year	1/1206 (<0.1%)	120603
	3rd year	1/1012 (<0.1%)	241229
	Overall	3/1452 (0.2%)	120603, 161019, 241229
Iron deficiency anaemia	1st year	1/1452 (<0.1%)	140812
	Overall	1/1452 (<0.1%)	140812
Lymphadenopathy	1st year	1/1452 (<0.1%)	245922
	3rd year	2/1012 (0.2%)	160552, 200930
	Overall	3/1452 (0.2%)	160552, 200930, 245922
Pancytopenia	2nd year	1/1206 (<0.1%)	242704
	Overall	1/1452 (<0.1%)	242704
Cardiac disorders	1st year	8/1452 (0.6%)	
	2nd year	4/1206 (0.3%)	
	3rd year	3/1012 (0.3%)	
	Overall	14/1452 (1.0%)	
Arrhythmia	1st year	2/1452 (0.1%)	160115, 161405
	Overall	2/1452 (0.1%)	160115, 161405
Extrasystoles	1st year	1/1452 (<0.1%)	160942
	3rd year	1/1012 (<0.1%)	160950
	Overall	2/1452 (0.1%)	160942, 160950

Table 14.3.1 / 10: Number of subjects with adverse events including subject identifier and year by treatment (FAS) (cont.)

TREATMENT: LCS16

Primary system organ class<br>Preferred term<br>MedDRA version 14.0	YEAR OF ONSET	N (%)	subject identifier
Mitral valve prolapse	1st year	1/1452 (<0.1%)	180629
	2nd year	1/1206 (<0.1%)	180660
	Overall	2/1452 (0.1%)	180629, 180660
Palpitations	1st year	2/1452 (0.1%)	180660, 241555
	2nd year	3/1206 (0.2%)	200510, 242912, 244704
	3rd year	1/1012 (<0.1%)	161432
	Overall	6/1452 (0.4%)	161432, 180660, 200510, 241555, 242912, 244704
Sinus tachycardia	1st year	1/1452 (<0.1%)	180629
	Overall	1/1452 (<0.1%)	180629
Tachycardia	1st year	2/1452 (0.1%)	120614, 160802
	Overall	2/1452 (0.1%)	120614, 160802
Ventricular extrasystoles	3rd year	1/1012 (<0.1%)	240734
	Overall	1/1452 (<0.1%)	240734
Congenital, familial and genetic disorders	1st year	1/1452 (<0.1%)	
	Overall	1/1452 (<0.1%)	
Myotonia congenita	1st year	1/1452 (<0.1%)	244435
	Overall	1/1452 (<0.1%)	244435
Ear and labyrinth disorders	1st year	5/1452 (0.3%)	
	2nd year	3/1206 (0.2%)	
	3rd year	7/1012 (0.7%)	
	Overall	14/1452 (1.0%)	
Ear pain	1st year	1/1452 (<0.1%)	140812
	3rd year	2/1012 (0.2%)	160636, 161524
	Overall	3/1452 (0.2%)	140812, 160636, 161524
Meniere's disease	3rd year	1/1012 (<0.1%)	160543
	Overall	1/1452 (<0.1%)	160543

Table 14.3.1 / 10: Number of subjects with adverse events including subject identifier and year by treatment (FAS) (cont.)

TREATMENT: LCS16

Primary system organ class<br>Preferred term<br>MedDRA version 14.0	YEAR OF ONSET	N (%)	subject identifier
Middle ear effusion	1st year	1/1452 (<0.1%)	244433
	2nd year	1/1206 (<0.1%)	244605
	Overall	2/1452 (0.1%)	244433, 244605
Motion sickness	1st year	1/1452 (<0.1%)	244514
	3rd year	2/1012 (0.2%)	161107, 161524
	Overall	3/1452 (0.2%)	161107, 161524, 244514
Otorrhoea	2nd year	1/1206 (<0.1%)	244429
	Overall	1/1452 (<0.1%)	244429
Vertigo	1st year	2/1452 (0.1%)	180625, 180641
	2nd year	1/1206 (<0.1%)	243331
	3rd year	3/1012 (0.3%)	161123, 190214, 244433
	Overall	6/1452 (0.4%)	161123, 180625, 180641, 190214, 243331, 244433
Endocrine disorders	1st year	14/1452 (1.0%)	
	2nd year	1/1206 (<0.1%)	
	3rd year	6/1012 (0.6%)	
	Overall	21/1452 (1.4%)	
Goitre	1st year	3/1452 (0.2%)	160629, 241402, 243937
	2nd year	1/1206 (<0.1%)	160543
	3rd year	1/1012 (<0.1%)	160562
	Overall	5/1452 (0.3%)	160543, 160562, 160629, 241402, 243937
Hyperthyroidism	1st year	1/1452 (<0.1%)	180602
	3rd year	1/1012 (<0.1%)	240631
	Overall	2/1452 (0.1%)	180602, 240631
Hypothyroidism	1st year	9/1452 (0.6%)	120122, 120426, 120615, 161507, 230207, 241155, 241543, 243963, 244923
	3rd year	4/1012 (0.4%)	120405, 150137, 230516, 241403
	Overall	13/1452 (0.9%)	120122, 120405, 120426, 120615, 150137, 161507, 230207, 230516, 241155, 241403, 241543, 243963, 244923

Table 14.3.1 / 10: Number of subjects with adverse events including subject identifier and year by treatment (FAS) (cont.)

TREATMENT: LCS16

Primary system organ class<br>Preferred term<br>MedDRA version 14.0	YEAR OF ONSET	N (%)	subject identifier
Thyroid mass	1st year	1/1452 (<0.1%)	160502
	Overall	1/1452 (<0.1%)	160502
Eye disorders	1st year	11/1452 (0.8%)	
	2nd year	5/1206 (0.4%)	
	3rd year	7/1012 (0.7%)	
	Overall	23/1452 (1.6%)	
Blepharitis	1st year	2/1452 (0.1%)	140803, 245922
	Overall	2/1452 (0.1%)	140803, 245922
Chalazion	1st year	1/1452 (<0.1%)	140803
	Overall	1/1452 (<0.1%)	140803
Conjunctivitis	1st year	6/1452 (0.4%)	160123, 160508, 160808, 161404, 210408, 245922
	2nd year	4/1206 (0.3%)	161115, 161310, 242208, 242918
	3rd year	5/1012 (0.5%)	160314, 160428, 161123, 161311, 161315
	Overall	15/1452 (1.0%)	160123, 160314, 160428, 160508, 160808, 161115, 161123, 161310, 161311, 161315, 161404, 210408, 242208, 242918, 245922
Conjunctivitis allergic	3rd year	2/1012 (0.2%)	160744, 161131
	Overall	2/1452 (0.1%)	160744, 161131
Eye inflammation	2nd year	1/1206 (<0.1%)	161310
	Overall	1/1452 (<0.1%)	161310
Eyelid cyst	1st year	1/1452 (<0.1%)	245513
	Overall	1/1452 (<0.1%)	245513
Iritis	1st year	1/1452 (<0.1%)	160715
	2nd year	1/1206 (<0.1%)	242503
	Overall	2/1452 (0.1%)	160715, 242503
Myopia	1st year	1/1452 (<0.1%)	160521
	Overall	1/1452 (<0.1%)	160521

Table 14.3.1 / 10: Number of subjects with adverse events including subject identifier and year by treatment (FAS) (cont.)

TREATMENT: LCS16

Primary system organ class<br>Preferred term<br>MedDRA version 14.0	YEAR OF ONSET	N (%)	subject identifier
Ocular icterus	1st year	1/1452 (<0.1%)	200707
	Overall	1/1452 (<0.1%)	200707
Gastrointestinal disorders	1st year	218/1452 (15.0%)	
	2nd year	93/1206 (7.7%)	
	3rd year	63/1012 (6.2%)	
	Overall	320/1452 (22.0%)	
Abdominal discomfort	1st year	3/1452 (0.2%)	160906, 161529, 200922
	2nd year	3/1206 (0.2%)	244424, 244516, 245526
	Overall	6/1452 (0.4%)	160906, 161529, 200922, 244424, 244516, 245526
Abdominal distension	1st year	10/1452 (0.7%)	140120, 141411, 160622, 161119, 240807, 241522, 243402, 243823, 243905, 243964
	2nd year	2/1206 (0.2%)	243402, 245526
	Overall	11/1452 (0.8%)	140120, 141411, 160622, 161119, 240807, 241522, 243402, 243823, 243905, 243964, 245526

Table 14.3.1 / 10: Number of subjects with adverse events including subject identifier and year by treatment (FAS) (cont.)

TREATMENT: LCS16

Primary system organ class<br>Preferred term<br>MedDRA version 14.0	YEAR OF ONSET	N (%)	subject identifier
Abdominal pain	1st year	60/1452 (4.1%)	120402, 120424, 120425, 120606, 120609, 120617, 120631, 140106, 140110, 140120, 140810, 150161, 160107, 160214, 160441, 160501, 160620, 160628, 160706, 160707, 160737, 160744, 161502, 161505, 161520,<br>161524, 161526, 161528, 170502, 180713, 190419, 200226, 200302, 200514, 200906, 200917, 200926, 230102, 230309, 230506, 230610, 230612, 230614, 240621, 240626, 240722, 240734, 240737, 241418, 241422,<br>241430, 241510, 243034, 243112, 243217, 243509, 244519, 244525, 244709, 245002
	2nd year	29/1206 (2.4%)	120309, 120410, 120609, 140119, 140601, 140810, 141415, 160529, 160602, 180112, 180135, 190214, 200401, 200522, 200609, 200620, 240709, 240722, 241403, 241509, 241709, 241719, 241731, 243501, 243960,<br>244709, 244716, 245526, 245908
	3rd year	21/1012 (2.1%)	120229, 120325, 120402, 120609, 140812, 160128, 160221, 160529, 160533, 180117, 180124, 180132, 190134, 200930, 230506, 230516, 240114, 240626, 241546, 243707, 245522
	Overall	101/1452 (7.0%)	120229, 120309, 120325, 120402, 120410, 120424, 120425, 120606, 120609, 120617, 120631, 140106, 140110, 140119, 140120, 140601, 140810, 140812, 141415, 150161, 160107, 160128, 160214, 160221, 160441,<br>160501, 160529, 160533, 160602, 160620, 160628, 160706, 160707, 160737, 160744, 161502, 161505, 161520, 161524, 161526, 161528, 170502, 180112, 180117, 180124, 180132, 180135, 180713, 190134, 190214,<br>190419, 200226, 200302, 200401, 200514, 200522, 200609, 200620, 200906, 200917, 200926, 200930, 230102, 230309, 230506, 230516, 230610, 230612, 230614, 240114, 240621, 240626, 240709, 240722, 240734,<br>240737, 241403, 241418, 241422, 241430, 241509, 241510, 241546, 241709, 241719, 241731, 243034, 243112, 243217, 243501, 243509, 243707, 243960, 244519, 244525, 244709, 244716, 245002, 245522, 245526,

Table 14.3.1 / 10: Number of subjects with adverse events including subject identifier and year by treatment (FAS) (cont.)

TREATMENT: LCS16

Primary system organ class<br>Preferred term<br>MedDRA version 14.0	YEAR OF ONSET	N (%)	subject identifier
			245908
Abdominal pain lower	1st year	43/1452 (3.0%)	120420, 141035, 150121, 160116, 160211, 160314, 160510, 160543, 160545, 160552, 160608, 160622, 160630, 160635, 160636, 160641, 160642, 160912, 160975, 161107, 161108, 161222, 161311, 161315, 161503, 161524, 180602, 180674, 190127, 200410, 200707, 200806, 200907, 230319, 230807, 240703, 240730, 240907, 241403, 242904, 243321, 245031, 245922
	2nd year	16/1206 (1.3%)	160552, 160608, 160621, 160630, 160636, 160978, 161524, 200410, 240708, 240722, 241008, 241519, 242908, 243313, 243316, 244435
	3rd year	9/1012 (0.9%)	160929, 160978, 161005, 190134, 190142, 240632, 240711, 241724, 243937
	Overall	61/1452 (4.2%)	120420, 141035, 150121, 160116, 160211, 160314, 160510, 160543, 160545, 160552, 160608, 160621, 160622, 160630, 160635, 160636, 160641, 160642, 160912, 160929, 160975, 160978, 161005, 161107, 161108, 161222, 161311, 161315, 161503, 161524, 180602, 180674, 190127, 190134, 190142, 200410, 200707, 200806, 200907, 230319, 230807, 240632, 240703, 240708, 240711, 240722, 240730, 240907, 241008, 241403, 241519, 241724, 242904, 242908, 243313, 243316, 243321, 243937, 244435, 245031, 245922
Abdominal pain upper	1st year	7/1452 (0.5%)	140106, 160748, 160966, 161405, 161506, 180629, 230823
	2nd year	2/1206 (0.2%)	161524, 245908
	3rd year	2/1012 (0.2%)	140119, 244902
	Overall	11/1452 (0.8%)	140106, 140119, 160748, 160966, 161405, 161506, 161524, 180629, 230823, 244902, 245908
Anal fissure	1st year	1/1452 (<0.1%)	150126
	2nd year	1/1206 (<0.1%)	241420
	Overall	2/1452 (0.1%)	150126, 241420

Table 14.3.1 / 10: Number of subjects with adverse events including subject identifier and year by treatment (FAS) (cont.)

TREATMENT: LCS16

Primary system organ class<br>Preferred term<br>MedDRA version 14.0	YEAR OF ONSET	N (%)	subject identifier
Anal pruritus	2nd year	1/1206 (<0.1%)	241420
	3rd year	1/1012 (<0.1%)	241117
	Overall	2/1452 (0.1%)	241117, 241420
Anal skin tags	2nd year	1/1206 (<0.1%)	241420
	Overall	1/1452 (<0.1%)	241420
Aphthous stomatitis	1st year	1/1452 (<0.1%)	242312
	Overall	1/1452 (<0.1%)	242312
Colitis	1st year	2/1452 (0.1%)	190206, 190207
	2nd year	1/1206 (<0.1%)	190214
	Overall	3/1452 (0.2%)	190206, 190207, 190214
Constipation	1st year	9/1452 (0.6%)	160123, 161131, 200928, 240645, 240807, 243956, 243977, 245922, 246103
	2nd year	6/1206 (0.5%)	141035, 200907, 200922, 241139, 243316, 244424
	3rd year	3/1012 (0.3%)	140805, 242321, 243964
	Overall	18/1452 (1.2%)	140805, 141035, 160123, 161131, 200907, 200922, 200928, 240645, 240807, 241139, 242321, 243316, 243956, 243964, 243977, 244424, 245922, 246103
Dental caries	1st year	1/1452 (<0.1%)	244916
	3rd year	2/1012 (0.2%)	242317, 242321
	Overall	3/1452 (0.2%)	242317, 242321, 244916
Diarrhoea	1st year	16/1452 (1.1%)	120606, 120609, 120612, 140810, 141025, 150147, 160709, 161016, 161321, 161419, 200514, 200917, 210110, 230811, 230815, 244606
	2nd year	7/1206 (0.6%)	140106, 161025, 161526, 230305, 242813, 242918, 243976
	3rd year	5/1012 (0.5%)	161115, 241546, 242918, 243934, 244424
	Overall	27/1452 (1.9%)	120606, 120609, 120612, 140106, 140810, 141025, 150147, 160709, 161016, 161025, 161115, 161321, 161419, 161526, 200514, 200917, 210110, 230305, 230811, 230815, 241546, 242813, 242918, 243934, 243976, 244424, 244606

Table 14.3.1 / 10: Number of subjects with adverse events including subject identifier and year by treatment (FAS) (cont.)

TREATMENT: LCS16

Primary system organ class<br>Preferred term<br>MedDRA version 14.0	YEAR OF ONSET	N (%)	subject identifier
Dry mouth	3rd year	1/1012 (<0.1%)	245806
	Overall	1/1452 (<0.1%)	245806
Dyspepsia	1st year	7/1452 (0.5%)	120609, 141415, 160553, 161306, 210604, 241430, 242904
	2nd year	3/1206 (0.2%)	141016, 160611, 161526
	3rd year	2/1012 (0.2%)	160603, 243707
	Overall	12/1452 (0.8%)	120609, 141016, 141415, 160553, 160603, 160611, 161306, 161526, 210604, 241430, 242904, 243707
Dysphagia	2nd year	1/1206 (<0.1%)	244801
	Overall	1/1452 (<0.1%)	244801
Flatulence	1st year	2/1452 (0.1%)	160748, 243964
	Overall	2/1452 (0.1%)	160748, 243964
Food poisoning	1st year	3/1452 (0.2%)	140805, 200529, 242317
	2nd year	4/1206 (0.3%)	161025, 161108, 200529, 200907
	Overall	6/1452 (0.4%)	140805, 161025, 161108, 200529, 200907, 242317
Gastric ulcer	1st year	1/1452 (<0.1%)	245707
	Overall	1/1452 (<0.1%)	245707
Gastritis	1st year	5/1452 (0.3%)	120609, 161401, 161417, 190113, 230915
	2nd year	8/1206 (0.7%)	120606, 150122, 150137, 160426, 160737, 190214, 230717, 240517
	3rd year	3/1012 (0.3%)	120607, 120612, 241197
	Overall	16/1452 (1.1%)	120606, 120607, 120609, 120612, 150122, 150137, 160426, 160737, 161401, 161417, 190113, 190214, 230717, 230915, 240517, 241197
Gastrointestinal pain	1st year	1/1452 (<0.1%)	242811
	Overall	1/1452 (<0.1%)	242811

Table 14.3.1 / 10: Number of subjects with adverse events including subject identifier and year by treatment (FAS) (cont.)

TREATMENT: LCS16

Primary system organ class<br>Preferred term<br>MedDRA version 14.0	YEAR OF ONSET	N (%)	subject identifier
Gastrooesophageal reflux disease	1st year	6/1452 (0.4%)	161019, 190134, 241141, 241236, 241403, 244113
	2nd year	3/1206 (0.2%)	141411, 240220, 243954
	3rd year	2/1012 (0.2%)	161004, 241546
	Overall	11/1452 (0.8%)	141411, 161004, 161019, 190134, 240220, 241141, 241236, 241403, 241546, 243954, 244113
Gingivitis	2nd year	1/1206 (<0.1%)	180708
	Overall	1/1452 (<0.1%)	180708
Haematochezia	2nd year	1/1206 (<0.1%)	241420
	Overall	1/1452 (<0.1%)	241420
Haemorrhoids	1st year	5/1452 (0.3%)	160438, 160642, 161321, 240802, 241031
	2nd year	3/1206 (0.2%)	161004, 230601, 241420
	Overall	8/1452 (0.6%)	160438, 160642, 161004, 161321, 230601, 240802, 241031, 241420
Hiatus hernia	2nd year	1/1206 (<0.1%)	245434
	Overall	1/1452 (<0.1%)	245434
Hyperchlorhydria	1st year	1/1452 (<0.1%)	120612
	Overall	1/1452 (<0.1%)	120612
Inflammatory bowel disease	3rd year	1/1012 (<0.1%)	190603
	Overall	1/1452 (<0.1%)	190603
Irritable bowel syndrome	1st year	2/1452 (0.1%)	150126, 150137
	2nd year	1/1206 (<0.1%)	150137
	3rd year	3/1012 (0.3%)	150140, 161306, 200704
	Overall	5/1452 (0.3%)	150126, 150137, 150140, 161306, 200704
Mouth ulceration	1st year	2/1452 (0.1%)	120404, 244916
	Overall	2/1452 (0.1%)	120404, 244916

Table 14.3.1 / 10: Number of subjects with adverse events including subject identifier and year by treatment (FAS) (cont.)

TREATMENT: LCS16

Primary system organ class<br>Preferred term<br>MedDRA version 14.0	YEAR OF ONSET	N (%)	subject identifier
Nausea	1st year	41/1452 (2.8%)	120313, 120336, 140810, 141415, 160423, 160634, 160966, 161019, 170601, 180310, 200508, 200926, 210410, 230111, 240626, 240803, 241030, 241103, 241116, 241118, 241176, 241511, 241555, 242104, 243109, 243209, 243303, 243703, 243707, 243714, 243909, 243977, 244309, 244514, 244516, 244619, 244703, 244706, 244715, 246127, 246207
	2nd year	8/1206 (0.7%)	140810, 160543, 240708, 240722, 240802, 241726, 243313, 245908
	3rd year	12/1012 (1.2%)	120404, 240618, 240626, 241401, 241412, 241522, 241546, 242104, 242321, 242918, 244424, 245522
	Overall	58/1452 (4.0%)	120313, 120336, 120404, 140810, 141415, 160423, 160543, 160634, 160966, 161019, 170601, 180310, 200508, 200926, 210410, 230111, 240618, 240626, 240708, 240722, 240802, 240803, 241030, 241103, 241116, 241118, 241176, 241401, 241412, 241511, 241522, 241546, 241555, 241726, 242104, 242321, 242918, 243109, 243209, 243303, 243313, 243703, 243707, 243714, 243909, 243977, 244309, 244424, 244514, 244516, 244619, 244703, 244706, 244715, 245522, 245908, 246127, 246207
Odynophagia	2nd year	1/1206 (<0.1%)	244801
	Overall	1/1452 (<0.1%)	244801
Oesophageal stenosis	2nd year	1/1206 (<0.1%)	140804
	Overall	1/1452 (<0.1%)	140804
Oral pain	1st year	1/1452 (<0.1%)	243205
	Overall	1/1452 (<0.1%)	243205
Periodontitis	3rd year	1/1012 (<0.1%)	161534
	Overall	1/1452 (<0.1%)	161534
Proctalgia	2nd year	1/1206 (<0.1%)	241420
	Overall	1/1452 (<0.1%)	241420

Table 14.3.1 / 10: Number of subjects with adverse events including subject identifier and year by treatment (FAS) (cont.)

TREATMENT: LCS16

Primary system organ class Preferred term MedDRA version 14.0	YEAR OF ONSET	N (%)	subject identifier
Rectal haemorrhage	1st year	2/1452 (0.1%)	244709, 245905
	2nd year	1/1206 (<0.1%)	241420
	Overall	3/1452 (0.2%)	241420, 244709, 245905
Reflux oesophagitis	1st year	1/1452 (<0.1%)	161123
	2nd year	1/1206 (<0.1%)	161123
	Overall	1/1452 (<0.1%)	161123
Salivary gland calculus	1st year	1/1452 (<0.1%)	243975
	Overall	1/1452 (<0.1%)	243975
Tooth disorder	2nd year	1/1206 (<0.1%)	160107
	Overall	1/1452 (<0.1%)	160107
Toothache	1st year	15/1452 (1.0%)	120101, 120102, 120606, 120615, 120627, 160115, 160314, 160632, 180678, 210123, 241194, 241510, 243966, 244519, 244916
	2nd year	9/1206 (0.7%)	120402, 120606, 150103, 160115, 160703, 180730, 241301, 241801, 244519
	3rd year	5/1012 (0.5%)	120603, 160602, 160907, 161211, 244435
	Overall	26/1452 (1.8%)	120101, 120102, 120402, 120603, 120606, 120615, 120627, 150103, 160115, 160314, 160602, 160632, 160703, 160907, 161211, 180678, 180730, 210123, 241194, 241301, 241510, 241801, 243966, 244435, 244519, 244916
Umbilical hernia	3rd year	1/1012 (<0.1%)	120404
	Overall	1/1452 (<0.1%)	120404
Vomiting	1st year	13/1452 (0.9%)	140810, 160546, 161315, 230203, 230220, 240626, 240706, 243303, 243406, 244619, 244715, 246127, 246207
	2nd year	6/1206 (0.5%)	141415, 230305, 240708, 240722, 241731, 245507
	3rd year	5/1012 (0.5%)	242918, 243223, 243707, 243934, 245522
	Overall	24/1452 (1.7%)	140810, 141415, 160546, 161315, 230203, 230220, 230305, 240626, 240706, 240708, 240722, 241731, 242918, 243223, 243303, 243406, 243707, 243934, 244619, 244715, 245507, 245522, 246127, 246207

Table 14.3.1 / 10: Number of subjects with adverse events including subject identifier and year by treatment (FAS) (cont.)

TREATMENT: LCS16

Primary system organ class / Preferred term / MedDRA version 14.0	YEAR OF ONSET	N (%)	subject identifier
General disorders and administration site conditions	1st year	72/1452 (5.0%)	
	2nd year	30/1206 (2.5%)	
	3rd year	20/1012 (2.0%)	
	Overall	119/1452 (8.2%)	
Asthenia	1st year	1/1452 (<0.1%)	120410
	Overall	1/1452 (<0.1%)	120410
Axillary pain	2nd year	1/1206 (<0.1%)	243205
	Overall	1/1452 (<0.1%)	243205
Chest pain	1st year	2/1452 (0.1%)	241236, 242811
	2nd year	1/1206 (<0.1%)	120311
	3rd year	4/1012 (0.4%)	140112, 160919, 241412, 244805
	Overall	7/1452 (0.5%)	120311, 140112, 160919, 241236, 241412, 242811, 244805
Chills	1st year	2/1452 (0.1%)	140508, 245511
	Overall	2/1452 (0.1%)	140508, 245511
Cyst	3rd year	1/1012 (<0.1%)	245930
	Overall	1/1452 (<0.1%)	245930
Device dislocation	1st year	3/1452 (0.2%)	243108, 244905, 245404
	2nd year	1/1206 (<0.1%)	160978
	3rd year	1/1012 (<0.1%)	243918
	Overall	5/1452 (0.3%)	160978, 243108, 243918, 244905, 245404

Table 14.3.1 / 10: Number of subjects with adverse events including subject identifier and year by treatment (FAS) (cont.)

TREATMENT: LCS16

Primary system organ class<br>Preferred term<br>MedDRA version 14.0	YEAR OF ONSET	N (%)	subject identifier
Device expulsion	1st year	23/1452 (1.6%)	120319, 160318, 160621, 170103, 180219, 180634, 180653, 180656, 180679, 180682, 190627, 240354, 240822, 240901, 241195, 241540, 241732, 242507, 244114, 244309, 244715, 244933, 245406
	2nd year	7/1206 (0.6%)	180121, 180134, 180226, 240133, 243034, 243208, 243936
	3rd year	7/1012 (0.7%)	120318, 170303, 243101, 243703, 243954, 245408, 246120
	Overall	37/1452 (2.5%)	120318, 120319, 160318, 160621, 170103, 170303, 180121, 180134, 180219, 180226, 180634, 180653, 180656, 180679, 180682, 190627, 240133, 240354, 240822, 240901, 241195, 241540, 241732, 242507, 243034, 243101, 243208, 243703, 243936, 243954, 244114, 244309, 244715, 244933, 245406, 245408, 246120
Discomfort	2nd year	1/1206 (<0.1%)	242704
	Overall	1/1452 (<0.1%)	242704
Fatigue	1st year	19/1452 (1.3%)	140201, 140805, 140810, 140812, 140818, 160561, 160966, 180664, 200508, 200906, 230211, 230514, 230516, 240330, 241030, 241418, 241420, 244516, 245035
	2nd year	6/1206 (0.5%)	141007, 180641, 230309, 243215, 243819, 245905
	3rd year	2/1012 (0.2%)	241403, 245522
	Overall	27/1452 (1.9%)	140201, 140805, 140810, 140812, 140818, 141007, 160561, 160966, 180641, 180664, 200508, 200906, 230211, 230309, 230514, 230516, 240330, 241030, 241403, 241418, 241420, 243215, 243819, 244516, 245035, 245522, 245905
Feeling cold	1st year	1/1452 (<0.1%)	242915
	2nd year	1/1206 (<0.1%)	240722
	Overall	2/1452 (0.1%)	240722, 242915
Feeling hot	1st year	1/1452 (<0.1%)	230505
	Overall	1/1452 (<0.1%)	230505

Table 14.3.1 / 10: Number of subjects with adverse events including subject identifier and year by treatment (FAS) (cont.)

TREATMENT: LCS16

Primary system organ class<br>Preferred term<br>MedDRA version 14.0	YEAR OF ONSET	N (%)	subject identifier
Hunger	2nd year	1/1206 (<0.1%)	243402
	Overall	1/1452 (<0.1%)	243402
Inflammation	3rd year	1/1012 (<0.1%)	160755
	Overall	1/1452 (<0.1%)	160755
Influenza like illness	2nd year	1/1206 (<0.1%)	242501
	Overall	1/1452 (<0.1%)	242501
Injury associated with device	1st year	1/1452 (<0.1%)	231110
	Overall	1/1452 (<0.1%)	231110
Irritability	1st year	4/1452 (0.3%)	140112, 140818, 160513, 230610
	2nd year	1/1206 (<0.1%)	141411
	3rd year	1/1012 (<0.1%)	240840
	Overall	6/1452 (0.4%)	140112, 140818, 141411, 160513, 230610, 240840
Localised oedema	1st year	1/1452 (<0.1%)	161524
	2nd year	1/1206 (<0.1%)	161524
	Overall	1/1452 (<0.1%)	161524
Malaise	1st year	1/1452 (<0.1%)	190214
	Overall	1/1452 (<0.1%)	190214
Oedema	1st year	1/1452 (<0.1%)	160802
	Overall	1/1452 (<0.1%)	160802
Oedema peripheral	1st year	4/1452 (0.3%)	160101, 241901, 242702, 243407
	2nd year	1/1206 (<0.1%)	160219
	3rd year	1/1012 (<0.1%)	200704
	Overall	6/1452 (0.4%)	160101, 160219, 200704, 241901, 242702, 243407
Pain	1st year	3/1452 (0.2%)	120410, 241535, 245511
	2nd year	1/1206 (<0.1%)	244424
	3rd year	1/1012 (<0.1%)	170303
	Overall	5/1452 (0.3%)	120410, 170303, 241535, 244424, 245511

Table 14.3.1 / 10: Number of subjects with adverse events including subject identifier and year by treatment (FAS) (cont.)

TREATMENT: LCS16

Primary system organ class<br>Preferred term<br>MedDRA version 14.0	YEAR OF ONSET	N (%)	subject identifier
Pyrexia	1st year	10/1452 (0.7%)	140225, 160221, 161526, 161535, 210405, 230220, 230222, 241107, 241418, 245511
	2nd year	5/1206 (0.4%)	160126, 160629, 200707, 210123, 230811
	3rd year	2/1012 (0.2%)	210103, 210123
	Overall	16/1452 (1.1%)	140225, 160126, 160221, 160629, 161526, 161535, 200707, 210103, 210123, 210405, 230220, 230222, 230811, 241107, 241418, 245511
Vaccination site pain	2nd year	1/1206 (<0.1%)	230704
	Overall	1/1452 (<0.1%)	230704
Hepatobiliary disorders	1st year	6/1452 (0.4%)	
	2nd year	3/1206 (0.2%)	
	3rd year	2/1012 (0.2%)	
	Overall	11/1452 (0.8%)	
Biliary colic	1st year	2/1452 (0.1%)	120425, 243303
	Overall	2/1452 (0.1%)	120425, 243303
Biliary dyskinesia	2nd year	1/1206 (<0.1%)	243020
	Overall	1/1452 (<0.1%)	243020
Cholecystitis	2nd year	1/1206 (<0.1%)	243011
	3rd year	1/1012 (<0.1%)	243707
	Overall	2/1452 (0.1%)	243011, 243707
Cholelithiasis	1st year	1/1452 (<0.1%)	245707
	2nd year	1/1206 (<0.1%)	241726
	3rd year	2/1012 (0.2%)	160221, 243707
	Overall	4/1452 (0.3%)	160221, 241726, 243707, 245707
Gallbladder pain	1st year	1/1452 (<0.1%)	241901
	Overall	1/1452 (<0.1%)	241901
Hepatic steatosis	1st year	1/1452 (<0.1%)	245517
	Overall	1/1452 (<0.1%)	245517

Table 14.3.1 / 10: Number of subjects with adverse events including subject identifier and year by treatment (FAS) (cont.)

TREATMENT: LCS16

Primary system organ class<br>Preferred term<br>MedDRA version 14.0	YEAR OF ONSET	N (%)	subject identifier
Hyperbilirubinaemia	1st year	1/1452 (<0.1%)	200707
	Overall	1/1452 (<0.1%)	200707
Immune system disorders	1st year	37/1452 (2.5%)	
	2nd year	22/1206 (1.8%)	
	3rd year	21/1012 (2.1%)	
	Overall	66/1452 (4.5%)	
Allergy to animal	1st year	2/1452 (0.1%)	160324, 161306
	Overall	2/1452 (0.1%)	160324, 161306
Allergy to arthropod bite	3rd year	1/1012 (<0.1%)	240517
	Overall	1/1452 (<0.1%)	240517
Allergy to arthropod sting	3rd year	1/1012 (<0.1%)	161311
	Overall	1/1452 (<0.1%)	161311
Anaphylactic reaction	3rd year	1/1012 (<0.1%)	244706
	Overall	1/1452 (<0.1%)	244706
Drug hypersensitivity	1st year	4/1452 (0.3%)	140205, 160621, 200610, 243319
	3rd year	2/1012 (0.2%)	140812, 240517
	Overall	6/1452 (0.4%)	140205, 140812, 160621, 200610, 240517, 243319
Food allergy	1st year	1/1452 (<0.1%)	241603
	Overall	1/1452 (<0.1%)	241603
Hypersensitivity	1st year	6/1452 (0.4%)	120612, 120618, 161116, 161211, 200231, 210413
	2nd year	5/1206 (0.4%)	120612, 160747, 161204, 240303, 245922
	3rd year	1/1012 (<0.1%)	120603
	Overall	11/1452 (0.8%)	120603, 120612, 120618, 160747, 161116, 161204, 161211, 200231, 210413, 240303, 245922

Table 14.3.1 / 10: Number of subjects with adverse events including subject identifier and year by treatment (FAS) (cont.)

TREATMENT: LCS16

Primary system organ class<br>Preferred term<br>MedDRA version 14.0	YEAR OF ONSET	N (%)	subject identifier
Seasonal allergy	1st year	24/1452 (1.7%)	140805, 160115, 160426, 160428, 160641, 160723, 160728, 160744, 160759, 160802, 160919, 160921, 161004, 161503, 161524, 161536, 200712, 230222, 230309, 241145, 241809, 243604, 244905, 245704
	2nd year	17/1206 (1.4%)	141111, 160115, 160428, 160429, 160642, 160744, 160759, 160919, 161004, 161432, 161502, 230506, 240133, 241420, 242918, 244424, 244902
	3rd year	16/1012 (1.6%)	160214, 160314, 160428, 160735, 160921, 161004, 161417, 161432, 161524, 200712, 230506, 240620, 241177, 242501, 245504, 246218
	Overall	44/1452 (3.0%)	140805, 141111, 160115, 160214, 160314, 160426, 160428, 160429, 160641, 160642, 160723, 160728, 160735, 160744, 160759, 160802, 160919, 160921, 161004, 161417, 161432, 161502, 161503, 161524, 161536, 200712, 230222, 230309, 230506, 240133, 240620, 241145, 241177, 241420, 241809, 242501, 242918, 243604, 244424, 244902, 244905, 245504, 245704, 246218
Infections and infestations	1st year	529/1452 (36.4%)	
	2nd year	335/1206 (27.8%)	
	3rd year	259/1012 (25.6%)	
	Overall	736/1452 (50.7%)	
Abscess limb	1st year	1/1452 (<0.1%)	242912
	Overall	1/1452 (<0.1%)	242912
Acute sinusitis	2nd year	2/1206 (0.2%)	161209, 240523
	3rd year	1/1012 (<0.1%)	161118
	Overall	3/1452 (0.2%)	161118, 161209, 240523
Acute tonsillitis	1st year	1/1452 (<0.1%)	160919
	2nd year	2/1206 (0.2%)	150140, 200902
	3rd year	2/1012 (0.2%)	160120, 160907
	Overall	5/1452 (0.3%)	150140, 160120, 160907, 160919, 200902

Table 14.3.1 / 10: Number of subjects with adverse events including subject identifier and year by treatment (FAS) (cont.)

TREATMENT: LCS16

Primary system organ class<br>Preferred term<br>MedDRA version 14.0	YEAR OF ONSET	N (%)	subject identifier
Anogenital warts	1st year	4/1452 (0.3%)	120314, 140101, 200401, 230918
	2nd year	3/1206 (0.2%)	210103, 230416, 245517
	3rd year	3/1012 (0.3%)	120334, 160210, 160748
	Overall	10/1452 (0.7%)	120314, 120334, 140101, 160210, 160748, 200401, 210103, 230416, 230918, 245517
Appendicitis	1st year	2/1452 (0.1%)	190202, 244433
	2nd year	3/1206 (0.2%)	141415, 200401, 243205
	3rd year	2/1012 (0.2%)	240905, 245805
	Overall	7/1452 (0.5%)	141415, 190202, 200401, 240905, 243205, 244433, 245805
Blastocystis infection	3rd year	1/1012 (<0.1%)	141035
	Overall	1/1452 (<0.1%)	141035
Body tinea	1st year	2/1452 (0.1%)	242503, 244902
	Overall	2/1452 (0.1%)	242503, 244902
Bronchitis	1st year	21/1452 (1.4%)	120101, 120334, 140610, 140920, 150139, 160202, 160332, 160532, 160608, 160972, 161016, 161306, 161528, 170703, 200920, 230516, 240320, 241157, 241524, 243228, 245918
	2nd year	15/1206 (1.2%)	140804, 150204, 160533, 160549, 160603, 160608, 160735, 161016, 161115, 161118, 230211, 241420, 241802, 244409, 244704
	3rd year	5/1012 (0.5%)	120334, 160324, 160748, 160921, 161529
	Overall	38/1452 (2.6%)	120101, 120334, 140610, 140804, 140920, 150139, 150204, 160202, 160324, 160332, 160532, 160533, 160549, 160603, 160608, 160735, 160748, 160921, 160972, 161016, 161115, 161118, 161306, 161528, 161529, 170703, 200920, 230211, 230516, 240320, 241157, 241420, 241524, 241802, 243228, 244409, 244704, 245918
Campylobacter intestinal infection	1st year	1/1452 (<0.1%)	230215
	Overall	1/1452 (<0.1%)	230215

Table 14.3.1 / 10: Number of subjects with adverse events including subject identifier and year by treatment (FAS) (cont.)

TREATMENT: LCS16

Primary system organ class<br>Preferred term<br>MedDRA version 14.0	YEAR OF ONSET	N (%)	subject identifier
Candidiasis	1st year	5/1452 (0.3%)	160126, 190103, 200605, 230717, 244305
	2nd year	2/1206 (0.2%)	161526, 246140
	3rd year	2/1012 (0.2%)	190212, 200922
	Overall	9/1452 (0.6%)	160126, 161526, 190103, 190212, 200605, 200922, 230717, 244305, 246140
Carbuncle	1st year	1/1452 (<0.1%)	160919
	Overall	1/1452 (<0.1%)	160919
Cellulitis	1st year	3/1452 (0.2%)	240616, 242912, 243027
	2nd year	1/1206 (<0.1%)	240616
	3rd year	1/1012 (<0.1%)	120627
	Overall	4/1452 (0.3%)	120627, 240616, 242912, 243027
Cervicitis	1st year	11/1452 (0.8%)	140112, 140805, 160503, 190103, 190108, 190404, 190416, 230315, 243001, 244802, 245429
	2nd year	2/1206 (0.2%)	120408, 140818
	3rd year	3/1012 (0.3%)	161118, 190103, 190414
	Overall	15/1452 (1.0%)	120408, 140112, 140805, 140818, 160503, 161118, 190103, 190108, 190404, 190414, 190416, 230315, 243001, 244802, 245429
Chlamydial cervicitis	1st year	1/1452 (<0.1%)	243909
	2nd year	3/1206 (0.2%)	231117, 240632, 241139
	3rd year	2/1012 (0.2%)	161123, 241219
	Overall	6/1452 (0.4%)	161123, 231117, 240632, 241139, 241219, 243909
Chlamydial infection	1st year	2/1452 (0.1%)	161008, 230513
	2nd year	1/1206 (<0.1%)	210206
	3rd year	1/1012 (<0.1%)	243967
	Overall	4/1452 (0.3%)	161008, 210206, 230513, 243967
Chronic tonsillitis	3rd year	1/1012 (<0.1%)	160126
	Overall	1/1452 (<0.1%)	160126
Conjunctivitis bacterial	1st year	1/1452 (<0.1%)	160969
	2nd year	1/1206 (<0.1%)	160602
	Overall	2/1452 (0.1%)	160602, 160969

Table 14.3.1 / 10: Number of subjects with adverse events including subject identifier and year by treatment (FAS) (cont.)

TREATMENT: LCS16

Primary system organ class / Preferred term / MedDRA version 14.0	YEAR OF ONSET	N (%)	subject identifier
Conjunctivitis viral	1st year	1/1452 (<0.1%)	120607
	Overall	1/1452 (<0.1%)	120607
Coxsackie viral infection	1st year	1/1452 (<0.1%)	161529
	Overall	1/1452 (<0.1%)	161529
Cystitis	1st year	13/1452 (0.9%)	140804, 160760, 161306, 180112, 180720, 200103, 200110, 200212, 200514, 200610, 200711, 243208, 245030
	2nd year	13/1206 (1.1%)	120303, 140812, 141411, 160602, 160752, 160760, 180112, 180314, 200110, 200513, 241117, 241121, 241141
	3rd year	6/1012 (0.6%)	140804, 160602, 160611, 200920, 241117, 241705
	Overall	26/1452 (1.8%)	120303, 140804, 140812, 141411, 160602, 160611, 160752, 160760, 161306, 180112, 180314, 180720, 200103, 200110, 200212, 200513, 200514, 200610, 200711, 200920, 241117, 241121, 241141, 241705, 243208, 245030
Diarrhoea infectious	1st year	1/1452 (<0.1%)	244521
	3rd year	1/1012 (<0.1%)	140512
	Overall	2/1452 (0.1%)	140512, 244521
Diverticulitis	2nd year	1/1206 (<0.1%)	240133
	Overall	1/1452 (<0.1%)	240133
Ear infection	1st year	7/1452 (0.5%)	141020, 141401, 150126, 160123, 161528, 170607, 243960
	2nd year	5/1206 (0.4%)	141020, 161118, 161204, 161306, 245806
	3rd year	3/1012 (0.3%)	141020, 161524, 200605
	Overall	13/1452 (0.9%)	141020, 141401, 150126, 160123, 161118, 161204, 161306, 161524, 161528, 170607, 200605, 243960, 245806

Table 14.3.1 / 10: Number of subjects with adverse events including subject identifier and year by treatment (FAS) (cont.)

TREATMENT: LCS16

Primary system organ class<br>Preferred term<br>MedDRA version 14.0	YEAR OF ONSET	N (%)	subject identifier
Endometritis	1st year	9/1452 (0.6%)	160504, 160718, 160727, 160748, 180661, 180678, 230909, 230916, 241726
	2nd year	3/1206 (0.2%)	160635, 160748, 240626
	Overall	11/1452 (0.8%)	160504, 160635, 160718, 160727, 160748, 180661, 180678, 230909, 230916, 240626, 241726
Enterobiasis	1st year	1/1452 (<0.1%)	161211
	Overall	1/1452 (<0.1%)	161211
Epstein-Barr virus infection	1st year	1/1452 (<0.1%)	140504
	Overall	1/1452 (<0.1%)	140504
Escherichia urinary tract infection	1st year	1/1452 (<0.1%)	170411
	Overall	1/1452 (<0.1%)	170411
Eye infection	3rd year	1/1012 (<0.1%)	160526
	Overall	1/1452 (<0.1%)	160526
Folliculitis	1st year	2/1452 (0.1%)	244606, 244801
	3rd year	4/1012 (0.4%)	140103, 161101, 161105, 200605
	Overall	6/1452 (0.4%)	140103, 161101, 161105, 200605, 244606, 244801
Fungal infection	1st year	6/1452 (0.4%)	160533, 170602, 170607, 190129, 243504, 243518
	2nd year	2/1206 (0.2%)	160533, 241714
	3rd year	1/1012 (<0.1%)	240605
	Overall	8/1452 (0.6%)	160533, 170602, 170607, 190129, 240605, 241714, 243504, 243518
Fungal skin infection	2nd year	1/1206 (<0.1%)	200520
	Overall	1/1452 (<0.1%)	200520
Furuncle	1st year	1/1452 (<0.1%)	244614
	Overall	1/1452 (<0.1%)	244614
Gastritis bacterial	1st year	1/1452 (<0.1%)	241211
	Overall	1/1452 (<0.1%)	241211

Table 14.3.1 / 10: Number of subjects with adverse events including subject identifier and year by treatment (FAS) (cont.)

TREATMENT: LCS16

Primary system organ class<br>Preferred term<br>MedDRA version 14.0	YEAR OF ONSET	N (%)	subject identifier
Gastritis viral	1st year	1/1452 (<0.1%)	140607
	Overall	1/1452 (<0.1%)	140607
Gastroenteritis	1st year	16/1452 (1.1%)	120606, 120612, 140526, 140818, 150154, 160109, 160219, 160632, 160808, 161118, 161226, 200906, 200930, 230215, 242505, 243319
	2nd year	6/1206 (0.5%)	120311, 140512, 160709, 161302, 170604, 243316
	3rd year	7/1012 (0.7%)	150137, 160735, 160760, 170502, 200922, 230514, 241809
	Overall	29/1452 (2.0%)	120311, 120606, 120612, 140512, 140526, 140818, 150137, 150154, 160109, 160219, 160632, 160709, 160735, 160760, 160808, 161118, 161226, 161302, 170502, 170604, 200906, 200922, 200930, 230215, 230514, 241809, 242505, 243316, 243319
Gastroenteritis rotavirus	1st year	1/1452 (<0.1%)	150122
	Overall	1/1452 (<0.1%)	150122
Gastroenteritis viral	1st year	4/1452 (0.3%)	240708, 242503, 243902, 245806
	2nd year	4/1206 (0.3%)	241401, 241417, 243316, 245913
	3rd year	1/1012 (<0.1%)	242503
	Overall	8/1452 (0.6%)	240708, 241401, 241417, 242503, 243316, 243902, 245806, 245913
Gastrointestinal bacterial infection	1st year	1/1452 (<0.1%)	160530
	Overall	1/1452 (<0.1%)	160530
Gastrointestinal viral infection	2nd year	1/1206 (<0.1%)	243331
	3rd year	1/1012 (<0.1%)	241308
	Overall	2/1452 (0.1%)	241308, 243331
Genital candidiasis	1st year	2/1452 (0.1%)	230416, 230909
	2nd year	1/1206 (<0.1%)	160114
	Overall	3/1452 (0.2%)	160114, 230416, 230909

Table 14.3.1 / 10: Number of subjects with adverse events including subject identifier and year by treatment (FAS) (cont.)

TREATMENT: LCS16

Primary system organ class<br>Preferred term<br>MedDRA version 14.0	YEAR OF ONSET	N (%)	subject identifier
Genital herpes	1st year	8/1452 (0.6%)	141415, 160114, 160748, 161118, 210212, 241137, 241415, 241546
	2nd year	3/1206 (0.2%)	160955, 160978, 241420
	3rd year	4/1012 (0.4%)	160546, 160760, 240636, 241546
	Overall	14/1452 (1.0%)	141415, 160114, 160546, 160748, 160760, 160955, 160978, 161118, 210212, 240636, 241137, 241415, 241420, 241546
Gonorrhoea	1st year	1/1452 (<0.1%)	243034
	Overall	1/1452 (<0.1%)	243034
Gynaecological chlamydia infection	1st year	2/1452 (0.1%)	140810, 245704
	2nd year	2/1206 (0.2%)	161214, 210212
	3rd year	2/1012 (0.2%)	160714, 161214
	Overall	5/1452 (0.3%)	140810, 160714, 161214, 210212, 245704
H1N1 influenza	2nd year	5/1206 (0.4%)	140912, 150118, 160316, 160759, 242808
	Overall	5/1452 (0.3%)	140912, 150118, 160316, 160759, 242808
Haemophilus infection	1st year	1/1452 (<0.1%)	243909
	Overall	1/1452 (<0.1%)	243909
Hand-foot-and-mouth disease	3rd year	1/1012 (<0.1%)	160543
	Overall	1/1452 (<0.1%)	160543
Helicobacter gastritis	2nd year	1/1206 (<0.1%)	241170
	Overall	1/1452 (<0.1%)	241170
Herpes dermatitis	1st year	1/1452 (<0.1%)	210405
	Overall	1/1452 (<0.1%)	210405
Herpes simplex	2nd year	2/1206 (0.2%)	210405, 245513
	3rd year	1/1012 (<0.1%)	141016
	Overall	3/1452 (0.2%)	141016, 210405, 245513

Table 14.3.1 / 10: Number of subjects with adverse events including subject identifier and year by treatment (FAS) (cont.)

TREATMENT: LCS16

Primary system organ class<br>Preferred term<br>MedDRA version 14.0	YEAR OF ONSET	N (%)	subject identifier
Herpes zoster	1st year	1/1452 (<0.1%)	160123
	2nd year	2/1206 (0.2%)	161401, 245908
	3rd year	1/1012 (<0.1%)	161107
	Overall	4/1452 (0.3%)	160123, 161107, 161401, 245908
Impetigo	1st year	1/1452 (<0.1%)	161502
	2nd year	1/1206 (<0.1%)	160969
	Overall	2/1452 (0.1%)	160969, 161502
Infected cyst	2nd year	1/1206 (<0.1%)	140112
	Overall	1/1452 (<0.1%)	140112
Infection	2nd year	1/1206 (<0.1%)	160438
	Overall	1/1452 (<0.1%)	160438
Infectious mononucleosis	1st year	2/1452 (0.1%)	141420, 200603
	2nd year	4/1206 (0.3%)	161417, 230402, 243504, 243902
	Overall	6/1452 (0.4%)	141420, 161417, 200603, 230402, 243504, 243902

Table 14.3.1 / 10: Number of subjects with adverse events including subject identifier and year by treatment (FAS) (cont.)

TREATMENT: LCS16

Primary system organ class<br>Preferred term<br>MedDRA version 14.0	YEAR OF ONSET	N (%)	subject identifier
Influenza	1st year	59/1452 (4.1%)	120103, 120303, 120317, 120328, 120404, 120425, 120612, 140101, 140103, 140205, 140524, 141415, 160307, 160314, 160319, 160324, 160508, 160546, 160603, 160611, 160618, 160621, 160622, 160627, 160629, 160635, 160636, 160730, 160751, 161223, 161302, 161310, 161311, 161320, 161321, 190102, 200102, 200116, 200225, 200509, 200513, 200525, 210103, 210105, 210112, 210133, 210212, 210605, 230119, 230305, 230609, 230811, 231109, 241503, 241901, 242312, 243211, 243225, 244309
	2nd year	32/1206 (2.7%)	120317, 120334, 120410, 120425, 120609, 140506, 141411, 160219, 160428, 160563, 160629, 160632, 160636, 160745, 160748, 160752, 161211, 170403, 170604, 200118, 210103, 210123, 210413, 210605, 230317, 240133, 241418, 241731, 242317, 242501, 243714, 243976
	3rd year	14/1012 (1.4%)	120309, 120317, 120410, 160332, 160553, 160602, 160611, 160618, 160632, 160906, 180717, 240631, 241809, 243316
	Overall	94/1452 (6.5%)	120103, 120303, 120309, 120317, 120328, 120334, 120404, 120410, 120425, 120609, 120612, 140101, 140103, 140205, 140506, 140524, 141411, 141415, 160219, 160307, 160314, 160319, 160324, 160332, 160428, 160508, 160546, 160553, 160563, 160602, 160603, 160611, 160618, 160621, 160622, 160627, 160629, 160632, 160635, 160636, 160730, 160745, 160748, 160751, 160752, 160906, 161211, 161223, 161302, 161310, 161311, 161320, 161321, 170403, 170604, 180717, 190102, 200102, 200116, 200118, 200225, 200509, 200513, 200525, 210103, 210105, 210112, 210123, 210133, 210212, 210413, 210605, 230119, 230305, 230317, 230609, 230811, 231109, 240133, 240631, 241418, 241503, 241731, 241809, 241901, 242312, 242317, 242501, 243211, 243225, 243316, 243714, 243976, 244309

Table 14.3.1 / 10: Number of subjects with adverse events including subject identifier and year by treatment (FAS) (cont.)

TREATMENT: LCS16

Primary system organ class<br>Preferred term<br>MedDRA version 14.0	YEAR OF ONSET	N (%)	subject identifier
Keratitis viral	1st year	1/1452 (<0.1%)	140812
	Overall	1/1452 (<0.1%)	140812
Kidney infection	1st year	1/1452 (<0.1%)	245924
	2nd year	3/1206 (0.2%)	243934, 244446, 245908
	Overall	4/1452 (0.3%)	243934, 244446, 245908, 245924
Labyrinthitis	1st year	1/1452 (<0.1%)	141102
	Overall	1/1452 (<0.1%)	141102
Laryngitis	1st year	5/1452 (0.3%)	120408, 160737, 161526, 200212, 243316
	2nd year	4/1206 (0.3%)	160628, 160724, 161004, 170408
	3rd year	2/1012 (0.2%)	161004, 200922
	Overall	10/1452 (0.7%)	120408, 160628, 160724, 160737, 161004, 161526, 170408, 200212, 200922, 243316
Laryngitis bacterial	1st year	1/1452 (<0.1%)	140101
	Overall	1/1452 (<0.1%)	140101
Localised infection	1st year	2/1452 (0.1%)	200702, 242317
	Overall	2/1452 (0.1%)	200702, 242317
Lower respiratory tract infection	2nd year	1/1206 (<0.1%)	245522
	Overall	1/1452 (<0.1%)	245522
Malaria	2nd year	1/1206 (<0.1%)	161025
	Overall	1/1452 (<0.1%)	161025
Mastitis	1st year	1/1452 (<0.1%)	120635
	Overall	1/1452 (<0.1%)	120635
Meningitis viral	3rd year	1/1012 (<0.1%)	245427
	Overall	1/1452 (<0.1%)	245427
Molluscum contagiosum	3rd year	1/1012 (<0.1%)	150120
	Overall	1/1452 (<0.1%)	150120

Table 14.3.1 / 10: Number of subjects with adverse events including subject identifier and year by treatment (FAS) (cont.)

TREATMENT: LCS16

Primary system organ class<br>Preferred term<br>MedDRA version 14.0	YEAR OF ONSET	N (%)	subject identifier
Nail infection	1st year	1/1452 (<0.1%)	245526
	Overall	1/1452 (<0.1%)	245526

Table 14.3.1 / 10: Number of subjects with adverse events including subject identifier and year by treatment (FAS) (cont.)

TREATMENT: LCS16

Primary system organ class<br>Preferred term<br>MedDRA version 14.0	YEAR OF ONSET	N (%)	subject identifier
Nasopharyngitis	1st year	73/1452 (5.0%)	120334, 120606, 140101, 140211, 140906, 141304, 141410, 150137, 150139, 150159, 160114, 160115, 160126, 160724, 160735, 160748, 161302, 161320, 161321, 161535, 180702, 180718, 180720, 180737, 210102, 210121, 210212, 210410, 230105, 230118, 230119, 230309, 230414, 230503, 230505, 230507, 230610, 230612, 230614, 230810, 230905, 230915, 231011, 231020, 231104, 240127, 240133, 240504, 241137, 241138, 241152, 241709, 241809, 242301, 242310, 242503, 242908, 242918, 243205, 243606, 243923, 243936, 243949, 243971, 243975, 244123, 244305, 244315, 244317, 244902, 244923, 244926, 245511
	2nd year	36/1206 (3.0%)	120324, 120328, 140101, 140106, 140120, 140205, 140504, 140506, 141304, 141401, 141408, 150141, 150159, 160702, 160709, 160716, 160761, 161306, 161320, 161529, 210413, 230111, 230507, 230609, 230614, 230822, 230905, 231001, 231020, 231104, 241809, 242918, 243225, 244106, 244123, 244401
	3rd year	24/1012 (2.4%)	140119, 140805, 141420, 150115, 150118, 150137, 160709, 160719, 161306, 161320, 180718, 210121, 210413, 230305, 230309, 230503, 240127, 242904, 242918, 243928, 243960, 244130, 244317, 244410
	Overall	109/1452 (7.5%)	120324, 120328, 120334, 120606, 140101, 140106, 140119, 140120, 140205, 140211, 140504, 140506, 140805, 140906, 141304, 141401, 141408, 141410, 141420, 150115, 150118, 150137, 150139, 150141, 150159, 160114, 160115, 160126, 160702, 160709, 160716, 160719, 160724, 160735, 160748, 160761, 161302, 161306, 161320, 161321, 161529, 161535, 180702, 180718, 180720, 180737, 210102, 210121, 210212, 210410, 210413, 230105, 230111, 230118, 230119, 230305, 230309, 230414, 230503, 230505, 230507, 230609, 230610, 230612, 230614, 230810, 230822, 230905, 230915, 231001, 231011, 231020, 231104, 240127, 240133,

Table 14.3.1 / 10: Number of subjects with adverse events including subject identifier and year by treatment (FAS) (cont.)

TREATMENT: LCS16

Primary system organ class<br>Preferred term<br>MedDRA version 14.0	YEAR OF ONSET	N (%)	subject identifier
			240504, 241137, 241138, 241152, 241709, 241809, 242301, 242310, 242503, 242904, 242908, 242918, 243205, 243225, 243606, 243923, 243928, 243936, 243949, 243960, 243971, 243975, 244106, 244123, 244130, 244305, 244315, 244317, 244401, 244410, 244902, 244923, 244926, 245511
Nipple infection	3rd year	1/1012 (<0.1%)	241705
	Overall	1/1452 (<0.1%)	241705
Omphalitis	1st year	1/1452 (<0.1%)	150126
	Overall	1/1452 (<0.1%)	150126
Onychomycosis	1st year	2/1452 (0.1%)	161433, 242324
	2nd year	2/1206 (0.2%)	120308, 180732
	Overall	4/1452 (0.3%)	120308, 161433, 180732, 242324
Oophoritis	1st year	1/1452 (<0.1%)	180112
	Overall	1/1452 (<0.1%)	180112
Oral candidiasis	2nd year	1/1206 (<0.1%)	160635
	Overall	1/1452 (<0.1%)	160635
Oral herpes	1st year	3/1452 (0.2%)	120334, 140810, 245511
	2nd year	4/1206 (0.3%)	141016, 160115, 160969, 161534
	3rd year	4/1012 (0.4%)	140805, 150114, 160115, 210405
	Overall	10/1452 (0.7%)	120334, 140805, 140810, 141016, 150114, 160115, 160969, 161534, 210405, 245511
Osteomyelitis	1st year	1/1452 (<0.1%)	231011
	2nd year	1/1206 (<0.1%)	231011
	Overall	1/1452 (<0.1%)	231011
Otitis externa	2nd year	2/1206 (0.2%)	140601, 160642
	3rd year	2/1012 (0.2%)	160526, 160642
	Overall	3/1452 (0.2%)	140601, 160526, 160642

Table 14.3.1 / 10: Number of subjects with adverse events including subject identifier and year by treatment (FAS) (cont.)

TREATMENT: LCS16

Primary system organ class<br>Preferred term<br>MedDRA version 14.0	YEAR OF ONSET	N (%)	subject identifier
Otitis media	1st year	3/1452 (0.2%)	160332, 160627, 161423
	2nd year	3/1206 (0.2%)	160221, 160627, 161434
	3rd year	1/1012 (<0.1%)	160324
	Overall	6/1452 (0.4%)	160221, 160324, 160332, 160627, 161423, 161434
Otitis media acute	1st year	1/1452 (<0.1%)	200510
	Overall	1/1452 (<0.1%)	200510
Papilloma viral infection	1st year	1/1452 (<0.1%)	230415
	Overall	1/1452 (<0.1%)	230415
Paronychia	1st year	1/1452 (<0.1%)	160961
	2nd year	1/1206 (<0.1%)	160934
	Overall	2/1452 (0.1%)	160934, 160961
Pelvic infection	1st year	1/1452 (<0.1%)	170602
	Overall	1/1452 (<0.1%)	170602
Pelvic inflammatory disease	1st year	4/1452 (0.3%)	141407, 160410, 161024, 180629
	3rd year	1/1012 (<0.1%)	180667
	Overall	5/1452 (0.3%)	141407, 160410, 161024, 180629, 180667
Peritonsillar abscess	1st year	1/1452 (<0.1%)	160919
	Overall	1/1452 (<0.1%)	160919
Pharyngitis	1st year	14/1452 (1.0%)	120103, 120309, 120406, 120410, 120420, 120606, 120607, 120622, 140604, 150114, 160327, 160761, 190408, 200603
	2nd year	4/1206 (0.3%)	120308, 120328, 160126, 161204
	3rd year	4/1012 (0.4%)	120308, 140112, 190123, 244802
	Overall	21/1452 (1.4%)	120103, 120308, 120309, 120328, 120406, 120410, 120420, 120606, 120607, 120622, 140112, 140604, 150114, 160126, 160327, 160761, 161204, 190123, 190408, 200603, 244802

Table 14.3.1 / 10: Number of subjects with adverse events including subject identifier and year by treatment (FAS) (cont.)

TREATMENT: LCS16

Primary system organ class<br>Preferred term<br>MedDRA version 14.0	YEAR OF ONSET	N (%)	subject identifier
Pharyngitis streptococcal	1st year	12/1452 (0.8%)	140909, 141411, 210103, 241114, 241205, 241243, 241401, 241533, 242905, 242918, 243223, 245927
	2nd year	5/1206 (0.4%)	140514, 141411, 243315, 243504, 243902
	3rd year	5/1012 (0.5%)	140112, 141304, 242310, 244435, 246218
	Overall	21/1452 (1.4%)	140112, 140514, 140909, 141304, 141411, 210103, 241114, 241205, 241243, 241401, 241533, 242310, 242905, 242918, 243223, 243315, 243504, 243902, 244435, 245927, 246218
Pneumonia	1st year	6/1452 (0.4%)	140805, 200217, 200510, 230305, 242905, 244305
	2nd year	8/1206 (0.7%)	120635, 140103, 180721, 230126, 230211, 230220, 230614, 243909
	3rd year	2/1012 (0.2%)	161123, 244305
	Overall	15/1452 (1.0%)	120635, 140103, 140805, 161123, 180721, 200217, 200510, 230126, 230211, 230220, 230305, 230614, 242905, 243909, 244305
Post procedural infection	3rd year	1/1012 (<0.1%)	161309
	Overall	1/1452 (<0.1%)	161309
Pulpitis dental	2nd year	1/1206 (<0.1%)	161123
	Overall	1/1452 (<0.1%)	161123
Pyelonephritis	1st year	2/1452 (0.1%)	140504, 141023
	2nd year	1/1206 (<0.1%)	200212
	Overall	3/1452 (0.2%)	140504, 141023, 200212
Rash pustular	1st year	1/1452 (<0.1%)	242915
	Overall	1/1452 (<0.1%)	242915
Respiratory tract infection	1st year	7/1452 (0.5%)	140909, 160702, 160707, 161116, 161302, 161306, 161502
	2nd year	5/1206 (0.4%)	120612, 161116, 161123, 161306, 161526
	3rd year	9/1012 (0.9%)	160747, 161105, 161107, 161115, 161123, 161214, 161306, 161526, 243923
	Overall	16/1452 (1.1%)	120612, 140909, 160702, 160707, 160747, 161105, 161107, 161115, 161116, 161123, 161214, 161302, 161306, 161502, 161526, 243923

Table 14.3.1 / 10: Number of subjects with adverse events including subject identifier and year by treatment (FAS) (cont.)

TREATMENT: LCS16

Primary system organ class<br>Preferred term<br>MedDRA version 14.0	YEAR OF ONSET	N (%)	subject identifier
Respiratory tract infection viral	1st year	1/1452 (<0.1%)	243319
	Overall	1/1452 (<0.1%)	243319
Rhinitis	1st year	7/1452 (0.5%)	120404, 120406, 120420, 160611, 161223, 210410, 241802
	2nd year	4/1206 (0.3%)	140611, 161025, 161131, 161535
	3rd year	1/1012 (<0.1%)	210605
	Overall	12/1452 (0.8%)	120404, 120406, 120420, 140611, 160611, 161025, 161131, 161223, 161535, 210410, 210605, 241802
Salpingo-oophoritis	1st year	3/1452 (0.2%)	180117, 180702, 180713
	Overall	3/1452 (0.2%)	180117, 180702, 180713
Sinobronchitis	3rd year	1/1012 (<0.1%)	161529
	Overall	1/1452 (<0.1%)	161529

Table 14.3.1 / 10: Number of subjects with adverse events including subject identifier and year by treatment (FAS) (cont.)

TREATMENT: LCS16

Primary system organ class<br>Preferred term<br>MedDRA version 14.0	YEAR OF ONSET	N (%)	subject identifier
Sinusitis	1st year	47/1452 (3.2%)	140106, 140514, 140805, 140818, 141030, 141411, 150122, 160319, 160504, 160508, 160531, 160608, 160621, 160632, 160808, 161204, 161217, 161508, 161534, 161535, 161536, 180708, 200620, 200632, 200701, 200920, 210133, 230309, 240313, 240633, 240705, 240803, 241155, 241301, 241417, 241501, 241535, 241802, 242310, 242321, 243215, 243901, 243957, 244514, 245522, 245801, 246218
	2nd year	34/1206 (2.8%)	140804, 140812, 141018, 160319, 160324, 160429, 160532, 160543, 160553, 160603, 160719, 161108, 161209, 161223, 161315, 161524, 161534, 161535, 161536, 170604, 180732, 200509, 200609, 200920, 200922, 230915, 240240, 240705, 243822, 244123, 244202, 244433, 245905, 245927
	3rd year	29/1012 (2.9%)	120617, 160319, 160543, 160608, 160755, 160906, 160919, 160921, 160987, 161223, 161306, 161311, 161315, 161434, 161524, 200509, 210601, 230222, 230305, 240614, 240626, 240631, 241401, 241809, 242705, 244315, 244517, 245908, 246218
	Overall	96/1452 (6.6%)	120617, 140106, 140514, 140804, 140805, 140812, 140818, 141018, 141030, 141411, 150122, 160319, 160324, 160429, 160504, 160508, 160531, 160532, 160543, 160553, 160603, 160608, 160621, 160632, 160719, 160755, 160808, 160906, 160919, 160921, 160987, 161108, 161204, 161209, 161217, 161223, 161306, 161311, 161315, 161434, 161508, 161524, 161534, 161535, 161536, 170604, 180708, 180732, 200509, 200609, 200620, 200632, 200701, 200920, 200922, 210133, 210601, 230222, 230305, 230309, 230915, 240240, 240313, 240614, 240626, 240631, 240633, 240705, 240803, 241155, 241301, 241401, 241417, 241501, 241535, 241802, 241809, 242310, 242321, 242705, 243215, 243822, 243901, 243957, 244123, 244202, 244315, 244433, 244514, 244517, 245522, 245801, 245905, 245908, 245927, 246218

Table 14.3.1 / 10: Number of subjects with adverse events including subject identifier and year by treatment (FAS) (cont.)

TREATMENT: LCS16

Primary system organ class<br>Preferred term<br>MedDRA version 14.0	YEAR OF ONSET	N (%)	subject identifier
Skin bacterial infection	1st year	1/1452 (<0.1%)	243319
	3rd year	1/1012 (<0.1%)	141108
	Overall	2/1452 (0.1%)	141108, 243319
Skin infection	1st year	2/1452 (0.1%)	150117, 245526
	2nd year	2/1206 (0.2%)	161306, 210405
	3rd year	2/1012 (0.2%)	230601, 241603
	Overall	6/1452 (0.4%)	150117, 161306, 210405, 230601, 241603, 245526
Small intestinal bacterial overgrowth	3rd year	1/1012 (<0.1%)	243501
	Overall	1/1452 (<0.1%)	243501
Staphylococcal infection	1st year	1/1452 (<0.1%)	240633
	Overall	1/1452 (<0.1%)	240633
Subcutaneous abscess	2nd year	2/1206 (0.2%)	120229, 243205
	Overall	2/1452 (0.1%)	120229, 243205
Tinea infection	3rd year	2/1012 (0.2%)	140526, 244410
	Overall	2/1452 (0.1%)	140526, 244410
Tinea pedis	1st year	1/1452 (<0.1%)	161422
	Overall	1/1452 (<0.1%)	161422
Tinea versicolour	1st year	1/1452 (<0.1%)	241556
	Overall	1/1452 (<0.1%)	241556
Tonsillitis	1st year	8/1452 (0.6%)	120631, 160611, 160636, 210501, 230305, 230404, 230811, 230814
	2nd year	8/1206 (0.7%)	120635, 141016, 141030, 150118, 160987, 161016, 161306, 243902
	3rd year	8/1012 (0.8%)	120410, 161506, 180625, 190114, 230609, 230614, 230704, 245806
	Overall	24/1452 (1.7%)	120410, 120631, 120635, 141016, 141030, 150118, 160611, 160636, 160987, 161016, 161306, 161506, 180625, 190114, 210501, 230305, 230404, 230609, 230614, 230704, 230811, 230814, 243902, 245806

Table 14.3.1 / 10: Number of subjects with adverse events including subject identifier and year by treatment (FAS) (cont.)

TREATMENT: LCS16

Primary system organ class<br>Preferred term<br>MedDRA version 14.0	YEAR OF ONSET	N (%)	subject identifier
Tooth abscess	1st year	3/1452 (0.2%)	242505, 243101, 244916
	2nd year	1/1206 (<0.1%)	245918
	Overall	4/1452 (0.3%)	242505, 243101, 244916, 245918
Tooth infection	1st year	7/1452 (0.5%)	120607, 150165, 160716, 161211, 210212, 231011, 231021
	2nd year	3/1206 (0.2%)	210405, 243813, 244519
	3rd year	6/1012 (0.6%)	120425, 120631, 140915, 210128, 210405, 244123
	Overall	15/1452 (1.0%)	120425, 120607, 120631, 140915, 150165, 160716, 161211, 210128, 210212, 210405, 231011, 231021, 243813, 244123, 244519
Trichomoniasis	2nd year	1/1206 (<0.1%)	241139
	3rd year	1/1012 (<0.1%)	241709
	Overall	2/1452 (0.1%)	241139, 241709
Typhoid fever	1st year	1/1452 (<0.1%)	190108
	Overall	1/1452 (<0.1%)	190108

Table 14.3.1 / 10: Number of subjects with adverse events including subject identifier and year by treatment (FAS) (cont.)

TREATMENT: LCS16

Primary system organ class<br>Preferred term<br>MedDRA version 14.0	YEAR OF ONSET	N (%)	subject identifier
Upper respiratory tract infection	1st year	41/1452 (2.8%)	140810, 140818, 160737, 160975, 161004, 161116, 230209, 230514, 240506, 240522, 240614, 241308, 241310, 241401, 241415, 241417, 241533, 241901, 242918, 243301, 243303, 243315, 243321, 243501, 243608, 244401, 244410, 244424, 244437, 244449, 244501, 244514, 244517, 244519, 244520, 244619, 244935, 245524, 245922, 245927, 245930
	2nd year	20/1206 (1.7%)	161004, 161016, 161025, 161116, 161131, 161402, 240717, 241420, 241430, 242904, 243301, 243401, 243822, 244424, 244519, 244520, 244525, 244619, 244808, 245908
	3rd year	8/1012 (0.8%)	161116, 230209, 240333, 241308, 241310, 241401, 241412, 241731
	Overall	57/1452 (3.9%)	140810, 140818, 160737, 160975, 161004, 161016, 161025, 161116, 161131, 161402, 230209, 230514, 240333, 240506, 240522, 240614, 240717, 241308, 241310, 241401, 241412, 241415, 241417, 241420, 241430, 241533, 241731, 241901, 242904, 242918, 243301, 243303, 243315, 243321, 243401, 243501, 243608, 243822, 244401, 244410, 244424, 244437, 244449, 244501, 244514, 244517, 244519, 244520, 244525, 244619, 244808, 244935, 245524, 245908, 245922, 245927, 245930

Table 14.3.1 / 10: Number of subjects with adverse events including subject identifier and year by treatment (FAS) (cont.)

TREATMENT: LCS16

Primary system organ class<br>Preferred term<br>MedDRA version 14.0	YEAR OF ONSET	N (%)	subject identifier
Urinary tract infection	1st year	87/1452 (6.0%)	120102, 120309, 120415, 120606, 120617, 120618, 140526, 140610, 140720, 140915, 150113, 150120, 150121, 150137, 150141, 150150, 150160, 150211, 160109, 160122, 160210, 160503, 160602, 160628, 160632, 160642, 160718, 160727, 160955, 160966, 160987, 161008, 161113, 161307, 161401, 161402, 161415, 161433, 190142, 210105, 210405, 210413, 230121, 230207, 230305, 230306, 230601, 230614, 231013, 231015, 231110, 240523, 240620, 240708, 240803, 241114, 241140, 241152, 241235, 241301, 241403, 241537, 241608, 241716, 241726, 242813, 243024, 243211, 243321, 243601, 243604, 243909, 243964, 244120, 244128, 244409, 244433, 244449, 244521, 244703, 244801, 245409, 245504, 245526, 245704, 245908, 245927
	2nd year	38/1206 (3.2%)	120612, 120635, 140506, 140509, 141413, 150113, 150126, 150141, 150504, 160534, 160603, 160723, 161008, 161302, 161402, 210132, 230416, 230807, 230811, 231021, 240103, 240133, 240620, 240632, 240722, 241310, 241519, 242501, 243316, 243911, 243936, 244429, 244514, 244521, 244616, 244801, 245801, 246201
	3rd year	38/1012 (3.8%)	120328, 140216, 140509, 140601, 140611, 140812, 150126, 150141, 160419, 160529, 160534, 160603, 160642, 160961, 161311, 161538, 170408, 180640, 180659, 230106, 230207, 230807, 231011, 240711, 241173, 241510, 241546, 242505, 242801, 242908, 243319, 243937, 243976, 244406, 244703, 244802, 245927, 246201
	Overall	145/1452 (10.0%)	120102, 120309, 120328, 120415, 120606, 120612, 120617, 120618, 120635, 140216, 140506, 140509, 140526, 140601, 140610, 140611, 140720, 140812, 140915, 141413, 150113, 150120, 150121, 150126, 150137,

Table 14.3.1 / 10: Number of subjects with adverse events including subject identifier and year by treatment (FAS) (cont.)

TREATMENT: LCS16

Primary system organ class<br>Preferred term<br>MedDRA version 14.0	YEAR OF ONSET	N (%)	subject identifier
			150141, 150150, 150160, 150211, 150504, 160109, 160122, 160210, 160419, 160503, 160529, 160534, 160602, 160603, 160628, 160632, 160642, 160718, 160723, 160727, 160955, 160961, 160966, 160987, 161008, 161113, 161302, 161307, 161311, 161401, 161402, 161415, 161433, 161538, 170408, 180640, 180659, 190142, 210105, 210132, 210405, 210413, 230106, 230121, 230207, 230305, 230306, 230416, 230601, 230614, 230807, 230811, 231011, 231013, 231015, 231021, 231110, 240103, 240133, 240523, 240620, 240632, 240708, 240711, 240722, 240803, 241114, 241140, 241152, 241173, 241235, 241301, 241310, 241403, 241510, 241519, 241537, 241546, 241608, 241716, 241726, 242501, 242505, 242801, 242813, 242908, 243024, 243211, 243316, 243319, 243321, 243601, 243604, 243909, 243911, 243936, 243937, 243964, 243976, 244120, 244128, 244406, 244409, 244429, 244433, 244449, 244514, 244521, 244616, 244703, 244801, 244802, 245409, 245504, 245526, 245704, 245801, 245908, 245927, 246201
Uterine infection	1st year	1/1452 (<0.1%)	244804
	Overall	1/1452 (<0.1%)	244804

Table 14.3.1 / 10: Number of subjects with adverse events including subject identifier and year by treatment (FAS) (cont.)

TREATMENT: LCS16

Primary system organ class<br>Preferred term<br>MedDRA version 14.0	YEAR OF ONSET	N (%)	subject identifier
Vaginal infection	1st year	40/1452 (2.8%)	120125, 120408, 140506, 140512, 140514, 140526, 140801, 140810, 140812, 150157, 160312, 160441, 160503, 160529, 160608, 160622, 160632, 160635, 160641, 160961, 161008, 161223, 161429, 180112, 180708, 190114, 190122, 190142, 190202, 190206, 190207, 190212, 190213, 190214, 190227, 210117, 210123, 230717, 241415, 244801
	2nd year	10/1206 (0.8%)	140120, 140812, 141024, 160319, 160552, 170701, 180708, 180738, 243315, 244616
	3rd year	15/1012 (1.5%)	140610, 140812, 141030, 141304, 160570, 160612, 160632, 161302, 180340, 190134, 190142, 190604, 240636, 244202, 246134
	Overall	60/1452 (4.1%)	120125, 120408, 140120, 140506, 140512, 140514, 140526, 140610, 140801, 140810, 140812, 141024, 141030, 141304, 150157, 160312, 160319, 160441, 160503, 160529, 160552, 160570, 160608, 160612, 160622, 160632, 160635, 160641, 160961, 161008, 161223, 161302, 161429, 170701, 180112, 180340, 180708, 180738, 190114, 190122, 190134, 190142, 190202, 190206, 190207, 190212, 190213, 190214, 190227, 190604, 210117, 210123, 230717, 240636, 241415, 243315, 244202, 244616, 244801, 246134

Table 14.3.1 / 10: Number of subjects with adverse events including subject identifier and year by treatment (FAS) (cont.)

TREATMENT: LCS16

Primary system organ class<br>Preferred term<br>MedDRA version 14.0	YEAR OF ONSET	N (%)	subject identifier
Vaginitis bacterial	1st year	61/1452 (4.2%)	120337, 120402, 140610, 140801, 140804, 140916, 141111, 150113, 150126, 150140, 150150, 160602, 160718, 160762, 180632, 180678, 210112, 210133, 230416, 240114, 240128, 240233, 240501, 240506, 240522, 240621, 240624, 240636, 240708, 241008, 241022, 241301, 241503, 241522, 241537, 241546, 242817, 243032, 243034, 243406, 243956, 243966, 243967, 243977, 244113, 244120, 244133, 244303, 244716, 244801, 244802, 244916, 244922, 244926, 244935, 245002, 245013, 245030, 245704, 246113, 246146
	2nd year	50/1206 (4.1%)	120229, 140804, 141401, 150164, 160762, 160945, 161113, 190604, 190607, 200301, 210112, 240106, 240605, 240626, 240632, 240803, 240906, 241002, 241101, 241139, 241510, 241519, 241522, 241535, 241709, 241714, 242317, 242801, 243017, 243703, 243827, 243906, 243934, 243937, 243967, 244113, 244437, 244520, 244801, 244802, 244805, 244936, 245018, 245513, 245517, 245909, 245918, 246123, 246137, 246207
	3rd year	45/1012 (4.4%)	140804, 140812, 140901, 150160, 160128, 160312, 160314, 160762, 160916, 160945, 160955, 160961, 190206, 190633, 200702, 210110, 210511, 230402, 230715, 240103, 240126, 240523, 240605, 240632, 240636, 241103, 241301, 241418, 241420, 241546, 241714, 242801, 243315, 243501, 243703, 243934, 243937, 244113, 244520, 244802, 245018, 245030, 245517, 246123, 246137
	Overall	127/1452 (8.7%)	120229, 120337, 120402, 140610, 140801, 140804, 140812, 140901, 140916, 141111, 141401, 150113, 150126, 150140, 150150, 150160, 150164, 160128, 160312, 160314, 160602, 160718, 160762, 160916, 160945, 160955, 160961, 161113, 180632, 180678, 190206, 190604, 190607, 190633, 200301, 200702, 210110, 210112, 210133, 210511, 230402, 230416, 230715, 240103, 240106, 240114, 240126, 240128, 240233, 240501,

Table 14.3.1 / 10: Number of subjects with adverse events including subject identifier and year by treatment (FAS) (cont.)

TREATMENT: LCS16

Primary system organ class<br>Preferred term<br>MedDRA version 14.0	YEAR OF ONSET	N (%)	subject identifier
			240506, 240522, 240523, 240605, 240621, 240624, 240626, 240632, 240636, 240708, 240803, 240906, 241002, 241008, 241022, 241101, 241103, 241139, 241301, 241418, 241420, 241503, 241510, 241519, 241522, 241535, 241537, 241546, 241709, 241714, 242317, 242801, 242817, 243017, 243032, 243034, 243315, 243406, 243501, 243703, 243827, 243906, 243934, 243937, 243956, 243966, 243967, 243977, 244113, 244120, 244133, 244303, 244437, 244520, 244716, 244801, 244802, 244805, 244916, 244922, 244926, 244935, 244936, 245002, 245013, 245018, 245030, 245513, 245517, 245704, 245909, 245918, 246113, 246123, 246137, 246146, 246207
Vaginitis chlamydial	1st year	1/1452 (<0.1%)	231010
	Overall	1/1452 (<0.1%)	231010
Vaginitis gardnerella	1st year	2/1452 (0.1%)	230609, 240708
	2nd year	1/1206 (<0.1%)	200707
	3rd year	1/1012 (<0.1%)	200302
	Overall	4/1452 (0.3%)	200302, 200707, 230609, 240708
Viral infection	1st year	2/1452 (0.1%)	242801, 244516
	2nd year	1/1206 (<0.1%)	242704
	Overall	3/1452 (0.2%)	242704, 242801, 244516
Viral pharyngitis	1st year	1/1452 (<0.1%)	140812
	Overall	1/1452 (<0.1%)	140812

Table 14.3.1 / 10: Number of subjects with adverse events including subject identifier and year by treatment (FAS) (cont.)

TREATMENT: LCS16

Primary system organ class<br>Preferred term<br>MedDRA version 14.0	YEAR OF ONSET	N (%)	subject identifier
Viral upper respiratory tract infection	1st year	7/1452 (0.5%)	161401, 161402, 161404, 161415, 161430, 161432, 161434
	2nd year	9/1206 (0.7%)	161415, 161417, 161418, 161419, 161422, 161423, 161430, 161432, 243319
	3rd year	3/1012 (0.3%)	161417, 161432, 242310
	Overall	14/1452 (1.0%)	161401, 161402, 161404, 161415, 161417, 161418, 161419, 161422, 161423, 161430, 161432, 161434, 242310, 243319
Vulval abscess	1st year	1/1452 (<0.1%)	243932
	Overall	1/1452 (<0.1%)	243932
Vulvitis	1st year	5/1452 (0.3%)	150139, 150160, 160324, 160602, 244106
	2nd year	1/1206 (<0.1%)	120629
	3rd year	1/1012 (<0.1%)	150116
	Overall	7/1452 (0.5%)	120629, 150116, 150139, 150160, 160324, 160602, 244106

Table 14.3.1 / 10: Number of subjects with adverse events including subject identifier and year by treatment (FAS) (cont.)

TREATMENT: LCS16

Primary system organ class<br>Preferred term<br>MedDRA version 14.0	YEAR OF ONSET	N (%)	subject identifier
Vulvovaginal candidiasis	1st year	39/1452 (2.7%)	140223, 140801, 150137, 160128, 160316, 160324, 160326, 160332, 160438, 160602, 160608, 160907, 160912, 160969, 160972, 160987, 161201, 161536, 190227, 190408, 200701, 200702, 230106, 230121, 230213, 230516, 230918, 231004, 231010, 231011, 231110, 240506, 241537, 242707, 244525, 245002, 245030, 245526, 245927
	2nd year	30/1206 (2.5%)	140514, 140912, 160215, 160326, 160438, 160602, 160608, 160620, 160635, 160969, 160986, 160987, 161223, 161417, 161535, 190622, 200107, 200509, 200628, 230415, 230514, 230823, 231007, 231115, 243316, 244519, 244520, 245017, 245023, 245030
	3rd year	19/1012 (1.9%)	140223, 140509, 141035, 160128, 160210, 160531, 160563, 160602, 160907, 160920, 160942, 160969, 160986, 160987, 161524, 190408, 190604, 231115, 243813
	Overall	72/1452 (5.0%)	140223, 140509, 140514, 140801, 140912, 141035, 150137, 160128, 160210, 160215, 160316, 160324, 160326, 160332, 160438, 160531, 160563, 160602, 160608, 160620, 160635, 160907, 160912, 160920, 160942, 160969, 160972, 160986, 160987, 161201, 161223, 161417, 161524, 161535, 161536, 190227, 190408, 190604, 190622, 200107, 200509, 200628, 200701, 200702, 230106, 230121, 230213, 230415, 230514, 230516, 230823, 230918, 231004, 231007, 231010, 231011, 231110, 231115, 240506, 241537, 242707, 243316, 243813, 244519, 244520, 244525, 245002, 245017, 245023, 245030, 245526, 245927

Table 14.3.1 / 10: Number of subjects with adverse events including subject identifier and year by treatment (FAS) (cont.)

TREATMENT: LCS16

Primary system organ class<br>Preferred term<br>MedDRA version 14.0	YEAR OF ONSET	N (%)	subject identifier
Vulvovaginal mycotic infection	1st year	67/1452 (4.6%)	120308, 140112, 150103, 150113, 150115, 150120, 150126, 150131, 150137, 150146, 150157, 150164, 160503, 160526, 160529, 160533, 160540, 160634, 160707, 160714, 160718, 160727, 160737, 160745, 160748,<br>160755, 160761, 161008, 161311, 170104, 180660, 210110, 230306, 240603, 240605, 240636, 240905, 241155, 241301, 241310, 241417, 241508, 241546, 241726, 242324, 242904, 243008, 243024, 243025, 243301,<br>243504, 243507, 243518, 243703, 243707, 243963, 244106, 244123, 244128, 244418, 244435, 244446, 244505, 244614, 245409, 245416, 245927
	2nd year	40/1206 (3.3%)	140611, 150103, 150154, 150157, 160529, 160531, 160532, 160540, 160702, 160716, 160760, 161008, 161016, 170602, 210132, 240355, 240636, 240840, 241029, 241139, 241310, 241417, 241726, 242324, 242501,<br>242502, 242918, 243301, 243306, 243615, 244202, 244429, 244435, 244520, 244801, 244936, 245513, 245517, 245522, 245918
	3rd year	31/1012 (3.1%)	141411, 150114, 150115, 150117, 150126, 150137, 150139, 150159, 150160, 160529, 160540, 160702, 160755, 160760, 240614, 240626, 240636, 240711, 240905, 241026, 241139, 241418, 241546, 241713, 242501,<br>243301, 243937, 244435, 244802, 244936, 245908
	Overall	110/1452 (7.6%)	120308, 140112, 140611, 141411, 150103, 150113, 150114, 150115, 150117, 150120, 150126, 150131, 150137, 150139, 150146, 150154, 150157, 150159, 150160, 150164, 160503, 160526, 160529, 160531, 160532,<br>160533, 160540, 160634, 160702, 160707, 160714, 160716, 160718, 160727, 160737, 160745, 160748, 160755, 160760, 160761, 161008, 161016, 161311, 170104, 170602, 180660, 210110, 210132, 230306, 240355,<br>240603, 240605, 240614, 240626, 240636, 240711, 240840, 240905, 241026, 241029, 241139, 241155, 241301, 241310, 241417, 241418, 241508, 241546, 241713, 241726, 242324, 242501, 242502, 242904, 242918,

Table 14.3.1 / 10: Number of subjects with adverse events including subject identifier and year by treatment (FAS) (cont.)

TREATMENT: LCS16

Primary system organ class<br>Preferred term<br>MedDRA version 14.0	YEAR OF ONSET	N (%)	subject identifier
			243008, 243024, 243025, 243301, 243306, 243504, 243507, 243518, 243615, 243703, 243707, 243937, 243963, 244106, 244123, 244128, 244202, 244418, 244429, 244435, 244446, 244505, 244520, 244614, 244801, 244802, 244936, 245409, 245416, 245513, 245517, 245522, 245908, 245918, 245927
Vulvovaginitis	1st year	5/1452 (0.3%)	120402, 120603, 140801, 150204, 180334
	2nd year	1/1206 (<0.1%)	160324
	3rd year	2/1012 (0.2%)	160324, 241714
	Overall	7/1452 (0.5%)	120402, 120603, 140801, 150204, 160324, 180334, 241714
Vulvovaginitis chlamydial	2nd year	1/1206 (<0.1%)	245513
	Overall	1/1452 (<0.1%)	245513
Vulvovaginitis streptococcal	1st year	1/1452 (<0.1%)	245023
	3rd year	1/1012 (<0.1%)	244936
	Overall	2/1452 (0.1%)	244936, 245023
Vulvovaginitis trichomonal	1st year	2/1452 (0.1%)	246113, 246127
	2nd year	1/1206 (<0.1%)	243006
	3rd year	1/1012 (<0.1%)	246134
	Overall	4/1452 (0.3%)	243006, 246113, 246127, 246134
Wound infection	1st year	1/1452 (<0.1%)	161118
	Overall	1/1452 (<0.1%)	161118
Wound infection staphylococcal	3rd year	1/1012 (<0.1%)	230106
	Overall	1/1452 (<0.1%)	230106
Injury, poisoning and procedural complications	1st year	88/1452 (6.1%)	
	2nd year	29/1206 (2.4%)	
	3rd year	30/1012 (3.0%)	
	Overall	133/1452 (9.2%)	

Table 14.3.1 / 10: Number of subjects with adverse events including subject identifier and year by treatment (FAS) (cont.)

TREATMENT: LCS16

Primary system organ class<br>Preferred term<br>MedDRA version 14.0	YEAR OF ONSET	N (%)	subject identifier
Abdominal wound dehiscence	3rd year	1/1012 (<0.1%)	242321
	Overall	1/1452 (<0.1%)	242321
Accident	2nd year	1/1206 (<0.1%)	200701
	Overall	1/1452 (<0.1%)	200701
Animal bite	1st year	1/1452 (<0.1%)	200702
	2nd year	1/1206 (<0.1%)	200702
	3rd year	1/1012 (<0.1%)	161430
	Overall	2/1452 (0.1%)	161430, 200702
Ankle fracture	1st year	1/1452 (<0.1%)	161302
	2nd year	1/1206 (<0.1%)	140702
	Overall	2/1452 (0.1%)	140702, 161302
Arthropod bite	1st year	2/1452 (0.1%)	160611, 160907
	Overall	2/1452 (0.1%)	160611, 160907
Arthropod sting	1st year	1/1452 (<0.1%)	161524
	3rd year	1/1012 (<0.1%)	120612
	Overall	2/1452 (0.1%)	120612, 161524
Back injury	2nd year	1/1206 (<0.1%)	244305
	Overall	1/1452 (<0.1%)	244305
Burns second degree	2nd year	1/1206 (<0.1%)	246127
	Overall	1/1452 (<0.1%)	246127
Cartilage injury	2nd year	1/1206 (<0.1%)	241117
	Overall	1/1452 (<0.1%)	241117
Concussion	1st year	6/1452 (0.4%)	140601, 140810, 161107, 200225, 230807, 241301
	2nd year	1/1206 (<0.1%)	160428
	3rd year	1/1012 (<0.1%)	161404
	Overall	8/1452 (0.6%)	140601, 140810, 160428, 161107, 161404, 200225, 230807, 241301

Table 14.3.1 / 10: Number of subjects with adverse events including subject identifier and year by treatment (FAS) (cont.)

TREATMENT: LCS16

Primary system organ class<br>Preferred term<br>MedDRA version 14.0	YEAR OF ONSET	N (%)	subject identifier
Contusion	1st year	2/1452 (0.1%)	230514, 240501
	2nd year	2/1206 (0.2%)	161125, 241418
	3rd year	1/1012 (<0.1%)	200605
	Overall	5/1452 (0.3%)	161125, 200605, 230514, 240501, 241418
Epicondylitis	1st year	3/1452 (0.2%)	141012, 161529, 161535
	2nd year	1/1206 (<0.1%)	200525
	Overall	4/1452 (0.3%)	141012, 161529, 161535, 200525
Excoriation	1st year	1/1452 (<0.1%)	246001
	Overall	1/1452 (<0.1%)	246001
Eye injury	1st year	1/1452 (<0.1%)	243216
	Overall	1/1452 (<0.1%)	243216
Fall	3rd year	1/1012 (<0.1%)	190611
	Overall	1/1452 (<0.1%)	190611
Foot fracture	1st year	3/1452 (0.2%)	140225, 180659, 242317
	Overall	3/1452 (0.2%)	140225, 180659, 242317
Hand fracture	2nd year	1/1206 (<0.1%)	161309
	3rd year	2/1012 (0.2%)	160312, 160737
	Overall	3/1452 (0.2%)	160312, 160737, 161309
Head injury	3rd year	1/1012 (<0.1%)	140804
	Overall	1/1452 (<0.1%)	140804
Humerus fracture	3rd year	1/1012 (<0.1%)	161536
	Overall	1/1452 (<0.1%)	161536
Injury	1st year	1/1452 (<0.1%)	140919
	Overall	1/1452 (<0.1%)	140919
Joint dislocation	1st year	1/1452 (<0.1%)	241170
	Overall	1/1452 (<0.1%)	241170

Table 14.3.1 / 10: Number of subjects with adverse events including subject identifier and year by treatment (FAS) (cont.)

TREATMENT: LCS16

Primary system organ class<br>Preferred term<br>MedDRA version 14.0	YEAR OF ONSET	N (%)	subject identifier
Joint injury	1st year	2/1452 (0.1%)	140915, 244517
	2nd year	1/1206 (<0.1%)	161107
	3rd year	3/1012 (0.3%)	120607, 161404, 161430
	Overall	6/1452 (0.4%)	120607, 140915, 161107, 161404, 161430, 244517
Joint sprain	1st year	3/1452 (0.2%)	160611, 161306, 245806
	3rd year	3/1012 (0.3%)	141030, 150165, 240708
	Overall	6/1452 (0.4%)	141030, 150165, 160611, 161306, 240708, 245806
Laceration	1st year	2/1452 (0.1%)	241206, 243215
	Overall	2/1452 (0.1%)	241206, 243215
Ligament rupture	1st year	1/1452 (<0.1%)	243928
	Overall	1/1452 (<0.1%)	243928
Ligament sprain	2nd year	1/1206 (<0.1%)	161535
	Overall	1/1452 (<0.1%)	161535
Limb injury	1st year	3/1452 (0.2%)	161422, 161432, 241731
	3rd year	1/1012 (<0.1%)	161417
	Overall	4/1452 (0.3%)	161417, 161422, 161432, 241731
Lumbar vertebral fracture	3rd year	1/1012 (<0.1%)	140805
	Overall	1/1452 (<0.1%)	140805
Muscle injury	1st year	1/1452 (<0.1%)	140211
	2nd year	1/1206 (<0.1%)	140106
	Overall	2/1452 (0.1%)	140106, 140211
Muscle rupture	2nd year	1/1206 (<0.1%)	245905
	Overall	1/1452 (<0.1%)	245905
Muscle strain	1st year	5/1452 (0.3%)	140810, 160737, 161430, 161529, 241722
	2nd year	1/1206 (<0.1%)	241418
	3rd year	4/1012 (0.4%)	140804, 240358, 240708, 242803
	Overall	10/1452 (0.7%)	140804, 140810, 160737, 161430, 161529, 240358, 240708, 241418, 241722, 242803

Table 14.3.1 / 10: Number of subjects with adverse events including subject identifier and year by treatment (FAS) (cont.)

TREATMENT: LCS16

Primary system organ class<br>Preferred term<br>MedDRA version 14.0	YEAR OF ONSET	N (%)	subject identifier
Post concussion syndrome	2nd year	1/1206 (<0.1%)	140810
	Overall	1/1452 (<0.1%)	140810
Post procedural haemorrhage	1st year	1/1452 (<0.1%)	160706
	3rd year	1/1012 (<0.1%)	200403
	Overall	2/1452 (0.1%)	160706, 200403
Post-traumatic pain	1st year	3/1452 (0.2%)	161314, 170703, 240333
	2nd year	1/1206 (<0.1%)	200701
	Overall	4/1452 (0.3%)	161314, 170703, 200701, 240333
Postoperative ileus	3rd year	1/1012 (<0.1%)	242321
	Overall	1/1452 (<0.1%)	242321
Procedural pain	1st year	43/1452 (3.0%)	141103, 160206, 160546, 160602, 160707, 160709, 160714, 160727, 160731, 160748, 160750, 160762, 161103, 161107, 161116, 161119, 161123, 161131, 161302, 161309, 161311, 161314, 161321, 170703, 200510, 200607, 200617, 210121, 210123, 230106, 230207, 230612, 230614, 240118, 240515, 240522, 240523, 241114, 241126, 241522, 241535, 242911, 244410
	2nd year	6/1206 (0.5%)	160556, 161131, 241603, 242303, 243205, 245517
	3rd year	7/1012 (0.7%)	160629, 200510, 240333, 240905, 242321, 243707, 245909
	Overall	54/1452 (3.7%)	141103, 160206, 160546, 160556, 160602, 160629, 160707, 160709, 160714, 160727, 160731, 160748, 160750, 160762, 161103, 161107, 161116, 161119, 161123, 161131, 161302, 161309, 161311, 161314, 161321, 170703, 200510, 200607, 200617, 210121, 210123, 230106, 230207, 230612, 230614, 240118, 240333, 240515, 240522, 240523, 240905, 241114, 241126, 241522, 241535, 241603, 242303, 242321, 242911, 243205, 243707, 244410, 245517, 245909
Radius fracture	1st year	1/1452 (<0.1%)	242702
	Overall	1/1452 (<0.1%)	242702

Table 14.3.1 / 10: Number of subjects with adverse events including subject identifier and year by treatment (FAS) (cont.)

TREATMENT: LCS16

Primary system organ class<br>Preferred term<br>MedDRA version 14.0	YEAR OF ONSET	N (%)	subject identifier
Rib fracture	1st year	1/1452 (<0.1%)	161019
	3rd year	1/1012 (<0.1%)	170701
	Overall	2/1452 (0.1%)	161019, 170701
Road traffic accident	3rd year	2/1012 (0.2%)	240358, 240708
	Overall	2/1452 (0.1%)	240358, 240708
Skeletal injury	3rd year	1/1012 (<0.1%)	240708
	Overall	1/1452 (<0.1%)	240708
Skin injury	2nd year	1/1206 (<0.1%)	161401
	Overall	1/1452 (<0.1%)	161401
Spinal column injury	1st year	2/1452 (0.1%)	140901, 150126
	Overall	2/1452 (0.1%)	140901, 150126
Stress fracture	2nd year	1/1206 (<0.1%)	160737
	Overall	1/1452 (<0.1%)	160737
Tendon injury	1st year	1/1452 (<0.1%)	243911
	Overall	1/1452 (<0.1%)	243911
Tendon rupture	1st year	1/1452 (<0.1%)	161223
	Overall	1/1452 (<0.1%)	161223
Thermal burn	2nd year	1/1206 (<0.1%)	161306
	3rd year	2/1012 (0.2%)	240708, 242104
	Overall	3/1452 (0.2%)	161306, 240708, 242104
Tibia fracture	2nd year	1/1206 (<0.1%)	161107
	Overall	1/1452 (<0.1%)	161107
Upper limb fracture	2nd year	1/1206 (<0.1%)	161536
	3rd year	1/1012 (<0.1%)	230119
	Overall	2/1452 (0.1%)	161536, 230119

Table 14.3.1 / 10: Number of subjects with adverse events including subject identifier and year by treatment (FAS) (cont.)

TREATMENT: LCS16

Primary system organ class<br>Preferred term<br>MedDRA version 14.0	YEAR OF ONSET	N (%)	subject identifier
Vulval laceration	3rd year	1/1012 (<0.1%)	245806
	Overall	1/1452 (<0.1%)	245806
Whiplash injury	1st year	2/1452 (0.1%)	140601, 243932
	2nd year	2/1206 (0.2%)	160314, 244315
	3rd year	1/1012 (<0.1%)	246131
	Overall	5/1452 (0.3%)	140601, 160314, 243932, 244315, 246131
Wrist fracture	1st year	1/1452 (<0.1%)	120603
	2nd year	1/1206 (<0.1%)	240805
	Overall	2/1452 (0.1%)	120603, 240805
Investigations	1st year	64/1452 (4.4%)	
	2nd year	42/1206 (3.5%)	
	3rd year	60/1012 (5.9%)	
	Overall	158/1452 (10.9%)	
Alanine aminotransferase increased	1st year	1/1452 (<0.1%)	245517
	2nd year	1/1206 (<0.1%)	230118
	3rd year	8/1012 (0.8%)	120232, 150139, 150164, 160526, 161118, 161314, 243977, 244317
	Overall	10/1452 (0.7%)	120232, 150139, 150164, 160526, 161118, 161314, 230118, 243977, 244317, 245517
Aspartate aminotransferase increased	2nd year	1/1206 (<0.1%)	230118
	3rd year	3/1012 (0.3%)	161314, 243977, 244317
	Overall	4/1452 (0.3%)	161314, 230118, 243977, 244317
Blood amylase increased	1st year	1/1452 (<0.1%)	180663
	Overall	1/1452 (<0.1%)	180663
Blood cholesterol increased	3rd year	1/1012 (<0.1%)	160417
	Overall	1/1452 (<0.1%)	160417
Blood iron decreased	1st year	1/1452 (<0.1%)	180663
	Overall	1/1452 (<0.1%)	180663

Table 14.3.1 / 10: Number of subjects with adverse events including subject identifier and year by treatment (FAS) (cont.)

TREATMENT: LCS16

Primary system organ class<br>Preferred term<br>MedDRA version 14.0	YEAR OF ONSET	N (%)	subject identifier
Blood potassium increased	1st year	1/1452 (<0.1%)	200236
	Overall	1/1452 (<0.1%)	200236
Blood pressure diastolic increased	1st year	1/1452 (<0.1%)	241501
	Overall	1/1452 (<0.1%)	241501
Blood pressure increased	3rd year	1/1012 (<0.1%)	120404
	Overall	1/1452 (<0.1%)	120404
Blood pressure systolic increased	2nd year	1/1206 (<0.1%)	231104
	Overall	1/1452 (<0.1%)	231104
Blood triglycerides increased	1st year	1/1452 (<0.1%)	190227
	3rd year	4/1012 (0.4%)	120333, 141111, 180434, 244305
	Overall	5/1452 (0.3%)	120333, 141111, 180434, 190227, 244305
Blood urine present	3rd year	1/1012 (<0.1%)	161320
	Overall	1/1452 (<0.1%)	161320
Chlamydia test positive	2nd year	1/1206 (<0.1%)	241418
	Overall	1/1452 (<0.1%)	241418
Gamma-glutamyltransferase increased	1st year	2/1452 (0.1%)	243824, 245517
	2nd year	1/1206 (<0.1%)	230118
	3rd year	10/1012 (1.0%)	120232, 150126, 150139, 150164, 161306, 190142, 243518, 243833, 245407, 245507
	Overall	13/1452 (0.9%)	120232, 150126, 150139, 150164, 161306, 190142, 230118, 243518, 243824, 243833, 245407, 245507, 245517
Gastric pH decreased	2nd year	1/1206 (<0.1%)	243928
	Overall	1/1452 (<0.1%)	243928
Glucose urine present	3rd year	1/1012 (<0.1%)	244305
	Overall	1/1452 (<0.1%)	244305
Haematocrit decreased	2nd year	1/1206 (<0.1%)	244808
	Overall	1/1452 (<0.1%)	244808

Table 14.3.1 / 10: Number of subjects with adverse events including subject identifier and year by treatment (FAS) (cont.)

TREATMENT: LCS16

Primary system organ class<br>Preferred term<br>MedDRA version 14.0	YEAR OF ONSET	N (%)	subject identifier
Haematocrit increased	3rd year	2/1012 (0.2%)	244416, 245522
	Overall	2/1452 (0.1%)	244416, 245522
Haemoglobin decreased	2nd year	2/1206 (0.2%)	160546, 244808
	3rd year	3/1012 (0.3%)	150131, 150141, 242321
	Overall	5/1452 (0.3%)	150131, 150141, 160546, 242321, 244808
Heart rate increased	1st year	1/1452 (<0.1%)	241401
	Overall	1/1452 (<0.1%)	241401
Hepatic enzyme increased	1st year	1/1452 (<0.1%)	245703
	Overall	1/1452 (<0.1%)	245703
High density lipoprotein decreased	1st year	1/1452 (<0.1%)	190227
	3rd year	5/1012 (0.5%)	150117, 150137, 150164, 160417, 190142
	Overall	6/1452 (0.4%)	150117, 150137, 150164, 160417, 190142, 190227
High density lipoprotein increased	3rd year	1/1012 (<0.1%)	150140
	Overall	1/1452 (<0.1%)	150140
Human papilloma virus test positive	1st year	2/1452 (0.1%)	240645, 243963
	2nd year	1/1206 (<0.1%)	200235
	3rd year	2/1012 (0.2%)	243017, 243401
	Overall	5/1452 (0.3%)	200235, 240645, 243017, 243401, 243963
Liver function test abnormal	1st year	1/1452 (<0.1%)	243217
	3rd year	1/1012 (<0.1%)	244424
	Overall	2/1452 (0.1%)	243217, 244424
Low density lipoprotein increased	1st year	1/1452 (<0.1%)	190627
	3rd year	1/1012 (<0.1%)	160417
	Overall	2/1452 (0.1%)	160417, 190627
Platelet count decreased	3rd year	3/1012 (0.3%)	160563, 241140, 244305
	Overall	3/1452 (0.2%)	160563, 241140, 244305
Protein total decreased	3rd year	1/1012 (<0.1%)	241138
	Overall	1/1452 (<0.1%)	241138

Table 14.3.1 / 10: Number of subjects with adverse events including subject identifier and year by treatment (FAS) (cont.)

TREATMENT: LCS16

Primary system organ class<br>Preferred term<br>MedDRA version 14.0	YEAR OF ONSET	N (%)	subject identifier
Protein urine present	3rd year	1/1012 (<0.1%)	241173
	Overall	1/1452 (<0.1%)	241173
Red blood cell count decreased	2nd year	1/1206 (<0.1%)	244808
	3rd year	1/1012 (<0.1%)	150157
	Overall	2/1452 (0.1%)	150157, 244808
Simplex virus test positive	1st year	1/1452 (<0.1%)	243934
	Overall	1/1452 (<0.1%)	243934
Smear cervix abnormal	1st year	7/1452 (0.5%)	140712, 160718, 180438, 180629, 241179, 242502, 246134
	2nd year	9/1206 (0.7%)	150115, 150160, 160202, 180660, 200621, 200907, 240523, 241608, 245429
	3rd year	12/1012 (1.2%)	180810, 200103, 200203, 200212, 200231, 200235, 200403, 200411, 240506, 240724, 240815, 244501
	Overall	28/1452 (1.9%)	140712, 150115, 150160, 160202, 160718, 180438, 180629, 180660, 180810, 200103, 200203, 200212, 200231, 200235, 200403, 200411, 200621, 200907, 240506, 240523, 240724, 240815, 241179, 241608, 242502, 244501, 245429, 246134
Transaminases increased	2nd year	1/1206 (<0.1%)	160513
	Overall	1/1452 (<0.1%)	160513
Urine leukocyte esterase positive	1st year	1/1452 (<0.1%)	241189
	Overall	1/1452 (<0.1%)	241189
Vitamin D decreased	3rd year	1/1012 (<0.1%)	241603
	Overall	1/1452 (<0.1%)	241603
Weight decreased	1st year	3/1452 (0.2%)	120635, 210110, 246207
	2nd year	3/1206 (0.2%)	140205, 200711, 242505
	3rd year	1/1012 (<0.1%)	200902
	Overall	7/1452 (0.5%)	120635, 140205, 200711, 200902, 210110, 242505, 246207

Table 14.3.1 / 10: Number of subjects with adverse events including subject identifier and year by treatment (FAS) (cont.)

TREATMENT: LCS16

Primary system organ class<br>Preferred term<br>MedDRA version 14.0	YEAR OF ONSET	N (%)	subject identifier
Weight increased	1st year	41/1452 (2.8%)	120303, 120324, 140112, 140120, 141410, 141414, 150113, 150131, 160621, 160627, 160728, 160748, 160762, 160802, 160902, 160958, 161214, 161216, 180429, 230516, 230910, 230911, 231013, 240802, 240823, 241103, 241137, 241155, 241189, 241430, 241503, 242702, 243301, 243901, 243967, 244110, 244113, 244433, 244922, 245513, 246219
	2nd year	22/1206 (1.8%)	120222, 120337, 141411, 150154, 160428, 230215, 230220, 230404, 240303, 240325, 240506, 241002, 241109, 241158, 241176, 241403, 241535, 241537, 242502, 245522, 245602, 246207
	3rd year	5/1012 (0.5%)	241194, 243017, 243928, 243966, 244916
	Overall	68/1452 (4.7%)	120222, 120303, 120324, 120337, 140112, 140120, 141410, 141411, 141414, 150113, 150131, 150154, 160428, 160621, 160627, 160728, 160748, 160762, 160802, 160902, 160958, 161214, 161216, 180429, 230215, 230220, 230404, 230516, 230910, 230911, 231013, 240303, 240325, 240506, 240802, 240823, 241002, 241103, 241109, 241137, 241155, 241158, 241176, 241189, 241194, 241403, 241430, 241503, 241535, 241537, 242502, 242702, 243017, 243301, 243901, 243928, 243966, 243967, 244110, 244113, 244433, 244916, 244922, 245513, 245522, 245602, 246207, 246219
White blood cell count decreased	3rd year	2/1012 (0.2%)	180407, 244429
	Overall	2/1452 (0.1%)	180407, 244429
White blood cell count increased	3rd year	2/1012 (0.2%)	160428, 190405
	Overall	2/1452 (0.1%)	160428, 190405
White blood cells urine positive	3rd year	2/1012 (0.2%)	241140, 241173
	Overall	2/1452 (0.1%)	241140, 241173
Metabolism and nutrition disorders	1st year	11/1452 (0.8%)	
	2nd year	4/1206 (0.3%)	
	3rd year	3/1012 (0.3%)	
	Overall	17/1452 (1.2%)	

Table 14.3.1 / 10: Number of subjects with adverse events including subject identifier and year by treatment (FAS) (cont.)

TREATMENT: LCS16

Primary system organ class<br>Preferred term<br>MedDRA version 14.0	YEAR OF ONSET	N (%)	subject identifier
Abnormal loss of weight	1st year	1/1452 (<0.1%)	141413
	Overall	1/1452 (<0.1%)	141413
Abnormal weight gain	1st year	1/1452 (<0.1%)	240614
	Overall	1/1452 (<0.1%)	240614
Decreased appetite	1st year	1/1452 (<0.1%)	140810
	Overall	1/1452 (<0.1%)	140810
Dehydration	1st year	1/1452 (<0.1%)	245505
	3rd year	1/1012 (<0.1%)	242918
	Overall	2/1452 (0.1%)	242918, 245505
Diabetic ketoacidosis	2nd year	1/1206 (<0.1%)	141413
	Overall	1/1452 (<0.1%)	141413
Dyslipidaemia	3rd year	1/1012 (<0.1%)	150122
	Overall	1/1452 (<0.1%)	150122
Fluid retention	1st year	2/1452 (0.1%)	210413, 230315
	Overall	2/1452 (0.1%)	210413, 230315
Increased appetite	1st year	2/1452 (0.1%)	210110, 244113
	Overall	2/1452 (0.1%)	210110, 244113
Insulin resistance	2nd year	1/1206 (<0.1%)	150154
	Overall	1/1452 (<0.1%)	150154
Obesity	1st year	1/1452 (<0.1%)	244410
	Overall	1/1452 (<0.1%)	244410
Overweight	1st year	1/1452 (<0.1%)	160718
	Overall	1/1452 (<0.1%)	160718
Vitamin B12 deficiency	2nd year	1/1206 (<0.1%)	240603
	Overall	1/1452 (<0.1%)	240603

Table 14.3.1 / 10: Number of subjects with adverse events including subject identifier and year by treatment (FAS) (cont.)

TREATMENT: LCS16

Primary system organ class<br>Preferred term<br>MedDRA version 14.0	YEAR OF ONSET	N (%)	subject identifier
Vitamin D deficiency	2nd year	1/1206 (<0.1%)	245703
	3rd year	1/1012 (<0.1%)	240325
	Overall	2/1452 (0.1%)	240325, 245703
Weight fluctuation	1st year	1/1452 (<0.1%)	230915
	Overall	1/1452 (<0.1%)	230915
Musculoskeletal and connective tissue disorders	1st year	109/1452 (7.5%)	
	2nd year	50/1206 (4.1%)	
	3rd year	55/1012 (5.4%)	
	Overall	187/1452 (12.9%)	
Arthralgia	1st year	19/1452 (1.3%)	120614, 140722, 160620, 160745, 160747, 161306, 161417, 210105, 240524, 240645, 240803, 241237, 241301, 242813, 243211, 244521, 244808, 245505, 246201
	2nd year	8/1206 (0.7%)	140119, 141420, 160202, 160219, 160419, 160629, 240240, 244935
	3rd year	5/1012 (0.5%)	120614, 141411, 160630, 210605, 244902
	Overall	31/1452 (2.1%)	120614, 140119, 140722, 141411, 141420, 160202, 160219, 160419, 160620, 160629, 160630, 160745, 160747, 161306, 161417, 210105, 210605, 240240, 240524, 240645, 240803, 241237, 241301, 242813, 243211, 244521, 244808, 244902, 244935, 245505, 246201
Arthritis	1st year	1/1452 (<0.1%)	180659
	2nd year	2/1206 (0.2%)	120635, 240741
	3rd year	2/1012 (0.2%)	140909, 161315
	Overall	5/1452 (0.3%)	120635, 140909, 161315, 180659, 240741
Axillary mass	2nd year	1/1206 (<0.1%)	160556
	Overall	1/1452 (<0.1%)	160556

Table 14.3.1 / 10: Number of subjects with adverse events including subject identifier and year by treatment (FAS) (cont.)

TREATMENT: LCS16

Primary system organ class<br>Preferred term<br>MedDRA version 14.0	YEAR OF ONSET	N (%)	subject identifier
Back pain	1st year	41/1452 (2.8%)	120333, 120336, 120618, 140805, 141023, 141410, 150161, 160202, 160210, 160221, 160307, 160513, 160602, 160608, 160618, 160621, 160719, 160727, 160802, 160808, 160934, 161204, 161309, 161401, 161535, 200633, 200701, 200902, 230215, 230513, 241109, 241219, 241301, 243228, 243402, 243960, 243963, 243964, 244406, 245505, 246127
	2nd year	15/1206 (1.2%)	120618, 140805, 160611, 160612, 160730, 160735, 160919, 161008, 161529, 200702, 230416, 240632, 243229, 244433, 245905
	3rd year	14/1012 (1.4%)	120311, 120314, 160602, 160919, 161314, 161430, 230513, 240703, 241401, 241546, 242918, 243954, 245522, 246134
	Overall	65/1452 (4.5%)	120311, 120314, 120333, 120336, 120618, 140805, 141023, 141410, 150161, 160202, 160210, 160221, 160307, 160513, 160602, 160608, 160611, 160612, 160618, 160621, 160719, 160727, 160730, 160735, 160802, 160808, 160919, 160934, 161008, 161204, 161309, 161314, 161401, 161430, 161529, 161535, 200633, 200701, 200702, 200902, 230215, 230416, 230513, 240632, 240703, 241109, 241219, 241301, 241401, 241546, 242918, 243228, 243229, 243402, 243954, 243960, 243963, 243964, 244406, 244433, 245505, 245522, 245905, 246127, 246134
Bone pain	1st year	1/1452 (<0.1%)	244113
	3rd year	2/1012 (0.2%)	120311, 200920
	Overall	3/1452 (0.2%)	120311, 200920, 244113
Bursitis	1st year	1/1452 (<0.1%)	240905
	Overall	1/1452 (<0.1%)	240905
Compartment syndrome	2nd year	1/1206 (<0.1%)	161432
	Overall	1/1452 (<0.1%)	161432
Costochondritis	3rd year	1/1012 (<0.1%)	140812
	Overall	1/1452 (<0.1%)	140812

Table 14.3.1 / 10: Number of subjects with adverse events including subject identifier and year by treatment (FAS) (cont.)

TREATMENT: LCS16

Primary system organ class<br>Preferred term<br>MedDRA version 14.0	YEAR OF ONSET	N (%)	subject identifier
Exostosis	1st year	1/1452 (<0.1%)	243501
	2nd year	2/1206 (0.2%)	240626, 243928
	Overall	3/1452 (0.2%)	240626, 243501, 243928
Fibromyalgia	2nd year	1/1206 (<0.1%)	161004
	3rd year	1/1012 (<0.1%)	240325
	Overall	2/1452 (0.1%)	161004, 240325
Flank pain	1st year	2/1452 (0.1%)	161302, 241401
	3rd year	1/1012 (<0.1%)	160611
	Overall	3/1452 (0.2%)	160611, 161302, 241401
Groin pain	1st year	2/1452 (0.1%)	230609, 244933
	3rd year	2/1012 (0.2%)	120226, 160987
	Overall	4/1452 (0.3%)	120226, 160987, 230609, 244933
Hand deformity	1st year	1/1452 (<0.1%)	241237
	Overall	1/1452 (<0.1%)	241237
Intervertebral disc disorder	1st year	1/1452 (<0.1%)	161321
	Overall	1/1452 (<0.1%)	161321
Intervertebral disc protrusion	2nd year	2/1206 (0.2%)	120426, 245707
	3rd year	1/1012 (<0.1%)	140106
	Overall	3/1452 (0.2%)	120426, 140106, 245707
Joint instability	2nd year	1/1206 (<0.1%)	140704
	3rd year	1/1012 (<0.1%)	140704
	Overall	1/1452 (<0.1%)	140704
Joint stiffness	2nd year	1/1206 (<0.1%)	245905
	Overall	1/1452 (<0.1%)	245905
Joint swelling	1st year	1/1452 (<0.1%)	161432
	Overall	1/1452 (<0.1%)	161432
Loose body in joint	2nd year	1/1206 (<0.1%)	160419
	Overall	1/1452 (<0.1%)	160419

Table 14.3.1 / 10: Number of subjects with adverse events including subject identifier and year by treatment (FAS) (cont.)

TREATMENT: LCS16

Primary system organ class / Preferred term / MedDRA version 14.0	YEAR OF ONSET	N (%)	subject identifier
Medial tibial stress syndrome	2nd year	1/1206 (<0.1%)	160936
	3rd year	1/1012 (<0.1%)	161306
	Overall	2/1452 (0.1%)	160936, 161306
Muscle contracture	1st year	1/1452 (<0.1%)	120415
	2nd year	1/1206 (<0.1%)	120609
	Overall	2/1452 (0.1%)	120415, 120609
Muscle spasms	1st year	6/1452 (0.4%)	241173, 241403, 242104, 243949, 243963, 245507
	3rd year	2/1012 (0.2%)	161116, 241401
	Overall	8/1452 (0.6%)	161116, 241173, 241401, 241403, 242104, 243949, 243963, 245507
Muscle tightness	1st year	4/1452 (0.3%)	160627, 161004, 161020, 161309
	2nd year	1/1206 (<0.1%)	161004
	3rd year	6/1012 (0.6%)	160441, 160709, 160752, 161125, 161302, 244619
	Overall	10/1452 (0.7%)	160441, 160627, 160709, 160752, 161004, 161020, 161125, 161302, 161309, 244619
Musculoskeletal chest pain	1st year	2/1452 (0.1%)	243216, 245511
	Overall	2/1452 (0.1%)	243216, 245511
Musculoskeletal discomfort	2nd year	1/1206 (<0.1%)	244902
	Overall	1/1452 (<0.1%)	244902
Musculoskeletal pain	1st year	7/1452 (0.5%)	160622, 161128, 161201, 161314, 161404, 161429, 200920
	2nd year	4/1206 (0.3%)	161430, 180112, 200920, 243223
	3rd year	1/1012 (<0.1%)	200702
	Overall	11/1452 (0.8%)	160622, 161128, 161201, 161314, 161404, 161429, 161430, 180112, 200702, 200920, 243223
Musculoskeletal stiffness	2nd year	1/1206 (<0.1%)	245905
	Overall	1/1452 (<0.1%)	245905

Table 14.3.1 / 10: Number of subjects with adverse events including subject identifier and year by treatment (FAS) (cont.)

TREATMENT: LCS16

Primary system organ class<br>Preferred term<br>MedDRA version 14.0	YEAR OF ONSET	N (%)	subject identifier
Myalgia	1st year	8/1452 (0.6%)	140119, 160562, 200902, 210103, 210601, 243902, 244106, 245917
	2nd year	2/1206 (0.2%)	230514, 230915
	3rd year	2/1012 (0.2%)	160510, 242321
	Overall	12/1452 (0.8%)	140119, 160510, 160562, 200902, 210103, 210601, 230514, 230915, 242321, 243902, 244106, 245917
Myositis	1st year	1/1452 (<0.1%)	230807
	Overall	1/1452 (<0.1%)	230807
Neck pain	1st year	6/1452 (0.4%)	120618, 140523, 160755, 242317, 242801, 243960
	2nd year	2/1206 (0.2%)	120336, 150126
	3rd year	2/1012 (0.2%)	120318, 240703
	Overall	10/1452 (0.7%)	120318, 120336, 120618, 140523, 150126, 160755, 240703, 242317, 242801, 243960
Osteoarthritis	1st year	2/1452 (0.1%)	160921, 240325
	Overall	2/1452 (0.1%)	160921, 240325
Osteochondrosis	3rd year	2/1012 (0.2%)	161417, 240708
	Overall	2/1452 (0.1%)	161417, 240708
Osteopenia	2nd year	1/1206 (<0.1%)	160219
	Overall	1/1452 (<0.1%)	160219
Pain in extremity	1st year	8/1452 (0.6%)	160703, 160808, 210105, 241301, 243936, 244519, 244933, 245505
	2nd year	3/1206 (0.2%)	241170, 244305, 245011
	3rd year	4/1012 (0.4%)	161025, 161432, 241555, 244923
	Overall	15/1452 (1.0%)	160703, 160808, 161025, 161432, 210105, 241170, 241301, 241555, 243936, 244305, 244519, 244923, 244933, 245011, 245505
Pain in jaw	3rd year	1/1012 (<0.1%)	160324
	Overall	1/1452 (<0.1%)	160324
Plantar fasciitis	1st year	1/1452 (<0.1%)	241310
	Overall	1/1452 (<0.1%)	241310

Table 14.3.1 / 10: Number of subjects with adverse events including subject identifier and year by treatment (FAS) (cont.)

TREATMENT: LCS16

Primary system organ class<br>Preferred term<br>MedDRA version 14.0	YEAR OF ONSET	N (%)	subject identifier
Rotator cuff syndrome	1st year	1/1452 (<0.1%)	161535
	2nd year	3/1206 (0.2%)	160978, 241117, 243928
	3rd year	2/1012 (0.2%)	160628, 160632
	Overall	6/1452 (0.4%)	160628, 160632, 160978, 161535, 241117, 243928
Sensation of heaviness	3rd year	1/1012 (<0.1%)	120617
	Overall	1/1452 (<0.1%)	120617
Temporomandibular joint syndrome	3rd year	1/1012 (<0.1%)	140804
	Overall	1/1452 (<0.1%)	140804
Tendon pain	1st year	1/1452 (<0.1%)	160980
	Overall	1/1452 (<0.1%)	160980
Tendonitis	1st year	4/1452 (0.3%)	120404, 200902, 242814, 243801
	3rd year	1/1012 (<0.1%)	160936
	Overall	5/1452 (0.3%)	120404, 160936, 200902, 242814, 243801
Tenosynovitis	1st year	1/1452 (<0.1%)	161118
	3rd year	1/1012 (<0.1%)	161118
	Overall	1/1452 (<0.1%)	161118
Torticollis	2nd year	1/1206 (<0.1%)	160961
	3rd year	1/1012 (<0.1%)	150118
	Overall	2/1452 (0.1%)	150118, 160961
Neoplasms benign, malignant and unspecified (incl cysts and polyps)	1st year	17/1452 (1.2%)	
	2nd year	18/1206 (1.5%)	
	3rd year	10/1012 (1.0%)	
	Overall	43/1452 (3.0%)	
Acoustic neuroma	3rd year	1/1012 (<0.1%)	160561
	Overall	1/1452 (<0.1%)	160561

Table 14.3.1 / 10: Number of subjects with adverse events including subject identifier and year by treatment (FAS) (cont.)

TREATMENT: LCS16

Primary system organ class<br>Preferred term<br>MedDRA version 14.0	YEAR OF ONSET	N (%)	subject identifier
Acrochordon	1st year	1/1452 (<0.1%)	245704
	2nd year	1/1206 (<0.1%)	200529
	3rd year	1/1012 (<0.1%)	161417
	Overall	3/1452 (0.2%)	161417, 200529, 245704
Adenoma benign	2nd year	1/1206 (<0.1%)	242813
	Overall	1/1452 (<0.1%)	242813
Benign breast neoplasm	1st year	1/1452 (<0.1%)	230106
	Overall	1/1452 (<0.1%)	230106
Cervicitis human papilloma virus	1st year	1/1452 (<0.1%)	242314
	2nd year	3/1206 (0.2%)	120635, 190202, 241101
	Overall	4/1452 (0.3%)	120635, 190202, 241101, 242314
Cervix carcinoma stage 0	3rd year	1/1012 (<0.1%)	230309
	Overall	1/1452 (<0.1%)	230309
Enchondroma	2nd year	1/1206 (<0.1%)	160629
	Overall	1/1452 (<0.1%)	160629
Fibroadenoma of breast	1st year	1/1452 (<0.1%)	140607
	2nd year	1/1206 (<0.1%)	241412
	Overall	2/1452 (0.1%)	140607, 241412
Haemangioma of liver	2nd year	1/1206 (<0.1%)	241731
	Overall	1/1452 (<0.1%)	241731
Leiomyoma	2nd year	1/1206 (<0.1%)	243966
	Overall	1/1452 (<0.1%)	243966
Malignant melanoma	2nd year	1/1206 (<0.1%)	243229
	Overall	1/1452 (<0.1%)	243229
Melanocytic naevus	1st year	2/1452 (0.1%)	161432, 180663
	2nd year	1/1206 (<0.1%)	245905
	3rd year	1/1012 (<0.1%)	160517
	Overall	4/1452 (0.3%)	160517, 161432, 180663, 245905

Table 14.3.1 / 10: Number of subjects with adverse events including subject identifier and year by treatment (FAS) (cont.)

TREATMENT: LCS16

Primary system organ class<br>Preferred term<br>MedDRA version 14.0	YEAR OF ONSET	N (%)	subject identifier
Neurilemmoma	1st year	1/1452 (<0.1%)	160556
	Overall	1/1452 (<0.1%)	160556
Ovarian germ cell teratoma benign	1st year	3/1452 (0.2%)	180422, 200409, 241714
	Overall	3/1452 (0.2%)	180422, 200409, 241714
Ovarian neoplasm	1st year	2/1452 (0.1%)	160545, 180422
	Overall	2/1452 (0.1%)	160545, 180422
Pancreatic carcinoma	1st year	1/1452 (<0.1%)	210604
	Overall	1/1452 (<0.1%)	210604
Skin papilloma	1st year	1/1452 (<0.1%)	244433
	2nd year	1/1206 (<0.1%)	141025
	Overall	2/1452 (0.1%)	141025, 244433
Teratoma benign	2nd year	1/1206 (<0.1%)	190622
	Overall	1/1452 (<0.1%)	190622
Thyroid neoplasm	2nd year	1/1206 (<0.1%)	243016
	3rd year	1/1012 (<0.1%)	141425
	Overall	2/1452 (0.1%)	141425, 243016
Uterine leiomyoma	1st year	2/1452 (0.1%)	190603, 243001
	2nd year	2/1206 (0.2%)	190123, 200529
	3rd year	4/1012 (0.4%)	190123, 190603, 241546, 243918
	Overall	6/1452 (0.4%)	190123, 190603, 200529, 241546, 243001, 243918
Vulvovaginal human papilloma virus infection	1st year	2/1452 (0.1%)	140223, 230209
	2nd year	3/1206 (0.2%)	160761, 190142, 245517
	3rd year	1/1012 (<0.1%)	160762
	Overall	6/1452 (0.4%)	140223, 160761, 160762, 190142, 230209, 245517
Nervous system disorders	1st year	170/1452 (11.7%)	
	2nd year	48/1206 (4.0%)	
	3rd year	39/1012 (3.9%)	
	Overall	221/1452 (15.2%)	

Table 14.3.1 / 10: Number of subjects with adverse events including subject identifier and year by treatment (FAS) (cont.)

TREATMENT: LCS16

Primary system organ class<br>Preferred term<br>MedDRA version 14.0	YEAR OF ONSET	N (%)	subject identifier
Amnesia	2nd year	1/1206 (<0.1%)	241555
	Overall	1/1452 (<0.1%)	241555
Aphonia	1st year	1/1452 (<0.1%)	140101
	Overall	1/1452 (<0.1%)	140101
Carpal tunnel syndrome	1st year	4/1452 (0.3%)	160303, 160608, 243963, 245918
	2nd year	2/1206 (0.2%)	141411, 241533
	Overall	6/1452 (0.4%)	141411, 160303, 160608, 241533, 243963, 245918
Cervicobrachial syndrome	1st year	1/1452 (<0.1%)	241206
	Overall	1/1452 (<0.1%)	241206
Cluster headache	2nd year	1/1206 (<0.1%)	244010
	Overall	1/1452 (<0.1%)	244010
Convulsion	1st year	2/1452 (0.1%)	245407, 245905
	Overall	2/1452 (0.1%)	245407, 245905
Disturbance in attention	2nd year	1/1206 (<0.1%)	241420
	3rd year	1/1012 (<0.1%)	141304
	Overall	2/1452 (0.1%)	141304, 241420
Dizziness	1st year	14/1452 (1.0%)	120336, 120603, 120614, 120616, 140810, 141407, 200302, 210410, 230203, 240329, 242915, 243109, 243703, 245511
	2nd year	3/1206 (0.2%)	140112, 160543, 241726
	3rd year	5/1012 (0.5%)	150137, 161223, 190633, 230207, 245522
	Overall	22/1452 (1.5%)	120336, 120603, 120614, 120616, 140112, 140810, 141407, 150137, 160543, 161223, 190633, 200302, 210410, 230203, 230207, 240329, 241726, 242915, 243109, 243703, 245511, 245522

Table 14.3.1 / 10: Number of subjects with adverse events including subject identifier and year by treatment (FAS) (cont.)

TREATMENT: LCS16

Primary system organ class<br>Preferred term<br>MedDRA version 14.0	YEAR OF ONSET	N (%)	subject identifier
Headache	1st year	108/1452 (7.4%)	120101, 120313, 120328, 120334, 120336, 120404, 120406, 120603, 120606, 120607, 120609, 120612, 120615, 120618, 120622, 120627, 120629, 120631, 120635, 140120, 140504, 140508, 140523, 140601, 140603, 140801, 140810, 150161, 160124, 160303, 160324, 160438, 160504, 160508, 160553, 160602, 160603, 160612, 160618, 160621, 160627, 160628, 160629, 160634, 160635, 160636, 160727, 160737, 160966, 161016, 161128, 161216, 161302, 161306, 161309, 161311, 161315, 161524, 161526, 161528, 161535, 170701, 180310, 180625, 190620, 200508, 200510, 200620, 200628, 200917, 200926, 210405, 210408, 210413, 210601, 230305, 230306, 230601, 230608, 230610, 230614, 230705, 230811, 230905, 230916, 241002, 241145, 241179, 241418, 241503, 241529, 241537, 241801, 242306, 243606, 243707, 243719, 243909, 243934, 243954, 243966, 244113, 244114, 244410, 244517, 244703, 245707, 245908
	2nd year	28/1206 (2.3%)	120303, 120410, 120425, 120603, 120612, 120618, 160602, 160627, 160728, 161116, 161307, 161524, 161526, 161535, 190603, 190622, 190624, 230306, 230610, 230811, 242104, 242310, 242912, 243703, 244113, 244123, 244516, 245905
	3rd year	23/1012 (2.3%)	120102, 120302, 120303, 120311, 120337, 120410, 120612, 160602, 160608, 160716, 161223, 161526, 190214, 190603, 190633, 210405, 240127, 242104, 243703, 243964, 244517, 244805, 244902
	Overall	137/1452 (9.4%)	120101, 120102, 120302, 120303, 120311, 120313, 120328, 120334, 120336, 120337, 120404, 120406, 120410, 120425, 120603, 120606, 120607, 120609, 120612, 120615, 120618, 120622, 120627, 120629, 120631, 120635, 140120, 140504, 140508, 140523, 140601, 140603, 140801, 140810, 150161, 160124, 160303, 160324, 160438, 160504, 160508, 160553, 160602, 160603, 160608, 160612, 160618, 160621, 160627, 160628,

Table 14.3.1 / 10: Number of subjects with adverse events including subject identifier and year by treatment (FAS) (cont.)

TREATMENT: LCS16

Primary system organ class<br>Preferred term<br>MedDRA version 14.0	YEAR OF ONSET	N (%)	subject identifier
			160629, 160634, 160635, 160636, 160716, 160727, 160728, 160737, 160966, 161016, 161116, 161128, 161216, 161223, 161302, 161306, 161307, 161309, 161311, 161315, 161524, 161526, 161528, 161535, 170701, 180310, 180625, 190214, 190603, 190620, 190622, 190624, 190633, 200508, 200510, 200620, 200628, 200917, 200926, 210405, 210408, 210413, 210601, 230305, 230306, 230601, 230608, 230610, 230614, 230705, 230811, 230905, 230916, 240127, 241002, 241145, 241179, 241418, 241503, 241529, 241537, 241801, 242104, 242306, 242310, 242912, 243606, 243703, 243707, 243719, 243909, 243934, 243954, 243964, 243966, 244113, 244114, 244123, 244410, 244516, 244517, 244703, 244805, 244902, 245707, 245905, 245908
Hypoaesthesia	1st year	2/1452 (0.1%)	230305, 241173
	2nd year	1/1206 (<0.1%)	240220
	3rd year	1/1012 (<0.1%)	230220
	Overall	4/1452 (0.3%)	230220, 230305, 240220, 241173
Loss of consciousness	1st year	1/1452 (<0.1%)	245511
	Overall	1/1452 (<0.1%)	245511

Table 14.3.1 / 10: Number of subjects with adverse events including subject identifier and year by treatment (FAS) (cont.)

TREATMENT: LCS16

Primary system organ class<br>Preferred term<br>MedDRA version 14.0	YEAR OF ONSET	N (%)	subject identifier
Migraine	1st year	25/1452 (1.7%)	140207, 140227, 140514, 140526, 140801, 141415, 160314, 160429, 160502, 160513, 160731, 160752, 160802, 161005, 161101, 161118, 200707, 210128, 230402, 230705, 230823, 243406, 243957, 243966, 245009
	2nd year	8/1206 (0.7%)	160936, 161526, 180659, 180706, 200632, 210405, 230305, 245905
	3rd year	6/1012 (0.6%)	140804, 160629, 160948, 161101, 161429, 244410
	Overall	38/1452 (2.6%)	140207, 140227, 140514, 140526, 140801, 140804, 141415, 160314, 160429, 160502, 160513, 160629, 160731, 160752, 160802, 160936, 160948, 161005, 161101, 161118, 161429, 161526, 180659, 180706, 200632, 200707, 210128, 210405, 230305, 230402, 230705, 230823, 243406, 243957, 243966, 244410, 245009, 245905
Migraine with aura	1st year	1/1452 (<0.1%)	161107
	3rd year	1/1012 (<0.1%)	244619
	Overall	2/1452 (0.1%)	161107, 244619
Muscle contractions involuntary	1st year	1/1452 (<0.1%)	242104
	Overall	1/1452 (<0.1%)	242104
Nervous system disorder	1st year	1/1452 (<0.1%)	200525
	Overall	1/1452 (<0.1%)	200525
Paraesthesia	1st year	4/1452 (0.3%)	161008, 200917, 230215, 230305
	3rd year	1/1012 (<0.1%)	240127
	Overall	5/1452 (0.3%)	161008, 200917, 230215, 230305, 240127
Presyncope	1st year	8/1452 (0.6%)	140513, 160912, 160932, 210212, 230319, 240506, 241802, 244922
	3rd year	1/1012 (<0.1%)	210405
	Overall	9/1452 (0.6%)	140513, 160912, 160932, 210212, 210405, 230319, 240506, 241802, 244922
Sciatica	1st year	3/1452 (0.2%)	160504, 160612, 180659
	Overall	3/1452 (0.2%)	160504, 160612, 180659

Table 14.3.1 / 10: Number of subjects with adverse events including subject identifier and year by treatment (FAS) (cont.)

TREATMENT: LCS16

Primary system organ class<br>Preferred term<br>MedDRA version 14.0	YEAR OF ONSET	N (%)	subject identifier
Sinus headache	2nd year	1/1206 (<0.1%)	244516
	3rd year	1/1012 (<0.1%)	245507
	Overall	2/1452 (0.1%)	244516, 245507
Somnolence	2nd year	1/1206 (<0.1%)	244902
	Overall	1/1452 (<0.1%)	244902
Spinal cord herniation	2nd year	1/1206 (<0.1%)	200114
	Overall	1/1452 (<0.1%)	200114
Syncope	1st year	4/1452 (0.3%)	140810, 240221, 241301, 243905
	3rd year	1/1012 (<0.1%)	210405
	Overall	5/1452 (0.3%)	140810, 210405, 240221, 241301, 243905
Tension headache	1st year	4/1452 (0.3%)	140818, 161105, 161320, 243229
	2nd year	3/1206 (0.2%)	150121, 243316, 244616
	3rd year	3/1012 (0.3%)	161105, 161107, 241555
	Overall	9/1452 (0.6%)	140818, 150121, 161105, 161107, 161320, 241555, 243229, 243316, 244616
Pregnancy, puerperium and perinatal conditions	1st year	3/1452 (0.2%)	
	2nd year	3/1206 (0.2%)	
	3rd year	4/1012 (0.4%)	
	Overall	10/1452 (0.7%)	
Abortion spontaneous incomplete	2nd year	1/1206 (<0.1%)	180112
	Overall	1/1452 (<0.1%)	180112
Blighted ovum	3rd year	1/1012 (<0.1%)	160927
	Overall	1/1452 (<0.1%)	160927
Ectopic pregnancy	1st year	2/1452 (0.1%)	160423, 243228
	2nd year	2/1206 (0.2%)	180317, 200609
	3rd year	2/1012 (0.2%)	243932, 244519
	Overall	6/1452 (0.4%)	160423, 180317, 200609, 243228, 243932, 244519
Ruptured ectopic pregnancy	3rd year	1/1012 (<0.1%)	242321
	Overall	1/1452 (<0.1%)	242321

Table 14.3.1 / 10: Number of subjects with adverse events including subject identifier and year by treatment (FAS) (cont.)

TREATMENT: LCS16

Primary system organ class<br>Preferred term<br>MedDRA version 14.0	YEAR OF ONSET	N (%)	subject identifier
Uterine contractions abnormal	1st year	1/1452 (<0.1%)	230309
	Overall	1/1452 (<0.1%)	230309
Psychiatric disorders	1st year	98/1452 (6.7%)	
	2nd year	53/1206 (4.4%)	
	3rd year	37/1012 (3.7%)	
	Overall	172/1452 (11.8%)	
Acute stress disorder	3rd year	1/1012 (<0.1%)	200513
	Overall	1/1452 (<0.1%)	200513
Affect lability	1st year	1/1452 (<0.1%)	243823
	Overall	1/1452 (<0.1%)	243823
Affective disorder	2nd year	1/1206 (<0.1%)	244424
	Overall	1/1452 (<0.1%)	244424
Alcohol abuse	2nd year	1/1206 (<0.1%)	161214
	Overall	1/1452 (<0.1%)	161214
Alcoholism	2nd year	1/1206 (<0.1%)	244424
	Overall	1/1452 (<0.1%)	244424
Anxiety	1st year	17/1452 (1.2%)	140805, 141304, 150137, 160612, 200220, 240329, 240717, 241155, 241170, 241705, 242811, 243319, 243909, 243963, 243967, 244619, 244703
	2nd year	12/1206 (1.0%)	140720, 161311, 180720, 240703, 241403, 241420, 243001, 243507, 243966, 244424, 244519, 245513
	3rd year	10/1012 (1.0%)	140106, 140601, 140805, 141415, 240626, 241308, 241419, 243319, 243504, 245427
	Overall	37/1452 (2.5%)	140106, 140601, 140720, 140805, 141304, 141415, 150137, 160612, 161311, 180720, 200220, 240329, 240626, 240703, 240717, 241155, 241170, 241308, 241403, 241419, 241420, 241705, 242811, 243001, 243319, 243504, 243507, 243909, 243963, 243966, 243967, 244424, 244519, 244619, 244703, 245427, 245513

Table 14.3.1 / 10: Number of subjects with adverse events including subject identifier and year by treatment (FAS) (cont.)

TREATMENT: LCS16

Primary system organ class<br>Preferred term<br>MedDRA version 14.0	YEAR OF ONSET	N (%)	subject identifier
Anxiety disorder	1st year	2/1452 (0.1%)	150120, 244305
	2nd year	1/1206 (<0.1%)	230801
	3rd year	1/1012 (<0.1%)	140913
	Overall	4/1452 (0.3%)	140913, 150120, 230801, 244305
Attention deficit/hyperactivity disorder	1st year	3/1452 (0.2%)	240706, 246135, 246219
	2nd year	1/1206 (<0.1%)	243902
	Overall	4/1452 (0.3%)	240706, 243902, 246135, 246219
Bipolar disorder	1st year	1/1452 (<0.1%)	244317
	2nd year	2/1206 (0.2%)	243966, 244424
	3rd year	3/1012 (0.3%)	240502, 240807, 245908
	Overall	6/1452 (0.4%)	240502, 240807, 243966, 244317, 244424, 245908
Bulimia nervosa	1st year	1/1452 (<0.1%)	161502
	Overall	1/1452 (<0.1%)	161502
Burnout syndrome	1st year	1/1452 (<0.1%)	140106
	2nd year	1/1206 (<0.1%)	160747
	Overall	2/1452 (0.1%)	140106, 160747
Completed suicide	3rd year	1/1012 (<0.1%)	210112
	Overall	1/1452 (<0.1%)	210112
Depressed mood	1st year	3/1452 (0.2%)	160513, 210122, 243601
	Overall	3/1452 (0.2%)	160513, 210122, 243601

Table 14.3.1 / 10: Number of subjects with adverse events including subject identifier and year by treatment (FAS) (cont.)

TREATMENT: LCS16

Primary system organ class<br>Preferred term<br>MedDRA version 14.0	YEAR OF ONSET	N (%)	subject identifier
Depression	1st year	26/1452 (1.8%)	141111, 160969, 161417, 161502, 170803, 200515, 200907, 210121, 230305, 230905, 240128, 240501, 241197, 241219, 241418, 241801, 243225, 243306, 243319, 243508, 243956, 244126, 244305, 244432, 245035, 245905
	2nd year	14/1206 (1.2%)	141415, 150103, 150121, 150137, 160529, 200632, 230319, 231113, 241194, 241403, 243225, 243819, 243902, 243937
	3rd year	12/1012 (1.2%)	120615, 120616, 160562, 161302, 161432, 161538, 190214, 200711, 240523, 243225, 243306, 244130
	Overall	49/1452 (3.4%)	120615, 120616, 141111, 141415, 150103, 150121, 150137, 160529, 160562, 160969, 161302, 161417, 161432, 161502, 161538, 170803, 190214, 200515, 200632, 200711, 200907, 210121, 230305, 230319, 230905, 231113, 240128, 240501, 240523, 241194, 241197, 241219, 241403, 241418, 241801, 243225, 243306, 243319, 243508, 243819, 243902, 243937, 243956, 244126, 244130, 244305, 244432, 245035, 245905
Disorientation	1st year	1/1452 (<0.1%)	160403
	Overall	1/1452 (<0.1%)	160403
Drug dependence	2nd year	1/1206 (<0.1%)	244424
	Overall	1/1452 (<0.1%)	244424
Fear	1st year	1/1452 (<0.1%)	200510
	Overall	1/1452 (<0.1%)	200510

Table 14.3.1 / 10: Number of subjects with adverse events including subject identifier and year by treatment (FAS) (cont.)

TREATMENT: LCS16

Primary system organ class<br>Preferred term<br>MedDRA version 14.0	YEAR OF ONSET	N (%)	subject identifier
Insomnia	1st year	17/1452 (1.2%)	120612, 140103, 140106, 140810, 140920, 141012, 141425, 160123, 160611, 161213, 241418, 242324, 243228, 244202, 244424, 244432, 245504
	2nd year	10/1206 (0.8%)	140101, 140810, 140901, 140902, 160512, 161214, 161311, 210413, 241419, 241420
	3rd year	4/1012 (0.4%)	161213, 161302, 240325, 241403
	Overall	29/1452 (2.0%)	120612, 140101, 140103, 140106, 140810, 140901, 140902, 140920, 141012, 141425, 160123, 160512, 160611, 161213, 161214, 161302, 161311, 210413, 240325, 241403, 241418, 241419, 241420, 242324, 243228, 244202, 244424, 244432, 245504
Libido decreased	1st year	16/1452 (1.1%)	120303, 120313, 160203, 160703, 160723, 160755, 161510, 230601, 230916, 240221, 240645, 242811, 243303, 243976, 244002, 244451
	2nd year	8/1206 (0.7%)	120406, 150120, 150122, 161433, 180641, 230909, 240504, 243215
	3rd year	1/1012 (<0.1%)	241176
	Overall	25/1452 (1.7%)	120303, 120313, 120406, 150120, 150122, 160203, 160703, 160723, 160755, 161433, 161510, 180641, 230601, 230909, 230916, 240221, 240504, 240645, 241176, 242811, 243215, 243303, 243976, 244002, 244451
Libido increased	1st year	1/1452 (<0.1%)	140810
	2nd year	1/1206 (<0.1%)	230402
	Overall	2/1452 (0.1%)	140810, 230402
Loss of libido	1st year	1/1452 (<0.1%)	161538
	2nd year	1/1206 (<0.1%)	200632
	Overall	2/1452 (0.1%)	161538, 200632
Mental status changes	1st year	1/1452 (<0.1%)	161510
	Overall	1/1452 (<0.1%)	161510

Table 14.3.1 / 10: Number of subjects with adverse events including subject identifier and year by treatment (FAS) (cont.)

TREATMENT: LCS16

Primary system organ class<br>Preferred term<br>MedDRA version 14.0	YEAR OF ONSET	N (%)	subject identifier
Mood altered	1st year	8/1452 (0.6%)	120404, 141304, 160723, 230609, 230815, 231021, 240221, 245035
	2nd year	2/1206 (0.2%)	120318, 230909
	Overall	10/1452 (0.7%)	120318, 120404, 141304, 160723, 230609, 230815, 230909, 231021, 240221, 245035
Mood swings	1st year	8/1452 (0.6%)	200630, 200926, 240626, 242321, 243901, 244110, 244432, 244703
	3rd year	1/1012 (<0.1%)	241508
	Overall	9/1452 (0.6%)	200630, 200926, 240626, 241508, 242321, 243901, 244110, 244432, 244703
Nervousness	1st year	1/1452 (<0.1%)	161019
	2nd year	1/1206 (<0.1%)	120606
	Overall	2/1452 (0.1%)	120606, 161019
Obsessive-compulsive personality disorder	1st year	1/1452 (<0.1%)	240631
	Overall	1/1452 (<0.1%)	240631
Panic attack	1st year	1/1452 (<0.1%)	161019
	Overall	1/1452 (<0.1%)	161019
Panic disorder	1st year	1/1452 (<0.1%)	230601
	2nd year	1/1206 (<0.1%)	161108
	3rd year	3/1012 (0.3%)	160107, 160961, 161432
	Overall	5/1452 (0.3%)	160107, 160961, 161108, 161432, 230601
Phobia of flying	3rd year	1/1012 (<0.1%)	160636
	Overall	1/1452 (<0.1%)	160636
Polysubstance dependence	2nd year	1/1206 (<0.1%)	244424
	Overall	1/1452 (<0.1%)	244424
Post-traumatic stress disorder	2nd year	1/1206 (<0.1%)	141102
	Overall	1/1452 (<0.1%)	141102
Psychotic disorder	2nd year	1/1206 (<0.1%)	200621
	Overall	1/1452 (<0.1%)	200621

Table 14.3.1 / 10: Number of subjects with adverse events including subject identifier and year by treatment (FAS) (cont.)

TREATMENT: LCS16

Primary system organ class<br>Preferred term<br>MedDRA version 14.0	YEAR OF ONSET	N (%)	subject identifier
Sleep disorder	1st year	1/1452 (<0.1%)	161008
	2nd year	1/1206 (<0.1%)	160948
	Overall	2/1452 (0.1%)	160948, 161008
Social phobia	1st year	1/1452 (<0.1%)	240372
	Overall	1/1452 (<0.1%)	240372
Stress	2nd year	3/1206 (0.2%)	120609, 170703, 246115
	3rd year	1/1012 (<0.1%)	241111
	Overall	4/1452 (0.3%)	120609, 170703, 241111, 246115
Renal and urinary disorders	1st year	16/1452 (1.1%)	
	2nd year	8/1206 (0.7%)	
	3rd year	9/1012 (0.9%)	
	Overall	31/1452 (2.1%)	
Calculus urinary	1st year	1/1452 (<0.1%)	240803
	Overall	1/1452 (<0.1%)	240803
Chromaturia	1st year	1/1452 (<0.1%)	244128
	Overall	1/1452 (<0.1%)	244128
Dysuria	1st year	5/1452 (0.3%)	120207, 120606, 160426, 241540, 242207
	2nd year	2/1206 (0.2%)	241401, 244521
	Overall	7/1452 (0.5%)	120207, 120606, 160426, 241401, 241540, 242207, 244521
Haematuria	1st year	1/1452 (<0.1%)	241403
	3rd year	1/1012 (<0.1%)	240738
	Overall	2/1452 (0.1%)	240738, 241403
Hypertonic bladder	3rd year	2/1012 (0.2%)	160942, 245427
	Overall	2/1452 (0.1%)	160942, 245427
Micturition urgency	2nd year	2/1206 (0.2%)	160608, 244714
	3rd year	1/1012 (<0.1%)	241403
	Overall	3/1452 (0.2%)	160608, 241403, 244714

Table 14.3.1 / 10: Number of subjects with adverse events including subject identifier and year by treatment (FAS) (cont.)

TREATMENT: LCS16

Primary system organ class<br>Preferred term<br>MedDRA version 14.0	YEAR OF ONSET	N (%)	subject identifier
Nephrolithiasis	2nd year	2/1206 (0.2%)	180702, 244706
	3rd year	1/1012 (<0.1%)	245805
	Overall	3/1452 (0.2%)	180702, 244706, 245805
Pollakiuria	1st year	7/1452 (0.5%)	141415, 200906, 241540, 242104, 242207, 243902, 244120
	2nd year	1/1206 (<0.1%)	241008
	Overall	8/1452 (0.6%)	141415, 200906, 241008, 241540, 242104, 242207, 243902, 244120
Polyuria	1st year	1/1452 (<0.1%)	150154
	Overall	1/1452 (<0.1%)	150154
Renal cyst	3rd year	1/1012 (<0.1%)	241546
	Overall	1/1452 (<0.1%)	241546
Stress urinary incontinence	1st year	2/1452 (0.1%)	241243, 243902
	Overall	2/1452 (0.1%)	241243, 243902
Ureteric obstruction	1st year	1/1452 (<0.1%)	240803
	Overall	1/1452 (<0.1%)	240803
Urethral cyst	3rd year	1/1012 (<0.1%)	141413
	Overall	1/1452 (<0.1%)	141413
Urinary incontinence	2nd year	1/1206 (<0.1%)	120605
	3rd year	1/1012 (<0.1%)	120605
	Overall	1/1452 (<0.1%)	120605
Urinary retention	3rd year	1/1012 (<0.1%)	243937
	Overall	1/1452 (<0.1%)	243937
Urinary tract pain	1st year	1/1452 (<0.1%)	150121
	Overall	1/1452 (<0.1%)	150121
Urine odour abnormal	1st year	1/1452 (<0.1%)	244128
	Overall	1/1452 (<0.1%)	244128

Table 14.3.1 / 10: Number of subjects with adverse events including subject identifier and year by treatment (FAS) (cont.)

TREATMENT: LCS16

Primary system organ class<br>Preferred term<br>MedDRA version 14.0	YEAR OF ONSET	N (%)	subject identifier
Reproductive system and breast disorders	1st year	536/1452 (36.9%)	
	2nd year	257/1206 (21.3%)	
	3rd year	195/1012 (19.3%)	
	Overall	763/1452 (52.5%)	
Adnexa uteri pain	1st year	3/1452 (0.2%)	140810, 241010, 241711
	2nd year	2/1206 (0.2%)	180124, 244936
	Overall	5/1452 (0.3%)	140810, 180124, 241010, 241711, 244936
Amenorrhoea	1st year	2/1452 (0.1%)	180518, 190206
	2nd year	4/1206 (0.3%)	170602, 190201, 190214, 190604
	Overall	6/1452 (0.4%)	170602, 180518, 190201, 190206, 190214, 190604
Bartholin's cyst	3rd year	1/1012 (<0.1%)	120324
	Overall	1/1452 (<0.1%)	120324
Breast cyst	2nd year	2/1206 (0.2%)	160977, 241546
	Overall	2/1452 (0.1%)	160977, 241546
Breast discharge	1st year	5/1452 (0.3%)	241237, 242705, 243032, 243216, 244808
	2nd year	2/1206 (0.2%)	240212, 240235
	3rd year	3/1012 (0.3%)	160919, 180509, 240626
	Overall	10/1452 (0.7%)	160919, 180509, 240212, 240235, 240626, 241237, 242705, 243032, 243216, 244808
Breast discomfort	1st year	6/1452 (0.4%)	161201, 161216, 180139, 230402, 230505, 230516
	2nd year	1/1206 (<0.1%)	120318
	3rd year	1/1012 (<0.1%)	243215
	Overall	8/1452 (0.6%)	120318, 161201, 161216, 180139, 230402, 230505, 230516, 243215
Breast disorder female	2nd year	1/1206 (<0.1%)	245703
	Overall	1/1452 (<0.1%)	245703
Breast enlargement	1st year	2/1452 (0.1%)	140101, 200707
	2nd year	2/1206 (0.2%)	244916, 246218
	Overall	4/1452 (0.3%)	140101, 200707, 244916, 246218

Table 14.3.1 / 10: Number of subjects with adverse events including subject identifier and year by treatment (FAS) (cont.)

TREATMENT: LCS16

Primary system organ class<br>Preferred term<br>MedDRA version 14.0	YEAR OF ONSET	N (%)	subject identifier
Breast mass	1st year	2/1452 (0.1%)	230309, 241170
	2nd year	3/1206 (0.2%)	243306, 243318, 244704
	3rd year	2/1012 (0.2%)	210405, 243801
	Overall	7/1452 (0.5%)	210405, 230309, 241170, 243306, 243318, 243801, 244704
Breast pain	1st year	23/1452 (1.6%)	120107, 120402, 120424, 120617, 140513, 140526, 150126, 150150, 150154, 160628, 170601, 170604, 190122, 200218, 200236, 200701, 200704, 200707, 200920, 241237, 241503, 244406, 245504
	2nd year	11/1206 (0.9%)	120337, 140119, 140810, 160760, 161534, 170408, 170602, 190603, 200704, 242321, 244113
	3rd year	12/1012 (1.2%)	150120, 160314, 160316, 161009, 170703, 190123, 190603, 200509, 200605, 200920, 242324, 243014
	Overall	43/1452 (3.0%)	120107, 120337, 120402, 120424, 120617, 140119, 140513, 140526, 140810, 150120, 150126, 150150, 150154, 160314, 160316, 160628, 160760, 161009, 161534, 170408, 170601, 170602, 170604, 170703, 190122, 190123, 190603, 200218, 200236, 200509, 200605, 200701, 200704, 200707, 200920, 241237, 241503, 242321, 242324, 243014, 244113, 244406, 245504
Breast swelling	1st year	2/1452 (0.1%)	140112, 243228
	Overall	2/1452 (0.1%)	140112, 243228
Breast tenderness	1st year	22/1452 (1.5%)	120426, 120612, 140810, 141103, 160128, 160608, 160630, 160635, 160978, 161405, 241103, 241109, 241170, 241503, 242104, 242321, 242801, 243228, 244433, 244619, 244704, 245930
	2nd year	11/1206 (0.9%)	120402, 120404, 141108, 160128, 161534, 240802, 242502, 242912, 243402, 243957, 244916
	3rd year	4/1012 (0.4%)	120233, 160128, 160950, 240626
	Overall	35/1452 (2.4%)	120233, 120402, 120404, 120426, 120612, 140810, 141103, 141108, 160128, 160608, 160630, 160635, 160950, 160978, 161405, 161534, 240626, 240802, 241103, 241109, 241170, 241503, 242104, 242321, 242502, 242801, 242912, 243228, 243402, 243957, 244433, 244619, 244704, 244916, 245930

Table 14.3.1 / 10: Number of subjects with adverse events including subject identifier and year by treatment (FAS) (cont.)

TREATMENT: LCS16

Primary system organ class<br>Preferred term<br>MedDRA version 14.0	YEAR OF ONSET	N (%)	subject identifier
Cervical cyst	1st year	1/1452 (<0.1%)	190414
	Overall	1/1452 (<0.1%)	190414

Table 14.3.1 / 10: Number of subjects with adverse events including subject identifier and year by treatment (FAS) (cont.)

TREATMENT: LCS16

Primary system organ class<br>Preferred term<br>MedDRA version 14.0	YEAR OF ONSET	N (%)	subject identifier
Cervical dysplasia	1st year	24/1452 (1.7%)	120210, 120314, 140514, 141108, 160319, 160641, 160759, 160808, 170103, 170104, 180518, 180627, 190142, 210513, 230610, 230911, 231001, 240645, 241205, 241501, 243507, 243949, 243963, 246120
	2nd year	53/1206 (4.4%)	120408, 120426, 120635, 140509, 140512, 160326, 160332, 160523, 160752, 160902, 160961, 160986, 161020, 161223, 161321, 180413, 180417, 180517, 180521, 180723, 180737, 180738, 190624, 200215, 210103, 210601, 210604, 230516, 230810, 231011, 240208, 240212, 240220, 240605, 240620, 240807, 241031, 241101, 241230, 241412, 241543, 241709, 242211, 243014, 243316, 243507, 243703, 243813, 243949, 244926, 245513, 245918, 246127
	3rd year	48/1012 (4.7%)	120216, 120328, 120424, 120609, 140514, 140702, 140901, 150116, 160128, 160419, 160510, 160523, 160611, 160719, 160961, 180217, 180224, 180305, 180406, 180417, 180706, 180723, 200702, 200711, 210103, 210212, 210520, 230402, 230823, 231007, 231011, 231019, 240114, 240126, 240313, 240318, 240632, 240807, 241236, 241719, 243001, 243006, 243017, 243401, 243501, 245419, 245805, 246135
	Overall	115/1452 (7.9%)	120210, 120216, 120314, 120328, 120408, 120424, 120426, 120609, 120635, 140509, 140512, 140514, 140702, 140901, 141108, 150116, 160128, 160319, 160326, 160332, 160419, 160510, 160523, 160611, 160641, 160719, 160752, 160759, 160808, 160902, 160961, 160986, 161020, 161223, 161321, 170103, 170104, 180217, 180224, 180305, 180406, 180413, 180417, 180517, 180518, 180521, 180627, 180706, 180723, 180737, 180738, 190142, 190624, 200215, 200702, 200711, 210103, 210212, 210513, 210520, 210601, 210604, 230402, 230516, 230610, 230810, 230823, 230911, 231001, 231007, 231011, 231019, 240114, 240126, 240208,

Table 14.3.1 / 10: Number of subjects with adverse events including subject identifier and year by treatment (FAS) (cont.)

TREATMENT: LCS16

Primary system organ class<br>Preferred term<br>MedDRA version 14.0	YEAR OF ONSET	N (%)	subject identifier
			240212, 240220, 240313, 240318, 240605, 240620, 240632, 240645, 240807, 241031, 241101, 241205, 241230, 241236, 241412, 241501, 241543, 241709, 241719, 242211, 243001, 243006, 243014, 243017, 243316, 243401, 243501, 243507, 243703, 243813, 243949, 243963, 244926, 245419, 245513, 245805, 245918, 246120, 246127, 246135
Cervical friability	2nd year	1/1206 (<0.1%)	200632
	Overall	1/1452 (<0.1%)	200632
Cervical polyp	3rd year	2/1012 (0.2%)	200507, 244501
	Overall	2/1452 (0.1%)	200507, 244501
Cervix disorder	1st year	1/1452 (<0.1%)	160702
	Overall	1/1452 (<0.1%)	160702
Cervix erythema	1st year	1/1452 (<0.1%)	140818
	3rd year	1/1012 (<0.1%)	140805
	Overall	2/1452 (0.1%)	140805, 140818
Cervix haemorrhage uterine	1st year	1/1452 (<0.1%)	160562
	Overall	1/1452 (<0.1%)	160562
Cervix oedema	2nd year	1/1206 (<0.1%)	243017
	Overall	1/1452 (<0.1%)	243017
Coital bleeding	1st year	7/1452 (0.5%)	140112, 140504, 161009, 200529, 244435, 244802, 245507
	2nd year	4/1206 (0.3%)	160332, 200902, 230707, 245505
	3rd year	3/1012 (0.3%)	230717, 242811, 243229
	Overall	14/1452 (1.0%)	140112, 140504, 160332, 161009, 200529, 200902, 230707, 230717, 242811, 243229, 244435, 244802, 245505, 245507

Table 14.3.1 / 10: Number of subjects with adverse events including subject identifier and year by treatment (FAS) (cont.)

TREATMENT: LCS16

Primary system organ class<br>Preferred term<br>MedDRA version 14.0	YEAR OF ONSET	N (%)	subject identifier
Dysfunctional uterine bleeding	1st year	3/1452 (0.2%)	161405, 244113, 246102
	2nd year	1/1206 (<0.1%)	242317
	Overall	4/1452 (0.3%)	161405, 242317, 244113, 246102

Table 14.3.1 / 10: Number of subjects with adverse events including subject identifier and year by treatment (FAS) (cont.)

TREATMENT: LCS16

Primary system organ class<br>Preferred term<br>MedDRA version 14.0	YEAR OF ONSET	N (%)	subject identifier
Dysmenorrhoea	1st year	75/1452 (5.2%)	120309, 120313, 120402, 120606, 120609, 120612, 120614, 140214, 140818, 141420, 150103, 150147, 160107, 160303, 160307, 160314, 160332, 160417, 160603, 160706, 160723, 160760, 160802, 160920, 160929, 161019, 161108, 161118, 161123, 161131, 161315, 161321, 161415, 161434, 161502, 190206, 190227, 200103, 200513, 200609, 200617, 200620, 200630, 200632, 200930, 210102, 210113, 210115, 210133, 210605, 230319, 230402, 230614, 230708, 230811, 230815, 230820, 230823, 230916, 240618, 240633, 241555, 241711, 241716, 243819, 243827, 244130, 244406, 244410, 244429, 244517, 244609, 245412, 245515, 245909
	2nd year	26/1206 (2.2%)	120334, 120415, 140524, 140805, 160603, 160611, 160636, 160728, 160744, 160760, 161118, 161309, 161526, 190201, 190213, 200633, 200711, 210513, 242317, 242502, 242704, 243401, 243819, 243977, 244614, 246123
	3rd year	14/1012 (1.4%)	120328, 120334, 120616, 160744, 190227, 200509, 200510, 210405, 241546, 244401, 244437, 244805, 245507, 246120
	Overall	108/1452 (7.4%)	120309, 120313, 120328, 120334, 120402, 120415, 120606, 120609, 120612, 120614, 120616, 140214, 140524, 140805, 140818, 141420, 150103, 150147, 160107, 160303, 160307, 160314, 160332, 160417, 160603, 160611, 160636, 160706, 160723, 160728, 160744, 160760, 160802, 160920, 160929, 161019, 161108, 161118, 161123, 161131, 161309, 161315, 161321, 161415, 161434, 161502, 161526, 190201, 190206, 190213, 190227, 200103, 200509, 200510, 200513, 200609, 200617, 200620, 200630, 200632, 200633, 200711, 200930, 210102, 210113, 210115, 210133, 210405, 210513, 210605, 230319, 230402, 230614, 230708, 230811,

Table 14.3.1 / 10: Number of subjects with adverse events including subject identifier and year by treatment (FAS) (cont.)

TREATMENT: LCS16

Primary system organ class<br>Preferred term<br>MedDRA version 14.0	YEAR OF ONSET	N (%)	subject identifier
			230815, 230820, 230823, 230916, 240618, 240633, 241546, 241555, 241711, 241716, 242317, 242502, 242704, 243401, 243819, 243827, 243977, 244130, 244401, 244406, 244410, 244429, 244437, 244517, 244609,<br>244614, 244805, 245412, 245507, 245515, 245909, 246120, 246123
Dyspareunia	1st year	18/1452 (1.2%)	140112, 140805, 141111, 150102, 150150, 160719, 161432, 200707, 200906, 200917, 200928, 241522, 242317, 242817, 244106, 244505, 245030, 245513
	2nd year	6/1206 (0.5%)	120230, 140526, 190214, 190603, 230801, 242918
	3rd year	4/1012 (0.4%)	140804, 150114, 161530, 230106
	Overall	28/1452 (1.9%)	120230, 140112, 140526, 140804, 140805, 141111, 150102, 150114, 150150, 160719, 161432, 161530, 190214, 190603, 200707, 200906, 200917, 200928, 230106, 230801, 241522, 242317, 242817, 242918, 244106,<br>244505, 245030, 245513
Ectropion of cervix	1st year	1/1452 (<0.1%)	190109
	Overall	1/1452 (<0.1%)	190109
Endometriosis	1st year	1/1452 (<0.1%)	200606
	2nd year	1/1206 (<0.1%)	190622
	3rd year	1/1012 (<0.1%)	160533
	Overall	3/1452 (0.2%)	160533, 190622, 200606
Fibrocystic breast disease	1st year	1/1452 (<0.1%)	241524
	2nd year	3/1206 (0.2%)	241809, 242801, 244128
	3rd year	1/1012 (<0.1%)	241731
	Overall	5/1452 (0.3%)	241524, 241731, 241809, 242801, 244128
Galactorrhoea	1st year	5/1452 (0.3%)	190134, 241524, 244002, 244808, 245801
	2nd year	1/1206 (<0.1%)	160521
	3rd year	2/1012 (0.2%)	150120, 161432
	Overall	8/1452 (0.6%)	150120, 160521, 161432, 190134, 241524, 244002, 244808, 245801

Table 14.3.1 / 10: Number of subjects with adverse events including subject identifier and year by treatment (FAS) (cont.)

TREATMENT: LCS16

Primary system organ class<br>Preferred term<br>MedDRA version 14.0	YEAR OF ONSET	N (%)	subject identifier
Genital cyst	1st year	1/1452 (<0.1%)	180625
	Overall	1/1452 (<0.1%)	180625
Genital discharge	1st year	4/1452 (0.3%)	140810, 160521, 160570, 160703
	2nd year	5/1206 (0.4%)	140106, 150118, 150120, 190202, 190206
	Overall	9/1452 (0.6%)	140106, 140810, 150118, 150120, 160521, 160570, 160703, 190202, 190206
Genital rash	2nd year	1/1206 (<0.1%)	243504
	Overall	1/1452 (<0.1%)	243504
Haemorrhagic ovarian cyst	1st year	11/1452 (0.8%)	120101, 120102, 150121, 160612, 200902, 240524, 241030, 241509, 242702, 244606, 244926
	2nd year	4/1206 (0.3%)	120402, 240523, 243313, 245918
	3rd year	4/1012 (0.4%)	190114, 190134, 241705, 242803
	Overall	19/1452 (1.3%)	120101, 120102, 120402, 150121, 160612, 190114, 190134, 200902, 240523, 240524, 241030, 241509, 241705, 242702, 242803, 243313, 244606, 244926, 245918
Hypomenorrhoea	3rd year	1/1012 (<0.1%)	190212
	Overall	1/1452 (<0.1%)	190212
Menorrhagia	1st year	9/1452 (0.6%)	150147, 161402, 200609, 200630, 200917, 210102, 241179, 241501, 243944
	3rd year	1/1012 (<0.1%)	200301
	Overall	10/1452 (0.7%)	150147, 161402, 200301, 200609, 200630, 200917, 210102, 241179, 241501, 243944
Menstrual disorder	3rd year	1/1012 (<0.1%)	244437
	Overall	1/1452 (<0.1%)	244437
Menstruation irregular	1st year	3/1452 (0.2%)	160433, 200514, 243957
	Overall	3/1452 (0.2%)	160433, 200514, 243957

Table 14.3.1 / 10: Number of subjects with adverse events including subject identifier and year by treatment (FAS) (cont.)

TREATMENT: LCS16

Primary system organ class<br>Preferred term<br>MedDRA version 14.0	YEAR OF ONSET	N (%)	subject identifier
Metrorrhagia	1st year	10/1452 (0.7%)	120406, 120408, 150102, 161415, 170301, 210517, 230907, 242301, 243013, 243029
	2nd year	1/1206 (<0.1%)	245505
	3rd year	1/1012 (<0.1%)	244401
	Overall	12/1452 (0.8%)	120406, 120408, 150102, 161415, 170301, 210517, 230907, 242301, 243013, 243029, 244401, 245505
Nipple exudate bloody	3rd year	1/1012 (<0.1%)	161009
	Overall	1/1452 (<0.1%)	161009
Nipple pain	1st year	1/1452 (<0.1%)	141108
	2nd year	1/1206 (<0.1%)	160737
	Overall	2/1452 (0.1%)	141108, 160737
Oligomenorrhoea	1st year	2/1452 (0.1%)	245524, 245530
	Overall	2/1452 (0.1%)	245524, 245530

Table 14.3.1 / 10: Number of subjects with adverse events including subject identifier and year by treatment (FAS) (cont.)

TREATMENT: LCS16

Primary system organ class<br>Preferred term<br>MedDRA version 14.0	YEAR OF ONSET	N (%)	subject identifier
Ovarian cyst	1st year	204/1452 (14.0%)	120229, 120237, 120241, 120306, 120317, 120333, 120334, 120402, 120405, 120603, 120605, 120612, 120627, 120629, 120631, 140223, 140603, 140704, 140810, 140906, 140916, 141003, 150116, 150140, 150141, 150504, 160107, 160116, 160211, 160312, 160315, 160326, 160411, 160412, 160441, 160517, 160533, 160549, 160553, 160563, 160608, 160611, 160632, 160641, 160735, 160761, 160802, 160808, 160901, 160919, 160921, 160932, 160950, 161004, 161008, 161009, 161025, 161116, 161128, 161217, 161315, 161320, 161502, 161503, 161510, 161524, 170301, 170303, 170308, 170601, 170602, 180124, 180333, 180640, 180667, 180720, 180732, 190108, 190109, 190114, 190129, 190202, 190212, 190213, 190214, 190607, 190622, 190640, 200118, 200203, 200212, 200217, 200229, 200305, 200509, 200514, 200704, 200902, 210115, 210206, 230203, 230209, 230215, 230301, 230319, 230409, 230414, 230505, 230609, 230612, 230807, 230814, 230915, 231019, 231020, 231104, 231109, 240114, 240213, 240303, 240305, 240325, 240331, 240343, 240369, 240504, 240511, 240624, 240626, 240631, 240632, 240633, 240645, 240722, 240727, 240833, 240906, 241010, 241029, 241101, 241103, 241114, 241117, 241125, 241147, 241189, 241220, 241236, 241301, 241402, 241430, 241501, 241508, 241509, 241511, 241529, 241533, 241537, 241608, 241703, 241709, 241711, 241716, 241719, 241726, 242310, 242317, 242321, 242803, 242904, 242908, 242911, 243321, 243331, 243407, 243601, 243615, 243717, 243801, 243804, 243822, 243833, 243906, 243923, 243954, 243959, 244113, 244120, 244202, 244303, 244406, 244451, 244514, 244802, 244805, 244925, 245002, 245409, 245524, 245801, 245925, 245926, 246127, 246146

Table 14.3.1 / 10: Number of subjects with adverse events including subject identifier and year by treatment (FAS) (cont.)

TREATMENT: LCS16

Primary system organ class<br>Preferred term<br>MedDRA version 14.0	YEAR OF ONSET	N (%)	subject identifier
	2nd year	76/1206 (6.3%)	120102, 120107, 120410, 120426, 120605, 120617, 140110, 140120, 140704, 140902, 140906, 141411, 150201, 150211, 160533, 160545, 160549, 160608, 160628, 160642, 160703, 160745, 160956, 161025, 161306, 161320, 170604, 180212, 180431, 180732, 190108, 190113, 190212, 190408, 190607, 190640, 200215, 200217, 200301, 200410, 200507, 200707, 210410, 230204, 230211, 230414, 230705, 230707, 231113, 240106, 240240, 240523, 240840, 241020, 241030, 241117, 241145, 241155, 241197, 241230, 241550, 241705, 242310, 242324, 242904, 243815, 243827, 243911, 243957, 243960, 243971, 244446, 244936, 245908, 246123, 246127
	3rd year	71/1012 (7.0%)	120302, 120333, 120410, 120605, 120607, 120616, 140216, 141003, 150141, 150504, 160128, 160546, 160549, 160563, 160735, 160747, 161204, 161222, 161310, 161524, 161534, 170308, 180115, 180117, 180404, 190108, 190213, 190227, 190414, 190603, 190611, 190622, 200218, 200302, 200605, 200711, 200930, 210128, 230222, 230301, 230614, 230705, 230810, 240235, 240307, 240325, 240511, 241020, 241140, 241145, 241236, 241508, 241537, 241543, 241546, 241603, 241719, 242803, 242908, 243225, 243604, 243911, 243967, 244303, 244305, 245428, 245504, 245517, 245522, 246135, 246219
	Overall	304/1452 (20.9%)	120102, 120107, 120229, 120237, 120241, 120302, 120306, 120317, 120333, 120334, 120402, 120405, 120410, 120426, 120603, 120605, 120607, 120612, 120616, 120617, 120627, 120629, 120631, 140110, 140120, 140216, 140223, 140603, 140704, 140810, 140902, 140906, 140916, 141003, 141411, 150116, 150140, 150141, 150201, 150211, 150504, 160107, 160116, 160128, 160211, 160312, 160315, 160326, 160411, 160412,

Table 14.3.1 / 10: Number of subjects with adverse events including subject identifier and year by treatment (FAS) (cont.)

TREATMENT: LCS16

Primary system organ class<br>Preferred term<br>MedDRA version 14.0	YEAR OF ONSET	N (%)	subject identifier
			160441, 160517, 160533, 160545, 160546, 160549, 160553, 160563, 160608, 160611, 160628, 160632, 160641, 160642, 160703, 160735, 160745, 160747, 160761, 160802, 160808, 160901, 160919, 160921, 160932,
			160950, 160956, 161004, 161008, 161009, 161025, 161116, 161128, 161204, 161217, 161222, 161306, 161310, 161315, 161320, 161502, 161503, 161510, 161524, 161534, 170301, 170303, 170308, 170601, 170602,
			170604, 180115, 180117, 180124, 180212, 180333, 180404, 180431, 180640, 180667, 180720, 180732, 190108, 190109, 190113, 190114, 190129, 190202, 190212, 190213, 190214, 190227, 190408, 190414, 190603,
			190607, 190611, 190622, 190640, 200118, 200203, 200212, 200215, 200217, 200218, 200229, 200301, 200302, 200305, 200410, 200507, 200509, 200514, 200605, 200704, 200707, 200711, 200902, 200930, 210115,
			210128, 210206, 210410, 230203, 230204, 230209, 230211, 230215, 230222, 230301, 230319, 230409, 230414, 230505, 230609, 230612, 230614, 230705, 230707, 230807, 230810, 230814, 230915, 231019, 231020,
			231104, 231109, 231113, 240106, 240114, 240213, 240235, 240240, 240303, 240305, 240307, 240325, 240331, 240343, 240369, 240504, 240511, 240523, 240624, 240626, 240631, 240632, 240633, 240645, 240722,
			240727, 240833, 240840, 240906, 241010, 241020, 241029, 241030, 241101, 241103, 241114, 241117, 241125, 241140, 241145, 241147, 241155, 241189, 241197, 241220, 241230, 241236, 241301, 241402, 241430,
			241501, 241508, 241509, 241511, 241529, 241533, 241537, 241543, 241546, 241550, 241603, 241608, 241703, 241705, 241709, 241711, 241716, 241719, 241726, 242310, 242317, 242321, 242324, 242803, 242904,

Table 14.3.1 / 10: Number of subjects with adverse events including subject identifier and year by treatment (FAS) (cont.)

TREATMENT: LCS16

Primary system organ class<br>Preferred term<br>MedDRA version 14.0	YEAR OF ONSET	N (%)	subject identifier
			242908, 242911, 243225, 243321, 243331, 243407, 243601, 243604, 243615, 243717, 243801, 243804, 243815, 243822, 243827, 243833, 243906, 243911, 243923, 243954, 243957, 243959, 243960, 243967, 243971, 244113, 244120, 244202, 244303, 244305, 244406, 244446, 244451, 244514, 244802, 244805, 244925, 244936, 245002, 245409, 245428, 245504, 245517, 245522, 245524, 245801, 245908, 245925, 245926, 246123, 246127, 246135, 246146, 246219
Ovarian cyst ruptured	1st year	6/1452 (0.4%)	140801, 160211, 161217, 240133, 241511, 246135
	2nd year	1/1206 (<0.1%)	244936
	Overall	7/1452 (0.5%)	140801, 160211, 161217, 240133, 241511, 244936, 246135
Ovarian cyst torsion	1st year	1/1452 (<0.1%)	190202
	Overall	1/1452 (<0.1%)	190202
Ovarian disorder	3rd year	1/1012 (<0.1%)	160563
	Overall	1/1452 (<0.1%)	160563
Ovarian enlargement	2nd year	1/1206 (<0.1%)	244936
	Overall	1/1452 (<0.1%)	244936
Ovarian mass	2nd year	2/1206 (0.2%)	243815, 244406
	Overall	2/1452 (0.1%)	243815, 244406
Ovulation pain	1st year	2/1452 (0.1%)	230909, 244505
	Overall	2/1452 (0.1%)	230909, 244505
Pelvic adhesions	3rd year	1/1012 (<0.1%)	180117
	Overall	1/1452 (<0.1%)	180117
Pelvic discomfort	2nd year	1/1206 (<0.1%)	241008
	3rd year	1/1012 (<0.1%)	240303
	Overall	2/1452 (0.1%)	240303, 241008

Table 14.3.1 / 10: Number of subjects with adverse events including subject identifier and year by treatment (FAS) (cont.)

TREATMENT: LCS16

Primary system organ class Preferred term MedDRA version 14.0	YEAR OF ONSET	N (%)	subject identifier
Pelvic pain	1st year	97/1452 (6.7%)	120210, 120221, 120325, 120328, 120334, 120420, 120606, 120612, 120614, 120615, 120616, 120617, 120622, 120629, 140223, 141015, 150102, 150116, 150118, 150120, 150140, 150141, 150146, 150150, 150161, 150162, 150164, 150504, 160568, 160727, 160730, 160731, 160760, 160761, 160762, 161009, 161103, 161118, 161119, 161123, 161128, 161213, 161216, 161217, 161222, 161226, 170305, 170412, 170601, 200206, 200220, 200907, 200925, 200928, 210210, 230108, 230121, 230202, 230305, 230708, 230718, 230909, 240315, 240524, 240805, 240833, 241107, 241114, 241147, 241179, 241189, 241509, 241529, 241555, 242104, 242306, 242702, 242914, 242915, 243014, 243029, 243216, 243402, 243802, 243905, 243944, 244432, 244449, 244706, 244922, 244926, 245511, 245515, 245524, 246103, 246146, 246207
	2nd year	24/1206 (2.0%)	120328, 120617, 141007, 150141, 150201, 160716, 160730, 170412, 190603, 190604, 200515, 230705, 241020, 241139, 241509, 241726, 242104, 242904, 243957, 244429, 244936, 245918, 246201, 246207
	3rd year	12/1012 (1.2%)	160737, 160761, 161118, 180304, 242222, 242908, 243229, 244517, 245507, 246117, 246127, 246134
	Overall	123/1452 (8.5%)	120210, 120221, 120325, 120328, 120334, 120420, 120606, 120612, 120614, 120615, 120616, 120617, 120622, 120629, 140223, 141007, 141015, 150102, 150116, 150118, 150120, 150140, 150141, 150146, 150150, 150161, 150162, 150164, 150201, 150504, 160568, 160716, 160727, 160730, 160731, 160737, 160760, 160761, 160762, 161009, 161103, 161118, 161119, 161123, 161128, 161213, 161216, 161217, 161222, 161226, 170305, 170412, 170601, 180304, 190603, 190604, 200206, 200220, 200515, 200907, 200925, 200928, 210210, 230108, 230121, 230202, 230305, 230705, 230708, 230718, 230909, 240315, 240524, 240805, 240833,

Table 14.3.1 / 10: Number of subjects with adverse events including subject identifier and year by treatment (FAS) (cont.)

TREATMENT: LCS16

Primary system organ class<br>Preferred term<br>MedDRA version 14.0	YEAR OF ONSET	N (%)	subject identifier
			241020, 241107, 241114, 241139, 241147, 241179, 241189, 241509, 241529, 241555, 241726, 242104, 242222, 242306, 242702, 242904, 242908, 242914, 242915, 243014, 243029, 243216, 243229, 243402, 243802, 243905, 243944, 243957, 244429, 244432, 244449, 244517, 244706, 244922, 244926, 244936, 245507, 245511, 245515, 245524, 245918, 246103, 246117, 246127, 246134, 246146, 246201, 246207
Polycystic ovaries	1st year	1/1452 (<0.1%)	150504
	2nd year	1/1206 (<0.1%)	241722
	Overall	2/1452 (0.1%)	150504, 241722
Polymenorrhoea	2nd year	1/1206 (<0.1%)	240113
	Overall	1/1452 (<0.1%)	240113
Premenstrual syndrome	1st year	8/1452 (0.6%)	160921, 161019, 200507, 200522, 210605, 230309, 230911, 241537
	2nd year	2/1206 (0.2%)	160728, 244703
	3rd year	2/1012 (0.2%)	160942, 244437
	Overall	12/1452 (0.8%)	160728, 160921, 160942, 161019, 200507, 200522, 210605, 230309, 230911, 241537, 244437, 244703
Uterine haemorrhage	1st year	4/1452 (0.3%)	240501, 240515, 240730, 244802
	2nd year	2/1206 (0.2%)	170803, 246123
	3rd year	1/1012 (<0.1%)	240502
	Overall	7/1452 (0.5%)	170803, 240501, 240502, 240515, 240730, 244802, 246123
Uterine inflammation	2nd year	1/1206 (<0.1%)	180212
	Overall	1/1452 (<0.1%)	180212
Uterine pain	1st year	2/1452 (0.1%)	210122, 243963
	Overall	2/1452 (0.1%)	210122, 243963
Uterine polyp	3rd year	1/1012 (<0.1%)	160531
	Overall	1/1452 (<0.1%)	160531

Table 14.3.1 / 10: Number of subjects with adverse events including subject identifier and year by treatment (FAS) (cont.)

TREATMENT: LCS16

Primary system organ class<br>Preferred term<br>MedDRA version 14.0	YEAR OF ONSET	N (%)	subject identifier
Uterine spasm	1st year	37/1452 (2.5%)	161401, 161402, 161404, 161405, 161412, 161415, 161417, 161419, 161422, 161423, 161429, 161430, 161432, 161433, 161434, 170408, 200305, 210405, 230203, 241301, 241310, 242703, 242704, 242810, 242814, 243406, 243601, 243823, 243921, 243932, 244429, 244525, 244606, 244612, 244712, 244801, 244804
	3rd year	2/1012 (0.2%)	240235, 243407
	Overall	39/1452 (2.7%)	161401, 161402, 161404, 161405, 161412, 161415, 161417, 161419, 161422, 161423, 161429, 161430, 161432, 161433, 161434, 170408, 200305, 210405, 230203, 240235, 241301, 241310, 242703, 242704, 242810, 242814, 243406, 243407, 243601, 243823, 243921, 243932, 244429, 244525, 244606, 244612, 244712, 244801, 244804
Uterine tenderness	1st year	2/1452 (0.1%)	242915, 242918
	Overall	2/1452 (0.1%)	242915, 242918

Table 14.3.1 / 10: Number of subjects with adverse events including subject identifier and year by treatment (FAS) (cont.)

TREATMENT: LCS16

Primary system organ class<br>Preferred term<br>MedDRA version 14.0	YEAR OF ONSET	N (%)	subject identifier
Vaginal discharge	1st year	33/1452 (2.3%)	120102, 120226, 120302, 120306, 120317, 120336, 120402, 120405, 120607, 120614, 120616, 120618, 120627, 120631, 140610, 140720, 141002, 160916, 180133, 180663, 200206, 200226, 200801, 210105, 230513, 230814, 241529, 241801, 244106, 244128, 244202, 244614, 245018
	2nd year	24/1206 (2.0%)	120306, 120308, 120334, 120402, 120404, 120405, 120408, 120607, 120616, 120627, 160123, 161502, 180138, 200603, 240741, 241139, 241714, 241801, 243504, 243957, 244714, 244902, 245517, 246207
	3rd year	15/1012 (1.5%)	120226, 120325, 120404, 120405, 120614, 120631, 160916, 160942, 180113, 180117, 180138, 210413, 240502, 241508, 244123
	Overall	58/1452 (4.0%)	120102, 120226, 120302, 120306, 120308, 120317, 120325, 120334, 120336, 120402, 120404, 120405, 120408, 120607, 120614, 120616, 120618, 120627, 120631, 140610, 140720, 141002, 160123, 160916, 160942, 161502, 180113, 180117, 180133, 180138, 180663, 200206, 200226, 200603, 200801, 210105, 210413, 230513, 230814, 240502, 240741, 241139, 241508, 241529, 241714, 241801, 243504, 243957, 244106, 244123, 244128, 244202, 244614, 244714, 244902, 245018, 245517, 246207
Vaginal disorder	1st year	2/1452 (0.1%)	140810, 231010
	3rd year	2/1012 (0.2%)	150141, 230601
	Overall	4/1452 (0.3%)	140810, 150141, 230601, 231010

Table 14.3.1 / 10: Number of subjects with adverse events including subject identifier and year by treatment (FAS) (cont.)

TREATMENT: LCS16

Primary system organ class<br>Preferred term<br>MedDRA version 14.0	YEAR OF ONSET	N (%)	subject identifier
Vaginal haemorrhage	1st year	53/1452 (3.7%)	120308, 141407, 150162, 160303, 160307, 160502, 160503, 160634, 160641, 160703, 160718, 160723, 160727, 161505, 180105, 180202, 180309, 180314, 180326, 180807, 190206, 200507, 200806, 200926, 210105, 210110, 210115, 210117, 210133, 230110, 230612, 230909, 231013, 240211, 240324, 241107, 241171, 241218, 241501, 241603, 242306, 242312, 242508, 243228, 243601, 243719, 243905, 243959, 243975, 245002, 245031, 245429, 245925
	2nd year	15/1206 (1.2%)	150154, 160512, 160621, 160636, 160702, 160748, 200515, 200609, 230111, 241714, 241901, 242003, 243215, 244120, 245407
	3rd year	5/1012 (0.5%)	160529, 170301, 170303, 230416, 240626
	Overall	73/1452 (5.0%)	120308, 141407, 150154, 150162, 160303, 160307, 160502, 160503, 160512, 160529, 160621, 160634, 160636, 160641, 160702, 160703, 160718, 160723, 160727, 160748, 161505, 170301, 170303, 180105, 180202, 180309, 180314, 180326, 180807, 190206, 200507, 200515, 200609, 200806, 200926, 210105, 210110, 210115, 210117, 210133, 230110, 230111, 230416, 230612, 230909, 231013, 240211, 240324, 240626, 241107, 241171, 241218, 241501, 241603, 241714, 241901, 242003, 242306, 242312, 242508, 243215, 243228, 243601, 243719, 243905, 243959, 243975, 244120, 245002, 245031, 245407, 245429, 245925
Vaginal odour	1st year	5/1452 (0.3%)	160941, 241139, 241501, 243902, 244120
	2nd year	3/1206 (0.2%)	241139, 244120, 244133
	3rd year	1/1012 (<0.1%)	241420
	Overall	7/1452 (0.5%)	160941, 241139, 241420, 241501, 243902, 244120, 244133
Vaginal perforation	2nd year	1/1206 (<0.1%)	180521
	Overall	1/1452 (<0.1%)	180521

Table 14.3.1 / 10: Number of subjects with adverse events including subject identifier and year by treatment (FAS) (cont.)

TREATMENT: LCS16

Primary system organ class<br>Preferred term<br>MedDRA version 14.0	YEAR OF ONSET	N (%)	subject identifier
Vaginal ulceration	2nd year	1/1206 (<0.1%)	243303
	Overall	1/1452 (<0.1%)	243303
Vulvovaginal burning sensation	1st year	1/1452 (<0.1%)	160948
	2nd year	1/1206 (<0.1%)	244406
	3rd year	2/1012 (0.2%)	140804, 244113
	Overall	4/1452 (0.3%)	140804, 160948, 244113, 244406
Vulvovaginal discomfort	1st year	1/1452 (<0.1%)	241237
	2nd year	1/1206 (<0.1%)	244128
	Overall	2/1452 (0.1%)	241237, 244128
Vulvovaginal dryness	1st year	5/1452 (0.3%)	140801, 161509, 241546, 244106, 245409
	Overall	5/1452 (0.3%)	140801, 161509, 241546, 244106, 245409
Vulvovaginal erythema	1st year	1/1452 (<0.1%)	141002
	2nd year	1/1206 (<0.1%)	245020
	Overall	2/1452 (0.1%)	141002, 245020
Vulvovaginal pain	1st year	2/1452 (0.1%)	120306, 200214
	Overall	2/1452 (0.1%)	120306, 200214
Vulvovaginal pruritus	1st year	11/1452 (0.8%)	120615, 140810, 141414, 150150, 161415, 190114, 190207, 210105, 230823, 240128, 244709
	2nd year	4/1206 (0.3%)	120233, 141005, 150103, 190114
	3rd year	4/1012 (0.4%)	161417, 190207, 200712, 241176
	Overall	17/1452 (1.2%)	120233, 120615, 140810, 141005, 141414, 150103, 150150, 161415, 161417, 190114, 190207, 200712, 210105, 230823, 240128, 241176, 244709
Vulvovaginal swelling	2nd year	1/1206 (<0.1%)	190207
	Overall	1/1452 (<0.1%)	190207
Respiratory, thoracic and mediastinal disorders	1st year	40/1452 (2.8%)	
	2nd year	20/1206 (1.7%)	
	3rd year	18/1012 (1.8%)	
	Overall	70/1452 (4.8%)	

Table 14.3.1 / 10: Number of subjects with adverse events including subject identifier and year by treatment (FAS) (cont.)

TREATMENT: LCS16

Primary system organ class<br>Preferred term<br>MedDRA version 14.0	YEAR OF ONSET	N (%)	subject identifier
Allergic sinusitis	1st year	1/1452 (<0.1%)	241522
	Overall	1/1452 (<0.1%)	241522
Asthma	1st year	4/1452 (0.3%)	160629, 160808, 160950, 241418
	2nd year	4/1206 (0.3%)	160950, 161009, 161118, 244801
	3rd year	4/1012 (0.4%)	160324, 160919, 241412, 244802
	Overall	11/1452 (0.8%)	160324, 160629, 160808, 160919, 160950, 161009, 161118, 241412, 241418, 244801, 244802
Atelectasis	3rd year	1/1012 (<0.1%)	242321
	Overall	1/1452 (<0.1%)	242321
Bronchospasm	2nd year	1/1206 (<0.1%)	120609
	3rd year	1/1012 (<0.1%)	120617
	Overall	2/1452 (0.1%)	120609, 120617
Cough	1st year	11/1452 (0.8%)	160307, 160426, 160629, 161302, 190202, 210405, 210413, 230820, 230905, 242317, 244446
	2nd year	3/1206 (0.2%)	230905, 241420, 242908
	3rd year	1/1012 (<0.1%)	161302
	Overall	13/1452 (0.9%)	160307, 160426, 160629, 161302, 190202, 210405, 210413, 230820, 230905, 241420, 242317, 242908, 244446
Diaphragmatic hernia	1st year	1/1452 (<0.1%)	161008
	Overall	1/1452 (<0.1%)	161008
Dyspnoea	3rd year	2/1012 (0.2%)	160602, 241412
	Overall	2/1452 (0.1%)	160602, 241412
Epistaxis	3rd year	1/1012 (<0.1%)	243016
	Overall	1/1452 (<0.1%)	243016
Hyperventilation	1st year	1/1452 (<0.1%)	200922
	Overall	1/1452 (<0.1%)	200922
Hypoxia	3rd year	1/1012 (<0.1%)	241412
	Overall	1/1452 (<0.1%)	241412

Table 14.3.1 / 10: Number of subjects with adverse events including subject identifier and year by treatment (FAS) (cont.)

TREATMENT: LCS16

Primary system organ class<br>Preferred term<br>MedDRA version 14.0	YEAR OF ONSET	N (%)	subject identifier
Nasal congestion	1st year	1/1452 (<0.1%)	230305
	Overall	1/1452 (<0.1%)	230305
Nasal polyps	2nd year	1/1206 (<0.1%)	160735
	Overall	1/1452 (<0.1%)	160735
Oropharyngeal pain	1st year	10/1452 (0.7%)	120612, 120618, 140607, 160636, 170408, 170703, 230207, 240706, 241802, 242912
	2nd year	7/1206 (0.6%)	120424, 120609, 120612, 120631, 170703, 242908, 244517
	3rd year	2/1012 (0.2%)	231113, 241308
	Overall	17/1452 (1.2%)	120424, 120609, 120612, 120618, 120631, 140607, 160636, 170408, 170703, 230207, 231113, 240706, 241308, 241802, 242908, 242912, 244517
Pleural effusion	3rd year	1/1012 (<0.1%)	242321
	Overall	1/1452 (<0.1%)	242321
Pleurisy	1st year	1/1452 (<0.1%)	241709
	Overall	1/1452 (<0.1%)	241709
Pneumonitis	1st year	1/1452 (<0.1%)	120622
	Overall	1/1452 (<0.1%)	120622
Rhinitis allergic	1st year	7/1452 (0.5%)	150120, 150126, 150139, 160561, 161131, 230614, 241415
	2nd year	2/1206 (0.2%)	160723, 161223
	3rd year	4/1012 (0.4%)	150141, 160540, 160561, 230614
	Overall	11/1452 (0.8%)	150120, 150126, 150139, 150141, 160540, 160561, 160723, 161131, 161223, 230614, 241415
Rhinitis seasonal	1st year	2/1452 (0.1%)	243901, 243936
	Overall	2/1452 (0.1%)	243901, 243936
Sinus congestion	2nd year	2/1206 (0.2%)	242803, 242817
	Overall	2/1452 (0.1%)	242803, 242817

Table 14.3.1 / 10: Number of subjects with adverse events including subject identifier and year by treatment (FAS) (cont.)

TREATMENT: LCS16

Primary system organ class<br>Preferred term<br>MedDRA version 14.0	YEAR OF ONSET	N (%)	subject identifier
Sinus polyp	2nd year	1/1206 (<0.1%)	244429
	Overall	1/1452 (<0.1%)	244429
Sleep apnoea syndrome	3rd year	1/1012 (<0.1%)	245806
	Overall	1/1452 (<0.1%)	245806
Tonsillar disorder	3rd year	1/1012 (<0.1%)	140804
	Overall	1/1452 (<0.1%)	140804
Upper respiratory tract inflammation	1st year	1/1452 (<0.1%)	160934
	2nd year	1/1206 (<0.1%)	160986
	3rd year	1/1012 (<0.1%)	160986
	Overall	2/1452 (0.1%)	160934, 160986
Skin and subcutaneous tissue disorders	1st year	183/1452 (12.6%)	
	2nd year	65/1206 (5.4%)	
	3rd year	28/1012 (2.8%)	
	Overall	254/1452 (17.5%)	

Table 14.3.1 / 10: Number of subjects with adverse events including subject identifier and year by treatment (FAS) (cont.)

TREATMENT: LCS16

Primary system organ class<br>Preferred term<br>MedDRA version 14.0	YEAR OF ONSET	N (%)	subject identifier
Acne	1st year	128/1452 (8.8%)	120609, 140103, 140106, 140112, 140504, 140508, 140523, 140803, 141002, 141025, 141105, 141108, 141415, 150117, 150120, 150146, 150159, 160124, 160126, 160202, 160211, 160314, 160326, 160330, 160417, 160534, 160543, 160602, 160621, 160622, 160634, 160636, 160642, 160728, 160735, 160750, 160752, 160759, 160761, 160920, 160929, 160932, 160936, 160947, 160958, 160968, 160969, 160978, 160986, 161103, 161201, 161302, 161307, 161315, 161405, 161509, 161538, 170601, 170602, 170703, 180601, 180602, 180625, 180632, 180641, 180659, 180738, 190620, 190640, 200229, 200411, 200520, 200605, 200632, 200711, 210122, 210133, 210206, 230110, 230215, 230306, 230315, 230409, 230503, 230505, 230612, 230707, 230814, 230823, 230918, 231104, 240103, 240332, 240511, 240706, 240823, 241030, 241111, 241116, 241155, 241176, 241195, 241529, 241556, 242216, 242702, 243020, 243123, 243208, 243228, 243959, 244114, 244312, 244424, 244446, 244519, 244611, 244916, 244922, 244935, 244936, 245505, 245513, 245925, 245927, 245930, 246201, 246218
	2nd year	33/1206 (2.7%)	120404, 140514, 140702, 160114, 160403, 160413, 160531, 160907, 160970, 180608, 180622, 180628, 190641, 200632, 230305, 240741, 240810, 241310, 241403, 242104, 242904, 242912, 242918, 243023, 243402, 243928, 243936, 243957, 244606, 244714, 245522, 245909, 246201
	3rd year	13/1012 (1.3%)	120616, 160114, 160115, 161009, 161524, 200509, 240369, 240840, 241308, 241709, 242503, 243928, 245930
	Overall	169/1452 (11.6%)	120404, 120609, 120616, 140103, 140106, 140112, 140504, 140508, 140514, 140523, 140702, 140803, 141002, 141025, 141105, 141108, 141415, 150117, 150120, 150146, 150159, 160114, 160115, 160124, 160126,

Table 14.3.1 / 10: Number of subjects with adverse events including subject identifier and year by treatment (FAS) (cont.)

TREATMENT: LCS16

Primary system organ class<br>Preferred term<br>MedDRA version 14.0	YEAR OF ONSET	N (%)	subject identifier
			160202, 160211, 160314, 160326, 160330, 160403, 160413, 160417, 160531, 160534, 160543, 160602, 160621, 160622, 160634, 160636, 160642, 160728, 160735, 160750, 160752, 160759, 160761, 160907, 160920, 160929, 160932, 160936, 160947, 160958, 160968, 160969, 160970, 160978, 160986, 161009, 161103, 161201, 161302, 161307, 161315, 161405, 161509, 161524, 161538, 170601, 170602, 170703, 180601, 180602, 180608, 180622, 180625, 180628, 180632, 180641, 180659, 180738, 190620, 190640, 190641, 200229, 200411, 200509, 200520, 200605, 200632, 200711, 210122, 210133, 210206, 230110, 230215, 230305, 230306, 230315, 230409, 230503, 230505, 230612, 230707, 230814, 230823, 230918, 231104, 240103, 240332, 240369, 240511, 240706, 240741, 240810, 240823, 240840, 241030, 241111, 241116, 241155, 241176, 241195, 241308, 241310, 241403, 241529, 241556, 241709, 242104, 242216, 242503, 242702, 242904, 242912, 242918, 243020, 243023, 243123, 243208, 243228, 243402, 243928, 243936, 243957, 243959, 244114, 244312, 244424, 244446, 244519, 244606, 244611, 244714, 244916, 244922, 244935, 244936, 245505, 245513, 245522, 245909, 245925, 245927, 245930, 246201, 246218
Alopecia	1st year	10/1452 (0.7%)	160630, 180138, 180660, 200628, 200925, 230814, 241030, 241171, 241555, 244418
	2nd year	1/1206 (<0.1%)	180103
	3rd year	2/1012 (0.2%)	180683, 230207
	Overall	13/1452 (0.9%)	160630, 180103, 180138, 180660, 180683, 200628, 200925, 230207, 230814, 241030, 241171, 241555, 244418
Dandruff	1st year	1/1452 (<0.1%)	160642
	Overall	1/1452 (<0.1%)	160642

Table 14.3.1 / 10: Number of subjects with adverse events including subject identifier and year by treatment (FAS) (cont.)

TREATMENT: LCS16

Primary system organ class<br>Preferred term<br>MedDRA version 14.0	YEAR OF ONSET	N (%)	subject identifier
Dermatitis	1st year	5/1452 (0.3%)	120420, 160628, 161004, 190114, 230506
	2nd year	2/1206 (0.2%)	161429, 243318
	Overall	7/1452 (0.5%)	120420, 160628, 161004, 161429, 190114, 230506, 243318
Dermatitis allergic	1st year	1/1452 (<0.1%)	241155
	2nd year	2/1206 (0.2%)	160629, 161008
	3rd year	2/1012 (0.2%)	161118, 230822
	Overall	5/1452 (0.3%)	160629, 161008, 161118, 230822, 241155
Dermatitis atopic	1st year	3/1452 (0.2%)	150139, 150150, 242321
	2nd year	1/1206 (<0.1%)	242321
	3rd year	2/1012 (0.2%)	160752, 244202
	Overall	5/1452 (0.3%)	150139, 150150, 160752, 242321, 244202
Dermatitis contact	1st year	3/1452 (0.2%)	141411, 244517, 245913
	3rd year	2/1012 (0.2%)	243008, 245801
	Overall	5/1452 (0.3%)	141411, 243008, 244517, 245801, 245913
Dry skin	2nd year	1/1206 (<0.1%)	245927
	Overall	1/1452 (<0.1%)	245927
Eczema	1st year	4/1452 (0.3%)	140810, 160523, 161529, 210134
	2nd year	3/1206 (0.2%)	160115, 210134, 241420
	3rd year	2/1012 (0.2%)	160907, 230503
	Overall	8/1452 (0.6%)	140810, 160115, 160523, 160907, 161529, 210134, 230503, 241420
Hirsutism	1st year	5/1452 (0.3%)	140120, 141410, 160206, 200509, 210131
	2nd year	6/1206 (0.5%)	141401, 141411, 141415, 210131, 230402, 244606
	Overall	10/1452 (0.7%)	140120, 141401, 141410, 141411, 141415, 160206, 200509, 210131, 230402, 244606
Hyperhidrosis	1st year	2/1452 (0.1%)	245507, 245511
	Overall	2/1452 (0.1%)	245507, 245511
Hypertrichosis	2nd year	1/1206 (<0.1%)	245909
	Overall	1/1452 (<0.1%)	245909

Table 14.3.1 / 10: Number of subjects with adverse events including subject identifier and year by treatment (FAS) (cont.)

TREATMENT: LCS16

Primary system organ class<br>Preferred term<br>MedDRA version 14.0	YEAR OF ONSET	N (%)	subject identifier
Lichen sclerosus	1st year	2/1452 (0.1%)	160932, 161311
	Overall	2/1452 (0.1%)	160932, 161311
Neurodermatitis	1st year	1/1452 (<0.1%)	241522
	Overall	1/1452 (<0.1%)	241522
Night sweats	2nd year	1/1206 (<0.1%)	242811
	Overall	1/1452 (<0.1%)	242811
Photosensitivity reaction	2nd year	1/1206 (<0.1%)	160950
	3rd year	1/1012 (<0.1%)	160950
	Overall	1/1452 (<0.1%)	160950
Pityriasis rosea	1st year	1/1452 (<0.1%)	161113
	2nd year	1/1206 (<0.1%)	141025
	Overall	2/1452 (0.1%)	141025, 161113
Precancerous skin lesion	2nd year	1/1206 (<0.1%)	243301
	Overall	1/1452 (<0.1%)	243301
Pruritus	1st year	1/1452 (<0.1%)	140504
	2nd year	2/1206 (0.2%)	160529, 242704
	Overall	3/1452 (0.2%)	140504, 160529, 242704
Pruritus allergic	1st year	1/1452 (<0.1%)	150164
	2nd year	1/1206 (<0.1%)	150117
	Overall	2/1452 (0.1%)	150117, 150164
Psoriasis	1st year	1/1452 (<0.1%)	120622
	2nd year	1/1206 (<0.1%)	200618
	Overall	2/1452 (0.1%)	120622, 200618
Rash	1st year	7/1452 (0.5%)	140101, 140818, 160513, 230416, 242202, 243509, 243703
	3rd year	1/1012 (<0.1%)	243833
	Overall	8/1452 (0.6%)	140101, 140818, 160513, 230416, 242202, 243509, 243703, 243833

Table 14.3.1 / 10: Number of subjects with adverse events including subject identifier and year by treatment (FAS) (cont.)

TREATMENT: LCS16

Primary system organ class Preferred term MedDRA version 14.0	YEAR OF ONSET	N (%)	subject identifier
Rash macular	3rd year	1/1012 (<0.1%)	243833
	Overall	1/1452 (<0.1%)	243833
Rash papular	2nd year	1/1206 (<0.1%)	243976
	Overall	1/1452 (<0.1%)	243976
Rosacea	1st year	2/1452 (0.1%)	140803, 242317
	Overall	2/1452 (0.1%)	140803, 242317
Scar	2nd year	1/1206 (<0.1%)	210605
	Overall	1/1452 (<0.1%)	210605
Seborrhoea	1st year	8/1452 (0.6%)	150146, 160115, 160517, 160627, 161108, 161116, 246115, 246139
	2nd year	4/1206 (0.3%)	120306, 120318, 160517, 161116
	Overall	10/1452 (0.7%)	120306, 120318, 150146, 160115, 160517, 160627, 161108, 161116, 246115, 246139
Skin fissures	1st year	1/1452 (<0.1%)	160761
	2nd year	1/1206 (<0.1%)	141025
	Overall	2/1452 (0.1%)	141025, 160761
Skin hypopigmentation	3rd year	1/1012 (<0.1%)	243833
	Overall	1/1452 (<0.1%)	243833
Skin irritation	1st year	2/1452 (0.1%)	200902, 242508
	Overall	2/1452 (0.1%)	200902, 242508
Skin lesion	3rd year	1/1012 (<0.1%)	244902
	Overall	1/1452 (<0.1%)	244902
Skin odour abnormal	2nd year	1/1206 (<0.1%)	161211
	Overall	1/1452 (<0.1%)	161211

Table 14.3.1 / 10: Number of subjects with adverse events including subject identifier and year by treatment (FAS) (cont.)

TREATMENT: LCS16

Primary system organ class<br>Preferred term<br>MedDRA version 14.0	YEAR OF ONSET	N (%)	subject identifier
Urticaria	1st year	5/1452 (0.3%)	160330, 160972, 170701, 210110, 244519
	2nd year	2/1206 (0.2%)	200930, 243305
	3rd year	3/1012 (0.3%)	140512, 200509, 243960
	Overall	10/1452 (0.7%)	140512, 160330, 160972, 170701, 200509, 200930, 210110, 243305, 243960, 244519
Vitiligo	3rd year	1/1012 (<0.1%)	243833
	Overall	1/1452 (<0.1%)	243833
Social circumstances	1st year	1/1452 (<0.1%)	
	Overall	1/1452 (<0.1%)	
Hearing disability	1st year	1/1452 (<0.1%)	160561
	Overall	1/1452 (<0.1%)	160561
Surgical and medical procedures	1st year	22/1452 (1.5%)	
	2nd year	18/1206 (1.5%)	
	3rd year	8/1012 (0.8%)	
	Overall	45/1452 (3.1%)	
Abdominoplasty	1st year	1/1452 (<0.1%)	244410
	Overall	1/1452 (<0.1%)	244410
Anal sphincterotomy	2nd year	1/1206 (<0.1%)	241420
	Overall	1/1452 (<0.1%)	241420
Apicectomy	1st year	1/1452 (<0.1%)	200513
	2nd year	1/1206 (<0.1%)	200513
	Overall	1/1452 (<0.1%)	200513
Breast prosthesis implantation	3rd year	1/1012 (<0.1%)	120426
	Overall	1/1452 (<0.1%)	120426
Bunion operation	3rd year	1/1012 (<0.1%)	200510
	Overall	1/1452 (<0.1%)	200510
Cervix cautery	1st year	1/1452 (<0.1%)	190123
	Overall	1/1452 (<0.1%)	190123

Table 14.3.1 / 10: Number of subjects with adverse events including subject identifier and year by treatment (FAS) (cont.)

TREATMENT: LCS16

Primary system organ class<br>Preferred term<br>MedDRA version 14.0	YEAR OF ONSET	N (%)	subject identifier
Dental operation	1st year	3/1452 (0.2%)	120309, 160115, 160123
	Overall	3/1452 (0.2%)	120309, 160115, 160123
Detoxification	2nd year	1/1206 (<0.1%)	244424
	Overall	1/1452 (<0.1%)	244424
Endodontic procedure	1st year	1/1452 (<0.1%)	150131
	2nd year	1/1206 (<0.1%)	160107
	Overall	2/1452 (0.1%)	150131, 160107
Eye laser surgery	2nd year	1/1206 (<0.1%)	160919
	Overall	1/1452 (<0.1%)	160919
Gallbladder operation	1st year	1/1452 (<0.1%)	230612
	Overall	1/1452 (<0.1%)	230612
Gastric bypass	2nd year	1/1206 (<0.1%)	160942
	Overall	1/1452 (<0.1%)	160942
Haemorrhoid operation	2nd year	1/1206 (<0.1%)	160561
	Overall	1/1452 (<0.1%)	160561
Keratomileusis	3rd year	1/1012 (<0.1%)	242317
	Overall	1/1452 (<0.1%)	242317
Mammoplasty	3rd year	3/1012 (0.3%)	160702, 160969, 200902
	Overall	3/1452 (0.2%)	160702, 160969, 200902
Meniscus operation	2nd year	1/1206 (<0.1%)	140119
	Overall	1/1452 (<0.1%)	140119
Mole excision	1st year	1/1452 (<0.1%)	160123
	2nd year	1/1206 (<0.1%)	243229
	Overall	2/1452 (0.1%)	160123, 243229
Nasal polypectomy	1st year	1/1452 (<0.1%)	160735
	Overall	1/1452 (<0.1%)	160735

Table 14.3.1 / 10: Number of subjects with adverse events including subject identifier and year by treatment (FAS) (cont.)

TREATMENT: LCS16

Primary system organ class<br>Preferred term<br>MedDRA version 14.0	YEAR OF ONSET	N (%)	subject identifier
Parasitic infection prophylaxis	1st year	1/1452 (<0.1%)	160950
	Overall	1/1452 (<0.1%)	160950
Plastic surgery	1st year	1/1452 (<0.1%)	230915
	Overall	1/1452 (<0.1%)	230915
Rotator cuff repair	1st year	1/1452 (<0.1%)	242703
	2nd year	1/1206 (<0.1%)	240626
	Overall	2/1452 (0.1%)	240626, 242703
Scar excision	2nd year	1/1206 (<0.1%)	160805
	Overall	1/1452 (<0.1%)	160805
Skin neoplasm excision	2nd year	1/1206 (<0.1%)	243229
	Overall	1/1452 (<0.1%)	243229
Tenolysis	3rd year	1/1012 (<0.1%)	241603
	Overall	1/1452 (<0.1%)	241603
Tonsillectomy	1st year	1/1452 (<0.1%)	160706
	2nd year	1/1206 (<0.1%)	244801
	Overall	2/1452 (0.1%)	160706, 244801
Tooth extraction	1st year	5/1452 (0.3%)	160611, 160987, 200510, 210123, 241194
	2nd year	3/1206 (0.2%)	120603, 120606, 241730
	3rd year	1/1012 (<0.1%)	170405
	Overall	9/1452 (0.6%)	120603, 120606, 160611, 160987, 170405, 200510, 210123, 241194, 241730
Umbilical hernia repair	1st year	1/1452 (<0.1%)	244410
	Overall	1/1452 (<0.1%)	244410
Wisdom teeth removal	1st year	3/1452 (0.2%)	161404, 161423, 244449
	2nd year	3/1206 (0.2%)	140103, 141024, 161423
	Overall	5/1452 (0.3%)	140103, 141024, 161404, 161423, 244449
Wrist surgery	1st year	1/1452 (<0.1%)	160608
	Overall	1/1452 (<0.1%)	160608

Table 14.3.1 / 10: Number of subjects with adverse events including subject identifier and year by treatment (FAS) (cont.)

TREATMENT: LCS16

Primary system organ class<br>Preferred term<br>MedDRA version 14.0	YEAR OF ONSET	N (%)	subject identifier
Vascular disorders	1st year	11/1452 (0.8%)	
	2nd year	11/1206 (0.9%)	
	3rd year	6/1012 (0.6%)	
	Overall	26/1452 (1.8%)	
Hot flush	1st year	1/1452 (<0.1%)	140810
	2nd year	1/1206 (<0.1%)	140810
	3rd year	2/1012 (0.2%)	160533, 240618
	Overall	3/1452 (0.2%)	140810, 160533, 240618
Hypertension	1st year	6/1452 (0.4%)	150126, 160508, 160966, 241236, 243901, 244905
	2nd year	7/1206 (0.6%)	180609, 200712, 240741, 243615, 244305, 244706, 244802
	3rd year	3/1012 (0.3%)	150504, 240325, 244446
	Overall	16/1452 (1.1%)	150126, 150504, 160508, 160966, 180609, 200712, 240325, 240741, 241236, 243615, 243901, 244305, 244446, 244706, 244802, 244905
Hypotension	1st year	2/1452 (0.1%)	120211, 120405
	Overall	2/1452 (0.1%)	120211, 120405
Phlebitis	2nd year	1/1206 (<0.1%)	242704
	Overall	1/1452 (<0.1%)	242704
Varicose vein	1st year	2/1452 (0.1%)	160534, 180609
	2nd year	3/1206 (0.2%)	180609, 180732, 244704
	3rd year	1/1012 (<0.1%)	170104
	Overall	5/1452 (0.3%)	160534, 170104, 180609, 180732, 244704
Vein pain	2nd year	1/1206 (<0.1%)	180609
	Overall	1/1452 (<0.1%)	180609

Note: A subject is counted only once within each preferred term of any primary SOC.
Note: Denominator is number of subjects available at the START of the year or all subjects for overall
Note: Adverse events are sorted in alphabetical order by primary SOC and preferred term.
Global Biostatistics: /by-sasp/patdb/projects/de04209/310442/stat/prod_interim03/pgms/t-ae-cat.sas epkll 12OCT2011 11:21
End of table

Table 14.3.1 / 11: Number of subjects with study drug-related adverse events by primary system organ class and preferred term (FAS)

Primary system organ class<br>Preferred term<br>MedDRA version 14.0	LCS12<br>N=1432 (100%)	LCS16<br>N=1452 (100%)	Total<br>N=2884 (100%)
Number of subjects (%) with at least one such adverse event	710 (49.6%)	756 (52.1%)	1466 (50.8%)
Blood and lymphatic system disorders	4 (0.3%)	1 (<0.1%)	5 (0.2%)
Anaemia	3 (0.2%)	1 (<0.1%)	4 (0.1%)
Leukocytosis	1 (<0.1%)	0	1 (<0.1%)
Lymphadenitis	1 (<0.1%)	0	1 (<0.1%)
Ear and labyrinth disorders	2 (0.1%)	2 (0.1%)	4 (0.1%)
Motion sickness	1 (<0.1%)	0	1 (<0.1%)
Vertigo	1 (<0.1%)	2 (0.1%)	3 (0.1%)
Endocrine disorders	0	1 (<0.1%)	1 (<0.1%)
Goitre	0	1 (<0.1%)	1 (<0.1%)
Eye disorders	0	1 (<0.1%)	1 (<0.1%)
Blepharitis	0	1 (<0.1%)	1 (<0.1%)
Chalazion	0	1 (<0.1%)	1 (<0.1%)
Gastrointestinal disorders	112 (7.8%)	90 (6.2%)	202 (7.0%)
Abdominal discomfort	0	1 (<0.1%)	1 (<0.1%)
Abdominal distension	11 (0.8%)	7 (0.5%)	18 (0.6%)
Abdominal pain	48 (3.4%)	37 (2.5%)	85 (2.9%)
Abdominal pain lower	30 (2.1%)	31 (2.1%)	61 (2.1%)
Abdominal pain upper	1 (<0.1%)	1 (<0.1%)	2 (<0.1%)
Abdominal tenderness	1 (<0.1%)	0	1 (<0.1%)
Aphthous stomatitis	0	1 (<0.1%)	1 (<0.1%)
Constipation	3 (0.2%)	1 (<0.1%)	4 (0.1%)
Dyspepsia	0	1 (<0.1%)	1 (<0.1%)
Flatulence	2 (0.1%)	0	2 (<0.1%)
Frequent bowel movements	1 (<0.1%)	0	1 (<0.1%)
Gastrointestinal disorder	1 (<0.1%)	0	1 (<0.1%)
Gastrooesophageal reflux disease	0	1 (<0.1%)	1 (<0.1%)
Nausea	24 (1.7%)	15 (1.0%)	39 (1.4%)
Vomiting	3 (0.2%)	4 (0.3%)	7 (0.2%)

Table 14.3.1 / 11: Number of subjects with study drug-related adverse events by primary system organ class and preferred term (FAS)

Primary system organ class<br>Preferred term<br>MedDRA version 14.0	LCS12<br>N=1432 (100%)	LCS16<br>N=1452 (100%)	Total<br>N=2884 (100%)
General disorders and administration site conditions	35 (2.4%)	48 (3.3%)	83 (2.9%)
Cyst	1 (<0.1%)	0	1 (<0.1%)
Device dislocation	2 (0.1%)	4 (0.3%)	6 (0.2%)
Device expulsion	21 (1.5%)	25 (1.7%)	46 (1.6%)
Fatigue	6 (0.4%)	6 (0.4%)	12 (0.4%)
Feeling cold	0	1 (<0.1%)	1 (<0.1%)
Feeling hot	0	1 (<0.1%)	1 (<0.1%)
Generalised oedema	1 (<0.1%)	0	1 (<0.1%)
Hunger	0	1 (<0.1%)	1 (<0.1%)
Irritability	2 (0.1%)	6 (0.4%)	8 (0.3%)
Localised oedema	0	1 (<0.1%)	1 (<0.1%)
Oedema	0	1 (<0.1%)	1 (<0.1%)
Oedema peripheral	1 (<0.1%)	1 (<0.1%)	2 (<0.1%)
Pain	2 (0.1%)	1 (<0.1%)	3 (0.1%)
Pyrexia	2 (0.1%)	1 (<0.1%)	3 (0.1%)
Infections and infestations	85 (5.9%)	85 (5.9%)	170 (5.9%)
Candidiasis	4 (0.3%)	1 (<0.1%)	5 (0.2%)
Cervicitis	1 (<0.1%)	7 (0.5%)	8 (0.3%)
Cystitis	2 (0.1%)	1 (<0.1%)	3 (0.1%)
Endometritis	8 (0.6%)	10 (0.7%)	18 (0.6%)
Escherichia vaginitis	1 (<0.1%)	0	1 (<0.1%)
Fungal infection	1 (<0.1%)	1 (<0.1%)	2 (<0.1%)
Gastroenteritis	1 (<0.1%)	0	1 (<0.1%)
Genital candidiasis	0	1 (<0.1%)	1 (<0.1%)
Pelvic inflammatory disease	4 (0.3%)	5 (0.3%)	9 (0.3%)
Pyelonephritis	1 (<0.1%)	0	1 (<0.1%)
Salpingitis	1 (<0.1%)	0	1 (<0.1%)
Salpingo-oophoritis	1 (<0.1%)	3 (0.2%)	4 (0.1%)
Tonsillitis	1 (<0.1%)	0	1 (<0.1%)
Tubo-ovarian abscess	1 (<0.1%)	0	1 (<0.1%)
Upper respiratory tract infection	1 (<0.1%)	0	1 (<0.1%)
Urinary tract infection	6 (0.4%)	2 (0.1%)	8 (0.3%)
Uterine infection	0	1 (<0.1%)	1 (<0.1%)
Vaginal infection	10 (0.7%)	10 (0.7%)	20 (0.7%)
Vaginitis bacterial	24 (1.7%)	27 (1.9%)	51 (1.8%)
Vulvitis	2 (0.1%)	1 (<0.1%)	3 (0.1%)
Vulvovaginal candidiasis	9 (0.6%)	8 (0.6%)	17 (0.6%)
Vulvovaginal mycotic infection	19 (1.3%)	19 (1.3%)	38 (1.3%)
Vulvovaginitis	1 (<0.1%)	3 (0.2%)	4 (0.1%)

Table 14.3.1 / 11: Number of subjects with study drug-related adverse events by primary system organ class and preferred term (FAS)

Primary system organ class<br>Preferred term<br>MedDRA version 14.0	LCS12<br>N=1432 (100%)	LCS16<br>N=1452 (100%)	Total<br>N=2884 (100%)
Injury, poisoning and procedural complications	29 (2.0%)	24 (1.7%)	53 (1.8%)
Post procedural haemorrhage	1 (<0.1%)	0	1 (<0.1%)
Procedural pain	28 (2.0%)	24 (1.7%)	52 (1.8%)
Investigations	44 (3.1%)	56 (3.9%)	100 (3.5%)
Alanine aminotransferase increased	3 (0.2%)	2 (0.1%)	5 (0.2%)
Aspartate aminotransferase increased	3 (0.2%)	1 (<0.1%)	4 (0.1%)
Blood cholesterol increased	1 (<0.1%)	0	1 (<0.1%)
Blood triglycerides increased	1 (<0.1%)	2 (0.1%)	3 (0.1%)
Gamma-glutamyltransferase increased	0	2 (0.1%)	2 (<0.1%)
Glycosylated haemoglobin increased	1 (<0.1%)	0	1 (<0.1%)
Haemoglobin decreased	1 (<0.1%)	1 (<0.1%)	2 (<0.1%)
Smear cervix abnormal	2 (0.1%)	1 (<0.1%)	3 (0.1%)
Ultrasound ovary abnormal	1 (<0.1%)	0	1 (<0.1%)
Weight decreased	0	1 (<0.1%)	1 (<0.1%)
Weight increased	34 (2.4%)	48 (3.3%)	82 (2.8%)
White blood cell count increased	1 (<0.1%)	0	1 (<0.1%)
Metabolism and nutrition disorders	3 (0.2%)	3 (0.2%)	6 (0.2%)
Fluid retention	1 (<0.1%)	1 (<0.1%)	2 (<0.1%)
Hypokalaemia	1 (<0.1%)	0	1 (<0.1%)
Hyponatraemia	1 (<0.1%)	0	1 (<0.1%)
Increased appetite	1 (<0.1%)	1 (<0.1%)	2 (<0.1%)
Weight fluctuation	0	1 (<0.1%)	1 (<0.1%)
Musculoskeletal and connective tissue disorders	7 (0.5%)	11 (0.8%)	18 (0.6%)
Back pain	6 (0.4%)	10 (0.7%)	16 (0.6%)
Muscle spasms	0	1 (<0.1%)	1 (<0.1%)
Musculoskeletal pain	1 (<0.1%)	0	1 (<0.1%)
Neoplasms benign, malignant and unspecified (incl cysts and polyps)	7 (0.5%)	4 (0.3%)	11 (0.4%)
Fibroadenoma of breast	2 (0.1%)	0	2 (<0.1%)
Haemangioma	1 (<0.1%)	0	1 (<0.1%)
Melanocytic naevus	0	1 (<0.1%)	1 (<0.1%)
Uterine leiomyoma	4 (0.3%)	3 (0.2%)	7 (0.2%)

Table 14.3.1 / 11: Number of subjects with study drug-related adverse events by primary system organ class and preferred term (FAS)

Primary system organ class<br>Preferred term<br>MedDRA version 14.0	LCS12<br>N=1432 (100%)	LCS16<br>N=1452 (100%)	Total<br>N=2884 (100%)
Nervous system disorders	60 (4.2%)	60 (4.1%)	120 (4.2%)
Dizziness	1 (<0.1%)	2 (0.1%)	3 (0.1%)
Headache	47 (3.3%)	48 (3.3%)	95 (3.3%)
Migraine	11 (0.8%)	8 (0.6%)	19 (0.7%)
Migraine with aura	2 (0.1%)	0	2 (<0.1%)
Paraesthesia	0	1 (<0.1%)	1 (<0.1%)
Presyncope	0	2 (0.1%)	2 (<0.1%)
Somnolence	1 (<0.1%)	0	1 (<0.1%)
Pregnancy, puerperium and perinatal conditions	3 (0.2%)	8 (0.6%)	11 (0.4%)
Abortion spontaneous	1 (<0.1%)	0	1 (<0.1%)
Abortion spontaneous incomplete	0	1 (<0.1%)	1 (<0.1%)
Ectopic pregnancy	2 (0.1%)	6 (0.4%)	8 (0.3%)
Premature separation of placenta	1 (<0.1%)	0	1 (<0.1%)
Ruptured ectopic pregnancy	0	1 (<0.1%)	1 (<0.1%)
Psychiatric disorders	68 (4.7%)	43 (3.0%)	111 (3.8%)
Affect lability	4 (0.3%)	1 (<0.1%)	5 (0.2%)
Aggression	1 (<0.1%)	0	1 (<0.1%)
Anxiety	6 (0.4%)	2 (0.1%)	8 (0.3%)
Depressed mood	4 (0.3%)	2 (0.1%)	6 (0.2%)
Depression	13 (0.9%)	8 (0.6%)	21 (0.7%)
Depressive symptom	1 (<0.1%)	0	1 (<0.1%)
Insomnia	3 (0.2%)	1 (<0.1%)	4 (0.1%)
Libido decreased	22 (1.5%)	17 (1.2%)	39 (1.4%)
Libido increased	1 (<0.1%)	1 (<0.1%)	2 (<0.1%)
Loss of libido	2 (0.1%)	0	2 (<0.1%)
Mood altered	12 (0.8%)	10 (0.7%)	22 (0.8%)
Mood swings	11 (0.8%)	6 (0.4%)	17 (0.6%)
Nervousness	0	1 (<0.1%)	1 (<0.1%)
Panic attack	0	1 (<0.1%)	1 (<0.1%)
Stress	1 (<0.1%)	0	1 (<0.1%)
Renal and urinary disorders	2 (0.1%)	1 (<0.1%)	3 (0.1%)
Chromaturia	0	1 (<0.1%)	1 (<0.1%)
Dysuria	1 (<0.1%)	0	1 (<0.1%)
Urinary tract disorder	1 (<0.1%)	0	1 (<0.1%)
Urine odour abnormal	0	1 (<0.1%)	1 (<0.1%)

Table 14.3.1 / 11: Number of subjects with study drug-related adverse events by primary system organ class and preferred term (FAS)

Primary system organ class<br>Preferred term<br>MedDRA version 14.0	LCS12<br>N=1432 (100%)	LCS16<br>N=1452 (100%)	Total<br>N=2884 (100%)
Reproductive system and breast disorders	438 (30.6%)	502 (34.6%)	940 (32.6%)
Adnexa uteri pain	1 (<0.1%)	1 (<0.1%)	2 (<0.1%)
Amenorrhoea	0	5 (0.3%)	5 (0.2%)
Atrophic vulvovaginitis	1 (<0.1%)	0	1 (<0.1%)
Breast cyst	1 (<0.1%)	0	1 (<0.1%)
Breast discharge	0	3 (0.2%)	3 (0.1%)
Breast discomfort	4 (0.3%)	7 (0.5%)	11 (0.4%)
Breast engorgement	1 (<0.1%)	0	1 (<0.1%)
Breast enlargement	0	2 (0.1%)	2 (<0.1%)
Breast mass	5 (0.3%)	2 (0.1%)	7 (0.2%)
Breast pain	23 (1.6%)	24 (1.7%)	47 (1.6%)
Breast swelling	1 (<0.1%)	1 (<0.1%)	2 (<0.1%)
Breast tenderness	21 (1.5%)	30 (2.1%)	51 (1.8%)
Cervical discharge	1 (<0.1%)	0	1 (<0.1%)
Cervical dysplasia	6 (0.4%)	4 (0.3%)	10 (0.3%)
Cervical polyp	0	1 (<0.1%)	1 (<0.1%)
Cervix erythema	0	1 (<0.1%)	1 (<0.1%)
Coital bleeding	9 (0.6%)	10 (0.7%)	19 (0.7%)
Dysfunctional uterine bleeding	2 (0.1%)	3 (0.2%)	5 (0.2%)
Dysmenorrhoea	98 (6.8%)	76 (5.2%)	174 (6.0%)
Dyspareunia	16 (1.1%)	13 (0.9%)	29 (1.0%)
Ectropion of cervix	1 (<0.1%)	1 (<0.1%)	2 (<0.1%)
Endometriosis	0	1 (<0.1%)	1 (<0.1%)
Fibrocystic breast disease	0	1 (<0.1%)	1 (<0.1%)
Galactorrhoea	1 (<0.1%)	1 (<0.1%)	2 (<0.1%)
Genital discharge	5 (0.3%)	2 (0.1%)	7 (0.2%)
Genital haemorrhage	1 (<0.1%)	0	1 (<0.1%)
Haemorrhagic ovarian cyst	8 (0.6%)	9 (0.6%)	17 (0.6%)
Hydrometra	2 (0.1%)	0	2 (<0.1%)
Hypomenorrhoea	2 (0.1%)	1 (<0.1%)	3 (0.1%)
Menometrorrhagia	2 (0.1%)	0	2 (<0.1%)
Menorrhagia	4 (0.3%)	9 (0.6%)	13 (0.5%)
Menstrual disorder	3 (0.2%)	1 (<0.1%)	4 (0.1%)
Menstruation irregular	4 (0.3%)	3 (0.2%)	7 (0.2%)
Metrorrhagia	13 (0.9%)	11 (0.8%)	24 (0.8%)
Nipple exudate bloody	0	1 (<0.1%)	1 (<0.1%)
Nipple pain	2 (0.1%)	1 (<0.1%)	3 (0.1%)
Oligomenorrhoea	0	2 (0.1%)	2 (<0.1%)
Ovarian cyst	110 (7.7%)	201 (13.8%)	311 (10.8%)
Ovarian cyst ruptured	1 (<0.1%)	6 (0.4%)	7 (0.2%)
Ovarian enlargement	0	1 (<0.1%)	1 (<0.1%)
Ovarian mass	0	2 (0.1%)	2 (<0.1%)
Ovulation pain	4 (0.3%)	0	4 (0.1%)

Table 14.3.1 / 11: Number of subjects with study drug-related adverse events by primary system organ class and preferred term (FAS)

Primary system organ class<br>Preferred term<br>MedDRA version 14.0	LCS12<br>N=1432 (100%)	LCS16<br>N=1452 (100%)	Total<br>N=2884 (100%)
Pelvic congestion	1 (<0.1%)	0	1 (<0.1%)
Pelvic discomfort	3 (0.2%)	1 (<0.1%)	4 (0.1%)
Pelvic fluid collection	1 (<0.1%)	0	1 (<0.1%)
Pelvic pain	68 (4.7%)	87 (6.0%)	155 (5.4%)
Polycystic ovaries	1 (<0.1%)	0	1 (<0.1%)
Polymenorrhoea	1 (<0.1%)	1 (<0.1%)	2 (<0.1%)
Premenstrual syndrome	10 (0.7%)	9 (0.6%)	19 (0.7%)
Uterine cervical erosion	1 (<0.1%)	0	1 (<0.1%)
Uterine cervical pain	1 (<0.1%)	0	1 (<0.1%)
Uterine haemorrhage	7 (0.5%)	6 (0.4%)	13 (0.5%)
Uterine inflammation	0	1 (<0.1%)	1 (<0.1%)
Uterine pain	1 (<0.1%)	2 (0.1%)	3 (0.1%)
Uterine polyp	2 (0.1%)	0	2 (<0.1%)
Uterine spasm	28 (2.0%)	37 (2.5%)	65 (2.3%)
Uterine tenderness	1 (<0.1%)	2 (0.1%)	3 (0.1%)
Vaginal discharge	28 (2.0%)	28 (1.9%)	56 (1.9%)
Vaginal disorder	1 (<0.1%)	0	1 (<0.1%)
Vaginal haemorrhage	65 (4.5%)	69 (4.8%)	134 (4.6%)
Vaginal odour	1 (<0.1%)	1 (<0.1%)	2 (<0.1%)
Vaginal ulceration	0	1 (<0.1%)	1 (<0.1%)
Vulvovaginal burning sensation	1 (<0.1%)	0	1 (<0.1%)
Vulvovaginal discomfort	1 (<0.1%)	0	1 (<0.1%)
Vulvovaginal dryness	2 (0.1%)	5 (0.3%)	7 (0.2%)
Vulvovaginal pain	2 (0.1%)	0	2 (<0.1%)
Vulvovaginal pruritus	4 (0.3%)	2 (0.1%)	6 (0.2%)

Table 14.3.1 / 11: Number of subjects with study drug-related adverse events by primary system organ class and preferred term (FAS)

Primary system organ class<br>Preferred term<br>MedDRA version 14.0	LCS12<br>N=1432 (100%)	LCS16<br>N=1452 (100%)	Total<br>N=2884 (100%)
Skin and subcutaneous tissue disorders	162 (11.3%)	170 (11.7%)	332 (11.5%)
Acne	144 (10.1%)	144 (9.9%)	288 (10.0%)
Acne cystic	1 (<0.1%)	0	1 (<0.1%)
Alopecia	8 (0.6%)	6 (0.4%)	14 (0.5%)
Chloasma	3 (0.2%)	0	3 (0.1%)
Dandruff	1 (<0.1%)	1 (<0.1%)	2 (<0.1%)
Dermatitis	0	1 (<0.1%)	1 (<0.1%)
Eczema	0	1 (<0.1%)	1 (<0.1%)
Hirsutism	7 (0.5%)	8 (0.6%)	15 (0.5%)
Hyperhidrosis	1 (<0.1%)	0	1 (<0.1%)
Hypertrichosis	2 (0.1%)	1 (<0.1%)	3 (0.1%)
Psoriasis	0	1 (<0.1%)	1 (<0.1%)
Rash	0	1 (<0.1%)	1 (<0.1%)
Rash papular	1 (<0.1%)	0	1 (<0.1%)
Rosacea	0	1 (<0.1%)	1 (<0.1%)
Seborrhoea	6 (0.4%)	10 (0.7%)	16 (0.6%)
Skin irritation	0	1 (<0.1%)	1 (<0.1%)
Skin odour abnormal	0	1 (<0.1%)	1 (<0.1%)
Urticaria	1 (<0.1%)	0	1 (<0.1%)
Vascular disorders	3 (0.2%)	4 (0.3%)	7 (0.2%)
Hot flush	2 (0.1%)	1 (<0.1%)	3 (0.1%)
Hypertension	0	2 (0.1%)	2 (<0.1%)
Phlebitis	0	1 (<0.1%)	1 (<0.1%)
Varicose vein	1 (<0.1%)	0	1 (<0.1%)

Note: A subject is counted only once within each preferred term of any primary SOC.
Note: Adverse events are sorted in alphabetical order by primary SOC and preferred term.
Note: Missing, possible, probable and definite for drug relationship is counted as drug-related.
Global Biostatistics: /by-sasp/patdb/projects/de04209/310442/stat/prod_interim03/pgms/t-ae.sas epkll 12OCT2011 11:24
End of table

Table 14.3.1 / 12: Number of subjects with adverse events causing discontinuation of study drug by primary system organ class and preferred term (FAS)

Primary system organ class<br>Preferred term<br>MedDRA version 14.0	LCS12<br>N=1432 (100%)	LCS16<br>N=1452 (100%)	Total<br>N=2884 (100%)
Number of subjects (%) with at least one such adverse event	319 (22.3%)	290 (20.0%)	609 (21.1%)
Blood and lymphatic system disorders	2 (0.1%)	0	2 (<0.1%)
Lymphadenitis	2 (0.1%)	0	2 (<0.1%)
Cardiac disorders	0	1 (<0.1%)	1 (<0.1%)
Palpitations	0	1 (<0.1%)	1 (<0.1%)
Ear and labyrinth disorders	0	1 (<0.1%)	1 (<0.1%)
Vertigo	0	1 (<0.1%)	1 (<0.1%)
Eye disorders	1 (<0.1%)	1 (<0.1%)	2 (<0.1%)
Blepharitis	0	1 (<0.1%)	1 (<0.1%)
Chalazion	0	1 (<0.1%)	1 (<0.1%)
Iridocyclitis	1 (<0.1%)	0	1 (<0.1%)
Gastrointestinal disorders	49 (3.4%)	28 (1.9%)	77 (2.7%)
Abdominal distension	8 (0.6%)	2 (0.1%)	10 (0.3%)
Abdominal pain	19 (1.3%)	14 (1.0%)	33 (1.1%)
Abdominal pain lower	17 (1.2%)	9 (0.6%)	26 (0.9%)
Constipation	1 (<0.1%)	0	1 (<0.1%)
Frequent bowel movements	1 (<0.1%)	0	1 (<0.1%)
Gastrooesophageal reflux disease	1 (<0.1%)	0	1 (<0.1%)
Irritable bowel syndrome	1 (<0.1%)	0	1 (<0.1%)
Nausea	6 (0.4%)	4 (0.3%)	10 (0.3%)
Vomiting	1 (<0.1%)	0	1 (<0.1%)
General disorders and administration site conditions	53 (3.7%)	46 (3.2%)	99 (3.4%)
Asthenia	1 (<0.1%)	0	1 (<0.1%)
Device dislocation	3 (0.2%)	5 (0.3%)	8 (0.3%)
Device expulsion	45 (3.1%)	36 (2.5%)	81 (2.8%)
Fatigue	3 (0.2%)	3 (0.2%)	6 (0.2%)
Irritability	1 (<0.1%)	0	1 (<0.1%)
Oedema	0	1 (<0.1%)	1 (<0.1%)
Oedema peripheral	0	1 (<0.1%)	1 (<0.1%)

Table 14.3.1 / 12: Number of subjects with adverse events causing discontinuation of study drug by primary system organ class and preferred term (FAS)

Primary system organ class<br>Preferred term<br>MedDRA version 14.0	LCS12<br>N=1432 (100%)	LCS16<br>N=1452 (100%)	Total<br>N=2884 (100%)
Infections and infestations	22 (1.5%)	24 (1.7%)	46 (1.6%)
Cervicitis	0	1 (<0.1%)	1 (<0.1%)
Endometritis	1 (<0.1%)	2 (0.1%)	3 (0.1%)
Genital herpes	1 (<0.1%)	0	1 (<0.1%)
Gynaecological chlamydia infection	1 (<0.1%)	1 (<0.1%)	2 (<0.1%)
Influenza	1 (<0.1%)	0	1 (<0.1%)
Pelvic inflammatory disease	5 (0.3%)	5 (0.3%)	10 (0.3%)
Pyelonephritis	2 (0.1%)	1 (<0.1%)	3 (0.1%)
Salpingo-oophoritis	1 (<0.1%)	1 (<0.1%)	2 (<0.1%)
Tonsillitis	1 (<0.1%)	0	1 (<0.1%)
Tubo-ovarian abscess	1 (<0.1%)	0	1 (<0.1%)
Urinary tract infection	3 (0.2%)	3 (0.2%)	6 (0.2%)
Uterine infection	0	1 (<0.1%)	1 (<0.1%)
Vaginal infection	3 (0.2%)	1 (<0.1%)	4 (0.1%)
Vaginitis bacterial	4 (0.3%)	4 (0.3%)	8 (0.3%)
Viral infection	0	1 (<0.1%)	1 (<0.1%)
Vulvovaginal candidiasis	2 (0.1%)	0	2 (<0.1%)
Vulvovaginal mycotic infection	0	2 (0.1%)	2 (<0.1%)
Vulvovaginitis	0	2 (0.1%)	2 (<0.1%)
Injury, poisoning and procedural complications	1 (<0.1%)	0	1 (<0.1%)
Procedural pain	1 (<0.1%)	0	1 (<0.1%)
Investigations	11 (0.8%)	17 (1.2%)	28 (1.0%)
Alanine aminotransferase increased	1 (<0.1%)	0	1 (<0.1%)
Aspartate aminotransferase increased	1 (<0.1%)	0	1 (<0.1%)
Haemoglobin decreased	0	1 (<0.1%)	1 (<0.1%)
Human papilloma virus test positive	0	1 (<0.1%)	1 (<0.1%)
Smear cervix abnormal	0	1 (<0.1%)	1 (<0.1%)
Weight increased	10 (0.7%)	14 (1.0%)	24 (0.8%)
Metabolism and nutrition disorders	1 (<0.1%)	1 (<0.1%)	2 (<0.1%)
Fluid retention	1 (<0.1%)	1 (<0.1%)	2 (<0.1%)
Musculoskeletal and connective tissue disorders	4 (0.3%)	2 (0.1%)	6 (0.2%)
Arthralgia	1 (<0.1%)	0	1 (<0.1%)
Back pain	3 (0.2%)	1 (<0.1%)	4 (0.1%)
Pain in extremity	0	1 (<0.1%)	1 (<0.1%)

Table 14.3.1 / 12: Number of subjects with adverse events causing discontinuation of study drug by primary system organ class and preferred term (FAS)

Primary system organ class<br>Preferred term<br>MedDRA version 14.0	LCS12<br>N=1432 (100%)	LCS16<br>N=1452 (100%)	Total<br>N=2884 (100%)
Neoplasms benign, malignant and unspecified (incl cysts and polyps)	3 (0.2%)	3 (0.2%)	6 (0.2%)
Cervicitis human papilloma virus	0	1 (<0.1%)	1 (<0.1%)
Cervix carcinoma stage 0	0	1 (<0.1%)	1 (<0.1%)
Ovarian germ cell teratoma benign	1 (<0.1%)	0	1 (<0.1%)
Uterine leiomyoma	2 (0.1%)	1 (<0.1%)	3 (0.1%)
Nervous system disorders	9 (0.6%)	9 (0.6%)	18 (0.6%)
Amnesia	1 (<0.1%)	1 (<0.1%)	2 (<0.1%)
Dizziness	1 (<0.1%)	1 (<0.1%)	2 (<0.1%)
Headache	6 (0.4%)	7 (0.5%)	13 (0.5%)
Migraine	1 (<0.1%)	0	1 (<0.1%)
Paraesthesia	0	1 (<0.1%)	1 (<0.1%)
Tension headache	0	1 (<0.1%)	1 (<0.1%)
Pregnancy, puerperium and perinatal conditions	5 (0.3%)	9 (0.6%)	14 (0.5%)
Abortion spontaneous	2 (0.1%)	0	2 (<0.1%)
Abortion spontaneous incomplete	0	1 (<0.1%)	1 (<0.1%)
Blighted ovum	0	1 (<0.1%)	1 (<0.1%)
Ectopic pregnancy	3 (0.2%)	6 (0.4%)	9 (0.3%)
Premature separation of placenta	1 (<0.1%)	0	1 (<0.1%)
Ruptured ectopic pregnancy	0	1 (<0.1%)	1 (<0.1%)
Psychiatric disorders	27 (1.9%)	22 (1.5%)	49 (1.7%)
Affect lability	0	1 (<0.1%)	1 (<0.1%)
Anxiety	4 (0.3%)	1 (<0.1%)	5 (0.2%)
Depressed mood	1 (<0.1%)	2 (0.1%)	3 (0.1%)
Depression	6 (0.4%)	2 (0.1%)	8 (0.3%)
Insomnia	1 (<0.1%)	1 (<0.1%)	2 (<0.1%)
Libido decreased	8 (0.6%)	10 (0.7%)	18 (0.6%)
Loss of libido	1 (<0.1%)	0	1 (<0.1%)
Mental status changes	0	1 (<0.1%)	1 (<0.1%)
Mood altered	7 (0.5%)	4 (0.3%)	11 (0.4%)
Mood swings	5 (0.3%)	5 (0.3%)	10 (0.3%)
Nervousness	0	1 (<0.1%)	1 (<0.1%)
Panic attack	0	1 (<0.1%)	1 (<0.1%)
Renal and urinary disorders	2 (0.1%)	0	2 (<0.1%)
Pollakiuria	1 (<0.1%)	0	1 (<0.1%)
Stress urinary incontinence	1 (<0.1%)	0	1 (<0.1%)

Table 14.3.1 / 12: Number of subjects with adverse events causing discontinuation of study drug by primary system organ class and preferred term (FAS)

Primary system organ class<br>Preferred term<br>MedDRA version 14.0	LCS12<br>N=1432 (100%)	LCS16<br>N=1452 (100%)	Total<br>N=2884 (100%)
Reproductive system and breast disorders	142 (9.9%)	147 (10.1%)	289 (10.0%)
Adnexa uteri pain	0	1 (<0.1%)	1 (<0.1%)
Amenorrhoea	0	2 (0.1%)	2 (<0.1%)
Atrophic vulvovaginitis	1 (<0.1%)	0	1 (<0.1%)
Breast discomfort	1 (<0.1%)	1 (<0.1%)	2 (<0.1%)
Breast pain	5 (0.3%)	1 (<0.1%)	6 (0.2%)
Breast tenderness	1 (<0.1%)	0	1 (<0.1%)
Cervical dysplasia	1 (<0.1%)	4 (0.3%)	5 (0.2%)
Coital bleeding	4 (0.3%)	0	4 (0.1%)
Dysfunctional uterine bleeding	1 (<0.1%)	2 (0.1%)	3 (0.1%)
Dysmenorrhoea	20 (1.4%)	12 (0.8%)	32 (1.1%)
Dyspareunia	8 (0.6%)	7 (0.5%)	15 (0.5%)
Ectropion of cervix	1 (<0.1%)	1 (<0.1%)	2 (<0.1%)
Endometriosis	3 (0.2%)	2 (0.1%)	5 (0.2%)
Galactorrhoea	0	1 (<0.1%)	1 (<0.1%)
Genital discharge	0	1 (<0.1%)	1 (<0.1%)
Haemorrhagic ovarian cyst	2 (0.1%)	0	2 (<0.1%)
Menometrorrhagia	1 (<0.1%)	0	1 (<0.1%)
Menorrhagia	2 (0.1%)	6 (0.4%)	8 (0.3%)
Menstrual disorder	1 (<0.1%)	0	1 (<0.1%)
Menstruation irregular	1 (<0.1%)	2 (0.1%)	3 (0.1%)
Metrorrhagia	6 (0.4%)	5 (0.3%)	11 (0.4%)
Oligomenorrhoea	0	2 (0.1%)	2 (<0.1%)
Ovarian cyst	3 (0.2%)	5 (0.3%)	8 (0.3%)
Ovulation pain	1 (<0.1%)	2 (0.1%)	3 (0.1%)
Pelvic discomfort	1 (<0.1%)	1 (<0.1%)	2 (<0.1%)
Pelvic pain	29 (2.0%)	39 (2.7%)	68 (2.4%)
Pelvic prolapse	1 (<0.1%)	0	1 (<0.1%)
Polymenorrhoea	0	1 (<0.1%)	1 (<0.1%)
Premenstrual syndrome	0	2 (0.1%)	2 (<0.1%)
Uterine haemorrhage	6 (0.4%)	2 (0.1%)	8 (0.3%)
Uterine inflammation	0	1 (<0.1%)	1 (<0.1%)
Uterine prolapse	1 (<0.1%)	0	1 (<0.1%)
Uterine spasm	10 (0.7%)	8 (0.6%)	18 (0.6%)
Uterine tenderness	1 (<0.1%)	0	1 (<0.1%)
Vaginal discharge	1 (<0.1%)	4 (0.3%)	5 (0.2%)
Vaginal haemorrhage	48 (3.4%)	48 (3.3%)	96 (3.3%)
Vaginal odour	0	1 (<0.1%)	1 (<0.1%)
Vulvovaginal dryness	1 (<0.1%)	2 (0.1%)	3 (0.1%)
Vulvovaginal pain	1 (<0.1%)	0	1 (<0.1%)

Table 14.3.1 / 12: Number of subjects with adverse events causing discontinuation of study drug by primary system organ class and preferred term (FAS)

Primary system organ class<br>Preferred term<br>MedDRA version 14.0	LCS12<br>N=1432 (100%)	LCS16<br>N=1452 (100%)	Total<br>N=2884 (100%)
Skin and subcutaneous tissue disorders	47 (3.3%)	33 (2.3%)	80 (2.8%)
Acne	39 (2.7%)	25 (1.7%)	64 (2.2%)
Acne cystic	1 (<0.1%)	0	1 (<0.1%)
Alopecia	4 (0.3%)	3 (0.2%)	7 (0.2%)
Hirsutism	2 (0.1%)	2 (0.1%)	4 (0.1%)
Hyperhidrosis	1 (<0.1%)	0	1 (<0.1%)
Hypertrichosis	1 (<0.1%)	0	1 (<0.1%)
Psoriasis	0	1 (<0.1%)	1 (<0.1%)
Seborrhoea	3 (0.2%)	2 (0.1%)	5 (0.2%)
Skin irritation	0	1 (<0.1%)	1 (<0.1%)
Skin odour abnormal	0	1 (<0.1%)	1 (<0.1%)
Urticaria	1 (<0.1%)	0	1 (<0.1%)
Vascular disorders	1 (<0.1%)	1 (<0.1%)	2 (<0.1%)
Deep vein thrombosis	1 (<0.1%)	0	1 (<0.1%)
Hypertension	0	1 (<0.1%)	1 (<0.1%)

Note: A subject is counted only once within each preferred term of any primary SOC.
Note: Adverse events are sorted in alphabetical order by primary SOC and preferred term.
Global Biostatistics: /by-sasp/patdb/projects/de04209/310442/stat/prod_interim03/pgms/t-ae.sas epkll 12OCT2011 11:24
End of table

Table 14.3.1 / 13: Number of subjects with study drug-related adverse events causing discontinuation of study drug by primary system organ class and preferred term (FAS)

Primary system organ class<br>Preferred term<br>MedDRA version 14.0	LCS12<br>N=1432 (100%)	LCS16<br>N=1452 (100%)	Total<br>N=2884 (100%)
Number of subjects (%) with at least one such adverse event	249 (17.4%)	232 (16.0%)	481 (16.7%)
Blood and lymphatic system disorders	1 (<0.1%)	0	1 (<0.1%)
Lymphadenitis	1 (<0.1%)	0	1 (<0.1%)
Ear and labyrinth disorders	0	1 (<0.1%)	1 (<0.1%)
Vertigo	0	1 (<0.1%)	1 (<0.1%)
Eye disorders	0	1 (<0.1%)	1 (<0.1%)
Blepharitis	0	1 (<0.1%)	1 (<0.1%)
Chalazion	0	1 (<0.1%)	1 (<0.1%)
Gastrointestinal disorders	37 (2.6%)	23 (1.6%)	60 (2.1%)
Abdominal distension	7 (0.5%)	2 (0.1%)	9 (0.3%)
Abdominal pain	15 (1.0%)	10 (0.7%)	25 (0.9%)
Abdominal pain lower	11 (0.8%)	8 (0.6%)	19 (0.7%)
Constipation	1 (<0.1%)	0	1 (<0.1%)
Frequent bowel movements	1 (<0.1%)	0	1 (<0.1%)
Nausea	6 (0.4%)	3 (0.2%)	9 (0.3%)
General disorders and administration site conditions	26 (1.8%)	31 (2.1%)	57 (2.0%)
Device dislocation	2 (0.1%)	4 (0.3%)	6 (0.2%)
Device expulsion	21 (1.5%)	25 (1.7%)	46 (1.6%)
Fatigue	2 (0.1%)	1 (<0.1%)	3 (0.1%)
Irritability	1 (<0.1%)	0	1 (<0.1%)
Oedema	0	1 (<0.1%)	1 (<0.1%)

Table 14.3.1 / 13: Number of subjects with study drug-related adverse events causing discontinuation of study drug by primary system organ class and preferred term (FAS)

Primary system organ class<br>Preferred term<br>MedDRA version 14.0	LCS12<br>N=1432 (100%)	LCS16<br>N=1452 (100%)	Total<br>N=2884 (100%)
Infections and infestations	11 (0.8%)	16 (1.1%)	27 (0.9%)
Cervicitis	0	1 (<0.1%)	1 (<0.1%)
Endometritis	1 (<0.1%)	2 (0.1%)	3 (0.1%)
Pelvic inflammatory disease	4 (0.3%)	5 (0.3%)	9 (0.3%)
Pyelonephritis	1 (<0.1%)	0	1 (<0.1%)
Salpingo-oophoritis	0	1 (<0.1%)	1 (<0.1%)
Tonsillitis	1 (<0.1%)	0	1 (<0.1%)
Tubo-ovarian abscess	1 (<0.1%)	0	1 (<0.1%)
Urinary tract infection	0	1 (<0.1%)	1 (<0.1%)
Uterine infection	0	1 (<0.1%)	1 (<0.1%)
Vaginal infection	3 (0.2%)	1 (<0.1%)	4 (0.1%)
Vaginitis bacterial	1 (<0.1%)	2 (0.1%)	3 (0.1%)
Vulvovaginal mycotic infection	0	2 (0.1%)	2 (<0.1%)
Vulvovaginitis	0	1 (<0.1%)	1 (<0.1%)
Injury, poisoning and procedural complications	1 (<0.1%)	0	1 (<0.1%)
Procedural pain	1 (<0.1%)	0	1 (<0.1%)
Investigations	9 (0.6%)	10 (0.7%)	19 (0.7%)
Alanine aminotransferase increased	1 (<0.1%)	0	1 (<0.1%)
Aspartate aminotransferase increased	1 (<0.1%)	0	1 (<0.1%)
Weight increased	8 (0.6%)	10 (0.7%)	18 (0.6%)
Metabolism and nutrition disorders	0	1 (<0.1%)	1 (<0.1%)
Fluid retention	0	1 (<0.1%)	1 (<0.1%)
Musculoskeletal and connective tissue disorders	1 (<0.1%)	0	1 (<0.1%)
Back pain	1 (<0.1%)	0	1 (<0.1%)
Neoplasms benign, malignant and unspecified (incl cysts and polyps)	0	1 (<0.1%)	1 (<0.1%)
Uterine leiomyoma	0	1 (<0.1%)	1 (<0.1%)
Nervous system disorders	6 (0.4%)	5 (0.3%)	11 (0.4%)
Dizziness	1 (<0.1%)	0	1 (<0.1%)
Headache	4 (0.3%)	5 (0.3%)	9 (0.3%)
Migraine	1 (<0.1%)	0	1 (<0.1%)

Table 14.3.1 / 13: Number of subjects with study drug-related adverse events causing discontinuation of study drug by primary system organ class and preferred term (FAS)

Primary system organ class<br>Preferred term<br>MedDRA version 14.0	LCS12<br>N=1432 (100%)	LCS16<br>N=1452 (100%)	Total<br>N=2884 (100%)
Pregnancy, puerperium and perinatal conditions	3 (0.2%)	8 (0.6%)	11 (0.4%)
Abortion spontaneous	1 (<0.1%)	0	1 (<0.1%)
Abortion spontaneous incomplete	0	1 (<0.1%)	1 (<0.1%)
Ectopic pregnancy	2 (0.1%)	6 (0.4%)	8 (0.3%)
Premature separation of placenta	1 (<0.1%)	0	1 (<0.1%)
Ruptured ectopic pregnancy	0	1 (<0.1%)	1 (<0.1%)
Psychiatric disorders	24 (1.7%)	16 (1.1%)	40 (1.4%)
Affect lability	0	1 (<0.1%)	1 (<0.1%)
Anxiety	4 (0.3%)	0	4 (0.1%)
Depressed mood	1 (<0.1%)	1 (<0.1%)	2 (<0.1%)
Depression	4 (0.3%)	2 (0.1%)	6 (0.2%)
Insomnia	0	1 (<0.1%)	1 (<0.1%)
Libido decreased	8 (0.6%)	7 (0.5%)	15 (0.5%)
Mood altered	7 (0.5%)	4 (0.3%)	11 (0.4%)
Mood swings	5 (0.3%)	4 (0.3%)	9 (0.3%)
Nervousness	0	1 (<0.1%)	1 (<0.1%)
Panic attack	0	1 (<0.1%)	1 (<0.1%)

Table 14.3.1 / 13: Number of subjects with study drug-related adverse events causing discontinuation of study drug by primary system organ class and preferred term (FAS)

Primary system organ class<br>Preferred term<br>MedDRA version 14.0	LCS12<br>N=1432 (100%)	LCS16<br>N=1452 (100%)	Total<br>N=2884 (100%)
Reproductive system and breast disorders	126 (8.8%)	127 (8.7%)	253 (8.8%)
Adnexa uteri pain	0	1 (<0.1%)	1 (<0.1%)
Amenorrhoea	0	1 (<0.1%)	1 (<0.1%)
Atrophic vulvovaginitis	1 (<0.1%)	0	1 (<0.1%)
Breast discomfort	1 (<0.1%)	1 (<0.1%)	2 (<0.1%)
Breast pain	5 (0.3%)	1 (<0.1%)	6 (0.2%)
Coital bleeding	3 (0.2%)	0	3 (0.1%)
Dysfunctional uterine bleeding	1 (<0.1%)	2 (0.1%)	3 (0.1%)
Dysmenorrhoea	17 (1.2%)	12 (0.8%)	29 (1.0%)
Dyspareunia	7 (0.5%)	3 (0.2%)	10 (0.3%)
Ectropion of cervix	1 (<0.1%)	1 (<0.1%)	2 (<0.1%)
Endometriosis	0	1 (<0.1%)	1 (<0.1%)
Genital discharge	0	1 (<0.1%)	1 (<0.1%)
Haemorrhagic ovarian cyst	1 (<0.1%)	0	1 (<0.1%)
Menometrorrhagia	1 (<0.1%)	0	1 (<0.1%)
Menorrhagia	2 (0.1%)	6 (0.4%)	8 (0.3%)
Menstrual disorder	1 (<0.1%)	0	1 (<0.1%)
Menstruation irregular	1 (<0.1%)	2 (0.1%)	3 (0.1%)
Metrorrhagia	5 (0.3%)	4 (0.3%)	9 (0.3%)
Oligomenorrhoea	0	2 (0.1%)	2 (<0.1%)
Ovarian cyst	2 (0.1%)	2 (0.1%)	4 (0.1%)
Ovulation pain	1 (<0.1%)	0	1 (<0.1%)
Pelvic discomfort	0	1 (<0.1%)	1 (<0.1%)
Pelvic pain	25 (1.7%)	34 (2.3%)	59 (2.0%)
Polymenorrhoea	0	1 (<0.1%)	1 (<0.1%)
Premenstrual syndrome	0	2 (0.1%)	2 (<0.1%)
Uterine haemorrhage	5 (0.3%)	2 (0.1%)	7 (0.2%)
Uterine inflammation	0	1 (<0.1%)	1 (<0.1%)
Uterine spasm	9 (0.6%)	8 (0.6%)	17 (0.6%)
Uterine tenderness	1 (<0.1%)	0	1 (<0.1%)
Vaginal discharge	1 (<0.1%)	2 (0.1%)	3 (0.1%)
Vaginal haemorrhage	47 (3.3%)	46 (3.2%)	93 (3.2%)
Vaginal odour	0	1 (<0.1%)	1 (<0.1%)
Vulvovaginal dryness	1 (<0.1%)	2 (0.1%)	3 (0.1%)
Vulvovaginal pain	1 (<0.1%)	0	1 (<0.1%)

Table 14.3.1 / 13: Number of subjects with study drug-related adverse events causing discontinuation of study drug by primary system organ class and preferred term (FAS)

Primary system organ class<br>Preferred term<br>MedDRA version 14.0	LCS12<br>N=1432 (100%)	LCS16<br>N=1452 (100%)	Total<br>N=2884 (100%)
Skin and subcutaneous tissue disorders	46 (3.2%)	29 (2.0%)	75 (2.6%)
Acne	39 (2.7%)	23 (1.6%)	62 (2.1%)
Alopecia	4 (0.3%)	1 (<0.1%)	5 (0.2%)
Hirsutism	2 (0.1%)	2 (0.1%)	4 (0.1%)
Hyperhidrosis	1 (<0.1%)	0	1 (<0.1%)
Hypertrichosis	1 (<0.1%)	0	1 (<0.1%)
Psoriasis	0	1 (<0.1%)	1 (<0.1%)
Seborrhoea	3 (0.2%)	2 (0.1%)	5 (0.2%)
Skin irritation	0	1 (<0.1%)	1 (<0.1%)
Skin odour abnormal	0	1 (<0.1%)	1 (<0.1%)
Urticaria	1 (<0.1%)	0	1 (<0.1%)
Vascular disorders	0	1 (<0.1%)	1 (<0.1%)
Hypertension	0	1 (<0.1%)	1 (<0.1%)

Note: A subject is counted only once within each preferred term of any primary SOC.
Note: Adverse events are sorted in alphabetical order by primary SOC and preferred term.
Note: Missing, possible, probable and definite for drug relationship is counted as drug-related.
Global Biostatistics: /by-sasp/patdb/projects/de04209/310442/stat/prod_interim03/pgms/t-ae.sas epkll 12OCT2011 11:24
End of table

Table 14.3.1 / 14: Number of subjects with adverse events by outcome, primary system organ class and preferred term (FAS)

Primary system organ class<br>Preferred term<br>MedDRA version 14.0	Maximum Derived AE outcome	LCS12<br>N=1432 (100%)	LCS16<br>N=1452 (100%)	Total<br>N=2884 (100%)
Number of subjects (%) with at least one AE	recovered/resolved	595 (41.6%)	641 (44.1%)	1236 (42.9%)
	recovering/resolving	42 (2.9%)	46 (3.2%)	88 (3.1%)
	not recovered/not resolved	523 (36.5%)	529 (36.4%)	1052 (36.5%)
	recovered/resolved with resid. effects	11 (0.8%)	7 (0.5%)	18 (0.6%)
	fatal	0	1 (<0.1%)	1 (<0.1%)
Blood and lymphatic system disorders	recovered/resolved	10 (0.7%)	4 (0.3%)	14 (0.5%)
	recovering/resolving	1 (<0.1%)	0	1 (<0.1%)
	not recovered/not resolved	4 (0.3%)	4 (0.3%)	8 (0.3%)
Anaemia	recovered/resolved	4 (0.3%)	1 (<0.1%)	5 (0.2%)
	not recovered/not resolved	2 (0.1%)	2 (0.1%)	4 (0.1%)
Iron deficiency anaemia	recovered/resolved	1 (<0.1%)	1 (<0.1%)	2 (<0.1%)
Leukocytosis	recovered/resolved	1 (<0.1%)	0	1 (<0.1%)
Lymphadenitis	recovered/resolved	1 (<0.1%)	0	1 (<0.1%)
	recovering/resolving	1 (<0.1%)	0	1 (<0.1%)
	not recovered/not resolved	1 (<0.1%)	0	1 (<0.1%)
Lymphadenopathy	recovered/resolved	3 (0.2%)	1 (<0.1%)	4 (0.1%)
	not recovered/not resolved	0	2 (0.1%)	2 (<0.1%)
Pancytopenia	recovered/resolved	0	1 (<0.1%)	1 (<0.1%)
Spherocytic anaemia	recovered/resolved	1 (<0.1%)	0	1 (<0.1%)
Splenic cyst	not recovered/not resolved	1 (<0.1%)	0	1 (<0.1%)
Splenomegaly	recovered/resolved	1 (<0.1%)	0	1 (<0.1%)
Cardiac disorders	recovered/resolved	7 (0.5%)	9 (0.6%)	16 (0.6%)
	not recovered/not resolved	2 (0.1%)	5 (0.3%)	7 (0.2%)
Arrhythmia	recovered/resolved	1 (<0.1%)	1 (<0.1%)	2 (<0.1%)
	not recovered/not resolved	0	1 (<0.1%)	1 (<0.1%)

Table 14.3.1 / 14: Number of subjects with adverse events by outcome, primary system organ class and preferred term (FAS)

Primary system organ class<br>Preferred term<br>MedDRA version 14.0	Maximum Derived AE outcome	LCS12<br>N=1432 (100%)	LCS16<br>N=1452 (100%)	Total<br>N=2884 (100%)
Extrasystoles	recovered/resolved	0	2 (0.1%)	2 (<0.1%)
Mitral valve prolapse	not recovered/not resolved	0	2 (0.1%)	2 (<0.1%)
Palpitations	recovered/resolved	4 (0.3%)	3 (0.2%)	7 (0.2%)
	not recovered/not resolved	0	3 (0.2%)	3 (0.1%)
Sinus tachycardia	recovered/resolved	0	1 (<0.1%)	1 (<0.1%)
Tachycardia	recovered/resolved	2 (0.1%)	2 (0.1%)	4 (0.1%)
	not recovered/not resolved	2 (0.1%)	0	2 (<0.1%)
Ventricular extrasystoles	recovered/resolved	0	1 (<0.1%)	1 (<0.1%)
Congenital, familial and genetic disorders	recovered/resolved	1 (<0.1%)	0	1 (<0.1%)
	not recovered/not resolved	2 (0.1%)	1 (<0.1%)	3 (0.1%)
Congenital hydronephrosis	not recovered/not resolved	1 (<0.1%)	0	1 (<0.1%)
Dermoid cyst	not recovered/not resolved	1 (<0.1%)	0	1 (<0.1%)
Myotonia congenita	not recovered/not resolved	0	1 (<0.1%)	1 (<0.1%)
Urethral intrinsic sphincter deficiency	recovered/resolved	1 (<0.1%)	0	1 (<0.1%)
Ear and labyrinth disorders	recovered/resolved	12 (0.8%)	12 (0.8%)	24 (0.8%)
	not recovered/not resolved	4 (0.3%)	2 (0.1%)	6 (0.2%)
Deafness bilateral	recovered/resolved	1 (<0.1%)	0	1 (<0.1%)
Ear pain	recovered/resolved	0	3 (0.2%)	3 (0.1%)
Ear pruritus	recovered/resolved	1 (<0.1%)	0	1 (<0.1%)
Meniere's disease	not recovered/not resolved	0	1 (<0.1%)	1 (<0.1%)
Middle ear effusion	recovered/resolved	0	2 (0.1%)	2 (<0.1%)

Table 14.3.1 / 14: Number of subjects with adverse events by outcome, primary system organ class and preferred term (FAS)

Primary system organ class<br>Preferred term<br>MedDRA version 14.0	Maximum Derived AE outcome	LCS12<br>N=1432 (100%)	LCS16<br>N=1452 (100%)	Total<br>N=2884 (100%)
Motion sickness	recovered/resolved	3 (0.2%)	3 (0.2%)	6 (0.2%)
	not recovered/not resolved	1 (<0.1%)	0	1 (<0.1%)
Otorrhoea	recovered/resolved	1 (<0.1%)	1 (<0.1%)	2 (<0.1%)
Sudden hearing loss	recovered/resolved	1 (<0.1%)	0	1 (<0.1%)
Tinnitus	not recovered/not resolved	2 (0.1%)	0	2 (<0.1%)
Tympanic membrane perforation	recovered/resolved	2 (0.1%)	0	2 (<0.1%)
Vertigo	recovered/resolved	3 (0.2%)	5 (0.3%)	8 (0.3%)
	not recovered/not resolved	1 (<0.1%)	1 (<0.1%)	2 (<0.1%)
Vertigo labyrinthine	recovered/resolved	1 (<0.1%)	0	1 (<0.1%)
Vertigo positional	recovered/resolved	2 (0.1%)	0	2 (<0.1%)
Endocrine disorders	recovered/resolved	1 (<0.1%)	1 (<0.1%)	2 (<0.1%)
	recovering/resolving	1 (<0.1%)	2 (0.1%)	3 (0.1%)
	not recovered/not resolved	7 (0.5%)	18 (1.2%)	25 (0.9%)
Goitre	recovered/resolved	1 (<0.1%)	1 (<0.1%)	2 (<0.1%)
	recovering/resolving	0	1 (<0.1%)	1 (<0.1%)
	not recovered/not resolved	1 (<0.1%)	3 (0.2%)	4 (0.1%)
Hyperthyroidism	not recovered/not resolved	0	2 (0.1%)	2 (<0.1%)
Hypothyroidism	recovering/resolving	1 (<0.1%)	1 (<0.1%)	2 (<0.1%)
	not recovered/not resolved	5 (0.3%)	12 (0.8%)	17 (0.6%)
Thyroid mass	not recovered/not resolved	0	1 (<0.1%)	1 (<0.1%)
Thyroiditis	not recovered/not resolved	1 (<0.1%)	0	1 (<0.1%)
Eye disorders	recovered/resolved	24 (1.7%)	21 (1.4%)	45 (1.6%)
	not recovered/not resolved	1 (<0.1%)	2 (0.1%)	3 (0.1%)

Table 14.3.1 / 14: Number of subjects with adverse events by outcome, primary system organ class and preferred term (FAS)

Primary system organ class<br>Preferred term<br>MedDRA version 14.0	Maximum Derived AE outcome	LCS12<br>N=1432 (100%)	LCS16<br>N=1452 (100%)	Total<br>N=2884 (100%)
Blepharitis	recovered/resolved	1 (<0.1%)	1 (<0.1%)	2 (<0.1%)
	not recovered/not resolved	0	1 (<0.1%)	1 (<0.1%)
Chalazion	not recovered/not resolved	0	1 (<0.1%)	1 (<0.1%)
Conjunctivitis	recovered/resolved	15 (1.0%)	15 (1.0%)	30 (1.0%)
Conjunctivitis allergic	recovered/resolved	2 (0.1%)	2 (0.1%)	4 (0.1%)
Dry eye	recovered/resolved	1 (<0.1%)	0	1 (<0.1%)
	not recovered/not resolved	1 (<0.1%)	0	1 (<0.1%)
Eye inflammation	recovered/resolved	0	1 (<0.1%)	1 (<0.1%)
Eyelid cyst	not recovered/not resolved	0	1 (<0.1%)	1 (<0.1%)
Heterophoria	recovered/resolved	1 (<0.1%)	0	1 (<0.1%)
Iridocyclitis	recovered/resolved	1 (<0.1%)	0	1 (<0.1%)
Iritis	recovered/resolved	0	2 (0.1%)	2 (<0.1%)
Meibomianitis	recovered/resolved	1 (<0.1%)	0	1 (<0.1%)
Myopia	recovered/resolved	0	1 (<0.1%)	1 (<0.1%)
Ocular icterus	recovered/resolved	0	1 (<0.1%)	1 (<0.1%)
Panophthalmitis	recovered/resolved	1 (<0.1%)	0	1 (<0.1%)
Scotoma	recovered/resolved	1 (<0.1%)	0	1 (<0.1%)
Vision blurred	recovered/resolved	1 (<0.1%)	0	1 (<0.1%)
Gastrointestinal disorders	recovered/resolved	235 (16.4%)	256 (17.6%)	491 (17.0%)
	recovering/resolving	14 (1.0%)	9 (0.6%)	23 (0.8%)
	not recovered/not resolved	76 (5.3%)	49 (3.4%)	125 (4.3%)

Table 14.3.1 / 14: Number of subjects with adverse events by outcome, primary system organ class and preferred term (FAS)

Primary system organ class<br>Preferred term<br>MedDRA version 14.0	Maximum Derived AE outcome	LCS12<br>N=1432 (100%)	LCS16<br>N=1452 (100%)	Total<br>N=2884 (100%)
Abdominal adhesions	recovered/resolved	1 (<0.1%)	0	1 (<0.1%)
Abdominal discomfort	recovered/resolved	5 (0.3%)	5 (0.3%)	10 (0.3%)
Abdominal distension	recovered/resolved	8 (0.6%)	7 (0.5%)	15 (0.5%)
	recovering/resolving	2 (0.1%)	0	2 (<0.1%)
	not recovered/not resolved	8 (0.6%)	4 (0.3%)	12 (0.4%)
Abdominal hernia	recovered/resolved	2 (0.1%)	0	2 (<0.1%)
Abdominal mass	recovered/resolved	1 (<0.1%)	0	1 (<0.1%)
Abdominal pain	recovered/resolved	74 (5.2%)	84 (5.8%)	158 (5.5%)
	recovering/resolving	4 (0.3%)	6 (0.4%)	10 (0.3%)
	not recovered/not resolved	19 (1.3%)	11 (0.8%)	30 (1.0%)
Abdominal pain lower	recovered/resolved	47 (3.3%)	50 (3.4%)	97 (3.4%)
	recovering/resolving	2 (0.1%)	0	2 (<0.1%)
	not recovered/not resolved	16 (1.1%)	11 (0.8%)	27 (0.9%)
Abdominal pain upper	recovered/resolved	6 (0.4%)	11 (0.8%)	17 (0.6%)
	not recovered/not resolved	1 (<0.1%)	0	1 (<0.1%)
Abdominal tenderness	recovered/resolved	1 (<0.1%)	0	1 (<0.1%)
	recovering/resolving	1 (<0.1%)	0	1 (<0.1%)
Anal fissure	recovered/resolved	1 (<0.1%)	2 (0.1%)	3 (0.1%)
Anal pruritus	recovered/resolved	3 (0.2%)	2 (0.1%)	5 (0.2%)
Anal skin tags	recovered/resolved	0	1 (<0.1%)	1 (<0.1%)
Aphthous stomatitis	not recovered/not resolved	0	1 (<0.1%)	1 (<0.1%)
Breath odour	recovered/resolved	1 (<0.1%)	0	1 (<0.1%)
Coeliac disease	not recovered/not resolved	1 (<0.1%)	0	1 (<0.1%)

Table 14.3.1 / 14: Number of subjects with adverse events by outcome, primary system organ class and preferred term (FAS)

Primary system organ class<br>Preferred term<br>MedDRA version 14.0	Maximum Derived AE outcome	LCS12<br>N=1432 (100%)	LCS16<br>N=1452 (100%)	Total<br>N=2884 (100%)
Colitis	recovered/resolved	1 (<0.1%)	3 (0.2%)	4 (0.1%)
Colitis ulcerative	not recovered/not resolved	2 (0.1%)	0	2 (<0.1%)
Colonic polyp	recovered/resolved	1 (<0.1%)	0	1 (<0.1%)
Constipation	recovered/resolved	8 (0.6%)	11 (0.8%)	19 (0.7%)
	recovering/resolving	4 (0.3%)	2 (0.1%)	6 (0.2%)
	not recovered/not resolved	4 (0.3%)	4 (0.3%)	8 (0.3%)
Crohn's disease	recovering/resolving	1 (<0.1%)	0	1 (<0.1%)
	not recovered/not resolved	1 (<0.1%)	0	1 (<0.1%)
Dental caries	recovered/resolved	1 (<0.1%)	3 (0.2%)	4 (0.1%)
Diarrhoea	recovered/resolved	16 (1.1%)	24 (1.7%)	40 (1.4%)
	not recovered/not resolved	2 (0.1%)	2 (0.1%)	4 (0.1%)
Dry mouth	recovered/resolved	0	1 (<0.1%)	1 (<0.1%)
Duodenal ulcer	recovered/resolved	1 (<0.1%)	0	1 (<0.1%)
Dyspepsia	recovered/resolved	8 (0.6%)	8 (0.6%)	16 (0.6%)
	recovering/resolving	1 (<0.1%)	0	1 (<0.1%)
	not recovered/not resolved	5 (0.3%)	4 (0.3%)	9 (0.3%)
Dysphagia	recovered/resolved	1 (<0.1%)	1 (<0.1%)	2 (<0.1%)
Flatulence	recovered/resolved	2 (0.1%)	2 (0.1%)	4 (0.1%)
Food poisoning	recovered/resolved	1 (<0.1%)	6 (0.4%)	7 (0.2%)
Frequent bowel movements	recovering/resolving	1 (<0.1%)	0	1 (<0.1%)
	not recovered/not resolved	1 (<0.1%)	0	1 (<0.1%)
Gastric ulcer	recovered/resolved	2 (0.1%)	0	2 (<0.1%)
	not recovered/not resolved	0	1 (<0.1%)	1 (<0.1%)

Table 14.3.1 / 14: Number of subjects with adverse events by outcome, primary system organ class and preferred term (FAS)

Primary system organ class<br>Preferred term<br>MedDRA version 14.0	Maximum Derived AE outcome	LCS12<br>N=1432 (100%)	LCS16<br>N=1452 (100%)	Total<br>N=2884 (100%)
Gastritis	recovered/resolved	10 (0.7%)	14 (1.0%)	24 (0.8%)
	not recovered/not resolved	2 (0.1%)	2 (0.1%)	4 (0.1%)
Gastrointestinal disorder	recovered/resolved	1 (<0.1%)	0	1 (<0.1%)
Gastrointestinal pain	recovered/resolved	1 (<0.1%)	1 (<0.1%)	2 (<0.1%)
Gastrooesophageal reflux disease	recovered/resolved	4 (0.3%)	7 (0.5%)	11 (0.4%)
	recovering/resolving	1 (<0.1%)	0	1 (<0.1%)
	not recovered/not resolved	3 (0.2%)	3 (0.2%)	6 (0.2%)
Gingival oedema	recovered/resolved	1 (<0.1%)	0	1 (<0.1%)
Gingival recession	recovered/resolved	1 (<0.1%)	0	1 (<0.1%)
Gingivitis	recovered/resolved	1 (<0.1%)	1 (<0.1%)	2 (<0.1%)
Haematochezia	recovered/resolved	1 (<0.1%)	1 (<0.1%)	2 (<0.1%)
Haemorrhoids	recovered/resolved	6 (0.4%)	6 (0.4%)	12 (0.4%)
	not recovered/not resolved	1 (<0.1%)	1 (<0.1%)	2 (<0.1%)
Hiatus hernia	recovered/resolved	0	1 (<0.1%)	1 (<0.1%)
Hyperchlorhydria	recovered/resolved	2 (0.1%)	1 (<0.1%)	3 (0.1%)
Impaired gastric emptying	recovering/resolving	1 (<0.1%)	0	1 (<0.1%)
Inguinal hernia	recovered/resolved	1 (<0.1%)	0	1 (<0.1%)
Irritable bowel syndrome	recovered/resolved	4 (0.3%)	1 (<0.1%)	5 (0.2%)
	not recovered/not resolved	7 (0.5%)	4 (0.3%)	11 (0.4%)
Mouth ulceration	recovered/resolved	0	2 (0.1%)	2 (<0.1%)

Table 14.3.1 / 14: Number of subjects with adverse events by outcome, primary system organ class and preferred term (FAS)

Primary system organ class<br>Preferred term<br>MedDRA version 14.0	Maximum Derived AE outcome	LCS12<br>N=1432 (100%)	LCS16<br>N=1452 (100%)	Total<br>N=2884 (100%)
Nausea	recovered/resolved	61 (4.3%)	47 (3.2%)	108 (3.7%)
	recovering/resolving	1 (<0.1%)	2 (0.1%)	3 (0.1%)
	not recovered/not resolved	11 (0.8%)	5 (0.3%)	16 (0.6%)
Odynophagia	recovered/resolved	0	1 (<0.1%)	1 (<0.1%)
Oesophageal stenosis	not recovered/not resolved	0	1 (<0.1%)	1 (<0.1%)
Oesophagitis	recovering/resolving	1 (<0.1%)	0	1 (<0.1%)
Oral pain	recovered/resolved	0	1 (<0.1%)	1 (<0.1%)
Peptic ulcer	recovered/resolved	1 (<0.1%)	0	1 (<0.1%)
Periodontitis	recovered/resolved	2 (0.1%)	1 (<0.1%)	3 (0.1%)
Peritonitis	recovered/resolved	1 (<0.1%)	0	1 (<0.1%)
Proctalgia	recovered/resolved	2 (0.1%)	1 (<0.1%)	3 (0.1%)
Rectal haemorrhage	recovered/resolved	0	3 (0.2%)	3 (0.1%)
Reflux oesophagitis	not recovered/not resolved	0	1 (<0.1%)	1 (<0.1%)
Salivary gland calculus	not recovered/not resolved	0	1 (<0.1%)	1 (<0.1%)
Tooth disorder	recovered/resolved	3 (0.2%)	1 (<0.1%)	4 (0.1%)
Tooth impacted	recovered/resolved	2 (0.1%)	0	2 (<0.1%)
Toothache	recovered/resolved	17 (1.2%)	26 (1.8%)	43 (1.5%)
Umbilical hernia	recovered/resolved	0	1 (<0.1%)	1 (<0.1%)
Vomiting	recovered/resolved	15 (1.0%)	23 (1.6%)	38 (1.3%)
	recovering/resolving	1 (<0.1%)	0	1 (<0.1%)
	not recovered/not resolved	1 (<0.1%)	0	1 (<0.1%)

Table 14.3.1 / 14: Number of subjects with adverse events by outcome, primary system organ class and preferred term (FAS)

Primary system organ class<br>Preferred term<br>MedDRA version 14.0	Maximum Derived AE outcome	LCS12<br>N=1432 (100%)	LCS16<br>N=1452 (100%)	Total<br>N=2884 (100%)
General disorders and administration site conditions	recovered/resolved	96 (6.7%)	99 (6.8%)	195 (6.8%)
	recovering/resolving	2 (0.1%)	2 (0.1%)	4 (0.1%)
	not recovered/not resolved	12 (0.8%)	15 (1.0%)	27 (0.9%)
	recovered/resolved with resid. effects	1 (<0.1%)	0	1 (<0.1%)
Asthenia	recovered/resolved	1 (<0.1%)	1 (<0.1%)	2 (<0.1%)
	not recovered/not resolved	1 (<0.1%)	0	1 (<0.1%)
Axillary pain	recovered/resolved	0	1 (<0.1%)	1 (<0.1%)
	not recovered/not resolved	1 (<0.1%)	0	1 (<0.1%)
Chest pain	recovered/resolved	5 (0.3%)	7 (0.5%)	12 (0.4%)
Chills	recovered/resolved	0	2 (0.1%)	2 (<0.1%)
Cyst	recovered/resolved	2 (0.1%)	1 (<0.1%)	3 (0.1%)
	not recovered/not resolved	1 (<0.1%)	0	1 (<0.1%)
Device dislocation	recovered/resolved	3 (0.2%)	5 (0.3%)	8 (0.3%)
Device expulsion	recovered/resolved	46 (3.2%)	36 (2.5%)	82 (2.8%)
Discomfort	recovered/resolved	2 (0.1%)	0	2 (<0.1%)
	not recovered/not resolved	0	1 (<0.1%)	1 (<0.1%)
Fatigue	recovered/resolved	14 (1.0%)	19 (1.3%)	33 (1.1%)
	recovering/resolving	2 (0.1%)	0	2 (<0.1%)
	not recovered/not resolved	6 (0.4%)	8 (0.6%)	14 (0.5%)
Feeling cold	recovered/resolved	0	1 (<0.1%)	1 (<0.1%)
	not recovered/not resolved	0	1 (<0.1%)	1 (<0.1%)
Feeling hot	recovered/resolved	0	1 (<0.1%)	1 (<0.1%)
Generalised oedema	not recovered/not resolved	1 (<0.1%)	0	1 (<0.1%)
Hangover	recovered/resolved	1 (<0.1%)	0	1 (<0.1%)

Table 14.3.1 / 14: Number of subjects with adverse events by outcome, primary system organ class and preferred term (FAS)

Primary system organ class<br>Preferred term<br>MedDRA version 14.0	Maximum Derived AE outcome	LCS12<br>N=1432 (100%)	LCS16<br>N=1452 (100%)	Total<br>N=2884 (100%)
Hunger	recovered/resolved	0	1 (<0.1%)	1 (<0.1%)
Inflammation	recovered/resolved	1 (<0.1%)	1 (<0.1%)	2 (<0.1%)
Influenza like illness	recovered/resolved	1 (<0.1%)	1 (<0.1%)	2 (<0.1%)
Injury associated with device	recovered/resolved	0	1 (<0.1%)	1 (<0.1%)
Irritability	recovered/resolved	1 (<0.1%)	4 (0.3%)	5 (0.2%)
	not recovered/not resolved	1 (<0.1%)	1 (<0.1%)	2 (<0.1%)
Localised oedema	recovering/resolving	0	1 (<0.1%)	1 (<0.1%)
Oedema	not recovered/not resolved	0	1 (<0.1%)	1 (<0.1%)
Oedema peripheral	recovered/resolved	3 (0.2%)	4 (0.3%)	7 (0.2%)
	recovering/resolving	0	1 (<0.1%)	1 (<0.1%)
	not recovered/not resolved	1 (<0.1%)	1 (<0.1%)	2 (<0.1%)
Pain	recovered/resolved	4 (0.3%)	4 (0.3%)	8 (0.3%)
	not recovered/not resolved	1 (<0.1%)	1 (<0.1%)	2 (<0.1%)
	recovered/resolved with resid. effects	1 (<0.1%)	0	1 (<0.1%)
Pyrexia	recovered/resolved	18 (1.3%)	15 (1.0%)	33 (1.1%)
	not recovered/not resolved	0	1 (<0.1%)	1 (<0.1%)
Vaccination site pain	recovered/resolved	1 (<0.1%)	1 (<0.1%)	2 (<0.1%)
Hepatobiliary disorders	recovered/resolved	6 (0.4%)	9 (0.6%)	15 (0.5%)
	not recovered/not resolved	1 (<0.1%)	1 (<0.1%)	2 (<0.1%)
	recovered/resolved with resid. effects	1 (<0.1%)	0	1 (<0.1%)
Biliary colic	recovered/resolved	0	1 (<0.1%)	1 (<0.1%)
Biliary dyskinesia	recovered/resolved	1 (<0.1%)	1 (<0.1%)	2 (<0.1%)
Cholecystitis	recovered/resolved	2 (0.1%)	2 (0.1%)	4 (0.1%)
	recovered/resolved with resid. effects	1 (<0.1%)	0	1 (<0.1%)

Table 14.3.1 / 14: Number of subjects with adverse events by outcome, primary system organ class and preferred term (FAS)

Primary system organ class<br>Preferred term<br>MedDRA version 14.0	Maximum Derived AE outcome	LCS12<br>N=1432 (100%)	LCS16<br>N=1452 (100%)	Total<br>N=2884 (100%)
Cholecystitis chronic	recovered/resolved	2 (0.1%)	0	2 (<0.1%)
Cholelithiasis	recovered/resolved	3 (0.2%)	4 (0.3%)	7 (0.2%)
	not recovered/not resolved	1 (<0.1%)	0	1 (<0.1%)
Gallbladder pain	recovered/resolved	0	1 (<0.1%)	1 (<0.1%)
Gallbladder polyp	recovered/resolved	1 (<0.1%)	0	1 (<0.1%)
Hepatic steatosis	recovered/resolved	0	1 (<0.1%)	1 (<0.1%)
Hyperbilirubinaemia	not recovered/not resolved	0	1 (<0.1%)	1 (<0.1%)
Immune system disorders	recovered/resolved	50 (3.5%)	44 (3.0%)	94 (3.3%)
	not recovered/not resolved	10 (0.7%)	21 (1.4%)	31 (1.1%)
Allergy to animal	recovered/resolved	1 (<0.1%)	1 (<0.1%)	2 (<0.1%)
	not recovered/not resolved	0	1 (<0.1%)	1 (<0.1%)
Allergy to arthropod bite	recovered/resolved	1 (<0.1%)	1 (<0.1%)	2 (<0.1%)
Allergy to arthropod sting	recovered/resolved	0	1 (<0.1%)	1 (<0.1%)
Allergy to chemicals	recovered/resolved	1 (<0.1%)	0	1 (<0.1%)
Anaphylactic reaction	recovered/resolved	0	1 (<0.1%)	1 (<0.1%)
Drug hypersensitivity	recovered/resolved	7 (0.5%)	6 (0.4%)	13 (0.5%)
	not recovered/not resolved	1 (<0.1%)	0	1 (<0.1%)
Food allergy	recovered/resolved	1 (<0.1%)	1 (<0.1%)	2 (<0.1%)
Hypersensitivity	recovered/resolved	13 (0.9%)	8 (0.6%)	21 (0.7%)
	not recovered/not resolved	0	3 (0.2%)	3 (0.1%)
Seasonal allergy	recovered/resolved	28 (2.0%)	26 (1.8%)	54 (1.9%)
	not recovered/not resolved	9 (0.6%)	17 (1.2%)	26 (0.9%)

Table 14.3.1 / 14: Number of subjects with adverse events by outcome, primary system organ class and preferred term (FAS)

Primary system organ class<br>Preferred term<br>MedDRA version 14.0	Maximum Derived AE outcome	LCS12<br>N=1432 (100%)	LCS16<br>N=1452 (100%)	Total<br>N=2884 (100%)
Infections and infestations	recovered/resolved	653 (45.6%)	683 (47.0%)	1336 (46.3%)
	recovering/resolving	8 (0.6%)	8 (0.6%)	16 (0.6%)
	not recovered/not resolved	55 (3.8%)	42 (2.9%)	97 (3.4%)
	recovered/resolved with resid. effects	3 (0.2%)	1 (<0.1%)	4 (0.1%)
Abscess limb	recovered/resolved	0	1 (<0.1%)	1 (<0.1%)
Acarodermatitis	recovered/resolved	2 (0.1%)	0	2 (<0.1%)
Acute sinusitis	recovered/resolved	6 (0.4%)	3 (0.2%)	9 (0.3%)
	not recovered/not resolved	1 (<0.1%)	0	1 (<0.1%)
Acute tonsillitis	Continuing with change	0	1 (<0.1%)	1 (<0.1%)
	recovered/resolved	9 (0.6%)	4 (0.3%)	13 (0.5%)
	recovering/resolving	1 (<0.1%)	0	1 (<0.1%)
Anogenital warts	recovered/resolved	10 (0.7%)	9 (0.6%)	19 (0.7%)
	not recovered/not resolved	1 (<0.1%)	1 (<0.1%)	2 (<0.1%)
Appendicitis	recovered/resolved	6 (0.4%)	6 (0.4%)	12 (0.4%)
	recovered/resolved with resid. effects	0	1 (<0.1%)	1 (<0.1%)
Arthritis rubella	recovered/resolved	1 (<0.1%)	0	1 (<0.1%)
Bartholin's abscess	recovered/resolved	2 (0.1%)	0	2 (<0.1%)
Blastocystis infection	recovered/resolved	0	1 (<0.1%)	1 (<0.1%)
Body tinea	recovered/resolved	1 (<0.1%)	2 (0.1%)	3 (0.1%)
	not recovered/not resolved	1 (<0.1%)	0	1 (<0.1%)
Bronchitis	recovered/resolved	57 (4.0%)	38 (2.6%)	95 (3.3%)
	not recovered/not resolved	1 (<0.1%)	0	1 (<0.1%)
Bronchitis viral	recovered/resolved	1 (<0.1%)	0	1 (<0.1%)
Bronchopneumonia	recovered/resolved	1 (<0.1%)	0	1 (<0.1%)

Table 14.3.1 / 14: Number of subjects with adverse events by outcome, primary system organ class and preferred term (FAS)

Primary system organ class<br>Preferred term<br>MedDRA version 14.0	Maximum Derived AE outcome	LCS12<br>N=1432 (100%)	LCS16<br>N=1452 (100%)	Total<br>N=2884 (100%)
Bursitis infective	recovered/resolved	1 (<0.1%)	0	1 (<0.1%)
Campylobacter infection	recovered/resolved	1 (<0.1%)	0	1 (<0.1%)
Campylobacter intestinal infection	recovered/resolved	0	1 (<0.1%)	1 (<0.1%)
Candidiasis	recovered/resolved	9 (0.6%)	8 (0.6%)	17 (0.6%)
	not recovered/not resolved	2 (0.1%)	1 (<0.1%)	3 (0.1%)
Carbuncle	recovered/resolved	0	1 (<0.1%)	1 (<0.1%)
Cellulitis	recovered/resolved	3 (0.2%)	4 (0.3%)	7 (0.2%)
Cervicitis	recovered/resolved	5 (0.3%)	14 (1.0%)	19 (0.7%)
	not recovered/not resolved	0	1 (<0.1%)	1 (<0.1%)
Cervicitis gonococcal	recovered/resolved	1 (<0.1%)	0	1 (<0.1%)
Chlamydial cervicitis	recovered/resolved	2 (0.1%)	5 (0.3%)	7 (0.2%)
	not recovered/not resolved	0	1 (<0.1%)	1 (<0.1%)
Chlamydial infection	recovered/resolved	1 (<0.1%)	4 (0.3%)	5 (0.2%)
Chronic tonsillitis	recovered/resolved	0	1 (<0.1%)	1 (<0.1%)
Clitoris abscess	recovered/resolved	1 (<0.1%)	0	1 (<0.1%)
Conjunctivitis bacterial	recovered/resolved	1 (<0.1%)	2 (0.1%)	3 (0.1%)
Conjunctivitis infective	recovered/resolved	2 (0.1%)	0	2 (<0.1%)
Conjunctivitis viral	recovered/resolved	1 (<0.1%)	1 (<0.1%)	2 (<0.1%)
Coxsackie viral infection	recovered/resolved	2 (0.1%)	1 (<0.1%)	3 (0.1%)
Cystitis	recovered/resolved	30 (2.1%)	25 (1.7%)	55 (1.9%)
	recovering/resolving	0	1 (<0.1%)	1 (<0.1%)

Table 14.3.1 / 14: Number of subjects with adverse events by outcome, primary system organ class and preferred term (FAS)

Primary system organ class<br>Preferred term<br>MedDRA version 14.0	Maximum Derived AE outcome	LCS12<br>N=1432 (100%)	LCS16<br>N=1452 (100%)	Total<br>N=2884 (100%)
Dengue fever	recovered/resolved	2 (0.1%)	0	2 (<0.1%)
Diarrhoea infectious	recovered/resolved	1 (<0.1%)	2 (0.1%)	3 (0.1%)
Diverticulitis	recovered/resolved	1 (<0.1%)	1 (<0.1%)	2 (<0.1%)
Ear infection	recovered/resolved	27 (1.9%)	11 (0.8%)	38 (1.3%)
	not recovered/not resolved	0	2 (0.1%)	2 (<0.1%)
Ear infection bacterial	recovered/resolved	1 (<0.1%)	0	1 (<0.1%)
Endometritis	recovered/resolved	9 (0.6%)	10 (0.7%)	19 (0.7%)
	recovering/resolving	0	1 (<0.1%)	1 (<0.1%)
	not recovered/not resolved	2 (0.1%)	0	2 (<0.1%)
Enterobiasis	recovered/resolved	3 (0.2%)	1 (<0.1%)	4 (0.1%)
Enterocolitis infectious	recovered/resolved	2 (0.1%)	0	2 (<0.1%)
Epstein-Barr virus infection	recovered/resolved	0	1 (<0.1%)	1 (<0.1%)
Erysipelas	recovered/resolved	1 (<0.1%)	0	1 (<0.1%)
Erythema infectiosum	recovered/resolved	1 (<0.1%)	0	1 (<0.1%)
Escherichia urinary tract infection	recovered/resolved	0	1 (<0.1%)	1 (<0.1%)
Escherichia vaginitis	recovered/resolved	1 (<0.1%)	0	1 (<0.1%)
Eye infection	recovered/resolved	0	1 (<0.1%)	1 (<0.1%)
Eyelid infection	recovered/resolved	1 (<0.1%)	0	1 (<0.1%)
Folliculitis	recovered/resolved	3 (0.2%)	5 (0.3%)	8 (0.3%)
	not recovered/not resolved	2 (0.1%)	1 (<0.1%)	3 (0.1%)

Table 14.3.1 / 14: Number of subjects with adverse events by outcome, primary system organ class and preferred term (FAS)

Primary system organ class<br>Preferred term<br>MedDRA version 14.0	Maximum Derived AE outcome	LCS12<br>N=1432 (100%)	LCS16<br>N=1452 (100%)	Total<br>N=2884 (100%)
Fungal infection	recovered/resolved	5 (0.3%)	7 (0.5%)	12 (0.4%)
	not recovered/not resolved	0	1 (<0.1%)	1 (<0.1%)
Fungal skin infection	recovered/resolved	3 (0.2%)	1 (<0.1%)	4 (0.1%)
Furuncle	recovered/resolved	1 (<0.1%)	1 (<0.1%)	2 (<0.1%)
Gastritis bacterial	recovered/resolved	0	1 (<0.1%)	1 (<0.1%)
Gastritis viral	recovered/resolved	0	1 (<0.1%)	1 (<0.1%)
Gastroenteritis	Continuing with change	0	1 (<0.1%)	1 (<0.1%)
	recovered/resolved	31 (2.2%)	28 (1.9%)	59 (2.0%)
Gastroenteritis rotavirus	recovered/resolved	0	1 (<0.1%)	1 (<0.1%)
Gastroenteritis viral	recovered/resolved	7 (0.5%)	8 (0.6%)	15 (0.5%)
Gastrointestinal bacterial infection	recovered/resolved	0	1 (<0.1%)	1 (<0.1%)
Gastrointestinal viral infection	recovered/resolved	1 (<0.1%)	2 (0.1%)	3 (0.1%)
Genital candidiasis	recovered/resolved	1 (<0.1%)	2 (0.1%)	3 (0.1%)
	not recovered/not resolved	0	1 (<0.1%)	1 (<0.1%)
Genital herpes	recovered/resolved	8 (0.6%)	12 (0.8%)	20 (0.7%)
	not recovered/not resolved	2 (0.1%)	2 (0.1%)	4 (0.1%)
Genital infection fungal	recovered/resolved	1 (<0.1%)	0	1 (<0.1%)
Giardiasis	recovered/resolved	1 (<0.1%)	0	1 (<0.1%)
Gonorrhoea	recovered/resolved	1 (<0.1%)	1 (<0.1%)	2 (<0.1%)
Groin infection	recovered/resolved	2 (0.1%)	0	2 (<0.1%)

Table 14.3.1 / 14: Number of subjects with adverse events by outcome, primary system organ class and preferred term (FAS)

Primary system organ class<br>Preferred term<br>MedDRA version 14.0	Maximum Derived AE outcome	LCS12<br>N=1432 (100%)	LCS16<br>N=1452 (100%)	Total<br>N=2884 (100%)
Gynaecological chlamydia infection	recovered/resolved	2 (0.1%)	5 (0.3%)	7 (0.2%)
	not recovered/not resolved	1 (<0.1%)	0	1 (<0.1%)
H1N1 influenza	recovered/resolved	7 (0.5%)	5 (0.3%)	12 (0.4%)
Haemophilus infection	recovered/resolved	0	1 (<0.1%)	1 (<0.1%)
Hand-foot-and-mouth disease	recovered/resolved	0	1 (<0.1%)	1 (<0.1%)
Helicobacter gastritis	recovered/resolved	1 (<0.1%)	1 (<0.1%)	2 (<0.1%)
Helicobacter infection	recovered/resolved	1 (<0.1%)	0	1 (<0.1%)
Herpes dermatitis	recovered/resolved	1 (<0.1%)	1 (<0.1%)	2 (<0.1%)
Herpes simplex	recovered/resolved	1 (<0.1%)	1 (<0.1%)	2 (<0.1%)
	not recovered/not resolved	0	2 (0.1%)	2 (<0.1%)
Herpes zoster	recovered/resolved	7 (0.5%)	4 (0.3%)	11 (0.4%)
Impetigo	recovered/resolved	3 (0.2%)	2 (0.1%)	5 (0.2%)
	recovering/resolving	1 (<0.1%)	0	1 (<0.1%)
Infected bites	recovered/resolved	2 (0.1%)	0	2 (<0.1%)
Infected cyst	recovered/resolved	0	1 (<0.1%)	1 (<0.1%)
Infection	recovered/resolved	0	1 (<0.1%)	1 (<0.1%)
Infectious mononucleosis	recovered/resolved	0	6 (0.4%)	6 (0.2%)
	not recovered/not resolved	1 (<0.1%)	0	1 (<0.1%)
Influenza	Continuing with change	0	1 (<0.1%)	1 (<0.1%)
	recovered/resolved	70 (4.9%)	93 (6.4%)	163 (5.7%)
	not recovered/not resolved	1 (<0.1%)	0	1 (<0.1%)
	recovered/resolved with resid. effects	1 (<0.1%)	0	1 (<0.1%)

Table 14.3.1 / 14: Number of subjects with adverse events by outcome, primary system organ class and preferred term (FAS)

Primary system organ class<br>Preferred term<br>MedDRA version 14.0	Maximum Derived AE outcome	LCS12<br>N=1432 (100%)	LCS16<br>N=1452 (100%)	Total<br>N=2884 (100%)
Keratitis viral	recovered/resolved	0	1 (<0.1%)	1 (<0.1%)
Kidney infection	recovered/resolved	2 (0.1%)	4 (0.3%)	6 (0.2%)
Labyrinthitis	recovered/resolved	1 (<0.1%)	1 (<0.1%)	2 (<0.1%)
Laryngitis	recovered/resolved	7 (0.5%)	10 (0.7%)	17 (0.6%)
Laryngitis bacterial	recovered/resolved	0	1 (<0.1%)	1 (<0.1%)
Localised infection	recovered/resolved	3 (0.2%)	2 (0.1%)	5 (0.2%)
	recovering/resolving	1 (<0.1%)	0	1 (<0.1%)
Lower respiratory tract infection	recovered/resolved	3 (0.2%)	1 (<0.1%)	4 (0.1%)
Lower respiratory tract infection bacterial	recovered/resolved	1 (<0.1%)	0	1 (<0.1%)
Lymph gland infection	recovered/resolved	1 (<0.1%)	0	1 (<0.1%)
Malaria	recovered/resolved	1 (<0.1%)	1 (<0.1%)	2 (<0.1%)
Mastitis	recovered/resolved	3 (0.2%)	1 (<0.1%)	4 (0.1%)
Meningitis	recovered/resolved	1 (<0.1%)	0	1 (<0.1%)
Meningitis viral	recovered/resolved	0	1 (<0.1%)	1 (<0.1%)
Molluscum contagiosum	not recovered/not resolved	0	1 (<0.1%)	1 (<0.1%)
Nail infection	recovered/resolved	2 (0.1%)	1 (<0.1%)	3 (0.1%)
Nasopharyngitis	Continuing with change	1 (<0.1%)	1 (<0.1%)	2 (<0.1%)
	recovered/resolved	100 (7.0%)	106 (7.3%)	206 (7.1%)
	recovering/resolving	1 (<0.1%)	1 (<0.1%)	2 (<0.1%)
	not recovered/not resolved	1 (<0.1%)	1 (<0.1%)	2 (<0.1%)
Nipple infection	recovering/resolving	0	1 (<0.1%)	1 (<0.1%)

Table 14.3.1 / 14: Number of subjects with adverse events by outcome, primary system organ class and preferred term (FAS)

Primary system organ class<br>Preferred term<br>MedDRA version 14.0	Maximum Derived AE outcome	LCS12<br>N=1432 (100%)	LCS16<br>N=1452 (100%)	Total<br>N=2884 (100%)
Omphalitis	recovered/resolved	0	1 (<0.1%)	1 (<0.1%)
Onychomycosis	recovered/resolved	2 (0.1%)	3 (0.2%)	5 (0.2%)
	not recovered/not resolved	1 (<0.1%)	1 (<0.1%)	2 (<0.1%)
Oophoritis	recovered/resolved	0	1 (<0.1%)	1 (<0.1%)
Oral candidiasis	recovered/resolved	0	1 (<0.1%)	1 (<0.1%)
Oral herpes	recovered/resolved	9 (0.6%)	9 (0.6%)	18 (0.6%)
	not recovered/not resolved	2 (0.1%)	1 (<0.1%)	3 (0.1%)
Osteomyelitis	recovered/resolved	0	1 (<0.1%)	1 (<0.1%)
Otitis externa	recovered/resolved	0	3 (0.2%)	3 (0.1%)
Otitis media	recovered/resolved	5 (0.3%)	6 (0.4%)	11 (0.4%)
Otitis media acute	recovered/resolved	0	1 (<0.1%)	1 (<0.1%)
Papilloma viral infection	recovered/resolved	1 (<0.1%)	1 (<0.1%)	2 (<0.1%)
Parasitic gastroenteritis	recovered/resolved	1 (<0.1%)	0	1 (<0.1%)
Paronychia	recovered/resolved	1 (<0.1%)	2 (0.1%)	3 (0.1%)
Pelvic infection	recovered/resolved	0	1 (<0.1%)	1 (<0.1%)
Pelvic inflammatory disease	recovered/resolved	4 (0.3%)	5 (0.3%)	9 (0.3%)
	not recovered/not resolved	1 (<0.1%)	0	1 (<0.1%)
Peritoneal abscess	recovered/resolved	1 (<0.1%)	0	1 (<0.1%)
Peritonsillar abscess	recovered/resolved	2 (0.1%)	1 (<0.1%)	3 (0.1%)
Pharyngeal abscess	recovered/resolved	1 (<0.1%)	0	1 (<0.1%)

Table 14.3.1 / 14: Number of subjects with adverse events by outcome, primary system organ class and preferred term (FAS)

Primary system organ class<br>Preferred term<br>MedDRA version 14.0	Maximum Derived AE outcome	LCS12<br>N=1432 (100%)	LCS16<br>N=1452 (100%)	Total<br>N=2884 (100%)
Pharyngitis	recovered/resolved	13 (0.9%)	21 (1.4%)	34 (1.2%)
Pharyngitis streptococcal	recovered/resolved	23 (1.6%)	20 (1.4%)	43 (1.5%)
	not recovered/not resolved	0	1 (<0.1%)	1 (<0.1%)
Pharyngotonsillitis	recovered/resolved	1 (<0.1%)	0	1 (<0.1%)
Pneumonia	recovered/resolved	13 (0.9%)	14 (1.0%)	27 (0.9%)
	recovering/resolving	0	1 (<0.1%)	1 (<0.1%)
	recovered/resolved with resid. effects	1 (<0.1%)	0	1 (<0.1%)
Pneumonia mycoplasmal	recovered/resolved	1 (<0.1%)	0	1 (<0.1%)
Pogosta disease	recovered/resolved	1 (<0.1%)	0	1 (<0.1%)
Post procedural infection	recovered/resolved	2 (0.1%)	1 (<0.1%)	3 (0.1%)
	recovered/resolved with resid. effects	1 (<0.1%)	0	1 (<0.1%)
Pulpitis dental	recovered/resolved	0	1 (<0.1%)	1 (<0.1%)
Pyelonephritis	recovered/resolved	8 (0.6%)	3 (0.2%)	11 (0.4%)
	not recovered/not resolved	1 (<0.1%)	0	1 (<0.1%)
Pyelonephritis acute	recovered/resolved	2 (0.1%)	0	2 (<0.1%)
Q fever	recovered/resolved	1 (<0.1%)	0	1 (<0.1%)
Rash pustular	not recovered/not resolved	0	1 (<0.1%)	1 (<0.1%)
Respiratory tract infection	recovered/resolved	10 (0.7%)	15 (1.0%)	25 (0.9%)
	not recovered/not resolved	0	1 (<0.1%)	1 (<0.1%)
Respiratory tract infection viral	recovered/resolved	2 (0.1%)	1 (<0.1%)	3 (0.1%)
Rhinitis	recovered/resolved	6 (0.4%)	11 (0.8%)	17 (0.6%)
	not recovered/not resolved	0	1 (<0.1%)	1 (<0.1%)

Table 14.3.1 / 14: Number of subjects with adverse events by outcome, primary system organ class and preferred term (FAS)

Primary system organ class<br>Preferred term<br>MedDRA version 14.0	Maximum Derived AE outcome	LCS12<br>N=1432 (100%)	LCS16<br>N=1452 (100%)	Total<br>N=2884 (100%)
Salpingitis	not recovered/not resolved	1 (<0.1%)	0	1 (<0.1%)
Salpingo-oophoritis	recovered/resolved	2 (0.1%)	3 (0.2%)	5 (0.2%)
Sialoadenitis	recovered/resolved	1 (<0.1%)	0	1 (<0.1%)
Sinobronchitis	recovered/resolved	0	1 (<0.1%)	1 (<0.1%)
Sinusitis	recovered/resolved	86 (6.0%)	94 (6.5%)	180 (6.2%)
	recovering/resolving	1 (<0.1%)	1 (<0.1%)	2 (<0.1%)
	not recovered/not resolved	3 (0.2%)	1 (<0.1%)	4 (0.1%)
Skin bacterial infection	recovered/resolved	1 (<0.1%)	2 (0.1%)	3 (0.1%)
Skin infection	recovered/resolved	1 (<0.1%)	6 (0.4%)	7 (0.2%)
Small intestinal bacterial overgrowth	not recovered/not resolved	0	1 (<0.1%)	1 (<0.1%)
Staphylococcal infection	recovered/resolved	1 (<0.1%)	1 (<0.1%)	2 (<0.1%)
Staphylococcal skin infection	recovered/resolved	1 (<0.1%)	0	1 (<0.1%)
Streptococcal infection	recovered/resolved	1 (<0.1%)	0	1 (<0.1%)
Streptococcal sepsis	recovered/resolved	1 (<0.1%)	0	1 (<0.1%)
Subcutaneous abscess	recovered/resolved	3 (0.2%)	2 (0.1%)	5 (0.2%)
Sweat gland infection	recovered/resolved	1 (<0.1%)	0	1 (<0.1%)
Tinea infection	recovered/resolved	1 (<0.1%)	2 (0.1%)	3 (0.1%)
Tinea pedis	recovered/resolved	0	1 (<0.1%)	1 (<0.1%)
	not recovered/not resolved	1 (<0.1%)	0	1 (<0.1%)
Tinea versicolour	recovered/resolved	0	1 (<0.1%)	1 (<0.1%)

Table 14.3.1 / 14: Number of subjects with adverse events by outcome, primary system organ class and preferred term (FAS)

Primary system organ class<br>Preferred term<br>MedDRA version 14.0	Maximum Derived AE outcome	LCS12<br>N=1432 (100%)	LCS16<br>N=1452 (100%)	Total<br>N=2884 (100%)
Tonsillitis	recovered/resolved	24 (1.7%)	22 (1.5%)	46 (1.6%)
	recovering/resolving	0	1 (<0.1%)	1 (<0.1%)
	not recovered/not resolved	1 (<0.1%)	0	1 (<0.1%)
Tonsillitis streptococcal	recovered/resolved	1 (<0.1%)	0	1 (<0.1%)
Tooth abscess	recovered/resolved	5 (0.3%)	4 (0.3%)	9 (0.3%)
Tooth infection	recovered/resolved	24 (1.7%)	15 (1.0%)	39 (1.4%)
	recovering/resolving	1 (<0.1%)	0	1 (<0.1%)
Tracheitis	recovered/resolved	1 (<0.1%)	0	1 (<0.1%)
Trichomoniasis	recovered/resolved	2 (0.1%)	2 (0.1%)	4 (0.1%)
Tuberculosis	recovered/resolved	1 (<0.1%)	0	1 (<0.1%)
Tubo-ovarian abscess	recovered/resolved	1 (<0.1%)	0	1 (<0.1%)
Typhoid fever	recovered/resolved	0	1 (<0.1%)	1 (<0.1%)
Upper respiratory tract infection	recovered/resolved	50 (3.5%)	56 (3.9%)	106 (3.7%)
	not recovered/not resolved	0	1 (<0.1%)	1 (<0.1%)
Ureaplasma infection	recovered/resolved	1 (<0.1%)	0	1 (<0.1%)
Urinary tract infection	recovered/resolved	149 (10.4%)	138 (9.5%)	287 (10.0%)
	recovering/resolving	1 (<0.1%)	1 (<0.1%)	2 (<0.1%)
	not recovered/not resolved	8 (0.6%)	3 (0.2%)	11 (0.4%)
Uterine infection	recovered/resolved	0	1 (<0.1%)	1 (<0.1%)
Vaginal infection	recovered/resolved	45 (3.1%)	58 (4.0%)	103 (3.6%)
	recovering/resolving	1 (<0.1%)	0	1 (<0.1%)
	not recovered/not resolved	2 (0.1%)	2 (0.1%)	4 (0.1%)

Table 14.3.1 / 14: Number of subjects with adverse events by outcome, primary system organ class and preferred term (FAS)

Primary system organ class<br>Preferred term<br>MedDRA version 14.0	Maximum Derived AE outcome	LCS12<br>N=1432 (100%)	LCS16<br>N=1452 (100%)	Total<br>N=2884 (100%)
Vaginitis bacterial	recovered/resolved	94 (6.6%)	119 (8.2%)	213 (7.4%)
	recovering/resolving	0	1 (<0.1%)	1 (<0.1%)
	not recovered/not resolved	10 (0.7%)	7 (0.5%)	17 (0.6%)
Vaginitis chlamydial	recovered/resolved	2 (0.1%)	1 (<0.1%)	3 (0.1%)
Vaginitis gardnerella	recovered/resolved	0	3 (0.2%)	3 (0.1%)
	not recovered/not resolved	0	1 (<0.1%)	1 (<0.1%)
Vestibular neuronitis	recovered/resolved	2 (0.1%)	0	2 (<0.1%)
Viral infection	recovered/resolved	3 (0.2%)	3 (0.2%)	6 (0.2%)
Viral pharyngitis	recovered/resolved	1 (<0.1%)	1 (<0.1%)	2 (<0.1%)
Viral rhinitis	recovered/resolved	1 (<0.1%)	0	1 (<0.1%)
Viral upper respiratory tract infection	recovered/resolved	15 (1.0%)	14 (1.0%)	29 (1.0%)
Vulval abscess	recovered/resolved	0	1 (<0.1%)	1 (<0.1%)
Vulvitis	recovered/resolved	7 (0.5%)	7 (0.5%)	14 (0.5%)
	not recovered/not resolved	1 (<0.1%)	0	1 (<0.1%)
Vulvovaginal candidiasis	recovered/resolved	66 (4.6%)	70 (4.8%)	136 (4.7%)
	recovering/resolving	0	1 (<0.1%)	1 (<0.1%)
	not recovered/not resolved	6 (0.4%)	1 (<0.1%)	7 (0.2%)
Vulvovaginal mycotic infection	recovered/resolved	94 (6.6%)	103 (7.1%)	197 (6.8%)
	recovering/resolving	0	1 (<0.1%)	1 (<0.1%)
	not recovered/not resolved	4 (0.3%)	6 (0.4%)	10 (0.3%)
Vulvovaginitis	recovered/resolved	6 (0.4%)	5 (0.3%)	11 (0.4%)
	not recovered/not resolved	0	2 (0.1%)	2 (<0.1%)
Vulvovaginitis chlamydial	recovered/resolved	0	1 (<0.1%)	1 (<0.1%)
Vulvovaginitis streptococcal	recovered/resolved	1 (<0.1%)	2 (0.1%)	3 (0.1%)

Table 14.3.1 / 14: Number of subjects with adverse events by outcome, primary system organ class and preferred term (FAS)

Primary system organ class<br>Preferred term<br>MedDRA version 14.0	Maximum Derived AE outcome	LCS12<br>N=1432 (100%)	LCS16<br>N=1452 (100%)	Total<br>N=2884 (100%)
Vulvovaginitis trichomonal	recovered/resolved	4 (0.3%)	4 (0.3%)	8 (0.3%)
Wound infection	recovered/resolved	3 (0.2%)	1 (<0.1%)	4 (0.1%)
Wound infection staphylococcal	recovered/resolved	0	1 (<0.1%)	1 (<0.1%)
Injury, poisoning and procedural complications	recovered/resolved	141 (9.8%)	121 (8.3%)	262 (9.1%)
	recovering/resolving	2 (0.1%)	6 (0.4%)	8 (0.3%)
	not recovered/not resolved	5 (0.3%)	5 (0.3%)	10 (0.3%)
	recovered/resolved with resid. effects	1 (<0.1%)	0	1 (<0.1%)
Abdominal wound dehiscence	recovering/resolving	0	1 (<0.1%)	1 (<0.1%)
Accident	recovered/resolved	0	1 (<0.1%)	1 (<0.1%)
Animal bite	recovered/resolved	3 (0.2%)	2 (0.1%)	5 (0.2%)
Ankle fracture	recovered/resolved	1 (<0.1%)	2 (0.1%)	3 (0.1%)
Arthropod bite	recovered/resolved	5 (0.3%)	2 (0.1%)	7 (0.2%)
Arthropod sting	recovered/resolved	0	2 (0.1%)	2 (<0.1%)
Back injury	not recovered/not resolved	0	1 (<0.1%)	1 (<0.1%)
Burns second degree	recovered/resolved	0	1 (<0.1%)	1 (<0.1%)
Burns third degree	recovered/resolved	1 (<0.1%)	0	1 (<0.1%)
Cartilage injury	recovered/resolved	0	1 (<0.1%)	1 (<0.1%)
Clavicle fracture	recovered/resolved	1 (<0.1%)	0	1 (<0.1%)
Concussion	recovered/resolved	3 (0.2%)	8 (0.6%)	11 (0.4%)
Contusion	recovered/resolved	7 (0.5%)	4 (0.3%)	11 (0.4%)
	recovering/resolving	0	1 (<0.1%)	1 (<0.1%)

Table 14.3.1 / 14: Number of subjects with adverse events by outcome, primary system organ class and preferred term (FAS)

Primary system organ class<br>Preferred term<br>MedDRA version 14.0	Maximum Derived AE outcome	LCS12<br>N=1432 (100%)	LCS16<br>N=1452 (100%)	Total<br>N=2884 (100%)
Epicondylitis	recovered/resolved	2 (0.1%)	4 (0.3%)	6 (0.2%)
Excoriation	recovered/resolved	0	1 (<0.1%)	1 (<0.1%)
Eye injury	recovered/resolved	1 (<0.1%)	1 (<0.1%)	2 (<0.1%)
Facial bones fracture	recovered/resolved	1 (<0.1%)	0	1 (<0.1%)
	not recovered/not resolved	1 (<0.1%)	0	1 (<0.1%)
Fall	recovered/resolved	0	1 (<0.1%)	1 (<0.1%)
Foot fracture	recovered/resolved	9 (0.6%)	3 (0.2%)	12 (0.4%)
Forearm fracture	recovered/resolved with resid. effects	1 (<0.1%)	0	1 (<0.1%)
Frostbite	recovered/resolved	1 (<0.1%)	0	1 (<0.1%)
Hand fracture	recovered/resolved	0	3 (0.2%)	3 (0.1%)
	not recovered/not resolved	1 (<0.1%)	0	1 (<0.1%)
Head injury	recovered/resolved	1 (<0.1%)	1 (<0.1%)	2 (<0.1%)
Humerus fracture	recovered/resolved	0	1 (<0.1%)	1 (<0.1%)
Injury	recovered/resolved	0	1 (<0.1%)	1 (<0.1%)
Joint dislocation	recovered/resolved	5 (0.3%)	1 (<0.1%)	6 (0.2%)
Joint injury	Continuing with change	1 (<0.1%)	0	1 (<0.1%)
	recovered/resolved	6 (0.4%)	6 (0.4%)	12 (0.4%)
Joint sprain	recovered/resolved	11 (0.8%)	6 (0.4%)	17 (0.6%)
	not recovered/not resolved	2 (0.1%)	0	2 (<0.1%)
Laceration	recovered/resolved	3 (0.2%)	2 (0.1%)	5 (0.2%)
Laryngeal injury	recovered/resolved	1 (<0.1%)	0	1 (<0.1%)

Table 14.3.1 / 14: Number of subjects with adverse events by outcome, primary system organ class and preferred term (FAS)

Primary system organ class<br>Preferred term<br>MedDRA version 14.0	Maximum Derived AE outcome	LCS12<br>N=1432 (100%)	LCS16<br>N=1452 (100%)	Total<br>N=2884 (100%)
Ligament rupture	recovered/resolved	3 (0.2%)	1 (<0.1%)	4 (0.1%)
Ligament sprain	recovered/resolved	0	1 (<0.1%)	1 (<0.1%)
Limb crushing injury	recovered/resolved	1 (<0.1%)	0	1 (<0.1%)
Limb injury	recovered/resolved	1 (<0.1%)	4 (0.3%)	5 (0.2%)
Lower limb fracture	recovered/resolved	1 (<0.1%)	0	1 (<0.1%)
Lumbar vertebral fracture	recovering/resolving	0	1 (<0.1%)	1 (<0.1%)
Meniscus lesion	recovered/resolved	3 (0.2%)	0	3 (0.1%)
Muscle injury	recovered/resolved	4 (0.3%)	2 (0.1%)	6 (0.2%)
	not recovered/not resolved	1 (<0.1%)	0	1 (<0.1%)
Muscle rupture	recovered/resolved	0	1 (<0.1%)	1 (<0.1%)
Muscle strain	recovered/resolved	6 (0.4%)	7 (0.5%)	13 (0.5%)
	recovering/resolving	1 (<0.1%)	1 (<0.1%)	2 (<0.1%)
	not recovered/not resolved	0	2 (0.1%)	2 (<0.1%)
Nail avulsion	recovered/resolved	1 (<0.1%)	0	1 (<0.1%)
Overdose	recovering/resolving	1 (<0.1%)	0	1 (<0.1%)
Post concussion syndrome	recovering/resolving	0	1 (<0.1%)	1 (<0.1%)
Post procedural haemorrhage	recovered/resolved	2 (0.1%)	2 (0.1%)	4 (0.1%)
Post-traumatic pain	recovered/resolved	2 (0.1%)	4 (0.3%)	6 (0.2%)
	not recovered/not resolved	1 (<0.1%)	0	1 (<0.1%)
Postoperative ileus	recovered/resolved	0	1 (<0.1%)	1 (<0.1%)

Table 14.3.1 / 14: Number of subjects with adverse events by outcome, primary system organ class and preferred term (FAS)

Primary system organ class<br>Preferred term<br>MedDRA version 14.0	Maximum Derived AE outcome	LCS12<br>N=1432 (100%)	LCS16<br>N=1452 (100%)	Total<br>N=2884 (100%)
Procedural pain	recovered/resolved	58 (4.1%)	51 (3.5%)	109 (3.8%)
	recovering/resolving	0	1 (<0.1%)	1 (<0.1%)
	not recovered/not resolved	0	1 (<0.1%)	1 (<0.1%)
Procedural vomiting	recovered/resolved	1 (<0.1%)	0	1 (<0.1%)
Radius fracture	recovered/resolved	1 (<0.1%)	1 (<0.1%)	2 (<0.1%)
Rib fracture	recovered/resolved	1 (<0.1%)	2 (0.1%)	3 (0.1%)
Road traffic accident	recovered/resolved	3 (0.2%)	2 (0.1%)	5 (0.2%)
Seroma	recovered/resolved	1 (<0.1%)	0	1 (<0.1%)
Shunt occlusion	recovered/resolved	1 (<0.1%)	0	1 (<0.1%)
Skeletal injury	recovered/resolved	0	1 (<0.1%)	1 (<0.1%)
Skin injury	recovered/resolved	0	1 (<0.1%)	1 (<0.1%)
Spinal column injury	recovered/resolved	0	2 (0.1%)	2 (<0.1%)
Stress fracture	recovered/resolved	0	1 (<0.1%)	1 (<0.1%)
Subdural haematoma	recovered/resolved	1 (<0.1%)	0	1 (<0.1%)
Tendon injury	recovered/resolved	0	1 (<0.1%)	1 (<0.1%)
Tendon rupture	recovered/resolved	3 (0.2%)	1 (<0.1%)	4 (0.1%)
Thermal burn	recovered/resolved	0	3 (0.2%)	3 (0.1%)
Tibia fracture	recovered/resolved	1 (<0.1%)	1 (<0.1%)	2 (<0.1%)
Traumatic fracture	recovered/resolved	1 (<0.1%)	0	1 (<0.1%)

Table 14.3.1 / 14: Number of subjects with adverse events by outcome, primary system organ class and preferred term (FAS)

Primary system organ class<br>Preferred term<br>MedDRA version 14.0	Maximum Derived AE outcome	LCS12<br>N=1432 (100%)	LCS16<br>N=1452 (100%)	Total<br>N=2884 (100%)
Upper limb fracture	recovered/resolved	1 (<0.1%)	1 (<0.1%)	2 (<0.1%)
	not recovered/not resolved	0	1 (<0.1%)	1 (<0.1%)
Vulval laceration	recovered/resolved	0	1 (<0.1%)	1 (<0.1%)
Whiplash injury	recovered/resolved	1 (<0.1%)	5 (0.3%)	6 (0.2%)
Wound	recovered/resolved	1 (<0.1%)	0	1 (<0.1%)
Wrist fracture	recovered/resolved	0	1 (<0.1%)	1 (<0.1%)
	recovering/resolving	0	1 (<0.1%)	1 (<0.1%)
Investigations	recovered/resolved	65 (4.5%)	60 (4.1%)	125 (4.3%)
	recovering/resolving	5 (0.3%)	6 (0.4%)	11 (0.4%)
	not recovered/not resolved	68 (4.7%)	79 (5.4%)	147 (5.1%)
	recovered/resolved with resid. effects	0	1 (<0.1%)	1 (<0.1%)
Alanine aminotransferase increased	recovered/resolved	3 (0.2%)	5 (0.3%)	8 (0.3%)
	not recovered/not resolved	5 (0.3%)	5 (0.3%)	10 (0.3%)
Antibiotic resistant Staphylococcus test positive	recovered/resolved	2 (0.1%)	0	2 (<0.1%)
Aspartate aminotransferase increased	recovered/resolved	2 (0.1%)	2 (0.1%)	4 (0.1%)
	not recovered/not resolved	3 (0.2%)	2 (0.1%)	5 (0.2%)
Biopsy muscle	recovered/resolved	1 (<0.1%)	0	1 (<0.1%)
Blood alkaline phosphatase decreased	not recovered/not resolved	1 (<0.1%)	0	1 (<0.1%)
Blood amylase increased	not recovered/not resolved	0	1 (<0.1%)	1 (<0.1%)
Blood cholesterol increased	recovered/resolved	2 (0.1%)	0	2 (<0.1%)
	not recovered/not resolved	1 (<0.1%)	1 (<0.1%)	2 (<0.1%)
Blood iron decreased	not recovered/not resolved	0	1 (<0.1%)	1 (<0.1%)
Blood pressure diastolic increased	recovering/resolving	0	1 (<0.1%)	1 (<0.1%)

Table 14.3.1 / 14: Number of subjects with adverse events by outcome, primary system organ class and preferred term (FAS)

Primary system organ class<br>Preferred term<br>MedDRA version 14.0	Maximum Derived AE outcome	LCS12<br>N=1432 (100%)	LCS16<br>N=1452 (100%)	Total<br>N=2884 (100%)
Blood pressure increased	recovered/resolved	2 (0.1%)	1 (<0.1%)	3 (0.1%)
	not recovered/not resolved	1 (<0.1%)	0	1 (<0.1%)
Blood pressure systolic increased	recovered/resolved	0	1 (<0.1%)	1 (<0.1%)
Blood sodium decreased	not recovered/not resolved	1 (<0.1%)	0	1 (<0.1%)
Blood triglycerides increased	recovered/resolved	1 (<0.1%)	1 (<0.1%)	2 (<0.1%)
	recovering/resolving	0	1 (<0.1%)	1 (<0.1%)
	not recovered/not resolved	0	3 (0.2%)	3 (0.1%)
Blood urine present	recovered/resolved	1 (<0.1%)	1 (<0.1%)	2 (<0.1%)
	not recovered/not resolved	2 (0.1%)	0	2 (<0.1%)
Carbohydrate antigen 125 increased	recovered/resolved	1 (<0.1%)	0	1 (<0.1%)
Cardiac murmur	not recovered/not resolved	1 (<0.1%)	0	1 (<0.1%)
Chlamydia test positive	recovered/resolved	7 (0.5%)	1 (<0.1%)	8 (0.3%)
Escherichia test positive	recovered/resolved	1 (<0.1%)	0	1 (<0.1%)
Gamma-glutamyltransferase increased	recovered/resolved	1 (<0.1%)	1 (<0.1%)	2 (<0.1%)
	recovering/resolving	1 (<0.1%)	0	1 (<0.1%)
	not recovered/not resolved	2 (0.1%)	9 (0.6%)	11 (0.4%)
	recovered/resolved with resid. effects	0	1 (<0.1%)	1 (<0.1%)
Gastric pH decreased	recovered/resolved	0	1 (<0.1%)	1 (<0.1%)
Glucose urine present	not recovered/not resolved	0	1 (<0.1%)	1 (<0.1%)
Glycosylated haemoglobin increased	not recovered/not resolved	5 (0.3%)	0	5 (0.2%)
Haematocrit decreased	recovering/resolving	0	1 (<0.1%)	1 (<0.1%)
Haematocrit increased	not recovered/not resolved	2 (0.1%)	2 (0.1%)	4 (0.1%)

Table 14.3.1 / 14: Number of subjects with adverse events by outcome, primary system organ class and preferred term (FAS)

Primary system organ class<br>Preferred term<br>MedDRA version 14.0	Maximum Derived AE outcome	LCS12<br>N=1432 (100%)	LCS16<br>N=1452 (100%)	Total<br>N=2884 (100%)
Haemoglobin decreased	recovered/resolved	2 (0.1%)	2 (0.1%)	4 (0.1%)
	recovering/resolving	0	1 (<0.1%)	1 (<0.1%)
	not recovered/not resolved	2 (0.1%)	2 (0.1%)	4 (0.1%)
Heart rate increased	recovered/resolved	0	1 (<0.1%)	1 (<0.1%)
Hepatic enzyme increased	recovered/resolved	0	1 (<0.1%)	1 (<0.1%)
	not recovered/not resolved	1 (<0.1%)	0	1 (<0.1%)
High density lipoprotein decreased	recovered/resolved	0	1 (<0.1%)	1 (<0.1%)
	not recovered/not resolved	3 (0.2%)	5 (0.3%)	8 (0.3%)
High density lipoprotein increased	not recovered/not resolved	0	1 (<0.1%)	1 (<0.1%)
Human papilloma virus test positive	recovered/resolved	2 (0.1%)	4 (0.3%)	6 (0.2%)
	recovering/resolving	1 (<0.1%)	1 (<0.1%)	2 (<0.1%)
	not recovered/not resolved	2 (0.1%)	0	2 (<0.1%)
Liver function test abnormal	recovered/resolved	0	1 (<0.1%)	1 (<0.1%)
	not recovered/not resolved	0	1 (<0.1%)	1 (<0.1%)
Low density lipoprotein increased	not recovered/not resolved	1 (<0.1%)	1 (<0.1%)	2 (<0.1%)
Neisseria test positive	recovered/resolved	2 (0.1%)	0	2 (<0.1%)
Platelet count decreased	recovered/resolved	0	1 (<0.1%)	1 (<0.1%)
	not recovered/not resolved	2 (0.1%)	2 (0.1%)	4 (0.1%)
Protein total decreased	not recovered/not resolved	0	1 (<0.1%)	1 (<0.1%)
Protein urine present	recovered/resolved	0	1 (<0.1%)	1 (<0.1%)
	not recovered/not resolved	1 (<0.1%)	0	1 (<0.1%)
Red blood cell count decreased	recovering/resolving	0	1 (<0.1%)	1 (<0.1%)
	not recovered/not resolved	0	1 (<0.1%)	1 (<0.1%)
Red blood cell count increased	not recovered/not resolved	1 (<0.1%)	0	1 (<0.1%)

Table 14.3.1 / 14: Number of subjects with adverse events by outcome, primary system organ class and preferred term (FAS)

Primary system organ class<br>Preferred term<br>MedDRA version 14.0	Maximum Derived AE outcome	LCS12<br>N=1432 (100%)	LCS16<br>N=1452 (100%)	Total<br>N=2884 (100%)
Simplex virus test positive	recovered/resolved	1 (<0.1%)	0	1 (<0.1%)
	not recovered/not resolved	0	1 (<0.1%)	1 (<0.1%)
Smear cervix abnormal	recovered/resolved	11 (0.8%)	21 (1.4%)	32 (1.1%)
	recovering/resolving	2 (0.1%)	0	2 (<0.1%)
	not recovered/not resolved	5 (0.3%)	4 (0.3%)	9 (0.3%)
Streptococcus test positive	recovered/resolved	1 (<0.1%)	0	1 (<0.1%)
Ultrasound ovary abnormal	recovered/resolved	1 (<0.1%)	0	1 (<0.1%)
Urine leukocyte esterase positive	recovered/resolved	0	1 (<0.1%)	1 (<0.1%)
Vitamin D decreased	recovered/resolved	0	1 (<0.1%)	1 (<0.1%)
Weight decreased	recovered/resolved	7 (0.5%)	5 (0.3%)	12 (0.4%)
	recovering/resolving	1 (<0.1%)	0	1 (<0.1%)
	not recovered/not resolved	1 (<0.1%)	2 (0.1%)	3 (0.1%)
Weight increased	recovered/resolved	21 (1.5%)	17 (1.2%)	38 (1.3%)
	recovering/resolving	0	2 (0.1%)	2 (<0.1%)
	not recovered/not resolved	28 (2.0%)	43 (3.0%)	71 (2.5%)
White blood cell count decreased	recovered/resolved	1 (<0.1%)	2 (0.1%)	3 (0.1%)
	recovering/resolving	1 (<0.1%)	0	1 (<0.1%)
	not recovered/not resolved	1 (<0.1%)	0	1 (<0.1%)
White blood cell count increased	recovered/resolved	1 (<0.1%)	0	1 (<0.1%)
	not recovered/not resolved	4 (0.3%)	2 (0.1%)	6 (0.2%)
White blood cells urine positive	recovered/resolved	3 (0.2%)	1 (<0.1%)	4 (0.1%)
	not recovered/not resolved	0	1 (<0.1%)	1 (<0.1%)
Metabolism and nutrition disorders	recovered/resolved	10 (0.7%)	8 (0.6%)	18 (0.6%)
	recovering/resolving	0	3 (0.2%)	3 (0.1%)
	not recovered/not resolved	7 (0.5%)	5 (0.3%)	12 (0.4%)
Abnormal loss of weight	recovered/resolved	0	1 (<0.1%)	1 (<0.1%)

Table 14.3.1 / 14: Number of subjects with adverse events by outcome, primary system organ class and preferred term (FAS)

Primary system organ class<br>Preferred term<br>MedDRA version 14.0	Maximum Derived AE outcome	LCS12<br>N=1432 (100%)	LCS16<br>N=1452 (100%)	Total<br>N=2884 (100%)
Abnormal weight gain	recovering/resolving	0	1 (<0.1%)	1 (<0.1%)
Cell death	recovered/resolved	1 (<0.1%)	0	1 (<0.1%)
Cholesterosis	recovered/resolved	1 (<0.1%)	0	1 (<0.1%)
Decreased appetite	recovered/resolved	2 (0.1%)	1 (<0.1%)	3 (0.1%)
Dehydration	recovered/resolved	0	2 (0.1%)	2 (<0.1%)
Diabetic ketoacidosis	recovered/resolved	0	1 (<0.1%)	1 (<0.1%)
Dyslipidaemia	not recovered/not resolved	0	1 (<0.1%)	1 (<0.1%)
Fluid retention	recovered/resolved	1 (<0.1%)	1 (<0.1%)	2 (<0.1%)
	recovering/resolving	0	1 (<0.1%)	1 (<0.1%)
	not recovered/not resolved	1 (<0.1%)	0	1 (<0.1%)
Glucose tolerance impaired	not recovered/not resolved	1 (<0.1%)	0	1 (<0.1%)
Hypokalaemia	recovered/resolved	2 (0.1%)	0	2 (<0.1%)
Hyponatraemia	recovered/resolved	1 (<0.1%)	0	1 (<0.1%)
Increased appetite	recovered/resolved	1 (<0.1%)	1 (<0.1%)	2 (<0.1%)
	recovering/resolving	0	1 (<0.1%)	1 (<0.1%)
Insulin resistance	not recovered/not resolved	2 (0.1%)	1 (<0.1%)	3 (0.1%)
Lactose intolerance	recovered/resolved	1 (<0.1%)	0	1 (<0.1%)
	not recovered/not resolved	1 (<0.1%)	0	1 (<0.1%)
Obesity	recovered/resolved	0	1 (<0.1%)	1 (<0.1%)
Overweight	not recovered/not resolved	0	1 (<0.1%)	1 (<0.1%)
Type 2 diabetes mellitus	not recovered/not resolved	1 (<0.1%)	0	1 (<0.1%)

Table 14.3.1 / 14: Number of subjects with adverse events by outcome, primary system organ class and preferred term (FAS)

Primary system organ class<br>Preferred term<br>MedDRA version 14.0	Maximum Derived AE outcome	LCS12<br>N=1432 (100%)	LCS16<br>N=1452 (100%)	Total<br>N=2884 (100%)
Vitamin B12 deficiency	recovered/resolved	1 (<0.1%)	0	1 (<0.1%)
	not recovered/not resolved	0	1 (<0.1%)	1 (<0.1%)
Vitamin D deficiency	not recovered/not resolved	1 (<0.1%)	1 (<0.1%)	2 (<0.1%)
Weight fluctuation	recovered/resolved	0	1 (<0.1%)	1 (<0.1%)
Musculoskeletal and connective tissue disorders	recovered/resolved	102 (7.1%)	141 (9.7%)	243 (8.4%)
	recovering/resolving	4 (0.3%)	5 (0.3%)	9 (0.3%)
	not recovered/not resolved	33 (2.3%)	36 (2.5%)	69 (2.4%)
	recovered/resolved with resid. effects	0	1 (<0.1%)	1 (<0.1%)
Ankylosing spondylitis	not recovered/not resolved	1 (<0.1%)	0	1 (<0.1%)
Arthralgia	recovered/resolved	16 (1.1%)	25 (1.7%)	41 (1.4%)
	not recovered/not resolved	5 (0.3%)	6 (0.4%)	11 (0.4%)
Arthritis	recovered/resolved	0	3 (0.2%)	3 (0.1%)
	not recovered/not resolved	1 (<0.1%)	2 (0.1%)	3 (0.1%)
Axillary mass	recovered/resolved	0	1 (<0.1%)	1 (<0.1%)
Back pain	recovered/resolved	41 (2.9%)	51 (3.5%)	92 (3.2%)
	recovering/resolving	1 (<0.1%)	2 (0.1%)	3 (0.1%)
	not recovered/not resolved	15 (1.0%)	11 (0.8%)	26 (0.9%)
Bone pain	recovered/resolved	1 (<0.1%)	3 (0.2%)	4 (0.1%)
Bursitis	recovered/resolved	1 (<0.1%)	1 (<0.1%)	2 (<0.1%)
	not recovered/not resolved	1 (<0.1%)	0	1 (<0.1%)
Compartment syndrome	recovered/resolved	0	1 (<0.1%)	1 (<0.1%)
Costochondritis	recovered/resolved	2 (0.1%)	1 (<0.1%)	3 (0.1%)
	not recovered/not resolved	1 (<0.1%)	0	1 (<0.1%)
Exostosis	recovered/resolved	0	3 (0.2%)	3 (0.1%)
	not recovered/not resolved	1 (<0.1%)	0	1 (<0.1%)

Table 14.3.1 / 14: Number of subjects with adverse events by outcome, primary system organ class and preferred term (FAS)

Primary system organ class<br>Preferred term<br>MedDRA version 14.0	Maximum Derived AE outcome	LCS12<br>N=1432 (100%)	LCS16<br>N=1452 (100%)	Total<br>N=2884 (100%)
Fibromyalgia	not recovered/not resolved	0	2 (0.1%)	2 (<0.1%)
Flank pain	recovered/resolved	2 (0.1%)	3 (0.2%)	5 (0.2%)
Groin pain	recovered/resolved	2 (0.1%)	4 (0.3%)	6 (0.2%)
Hand deformity	recovered/resolved	0	1 (<0.1%)	1 (<0.1%)
Intervertebral disc disorder	recovered/resolved	1 (<0.1%)	1 (<0.1%)	2 (<0.1%)
Intervertebral disc protrusion	recovered/resolved	0	1 (<0.1%)	1 (<0.1%)
	not recovered/not resolved	1 (<0.1%)	2 (0.1%)	3 (0.1%)
Joint effusion	recovered/resolved	1 (<0.1%)	0	1 (<0.1%)
Joint instability	recovered/resolved	1 (<0.1%)	0	1 (<0.1%)
	recovering/resolving	0	1 (<0.1%)	1 (<0.1%)
Joint stiffness	not recovered/not resolved	0	1 (<0.1%)	1 (<0.1%)
Joint swelling	recovered/resolved	0	1 (<0.1%)	1 (<0.1%)
Loose body in joint	recovered/resolved with resid. effects	0	1 (<0.1%)	1 (<0.1%)
Medial tibial stress syndrome	recovered/resolved	1 (<0.1%)	2 (0.1%)	3 (0.1%)
	recovering/resolving	1 (<0.1%)	0	1 (<0.1%)
Muscle contracture	recovered/resolved	1 (<0.1%)	2 (0.1%)	3 (0.1%)
Muscle spasms	recovered/resolved	5 (0.3%)	7 (0.5%)	12 (0.4%)
	not recovered/not resolved	0	1 (<0.1%)	1 (<0.1%)
Muscle tightness	recovered/resolved	6 (0.4%)	6 (0.4%)	12 (0.4%)
	not recovered/not resolved	2 (0.1%)	4 (0.3%)	6 (0.2%)
Musculoskeletal chest pain	recovered/resolved	0	2 (0.1%)	2 (<0.1%)
	not recovered/not resolved	2 (0.1%)	0	2 (<0.1%)

Table 14.3.1 / 14: Number of subjects with adverse events by outcome, primary system organ class and preferred term (FAS)

Primary system organ class<br>Preferred term<br>MedDRA version 14.0	Maximum Derived AE outcome	LCS12<br>N=1432 (100%)	LCS16<br>N=1452 (100%)	Total<br>N=2884 (100%)
Musculoskeletal discomfort	recovered/resolved	0	1 (<0.1%)	1 (<0.1%)
Musculoskeletal pain	recovered/resolved	5 (0.3%)	10 (0.7%)	15 (0.5%)
	not recovered/not resolved	2 (0.1%)	0	2 (<0.1%)
Musculoskeletal stiffness	recovered/resolved	0	1 (<0.1%)	1 (<0.1%)
Myalgia	recovered/resolved	7 (0.5%)	11 (0.8%)	18 (0.6%)
	recovering/resolving	1 (<0.1%)	1 (<0.1%)	2 (<0.1%)
	not recovered/not resolved	1 (<0.1%)	0	1 (<0.1%)
Myositis	recovered/resolved	0	1 (<0.1%)	1 (<0.1%)
Neck pain	recovered/resolved	12 (0.8%)	9 (0.6%)	21 (0.7%)
Osteitis	recovered/resolved	2 (0.1%)	0	2 (<0.1%)
Osteoarthritis	not recovered/not resolved	0	2 (0.1%)	2 (<0.1%)
Osteochondrosis	recovered/resolved	0	2 (0.1%)	2 (<0.1%)
Osteopenia	not recovered/not resolved	0	1 (<0.1%)	1 (<0.1%)
Pain in extremity	recovered/resolved	6 (0.4%)	11 (0.8%)	17 (0.6%)
	recovering/resolving	0	1 (<0.1%)	1 (<0.1%)
	not recovered/not resolved	0	3 (0.2%)	3 (0.1%)
Pain in jaw	recovered/resolved	0	1 (<0.1%)	1 (<0.1%)
Patellofemoral pain syndrome	not recovered/not resolved	1 (<0.1%)	0	1 (<0.1%)
Plantar fasciitis	recovered/resolved	0	1 (<0.1%)	1 (<0.1%)
Psoriatic arthropathy	recovering/resolving	1 (<0.1%)	0	1 (<0.1%)
Rotator cuff syndrome	recovered/resolved	1 (<0.1%)	4 (0.3%)	5 (0.2%)
	not recovered/not resolved	0	2 (0.1%)	2 (<0.1%)

Table 14.3.1 / 14: Number of subjects with adverse events by outcome, primary system organ class and preferred term (FAS)

Primary system organ class<br>Preferred term<br>MedDRA version 14.0	Maximum Derived AE outcome	LCS12<br>N=1432 (100%)	LCS16<br>N=1452 (100%)	Total<br>N=2884 (100%)
Scleroderma	recovered/resolved	1 (<0.1%)	0	1 (<0.1%)
Sensation of heaviness	recovered/resolved	0	1 (<0.1%)	1 (<0.1%)
Spinal osteoarthritis	recovered/resolved	1 (<0.1%)	0	1 (<0.1%)
Synovitis	recovered/resolved	1 (<0.1%)	0	1 (<0.1%)
Temporomandibular joint syndrome	recovered/resolved	1 (<0.1%)	0	1 (<0.1%)
	not recovered/not resolved	0	1 (<0.1%)	1 (<0.1%)
Tendon pain	recovered/resolved	1 (<0.1%)	1 (<0.1%)	2 (<0.1%)
Tendonitis	recovered/resolved	4 (0.3%)	2 (0.1%)	6 (0.2%)
	not recovered/not resolved	1 (<0.1%)	2 (0.1%)	3 (0.1%)
Tenosynovitis	recovered/resolved	0	1 (<0.1%)	1 (<0.1%)
Torticollis	recovered/resolved	1 (<0.1%)	2 (0.1%)	3 (0.1%)
Neoplasms benign, malignant and unspecified (incl cysts and polyps)	Continuing with change	0	3 (0.2%)	3 (0.1%)
	recovered/resolved	24 (1.7%)	26 (1.8%)	50 (1.7%)
	recovering/resolving	0	1 (<0.1%)	1 (<0.1%)
	not recovered/not resolved	16 (1.1%)	9 (0.6%)	25 (0.9%)
	recovered/resolved with resid. effects	3 (0.2%)	1 (<0.1%)	4 (0.1%)
Acoustic neuroma	not recovered/not resolved	0	1 (<0.1%)	1 (<0.1%)
Acrochordon	recovered/resolved	1 (<0.1%)	3 (0.2%)	4 (0.1%)
Acute leukaemia	not recovered/not resolved	1 (<0.1%)	0	1 (<0.1%)
Adenoma benign	recovered/resolved	0	1 (<0.1%)	1 (<0.1%)
Astrocytoma, low grade	recovered/resolved	1 (<0.1%)	0	1 (<0.1%)

Table 14.3.1 / 14: Number of subjects with adverse events by outcome, primary system organ class and preferred term (FAS)

Primary system organ class<br>Preferred term<br>MedDRA version 14.0	Maximum Derived AE outcome	LCS12<br>N=1432 (100%)	LCS16<br>N=1452 (100%)	Total<br>N=2884 (100%)
Benign breast neoplasm	recovered/resolved	2 (0.1%)	0	2 (<0.1%)
	recovered/resolved with resid. effects	0	1 (<0.1%)	1 (<0.1%)
Cervicitis human papilloma virus	recovered/resolved	0	1 (<0.1%)	1 (<0.1%)
	not recovered/not resolved	1 (<0.1%)	1 (<0.1%)	2 (<0.1%)
Cervix carcinoma stage 0	recovering/resolving	0	1 (<0.1%)	1 (<0.1%)
Cervix neoplasm	recovered/resolved with resid. effects	1 (<0.1%)	0	1 (<0.1%)
Enchondroma	recovered/resolved	0	1 (<0.1%)	1 (<0.1%)
Fibroadenoma of breast	recovered/resolved	1 (<0.1%)	2 (0.1%)	3 (0.1%)
	not recovered/not resolved	5 (0.3%)	0	5 (0.2%)
	recovered/resolved with resid. effects	1 (<0.1%)	0	1 (<0.1%)
Glomus tumour	recovered/resolved	1 (<0.1%)	0	1 (<0.1%)
Haemangioma	recovered/resolved	1 (<0.1%)	0	1 (<0.1%)
Haemangioma of liver	not recovered/not resolved	0	1 (<0.1%)	1 (<0.1%)
Lipoma of breast	recovered/resolved	1 (<0.1%)	0	1 (<0.1%)
	not recovered/not resolved	1 (<0.1%)	0	1 (<0.1%)
Malignant melanoma	recovered/resolved	0	1 (<0.1%)	1 (<0.1%)
Melanocytic naevus	recovered/resolved	6 (0.4%)	2 (0.1%)	8 (0.3%)
	not recovered/not resolved	0	2 (0.1%)	2 (<0.1%)
Neurilemmoma	Continuing with change	0	1 (<0.1%)	1 (<0.1%)
Ovarian germ cell teratoma benign	recovered/resolved	4 (0.3%)	3 (0.2%)	7 (0.2%)
	not recovered/not resolved	1 (<0.1%)	0	1 (<0.1%)
Ovarian neoplasm	recovered/resolved	0	2 (0.1%)	2 (<0.1%)

Table 14.3.1 / 14: Number of subjects with adverse events by outcome, primary system organ class and preferred term (FAS)

Primary system organ class<br>Preferred term<br>MedDRA version 14.0	Maximum Derived AE outcome	LCS12<br>N=1432 (100%)	LCS16<br>N=1452 (100%)	Total<br>N=2884 (100%)
Pancreatic carcinoma	not recovered/not resolved	0	1 (<0.1%)	1 (<0.1%)
Skin papilloma	recovered/resolved	1 (<0.1%)	2 (0.1%)	3 (0.1%)
Teratoma benign	recovered/resolved	0	1 (<0.1%)	1 (<0.1%)
Thyroid cancer	recovered/resolved with resid. effects	1 (<0.1%)	0	1 (<0.1%)
Thyroid neoplasm	recovered/resolved	0	1 (<0.1%)	1 (<0.1%)
	not recovered/not resolved	0	1 (<0.1%)	1 (<0.1%)
Uterine leiomyoma	Continuing with change	0	2 (0.1%)	2 (<0.1%)
	recovered/resolved	2 (0.1%)	2 (0.1%)	4 (0.1%)
	not recovered/not resolved	5 (0.3%)	2 (0.1%)	7 (0.2%)
Vulvovaginal human papilloma virus infection	recovered/resolved	3 (0.2%)	6 (0.4%)	9 (0.3%)
	not recovered/not resolved	2 (0.1%)	0	2 (<0.1%)
Nervous system disorders	recovered/resolved	134 (9.4%)	158 (10.9%)	292 (10.1%)
	recovering/resolving	4 (0.3%)	4 (0.3%)	8 (0.3%)
	not recovered/not resolved	57 (4.0%)	52 (3.6%)	109 (3.8%)
	recovered/resolved with resid. effects	1 (<0.1%)	0	1 (<0.1%)
Amnesia	recovering/resolving	1 (<0.1%)	0	1 (<0.1%)
	not recovered/not resolved	0	1 (<0.1%)	1 (<0.1%)
Aphonia	recovered/resolved	0	1 (<0.1%)	1 (<0.1%)
Burning sensation	recovered/resolved	1 (<0.1%)	0	1 (<0.1%)
Carpal tunnel syndrome	recovered/resolved	0	3 (0.2%)	3 (0.1%)
	not recovered/not resolved	1 (<0.1%)	3 (0.2%)	4 (0.1%)
Cervicogenic headache	recovered/resolved	1 (<0.1%)	0	1 (<0.1%)
Cluster headache	recovered/resolved	0	1 (<0.1%)	1 (<0.1%)

Table 14.3.1 / 14: Number of subjects with adverse events by outcome, primary system organ class and preferred term (FAS)

Primary system organ class<br>Preferred term<br>MedDRA version 14.0	Maximum Derived AE outcome	LCS12<br>N=1432 (100%)	LCS16<br>N=1452 (100%)	Total<br>N=2884 (100%)
Convulsion	recovered/resolved	1 (<0.1%)	1 (<0.1%)	2 (<0.1%)
	not recovered/not resolved	0	1 (<0.1%)	1 (<0.1%)
Disturbance in attention	recovered/resolved	0	1 (<0.1%)	1 (<0.1%)
	not recovered/not resolved	0	1 (<0.1%)	1 (<0.1%)
Dizziness	recovered/resolved	7 (0.5%)	19 (1.3%)	26 (0.9%)
	recovering/resolving	0	1 (<0.1%)	1 (<0.1%)
	not recovered/not resolved	2 (0.1%)	1 (<0.1%)	3 (0.1%)
Dizziness postural	recovered/resolved	1 (<0.1%)	0	1 (<0.1%)
Facial spasm	recovered/resolved	1 (<0.1%)	0	1 (<0.1%)
Headache	Continuing with change	0	1 (<0.1%)	1 (<0.1%)
	recovered/resolved	87 (6.1%)	102 (7.0%)	189 (6.6%)
	recovering/resolving	2 (0.1%)	3 (0.2%)	5 (0.2%)
	not recovered/not resolved	39 (2.7%)	26 (1.8%)	65 (2.3%)
	recovered/resolved with resid. effects	1 (<0.1%)	0	1 (<0.1%)
Hypoaesthesia	recovered/resolved	1 (<0.1%)	3 (0.2%)	4 (0.1%)
	not recovered/not resolved	1 (<0.1%)	1 (<0.1%)	2 (<0.1%)
Loss of consciousness	recovered/resolved	0	1 (<0.1%)	1 (<0.1%)
Migraine	Continuing with change	1 (<0.1%)	0	1 (<0.1%)
	recovered/resolved	20 (1.4%)	23 (1.6%)	43 (1.5%)
	recovering/resolving	1 (<0.1%)	1 (<0.1%)	2 (<0.1%)
	not recovered/not resolved	11 (0.8%)	13 (0.9%)	24 (0.8%)
Migraine with aura	recovered/resolved	3 (0.2%)	1 (<0.1%)	4 (0.1%)
	not recovered/not resolved	1 (<0.1%)	1 (<0.1%)	2 (<0.1%)
Migraine without aura	recovered/resolved	1 (<0.1%)	0	1 (<0.1%)
Morton's neuralgia	not recovered/not resolved	1 (<0.1%)	0	1 (<0.1%)
Multiple sclerosis	not recovered/not resolved	1 (<0.1%)	0	1 (<0.1%)

Table 14.3.1 / 14: Number of subjects with adverse events by outcome, primary system organ class and preferred term (FAS)

Primary system organ class<br>Preferred term<br>MedDRA version 14.0	Maximum Derived AE outcome	LCS12<br>N=1432 (100%)	LCS16<br>N=1452 (100%)	Total<br>N=2884 (100%)
Muscle contractions involuntary	recovered/resolved	0	1 (<0.1%)	1 (<0.1%)
Nerve compression	recovered/resolved	2 (0.1%)	0	2 (<0.1%)
Nervous system disorder	not recovered/not resolved	0	1 (<0.1%)	1 (<0.1%)
Paraesthesia	recovered/resolved	0	4 (0.3%)	4 (0.1%)
	not recovered/not resolved	0	1 (<0.1%)	1 (<0.1%)
Presyncope	recovered/resolved	4 (0.3%)	9 (0.6%)	13 (0.5%)
Restless legs syndrome	not recovered/not resolved	1 (<0.1%)	0	1 (<0.1%)
Sciatica	recovered/resolved	4 (0.3%)	2 (0.1%)	6 (0.2%)
	not recovered/not resolved	0	1 (<0.1%)	1 (<0.1%)
Sinus headache	recovered/resolved	1 (<0.1%)	1 (<0.1%)	2 (<0.1%)
	not recovered/not resolved	0	1 (<0.1%)	1 (<0.1%)
Somnolence	recovered/resolved	1 (<0.1%)	1 (<0.1%)	2 (<0.1%)
	not recovered/not resolved	1 (<0.1%)	0	1 (<0.1%)
Spinal cord herniation	recovered/resolved	0	1 (<0.1%)	1 (<0.1%)
Status migrainosus	recovered/resolved	1 (<0.1%)	0	1 (<0.1%)
Syncope	recovered/resolved	6 (0.4%)	5 (0.3%)	11 (0.4%)
Tension headache	recovered/resolved	6 (0.4%)	7 (0.5%)	13 (0.5%)
	not recovered/not resolved	6 (0.4%)	2 (0.1%)	8 (0.3%)
Thoracic outlet syndrome	not recovered/not resolved	1 (<0.1%)	0	1 (<0.1%)
VIIth nerve paralysis	recovered/resolved	2 (0.1%)	0	2 (<0.1%)
Pregnancy, puerperium and perinatal conditions	recovered/resolved	6 (0.4%)	10 (0.7%)	16 (0.6%)

Table 14.3.1 / 14: Number of subjects with adverse events by outcome, primary system organ class and preferred term (FAS)

Primary system organ class / Preferred term / MedDRA version 14.0	Maximum Derived AE outcome	LCS12 N=1432 (100%)	LCS16 N=1452 (100%)	Total N=2884 (100%)
Abortion spontaneous	recovered/resolved	3 (0.2%)	0	3 (0.1%)
Abortion spontaneous incomplete	recovered/resolved	0	1 (<0.1%)	1 (<0.1%)
Blighted ovum	recovered/resolved	0	1 (<0.1%)	1 (<0.1%)
Ectopic pregnancy	recovered/resolved	3 (0.2%)	6 (0.4%)	9 (0.3%)
Premature separation of placenta	recovered/resolved	1 (<0.1%)	0	1 (<0.1%)
Ruptured ectopic pregnancy	recovered/resolved	0	1 (<0.1%)	1 (<0.1%)
Uterine contractions abnormal	recovered/resolved	0	1 (<0.1%)	1 (<0.1%)
Psychiatric disorders	recovered/resolved	71 (5.0%)	63 (4.3%)	134 (4.6%)
	recovering/resolving	10 (0.7%)	10 (0.7%)	20 (0.7%)
	not recovered/not resolved	83 (5.8%)	90 (6.2%)	173 (6.0%)
	fatal	0	1 (<0.1%)	1 (<0.1%)
Acute stress disorder	recovered/resolved	0	1 (<0.1%)	1 (<0.1%)
Adjustment disorder	recovered/resolved	1 (<0.1%)	0	1 (<0.1%)
Affect lability	recovered/resolved	3 (0.2%)	0	3 (0.1%)
	not recovered/not resolved	1 (<0.1%)	1 (<0.1%)	2 (<0.1%)
Affective disorder	recovered/resolved	0	1 (<0.1%)	1 (<0.1%)
Aggression	recovered/resolved	1 (<0.1%)	0	1 (<0.1%)
Alcohol abuse	recovered/resolved	0	1 (<0.1%)	1 (<0.1%)
Alcoholism	recovering/resolving	0	1 (<0.1%)	1 (<0.1%)
Anxiety	recovered/resolved	12 (0.8%)	14 (1.0%)	26 (0.9%)
	recovering/resolving	2 (0.1%)	5 (0.3%)	7 (0.2%)
	not recovered/not resolved	21 (1.5%)	18 (1.2%)	39 (1.4%)

Table 14.3.1 / 14: Number of subjects with adverse events by outcome, primary system organ class and preferred term (FAS)

Primary system organ class<br>Preferred term<br>MedDRA version 14.0	Maximum Derived AE outcome	LCS12<br>N=1432 (100%)	LCS16<br>N=1452 (100%)	Total<br>N=2884 (100%)
Anxiety disorder	recovered/resolved	0	1 (<0.1%)	1 (<0.1%)
	not recovered/not resolved	1 (<0.1%)	3 (0.2%)	4 (0.1%)
Attention deficit/hyperactivity disorder	not recovered/not resolved	4 (0.3%)	4 (0.3%)	8 (0.3%)
Bipolar I disorder	not recovered/not resolved	1 (<0.1%)	0	1 (<0.1%)
Bipolar disorder	not recovered/not resolved	1 (<0.1%)	5 (0.3%)	6 (0.2%)
Bulimia nervosa	recovered/resolved	0	1 (<0.1%)	1 (<0.1%)
Burnout syndrome	recovered/resolved	1 (<0.1%)	2 (0.1%)	3 (0.1%)
Completed suicide	fatal	0	1 (<0.1%)	1 (<0.1%)
Daydreaming	recovered/resolved	1 (<0.1%)	0	1 (<0.1%)
Depressed mood	recovered/resolved	2 (0.1%)	1 (<0.1%)	3 (0.1%)
	not recovered/not resolved	3 (0.2%)	1 (<0.1%)	4 (0.1%)
Depression	recovered/resolved	23 (1.6%)	20 (1.4%)	43 (1.5%)
	recovering/resolving	3 (0.2%)	2 (0.1%)	5 (0.2%)
	not recovered/not resolved	23 (1.6%)	25 (1.7%)	48 (1.7%)
Depression suicidal	Continuing with change	1 (<0.1%)	0	1 (<0.1%)
Depressive symptom	recovered/resolved	1 (<0.1%)	0	1 (<0.1%)
Disorientation	recovered/resolved	0	1 (<0.1%)	1 (<0.1%)
Drug dependence	recovering/resolving	0	1 (<0.1%)	1 (<0.1%)
	not recovered/not resolved	1 (<0.1%)	0	1 (<0.1%)
Fear	recovered/resolved	1 (<0.1%)	1 (<0.1%)	2 (<0.1%)
Generalised anxiety disorder	recovering/resolving	2 (0.1%)	0	2 (<0.1%)
	not recovered/not resolved	1 (<0.1%)	0	1 (<0.1%)

Table 14.3.1 / 14: Number of subjects with adverse events by outcome, primary system organ class and preferred term (FAS)

Primary system organ class<br>Preferred term<br>MedDRA version 14.0	Maximum Derived AE outcome	LCS12<br>N=1432 (100%)	LCS16<br>N=1452 (100%)	Total<br>N=2884 (100%)
Hallucination, auditory	recovered/resolved	1 (<0.1%)	0	1 (<0.1%)
Insomnia	recovered/resolved	13 (0.9%)	16 (1.1%)	29 (1.0%)
	recovering/resolving	1 (<0.1%)	0	1 (<0.1%)
	not recovered/not resolved	7 (0.5%)	13 (0.9%)	20 (0.7%)
Libido decreased	recovered/resolved	9 (0.6%)	6 (0.4%)	15 (0.5%)
	recovering/resolving	0	2 (0.1%)	2 (<0.1%)
	not recovered/not resolved	14 (1.0%)	14 (1.0%)	28 (1.0%)
Libido increased	recovered/resolved	1 (<0.1%)	1 (<0.1%)	2 (<0.1%)
	not recovered/not resolved	1 (<0.1%)	1 (<0.1%)	2 (<0.1%)
Loss of libido	recovered/resolved	2 (0.1%)	1 (<0.1%)	3 (0.1%)
	not recovered/not resolved	2 (0.1%)	1 (<0.1%)	3 (0.1%)
Mental status changes	not recovered/not resolved	0	1 (<0.1%)	1 (<0.1%)
Mood altered	recovered/resolved	4 (0.3%)	6 (0.4%)	10 (0.3%)
	recovering/resolving	1 (<0.1%)	0	1 (<0.1%)
	not recovered/not resolved	8 (0.6%)	2 (0.1%)	10 (0.3%)
Mood swings	recovered/resolved	3 (0.2%)	7 (0.5%)	10 (0.3%)
	recovering/resolving	2 (0.1%)	0	2 (<0.1%)
	not recovered/not resolved	7 (0.5%)	2 (0.1%)	9 (0.3%)
Nervousness	recovered/resolved	0	1 (<0.1%)	1 (<0.1%)
	not recovered/not resolved	0	1 (<0.1%)	1 (<0.1%)
Nicotine dependence	recovered/resolved	1 (<0.1%)	0	1 (<0.1%)
Obsessive-compulsive personality disorder	not recovered/not resolved	0	1 (<0.1%)	1 (<0.1%)
Panic attack	recovered/resolved	1 (<0.1%)	0	1 (<0.1%)
	not recovered/not resolved	0	1 (<0.1%)	1 (<0.1%)
Panic disorder	recovered/resolved	0	1 (<0.1%)	1 (<0.1%)
	not recovered/not resolved	1 (<0.1%)	4 (0.3%)	5 (0.2%)

Table 14.3.1 / 14: Number of subjects with adverse events by outcome, primary system organ class and preferred term (FAS)

Primary system organ class<br>Preferred term<br>MedDRA version 14.0	Maximum Derived AE outcome	LCS12<br>N=1432 (100%)	LCS16<br>N=1452 (100%)	Total<br>N=2884 (100%)
Personality disorder	recovered/resolved	1 (<0.1%)	0	1 (<0.1%)
Phobia of flying	not recovered/not resolved	0	1 (<0.1%)	1 (<0.1%)
Polysubstance dependence	recovered/resolved	0	1 (<0.1%)	1 (<0.1%)
Post-traumatic stress disorder	recovering/resolving	0	1 (<0.1%)	1 (<0.1%)
	not recovered/not resolved	1 (<0.1%)	0	1 (<0.1%)
Psychotic disorder	not recovered/not resolved	0	1 (<0.1%)	1 (<0.1%)
Sleep disorder	recovered/resolved	4 (0.3%)	1 (<0.1%)	5 (0.2%)
	not recovered/not resolved	1 (<0.1%)	1 (<0.1%)	2 (<0.1%)
Social phobia	recovered/resolved	0	1 (<0.1%)	1 (<0.1%)
Stress	recovered/resolved	2 (0.1%)	2 (0.1%)	4 (0.1%)
	not recovered/not resolved	1 (<0.1%)	2 (0.1%)	3 (0.1%)
Suicide attempt	recovered/resolved	1 (<0.1%)	0	1 (<0.1%)
Renal and urinary disorders	recovered/resolved	36 (2.5%)	24 (1.7%)	60 (2.1%)
	recovering/resolving	1 (<0.1%)	0	1 (<0.1%)
	not recovered/not resolved	3 (0.2%)	5 (0.3%)	8 (0.3%)
Calculus bladder	recovered/resolved	1 (<0.1%)	0	1 (<0.1%)
Calculus urinary	recovered/resolved	0	1 (<0.1%)	1 (<0.1%)
Chromaturia	recovered/resolved	0	1 (<0.1%)	1 (<0.1%)
Cystitis haemorrhagic	recovered/resolved	1 (<0.1%)	0	1 (<0.1%)
Dysuria	recovered/resolved	13 (0.9%)	7 (0.5%)	20 (0.7%)
	recovering/resolving	1 (<0.1%)	0	1 (<0.1%)
Haematuria	recovered/resolved	2 (0.1%)	2 (0.1%)	4 (0.1%)

Table 14.3.1 / 14: Number of subjects with adverse events by outcome, primary system organ class and preferred term (FAS)

Primary system organ class<br>Preferred term<br>MedDRA version 14.0	Maximum Derived AE outcome	LCS12<br>N=1432 (100%)	LCS16<br>N=1452 (100%)	Total<br>N=2884 (100%)
Hypertonic bladder	recovered/resolved	0	1 (<0.1%)	1 (<0.1%)
	not recovered/not resolved	0	1 (<0.1%)	1 (<0.1%)
Micturition urgency	recovered/resolved	2 (0.1%)	3 (0.2%)	5 (0.2%)
Nephrolithiasis	recovered/resolved	7 (0.5%)	2 (0.1%)	9 (0.3%)
	not recovered/not resolved	1 (<0.1%)	0	1 (<0.1%)
Pollakiuria	recovered/resolved	2 (0.1%)	7 (0.5%)	9 (0.3%)
	recovering/resolving	1 (<0.1%)	0	1 (<0.1%)
	not recovered/not resolved	1 (<0.1%)	1 (<0.1%)	2 (<0.1%)
Polyuria	recovered/resolved	1 (<0.1%)	1 (<0.1%)	2 (<0.1%)
Renal pain	recovered/resolved	1 (<0.1%)	0	1 (<0.1%)
Strangury	recovered/resolved	1 (<0.1%)	0	1 (<0.1%)
Stress urinary incontinence	recovered/resolved	1 (<0.1%)	0	1 (<0.1%)
	not recovered/not resolved	0	2 (0.1%)	2 (<0.1%)
Ureteric obstruction	recovered/resolved	0	1 (<0.1%)	1 (<0.1%)
Urethral cyst	not recovered/not resolved	0	1 (<0.1%)	1 (<0.1%)
Urinary incontinence	recovered/resolved	2 (0.1%)	1 (<0.1%)	3 (0.1%)
	not recovered/not resolved	1 (<0.1%)	0	1 (<0.1%)
Urinary retention	recovered/resolved	0	1 (<0.1%)	1 (<0.1%)
Urinary tract inflammation	recovered/resolved	1 (<0.1%)	0	1 (<0.1%)
Urinary tract pain	recovered/resolved	2 (0.1%)	1 (<0.1%)	3 (0.1%)
Urine odour abnormal	recovered/resolved	0	1 (<0.1%)	1 (<0.1%)

Table 14.3.1 / 14: Number of subjects with adverse events by outcome, primary system organ class and preferred term (FAS)

Primary system organ class<br>Preferred term<br>MedDRA version 14.0	Maximum Derived AE outcome	LCS12<br>N=1432 (100%)	LCS16<br>N=1452 (100%)	Total<br>N=2884 (100%)
Reproductive system and breast disorders	recovered/resolved	425 (29.7%)	494 (34.0%)	919 (31.9%)
	recovering/resolving	26 (1.8%)	23 (1.6%)	49 (1.7%)
	not recovered/not resolved	202 (14.1%)	223 (15.4%)	425 (14.7%)
	recovered/resolved with resid. effects	1 (<0.1%)	2 (0.1%)	3 (0.1%)
Adenomyosis	not recovered/not resolved	1 (<0.1%)	0	1 (<0.1%)
Adnexa uteri mass	recovered/resolved	1 (<0.1%)	0	1 (<0.1%)
Adnexa uteri pain	recovered/resolved	2 (0.1%)	5 (0.3%)	7 (0.2%)
Amenorrhoea	recovered/resolved	0	4 (0.3%)	4 (0.1%)
	not recovered/not resolved	0	1 (<0.1%)	1 (<0.1%)
Atrophic vulvovaginitis	not recovered/not resolved	1 (<0.1%)	0	1 (<0.1%)
Bartholin's cyst	not recovered/not resolved	0	1 (<0.1%)	1 (<0.1%)
Breast calcifications	recovered/resolved	1 (<0.1%)	0	1 (<0.1%)
Breast cyst	recovered/resolved	1 (<0.1%)	2 (0.1%)	3 (0.1%)
	not recovered/not resolved	2 (0.1%)	0	2 (<0.1%)
Breast discharge	recovered/resolved	0	9 (0.6%)	9 (0.3%)
	not recovered/not resolved	0	1 (<0.1%)	1 (<0.1%)
Breast discomfort	recovered/resolved	3 (0.2%)	5 (0.3%)	8 (0.3%)
	not recovered/not resolved	0	3 (0.2%)	3 (0.1%)
Breast disorder female	recovered/resolved	0	1 (<0.1%)	1 (<0.1%)
Breast dysplasia	recovered/resolved	1 (<0.1%)	0	1 (<0.1%)
Breast engorgement	recovered/resolved	1 (<0.1%)	0	1 (<0.1%)
Breast enlargement	recovered/resolved	0	1 (<0.1%)	1 (<0.1%)
	not recovered/not resolved	0	3 (0.2%)	3 (0.1%)

Table 14.3.1 / 14: Number of subjects with adverse events by outcome, primary system organ class and preferred term (FAS)

Primary system organ class<br>Preferred term<br>MedDRA version 14.0	Maximum Derived AE outcome	LCS12<br>N=1432 (100%)	LCS16<br>N=1452 (100%)	Total<br>N=2884 (100%)
Breast mass	recovered/resolved	4 (0.3%)	5 (0.3%)	9 (0.3%)
	not recovered/not resolved	2 (0.1%)	1 (<0.1%)	3 (0.1%)
Breast pain	recovered/resolved	28 (2.0%)	30 (2.1%)	58 (2.0%)
	recovering/resolving	2 (0.1%)	0	2 (<0.1%)
	not recovered/not resolved	6 (0.4%)	12 (0.8%)	18 (0.6%)
	recovered/resolved with resid. effects	0	1 (<0.1%)	1 (<0.1%)
Breast swelling	recovered/resolved	1 (<0.1%)	2 (0.1%)	3 (0.1%)
Breast tenderness	recovered/resolved	23 (1.6%)	25 (1.7%)	48 (1.7%)
	recovering/resolving	1 (<0.1%)	1 (<0.1%)	2 (<0.1%)
	not recovered/not resolved	8 (0.6%)	7 (0.5%)	15 (0.5%)
Cervical cyst	recovered/resolved	0	1 (<0.1%)	1 (<0.1%)
Cervical discharge	recovering/resolving	1 (<0.1%)	0	1 (<0.1%)
Cervical dysplasia	recovered/resolved	68 (4.7%)	77 (5.3%)	145 (5.0%)
	recovering/resolving	1 (<0.1%)	4 (0.3%)	5 (0.2%)
	not recovered/not resolved	32 (2.2%)	29 (2.0%)	61 (2.1%)
	recovered/resolved with resid. effects	1 (<0.1%)	1 (<0.1%)	2 (<0.1%)
Cervical friability	recovered/resolved	0	1 (<0.1%)	1 (<0.1%)
Cervical polyp	recovered/resolved	1 (<0.1%)	2 (0.1%)	3 (0.1%)
Cervix disorder	recovered/resolved	0	1 (<0.1%)	1 (<0.1%)
Cervix erythema	recovered/resolved	0	1 (<0.1%)	1 (<0.1%)
	not recovered/not resolved	0	1 (<0.1%)	1 (<0.1%)
Cervix haemorrhage uterine	recovered/resolved	0	1 (<0.1%)	1 (<0.1%)
Cervix inflammation	recovered/resolved	1 (<0.1%)	0	1 (<0.1%)
Cervix oedema	recovered/resolved	0	1 (<0.1%)	1 (<0.1%)

Table 14.3.1 / 14: Number of subjects with adverse events by outcome, primary system organ class and preferred term (FAS)

Primary system organ class<br>Preferred term<br>MedDRA version 14.0	Maximum Derived AE outcome	LCS12<br>N=1432 (100%)	LCS16<br>N=1452 (100%)	Total<br>N=2884 (100%)
Coital bleeding	recovered/resolved	7 (0.5%)	10 (0.7%)	17 (0.6%)
	recovering/resolving	1 (<0.1%)	0	1 (<0.1%)
	not recovered/not resolved	6 (0.4%)	4 (0.3%)	10 (0.3%)
Dysfunctional uterine bleeding	recovered/resolved	2 (0.1%)	2 (0.1%)	4 (0.1%)
	not recovered/not resolved	1 (<0.1%)	2 (0.1%)	3 (0.1%)
Dysmenorrhoea	recovered/resolved	77 (5.4%)	74 (5.1%)	151 (5.2%)
	recovering/resolving	4 (0.3%)	2 (0.1%)	6 (0.2%)
	not recovered/not resolved	45 (3.1%)	30 (2.1%)	75 (2.6%)
Dyspareunia	recovered/resolved	19 (1.3%)	19 (1.3%)	38 (1.3%)
	recovering/resolving	2 (0.1%)	2 (0.1%)	4 (0.1%)
	not recovered/not resolved	9 (0.6%)	6 (0.4%)	15 (0.5%)
Ectropion of cervix	recovered/resolved	1 (<0.1%)	1 (<0.1%)	2 (<0.1%)
	not recovered/not resolved	1 (<0.1%)	0	1 (<0.1%)
Endometriosis	Continuing with change	0	1 (<0.1%)	1 (<0.1%)
	recovered/resolved	2 (0.1%)	0	2 (<0.1%)
	not recovered/not resolved	4 (0.3%)	2 (0.1%)	6 (0.2%)
Fibrocystic breast disease	recovered/resolved	2 (0.1%)	2 (0.1%)	4 (0.1%)
	not recovered/not resolved	1 (<0.1%)	1 (<0.1%)	2 (<0.1%)
Galactorrhoea	recovered/resolved	1 (<0.1%)	7 (0.5%)	8 (0.3%)
	not recovered/not resolved	3 (0.2%)	1 (<0.1%)	4 (0.1%)
Genital cyst	not recovered/not resolved	0	1 (<0.1%)	1 (<0.1%)
Genital discharge	recovered/resolved	7 (0.5%)	7 (0.5%)	14 (0.5%)
	not recovered/not resolved	1 (<0.1%)	2 (0.1%)	3 (0.1%)
Genital haemorrhage	recovered/resolved	1 (<0.1%)	0	1 (<0.1%)
Genital lesion	recovered/resolved	2 (0.1%)	0	2 (<0.1%)
Genital rash	recovered/resolved	0	1 (<0.1%)	1 (<0.1%)

Table 14.3.1 / 14: Number of subjects with adverse events by outcome, primary system organ class and preferred term (FAS)

Primary system organ class<br>Preferred term<br>MedDRA version 14.0	Maximum Derived AE outcome	LCS12<br>N=1432 (100%)	LCS16<br>N=1452 (100%)	Total<br>N=2884 (100%)
Haemorrhagic ovarian cyst	recovered/resolved	10 (0.7%)	17 (1.2%)	27 (0.9%)
	recovering/resolving	2 (0.1%)	0	2 (<0.1%)
	not recovered/not resolved	1 (<0.1%)	2 (0.1%)	3 (0.1%)
Hydrometra	recovered/resolved	2 (0.1%)	0	2 (<0.1%)
Hypomenorrhoea	recovered/resolved	1 (<0.1%)	0	1 (<0.1%)
	not recovered/not resolved	1 (<0.1%)	1 (<0.1%)	2 (<0.1%)
Menometrorrhagia	not recovered/not resolved	2 (0.1%)	0	2 (<0.1%)
Menorrhagia	recovered/resolved	3 (0.2%)	3 (0.2%)	6 (0.2%)
	recovering/resolving	0	1 (<0.1%)	1 (<0.1%)
	not recovered/not resolved	3 (0.2%)	4 (0.3%)	7 (0.2%)
Menstrual disorder	recovered/resolved	1 (<0.1%)	1 (<0.1%)	2 (<0.1%)
	not recovered/not resolved	2 (0.1%)	0	2 (<0.1%)
Menstruation irregular	recovered/resolved	4 (0.3%)	0	4 (0.1%)
	recovering/resolving	0	1 (<0.1%)	1 (<0.1%)
	not recovered/not resolved	0	2 (0.1%)	2 (<0.1%)
Metrorrhagia	recovered/resolved	12 (0.8%)	7 (0.5%)	19 (0.7%)
	recovering/resolving	1 (<0.1%)	0	1 (<0.1%)
	not recovered/not resolved	2 (0.1%)	3 (0.2%)	5 (0.2%)
Nipple exudate bloody	recovered/resolved	0	1 (<0.1%)	1 (<0.1%)
Nipple pain	recovered/resolved	3 (0.2%)	2 (0.1%)	5 (0.2%)
Oligomenorrhoea	recovered/resolved	0	2 (0.1%)	2 (<0.1%)
Ovarian cyst	recovered/resolved	147 (10.3%)	251 (17.3%)	398 (13.8%)
	recovering/resolving	3 (0.2%)	4 (0.3%)	7 (0.2%)
	not recovered/not resolved	26 (1.8%)	45 (3.1%)	71 (2.5%)
Ovarian cyst ruptured	recovered/resolved	2 (0.1%)	6 (0.4%)	8 (0.3%)
	recovering/resolving	0	1 (<0.1%)	1 (<0.1%)

Table 14.3.1 / 14: Number of subjects with adverse events by outcome, primary system organ class and preferred term (FAS)

Primary system organ class<br>Preferred term<br>MedDRA version 14.0	Maximum Derived AE outcome	LCS12<br>N=1432 (100%)	LCS16<br>N=1452 (100%)	Total<br>N=2884 (100%)
Ovarian cyst torsion	recovered/resolved	0	1 (<0.1%)	1 (<0.1%)
Ovarian disorder	not recovered/not resolved	0	1 (<0.1%)	1 (<0.1%)
Ovarian enlargement	recovered/resolved	1 (<0.1%)	1 (<0.1%)	2 (<0.1%)
Ovarian mass	recovered/resolved	1 (<0.1%)	2 (0.1%)	3 (0.1%)
Ovulation pain	recovered/resolved	4 (0.3%)	1 (<0.1%)	5 (0.2%)
	recovering/resolving	2 (0.1%)	0	2 (<0.1%)
	not recovered/not resolved	0	1 (<0.1%)	1 (<0.1%)
Parovarian cyst	recovered/resolved	2 (0.1%)	0	2 (<0.1%)
Pelvic adhesions	recovered/resolved	0	1 (<0.1%)	1 (<0.1%)
Pelvic congestion	recovered/resolved	1 (<0.1%)	0	1 (<0.1%)
Pelvic discomfort	recovered/resolved	4 (0.3%)	1 (<0.1%)	5 (0.2%)
	not recovered/not resolved	1 (<0.1%)	1 (<0.1%)	2 (<0.1%)
Pelvic fluid collection	recovered/resolved	1 (<0.1%)	0	1 (<0.1%)
Pelvic pain	Continuing with change	1 (<0.1%)	1 (<0.1%)	2 (<0.1%)
	recovered/resolved	73 (5.1%)	80 (5.5%)	153 (5.3%)
	recovering/resolving	2 (0.1%)	6 (0.4%)	8 (0.3%)
	not recovered/not resolved	14 (1.0%)	31 (2.1%)	45 (1.6%)
Pelvic prolapse	recovered/resolved	1 (<0.1%)	0	1 (<0.1%)
Perineal pain	not recovered/not resolved	1 (<0.1%)	0	1 (<0.1%)
Polycystic ovaries	recovered/resolved	2 (0.1%)	1 (<0.1%)	3 (0.1%)
	not recovered/not resolved	2 (0.1%)	1 (<0.1%)	3 (0.1%)
Polymenorrhoea	recovered/resolved	1 (<0.1%)	0	1 (<0.1%)
	not recovered/not resolved	0	1 (<0.1%)	1 (<0.1%)

Table 14.3.1 / 14: Number of subjects with adverse events by outcome, primary system organ class and preferred term (FAS)

Primary system organ class<br>Preferred term<br>MedDRA version 14.0	Maximum Derived AE outcome	LCS12<br>N=1432 (100%)	LCS16<br>N=1452 (100%)	Total<br>N=2884 (100%)
Premenstrual syndrome	recovered/resolved	9 (0.6%)	3 (0.2%)	12 (0.4%)
	not recovered/not resolved	8 (0.6%)	9 (0.6%)	17 (0.6%)
Pruritus genital	recovered/resolved	1 (<0.1%)	0	1 (<0.1%)
Uterine cervical erosion	recovered/resolved	1 (<0.1%)	0	1 (<0.1%)
Uterine cervical pain	recovered/resolved	2 (0.1%)	0	2 (<0.1%)
Uterine haemorrhage	recovered/resolved	4 (0.3%)	5 (0.3%)	9 (0.3%)
	not recovered/not resolved	3 (0.2%)	1 (<0.1%)	4 (0.1%)
Uterine inflammation	recovered/resolved	0	1 (<0.1%)	1 (<0.1%)
Uterine pain	recovered/resolved	2 (0.1%)	0	2 (<0.1%)
	recovering/resolving	0	1 (<0.1%)	1 (<0.1%)
	not recovered/not resolved	0	1 (<0.1%)	1 (<0.1%)
Uterine polyp	recovered/resolved	2 (0.1%)	1 (<0.1%)	3 (0.1%)
Uterine prolapse	recovered/resolved	1 (<0.1%)	0	1 (<0.1%)
Uterine spasm	recovered/resolved	23 (1.6%)	31 (2.1%)	54 (1.9%)
	recovering/resolving	2 (0.1%)	2 (0.1%)	4 (0.1%)
	not recovered/not resolved	5 (0.3%)	5 (0.3%)	10 (0.3%)
Uterine tenderness	recovered/resolved	0	1 (<0.1%)	1 (<0.1%)
	not recovered/not resolved	0	1 (<0.1%)	1 (<0.1%)
Vaginal cyst	not recovered/not resolved	1 (<0.1%)	0	1 (<0.1%)
Vaginal discharge	recovered/resolved	40 (2.8%)	52 (3.6%)	92 (3.2%)
	recovering/resolving	3 (0.2%)	2 (0.1%)	5 (0.2%)
	not recovered/not resolved	10 (0.7%)	4 (0.3%)	14 (0.5%)
Vaginal disorder	recovered/resolved	4 (0.3%)	4 (0.3%)	8 (0.3%)

Table 14.3.1 / 14: Number of subjects with adverse events by outcome, primary system organ class and preferred term (FAS)

Primary system organ class<br>Preferred term<br>MedDRA version 14.0	Maximum Derived AE outcome	LCS12<br>N=1432 (100%)	LCS16<br>N=1452 (100%)	Total<br>N=2884 (100%)
Vaginal haemorrhage	recovered/resolved	30 (2.1%)	37 (2.5%)	67 (2.3%)
	recovering/resolving	3 (0.2%)	4 (0.3%)	7 (0.2%)
	not recovered/not resolved	27 (1.9%)	28 (1.9%)	55 (1.9%)
Vaginal lesion	recovered/resolved	1 (<0.1%)	0	1 (<0.1%)
Vaginal odour	recovered/resolved	5 (0.3%)	7 (0.5%)	12 (0.4%)
	not recovered/not resolved	1 (<0.1%)	0	1 (<0.1%)
Vaginal perforation	recovered/resolved	0	1 (<0.1%)	1 (<0.1%)
Vaginal ulceration	recovered/resolved	0	1 (<0.1%)	1 (<0.1%)
Vaginal wall congestion	recovered/resolved	1 (<0.1%)	0	1 (<0.1%)
Vulval disorder	recovered/resolved	1 (<0.1%)	0	1 (<0.1%)
Vulvovaginal burning sensation	recovered/resolved	1 (<0.1%)	4 (0.3%)	5 (0.2%)
Vulvovaginal discomfort	recovered/resolved	5 (0.3%)	2 (0.1%)	7 (0.2%)
Vulvovaginal dryness	recovered/resolved	2 (0.1%)	2 (0.1%)	4 (0.1%)
	not recovered/not resolved	1 (<0.1%)	3 (0.2%)	4 (0.1%)
Vulvovaginal erythema	recovered/resolved	0	2 (0.1%)	2 (<0.1%)
Vulvovaginal pain	recovered/resolved	1 (<0.1%)	2 (0.1%)	3 (0.1%)
	not recovered/not resolved	2 (0.1%)	0	2 (<0.1%)
Vulvovaginal pruritus	recovered/resolved	14 (1.0%)	16 (1.1%)	30 (1.0%)
	recovering/resolving	2 (0.1%)	0	2 (<0.1%)
	not recovered/not resolved	0	1 (<0.1%)	1 (<0.1%)
Vulvovaginal swelling	recovered/resolved	1 (<0.1%)	1 (<0.1%)	2 (<0.1%)
Respiratory, thoracic and mediastinal disorders	recovered/resolved	55 (3.8%)	50 (3.4%)	105 (3.6%)
	recovering/resolving	0	1 (<0.1%)	1 (<0.1%)
	not recovered/not resolved	15 (1.0%)	18 (1.2%)	33 (1.1%)

Table 14.3.1 / 14: Number of subjects with adverse events by outcome, primary system organ class and preferred term (FAS)

Primary system organ class<br>Preferred term<br>MedDRA version 14.0	Maximum Derived AE outcome	LCS12<br>N=1432 (100%)	LCS16<br>N=1452 (100%)	Total<br>N=2884 (100%)
Allergic sinusitis	recovered/resolved	1 (<0.1%)	1 (<0.1%)	2 (<0.1%)
Asthma	recovered/resolved	3 (0.2%)	3 (0.2%)	6 (0.2%)
	not recovered/not resolved	8 (0.6%)	8 (0.6%)	16 (0.6%)
Atelectasis	recovered/resolved	0	1 (<0.1%)	1 (<0.1%)
Bronchospasm	recovered/resolved	2 (0.1%)	2 (0.1%)	4 (0.1%)
Cough	recovered/resolved	11 (0.8%)	12 (0.8%)	23 (0.8%)
	recovering/resolving	0	1 (<0.1%)	1 (<0.1%)
	not recovered/not resolved	2 (0.1%)	0	2 (<0.1%)
Diaphragmatic hernia	not recovered/not resolved	0	1 (<0.1%)	1 (<0.1%)
Dyspnoea	recovered/resolved	1 (<0.1%)	1 (<0.1%)	2 (<0.1%)
	not recovered/not resolved	0	1 (<0.1%)	1 (<0.1%)
Epiglottic cyst	recovered/resolved	1 (<0.1%)	0	1 (<0.1%)
Epistaxis	recovered/resolved	3 (0.2%)	1 (<0.1%)	4 (0.1%)
Hyperventilation	recovered/resolved	1 (<0.1%)	1 (<0.1%)	2 (<0.1%)
Hypoxia	recovered/resolved	0	1 (<0.1%)	1 (<0.1%)
Nasal congestion	recovered/resolved	1 (<0.1%)	1 (<0.1%)	2 (<0.1%)
	not recovered/not resolved	1 (<0.1%)	0	1 (<0.1%)
Nasal disorder	recovered/resolved	1 (<0.1%)	0	1 (<0.1%)
Nasal oedema	recovered/resolved	1 (<0.1%)	0	1 (<0.1%)
Nasal polyps	not recovered/not resolved	1 (<0.1%)	1 (<0.1%)	2 (<0.1%)
Oropharyngeal pain	recovered/resolved	16 (1.1%)	17 (1.2%)	33 (1.1%)

Table 14.3.1 / 14: Number of subjects with adverse events by outcome, primary system organ class and preferred term (FAS)

Primary system organ class<br>Preferred term<br>MedDRA version 14.0	Maximum Derived AE outcome	LCS12<br>N=1432 (100%)	LCS16<br>N=1452 (100%)	Total<br>N=2884 (100%)
Pleural effusion	recovered/resolved	0	1 (<0.1%)	1 (<0.1%)
Pleurisy	recovered/resolved	1 (<0.1%)	1 (<0.1%)	2 (<0.1%)
Pneumonitis	recovered/resolved	0	1 (<0.1%)	1 (<0.1%)
Pneumothorax	recovered/resolved	1 (<0.1%)	0	1 (<0.1%)
Pulmonary congestion	recovered/resolved	1 (<0.1%)	0	1 (<0.1%)
Respiratory tract congestion	recovered/resolved	1 (<0.1%)	0	1 (<0.1%)
Respiratory tract inflammation	recovered/resolved	1 (<0.1%)	0	1 (<0.1%)
Rhinitis allergic	recovered/resolved	7 (0.5%)	7 (0.5%)	14 (0.5%)
	not recovered/not resolved	3 (0.2%)	4 (0.3%)	7 (0.2%)
Rhinitis seasonal	not recovered/not resolved	1 (<0.1%)	1 (<0.1%)	2 (<0.1%)
Rhinorrhoea	recovered/resolved	1 (<0.1%)	0	1 (<0.1%)
Sinus congestion	recovered/resolved	5 (0.3%)	2 (0.1%)	7 (0.2%)
Sinus polyp	recovered/resolved	0	1 (<0.1%)	1 (<0.1%)
Sleep apnoea syndrome	not recovered/not resolved	0	1 (<0.1%)	1 (<0.1%)
Tonsillar disorder	not recovered/not resolved	0	1 (<0.1%)	1 (<0.1%)
Upper respiratory tract inflammation	recovered/resolved	0	2 (0.1%)	2 (<0.1%)
Wheezing	recovered/resolved	1 (<0.1%)	0	1 (<0.1%)
Skin and subcutaneous tissue disorders	Continuing with change	0	1 (<0.1%)	1 (<0.1%)
	recovered/resolved	115 (8.0%)	124 (8.5%)	239 (8.3%)
	recovering/resolving	9 (0.6%)	8 (0.6%)	17 (0.6%)
	not recovered/not resolved	113 (7.9%)	114 (7.9%)	227 (7.9%)

Table 14.3.1 / 14: Number of subjects with adverse events by outcome, primary system organ class and preferred term (FAS)

Primary system organ class<br>Preferred term<br>MedDRA version 14.0	Maximum Derived AE outcome	LCS12<br>N=1432 (100%)	LCS16<br>N=1452 (100%)	Total<br>N=2884 (100%)
Acne	Continuing with change	0	1 (<0.1%)	1 (<0.1%)
	recovered/resolved	67 (4.7%)	67 (4.6%)	134 (4.6%)
	recovering/resolving	7 (0.5%)	7 (0.5%)	14 (0.5%)
	not recovered/not resolved	85 (5.9%)	87 (6.0%)	172 (6.0%)
Acne cystic	not recovered/not resolved	2 (0.1%)	0	2 (<0.1%)
Alopecia	recovered/resolved	5 (0.3%)	7 (0.5%)	12 (0.4%)
	recovering/resolving	2 (0.1%)	0	2 (<0.1%)
	not recovered/not resolved	8 (0.6%)	6 (0.4%)	14 (0.5%)
Alopecia areata	recovered/resolved	1 (<0.1%)	0	1 (<0.1%)
	recovering/resolving	1 (<0.1%)	0	1 (<0.1%)
Chloasma	recovered/resolved	2 (0.1%)	0	2 (<0.1%)
	recovering/resolving	1 (<0.1%)	0	1 (<0.1%)
Cold sweat	recovered/resolved	1 (<0.1%)	0	1 (<0.1%)
Dandruff	not recovered/not resolved	1 (<0.1%)	1 (<0.1%)	2 (<0.1%)
Dermal cyst	recovered/resolved	2 (0.1%)	0	2 (<0.1%)
Dermatitis	recovered/resolved	7 (0.5%)	7 (0.5%)	14 (0.5%)
	not recovered/not resolved	1 (<0.1%)	0	1 (<0.1%)
Dermatitis allergic	recovered/resolved	5 (0.3%)	5 (0.3%)	10 (0.3%)
	not recovered/not resolved	1 (<0.1%)	0	1 (<0.1%)
Dermatitis atopic	recovered/resolved	1 (<0.1%)	3 (0.2%)	4 (0.1%)
	not recovered/not resolved	1 (<0.1%)	2 (0.1%)	3 (0.1%)
Dermatitis contact	recovered/resolved	2 (0.1%)	4 (0.3%)	6 (0.2%)
	not recovered/not resolved	0	1 (<0.1%)	1 (<0.1%)
Drug eruption	recovered/resolved	1 (<0.1%)	0	1 (<0.1%)
Dry skin	not recovered/not resolved	0	1 (<0.1%)	1 (<0.1%)

Table 14.3.1 / 14: Number of subjects with adverse events by outcome, primary system organ class and preferred term (FAS)

Primary system organ class<br>Preferred term<br>MedDRA version 14.0	Maximum Derived AE outcome	LCS12<br>N=1432 (100%)	LCS16<br>N=1452 (100%)	Total<br>N=2884 (100%)
Dyshidrosis	recovered/resolved	1 (<0.1%)	0	1 (<0.1%)
Eczema	recovered/resolved	3 (0.2%)	4 (0.3%)	7 (0.2%)
	not recovered/not resolved	3 (0.2%)	4 (0.3%)	7 (0.2%)
Erythema	recovered/resolved	2 (0.1%)	0	2 (<0.1%)
Erythema multiforme	recovered/resolved	1 (<0.1%)	0	1 (<0.1%)
Hidradenitis	recovered/resolved	1 (<0.1%)	0	1 (<0.1%)
Hirsutism	recovered/resolved	1 (<0.1%)	2 (0.1%)	3 (0.1%)
	recovering/resolving	1 (<0.1%)	0	1 (<0.1%)
	not recovered/not resolved	5 (0.3%)	8 (0.6%)	13 (0.5%)
Hyperhidrosis	recovered/resolved	2 (0.1%)	2 (0.1%)	4 (0.1%)
	not recovered/not resolved	1 (<0.1%)	0	1 (<0.1%)
Hyperkeratosis	not recovered/not resolved	1 (<0.1%)	0	1 (<0.1%)
Hypertrichosis	not recovered/not resolved	2 (0.1%)	1 (<0.1%)	3 (0.1%)
Idiopathic urticaria	recovered/resolved	1 (<0.1%)	0	1 (<0.1%)
Increased tendency to bruise	recovered/resolved	1 (<0.1%)	0	1 (<0.1%)
Ingrown hair	recovered/resolved	1 (<0.1%)	0	1 (<0.1%)
Intertrigo	recovered/resolved	1 (<0.1%)	0	1 (<0.1%)
Lentigo	recovered/resolved	1 (<0.1%)	0	1 (<0.1%)
Lichen sclerosus	recovered/resolved	0	2 (0.1%)	2 (<0.1%)
	not recovered/not resolved	1 (<0.1%)	0	1 (<0.1%)
Neurodermatitis	recovered/resolved	0	1 (<0.1%)	1 (<0.1%)

Table 14.3.1 / 14: Number of subjects with adverse events by outcome, primary system organ class and preferred term (FAS)

Primary system organ class<br>Preferred term<br>MedDRA version 14.0	Maximum Derived AE outcome	LCS12<br>N=1432 (100%)	LCS16<br>N=1452 (100%)	Total<br>N=2884 (100%)
Night sweats	not recovered/not resolved	0	1 (<0.1%)	1 (<0.1%)
Photosensitivity reaction	recovered/resolved	0	1 (<0.1%)	1 (<0.1%)
Pityriasis rosea	recovered/resolved	1 (<0.1%)	2 (0.1%)	3 (0.1%)
Precancerous skin lesion	recovered/resolved	0	1 (<0.1%)	1 (<0.1%)
Pruritus	recovered/resolved	4 (0.3%)	3 (0.2%)	7 (0.2%)
Pruritus allergic	recovered/resolved	0	2 (0.1%)	2 (<0.1%)
Psoriasis	not recovered/not resolved	0	2 (0.1%)	2 (<0.1%)
Rash	Continuing with change	1 (<0.1%)	0	1 (<0.1%)
	recovered/resolved	8 (0.6%)	6 (0.4%)	14 (0.5%)
	not recovered/not resolved	1 (<0.1%)	2 (0.1%)	3 (0.1%)
Rash macular	recovered/resolved	0	1 (<0.1%)	1 (<0.1%)
Rash papular	recovered/resolved	2 (0.1%)	1 (<0.1%)	3 (0.1%)
Rosacea	recovered/resolved	1 (<0.1%)	1 (<0.1%)	2 (<0.1%)
	not recovered/not resolved	0	1 (<0.1%)	1 (<0.1%)
Scar	recovered/resolved	0	1 (<0.1%)	1 (<0.1%)
Seborrhoea	recovered/resolved	3 (0.2%)	5 (0.3%)	8 (0.3%)
	not recovered/not resolved	3 (0.2%)	5 (0.3%)	8 (0.3%)
Skin dystrophy	not recovered/not resolved	1 (<0.1%)	0	1 (<0.1%)
Skin fissures	recovered/resolved	0	2 (0.1%)	2 (<0.1%)
Skin hypopigmentation	recovered/resolved	0	1 (<0.1%)	1 (<0.1%)
Skin irritation	recovered/resolved	1 (<0.1%)	2 (0.1%)	3 (0.1%)

Table 14.3.1 / 14: Number of subjects with adverse events by outcome, primary system organ class and preferred term (FAS)

Primary system organ class<br>Preferred term<br>MedDRA version 14.0	Maximum Derived AE outcome	LCS12<br>N=1432 (100%)	LCS16<br>N=1452 (100%)	Total<br>N=2884 (100%)
Skin lesion	recovered/resolved	0	1 (<0.1%)	1 (<0.1%)
Skin odour abnormal	not recovered/not resolved	0	1 (<0.1%)	1 (<0.1%)
Skin reaction	recovered/resolved	2 (0.1%)	0	2 (<0.1%)
Urticaria	recovered/resolved	9 (0.6%)	8 (0.6%)	17 (0.6%)
	recovering/resolving	0	2 (0.1%)	2 (<0.1%)
	not recovered/not resolved	2 (0.1%)	0	2 (<0.1%)
Vitiligo	recovered/resolved	0	1 (<0.1%)	1 (<0.1%)
Social circumstances	recovered/resolved	1 (<0.1%)	0	1 (<0.1%)
	not recovered/not resolved	0	1 (<0.1%)	1 (<0.1%)
Exposure to communicable disease	recovered/resolved	1 (<0.1%)	0	1 (<0.1%)
Hearing disability	not recovered/not resolved	0	1 (<0.1%)	1 (<0.1%)
Surgical and medical procedures	recovered/resolved	39 (2.7%)	43 (3.0%)	82 (2.8%)
	recovering/resolving	0	2 (0.1%)	2 (<0.1%)
	not recovered/not resolved	1 (<0.1%)	0	1 (<0.1%)
Abdominoplasty	recovered/resolved	4 (0.3%)	1 (<0.1%)	5 (0.2%)
Anal fissure excision	recovered/resolved	1 (<0.1%)	0	1 (<0.1%)
Anal sphincterotomy	recovered/resolved	0	1 (<0.1%)	1 (<0.1%)
Apicectomy	recovered/resolved	0	1 (<0.1%)	1 (<0.1%)
Breast prosthesis implantation	recovered/resolved	3 (0.2%)	0	3 (0.1%)
	recovering/resolving	0	1 (<0.1%)	1 (<0.1%)
Bunion operation	recovered/resolved	2 (0.1%)	1 (<0.1%)	3 (0.1%)
Cervix cautery	recovered/resolved	0	1 (<0.1%)	1 (<0.1%)

Table 14.3.1 / 14: Number of subjects with adverse events by outcome, primary system organ class and preferred term (FAS)

Primary system organ class<br>Preferred term<br>MedDRA version 14.0	Maximum Derived AE outcome	LCS12<br>N=1432 (100%)	LCS16<br>N=1452 (100%)	Total<br>N=2884 (100%)
Cholecystectomy	recovered/resolved	1 (<0.1%)	0	1 (<0.1%)
Dental cosmetic procedure	recovered/resolved	1 (<0.1%)	0	1 (<0.1%)
Dental operation	recovered/resolved	1 (<0.1%)	3 (0.2%)	4 (0.1%)
Detoxification	recovered/resolved	0	1 (<0.1%)	1 (<0.1%)
	not recovered/not resolved	1 (<0.1%)	0	1 (<0.1%)
Endodontic procedure	recovered/resolved	0	2 (0.1%)	2 (<0.1%)
Eye laser surgery	recovered/resolved	0	1 (<0.1%)	1 (<0.1%)
Gallbladder operation	recovered/resolved	0	1 (<0.1%)	1 (<0.1%)
Gastric banding	recovered/resolved	1 (<0.1%)	0	1 (<0.1%)
Gastric bypass	recovered/resolved	0	1 (<0.1%)	1 (<0.1%)
Haemorrhoid operation	recovered/resolved	0	1 (<0.1%)	1 (<0.1%)
Keratomileusis	recovered/resolved	0	1 (<0.1%)	1 (<0.1%)
Ligament operation	recovered/resolved	1 (<0.1%)	0	1 (<0.1%)
Lipoma excision	recovered/resolved	1 (<0.1%)	0	1 (<0.1%)
Mammoplasty	recovered/resolved	6 (0.4%)	3 (0.2%)	9 (0.3%)
Maxillary antrum operation	recovered/resolved	1 (<0.1%)	0	1 (<0.1%)
Meniscus operation	recovered/resolved	0	1 (<0.1%)	1 (<0.1%)
Mole excision	recovered/resolved	2 (0.1%)	2 (0.1%)	4 (0.1%)
Nasal polypectomy	recovered/resolved	0	1 (<0.1%)	1 (<0.1%)

Table 14.3.1 / 14: Number of subjects with adverse events by outcome, primary system organ class and preferred term (FAS)

Primary system organ class<br>Preferred term<br>MedDRA version 14.0	Maximum Derived AE outcome	LCS12<br>N=1432 (100%)	LCS16<br>N=1452 (100%)	Total<br>N=2884 (100%)
Parasitic infection prophylaxis	recovered/resolved	0	1 (<0.1%)	1 (<0.1%)
Plastic surgery	recovered/resolved	0	1 (<0.1%)	1 (<0.1%)
Postoperative analgesia	recovered/resolved	1 (<0.1%)	0	1 (<0.1%)
Rotator cuff repair	recovered/resolved	0	1 (<0.1%)	1 (<0.1%)
	recovering/resolving	0	1 (<0.1%)	1 (<0.1%)
Scar excision	recovered/resolved	0	1 (<0.1%)	1 (<0.1%)
Skin neoplasm excision	recovered/resolved	0	1 (<0.1%)	1 (<0.1%)
Tenolysis	recovered/resolved	0	1 (<0.1%)	1 (<0.1%)
Tonsillectomy	recovered/resolved	1 (<0.1%)	2 (0.1%)	3 (0.1%)
Tooth extraction	recovered/resolved	6 (0.4%)	9 (0.6%)	15 (0.5%)
Umbilical hernia repair	recovered/resolved	0	1 (<0.1%)	1 (<0.1%)
Varicose vein operation	recovered/resolved	1 (<0.1%)	0	1 (<0.1%)
Wisdom teeth removal	recovered/resolved	7 (0.5%)	5 (0.3%)	12 (0.4%)
Wrist surgery	recovered/resolved	0	1 (<0.1%)	1 (<0.1%)
Vascular disorders	recovered/resolved	10 (0.7%)	13 (0.9%)	23 (0.8%)
	recovering/resolving	2 (0.1%)	2 (0.1%)	4 (0.1%)
	not recovered/not resolved	7 (0.5%)	9 (0.6%)	16 (0.6%)
	recovered/resolved with resid. effects	1 (<0.1%)	1 (<0.1%)	2 (<0.1%)
Deep vein thrombosis	recovered/resolved	1 (<0.1%)	0	1 (<0.1%)
Hot flush	recovered/resolved	2 (0.1%)	1 (<0.1%)	3 (0.1%)
	not recovered/not resolved	2 (0.1%)	2 (0.1%)	4 (0.1%)

Table 14.3.1 / 14: Number of subjects with adverse events by outcome, primary system organ class and preferred term (FAS)

Primary system organ class<br>Preferred term<br>MedDRA version 14.0	Maximum Derived AE outcome	LCS12<br>N=1432 (100%)	LCS16<br>N=1452 (100%)	Total<br>N=2884 (100%)
Hypertension	recovered/resolved	3 (0.2%)	6 (0.4%)	9 (0.3%)
	recovering/resolving	2 (0.1%)	2 (0.1%)	4 (0.1%)
	not recovered/not resolved	5 (0.3%)	6 (0.4%)	11 (0.4%)
	recovered/resolved with resid. effects	1 (<0.1%)	1 (<0.1%)	2 (<0.1%)
Hypotension	recovered/resolved	1 (<0.1%)	2 (0.1%)	3 (0.1%)
Neurogenic shock	recovered/resolved	2 (0.1%)	0	2 (<0.1%)
Phlebitis	recovered/resolved	0	1 (<0.1%)	1 (<0.1%)
Thrombophlebitis superficial	recovered/resolved	1 (<0.1%)	0	1 (<0.1%)
Varicose vein	recovered/resolved	1 (<0.1%)	3 (0.2%)	4 (0.1%)
	not recovered/not resolved	0	2 (0.1%)	2 (<0.1%)
Vein pain	not recovered/not resolved	0	1 (<0.1%)	1 (<0.1%)

Note: A subject is counted only once within each preferred term of any primary SOC.
Note: Adverse events are sorted in alphabetical order by primary SOC and preferred term.
Note: Only the most severe outcome is counted for multiple occurrences of the same AE in one individual.
Code for outcome=continuing with change is recoded to 0.5 with the possible smallest code for outcome
Global Biostatistics: /by-sasp/patdb/projects/de04209/310442/stat/prod_interim03/pgms/t-ae.sas epkll 12OCT2011 11:24
End of table

Table 14.3.1 / 15: Number of subjects with common (=> 1%) adverse events by preferred term (FAS)

Preferred term<br>MedDRA version 14.0	LCS12<br>N=1432 (100%)	LCS16<br>N=1452 (100%)	Total<br>N=2884 (100%)
Ovarian cyst	186 (13.0%)	304 (20.9%)	490 (17.0%)
Acne	163 (11.4%)	169 (11.6%)	332 (11.5%)
Urinary tract infection	158 (11.0%)	145 (10.0%)	303 (10.5%)
Headache	133 (9.3%)	137 (9.4%)	270 (9.4%)
Dysmenorrhoea	130 (9.1%)	108 (7.4%)	238 (8.3%)
Cervical dysplasia	107 (7.5%)	115 (7.9%)	222 (7.7%)
Vaginitis bacterial	105 (7.3%)	127 (8.7%)	232 (8.0%)
Nasopharyngitis	103 (7.2%)	109 (7.5%)	212 (7.4%)
Abdominal pain	100 (7.0%)	101 (7.0%)	201 (7.0%)
Vulvovaginal mycotic infection	99 (6.9%)	110 (7.6%)	209 (7.2%)
Pelvic pain	96 (6.7%)	123 (8.5%)	219 (7.6%)
Sinusitis	90 (6.3%)	96 (6.6%)	186 (6.4%)
Nausea	74 (5.2%)	58 (4.0%)	132 (4.6%)
Influenza	72 (5.0%)	94 (6.5%)	166 (5.8%)
Vulvovaginal candidiasis	72 (5.0%)	72 (5.0%)	144 (5.0%)
Abdominal pain lower	67 (4.7%)	61 (4.2%)	128 (4.4%)
Vaginal haemorrhage	66 (4.6%)	73 (5.0%)	139 (4.8%)
Back pain	59 (4.1%)	65 (4.5%)	124 (4.3%)
Bronchitis	58 (4.1%)	38 (2.6%)	96 (3.3%)
Procedural pain	58 (4.1%)	54 (3.7%)	112 (3.9%)
Weight increased	56 (3.9%)	68 (4.7%)	124 (4.3%)
Vaginal discharge	55 (3.8%)	58 (4.0%)	113 (3.9%)
Depression	51 (3.6%)	49 (3.4%)	100 (3.5%)
Upper respiratory tract infection	51 (3.6%)	57 (3.9%)	108 (3.7%)
Vaginal infection	48 (3.4%)	60 (4.1%)	108 (3.7%)
Device expulsion	46 (3.2%)	37 (2.5%)	83 (2.9%)
Seasonal allergy	39 (2.7%)	44 (3.0%)	83 (2.9%)
Anxiety	37 (2.6%)	37 (2.5%)	74 (2.6%)
Breast pain	36 (2.5%)	43 (3.0%)	79 (2.7%)
Migraine	34 (2.4%)	38 (2.6%)	72 (2.5%)
Dyspareunia	33 (2.3%)	28 (1.9%)	61 (2.1%)
Breast tenderness	32 (2.2%)	35 (2.4%)	67 (2.3%)
Gastroenteritis	31 (2.2%)	29 (2.0%)	60 (2.1%)
Cystitis	30 (2.1%)	26 (1.8%)	56 (1.9%)
Uterine spasm	30 (2.1%)	39 (2.7%)	69 (2.4%)
Ear infection	27 (1.9%)	13 (0.9%)	40 (1.4%)
Libido decreased	27 (1.9%)	25 (1.7%)	52 (1.8%)
Tonsillitis	25 (1.7%)	24 (1.7%)	49 (1.7%)
Tooth infection	25 (1.7%)	15 (1.0%)	40 (1.4%)
Fatigue	24 (1.7%)	27 (1.9%)	51 (1.8%)
Pharyngitis streptococcal	23 (1.6%)	21 (1.4%)	44 (1.5%)
Insomnia	22 (1.5%)	29 (2.0%)	51 (1.8%)
Abdominal distension	21 (1.5%)	11 (0.8%)	32 (1.1%)
Arthralgia	21 (1.5%)	31 (2.1%)	52 (1.8%)
Diarrhoea	19 (1.3%)	27 (1.9%)	46 (1.6%)

Table 14.3.1 / 15: Number of subjects with common (=> 1%) adverse events by preferred term (FAS)

Preferred term<br>MedDRA version 14.0	LCS12<br>N=1432 (100%)	LCS16<br>N=1452 (100%)	Total<br>N=2884 (100%)
Pyrexia	18 (1.3%)	16 (1.1%)	34 (1.2%)
Smear cervix abnormal	18 (1.3%)	28 (1.9%)	46 (1.6%)
Oropharyngeal pain	17 (1.2%)	17 (1.2%)	34 (1.2%)
Premenstrual syndrome	17 (1.2%)	12 (0.8%)	29 (1.0%)
Toothache	17 (1.2%)	26 (1.8%)	43 (1.5%)
Vomiting	17 (1.2%)	24 (1.7%)	41 (1.4%)
Constipation	16 (1.1%)	18 (1.2%)	34 (1.2%)
Metrorrhagia	16 (1.1%)	12 (0.8%)	28 (1.0%)
Vulvovaginal pruritus	16 (1.1%)	17 (1.2%)	33 (1.1%)
Alopecia	15 (1.0%)	13 (0.9%)	28 (1.0%)
Conjunctivitis	15 (1.0%)	15 (1.0%)	30 (1.0%)
Mood altered	15 (1.0%)	10 (0.7%)	25 (0.9%)
Viral upper respiratory tract infection	15 (1.0%)	14 (1.0%)	29 (1.0%)
Pneumonia	14 (1.0%)	15 (1.0%)	29 (1.0%)
Gastritis	13 (0.9%)	16 (1.1%)	29 (1.0%)
Haemorrhagic ovarian cyst	13 (0.9%)	19 (1.3%)	32 (1.1%)
Pharyngitis	13 (0.9%)	21 (1.4%)	34 (1.2%)
Hypertension	11 (0.8%)	16 (1.1%)	27 (0.9%)
Respiratory tract infection	10 (0.7%)	16 (1.1%)	26 (0.9%)
Dizziness	9 (0.6%)	22 (1.5%)	31 (1.1%)
Pain in extremity	6 (0.4%)	15 (1.0%)	21 (0.7%)
Cervicitis	5 (0.3%)	15 (1.0%)	20 (0.7%)

Note: A subject is counted only once within each preferred term.
Note: Adverse events are sorted by decreasing frequency of the LCS12 treatment arm.
Global Biostatistics: /by-sasp/patdb/projects/de04209/310442/stat/prod_interim03/pgms/t-ae.sas epkll 12OCT2011 11:24
End of table

Table 14.3.1 / 16: Number of subjects with adverse events by maximum intensity, primary system organ class and preferred term (FAS)

Primary system organ class<br>Preferred term<br>MedDRA version 14.0	Maximum AE<br>maximum<br>intensity	LCS12<br>N=1432 (100%)	LCS16<br>N=1452 (100%)	Total<br>N=2884 (100%)
Number of subjects (%) with at least one AE	mild	313 (21.9%)	353 (24.3%)	666 (23.1%)
	moderate	616 (43.0%)	633 (43.6%)	1249 (43.3%)
	severe	261 (18.2%)	250 (17.2%)	511 (17.7%)
Blood and lymphatic system disorders	mild	12 (0.8%)	6 (0.4%)	18 (0.6%)
	moderate	2 (0.1%)	2 (0.1%)	4 (0.1%)
	severe	1 (<0.1%)	0	1 (<0.1%)
Anaemia	mild	5 (0.3%)	3 (0.2%)	8 (0.3%)
	moderate	1 (<0.1%)	0	1 (<0.1%)
Iron deficiency anaemia	mild	1 (<0.1%)	1 (<0.1%)	2 (<0.1%)
Leukocytosis	moderate	1 (<0.1%)	0	1 (<0.1%)
Lymphadenitis	mild	2 (0.1%)	0	2 (<0.1%)
	moderate	1 (<0.1%)	0	1 (<0.1%)
Lymphadenopathy	mild	3 (0.2%)	1 (<0.1%)	4 (0.1%)
	moderate	0	2 (0.1%)	2 (<0.1%)
Pancytopenia	mild	0	1 (<0.1%)	1 (<0.1%)
Spherocytic anaemia	severe	1 (<0.1%)	0	1 (<0.1%)
Splenic cyst	mild	1 (<0.1%)	0	1 (<0.1%)
Splenomegaly	moderate	1 (<0.1%)	0	1 (<0.1%)
Cardiac disorders	mild	6 (0.4%)	10 (0.7%)	16 (0.6%)
	moderate	3 (0.2%)	3 (0.2%)	6 (0.2%)
	severe	0	1 (<0.1%)	1 (<0.1%)
Arrhythmia	mild	0	2 (0.1%)	2 (<0.1%)
	moderate	1 (<0.1%)	0	1 (<0.1%)
Extrasystoles	mild	0	2 (0.1%)	2 (<0.1%)

Table 14.3.1 / 16: Number of subjects with adverse events by maximum intensity, primary system organ class and preferred term (FAS)

Primary system organ class<br>Preferred term<br>MedDRA version 14.0	Maximum AE maximum intensity	LCS12<br>N=1432 (100%)	LCS16<br>N=1452 (100%)	Total<br>N=2884 (100%)
Mitral valve prolapse	mild	0	2 (0.1%)	2 (<0.1%)
Palpitations	mild	2 (0.1%)	4 (0.3%)	6 (0.2%)
	moderate	2 (0.1%)	1 (<0.1%)	3 (0.1%)
	severe	0	1 (<0.1%)	1 (<0.1%)
Sinus tachycardia	moderate	0	1 (<0.1%)	1 (<0.1%)
Tachycardia	mild	4 (0.3%)	1 (<0.1%)	5 (0.2%)
	moderate	0	1 (<0.1%)	1 (<0.1%)
Ventricular extrasystoles	mild	0	1 (<0.1%)	1 (<0.1%)
Congenital, familial and genetic disorders	mild	3 (0.2%)	1 (<0.1%)	4 (0.1%)
Congenital hydronephrosis	mild	1 (<0.1%)	0	1 (<0.1%)
Dermoid cyst	mild	1 (<0.1%)	0	1 (<0.1%)
Myotonia congenita	mild	0	1 (<0.1%)	1 (<0.1%)
Urethral intrinsic sphincter deficiency	mild	1 (<0.1%)	0	1 (<0.1%)
Ear and labyrinth disorders	mild	10 (0.7%)	7 (0.5%)	17 (0.6%)
	moderate	4 (0.3%)	6 (0.4%)	10 (0.3%)
	severe	2 (0.1%)	1 (<0.1%)	3 (0.1%)
Deafness bilateral	severe	1 (<0.1%)	0	1 (<0.1%)
Ear pain	mild	0	2 (0.1%)	2 (<0.1%)
	moderate	0	1 (<0.1%)	1 (<0.1%)
Ear pruritus	mild	1 (<0.1%)	0	1 (<0.1%)
Meniere's disease	moderate	0	1 (<0.1%)	1 (<0.1%)
Middle ear effusion	mild	0	2 (0.1%)	2 (<0.1%)

Table 14.3.1 / 16: Number of subjects with adverse events by maximum intensity, primary system organ class and preferred term (FAS)

Primary system organ class<br>Preferred term<br>MedDRA version 14.0	Maximum AE<br>maximum<br>intensity	LCS12<br>N=1432 (100%)	LCS16<br>N=1452 (100%)	Total<br>N=2884 (100%)
Motion sickness	mild	4 (0.3%)	2 (0.1%)	6 (0.2%)
	moderate	0	1 (<0.1%)	1 (<0.1%)
Otorrhoea	moderate	1 (<0.1%)	1 (<0.1%)	2 (<0.1%)
Sudden hearing loss	moderate	1 (<0.1%)	0	1 (<0.1%)
Tinnitus	mild	2 (0.1%)	0	2 (<0.1%)
Tympanic membrane perforation	moderate	1 (<0.1%)	0	1 (<0.1%)
	severe	1 (<0.1%)	0	1 (<0.1%)
Vertigo	mild	3 (0.2%)	3 (0.2%)	6 (0.2%)
	moderate	1 (<0.1%)	2 (0.1%)	3 (0.1%)
	severe	0	1 (<0.1%)	1 (<0.1%)
Vertigo labyrinthine	mild	1 (<0.1%)	0	1 (<0.1%)
Vertigo positional	mild	1 (<0.1%)	0	1 (<0.1%)
	moderate	1 (<0.1%)	0	1 (<0.1%)
Endocrine disorders	mild	8 (0.6%)	14 (1.0%)	22 (0.8%)
	moderate	1 (<0.1%)	6 (0.4%)	7 (0.2%)
	severe	1 (<0.1%)	1 (<0.1%)	2 (<0.1%)
Goitre	mild	1 (<0.1%)	2 (0.1%)	3 (0.1%)
	moderate	0	3 (0.2%)	3 (0.1%)
	severe	1 (<0.1%)	0	1 (<0.1%)
Hyperthyroidism	mild	0	1 (<0.1%)	1 (<0.1%)
	moderate	0	1 (<0.1%)	1 (<0.1%)
Hypothyroidism	mild	6 (0.4%)	10 (0.7%)	16 (0.6%)
	moderate	1 (<0.1%)	2 (0.1%)	3 (0.1%)
	severe	0	1 (<0.1%)	1 (<0.1%)
Thyroid mass	mild	0	1 (<0.1%)	1 (<0.1%)

Table 14.3.1 / 16: Number of subjects with adverse events by maximum intensity, primary system organ class and preferred term (FAS)

Primary system organ class<br>Preferred term<br>MedDRA version 14.0	Maximum AE maximum intensity	LCS12<br>N=1432 (100%)	LCS16<br>N=1452 (100%)	Total<br>N=2884 (100%)
Thyroiditis	mild	1 (<0.1%)	0	1 (<0.1%)
Eye disorders	mild	9 (0.6%)	9 (0.6%)	18 (0.6%)
	moderate	15 (1.0%)	13 (0.9%)	28 (1.0%)
	severe	1 (<0.1%)	1 (<0.1%)	2 (<0.1%)
Blepharitis	mild	0	1 (<0.1%)	1 (<0.1%)
	severe	1 (<0.1%)	1 (<0.1%)	2 (<0.1%)
Chalazion	mild	0	1 (<0.1%)	1 (<0.1%)
Conjunctivitis	mild	7 (0.5%)	7 (0.5%)	14 (0.5%)
	moderate	8 (0.6%)	7 (0.5%)	15 (0.5%)
	severe	0	1 (<0.1%)	1 (<0.1%)
Conjunctivitis allergic	moderate	2 (0.1%)	2 (0.1%)	4 (0.1%)
Dry eye	moderate	2 (0.1%)	0	2 (<0.1%)
Eye inflammation	moderate	0	1 (<0.1%)	1 (<0.1%)
Eyelid cyst	moderate	0	1 (<0.1%)	1 (<0.1%)
Heterophoria	moderate	1 (<0.1%)	0	1 (<0.1%)
Iridocyclitis	moderate	1 (<0.1%)	0	1 (<0.1%)
Iritis	moderate	0	2 (0.1%)	2 (<0.1%)
Meibomianitis	mild	1 (<0.1%)	0	1 (<0.1%)
Myopia	moderate	0	1 (<0.1%)	1 (<0.1%)
Ocular icterus	mild	0	1 (<0.1%)	1 (<0.1%)
Panophthalmitis	mild	1 (<0.1%)	0	1 (<0.1%)

Table 14.3.1 / 16: Number of subjects with adverse events by maximum intensity, primary system organ class and preferred term (FAS)

Primary system organ class<br>Preferred term<br>MedDRA version 14.0	Maximum AE<br>maximum<br>intensity	LCS12<br>N=1432 (100%)	LCS16<br>N=1452 (100%)	Total<br>N=2884 (100%)
Scotoma	moderate	1 (<0.1%)	0	1 (<0.1%)
Vision blurred	mild	1 (<0.1%)	0	1 (<0.1%)
Gastrointestinal disorders	mild	129 (9.0%)	107 (7.4%)	236 (8.2%)
	moderate	146 (10.2%)	172 (11.8%)	318 (11.0%)
	severe	59 (4.1%)	40 (2.8%)	99 (3.4%)
Abdominal adhesions	severe	1 (<0.1%)	0	1 (<0.1%)
Abdominal discomfort	mild	3 (0.2%)	4 (0.3%)	7 (0.2%)
	moderate	1 (<0.1%)	2 (0.1%)	3 (0.1%)
	severe	1 (<0.1%)	0	1 (<0.1%)
Abdominal distension	mild	9 (0.6%)	8 (0.6%)	17 (0.6%)
	moderate	11 (0.8%)	3 (0.2%)	14 (0.5%)
	severe	1 (<0.1%)	0	1 (<0.1%)
Abdominal hernia	moderate	2 (0.1%)	0	2 (<0.1%)
Abdominal mass	mild	1 (<0.1%)	0	1 (<0.1%)
Abdominal pain	mild	27 (1.9%)	27 (1.9%)	54 (1.9%)
	moderate	44 (3.1%)	55 (3.8%)	99 (3.4%)
	severe	28 (2.0%)	19 (1.3%)	47 (1.6%)
Abdominal pain lower	mild	20 (1.4%)	14 (1.0%)	34 (1.2%)
	moderate	38 (2.7%)	41 (2.8%)	79 (2.7%)
	severe	9 (0.6%)	6 (0.4%)	15 (0.5%)
Abdominal pain upper	mild	3 (0.2%)	6 (0.4%)	9 (0.3%)
	moderate	3 (0.2%)	4 (0.3%)	7 (0.2%)
	severe	1 (<0.1%)	1 (<0.1%)	2 (<0.1%)
Abdominal tenderness	mild	1 (<0.1%)	0	1 (<0.1%)
	moderate	1 (<0.1%)	0	1 (<0.1%)

Table 14.3.1 / 16: Number of subjects with adverse events by maximum intensity, primary system organ class and preferred term (FAS)

Primary system organ class<br>Preferred term<br>MedDRA version 14.0	Maximum AE<br>maximum<br>intensity	LCS12<br>N=1432 (100%)	LCS16<br>N=1452 (100%)	Total<br>N=2884 (100%)
Anal fissure	mild	0	1 (<0.1%)	1 (<0.1%)
	moderate	0	1 (<0.1%)	1 (<0.1%)
	severe	1 (<0.1%)	0	1 (<0.1%)
Anal pruritus	mild	2 (0.1%)	1 (<0.1%)	3 (0.1%)
	moderate	1 (<0.1%)	1 (<0.1%)	2 (<0.1%)
Anal skin tags	moderate	0	1 (<0.1%)	1 (<0.1%)
Aphthous stomatitis	severe	0	1 (<0.1%)	1 (<0.1%)
Breath odour	mild	1 (<0.1%)	0	1 (<0.1%)
Coeliac disease	mild	1 (<0.1%)	0	1 (<0.1%)
Colitis	mild	0	1 (<0.1%)	1 (<0.1%)
	moderate	1 (<0.1%)	2 (0.1%)	3 (0.1%)
Colitis ulcerative	mild	1 (<0.1%)	0	1 (<0.1%)
	moderate	1 (<0.1%)	0	1 (<0.1%)
Colonic polyp	moderate	1 (<0.1%)	0	1 (<0.1%)
Constipation	mild	7 (0.5%)	6 (0.4%)	13 (0.5%)
	moderate	6 (0.4%)	9 (0.6%)	15 (0.5%)
	severe	3 (0.2%)	3 (0.2%)	6 (0.2%)
Crohn's disease	moderate	1 (<0.1%)	0	1 (<0.1%)
	severe	1 (<0.1%)	0	1 (<0.1%)
Dental caries	mild	1 (<0.1%)	0	1 (<0.1%)
	moderate	0	3 (0.2%)	3 (0.1%)
Diarrhoea	mild	11 (0.8%)	11 (0.8%)	22 (0.8%)
	moderate	7 (0.5%)	14 (1.0%)	21 (0.7%)
	severe	1 (<0.1%)	2 (0.1%)	3 (0.1%)
Dry mouth	mild	0	1 (<0.1%)	1 (<0.1%)

Table 14.3.1 / 16: Number of subjects with adverse events by maximum intensity, primary system organ class and preferred term (FAS)

Primary system organ class<br>Preferred term<br>MedDRA version 14.0	Maximum AE<br>maximum<br>intensity	LCS12<br>N=1432 (100%)	LCS16<br>N=1452 (100%)	Total<br>N=2884 (100%)
Duodenal ulcer	mild	1 (<0.1%)	0	1 (<0.1%)
Dyspepsia	mild	8 (0.6%)	6 (0.4%)	14 (0.5%)
	moderate	5 (0.3%)	6 (0.4%)	11 (0.4%)
	severe	1 (<0.1%)	0	1 (<0.1%)
Dysphagia	moderate	1 (<0.1%)	0	1 (<0.1%)
	severe	0	1 (<0.1%)	1 (<0.1%)
Flatulence	mild	2 (0.1%)	1 (<0.1%)	3 (0.1%)
	moderate	0	1 (<0.1%)	1 (<0.1%)
Food poisoning	moderate	1 (<0.1%)	4 (0.3%)	5 (0.2%)
	severe	0	2 (0.1%)	2 (<0.1%)
Frequent bowel movements	mild	1 (<0.1%)	0	1 (<0.1%)
	moderate	1 (<0.1%)	0	1 (<0.1%)
Gastric ulcer	mild	2 (0.1%)	0	2 (<0.1%)
	moderate	0	1 (<0.1%)	1 (<0.1%)
Gastritis	mild	6 (0.4%)	6 (0.4%)	12 (0.4%)
	moderate	5 (0.3%)	8 (0.6%)	13 (0.5%)
	severe	2 (0.1%)	2 (0.1%)	4 (0.1%)
Gastrointestinal disorder	moderate	1 (<0.1%)	0	1 (<0.1%)
Gastrointestinal pain	mild	1 (<0.1%)	0	1 (<0.1%)
	moderate	0	1 (<0.1%)	1 (<0.1%)
Gastrooesophageal reflux disease	mild	4 (0.3%)	7 (0.5%)	11 (0.4%)
	moderate	5 (0.3%)	4 (0.3%)	9 (0.3%)
Gingival oedema	mild	1 (<0.1%)	0	1 (<0.1%)
Gingival recession	moderate	1 (<0.1%)	0	1 (<0.1%)

Table 14.3.1 / 16: Number of subjects with adverse events by maximum intensity, primary system organ class and preferred term (FAS)

Primary system organ class<br>Preferred term<br>MedDRA version 14.0	Maximum AE maximum intensity	LCS12<br>N=1432 (100%)	LCS16<br>N=1452 (100%)	Total<br>N=2884 (100%)
Gingivitis	mild	0	1 (<0.1%)	1 (<0.1%)
	moderate	1 (<0.1%)	0	1 (<0.1%)
Haematochezia	mild	1 (<0.1%)	0	1 (<0.1%)
	moderate	0	1 (<0.1%)	1 (<0.1%)
Haemorrhoids	mild	3 (0.2%)	5 (0.3%)	8 (0.3%)
	moderate	3 (0.2%)	3 (0.2%)	6 (0.2%)
	severe	1 (<0.1%)	0	1 (<0.1%)
Hiatus hernia	moderate	0	1 (<0.1%)	1 (<0.1%)
Hyperchlorhydria	mild	2 (0.1%)	1 (<0.1%)	3 (0.1%)
Impaired gastric emptying	severe	1 (<0.1%)	0	1 (<0.1%)
Inflammatory bowel disease	mild	0	1 (<0.1%)	1 (<0.1%)
Inguinal hernia	moderate	1 (<0.1%)	0	1 (<0.1%)
Irritable bowel syndrome	mild	8 (0.6%)	5 (0.3%)	13 (0.5%)
	moderate	1 (<0.1%)	0	1 (<0.1%)
	severe	2 (0.1%)	0	2 (<0.1%)
Mouth ulceration	mild	0	1 (<0.1%)	1 (<0.1%)
	moderate	0	1 (<0.1%)	1 (<0.1%)
Nausea	mild	44 (3.1%)	31 (2.1%)	75 (2.6%)
	moderate	23 (1.6%)	25 (1.7%)	48 (1.7%)
	severe	7 (0.5%)	1 (<0.1%)	8 (0.3%)
Odynophagia	severe	0	1 (<0.1%)	1 (<0.1%)
Oesophageal stenosis	moderate	0	1 (<0.1%)	1 (<0.1%)
Oesophagitis	mild	1 (<0.1%)	0	1 (<0.1%)
Oral pain	moderate	0	1 (<0.1%)	1 (<0.1%)

Table 14.3.1 / 16: Number of subjects with adverse events by maximum intensity, primary system organ class and preferred term (FAS)

Primary system organ class<br>Preferred term<br>MedDRA version 14.0	Maximum AE<br>maximum<br>intensity	LCS12<br>N=1432 (100%)	LCS16<br>N=1452 (100%)	Total<br>N=2884 (100%)
Peptic ulcer	moderate	1 (<0.1%)	0	1 (<0.1%)
Periodontitis	mild	2 (0.1%)	0	2 (<0.1%)
	moderate	0	1 (<0.1%)	1 (<0.1%)
Peritonitis	severe	1 (<0.1%)	0	1 (<0.1%)
Proctalgia	moderate	2 (0.1%)	1 (<0.1%)	3 (0.1%)
Rectal haemorrhage	mild	0	1 (<0.1%)	1 (<0.1%)
	moderate	0	2 (0.1%)	2 (<0.1%)
Reflux oesophagitis	moderate	0	1 (<0.1%)	1 (<0.1%)
Salivary gland calculus	severe	0	1 (<0.1%)	1 (<0.1%)
Tooth disorder	mild	1 (<0.1%)	1 (<0.1%)	2 (<0.1%)
	moderate	2 (0.1%)	0	2 (<0.1%)
Tooth impacted	mild	1 (<0.1%)	0	1 (<0.1%)
	moderate	1 (<0.1%)	0	1 (<0.1%)
Toothache	mild	2 (0.1%)	9 (0.6%)	11 (0.4%)
	moderate	10 (0.7%)	13 (0.9%)	23 (0.8%)
	severe	5 (0.3%)	4 (0.3%)	9 (0.3%)
Umbilical hernia	moderate	0	1 (<0.1%)	1 (<0.1%)
Vomiting	mild	7 (0.5%)	9 (0.6%)	16 (0.6%)
	moderate	8 (0.6%)	14 (1.0%)	22 (0.8%)
	severe	2 (0.1%)	1 (<0.1%)	3 (0.1%)
General disorders and administration site conditions	mild	60 (4.2%)	70 (4.8%)	130 (4.5%)
	moderate	41 (2.9%)	37 (2.5%)	78 (2.7%)
	severe	10 (0.7%)	10 (0.7%)	20 (0.7%)
Asthenia	mild	1 (<0.1%)	0	1 (<0.1%)
	moderate	1 (<0.1%)	1 (<0.1%)	2 (<0.1%)

Table 14.3.1 / 16: Number of subjects with adverse events by maximum intensity, primary system organ class and preferred term (FAS)

Primary system organ class<br>Preferred term<br>MedDRA version 14.0	Maximum AE<br>maximum<br>intensity	LCS12<br>N=1432 (100%)	LCS16<br>N=1452 (100%)	Total<br>N=2884 (100%)
Axillary pain	mild	1 (<0.1%)	0	1 (<0.1%)
	severe	0	1 (<0.1%)	1 (<0.1%)
Chest pain	mild	1 (<0.1%)	4 (0.3%)	5 (0.2%)
	moderate	2 (0.1%)	3 (0.2%)	5 (0.2%)
	severe	2 (0.1%)	0	2 (<0.1%)
Chills	mild	0	1 (<0.1%)	1 (<0.1%)
	moderate	0	1 (<0.1%)	1 (<0.1%)
Cyst	mild	3 (0.2%)	1 (<0.1%)	4 (0.1%)
Device dislocation	mild	3 (0.2%)	2 (0.1%)	5 (0.2%)
	moderate	0	3 (0.2%)	3 (0.1%)
Device expulsion	mild	34 (2.4%)	28 (1.9%)	62 (2.1%)
	moderate	6 (0.4%)	1 (<0.1%)	7 (0.2%)
	severe	4 (0.3%)	6 (0.4%)	10 (0.3%)
Discomfort	mild	1 (<0.1%)	1 (<0.1%)	2 (<0.1%)
	moderate	1 (<0.1%)	0	1 (<0.1%)
Drug withdrawal syndrome	severe	1 (<0.1%)	0	1 (<0.1%)
Fatigue	mild	5 (0.3%)	16 (1.1%)	21 (0.7%)
	moderate	17 (1.2%)	9 (0.6%)	26 (0.9%)
	severe	2 (0.1%)	2 (0.1%)	4 (0.1%)
Feeling cold	mild	0	2 (0.1%)	2 (<0.1%)
Feeling hot	mild	0	1 (<0.1%)	1 (<0.1%)
Generalised oedema	moderate	1 (<0.1%)	0	1 (<0.1%)
Hangover	moderate	1 (<0.1%)	0	1 (<0.1%)
Hunger	severe	0	1 (<0.1%)	1 (<0.1%)

Table 14.3.1 / 16: Number of subjects with adverse events by maximum intensity, primary system organ class and preferred term (FAS)

Primary system organ class<br>Preferred term<br>MedDRA version 14.0	Maximum AE maximum intensity	LCS12<br>N=1432 (100%)	LCS16<br>N=1452 (100%)	Total<br>N=2884 (100%)
Inflammation	mild	0	1 (<0.1%)	1 (<0.1%)
	moderate	1 (<0.1%)	0	1 (<0.1%)
Influenza like illness	moderate	1 (<0.1%)	1 (<0.1%)	2 (<0.1%)
Injury associated with device	mild	0	1 (<0.1%)	1 (<0.1%)
Irritability	mild	2 (0.1%)	1 (<0.1%)	3 (0.1%)
	moderate	0	5 (0.3%)	5 (0.2%)
Localised oedema	mild	0	1 (<0.1%)	1 (<0.1%)
Malaise	moderate	0	1 (<0.1%)	1 (<0.1%)
Oedema	mild	0	1 (<0.1%)	1 (<0.1%)
Oedema peripheral	mild	1 (<0.1%)	5 (0.3%)	6 (0.2%)
	moderate	3 (0.2%)	1 (<0.1%)	4 (0.1%)
Pain	mild	4 (0.3%)	1 (<0.1%)	5 (0.2%)
	moderate	1 (<0.1%)	4 (0.3%)	5 (0.2%)
	severe	1 (<0.1%)	0	1 (<0.1%)
Pyrexia	mild	8 (0.6%)	7 (0.5%)	15 (0.5%)
	moderate	10 (0.7%)	9 (0.6%)	19 (0.7%)
Vaccination site pain	mild	1 (<0.1%)	1 (<0.1%)	2 (<0.1%)
Hepatobiliary disorders	mild	2 (0.1%)	2 (0.1%)	4 (0.1%)
	moderate	2 (0.1%)	5 (0.3%)	7 (0.2%)
	severe	4 (0.3%)	4 (0.3%)	8 (0.3%)
Biliary colic	moderate	0	1 (<0.1%)	1 (<0.1%)
	severe	0	1 (<0.1%)	1 (<0.1%)
Biliary dyskinesia	moderate	1 (<0.1%)	0	1 (<0.1%)
	severe	0	1 (<0.1%)	1 (<0.1%)

Table 14.3.1 / 16: Number of subjects with adverse events by maximum intensity, primary system organ class and preferred term (FAS)

Primary system organ class<br>Preferred term<br>MedDRA version 14.0	Maximum AE<br>maximum<br>intensity	LCS12<br>N=1432 (100%)	LCS16<br>N=1452 (100%)	Total<br>N=2884 (100%)
Cholecystitis	moderate	1 (<0.1%)	1 (<0.1%)	2 (<0.1%)
	severe	2 (0.1%)	1 (<0.1%)	3 (0.1%)
Cholecystitis chronic	mild	1 (<0.1%)	0	1 (<0.1%)
	severe	1 (<0.1%)	0	1 (<0.1%)
Cholelithiasis	mild	1 (<0.1%)	0	1 (<0.1%)
	moderate	2 (0.1%)	3 (0.2%)	5 (0.2%)
	severe	1 (<0.1%)	1 (<0.1%)	2 (<0.1%)
Gallbladder pain	moderate	0	1 (<0.1%)	1 (<0.1%)
Gallbladder polyp	moderate	1 (<0.1%)	0	1 (<0.1%)
Hepatic steatosis	mild	0	1 (<0.1%)	1 (<0.1%)
Hyperbilirubinaemia	mild	0	1 (<0.1%)	1 (<0.1%)
Immune system disorders	mild	31 (2.2%)	40 (2.8%)	71 (2.5%)
	moderate	30 (2.1%)	24 (1.7%)	54 (1.9%)
	severe	2 (0.1%)	2 (0.1%)	4 (0.1%)
Allergy to animal	mild	0	2 (0.1%)	2 (<0.1%)
	moderate	1 (<0.1%)	0	1 (<0.1%)
Allergy to arthropod bite	moderate	1 (<0.1%)	1 (<0.1%)	2 (<0.1%)
Allergy to arthropod sting	moderate	0	1 (<0.1%)	1 (<0.1%)
Allergy to chemicals	mild	1 (<0.1%)	0	1 (<0.1%)
Anaphylactic reaction	severe	0	1 (<0.1%)	1 (<0.1%)
Drug hypersensitivity	mild	4 (0.3%)	1 (<0.1%)	5 (0.2%)
	moderate	4 (0.3%)	5 (0.3%)	9 (0.3%)
	severe	1 (<0.1%)	0	1 (<0.1%)

Table 14.3.1 / 16: Number of subjects with adverse events by maximum intensity, primary system organ class and preferred term (FAS)

Primary system organ class<br>Preferred term<br>MedDRA version 14.0	Maximum AE maximum intensity	LCS12<br>N=1432 (100%)	LCS16<br>N=1452 (100%)	Total<br>N=2884 (100%)
Food allergy	mild	0	1 (<0.1%)	1 (<0.1%)
	moderate	1 (<0.1%)	0	1 (<0.1%)
Hypersensitivity	mild	5 (0.3%)	4 (0.3%)	9 (0.3%)
	moderate	7 (0.5%)	6 (0.4%)	13 (0.5%)
	severe	1 (<0.1%)	1 (<0.1%)	2 (<0.1%)
Seasonal allergy	mild	23 (1.6%)	32 (2.2%)	55 (1.9%)
	moderate	16 (1.1%)	12 (0.8%)	28 (1.0%)
Infections and infestations	mild	270 (18.9%)	290 (20.0%)	560 (19.4%)
	moderate	389 (27.2%)	392 (27.0%)	781 (27.1%)
	severe	63 (4.4%)	54 (3.7%)	117 (4.1%)
Abscess limb	mild	0	1 (<0.1%)	1 (<0.1%)
Acarodermatitis	moderate	1 (<0.1%)	0	1 (<0.1%)
	severe	1 (<0.1%)	0	1 (<0.1%)
Acute sinusitis	mild	3 (0.2%)	1 (<0.1%)	4 (0.1%)
	moderate	4 (0.3%)	2 (0.1%)	6 (0.2%)
Acute tonsillitis	mild	6 (0.4%)	4 (0.3%)	10 (0.3%)
	moderate	4 (0.3%)	1 (<0.1%)	5 (0.2%)
Anogenital warts	mild	7 (0.5%)	8 (0.6%)	15 (0.5%)
	moderate	3 (0.2%)	2 (0.1%)	5 (0.2%)
	severe	1 (<0.1%)	0	1 (<0.1%)
Appendicitis	moderate	2 (0.1%)	1 (<0.1%)	3 (0.1%)
	severe	4 (0.3%)	6 (0.4%)	10 (0.3%)
Arthritis rubella	moderate	1 (<0.1%)	0	1 (<0.1%)
Bartholin's abscess	mild	2 (0.1%)	0	2 (<0.1%)
	moderate	1 (<0.1%)	0	1 (<0.1%)
Blastocystis infection	moderate	0	1 (<0.1%)	1 (<0.1%)

Table 14.3.1 / 16: Number of subjects with adverse events by maximum intensity, primary system organ class and preferred term (FAS)

Primary system organ class<br>Preferred term<br>MedDRA version 14.0	Maximum AE<br>maximum<br>intensity	LCS12<br>N=1432 (100%)	LCS16<br>N=1452 (100%)	Total<br>N=2884 (100%)
Body tinea	mild	1 (<0.1%)	2 (0.1%)	3 (0.1%)
	moderate	1 (<0.1%)	0	1 (<0.1%)
Breast abscess	moderate	1 (<0.1%)	0	1 (<0.1%)
Bronchitis	mild	21 (1.5%)	13 (0.9%)	34 (1.2%)
	moderate	36 (2.5%)	23 (1.6%)	59 (2.0%)
	severe	1 (<0.1%)	2 (0.1%)	3 (0.1%)
Bronchitis viral	mild	1 (<0.1%)	0	1 (<0.1%)
Bronchopneumonia	severe	1 (<0.1%)	0	1 (<0.1%)
Bursitis infective	mild	1 (<0.1%)	0	1 (<0.1%)
Campylobacter infection	moderate	1 (<0.1%)	0	1 (<0.1%)
Campylobacter intestinal infection	severe	0	1 (<0.1%)	1 (<0.1%)
Candidiasis	mild	8 (0.6%)	7 (0.5%)	15 (0.5%)
	moderate	2 (0.1%)	1 (<0.1%)	3 (0.1%)
	severe	1 (<0.1%)	1 (<0.1%)	2 (<0.1%)
Carbuncle	mild	0	1 (<0.1%)	1 (<0.1%)
Cellulitis	mild	0	1 (<0.1%)	1 (<0.1%)
	moderate	2 (0.1%)	2 (0.1%)	4 (0.1%)
	severe	1 (<0.1%)	1 (<0.1%)	2 (<0.1%)
Cervicitis	mild	4 (0.3%)	10 (0.7%)	14 (0.5%)
	moderate	1 (<0.1%)	4 (0.3%)	5 (0.2%)
	severe	0	1 (<0.1%)	1 (<0.1%)
Cervicitis gonococcal	mild	1 (<0.1%)	0	1 (<0.1%)
Chlamydial cervicitis	mild	2 (0.1%)	4 (0.3%)	6 (0.2%)
	moderate	0	2 (0.1%)	2 (<0.1%)

Table 14.3.1 / 16: Number of subjects with adverse events by maximum intensity, primary system organ class and preferred term (FAS)

Primary system organ class<br>Preferred term<br>MedDRA version 14.0	Maximum AE maximum intensity	LCS12<br>N=1432 (100%)	LCS16<br>N=1452 (100%)	Total<br>N=2884 (100%)
Chlamydial infection	mild	1 (<0.1%)	3 (0.2%)	4 (0.1%)
	moderate	0	1 (<0.1%)	1 (<0.1%)
Chronic tonsillitis	moderate	0	1 (<0.1%)	1 (<0.1%)
Clitoris abscess	moderate	1 (<0.1%)	0	1 (<0.1%)
Conjunctivitis bacterial	mild	0	1 (<0.1%)	1 (<0.1%)
	moderate	1 (<0.1%)	1 (<0.1%)	2 (<0.1%)
Conjunctivitis infective	mild	1 (<0.1%)	0	1 (<0.1%)
	moderate	1 (<0.1%)	0	1 (<0.1%)
Conjunctivitis viral	mild	1 (<0.1%)	0	1 (<0.1%)
	moderate	0	1 (<0.1%)	1 (<0.1%)
Coxsackie viral infection	mild	1 (<0.1%)	0	1 (<0.1%)
	moderate	1 (<0.1%)	1 (<0.1%)	2 (<0.1%)
Cystitis	mild	11 (0.8%)	14 (1.0%)	25 (0.9%)
	moderate	16 (1.1%)	10 (0.7%)	26 (0.9%)
	severe	3 (0.2%)	2 (0.1%)	5 (0.2%)
Dengue fever	mild	1 (<0.1%)	0	1 (<0.1%)
	moderate	1 (<0.1%)	0	1 (<0.1%)
Diarrhoea infectious	mild	1 (<0.1%)	1 (<0.1%)	2 (<0.1%)
	severe	0	1 (<0.1%)	1 (<0.1%)
Diverticulitis	moderate	0	1 (<0.1%)	1 (<0.1%)
	severe	1 (<0.1%)	0	1 (<0.1%)
Ear infection	mild	8 (0.6%)	1 (<0.1%)	9 (0.3%)
	moderate	16 (1.1%)	11 (0.8%)	27 (0.9%)
	severe	3 (0.2%)	1 (<0.1%)	4 (0.1%)
Ear infection bacterial	moderate	1 (<0.1%)	0	1 (<0.1%)

Table 14.3.1 / 16: Number of subjects with adverse events by maximum intensity, primary system organ class and preferred term (FAS)

Primary system organ class<br>Preferred term<br>MedDRA version 14.0	Maximum AE<br>maximum<br>intensity	LCS12<br>N=1432 (100%)	LCS16<br>N=1452 (100%)	Total<br>N=2884 (100%)
Endometritis	mild	5 (0.3%)	3 (0.2%)	8 (0.3%)
	moderate	5 (0.3%)	8 (0.6%)	13 (0.5%)
	severe	1 (<0.1%)	0	1 (<0.1%)
Enterobiasis	mild	3 (0.2%)	0	3 (0.1%)
	moderate	0	1 (<0.1%)	1 (<0.1%)
Enterocolitis infectious	moderate	2 (0.1%)	0	2 (<0.1%)
Epstein-Barr virus infection	mild	0	1 (<0.1%)	1 (<0.1%)
Erysipelas	mild	1 (<0.1%)	0	1 (<0.1%)
Erythema infectiosum	moderate	1 (<0.1%)	0	1 (<0.1%)
Escherichia urinary tract infection	mild	0	1 (<0.1%)	1 (<0.1%)
Escherichia vaginitis	mild	1 (<0.1%)	0	1 (<0.1%)
Eye infection	mild	0	1 (<0.1%)	1 (<0.1%)
Eyelid infection	mild	1 (<0.1%)	0	1 (<0.1%)
Folliculitis	mild	3 (0.2%)	3 (0.2%)	6 (0.2%)
	moderate	2 (0.1%)	3 (0.2%)	5 (0.2%)
Fungal infection	mild	3 (0.2%)	7 (0.5%)	10 (0.3%)
	moderate	2 (0.1%)	1 (<0.1%)	3 (0.1%)
Fungal skin infection	mild	2 (0.1%)	1 (<0.1%)	3 (0.1%)
	moderate	1 (<0.1%)	0	1 (<0.1%)
Furuncle	mild	1 (<0.1%)	0	1 (<0.1%)
	moderate	0	1 (<0.1%)	1 (<0.1%)
Gastritis bacterial	mild	0	1 (<0.1%)	1 (<0.1%)

Table 14.3.1 / 16: Number of subjects with adverse events by maximum intensity, primary system organ class and preferred term (FAS)

Primary system organ class<br>Preferred term<br>MedDRA version 14.0	Maximum AE maximum intensity	LCS12<br>N=1432 (100%)	LCS16<br>N=1452 (100%)	Total<br>N=2884 (100%)
Gastritis viral	mild	0	1 (<0.1%)	1 (<0.1%)
Gastroenteritis	mild	9 (0.6%)	9 (0.6%)	18 (0.6%)
	moderate	19 (1.3%)	18 (1.2%)	37 (1.3%)
	severe	3 (0.2%)	2 (0.1%)	5 (0.2%)
Gastroenteritis rotavirus	mild	0	1 (<0.1%)	1 (<0.1%)
Gastroenteritis viral	mild	3 (0.2%)	2 (0.1%)	5 (0.2%)
	moderate	3 (0.2%)	5 (0.3%)	8 (0.3%)
	severe	1 (<0.1%)	1 (<0.1%)	2 (<0.1%)
Gastrointestinal bacterial infection	mild	0	1 (<0.1%)	1 (<0.1%)
Gastrointestinal viral infection	mild	0	1 (<0.1%)	1 (<0.1%)
	moderate	1 (<0.1%)	1 (<0.1%)	2 (<0.1%)
Genital candidiasis	mild	1 (<0.1%)	1 (<0.1%)	2 (<0.1%)
	moderate	0	2 (0.1%)	2 (<0.1%)
Genital herpes	mild	4 (0.3%)	4 (0.3%)	8 (0.3%)
	moderate	5 (0.3%)	10 (0.7%)	15 (0.5%)
	severe	1 (<0.1%)	0	1 (<0.1%)
Genital infection fungal	moderate	1 (<0.1%)	0	1 (<0.1%)
Giardiasis	mild	1 (<0.1%)	0	1 (<0.1%)
Gonorrhoea	mild	0	1 (<0.1%)	1 (<0.1%)
	moderate	1 (<0.1%)	0	1 (<0.1%)
Groin infection	mild	2 (0.1%)	0	2 (<0.1%)
Gynaecological chlamydia infection	mild	2 (0.1%)	3 (0.2%)	5 (0.2%)
	moderate	1 (<0.1%)	2 (0.1%)	3 (0.1%)

Table 14.3.1 / 16: Number of subjects with adverse events by maximum intensity, primary system organ class and preferred term (FAS)

Primary system organ class<br>Preferred term<br>MedDRA version 14.0	Maximum AE maximum intensity	LCS12<br>N=1432 (100%)	LCS16<br>N=1452 (100%)	Total<br>N=2884 (100%)
H1N1 influenza	mild	2 (0.1%)	1 (<0.1%)	3 (0.1%)
	moderate	4 (0.3%)	4 (0.3%)	8 (0.3%)
	severe	1 (<0.1%)	0	1 (<0.1%)
Haemophilus infection	mild	0	1 (<0.1%)	1 (<0.1%)
Hand-foot-and-mouth disease	moderate	0	1 (<0.1%)	1 (<0.1%)
Helicobacter gastritis	mild	1 (<0.1%)	1 (<0.1%)	2 (<0.1%)
Helicobacter infection	mild	1 (<0.1%)	0	1 (<0.1%)
Herpes dermatitis	moderate	1 (<0.1%)	1 (<0.1%)	2 (<0.1%)
Herpes simplex	mild	0	1 (<0.1%)	1 (<0.1%)
	moderate	0	2 (0.1%)	2 (<0.1%)
	severe	1 (<0.1%)	0	1 (<0.1%)
Herpes zoster	mild	2 (0.1%)	0	2 (<0.1%)
	moderate	4 (0.3%)	4 (0.3%)	8 (0.3%)
	severe	1 (<0.1%)	0	1 (<0.1%)
Impetigo	mild	1 (<0.1%)	2 (0.1%)	3 (0.1%)
	moderate	3 (0.2%)	0	3 (0.1%)
Infected bites	mild	2 (0.1%)	0	2 (<0.1%)
Infected cyst	mild	0	1 (<0.1%)	1 (<0.1%)
Infection	moderate	0	1 (<0.1%)	1 (<0.1%)
Infectious mononucleosis	mild	0	2 (0.1%)	2 (<0.1%)
	moderate	1 (<0.1%)	4 (0.3%)	5 (0.2%)
Influenza	mild	25 (1.7%)	31 (2.1%)	56 (1.9%)
	moderate	42 (2.9%)	55 (3.8%)	97 (3.4%)
	severe	5 (0.3%)	8 (0.6%)	13 (0.5%)

Table 14.3.1 / 16: Number of subjects with adverse events by maximum intensity, primary system organ class and preferred term (FAS)

Primary system organ class<br>Preferred term<br>MedDRA version 14.0	Maximum AE<br>maximum<br>intensity	LCS12<br>N=1432 (100%)	LCS16<br>N=1452 (100%)	Total<br>N=2884 (100%)
Keratitis viral	moderate	0	1 (<0.1%)	1 (<0.1%)
Kidney infection	mild	1 (<0.1%)	0	1 (<0.1%)
	moderate	1 (<0.1%)	4 (0.3%)	5 (0.2%)
Labyrinthitis	moderate	1 (<0.1%)	1 (<0.1%)	2 (<0.1%)
Laryngitis	mild	2 (0.1%)	5 (0.3%)	7 (0.2%)
	moderate	4 (0.3%)	4 (0.3%)	8 (0.3%)
	severe	1 (<0.1%)	1 (<0.1%)	2 (<0.1%)
Laryngitis bacterial	moderate	0	1 (<0.1%)	1 (<0.1%)
Localised infection	mild	3 (0.2%)	1 (<0.1%)	4 (0.1%)
	moderate	1 (<0.1%)	0	1 (<0.1%)
	severe	0	1 (<0.1%)	1 (<0.1%)
Lower respiratory tract infection	mild	1 (<0.1%)	0	1 (<0.1%)
	moderate	2 (0.1%)	1 (<0.1%)	3 (0.1%)
Lower respiratory tract infection bacterial	moderate	1 (<0.1%)	0	1 (<0.1%)
Lymph gland infection	moderate	1 (<0.1%)	0	1 (<0.1%)
Malaria	moderate	1 (<0.1%)	1 (<0.1%)	2 (<0.1%)
Mastitis	mild	1 (<0.1%)	0	1 (<0.1%)
	moderate	2 (0.1%)	1 (<0.1%)	3 (0.1%)
Meningitis	moderate	1 (<0.1%)	0	1 (<0.1%)
Meningitis viral	severe	0	1 (<0.1%)	1 (<0.1%)
Molluscum contagiosum	mild	0	1 (<0.1%)	1 (<0.1%)
Nail infection	moderate	2 (0.1%)	1 (<0.1%)	3 (0.1%)

Table 14.3.1 / 16: Number of subjects with adverse events by maximum intensity, primary system organ class and preferred term (FAS)

Primary system organ class Preferred term MedDRA version 14.0	Maximum AE maximum intensity	LCS12 N=1432 (100%)	LCS16 N=1452 (100%)	Total N=2884 (100%)
Nasopharyngitis	mild	65 (4.5%)	61 (4.2%)	126 (4.4%)
	moderate	35 (2.4%)	45 (3.1%)	80 (2.8%)
	severe	3 (0.2%)	3 (0.2%)	6 (0.2%)
Nipple infection	moderate	0	1 (<0.1%)	1 (<0.1%)
Omphalitis	mild	0	1 (<0.1%)	1 (<0.1%)
Onychomycosis	mild	1 (<0.1%)	3 (0.2%)	4 (0.1%)
	moderate	2 (0.1%)	1 (<0.1%)	3 (0.1%)
Oophoritis	mild	0	1 (<0.1%)	1 (<0.1%)
Oral candidiasis	mild	0	1 (<0.1%)	1 (<0.1%)
Oral herpes	mild	6 (0.4%)	7 (0.5%)	13 (0.5%)
	moderate	5 (0.3%)	3 (0.2%)	8 (0.3%)
Osteomyelitis	severe	0	1 (<0.1%)	1 (<0.1%)
Otitis externa	mild	0	1 (<0.1%)	1 (<0.1%)
	moderate	0	2 (0.1%)	2 (<0.1%)
Otitis media	mild	0	3 (0.2%)	3 (0.1%)
	moderate	5 (0.3%)	3 (0.2%)	8 (0.3%)
Otitis media acute	moderate	0	1 (<0.1%)	1 (<0.1%)
Papilloma viral infection	mild	2 (0.1%)	1 (<0.1%)	3 (0.1%)
Parasitic gastroenteritis	mild	1 (<0.1%)	0	1 (<0.1%)
Paronychia	mild	0	2 (0.1%)	2 (<0.1%)
	moderate	1 (<0.1%)	0	1 (<0.1%)
Pelvic infection	mild	0	1 (<0.1%)	1 (<0.1%)

Table 14.3.1 / 16: Number of subjects with adverse events by maximum intensity, primary system organ class and preferred term (FAS)

Primary system organ class<br>Preferred term<br>MedDRA version 14.0	Maximum AE<br>maximum<br>intensity	LCS12<br>N=1432 (100%)	LCS16<br>N=1452 (100%)	Total<br>N=2884 (100%)
Pelvic inflammatory disease	mild	0	1 (<0.1%)	1 (<0.1%)
	moderate	3 (0.2%)	2 (0.1%)	5 (0.2%)
	severe	2 (0.1%)	2 (0.1%)	4 (0.1%)
Peritoneal abscess	moderate	1 (<0.1%)	0	1 (<0.1%)
Peritonsillar abscess	moderate	2 (0.1%)	0	2 (<0.1%)
	severe	0	1 (<0.1%)	1 (<0.1%)
Pharyngeal abscess	moderate	1 (<0.1%)	0	1 (<0.1%)
Pharyngitis	mild	4 (0.3%)	9 (0.6%)	13 (0.5%)
	moderate	9 (0.6%)	12 (0.8%)	21 (0.7%)
Pharyngitis streptococcal	mild	6 (0.4%)	10 (0.7%)	16 (0.6%)
	moderate	15 (1.0%)	9 (0.6%)	24 (0.8%)
	severe	2 (0.1%)	2 (0.1%)	4 (0.1%)
Pharyngotonsillitis	mild	1 (<0.1%)	0	1 (<0.1%)
Pilonidal cyst	moderate	1 (<0.1%)	0	1 (<0.1%)
Pneumonia	mild	1 (<0.1%)	1 (<0.1%)	2 (<0.1%)
	moderate	11 (0.8%)	11 (0.8%)	22 (0.8%)
	severe	2 (0.1%)	3 (0.2%)	5 (0.2%)
Pneumonia mycoplasmal	moderate	1 (<0.1%)	0	1 (<0.1%)
Pogosta disease	mild	1 (<0.1%)	0	1 (<0.1%)
Post procedural infection	moderate	2 (0.1%)	1 (<0.1%)	3 (0.1%)
	severe	1 (<0.1%)	0	1 (<0.1%)
Pulpitis dental	severe	0	1 (<0.1%)	1 (<0.1%)
Pyelonephritis	mild	1 (<0.1%)	0	1 (<0.1%)
	moderate	4 (0.3%)	1 (<0.1%)	5 (0.2%)
	severe	4 (0.3%)	2 (0.1%)	6 (0.2%)

Table 14.3.1 / 16: Number of subjects with adverse events by maximum intensity, primary system organ class and preferred term (FAS)

Primary system organ class<br>Preferred term<br>MedDRA version 14.0	Maximum AE<br>maximum<br>intensity	LCS12<br>N=1432 (100%)	LCS16<br>N=1452 (100%)	Total<br>N=2884 (100%)
Pyelonephritis acute	moderate	1 (<0.1%)	0	1 (<0.1%)
	severe	1 (<0.1%)	0	1 (<0.1%)
Q fever	severe	1 (<0.1%)	0	1 (<0.1%)
Rash pustular	mild	0	1 (<0.1%)	1 (<0.1%)
Respiratory tract infection	mild	4 (0.3%)	4 (0.3%)	8 (0.3%)
	moderate	6 (0.4%)	11 (0.8%)	17 (0.6%)
	severe	0	1 (<0.1%)	1 (<0.1%)
Respiratory tract infection viral	moderate	2 (0.1%)	1 (<0.1%)	3 (0.1%)
Rhinitis	mild	5 (0.3%)	8 (0.6%)	13 (0.5%)
	moderate	1 (<0.1%)	4 (0.3%)	5 (0.2%)
Salpingitis	mild	1 (<0.1%)	0	1 (<0.1%)
Salpingo-oophoritis	mild	1 (<0.1%)	3 (0.2%)	4 (0.1%)
	moderate	1 (<0.1%)	0	1 (<0.1%)
Sialoadenitis	moderate	1 (<0.1%)	0	1 (<0.1%)
Sinobronchitis	moderate	0	1 (<0.1%)	1 (<0.1%)
Sinusitis	mild	26 (1.8%)	29 (2.0%)	55 (1.9%)
	moderate	62 (4.3%)	62 (4.3%)	124 (4.3%)
	severe	2 (0.1%)	5 (0.3%)	7 (0.2%)
Skin bacterial infection	mild	0	1 (<0.1%)	1 (<0.1%)
	moderate	1 (<0.1%)	1 (<0.1%)	2 (<0.1%)
Skin candida	mild	1 (<0.1%)	0	1 (<0.1%)
Skin infection	mild	1 (<0.1%)	3 (0.2%)	4 (0.1%)
	moderate	0	3 (0.2%)	3 (0.1%)
Small intestinal bacterial overgrowth	moderate	0	1 (<0.1%)	1 (<0.1%)

Table 14.3.1 / 16: Number of subjects with adverse events by maximum intensity, primary system organ class and preferred term (FAS)

Primary system organ class<br>Preferred term<br>MedDRA version 14.0	Maximum AE maximum intensity	LCS12<br>N=1432 (100%)	LCS16<br>N=1452 (100%)	Total<br>N=2884 (100%)
Staphylococcal infection	moderate	1 (<0.1%)	1 (<0.1%)	2 (<0.1%)
Staphylococcal skin infection	moderate	1 (<0.1%)	0	1 (<0.1%)
Streptococcal infection	moderate	1 (<0.1%)	0	1 (<0.1%)
Streptococcal sepsis	severe	1 (<0.1%)	0	1 (<0.1%)
Subcutaneous abscess	mild	0	1 (<0.1%)	1 (<0.1%)
	moderate	3 (0.2%)	0	3 (0.1%)
	severe	0	1 (<0.1%)	1 (<0.1%)
Sweat gland infection	moderate	1 (<0.1%)	0	1 (<0.1%)
Tinea infection	mild	1 (<0.1%)	0	1 (<0.1%)
	moderate	0	2 (0.1%)	2 (<0.1%)
Tinea pedis	mild	0	1 (<0.1%)	1 (<0.1%)
	moderate	1 (<0.1%)	0	1 (<0.1%)
Tinea versicolour	mild	0	1 (<0.1%)	1 (<0.1%)
Tonsillitis	mild	8 (0.6%)	4 (0.3%)	12 (0.4%)
	moderate	16 (1.1%)	19 (1.3%)	35 (1.2%)
	severe	1 (<0.1%)	1 (<0.1%)	2 (<0.1%)
Tonsillitis streptococcal	moderate	1 (<0.1%)	0	1 (<0.1%)
Tooth abscess	mild	0	1 (<0.1%)	1 (<0.1%)
	moderate	3 (0.2%)	2 (0.1%)	5 (0.2%)
	severe	2 (0.1%)	1 (<0.1%)	3 (0.1%)
Tooth infection	mild	12 (0.8%)	4 (0.3%)	16 (0.6%)
	moderate	10 (0.7%)	9 (0.6%)	19 (0.7%)
	severe	3 (0.2%)	2 (0.1%)	5 (0.2%)
Tracheitis	moderate	1 (<0.1%)	0	1 (<0.1%)

Table 14.3.1 / 16: Number of subjects with adverse events by maximum intensity, primary system organ class and preferred term (FAS)

Primary system organ class<br>Preferred term<br>MedDRA version 14.0	Maximum AE maximum intensity	LCS12<br>N=1432 (100%)	LCS16<br>N=1452 (100%)	Total<br>N=2884 (100%)
Trichomoniasis	mild	2 (0.1%)	1 (<0.1%)	3 (0.1%)
	moderate	0	1 (<0.1%)	1 (<0.1%)
Tuberculosis	moderate	1 (<0.1%)	0	1 (<0.1%)
Tubo-ovarian abscess	severe	1 (<0.1%)	0	1 (<0.1%)
Typhoid fever	mild	0	1 (<0.1%)	1 (<0.1%)
Upper respiratory tract infection	mild	28 (2.0%)	32 (2.2%)	60 (2.1%)
	moderate	22 (1.5%)	24 (1.7%)	46 (1.6%)
	severe	1 (<0.1%)	1 (<0.1%)	2 (<0.1%)
Ureaplasma infection	mild	1 (<0.1%)	0	1 (<0.1%)
Urinary tract infection	mild	71 (5.0%)	70 (4.8%)	141 (4.9%)
	moderate	79 (5.5%)	73 (5.0%)	152 (5.3%)
	severe	8 (0.6%)	2 (0.1%)	10 (0.3%)
Uterine infection	severe	0	1 (<0.1%)	1 (<0.1%)
Vaginal infection	mild	28 (2.0%)	36 (2.5%)	64 (2.2%)
	moderate	19 (1.3%)	22 (1.5%)	41 (1.4%)
	severe	1 (<0.1%)	2 (0.1%)	3 (0.1%)
Vaginitis bacterial	mild	73 (5.1%)	84 (5.8%)	157 (5.4%)
	moderate	30 (2.1%)	43 (3.0%)	73 (2.5%)
	severe	2 (0.1%)	0	2 (<0.1%)
Vaginitis chlamydial	mild	2 (0.1%)	1 (<0.1%)	3 (0.1%)
Vaginitis gardnerella	mild	0	3 (0.2%)	3 (0.1%)
	moderate	0	1 (<0.1%)	1 (<0.1%)
Vestibular neuronitis	moderate	1 (<0.1%)	0	1 (<0.1%)
	severe	1 (<0.1%)	0	1 (<0.1%)

Table 14.3.1 / 16: Number of subjects with adverse events by maximum intensity, primary system organ class and preferred term (FAS)

Primary system organ class<br>Preferred term<br>MedDRA version 14.0	Maximum AE maximum intensity	LCS12<br>N=1432 (100%)	LCS16<br>N=1452 (100%)	Total<br>N=2884 (100%)
Viral infection	mild	1 (<0.1%)	1 (<0.1%)	2 (<0.1%)
	moderate	2 (0.1%)	1 (<0.1%)	3 (0.1%)
	severe	0	1 (<0.1%)	1 (<0.1%)
Viral pharyngitis	mild	1 (<0.1%)	1 (<0.1%)	2 (<0.1%)
Viral rhinitis	mild	1 (<0.1%)	0	1 (<0.1%)
Viral upper respiratory tract infection	mild	6 (0.4%)	3 (0.2%)	9 (0.3%)
	moderate	9 (0.6%)	11 (0.8%)	20 (0.7%)
Vulval abscess	moderate	0	1 (<0.1%)	1 (<0.1%)
Vulvitis	mild	4 (0.3%)	5 (0.3%)	9 (0.3%)
	moderate	4 (0.3%)	2 (0.1%)	6 (0.2%)
Vulvovaginal candidiasis	mild	39 (2.7%)	48 (3.3%)	87 (3.0%)
	moderate	32 (2.2%)	24 (1.7%)	56 (1.9%)
	severe	1 (<0.1%)	0	1 (<0.1%)
Vulvovaginal mycotic infection	mild	69 (4.8%)	73 (5.0%)	142 (4.9%)
	moderate	28 (2.0%)	36 (2.5%)	64 (2.2%)
	severe	2 (0.1%)	1 (<0.1%)	3 (0.1%)
Vulvovaginitis	mild	3 (0.2%)	3 (0.2%)	6 (0.2%)
	moderate	2 (0.1%)	4 (0.3%)	6 (0.2%)
	severe	1 (<0.1%)	0	1 (<0.1%)
Vulvovaginitis chlamydial	moderate	0	1 (<0.1%)	1 (<0.1%)
Vulvovaginitis streptococcal	mild	0	1 (<0.1%)	1 (<0.1%)
	moderate	1 (<0.1%)	1 (<0.1%)	2 (<0.1%)
Vulvovaginitis trichomonal	mild	2 (0.1%)	3 (0.2%)	5 (0.2%)
	moderate	1 (<0.1%)	1 (<0.1%)	2 (<0.1%)
	severe	1 (<0.1%)	0	1 (<0.1%)

Table 14.3.1 / 16: Number of subjects with adverse events by maximum intensity, primary system organ class and preferred term (FAS)

Primary system organ class<br>Preferred term<br>MedDRA version 14.0	Maximum AE<br>maximum<br>intensity	LCS12<br>N=1432 (100%)	LCS16<br>N=1452 (100%)	Total<br>N=2884 (100%)
Wound infection	mild	2 (0.1%)	0	2 (<0.1%)
	moderate	1 (<0.1%)	1 (<0.1%)	2 (<0.1%)
Wound infection staphylococcal	mild	0	1 (<0.1%)	1 (<0.1%)
Injury, poisoning and procedural complications	mild	38 (2.7%)	35 (2.4%)	73 (2.5%)
	moderate	80 (5.6%)	80 (5.5%)	160 (5.5%)
	severe	31 (2.2%)	18 (1.2%)	49 (1.7%)
Abdominal wound dehiscence	mild	0	1 (<0.1%)	1 (<0.1%)
Accident	severe	0	1 (<0.1%)	1 (<0.1%)
Animal bite	mild	3 (0.2%)	2 (0.1%)	5 (0.2%)
Ankle fracture	mild	0	1 (<0.1%)	1 (<0.1%)
	moderate	0	1 (<0.1%)	1 (<0.1%)
	severe	1 (<0.1%)	0	1 (<0.1%)
Arthropod bite	mild	2 (0.1%)	1 (<0.1%)	3 (0.1%)
	moderate	2 (0.1%)	1 (<0.1%)	3 (0.1%)
	severe	1 (<0.1%)	0	1 (<0.1%)
Arthropod sting	moderate	0	2 (0.1%)	2 (<0.1%)
Back injury	moderate	0	1 (<0.1%)	1 (<0.1%)
Burns second degree	severe	0	1 (<0.1%)	1 (<0.1%)
Burns third degree	severe	1 (<0.1%)	0	1 (<0.1%)
Cartilage injury	mild	0	1 (<0.1%)	1 (<0.1%)
Clavicle fracture	moderate	1 (<0.1%)	0	1 (<0.1%)
Concussion	mild	0	4 (0.3%)	4 (0.1%)
	moderate	2 (0.1%)	2 (0.1%)	4 (0.1%)
	severe	1 (<0.1%)	2 (0.1%)	3 (0.1%)

Table 14.3.1 / 16: Number of subjects with adverse events by maximum intensity, primary system organ class and preferred term (FAS)

Primary system organ class<br>Preferred term<br>MedDRA version 14.0	Maximum AE<br>maximum<br>intensity	LCS12<br>N=1432 (100%)	LCS16<br>N=1452 (100%)	Total<br>N=2884 (100%)
Contusion	mild	2 (0.1%)	3 (0.2%)	5 (0.2%)
	moderate	5 (0.3%)	2 (0.1%)	7 (0.2%)
Epicondylitis	mild	1 (<0.1%)	0	1 (<0.1%)
	moderate	1 (<0.1%)	3 (0.2%)	4 (0.1%)
	severe	0	1 (<0.1%)	1 (<0.1%)
Excoriation	mild	0	1 (<0.1%)	1 (<0.1%)
Eye injury	mild	1 (<0.1%)	0	1 (<0.1%)
	moderate	0	1 (<0.1%)	1 (<0.1%)
Facial bones fracture	mild	1 (<0.1%)	0	1 (<0.1%)
	moderate	1 (<0.1%)	0	1 (<0.1%)
Fall	mild	0	1 (<0.1%)	1 (<0.1%)
Foot fracture	mild	2 (0.1%)	1 (<0.1%)	3 (0.1%)
	moderate	4 (0.3%)	1 (<0.1%)	5 (0.2%)
	severe	3 (0.2%)	1 (<0.1%)	4 (0.1%)
Forearm fracture	severe	1 (<0.1%)	0	1 (<0.1%)
Frostbite	moderate	1 (<0.1%)	0	1 (<0.1%)
Hand fracture	moderate	1 (<0.1%)	3 (0.2%)	4 (0.1%)
Head injury	mild	1 (<0.1%)	0	1 (<0.1%)
	moderate	0	1 (<0.1%)	1 (<0.1%)
Humerus fracture	moderate	0	1 (<0.1%)	1 (<0.1%)
Injury	moderate	0	1 (<0.1%)	1 (<0.1%)
Joint dislocation	mild	2 (0.1%)	1 (<0.1%)	3 (0.1%)
	moderate	3 (0.2%)	0	3 (0.1%)

Table 14.3.1 / 16: Number of subjects with adverse events by maximum intensity, primary system organ class and preferred term (FAS)

Primary system organ class<br>Preferred term<br>MedDRA version 14.0	Maximum AE<br>maximum<br>intensity	LCS12<br>N=1432 (100%)	LCS16<br>N=1452 (100%)	Total<br>N=2884 (100%)
Joint injury	mild	2 (0.1%)	2 (0.1%)	4 (0.1%)
	moderate	3 (0.2%)	4 (0.3%)	7 (0.2%)
	severe	2 (0.1%)	0	2 (<0.1%)
Joint sprain	mild	5 (0.3%)	2 (0.1%)	7 (0.2%)
	moderate	5 (0.3%)	4 (0.3%)	9 (0.3%)
	severe	3 (0.2%)	0	3 (0.1%)
Laceration	mild	1 (<0.1%)	1 (<0.1%)	2 (<0.1%)
	moderate	2 (0.1%)	1 (<0.1%)	3 (0.1%)
Laryngeal injury	moderate	1 (<0.1%)	0	1 (<0.1%)
Ligament rupture	mild	0	1 (<0.1%)	1 (<0.1%)
	moderate	1 (<0.1%)	0	1 (<0.1%)
	severe	2 (0.1%)	0	2 (<0.1%)
Ligament sprain	moderate	0	1 (<0.1%)	1 (<0.1%)
Limb crushing injury	mild	1 (<0.1%)	0	1 (<0.1%)
Limb injury	mild	1 (<0.1%)	2 (0.1%)	3 (0.1%)
	moderate	0	2 (0.1%)	2 (<0.1%)
Lower limb fracture	moderate	1 (<0.1%)	0	1 (<0.1%)
Lumbar vertebral fracture	moderate	0	1 (<0.1%)	1 (<0.1%)
Meniscus lesion	mild	1 (<0.1%)	0	1 (<0.1%)
	moderate	2 (0.1%)	0	2 (<0.1%)
Muscle injury	moderate	4 (0.3%)	1 (<0.1%)	5 (0.2%)
	severe	1 (<0.1%)	1 (<0.1%)	2 (<0.1%)
Muscle rupture	moderate	0	1 (<0.1%)	1 (<0.1%)

Table 14.3.1 / 16: Number of subjects with adverse events by maximum intensity, primary system organ class and preferred term (FAS)

Primary system organ class<br>Preferred term<br>MedDRA version 14.0	Maximum AE maximum intensity	LCS12<br>N=1432 (100%)	LCS16<br>N=1452 (100%)	Total<br>N=2884 (100%)
Muscle strain	mild	3 (0.2%)	3 (0.2%)	6 (0.2%)
	moderate	2 (0.1%)	5 (0.3%)	7 (0.2%)
	severe	2 (0.1%)	2 (0.1%)	4 (0.1%)
Nail avulsion	severe	1 (<0.1%)	0	1 (<0.1%)
Overdose	severe	1 (<0.1%)	0	1 (<0.1%)
Post concussion syndrome	moderate	0	1 (<0.1%)	1 (<0.1%)
Post procedural haemorrhage	mild	1 (<0.1%)	1 (<0.1%)	2 (<0.1%)
	moderate	1 (<0.1%)	1 (<0.1%)	2 (<0.1%)
Post-traumatic pain	mild	0	1 (<0.1%)	1 (<0.1%)
	moderate	3 (0.2%)	1 (<0.1%)	4 (0.1%)
	severe	0	2 (0.1%)	2 (<0.1%)
Postoperative ileus	moderate	0	1 (<0.1%)	1 (<0.1%)
Procedural pain	mild	13 (0.9%)	14 (1.0%)	27 (0.9%)
	moderate	35 (2.4%)	33 (2.3%)	68 (2.4%)
	severe	10 (0.7%)	7 (0.5%)	17 (0.6%)
Procedural vomiting	severe	1 (<0.1%)	0	1 (<0.1%)
Radius fracture	moderate	1 (<0.1%)	1 (<0.1%)	2 (<0.1%)
Rib fracture	moderate	1 (<0.1%)	2 (0.1%)	3 (0.1%)
Road traffic accident	mild	1 (<0.1%)	1 (<0.1%)	2 (<0.1%)
	moderate	2 (0.1%)	1 (<0.1%)	3 (0.1%)
Seroma	mild	1 (<0.1%)	0	1 (<0.1%)
Shunt occlusion	severe	1 (<0.1%)	0	1 (<0.1%)
Skeletal injury	mild	0	1 (<0.1%)	1 (<0.1%)

Table 14.3.1 / 16: Number of subjects with adverse events by maximum intensity, primary system organ class and preferred term (FAS)

Primary system organ class<br>Preferred term<br>MedDRA version 14.0	Maximum AE maximum intensity	LCS12<br>N=1432 (100%)	LCS16<br>N=1452 (100%)	Total<br>N=2884 (100%)
Skin injury	moderate	0	1 (<0.1%)	1 (<0.1%)
Spinal column injury	moderate	0	2 (0.1%)	2 (<0.1%)
Stress fracture	moderate	0	1 (<0.1%)	1 (<0.1%)
Subdural haematoma	severe	1 (<0.1%)	0	1 (<0.1%)
Tendon injury	moderate	0	1 (<0.1%)	1 (<0.1%)
Tendon rupture	mild	1 (<0.1%)	0	1 (<0.1%)
	moderate	2 (0.1%)	0	2 (<0.1%)
	severe	0	1 (<0.1%)	1 (<0.1%)
Thermal burn	mild	0	1 (<0.1%)	1 (<0.1%)
	moderate	0	1 (<0.1%)	1 (<0.1%)
	severe	0	1 (<0.1%)	1 (<0.1%)
Tibia fracture	moderate	1 (<0.1%)	1 (<0.1%)	2 (<0.1%)
Traumatic fracture	mild	1 (<0.1%)	0	1 (<0.1%)
Upper limb fracture	moderate	0	2 (0.1%)	2 (<0.1%)
	severe	1 (<0.1%)	0	1 (<0.1%)
Vulval laceration	mild	0	1 (<0.1%)	1 (<0.1%)
Whiplash injury	mild	0	1 (<0.1%)	1 (<0.1%)
	moderate	1 (<0.1%)	4 (0.3%)	5 (0.2%)
Wound	mild	1 (<0.1%)	0	1 (<0.1%)
Wrist fracture	moderate	0	1 (<0.1%)	1 (<0.1%)
	severe	0	1 (<0.1%)	1 (<0.1%)
Investigations	mild	65 (4.5%)	68 (4.7%)	133 (4.6%)
	moderate	39 (2.7%)	44 (3.0%)	83 (2.9%)
	severe	4 (0.3%)	2 (0.1%)	6 (0.2%)

Table 14.3.1 / 16: Number of subjects with adverse events by maximum intensity, primary system organ class and preferred term (FAS)

Primary system organ class<br>Preferred term<br>MedDRA version 14.0	Maximum AE maximum intensity	LCS12<br>N=1432 (100%)	LCS16<br>N=1452 (100%)	Total<br>N=2884 (100%)
Alanine aminotransferase increased	mild	1 (<0.1%)	1 (<0.1%)	2 (<0.1%)
Antibiotic resistant Staphylococcus test positive	mild	1 (<0.1%)	0	1 (<0.1%)
	moderate	1 (<0.1%)	0	1 (<0.1%)
Biopsy muscle	severe	1 (<0.1%)	0	1 (<0.1%)
Blood cholesterol increased	mild	1 (<0.1%)	0	1 (<0.1%)
	severe	1 (<0.1%)	0	1 (<0.1%)
Blood pressure diastolic increased	mild	0	1 (<0.1%)	1 (<0.1%)
Blood pressure increased	mild	2 (0.1%)	1 (<0.1%)	3 (0.1%)
	moderate	1 (<0.1%)	0	1 (<0.1%)
Blood pressure systolic increased	mild	0	1 (<0.1%)	1 (<0.1%)
Cardiac murmur	mild	1 (<0.1%)	0	1 (<0.1%)
Chlamydia test positive	mild	6 (0.4%)	1 (<0.1%)	7 (0.2%)
	moderate	1 (<0.1%)	0	1 (<0.1%)
Gamma-glutamyltransferase increased	mild	1 (<0.1%)	0	1 (<0.1%)
Gastric pH decreased	mild	0	1 (<0.1%)	1 (<0.1%)
Haemoglobin decreased	mild	2 (0.1%)	1 (<0.1%)	3 (0.1%)
	severe	0	1 (<0.1%)	1 (<0.1%)
Heart rate increased	moderate	0	1 (<0.1%)	1 (<0.1%)
Human papilloma virus test positive	mild	4 (0.3%)	4 (0.3%)	8 (0.3%)
	moderate	1 (<0.1%)	1 (<0.1%)	2 (<0.1%)
Liver function test abnormal	mild	0	1 (<0.1%)	1 (<0.1%)
	moderate	0	1 (<0.1%)	1 (<0.1%)

Table 14.3.1 / 16: Number of subjects with adverse events by maximum intensity, primary system organ class and preferred term (FAS)

Primary system organ class<br>Preferred term<br>MedDRA version 14.0	Maximum AE maximum intensity	LCS12<br>N=1432 (100%)	LCS16<br>N=1452 (100%)	Total<br>N=2884 (100%)
Neisseria test positive	moderate	2 (0.1%)	0	2 (<0.1%)
Simplex virus test positive	mild	1 (<0.1%)	1 (<0.1%)	2 (<0.1%)
	moderate	1 (<0.1%)	0	1 (<0.1%)
Smear cervix abnormal	mild	14 (1.0%)	23 (1.6%)	37 (1.3%)
	moderate	4 (0.3%)	4 (0.3%)	8 (0.3%)
Streptococcus test positive	mild	1 (<0.1%)	0	1 (<0.1%)
Ultrasound ovary abnormal	mild	1 (<0.1%)	0	1 (<0.1%)
Vitamin D decreased	moderate	0	1 (<0.1%)	1 (<0.1%)
Weight decreased	mild	5 (0.3%)	3 (0.2%)	8 (0.3%)
	moderate	4 (0.3%)	4 (0.3%)	8 (0.3%)
Weight increased	mild	27 (1.9%)	34 (2.3%)	61 (2.1%)
	moderate	27 (1.9%)	33 (2.3%)	60 (2.1%)
	severe	2 (0.1%)	1 (<0.1%)	3 (0.1%)
White blood cell count increased	mild	1 (<0.1%)	0	1 (<0.1%)
Metabolism and nutrition disorders	mild	13 (0.9%)	8 (0.6%)	21 (0.7%)
	moderate	5 (0.3%)	9 (0.6%)	14 (0.5%)
Abnormal loss of weight	mild	0	1 (<0.1%)	1 (<0.1%)
Abnormal weight gain	moderate	0	1 (<0.1%)	1 (<0.1%)
Cell death	mild	1 (<0.1%)	0	1 (<0.1%)
Cholesterosis	mild	1 (<0.1%)	0	1 (<0.1%)
Decreased appetite	mild	1 (<0.1%)	1 (<0.1%)	2 (<0.1%)
	moderate	1 (<0.1%)	0	1 (<0.1%)

Table 14.3.1 / 16: Number of subjects with adverse events by maximum intensity, primary system organ class and preferred term (FAS)

Primary system organ class<br>Preferred term<br>MedDRA version 14.0	Maximum AE<br>maximum<br>intensity	LCS12<br>N=1432 (100%)	LCS16<br>N=1452 (100%)	Total<br>N=2884 (100%)
Dehydration	mild	0	1 (<0.1%)	1 (<0.1%)
	moderate	0	1 (<0.1%)	1 (<0.1%)
Diabetic ketoacidosis	moderate	0	1 (<0.1%)	1 (<0.1%)
Dyslipidaemia	mild	0	1 (<0.1%)	1 (<0.1%)
Fluid retention	mild	2 (0.1%)	0	2 (<0.1%)
	moderate	1 (<0.1%)	2 (0.1%)	3 (0.1%)
Glucose tolerance impaired	mild	1 (<0.1%)	0	1 (<0.1%)
Hypokalaemia	moderate	2 (0.1%)	0	2 (<0.1%)
Hyponatraemia	moderate	1 (<0.1%)	0	1 (<0.1%)
Increased appetite	mild	1 (<0.1%)	1 (<0.1%)	2 (<0.1%)
	moderate	0	1 (<0.1%)	1 (<0.1%)
Insulin resistance	mild	2 (0.1%)	1 (<0.1%)	3 (0.1%)
Lactose intolerance	mild	2 (0.1%)	0	2 (<0.1%)
Obesity	mild	0	1 (<0.1%)	1 (<0.1%)
Overweight	moderate	0	1 (<0.1%)	1 (<0.1%)
Type 2 diabetes mellitus	mild	1 (<0.1%)	0	1 (<0.1%)
Vitamin B12 deficiency	mild	1 (<0.1%)	0	1 (<0.1%)
	moderate	0	1 (<0.1%)	1 (<0.1%)
Vitamin D deficiency	mild	0	2 (0.1%)	2 (<0.1%)
	moderate	1 (<0.1%)	0	1 (<0.1%)
Weight fluctuation	moderate	0	1 (<0.1%)	1 (<0.1%)

Table 14.3.1 / 16: Number of subjects with adverse events by maximum intensity, primary system organ class and preferred term (FAS)

Primary system organ class<br>Preferred term<br>MedDRA version 14.0	Maximum AE maximum intensity	LCS12<br>N=1432 (100%)	LCS16<br>N=1452 (100%)	Total<br>N=2884 (100%)
Musculoskeletal and connective tissue disorders	mild	52 (3.6%)	65 (4.5%)	117 (4.1%)
	moderate	76 (5.3%)	108 (7.4%)	184 (6.4%)
	severe	16 (1.1%)	14 (1.0%)	30 (1.0%)
Ankylosing spondylitis	moderate	1 (<0.1%)	0	1 (<0.1%)
Arthralgia	mild	10 (0.7%)	12 (0.8%)	22 (0.8%)
	moderate	10 (0.7%)	18 (1.2%)	28 (1.0%)
	severe	1 (<0.1%)	1 (<0.1%)	2 (<0.1%)
Arthritis	mild	0	2 (0.1%)	2 (<0.1%)
	moderate	0	2 (0.1%)	2 (<0.1%)
	severe	1 (<0.1%)	1 (<0.1%)	2 (<0.1%)
Axillary mass	moderate	0	1 (<0.1%)	1 (<0.1%)
Back pain	mild	19 (1.3%)	21 (1.4%)	40 (1.4%)
	moderate	35 (2.4%)	38 (2.6%)	73 (2.5%)
	severe	5 (0.3%)	6 (0.4%)	11 (0.4%)
Bone pain	mild	1 (<0.1%)	1 (<0.1%)	2 (<0.1%)
	moderate	0	1 (<0.1%)	1 (<0.1%)
	severe	0	1 (<0.1%)	1 (<0.1%)
Bursitis	mild	1 (<0.1%)	1 (<0.1%)	2 (<0.1%)
	moderate	1 (<0.1%)	0	1 (<0.1%)
Compartment syndrome	moderate	0	1 (<0.1%)	1 (<0.1%)
Costochondritis	mild	1 (<0.1%)	0	1 (<0.1%)
	moderate	1 (<0.1%)	1 (<0.1%)	2 (<0.1%)
	severe	1 (<0.1%)	0	1 (<0.1%)
Exostosis	mild	1 (<0.1%)	1 (<0.1%)	2 (<0.1%)
	moderate	0	2 (0.1%)	2 (<0.1%)
Fibromyalgia	mild	1 (<0.1%)	2 (0.1%)	3 (0.1%)

Table 14.3.1 / 16: Number of subjects with adverse events by maximum intensity, primary system organ class and preferred term (FAS)

Primary system organ class<br>Preferred term<br>MedDRA version 14.0	Maximum AE<br>maximum<br>intensity	LCS12<br>N=1432 (100%)	LCS16<br>N=1452 (100%)	Total<br>N=2884 (100%)
Flank pain	moderate	2 (0.1%)	3 (0.2%)	5 (0.2%)
Groin pain	mild	2 (0.1%)	3 (0.2%)	5 (0.2%)
	severe	0	1 (<0.1%)	1 (<0.1%)
Hand deformity	mild	0	1 (<0.1%)	1 (<0.1%)
Intervertebral disc disorder	moderate	0	1 (<0.1%)	1 (<0.1%)
	severe	1 (<0.1%)	0	1 (<0.1%)
Intervertebral disc protrusion	moderate	1 (<0.1%)	2 (0.1%)	3 (0.1%)
	severe	0	1 (<0.1%)	1 (<0.1%)
Joint effusion	severe	1 (<0.1%)	0	1 (<0.1%)
Joint instability	moderate	1 (<0.1%)	1 (<0.1%)	2 (<0.1%)
Joint stiffness	moderate	0	1 (<0.1%)	1 (<0.1%)
Joint swelling	moderate	1 (<0.1%)	1 (<0.1%)	2 (<0.1%)
Loose body in joint	moderate	0	1 (<0.1%)	1 (<0.1%)
Medial tibial stress syndrome	mild	1 (<0.1%)	1 (<0.1%)	2 (<0.1%)
	moderate	1 (<0.1%)	1 (<0.1%)	2 (<0.1%)
Muscle contracture	mild	0	1 (<0.1%)	1 (<0.1%)
	moderate	0	1 (<0.1%)	1 (<0.1%)
	severe	1 (<0.1%)	0	1 (<0.1%)
Muscle spasms	mild	3 (0.2%)	3 (0.2%)	6 (0.2%)
	moderate	2 (0.1%)	5 (0.3%)	7 (0.2%)
Muscle tightness	mild	3 (0.2%)	2 (0.1%)	5 (0.2%)
	moderate	5 (0.3%)	8 (0.6%)	13 (0.5%)
Musculoskeletal chest pain	moderate	2 (0.1%)	2 (0.1%)	4 (0.1%)

Table 14.3.1 / 16: Number of subjects with adverse events by maximum intensity, primary system organ class and preferred term (FAS)

Primary system organ class<br>Preferred term<br>MedDRA version 14.0	Maximum AE<br>maximum<br>intensity	LCS12<br>N=1432 (100%)	LCS16<br>N=1452 (100%)	Total<br>N=2884 (100%)
Musculoskeletal discomfort	mild	0	1 (<0.1%)	1 (<0.1%)
Musculoskeletal pain	mild	5 (0.3%)	2 (0.1%)	7 (0.2%)
	moderate	3 (0.2%)	8 (0.6%)	11 (0.4%)
	severe	1 (<0.1%)	1 (<0.1%)	2 (<0.1%)
Musculoskeletal stiffness	moderate	0	1 (<0.1%)	1 (<0.1%)
Myalgia	mild	4 (0.3%)	5 (0.3%)	9 (0.3%)
	moderate	5 (0.3%)	6 (0.4%)	11 (0.4%)
	severe	0	1 (<0.1%)	1 (<0.1%)
Myositis	moderate	0	1 (<0.1%)	1 (<0.1%)
Neck pain	mild	5 (0.3%)	3 (0.2%)	8 (0.3%)
	moderate	7 (0.5%)	6 (0.4%)	13 (0.5%)
	severe	0	1 (<0.1%)	1 (<0.1%)
Osteitis	mild	1 (<0.1%)	0	1 (<0.1%)
	severe	1 (<0.1%)	0	1 (<0.1%)
Osteoarthritis	moderate	0	2 (0.1%)	2 (<0.1%)
Osteochondrosis	mild	0	1 (<0.1%)	1 (<0.1%)
	severe	0	1 (<0.1%)	1 (<0.1%)
Osteopenia	mild	0	1 (<0.1%)	1 (<0.1%)
Pain in extremity	mild	2 (0.1%)	10 (0.7%)	12 (0.4%)
	moderate	4 (0.3%)	5 (0.3%)	9 (0.3%)
Pain in jaw	moderate	0	1 (<0.1%)	1 (<0.1%)
Patellofemoral pain syndrome	moderate	1 (<0.1%)	0	1 (<0.1%)
Plantar fasciitis	moderate	0	1 (<0.1%)	1 (<0.1%)
Psoriatic arthropathy	severe	1 (<0.1%)	0	1 (<0.1%)

Table 14.3.1 / 16: Number of subjects with adverse events by maximum intensity, primary system organ class and preferred term (FAS)

Primary system organ class<br>Preferred term<br>MedDRA version 14.0	Maximum AE maximum intensity	LCS12<br>N=1432 (100%)	LCS16<br>N=1452 (100%)	Total<br>N=2884 (100%)
Rotator cuff syndrome	mild	1 (<0.1%)	2 (0.1%)	3 (0.1%)
	moderate	0	4 (0.3%)	4 (0.1%)
Scleroderma	mild	1 (<0.1%)	0	1 (<0.1%)
Sensation of heaviness	moderate	0	1 (<0.1%)	1 (<0.1%)
Spinal osteoarthritis	mild	1 (<0.1%)	0	1 (<0.1%)
Synovitis	severe	1 (<0.1%)	0	1 (<0.1%)
Temporomandibular joint syndrome	mild	1 (<0.1%)	1 (<0.1%)	2 (<0.1%)
Tendon pain	mild	0	1 (<0.1%)	1 (<0.1%)
	moderate	1 (<0.1%)	0	1 (<0.1%)
Tendonitis	mild	3 (0.2%)	1 (<0.1%)	4 (0.1%)
	moderate	1 (<0.1%)	4 (0.3%)	5 (0.2%)
	severe	1 (<0.1%)	0	1 (<0.1%)
Tenosynovitis	moderate	0	1 (<0.1%)	1 (<0.1%)
Torticollis	mild	0	2 (0.1%)	2 (<0.1%)
	moderate	1 (<0.1%)	0	1 (<0.1%)
Neoplasms benign, malignant and unspecified (incl cysts and polyps)	mild	33 (2.3%)	29 (2.0%)	62 (2.1%)
	moderate	8 (0.6%)	11 (0.8%)	19 (0.7%)
	severe	4 (0.3%)	3 (0.2%)	7 (0.2%)
Acoustic neuroma	moderate	0	1 (<0.1%)	1 (<0.1%)
Acrochordon	mild	1 (<0.1%)	3 (0.2%)	4 (0.1%)
Acute leukaemia	severe	1 (<0.1%)	0	1 (<0.1%)
Adenoma benign	mild	0	1 (<0.1%)	1 (<0.1%)

Table 14.3.1 / 16: Number of subjects with adverse events by maximum intensity, primary system organ class and preferred term (FAS)

Primary system organ class<br>Preferred term<br>MedDRA version 14.0	Maximum AE maximum intensity	LCS12<br>N=1432 (100%)	LCS16<br>N=1452 (100%)	Total<br>N=2884 (100%)
Astrocytoma, low grade	severe	1 (<0.1%)	0	1 (<0.1%)
Benign breast neoplasm	mild	1 (<0.1%)	1 (<0.1%)	2 (<0.1%)
	moderate	1 (<0.1%)	0	1 (<0.1%)
Cervicitis human papilloma virus	mild	1 (<0.1%)	4 (0.3%)	5 (0.2%)
Cervix carcinoma stage 0	severe	0	1 (<0.1%)	1 (<0.1%)
Cervix neoplasm	moderate	1 (<0.1%)	0	1 (<0.1%)
Enchondroma	moderate	0	1 (<0.1%)	1 (<0.1%)
Fibroadenoma of breast	mild	6 (0.4%)	1 (<0.1%)	7 (0.2%)
	moderate	1 (<0.1%)	1 (<0.1%)	2 (<0.1%)
Glomus tumour	moderate	1 (<0.1%)	0	1 (<0.1%)
Haemangioma	mild	1 (<0.1%)	0	1 (<0.1%)
Haemangioma of liver	moderate	0	1 (<0.1%)	1 (<0.1%)
Leiomyoma	mild	0	1 (<0.1%)	1 (<0.1%)
Lipoma of breast	mild	2 (0.1%)	0	2 (<0.1%)
Malignant melanoma	severe	0	1 (<0.1%)	1 (<0.1%)
Melanocytic naevus	mild	6 (0.4%)	3 (0.2%)	9 (0.3%)
	moderate	0	1 (<0.1%)	1 (<0.1%)
Neurilemmoma	moderate	0	1 (<0.1%)	1 (<0.1%)
Ovarian germ cell teratoma benign	mild	3 (0.2%)	1 (<0.1%)	4 (0.1%)
	moderate	2 (0.1%)	2 (0.1%)	4 (0.1%)
Ovarian neoplasm	mild	0	2 (0.1%)	2 (<0.1%)

Table 14.3.1 / 16: Number of subjects with adverse events by maximum intensity, primary system organ class and preferred term (FAS)

Primary system organ class<br>Preferred term<br>MedDRA version 14.0	Maximum AE maximum intensity	LCS12<br>N=1432 (100%)	LCS16<br>N=1452 (100%)	Total<br>N=2884 (100%)
Pancreatic carcinoma	severe	0	1 (<0.1%)	1 (<0.1%)
Skin papilloma	mild	0	2 (0.1%)	2 (<0.1%)
	moderate	1 (<0.1%)	0	1 (<0.1%)
Teratoma benign	mild	0	1 (<0.1%)	1 (<0.1%)
Thyroid cancer	severe	2 (0.1%)	0	2 (<0.1%)
Thyroid neoplasm	mild	0	2 (0.1%)	2 (<0.1%)
Uterine leiomyoma	mild	7 (0.5%)	4 (0.3%)	11 (0.4%)
	moderate	1 (<0.1%)	2 (0.1%)	3 (0.1%)
Vulvovaginal human papilloma virus infection	mild	5 (0.3%)	5 (0.3%)	10 (0.3%)
	moderate	0	1 (<0.1%)	1 (<0.1%)
Nervous system disorders	mild	83 (5.8%)	80 (5.5%)	163 (5.7%)
	moderate	85 (5.9%)	108 (7.4%)	193 (6.7%)
	severe	32 (2.2%)	32 (2.2%)	64 (2.2%)
Amnesia	mild	0	1 (<0.1%)	1 (<0.1%)
	severe	1 (<0.1%)	0	1 (<0.1%)
Aphonia	moderate	0	1 (<0.1%)	1 (<0.1%)
Burning sensation	moderate	1 (<0.1%)	0	1 (<0.1%)
Carpal tunnel syndrome	moderate	1 (<0.1%)	6 (0.4%)	7 (0.2%)
Cervicobrachial syndrome	mild	0	1 (<0.1%)	1 (<0.1%)
Cervicogenic headache	moderate	1 (<0.1%)	0	1 (<0.1%)
Cluster headache	moderate	0	1 (<0.1%)	1 (<0.1%)

Table 14.3.1 / 16: Number of subjects with adverse events by maximum intensity, primary system organ class and preferred term (FAS)

Primary system organ class<br>Preferred term<br>MedDRA version 14.0	Maximum AE<br>maximum<br>intensity	LCS12<br>N=1432 (100%)	LCS16<br>N=1452 (100%)	Total<br>N=2884 (100%)
Convulsion	mild	1 (<0.1%)	1 (<0.1%)	2 (<0.1%)
	moderate	0	1 (<0.1%)	1 (<0.1%)
Disturbance in attention	mild	0	1 (<0.1%)	1 (<0.1%)
	moderate	0	1 (<0.1%)	1 (<0.1%)
Dizziness	mild	5 (0.3%)	9 (0.6%)	14 (0.5%)
	moderate	3 (0.2%)	11 (0.8%)	14 (0.5%)
	severe	1 (<0.1%)	1 (<0.1%)	2 (<0.1%)
Dizziness postural	mild	1 (<0.1%)	0	1 (<0.1%)
Facial spasm	severe	1 (<0.1%)	0	1 (<0.1%)
Headache	mild	65 (4.5%)	56 (3.9%)	121 (4.2%)
	moderate	50 (3.5%)	59 (4.1%)	109 (3.8%)
	severe	18 (1.3%)	22 (1.5%)	40 (1.4%)
Hypoaesthesia	mild	1 (<0.1%)	2 (0.1%)	3 (0.1%)
	moderate	1 (<0.1%)	2 (0.1%)	3 (0.1%)
Loss of consciousness	mild	0	1 (<0.1%)	1 (<0.1%)
Migraine	mild	10 (0.7%)	10 (0.7%)	20 (0.7%)
	moderate	18 (1.3%)	19 (1.3%)	37 (1.3%)
	severe	6 (0.4%)	9 (0.6%)	15 (0.5%)
Migraine with aura	mild	1 (<0.1%)	0	1 (<0.1%)
	moderate	3 (0.2%)	2 (0.1%)	5 (0.2%)
Migraine without aura	mild	1 (<0.1%)	0	1 (<0.1%)
Morton's neuralgia	mild	1 (<0.1%)	0	1 (<0.1%)
Multiple sclerosis	mild	1 (<0.1%)	0	1 (<0.1%)
Muscle contractions involuntary	mild	0	1 (<0.1%)	1 (<0.1%)

Table 14.3.1 / 16: Number of subjects with adverse events by maximum intensity, primary system organ class and preferred term (FAS)

Primary system organ class<br>Preferred term<br>MedDRA version 14.0	Maximum AE maximum intensity	LCS12<br>N=1432 (100%)	LCS16<br>N=1452 (100%)	Total<br>N=2884 (100%)
Nerve compression	moderate	2 (0.1%)	0	2 (<0.1%)
Nervous system disorder	moderate	0	1 (<0.1%)	1 (<0.1%)
Paraesthesia	mild	0	2 (0.1%)	2 (<0.1%)
	moderate	0	2 (0.1%)	2 (<0.1%)
	severe	0	1 (<0.1%)	1 (<0.1%)
Presyncope	mild	0	5 (0.3%)	5 (0.2%)
	moderate	3 (0.2%)	4 (0.3%)	7 (0.2%)
	severe	1 (<0.1%)	0	1 (<0.1%)
Restless legs syndrome	mild	1 (<0.1%)	0	1 (<0.1%)
Sciatica	mild	2 (0.1%)	1 (<0.1%)	3 (0.1%)
	moderate	1 (<0.1%)	2 (0.1%)	3 (0.1%)
	severe	1 (<0.1%)	0	1 (<0.1%)
Sinus headache	mild	1 (<0.1%)	1 (<0.1%)	2 (<0.1%)
	moderate	0	1 (<0.1%)	1 (<0.1%)
Somnolence	mild	1 (<0.1%)	1 (<0.1%)	2 (<0.1%)
	moderate	1 (<0.1%)	0	1 (<0.1%)
Spinal cord herniation	moderate	0	1 (<0.1%)	1 (<0.1%)
Status migrainosus	severe	1 (<0.1%)	0	1 (<0.1%)
Syncope	mild	3 (0.2%)	3 (0.2%)	6 (0.2%)
	moderate	2 (0.1%)	2 (0.1%)	4 (0.1%)
	severe	1 (<0.1%)	0	1 (<0.1%)
Tension headache	mild	4 (0.3%)	3 (0.2%)	7 (0.2%)
	moderate	7 (0.5%)	6 (0.4%)	13 (0.5%)
	severe	1 (<0.1%)	0	1 (<0.1%)
Thoracic outlet syndrome	moderate	1 (<0.1%)	0	1 (<0.1%)

Table 14.3.1 / 16: Number of subjects with adverse events by maximum intensity, primary system organ class and preferred term (FAS)

Primary system organ class<br>Preferred term<br>MedDRA version 14.0	Maximum AE maximum intensity	LCS12<br>N=1432 (100%)	LCS16<br>N=1452 (100%)	Total<br>N=2884 (100%)
VIIth nerve paralysis	mild	1 (<0.1%)	0	1 (<0.1%)
	severe	1 (<0.1%)	0	1 (<0.1%)
Pregnancy, puerperium and perinatal conditions	mild	3 (0.2%)	1 (<0.1%)	4 (0.1%)
	moderate	1 (<0.1%)	4 (0.3%)	5 (0.2%)
	severe	2 (0.1%)	5 (0.3%)	7 (0.2%)
Abortion spontaneous	mild	1 (<0.1%)	0	1 (<0.1%)
	moderate	1 (<0.1%)	0	1 (<0.1%)
	severe	1 (<0.1%)	0	1 (<0.1%)
Abortion spontaneous incomplete	moderate	0	1 (<0.1%)	1 (<0.1%)
Blighted ovum	severe	0	1 (<0.1%)	1 (<0.1%)
Ectopic pregnancy	mild	2 (0.1%)	1 (<0.1%)	3 (0.1%)
	moderate	0	2 (0.1%)	2 (<0.1%)
	severe	1 (<0.1%)	3 (0.2%)	4 (0.1%)
Premature separation of placenta	moderate	1 (<0.1%)	0	1 (<0.1%)
Ruptured ectopic pregnancy	severe	0	1 (<0.1%)	1 (<0.1%)
Uterine contractions abnormal	moderate	0	1 (<0.1%)	1 (<0.1%)
Psychiatric disorders	mild	76 (5.3%)	85 (5.9%)	161 (5.6%)
	moderate	84 (5.9%)	72 (5.0%)	156 (5.4%)
	severe	13 (0.9%)	15 (1.0%)	28 (1.0%)
Acute stress disorder	moderate	0	1 (<0.1%)	1 (<0.1%)
Adjustment disorder	mild	1 (<0.1%)	0	1 (<0.1%)
Affect lability	mild	2 (0.1%)	1 (<0.1%)	3 (0.1%)
	moderate	2 (0.1%)	0	2 (<0.1%)
Affective disorder	severe	0	1 (<0.1%)	1 (<0.1%)

Table 14.3.1 / 16: Number of subjects with adverse events by maximum intensity, primary system organ class and preferred term (FAS)

Primary system organ class<br>Preferred term<br>MedDRA version 14.0	Maximum AE<br>maximum<br>intensity	LCS12<br>N=1432 (100%)	LCS16<br>N=1452 (100%)	Total<br>N=2884 (100%)
Aggression	moderate	1 (<0.1%)	0	1 (<0.1%)
Alcohol abuse	mild	0	1 (<0.1%)	1 (<0.1%)
Alcoholism	severe	0	1 (<0.1%)	1 (<0.1%)
Anxiety	mild	16 (1.1%)	13 (0.9%)	29 (1.0%)
	moderate	17 (1.2%)	21 (1.4%)	38 (1.3%)
	severe	4 (0.3%)	3 (0.2%)	7 (0.2%)
Anxiety disorder	mild	1 (<0.1%)	3 (0.2%)	4 (0.1%)
	moderate	0	1 (<0.1%)	1 (<0.1%)
Attention deficit/hyperactivity disorder	mild	0	3 (0.2%)	3 (0.1%)
	moderate	4 (0.3%)	1 (<0.1%)	5 (0.2%)
Bipolar I disorder	moderate	1 (<0.1%)	0	1 (<0.1%)
Bipolar disorder	mild	0	4 (0.3%)	4 (0.1%)
	moderate	1 (<0.1%)	1 (<0.1%)	2 (<0.1%)
	severe	0	1 (<0.1%)	1 (<0.1%)
Bulimia nervosa	mild	0	1 (<0.1%)	1 (<0.1%)
Burnout syndrome	mild	0	1 (<0.1%)	1 (<0.1%)
	moderate	1 (<0.1%)	1 (<0.1%)	2 (<0.1%)
Completed suicide	severe	0	1 (<0.1%)	1 (<0.1%)
Daydreaming	moderate	1 (<0.1%)	0	1 (<0.1%)
Depressed mood	mild	2 (0.1%)	1 (<0.1%)	3 (0.1%)
	moderate	3 (0.2%)	2 (0.1%)	5 (0.2%)
Depression	mild	25 (1.7%)	27 (1.9%)	52 (1.8%)
	moderate	22 (1.5%)	18 (1.2%)	40 (1.4%)
	severe	4 (0.3%)	4 (0.3%)	8 (0.3%)

Table 14.3.1 / 16: Number of subjects with adverse events by maximum intensity, primary system organ class and preferred term (FAS)

Primary system organ class<br>Preferred term<br>MedDRA version 14.0	Maximum AE<br>maximum<br>intensity	LCS12<br>N=1432 (100%)	LCS16<br>N=1452 (100%)	Total<br>N=2884 (100%)
Depression suicidal	severe	1 (<0.1%)	0	1 (<0.1%)
Depressive symptom	mild	1 (<0.1%)	0	1 (<0.1%)
Disorientation	moderate	0	1 (<0.1%)	1 (<0.1%)
Drug dependence	moderate	1 (<0.1%)	0	1 (<0.1%)
	severe	0	1 (<0.1%)	1 (<0.1%)
Fear	mild	1 (<0.1%)	0	1 (<0.1%)
	moderate	0	1 (<0.1%)	1 (<0.1%)
Generalised anxiety disorder	mild	2 (0.1%)	0	2 (<0.1%)
	moderate	1 (<0.1%)	0	1 (<0.1%)
Hallucination, auditory	moderate	1 (<0.1%)	0	1 (<0.1%)
Insomnia	mild	14 (1.0%)	14 (1.0%)	28 (1.0%)
	moderate	6 (0.4%)	13 (0.9%)	19 (0.7%)
	severe	2 (0.1%)	2 (0.1%)	4 (0.1%)
Libido decreased	mild	16 (1.1%)	16 (1.1%)	32 (1.1%)
	moderate	9 (0.6%)	9 (0.6%)	18 (0.6%)
	severe	2 (0.1%)	0	2 (<0.1%)
Libido increased	mild	0	2 (0.1%)	2 (<0.1%)
	moderate	2 (0.1%)	0	2 (<0.1%)
Loss of libido	mild	0	1 (<0.1%)	1 (<0.1%)
	moderate	4 (0.3%)	0	4 (0.1%)
	severe	0	1 (<0.1%)	1 (<0.1%)
Mental status changes	mild	0	1 (<0.1%)	1 (<0.1%)
Mood altered	mild	5 (0.3%)	7 (0.5%)	12 (0.4%)
	moderate	9 (0.6%)	3 (0.2%)	12 (0.4%)
	severe	1 (<0.1%)	0	1 (<0.1%)

Table 14.3.1 / 16: Number of subjects with adverse events by maximum intensity, primary system organ class and preferred term (FAS)

Primary system organ class<br>Preferred term<br>MedDRA version 14.0	Maximum AE maximum intensity	LCS12<br>N=1432 (100%)	LCS16<br>N=1452 (100%)	Total<br>N=2884 (100%)
Mood swings	mild	6 (0.4%)	3 (0.2%)	9 (0.3%)
	moderate	5 (0.3%)	4 (0.3%)	9 (0.3%)
	severe	1 (<0.1%)	2 (0.1%)	3 (0.1%)
Nervousness	mild	0	1 (<0.1%)	1 (<0.1%)
	moderate	0	1 (<0.1%)	1 (<0.1%)
Nicotine dependence	moderate	1 (<0.1%)	0	1 (<0.1%)
Obsessive-compulsive personality disorder	moderate	0	1 (<0.1%)	1 (<0.1%)
Panic attack	mild	1 (<0.1%)	1 (<0.1%)	2 (<0.1%)
Panic disorder	mild	0	2 (0.1%)	2 (<0.1%)
	moderate	1 (<0.1%)	1 (<0.1%)	2 (<0.1%)
	severe	0	2 (0.1%)	2 (<0.1%)
Personality disorder	mild	1 (<0.1%)	0	1 (<0.1%)
Phobia of flying	mild	0	1 (<0.1%)	1 (<0.1%)
Polysubstance dependence	severe	0	1 (<0.1%)	1 (<0.1%)
Post-traumatic stress disorder	mild	0	1 (<0.1%)	1 (<0.1%)
	moderate	1 (<0.1%)	0	1 (<0.1%)
Psychotic disorder	severe	0	1 (<0.1%)	1 (<0.1%)
Sleep disorder	mild	3 (0.2%)	1 (<0.1%)	4 (0.1%)
	moderate	2 (0.1%)	1 (<0.1%)	3 (0.1%)
Social phobia	moderate	0	1 (<0.1%)	1 (<0.1%)
Stress	mild	0	2 (0.1%)	2 (<0.1%)
	moderate	2 (0.1%)	2 (0.1%)	4 (0.1%)
	severe	1 (<0.1%)	0	1 (<0.1%)
Suicide attempt	severe	1 (<0.1%)	0	1 (<0.1%)

Table 14.3.1 / 16: Number of subjects with adverse events by maximum intensity, primary system organ class and preferred term (FAS)

Primary system organ class<br>Preferred term<br>MedDRA version 14.0	Maximum AE<br>maximum<br>intensity	LCS12<br>N=1432 (100%)	LCS16<br>N=1452 (100%)	Total<br>N=2884 (100%)
Renal and urinary disorders	mild	18 (1.3%)	21 (1.4%)	39 (1.4%)
	moderate	14 (1.0%)	7 (0.5%)	21 (0.7%)
	severe	8 (0.6%)	3 (0.2%)	11 (0.4%)
Calculus bladder	severe	1 (<0.1%)	0	1 (<0.1%)
Calculus urinary	mild	0	1 (<0.1%)	1 (<0.1%)
Chromaturia	mild	0	1 (<0.1%)	1 (<0.1%)
Cystitis haemorrhagic	severe	1 (<0.1%)	0	1 (<0.1%)
Dysuria	mild	8 (0.6%)	6 (0.4%)	14 (0.5%)
	moderate	4 (0.3%)	1 (<0.1%)	5 (0.2%)
	severe	2 (0.1%)	0	2 (<0.1%)
Haematuria	mild	0	2 (0.1%)	2 (<0.1%)
	severe	1 (<0.1%)	0	1 (<0.1%)
Hypertonic bladder	mild	0	2 (0.1%)	2 (<0.1%)
Micturition urgency	mild	2 (0.1%)	3 (0.2%)	5 (0.2%)
Nephrolithiasis	mild	2 (0.1%)	1 (<0.1%)	3 (0.1%)
	moderate	4 (0.3%)	1 (<0.1%)	5 (0.2%)
	severe	2 (0.1%)	1 (<0.1%)	3 (0.1%)
Pollakiuria	mild	2 (0.1%)	4 (0.3%)	6 (0.2%)
	moderate	2 (0.1%)	4 (0.3%)	6 (0.2%)
Polyuria	mild	1 (<0.1%)	1 (<0.1%)	2 (<0.1%)
Renal cyst	mild	0	1 (<0.1%)	1 (<0.1%)
Renal pain	moderate	1 (<0.1%)	0	1 (<0.1%)
Strangury	moderate	1 (<0.1%)	0	1 (<0.1%)

Table 14.3.1 / 16: Number of subjects with adverse events by maximum intensity, primary system organ class and preferred term (FAS)

Primary system organ class<br>Preferred term<br>MedDRA version 14.0	Maximum AE maximum intensity	LCS12<br>N=1432 (100%)	LCS16<br>N=1452 (100%)	Total<br>N=2884 (100%)
Stress urinary incontinence	mild	0	1 (<0.1%)	1 (<0.1%)
	severe	1 (<0.1%)	1 (<0.1%)	2 (<0.1%)
Ureteric obstruction	moderate	0	1 (<0.1%)	1 (<0.1%)
Urethral cyst	mild	0	1 (<0.1%)	1 (<0.1%)
Urinary incontinence	mild	2 (0.1%)	0	2 (<0.1%)
	moderate	1 (<0.1%)	0	1 (<0.1%)
	severe	0	1 (<0.1%)	1 (<0.1%)
Urinary retention	mild	0	1 (<0.1%)	1 (<0.1%)
Urinary tract disorder	moderate	1 (<0.1%)	0	1 (<0.1%)
Urinary tract inflammation	mild	1 (<0.1%)	0	1 (<0.1%)
Urinary tract pain	mild	2 (0.1%)	1 (<0.1%)	3 (0.1%)
Urine odour abnormal	mild	0	1 (<0.1%)	1 (<0.1%)
Reproductive system and breast disorders	mild	323 (22.6%)	378 (26.0%)	701 (24.3%)
	moderate	269 (18.8%)	292 (20.1%)	561 (19.5%)
	severe	88 (6.1%)	93 (6.4%)	181 (6.3%)
Adenomyosis	mild	1 (<0.1%)	0	1 (<0.1%)
Adnexa uteri mass	moderate	1 (<0.1%)	0	1 (<0.1%)
Adnexa uteri pain	mild	0	2 (0.1%)	2 (<0.1%)
	moderate	3 (0.2%)	2 (0.1%)	5 (0.2%)
	severe	0	1 (<0.1%)	1 (<0.1%)
Amenorrhoea	mild	0	5 (0.3%)	5 (0.2%)
	moderate	0	1 (<0.1%)	1 (<0.1%)
Atrophic vulvovaginitis	mild	1 (<0.1%)	0	1 (<0.1%)

Table 14.3.1 / 16: Number of subjects with adverse events by maximum intensity, primary system organ class and preferred term (FAS)

Primary system organ class<br>Preferred term<br>MedDRA version 14.0	Maximum AE maximum intensity	LCS12<br>N=1432 (100%)	LCS16<br>N=1452 (100%)	Total<br>N=2884 (100%)
Bartholin's cyst	mild	0	1 (<0.1%)	1 (<0.1%)
Breast calcifications	mild	1 (<0.1%)	0	1 (<0.1%)
Breast cyst	mild	3 (0.2%)	2 (0.1%)	5 (0.2%)
Breast discharge	mild	0	8 (0.6%)	8 (0.3%)
	moderate	0	2 (0.1%)	2 (<0.1%)
Breast discomfort	mild	3 (0.2%)	2 (0.1%)	5 (0.2%)
	moderate	1 (<0.1%)	6 (0.4%)	7 (0.2%)
Breast disorder female	mild	0	1 (<0.1%)	1 (<0.1%)
Breast dysplasia	moderate	1 (<0.1%)	0	1 (<0.1%)
Breast engorgement	mild	1 (<0.1%)	0	1 (<0.1%)
Breast enlargement	mild	0	3 (0.2%)	3 (0.1%)
	severe	0	1 (<0.1%)	1 (<0.1%)
Breast mass	mild	6 (0.4%)	5 (0.3%)	11 (0.4%)
	moderate	0	2 (0.1%)	2 (<0.1%)
	severe	1 (<0.1%)	0	1 (<0.1%)
Breast pain	mild	21 (1.5%)	17 (1.2%)	38 (1.3%)
	moderate	13 (0.9%)	21 (1.4%)	34 (1.2%)
	severe	2 (0.1%)	5 (0.3%)	7 (0.2%)
Breast swelling	mild	0	1 (<0.1%)	1 (<0.1%)
	moderate	1 (<0.1%)	1 (<0.1%)	2 (<0.1%)
Breast tenderness	mild	20 (1.4%)	20 (1.4%)	40 (1.4%)
	moderate	11 (0.8%)	12 (0.8%)	23 (0.8%)
	severe	1 (<0.1%)	3 (0.2%)	4 (0.1%)
Cervical cyst	mild	0	1 (<0.1%)	1 (<0.1%)

Table 14.3.1 / 16: Number of subjects with adverse events by maximum intensity, primary system organ class and preferred term (FAS)

Primary system organ class<br>Preferred term<br>MedDRA version 14.0	Maximum AE maximum intensity	LCS12<br>N=1432 (100%)	LCS16<br>N=1452 (100%)	Total<br>N=2884 (100%)
Cervical discharge	mild	2 (0.1%)	0	2 (<0.1%)
Cervical dysplasia	mild	79 (5.5%)	89 (6.1%)	168 (5.8%)
	moderate	26 (1.8%)	25 (1.7%)	51 (1.8%)
	severe	1 (<0.1%)	1 (<0.1%)	2 (<0.1%)
Cervical friability	mild	0	1 (<0.1%)	1 (<0.1%)
Cervical polyp	mild	1 (<0.1%)	2 (0.1%)	3 (0.1%)
Cervix disorder	severe	0	1 (<0.1%)	1 (<0.1%)
Cervix erythema	mild	0	2 (0.1%)	2 (<0.1%)
Cervix haemorrhage uterine	mild	0	1 (<0.1%)	1 (<0.1%)
Cervix inflammation	mild	1 (<0.1%)	0	1 (<0.1%)
Cervix oedema	moderate	0	1 (<0.1%)	1 (<0.1%)
Coital bleeding	mild	8 (0.6%)	11 (0.8%)	19 (0.7%)
	moderate	6 (0.4%)	2 (0.1%)	8 (0.3%)
	severe	0	1 (<0.1%)	1 (<0.1%)
Dysfunctional uterine bleeding	mild	1 (<0.1%)	0	1 (<0.1%)
	moderate	2 (0.1%)	4 (0.3%)	6 (0.2%)
Dysmenorrhoea	mild	43 (3.0%)	24 (1.7%)	67 (2.3%)
	moderate	66 (4.6%)	61 (4.2%)	127 (4.4%)
	severe	21 (1.5%)	23 (1.6%)	44 (1.5%)
Dyspareunia	mild	12 (0.8%)	11 (0.8%)	23 (0.8%)
	moderate	19 (1.3%)	14 (1.0%)	33 (1.1%)
	severe	2 (0.1%)	3 (0.2%)	5 (0.2%)
Ectropion of cervix	mild	1 (<0.1%)	0	1 (<0.1%)
	severe	1 (<0.1%)	1 (<0.1%)	2 (<0.1%)

Table 14.3.1 / 16: Number of subjects with adverse events by maximum intensity, primary system organ class and preferred term (FAS)

Primary system organ class<br>Preferred term<br>MedDRA version 14.0	Maximum AE maximum intensity	LCS12<br>N=1432 (100%)	LCS16<br>N=1452 (100%)	Total<br>N=2884 (100%)
Endometriosis	mild	3 (0.2%)	1 (<0.1%)	4 (0.1%)
	moderate	2 (0.1%)	1 (<0.1%)	3 (0.1%)
	severe	3 (0.2%)	1 (<0.1%)	4 (0.1%)
Fibrocystic breast disease	mild	3 (0.2%)	2 (0.1%)	5 (0.2%)
	moderate	0	3 (0.2%)	3 (0.1%)
Galactorrhoea	mild	4 (0.3%)	8 (0.6%)	12 (0.4%)
Genital cyst	mild	0	1 (<0.1%)	1 (<0.1%)
Genital discharge	mild	7 (0.5%)	7 (0.5%)	14 (0.5%)
	moderate	2 (0.1%)	2 (0.1%)	4 (0.1%)
Genital haemorrhage	mild	1 (<0.1%)	0	1 (<0.1%)
Genital lesion	mild	2 (0.1%)	0	2 (<0.1%)
Genital rash	mild	0	1 (<0.1%)	1 (<0.1%)
Haemorrhagic ovarian cyst	mild	8 (0.6%)	12 (0.8%)	20 (0.7%)
	moderate	3 (0.2%)	5 (0.3%)	8 (0.3%)
	severe	2 (0.1%)	2 (0.1%)	4 (0.1%)
Hydrometra	mild	2 (0.1%)	0	2 (<0.1%)
Hypomenorrhoea	mild	2 (0.1%)	1 (<0.1%)	3 (0.1%)
Menometrorrhagia	severe	2 (0.1%)	0	2 (<0.1%)
Menorrhagia	mild	1 (<0.1%)	3 (0.2%)	4 (0.1%)
	moderate	3 (0.2%)	5 (0.3%)	8 (0.3%)
	severe	2 (0.1%)	2 (0.1%)	4 (0.1%)
Menstrual disorder	mild	3 (0.2%)	0	3 (0.1%)
	moderate	0	1 (<0.1%)	1 (<0.1%)

Table 14.3.1 / 16: Number of subjects with adverse events by maximum intensity, primary system organ class and preferred term (FAS)

Primary system organ class<br>Preferred term<br>MedDRA version 14.0	Maximum AE<br>maximum<br>intensity	LCS12<br>N=1432 (100%)	LCS16<br>N=1452 (100%)	Total<br>N=2884 (100%)
Menstruation irregular	mild	1 (<0.1%)	1 (<0.1%)	2 (<0.1%)
	moderate	3 (0.2%)	2 (0.1%)	5 (0.2%)
Metrorrhagia	mild	6 (0.4%)	7 (0.5%)	13 (0.5%)
	moderate	8 (0.6%)	3 (0.2%)	11 (0.4%)
	severe	2 (0.1%)	2 (0.1%)	4 (0.1%)
Nipple exudate bloody	mild	0	1 (<0.1%)	1 (<0.1%)
Nipple pain	mild	1 (<0.1%)	1 (<0.1%)	2 (<0.1%)
	moderate	1 (<0.1%)	1 (<0.1%)	2 (<0.1%)
	severe	1 (<0.1%)	0	1 (<0.1%)
Oligomenorrhoea	moderate	0	2 (0.1%)	2 (<0.1%)
Ovarian cyst	mild	154 (10.8%)	247 (17.0%)	401 (13.9%)
	moderate	25 (1.7%)	50 (3.4%)	75 (2.6%)
	severe	7 (0.5%)	7 (0.5%)	14 (0.5%)
Ovarian cyst ruptured	mild	0	1 (<0.1%)	1 (<0.1%)
	moderate	1 (<0.1%)	2 (0.1%)	3 (0.1%)
	severe	1 (<0.1%)	4 (0.3%)	5 (0.2%)
Ovarian cyst torsion	severe	0	1 (<0.1%)	1 (<0.1%)
Ovarian disorder	mild	0	1 (<0.1%)	1 (<0.1%)
Ovarian enlargement	mild	1 (<0.1%)	1 (<0.1%)	2 (<0.1%)
Ovarian mass	mild	1 (<0.1%)	2 (0.1%)	3 (0.1%)
Ovulation pain	mild	1 (<0.1%)	2 (0.1%)	3 (0.1%)
	moderate	5 (0.3%)	0	5 (0.2%)
Parovarian cyst	mild	2 (0.1%)	0	2 (<0.1%)
	moderate	1 (<0.1%)	0	1 (<0.1%)
Pelvic adhesions	moderate	0	1 (<0.1%)	1 (<0.1%)

Table 14.3.1 / 16: Number of subjects with adverse events by maximum intensity, primary system organ class and preferred term (FAS)

Primary system organ class<br>Preferred term<br>MedDRA version 14.0	Maximum AE<br>maximum<br>intensity	LCS12<br>N=1432 (100%)	LCS16<br>N=1452 (100%)	Total<br>N=2884 (100%)
Pelvic congestion	mild	1 (<0.1%)	0	1 (<0.1%)
Pelvic discomfort	mild	0	1 (<0.1%)	1 (<0.1%)
	moderate	6 (0.4%)	1 (<0.1%)	7 (0.2%)
Pelvic fluid collection	moderate	1 (<0.1%)	0	1 (<0.1%)
Pelvic pain	mild	18 (1.3%)	32 (2.2%)	50 (1.7%)
	moderate	52 (3.6%)	59 (4.1%)	111 (3.8%)
	severe	26 (1.8%)	32 (2.2%)	58 (2.0%)
Pelvic prolapse	moderate	1 (<0.1%)	0	1 (<0.1%)
Perineal pain	moderate	1 (<0.1%)	0	1 (<0.1%)
Polycystic ovaries	mild	4 (0.3%)	2 (0.1%)	6 (0.2%)
Polymenorrhoea	mild	1 (<0.1%)	1 (<0.1%)	2 (<0.1%)
Premenstrual syndrome	mild	8 (0.6%)	4 (0.3%)	12 (0.4%)
	moderate	8 (0.6%)	6 (0.4%)	14 (0.5%)
	severe	1 (<0.1%)	2 (0.1%)	3 (0.1%)
Pruritus genital	mild	1 (<0.1%)	0	1 (<0.1%)
Uterine cervical erosion	mild	1 (<0.1%)	0	1 (<0.1%)
Uterine cervical pain	mild	2 (0.1%)	0	2 (<0.1%)
Uterine haemorrhage	mild	1 (<0.1%)	5 (0.3%)	6 (0.2%)
	moderate	4 (0.3%)	2 (0.1%)	6 (0.2%)
	severe	3 (0.2%)	0	3 (0.1%)
Uterine inflammation	mild	0	1 (<0.1%)	1 (<0.1%)
Uterine pain	mild	1 (<0.1%)	1 (<0.1%)	2 (<0.1%)
	moderate	1 (<0.1%)	1 (<0.1%)	2 (<0.1%)

Table 14.3.1 / 16: Number of subjects with adverse events by maximum intensity, primary system organ class and preferred term (FAS)

Primary system organ class<br>Preferred term<br>MedDRA version 14.0	Maximum AE<br>maximum<br>intensity	LCS12<br>N=1432 (100%)	LCS16<br>N=1452 (100%)	Total<br>N=2884 (100%)
Uterine polyp	mild	2 (0.1%)	1 (<0.1%)	3 (0.1%)
Uterine prolapse	severe	1 (<0.1%)	0	1 (<0.1%)
Uterine spasm	mild	6 (0.4%)	9 (0.6%)	15 (0.5%)
	moderate	15 (1.0%)	21 (1.4%)	36 (1.2%)
	severe	9 (0.6%)	9 (0.6%)	18 (0.6%)
Uterine tenderness	mild	1 (<0.1%)	1 (<0.1%)	2 (<0.1%)
	moderate	0	1 (<0.1%)	1 (<0.1%)
Vaginal cyst	mild	1 (<0.1%)	0	1 (<0.1%)
Vaginal discharge	mild	33 (2.3%)	35 (2.4%)	68 (2.4%)
	moderate	18 (1.3%)	18 (1.2%)	36 (1.2%)
	severe	4 (0.3%)	5 (0.3%)	9 (0.3%)
Vaginal disorder	mild	3 (0.2%)	3 (0.2%)	6 (0.2%)
	moderate	1 (<0.1%)	1 (<0.1%)	2 (<0.1%)
Vaginal haemorrhage	mild	28 (2.0%)	31 (2.1%)	59 (2.0%)
	moderate	36 (2.5%)	39 (2.7%)	75 (2.6%)
	severe	2 (0.1%)	3 (0.2%)	5 (0.2%)
Vaginal lesion	moderate	1 (<0.1%)	0	1 (<0.1%)
Vaginal odour	mild	5 (0.3%)	6 (0.4%)	11 (0.4%)
	moderate	1 (<0.1%)	1 (<0.1%)	2 (<0.1%)
Vaginal perforation	mild	0	1 (<0.1%)	1 (<0.1%)
Vaginal ulceration	mild	0	1 (<0.1%)	1 (<0.1%)
Vaginal wall congestion	mild	1 (<0.1%)	0	1 (<0.1%)
Vulval disorder	moderate	1 (<0.1%)	0	1 (<0.1%)

Table 14.3.1 / 16: Number of subjects with adverse events by maximum intensity, primary system organ class and preferred term (FAS)

Primary system organ class<br>Preferred term<br>MedDRA version 14.0	Maximum AE<br>maximum<br>intensity	LCS12<br>N=1432 (100%)	LCS16<br>N=1452 (100%)	Total<br>N=2884 (100%)
Vulvovaginal burning sensation	mild	1 (<0.1%)	3 (0.2%)	4 (0.1%)
	moderate	0	1 (<0.1%)	1 (<0.1%)
Vulvovaginal discomfort	mild	2 (0.1%)	2 (0.1%)	4 (0.1%)
	moderate	4 (0.3%)	0	4 (0.1%)
Vulvovaginal dryness	mild	1 (<0.1%)	4 (0.3%)	5 (0.2%)
	moderate	2 (0.1%)	1 (<0.1%)	3 (0.1%)
Vulvovaginal erythema	mild	0	2 (0.1%)	2 (<0.1%)
Vulvovaginal pain	mild	2 (0.1%)	1 (<0.1%)	3 (0.1%)
	moderate	1 (<0.1%)	1 (<0.1%)	2 (<0.1%)
Vulvovaginal pruritus	mild	8 (0.6%)	14 (1.0%)	22 (0.8%)
	moderate	7 (0.5%)	3 (0.2%)	10 (0.3%)
	severe	1 (<0.1%)	0	1 (<0.1%)
Vulvovaginal swelling	mild	1 (<0.1%)	0	1 (<0.1%)
	moderate	0	1 (<0.1%)	1 (<0.1%)
Respiratory, thoracic and mediastinal disorders	mild	33 (2.3%)	27 (1.9%)	60 (2.1%)
	moderate	31 (2.2%)	34 (2.3%)	65 (2.3%)
	severe	7 (0.5%)	9 (0.6%)	16 (0.6%)
Allergic sinusitis	mild	1 (<0.1%)	1 (<0.1%)	2 (<0.1%)
Asthma	mild	4 (0.3%)	1 (<0.1%)	5 (0.2%)
	moderate	6 (0.4%)	8 (0.6%)	14 (0.5%)
	severe	1 (<0.1%)	2 (0.1%)	3 (0.1%)
Atelectasis	moderate	0	1 (<0.1%)	1 (<0.1%)
Bronchospasm	moderate	2 (0.1%)	1 (<0.1%)	3 (0.1%)
	severe	0	1 (<0.1%)	1 (<0.1%)

Table 14.3.1 / 16: Number of subjects with adverse events by maximum intensity, primary system organ class and preferred term (FAS)

Primary system organ class<br>Preferred term<br>MedDRA version 14.0	Maximum AE<br>maximum<br>intensity	LCS12<br>N=1432 (100%)	LCS16<br>N=1452 (100%)	Total<br>N=2884 (100%)
Cough	mild	6 (0.4%)	4 (0.3%)	10 (0.3%)
	moderate	5 (0.3%)	7 (0.5%)	12 (0.4%)
	severe	2 (0.1%)	2 (0.1%)	4 (0.1%)
Diaphragmatic hernia	moderate	0	1 (<0.1%)	1 (<0.1%)
Dyspnoea	mild	0	1 (<0.1%)	1 (<0.1%)
	moderate	1 (<0.1%)	0	1 (<0.1%)
	severe	0	1 (<0.1%)	1 (<0.1%)
Epiglottic cyst	moderate	1 (<0.1%)	0	1 (<0.1%)
Epistaxis	mild	3 (0.2%)	0	3 (0.1%)
	moderate	0	1 (<0.1%)	1 (<0.1%)
Hyperventilation	mild	1 (<0.1%)	0	1 (<0.1%)
	severe	0	1 (<0.1%)	1 (<0.1%)
Hypoxia	moderate	0	1 (<0.1%)	1 (<0.1%)
Nasal congestion	mild	1 (<0.1%)	0	1 (<0.1%)
	moderate	1 (<0.1%)	1 (<0.1%)	2 (<0.1%)
Nasal disorder	mild	1 (<0.1%)	0	1 (<0.1%)
Nasal oedema	moderate	1 (<0.1%)	0	1 (<0.1%)
Nasal polyps	moderate	1 (<0.1%)	1 (<0.1%)	2 (<0.1%)
Oropharyngeal pain	mild	5 (0.3%)	9 (0.6%)	14 (0.5%)
	moderate	8 (0.6%)	8 (0.6%)	16 (0.6%)
	severe	4 (0.3%)	0	4 (0.1%)
Pleural effusion	moderate	0	1 (<0.1%)	1 (<0.1%)
Pleurisy	severe	1 (<0.1%)	1 (<0.1%)	2 (<0.1%)
Pneumonitis	moderate	0	1 (<0.1%)	1 (<0.1%)

Table 14.3.1 / 16: Number of subjects with adverse events by maximum intensity, primary system organ class and preferred term (FAS)

Primary system organ class<br>Preferred term<br>MedDRA version 14.0	Maximum AE maximum intensity	LCS12<br>N=1432 (100%)	LCS16<br>N=1452 (100%)	Total<br>N=2884 (100%)
Pneumothorax	moderate	1 (<0.1%)	0	1 (<0.1%)
Pulmonary congestion	mild	1 (<0.1%)	0	1 (<0.1%)
Respiratory tract congestion	moderate	1 (<0.1%)	0	1 (<0.1%)
Respiratory tract inflammation	mild	1 (<0.1%)	0	1 (<0.1%)
Rhinitis allergic	mild	6 (0.4%)	6 (0.4%)	12 (0.4%)
	moderate	4 (0.3%)	5 (0.3%)	9 (0.3%)
Rhinitis seasonal	mild	0	1 (<0.1%)	1 (<0.1%)
	moderate	1 (<0.1%)	1 (<0.1%)	2 (<0.1%)
Rhinorrhoea	mild	1 (<0.1%)	0	1 (<0.1%)
Sinus congestion	mild	5 (0.3%)	2 (0.1%)	7 (0.2%)
	moderate	1 (<0.1%)	0	1 (<0.1%)
Sinus polyp	severe	0	1 (<0.1%)	1 (<0.1%)
Sleep apnoea syndrome	severe	0	1 (<0.1%)	1 (<0.1%)
Tonsillar disorder	mild	0	1 (<0.1%)	1 (<0.1%)
Upper respiratory tract inflammation	mild	0	2 (0.1%)	2 (<0.1%)
Wheezing	mild	1 (<0.1%)	0	1 (<0.1%)
Skin and subcutaneous tissue disorders	mild	120 (8.4%)	145 (10.0%)	265 (9.2%)
	moderate	104 (7.3%)	98 (6.7%)	202 (7.0%)
	severe	18 (1.3%)	11 (0.8%)	29 (1.0%)
Acne	mild	81 (5.7%)	104 (7.2%)	185 (6.4%)
	moderate	70 (4.9%)	58 (4.0%)	128 (4.4%)
	severe	12 (0.8%)	7 (0.5%)	19 (0.7%)

Table 14.3.1 / 16: Number of subjects with adverse events by maximum intensity, primary system organ class and preferred term (FAS)

Primary system organ class<br>Preferred term<br>MedDRA version 14.0	Maximum AE maximum intensity	LCS12<br>N=1432 (100%)	LCS16<br>N=1452 (100%)	Total<br>N=2884 (100%)
Acne cystic	mild	1 (<0.1%)	0	1 (<0.1%)
	severe	1 (<0.1%)	0	1 (<0.1%)
Alopecia	mild	10 (0.7%)	8 (0.6%)	18 (0.6%)
	moderate	4 (0.3%)	4 (0.3%)	8 (0.3%)
	severe	1 (<0.1%)	1 (<0.1%)	2 (<0.1%)
Alopecia areata	mild	1 (<0.1%)	0	1 (<0.1%)
	moderate	1 (<0.1%)	0	1 (<0.1%)
Chloasma	mild	2 (0.1%)	0	2 (<0.1%)
	moderate	1 (<0.1%)	0	1 (<0.1%)
Cold sweat	mild	1 (<0.1%)	0	1 (<0.1%)
Dandruff	mild	1 (<0.1%)	1 (<0.1%)	2 (<0.1%)
Dermal cyst	mild	2 (0.1%)	0	2 (<0.1%)
Dermatitis	mild	7 (0.5%)	3 (0.2%)	10 (0.3%)
	moderate	1 (<0.1%)	4 (0.3%)	5 (0.2%)
Dermatitis allergic	mild	3 (0.2%)	2 (0.1%)	5 (0.2%)
	moderate	3 (0.2%)	3 (0.2%)	6 (0.2%)
Dermatitis atopic	mild	1 (<0.1%)	3 (0.2%)	4 (0.1%)
	moderate	1 (<0.1%)	2 (0.1%)	3 (0.1%)
Dermatitis contact	mild	1 (<0.1%)	2 (0.1%)	3 (0.1%)
	moderate	0	3 (0.2%)	3 (0.1%)
	severe	1 (<0.1%)	0	1 (<0.1%)
Drug eruption	severe	1 (<0.1%)	0	1 (<0.1%)
Dry skin	mild	0	1 (<0.1%)	1 (<0.1%)
Dyshidrosis	moderate	1 (<0.1%)	0	1 (<0.1%)

Table 14.3.1 / 16: Number of subjects with adverse events by maximum intensity, primary system organ class and preferred term (FAS)

Primary system organ class<br>Preferred term<br>MedDRA version 14.0	Maximum AE maximum intensity	LCS12<br>N=1432 (100%)	LCS16<br>N=1452 (100%)	Total<br>N=2884 (100%)
Eczema	mild	4 (0.3%)	6 (0.4%)	10 (0.3%)
	moderate	2 (0.1%)	2 (0.1%)	4 (0.1%)
Erythema	mild	2 (0.1%)	0	2 (<0.1%)
Erythema multiforme	moderate	1 (<0.1%)	0	1 (<0.1%)
Hidradenitis	mild	1 (<0.1%)	0	1 (<0.1%)
Hirsutism	mild	3 (0.2%)	7 (0.5%)	10 (0.3%)
	moderate	4 (0.3%)	3 (0.2%)	7 (0.2%)
Hyperhidrosis	mild	1 (<0.1%)	1 (<0.1%)	2 (<0.1%)
	moderate	2 (0.1%)	1 (<0.1%)	3 (0.1%)
Hyperkeratosis	mild	1 (<0.1%)	0	1 (<0.1%)
Hypertrichosis	mild	2 (0.1%)	1 (<0.1%)	3 (0.1%)
Idiopathic urticaria	moderate	1 (<0.1%)	0	1 (<0.1%)
Increased tendency to bruise	mild	1 (<0.1%)	0	1 (<0.1%)
Ingrown hair	mild	1 (<0.1%)	0	1 (<0.1%)
Intertrigo	mild	1 (<0.1%)	0	1 (<0.1%)
Lentigo	mild	1 (<0.1%)	0	1 (<0.1%)
Lichen sclerosus	mild	1 (<0.1%)	1 (<0.1%)	2 (<0.1%)
	moderate	0	1 (<0.1%)	1 (<0.1%)
Neurodermatitis	mild	0	1 (<0.1%)	1 (<0.1%)
Night sweats	moderate	0	1 (<0.1%)	1 (<0.1%)
Photosensitivity reaction	moderate	0	1 (<0.1%)	1 (<0.1%)

Table 14.3.1 / 16: Number of subjects with adverse events by maximum intensity, primary system organ class and preferred term (FAS)

Primary system organ class<br>Preferred term<br>MedDRA version 14.0	Maximum AE<br>maximum<br>intensity	LCS12<br>N=1432 (100%)	LCS16<br>N=1452 (100%)	Total<br>N=2884 (100%)
Pityriasis rosea	mild	1 (<0.1%)	1 (<0.1%)	2 (<0.1%)
	severe	0	1 (<0.1%)	1 (<0.1%)
Precancerous skin lesion	moderate	0	1 (<0.1%)	1 (<0.1%)
Pruritus	mild	2 (0.1%)	3 (0.2%)	5 (0.2%)
	moderate	1 (<0.1%)	0	1 (<0.1%)
	severe	1 (<0.1%)	0	1 (<0.1%)
Pruritus allergic	mild	0	2 (0.1%)	2 (<0.1%)
Psoriasis	mild	0	1 (<0.1%)	1 (<0.1%)
	severe	0	1 (<0.1%)	1 (<0.1%)
Rash	mild	6 (0.4%)	4 (0.3%)	10 (0.3%)
	moderate	4 (0.3%)	4 (0.3%)	8 (0.3%)
Rash macular	mild	0	1 (<0.1%)	1 (<0.1%)
Rash papular	mild	1 (<0.1%)	1 (<0.1%)	2 (<0.1%)
	moderate	1 (<0.1%)	0	1 (<0.1%)
Rosacea	mild	1 (<0.1%)	1 (<0.1%)	2 (<0.1%)
	severe	0	1 (<0.1%)	1 (<0.1%)
Scar	moderate	0	1 (<0.1%)	1 (<0.1%)
Seborrhoea	mild	4 (0.3%)	5 (0.3%)	9 (0.3%)
	moderate	3 (0.2%)	5 (0.3%)	8 (0.3%)
Skin dystrophy	moderate	1 (<0.1%)	0	1 (<0.1%)
Skin fissures	mild	0	2 (0.1%)	2 (<0.1%)
Skin hypopigmentation	mild	0	1 (<0.1%)	1 (<0.1%)
Skin irritation	mild	1 (<0.1%)	0	1 (<0.1%)
	moderate	0	2 (0.1%)	2 (<0.1%)

Table 14.3.1 / 16: Number of subjects with adverse events by maximum intensity, primary system organ class and preferred term (FAS)

Primary system organ class<br>Preferred term<br>MedDRA version 14.0	Maximum AE maximum intensity	LCS12<br>N=1432 (100%)	LCS16<br>N=1452 (100%)	Total<br>N=2884 (100%)
Skin lesion	mild	0	1 (<0.1%)	1 (<0.1%)
Skin odour abnormal	moderate	0	1 (<0.1%)	1 (<0.1%)
Skin reaction	mild	1 (<0.1%)	0	1 (<0.1%)
	severe	1 (<0.1%)	0	1 (<0.1%)
Urticaria	mild	2 (0.1%)	2 (0.1%)	4 (0.1%)
	moderate	9 (0.6%)	8 (0.6%)	17 (0.6%)
Vitiligo	mild	0	1 (<0.1%)	1 (<0.1%)
Social circumstances	mild	1 (<0.1%)	1 (<0.1%)	2 (<0.1%)
Exposure to communicable disease	mild	1 (<0.1%)	0	1 (<0.1%)
Hearing disability	mild	0	1 (<0.1%)	1 (<0.1%)
Surgical and medical procedures	mild	20 (1.4%)	17 (1.2%)	37 (1.3%)
	moderate	14 (1.0%)	19 (1.3%)	33 (1.1%)
	severe	6 (0.4%)	9 (0.6%)	15 (0.5%)
Abdominoplasty	mild	3 (0.2%)	1 (<0.1%)	4 (0.1%)
	severe	1 (<0.1%)	0	1 (<0.1%)
Anal fissure excision	severe	1 (<0.1%)	0	1 (<0.1%)
Anal sphincterotomy	moderate	0	1 (<0.1%)	1 (<0.1%)
Apicectomy	severe	0	1 (<0.1%)	1 (<0.1%)
Breast prosthesis implantation	mild	1 (<0.1%)	0	1 (<0.1%)
	moderate	1 (<0.1%)	1 (<0.1%)	2 (<0.1%)
	severe	1 (<0.1%)	0	1 (<0.1%)
Bunion operation	moderate	2 (0.1%)	0	2 (<0.1%)
	severe	0	1 (<0.1%)	1 (<0.1%)

Table 14.3.1 / 16: Number of subjects with adverse events by maximum intensity, primary system organ class and preferred term (FAS)

Primary system organ class<br>Preferred term<br>MedDRA version 14.0	Maximum AE<br>maximum<br>intensity	LCS12<br>N=1432 (100%)	LCS16<br>N=1452 (100%)	Total<br>N=2884 (100%)
Cervix cautery	moderate	0	1 (<0.1%)	1 (<0.1%)
Cholecystectomy	severe	1 (<0.1%)	0	1 (<0.1%)
Dental cosmetic procedure	mild	1 (<0.1%)	0	1 (<0.1%)
Dental operation	mild	1 (<0.1%)	3 (0.2%)	4 (0.1%)
Detoxification	severe	1 (<0.1%)	1 (<0.1%)	2 (<0.1%)
Endodontic procedure	mild	0	1 (<0.1%)	1 (<0.1%)
	moderate	0	1 (<0.1%)	1 (<0.1%)
Eye laser surgery	mild	0	1 (<0.1%)	1 (<0.1%)
Gallbladder operation	severe	0	1 (<0.1%)	1 (<0.1%)
Gastric banding	moderate	1 (<0.1%)	0	1 (<0.1%)
Gastric bypass	mild	0	1 (<0.1%)	1 (<0.1%)
Haemorrhoid operation	mild	0	1 (<0.1%)	1 (<0.1%)
Keratomileusis	moderate	0	1 (<0.1%)	1 (<0.1%)
Ligament operation	moderate	1 (<0.1%)	0	1 (<0.1%)
Lipoma excision	mild	1 (<0.1%)	0	1 (<0.1%)
Mammoplasty	mild	4 (0.3%)	0	4 (0.1%)
	moderate	2 (0.1%)	2 (0.1%)	4 (0.1%)
	severe	0	1 (<0.1%)	1 (<0.1%)
Maxillary antrum operation	severe	1 (<0.1%)	0	1 (<0.1%)
Meniscus operation	mild	0	1 (<0.1%)	1 (<0.1%)

Table 14.3.1 / 16: Number of subjects with adverse events by maximum intensity, primary system organ class and preferred term (FAS)

Primary system organ class<br>Preferred term<br>MedDRA version 14.0	Maximum AE maximum intensity	LCS12<br>N=1432 (100%)	LCS16<br>N=1452 (100%)	Total<br>N=2884 (100%)
Mole excision	mild	1 (<0.1%)	1 (<0.1%)	2 (<0.1%)
	moderate	1 (<0.1%)	1 (<0.1%)	2 (<0.1%)
Nasal polypectomy	moderate	0	1 (<0.1%)	1 (<0.1%)
Parasitic infection prophylaxis	mild	0	1 (<0.1%)	1 (<0.1%)
Plastic surgery	mild	0	1 (<0.1%)	1 (<0.1%)
Postoperative analgesia	moderate	1 (<0.1%)	0	1 (<0.1%)
Rotator cuff repair	moderate	0	1 (<0.1%)	1 (<0.1%)
	severe	0	1 (<0.1%)	1 (<0.1%)
Scar excision	mild	0	1 (<0.1%)	1 (<0.1%)
Skin neoplasm excision	severe	0	1 (<0.1%)	1 (<0.1%)
Tenolysis	moderate	0	1 (<0.1%)	1 (<0.1%)
Tonsillectomy	moderate	1 (<0.1%)	1 (<0.1%)	2 (<0.1%)
	severe	0	1 (<0.1%)	1 (<0.1%)
Tooth extraction	mild	3 (0.2%)	4 (0.3%)	7 (0.2%)
	moderate	2 (0.1%)	4 (0.3%)	6 (0.2%)
	severe	1 (<0.1%)	1 (<0.1%)	2 (<0.1%)
Umbilical hernia repair	mild	0	1 (<0.1%)	1 (<0.1%)
Varicose vein operation	moderate	1 (<0.1%)	0	1 (<0.1%)
Wisdom teeth removal	mild	6 (0.4%)	1 (<0.1%)	7 (0.2%)
	moderate	1 (<0.1%)	4 (0.3%)	5 (0.2%)
Wrist surgery	moderate	0	1 (<0.1%)	1 (<0.1%)

Table 14.3.1 / 16: Number of subjects with adverse events by maximum intensity, primary system organ class and preferred term (FAS)

Primary system organ class<br>Preferred term<br>MedDRA version 14.0	Maximum AE maximum intensity	LCS12<br>N=1432 (100%)	LCS16<br>N=1452 (100%)	Total<br>N=2884 (100%)
Vascular disorders	mild	7 (0.5%)	15 (1.0%)	22 (0.8%)
	moderate	10 (0.7%)	9 (0.6%)	19 (0.7%)
	severe	3 (0.2%)	2 (0.1%)	5 (0.2%)
Deep vein thrombosis	severe	1 (<0.1%)	0	1 (<0.1%)
Hot flush	mild	1 (<0.1%)	1 (<0.1%)	2 (<0.1%)
	moderate	3 (0.2%)	2 (0.1%)	5 (0.2%)
Hypertension	mild	6 (0.4%)	13 (0.9%)	19 (0.7%)
	moderate	3 (0.2%)	3 (0.2%)	6 (0.2%)
	severe	2 (0.1%)	0	2 (<0.1%)
Hypotension	mild	0	1 (<0.1%)	1 (<0.1%)
	moderate	1 (<0.1%)	0	1 (<0.1%)
	severe	0	1 (<0.1%)	1 (<0.1%)
Neurogenic shock	moderate	2 (0.1%)	0	2 (<0.1%)
Phlebitis	moderate	0	1 (<0.1%)	1 (<0.1%)
Thrombophlebitis superficial	mild	1 (<0.1%)	0	1 (<0.1%)
Varicose vein	mild	0	1 (<0.1%)	1 (<0.1%)
	moderate	1 (<0.1%)	3 (0.2%)	4 (0.1%)
	severe	0	1 (<0.1%)	1 (<0.1%)
Vein pain	moderate	0	1 (<0.1%)	1 (<0.1%)

Note: A subject is counted only once within each preferred term of any primary SOC.
Note: Adverse events are sorted in alphabetical order by primary SOC and preferred term.
Note: Only the most severe intensity is counted for multiple occurrences of the same AE in one individual.
Note: laboratory AEs are not covered by this table as no intensity could be given on CRF
Global Biostatistics: /by-sasp/patdb/projects/de04209/310442/stat/prod_interim03/pgms/t-ae.sas epkll 12OCT2011 11:24
End of table

Table 14.3.1 / 17: Number of subjects with serious adverse events by primary system organ class and preferred term (FAS)

Primary system organ class<br>Preferred term<br>MedDRA version 14.0	LCS12<br>N=1432 (100%)	LCS16<br>N=1452 (100%)	Total<br>N=2884 (100%)
Number of subjects (%) with at least one SAE	66 (4.6%)	71 (4.9%)	137 (4.8%)
Blood and lymphatic system disorders	1 (<0.1%)	0	1 (<0.1%)
Spherocytic anaemia	1 (<0.1%)	0	1 (<0.1%)
Splenomegaly	1 (<0.1%)	0	1 (<0.1%)
Cardiac disorders	0	1 (<0.1%)	1 (<0.1%)
Mitral valve prolapse	0	1 (<0.1%)	1 (<0.1%)
Sinus tachycardia	0	1 (<0.1%)	1 (<0.1%)
Ear and labyrinth disorders	0	1 (<0.1%)	1 (<0.1%)
Vertigo	0	1 (<0.1%)	1 (<0.1%)
Endocrine disorders	1 (<0.1%)	1 (<0.1%)	2 (<0.1%)
Goitre	1 (<0.1%)	1 (<0.1%)	2 (<0.1%)
Eye disorders	1 (<0.1%)	0	1 (<0.1%)
Iridocyclitis	1 (<0.1%)	0	1 (<0.1%)
Gastrointestinal disorders	9 (0.6%)	9 (0.6%)	18 (0.6%)
Abdominal adhesions	1 (<0.1%)	0	1 (<0.1%)
Abdominal hernia	1 (<0.1%)	0	1 (<0.1%)
Abdominal pain	5 (0.3%)	4 (0.3%)	9 (0.3%)
Constipation	1 (<0.1%)	0	1 (<0.1%)
Crohn's disease	1 (<0.1%)	0	1 (<0.1%)
Dysphagia	0	1 (<0.1%)	1 (<0.1%)
Gastritis	0	1 (<0.1%)	1 (<0.1%)
Impaired gastric emptying	1 (<0.1%)	0	1 (<0.1%)
Irritable bowel syndrome	1 (<0.1%)	0	1 (<0.1%)
Odynophagia	0	1 (<0.1%)	1 (<0.1%)
Peritonitis	1 (<0.1%)	0	1 (<0.1%)
Rectal haemorrhage	0	1 (<0.1%)	1 (<0.1%)
Salivary gland calculus	0	1 (<0.1%)	1 (<0.1%)
Umbilical hernia	0	1 (<0.1%)	1 (<0.1%)
General disorders and administration site conditions	2 (0.1%)	1 (<0.1%)	3 (0.1%)
Chest pain	1 (<0.1%)	0	1 (<0.1%)
Device dislocation	0	1 (<0.1%)	1 (<0.1%)
Pyrexia	1 (<0.1%)	0	1 (<0.1%)

Table 14.3.1 / 17: Number of subjects with serious adverse events by primary system organ class and preferred term (FAS)

Primary system organ class<br>Preferred term<br>MedDRA version 14.0	LCS12<br>N=1432 (100%)	LCS16<br>N=1452 (100%)	Total<br>N=2884 (100%)
Hepatobiliary disorders	4 (0.3%)	3 (0.2%)	7 (0.2%)
Cholecystitis	2 (0.1%)	2 (0.1%)	4 (0.1%)
Cholecystitis chronic	1 (<0.1%)	0	1 (<0.1%)
Cholelithiasis	1 (<0.1%)	2 (0.1%)	3 (0.1%)
Gallbladder polyp	1 (<0.1%)	0	1 (<0.1%)
Immune system disorders	1 (<0.1%)	1 (<0.1%)	2 (<0.1%)
Anaphylactic reaction	0	1 (<0.1%)	1 (<0.1%)
Drug hypersensitivity	1 (<0.1%)	0	1 (<0.1%)
Infections and infestations	24 (1.7%)	20 (1.4%)	44 (1.5%)
Appendicitis	6 (0.4%)	7 (0.5%)	13 (0.5%)
Campylobacter intestinal infection	0	1 (<0.1%)	1 (<0.1%)
Cellulitis	1 (<0.1%)	1 (<0.1%)	2 (<0.1%)
Cystitis	1 (<0.1%)	0	1 (<0.1%)
Diverticulitis	1 (<0.1%)	0	1 (<0.1%)
Enterocolitis infectious	1 (<0.1%)	0	1 (<0.1%)
Gastroenteritis	1 (<0.1%)	0	1 (<0.1%)
Genital herpes	1 (<0.1%)	0	1 (<0.1%)
Herpes dermatitis	1 (<0.1%)	0	1 (<0.1%)
Infection	0	1 (<0.1%)	1 (<0.1%)
Meningitis	1 (<0.1%)	0	1 (<0.1%)
Meningitis viral	0	1 (<0.1%)	1 (<0.1%)
Pelvic inflammatory disease	2 (0.1%)	4 (0.3%)	6 (0.2%)
Peritoneal abscess	1 (<0.1%)	0	1 (<0.1%)
Peritonsillar abscess	1 (<0.1%)	1 (<0.1%)	2 (<0.1%)
Pneumonia	2 (0.1%)	1 (<0.1%)	3 (0.1%)
Pyelonephritis	1 (<0.1%)	1 (<0.1%)	2 (<0.1%)
Pyelonephritis acute	1 (<0.1%)	0	1 (<0.1%)
Sinusitis	0	1 (<0.1%)	1 (<0.1%)
Streptococcal sepsis	1 (<0.1%)	0	1 (<0.1%)
Tonsillitis	1 (<0.1%)	0	1 (<0.1%)
Tubo-ovarian abscess	1 (<0.1%)	0	1 (<0.1%)
Upper respiratory tract infection	1 (<0.1%)	0	1 (<0.1%)
Urinary tract infection	2 (0.1%)	0	2 (<0.1%)
Vestibular neuronitis	1 (<0.1%)	0	1 (<0.1%)
Viral infection	0	1 (<0.1%)	1 (<0.1%)

Table 14.3.1 / 17: Number of subjects with serious adverse events by primary system organ class and preferred term (FAS)

Primary system organ class<br>Preferred term<br>MedDRA version 14.0	LCS12<br>N=1432 (100%)	LCS16<br>N=1452 (100%)	Total<br>N=2884 (100%)
Injury, poisoning and procedural complications	5 (0.3%)	6 (0.4%)	11 (0.4%)
Ankle fracture	0	1 (<0.1%)	1 (<0.1%)
Foot fracture	0	1 (<0.1%)	1 (<0.1%)
Forearm fracture	1 (<0.1%)	0	1 (<0.1%)
Joint sprain	1 (<0.1%)	0	1 (<0.1%)
Meniscus lesion	1 (<0.1%)	0	1 (<0.1%)
Overdose	1 (<0.1%)	0	1 (<0.1%)
Procedural pain	0	2 (0.1%)	2 (<0.1%)
Subdural haematoma	1 (<0.1%)	0	1 (<0.1%)
Tendon rupture	0	1 (<0.1%)	1 (<0.1%)
Thermal burn	0	1 (<0.1%)	1 (<0.1%)
Investigations	1 (<0.1%)	1 (<0.1%)	2 (<0.1%)
Haemoglobin decreased	0	1 (<0.1%)	1 (<0.1%)
Weight increased	1 (<0.1%)	0	1 (<0.1%)
Metabolism and nutrition disorders	0	1 (<0.1%)	1 (<0.1%)
Diabetic ketoacidosis	0	1 (<0.1%)	1 (<0.1%)
Musculoskeletal and connective tissue disorders	1 (<0.1%)	4 (0.3%)	5 (0.2%)
Arthritis	0	1 (<0.1%)	1 (<0.1%)
Axillary mass	0	1 (<0.1%)	1 (<0.1%)
Joint instability	0	1 (<0.1%)	1 (<0.1%)
Loose body in joint	0	1 (<0.1%)	1 (<0.1%)
Synovitis	1 (<0.1%)	0	1 (<0.1%)
Neoplasms benign, malignant and unspecified (incl cysts and polyps)	7 (0.5%)	5 (0.3%)	12 (0.4%)
Acute leukaemia	1 (<0.1%)	0	1 (<0.1%)
Astrocytoma, low grade	1 (<0.1%)	0	1 (<0.1%)
Cervix carcinoma stage 0	0	1 (<0.1%)	1 (<0.1%)
Ovarian germ cell teratoma benign	2 (0.1%)	2 (0.1%)	4 (0.1%)
Pancreatic carcinoma	0	1 (<0.1%)	1 (<0.1%)
Teratoma benign	0	1 (<0.1%)	1 (<0.1%)
Thyroid cancer	2 (0.1%)	0	2 (<0.1%)
Uterine leiomyoma	1 (<0.1%)	0	1 (<0.1%)

Table 14.3.1 / 17: Number of subjects with serious adverse events by primary system organ class and preferred term (FAS)

Primary system organ class<br>Preferred term<br>MedDRA version 14.0	LCS12<br>N=1432 (100%)	LCS16<br>N=1452 (100%)	Total<br>N=2884 (100%)
Nervous system disorders	1 (<0.1%)	5 (0.3%)	6 (0.2%)
Convulsion	0	1 (<0.1%)	1 (<0.1%)
Dizziness	0	2 (0.1%)	2 (<0.1%)
Headache	0	1 (<0.1%)	1 (<0.1%)
Paraesthesia	0	1 (<0.1%)	1 (<0.1%)
Sciatica	0	1 (<0.1%)	1 (<0.1%)
Status migrainosus	1 (<0.1%)	0	1 (<0.1%)
Pregnancy, puerperium and perinatal conditions	6 (0.4%)	9 (0.6%)	15 (0.5%)
Abortion spontaneous	3 (0.2%)	0	3 (0.1%)
Abortion spontaneous incomplete	0	1 (<0.1%)	1 (<0.1%)
Blighted ovum	0	1 (<0.1%)	1 (<0.1%)
Ectopic pregnancy	3 (0.2%)	6 (0.4%)	9 (0.3%)
Premature separation of placenta	1 (<0.1%)	0	1 (<0.1%)
Ruptured ectopic pregnancy	0	1 (<0.1%)	1 (<0.1%)
Psychiatric disorders	3 (0.2%)	5 (0.3%)	8 (0.3%)
Affective disorder	0	1 (<0.1%)	1 (<0.1%)
Alcoholism	0	1 (<0.1%)	1 (<0.1%)
Anxiety	1 (<0.1%)	1 (<0.1%)	2 (<0.1%)
Bipolar disorder	0	1 (<0.1%)	1 (<0.1%)
Completed suicide	0	1 (<0.1%)	1 (<0.1%)
Depression	1 (<0.1%)	1 (<0.1%)	2 (<0.1%)
Depression suicidal	1 (<0.1%)	0	1 (<0.1%)
Drug dependence	0	1 (<0.1%)	1 (<0.1%)
Hallucination, auditory	1 (<0.1%)	0	1 (<0.1%)
Panic disorder	0	1 (<0.1%)	1 (<0.1%)
Polysubstance dependence	0	1 (<0.1%)	1 (<0.1%)
Suicide attempt	1 (<0.1%)	0	1 (<0.1%)
Renal and urinary disorders	3 (0.2%)	0	3 (0.1%)
Haematuria	1 (<0.1%)	0	1 (<0.1%)
Strangury	1 (<0.1%)	0	1 (<0.1%)
Stress urinary incontinence	1 (<0.1%)	0	1 (<0.1%)

Table 14.3.1 / 17: Number of subjects with serious adverse events by primary system organ class and preferred term (FAS)

Primary system organ class<br>Preferred term<br>MedDRA version 14.0	LCS12<br>N=1432 (100%)	LCS16<br>N=1452 (100%)	Total<br>N=2884 (100%)
Reproductive system and breast disorders	7 (0.5%)	5 (0.3%)	12 (0.4%)
Endometriosis	1 (<0.1%)	0	1 (<0.1%)
Haemorrhagic ovarian cyst	2 (0.1%)	0	2 (<0.1%)
Ovarian cyst	1 (<0.1%)	2 (0.1%)	3 (0.1%)
Ovarian cyst ruptured	1 (<0.1%)	1 (<0.1%)	2 (<0.1%)
Ovarian cyst torsion	0	1 (<0.1%)	1 (<0.1%)
Pelvic adhesions	0	1 (<0.1%)	1 (<0.1%)
Uterine prolapse	1 (<0.1%)	0	1 (<0.1%)
Uterine spasm	1 (<0.1%)	0	1 (<0.1%)
Vaginal perforation	0	1 (<0.1%)	1 (<0.1%)
Respiratory, thoracic and mediastinal disorders	2 (0.1%)	1 (<0.1%)	3 (0.1%)
Asthma	1 (<0.1%)	1 (<0.1%)	2 (<0.1%)
Dyspnoea	0	1 (<0.1%)	1 (<0.1%)
Hypoxia	0	1 (<0.1%)	1 (<0.1%)
Pneumothorax	1 (<0.1%)	0	1 (<0.1%)
Skin and subcutaneous tissue disorders	1 (<0.1%)	1 (<0.1%)	2 (<0.1%)
Erythema multiforme	1 (<0.1%)	0	1 (<0.1%)
Urticaria	0	1 (<0.1%)	1 (<0.1%)
Surgical and medical procedures	1 (<0.1%)	1 (<0.1%)	2 (<0.1%)
Detoxification	1 (<0.1%)	1 (<0.1%)	2 (<0.1%)
Vascular disorders	2 (0.1%)	0	2 (<0.1%)
Deep vein thrombosis	1 (<0.1%)	0	1 (<0.1%)
Hypertension	1 (<0.1%)	0	1 (<0.1%)

Note: A subject is counted only once within each preferred term of any primary SOC.
Note: Adverse events are sorted in alphabetical order by primary SOC and preferred term.
Global Biostatistics: /by-sasp/patdb/projects/de04209/310442/stat/prod_interim03/pgms/t-ae.sas epkll 12OCT2011 11:24
End of table

Table 14.3.1 / 18: Number of subjects with study drug-related serious adverse events by primary system organ class and preferred term (FAS)

Primary system organ class<br>Preferred term<br>MedDRA version 14.0	LCS12<br>N=1432 (100%)	LCS16<br>N=1452 (100%)	Total<br>N=2884 (100%)
Number of subjects (%) with at least one SAE	8 (0.6%)	15 (1.0%)	23 (0.8%)
Gastrointestinal disorders	1 (<0.1%)	2 (0.1%)	3 (0.1%)
Abdominal pain	1 (<0.1%)	2 (0.1%)	3 (0.1%)
General disorders and administration site conditions	0	1 (<0.1%)	1 (<0.1%)
Device dislocation	0	1 (<0.1%)	1 (<0.1%)
Infections and infestations	2 (0.1%)	4 (0.3%)	6 (0.2%)
Pelvic inflammatory disease	2 (0.1%)	4 (0.3%)	6 (0.2%)
Tubo-ovarian abscess	1 (<0.1%)	0	1 (<0.1%)
Pregnancy, puerperium and perinatal conditions	3 (0.2%)	8 (0.6%)	11 (0.4%)
Abortion spontaneous	1 (<0.1%)	0	1 (<0.1%)
Abortion spontaneous incomplete	0	1 (<0.1%)	1 (<0.1%)
Ectopic pregnancy	2 (0.1%)	6 (0.4%)	8 (0.3%)
Premature separation of placenta	1 (<0.1%)	0	1 (<0.1%)
Ruptured ectopic pregnancy	0	1 (<0.1%)	1 (<0.1%)
Reproductive system and breast disorders	2 (0.1%)	1 (<0.1%)	3 (0.1%)
Haemorrhagic ovarian cyst	1 (<0.1%)	0	1 (<0.1%)
Ovarian cyst	0	1 (<0.1%)	1 (<0.1%)
Ovarian cyst ruptured	1 (<0.1%)	0	1 (<0.1%)

Note: A subject is counted only once within each preferred term of any primary SOC.
Note: Adverse events are sorted in alphabetical order by primary SOC and preferred term.
Global Biostatistics: /by-sasp/patdb/projects/de04209/310442/stat/prod_interim03/pgms/t-ae.sas epkll 12OCT2011 11:24
End of table

14.3.2 Listings of deaths, other serious and significant adverse events

Table 14.3.2 / 1: Deaths - Subjects listing (FAS)

TREATMENT	Subject/age/sex/race	IUS insertion date	Last day on study medication (imputed)	Death date text	Relative to start day/relative to stop day	Cause of death AE text	MedDRA preferred term with fatal outcome	AE study drug relation	Cause of death text
LCS16	210112/ 20/female/Caucasian	11JAN08	15JUL10	JUL2010			Completed suicide	no	REASONS UNKNOWN SUCIDE.

Note: Relative to start day = date of death minus date of first study drug administration plus 1 day.
Relative to stop day = date of death minus date of last study drug administration plus 1 day.
Note: Dictionary/ MedDRA Version 14.0
Global Biostatistics: /by-sasp/patdb/projects/de04209/310442/stat/prod_interim03/pgms/t-sae.sas epkll 12OCT2011 11:27
End of table

Table 14.3.2 / 2: Serious adverse events - Subject listing (FAS)

TREATMENT: LCS12

Subject/ age/sex/race	Primary SOC/preferred term/AEX	AE maximum intensity	Related to study drug/protocol-required procedure	Onset date/stop date/study day	AE Duration (days)	AE related study drug action	AE related drug treatment/non-drug treatment	AE outcome
120330/ 21/female/other	Gastrointestinal disorders/Peritonitis/PERITONITIS	severe	NO/NO	11SEP2009/15SEP2009/ 519	5	dose not changed	YES/YES	recovered/ resolved
	Infections and infestations/Appendicitis/[ACUTE APENDICITIS]	severe	NO/NO	11SEP2009/15SEP2009/ 519	5	dose not changed	YES/YES	recovered/ resolved
	Infections and infestations/Urinary tract infection/URINARY INFECTION	severe	NO/NO	16SEP2009/20SEP2009/ 524	5	dose not changed	YES/NO	recovered/ resolved
120419/ 30/female/Caucasian	Pregnancy, puerperium and perinatal conditions/Ectopic pregnancy/ECTOPIC PREGNANCY	severe	YES/NO	27JAN2009/02FEB2009/ 259	7	drug withdrawn	NO/YES	recovered/ resolved
140114/ 32/female/Caucasian	Psychiatric disorders/Hallucination, auditory/AUDITIVE HALLUCINATIONS	moderate	NO/NO	10APR2009/27APR2009/ 347	18	dose not changed	YES/NO	recovered/ resolved
140118/ 24/female/Caucasian	Infections and infestations/Cystitis/CYSTITIS	severe	NO/NO	13JUL2008/14JUL2008/ 55	2	dose not changed	YES/NO	recovered/ resolved

Table 14.3.2 / 2: Serious adverse events - Subject listing (FAS)

TREATMENT: LCS12

Subject/ age/sex/race	Primary SOC/preferred term/AEX	AE maximum intensity	Related to study drug/protocol-required procedure	Onset date/stop date/study day	AE Duration (days)	AE related study drug action	AE related drug treatment/non-drug treatment	AE outcome
140202/ 27/female/Caucasian	Pregnancy, puerperium and perinatal conditions/Abortion spontaneous/SPONTANEOUS ABORTION	mild	NO/NO	08SEP2010/08SEP2010/ 932	1	drug withdrawn	NO/NO	recovered/ resolved
140520/ 30/female/Caucasian	Injury, poisoning and procedural complications/Forearm fracture/LEFT LOWER ARM FRACTURE	severe	NO/NO	28JUN2008/24NOV2008/ 53	150	dose not changed	YES/YES	recovered/ resolved with resid. effects
140807/ 33/female/Caucasian	Vascular disorders/Deep vein thrombosis/LEFT LEG DEEP VEIN THROMBOSIS	severe	NO/NO	14NOV2008/28NOV2008/ 201	15	drug withdrawn	YES/NO	recovered/ resolved
150111/ 30/female/Caucasian	Infections and infestations/Appendicitis/ACUTE APPENDICITIS	severe	NO/NO	21MAR2008/22MAR2008/ 45	2	dose not changed	YES/YES	recovered/ resolved
150123/ 22/female/Caucasian	Infections and infestations/Tonsillitis/TONSILLITIS	moderate	NO/NO	23MAY2008/11JUN2008/ 115	20	dose not changed	YES/YES	recovered/ resolved

Table 14.3.2 / 2: Serious adverse events - Subject listing (FAS)

TREATMENT: LCS12

Subject/ age/sex/race	Primary SOC/preferred term/AEX	AE maximum intensity	Related to study drug/protocol-required procedure	Onset date/stop date/study day	AE Duration (days)	AE related study drug action	AE related drug treatment/non-drug treatment	AE outcome
150138/ 33/female/Caucasian	Gastrointestinal disorders/Irritable bowel syndrome/IRRITABLE COLON CRISIS	severe	NO/NO	21NOV2009/24NOV2009/ 656	4	dose not changed	YES/NO	recovered/ resolved
160317/ 26/female/Caucasian	Neoplasms benign, malignant and unspecified (incl cysts and polyps)/Ovarian germ cell teratoma benign/DERMOID CYST (RIGHT OVARY)	moderate	NO/NO	16AUG2010/25OCT2010/ 939	71	dose not changed	YES/YES	recovered/ resolved
160329/ 21/female/Caucasian	Infections and infestations/Meningitis/MENINGITIS POST OPERATIVE	moderate	NO/NO	25MAR2008/15APR2008/ 51	22	dose not changed	YES/NO	recovered/ resolved
	Injury, poisoning and procedural complications/Subdural haematoma/HEMATOMA SUBDURALE POSTOPERATIVE	severe	NO/NO	19MAR2008/15APR2008/ 45	28	dose not changed	NO/YES	recovered/ resolved

Table 14.3.2 / 2: Serious adverse events - Subject listing (FAS)

TREATMENT: LCS12

Subject/ age/sex/r ace	Primary SOC/preferred term/AEX	AE maximum intensity	Related to study drug/protocol-required procedure	Onset date/stop date/study day	AE Duration (days)	AE related study drug action	AE related drug treatment/non -drug treatment	AE outcome
	Neoplasms benign, malignant and unspecified (incl cysts and polyps)/Astrocytoma, low grade/PILOCYTIC ASTROCYTOMA GR 1	severe	NO/NO	13MAR2008/15APR2008/ 39	34	dose not changed	YES/YES	recovered/ resolved
160416/ 26/female/Caucasian	Infections and infestations/Appendicitis/APPENDICITIS	moderate	NO/NO	27AUG2008/10SEP2008/ 293	15	dose not changed	YES/YES	recovered/ resolved
160427/ 29/female/Caucasian	Gastrointestinal disorders/Abdominal pain/DOLORES ABDOMINI NUD=ABDOMINAL PAIN	moderate	NO/NO	04JAN2010/05JAN2010/ 725	2	dose not changed	YES/YES	recovered/ resolved
160519/ 31/female/Caucasian	Neoplasms benign, malignant and unspecified (incl cysts and polyps)/Thyroid cancer/CARSINOMA (CANCER) THYROIDEA	severe	NO/NO	13SEP2010/16SEP2010/ 1079	4	dose not changed	YES/YES	recovered/ resolved with resid. effects
160576/ 31/female/Caucasian	Respiratory, thoracic and mediastinal disorders/Asthma/ WORSENING OF ASTHMA	moderate	NO/NO	11FEB2011/21FEB2011/ 985	11	dose not changed	YES/YES	recovered/ resolved

Table 14.3.2 / 2: Serious adverse events - Subject listing (FAS)

TREATMENT: LCS12

Subject/ age/sex/race	Primary SOC/preferred term/AEX	AE maximum intensity	Related to study drug/protocol-required procedure	Onset date/stop date/study day	AE Duration (days)	AE related study drug action	AE related drug treatment/non-drug treatment	AE outcome
160613/ 34/female/Caucasian	Gastrointestinal disorders/Abdominal hernia/HERNIA VENTRALIS	moderate	NO/NO	17AUG2010/18AUG2010/ 978	2	dose not changed	YES/NO	recovered/ resolved
160743/ 18/female/Caucasian	Pregnancy, puerperium and perinatal conditions/Ectopic pregnancy/EXTRA UTERINE PREGNANCY	mild	YES/NO	23APR2008/05MAY2008/ 97	13	drug withdrawn	NO/YES	recovered/ resolved
160757/ 24/female/Caucasian	Infections and infestations/Gastroenteritis/GASTRO ENTERITIS	moderate	NO/NO	21OCT2008/ ./ 245	.	dose not changed	YES/YES	continuing with change
160803/ 23/female/Caucasian	Injury, poisoning and procedural complications/Joint sprain/DISTORSION OF LEFT KNEE	moderate	NO/NO	20AUG2009/ ./ 540	.	dose not changed	YES/YES	continuing with change
160904/ 21/female/Caucasian	Infections and infestations/Peritonsillar abscess/PERITONSILLARY ABSCESS	moderate	NO/NO	24OCT2008/06NOV2008/ 354	14	dose not changed	YES/YES	recovered/ resolved
160973/ 30/female/Caucasian	Infections and infestations/Appendicitis/ACUTE APPENDICITIS	moderate	NO/NO	02NOV2009/15NOV2009/ 687	14	dose not changed	YES/YES	recovered/ resolved

Table 14.3.2 / 2: Serious adverse events - Subject listing (FAS)

TREATMENT: LCS12

Subject/ age/sex/r ace	Primary SOC/preferred term/AEX	AE maximum intensity	Related to study drug/protocol-required procedure	Onset date/stop date/study day	AE Duration (days)	AE related study drug action	AE related drug treatment/non -drug treatment	AE outcome
	Infections and infestations/Peritoneal abscess/PERITONEAL ABSCESS	moderate	NO/NO	02NOV2009/15NOV2009/ 687	14	dose not changed	YES/YES	recovered/ resolved
	Reproductive system and breast disorders/Endometriosis/ENDOMETRIOSIS IN APPENDIX	moderate	NO/NO	02NOV2009/15NOV2009/ 687	14	dose not changed	YES/YES	recovered/ resolved
161003/ 19/female/Caucasian	Infections and infestations/Pneumonia/PNEUMONIA	moderate	NO/NO	23JAN2009/25JAN2009/ 478	3	dose not changed	YES/NO	recovered/ resolved
161027/ 22/female/Caucasian	Reproductive system and breast disorders/Haemorrhagic ovarian cyst/HAEMORRHAGIC CYST IN THE OVARY	moderate	YES/NO	20SEP2008/ ./ 293	.	dose not changed	YES/NO	continuing with change
		moderate	YES/NO	14DEC2008/ ./ 378	.	dose not changed	YES/NO	continuing with change

Table 14.3.2 / 2: Serious adverse events - Subject listing (FAS)

TREATMENT: LCS12

Subject/ age/sex/race	Primary SOC/preferred term/AEX	AE maximum intensity	Related to study drug/protocol-required procedure	Onset date/stop date/study day	AE Duration (days)	AE related study drug action	AE related drug treatment/non-drug treatment	AE outcome
161114/ 23/female/Caucasian	Neoplasms benign, malignant and unspecified (incl cysts and polyps)/Ovarian germ cell teratoma benign/LAPAROSCOPY DUE TO SUSPICION OF DERMOID CYST IN LEFT OVARY	moderate	NO/NO	14SEP2010/15SEP2010/ 1042	2	dose not changed	NO/NO	recovered/ resolved
161416/ 22/female/Caucasian	Reproductive system and breast disorders/Ovarian cyst ruptured/RUPTURED OVARIAN CYST	severe	YES/NO	06JAN2008/28JAN2008/ 39	23	dose not changed	NO/YES	recovered/ resolved
161512/ 18/female/Caucasian	Infections and infestations/Pyelonephritis acute/ACUTE PYELONEPHRITIS	severe	NO/NO	08MAR2008/17MAR2008/ 69	10	dose not changed	YES/NO	recovered/ resolved
161525/ 19/female/Caucasian	Infections and infestations/Genital herpes/HERPES GENITALIS	severe	NO/NO	04AUG2008/18AUG2008/ 197	15	drug withdrawn	YES/NO	recovered/ resolved
180116/ 30/female/Caucasian	Infections and infestations/Pelvic inflammatory disease/PID	moderate	YES/NO	14APR2010/20JUL2010/ 855	98	drug withdrawn	YES/YES	recovered/ resolved

Table 14.3.2 / 2: Serious adverse events - Subject listing (FAS)

TREATMENT: LCS12

Subject/ age/sex/race	Primary SOC/preferred term/AEX	AE maximum intensity	Related to study drug/protocol-required procedure	Onset date/stop date/study day	AE Duration (days)	AE related study drug action	AE related drug treatment/non-drug treatment	AE outcome
180420/ 35/female/Caucasian	Infections and infestations/Pneumonia/PNEUMONIA	severe	NO/NO	12DEC2010/28JAN2011/ 1074	48	dose not changed	YES/NO	recovered/ resolved
180501/ 31/female/Caucasian	Eye disorders/Iridocyclitis/IRIDOCYCLITIS	moderate	NO/NO	03JAN2008/14NOV2008/ 29	317	drug withdrawn	YES/NO	recovered/ resolved
180642/ 31/female/Caucasian	Injury, poisoning and procedural complications/Meniscus lesion/LESION OF LATERAL MENISC OF LEFT KNEE	moderate	NO/NO	21JUL2008/24JUL2008/ 227	4	dose not changed	YES/YES	recovered/ resolved
	Musculoskeletal and connective tissue disorders/Synovitis/SYNOVITIS OF LEFT KNEE	severe	NO/NO	21JUL2008/24JUL2008/ 227	4	dose not changed	YES/YES	recovered/ resolved
190636/ 23/female/Hispanic	General disorders and administration site conditions/Pyrexia/FEVER (38.5 DEGREE CELSIUS)	mild	NO/NO	22SEP2009/22SEP2009/ 492	1	dose not changed	YES/NO	recovered/ resolved

Table 14.3.2 / 2: Serious adverse events - Subject listing (FAS)

TREATMENT: LCS12

Subject/ age/sex/race	Primary SOC/preferred term/AEX	AE maximum intensity	Related to study drug/protocol-required procedure	Onset date/stop date/study day	AE Duration (days)	AE related study drug action	AE related drug treatment/non-drug treatment	AE outcome
	Pregnancy, puerperium and perinatal conditions/Abortion spontaneous/EARLY PREGNANCY LOSS	moderate	YES/NO	22SEP2009/24SEP2009/ 492	3	drug withdrawn	YES/YES	recovered/ resolved
	Pregnancy, puerperium and perinatal conditions/Premature separation of placenta/PLACENTAL ABRUPTION IN EARLY PREGNANCY	moderate	YES/NO	22SEP2009/24SEP2009/ 492	3	drug withdrawn	NO/YES	recovered/ resolved
200219/ 18/female/Caucasian	Reproductive system and breast disorders/Ovarian cyst/OVARIAN CYST RIGHT SIDE	severe	NO/NO	10MAR2009/10MAR2009/ 373	1	dose not changed	NO/YES	recovered/ resolved
200908/ 28/female/Caucasian	Gastrointestinal disorders/Abdominal adhesions/ADHESIONS INTRA ABDOMINAL	severe	NO/NO	10AUG2009/12AUG2009/ 574	3	dose not changed	YES/NO	recovered/ resolved

Table 14.3.2 / 2: Serious adverse events - Subject listing (FAS)

TREATMENT: LCS12

Subject/ age/sex/race	Primary SOC/preferred term/AEX	AE maximum intensity	Related to study drug/protocol-required procedure	Onset date/stop date/study day	AE Duration (days)	AE related study drug action	AE related drug treatment/non-drug treatment	AE outcome
	Gastrointestinal disorders/Abdominal pain/ABDOMINAL PAIN	severe	NO/NO	26FEB2008/28FEB2008/ 43	3	dose not changed	NO/NO	recovered/ resolved
	Gastrointestinal disorders/Constipation/OBSTIPATION	severe	NO/NO	26FEB2008/28FEB2008/ 43	3	dose not changed	YES/NO	recovered/ resolved
210203/ 28/female/Caucasian	Gastrointestinal disorders/Abdominal pain/ABDOMINAL PAIN	moderate	NO/NO	11MAR2010/13MAR2010/ 820	3	dose not changed	NO/NO	recovered/ resolved
210411/ 28/female/Caucasian	Reproductive system and breast disorders/Haemorrhagic ovarian cyst/CORPUS LUTEUM HEMORRHAGE (RIGHT OVARY)	severe	NO/NO	11DEC2008/11DEC2008/ 304	1	dose not changed	NO/YES	recovered/ resolved
230201/ 34/female/Caucasian	Hepatobiliary disorders/Cholecystitis/CHOLECYSTITIS	severe	NO/NO	13NOV2009/19NOV2009/ 768	7	dose not changed	YES/YES	recovered/ resolved with resid. effects
230303/ 20/female/Caucasian	Pregnancy, puerperium and perinatal conditions/Ectopic pregnancy/ECTOPIC PREGNANCY	mild	NO/NO	15JUN2009/18JUN2009/ 519	4	drug withdrawn	YES/YES	recovered/ resolved

Table 14.3.2 / 2: Serious adverse events - Subject listing (FAS)

TREATMENT: LCS12

Subject/ age/sex/race	Primary SOC/preferred term/AEX	AE maximum intensity	Related to study drug/protocol-required procedure	Onset date/stop date/study day	AE Duration (days)	AE related study drug action	AE related drug treatment/non-drug treatment	AE outcome
230408/ 31/female/Caucasian	Infections and infestations/Vestibular neuronitis/VESTIBULERIS NEURONITIS	severe	NO/NO	10MAR2008/09DEC2008/ 85	275	dose not changed	YES/NO	recovered/ resolved
230501/ 35/female/Hispanic	Investigations/Weight increased/WEIGHT GAIN	severe	NO/NO	14APR2010/28APR2010/ 850	15	dose not changed	YES/YES	recovered/ resolved
	Renal and urinary disorders/Strangury/STRANGURY	moderate	NO/NO	29OCT2008/30OCT2008/ 318	2	dose not changed	YES/YES	recovered/ resolved
230615/ 19/female/Caucasian	Reproductive system and breast disorders/Uterine spasm/UTERINE CRAMPS	severe	NO/YES	31JAN2008/05FEB2008/ 1	6	drug withdrawn	YES/NO	recovered/ resolved
230720/ 33/female/Caucasian	Injury, poisoning and procedural complications/Overdose/MEDICATION OVERDOSE	severe	NO/NO	14JAN2011/17JAN2011/ 1045	4	dose not changed	YES/YES	recovering/ resolving
	Psychiatric disorders/Anxiety/ANXIETY	severe	NO/NO	21OCT2009/19NOV2009/ 595	30	dose not changed	YES/NO	continuing with change
		severe	NO/NO	22FEB2010/13APR2010/ 719	51	dose not changed	YES/NO	continuing with change
	Psychiatric disorders/Anxiety/WORSENING OF ANXIETY	severe	NO/NO	16JUL2009/29JUL2009/ 498	14	dose not changed	YES/NO	continuing with change

Table 14.3.2 / 2: Serious adverse events - Subject listing (FAS)

TREATMENT: LCS12

Subject/ age/sex/r ace	Primary SOC/preferred term/AEX	AE maximum intensity	Related to study drug/protocol-required procedure	Onset date/stop date/study day	AE Duration (days)	AE related study drug action	AE related drug treatment/non -drug treatment	AE outcome
	Psychiatric disorders/Depression/DEPRESSION	severe	NO/NO	16JUL2009/29JUL2009/ 498	14	dose not changed	YES/NO	continuing with change
		severe	NO/NO	21OCT2009/19NOV2009/ 595	30	dose not changed	YES/NO	continuing with change
		severe	NO/NO	22FEB2010/13APR2010/ 719	51	dose not changed	YES/NO	continuing with change
	Psychiatric disorders/Depression/WORSENING OF DEPRESSION	severe	NO/NO	16JUL2009/ ./ 498	.	dose not changed	YES/NO	not recovered/ not resolved
240209/ 23/female/other	Gastrointestinal disorders/Crohn's disease/CROHN'S DISEASE	severe	NO/NO	25JUL2008/ ./ 233	.	dose not changed	YES/YES	continuing with change
	Gastrointestinal disorders/Impaired gastric emptying/GASTROPARESIS	severe	NO/NO	25JUL2008/ ./ 233	.	dose not changed	YES/YES	continuing with change
	Renal and urinary disorders/Haematuria/HEMATURIA	severe	NO/NO	25JUL2008/31JUL2008/ 233	7	dose not changed	NO/NO	recovered/ resolved
240215/ 26/female/Caucasian	Hepatobiliary disorders/Cholecystitis/CHOLECYSTITIS	severe	NO/NO	14NOV2008/16NOV2008/ 354	3	dose not changed	NO/YES	recovered/ resolved
	Hepatobiliary disorders/Gallbladder polyp/GALLBLADDER POLYP	moderate	NO/NO	14NOV2008/16NOV2008/ 354	3	dose not changed	NO/YES	recovered/ resolved

Table 14.3.2 / 2: Serious adverse events - Subject listing (FAS)

TREATMENT: LCS12

Subject/ age/sex/race	Primary SOC/preferred term/AEX	AE maximum intensity	Related to study drug/protocol-required procedure	Onset date/stop date/study day	AE Duration (days)	AE related study drug action	AE related drug treatment/non-drug treatment	AE outcome
240510/ 28/female/Caucasian	Respiratory, thoracic and mediastinal disorders/Pneumothorax/MULTIPLE RECURRENT SPONTANEOUS LEFT SIDED PNEUMOTHORACES	moderate	NO/NO	02SEP2009/04SEP2009/ 560	3	dose not changed	YES/YES	recovered/ resolved
240521/ 23/female/Caucasian	Infections and infestations/Diverticulitis/ACUTE DIVERTICULITIS	severe	NO/NO	05FEB2009/30MAR2009/ 283	54	dose not changed	YES/NO	recovered/ resolved
240841/ 33/female/Caucasian	Gastrointestinal disorders/Abdominal pain/ABDOMINAL PAIN	moderate	NO/NO	20AUG2008/03NOV2008/ 140	76	drug withdrawn	NO/YES	recovered/ resolved
	Neoplasms benign, malignant and unspecified (incl cysts and polyps)/Uterine leiomyoma/ENDOMETRIAL LEIOMYOMA	moderate	NO/NO	20AUG2008/04NOV2008/ 140	77	drug withdrawn	NO/YES	recovered/ resolved
241106/ 29/female/Caucasian	Infections and infestations/Appendicitis/APPENDICITIS	severe	NO/NO	31AUG2008/02SEP2008/ 314	3	dose not changed	YES/YES	recovered/ resolved

Table 14.3.2 / 2: Serious adverse events - Subject listing (FAS)

TREATMENT: LCS12

Subject/ age/sex/race	Primary SOC/preferred term/AEX	AE maximum intensity	Related to study drug/protocol-required procedure	Onset date/stop date/study day	AE Duration (days)	AE related study drug action	AE related drug treatment/non-drug treatment	AE outcome
241153/ 32/female/Caucasian	Neoplasms benign, malignant and unspecified (incl cysts and polyps)/Thyroid cancer/THYROID CANCER	severe	NO/NO	15AUG2009/ ./ 542	.	dose not changed	YES/YES	UNK
241172/ 35/female/Caucasian	Endocrine disorders/Goitre/ENLARGED THYROID	severe	NO/NO	15MAY2009/22MAY2009/ 452	8	dose not changed	YES/YES	recovered/ resolved
	Infections and infestations/Appendicitis/APPENDICITIS	severe	NO/NO	15OCT2008/16OCT2008/ 240	2	dose not changed	YES/YES	recovered/ resolved
241410/ 27/female/Caucasian	Gastrointestinal disorders/Abdominal pain/ABDOMINAL PAIN	severe	YES/NO	16JAN2008/01MAY2008/ 1	107	drug withdrawn	YES/YES	recovered/ resolved
	Infections and infestations/Pyelonephritis/POSSIBLE PYELONEPHRITIS	moderate	NO/NO	29APR2008/ ./ 105	.	drug withdrawn	YES/YES	not recovered/ not resolved
	Infections and infestations/Urinary tract infection/URINARY TRACT INFECTION	severe	NO/NO	29APR2008/10MAY2008/ 105	12	drug withdrawn	YES/YES	recovered/ resolved

Table 14.3.2 / 2: Serious adverse events - Subject listing (FAS)

TREATMENT: LCS12

Subject/ age/sex/race	Primary SOC/preferred term/AEX	AE maximum intensity	Related to study drug/protocol-required procedure	Onset date/stop date/study day	AE Duration (days)	AE related study drug action	AE related drug treatment/non-drug treatment	AE outcome
241536/ 28/female/other	Hepatobiliary disorders/Cholecystitis chronic/CHRONIC CHOLECYSTITIS MILD	mild	NO/NO	14MAY2008/22MAY2008/ 52	9	dose not changed	NO/YES	recovered/ resolved
	Infections and infestations/Cellulitis/RIGHT ABDOMINAL WALL CELLULITIS	moderate	NO/NO	04DEC2009/ ./ 621	.	dose not changed	YES/NO	continuing with change
		moderate	NO/NO	14DEC2009/ ./ 631	.	dose not changed	YES/NO	continuing with change
241708/ 24/female/Caucasian	Surgical and medical procedures/Detoxification/DETOXIFICATION	severe	NO/NO	01MAR2010/ ./ 788	.	dose not changed	YES/YES	not recovered/ not resolved
242212/ 27/female/Hispanic	Infections and infestations/Enterocolitis infectious/INFECTIOUS COLITIS	moderate	NO/NO	01AUG2009/ ./ 508	.	dose not changed	YES/YES	continuing with change
242320/ 23/female/Hispanic	General disorders and administration site conditions/Chest pain/ATYPICAL CHEST PAINS-BILATERAL	severe	NO/NO	04JAN2011/04JAN2011/ 1036	1	dose not changed	YES/NO	recovered/ resolved

Table 14.3.2 / 2: Serious adverse events - Subject listing (FAS)

TREATMENT: LCS12

Subject/ age/sex/race	Primary SOC/preferred term/AEX	AE maximum intensity	Related to study drug/protocol-required procedure	Onset date/stop date/study day	AE Duration (days)	AE related study drug action	AE related drug treatment/non-drug treatment	AE outcome
	Vascular disorders/Hypertension/HYPERTENSION	severe	NO/NO	04JAN2011/ ./ 1036	.	dose not changed	YES/NO	continuing with change
242807/ 21/female/Caucasian	Nervous system disorders/Status migrainosus/STATUS MIGRAINOUSUS	severe	NO/NO	24MAR2008/27MAR2008/ 11	4	dose not changed	YES/NO	recovered/ resolved
243938/ 20/female/Caucasian	Blood and lymphatic system disorders/Spherocytic anaemia/ANEMIA DUE TO SPHEROCYTOSIS	severe	NO/NO	22MAY2008/24MAY2008/ 56	3	dose not changed	YES/NO	recovered/ resolved
	Blood and lymphatic system disorders/Splenomegaly/SPLEENOMEGALY	moderate	NO/NO	23JUL2008/29JUL2008/ 118	7	dose not changed	YES/YES	recovered/ resolved
	Blood and lymphatic system disorders/Splenomegaly/SPLENOMEGALY	mild	NO/NO	22MAY2008/ ./ 56	.	dose not changed	YES/NO	continuing with change
	Hepatobiliary disorders/Cholelithiasis/CHOLELITHIASIS	mild	NO/NO	22MAY2008/ ./ 56	.	dose not changed	YES/NO	continuing with change
		mild	NO/NO	23JUL2008/29JUL2008/ 118	7	dose not changed	YES/YES	recovered/ resolved

Table 14.3.2 / 2: Serious adverse events - Subject listing (FAS)

TREATMENT: LCS12

Subject/ age/sex/race	Primary SOC/preferred term/AEX	AE maximum intensity	Related to study drug/protocol-required procedure	Onset date/stop date/study day	AE Duration (days)	AE related study drug action	AE related drug treatment/non-drug treatment	AE outcome
244422/ 20/female/Caucasian	Psychiatric disorders/Depression suicidal/DEPRESSION WITH SUICIDAL TENDANCIES	severe	NO/NO	04APR2009/ ./ 396	.	dose not changed	YES/NO	continuing with change
	Psychiatric disorders/Suicide attempt/SUICIDE ATTEMPT	severe	NO/NO	04APR2009/04APR2009/ 396	1	dose not changed	YES/NO	recovered/ resolved
244438/ 25/female/Caucasian	Infections and infestations/Pelvic inflammatory disease/PELVIC-INFLAMMATORY DISEASE.	severe	YES/YES	30APR2008/ ./ 27	.	drug withdrawn	YES/NO	continuing with change
	Infections and infestations/Tubo-ovarian abscess/TUBAL OVARIAN ABSCESS	severe	YES/YES	30APR2008/ ./ 27	.	drug withdrawn	YES/NO	continuing with change
244602/ 33/female/Black	Infections and infestations/Streptococcal sepsis/STREPTOCOCCAL SEPSIS SYNDROME	severe	NO/NO	03SEP2009/15SEP2009/ 519	13	dose not changed	YES/NO	recovered/ resolved

Table 14.3.2 / 2: Serious adverse events - Subject listing (FAS)

TREATMENT: LCS12

Subject/ age/sex/race	Primary SOC/preferred term/AEX	AE maximum intensity	Related to study drug/protocol-required procedure	Onset date/stop date/study day	AE Duration (days)	AE related study drug action	AE related drug treatment/non-drug treatment	AE outcome
	Infections and infestations/Upper respiratory tract infection/UPPER RESPIRATORY STEP INFECTION	severe	NO/NO	03SEP2009/15SEP2009/ 519	13	dose not changed	NO/NO	recovered/ resolved
245003/ 27/female/Caucasian	Pregnancy, puerperium and perinatal conditions/Abortion spontaneous/SPONTANEOUS ABORTION	severe	NO/NO	23NOV2008/23NOV2008/ 322	1	.	NO/NO	recovered/ resolved
245431/ 24/female/Hispanic	Infections and infestations/Herpes dermatitis/HERPES SIMPLEX VIRUS INFECTION ALL OVER BODY	moderate	NO/NO	16JUN2010/28JUN2010/ 749	13	dose not changed	YES/NO	recovered/ resolved
	Skin and subcutaneous tissue disorders/Erythema multiforme/ERYTHEMA MULTIFORME	moderate	NO/NO	16JUN2010/28JUN2010/ 749	13	dose not changed	YES/NO	recovered/ resolved
245503/ 32/female/Hispanic	Renal and urinary disorders/Stress urinary incontinence/STRESS INCONTINENCE	severe	NO/NO	01NOV2010/01NOV2010/ 1023	1	drug withdrawn	NO/YES	recovered/ resolved

Table 14.3.2 / 2: Serious adverse events - Subject listing (FAS)

TREATMENT: LCS12

Subject/ age/sex/r ace	Primary SOC/preferred term/AEX	AE maximum intensity	Related to study drug/protocol-required procedure	Onset date/stop date/study day	AE Duration (days)	AE related study drug action	AE related drug treatment/non -drug treatment	AE outcome
	Reproductive system and breast disorders/Uterine prolapse/UTERINE PROLAPSE	severe	NO/NO	01NOV2010/01NOV2010/ 1023	1	drug withdrawn	NO/YES	recovered/ resolved
245701/ 24/female/Caucasian	Immune system disorders/Drug hypersensitivity/ALLERGIC REACTION TO AMOXICILLIN	moderate	NO/NO	05JAN2011/05JAN2011/ 1039	1	dose not changed	YES/YES	recovered/ resolved
246212/ 20/female/Caucasian	Neoplasms benign, malignant and unspecified (incl cysts and polyps)/Acute leukaemia/ACUTE LEUKEMIA	severe	NO/NO	21NOV2010/ ./ 929	.	.	YES/NO	not recovered/ not resolved

Note: AEX=adverse event verbatim investigator term. SOC=System Organ Class.
Note: Study day is calculated from the first day of study drug administration. The formula for this calculation is: (onset day of AE minus medication day) plus 1 day.
Note: Dictionary/ MedDRA Version 14.0
Global Biostatistics: /by-sasp/patdb/projects/de04209/310442/stat/prod_interim03/pgms/t-sae.sas epkll 12OCT2011 11:27

Table 14.3.2 / 2: Serious adverse events - Subject listing (FAS) (cont.)

TREATMENT: LCS16

Subject/ age/sex/race	Primary SOC/preferred term/AEX	AE maximum intensity	Related to study drug/protocol-required procedure	Onset date/stop date/study day	AE Duration (days)	AE related study drug action	AE related drug treatment/non-drug treatment	AE outcome
120404/ 29/female/Caucasian	Gastrointestinal disorders/Umbilical hernia/UMBILICAL HERNIA	mild	NO/NO	14SEP2010/01OCT2010/ 837	18	dose not changed	YES/YES	recovered/ resolved
120607/ 33/female/Hispanic	Gastrointestinal disorders/Gastritis/ ACUTE GASTRITIS	severe	NO/NO	30OCT2010/01NOV2010/ 964	3	dose not changed	YES/NO	recovered/ resolved
140702/ 21/female/Caucasian	Injury, poisoning and procedural complications/Ankle fracture/FRACTURED RIGHT ANKLE	moderate	NO/NO	11JUL2009/13JUL2009/ 494	3	dose not changed	YES/YES	recovered/ resolved
140704/ 24/female/Caucasian	Musculoskeletal and connective tissue disorders/Joint instability/INSTABILITY LEFT SHOULDER	moderate	NO/NO	01FEB2011/ ./ 1055	.	dose not changed	YES/YES	recovering/ resolving
140805/ 24/female/Caucasian	Psychiatric disorders/Anxiety/ WORSENING ANXIETY	severe	NO/NO	11MAY2010/ ./ 813	.	dose not changed	YES/NO	continuing with change
141023/ 27/female/Caucasian	Infections and infestations/Pyelonephritis/PYELONEPHRITIS	severe	NO/NO	06AUG2008/09AUG2008/ 101	4	drug withdrawn	YES/NO	recovered/ resolved

Table 14.3.2 / 2: Serious adverse events - Subject listing (FAS) (cont.)

TREATMENT: LCS16

Subject/ age/sex/race	Primary SOC/preferred term/AEX	AE maximum intensity	Related to study drug/protocol-required procedure	Onset date/stop date/study day	AE Duration (days)	AE related study drug action	AE related drug treatment/non-drug treatment	AE outcome
141407/ 28/female/Caucasian	Infections and infestations/Pelvic inflammatory disease/PELVIC INFLAMMATORY DISEASE	moderate	YES/NO	04APR2008/11APR2008/ 17	8	drug withdrawn	YES/NO	recovered/ resolved
141413/ 21/female/Caucasian	Metabolism and nutrition disorders/Diabetic ketoacidosis/DIABETIC KETOACIDOSIS - WORSENING OF	moderate	NO/NO	10JAN2010/13JAN2010/ 655	4	dose not changed	YES/YES	recovered/ resolved
141415/ 21/female/other	Infections and infestations/Appendicitis/APPENDICITIS	moderate	NO/NO	21DEC2009/24DEC2009/ 623	4	dose not changed	YES/YES	recovered/ resolved
160410/ 35/female/Caucasian	Infections and infestations/Pelvic inflammatory disease/PID	severe	YES/YES	20DEC2007/24JAN2008/ 2	36	drug withdrawn	YES/NO	recovered/ resolved
160419/ 28/female/Caucasian	Musculoskeletal and connective tissue disorders/Loose body in joint/CORPUS LIBERA GENUS L DX	moderate	NO/NO	22JUN2009/23JUN2009/ 599	2	dose not changed	NO/YES	recovered/ resolved with resid. effects

Table 14.3.2 / 2: Serious adverse events - Subject listing (FAS) (cont.)

TREATMENT: LCS16

Subject/ age/sex/race	Primary SOC/preferred term/AEX	AE maximum intensity	Related to study drug/protocol-required procedure	Onset date/stop date/study day	AE Duration (days)	AE related study drug action	AE related drug treatment/non-drug treatment	AE outcome
160423/ 26/female/Caucasian	Pregnancy, puerperium and perinatal conditions/Ectopic pregnancy/ECTOPIC PREGNANCY	moderate	YES/YES	09OCT2008/28NOV2008/ 297	51	drug withdrawn	NO/YES	recovered/ resolved
160438/ 34/female/Caucasian	Infections and infestations/Infection/INFECTION IN BOTH HANDS	moderate	NO/NO	14JUL2009/18AUG2009/ 537	36	dose not changed	YES/NO	recovered/ resolved
160556/ 33/female/Caucasian	Musculoskeletal and connective tissue disorders/Axillary mass/CLUMP IN THE RIGHT ARMBIT OPERATED	moderate	NO/NO	13JAN2009/15JAN2009/ 450	3	dose not changed	NO/YES	recovered/ resolved
160919/ 33/female/Caucasian	Infections and infestations/Peritonsillar abscess/PERITONSILLARY ABSCESS	severe	NO/NO	08JUN2008/10JUN2008/ 229	3	dose not changed	YES/YES	recovered/ resolved
160927/ 33/female/Caucasian	Pregnancy, puerperium and perinatal conditions/Blighted ovum/BLIGHTED OVUM	severe	NO/NO	05JAN2010/11FEB2010/ 807	38	drug withdrawn	YES/YES	recovered/ resolved

Table 14.3.2 / 2: Serious adverse events - Subject listing (FAS) (cont.)

TREATMENT: LCS16

Subject/ age/sex/race	Primary SOC/preferred term/AEX	AE maximum intensity	Related to study drug/protocol-required procedure	Onset date/stop date/study day	AE Duration (days)	AE related study drug action	AE related drug treatment/non-drug treatment	AE outcome
160978/ 26/female/Caucasian	General disorders and administration site conditions/Device dislocation/PARTIAL PERFORATION (MYOMETRIUM)	moderate	YES/NO	07DEC2009/13JAN2010/ 722	38	drug withdrawn	NO/NO	recovered/ resolved
161024/ 34/female/Caucasian	Infections and infestations/Pelvic inflammatory disease/PELVIC INFLAMMATORY DISEASE	severe	YES/YES	26NOV2007/10DEC2007/ 1	15	drug withdrawn	YES/NO	recovered/ resolved
161123/ 23/female/Caucasian	Ear and labyrinth disorders/Vertigo/ VERTIGO	severe	NO/NO	18DEC2009/01JAN2010/ 758	15	dose not changed	YES/NO	recovered/ resolved
161223/ 28/female/Caucasian	Injury, poisoning and procedural complications/Tendon rupture/ACHILLES TEND. RUPTURE IN LEFT. LEG	severe	NO/NO	18OCT2008/01DEC2008/ 237	45	dose not changed	YES/YES	recovered/ resolved
	Nervous system disorders/Dizziness /DIZZINESS	moderate	NO/NO	27JUL2010/15OCT2010 / 884	81	dose not changed	NO/NO	recovered/ resolved

Table 14.3.2 / 2: Serious adverse events - Subject listing (FAS) (cont.)

TREATMENT: LCS16

Subject/ age/sex/race	Primary SOC/preferred term/AEX	AE maximum intensity	Related to study drug/protocol-required procedure	Onset date/stop date/study day	AE Duration (days)	AE related study drug action	AE related drug treatment/non-drug treatment	AE outcome
161306/ 27/female/Caucasian	Injury, poisoning and procedural complications/Thermal burn/BURN INJURY IN BOTH TIGHTS	severe	NO/NO	02JAN2009/19JAN2009 / 460	18	dose not changed	YES/YES	recovered/ resolved
161432/ 22/female/Caucasian	Psychiatric disorders/Depression/DEPRESSION, WORSENED	severe	NO/NO	10OCT2010/ ./ 971	.	dose not changed	YES/YES	not recovered/ not resolved
180112/ 33/female/Caucasian	Gastrointestinal disorders/Abdominal pain/ABDOMINAL PAIN	moderate	YES/YES	01JUN2009/04JUN2009 / 546	4	drug withdrawn	NO/NO	recovered/ resolved
	Pregnancy, puerperium and perinatal conditions/Abortion spontaneous incomplete/INCOMPLETE SPONTANEUS ABORTION	moderate	YES/YES	01JUN2009/04JUN2009 / 546	4	drug withdrawn	YES/NO	recovered/ resolved
180117/ 33/female/Caucasian	Gastrointestinal disorders/Abdominal pain/ABDOMINAL PAIN	moderate	NO/NO	14JUL2010/11AUG2010/ 929	29	dose not changed	NO/YES	recovered/ resolved

Table 14.3.2 / 2: Serious adverse events - Subject listing (FAS) (cont.)

TREATMENT: LCS16

Subject/ age/sex/race	Primary SOC/preferred term/AEX	AE maximum intensity	Related to study drug/protocol-required procedure	Onset date/stop date/study day	AE Duration (days)	AE related study drug action	AE related drug treatment/non-drug treatment	AE outcome
	Reproductive system and breast disorders/Pelvic adhesions/PELVIC ADHESIONS	moderate	NO/NO	14JUL2010/11AUG2010/ 929	29	dose not changed	NO/YES	recovered/ resolved
180317/ 33/female/Caucasian	Pregnancy, puerperium and perinatal conditions/Ectopic pregnancy/LEFT TUBAL EXTRAUTERIN PREGNANCY	severe	YES/YES	03JAN2009/05JAN2009/ 409	3	drug withdrawn	NO/YES	recovered/ resolved
180422/ 33/female/Caucasian	Neoplasms benign, malignant and unspecified (incl cysts and polyps)/Ovarian germ cell teratoma benign/WORSENING OF PRE-EXISTING DERMOID CYST OF THE RIGHT OVARY	mild	NO/NO	15JAN2008/19JAN2008/ 37	5	dose not changed	NO/YES	recovered/ resolved
180521/ 35/female/Caucasian	Reproductive system and breast disorders/Vaginal perforation/RUPTURA FORNIC VAGINAL POSTERIOR	mild	NO/NO	14DEC2009/15DEC2009/ 690	2	dose not changed	YES/YES	recovered/ resolved

Table 14.3.2 / 2: Serious adverse events - Subject listing (FAS) (cont.)

TREATMENT: LCS16

Subject/ age/sex/race	Primary SOC/preferred term/AEX	AE maximum intensity	Related to study drug/protocol-required procedure	Onset date/stop date/study day	AE Duration (days)	AE related study drug action	AE related drug treatment/non-drug treatment	AE outcome
180629/ 29/female/Caucasian	Cardiac disorders/Mitral valve prolapse/MITRAL PROLAPSE	mild	NO/NO	12MAR2008/ ./ 98	.	dose not changed	NO/NO	not recovered/ not resolved
	Cardiac disorders/Sinus tachycardia/SINUS TARCHYCARDIA	moderate	NO/NO	12MAR2008/19MAR2008/ 98	8	dose not changed	YES/NO	recovered/ resolved
	Infections and infestations/Pelvic inflammatory disease/PELVIC INFLAMMATORY DISEASE	mild	YES/YES	27MAR2008/03APR2008/ 113	8	drug withdrawn	YES/YES	recovered/ resolved
180659/ 34/female/Caucasian	Injury, poisoning and procedural complications/Foot fracture/MEDIAL SESAME BONE FRACTURE FOOT	severe	NO/NO	22SEP2008/25OCT2008/ 236	34	dose not changed	YES/NO	recovered/ resolved
	Injury, poisoning and procedural complications/Foot fracture/SESAME BONE FRACTURE LEFT LEG FOOT	severe	NO/NO	15NOV2008/05APR2009/ 290	142	dose not changed	YES/YES	recovered/ resolved

Table 14.3.2 / 2: Serious adverse events - Subject listing (FAS) (cont.)

TREATMENT: LCS16

Subject/ age/sex/race	Primary SOC/preferred term/AEX	AE maximum intensity	Related to study drug/protocol-required procedure	Onset date/stop date/study day	AE Duration (days)	AE related study drug action	AE related drug treatment/non-drug treatment	AE outcome
	Musculoskeletal and connective tissue disorders/Arthritis/ ARTHRITIS IN THE METACARPAL JOINT OF THE BIG TOE OF THE LEFT LEG	severe	NO/NO	22SEP2008/25OCT2008 / 236	34	dose not changed	YES/NO	recovered/ resolved
	Nervous system disorders/Sciatica/ LUMBOISCHIALGIA RIGHT SIDE	mild	NO/NO	22SEP2008/25OCT2008 / 236	34	dose not changed	NO/NO	recovered/ resolved
190202/ 35/female/Hispanic	Infections and infestations/Appendicitis/APPENDICITIS	severe	NO/NO	16JUN2008/18JUN2008 / 137	3	dose not changed	NO/YES	recovered/ resolved
	Reproductive system and breast disorders/Ovarian cyst torsion/TWISTED OVARIAN CYST	severe	NO/NO	16JUN2008/18JUN2008 / 137	3	dose not changed	NO/YES	recovered/ resolved
190622/ 26/female/Hispanic	Neoplasms benign, malignant and unspecified (incl cysts and polyps)/Teratoma benign/BENIGN TERATOMA	mild	NO/NO	28MAR2009/31MAR2009/ 382	4	dose not changed	NO/YES	recovered/ resolved

Table 14.3.2 / 2: Serious adverse events - Subject listing (FAS) (cont.)

TREATMENT: LCS16

Subject/ age/sex/race	Primary SOC/preferred term/AEX	AE maximum intensity	Related to study drug/protocol-required procedure	Onset date/stop date/study day	AE Duration (days)	AE related study drug action	AE related drug treatment/non-drug treatment	AE outcome
200301/ 18/female/Caucasian	Reproductive system and breast disorders/Ovarian cyst/CYSTE IN THE LEFT OVARY	severe	YES/NO	19JUL2009/20JUL2009/ 585	2	dose not changed	NO/YES	recovered/ resolved
200401/ 31/female/Caucasian	Infections and infestations/Appendicitis/APPENDICITIS ACUTA	severe	NO/NO	26AUG2009/10SEP2009/ 591	16	dose not changed	NO/YES	recovered/ resolved with resid. effects
200409/ 24/female/Caucasian	Neoplasms benign, malignant and unspecified (incl cysts and polyps)/Ovarian germ cell teratoma benign/OVARIAN TUMOR LEFT SIDE, BENIGN MATURE TERATOMA	moderate	NO/NO	10JUN2008/25JUN2008/ 93	16	dose not changed	NO/YES	recovered/ resolved
200509/ 32/female/Caucasian	Infections and infestations/Sinusitis/SINUSITIS MAXILLARIS	severe	NO/NO	13DEC2010/ ./ 1054	.	dose not changed	YES/YES	continuing with change
200609/ 32/female/Caucasian	Pregnancy, puerperium and perinatal conditions/Ectopic pregnancy/ECTOPIC MASS [ECTOPIC PREGNANCY]	severe	YES/NO	01DEC2009/21DEC2009/ 699	21	drug withdrawn	YES/YES	recovered/ resolved

Table 14.3.2 / 2: Serious adverse events - Subject listing (FAS) (cont.)

TREATMENT: LCS16

Subject/ age/sex/race	Primary SOC/preferred term/AEX	AE maximum intensity	Related to study drug/protocol-required procedure	Onset date/stop date/study day	AE Duration (days)	AE related study drug action	AE related drug treatment/non-drug treatment	AE outcome
210110/ 23/female/Caucasian	Skin and subcutaneous tissue disorders/Urticaria/ URTICARIA	moderate	NO/NO	15JUL2008/01DEC2008/ 191	140	dose not changed	YES/NO	recovered/ resolved
210112/ 20/female/Caucasian	Psychiatric disorders/Completed suicide/SUICIDE	severe	NO/NO	15JUL2010/15JUL2010/ 917	1	dose not changed	NO/NO	fatal
210604/ 32/female/Caucasian	Neoplasms benign, malignant and unspecified (incl cysts and polyps)/Pancreatic carcinoma/CANCER PANCREATIS	severe	NO/NO	28JAN2009/ ./ 332	.	dose not changed	YES/YES	not recovered/ not resolved
230203/ 24/female/Caucasian	Nervous system disorders/Dizziness /DIZZINESS	moderate	NO/NO	02APR2008/ ./ 175	.	dose not changed	NO/NO	continuing with change
230215/ 31/female/Caucasian	Infections and infestations/Campylobacter intestinal infection/ENTERITIS DUE TO CAMPYLOBACT INFECTION	severe	NO/NO	07MAR2008/13MAR2008/ 39	7	dose not changed	YES/NO	recovered/ resolved

Table 14.3.2 / 2: Serious adverse events - Subject listing (FAS) (cont.)

TREATMENT: LCS16

Subject/ age/sex/race	Primary SOC/preferred term/AEX	AE maximum intensity	Related to study drug/protocol-required procedure	Onset date/stop date/study day	AE Duration (days)	AE related study drug action	AE related drug treatment/non-drug treatment	AE outcome
230309/ 28/female/Caucasian	Neoplasms benign, malignant and unspecified (incl cysts and polyps)/Cervix carcinoma stage 0/CARCINOMA IN SITU CERVICIS UTERI.	severe	NO/NO	03MAR2010/ ./ 749	.	drug withdrawn	YES/YES	recovering/ resolving
230506/ 33/female/Caucasian	Gastrointestinal disorders/Abdominal pain/ABDOMINAL PAIN	moderate	YES/NO	10MAR2008/11MAR2008/ 119	2	dose not changed	YES/NO	recovered/ resolved
230601/ 20/female/Caucasian	Psychiatric disorders/Panic disorder/PANIC SYNDROME	severe	NO/NO	08AUG2008/09AUG2008/ 254	2	dose not changed	YES/NO	recovered/ resolved
230612/ 29/female/Caucasian	Injury, poisoning and procedural complications/Procedural pain/ABDOMINAL PAIN AFTER GALL BLADDER SURGERY	severe	NO/NO	06MAR2008/07MAR2008/ 71	2	dose not changed	NO/YES	recovered/ resolved
240127/ 33/female/Asian	Nervous system disorders/Headache/HEADACHE	mild	NO/NO	06JAN2011/ ./ 1037	.	drug withdrawn	YES/NO	continuing with change

Table 14.3.2 / 2: Serious adverse events - Subject listing (FAS) (cont.)

TREATMENT: LCS16

Subject/ age/sex/race	Primary SOC/preferred term/AEX	AE maximum intensity	Related to study drug/protocol-required procedure	Onset date/stop date/study day	AE Duration (days)	AE related study drug action	AE related drug treatment/non-drug treatment	AE outcome
	Nervous system disorders/Paraesthesia/PARATHESIAS	severe	NO/NO	06JAN2011/ ./ 1037	.	drug withdrawn	YES/NO	continuing with change
240905/ 33/female/Asian	Infections and infestations/Appendicitis/ACUTE APPENDICITIS	severe	NO/NO	13APR2011/14APR2011/ 1085	2	dose not changed	YES/YES	recovered/ resolved
241412/ 32/female/Caucasian	Respiratory, thoracic and mediastinal disorders/Asthma/ ASTHMA	severe	NO/NO	15NOV2010/ ./ 988	.	dose not changed	YES/NO	not recovered/ not resolved
	Respiratory, thoracic and mediastinal disorders/Dyspnoea/SHORTNESS OF BREATH	severe	NO/NO	14NOV2010/30NOV2010/ 987	17	dose not changed	YES/NO	recovered/ resolved
	Respiratory, thoracic and mediastinal disorders/Hypoxia/ HYPOXEMIA	moderate	NO/NO	28NOV2010/30NOV2010/ 1001	3	dose not changed	NO/NO	recovered/ resolved
241511/ 28/female/Hispanic	Reproductive system and breast disorders/Ovarian cyst ruptured/RIGHT OVARIAN CYST - RUPTURE.	severe	NO/NO	09SEP2008/11SEP2008/ 300	3	dose not changed	YES/NO	recovered/ resolved

Table 14.3.2 / 2: Serious adverse events - Subject listing (FAS) (cont.)

TREATMENT: LCS16

Subject/ age/sex/race	Primary SOC/preferred term/AEX	AE maximum intensity	Related to study drug/protocol-required procedure	Onset date/stop date/study day	AE Duration (days)	AE related study drug action	AE related drug treatment/non-drug treatment	AE outcome
	Reproductive system and breast disorders/Ovarian cyst/COMPLEX LEFT OVARIAN CYST 2.4CM	mild	NO/NO	09SEP2008/ ./ 300	.	dose not changed	NO/NO	continuing with change
241546/ 30/female/Black	Gastrointestinal disorders/Abdominal pain/ABDOMINAL PAIN	severe	NO/NO	10JUN2010/11JUN2010/ 753	2	dose not changed	YES/NO	recovered/ resolved
241603/ 23/female/Black	Injury, poisoning and procedural complications/Procedural pain/POST - OPERATIVE PAIN	severe	NO/NO	19MAY2009/30SEP2009/ 436	135	dose not changed	YES/NO	recovered/ resolved
	Injury, poisoning and procedural complications/Procedural pain/[POST-OPERATIVE PAIN AFTER ELECTIVE SURGERY]	severe	NO/NO	24NOV2009/30JUL2010/ 625	249	dose not changed	YES/YES	recovered/ resolved
241726/ 28/female/Caucasian	Hepatobiliary disorders/Cholelithiasis/CHOLELITHIASIS	severe	NO/NO	23APR2009/ ./ 402	.	dose not changed	YES/YES	continuing with change

Table 14.3.2 / 2: Serious adverse events - Subject listing (FAS) (cont.)

TREATMENT: LCS16

Subject/ age/sex/race	Primary SOC/preferred term/AEX	AE maximum intensity	Related to study drug/protocol-required procedure	Onset date/stop date/study day	AE Duration (days)	AE related study drug action	AE related drug treatment/non-drug treatment	AE outcome
242321/ 31/female/other	Investigations/Haemoglobin decreased/DECREASED HEMOGLOBIN	severe	NO/NO	02JUL2010/ ./ 803	.	drug withdrawn	YES/NO	continuing with change
	Pregnancy, puerperium and perinatal conditions/Ruptured ectopic pregnancy/ECTOPIC PREGNANCY-RUPTURED	severe	YES/NO	02JUL2010/02JUL2010/ 803	1	drug withdrawn	YES/YES	recovered/ resolved
242704/ 19/female/Caucasian	Infections and infestations/Viral infection/VIRAL SYNDROME	severe	NO/NO	29MAR2009/06APR2009/ 384	9	drug withdrawn	YES/NO	recovered/ resolved
243011/ 24/female/Caucasian	Hepatobiliary disorders/Cholecystitis/CHOLECYSTITIS	severe	NO/NO	23MAR2009/27MAR2009/ 427	5	dose not changed	YES/YES	recovered/ resolved
243027/ 35/female/Caucasian	Infections and infestations/Cellulitis/LEFT HAND CELLULITIS	severe	NO/NO	01MAY2009/ ./ 353	.	dose not changed	YES/YES	continuing with change
243205/ 26/female/Caucasian	Infections and infestations/Appendicitis/APPENDICITIS	severe	NO/NO	20MAR2009/21MAR2009/ 446	2	dose not changed	YES/YES	recovered/ resolved

Table 14.3.2 / 2: Serious adverse events - Subject listing (FAS) (cont.)

TREATMENT: LCS16

Subject/ age/sex/race	Primary SOC/preferred term/AEX	AE maximum intensity	Related to study drug/protocol-required procedure	Onset date/stop date/study day	AE Duration (days)	AE related study drug action	AE related drug treatment/non-drug treatment	AE outcome
243228/ 23/female/Caucasian	Pregnancy, puerperium and perinatal conditions/Ectopic pregnancy/RIGHT ECTOPIC PREGNANCY	severe	YES/NO	29APR2009/30APR2009/ 345	2	drug withdrawn	NO/YES	recovered/ resolved
243707/ 34/female/Hispanic	Hepatobiliary disorders/Cholecystitis/CHOLECYSTITIS	moderate	NO/NO	01JUL2010/03JUL2010/ 870	3	dose not changed	NO/YES	recovered/ resolved
	Hepatobiliary disorders/Cholelithiasis/CHOLELITHIASIS	moderate	NO/NO	01JUL2010/03JUL2010/ 870	3	dose not changed	NO/YES	recovered/ resolved
243932/ 29/female/Caucasian	Pregnancy, puerperium and perinatal conditions/Ectopic pregnancy/ECTOPIC PREGNANCY	moderate	YES/NO	15MAR2011/02JUN2011/ 1119	80	drug withdrawn	YES/NO	recovered/ resolved
243937/ 27/female/Caucasian	Endocrine disorders/Goitre/WORSENING OF PRE EXISTING GOITER	moderate	NO/NO	18APR2008/18APR2008/ 43	1	dose not changed	NO/YES	recovered/ resolved
243975/ 24/female/Caucasian	Gastrointestinal disorders/Salivary gland calculus/SIALOLITHIASIS	severe	NO/NO	25JUL2008/ ./ 68	.	dose not changed	NO/NO	not recovered/ not resolved

Table 14.3.2 / 2: Serious adverse events - Subject listing (FAS) (cont.)

TREATMENT: LCS16

Subject/ age/sex/race	Primary SOC/preferred term/AEX	AE maximum intensity	Related to study drug/protocol-required procedure	Onset date/stop date/study day	AE Duration (days)	AE related study drug action	AE related drug treatment/non-drug treatment	AE outcome
	Gastrointestinal disorders/Salivary gland calculus/WORSENIG OF SIALOLITHIASIS	severe	NO/NO	28JUL2008/ ./ 71	.	dose not changed	NO/NO	continuing with change
244305/ 35/female/Caucasian	Infections and infestations/Pneumonia/PNEUMONIA	moderate	NO/NO	14APR2008/29APR2008/ 75	16	dose not changed	YES/NO	recovered/ resolved
244424/ 26/female/Caucasian	Psychiatric disorders/Affective disorder/MOOD DISORDER	severe	NO/NO	02AUG2009/ ./ 516	.	dose not changed	YES/NO	continuing with change
	Psychiatric disorders/Alcoholism/ALCOHOL DEPENDENCE	severe	NO/NO	24AUG2009/ ./ 538	.	dose not changed	NO/YES	continuing with change
	Psychiatric disorders/Bipolar disorder/BIPOLAR DISORDER	severe	NO/NO	24AUG2009/ ./ 538	.	dose not changed	YES/YES	continuing with change
	Psychiatric disorders/Drug dependence/COCAINE DEPENDENCE	severe	NO/NO	24AUG2009/ ./ 538	.	dose not changed	NO/YES	continuing with change

Table 14.3.2 / 2: Serious adverse events - Subject listing (FAS) (cont.)

TREATMENT: LCS16

Subject/ age/sex/race	Primary SOC/preferred term/AEX	AE maximum intensity	Related to study drug/protocol-required procedure	Onset date/stop date/study day	AE Duration (days)	AE related study drug action	AE related drug treatment/non-drug treatment	AE outcome
	Psychiatric disorders/Polysubstance dependence/POLY SUBSTANCE DEPENDENCE	severe	NO/NO	02AUG2009/ ./ 516	.	dose not changed	NO/NO	continuing with change
	Surgical and medical procedures/Detoxification/DETOX ADMISSION	severe	NO/NO	15JUL2009/01JUL2009/ 498	-13	dose not changed	./.	recovered/ resolved
244433/ 28/female/other	Infections and infestations/Appendicitis/ACUTE APPENDICITIS	severe	NO/NO	03OCT2008/05OCT2008/ 185	3	dose not changed	YES/YES	recovered/ resolved
244519/ 25/female/Caucasian	Pregnancy, puerperium and perinatal conditions/Ectopic pregnancy/POSSIBLE ECTOPIC PREGNANCY	mild	YES/NO	17JUN2010/20JUN2010/ 799	4	drug withdrawn	YES/YES	recovered/ resolved
244706/ 34/female/Black	Immune system disorders/Anaphylactic reaction/ANAPHYLACTIC REACTION (TO SHELL FISH)	severe	NO/NO	02SEP2010/02SEP2010/ 912	1	dose not changed	YES/NO	recovered/ resolved
244801/ 20/female/Black	Gastrointestinal disorders/Dysphagia/DYSPHAGIA	severe	NO/NO	18AUG2009/19AUG2009/ 492	2	dose not changed	YES/YES	recovered/ resolved

Table 14.3.2 / 2: Serious adverse events - Subject listing (FAS) (cont.)

TREATMENT: LCS16

Subject/ age/sex/race	Primary SOC/preferred term/AEX	AE maximum intensity	Related to study drug/protocol-required procedure	Onset date/stop date/study day	AE Duration (days)	AE related study drug action	AE related drug treatment/non-drug treatment	AE outcome
	Gastrointestinal disorders/Odynophagia/ODYNOPHAGIA	severe	NO/NO	18AUG2009/19AUG2009/ 492	2	dose not changed	YES/YES	recovered/ resolved
245427/ 24/female/Caucasian	Infections and infestations/Meningitis viral/VIRAL MENINGITIS	severe	NO/NO	30APR2011/06MAY2011/ 1088	7	dose not changed	YES/NO	recovered/ resolved
245805/ 19/female/Caucasian	Infections and infestations/Appendicitis/APPENDICITIS	severe	NO/NO	13JUN2010/18JUN2010/ 776	6	dose not changed	YES/YES	recovered/ resolved
245905/ 33/female/Caucasian	Gastrointestinal disorders/Rectal haemorrhage/RECTAL BLEEDING	moderate	NO/NO	07FEB2009/09FEB2009/ 325	3	dose not changed	YES/NO	recovered/ resolved
	Nervous system disorders/Convulsion/SEIZURE	moderate	NO/NO	07FEB2009/07FEB2009/ 325	1	dose not changed	NO/NO	recovered/ resolved

Note: AEX=adverse event verbatim investigator term. SOC=System Organ Class.
Note: Study day is calculated from the first day of study drug administration. The formula for this calculation is: (onset day of AE minus medication day) plus 1 day.
Note: Dictionary/ MedDRA Version 14.0
Global Biostatistics: /by-sasp/patdb/projects/de04209/310442/stat/prod_interim03/pgms/t-sae.sas epkll 12OCT2011 11:27
End of table

14.3.3 Pregnancies (see efficacy)
