# Supplementary material for: The Effect of Age, Parity and Body Mass Index on the Efficacy, Safety, Placement and User Satisfaction Associated With Two Low-Dose Levonorgestrel Intrauterine Contraceptive Systems: Subgroup Analyses of Data From a Phase III Trial
Source: PLoS One. 2015 Sep 17;10(9):e0135309. doi: 10.1371/journal.pone.0135309 (PMC4574776; doi:10.1371/journal.pone.0135309)
Supplement: S2 Data — (PDF) [file pone.0135309.s003.pdf]

Table of contents

Table /1: Sound depth of the uterus by treatment and parity (FAS) 2
Table /2: Reasons for premature discontinuation by type of prior births (parous women only) - FAS 3
Table /3: Number of successful first insertion by treatment and parity (FAS) 4
Table /4: Number of successful first insertion by treatment and type of prior births (parous women only) (FAS) 5
Table /5: Number of successful second insertion by treatment and parity (FAS) 6
Table /6: Number of successful second insertion by treatment and type of prior births (parous women only) (FAS) 7
Table /7: Investigator's evaluation of IUS insertion procedure by parity (0 births vs. 1 or more) and treatment (FAS) 8
Table /8: Investigator's evaluation of IUS insertion procedure and type of prior births (parous women only) and treatment (FAS) 9
Table /9: Subject's evaluation of pain during IUS insertion procedure by parity (0 births vs. 1 or more) and treatment (FAS) 10
Table /10: Subject's evaluation of pain during IUS insertion procedure by type of prior births (parous women only) and treatment (FAS) 11
Table /11: Number of subjects with dilatation performed including 'when' by parity and treatment (FAS) 12
Table /12: Number of subjects with dilatation performed including 'when' by type of prior births (parous women only) (FAS) 13
Table /13: Number of subjects who prematurely discontinued the study medication (including main reason) after randomization by treatment and year (all randomized subjects) 14
Table /14: Number of subjects who prematurely discontinued the study medication (including main reason) after randomization by treatment, year and parity (all randomized subjects) 18
Table /15: Number of subjects who prematurely discontinued the study medication (including main reason) after randomization by treatment, year and age group (all randomized subjects) 22
Table /16: Reasons for premature discontinuation by parity - FAS 26
Table /17: Reasons for premature discontinuation by age group - FAS 27
Table /18: Reason for discontinuation of study medication due to withdrawal of consent or AE by treatment and parity status (FAS) 28
Table /19: Reason for discontinuation of study medication due to withdrawal of consent or AE by treatment and age group (FAS) 29
Table /20: User satisfaction questionnaire by parity (FAS) 30
Table /21: User satisfaction questionnaire by age group (FAS) 34

Table /1: Sound depth of the uterus by treatment and parity (FAS)

Parity			TREATMENT	n	Nmiss	Mean	SD	Min	Q1	Median	Q3	Max
0 births	Uterus depth	(cm)	LCS12	555	1	6.97	0.76	2.0	6.50	7.00	7.50	9.0
			LCS16	572	2	6.93	0.79	2.0	6.50	7.00	7.00	10.0
			Total	1127	3	6.95	0.77	2.0	6.50	7.00	7.20	10.0
1 birth or more	Uterus depth	(cm)	LCS12	871	5	7.46	0.91	3.2	7.00	7.50	8.00	11.0
			LCS16	878	0	7.43	0.93	3.0	7.00	7.50	8.00	11.0
			Total	1749	5	7.44	0.92	3.0	7.00	7.50	8.00	11.0

Bayer: /by-sasp/patdb/projects/de04209/310442/stat/test_query06/pgms/t-ius01-soundparity.sas sgrpp 14JUL2015 13:50
End of table

Table /2: Reasons for premature discontinuation by type of prior births (parous women only) - FAS

Ceasarian section only		LCS12	LCS16	Total
no	n	688 (100.0%)	709 (100.0%)	1397 (100.0%)
	Study medication, administration status			
	missing	1 (0.1%)	0	1 (<0.1%)
	pat. lost, no further information avail.	1 (0.1%)	0	1 (<0.1%)
	study medication never administered	0	0	0
	other	0	0	0
	completed	407 (59.2%)	72 (10.2%)	479 (34.3%)
	prematurely discontinued	280 (40.7%)	278 (39.2%)	558 (39.9%)
	withdrawal of consent	12 (1.7%)	14 (2.0%)	26 (1.9%)
	protocol deviation	8 (1.2%)	9 (1.3%)	17 (1.2%)
	adverse event	126 (18.3%)	129 (18.2%)	255 (18.3%)
	pat. lost, no further information avail.	42 (6.1%)	33 (4.7%)	75 (5.4%)
	pregnancy	5 (0.7%)	6 (0.8%)	11 (0.8%)
	wish for pregnancy	57 (8.3%)	62 (8.7%)	119 (8.5%)
	other	30 (4.4%)	25 (3.5%)	55 (3.9%)
	ongoing	0	359 (50.6%)	359 (25.7%)
yes	n	188 (100.0%)	169 (100.0%)	357 (100.0%)
	Study medication, administration status			
	missing	0	0	0
	pat. lost, no further information avail.	0	0	0
	study medication never administered	0	1 (0.6%)	1 (0.3%)
	other	0	1 (0.6%)	1 (0.3%)
	completed	110 (58.5%)	19 (11.2%)	129 (36.1%)
	prematurely discontinued	78 (41.5%)	63 (37.3%)	141 (39.5%)
	withdrawal of consent	4 (2.1%)	5 (3.0%)	9 (2.5%)
	protocol deviation	3 (1.6%)	2 (1.2%)	5 (1.4%)
	adverse event	42 (22.3%)	31 (18.3%)	73 (20.4%)
	pat. lost, no further information avail.	9 (4.8%)	7 (4.1%)	16 (4.5%)
	pregnancy	0	1 (0.6%)	1 (0.3%)
	wish for pregnancy	16 (8.5%)	11 (6.5%)	27 (7.6%)
	other	4 (2.1%)	6 (3.6%)	10 (2.8%)
	ongoing	0	86 (50.9%)	86 (24.1%)

Bayer: /by-sasp/patdb/projects/de04209/310442/stat/test_query06/pgms/t-eosm-dis-ces.sas sgrpp 14JUL2015 13:51
End of table

Table /3: Number of successful first insertion by treatment and parity (FAS)

Parity		LCS12	LCS16	Total
0 births	Number of insertions	556 (100.0%)	574 (100.0%)	1130 (100.0%)
	IUS insertion completed			
	no	28 (5.0%)	28 (4.9%)	56 (5.0%)
	yes	528 (95.0%)	546 (95.1%)	1074 (95.0%)
1 birth or more	Number of insertions	876 (100.0%)	878 (100.0%)	1754 (100.0%)
	IUS insertion completed			
	no	24 (2.7%)	34 (3.9%)	58 (3.3%)
	yes	852 (97.3%)	844 (96.1%)	1696 (96.7%)

Bayer: /by-sasp/patdb/projects/de04209/310442/stat/test_query06/pgms/t-ius-admin-compl-ces.sas sgrpp 14JUL2015 13:51
End of table

Table /4: Number of successful first insertion by treatment and type of prior births (parous women only) (FAS)

Ceasarian section only		LCS12	LCS16	Total
no	Number of insertions	688 (100.0%)	709 (100.0%)	1397 (100.0%)
	IUS insertion completed			
	no	17 (2.5%)	27 (3.8%)	44 (3.1%)
	yes	671 (97.5%)	682 (96.2%)	1353 (96.9%)
yes	Number of insertions	188 (100.0%)	169 (100.0%)	357 (100.0%)
	IUS insertion completed			
	no	7 (3.7%)	7 (4.1%)	14 (3.9%)
	yes	181 (96.3%)	162 (95.9%)	343 (96.1%)

Bayer: /by-sasp/patdb/projects/de04209/310442/stat/test_query06/pgms/t-ius-admin-compl-ces.sas sgrpp 14JUL2015 13:51
End of table

Table /5: Number of successful second insertion by treatment and parity (FAS)

Parity		LCS12	LCS16	Total
0 births	Number of insertions	25 (100.0%)	25 (100.0%)	50 (100.0%)
	IUS insertion completed			
	no	2 (8.0%)	1 (4.0%)	3 (6.0%)
	yes	23 (92.0%)	24 (96.0%)	47 (94.0%)
1 birth or more	Number of insertions	24 (100.0%)	32 (100.0%)	56 (100.0%)
	IUS insertion completed			
	no	1 (4.2%)	1 (3.1%)	2 (3.6%)
	yes	23 (95.8%)	31 (96.9%)	54 (96.4%)

Bayer: /by-sasp/patdb/projects/de04209/310442/stat/test_query06/pgms/t-ius-admin-compl-ces.sas sgrpp 14JUL2015 13:51
End of table

Table /6: Number of successful second insertion by treatment and type of prior births (parous women only) (FAS)

Ceasarian section only		LCS12	LCS16	Total
no	Number of insertions	17 (100.0%)	26 (100.0%)	43 (100.0%)
	IUS insertion completed			
	no	1 (5.9%)	0	1 (2.3%)
	yes	16 (94.1%)	26 (100.0%)	42 (97.7%)
yes	Number of insertions	7 (100.0%)	6 (100.0%)	13 (100.0%)
	IUS insertion completed			
	no	0	1 (16.7%)	1 (7.7%)
	yes	7 (100.0%)	5 (83.3%)	12 (92.3%)

Bayer: /by-sasp/patdb/projects/de04209/310442/stat/test_query06/pgms/t-ius-admin-compl-ces.sas sgrpp 14JUL2015 13:51
End of table

Table /7: Investigator's evaluation of IUS insertion procedure by parity (0 births vs. 1 or more) and treatment (FAS)

	LCS12	LCS16	Total
n	1432 (100.0%)	1452 (100.0%)	2884 (100.0%)
Parity			
0 births	556 (38.8%)	574 (39.5%)	1130 (39.2%)
- of these: easy	468 (84.2%)	484 (84.3%)	952 (84.2%)
slightly difficult	76 (13.7%)	78 (13.6%)	154 (13.6%)
very difficult	12 (2.2%)	12 (2.1%)	24 (2.1%)
1 birth or more	876 (61.2%)	878 (60.5%)	1754 (60.8%)
- of these: easy	815 (93.0%)	818 (93.2%)	1633 (93.1%)
slightly difficult	55 (6.3%)	54 (6.2%)	109 (6.2%)
very difficult	6 (0.7%)	6 (0.7%)	12 (0.7%)

Bayer: /by-sasp/patdb/projects/de04209/310442/stat/test_query06/pgms/t-ius-admin-compl-ces.sas sgrpp 14JUL2015 13:51
End of table

Table /8: Investigator's evaluation of IUS insertion procedure and type of prior births (parous women only) and treatment (FAS)

	LCS12	LCS16	Total
n	876 (100.0%)	878 (100.0%)	1754 (100.0%)
Ceasarian section only			
no	688 (78.5%)	709 (80.8%)	1397 (79.6%)
- of these: easy	651 (94.6%)	669 (94.4%)	1320 (94.5%)
slightly difficult	33 (4.8%)	37 (5.2%)	70 (5.0%)
very difficult	4 (0.6%)	3 (0.4%)	7 (0.5%)
yes	188 (21.5%)	169 (19.2%)	357 (20.4%)
- of these: easy	164 (87.2%)	149 (88.2%)	313 (87.7%)
slightly difficult	22 (11.7%)	17 (10.1%)	39 (10.9%)
very difficult	2 (1.1%)	3 (1.8%)	5 (1.4%)

Bayer: /by-sasp/patdb/projects/de04209/310442/stat/test_query06/pgms/t-ius-admin-compl-ces.sas sgrpp 14JUL2015 13:51
End of table

Table /9: Subject's evaluation of pain during IUS insertion procedure by parity (0 births vs. 1 or more) and treatment (FAS)

	LCS12	LCS16	Total
Number of insertions	1432 (100.0%)	1452 (100.0%)	2884 (100.0%)
Parity			
0 births	556 (38.8%)	574 (39.5%)	1130 (39.2%)
- of these: missing	0	1 (0.2%)	1 (<0.1%)
none	40 (7.2%)	29 (5.1%)	69 (6.1%)
mild	178 (32.0%)	225 (39.2%)	403 (35.7%)
moderate	246 (44.2%)	236 (41.1%)	482 (42.7%)
severe	92 (16.5%)	83 (14.5%)	175 (15.5%)
1 birth or more	876 (61.2%)	878 (60.5%)	1754 (60.8%)
- of these: none	255 (29.1%)	239 (27.2%)	494 (28.2%)
mild	451 (51.5%)	458 (52.2%)	909 (51.8%)
moderate	144 (16.4%)	164 (18.7%)	308 (17.6%)
severe	26 (3.0%)	17 (1.9%)	43 (2.5%)

Bayer: /by-sasp/patdb/projects/de04209/310442/stat/test_query06/pgms/t-ius-admin-compl-ces.sas sgrpp 14JUL2015 13:51
End of table

Table /10: Subject's evaluation of pain during IUS insertion procedure by type of prior births (parous women only) and treatment (FAS)

	LCS12	LCS16	Total
Number of insertions	876 (100.0%)	878 (100.0%)	1754 (100.0%)
Ceasarian section only			
no	688 (78.5%)	709 (80.8%)	1397 (79.6%)
- of these: none	218 (31.7%)	202 (28.5%)	420 (30.1%)
mild	356 (51.7%)	367 (51.8%)	723 (51.8%)
moderate	99 (14.4%)	128 (18.1%)	227 (16.2%)
severe	15 (2.2%)	12 (1.7%)	27 (1.9%)
yes	188 (21.5%)	169 (19.2%)	357 (20.4%)
- of these: none	37 (19.7%)	37 (21.9%)	74 (20.7%)
mild	95 (50.5%)	91 (53.8%)	186 (52.1%)
moderate	45 (23.9%)	36 (21.3%)	81 (22.7%)
severe	11 (5.9%)	5 (3.0%)	16 (4.5%)

Bayer: /by-sasp/patdb/projects/de04209/310442/stat/test_query06/pgms/t-ius-admin-compl-ces.sas sgrpp 14JUL2015 13:51
End of table

Table /11: Number of subjects with dilatation performed including 'when' by parity and treatment (FAS)

Parity		LCS12	LCS16	Total
0 births	Number of insertions	556 (100.0%)	574 (100.0%)	1130 (100.0%)
	IUS insertion, dilatation used			
	no	505 (90.8%)	521 (90.8%)	1026 (90.8%)
	missing	505 (90.8%)	520 (90.6%)	1025 (90.7%)
	before procedure was performed	0	1 (0.2%)	1 (<0.1%)
	yes	51 (9.2%)	53 (9.2%)	104 (9.2%)
	before procedure was performed	36 (6.5%)	31 (5.4%)	67 (5.9%)
	when procedure proved to be difficult	14 (2.5%)	22 (3.8%)	36 (3.2%)
	when procedure proved to be painful	1 (0.2%)	0	1 (<0.1%)
1 birth or more	Number of insertions	876 (100.0%)	878 (100.0%)	1754 (100.0%)
	IUS insertion, dilatation used			
	no	850 (97.0%)	849 (96.7%)	1699 (96.9%)
	missing	850 (97.0%)	849 (96.7%)	1699 (96.9%)
	before procedure was performed	0	0	0
	yes	26 (3.0%)	29 (3.3%)	55 (3.1%)
	before procedure was performed	18 (2.1%)	20 (2.3%)	38 (2.2%)
	when procedure proved to be difficult	8 (0.9%)	9 (1.0%)	17 (1.0%)
	when procedure proved to be painful	0	0	0

Bayer: /by-sasp/patdb/projects/de04209/310442/stat/test_query06/pgms/t-ius-admin-compl-ces.sas sgrpp 14JUL2015 13:51
End of table

Table /12: Number of subjects with dilatation performed including 'when' by type of prior births (parous women only) (FAS)

Ceasarian section only		LCS12	LCS16	Total
no	Number of insertions	688 (100.0%)	709 (100.0%)	1397 (100.0%)
	IUS insertion, dilatation used			
	no	672 (97.7%)	692 (97.6%)	1364 (97.6%)
	yes	16 (2.3%)	17 (2.4%)	33 (2.4%)
	before procedure was performed	11 (1.6%)	12 (1.7%)	23 (1.6%)
	when procedure proved to be difficult	5 (0.7%)	5 (0.7%)	10 (0.7%)
yes	Number of insertions	188 (100.0%)	169 (100.0%)	357 (100.0%)
	IUS insertion, dilatation used			
	no	178 (94.7%)	157 (92.9%)	335 (93.8%)
	yes	10 (5.3%)	12 (7.1%)	22 (6.2%)
	before procedure was performed	7 (3.7%)	8 (4.7%)	15 (4.2%)
	when procedure proved to be difficult	3 (1.6%)	4 (2.4%)	7 (2.0%)

Bayer: /by-sasp/patdb/projects/de04209/310442/stat/test_query06/pgms/t-ius-admin-compl-ces.sas sgrpp 14JUL2015 13:51
End of table

Table /13: Number of subjects who prematurely discontinued the study medication (including main reason) after randomization by treatment and year (all randomized subjects)

YEAR AFTER INSERTION: 1st year

	LCS12	LCS16	Total
Study medication, administration status			
n	1432 (100.0%)	1453 (100.0%)	2885 (100.0%)
study medication never administered	0	2 (0.1%)	2 (<0.1%)
completed	0	0	0
prematurely discontinued	266 (18.6%)	245 (16.9%)	511 (17.7%)
ongoing	1166 (81.4%)	1206 (83.0%)	2372 (82.2%)
missing	0	0	0
Premature EOSM or never taken, reason			
n	266 (100.0%)	247 (100.0%)	513 (100.0%)
withdrawal of consent	11 (4.1%)	9 (3.6%)	20 (3.9%)
protocol deviation	3 (1.1%)	0	3 (0.6%)
adverse event	175 (65.8%)	168 (68.0%)	343 (66.9%)
death	0	0	0
pat. lost, no further information avail.	25 (9.4%)	21 (8.5%)	46 (9.0%)
pregnancy	5 (1.9%)	2 (0.8%)	7 (1.4%)
other	47 (17.7%)	47 (19.0%)	94 (18.3%)

Note: Year is calculated using the formula year = (last day on study (imputed)- insertion date)/365
Bayer: /by-sasp/patdb/projects/de04209/310442/stat/test_query06/pgms/t-ds3-2-parity-age.sas sgrpp 14JUL2015 13:51

Table /13: Number of subjects who prematurely discontinued the study medication (including main reason) after randomization by treatment and year (all randomized subjects) (cont.)

YEAR AFTER INSERTION: 2nd year

	LCS12	LCS16	Total
Study medication, administration status			
n	1166 (100.0%)	1206 (100.0%)	2372 (100.0%)
study medication never administered	0	0	0
completed	0	0	0
prematurely discontinued	203 (17.4%)	194 (16.1%)	397 (16.7%)
ongoing	963 (82.6%)	1012 (83.9%)	1975 (83.3%)
missing	0	0	0
Premature EOSM or never taken, reason			
n	203 (100.0%)	194 (100.0%)	397 (100.0%)
withdrawal of consent	9 (4.4%)	14 (7.2%)	23 (5.8%)
protocol deviation	5 (2.5%)	8 (4.1%)	13 (3.3%)
adverse event	85 (41.9%)	66 (34.0%)	151 (38.0%)
death	0	0	0
pat. lost, no further information avail.	23 (11.3%)	21 (10.8%)	44 (11.1%)
pregnancy	3 (1.5%)	3 (1.5%)	6 (1.5%)
other	78 (38.4%)	82 (42.3%)	160 (40.3%)

Note: Year is calculated using the formula year = (last day on study (imputed)- insertion date)/365
Bayer: /by-sasp/patdb/projects/de04209/310442/stat/test_query06/pgms/t-ds3-2-parity-age.sas sgrpp 14JUL2015 13:51

Table /13: Number of subjects who prematurely discontinued the study medication (including main reason) after randomization by treatment and year (all randomized subjects) (cont.)

YEAR AFTER INSERTION: 3rd year

	LCS12	LCS16	Total
Study medication, administration status			
n	963 (100.0%)	1012 (100.0%)	1975 (100.0%)
study medication never administered	0	0	0
completed	819 (85.0%)	163 (16.1%)	982 (49.7%)
prematurely discontinued	143 (14.8%)	142 (14.0%)	285 (14.4%)
ongoing	0	707 (69.9%)	707 (35.8%)
missing	1 (0.1%)	0	1 (<0.1%)
Premature EOSM or never taken, reason			
n	144 (100.0%)	142 (100.0%)	286 (100.0%)
withdrawal of consent	6 (4.2%)	8 (5.6%)	14 (4.9%)
protocol deviation	8 (5.6%)	8 (5.6%)	16 (5.6%)
adverse event	53 (36.8%)	44 (31.0%)	97 (33.9%)
death	0	1 (0.7%)	1 (0.3%)
pat. lost, no further information avail.	15 (10.4%)	19 (13.4%)	34 (11.9%)
pregnancy	1 (0.7%)	5 (3.5%)	6 (2.1%)
other	61 (42.4%)	57 (40.1%)	118 (41.3%)

Note: Year is calculated using the formula year = (last day on study (imputed)- insertion date)/365
Bayer: /by-sasp/patdb/projects/de04209/310442/stat/test_query06/pgms/t-ds3-2-parity-age.sas sgrpp 14JUL2015 13:51

Table /13: Number of subjects who prematurely discontinued the study medication (including main reason) after randomization by treatment and year (all randomized subjects) (cont.)

YEAR AFTER INSERTION: Overall

	LCS12	LCS16	Total
Study medication, administration status			
n	1432 (100.0%)	1453 (100.0%)	2885 (100.0%)
study medication never administered	0	2 (0.1%)	2 (<0.1%)
completed	819 (57.2%)	163 (11.2%)	982 (34.0%)
prematurely discontinued	612 (42.7%)	581 (40.0%)	1193 (41.4%)
ongoing	0	707 (48.7%)	707 (24.5%)
missing	1 (<0.1%)	0	1 (<0.1%)
Premature EOSM or never taken, reason			
n	613 (100.0%)	583 (100.0%)	1196 (100.0%)
withdrawal of consent	26 (4.2%)	31 (5.3%)	57 (4.8%)
protocol deviation	16 (2.6%)	16 (2.7%)	32 (2.7%)
adverse event	313 (51.1%)	278 (47.7%)	591 (49.4%)
death	0	1 (0.2%)	1 (<0.1%)
pat. lost, no further information avail.	63 (10.3%)	61 (10.5%)	124 (10.4%)
pregnancy	9 (1.5%)	10 (1.7%)	19 (1.6%)
other	186 (30.3%)	186 (31.9%)	372 (31.1%)

Note: Year is calculated using the formula year = (last day on study (imputed)- insertion date)/365
Bayer: /by-sasp/patdb/projects/de04209/310442/stat/test_query06/pgms/t-ds3-2-parity-age.sas sgrpp 14JUL2015 13:51
End of table

Table /14: Number of subjects who prematurely discontinued the study medication (including main reason) after randomization by treatment, year and parity (all randomized subjects)

YEAR AFTER INSERTION: 1st year

Parity		LCS12	LCS16	Total
0 births	Study medication, administration status			
	n	556 (100.0%)	574 (100.0%)	1130 (100.0%)
	study medication never administered	0	0	0
	completed	0	0	0
	prematurely discontinued	118 (21.2%)	116 (20.2%)	234 (20.7%)
	ongoing	438 (78.8%)	458 (79.8%)	896 (79.3%)
	missing	0	0	0
	Premature EOSM or never taken, reason			
	n	118 (100.0%)	116 (100.0%)	234 (100.0%)
	withdrawal of consent	6 (5.1%)	4 (3.4%)	10 (4.3%)
	protocol deviation	1 (0.8%)	0	1 (0.4%)
	adverse event	87 (73.7%)	81 (69.8%)	168 (71.8%)
	death	0	0	0
	pat. lost, no further information avail.	4 (3.4%)	9 (7.8%)	13 (5.6%)
	pregnancy	2 (1.7%)	0	2 (0.9%)
	other	18 (15.3%)	22 (19.0%)	40 (17.1%)
1 birth or more	Study medication, administration status			
	n	876 (100.0%)	879 (100.0%)	1755 (100.0%)
	study medication never administered	0	2 (0.2%)	2 (0.1%)
	completed	0	0	0
	prematurely discontinued	148 (16.9%)	129 (14.7%)	277 (15.8%)
	ongoing	728 (83.1%)	748 (85.1%)	1476 (84.1%)
	missing	0	0	0
	Premature EOSM or never taken, reason			
	n	148 (100.0%)	131 (100.0%)	279 (100.0%)
	withdrawal of consent	5 (3.4%)	5 (3.8%)	10 (3.6%)
	protocol deviation	2 (1.4%)	0	2 (0.7%)
	adverse event	88 (59.5%)	87 (66.4%)	175 (62.7%)
	death	0	0	0
	pat. lost, no further information avail.	21 (14.2%)	12 (9.2%)	33 (11.8%)
	pregnancy	3 (2.0%)	2 (1.5%)	5 (1.8%)
	other	29 (19.6%)	25 (19.1%)	54 (19.4%)

Note: Year is calculated using the formula year = (last day on study (imputed)- insertion date)/365
Bayer: /by-sasp/patdb/projects/de04209/310442/stat/test_query06/pgms/t-ds3-2-parity-age.sas sgrpp 14JUL2015 13:51

Table /14: Number of subjects who prematurely discontinued the study medication (including main reason) after randomization by treatment, year and parity (all randomized subjects) (cont.)

YEAR AFTER INSERTION: 2nd year

Parity		LCS12	LCS16	Total
0 births	Study medication, administration status			
	n	438 (100.0%)	458 (100.0%)	896 (100.0%)
	study medication never administered	0	0	0
	completed	0	0	0
	prematurely discontinued	85 (19.4%)	76 (16.6%)	161 (18.0%)
	ongoing	353 (80.6%)	382 (83.4%)	735 (82.0%)
	missing	0	0	0
	Premature EOSM or never taken, reason			
	n	85 (100.0%)	76 (100.0%)	161 (100.0%)
	withdrawal of consent	2 (2.4%)	5 (6.6%)	7 (4.3%)
	protocol deviation	2 (2.4%)	1 (1.3%)	3 (1.9%)
	adverse event	41 (48.2%)	26 (34.2%)	67 (41.6%)
	death	0	0	0
	pat. lost, no further information avail.	3 (3.5%)	7 (9.2%)	10 (6.2%)
	pregnancy	1 (1.2%)	0	1 (0.6%)
	other	36 (42.4%)	37 (48.7%)	73 (45.3%)
1 birth or more	Study medication, administration status			
	n	728 (100.0%)	748 (100.0%)	1476 (100.0%)
	study medication never administered	0	0	0
	completed	0	0	0
	prematurely discontinued	118 (16.2%)	118 (15.8%)	236 (16.0%)
	ongoing	610 (83.8%)	630 (84.2%)	1240 (84.0%)
	missing	0	0	0
	Premature EOSM or never taken, reason			
	n	118 (100.0%)	118 (100.0%)	236 (100.0%)
	withdrawal of consent	7 (5.9%)	9 (7.6%)	16 (6.8%)
	protocol deviation	3 (2.5%)	7 (5.9%)	10 (4.2%)
	adverse event	44 (37.3%)	40 (33.9%)	84 (35.6%)
	death	0	0	0
	pat. lost, no further information avail.	20 (16.9%)	14 (11.9%)	34 (14.4%)
	pregnancy	2 (1.7%)	3 (2.5%)	5 (2.1%)
	other	42 (35.6%)	45 (38.1%)	87 (36.9%)

Note: Year is calculated using the formula year = (last day on study (imputed)- insertion date)/365
Bayer: /by-sasp/patdb/projects/de04209/310442/stat/test_query06/pgms/t-ds3-2-parity-age.sas sgrpp 14JUL2015 13:51

Table /14: Number of subjects who prematurely discontinued the study medication (including main reason) after randomization by treatment, year and parity (all randomized subjects) (cont.)

YEAR AFTER INSERTION: 3rd year

Parity		LCS12	LCS16	Total
0 births	Study medication, administration status			
	n	353 (100.0%)	382 (100.0%)	735 (100.0%)
	study medication never administered	0	0	0
	completed	302 (85.6%)	72 (18.8%)	374 (50.9%)
	prematurely discontinued	51 (14.4%)	48 (12.6%)	99 (13.5%)
	ongoing	0	262 (68.6%)	262 (35.6%)
	missing	0	0	0
	Premature EOSM or never taken, reason			
	n	51 (100.0%)	48 (100.0%)	99 (100.0%)
	withdrawal of consent	2 (3.9%)	3 (6.3%)	5 (5.1%)
	protocol deviation	2 (3.9%)	4 (8.3%)	6 (6.1%)
	adverse event	17 (33.3%)	11 (22.9%)	28 (28.3%)
	death	0	1 (2.1%)	1 (1.0%)
	pat. lost, no further information avail.	4 (7.8%)	5 (10.4%)	9 (9.1%)
	pregnancy	1 (2.0%)	3 (6.3%)	4 (4.0%)
	other	25 (49.0%)	21 (43.8%)	46 (46.5%)
1 birth or more	Study medication, administration status			
	n	610 (100.0%)	630 (100.0%)	1240 (100.0%)
	study medication never administered	0	0	0
	completed	517 (84.8%)	91 (14.4%)	608 (49.0%)
	prematurely discontinued	92 (15.1%)	94 (14.9%)	186 (15.0%)
	ongoing	0	445 (70.6%)	445 (35.9%)
	missing	1 (0.2%)	0	1 (<0.1%)
	Premature EOSM or never taken, reason			
	n	93 (100.0%)	94 (100.0%)	187 (100.0%)
	withdrawal of consent	4 (4.3%)	5 (5.3%)	9 (4.8%)
	protocol deviation	6 (6.5%)	4 (4.3%)	10 (5.3%)
	adverse event	36 (38.7%)	33 (35.1%)	69 (36.9%)
	death	0	0	0
	pat. lost, no further information avail.	11 (11.8%)	14 (14.9%)	25 (13.4%)
	pregnancy	0	2 (2.1%)	2 (1.1%)
	other	36 (38.7%)	36 (38.3%)	72 (38.5%)

Note: Year is calculated using the formula year = (last day on study (imputed)- insertion date)/365
Bayer: /by-sasp/patdb/projects/de04209/310442/stat/test_query06/pgms/t-ds3-2-parity-age.sas sgrpp 14JUL2015 13:51

Table /14: Number of subjects who prematurely discontinued the study medication (including main reason) after randomization by treatment, year and parity (all randomized subjects) (cont.)

YEAR AFTER INSERTION: Overall

Parity		LCS12	LCS16	Total
0 births	Study medication, administration status			
	n	556 (100.0%)	574 (100.0%)	1130 (100.0%)
	study medication never administered	0	0	0
	completed	302 (54.3%)	72 (12.5%)	374 (33.1%)
	prematurely discontinued	254 (45.7%)	240 (41.8%)	494 (43.7%)
	ongoing	0	262 (45.6%)	262 (23.2%)
	missing	0	0	0
	Premature EOSM or never taken, reason			
	n	254 (100.0%)	240 (100.0%)	494 (100.0%)
	withdrawal of consent	10 (3.9%)	12 (5.0%)	22 (4.5%)
	protocol deviation	5 (2.0%)	5 (2.1%)	10 (2.0%)
	adverse event	145 (57.1%)	118 (49.2%)	263 (53.2%)
	death	0	1 (0.4%)	1 (0.2%)
	pat. lost, no further information avail.	11 (4.3%)	21 (8.8%)	32 (6.5%)
	pregnancy	4 (1.6%)	3 (1.3%)	7 (1.4%)
	other	79 (31.1%)	80 (33.3%)	159 (32.2%)
1 birth or more	Study medication, administration status			
	n	876 (100.0%)	879 (100.0%)	1755 (100.0%)
	study medication never administered	0	2 (0.2%)	2 (0.1%)
	completed	517 (59.0%)	91 (10.4%)	608 (34.6%)
	prematurely discontinued	358 (40.9%)	341 (38.8%)	699 (39.8%)
	ongoing	0	445 (50.6%)	445 (25.4%)
	missing	1 (0.1%)	0	1 (<0.1%)
	Premature EOSM or never taken, reason			
	n	359 (100.0%)	343 (100.0%)	702 (100.0%)
	withdrawal of consent	16 (4.5%)	19 (5.5%)	35 (5.0%)
	protocol deviation	11 (3.1%)	11 (3.2%)	22 (3.1%)
	adverse event	168 (46.8%)	160 (46.6%)	328 (46.7%)
	death	0	0	0
	pat. lost, no further information avail.	52 (14.5%)	40 (11.7%)	92 (13.1%)
	pregnancy	5 (1.4%)	7 (2.0%)	12 (1.7%)
	other	107 (29.8%)	106 (30.9%)	213 (30.3%)

Note: Year is calculated using the formula year = (last day on study (imputed)- insertion date)/365
Bayer: /by-sasp/patdb/projects/de04209/310442/stat/test_query06/pgms/t-ds3-2-parity-age.sas sgrpp 14JUL2015 13:51
End of table

Table /15: Number of subjects who prematurely discontinued the study medication (including main reason) after randomization by treatment, year and age group (all randomized subjects)

YEAR AFTER INSERTION: 1st year

Age category		LCS12	LCS16	Total
age <= 25	Study medication, administration status			
	n	566 (100.0%)	564 (100.0%)	1130 (100.0%)
	study medication never administered	0	1 (0.2%)	1 (<0.1%)
	completed	0	0	0
	prematurely discontinued	129 (22.8%)	104 (18.4%)	233 (20.6%)
	ongoing	437 (77.2%)	459 (81.4%)	896 (79.3%)
	missing	0	0	0
	Premature EOSM or never taken, reason			
	n	129 (100.0%)	105 (100.0%)	234 (100.0%)
	withdrawal of consent	4 (3.1%)	6 (5.7%)	10 (4.3%)
	protocol deviation	1 (0.8%)	0	1 (0.4%)
	adverse event	82 (63.6%)	70 (66.7%)	152 (65.0%)
	death	0	0	0
	pat. lost, no further information avail.	15 (11.6%)	10 (9.5%)	25 (10.7%)
	pregnancy	1 (0.8%)	1 (1.0%)	2 (0.9%)
	other	26 (20.2%)	18 (17.1%)	44 (18.8%)
25 < age <= 35	Study medication, administration status			
	n	866 (100.0%)	889 (100.0%)	1755 (100.0%)
	study medication never administered	0	1 (0.1%)	1 (<0.1%)
	completed	0	0	0
	prematurely discontinued	137 (15.8%)	141 (15.9%)	278 (15.8%)
	ongoing	729 (84.2%)	747 (84.0%)	1476 (84.1%)
	missing	0	0	0
	Premature EOSM or never taken, reason			
	n	137 (100.0%)	142 (100.0%)	279 (100.0%)
	withdrawal of consent	7 (5.1%)	3 (2.1%)	10 (3.6%)
	protocol deviation	2 (1.5%)	0	2 (0.7%)
	adverse event	93 (67.9%)	98 (69.0%)	191 (68.5%)
	death	0	0	0
	pat. lost, no further information avail.	10 (7.3%)	11 (7.7%)	21 (7.5%)
	pregnancy	4 (2.9%)	1 (0.7%)	5 (1.8%)
	other	21 (15.3%)	29 (20.4%)	50 (17.9%)

Note: Year is calculated using the formula year = (last day on study (imputed)- insertion date)/365
Bayer: /by-sasp/patdb/projects/de04209/310442/stat/test_query06/pgms/t-ds3-2-parity-age.sas sgrpp 14JUL2015 13:51

Table /15: Number of subjects who prematurely discontinued the study medication (including main reason) after randomization by treatment, year and age group (all randomized subjects) (cont.)

YEAR AFTER INSERTION: 2nd year

Age category		LCS12	LCS16	Total
age <= 25	Study medication, administration status			
	n	437 (100.0%)	459 (100.0%)	896 (100.0%)
	study medication never administered	0	0	0
	completed	0	0	0
	prematurely discontinued	82 (18.8%)	75 (16.3%)	157 (17.5%)
	ongoing	355 (81.2%)	384 (83.7%)	739 (82.5%)
	missing	0	0	0
	Premature EOSM or never taken, reason			
	n	82 (100.0%)	75 (100.0%)	157 (100.0%)
	withdrawal of consent	1 (1.2%)	5 (6.7%)	6 (3.8%)
	protocol deviation	3 (3.7%)	3 (4.0%)	6 (3.8%)
	adverse event	36 (43.9%)	28 (37.3%)	64 (40.8%)
	death	0	0	0
	pat. lost, no further information avail.	11 (13.4%)	10 (13.3%)	21 (13.4%)
	pregnancy	2 (2.4%)	0	2 (1.3%)
	other	29 (35.4%)	29 (38.7%)	58 (36.9%)
25 < age <= 35	Study medication, administration status			
	n	729 (100.0%)	747 (100.0%)	1476 (100.0%)
	study medication never administered	0	0	0
	completed	0	0	0
	prematurely discontinued	121 (16.6%)	119 (15.9%)	240 (16.3%)
	ongoing	608 (83.4%)	628 (84.1%)	1236 (83.7%)
	missing	0	0	0
	Premature EOSM or never taken, reason			
	n	121 (100.0%)	119 (100.0%)	240 (100.0%)
	withdrawal of consent	8 (6.6%)	9 (7.6%)	17 (7.1%)
	protocol deviation	2 (1.7%)	5 (4.2%)	7 (2.9%)
	adverse event	49 (40.5%)	38 (31.9%)	87 (36.3%)
	death	0	0	0
	pat. lost, no further information avail.	12 (9.9%)	11 (9.2%)	23 (9.6%)
	pregnancy	1 (0.8%)	3 (2.5%)	4 (1.7%)
	other	49 (40.5%)	53 (44.5%)	102 (42.5%)

Note: Year is calculated using the formula year = (last day on study (imputed)- insertion date)/365
Bayer: /by-sasp/patdb/projects/de04209/310442/stat/test_query06/pgms/t-ds3-2-parity-age.sas sgrpp 14JUL2015 13:51

Table /15: Number of subjects who prematurely discontinued the study medication (including main reason) after randomization by treatment, year and age group (all randomized subjects) (cont.)

YEAR AFTER INSERTION: 3rd year

Age category		LCS12	LCS16	Total
age <= 25	Study medication, administration status			
	n	355 (100.0%)	384 (100.0%)	739 (100.0%)
	study medication never administered	0	0	0
	completed	288 (81.1%)	73 (19.0%)	361 (48.8%)
	prematurely discontinued	66 (18.6%)	57 (14.8%)	123 (16.6%)
	ongoing	0	254 (66.1%)	254 (34.4%)
	missing	1 (0.3%)	0	1 (0.1%)
	Premature EOSM or never taken, reason			
	n	67 (100.0%)	57 (100.0%)	124 (100.0%)
	withdrawal of consent	3 (4.5%)	4 (7.0%)	7 (5.6%)
	protocol deviation	4 (6.0%)	6 (10.5%)	10 (8.1%)
	adverse event	24 (35.8%)	15 (26.3%)	39 (31.5%)
	death	0	1 (1.8%)	1 (0.8%)
	pat. lost, no further information avail.	6 (9.0%)	10 (17.5%)	16 (12.9%)
	pregnancy	0	1 (1.8%)	1 (0.8%)
	other	30 (44.8%)	20 (35.1%)	50 (40.3%)
25 < age <= 35	Study medication, administration status			
	n	608 (100.0%)	628 (100.0%)	1236 (100.0%)
	study medication never administered	0	0	0
	completed	531 (87.3%)	90 (14.3%)	621 (50.2%)
	prematurely discontinued	77 (12.7%)	85 (13.5%)	162 (13.1%)
	ongoing	0	453 (72.1%)	453 (36.7%)
	missing	0	0	0
	Premature EOSM or never taken, reason			
	n	77 (100.0%)	85 (100.0%)	162 (100.0%)
	withdrawal of consent	3 (3.9%)	4 (4.7%)	7 (4.3%)
	protocol deviation	4 (5.2%)	2 (2.4%)	6 (3.7%)
	adverse event	29 (37.7%)	29 (34.1%)	58 (35.8%)
	death	0	0	0
	pat. lost, no further information avail.	9 (11.7%)	9 (10.6%)	18 (11.1%)
	pregnancy	1 (1.3%)	4 (4.7%)	5 (3.1%)
	other	31 (40.3%)	37 (43.5%)	68 (42.0%)

Note: Year is calculated using the formula year = (last day on study (imputed)- insertion date)/365
Bayer: /by-sasp/patdb/projects/de04209/310442/stat/test_query06/pgms/t-ds3-2-parity-age.sas sgrpp 14JUL2015 13:51

Table /15: Number of subjects who prematurely discontinued the study medication (including main reason) after randomization by treatment, year and age group (all randomized subjects) (cont.)

YEAR AFTER INSERTION: Overall

Age category		LCS12	LCS16	Total
age <= 25	Study medication, administration status			
	n	566 (100.0%)	564 (100.0%)	1130 (100.0%)
	study medication never administered	0	1 (0.2%)	1 (<0.1%)
	completed	288 (50.9%)	73 (12.9%)	361 (31.9%)
	prematurely discontinued	277 (48.9%)	236 (41.8%)	513 (45.4%)
	ongoing	0	254 (45.0%)	254 (22.5%)
	missing	1 (0.2%)	0	1 (<0.1%)
	Premature EOSM or never taken, reason			
	n	278 (100.0%)	237 (100.0%)	515 (100.0%)
	withdrawal of consent	8 (2.9%)	15 (6.3%)	23 (4.5%)
	protocol deviation	8 (2.9%)	9 (3.8%)	17 (3.3%)
	adverse event	142 (51.1%)	113 (47.7%)	255 (49.5%)
	death	0	1 (0.4%)	1 (0.2%)
	pat. lost, no further information avail.	32 (11.5%)	30 (12.7%)	62 (12.0%)
	pregnancy	3 (1.1%)	2 (0.8%)	5 (1.0%)
	other	85 (30.6%)	67 (28.3%)	152 (29.5%)
25 < age <= 35	Study medication, administration status			
	n	866 (100.0%)	889 (100.0%)	1755 (100.0%)
	study medication never administered	0	1 (0.1%)	1 (<0.1%)
	completed	531 (61.3%)	90 (10.1%)	621 (35.4%)
	prematurely discontinued	335 (38.7%)	345 (38.8%)	680 (38.7%)
	ongoing	0	453 (51.0%)	453 (25.8%)
	missing	0	0	0
	Premature EOSM or never taken, reason			
	n	335 (100.0%)	346 (100.0%)	681 (100.0%)
	withdrawal of consent	18 (5.4%)	16 (4.6%)	34 (5.0%)
	protocol deviation	8 (2.4%)	7 (2.0%)	15 (2.2%)
	adverse event	171 (51.0%)	165 (47.7%)	336 (49.3%)
	death	0	0	0
	pat. lost, no further information avail.	31 (9.3%)	31 (9.0%)	62 (9.1%)
	pregnancy	6 (1.8%)	8 (2.3%)	14 (2.1%)
	other	101 (30.1%)	119 (34.4%)	220 (32.3%)

Note: Year is calculated using the formula year = (last day on study (imputed)- insertion date)/365
Bayer: /by-sasp/patdb/projects/de04209/310442/stat/test_query06/pgms/t-ds3-2-parity-age.sas sgrpp 14JUL2015 13:51
End of table

Table /16: Reasons for premature discontinuation by parity - FAS

Parity		LCS12	LCS16	Total
0 births	n	556 (100.0%)	574 (100.0%)	1130 (100.0%)
	Study medication, administration status			
	missing	0	0	0
	pat. lost, no further information avail.	0	0	0
	study medication never administered	0	0	0
	other	0	0	0
	completed	302 (54.3%)	72 (12.5%)	374 (33.1%)
	prematurely discontinued	254 (45.7%)	240 (41.8%)	494 (43.7%)
	withdrawal of consent	10 (1.8%)	12 (2.1%)	22 (1.9%)
	protocol deviation	5 (0.9%)	5 (0.9%)	10 (0.9%)
	adverse event	145 (26.1%)	118 (20.6%)	263 (23.3%)
	death	0	1 (0.2%)	1 (<0.1%)
	pat. lost, no further information avail.	11 (2.0%)	21 (3.7%)	32 (2.8%)
	pregnancy	4 (0.7%)	3 (0.5%)	7 (0.6%)
	wish for pregnancy	42 (7.6%)	45 (7.8%)	87 (7.7%)
	other	37 (6.7%)	35 (6.1%)	72 (6.4%)
	ongoing	0	262 (45.6%)	262 (23.2%)
1 birth or more	n	876 (100.0%)	878 (100.0%)	1754 (100.0%)
	Study medication, administration status			
	missing	1 (0.1%)	0	1 (<0.1%)
	pat. lost, no further information avail.	1 (0.1%)	0	1 (<0.1%)
	study medication never administered	0	1 (0.1%)	1 (<0.1%)
	other	0	1 (0.1%)	1 (<0.1%)
	completed	517 (59.0%)	91 (10.4%)	608 (34.7%)
	prematurely discontinued	358 (40.9%)	341 (38.8%)	699 (39.9%)
	withdrawal of consent	16 (1.8%)	19 (2.2%)	35 (2.0%)
	protocol deviation	11 (1.3%)	11 (1.3%)	22 (1.3%)
	adverse event	168 (19.2%)	160 (18.2%)	328 (18.7%)
	death	0	0	0
	pat. lost, no further information avail.	51 (5.8%)	40 (4.6%)	91 (5.2%)
	pregnancy	5 (0.6%)	7 (0.8%)	12 (0.7%)
	wish for pregnancy	73 (8.3%)	73 (8.3%)	146 (8.3%)
	other	34 (3.9%)	31 (3.5%)	65 (3.7%)
	ongoing	0	445 (50.7%)	445 (25.4%)

Bayer: /by-sasp/patdb/projects/de04209/310442/stat/test_query06/pgms/t-eosmr-parity-age.sas sgrpp 14JUL2015 13:51
End of table

Table /17: Reasons for premature discontinuation by age group - FAS

Age category		LCS12	LCS16	Total
age <= 25	n	566 (100.0%)	564 (100.0%)	1130 (100.0%)
	Study medication, administration status			
	missing	1 (0.2%)	0	1 (<0.1%)
	pat. lost, no further information avail.	1 (0.2%)	0	1 (<0.1%)
	study medication never administered	0	1 (0.2%)	1 (<0.1%)
	other	0	1 (0.2%)	1 (<0.1%)
	completed	288 (50.9%)	73 (12.9%)	361 (31.9%)
	prematurely discontinued	277 (48.9%)	236 (41.8%)	513 (45.4%)
	withdrawal of consent	8 (1.4%)	15 (2.7%)	23 (2.0%)
	protocol deviation	8 (1.4%)	9 (1.6%)	17 (1.5%)
	adverse event	142 (25.1%)	113 (20.0%)	255 (22.6%)
	death	0	1 (0.2%)	1 (<0.1%)
	pat. lost, no further information avail.	31 (5.5%)	30 (5.3%)	61 (5.4%)
	pregnancy	3 (0.5%)	2 (0.4%)	5 (0.4%)
	wish for pregnancy	48 (8.5%)	39 (6.9%)	87 (7.7%)
	other	37 (6.5%)	27 (4.8%)	64 (5.7%)
	ongoing	0	254 (45.0%)	254 (22.5%)
25 < age <= 35	n	866 (100.0%)	888 (100.0%)	1754 (100.0%)
	Study medication, administration status			
	missing	0	0	0
	pat. lost, no further information avail.	0	0	0
	study medication never administered	0	0	0
	other	0	0	0
	completed	531 (61.3%)	90 (10.1%)	621 (35.4%)
	prematurely discontinued	335 (38.7%)	345 (38.9%)	680 (38.8%)
	withdrawal of consent	18 (2.1%)	16 (1.8%)	34 (1.9%)
	protocol deviation	8 (0.9%)	7 (0.8%)	15 (0.9%)
	adverse event	171 (19.7%)	165 (18.6%)	336 (19.2%)
	death	0	0	0
	pat. lost, no further information avail.	31 (3.6%)	31 (3.5%)	62 (3.5%)
	pregnancy	6 (0.7%)	8 (0.9%)	14 (0.8%)
	wish for pregnancy	67 (7.7%)	79 (8.9%)	146 (8.3%)
	other	34 (3.9%)	39 (4.4%)	73 (4.2%)
	ongoing	0	453 (51.0%)	453 (25.8%)

Bayer: /by-sasp/patdb/projects/de04209/310442/stat/test_query06/pgms/t-eosmr-parity-age.sas sgrpp 14JUL2015 13:51
End of table

Table /18: Reason for discontinuation of study medication due to withdrawal of consent or AE by treatment and parity status (FAS)

Parity		LCS12	LCS16	Total
0 births	n	556 (100.0%)	574 (100.0%)	1130 (100.0%)
	SM discontinued due to withdrawal of consent or AE			
	no	401 (72.1%)	444 (77.4%)	845 (74.8%)
	yes	155 (27.9%)	130 (22.6%)	285 (25.2%)
	missing	2 (0.4%)	1 (0.2%)	3 (0.3%)
	progestin-related side effect	21 (3.8%)	20 (3.5%)	41 (3.6%)
	bleeding/non bleeding problem	29 (5.2%)	32 (5.6%)	61 (5.4%)
	UNK	5 (0.9%)	5 (0.9%)	10 (0.9%)
	other	98 (17.6%)	72 (12.5%)	170 (15.0%)
1 birth or more	n	876 (100.0%)	878 (100.0%)	1754 (100.0%)
	SM discontinued due to withdrawal of consent or AE			
	no	692 (79.0%)	699 (79.6%)	1391 (79.3%)
	yes	184 (21.0%)	179 (20.4%)	363 (20.7%)
	missing	4 (0.5%)	2 (0.2%)	6 (0.3%)
	progestin-related side effect	27 (3.1%)	20 (2.3%)	47 (2.7%)
	bleeding/non bleeding problem	39 (4.5%)	39 (4.4%)	78 (4.4%)
	UNK	9 (1.0%)	11 (1.3%)	20 (1.1%)
	other	105 (12.0%)	107 (12.2%)	212 (12.1%)
total	n	1432 (100.0%)	1452 (100.0%)	2884 (100.0%)
	SM discontinued due to withdrawal of consent or AE			
	no	1093 (76.3%)	1143 (78.7%)	2236 (77.5%)
	yes	339 (23.7%)	309 (21.3%)	648 (22.5%)
	missing	6 (0.4%)	3 (0.2%)	9 (0.3%)
	progestin-related side effect	48 (3.4%)	40 (2.8%)	88 (3.1%)
	bleeding/non bleeding problem	68 (4.7%)	71 (4.9%)	139 (4.8%)
	UNK	14 (1.0%)	16 (1.1%)	30 (1.0%)
	other	203 (14.2%)	179 (12.3%)	382 (13.2%)

Note: SM = Study medication
Bayer: /by-sasp/patdb/projects/de04209/310442/stat/test_query06/pgms/t-ds3-3-parity-age.sas sgrpp 14JUL2015 13:51
End of table

Table /19: Reason for discontinuation of study medication due to withdrawal of consent or AE by treatment and age group (FAS)

Age category		LCS12	LCS16	Total
age <= 25	n	1132 (100.0%)	1128 (100.0%)	2260 (100.0%)
	SM discontinued due to withdrawal of consent or AE			
	no	832 (73.5%)	872 (77.3%)	1704 (75.4%)
	yes	300 (26.5%)	256 (22.7%)	556 (24.6%)
	missing	6 (0.5%)	4 (0.4%)	10 (0.4%)
	progestin-related side effect	38 (3.4%)	36 (3.2%)	74 (3.3%)
	bleeding/non bleeding problem	38 (3.4%)	50 (4.4%)	88 (3.9%)
	UNK	10 (0.9%)	14 (1.2%)	24 (1.1%)
	other	208 (18.4%)	152 (13.5%)	360 (15.9%)
25 < age <= 35	n	1732 (100.0%)	1776 (100.0%)	3508 (100.0%)
	SM discontinued due to withdrawal of consent or AE			
	no	1354 (78.2%)	1414 (79.6%)	2768 (78.9%)
	yes	378 (21.8%)	362 (20.4%)	740 (21.1%)
	missing	6 (0.3%)	2 (0.1%)	8 (0.2%)
	progestin-related side effect	58 (3.3%)	44 (2.5%)	102 (2.9%)
	bleeding/non bleeding problem	98 (5.7%)	92 (5.2%)	190 (5.4%)
	UNK	18 (1.0%)	18 (1.0%)	36 (1.0%)
	other	198 (11.4%)	206 (11.6%)	404 (11.5%)

Note: SM = Study medication
Bayer: /by-sasp/patdb/projects/de04209/310442/stat/test_query06/pgms/t-ds3-3-parity-age.sas sgrpp 14JUL2015 13:51
End of table

Table /20: User satisfaction questionnaire by parity (FAS)

Parity: 0 births

Volunteer satisf. questionn., questions	Volunteer satisfaction questionnaire	LCS12	LCS16	Total
study treat, overall satisfaction	n	397 (100.0%)	405 (100.0%)	802 (100.0%)
	very satisfied	283 (71.3%)	305 (75.3%)	588 (73.3%)
	somewhat satisfied	91 (22.9%)	83 (20.5%)	174 (21.7%)
	neither satisfied / dissatisfied	13 (3.3%)	6 (1.5%)	19 (2.4%)
	dissatisfied	9 (2.3%)	9 (2.2%)	18 (2.2%)
	very dissatisfied	1 (0.3%)	2 (0.5%)	3 (0.4%)
change of regimen, likelihood	n	397 (100.0%)	405 (100.0%)	802 (100.0%)
	missing	1 (0.3%)	0	1 (0.1%)
	continue with LCS	291 (73.3%)	323 (79.8%)	614 (76.6%)
	use a different horm. contr.	44 (11.1%)	24 (5.9%)	68 (8.5%)
	use a different contr. meth.	30 (7.6%)	25 (6.2%)	55 (6.9%)
	discontinue use of all types of c	13 (3.3%)	14 (3.5%)	27 (3.4%)
	don't know	18 (4.5%)	19 (4.7%)	37 (4.6%)
menstrual bleeding, comparison	n	397 (100.0%)	405 (100.0%)	802 (100.0%)
	missing	1 (0.3%)	0	1 (0.1%)
	decreased	338 (85.1%)	374 (92.3%)	712 (88.8%)
	no change	36 (9.1%)	17 (4.2%)	53 (6.6%)
	increased	22 (5.5%)	14 (3.5%)	36 (4.5%)
menstrual bleeding pattern	n	397 (100.0%)	405 (100.0%)	802 (100.0%)
	missing	0	2 (0.5%)	2 (0.2%)
	very satisfied	165 (41.6%)	183 (45.2%)	348 (43.4%)
	somewhat satisfied	124 (31.2%)	106 (26.2%)	230 (28.7%)
	neither satisfied / dissatisfied	52 (13.1%)	36 (8.9%)	88 (11.0%)
	dissatisfied	30 (7.6%)	26 (6.4%)	56 (7.0%)
	very dissatisfied	8 (2.0%)	6 (1.5%)	14 (1.7%)
	not applicable	18 (4.5%)	46 (11.4%)	64 (8.0%)

Table /20: User satisfaction questionnaire by parity (FAS)

Parity: 0 births

Volunteer satisf. questionn., questions	Volunteer satisfaction questionnaire	LCS12	LCS16	Total
menstrual bleeding, unexpected	n	397 (100.0%)	405 (100.0%)	802 (100.0%)
	missing	1 (0.3%)	0	1 (0.1%)
	never	118 (29.7%)	111 (27.4%)	229 (28.6%)
	seldom	237 (59.7%)	254 (62.7%)	491 (61.2%)
	often	35 (8.8%)	33 (8.1%)	68 (8.5%)
	very often	6 (1.5%)	7 (1.7%)	13 (1.6%)
menstrual bleeding, absence	n	397 (100.0%)	405 (100.0%)	802 (100.0%)
	missing	362 (91.2%)	311 (76.8%)	673 (83.9%)
	very satisfied	29 (7.3%)	79 (19.5%)	108 (13.5%)
	somewhat satisfied	5 (1.3%)	11 (2.7%)	16 (2.0%)
	neither satisfied / dissatisfied	0	4 (1.0%)	4 (0.5%)
	dissatisfied	1 (0.3%)	0	1 (0.1%)
menstrual pain, treatment	n	397 (100.0%)	405 (100.0%)	802 (100.0%)
	none	92 (23.2%)	116 (28.6%)	208 (25.9%)
	mild	194 (48.9%)	199 (49.1%)	393 (49.0%)
	moderate	90 (22.7%)	79 (19.5%)	169 (21.1%)
	severe	21 (5.3%)	11 (2.7%)	32 (4.0%)
menstrual pain, comparison	n	397 (100.0%)	405 (100.0%)	802 (100.0%)
	missing	43 (10.8%)	25 (6.2%)	68 (8.5%)
	decreased	197 (49.6%)	251 (62.0%)	448 (55.9%)
	no change	71 (17.9%)	81 (20.0%)	152 (19.0%)
	increased	86 (21.7%)	48 (11.9%)	134 (16.7%)

Note: Only collected after Amendment 3 to study protocol
Bayer: /by-sasp/patdb/projects/de04209/310442/stat/test_query06/pgms/t-usq.sas sgrpp 14JUL2015 13:52

Table /20: User satisfaction questionnaire by parity (FAS) (cont.)

Parity: 1 birth or more

Volunteer satisf. questionn., questions	Volunteer satisfaction questionnaire	LCS12	LCS16	Total
study treat, overall satisfaction	n	656 (100.0%)	658 (100.0%)	1314 (100.0%)
	missing	1 (0.2%)	1 (0.2%)	2 (0.2%)
	very satisfied	513 (78.2%)	537 (81.6%)	1050 (79.9%)
	somewhat satisfied	110 (16.8%)	94 (14.3%)	204 (15.5%)
	neither satisfied / dissatisfied	18 (2.7%)	16 (2.4%)	34 (2.6%)
	dissatisfied	14 (2.1%)	9 (1.4%)	23 (1.8%)
	very dissatisfied	0	1 (0.2%)	1 (<0.1%)
change of regimen, likelihood	n	656 (100.0%)	658 (100.0%)	1314 (100.0%)
	missing	1 (0.2%)	1 (0.2%)	2 (0.2%)
	continue with LCS	520 (79.3%)	549 (83.4%)	1069 (81.4%)
	use a different horm. contr.	42 (6.4%)	21 (3.2%)	63 (4.8%)
	use a different contr. meth.	49 (7.5%)	43 (6.5%)	92 (7.0%)
	discontinue use of all types of c	15 (2.3%)	13 (2.0%)	28 (2.1%)
	don't know	29 (4.4%)	31 (4.7%)	60 (4.6%)
menstrual bleeding, comparison	n	656 (100.0%)	658 (100.0%)	1314 (100.0%)
	missing	1 (0.2%)	1 (0.2%)	2 (0.2%)
	decreased	575 (87.7%)	611 (92.9%)	1186 (90.3%)
	no change	49 (7.5%)	31 (4.7%)	80 (6.1%)
	increased	31 (4.7%)	15 (2.3%)	46 (3.5%)
menstrual bleeding pattern	n	656 (100.0%)	658 (100.0%)	1314 (100.0%)
	missing	2 (0.3%)	3 (0.5%)	5 (0.4%)
	very satisfied	349 (53.2%)	354 (53.8%)	703 (53.5%)
	somewhat satisfied	169 (25.8%)	166 (25.2%)	335 (25.5%)
	neither satisfied / dissatisfied	48 (7.3%)	41 (6.2%)	89 (6.8%)
	dissatisfied	36 (5.5%)	33 (5.0%)	69 (5.3%)
	very dissatisfied	12 (1.8%)	7 (1.1%)	19 (1.4%)
	not applicable	40 (6.1%)	54 (8.2%)	94 (7.2%)

Table /20: User satisfaction questionnaire by parity (FAS) (cont.)

Parity: 1 birth or more

Volunteer satisf. questionn., questions	Volunteer satisfaction questionnaire	LCS12	LCS16	Total
menstrual bleeding, unexpected	n	656 (100.0%)	658 (100.0%)	1314 (100.0%)
	missing	2 (0.3%)	1 (0.2%)	3 (0.2%)
	never	224 (34.1%)	216 (32.8%)	440 (33.5%)
	seldom	352 (53.7%)	380 (57.8%)	732 (55.7%)
	often	63 (9.6%)	46 (7.0%)	109 (8.3%)
	very often	15 (2.3%)	15 (2.3%)	30 (2.3%)
menstrual bleeding, absence	n	656 (100.0%)	658 (100.0%)	1314 (100.0%)
	missing	556 (84.8%)	522 (79.3%)	1078 (82.0%)
	very satisfied	86 (13.1%)	110 (16.7%)	196 (14.9%)
	somewhat satisfied	7 (1.1%)	15 (2.3%)	22 (1.7%)
	neither satisfied / dissatisfied	6 (0.9%)	7 (1.1%)	13 (1.0%)
	dissatisfied	1 (0.2%)	4 (0.6%)	5 (0.4%)
menstrual pain, treatment	n	656 (100.0%)	658 (100.0%)	1314 (100.0%)
	missing	3 (0.5%)	1 (0.2%)	4 (0.3%)
	none	333 (50.8%)	332 (50.5%)	665 (50.6%)
	mild	275 (41.9%)	281 (42.7%)	556 (42.3%)
	moderate	40 (6.1%)	40 (6.1%)	80 (6.1%)
	severe	5 (0.8%)	4 (0.6%)	9 (0.7%)
menstrual pain, comparison	n	656 (100.0%)	658 (100.0%)	1314 (100.0%)
	missing	96 (14.6%)	89 (13.5%)	185 (14.1%)
	decreased	405 (61.7%)	430 (65.3%)	835 (63.5%)
	no change	124 (18.9%)	116 (17.6%)	240 (18.3%)
	increased	31 (4.7%)	23 (3.5%)	54 (4.1%)

Note: Only collected after Amendment 3 to study protocol
Bayer: /by-sasp/patdb/projects/de04209/310442/stat/test_query06/pgms/t-usq.sas sgrpp 14JUL2015 13:52
End of table

Table /21: User satisfaction questionnaire by age group (FAS)

Age category: age <= 25

Volunteer satisf. questionn., questions	Volunteer satisfaction questionnaire	LCS12	LCS16	Total
study treat, overall satisfaction	n	388 (100.0%)	396 (100.0%)	784 (100.0%)
	missing	0	1 (0.3%)	1 (0.1%)
	very satisfied	295 (76.0%)	313 (79.0%)	608 (77.6%)
	somewhat satisfied	79 (20.4%)	69 (17.4%)	148 (18.9%)
	neither satisfied / dissatisfied	9 (2.3%)	8 (2.0%)	17 (2.2%)
	dissatisfied	5 (1.3%)	5 (1.3%)	10 (1.3%)
change of regimen, likelihood	n	388 (100.0%)	396 (100.0%)	784 (100.0%)
	missing	1 (0.3%)	1 (0.3%)	2 (0.3%)
	continue with LCS	289 (74.5%)	311 (78.5%)	600 (76.5%)
	use a different horm. contr.	33 (8.5%)	22 (5.6%)	55 (7.0%)
	use a different contr. meth.	35 (9.0%)	31 (7.8%)	66 (8.4%)
	discontinue use of all types of c	13 (3.4%)	9 (2.3%)	22 (2.8%)
	don't know	17 (4.4%)	22 (5.6%)	39 (5.0%)
menstrual bleeding, comparison	n	388 (100.0%)	396 (100.0%)	784 (100.0%)
	missing	1 (0.3%)	1 (0.3%)	2 (0.3%)
	decreased	327 (84.3%)	365 (92.2%)	692 (88.3%)
	no change	39 (10.1%)	17 (4.3%)	56 (7.1%)
	increased	21 (5.4%)	13 (3.3%)	34 (4.3%)
menstrual bleeding pattern	n	388 (100.0%)	396 (100.0%)	784 (100.0%)
	missing	0	3 (0.8%)	3 (0.4%)
	very satisfied	183 (47.2%)	194 (49.0%)	377 (48.1%)
	somewhat satisfied	105 (27.1%)	107 (27.0%)	212 (27.0%)
	neither satisfied / dissatisfied	49 (12.6%)	35 (8.8%)	84 (10.7%)
	dissatisfied	27 (7.0%)	19 (4.8%)	46 (5.9%)
	very dissatisfied	6 (1.5%)	4 (1.0%)	10 (1.3%)
	not applicable	18 (4.6%)	34 (8.6%)	52 (6.6%)

Table /21: User satisfaction questionnaire by age group (FAS)

Age category: age <= 25

Volunteer satisf. questionn., questions	Volunteer satisfaction questionnaire	LCS12	LCS16	Total
menstrual bleeding, unexpected	n	388 (100.0%)	396 (100.0%)	784 (100.0%)
	missing	1 (0.3%)	1 (0.3%)	2 (0.3%)
	never	115 (29.6%)	104 (26.3%)	219 (27.9%)
	seldom	233 (60.1%)	253 (63.9%)	486 (62.0%)
	often	34 (8.8%)	29 (7.3%)	63 (8.0%)
	very often	5 (1.3%)	9 (2.3%)	14 (1.8%)
menstrual bleeding, absence	n	388 (100.0%)	396 (100.0%)	784 (100.0%)
	missing	345 (88.9%)	323 (81.6%)	668 (85.2%)
	very satisfied	36 (9.3%)	61 (15.4%)	97 (12.4%)
	somewhat satisfied	3 (0.8%)	7 (1.8%)	10 (1.3%)
	neither satisfied / dissatisfied	4 (1.0%)	5 (1.3%)	9 (1.1%)
menstrual pain, treatment	n	388 (100.0%)	396 (100.0%)	784 (100.0%)
	missing	1 (0.3%)	1 (0.3%)	2 (0.3%)
	none	113 (29.1%)	127 (32.1%)	240 (30.6%)
	mild	181 (46.6%)	198 (50.0%)	379 (48.3%)
	moderate	78 (20.1%)	61 (15.4%)	139 (17.7%)
	severe	15 (3.9%)	9 (2.3%)	24 (3.1%)
menstrual pain, comparison	n	388 (100.0%)	396 (100.0%)	784 (100.0%)
	missing	48 (12.4%)	34 (8.6%)	82 (10.5%)
	decreased	194 (50.0%)	244 (61.6%)	438 (55.9%)
	no change	82 (21.1%)	78 (19.7%)	160 (20.4%)
	increased	64 (16.5%)	40 (10.1%)	104 (13.3%)

Note: Only collected after Amendment 3 to study protocol
Bayer: /by-sasp/patdb/projects/de04209/310442/stat/test_query06/pgms/t-usq.sas sgrpp 14JUL2015 13:52

Table /21: User satisfaction questionnaire by age group (FAS) (cont.)

Age category: 25 < age <= 35

Volunteer satisf. questionn., questions	Volunteer satisfaction questionnaire	LCS12	LCS16	Total
study treat, overall satisfaction	n	665 (100.0%)	667 (100.0%)	1332 (100.0%)
	missing	1 (0.2%)	0	1 (<0.1%)
	very satisfied	501 (75.3%)	529 (79.3%)	1030 (77.3%)
	somewhat satisfied	122 (18.3%)	108 (16.2%)	230 (17.3%)
	neither satisfied / dissatisfied	22 (3.3%)	14 (2.1%)	36 (2.7%)
	dissatisfied	18 (2.7%)	13 (1.9%)	31 (2.3%)
	very dissatisfied	1 (0.2%)	3 (0.4%)	4 (0.3%)
change of regimen, likelihood	n	665 (100.0%)	667 (100.0%)	1332 (100.0%)
	missing	1 (0.2%)	0	1 (<0.1%)
	continue with LCS	522 (78.5%)	561 (84.1%)	1083 (81.3%)
	use a different horm. contr.	53 (8.0%)	23 (3.4%)	76 (5.7%)
	use a different contr. meth.	44 (6.6%)	37 (5.5%)	81 (6.1%)
	discontinue use of all types of c	15 (2.3%)	18 (2.7%)	33 (2.5%)
	don't know	30 (4.5%)	28 (4.2%)	58 (4.4%)
menstrual bleeding, comparison	n	665 (100.0%)	667 (100.0%)	1332 (100.0%)
	missing	1 (0.2%)	0	1 (<0.1%)
	decreased	586 (88.1%)	620 (93.0%)	1206 (90.5%)
	no change	46 (6.9%)	31 (4.6%)	77 (5.8%)
	increased	32 (4.8%)	16 (2.4%)	48 (3.6%)
menstrual bleeding pattern	n	665 (100.0%)	667 (100.0%)	1332 (100.0%)
	missing	2 (0.3%)	2 (0.3%)	4 (0.3%)
	very satisfied	331 (49.8%)	343 (51.4%)	674 (50.6%)
	somewhat satisfied	188 (28.3%)	165 (24.7%)	353 (26.5%)
	neither satisfied / dissatisfied	51 (7.7%)	42 (6.3%)	93 (7.0%)
	dissatisfied	39 (5.9%)	40 (6.0%)	79 (5.9%)
	very dissatisfied	14 (2.1%)	9 (1.3%)	23 (1.7%)
	not applicable	40 (6.0%)	66 (9.9%)	106 (8.0%)

Table /21: User satisfaction questionnaire by age group (FAS) (cont.)

Age category: 25 < age <= 35

Volunteer satisf. questionn., questions	Volunteer satisfaction questionnaire	LCS12	LCS16	Total
menstrual bleeding, unexpected	n	665 (100.0%)	667 (100.0%)	1332 (100.0%)
	missing	2 (0.3%)	0	2 (0.2%)
	never	227 (34.1%)	223 (33.4%)	450 (33.8%)
	seldom	356 (53.5%)	381 (57.1%)	737 (55.3%)
	often	64 (9.6%)	50 (7.5%)	114 (8.6%)
	very often	16 (2.4%)	13 (1.9%)	29 (2.2%)
menstrual bleeding, absence	n	665 (100.0%)	667 (100.0%)	1332 (100.0%)
	missing	573 (86.2%)	510 (76.5%)	1083 (81.3%)
	very satisfied	79 (11.9%)	128 (19.2%)	207 (15.5%)
	somewhat satisfied	9 (1.4%)	19 (2.8%)	28 (2.1%)
	neither satisfied / dissatisfied	2 (0.3%)	6 (0.9%)	8 (0.6%)
	dissatisfied	2 (0.3%)	4 (0.6%)	6 (0.5%)
menstrual pain, treatment	n	665 (100.0%)	667 (100.0%)	1332 (100.0%)
	missing	2 (0.3%)	0	2 (0.2%)
	none	312 (46.9%)	321 (48.1%)	633 (47.5%)
	mild	288 (43.3%)	282 (42.3%)	570 (42.8%)
	moderate	52 (7.8%)	58 (8.7%)	110 (8.3%)
	severe	11 (1.7%)	6 (0.9%)	17 (1.3%)
menstrual pain, comparison	n	665 (100.0%)	667 (100.0%)	1332 (100.0%)
	missing	91 (13.7%)	80 (12.0%)	171 (12.8%)
	decreased	408 (61.4%)	437 (65.5%)	845 (63.4%)
	no change	113 (17.0%)	119 (17.8%)	232 (17.4%)
	increased	53 (8.0%)	31 (4.6%)	84 (6.3%)

Note: Only collected after Amendment 3 to study protocol
Bayer: /by-sasp/patdb/projects/de04209/310442/stat/test_query06/pgms/t-usq.sas sgrpp 14JUL2015 13:52
End of table
