# Supplementary material for: The Effect of Age, Parity and Body Mass Index on the Efficacy, Safety, Placement and User Satisfaction Associated With Two Low-Dose Levonorgestrel Intrauterine Contraceptive Systems: Subgroup Analyses of Data From a Phase III Trial
Source: PLoS One. 2015 Sep 17;10(9):e0135309. doi: 10.1371/journal.pone.0135309 (PMC4574776; doi:10.1371/journal.pone.0135309)
Supplement: S1 Protocol — (PDF) [file pone.0135309.s004.pdf]

Title page

Multi-center, open-label, randomized study to assess the safety and contraceptive efficacy of two doses (*in vitro* 12 µg/24 h and 16 µg/24 h) of the ultra low dose levonorgestrel contraceptive intrauterine systems (LCS) for a maximum of 3 years in women 18 to 35 years of age

LCS Pearl Index study

**Study purpose:**	Efficacy and safety		
**Clinical study phase:**	3	**Date:**	28 Jun 2007
**EudraCT No.:**	2007-000420-40	**Status:**	Final external approved (Identical to the final internal approved version, 28 May 2007)

Authors: **Tiina Miettunen, Taru Kosonen**

The study will be conducted in compliance with the protocol, ICH-GCP and any applicable regulatory requirements.

Confidential
The information provided in this document is strictly confidential and is intended solely for the guidance of the clinical investigation. Reproduction or disclosure of this document - whether in part or in full - to parties not associated with the clinical investigation or its use for any other purpose without the prior written consent of the sponsor is not permitted.

The approval of this document is recorded in a separate signature document.
Throughout this document, symbols indicating proprietary names (®, TM) are not displayed. Hence, the appearance of product names without these symbols does not imply that these names are not protected.

Synopsis

**Study title**	Multi-center, open-label, randomized study to assess the safety and contraceptive efficacy of two doses (*in vitro* 12 µg/24 h and 16 µg/24 h) of the ultra low dose levonorgestrel contraceptive intrauterine systems (LCS) for a maximum of 3 years in women 18 to 35 years of age
**Short title**	Phase 3 efficacy and safety study of LCS for contraception
**Clinical study phase**	3
**Study objective(s)**	The objective of this study is to assess the safety, efficacy and pharmacokinetics of 2 doses of LNG, delivered locally by a new intrauterine contraceptive system suitable for use by women 18 to 35 years of age.
**Project code**	DE-04209
**Test product(s)**	Ultra low dose levonorgestrel contraceptive system (LCS)
**Name of active ingredient**	Levonorgestrel
**Dose(s)**	*In vitro* release rate: 12 µg/24 h and 16 µg/24 h
**Route of administration**	Intrauterine
**Duration of treatment**	3 years
**Indication**	Contraception
**Diagnosis and main criteria for inclusion**	Generally healthy, 18 – 35 year-old nulliparous or parous women in need of contraception
**Study design**	Multi-center, open-label, randomized, 2-arm, parallel–group study
**Methodology**	Occurrence of pregnancies monitored
**Type of control**	Uncontrolled
**Planned study dates**	**Start of study / recruitment** Aug 2007; **End of recruitment** May 2008; **End of study** May 2011
**Planned number of study centers / countries**	160 centers/15 countries
**Number of patients**	**Total:** 2820 subjects, 1410 subjects per dose group<br>**Minimum per center:** NA **Maximum per center:** NA<br>**Total number based on statistical rationale:** Yes
**Primary variable**	Pregnancy rate
**Plan for statistical analysis**	95 % confidence interval will be calculated for the Pearl Index (number of unintended pregnancies per 100 woman years) for both dose groups separately.

Table of contents

Title page 1

Synopsis 2
Table of contents 3

Table of text tables 7

Table of text figures 8

List of abbreviations 9

Study administrative structure 10

1. Introduction 13

2. Study objectives 18

3. Overview of methodology and design 18
3.1 Study design 18
3.2 Primary variable(s) 19
3.3 Justification of the design 19
3.4 Protocol adherence 20

4. Study population 20
4.1 Eligibility 20
4.1.1 Inclusion criteria 20
4.1.2 Exclusion criteria 21
4.2 Recruitment 22
4.3 Withdrawal of patients from study participation or medication 23
4.3.1 Withdrawal 23
4.3.2 Withdrawal of treatment arms 24
4.4 Patient identification 25

5. Study drug 25
5.1 Identity 25
5.2 Dosage and administration 26
5.2.1 Insertion of the LCS 27
5.3 Treatment assignment 27
5.4 Blinding 28

5.5 Packaging and labeling 28
5.6 Drug logistics and accountability 29
5.6.1 Supply, storage, dispensation and return 29
5.6.1.1 Removal of the LCS and return to the sponsor 30
5.6.2 Drug accountability 30
5.7 Treatment compliance 31

6. Therapies other than study drug 31
6.1 Prior and concomitant therapy 31
6.2 Post-study therapy 31

7. Schedule of evaluations and visit description 32
7.1 Schedule of evaluations 32
7.2 Visit description 34
7.2.1 General 34
7.2.2 Pre-screening contact (optional) 34
7.2.3 Visit 1 – Screening 35
7.2.4 Visit 2 – Baseline 35
7.2.5 Interim visits 3, 4, 5, 6, 7, 8 and 9 36
7.2.6 Visit 10 – End of study 36
7.3 End of the study 37

8. Procedures and variables 37
8.1 Population characteristics 37
8.1.1 Demographic and other baseline characteristics 37
8.1.2 Medical and surgical history 38
8.1.3 Prior and concomitant medication 38
8.2 Efficacy 38
8.2.1 Pregnancy rate –Primary efficacy variable 38
8.3 Pharmacokinetics 39
8.3.1 Population pharmacokinetics 39
8.3.2 Methods for measurements 40
8.3.3 Pharmacokinetic evaluation 40
8.3.4 Determination of *in vivo* LNG release rate 41
8.4 Safety 41
8.4.1 Baseline findings 41
8.4.1.1 Definition of baseline finding 41
8.4.1.2 Categories, assessments and documentation of baseline findings 42

8.4.1.3 Serious baseline findings 42
8.4.2 Adverse events 43
8.4.2.1 Definition of adverse event 43
8.4.2.1.1 Special events to be reported as AEs (/SAEs) in this study 43
8.4.2.1.2 Special events with conditional reporting as AEs (/SAEs) in this study 45
8.4.2.2 Categories for adverse event assessment 46
8.4.2.3 Assessments and documentation of adverse events 50
8.4.2.4 Expected adverse events 51
8.4.2.5 Serious adverse events 53
8.4.2.5.1 Actions and reporting obligations in case of serious adverse events 54
8.4.3 Further safety 57
8.4.3.1 Vaginal bleeding 57
8.4.3.2 Dysmenorrhea 57
8.4.3.3 Vital signs and weight 57
8.4.3.4 General physical examination 58
8.4.3.5 Gynecological examination 58
8.4.3.6 Vaginal ultrasound 58
8.4.3.7 Pregnancy test 59
8.4.3.8 Cervical smear 59
8.4.3.9 Test for *Chlamydia* 60
8.4.3.10 LCS insertion ease and pain 60
8.4.3.11 LCS removal ease and pain 60
8.4.3.12 Safety laboratory tests 60
8.5 Other procedures and variables 65
8.5.1 Compliance 65
8.5.2 Use of concomitant contraceptive methods 65
8.5.3 Need of contraception 65
8.6 Appropriateness of procedures / measurements 65

9. Statistical methods and determination of sample size 65
9.1 List of variables and population characteristics 65
9.1.1 Primary efficacy variable – pregnancy rate 65
9.1.2 Population characteristics 66
9.1.2.1 Demographic and baseline characteristics 66
9.1.2.2 Gynecological baseline characteristics 67

9.1.2.3 Medical and surgical history 67
9.1.2.4 Prior and concomitant medication 68
9.1.2.5 Baseline findings 68
9.1.3 Efficacy variables 68
9.1.4 Pharmacokinetic variables 68
9.1.4.1 *In vivo* LNG release rates 68
9.1.5 Safety variables 68
9.1.5.1 Adverse events 68
9.1.5.2 Bleeding pattern 68
9.1.5.3 Dysmenorrhea 70
9.1.5.4 Progestin-related side-effects 70
9.1.5.5 LCS insertion ease and pain 70
9.1.5.6 LCS removal ease and pain 70
9.1.5.7 Gynecological findings 71
9.1.5.8 Vital signs and weight 71
9.1.5.9 Laboratory variables 71
9.1.6 Other variables and evaluations 71
9.1.6.1 LCS expulsion rate 71
9.1.6.2 Compliance 71
9.1.6.3 Discontinuation rates 72
9.2 Statistical and analytical plans 72
9.2.1 General considerations 72
9.2.2 Analysis sets 72
9.2.3 Statistical analyses 73
9.2.3.1 Population characteristics 73
9.2.3.2 Efficacy variables 73
9.2.3.2.1 Pregnancy rate 73
9.2.3.2.2 Discontinuation rate 77
9.2.3.3 Pharmacokinetic variables 77
9.2.3.4 Safety variables 77
9.2.3.4.1 Adverse events 77
9.2.3.4.2 Laboratory parameters reflecting safety 77
9.2.3.4.3 Other safety variables 77
9.3 Determination of sample size 77

10. Data handling and quality assurance 78
10.1 Data recording 78

10.2 Monitoring 79
10.3 Data processing 80
10.4 Auditing 80
10.5 Archiving 81

11. Premature termination of the study 81

12. Ethical and legal aspects 82
12.1 Ethical and legal conduct of the study 82
12.2 Patient information and consent 82
12.3 Financing / financial disclosure 83
12.4 Publication policy 84
12.5 Compensation for health damage of patients / insurance 84

13. Reference list 84

Table of text tables

Table 1. The extrapolated *in vitro* and the average estimated *in vivo* LNG release 15

Table 2: Pharmaceutical information on study drugs 26

Table 3: The English master labels for Europe/Latin America and US/Canada 29

Table 4. Schedule of assessments/events 33

Table 5: Adverse drug reactions by MedDRA for Mirena 52

Table 6: Definitions of bleeding intensity 57

Table 7: Ranges for laboratory values and their consequences 64

Table 8. WHO definitions for bleeding intensities 69

Table 9. Examples of assessments of bleeding episodes 69

Table 10. Definition of crude exposure times for the unadjusted PIs 74

Table 11: Definition of crude exposure times for the adjusted PIs 75

Table of text figures

Figure 1. Ranges and flags for laboratory values during pre-treatment phase[1] 62

Figure 2. Ranges and flags for laboratory values during treatment phase[2] 63

List of abbreviations

A	Alert value (laboratory value)
AE	Adverse event
ADR	Adverse drug reaction
ALT	Alanine aminotransferase
AP	Alkaline phosphatase
APC	Activated protein C resistance
AST	Aspartate aminotransferase
AUC	Area under the curve
BMD	Bone mineral density
BMI	Body mass index
C	Concentration
C_{AV}	Average concentration
CCT	Corporate core text
CI	Confidence interval
C_{max}	Concentration maximum
C_{min}	Concentration minimum
CRA	Clinical research associate
CRF	Case report form
CRO	Contract research organization
CRP	C-reactive protein
DXA	Dual x-ray absorptiometry
E2	Estradiol
FAS	Full analysis set
EMEA	The European Medicines Agency
FDA	Food and Drug Administration
GCP	Good clinical practice
H	High (laboratory value)
HbA1c	Hemoglobin-A1C
HCG	Human chorionic gonadotropin
HDL	High density lipoprotein
HIV	Human immunodeficiency virus
IB	Investigator's brochure
ICH	International Conference for Harmonization
IEC	Independent ethics committee
IRB	Institutional review board
ITF	Investigator trial file
IUD	Intrauterine device
IUS	Intrauterine system
IVIVC	*In vitro*/*in vivo* correlation
L	Low (laboratory value)
LCS	Ultra low dose levonorgestrel contraceptive system
LDL	Low density lipoprotein
LH	Luteinizing hormone
LLOQ	Lower limit of quantitation
LLT	Lower level term
LNG	Levonorgestrel
LVLP	Last visit of the last subject
MedDRA	Medical Dictionary for Regulatory Activities
MLS	Menopausal levonorgestrel system
P	Non-inclusion value (laboratory value)
PG	Progesterone
PI	Pearl index
PID	Patient identification number
PPS	Per protocol set
PT	Preferred term
RIA	Radioimmunoassay
SAE	Serious adverse event
SDV	Source data verification
SHBG	Sex hormone binding globulin
SOC	System organ class
SOG	Study operations guide
S1	Subset 1
S2A	Subset 2A
S2B	Subset 2B
S3	Subset 3
S4	Subset 4
STD	Sexually transmitted disease
SUSAR	Suspected unexpected serious adverse reaction
TBD	To be decided
t_{max}	Time to concentration maximum
TMF	Trial master file
WHO	World Health Organization
WHODD	World Health Organization Drug Dictionary

Study administrative structure

Role	Name	Affiliation
**Sponsor's identity:**<br>**EUROPE and Latin America**	Dr. Rolf Schürmann	Bayer Schering Pharma<br>Muellerstrasse 178<br>D-13342 Berlin<br>GERMANY<br>Tel. +49 30 468 1111<br>rolf.schuermann@bayerhealthcare.com
**Sponsor's identity:**<br>**USA and CANADA**	Dr. Angelo Secci	Bayer HealthCare Pharmaceuticals<br>PO BOX 1000<br>Montville, NJ 07045-1000 226<br>USA<br>Tel. +1 973 487 20 00 (operator)<br>http://www.bayerus.com
**Clinical Team Lead**	Dr. Ilka Schellschmidt	Bayer Schering Pharma<br>Muellerstrasse 178<br>D-13342 Berlin<br>GERMANY<br>Tel. +49 30 468 17664<br>Fax +49 30 468 15348<br>ilka.schellschmidt@bayerhealthcare.com
**Study Team Lead**	Sakari Aropuu	Bayer Schering Pharma Oy (BSP subsidiary)<br>PO BOX 22<br>Keilaranta 12<br>FI-02151 Espoo<br>FINLAND<br>Tel. +358 20 785 8469<br>Fax +358 20 785 8403<br>Sakari.aropuu@bayer.fi
**Study Biometrician**	Dr. Michael Kunz	Bayer Schering Pharma<br>Muellerstrasse 178<br>D-13342 Berlin<br>GERMANY<br>Tel. +49 30 468 17322<br>michael.kunz@ bayerhealthcare.com
**Lead Clinical Research Associate (CRA)**	Aila Havukainen	Bayer Schering Pharma Oy (BSP subsidiary)<br>PO BOX 22<br>Keilaranta 12<br>FI-02151 Espoo<br>FINLAND<br>Tel. +358 20 785 8459<br>Fax +358 20 785 8403<br>aila.havukainen@bayer.fi

Role	Name	Affiliation
**Study Pharmacokinetist**	Dr. Christian Zurth	Bayer Schering Pharma<br>Muellerstrasse 178<br>D-13342 Berlin<br>GERMANY<br>Tel. +49 30 468 14333<br>christian.zurth@bayerhealthcare.com
**Drug Safety Manager**	Dr. Juliane Schöndorf	Global Pharmacovigilance Women's Healthcare<br>Drug Safety Bayer OY<br>PO Box 22<br>FI-02151 Espoo<br>FINLAND<br>Tel. +358 20 785 2921<br>juliane.schoendorf @bayer.fi
**Drug Safety Manager USA**	Charles Walsh	Global Pharmacovigilance<br>Bayer HealthCare Pharmaceuticals<br>P.O. Box 1000<br>Montville, NJ 07045-1000<br>USA<br><br>8:00 AM to 4:00 PM (ET) Monday - Friday<br>Phone: +1 888 237 5394, Option 3<br>Fax: +1 973 709 2185<br>Nights, Weekends, and Holidays:<br>Phone: 1-888-237-5394, Option 3
**Clinical Study Supply Management**	Daniela Gehrwald	Bayer Schering Pharma<br>Clinical Supply Management<br>Muellerstrasse 178<br>D-13342 Berlin<br>GERMANY<br>Tel. +49 30 468 192370<br>daniela.gehrwald@bayerhealthcare.com
**Central laboratory: EUROPE**	Laboratorium für Klinische Forschung GmbH (LKF)	Lise-Meitner-Strasse 25-29<br>D-24223 Raisdorf<br>Germany
**Collaborative laboratory: USA and CANADA**	Mayo Clinical Trial Services	3050 Superior Drive NW<br>Rochester, MN 55901<br>United States
**Collaborative laboratory: LATIN-AMERICA**	Laboratorio Bioquimica Medica	Av. Santa Fe 2534<br>C1425BGN<br>Buenos Aires, Argentina

**Countries planned for subject recruitment**	EUROPE:<br>Finland<br>Sweden<br>Norway<br>Hungary<br>Netherlands<br>France<br>Russia	NORTH-AMERICA:<br>USA<br>Canada	LATIN AMERICA:<br>Argentina<br>Chile<br>Brazil<br>Mexico

**Notification of serious adverse events (SAE)**
**Europe and Latin America:**<br>Drug Safety<br>Bayer Schering Pharma Oy<br>Pansiontie 47<br>PO BOX 415<br>FI-20101 Turku<br>FINLAND<br>Tel. + 358 20 785 21<br>**Fax: + 358 20 785 2025**<br>e-mail: DrugSafety.SOY@bayer.fi
**North America:**<br>Global Pharmacovigilance<br>Bayer HealthCare Pharmaceuticals<br>P.O. Box 1000<br>Montville, NJ 07045-1000<br>USA<br><br>8:00 AM to 4:00 PM (ET) Monday - Friday<br>Phone: +1 888 237 5394, Option 3<br>**Fax: +1 973 709 2185**<br>Nights, Weekends, and Holidays:<br>Phone: 1-888-237-5394, Option 3

The principal investigator of each center must sign the protocol signature sheet before patient recruitment may start at the respective center. Likewise, all protocol amendments must be signed and dated by the principal investigator before coming into effect at the respective center.

A complete list of all participating centers and their investigators, as well as all required signature documents, will be maintained in the trial master file (TMF).

The coordinating investigator who signs the clinical study report will be defined prior to the last subject completing treatment.

1. Introduction

Background

Various contraceptive methods make it possible for couples to plan the number and spacing of pregnancies in a responsible way. There is a large choice of contraceptive methods available: male/female sterilization, barrier and chemical methods, periodic abstinence, post-coital pills, hormone implants and injections, combined and progesterone-only pills, copper intrauterine devices (IUD) and hormone releasing intrauterine systems (IUS). Levonorgestrel (LNG)-releasing IUS (Mirena) is one of the most reliable contraceptive methods with a contraceptive effectiveness on the same level as with female sterilization.

The mode of action of intrauterine levonorgestrel-releasing systems is multifactorial, rendering difficult the interpretation of the relative importance of the individual elements. Most of the actions are localized in the uterus. In this composite action, several factors have been identified:

Firstly, the passage of sperm cells from the vagina to the Fallopian tubes is inhibited by progestational effects and foreign body reaction:

Thickening of cervical mucus (Jonsson et al 1991, Barbosa et al 1995), strong progestin suppression of the epithelial endometrium by doses of 5 - 30 µg LNG per day (Silverberg et al, 1986) and a local effect in the uterine cavity inducing local inflammatory mediators (foreign body effect, common to all IUDs and IUSs; Ämmälä et al 1995, Nilsson et al 1995).

Secondly, via intrauterine delivery by the LNG-releasing intrauterine IUS, low amounts of LNG are absorbed into the circulation. With a mean daily dose of 20 µg (Mirena), this has been shown to have an effect on the pituitary ovarian feedback system resulting in an inhibition of ovulation that varies during the treatment time in 25 - 55% of women (Xiao 1990, Barbosa 1995).

Thirdly, signs of suppression of the endometrial activity and a reduction of the endometrial thickness were found in women using Mirena. However, the knowledge on potential cellular or biochemical changes in the endometrium is insufficient to prove anti-implantational effects of Mirena.

Fourthly, some data are supportive of fertilization being inhibited during Mirena use: interference with normal development of ova (Alvarez et al 1988), and "closure" of the fertilization window. Glycodelin A inhibits the interaction between sperm and ova. Glycodelin A is present throughout the cycle in Mirena users (Mandelin et al 1997).

Mirena has been proven to be a reliable method of contraception over the 5 years of labeled use. Mirena initially releases 20 µg LNG per day *in vitro*. Over the 5-year usage period, the average *in vivo* release is 15 µg LNG per day.

In the present clinical development program, we will study two IUSs with lower doses of LNG and smaller sizes of both the system and its insertion tube when compared with Mirena. In a phase 2 study, two experimental doses, 12 and 16µg/day (in vitro), are being compared with Mirena in contraceptive users aged 21-40 years for 3 years, with interim analyses after 1 and 2 years of treatment. A 1-year interim analysis of the following endpoints has been conducted: Pearl Index, expulsion and discontinuation rate because of (non)bleeding problems. It yielded

Pearl Indices (with 95% confidence intervals) of 0.00 (0.00; 1.64) for the 12 µg/day group, 0.43 (0.01; 2.42, due to 1 pregnancy occurring after an unnoticed expulsion) for the 16 µg/day group, and 0.00 (0.00; 1.56) for Mirena (20µg). Additionally, to date in the second year of the Phase 2 study, 2 very early accidental pregnancies were found in the 16µg group with the LCs in situ. Both pregnancies were not vital and aborted within 2 weeks of diagnosis. In the Mirena® group, an ectopic pregnancy was diagnosed and treated. Expulsion rates were low: 0.0% (12 µg/day group), 1.22% (16 µg/day group) and 1.17% (Mirena). Discontinuation rates because of (non)bleeding problems were 2.08% in the 12 µg/day group, 1.63% in the 16 µg/day group and 2.34% in the Mirena group. These first-year experiences show no differences between the 2 experimental doses of LCS and also no differences between the LCS doses and Mirena. Except for one case of pelvic inflammatory disease in the 16 µg/day group and 4 cases of ovarian cysts larger than 5 cm in the Mirena group, there were no relevant safety aspects and no differences between the doses. The pregnancy (16 µg/day group) progressed normally to term.

Thus, in the present phase 3 study 2 IUSs called "ultra low dose levonorgestrel contraceptive systems" (LCS) releasing 12 µg and 16 µg of LNG per day *in vitro* will be studied for 3 years in 18-35 years old healthy women in need of contraception.

The present phase 3 study will apply the same methodology as the phase 2 study, and have the Pearl Index (PI) as the primary endpoint parameter.

The experimental LCS to be used in this study has the following dimensions: horizontal width of the T-body: 28 mm, vertical length of 30 mm (28 mm in the phase 2 study product) and an outer diameter of the insertion tube of 3.8 mm (Mirena: 32 x 32 mm and 4.75 mm, respectively). The vertical stem bears a silver ring that gives the system a better ultrasound visibility. The LCS comes in a preloaded inserter.

Further details on the LCS can be found in the investigator's brochure, which contains comprehensive information on the study drug.

Rationale for the study

The rationale for developing a lower dose IUS than Mirena developed from the observation that in fertile women, intrauterine LNG doses from 10 to 30 µg result in similar histological changes in the endometrium (Silverberg et al 1986). Lower intrauterine doses of 5 and 10 µg LNG had equally similar effect in perimenopausal women (Wollter-Svensson et al 1995).

Clinical experience with intrauterine doses lower than 20 µg of LNG for contraception is limited to only a few studies. A study by the World Health Organization (WHO) used a 2 µg LNG releasing system of unknown specifications. This study showed inferior efficacy compared with a copper releasing IUD (WHO 1987). In a study on immediate post-partum use (30 subjects) of a system releasing 10 µg LNG, one accidental pregnancy took place (Heikkilä 1982a). In another study of 30 lactating women, no accidental pregnancies occurred during the 1-year study (Heikkilä 1982b). Post-abortion insertion is not typical as uterine involution has not taken place yet, and was also the case in the above mentioned post-partum study in which the contraceptive effect of lactation may also have played an additional role (Heikkilä 1982b).

The minimum effective intrauterine dose of LNG for contraception has not been determined. Consequently, the efficacy of the new LCS with the proposed study doses is unknown. Experiences with a 10 µg LNG (*in vitro* release) IUS (Menopausal Levonorgestrel System [MLS]) in postmenopausal women taking estrogen has shown that in the third year of treatment, when the *in vivo* release of LNG averages 3.7 µg/day, the rate of endometrial proliferation increases, indicating a loss of suppression of the endometrium. This would impair the main contraceptive action of the IUS. The estimated *in vitro* and the average *in vivo* release of LNG (µg/day) over annual periods from the experimental LCSs are given in Table 1. These figures are based on observations during the first year of use in the ongoing phase 2 study and extrapolations for the second and third year. The amount of LNG released from the LCS decreases with time, both *in vitro* and *in vivo*.

Table 1. The extrapolated *in vitro* and the average estimated *in vivo* LNG release

Year	LCS 12 µg *in vitro* / *in vivo* LNG release, µg/day	LCS 16 µg *in vitro* / *in vivo* LNG release, µg/day
1	8.8 / 6.2	11.7 / 8.1
2	7.0 / 4.9	9.3 / 6.7
3	5.6 / 3.9	7.5 / 5.5

There are no studies with an inert T-shaped frame with the dimensions of the new LCS. Inert IUDs have been replaced by copper releasing IUDs. Available data with inert IUDs mostly concern the various types of the Lippes Loop. Overall failure rates of the latest model (D) were found to be 1.3/100 woman years. Failure rates in younger women (24 – 34 years) were higher: 2.3 – 2.4/100 woman years over the duration of use of 2 years or less to 4 years or more, than in women over 35: 0.0 – 1.8/100 woman years. Increasing age and duration of use appeared to improve the efficacy of inert IUDs (Vessey et al 1982). Inert IUDs therefore have a basic contraceptive efficacy that increases with age and duration of use.

The smaller T-shaped frame (28 x 30 mm) of the LCS is designed to fit smaller uterine cavities such as in nulliparous women. This frame has been proven stable in postmenopausal women whose uterine cavity also has reduced size (expulsion rates < 1 %; Sturdee et al 2004).

Benefits of the new LCS are a lower daily dose of LNG which should lead to a reduction of progestin-related side-effects and the use of a smaller product that is easier to insert and facilitates its use by women with smaller uterine dimensions. The insertion and retention experiences in postmenopausal women with MLS, which has approximately the same dimensions as LCS, have been positive (Sturdee et al 2004).

The clinical development program is designed so that the phase 3 study, aiming at sample sizes that will allow the determination of a “true” Pearl Index, runs a year behind the phase 2 study, allowing adjustments to the study design. For example, if one of the LCS doses would not have performed as expected in the phase 2 study during the first year of treatment, the dose group in question would have been terminated at the 1-year time-point and this dose would not have been included in the phase 3 study. The next interim analysis of the phase 2 study will take place after

2 years of treatment. In order to make adjusting of the phase 3 study possible, the design of the phase 2 and phase 3 studies are largely similar.

The currently most specific and recent regulatory guideline has been followed in the development of this protocol: The European Medicines Agency, Evaluation of Medicines for Human Use. Committee for Medicinal Products for Human Use. Guideline on Clinical Investigation of Steroid Contraceptives in Women. CHMP/EWP/519/98 Rev 1, July 2005.

Benefit-risk assessment

Benefits

When the development of the new LCS is complete, the benefits of LCS will be reliable contraception for 3 years with good user compliance, and, when compared to Mirena, a lower rate of progestin-related systemic side-effects and an easier and less inconvenient insertion due to the reduced dimensions of the frame is expected.

From an individual study subject point of view the subject will be provided with a reversible contraception not requiring user intervention for a period of 3 years. During the study, each study subject will be in close medical and gynecological surveillance.

Risks

The noteworthy risks for an individual study subject are as follows:

Contraceptive failure

Contraceptive failure is possible with all available contraceptive methods. In the phase 2 study, after 1 year of treatment there has been 1 pregnancy due to an unnoticed LCS expulsion in the 16 µg dose group. This resulted in a 1-year Pearl index (PI) of 0.43 (95% CI 0.01; 2.42) in the 16 µg group; the Pearl Index for the 12 µg/day group was 0.00 (95% CI 0.00; 1.64). These Pearl Indices are favorable but it must be remembered that the treatment groups are relatively small, about 240 subjects each, and the confidence intervals are therefore wide. Additionally, to date in the second year of the Phase 2 study, 2 very early accidental pregnancies were found in the 16µg group with the LCs in situ. Both pregnancies were not vital and aborted within 2 weeks of diagnosis. In the Mirena® group, an ectopic pregnancy was diagnosed and treated. The occurrence of accidental pregnancies will be monitored on an ongoing basis. Unacceptably high rates of accidental pregnancies in one or both dose groups will lead to termination of the treatment group(s) in question.

Expulsion

Expulsion of the LCS is possible in this study. Expulsion, especially when unnoticed, may be associated with an unwanted pregnancy. In the first year of the phase 2 study, expulsion rates were 0.00% for the 12 µg dose and 1.22% for the 16 µg dose. The expulsion rate in the Mirena group was 1.17%. These rates are much lower than previously observed with Mirena (≥ 1% to < 10%), and show good retention of the experimental doses of LCS.

Pelvic infection

Pelvic infection is possible in this study. Such infection is mainly associated with behavioral factors such as multiplicity of sexual partners. In Mirena users, the rate of pelvic infection has been between 0.1% and 1% (Andersson et al, 1994). Similar incidence rates are expected for the new LCS.

Subjects with multiple sexual partners, with a high risk for sexually transmitted disease (STD) or a history of pelvic inflammatory disease, salpingitis and endometritis will not be enrolled to this study. All subjects will be screened for *Chlamydia* and also for other genital infections as needed before study entry.

In the phase 2 study, one case of pelvic inflammatory disease was reported in the 16 µg group, approximately 6 months after insertion.

Ectopic pregnancy

Ectopic pregnancy is possible in this study. The rate of ectopic pregnancy in users of Mirena has been 0.06 per 100 woman years (Luukkainen and Toivonen 1995). With Mirena, the absolute risk of ectopic pregnancy is very low but when the pregnancy occurs with Mirena in situ, the relative risk of ectopic pregnancy is increased. In contraceptive studies with a 10 µg dose of LNG, no ectopic pregnancies were observed (Heikkilä 1982), but in the WHO study using a lower 2 µg LNG-dose, several were seen (WHO 1987). Ectopic pregnancies seen during the first 2 years of use of an inert IUD were at a PI of 0.087 (overall PI 1.9) (Sivin 1985). In all, ectopic pregnancy is very rare but cannot be completely excluded with the use of the new LCS.

To date, 1 ectopic pregnancy has occurred the phase 2 study in the Mirena group.

Perforations

Uterine perforation occurs in 1/1000 of insertions and at the same rate for all intrauterine devices and hormone releasing systems including Mirena (Sivin et al, 1984,1987; Andersson et al, 1998; Harrison-Woolwych 2003).

Other safety factors

Side-effects are possible in this study. The typical progestin-related side-effects are headache, nausea, bloating, edema, skin effects, breast pain and tension and weight gain. However, these side-effects are expected to occur less frequently with the new LCS compared to Mirena. A comprehensive list of side-effects and their occurrence with Mirena users are given in Section 8.4.2.4.

Bleeding profile

LNG-releasing IUSs are associated with scanty bleeding and spotting especially during the first 3 months of use whether used for contraception or endometrial protection. The new LCS is expected to show a similar bleeding profile. In one study, the lower dose of 10 µg LNG did not affect the cyclicity of bleeding in the first year of use as did a higher dose of 30 µg LNG: 86 %

as compared to 58 % (Heikkilä 1982). Possible bleeding problems caused by the LCS in this study will not pose a substantial risk to the subject's health but rather an inconvenience.

Overall assessment of benefits and risks

All available information supports a favorable benefit-risk ratio.

2. Study objectives

The objective of this study is to assess the safety, efficacy and pharmacokinetics of 2 doses of LNG, delivered locally by a new intrauterine contraceptive system suitable for use by women 18 to 35 years of age.

3. Overview of methodology and design

3.1 Study design

This is a multi-center, randomized, open-label, 2-arm, parallel-group phase 3 study. Two doses of intrauterine administered LNG, 12 µg and 16 µg per day, will be studied. There will be no control group.

A total of 2820 generally healthy women 18 to 35 years of age desiring contraception will be randomized to 2 equal-sized treatment arms (1410 subjects per dose). The screening period per subject is targeted to last from 2 weeks to 1 month. The maximum duration of treatment will be 3 years.

The number of pregnancies will be recorded and pregnancy rate as PI will be calculated as the primary efficacy variable in this study; a secondary analysis will also be performed using the Kaplan Meier method. The secondary efficacy variables – number of IUS expulsions and discontinuations due to problems related to menstrual bleeding or non-bleeding or progestin-related side-effects, and the number of overall discontinuations will be recorded and their rates calculated.

Bleeding pattern will be evaluated from the bleeding data obtained from subject-kept diaries. Occurrence of dysmenorrhea will be recorded in the diaries as well. The investigator will evaluate the ease of LCS insertion and removal. The subject will evaluate the pain experienced during LCS insertion and removal. Adverse events will be recorded as reported voluntarily by the subject or elicited. General safety will be assured prior to entry and monitored during the study.

Serum levels of LNG and sex hormone binding globulin (SHBG) will be monitored by means of sparse blood sampling and will be evaluated using a population pharmacokinetic approach. In addition, serum LNG and SHBG levels will also be determined at the LCS removal in all subjects discontinuing the study prematurely. A detailed determination of the LNG serum level - time course will be conducted in a subgroup of study subjects (subset 3) as described in detail in

the attachment 1. In addition, the release of LNG from the LCS dose variants will be determined by means of *ex vivo* residual content analysis of used LCSs collected from 690 randomly selected subjects (345 per treatment arm) who have completed the full 3 years of treatment and LCSs from all subjects discontinuing the study prematurely.

In addition to the above variables studied in the whole study population, additional variables will be studied in 4 subsets in (pre)selected centers. Efficacy evaluations will be conducted in subsets 1 and 2A, pharmacokinetics in subset 3 and safety in subsets 2B and 4. Note, that the subjects in subsets 2A and 2B are the same individuals.

The subsets are the following:

- **Subset 1 (S1)**: Ovarian and cervical function studied in **40 subjects** (20 per treatment arm)
- **Subsets 2A (S2A) and 2B (S2B)**: Endometrial histology studied in **60 subjects** (30 per treatment arm) (S2A), and assessment of hemostatic factors (S2B) (same 60 subjects)
- **Subset 3 (S3)**: Detailed pharmacokinetics studied in **24 subjects** (12 per treatment arm)
- **Subset 4 (S4)**: Bone mineral density (BMD) studied in **200 subjects** (100 per treatment arm)

Further details of the subsets can be found in Attachment 1.

The pregnancy, discontinuation and expulsion rates will be monitored continuously.

3.2 Primary variable(s)

The primary variable of this study is the pregnancy rate. The number of pregnancies in both treatment arms will be recorded and PIs with 95 %, 2-sided confidence intervals (CI) will be calculated.

3.3 Justification of the design

The present phase 3 study is a 2-dose comparison study to determine which would be the appropriate intrauterine dose of LNG administered via the LCS for contraception (see Objectives in section 2). In the absence of valid surrogate markers, each dose needs to be evaluated in a population large enough to allow determination of the PIs with sufficient precision for the entire proposed duration of the LCS use, but also separately for the 3 treatment years. Following the current regulatory guidelines, the study does not include a reference treatment.

The 2 doses to be tested were chosen based on the evidence that intrauterine doses of 5 µg to 30 µg LNG (*in vitro* release) result in similar endometrial changes in fertile/perimenopausal women. The technically feasible lower and upper limits for *in vitro* releases that can be obtained with the LCS are 8 and 16 µg LNG per day. However, IUSs releasing 10 µg LNG or less *in vitro* are not likely to deliver enough LNG *in vivo* over the 3 years to maintain endometrial suppression, as was observed with the 10-µg MLS.

This is an open study, because the 2 LCSs can be distinguished from each other by the length of the hormone reservoir. However, treatment allocation will be randomized.

The population chosen for the study represents the target population of the new LCS.

3.4 Protocol adherence

Strict adherence to all specifications laid down in this protocol is required for all aspects of the study conduct; the investigator may not modify or alter the procedures described in this protocol. If protocol modifications are necessary, all alterations that are not solely of an administrative nature require a formal protocol amendment (see Section 12.1 for the involvement of International Ethics Committee(s) IEC(s)/International Review Board(s) IRB(s)).

If an investigator has deviated from the protocol in order to eliminate an immediate hazard to subjects or for other inevitable medical reasons, the investigator shall document all such deviations, including the reasons for the deviation, and submit the document to the sponsor and the IRB/IEC.

4. Study population

4.1 Eligibility

4.1.1 Inclusion criteria

A subject can be included into the study if all of the following criteria are met:

1. Has signed informed consent.
2. Is of age between 18 and 35 years (inclusive), in good general health and requesting contraception.
3. Has, in the opinion of the investigator, suitable general and uterine conditions for inserting the LCS.
4. Has clinically normal safety laboratory results (i.e., inside the specified range for inclusion).
5. Is willing and able to attend the scheduled visits and to comply with the study procedures.
6. Has regular menstrual cycles (length of cycle 21-35 days) (i.e., endogenous cyclicity without hormonal contraceptive use).

4.1.2 Exclusion criteria

A subject cannot be included into the study if any of the following criteria are met:

1. Known or suspected pregnancy or is lactating.
2. Vaginal delivery, cesarean delivery, or abortion within 6 weeks prior to visit 1. Note: Postpartum insertions should be postponed until uterus is fully involuted, however not earlier than 6 weeks after delivery. If involution is substantially delayed, consider waiting until 12 weeks postpartum. In case of a difficult insertion and/or exceptional pain or bleeding during or after insertion, physical examination and ultrasound should be performed immediately to exclude perforation.
3. History of ectopic pregnancies.
4. Infected abortion or postpartum endometritis within 3 months prior to visit 1.
5. Abnormal uterine bleeding of unknown origin.
6. Any genital infection (until successfully treated).
7. Descriptive diagnoses of epithelial cell atypias (not benign atypias) or more serious disorder in cervical smear (according to the Bethesda System) at screening and not responding to treatment.
8. History of, or current, pelvic inflammatory disease.
9. Congenital or acquired uterine anomaly.
10. Any distortion of the uterine cavity (by e.g., fibroids) likely to cause problems (in the opinion of the investigator) during insertion, retention or removal of the LCS.
11. History of, diagnosed or suspected genital malignancy, and untreated cervical dysplasia.
12. Current deep venous thrombosis or thrombophlebitis; history of deep venous thrombosis.
13. Current endometrial polyp(s).
14. Ovarian cyst(s) with diameter > 3 cm.
15. Concomitant use of other sex-hormone containing preparations or intrauterine device.
16. Use of any long-acting injectable sex-hormone preparations within 12 months prior to start of study medication,
    and if entering subset 2 or subset 3: any sex-hormone administration within one month prior to start of the study medication
17. If entering subset 2: any drug that might affect the blood coagulation (e.g., heparin, coumarin) within one month prior to start of the study medication

18. If entering subset 2: any known condition that might affect the blood coagulation.
19. Established immunodeficiency.
20. Any known hypersensitivity to the constituents of the LCS.
21. Diagnosed or suspected malignant or premalignant disease at the screening.
22. Arterial hypertension not responding to treatment, with systolic pressure > 140 mmHg or diastolic pressure > 90 mmHg.
23. Current (or history of) severe hepatic diseases including benign or malignant tumors. There should be an interval of at least 3 months between the start of study treatment (i.e., LCS insertion) and the return of liver function values to normal.
24. History of chronic alcoholism, drug dependence or abuse, psychotic states or severe neurosis or any other condition that, by judgment of the investigator, might impair subject's ability to cooperate.
25. Known or suspected HIV infection or high risk for STD.
26. Any clinically significant condition or laboratory result that, in the opinion of the investigator, compromises subject's safety, might interfere with the evaluations or prevents the completion of the study. Non-inclusion laboratory values will be flagged by the laboratory based on predefined ranges (see section 8.4.3.12).
27. Participated in another clinical study or consumed another experimental drug within 1 month prior to visit 1.
28. Previous participation in this study.
29. A person with close affiliation with the investigational site; e.g., close relative of the investigator, dependent person, employee or student of the investigational site.

4.2 Recruitment

From the individual study center's subject population, the investigator will identify and approach potential candidates for enrollment into the study.

Candidates for this study may be recruited by advertising in appropriate local or national newspapers or magazines. Candidates may also be recruited by advertising on bulletin boards in the study site's waiting rooms and such. An advertisement about the study may also be posted to the study site's internet homepage.

Advertising will only be done in countries where advertising for clinical studies is allowed. Furthermore, only text as accepted by the IEC/IRB of the respective country / site may be used in an advertisement in a non-provocative manner. In the advertisement a telephone number (or other contact information) is given, at which an investigator, study nurse or other person of the

study staff specifically trained for this task will answer the calls during the hours posted. More details on the procedure is provided in section 7.2.2.

The text of the advertisement as accepted by the IEC of each country is given in the TMF and ITF (of the respective country).

The recruitment for this study will be competitive. This means that no minimum or maximum number of subjects to be recruited per study site is set. However, the total number of subjects to be recruited will be as stated in Section 3.1.

4.3 Withdrawal of patients from study participation or medication

4.3.1 Withdrawal

A subject <u>must</u> always be <u>withdrawn</u> from the study treatment and the study for the following reasons:

- Withdrawal of consent. Every subject or her legal representative has the right to refuse further participation in the study at any time and without providing reasons (see also Section 12.1). A subject's participation is to terminate immediately upon her request, without prior consultation with the sponsor. The investigator should seek to obtain the reason and report this on the case report form (CRF).
- Pregnancy
- After two failed LCS insertion attempts at visit 2
- Complete or partial expulsion of the LCS (after a successful insertion)
- Partial or total perforation of the uterus or cervix by the LCS
- Pelvic inflammatory disease
- Persisting ovarian cyst with diameter > 5 cm for 3 months or longer
- Genital malignancy
- Liver tumor
- Allergic reaction to the study medication
- Participation in any other clinical study during the duration of this study
- Non-attendance of visits (i.e., if a subject does not attend 2 consecutive scheduled visits without a major reason).

In addition **withdrawing of a subject from the study treatment and study should always be considered** for the following reasons (see Sections 8.4.2.3 and 8.4.2.5.1 for AE and SAE follow-up):

- In case of idiopathic abnormal uterine bleeding
- In case any of the (other than above) exclusion criteria apply during treatment
- In case any of the following conditions exist or arise for the first time: migraine, crescendo migraine, focal migraine with asymmetrical visual loss or other symptoms indicating transient cerebral ischemia, exceptionally severe headache; jaundice; marked increase of blood pressure; confirmed or suspected hormone dependent neoplasia including breast cancer, malignancies affecting the blood or leukemia. Appropriate diagnostic and therapeutic measures should be undertaken immediately, if there are symptoms or signs indicating retinal thrombosis: unexplained partial or complete loss of vision, onset of proptosis or diplopia, papilloedema, or retinal vascular lesions.
- Serious adverse event (SAE), adverse event (AE) or laboratory abnormality of considerable clinical concern, or considerable worsening of the subject's clinical symptoms.
- Investigator's discretion.

If, at the time of withdrawal, the study drug has already been administered, all efforts should be made to perform (before withdrawal) all assessments scheduled for the end of study visit. The reasons for any withdrawal are to be fully documented on the CRF.

The time to return to fertility should be followed for up to a year in all subjects discontinuing treatment for wish of pregnancy.

Replacement

Randomized subjects whether in the basic study or in one of the subsets who terminate their study participation prematurely will not be replaced.

4.3.2 Withdrawal of treatment arms

The sponsor will closely monitor the occurrence of pregnancies, and one or both treatment arms may be halted at any time during the study if an unacceptable number of accidental pregnancies is observed. This decision of withdrawing a treatment arm will be made by the sponsor.

For the premature termination of the study, see also Section 11.

4.4 Patient identification

Upon signing the informed consent form, each subject will be assigned a unique multi-digit patient identification number (PID) for unambiguous identification. The PID will be constructed as follows:

- digits 1 and 2: unique country code
- digits 3 and 4: center code (unique within each country)
- digits 5 and 6: subject code (unique within each center)

The sponsor will provide the centers with a sufficient amount of PIDs.

<u>Randomization number</u>

Upon randomization, each eligible subject will be assigned a unique randomization number (RNR).

5. Study drug

5.1 Identity

The pharmaceutical information on the study drugs is summarized in Table 2.

Table 2: Pharmaceutical information on study drugs

**Generic name:**	Levonorgestrel (LNG)						
**Substance code number (ZK):**	18206						
**Treatment arms**	**Formula-tion number (SH)**	***In vitro* release rate of LNG (µg/day)**	**Total LNG content (mg)**	**Dimensions of the LCS (mm)**	**Drug reservoir diameter (mm)**	**Drug reservoir length (mm)**	**Inserter diameter (mm)**
**Arm 1:**	G04209F	12	13.5	28 x 30	2.8	12	3.80
**Arm 2:**	G04209G	16	19.5	28 x 30	2.8	18	3.80
**Type of formulation**	Intrauterine system (IUS)						
**Route of administration**	Intrauterine						
**Sterile product**	Yes [sterilized with ethylene oxide (EtO)]						
**Description of the pharmaceutical preparation**	The LCS consists of a polyethylene T-shape frame with a drug reservoir cylinder around the vertical stem. This reservoir consists of a mixture of polydimethylsiloxane and LNG covered by a polydimethylsiloxane membrane to generate a constant LNG release rate. The T-shaped frame has flexible, curved, horizontal arms and a vertical arm that have overall lengths as provided above. The frame is impregnated with barium sulfate to make the system X-ray detectable. On top of the vertical arm, a thickening is placed to prevent upward movement of the hormone cylinder and above this thickening a thin silver ring is placed to facilitate detection by ultrasound. Removal threads are tied to the loop at the low-end of the vertical arm of the system. The period of use for the LCSs is targeted to be 3 years.						
**Packaging**	The LCS is packed in a thermoformable blister package together with a specially designed inserter making insertion a one-handed action. The primary package is packed in a secondary cardboard package.						
**Units per package**	1						
**Storage**	Room temperature.						
**Manufacturer of the pharmaceutical preparation**	Bayer Schering Pharma Oy (subsidiary of BSP), Turku, Finland						
**Manufacturer of the substance bulkware**	Bayer Schering Pharma AG, Bergkamen, Germany						

The batch numbers and the expiry dates of the study drugs will appear on the study drug labels. A complete record of batch numbers and expiry dates of all study medication will be maintained in the TMF.

5.2 Dosage and administration

Two different *in vitro* doses (daily release rates) of intrauterine LNG are studied in this study: Arm 1: 12 µg/day and arm 2: 16 µg/day. The total LNG content in these LCSs is 13.5 and 19.5 mg, respectively. For extrapolated *in vitro* and average estimated *in vivo* LNG release rates with the 2 doses of LCS over 3 years see Table 1.

Each subject is inserted with one of the LCSs. The insertion procedure is described in Section 5.2.1. After a successful insertion, the LCS is to remain in the uterine cavity until its removal at the end of the study or at premature discontinuation of the subject from the study (see Section 5.6.1.1). The maximum duration of treatment is 3 years.

5.2.1 Insertion of the LCS

The LCS is to be inserted according to separate insertion instructions provided by the sponsor. These instructions are provided for each investigator and filed in the ITF and TMF. After the insertion of the LCS, the peel-off lid (with the label) of the empty LCS blister must be stored with the subject's study files.

During screening, the investigator should evaluate the suitability of the subject for the insertion of the LCS. The LCS should always be inserted within 7 days from the onset of menstrual bleeding. Local anesthesia, dilatation or oral painkillers and such aids can be used, if appropriate.

Two insertions may be attempted per subject at visit 2. If the second insertion also fails, the subject must be withdrawn from the study. If the LCS becomes unsterile before the insertion, or the inserter is malfunctioning, a new LCS must be used. Any LCS taken from the blister but not inserted successfully must be stored appropriately until returned to the sponsor.

If, after a successful insertion, the subject leaves the site and later the LCS is partially or totally expelled, a new LCS may not be inserted and the subject must be withdrawn from the study (see Section 4.3.1). The same holds true if the LCS has partially or totally perforated the uterus/cervix. Both, expulsion of the LCS and perforation by the LCS are to be reported as AEs; perforation also as a serious adverse event (SAE).

5.3 Treatment assignment

The treatment allocation in this study will be done by providing a computerized randomization list. Randomization will be done by the sponsor's central randomization group after the randomization program has been set up by the study statistician. Randomization will be done in blocks using a 1:1 allocation ratio. The subjects will be randomized either to the 12 µg/day or to the 16 µg/day treatment arm.

A set of randomization cards and a randomization list will be prepared for each study site. The cards will contain the information on which of the 2 treatments the subject will be allocated. From the randomization list the sites will get the information on which visit the blood sample for the population pharmacokinetics will be drawn (for further details on randomization of blood sampling for the population pharmacokinetics see Section 8.3.1). If a subject is eligible for randomization (see Section 4.1 for selection criteria), at the baseline visit (visit 2), the investigator will remove the cover and scratch off the surface of the card with the next sequential randomization number. On the card he/she will find the information on which of the 2 treatments is to be allocated to the subject. **It is of utmost importance that the randomization cards are used in ascending order to ensure the planned randomization.** No card may be skipped or left unused. Once the card is opened, the subject cannot be enrolled in the study again with a new

randomization number. Any subject, who has been randomized in the study, cannot be replaced with another subject.

After the investigator has scratched off the surface of card, he/she will take the study drug (from the stock of study drugs provided for the site) as named inside the card and insert the LCS as described in Section 5.2.1. The investigator must immediately **record the randomization number** from the card and **the treatment arm on** the corresponding **CRF** of the subject. In addition, the **randomization number** should be recorded **also on the label** of both the primary (peelable lid) and secondary (cardboard) packages of the study drug as allocated by the card and used for the subject. Furthermore, he/she must also record the PID on the labels of the primary and secondary study drug package and on the randomization card. The investigator will also enter his/her name and dated signature on the randomization card to indicate the person removing the cover of the card.

Finally, the randomization number must be recorded on the study subject screening/enrollment log. Sample master study drug labels are provided in Table 3. The scratched randomization card will be stored with the subject's study files.

5.4 Blinding

This is an open study because the LCSs are distinguishable from each other because the length of the hormone reservoir on the vertical stem of the LCS is different depending on the dose (either 12 or 18 mm). However, as far as possible, all evaluators of efficacy or safety outcomes other than the investigators or study nurses such as the persons analyzing the safety laboratory and the LNG/SHBG concentrations, will be kept blinded to treatment during the study to eliminate bias.

The allocated treatment should not be revealed to the study subject prior to the end of the treatment.

5.5 Packaging and labeling

Packaging and labeling of the study drug (the LCS) will be performed by Schering Oy (subsidiary of BSP), Turku, Finland.

Each LCS is packaged in one primary and one secondary package. The primary study drug package is a thermoformed blister package with a peelable lid. This transparent (from the "tray" side) package will contain the LCS pre-loaded into a one-hand operable inserter. The study drug label will appear on the TYVEK film (i.e., peelable lid). The LCS is a sterilized product. <u>Note that the LCS inside the blister may not be used if the blister is damaged.</u> The primary study drug package is packed into a secondary cardboard package.

The labels will be in the respective language of the country and will be in accordance with local regulations. Label terminology may vary according to local regulations, and country-specific

remarks will be added as needed. The sample label presented in Table 3 is a master label in English. The label will be attached to both primary and secondary packages.

Table 3: The English master labels for Europe/Latin America and US/Canada

Europe/Latin America:

**Study 310442**	Subject no. (PID): _____	Kit no.: X
Random. no.: _____	IUS identification no.:_____	insert before: 01/2009
Ultra Low Dose Levonorgestrel Intrauterine System LCS		Batch: XXXXXX
Levonorgestrel release rate xx µg per 24 hours.		
To be inserted according to the separate insertion instructions. Sterile unless blister is damaged or open.		
Store at 20°C to 25°C (68°F to 77°F), excursions permitted between 15°C to 30°C (59°F to 86°F).		
Keep out of reach of children. For clinical trial use only.		EudraCT-Nr.: 2007-000420-40
Investigator: ______________________	Center: ______________________	
Manufacturer: Bayer Schering Pharma Oy, PO BOX 415, Finland, Tel. +358-20-785 21		
Sponsor: Bayer Schering Pharma Oy, PO BOX 415, Finland		

X = 1 - …

xx = 12 or 16

US/Canada:

**Protocol 310442**	PID number: ___________	Kit number: X
Randomization number:________	insert before: 01/2009	Batch: XXXXX
IUS identification no: ___________		
Ultra Low Dose Levonorgestrel Intrauterine System LCS		
Levonorgestrel release rate xx µg per 24 hours.		
To be inserted according to the separate insertion instructions. Sterile unless blister is damaged or open.		
Store at 20°C to 25°C (68°F to 77°F), excursions permitted between 15°C to 30°C (59°F to 86°F).		
Caution: New Drug--Limited by Federal (or United States) law to investigational use.		
Investigator: __________________________	Center: _____________________________	
Manufactured for: Bayer HealthCare Pharmaceuticals, Montville, NJ 07045		
Manufactured in Finland		

X = 1 - …

xx = 12 or 16

5.6 Drug logistics and accountability

5.6.1 Supply, storage, dispensation and return

The study drug will not be supplied to the investigator prior to approval of the study by IRB/IEC and, if applicable, regulatory authority.

Study drugs will be supplied to the individual centers either from a central stock or via regional central stocks approved by the sponsor. For the transport to the centers, selected providers of

courier services will be used. Room temperature conditions should be maintained throughout transportation. Furthermore, the study drug packages should be packed carefully for transportation to ensure that the blister of the sterile product (the LCS) does not get damaged during transportation. All efforts will be made to limit the duration of any shipment. If upon receipt at the study center the drug supply appears damaged and/or the limits for transport temperatures were violated, the drug shall not be used without the sponsor's authorization.

The central and regional stocks must ensure that the temperature and other conditions for storage are maintained.

At the study centers, drug supplies must be kept in a secure storage area to which only authorized personnel will be allowed access. Furthermore, they must be kept at room temperature.

5.6.1.1 Removal of the LCS and return to the sponsor

The LCS will be removed by gently pulling the removal threads with forceps.

After removal (or if the subject returns an expelled LCS to the study site) the LCS is to be rinsed in cold water, dried carefully with a clean paper towel and packaged in a black plastic pouch provided by the sponsor. The PID, the randomization number and the insertion and removal dates will be entered on the pouch label. All removed or expelled LCSs should be stored in their pouches at room temperature in locked storage facilities until returned to the sponsor. *Ex vivo* content analysis will be studied on selected LCSs (for further information see Section 8.3.4).

All unused study drugs, including the ones opened but not inserted, are to be returned to the sponsor.

For contraceptive measures to be taken prior to the removal of the LCS, please see Section 6.1.

5.6.2 Drug accountability

The investigator (or pharmacist in certain countries) will confirm receipt of the study drug in writing and will use the study drug only within the framework of this clinical study and in accordance with this study protocol. He/she will keep a record of the study drugs dispensed on the drug accountability record form provided by the sponsor.

Receipt, distribution and return of the study drug must be properly documented on the forms provided by the sponsor giving the following information: study protocol number, sender, receiver, date, mode of transport, quantity, batch number and expiration or retest date, if applicable.

On the drug accountability record-form the investigator should give account of all study drugs dispensed whether used or unused and they should be returned to the sponsor. The subjects should be encouraged always to return any expelled LCS to the study site.

5.7 Treatment compliance

The study drug compliance will be assured by ultrasound at all visits after insertion. The subject is compliant with the study treatment if the LCS is completely located in the uterine cavity ('*in situ*'), displaced intrauterine or displaced in the cervical canal.

If the LCS should be partially or totally expelled or it has perforated the uterus or cervix, the subject should be withdrawn from the study (see Section 4.3.1). An expulsion of an LCS and a perforation by an LCS must be reported as an AE (perforation also as a SAE). For diagnosis of expulsion and perforation, for the means to find an expelled LCS and for AE reporting of an expulsion or perforation see Section 8.4.2.1.1.

6. Therapies other than study drug

6.1 Prior and concomitant therapy

Prior (i.e., used during screening) medication used 1 month before the screening visit, and all concomitant (i.e., used during study treatment) medication will be reported on the CRF. The subject should be interviewed for her prior and concomitant medication. The following information will be reported on the CRF: brand name of the medication, indication, dose, frequency, route of administration and start and stop dates.

The term ‘concomitant’ implies either continuation of a treatment started already before the first administration of study drug (i.e., before LCS insertion) / enrollment to the study, or addition of a new treatment during the study treatment period.

For prior and/or concomitant therapy that is not allowed prior/during the study please see the exclusion criteria in Section 4.1.2 (criteria 15, 16, 17 and 27).

When the LCS removal is scheduled (at premature discontinuation/end of the study visit), the subject should be informed to start using condom or another barrier method as a contraceptive method at least 7 days before LCS removal, unless the removal takes place during the first days of menstruation. If the LCS is removed after menstruation or during the last days of menstruation and not immediately replaced with another IUS/IUD or other method (not as part of this study), there is a risk for pregnancy. If the LCS has to be removed without prior scheduling or the subject did not use condom or another barrier method as instructed, post-coital contraception should be considered if intercourse had taken place.

6.2 Post-study therapy

Before the end of the study, the investigator should counsel the subject about the contraceptive options available after the study. If other therapy is needed the subjects should receive treatment at the investigators' discretion according to current local medical practice. These actions will be taken without the sponsor’s involvement.

7. Schedule of evaluations and visit description

7.1 Schedule of evaluations

All activities and evaluations scheduled during the study are summarized in Table 4.

All subset evaluations (schedule of events and visit descriptions) are described in Attachment 1
.

Table 4. Schedule of assessments/events

Assessment / event	Pre-treatment			Treatment							End of study[2, 3]
Visit[1]	☎/✉	1	2	3	4	5	6	7	8	9	10
Months	1st contact[4]	Screening[5]	Baseline	3	6	9	12	18	24	30	36
**<u>Initiation / subject characteristics</u>**											
Questioning for preliminary suitability	●										
Distribution of subject information	●	●									
Informed consent		●									
Demographics and baseline characteristics		●									
Medical/surgical, gynecological and menstrual history		●									
Test for *Chlamydia*		●									
Baseline findings		●	●								
Entry criteria		●	●								
**<u>Safety</u>**											
Vital signs and weight		● +height					●		●		●
General physical examination		●									●
Gynecological examination		●	●	●	●	●	●	●	●	●	●
Breast palpation		●					●		●		●
Vaginal ultrasound		●	●	●	●	●	●	●	●	●	●
Cervical smear		●									●
Safety laboratory tests		●									●
Pregnancy test		●	●								●
Prior and concomitant medication		●	●	●	●	●	●	●	●	●	●
Adverse events				●	●	●	●	●	●	●	●
**<u>Other</u>**											
Serum sample for LNG/SHBG[6]				1 sample / subject at one of the time points (visits 3 – 10)							
Randomized treatment allocation, LCS insertion[7]			●								
LCS insertion ease and pain			●								
LCS removal ease and pain											●
Concomitant contraception				●	●	●	●	●	●	●	●
Need of contraception				●	●	●	●	●	●	●	●
Diary dispensed			●								
Diary pages collected				●	●	●	●	●	●	●	●
End of study medication / end of study											●

1 Visit 3 should be performed within ±2 weeks (visit 3 for S3 within ±1 week) and visits 4 - 9 within ±4 weeks from the given visit time line. Visit 10 should be performed at end of study within -2 weeks.
2 End of study visit (visit 10) must be performed also on all subjects discontinuing the study prematurely
3 Follow-up 'Return to fertility' up to 1 year for subjects discontinuing because of 'wish for pregnancy'. All pregnancies detected within 3 months after the end of study treatment should be reported to the sponsor. Subjects will be contacted by the study site, visit not needed.
4 Optional
5 The screening period should be kept at minimum (and screening visit should never be more than 12 weeks before start of treatment)
6 Always at the premature discontinuation of the study
7 The LCS should be inserted within 7 days from the start of a (menstrual) bleeding

7.2 Visit description

7.2.1 General

Scheduled visits

There will be 10 scheduled study visits: screening and baseline visits, 7 interim visits during the study treatment and the end of study visit.

The following deviations from the visit schedule are allowed. Screening period should be kept to a minimum. Typically, baseline visit will take place 2 to 4 weeks after the screening visit. Screening visit should never be more than 12 weeks before the start of study treatment. Visit 3 should be performed within ±2 weeks and visits 4, 5, 6, 7, 8 and 9 should be performed within ±4 weeks from the given visit time line. Visit 10 should be performed at end of study (within -2 weeks).

Unscheduled visits

If deemed necessary for individual subject, it is at the discretion of the investigator to arrange visits in addition to the scheduled study visits. Possible reasons for unscheduled visits are suspicion of pregnancy, suspicion of expulsion of the LCS, suspicion of pelvic inflammatory disease or any other safety concern.

Detailed descriptions of the study procedures are provided in Section 8 unless otherwise indicated.

7.2.2 Pre-screening contact (optional)

Prior to actual screening visit, an optional pre-screening contact can be arranged. A subject candidate may be contacted / she may contact the study site (e.g., as a response to an advertisement, where applicable) typically via telephone. During this contact, the subject may be interviewed for suitability to participate in the study in accordance with the entry criteria of the study. The pre-screening contact may be performed by the investigator, study nurse or other person of the study staff specifically trained for this task.

After the telephone discussion, the Subject Information -leaflet may be sent to the subject for further information. Regardless whether the subject information leaflet was sent to the subject, it will be thoroughly explained to her at the study site prior to subject's signing of the Informed Consent-form as described in Section 12.2.

If a candidate should not be willing to consider participating in the study further or she was not suitable for screening, she will be given advice about available contraceptive options, how they can be accessed, and where to contact for further information, or if applicable, directed to treatment as per standard care.

7.2.3 Visit 1 – Screening

At the screening visit, the following procedures and assessments will be performed:

- Informative discussion about the study
- Obtaining written informed consent
- Interview for the following:
  - Demographics and baseline characteristics
  - Medical / surgical history
  - Gynecological / menstrual history
  - Prior medications
- Baseline findings
- General physical examination
- Gynecological examination (including breast palpation)
- Vaginal ultrasound
- Vital signs, weight and height
- Cervical smear
- Test for *Chlamydia*
- Safety laboratory tests
- Serum pregnancy test
- Entry criteria checked

7.2.4 Visit 2 – Baseline

At the baseline visit, the following procedures and assessments will be performed:

- Interview for the following:
  - Prior and concomitant medications
- Baseline findings
- Gynecological examination (except for palpation of the breasts)
- Urine pregnancy test
- Date of last menstruation
- Re-checking the entry criteria
- Randomization to treatment
- Insertion of the LCS (i.e., start of study medication use)
- LCS insertion ease and pain evaluations
- Vaginal ultrasound (after LCS insertion and including LCS compliance check)
- Distribution of the diary

7.2.5 Interim visits 3, 4, 5, 6, 7, 8 and 9

At the interim visits 3 (3 months), 4 (6 months), 5 (9 months), 6 (12 months), 7 (18 months), 8 (24 months) and 9 (30 months), the following procedures and assessments will be performed:

- Interview for the following:
  - Adverse events
  - Concomitant medication
  - Concomitant contraception on monthly basis
  - Need for contraception
- Gynecological examination (palpation of breasts at visits 6 and 8 only)
- Vaginal ultrasound including LCS compliance check
- Collection of completed (monthly) diary pages including the information on vaginal bleeding
- Vital signs and weight at visits 6 and 8 only
- One blood sample for the assessment of LNG and SHBG per subject at one of the interim visits (within ±1 week for visit 3 and within ±4 weeks for other interim visits). The visit for the blood sampling from an individual subject is randomized separately (see Section 8.3.1)

7.2.6 Visit 10 – End of study

At the end of study visit, the procedures and assessments listed below will be performed. If a subject should be prematurely withdrawn from the study and at the time of withdrawal LCS had already been inserted, all efforts should be made to perform (before withdrawal) all assessments scheduled for this end of study visit (see also Section 4.3).

- Interview for the following:
  - Adverse events
  - Concomitant medication
  - Concomitant contraception on monthly basis
  - Need of contraception
- Gynecological examination (including breast palpation)
- Vaginal ultrasound prior to LCS removal, including LCS compliance check
- Safety laboratory tests
- Serum pregnancy test
- Blood sampling (within -2 weeks) for LNG and SHBG prior to LCS removal (all prematurely discontinuing subjects, and those randomized to the end of study visit for population pharmacokinetics)
- Removal of the LCS (i.e., end of study medication)
- LCS removal ease and pain evaluations
- Cervical smear

- General physical examination
- Vital signs and weight
- Collection of completed (monthly) diary pages including the information on vaginal bleeding
- Discussion on future contraception with the subject. For contraceptive measures to be used before the removal of the LCS see Section 6.1.

7.3 End of the study

The end of the study is defined as the last visit of the last subject (LVLP).

Each investigator will immediately inform the sponsor about his/her center's LVLP.

8. Procedures and variables

All study procedures are explained below. The outcome of the study procedures is to be reported on the CRF, unless otherwise indicated. The schedule of the procedures is summarized in Table 4 and presented by visit in Section 7.2. The variables derived from the procedures are presented in more detail in Section 9.1.

8.1 Population characteristics

8.1.1 Demographic and other baseline characteristics

Demographic and other baseline characteristics
The subject will be interviewed for her demographic data. These data are the date of birth (month/year), age, ethnic origin and height. She will also be interviewed for her sexual relation/activity, smoking habits and alcohol consumption.

Gynecological history
The subject will be interviewed for her gynecological history. The following items will be included: age at menarche, previous menstruation, number of births, vaginal deliveries, abortions, caesarean sections and ectopic pregnancies, date of last birth or abortion, current contraceptive method, and history of genital infections.

Menstrual history
The subject will be interviewed for her menstrual history. The following items will be included: regularity of the menstrual cycle, average length of cycle, average duration and intensity of menstrual bleeding and existence of intracyclic vaginal bleeding.

8.1.2 Medical and surgical history

The subject will be interviewed for her past medical and surgical history. Detailed instructions on the differentiation between (i) medical / surgical history and (ii) baseline findings can be found in Section 8.4.1.1.

8.1.3 Prior and concomitant medication

See section 6.1.

8.2 Efficacy

8.2.1 Pregnancy rate –Primary efficacy variable

The occurrence of pregnancies will be closely monitored throughout the study. The subject will be instructed to contact the investigator or the study nurse immediately in case a pregnancy is suspected or detected. In such case, the subject should be called for an unscheduled visit as soon as possible and the investigator should verify the pregnancy by a valid method (e.g., ultrasound, serum human chorionic gonadotropin (HCG) -test).

Reporting of a pregnancy to the sponsor
In case of a pregnancy, immediate reporting to the sponsor must follow. The investigator must submit a complete report for any pregnancy detected after insertion of the LCS to the sponsor's Drug Safety Unit. The "information package" includes the AE form, pregnancy report form, demographic data form and concomitant medication form. The report must be faxed to the sponsor’s Drug Safety **immediately**, at the latest within 48 hours of having gained knowledge of the event. See section 8.4.2.5.1 for the Drug Safety Unit contact information.

A pregnancy will be reported on a Pregnancy Report Form provided by the sponsor (the data from the Pregnancy Report forms will be entered into the Drug Safety Unit's database, not in the clinical data base). The investigator is required to document, as far as possible, the estimated date of conception, the reason for pregnancy, (e.g., LCS failure), implantation, location of the LCS and information regarding whether the LCS was removed during pregnancy. The investigator is also required to provide any further information as soon as it becomes available.

In addition to the expedited reporting of the pregnancy, pregnancy is also considered as an AE (ectopic pregnancy also a SAE) and must be reported as such on the AE-CRF. Also, information on the pregnancy (implantation, IUS location, estimated date of conception and the calculated due date, whether IUS was removed during pregnancy, and early termination) will be collected on a specific CRF for the clinical data base.

The investigator should submit the pregnancy report to the sponsor's Drug Safety Unit also in a case where pregnancy is reported by the subject as a result of a positive home pregnancy test, and not wait for verification if it cannot be achieved within the 48-hour time limit. If a pregnancy reported by the subject cannot be verified by a valid method (e.g., ultrasound, serum HCG), the

investigator should inform the sponsor's Drug Safety Unit about this immediately. If a pregnancy is detected prior to the study drug administration, the subject should not be enrolled.

Monitoring of pregnancies and the pregnancy rate

The sponsor will closely monitor the pregnancy rate (based on the expedited reporting of each pregnancy by the investigators to the sponsor's Drug Safety Unit, as described above) throughout the study and one or both treatment arms may be halted at any time during the study if an unacceptable number of accidental pregnancies is observed.

All pregnancies occurring after the LCS insertion will be followed up for the final outcome of both the subject (mother) and child, if delivered. To document the pregnancy outcome, a Pregnancy Outcome Form is to be filled out by the investigator and submitted to the sponsor as described above.

All pregnancies detected within 3 months after the end of the study treatment (i.e., LCS removal), regardless whether the subject had discontinued the study prematurely or not, should be reported to the sponsor. The subjects will be instructed to contact the investigator immediately if she should become pregnant within 3 months after the LCS removal. In case the date of conception is suspected to have taken place during treatment, further investigations will be conducted.

8.3 Pharmacokinetics

Pharmacokinetic analyses will be performed at, or delegated by, the department Function Pharmacokinetics of the sponsor.

Detailed instructions on how to take, prepare, store and send the serum samples for analysis in the central laboratory are provided in a separate laboratory manual filed in the ITF and TMF.

8.3.1 Population pharmacokinetics

A blood sample for the determination of serum LNG and SHBG concentrations for population pharmacokinetics will be taken at one of the interim study visits during treatment (within ±1 week for visit 3 and within ±4 weeks for other interim visits) or at the end of the study visit per each study subject. In order to achieve an approximately equal distribution of blood samples over the whole treatment period, the time point at which the sample is taken from each subject is randomized. This means that the sampling time point for each subject will be allocated to one of the 7 interim visits or the end-of-study visit (visits 3-10). Subjects will be allocated to one of these sampling time points with the same probability, i.e., with the probability 1/7. The allocation of subjects to sample time points for population pharmacokinetics will be done using a computer generated randomization list. The study statistician will set up this additional randomization program together with randomization program for the allocation of subjects to treatment arms. The time point at which the sample is to be taken from an individual subject can be found on the randomization list provided to the study site (see also Section 5.3).

In addition, a blood sample for the determination of serum LNG and SHBG concentration will be taken from all subjects who prematurely discontinued the study. This sample will always be taken prior to the LCS removal.

The amount of blood drawn for these pharmacokinetics measurements will be approximately 5 ml per time point. Maximally 2820 subjects x 1 time point + 930 samples from subjects who discontinued the study = 3750 blood samples will be collected for the population pharmacokinetics analysis during the study.

8.3.2 Methods for measurements

Determination of LNG in serum

The LNG serum samples will be assayed by a validated bioanalytical method. The assay quality will be monitored by the analysis of quality control samples (blank serum spiked with at least 3 different concentrations of LNG within each batch). The lower limit of quantitation (LLOQ) will be determined prior to the start of the analyses.

Determination of SHBG in serum

The SHBG serum samples will be assayed by a validated immunological method. The assay quality will be monitored by the analysis of quality control samples (blank serum spiked with different concentrations of SHBG within each batch). The LLOQ is 9.8 nmol/l.

8.3.3 Pharmacokinetic evaluation

For population pharmacokinetic evaluation, concentration data collected during the study will be analyzed using non-linear mixed-effects models.

Mixed effects models, or population-type pharmacokinetic models, describe the relationship between dose and time and variables such as drug serum concentrations. Both fixed (measurable factors, e.g., dose, time, age) and random effects (not measurable factors, e.g., model misspecification) are involved in this relationship. A population pharmacokinetic compartmental model will be developed using the serum concentration of the drug as the dependent variable.

Pharmacokinetic data will be evaluated as follows:

- Definition of an appropriate structural pharmacokinetic model to characterize the pharmacokinetics of LNG in women using LCS.
- Estimation of the typical population pharmacokinetic parameters, e.g., clearance and volume of distribution, and their associated precision and variability.
- Estimation of inter-individual variability in structural model parameters and residual variability between model-predicted and observed concentrations if appropriate.
- Investigation of the potential influence of demographic and (patho-)physiological covariates (e.g., age, ethnic group, body weight, SHBG concentration, concomitant medications) on the pharmacokinetics of LNG.

- Estimation of individual parameters based on the population pharmacokinetic model obtained. The analysis will include the following parameters but will not be limited to: Clearance and the LNG concentration after 3 months, and 1, 2 and 3 years ($C_{3\ months}$, $C_{1\ year}$, $C_{2\ years}$ and $C_{3\ years}$).

A separate data analysis plan, providing details of the model building process including model evaluation will be provided prior to the start of the population pharmacokinetic analysis.

8.3.4 Determination of *in vivo* LNG release rate

For the determination of the *in vivo* release rates of LNG, the residual content of LNG in used LCSs will be determined from subjects who discontinued the study treatment prematurely. In addition, the residual content of used LCSs will be determined from 690 randomly selected subjects (345 per dose) under treatment for the full 3 years of the study.

In order to describe the intrauterine exposure for the LCS formulations, the LNG-release rates (dA_0/dt) over the 3-year treatment period will be estimated for the start of treatment and at 1, 2 and 3 years after insertion of LCS. The release rates will be obtained by fitting the LNG content data (A_t) from used LCSs versus time (t) to a mono exponential curve and subsequent calculation of the first derivative of this equation:

$$A_t = A_0 \cdot e^{-k \cdot t}$$

$$\frac{dA_t}{dt} = -k \cdot A_0 \cdot e^{-k \cdot t}$$

8.4 Safety

8.4.1 Baseline findings

8.4.1.1 Definition of baseline finding

Definition of baseline finding

A baseline finding is defined as any untoward medical condition in a study subject who has signed the informed consent form but not yet received the first dose of the study drug. This includes conditions stabilized by treatment. By definition, a baseline finding cannot be causally related to study drug; however, it may be causally related to the study (e.g., caused by study-conduct-related investigations).

Differentiation between medical/surgical history and baseline findings

Conditions which started before signature of informed consent and for which no symptoms or treatment are present until the first administration of study drug (e.g., seasonal allergy without acute complaints), are recorded as medical/surgical history.

Conditions which started before signature of informed consent and for which symptoms or treatment are present between signature of informed consent and first administration of study drug (e.g., allergic pollinosis) are recorded as baseline findings.

Differentiation between baseline findings and adverse events

Conditions (e.g., abnormal physical examination findings, symptoms, diseases, laboratory, electrocardiogram (ECG) which were present before the first administration of study drug will be documented as baseline findings.

Conditions which started or deteriorated after the first administration of study drug will be documented as adverse events.

8.4.1.2 Categories, assessments and documentation of baseline findings

Baseline findings may be obtained by any means of baseline checking of the subject's health (i.e., by questioning, voluntarily told by the subject, observing, as a result of a laboratory test, etc.). Attention is to be paid to the occurrence of baseline findings after obtaining informed consent and before administration of the study drug (i.e., LCS insertion). Any baseline finding is to be documented in detail on the CRF. The following characteristics are to be documented for each baseline finding:

- Timing
For each baseline finding, the date of onset and end of the finding is to be recorded. The date of end may not be available if the event is still ongoing at the end of the study.

- Intensity
For each baseline finding, the investigator has to assess the intensity of the event at the end of pre-treatment phase according to the same criteria as used for AEs (see Section 8.4.2.2).

- Seriousness
For each baseline finding, the investigator has to determine whether it is of serious nature according to the criteria for SAEs and report them accordingly (see Section 8.4.2.5).

8.4.1.3 Serious baseline findings

Definition

Baseline findings will be regarded as serious if they meet the criteria used for defining SAEs (see Section 8.4.2.5).

Serious baseline findings will be reported on the SAE form described in Section 8.4.2.5. For actions and reporting obligations, see Section 8.4.2.5.

For serious baseline findings, causal relationship to study conduct will also be assessed according to the criteria as used for AEs (see Section 8.4.2.2).

8.4.2 Adverse events

8.4.2.1 Definition of adverse event

The definition below follows the International Conference on Harmonization (ICH) – Good Clinical Practice (GCP) (see also ICH Guideline for Clinical Safety Data Management: Definitions and Standards for Expedited Reporting):

Adverse event (AE)

Any untoward medical occurrence in a patient or clinical investigation subject administered a pharmaceutical product and which does not necessarily have a causal relationship with this treatment. An AE can therefore be any unfavorable and unintended sign (including an abnormal laboratory finding), symptom, or disease temporally associated with the use of a medicinal (investigational) product, whether or not considered related to the medicinal (investigational) product.

By definition, for this study, all AEs are regarded as 'treatment emergent', i.e., not seen before treatment or, if already present before treatment, worsened after start of treatment.

8.4.2.1.1 Special events to be reported as AEs (/SAEs) in this study

The following events must be reported as AEs and if any of these events should fulfill the criteria of a SAE, then they must be reported also as SAEs.

- **Pregnancy**

  Normal pregnancy will be considered as an AE. However, because any pregnancy that might occur during the use of the LCS, must be followed and reported to authorities, an **"information package"** (AE form, pregnancy report form, demographic data form and concomitant medication form) will be completed and the sponsor's Drug Safety unit notified. The report of a pregnancy must be faxed to the sponsor's Drug Safety **immediately** (at the latest within 48 hours of having gained knowledge of the event) (for notification see Section 8.4.2.5.1).

> **Warning!**
> LCS is not to be used during an existing or suspected pregnancy. If the subject becomes pregnant when using LCS, removal of the system is recommended, since any intrauterine contraceptive left *in situ* may increase the risk of abortion and preterm labor. Removal of the LCS or probing of the uterus may result in spontaneous abortion. If the LCS cannot be gently removed, termination of the pregnancy may be considered. If the subject wishes to continue the pregnancy and the system cannot be withdrawn, she should be informed about the risks and the possible consequences of premature birth to the infant. The course of such pregnancy should be closely monitored. The possibility of ectopic pregnancy should be excluded. The subject should be instructed to report all symptoms that suggest complications of the pregnancy, like cramping abdominal pain with fever.
>
> Because of the intrauterine administration and the local exposure to the hormone, teratogenicity (especially virilization) cannot be completely excluded. Clinical experience of the outcomes of pregnancies under Mirena is limited due to the high contraceptive efficacy, but the subject should be informed that to date, there is no evidence of birth defects caused by Mirena use in cases where pregnancy continued to term with Mirena in place.

Ectopic pregnancy is always considered an SAE (see Section 8.4.2.5).

All pregnancies occurring during the course of the study will be followed up for the final outcome of both the subject (mother) and child, if delivered. The outcome will be documented on a Pregnancy Outcome Form and added on the pregnancy report CRF (see Section 8.2.1).

- **Expulsion of the LCS**

Total and partial expulsions will be reported as AEs.

Total expulsion is confirmed if the LCS is observed in the vagina, and/or the LCS is not shown in the uterine cavity by ultrasound or the subject confirms that the system was expelled. If the removal threads cannot be seen, expulsion is suspected. (NOTE: threads can be drawn in while the LCS is *in situ*.) Expelled LCS should be returned to the sponsor, if possible (see Section 5.6.1.1). Possibilities of cervical or uterine perforations may be excluded by ultrasound, x-ray, laparoscopy or hysteroscopy before making a final diagnosis of total expulsion.

Partial expulsion is diagnosed if the LCS can be partially seen in the vagina. If the LCS is partially expelled it needs to be removed and the subject will discontinue the study.

After total or partial expulsion of the LCS the subject will discontinue the study treatment and the study.

- **Perforation by the LCS**

  All perforations (partial or total cervical, myometrial or peritoneal perforations by the LCS) will be reported as AEs and SAEs. The LCS needs to be removed and the subject will discontinue the study.

- **Pelvic inflammatory disease**

A diagnosis of pelvic inflammatory disease will be based on:

- Presence of tenderness on pelvic examination and
- Current lower abdominal pain and
- At least 2 of the following findings:
  - Purulent or abnormal vaginal discharge
  - Increased S-CRP (≥ 30 mg/l).
  - Increased body temperature (≥ 38°C)
  - Typical findings of laparoscopy, if other clinical evidence is controversial
  - Evidence of causative pathogen (e.g., *Chlamydia trachomatis* or *Neisseria Gonorrhea*) in the cervical canal

In a case of pelvic inflammatory disease, the LCS needs to be removed and the subject will discontinue the study. The pelvic inflammatory disease report form and AE CRF will be completed in all pelvic inflammatory disease cases. If classified as an SAE, the SAE information package (SAE form, AE form, pelvic inflammatory disease report form, concomitant medication form and demographic data form) will be completed and sponsor's Drug Safety unit notified (see Section 8.4.2.5.1).

8.4.2.1.2 Special events with conditional reporting as AEs (/SAEs) in this study

The following events will not be considered or reported as AEs (/SAEs), <u>if not specifically considered as such by the investigator</u>. This is to avoid redundancy and/or inconsistent reporting of AEs as these data are already recorded on the diary or on another CRF.

- **Fibroids**

Fibroids, or their increase in number or size, should not be reported as AEs unless they cause symptoms, for example pain or vaginal bleeding.

- **Ovarian cysts**

Ovarian cysts should only be reported as AEs if they are abnormal non-functional cysts and/or have a diameter > 3 cm.

- **Findings on the bleeding diary**
- **LCS insertion or removal pain**
- **Dysmenorrhea** only if rescue medication is taken to relieve the symptoms

8.4.2.2 Categories for adverse event assessment

All AEs will be assessed and documented by the investigator according to the categories detailed below.

Seriousness

For each AE, the seriousness must be determined according to the criteria given in Section 8.4.2.5.

Intensity

The intensity of an AE is classified according to the following categories, taking into account the possible range of the intensity of the event:

- Mild
- Moderate
- Severe

Main pattern

The main pattern of the AE is to be documented as follows:

Intermittent:	Regular or irregular repeating events that are clearly of the same kind and same cause, but not clearly time related to study drug administration
Continuous:	Events that are continuously present within the whole time period which is covered by the form, but not clearly time related to study drug administration
Other:	All other patterns, need to be specified in the following text field

Study drug action

Any potential study drug action to resolve the AEs is to be documented as follows

- Drug withdrawn
- Dose not changed

Drug treatment

Specific drug treatment of AE yes/no, drug to be specified on the Medication report CRF

Non-drug treatment

Specific non-drug treatment of AE (entered in free text)

Causal relationship to study drug

The possible causal relationship between the AE and the administration of the study drug is classified according to the following definitions:

None: The time course between administration of the study drug and occurrence or worsening of the AE rules out a causal relationship.
and / or
Another cause is confirmed and no indication of involvement of the study drug in the occurrence / worsening of the AE exists.

Unlikely: The time course between administration of the study drug and occurrence or worsening of the AE makes a causal relationship unlikely.
and / or
The known effects of the study drug or of the substance class provide no indication of involvement in the occurrence / worsening of the AE and another cause adequately explaining the AE is known.
and / or
Regarding the occurrence / worsening of the AE a plausible causal chain may be deduced from the known effects of the study drug or the substance class, but another cause is much more probable.
and / or
Another cause is confirmed and involvement of the study drug in the occurrence / worsening of the AE is unlikely.

Possible: Regarding the occurrence / worsening of the AE, a plausible causal chain may be deduced from the pharmacological properties of the study drug or the substance class, but another cause just as likely to be involved is also known.
or
Although the pharmacological properties of the study drug or the substance class provide no indication of involvement in the occurrence / worsening of the AE, no other cause gives adequate explanation.

Probable: The pharmacological properties of the study drug or of the substance class,
and / or
The course of the AE after dechallenge and, if applicable, after rechallenge,
and / or
Specific tests (e.g., positive allergy test, antibodies against study drug / metabolites) suggest involvement of the study drug in the occurrence / worsening of the AE, although another cause cannot be ruled out.

Definite: The pharmacological properties of the study drug or of the substance class,
and
The course of the AE after dechallenge and, if applicable, after rechallenge,
and
Specific tests (e.g., positive allergy test, antibodies against study drug / metabolites) indicate involvement of the study drug in the occurrence / worsening of the AE and no indication of other causes exists.

‘Related’ AEs comprise the categories ‘possible’, ‘probable’ and ‘definite’.

Causal relationship to study conduct

The possible causal relationship between the AE and any study-conduct-related procedures and activities required by the protocol is classified according to the following definitions:

None: The nature of the AE or the time course between study-conduct-related procedures and activities and occurrence or worsening of the AE rules out a causal relationship.
and / or
Another cause is confirmed and no indication of involvement of the study conduct in the occurrence / worsening of the AE exists.

Unlikely: The time course between study-conduct-related procedures and activities and occurrence or worsening of the AE makes a causal relationship unlikely.
and / or
The known risks of the study-conduct-related procedures and activities provide no indication of involvement in occurrence / worsening of the AE and another cause adequately explaining the AE is known.
and / or
Regarding the occurrence / worsening of the AE, a plausible causal relationship may be deduced from the known risks of the study-conduct-related procedures and activities, but another cause is much more probable.
and / or
Another cause is confirmed and involvement of the study-conduct-related procedures and activities in the occurrence / worsening of the AE is unlikely.

Possible: Regarding the occurrence / worsening of the AE, a plausible causal relationship may be deduced from the known risks of the study-conduct-related procedures and activities, but another cause just as likely to be involved is also known.
or
Although the known risks of the study-conduct-related procedures and activities provide no indication of involvement in the occurrence / worsening of the AE, no other cause gives adequate explanation.

Probable: Regarding the occurrence / worsening of the AE, a plausible causal relationship is suggested by the known risks of the study-conduct-related procedures and activities
or
No other cause is just as likely.

Definite: Regarding the occurrence / worsening of the AE, a plausible causal relationship is suggested by the known risks of the study-conduct-related procedures and activities and other causes can be ruled out.

‘Related’ AEs comprise the categories ‘possible’, ‘probable’ and ‘definite’.

Outcome
The outcome of the AE is to be documented as follows:

- Recovered / resolved
- Recovering / resolving
- Not recovered / not resolved
- Recovered / resolved with residual effects
- Fatal
- Continuing with change
- Unknown.

8.4.2.3 Assessments and documentation of adverse events

The AEs in this study are to be obtained by the following means: observed, voluntary reporting by the subject and elicited by open questioning. The AEs are to be documented as event-based. The recording period of AEs starts from the start of the study treatment (i.e., LCS insertion) and ends at the end-of-study visit (visit 10).

Attention is to be paid to the occurrence of AEs at all stages of the study. Thus, the subject should be closely observed by the investigator both during and after the examination. At each visit, the investigator has to assess and document all new AEs. For ongoing AEs, the following information has to be entered first:

- AE
- Date of onset

As soon as an AE has ended the following information has to be entered:

- Date of end of AE
- Maximum intensity
- Causal relationship to the study drug
- Causal relationship to the study conduct
- Drug/non-drug treatment due to AE
- Study drug action
- Outcome of the AE
- Main pattern of the AE
- AE related to medical history/concomitant disease/pre-existing condition
- Seriousness

AEs still ongoing at the end of the study should be followed up as seen medically appropriate. The follow-up of AEs after the end of the study will not be recorded on the CRF.

8.4.2.4 Expected adverse events

Expected adverse drug reactions

The definition below follows ICH-GCP (see also ICH Guideline for Clinical Safety Data Management: Definitions and Standards for Expedited Reporting):

Adverse drug reaction (ADR)

In the pre-approval clinical experience with a new medicinal product or its new usages, particularly as the therapeutic dose(s) may not be established: all noxious and unintended responses to a medicinal product related to any dose should be considered as ADR. The phrase 'responses to a medicinal product' means that a causal relationship between a medicinal product and an AE is at least a reasonable possibility, i.e., the relationship cannot be ruled out.

Regarding marketed medicinal products: a response to a drug which is noxious and unintended and which occurs at doses normally used in man for prophylaxis, diagnosis, or therapy of diseases or for modification of physiological function.

For this study, the Company Core Data Sheet (CCDS) for Mirena and the most recent Investigator's Brochure (IB) are taken as the reference documents for expected ADRs. See Table 5 for adverse reactions divided by system organ classes (SOC) Medical Dictionary for Regulatory Activities (MedDRA). The frequencies are based on clinical trial data.

Table 5: Adverse drug reactions by MedDRA for Mirena

Organ system	Very common ≥1/10	Common ≥1/100 to <1/10	Uncommon ≥1/1000 to <1/100	Rare ≥1/10000 to <1/1000
**Psychiatric disorders**		Depressed mood<br>Nervousness<br>Decreased libido	Altered mood	
**Nervous systems disorders**		Headache	Migraine	
**Gastrointestinal disorders**		Abdominal pain<br>Nausea	Abdominal distension	
**Skin and subcutaneous tissue disorders**		Acne	Alopecia<br>Hirsutism<br>Pruritus<br>Eczema	Rash<br>Urticaria
**Musculoskeletal, connective tissue and bone disorders**		Back pain		
**Reproductive system and breast disorders**	Uterine/vaginal bleeding (including spotting, oligomenorrhea and amenorrhea)<br>Benign ovarian cysts	Pelvic pain<br>Dysmenorrhea<br>Vaginal discharge<br>Vulvovaginitis<br>Breast tenderness<br>Breast pain<br>Intra-uterine contraceptive device expelled	Pelvic inflammatory disease<br>Endometritis<br>Cervicitis/ Papanicolaou smear normal, class II	Uterine perforation
**General disorders and administration site conditions**			Oedema	
**Investigations**		Weight increase		

In the Table 5 the most appropriate MedDRA term is used to describe a certain reaction and its synonyms and related conditions.

If a woman becomes pregnant with Mirena *in situ*, the relative risk of ectopic pregnancy is increased. In addition, cases of breast cancer have been reported (frequency unknown).

The sponsor will assess and document the expectedness of each AE.

Adverse events of special safety interest

In this study, progestin-related side-effects are defined as given below: The terms are given either by MedDRA preferred term (PT) or lower level term (LLT).

- Breast tension (LLT)
- Breast pain (PT)
- Headache (PT)
- Nausea (PT)
- Mood changes (LLT)
- Bloating (LLT)
- Oedema (PT)
- Weight gain (LLT)
- Acne (PT) or greasy skin (LLT)

Unexpected adverse drug reactions

The term 'unexpected' as used in this definition refers to an ADR currently not included in the defined reference documents (see Section 8.4.2.4); it does not refer to the perspective of such experience not being anticipated from the pharmacological properties of the study drug.

Also, an ADR is to be considered unexpected if it adds significant information on the specificity or intensity of an expected ADR listed in the defined reference documents.

The expectedness of an AE/ADR shall be determined by the sponsor according to the defined reference documents.

8.4.2.5 Serious adverse events

Definition of serious adverse events (SAE)

<u>Definition</u>
The following SAE definition is based on ICH guidelines and the final rule issued by the Food and Drug Administration (FDA) and effective 06 Apr 1998. It is to be applied to both AEs (defined in Section 8.4.2.1) and baseline findings (defined in Section 8.4.1.1).

An SAE is classified as any untoward medical occurrence that at any dose

- Results in death, or
- Is life threatening, or
- Requires inpatient hospitalization or prolongation of existing hospitalization, or
- Results in persistent or significant disability/incapacity, or
- Is a congenital anomaly/birth defect

The following events are also always **defined as SAEs in this study**:

- Ectopic pregnancy
- Perforation by the LCS

The term ‘life threatening’ in the definition refers to an event in which the patient was at risk of death at the time of the event, it does not refer to an event which hypothetically might have caused death if it were more severe.

Medical and scientific judgment should be exercised in deciding whether it is appropriate to report an AE as serious also in other situations, such as important medical events that may not be immediately life threatening or result in death or hospitalization but may jeopardize the patient or may require intervention to prevent one of the other outcomes listed in the definition above. These should also usually be considered serious. Examples of such events are intensive treatment in an emergency room or at home for allergic bronchospasm; blood dyscrasias or convulsions that do not result in inpatient hospitalization; or development of drug dependency or drug abuse.

The following types of events are excluded from SAE reporting:

- Hospitalizations for evaluation or treatment of pre-existing conditions which do not worsen in severity or frequency during the subject's participation in the study. Such conditions must have been present prior to the subject's participation in the study and reported as such in the corresponding CRF.
- Elective surgical procedures performed for cosmetic reasons or due to pre-existing conditions as defined in the previous section.

Based on the recorded data, the sponsor will perform its own assessment of the seriousness of the AE, in addition to the investigator’s assessment of seriousness.

8.4.2.5.1 Actions and reporting obligations in case of serious adverse events

The investigator should take appropriate diagnostic and therapeutic measures to minimize the risk to the subject. When appropriate, he/she should take diagnostic measures to collect evidence for clarification of the relationship between the SAE and the study drug.

The obligation for serious baseline finding reporting starts from the signing of the informed consent by the subject and continues until study treatment is administered (i.e., insertion of the LCS). The obligation for SAE reporting starts from the moment the study treatment is administered (i.e., insertion of the LCS). Duplicate reporting of a pre-existing serious baseline finding that has not changed when the study treatment is administered will not be done. Serious baseline findings are reported using the same form as for SAE reporting.

The investigator must follow up a subject with SAE until it has resolved or a final assessment can be carried out. Unless the initial SAE report is considered complete, follow-up reports should be submitted as soon as relevant new information becomes available. For SAEs which were reported to the sponsor with the seriousness criteria “fatal” or “life threatening”, the

investigator must provide a completed written follow-up report form at the latest 7 calendar days after having reported the initial SAE. If no further information has become available, the investigator should complete the follow-up report form stating no further information has become available at the latest 7 days after reporting the initial SAE.

Investigator's notification of the sponsor

The investigator must complete the appropriate report form for all serious baseline findings/SAEs, regardless of a possible causal relationship. This report must be faxed to the sponsor's Drug Safety **immediately** (at the latest within 48 hours of having gained knowledge of the event). The report should be as complete as possible, however, if not all pertinent information is available, 'Follow-up Reports' must be completed and sent to the sponsor's Drug Safety as soon as further information or information about the outcome becomes available (at the latest within 48 hours of having gained such knowledge). For all serious baseline findings/SAEs, the investigator is required to document in full the course of the serious baseline findings/SAE and any therapy given, including any relevant findings/records in the report.

With every SAE report, a **SAE information package** should be completed and forwarded to the sponsor's Drug safety. The SAE information package includes **copies of** the following CRF:

- SAE form
- AE CRF
- Demographic data CRF
- Ongoing medication CRF

Assessment of the causal relationship to study drug (SAE form)

The possible causal relationship between the SAE and the administration of the study drug is classified as 'yes'/'no' on the SAE form instead of the 5-level scale used for AEs. Please note that 'related' AEs comprise the categories 'possible', 'probable' and 'definite'.

All serious baseline findings must also be recorded on the "Baseline Finding Form" of the CRF.

Please note, that even if normal pregnancies occurring during the use of the LCS use are not considered as SAEs they must always be reported to the sponsor's Drug Safety like SAEs with a similar information package as for SAEs and within the timelines given for SAE reporting (for details see Section 8.4.2.1.1).

All SAEs must also be recorded on the "Adverse Event Form" of the CRF.

All SAEs (and also pregnancy reports as described in Section 8.2.1) are to be reported to the applicable sponsor's drug safety unit:

**Notification of Serious Adverse Events (SAE), including Serious Baseline Findings**
**EUROPE and Latin America**:<br><br>Drug Safety<br>Bayer Schering Pharma Oy<br>PO BOX 415<br>FI-20101 Turku<br>FINLAND<br>Tel. + 358 20 785 21<br>**Fax: + 358 20 785 2025**<br>e-mail: DrugSafety.SOY@bayer.fi<br><br>**North America**:<br><br>Global Pharmacovigilance<br>Bayer HealthCare Pharmaceuticals<br>P.O. Box 1000<br>Montville, NJ 07045-1000<br>USA<br><br>8:00 AM to 4:00 PM (ET) Monday - Friday<br>Phone: +1 888 237 5394, Option 3<br>**Fax: +1 973 709 2185**<br><br>Nights, Weekends, and Holidays:<br>Phone: 1-888-237-5394, Option 3

Notification of the IEC(s)/IRB(s)

Notification of the IEC(s)/IRB(s) about all relevant events [e.g., SAEs, suspected unexpected serious adverse reactions (SUSARs)] will be performed by the sponsor and/or by the investigator according to all applicable regulations.

Notification of the authorities

The processing and reporting of all relevant events (e.g., SAEs, SUSARs) to the authorities will be done by the sponsor according to all applicable regulations.

Sponsor's notification of the investigators

The sponsor will inform all investigators about reported relevant events (e.g., fatal or life-threatening SUSARs) according to all applicable regulations.

8.4.3 Further safety

8.4.3.1 Vaginal bleeding

The occurrence of bleeding will be recorded every day during the study using diaries provided by the sponsor. The diary will resemble a calendar. The subject will be given instructions as to how to use it and will be told to bring it back when she returns to the investigator's site. She will make daily entries in the diary according to the bleeding intensity definitions in Table 6 below.

Table 6: Definitions of bleeding intensity

Category	Definition
No	No vaginal bleeding.
Spotting	Less than associated with normal menstruation relative to the subject’s experience with no need for sanitary protection (except for panty liners).
Light	Less than associated with normal menstruation relative to the subject’s experience with need for sanitary protection.
Normal	Like normal menstruation relative to the subject’s experience.
Heavy	More than normal menstruation relative to the subject’s experience.

At each study visit the completed diary cards will be collected, reviewed and signed by the investigator. Should there be some data missing, or a diary card is not available, these data will be obtained by direct questioning and entered in a substitute diary card. The original diary will become part of the CRF.

To avoid redundancy, the findings on the bleeding diary will not be recorded as AEs unless specifically considered as such by the investigator.

8.4.3.2 Dysmenorrhea

The subject will record dysmenorrhea (intensity: none/mild/moderate/severe) in the diaries.

Dysmenorrhea will not be recorded as AE unless rescue medication is taken.

8.4.3.3 Vital signs and weight

Vital signs will include the following measurements:

- Heart rate (for 1 minute, after 5 minutes in sitting position)
- Systolic and diastolic blood pressure (after 5 minutes in sitting position)

In case of clinically relevant deviations, the measurements are to be repeated before documentation.

The subject will be weighed on a scale (without shoes and dressed with approximately similarly heavy clothes each time).

8.4.3.4 General physical examination

During the general physical examination, the investigator will assess/examine at least the following:

- General appearance
- Lungs
- Cardiovascular system
- Gastrointestinal system

Any physical examination finding, symptom, or disease observed prior to study drug administration should be recorded as baseline finding. After start of study drug any deterioration or new physical examination finding, symptom, or disease should be recorded as an AE.

8.4.3.5 Gynecological examination

During the gynecological examination, the investigator will examine the following:

- Breasts (by palpation and only annually)
- Vulva
- Vagina
- Cervix
- Uterus
- Ovaries

Any gynecological examination finding, symptom, or disease observed prior to study drug administration should be recorded as baseline finding. After start of study drug any deterioration or new gynecological examination finding, symptom, or disease should be recorded as AE.

8.4.3.6 Vaginal ultrasound

The investigator will perform a vaginal ultrasound examination at every visit to assess/examine the following:

<u>At screening only</u>:

- Dimensions of the uterus
- The investigator should check that there are no distortions of the uterine cavity by fibroids or by anything else.

<u>All visits:</u>

- Double-layer thickness of the endometrium
- Endometrial findings
- Fibroids of the uterus, if any, their type, number and size of the largest one
- Other uterine abnormalities

- Ovarian findings and size
- Cysts, their number and size of the largest one
- Location of the LCS (see also Section 8.5.1 for compliance with the LCS) (not at screening).

Any abnormality detected prior to study drug administration should be recorded as a baseline finding. After start of study treatment, any deterioration or new abnormality should be recorded as an AE.

Fibroids or their increase in number or size should not be considered as AEs unless they cause symptoms, for example pain or vaginal bleeding (see also Section 8.4.2.1.2).

Ovarian cysts should only be reported as AEs if they are abnormal non-functional cysts and/or have a diameter > 3 cm (see also Section 8.4.2.1.2).

An image from each ultrasound examination should be printed out. The image should include the PID, examination date, and be signed and dated by the investigator and filed with the source documentation.

8.4.3.7 Pregnancy test

A blood sample for serum pregnancy test (for determination of HCG) will be taken at screening and end of study (visits 1 and 10), and at any interim visits if needed. This sample will be analyzed in the central laboratory. Detailed instructions on taking, handling and shipping the sample are provided in a separate laboratory manual filed in the ITF and TMF.

The subject will also be provided a urine pregnancy test (with instructions) to be done at home in the morning of the baseline visit. She is instructed to bring the performed test slip with her to the baseline visit. If the subject did not bring the test slip with her or did not perform the test, the urine pregnancy test should be done at the center to exclude the possibility of a pregnancy prior to start of the study treatment. This test may be done at the center, provided that the urine has been in the bladder for long enough to get a reliable result.

8.4.3.8 Cervical smear

The cervical smear will be sent to a central laboratory for analysis and analyzed according to the Bethesda system.

Detailed instructions on how to take, prepare and send the smear sample for analysis to the central laboratory are provided in a separate laboratory manual filed in the ITF and TMF.

Any abnormality detected prior to study drug administration should be recorded as a baseline finding. After start of study treatment, any deterioration or new abnormality should be recorded as an AE.

8.4.3.9 Test for *Chlamydia*

At screening, the subjects will be tested for *Chlamydia*. If the test result is positive, gonorrhea should also be tested. A swab sample from the cervix will be used.

Detailed instructions on how to take, prepare and send the sample for analysis to the central laboratory are provided in a separate laboratory manual filed in the ITF and TMF.

8.4.3.10 LCS insertion ease and pain

The ease of the LCS insertion will be evaluated by the investigator as easy, slightly difficult or very difficult. The use of painkillers, (para)cervical blockade or dilation will also be documented. The subject will assess the pain caused by the insertion as none, mild, moderate or severe pain.

It will be recorded whether the insertion was successful.

To avoid redundancy any recorded insertion pain will not be recorded as an AE unless specifically considered as such by the investigator.

8.4.3.11 LCS removal ease and pain

The removal of the LCS will be evaluated by the investigator as easy, slightly difficult or very difficult. The subject will assess the pain caused by the removal as none, mild, moderate or severe pain.

To avoid redundancy any recorded removal pain will not be recorded as an AE unless specifically considered as such by the investigator.

8.4.3.12 Safety laboratory tests

The following safety laboratory tests will be done at screening and at the end of the study:

- **Hematology**
  - Erythrocytes
  - Hemoglobin
  - Hematocrit
  - Leukocytes
  - Platelets
- **Serum plasma chemistry**
  - Sodium
  - Potassium
  - Creatinine
  - Total protein
  - Albumin
- **Liver enzymes**
  - Alkaline phosphatase (AP)
  - Aspartate aminotransferase (AST)
  - Gamma-GT
  - Alanine aminotransferase (ALT)
- **Lipids**
  - Total cholesterol
  - Triglycerides
  - HDL– cholesterol
  - LDL–cholesterol
- **Carbohydrate metabolism**
  - HbA1C
- **Urinalyses (dip stick)**
  - U-glucose
  - U-protein
  - U-pH
  - U-erythrocytes
  - U-leukocytes

For the laboratory measurements, blood and urine samples will be obtained from the subjects. All blood and urine specimens will be drawn with the subject in a fasting state (10 hours without eating or drinking, except for water). The amount of blood drawn for safety laboratory measurements will be approximately 10 ml. Blood samples will be clearly labeled. Preparation, storage and transport of blood/serum samples will be performed under appropriate conditions. Detailed instructions provided by the central laboratory will be provided in a separate laboratory manual filed in the ITF and TMF.

The laboratory test assays (except for the urinalyses) will be performed at a central laboratory. The urinalyses will be done locally in each study center using dip sticks provided by the sponsor.

Non-inclusion and alert ranges

According to current ICH guidelines, deviations from the reference range should be evaluated for clinical significance in each individual case. To facilitate the investigator being able to recognize meaningful increases or decreases of values out of the vast amount of laboratory data generated, 2 additional ranges are used (predefined algorithms provided in the laboratory manual).

- **Non-inclusion range (valid for pre-treatment phase)**

The non-inclusion range defines which values below/above the reference range lead to **non-inclusion** of the subject in the study. These values are flagged with “P” on the laboratory report (see Figure 1). In case a laboratory value is flagged with “P” (non-inclusion value), the investigator will assess this laboratory value on the “Assessment of laboratory values” -CRF.

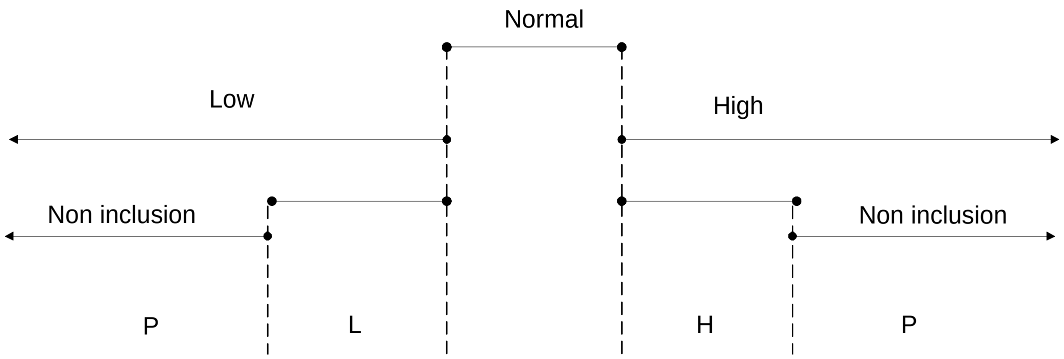

Figure 1. Ranges and flags for laboratory values during pre-treatment phase[1]

Normal = within the reference range of the respective laboratory
L = low
H = high
P = protocol deviation; value(s) within non-inclusion range
[1] Pre-treatment phase concerns all values measured before 1st administration of study medication to the individual subject

- **Alert range (valid for treatment phase including end-of-study visit)**

The alert range defines which values below/above the reference range are to be considered as significant reductions or increases. Such values have to be assessed on the CRF "Assessment of laboratory values" and to be documented as **AEs** (on laboratory AE CRF). These specific laboratory values can be related to a particular disease/symptom or can occur as an individual laboratory AE of unclear etiology. They are flagged with "A" on the laboratory report (see Figure 2).

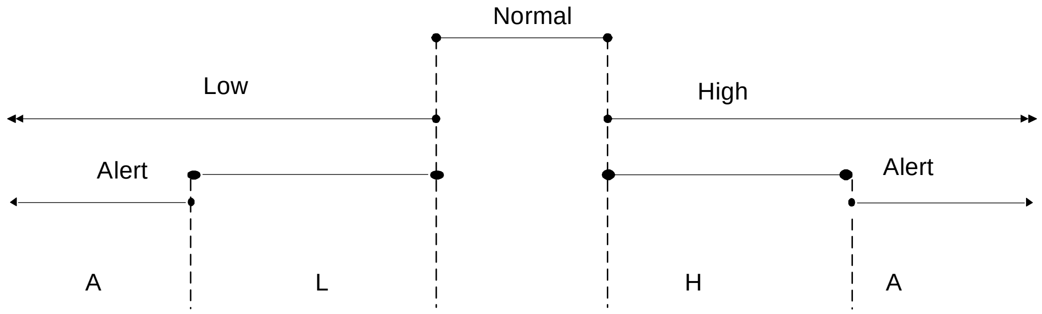

Figure 2. Ranges and flags for laboratory values during treatment phase[2]

Normal = within the reference range of the respective laboratory
L = low
H = high
A = Alert range
[2] Treatment phase: concerns all values after 1st administration of the study medication until the final visit

Non-inclusion and alert values are to be handled as summarized in Table 7.

Table 7: Ranges for laboratory values and their consequences

Flag	Term	Definition	Consequence
During entire study			
None	Reference range	Values are in the reference range	-
L	Low	Values are below the lower limit of the reference range	-
H	High	Values are above the upper limit of the reference range	-
Pre-treatment phase[1]			
P	Non-inclusion range<br><br>(Protocol deviation)	Values are below or above the reference range and reach a range not acceptable for inclusion in the study<br>(based on predefined algorithms applied to the reference range)	Subject may not be included<br><br>The investigator must assess this laboratory value on the "Assessments of laboratory values" CRF and record it as a baseline finding.<br><br>If a subject is nevertheless included in the study, this protocol deviation must be documented accordingly (as free text in CRF)<br><br>Subject may be included after re-test not flagged with P
Treatment phase[2]			
A	Alert range	Values are greatly below or above the reference range and predefined as significant abnormality (based on pre-defined algorithms applied to the reference range)	Must be documented as a laboratory AE, if the value(s) cannot be explained by an underlying disease/symptom or disturbing/influencing factor<br><br>A re-test should be performed if deemed as medically appropriate.

[1] Pre-treatment phase concerns all values measured before 1st administration of study medication to the individual subject
[2] Treatment phase: concerns all values after 1st administration of the study medication including the final visit

Further laboratory values (not flagged as alert) but assessed as clinically significant by the investigator should also be documented as laboratory AEs.

Follow-up

Alert values due to an underlying disease will be followed up as medically appropriate for the corresponding AE. Alert values of unclear etiology which led to an intervention, including withdrawal of drug treatment or significant additional concomitant therapy have to be followed up.

The reference range as well as the corresponding non-inclusion / alert ranges, the units and methods for each parameter will be provided by the central laboratory. They are described in detail in the laboratory manual filed in the ITF and TMF.

8.5 Other procedures and variables

8.5.1 Compliance

Compliance with the LCS treatment will be evaluated at every visit after LCS insertion by confirming the location of the LCS using vaginal ultrasound. In addition (at every visit after LCS insertion), the visibility of the retrieval threads should be confirmed.

The subject is considered compliant with the treatment when the location of the LCS is '*in situ*' or 'displaced in the uterine cavity'.

8.5.2 Use of concomitant contraceptive methods

At screening, the subject will be asked what contraceptive method she is currently using. During the study treatment, possible use of concomitant contraceptive method will be asked at each visit and recorded on the diary on monthly basis. The only allowed concomitant contraception is the use of a barrier method, e.g. condoms to prevent STD.

8.5.3 Need of contraception

During the study treatment period the subjects will be asked at every visit if there was a need for the contraception (i.e., if there were any coital events) since the last visit.

8.6 Appropriateness of procedures / measurements

The procedures chosen to measure the variables of this study are standard and/or widely used and generally recognized as reliable, accurate and relevant.

9. Statistical methods and determination of sample size

9.1 List of variables and population characteristics

9.1.1 Primary efficacy variable – pregnancy rate

Primary efficacy variable is the occurrence of pregnancy.

The Pearl Index (PI) is defined as the number of pregnancies per 100 woman years. The usual assumption for the calculation of the PI is a constant hazard for the event of becoming pregnant

over time. As this cannot necessarily be assumed for the experimental treatments of this study, several different PIs will be calculated.

The following PIs will be calculated:

- 'First year PI', PI obtained in the first year of treatment, i.e., number of pregnancies that occurred during the first year of treatment divided by time the women were under a risk of getting pregnant in the first year of treatment.
- 'Second year PI', PI obtained in the second year of treatment, i.e., number of pregnancies that occurred during the second year of treatment divided by time the women were under a risk of getting pregnant in the second year of treatment.
- 'Third year PI', PI obtained in the third year of treatment, i.e., number of pregnancies that occurred during the third year of treatment divided by time the women were under a risk of getting pregnant in the third year of treatment.
- 'Two years PI', PI obtained in the first two years of treatment, i.e., number of pregnancies that occurred during the first two years of treatment divided by time the women were under risk of getting pregnant in the first two years of treatment..
- 'Three years PI', PI obtained in the first three years of treatment, i.e., number of pregnancies that occurred during the first three years of treatment divided by time the women were under a risk of getting pregnant in the first three years of treatment.
- 'Overall PI', PI obtained during the whole study, i.e., number of pregnancies that occurred during treatment divided by the time the women were under a risk of getting pregnant

The exact rules regarding how the exposure time will be calculated are given in section 9.2.3.2.

9.1.2 Population characteristics

9.1.2.1 Demographic and baseline characteristics

The following demographic and baseline characteristics will be described descriptively:

- Age
- Ethnic group
- Height
- Sexual relation/activity
- Smoking habits
- Alcohol consumption

9.1.2.2 Gynecological baseline characteristics

The following gynecological baseline characteristics will be described descriptively:

Gynecological history:

- Age at menarche
- Previous menstruation
- Number of births
- Number of vaginal deliveries
- Number of abortions
- Number of cesarean sections
- Number of ectopic pregnancies
- Date of the last birth or abortion
- Current contraceptive method
- History of genital infections

Menstrual history:

- Regularity/cyclicity of cycle
- Average length of cycle
- Duration of menstruation
- Intensity of menstrual bleeding
- Intracyclic vaginal bleeding

Uterus dimensions (by ultrasound or sound):

- Length (portio-fundus)
- Maximum width anteroposterior
- Maximum width transverse
- Length of the cavity
- Fibroids distorting uterine cavity
- Sound measure

The parity is determined as:

- Nullipara = number of births is 0 (including vaginal deliveries and cesarean sections)
- Para (1, 2, 3… n) = number of births is 1, 2, 3 … n (including vaginal deliveries and cesarean sections).

The parity of a subject will be recorded on the CRF as
- Nullipara
- Para 1
- Para 2
- Para 3
- Para n.

9.1.2.3 Medical and surgical history

Medical and surgical history will be coded using MedDRA and described descriptively.

9.1.2.4 Prior and concomitant medication

Prior and concomitant medications will be coded using World Health Organization Drug Dictionary (WHODD) and described descriptively.

9.1.2.5 Baseline findings

Baseline findings will be coded using MedDRA and described.

9.1.3 Efficacy variables

Subset variables are described in Attachment 1.

9.1.4 Pharmacokinetic variables

Based on the population pharmacokinetic analysis, the following individual parameters will be determined but will not be limited to:

Clearance, $C_{3\ months}$, $C_{1\ year}$, $C_{2\ years}$ and $C_{3\ years}$.

The subset variables are described in Attachment 1.

9.1.4.1 *In vivo* LNG release rates

Based on the fitted parameters, initial amount of LNG A_0 in LCS and the rate constant k describing the decline of the residual content over time, the average daily LNG release rates at the time of insertion and after 1, 2 and 3 years will be calculated for LCS.

9.1.5 Safety variables

9.1.5.1 Adverse events

AEs coded by MedDRA terms and their characteristics will be analyzed.

9.1.5.2 Bleeding pattern

Bleeding intensities will be recorded by the subject on a daily basis using the categories presented in Table 6 in Section 8.4.3.1.

In addition to the registration of the absolute number of bleeding days, bleeding/spotting episodes will also be assessed according to the Bayer Schering Pharma bleeding intensity codes and WHO definitions provided in Table 8 below. This is possible because of the direct diary entry. The definitions for bleeding intensity and bleeding/spotting episodes are given in Table 6.

Table 8. WHO definitions for bleeding intensities

Bleeding intensity (BSP-codes)	WHO-definitions	
2 - 5	Bleeding/spotting episode	day(s) with bleeding/spotting preceded and followed by at least 2 bleed-free days
2	Spotting-only episode	day(s) with spotting preceded and followed by at least 2 bleed-free days
1	Bleeding/spotting-free interval	at least 2 day(s) without bleeding/spotting preceded and followed by at least 1 bleeding/spotting day

In addition to the WHO definitions, a ‘bleeding episode’ is defined as day(s) with bleeding/spotting of which at least one day is of intensity 3 or higher, preceded and followed by at least 2 bleed-free days.

The following few examples illustrate this procedure:

The assessment of episodes, which is based on current WHO-definitions, provides the information whether this event consisted of 5 consecutive bleeding days or whether these were separate events.

Table 9. Examples of assessments of bleeding episodes

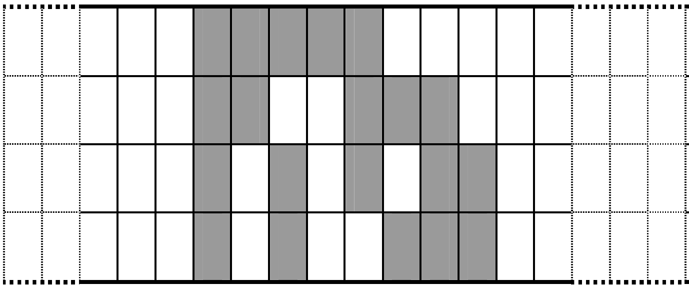

1 episode of 5 days
2 episodes, 1 of 2, and 1 of 3 days
1 episode of 8 days
2 episodes of 3 days each

Based on day-to-day data obtained from the diary cards, the bleeding pattern will be reported using reference periods of 90-days. The first 90-day reference period starts on the day of LCS insertion. For each woman and for each period, the number of bleeding/spotting days and bleeding/spotting episodes will be calculated. If the bleeding intensity is recorded as light, normal or heavy, this will be considered as a 'bleeding'.

For each woman and for each 90-day reference period, the following bleeding indices will be calculated:

- number of bleeding/spotting days
- number of bleeding days (excluding spotting)
- number of spotting only days
- number (mean length, maximal length, and range of length) of bleeding/spotting episodes
- number (mean length, maximal length, and range of length) of spotting only episodes (will only be calculated for those subjects who have at least 1 spotting-only episode)

9.1.5.3 Dysmenorrhea

Dysmenorrhea will be reported in the diaries and described with descriptive statistics.

9.1.5.4 Progestin-related side-effects

The defined progestin-related side-effects and their frequency will be analyzed.

9.1.5.5 LCS insertion ease and pain

The following variables will be described:

- Insertion ease
- Insertion pain
- Insertion success/failure and reason for failure

In addition, the use of the following during insertion will be described:

- Dilatation
- Local anesthesia
- Analgesics
- Other

9.1.5.6 LCS removal ease and pain

The following variables will be described:

- Removal ease
- Removal pain

9.1.5.7 Gynecological findings

Based on the vaginal ultrasound, the following items will be described:

Endometrium and other:
- Double layer thickness
- Endometrial findings
- Other uterine abnormalities

Fibroids:
- Number of
  - submucous fibroids
  - subserous fibroids
  - intramural fibroids
- Diameter of the largest fibroid

Ovary (left and right):
- Size
- Number of cysts
- Diameter of the largest cyst
- Other ovarian abnormalities

9.1.5.8 Vital signs and weight

Systolic and diastolic blood pressure and heart rate will be described. In addition, body weight, and calculated body mass index (BMI) will be described.

9.1.5.9 Laboratory variables

List of safety variables is given in Section 8.4.3.12. Normal (Low and High) as well as P- and A-values will be provided.

9.1.6 Other variables and evaluations

9.1.6.1 LCS expulsion rate

The LCS expulsion rate, based on the number of partial and total expulsions will be analyzed. The expulsion rate will also be analyzed for the two LCS dose groups separately. Therefore, the expulsion rates will be calculated as number of expulsions per number of subjects with successful LCS insertion per LCS dose group.

Data on expulsion rate will be used for decisions to continue the study at interim analyses after 1 and 2 years of treatment as described in Section 4.3.2.

9.1.6.2 Compliance

The compliance with the LCS will be assessed with gynecological examination and transvaginal ultrasound as follows:

- Compliant, location of the LCS =
  - *In situ*
  - Displaced intrauterine
  - Displaced in the cervical canal

- Non-compliant, location of the LCS =
  - Partially or totally expelled into vagina
  - Cervical perforation
  - Myometrial perforation
  - Peritoneal perforation
  - Absent
  - Other

9.1.6.3 Discontinuation rates

Discontinuation rates for the following categories will be analyzed:

- Overall discontinuation
- Discontinuation due to bleeding or non-bleeding problems including amenorrhea
- Discontinuation due to progestin-related side-effects
- Discontinuation due to LCS expulsions

The discontinuation rates will be calculated as number of subjects discontinuing per number of subjects that started treatment. However, discontinuations as a result of withdrawal of whole treatment arms as described in 4.3.2 should not be included in these categories.

9.2 Statistical and analytical plans

9.2.1 General considerations

Statistical analyses will be conducted by or under supervision of the designated study statistician.

All details of the statistical analyses will be provided in a statistical analysis plan that will be agreed upon before data release.

All variables will be analyzed according to the respective type of the data, i.e., at least arithmetic mean, standard deviation, median, minimum, and maximum will be provided for continuous variables and frequency counts and proportions will be provided for categorical data.

9.2.2 Analysis sets

Full analysis set (FAS)

All variables will be analyzed on the FAS. This analysis set will include all subjects randomized who received a device (LCS), using the treatment actually received. All safety evaluations will be conducted on the FAS.

Per protocol set (PPS)

No PPS will be defined, only analyses on the FAS will be carried out.

9.2.3 Statistical analyses

9.2.3.1 Population characteristics

Demographic and baseline characteristics will be presented in terms of descriptive statistics.

9.2.3.2 Efficacy variables

9.2.3.2.1 Pregnancy rate

The primary target variables are the PIs defined in Section 9.1.1.

Unadjusted and adjusted PIs will be calculated. For regulatory purposes, only the unadjusted PIs are relevant. For pregnancies that occur during the study the estimated date of conception will be provided, (i.e., a date format DDMMYY).

Definitions for unadjusted PIs are presented in Table 10.

Table 10. Definition of crude exposure times for the unadjusted PIs

PI	Reason for end of study/ continuation status	Crude exposure time
**First year PI, unadjusted**	Total expulsion	Date, when expulsion was realized – LCS insertion date +1
	Partial Expulsion/ LCS removal	Date of LCS removal, – LCS insertion date +1
	Pregnancy	Date of conception - LCS insertion date +1
	Lost to Follow up	Max (Date LCS last known in situ, 0)
	Continues into second year of treatment	365 days
**Second year PI, unadjusted**	Total expulsion	Date, when expulsion was realized – LCS insertion date +1-365
	Partial Expulsion/ LCS removal	Date of LCS removal, – LCS insertion date +1-365
	Pregnancy	Date of conception - LCS insertion date +1-365
	Lost to Follow up	Max (Date LCS last known in situ-365, 0)
	Continues into third year of treatment	365 days
**Third year PI, unadjusted**	Total expulsion	Date, when expulsion was realized – LCS insertion date +1-730
	Partial Expulsion/ LCS removal	Date of LCS removal – LCS insertion date +1-730
	Pregnancy	Date of conception - LCS insertion date +1-730
	Lost to Follow up	Max (Date LCS last known in situ-730, 0)
	Continues into forth year of treatment	365 days
**Two years PI, unadjusted**	Total expulsion	Date, when expulsion was realized – LCS insertion date +1
	Partial Expulsion/ LCS removal	Date LCS removal – LCS insertion date +1
	Pregnancy	Date of conception - LCS insertion date +1
	Lost to Follow up	Max (Date LCS last known in situ, 0)
	Continues into third year of treatment	730 days
**Three years PI, unadjusted**	Total expulsion	Date, when expulsion was realized – LCS insertion date +1
	Partial Expulsion/ LCS removal	Date of LCS removal, – LCS insertion date +1
	Pregnancy	Date of conception - LCS insertion date +1
	Lost to Follow up	Max (Date LCS last known in situ, 0)
	Continues into fourth year of treatment	1095 days
**Overall PI, unadjusted**	Total expulsion	Date, when expulsion was realized – LCS insertion date +1
	Partial Expulsion/ LCS removal	Date of LCS removal, – LCS insertion date +1
	Pregnancy	Date of conception - LCS insertion date +1
	Lost to Follow up	Max (Date LCS last known in situ, 0)

A pregnancy will be allocated to the time period(s) that are relevant for the calculation of the unadjusted PIs described above, e.g., a pregnancy that occurs on day 400 will be relevant for the second year PI, the two years PI, the three years PI, and the overall PI. Pregnancies that occur after the LCS was removed or an expulsion was realized will not count for any PI.

It should be noted that e.g., pregnancies that occur after partial expulsion, but before LCS removal will count for the unadjusted PIs.

Definitions for adjusted PIs are presented in Table 11.

Table 11: Definition of crude exposure times for the adjusted PIs

PI	Reason for end of study/ continuation status	Crude exposure time
**First year PI, adjusted**	Total expulsion	Date, when LCS was last known in situ or displaced intrauterine – LCS insertion date +1
	Partial Expulsion/ LCS removal	Date, when LCS was last known in situ or displaced intrauterine – LCS insertion date +1
	Pregnancy	Date of conception - LCS insertion date +1
	Lost to Follow up	Max (Date LCS last known in situ, 0)
	Continues into second year of treatment	365 days
**Second year PI, adjusted**	Total expulsion	Date, when LCS was last known in situ or displaced intrauterine – LCS insertion date +1
	Partial Expulsion/ LCS removal	Date, when LCS was last known in situ or displaced intrauterine – LCS insertion date +1
	Pregnancy	Date of conception - LCS insertion date +1-365
	Lost to Follow up	Max (Date LCS last known in situ-365, 0)
	Continues into third year of treatment	365 days
**Third year PI, adjusted**	Total expulsion	Date, when LCS was last known in situ or displaced intrauterine – LCS insertion date +1
	Partial Expulsion/ LCS removal	Date, when LCS was last known in situ or displaced intrauterine – LCS insertion date +1
	Pregnancy	Date of conception - LCS insertion date +1-730
	Lost to Follow up	Max (Date LCS last known in situ-730, 0)
	Continues into forth year of treatment	365 days
**Two years PI, adjusted**	Total expulsion	Date, when LCS was last known in situ or displaced intrauterine – LCS insertion date +1
	Partial Expulsion/ LCS removal	Date, when LCS was last known in situ or displaced intrauterine – LCS insertion date +1
	Pregnancy	Date of conception - LCS insertion date +1
	Lost to Follow up	Max (Date LCS last known in situ, 0)
	Continues into third year of treatment	730 days
**Three years PI, adjusted**	Total expulsion	Date, when LCS was last known in situ or displaced intrauterine – LCS insertion date +1
	Partial Expulsion/ LCS removal	Date, when LCS was last known in situ or displaced intrauterine – LCS insertion date +1
	Pregnancy	Date of conception - LCS insertion date +1
	Lost to Follow up	Max (Date LCS last known in situ, 0)
	Continues into forth year of treatment	1095 days
**Overall PI, adjusted**	Total expulsion	Date, when LCS was last known in situ or displaced intrauterine – LCS insertion date +1
	Partial Expulsion/ LCS removal	Date, when LCS was last known in situ or displaced intrauterine – LCS insertion date +1
	Pregnancy	Date of conception - LCS insertion date +1
	Lost to Follow up	Max (Date LCS last known in situ, 0)

Pregnancies that occur after the LCS was definitely not *in situ* and not displaced intrauterine (i.e., the subject was non-compliant) will not count for any adjusted PI.

Exposure times to be subtracted:

Subjects who apply additional concomitant contraception are not in risk or are under a lower risk of getting pregnant than subjects who do not apply additional contraception. In case a subject uses concomitant contraceptive methods (e.g., condoms to prevent STD), the period of additional contraceptive method use will be excluded from the exposure time.

Primary analysis

The PIs will be calculated using the model specified below. In addition, the number of pregnancies per 100 woman years and the corresponding 2-sided 95 % confidence interval (CI) will be calculated for both treatment groups separately. No direct comparisons between the two treatment groups will be made, as the assessment of efficacy is considered an estimation-problem, but not a testing problem.

Mathematical model for the calculation of the PI:

By assuming that the number of pregnancies follows a Poisson-distribution, the point estimate and the 95 % CI for the PI can be calculated as follows:

PI = x/E,

lower 95 % confidence limit of PI = $0.5 \times \chi^2_{(0.025,\, 2x)}/E$

upper 95 % confidence limit of PI = $0.5 \times \chi^2_{(0.975,\, 2(x+1))}/E$

where x = number of pregnancies,

E = exposure in 100 woman years (one woman year is 365 days of treatment exposure),

$\chi^2_{(alpha,df)}$ is the alpha quantile from χ^2-distribution with df degrees of freedom

Secondary analysis

In order to fulfill the European guideline EMEA-'Guideline on clinical investigation of steroid contraceptives in women' (EMEA/CPMP/EWP/519/ Rev1, July 2005.) the cumulative failure rate, i.e., the probability of getting pregnant will be calculated using the Kaplan Meier method in addition to calculation of the different PIs.

Hypothesis

No hypotheses are stated. The pregnancy rates will be calculated together with the 2-sided 95%-CIs as specified above.

9.2.3.2.2 Discontinuation rate

Discontinuation rates due to the following reasons will be calculated:

- LCS expulsions
- (Non-)bleeding problems
- Progestin-related side-effects
- Overall discontinuation

The Kaplan-Meier estimate will be used.

9.2.3.3 Pharmacokinetic variables

Measured serum concentrations of LNG and SHBG and derived pharmacokinetic parameters will be tabulated together with descriptive statistics.

9.2.3.4 Safety variables

Subset variables are described in Attachment 1.

9.2.3.4.1 Adverse events

All adverse events will be classified using MedDRA. The latest available version at the time of coding will be used. The results will be summarized at least on the level of system organ class (SOC) and preferred term (PT). Data will also be summarized according to severity and causality assessment.

9.2.3.4.2 Laboratory parameters reflecting safety

All safety laboratory data (including cervical smear) will be presented in terms of descriptive statistics.

9.2.3.4.3 Other safety variables

All safety data will be reported descriptively.

9.3 Determination of sample size

The EMEA-'Guideline on clinical investigation of steroid contraceptives in women' (EMEA/CPMP/EWP/519/ Rev1, July 2005.) requires conducting a study large enough to give the PI with a 2-sided 95% CI such that the difference between the upper limit of the CI and the point estimate (as pregnancies per 100 woman years) does not exceed 1.

The sample size for the study was chosen to be large enough to fulfill this requirement also for the third year alone in each of the two treatment arms. The assumptions are as follows:

- The true PI is 1.0.
- The annual drop out rate is 15%.
- Due to a use of an additional concomitant contraceptive method, the exposure time will be reduced by an additional 2%.

For a true PI of 1, the relevant exposure time should be 923 woman years, according to Benda et al 2004. Therefore, under the assumptions stated above, 923 / (0.85*0.85*0.925*0.98) = 1410 subjects should belong to both treatment arms to end with sufficient exposure time in the third year of treatment. With this sample size, the necessary exposure time would also be obtained, when assuming a true PI of 0.8, the same drop out rate as above, and a loss of exposure time due to additional concomitant contraceptive method of 11% (839(=exposure time needed) / (0.85*0.85*0.925*0.89)=1410).

For all other time intervals for which pregnancy rates shall be calculated, even more than the necessary exposure time is expected with this sample size.

10. Data handling and quality assurance

Steps will be taken at the study site and centrally to ensure the use of standard terminology and the collection of accurate, consistent, complete and reliable data, such as investigator meetings, training sessions, monitoring of investigators, instruction manuals, data verification, cross-checking, use of a central laboratory for certain tests and centralized evaluations, where applicable.

The quality assurance (including internal or external auditing procedures) and quality control systems will be implemented to assure the quality of the data and the whole study.

The investigator(s) and institution(s) will permit study-related monitoring, audits, review by IEC(s)/IRB(s) and regulatory inspections, providing direct access to source data / documents.

10.1 Data recording

Data required according to this protocol is to be recorded as soon as possible on the CRFs provided by the sponsor. Entries on the CRF must be made using a black or blue ballpoint pen and must be legible. Pencils and correction fluids must not be used.

If corrections are necessary, they will be entered by an authorized member of the investigator's staff. The wrong entry will be crossed out, although it must remain legible, and the correct entry will be placed next to it. Corrections will be initialed and dated. For corrections concerning AEs or the primary variable, a reason for any alteration must be provided.

To ensure proper source data verification, the investigator will document in the patient files (hospital files) at least the items listed below:

- Subject's identification details (name, address, date of birth)
- Date of subject's written informed consent and a statement that the consent was obtained prior to any study related procedures,
- Demographics and baseline characteristics (age, ethnic group, smoking habits, alcohol consumption, sexual relation/activity, education),
- Medical history (including diagnosis of indication being treated)
- The fact that the subject is enrolled in a clinical trial and the study number,
- Subject number (PID)/randomization number,
- Subject's visit dates including entrance and termination of the study,
- Information related to the inclusion/exclusion criteria,
- Confirmation of the diagnosis of the indication being treated (i.e., the subject is in need of contraception),
- SAEs and hospitalizations
- AEs (the CRF may contain more details than the patient file)
- Baseline findings
- Study drug administration (i.e., start and end of study treatment, reason for end if premature)
- Study drug description (i.e., levonorgestrel releasing IUS for contraception for 3 years)
- Prior and concomitant medications (the CRF may contain more details than the patient file)
- Any pregnancy occurring during the study
- Reports on centralized investigations (e.g., laboratory prints, cervical smear results) signed and dated by the investigator
- Prints of vaginal ultrasound images with PID and signed and dated by the investigator

The respective CRF/diary pages are considered as source documentation for the following data:

- Vital signs
- Recordings on the diary (e.g., vaginal bleeding)

10.2 Monitoring

This study will be monitored regularly, usually by a clinical research associate (CRA) from the sponsor or from a Contract Research Organization (CRO) (see TMF/ITF for a list of CROs). Monitoring procedures include one or more visits designed to clarify all prerequisites before the study commences. Interim monitoring visits will take place on a regular basis according to a schedule fixed by mutual agreement. During these visits, the CRA will check for completion of the entries on the CRFs, their compliance with the study protocol and with GCP, and will compare the CRF entries with the source data.

The CRA will collect originals of the completed CRFs. He/she will also verify the correct use of the study drug. The study drug will not be supplied to the investigational site prior to a favorable opinion from an IEC/IRB and regulatory authority (if applicable). At a final visit, the CRA will check all remaining material including the remaining quantities of the study drug and will organize its return.

In addition, the CRA will determine whether the investigator had appropriately reported all SAEs within the time periods required.

Depending on the variable, the following documents will be used for source data verification (SDV) (see Section 10.1 for specifications):

- Patient file
- CRF
- Diary

In case of electronic patient files, printouts will be prepared for monitoring purposes. These printouts will be signed and dated by the investigator. CRA will verify with his/her initials which printout was used for SDV purposes. Any later changes entered in the electronic patient file will be printed out for subsequent SDV.

A detailed guidance for CRAs will be given in the study operations guide (SOG) filed in the TMF.

10.3 Data processing

A Study Data Management Book (SDMB) will be maintained specifying all relevant aspects of data processing for the study (including data validation, cleaning, correcting, releasing). This SDMB will be stored in the TMF.

Medical/surgical history, baseline findings and AE data will be coded by MedDRA and medication data by WHODD.

10.4 Auditing

A member of the sponsor's (or a designated CRO) quality assurance unit may arrange to visit the investigator in order to audit the performance of the study at the study site and the study documents originating there. The auditor(s) will usually be accompanied by a CRA or the study team lead. The investigator will be informed about the outcome of the audit.

In addition, inspections by health authority representatives - including foreign authorities - and IEC(s) / IRB(s) are possible at any time. The investigator is to notify the sponsor of any such inspection immediately.

10.5 Archiving

The sponsor and the investigator/medical institution shall, in every case, retain the essential documents relating to this study for at least 15 years after its completion. They shall retain the documents for a longer period if required by other applicable regulatory requirements or by a separate agreement between the sponsor and the investigator. Essential documents shall be archived in a way that ensures that they are readily available upon authorities' request.

Patient (hospital) files will be archived according to local regulations and in accordance with the maximum period of time permitted by the hospital, institution or private practice. The ITF is not to be destroyed without the sponsor's approval. The investigator's contract will contain all regulations relevant for the study center.

11. Premature termination of the study

As detailed in this section, termination of the study at several levels may be decided (study as a whole, a specific treatment arm, at an individual center, for an individual subject). Should the study be discontinued prematurely at any level, all study materials (completed, partially completed and blank CRFs, study drug, etc.) must be returned to the sponsor. If, at the time of discontinuation of an individual subject, the study drug has already been administered the investigator should schedule a removal of the LCS at the earliest convenience and all efforts should be made to perform (before discontinuation of an individual subject) all assessments scheduled for the end of study visit.

Study as a whole

The sponsor retains the right to terminate single treatment arms prematurely or the study as a whole at any time if any of the following criteria is met:

- Based on the result of an interim analysis
- New toxicological or pharmacological findings or SAEs that invalidate the earlier positive benefit-risk assessment.
- Discontinuation of the drug development. In case the sponsor decides to discontinue the development of the study drug, the sponsor will ensure that premature termination of the study will not compromise the subjects' safety.
- The study cannot be carried out as agreed upon in the protocol.

In case of premature termination of the study as a whole, the study team lead, study manager or the local study coordinator will promptly inform the investigators/institutions, regulatory authorities and IEC(s)/IRB(s) of the termination or suspension and the reason.

Center

At any time, the study may be terminated at an individual center if:

- The center cannot comply with the requirements of the protocol.
- It is not possible for the center to comply with GCP standards.

- The center's first subject is not enrolled within 12 weeks after initiation of the center.

Subject

Individual subjects may be withdrawn from the study according to the criteria specified in Section 4.3.

12. Ethical and legal aspects

12.1 Ethical and legal conduct of the study

The study will be conducted in accordance with the following standards:

- Standard operating procedures for clinical investigation and documentation applicable at the sponsor's facilities
- Ethical principles that have their origin in the Declaration of Helsinki
- ICH-GCP Guidelines

The protocol and all protocol amendments must be submitted to the appropriate IEC/IRB and if applicable, health authorities. Protocol amendments implying substantial changes must be approved by the appropriate IEC/IRB and, if applicable, health authorities prior to implementation, except where immediate implementation in order to eliminate an imminent hazard to the subject is necessary.

The planning and conduct of this clinical study are subject to national laws. Only when all of the requirements of the appropriate regulatory authority have been fulfilled will the study begin. In particular, for each center, the study will commence only after the following conditions are met:

- Review and approval by the appropriate IEC/IRB
- Sponsor's receipt of the written notification of the IEC/IRB approval
- Signing of a contract between the sponsor and the investigator or his/her head of medical institution
- Approval by the appropriate regulatory authority as needed

Criteria for the premature termination of the study in part or as a whole are given in Section 11.

12.2 Patient information and consent

All relevant information on the study will be summarized in an integrated patient information and informed consent sheet provided by the sponsor. A sample patient information and informed consent form is provided as a document separate to this protocol.

Based on this patient information sheet, the investigator will explain all relevant aspects of the study to each subject, before her entry into the study (i.e., before examinations and procedures associated with selection for the study are performed).

The investigator will also mention that written approval of the IRB/IEC has been obtained.

Each subject will have ample time and opportunity to ask questions and will be informed about the right to withdraw from the study at any time without any disadvantage and without having to provide reasons for this decision.

Following this informative discussion, the subject will be asked if she is willing to sign and personally date a statement of informed consent, which includes consenting to the processing of her data as explained in the patient information sheet. Only if the subject agrees to sign the informed consent form and has done so, may she enter the study. Additionally, the investigator will also personally sign and date the form. The subject will receive a duplicate of the signed and dated form.

The signed informed consent statement is to remain in the ITF or, if locally required, in the patient's note/file of the medical institution.

The investigator will document on the CRF the date of obtaining informed consent. In the event that informed consent is obtained on the date that screening study procedures are performed, the study record or patient's clinical record must clearly show that informed consent was obtained prior to these procedures.

The informed consent form and any other written information provided to subjects will be revised whenever important new information becomes available that may be relevant to the subject's consent, or there is an amendment to the protocol which necessitates a change to the content of the patient information and/or the written informed consent form. The investigator will inform the subject of changes in a timely manner and will ask the subject to confirm her participation in the study by signing the revised informed consent form. Any revised written informed consent form and written information must receive the IRB/IEC's approval/favorable opinion in advance of use.

Subjects of 18 yeas of age onwards can be included in this study. In countries, where such persons are considered as minors or adults under legal protection the following applies: For minors or adults under legal protection, consent shall be given by the legal guardian(s). The consent of a minor or adult under legal protection shall also be requested. Her refusal or the withdrawal of her consent may not be disregarded.

All consent procedures will be adapted to all applicable local requirements.

12.3 Financing / financial disclosure

Financing

Funding for the study will be agreed between the investigator and the sponsor and must be confirmed in writing before the study commences.

Financial disclosure

Each investigator (including principal and/or any subinvestigators; as well as their spouses and dependent children) who is directly involved in the treatment or evaluation of research subjects has to provide a financial disclosure according to all applicable legal requirements. All relevant documentation will be filed in the TMF and/or ITF, as appropriate.

12.4 Publication policy

The sponsor is interested in the publication of the results of every study it performs. As some of the information concerning the study drug and the sponsor's development activities may be strictly confidential, any publication manuscript (including conference contributions, etc.) must first be reviewed by the sponsor before its submission or presentation.

Publication of subgroup data and single center data shall not be performed until the complete study has been published.

All relevant aspects regarding publication will be part of the contract between the sponsor and the investigator/institution.

The sponsor has committed to the global industry position on disclosure of information about clinical trials. The information regarding the study protocol is made publicly available on the internet at www.clinicaltrials.gov. This derives from the standards that international medical journal editors have established requiring protocol registration at the outset of the study as a prerequisite of consideration for publication.

12.5 Compensation for health damage of patients / insurance

Where required by the laws and regulations of the country in which the study is performed, insurance of patients against health impairment occurring as a result of participation in the study will be set up in accordance with said laws and regulations. All relevant documentation regarding such insurance will be filed in the TMF and/or ITF, as appropriate.

13. Reference list

Alvarez F, Brache V, Fernandez E, Guerrero B, Guiloff E, Hess R, Salvatierra AM, Zacharias S. New insights on the mode of action of intrauterine contraceptive devices in women. Fertil Steril 1988;49;768-73.

Andersson K, Odlind V, Rybo G. Levonorgestrel-releasing and copper-releasing (Nova T) IUDs during five years of use: A randomized comparative trial. Contraception 1994;49:56-72.

Andersson K, Ryde-Blomqvist E, Lindell K et al. Perforations with intrauterine devices. Report from a Swedish study. Contraception 1998;57:251-255.

Barbosa I, Olsson S-E, Odlind V, Goncalves T, Coutinho E. Ovarian function after seven years' use of a levonorgestrel IUD. Adv Contracept 1995;11:85-95.

Benda N, Gerlinger C, van der Meulen E.A., Endrikat J. Sample size calculation for Clinical Studies on the Efficacy of a New Contraceptive Method. Biometrical Journal 2004;46:141–150.

Harrison-Woolwych M, Zhou L, Coulter D. Insertion of intrauterine devices: a comparison with Mirena® and Multiload Cu 375® during post-marketing monitoring in New Zealand. The New Zealand Medical Journal 2003;1179:1-7

Heikkilä M. Puerperal insertion of a copper-releasing and a levonorgestrel-releasing intrauterine contraceptive device. Contraception 1982a; 25(6):561-572.

Heikkilä M, Lähteenmäki P, Luukkainen T. Immediate postabortal insertion of a levonorgestrel-releasing IUD. Contraception 1982b; 26(3):245-259.

Jonsson B, Landgren BM, Eneroth P. Effects of various IUDs on the composition of cervical mucus. Contraception 1991;43;447-58.

Luukkainen T, Toivonen J. Levonorgestrel-releasing IUD as a method of contraception with therapeutic properties. Contraception 1995;52:269-276.

Mandelin E, Koistinen H, Koistinen R, Affandi B, Seppala M. Levonorgestrel-releasing intrauterine device -wearing women express contraceptive glycodelin A in endometrium during midcycle: another contraceptive mechanism? Human Reprod 1997;12;2671-5.

Nilsson CG, Lähteenmäki P, Luukkainen T. Ovarian function in amenorrheic and menstruating users of a levonorgestrel-releasing intrauterine device. Fertil Steril 1984;41: 52-5.

Silverberg SG, Haukkamaa M, Arko H, Nilsson GC, Luukkainen T. Endometrial morphology during long-term use of levonorgestrel-releasing intrauterine devices. Int J Gynecol Pathology 1986;5:235-241.

Sivin I. IUD-associated ectopic pregnancies. In : Zatuchni G ed: Intrauterine Contraception, Advances and Future Prospects. Harper & Row, Philadelphia 1985;340-353.

Sivin I, Alvarez F, Diaz J et al. Intrauterine contraception with levonorgestrel and with copper: a randomized comparison of Tcu 380 Ag and levonorgestrel 20µg/day devices. Contraception 1984; 30:443-456.

Sivin I, Stern J, Diaz J et al. Two years of intrauterine contraception with levonorgestrel and with copper: a randomized comparison of the Tcu 380 Ag and levonorgestrel 20µg devices. Contraception 1987; 35:245-255.

Sturdee DW, Rantala ML, Colau J, Zahradnik HP, Riphagen FE. The acceptability of a small intrauterine progestogen-releasing system for continuous combined hormone therapy in early postmenopausal women. Climacteric 2004; 7:404-411.

Vessey M, Lawless M, Yeates D. Efficacy of different contraceptive methods. Lancet 1982; April: 841-842.

Wollter-Svensson LO, Stadberg E, Andersson K, Mattsson LA, Odlind V, Persson I. Intrauterine administration of levonorgestrel in two low doses in HRT. A randomized clinical trial during one year: effects on lipids and lipoprotein metabolism. Maturitas 1995; 22:199-205.

World Health Organization Special Programme of Research, Development and Research Training in Human Reproduction. Microdose intrauterine levonorgestrel for contraception. Contraception 1987; 35:363-379.

Xiao B, Zhou L, Zhang X, Jia M, Luukkainen T, Allonen H. Pharmacokinetic and pharmacodynamic studies of levonorgestrel-releasing intrauterine device. Contraception 1990; 41;353-62.

Ämmälä M, Nyman T, Strengel L, Rutanen E-M. Effect of intrauterine contraceptive devices on cytokine messenger ribonucleic acid expression in the human endometrium. Fertil Steril 1995; 63;773-8.
